# Cation affinity numbers of Lewis bases

**DOI:** 10.3762/bjoc.8.163

**Published:** 2012-08-31

**Authors:** Christoph Lindner, Raman Tandon, Boris Maryasin, Evgeny Larionov, Hendrik Zipse

**Affiliations:** 1 Department of Chemistry, Ludwigs-Maximilians-Universität München, Butenandstr. 5–13, D-81377 München, Germany

**Keywords:** ab initio, cation affinity, Lewis basicity, organocatalysis, proton affinity

## Abstract

Using selected theoretical methods the affinity of a large range of Lewis bases towards model cations has been quantified. The range of model cations includes the methyl cation as the smallest carbon-centered electrophile, the benzhydryl and trityl cations as models for electrophilic substrates encountered in Lewis base-catalyzed synthetic procedures, and the acetyl cation as a substrate model for acyl-transfer reactions. Affinities towards these cationic electrophiles are complemented by data for Lewis-base addition to Michael acceptors as prototypical neutral electrophiles.

## Introduction

Cation affinity values are important guidelines for the reactivity of Lewis and Brønstedt bases [1–3]. While proton affinity numbers (either as gas phase proton affinities or as solution phase p*K_a_* values) have been used for a long time in quantitative approaches to describe base-induced or base-catalyzed processes, affinity data towards carbon electrophiles have only recently been adopted as tools for the assessment of Lewis base reactivity [4]. This is mainly due to the scarcity of accurate experimentally measured or theoretically calculated data. The performance of various theoretical methods to provide accurate affinity data has recently been analyzed and a number of cost-efficient methods for the determination of accurate gas phase values have been identified [5–6]. Using these methods we now present a broad overview over the cation affinities of N- and P-based nucleophiles.

## Results and Discussion

### Methyl cation affinities (MCA)

**Scheme 1:**
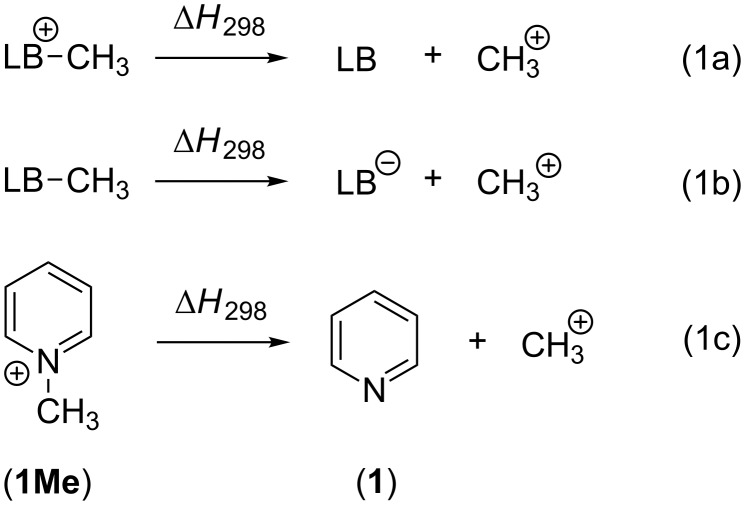
Reactions for the methyl cation affinity (MCA) of a neutral Lewis base (1a), an anionic Lewis base (1b) and pyridine (1c).

The methyl cation (CH_3_^+^) is the smallest carbocation which is useful as a chemical probe for Lewis bases. The respective methyl cation affinity of a given Lewis base (LB) is obtained as the reaction enthalpy at 298.15 K and 1 bar pressure for the reaction shown in equation 1a for a neutral Lewis base and in equation 1b for an anionic base (Scheme 1). This definition is in analogy to that for proton affinities (PA) and implies large positive energies for most of the P- and N-based Lewis bases used in catalytic processes. Using pyridine (**1**) as an example for a weak Lewis base, the methyl cation affinity corresponds to the enthalpy of the reaction in equation 1c and amounts to MCA(**1**) = +519.2 kJ/mol at the G3 level of theory [5].

A recent analysis of theoretical methods found that calculations at the MP2(FC)/6-31+G(2d,p)//B98/6-31G(d) level of theory (in short: "MP2-5") reproduce results obtained at the G3 level within 4.0 kJ/mol for selected small and medium-sized organocatalysts [5]. For pyridine (**1**) the MCA value obtained with this model amounts to MCA(**1**) = +518.7 kJ/mol, which is only 0.5 kJ/mol lower than the G3 value. The following discussion will thus be based on results obtained with the MP2-5 model, if not noted otherwise. Methyl cation affinity values obtained for N-centered Lewis bases using this approach are collected in Table 1. For organocatalytic processes especially the Lewis bases **12**, **14**, **18**, **24**, **44**, **45** and **52**-**65** are of note.

**Table 1:** MCA values for N-centered Lewis bases, ordered by increasing MCA values.

system	MCA [kJ/mol]	system	MCA [kJ/mol]

NH_3_ (**2**)	+436.5	NH_2_Me (**3**)	+488.7
NMePh_2_ (**4**)	+514.4	pyridine (**1**)	+518.7
N*c*-Pr_3_ (**5**)	+521.2	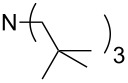 (**6**)	+521.4
NHMe_2_ (**7**)	+523.1	NMe*c*-Pr_2_ (**8**)	+523.7
NMe_2_Ph (**9**)	+527.7	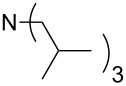 (**10**)	+529.4
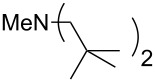 (**11**)	+531.1	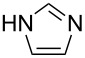 (**12**)	+531.7^a^
NMe_2_*c*-Pr (**13**)	+532.0	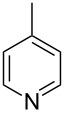 (**14**)	+532.8
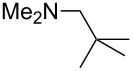 (**15**)	+535.9	N(iPr)_3_ (**16**)	+536.0
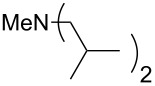 (**17**)	+538.2	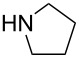 (**18**)	+539.8
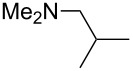 (**19**)	+541.5	NMe_3_ (**20**)	+543.5
N*t*-Bu_3_ (**21**)	+545.5	NMe_2_Et (**22**)	+548.6
NMe*t*-Bu_2_ (**23**)	+549.4	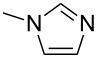 (**24**)	+550.0
NMe_2_*c*-Bu (**25**)	+551.2	NMe_2_(iPr) (**26**)	+551.7
NMe_2_*n*-Pr (**27**)	+552.1	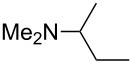 (**28**)	+552.5
NMe_2_*t*-Bu (**29**)	+552.6	NMe_2_*n*-Bu (**30**)	+553.8
NMe_2_*c*-Pen (**31**)	+554.3	NMe_2_*n*-Pen (**32**)	+554.5
NMe_2_*c*-Oct (**33**)	+554.6	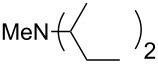 (**34**)	+555.2
NMeEt_2_ (**35**)	+555.3	NMe_2_*n*-Hex (**36**)	+555.4
NMe_2_*n*-Hep (**37**)	+555.7	N*c*-Hex_3_ (**38**)	+556.9
NMe(iPr)_2_ (**39**)	+557.3	NMe_2_*c*-Hep (**40**)	+560.3
NMe*c*-Bu_2_ (**41**)	+560.4	NMe_2_*c*-Hex (**42**)	+561.1
NMe*n*-Pr_2_ (**43**)	+561.7	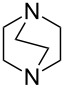 (**44**)	+562.2
NEt_3_ (**45**)	+562.3	NMe*n*-Bu_2_ (**46**)	+564.1
N*n*-Pr_3_ (**47**)	+567.5	N*c*-Pen_3_ (**48**)	+568.3
NMe*c*-Pen_2_ (**49**)	+570.6	N*c*-Bu_3_ (**50**)	+570.9
NMe*c*-Hex_2_ (**51**)	+572.0	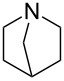 (**52**)	+576.0
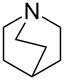 (**53**)	+580.6	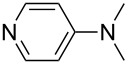 (**54**)	+581.2^b^
NPh_3_ (**55**)	+583.5	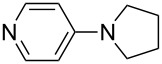 (**56**)	+590.1^b^
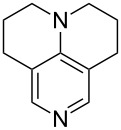 (**57**)	+602.7	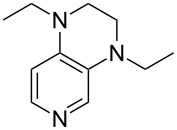 (**58**)	+609.0
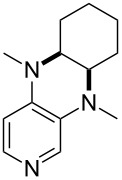 (**59**)	+609.1	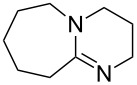 (**60**)	+609.6^c^
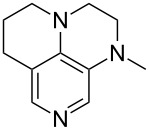 (**61**)	+611.0	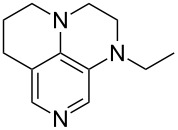 (**62**)	+613.3
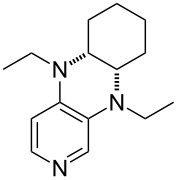 (**63**)	+616.0	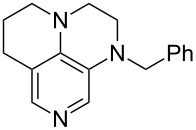 (**64**)	+620.8
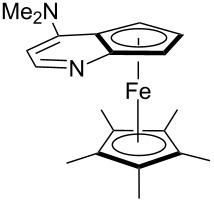 (**65**)	+624.1^b,d^		

^a^N3; ^b^pyridine nitrogen; ^c^N(sp²); ^d^see [7].

Pyridine is a comparatively weak nucleophile as already mentioned above. This also applies to imidazole (**12**), pyrrolidine (**18**) and a number of trialkylamines, all of which have MCA values below 550 kJ/mol. In the case of pyridine it is possible to increase the Lewis basicity by introducing electron-donating groups in *para*-position. The dialkylamino groups in 4-*N*,*N*-dimethylaminopyridine (DMAP, **54**) or in 4-pyrrolidinopyridine (PPY, **56**) increase the MCA values dramatically. This is in accordance with the much higher catalytic efficiency of **54** and **56** for e.g., acylation reactions [3,8–12]. The currently highest MCA value has been obtained for ferrocenyl DMAP-derivative **65** with MCA(**65**) = +624.1 kJ/mol [7]. This is approximately 40 kJ/mol more than the value for DMAP with MCA(**54**) = +581.2 kJ/mol and may be the reason for the outstanding catalytic potential of **65**. For the chiral Lewis bases **59**, **63**, and **65** only one enantiomer is listed in Table 1. Affinity values towards achiral electrophiles such as the MCA values collected in Table 1 are, of course, exactly identical for both enantiomers, and therefore we will in the following report affinity values for only one of the enantiomers of a given chiral Lewis base.

**Figure 1:**
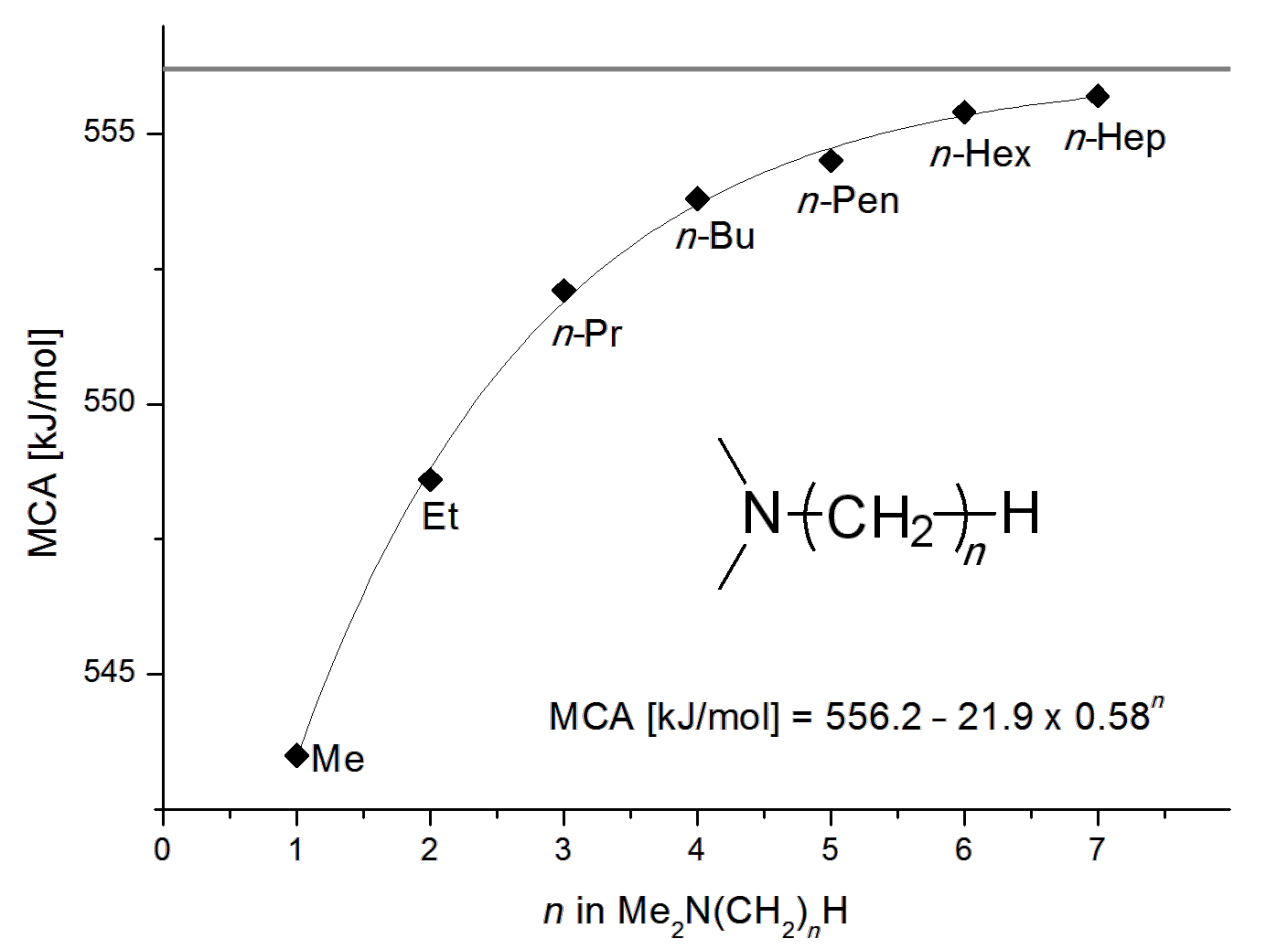
MCA values of monosubstituted amines of general formula Me_2_N(CH_2_)*_n_*H (*n* = 1–7, in kJ/mol).

**Scheme 2:**
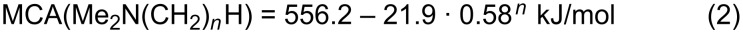
Systematic dependence of MCA.

The MCA values of trialkylamines depend in a systematic manner on the number and structure of the attached alkyl groups. The influence of the length of linear alkyl groups has been explored using alkyldimethylamines. As can be seen in Figure 1 the MCA values of these bases depend in an exponential manner on the length of the alkyl group. This systematic dependence can be expressed quantitatively by the equation given in Scheme 2.

**Scheme 3:**
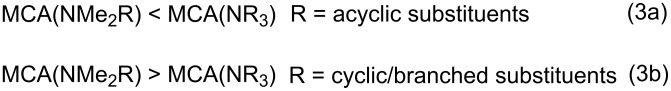
Trends in amine MCA values.

This relationship predicts a limiting MCA value of 556.2 kJ/mol for alkyldimethylamines with an alkyl substituent of infinite length. This is an increase of 12.7 kJ/mol compared to trimethylamine. In amines with three identical substituents – such as N*n*-Pr_3_ (**47**) with MCA(**47**) = 567.5 kJ/mol – the electron-donation effects induced by the alkyl groups are close to additive for linear alkyl chains, thus leading to systematically higher affinity values as compared to the respective mono-substituted amine (e.g., NMe_2_*n*-Pr (**27)** with MCA(**27**) = 552.1 kJ/mol). However, even in systems with linear alkyl substituents unfavorable steric effects appear to exist between the alkyl substituents, and confining the alkyl groups to a bicyclic cage structure as in quinuclidine (**53**) thus raises the MCA value considerably to MCA(**53**) = +580.6 kJ/mol. For amines with branched or cyclic substituents a further erosion of MCA values can be observed due to increasing steric effects in the methyl cation adducts. The following trends in amine MCA values can therefore be observed for a variety of systems (Scheme 3).

**Figure 2:**
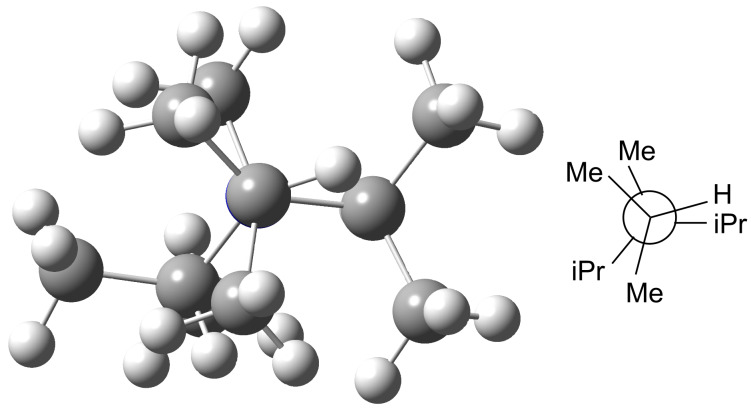
Eclipsing interactions in the best conformation of N^+^Me(iPr)_3_ (**16Me**) (left), and the corresponding Newman projection through the C–N bond (right).

For branched and cyclic substituents additional unfavorable steric interactions come into play, for the cationic methyl-adducts more than for the parent amines. In order to illustrate these effects a closer look at the best conformations of the simple most branched amines Me_2_N(iPr) (**26**, MCA = 551.7 kJ/mol), MeN(iPr)_2_ (**39**, MCA = 557.3 kJ/mol) and N(iPr)_3_ (**16**, MCA = 536.0 kJ/mol) is helpful. In the absence of steric effects a systematic increase in the MCA value is expected on replacing methyl by isopropyl substituents. However, the number of gauche interactions increases more rapidly for the methyl cation adducts than for the parent amines; this leads to an increase which is smaller than expected. For amine **26** (Me_2_N(iPr)) there are two gauche interactions in the neutral parent and four such interactions in the respective methyl cation adduct. For amine **39** (MeN(iPr)_2_) the number of unfavorable interactions increases to five in the neutral parent and to eight in the methyl cation adduct. The unexpectedly low MCA value for amine **16** (N(iPr)_3_) is a consequence of additional, more strongly repulsive *syn*-pentane interactions, whose magnitude is larger in the methyl cation adduct than in the neutral amine. Figure 2 shows the projection through the C–N bond of one of the isopropyl-groups in **16Me**.

Trialkyl- and triarylphosphanes are equally potent nucleophiles, whose use in catalytic processes is, however, often limited due to their oxygen sensitivity. Table 2 lists MCA values for a large number of trialkylphosphanes and alkyldiphenylphosphanes. For organocatalytic processes especially the phosphanes **89**, **98**, **117**, **120**–**124** are of note.

**Table 2:** MCA values of saturated trialkylphosphanes without heteroatoms, PPh_3_, PH_3_, PH_2_Me and PHMe_2_, ordered by increasing MCA values.

system	MCA [kJ/mol]	system	MCA [kJ/mol]

PH_3_ (**66**)	+448.4	PH_2_Me (**67**)	+513.0
PHMe_2_ (**68**)	+564.2	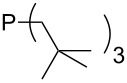 (**69**)	+603.3
PMe_3_ (**70**)	+604.2	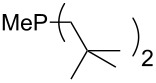 (**71**)	+606.9
Me_2_*c*-Pr (**72**)	+607.2	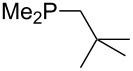 (**73**)	+607.9
PMe_2_Et (**74**)	+610.5	Me*c*-Pr_2_ (**75**)	+611.8
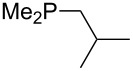 (**76**)	+611.9	PMe_2_(iPr) (**77**)	+613.5
PMe_2_*n*-Pr (**78**)	+614.3	PMeEt_2_ (**79**)	+616.1
PMe_2_*n*-Bu (**80**)	+616.3	PMe_2_*c*-Bu (**81**)	+616.7
PMe_2_*n*-Pen (**82**)	+617.3	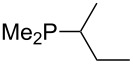 (**83**)	+617.3
PMe_2_*n*-Hex (**84**)	+617.7	PMe_2_*n*-Hep (**85**)	+617.9
PMe_2_*n*-Oct (**86**)	+618.1	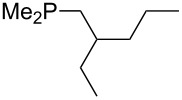 (**87**)	+618.2.
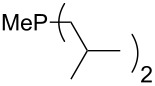 (**88**)	+618.5	PPh_3_ (**89**)	+618.7
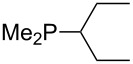 (**90**)	+618.8	PMe_2_*t*-Bu (**91**)	+619.4
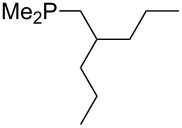 (**92**)	+619.5	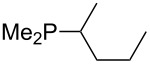 (**93**)	+619.6
PMe_2_*c*-Pen (**94**)	+620.4	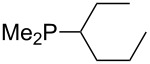 (**95**)	+620.8
P*c*-Pr_3_ (**96**)	+621.1	PMe_2_*c*-Hex (**97**)	+621.9
PEt_3_ (**98**)	+622.5	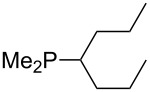 (**99**)	+623.3
PMe*n*-Pr_2_ (**100**)	+624.1	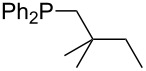 (**101**)	+624.5
PPh_2_*n*-Bu (**102**)	+624.6	PMe(iPr)_2_ (**103**)	+624.8
PMe_2_*c*-Hep (**104**)	+624.9	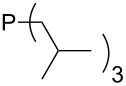 (**105**)	+625.7
PMe_2_*c*-Oct (**106**)	+626.1	PMe_2_*c*-Non (**107**)	+627.5
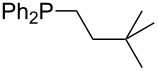 (**108**)	+627.7	PMe*n*-Bu_2_ (**109**)	+627.8
PMe*c*-Bu_2_ (**110**)	+628.7	PMe_2_*c*-Dec (**111**)	+628.9
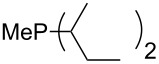 (**112**)	+630.5	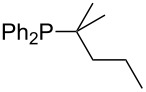 (**113**)	+631.7
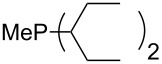 (**114**)	+631.8	P*n*-Pr_3_ (**115**)	+633.6
PMe*t*-Bu_2_ (**116**)	+635.1	P(iPr)_3_ (**117**)	+635.4
PMe*c*-Pen_2_ (**118**)	+637.1	P*c*-Bu_3_ (**119**)	+638.5
P*n*-Bu_3_ (**120**)	+639.5	PMe*c*-Hex_2_ (**121**)	+641.0
P*t*-Bu_3_ (**122**)	+648.3	P*c*-Pen_3_ (**123**)	+650.8
P*c*-Hex_3_ (**124**)	+655.7		

**Scheme 4:**
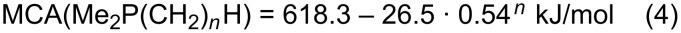
General expression for the chain-length dependence of MCA values.

**Figure 3:**
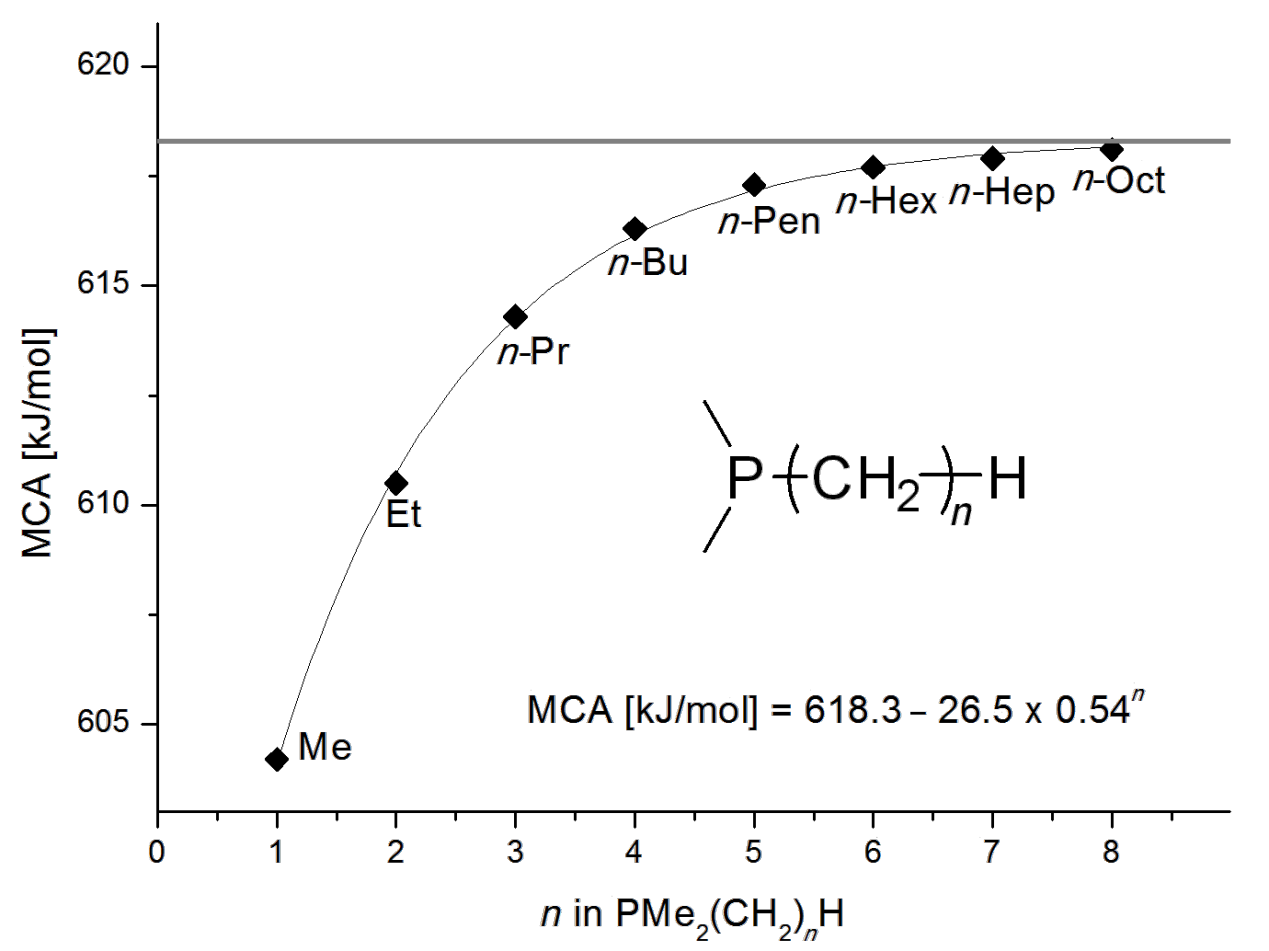
MCA values of monosubstituted phosphanes of general formula Me_2_P(CH_2_)*_n_*H (*n* = 1–8, in kJ/mol).

Analysis of the results for unbranched trialkylphosphanes indicates that longer alkyl chains increase the MCA values in a systematic manner. This was also found for trialkylamines and reflects inductive electron donation through alkyl substituents with variable length [13]. The results obtained for dimethylalkylphosphanes of general structure Me_2_P(CH_2_)*_n_*H with *n* = 1–8 lead to a general expression for the chain-length dependence of the MCA values that can again be derived as given in equation 4 (Scheme 4). This is shown together with the respective data points in Figure 3.

**Figure 4:**
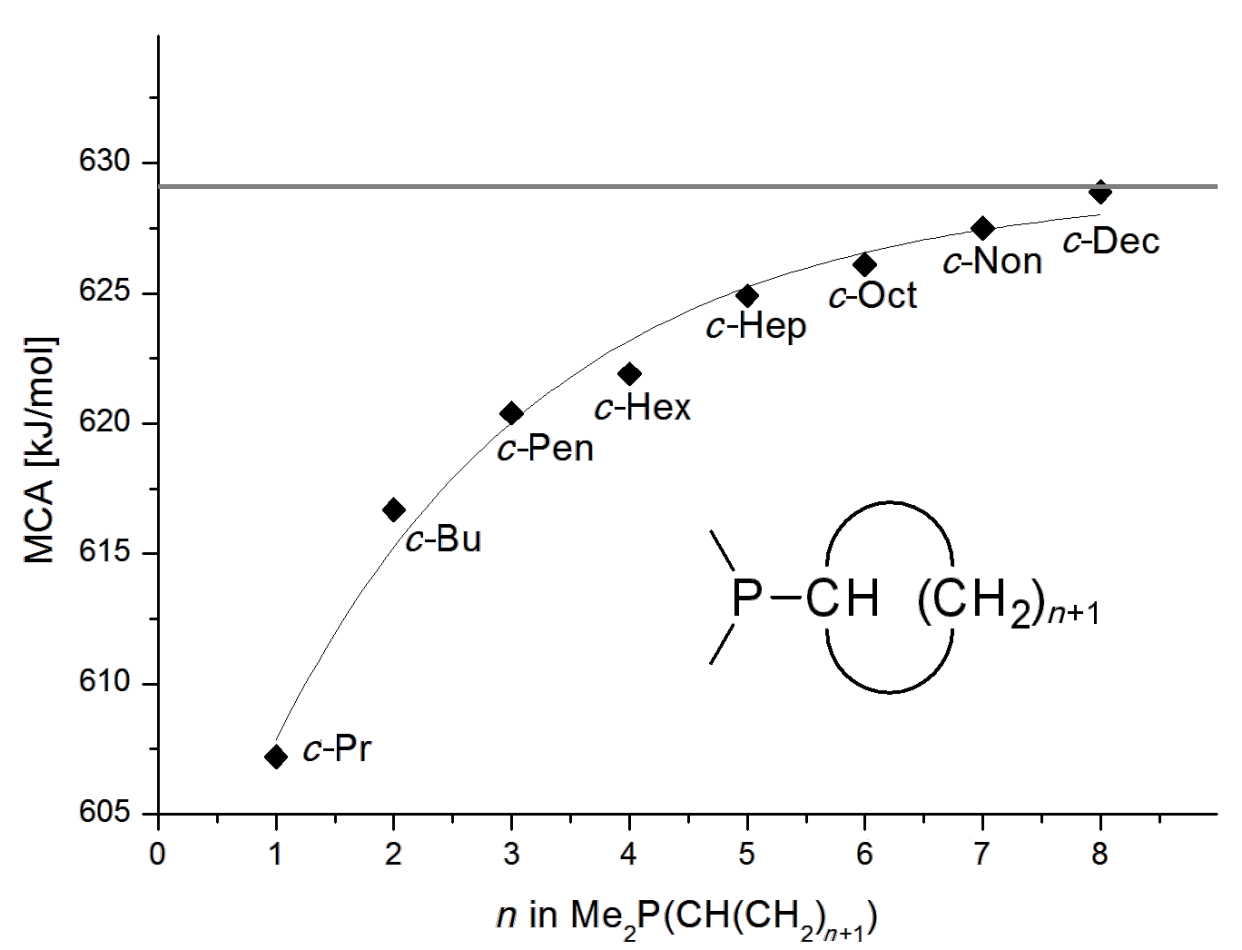
MCA values of monosubstituted phosphanes of general formula PMe_2_(CH(CH_2_)*_n_*_+1_) (*n* = 1–8, in kJ/mol).

On the basis of equation 4 it is possible to predict the MCA value for a phosphane Me_2_PR with one infinitively long alkyl substituent (MCA = 618.3 kJ/mol) and for a phosphane PR_3_ with three infinitively long alkyl substituents (MCA = 646.5 kJ/mol). Phosphanes with branched alkyl substituents show systematically larger MCA values as compared to unbranched systems of otherwise comparable structure. An α-branched substituent leads to a higher MCA value than a β-branched substituent; this can be illustrated with the phosphanes **76** and **83**. Both phosphanes are dimethyl(methylpropyl)phosphanes, but one in α-position (**83**, MCA = 617.3 kJ/mol) and one in β-position (**76**, MCA = 611.9 kJ/mol). Deviations from the regular behavior of the MCA values are due to steric interactions, mostly 1,5-*syn*-pentane interactions. The MCA values obtained for phosphanes with cycloalkyl substituents show similar trends as already observed for acyclic systems in that larger rings lead to higher MCA values. However, as shown in Figure 4 for phosphanes carrying one cyclic substituent, the correlation between ring size and MCA value is not quite as good as found for phosphanes with acyclic substituents.

**Figure 5:**
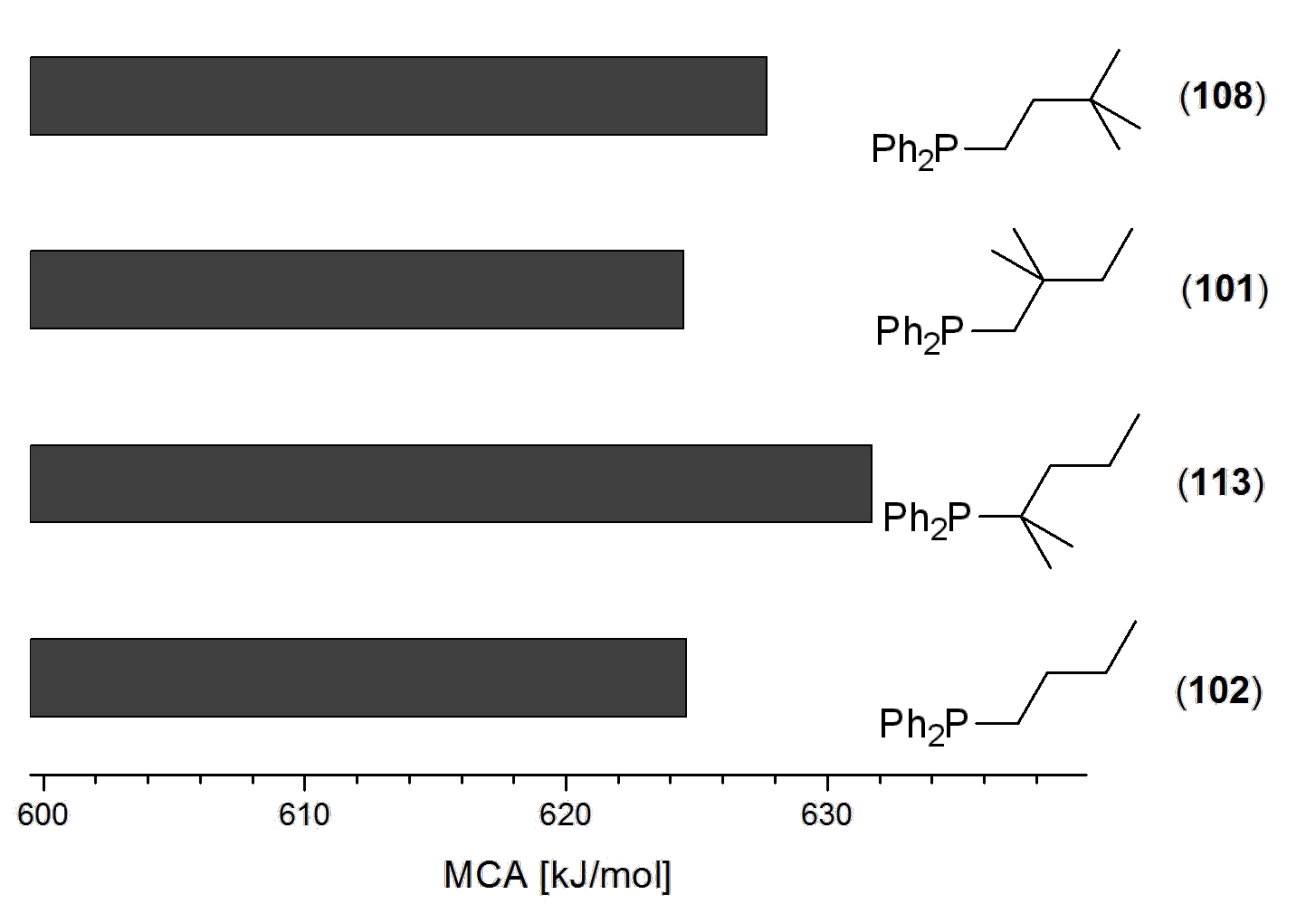
The MCA values of *n-*butyldiphenylphosphane (**102**) and its (αα-/ββ-/γγ-) dimethylated analogues.

A similar analysis applies to phosphanes combining one alkyl and two phenyl substituents with the general formula PPh_2_R. The results of phosphanes **101**, **102**, **108**, **113** are particularly interesting, because they contain an *n*-butyl substituent decorated with additional methyl groups in varying positions (Figure 5). With two methyl groups in α-position the MCA value is increased by 7 kJ/mol compared to the phosphane without methyl groups. In contrast, the positive inductive influence of two methyl groups in β-position is balanced by disfavorable steric interactions, which are similar to 1,5-*syn*-pentane interactions. But the methyl groups in γ-position lead again to a slight rise of the MCA value. The latter increase is just about 3 kJ/mol, because the γ-position is quite far away from the reaction center. Therefore, it can be summarized that branched phosphanes are most sensitive for disfavorable steric interactions when branching occurs in β-position.

Large MCA values can be expected for phosphanes and amines which carry substituents that are able to act as lone pair donors. This was explored for phosphanes which possess a nitrogen-containing moiety. Table 3 lists results for phosphanes containing a direct phosphorus–nitrogen bond. In the case of phosphanes with a second nucleophilic position (e.g., nitrogen atom) all MCA values are calculated for the reaction at the phosphorus atom, if not mentioned otherwise.

**Table 3:** MCA values of phosphanes containing one to three P–N bonds, ordered by increasing MCA values.

system	MCA [kJ/mol]	system	MCA [kJ/mol]

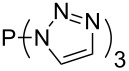 (**125**)	+418.4	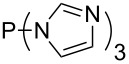 (**126**)	+451.3
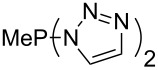 (**127**)	+466.1	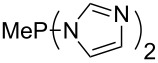 (**128**)	+487.0
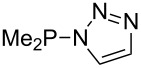 (**129**)	+523.3	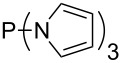 (**130**)	+524.6
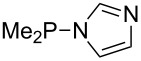 (**131**)	+535.5	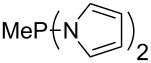 (**132**)	+541.2
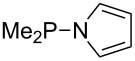 (**133**)	+563.8	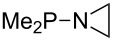 (**134**)	+605.4
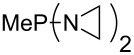 (**135**)	+611.9	Me_2_P–NMe_2_ (**136**)	+615.5
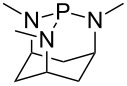 (**137**)	+620.0	Me_2_P–NMeEt (**138**)	+620.6
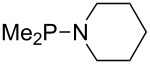 (**139**)	+624.7	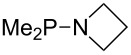 (**140**)	+624.8
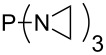 (**141**)	+626.8	Me_2_P–NEt_2_ (**142**)	+626.8
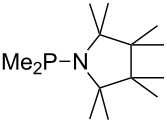 (**143**)	+626.9	Me_2_P–NEtPr (**144**)	+627.7
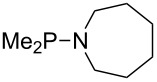 (**145**)	+629.6	Me_2_P–NPr_2_ (**146**)	+629.8
MeP(NMe_2_)_2_ (**147**)	+631.8	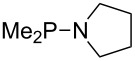 (**148**)	+634.2
P(NMe_2_)_3_ (**149**)	+642.3	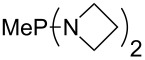 (**150**)	+642.7
MeP(NMeEt)_2_ (**151**)	+642.7	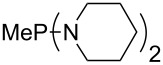 (**152**)	+648.7
MeP(NEt_2_)_2_ (**153**)	+653.2	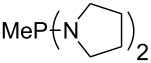 (**154**)	+661.6
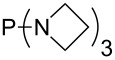 (**155**)	+666.4	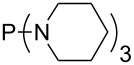 (**156**)	+666.5
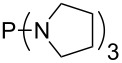 (**157**)	+686.6	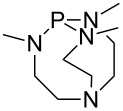 (**158**)	+701.1

**Figure 6:**
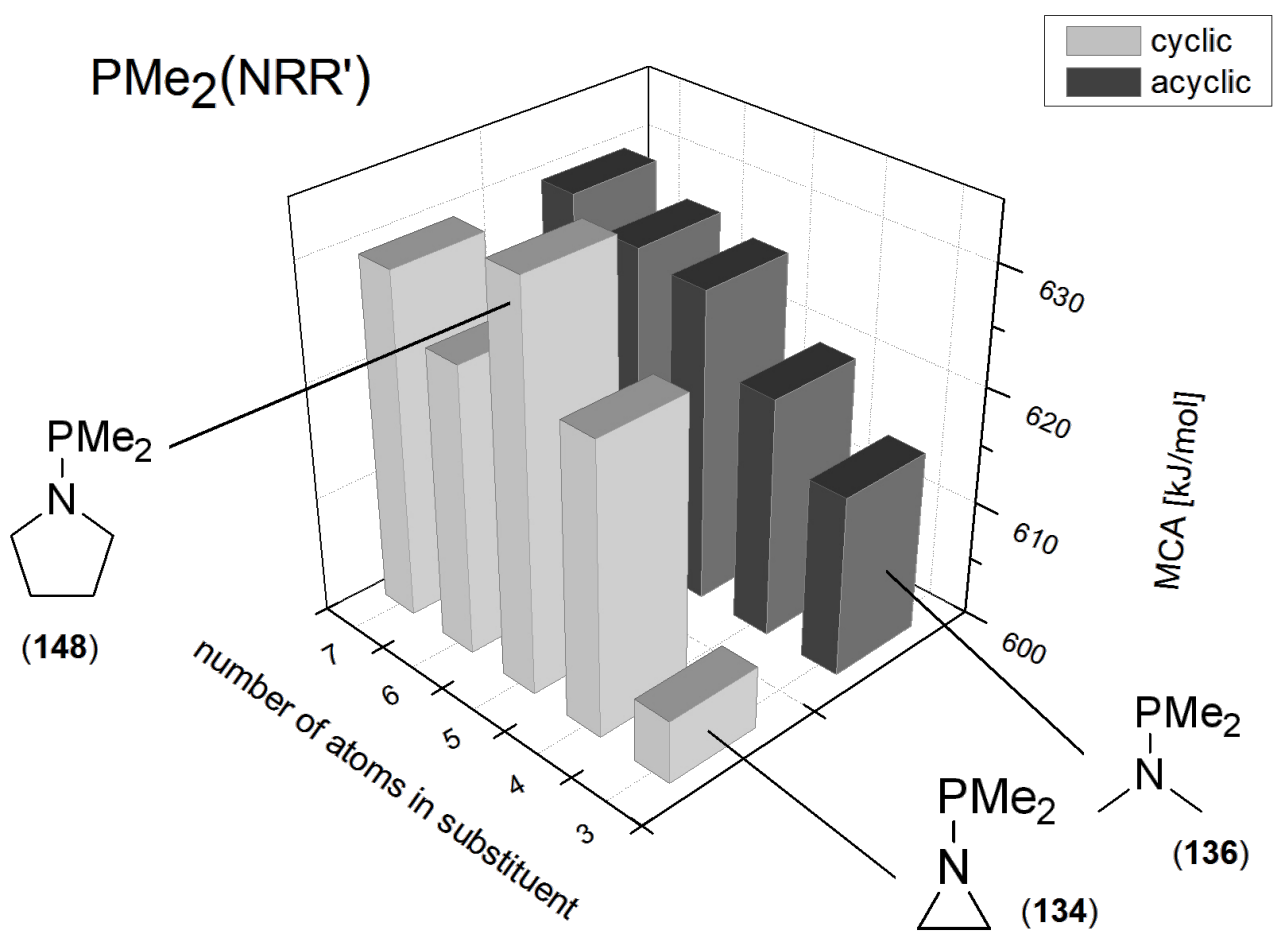
MCA values of phosphanes Me_2_P–NR_2_ with cyclic and acyclic amine substituents.

The largest increase in MCA value compared to the reference of trimethylphosphane (**70**) is obtained through the introduction of the pyrrolidino substituent present in phosphanes **148**, **154** and **157**. Introduction of the first pyrrolidino group as in phosphane **148** leads to MCA(**148**) = +634.2 kJ/mol, an increase of 30.0 kJ/mol compared to the reference systems PMe_3_ (**70**). Despite the fact that the MCA value increases somewhat more slowly on introduction of the second and third pyrrolidino substituent, the tris(pyrrolidino)phosphane **157** counts among the most Lewis-basic systems studied here with MCA(**157**) = +686.6 kJ/mol except the bicyclic, unusual phosphane **158**. For systems of general formula PMe_2_NRR’ the unsaturated substituents (pyrrole (**133**), imidazol (**131**), triazol (**129**)) show MCA values below 570 kJ/mol, whereas the saturated substituents show MCA values above 600 kJ/mol. In the case of saturated, acyclic substituents the trend ‘longer alkyl chains – higher MCA values’ is again observed (Figure 6). For saturated, cyclic substituents, however, the MCA values increase from aziridine (**134**) to azetidine (**140**) and pyrrolidine (**148**), but then decrease again for piperidine (**139**) and azepane (**145**).

Aside from phosphanes with a direct phosphorus–nitrogen bond, a variety of phosphanes with nitrogen-containing substituents can be envisioned in which P- and N-centers are separated by at least one carbon atom. Results for these phosphanes are listed in Table 4. All MCA values are calculated for reaction at the phosphorus atom, if not mentioned otherwise.

**Table 4:** MCA values of phosphanes with nitrogen-containing substituents lacking direct phosphorous–nitrogen bonds.

system	MCA [kJ/mol]	system	MCA [kJ/mol]

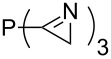 (**159**)	+431.3	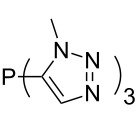 (**160**)	+470.2
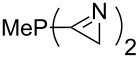 (**161**)	+480.1	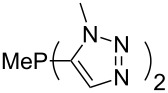 (**162**)	+510.5
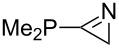 (**163**)	+539.2	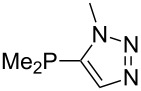 (**164**)	+548.8
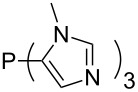 (**165**)	+556.6	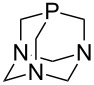 (**166**)	+559.5
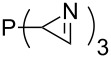 (**167**)	+565.1	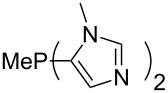 (**168**)	+571.0
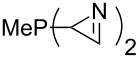 (**169**)	+571.7	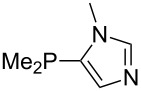 (**170**)	+582.0
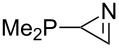 (171)	+588.4	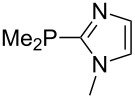 (**172**)	+591.4
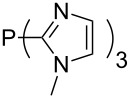 (**173**)	+594.4	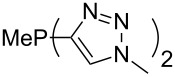 (**174**)	+596.1
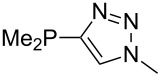 (**175**)	+596.7	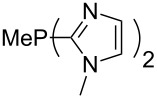 (**176**)	+601.1
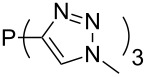 (**177**)	+603.5	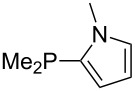 (**178**)	+605.6
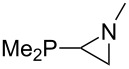 (**179**)	+610.7	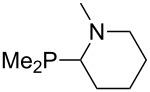 (**180**)	+614.9
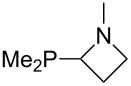 (**181**)	+615.1	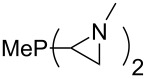 (**182**)	+616.2
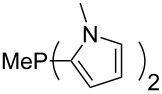 (**183**)	+618.1	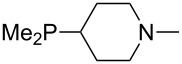 (**184**)	+619.2
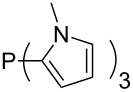 (**185**)	+620.2	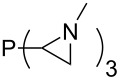 (**186**)	+621.1
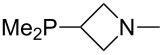 (**187**)	+622.4	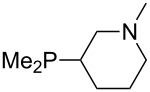 (**188**)	+623.9
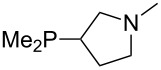 (**189**)	+625.9	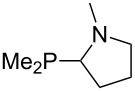 (**190**)	+626.0
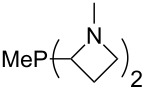 (**191**)	+626.1	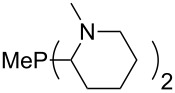 (**192**)	+627.2
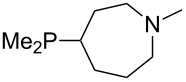 (**193**)	+627.8	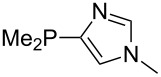 (**194**)	+628.7
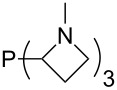 (**195**)	+632.0	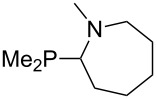 (**196**)	+633.0
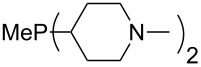 (**197**)	+634.7	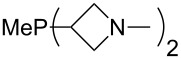 (**198**)	+635.7
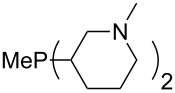 (**199**)	+636.1	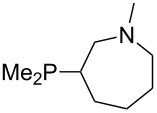 (**200**)	+636.7
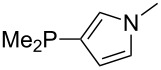 (**201**)	+638.2	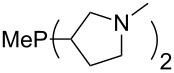 (**202**)	+644.4
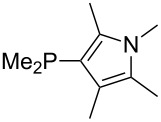 (**203**)	+645.3	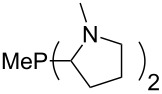 (**204**)	+645.8
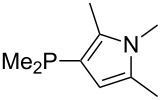 (**205**)	+646.4	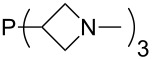 (**206**)	+650.0
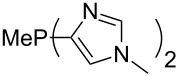 (**207**)	+656.1	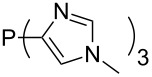 (**208**)	+672.4
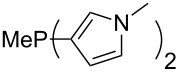 (**209**)	+673.5	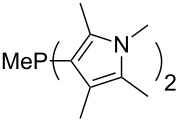 (**210**)	+688.8
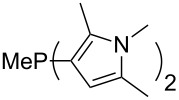 (**211**)	+694.3	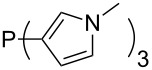 (**212**)	+702.9
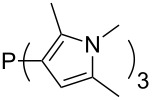 (**213**)	+722.3	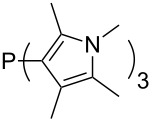 (**214**)	+726.8

**Figure 7:**
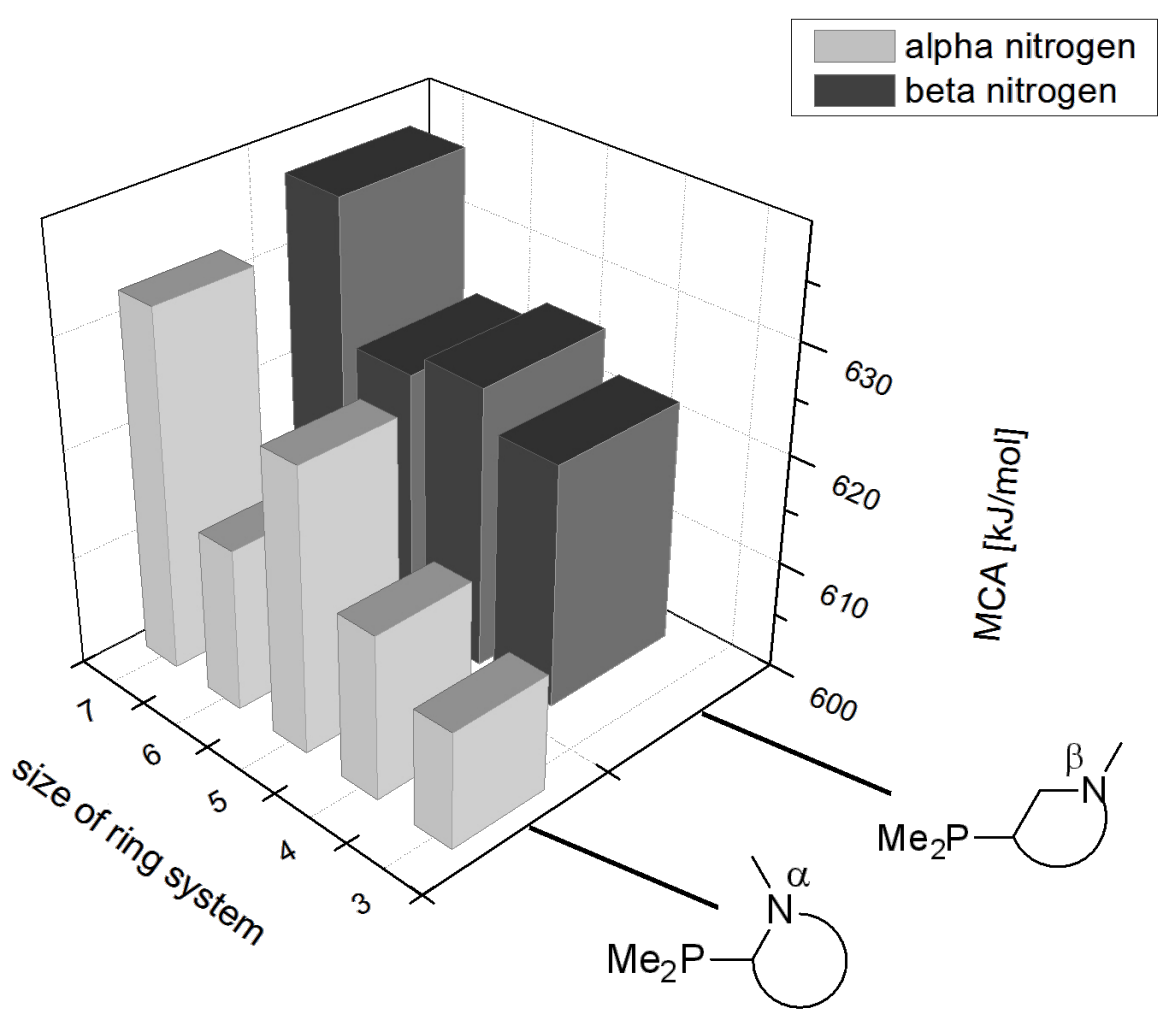
MCA values of phosphanes PMe_2_R connected to α,α- and β,β-position of nitrogen containing cyclic substituents.

For saturated substituents a general trend of higher MCA values with larger cyclic substituents can be observed. It is also visible that the β-connectivity usually leads to higher MCA values than the α-connectivity (Figure 7).

Most of the unsaturated substituents as 1-methyltriazoles (**160**, **162**, **164**, **174**, **175**, **177**) and 2-/5-(1-methylimidazoles) (**165**, **168**, **170**, **172**, **173**, **176**) lead to lower MCA values compared to the reference system PMe_3_ (**70**) due to their electron withdrawing character. The 4-(1-methylimidazole) substituents present in phosphanes **194**, **207**, and **208** are, however, electron donating. The most effective electron-donating effects are in this group found for methylated pyrrole substituents (**201**, **203**, **205**, **209**–**214**), particularly in systems connecting the phosphorous atom to the C3 position of the pyrrole ring. In these cases, the trisubstituted phosphanes reach MCA values above 720 kJ/mol. This is an extraordinary MCA value for neutral Lewis bases.

Phosphanes with cyclic substituents containing heteroatoms such as oxygen or sulfur are not quite as Lewis basic as the nitrogen-containing analogs (Table 5). All MCA values refer to reaction at the phosphorus atom, if not mentioned otherwise.

**Table 5:** MCA values of phosphanes with oxygen- and sulfur-containing cyclic substituents.

system	MCA [kJ/mol]	system	MCA [kJ/mol]

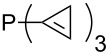 (**215**)	+559.4	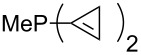 (**216**)	+570.5
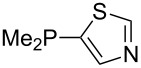 (**217**)	+572.1	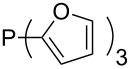 (**218**)	+576.6
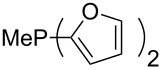 (**219**)	+578.2	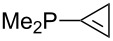 (**220**)	+585.8
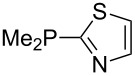 (**221**)	+586.4	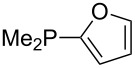 (**222**)	+588.2
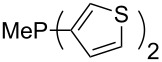 (**223**)	+595.1	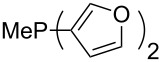 (**224**)	+595.3
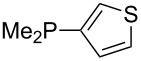 (**225**)	+596.0	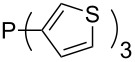 (**226**)	+596.2
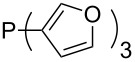 (**227**)	+596.7	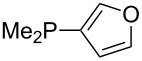 (**228**)	+596.8
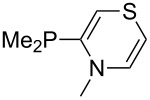 (**229**)	+598.1	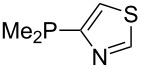 (**230**)	+598.5
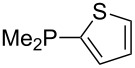 (**231**)	+606.0	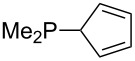 (**232**)	+607.8
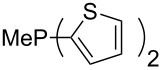 (**233**)	+608.5	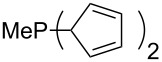 (**234**)	+610.1
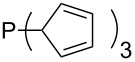 (**235**)	+611.0	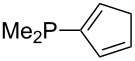 (**236**)	+611.5
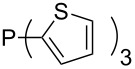 (**237**)	+612.9	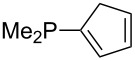 (**238**)	+613.9
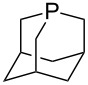 (**239**)	+613.9	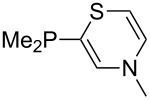 (**240**)	+614.1
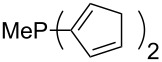 (**241**)	+617.1	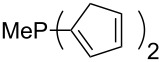 (**242**)	+618.7
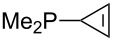 (**243**)	+619.4	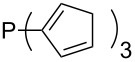 (**244**)	+626.3
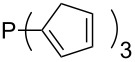 (**245**)	+633.9	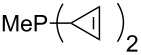 (**246**)	+634.5
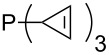 (**247**)	+647.3		

Surveying the MCA values obtained for phosphanes with furane (**218**, **219**, **222**, **224**, **227**, **228**), thiophene (**223**, **225**, **226**, **231**, **233**, **237**), cyclopentadiene (**232**, **234**–**236**, **238**, **241**, **242**, **244**, **245**), thiazole (**217**, **221**, **230**), and thiazine (**229**, **240**) substituents clearly illustrates that these substitution patterns lead to low or moderate MCA values.

Phosphanes with aromatic substituents are expected to display largely different MCA values depending on the functionalization pattern of these substituents. Results for this group of phosphanes are presented in Table 6 in which most of the phosphanes are interesting for organocatalysis. Again, all MCA values are calculated for reaction at the phosphorus atom if not mentioned otherwise.

**Table 6:** MCA values of aryl-substituted phosphanes.

system	MCA [kJ/mol]	system	MCA [kJ/mol]

PMe_3_O (**248**)	+463.5^a^	P(C_6_F_5_)_3_ (**249**)	+494.1
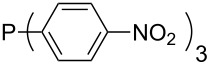 (**250**)	+526.6	Ph_2_P–NEt_2_ (**251**)	+545.5^b^
P(OPh)_3_ (**252**)	+575.7	PPh_2_H (**253**)	+577.2
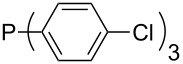 (**254**)	+586.5	P(OEt)_3_ (**255**)	+599.9
PPhMe_2_ (**256**)	+608.5	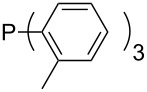 (**257**)	+610.7
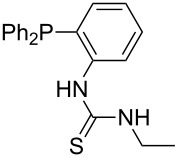 (**258**)	+612.3	PPh_2_Me (**259**)	+614.1
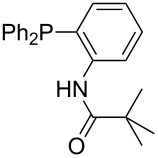 (**260**)	+616.6	PPhEt_2_ (**261**)	+617.8
PPh_3_ (**89**)	+618.7	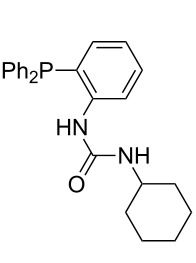 (**262**)	+619.7
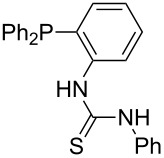 (**263**)	+619.8	PPh_2_Et (**264**)	+620.0
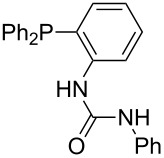 (**265**)	+620.6	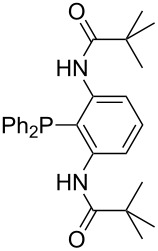 (**266**)	+621.3
PPh_2_*c*-Pr (**267**)	+622.4	PPh_2_(iPr) (**268**)	+623.0
PPh*n*-Pr_2_ (**269**)	+623.5	PPh_2_*n*-Pr (**270**)	+623.6
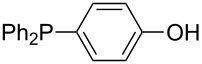 (**271**)	+624.2	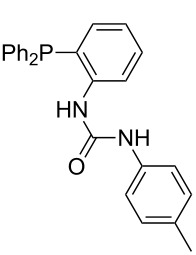 (**272**)	+624.2
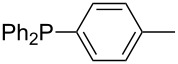 (**273**)	+624.9	Ph_2_P–NMe_2_ (**274**)	+624.9
PPh_2_*n*-Pen (**275**)	+625.3	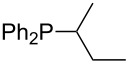 (**276**)	+625.8
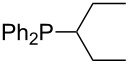 (**277**)	+625.8	PPh_2_*c*-Bu (**278**)	+626.1
PPh(iPr)_2_ (**279**)	+627.0	PPh*n*-Bu_2_ (**280**)	+627.7
PPh_2_*t*-Bu (**281**)	+628.5	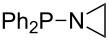 (**282**)	+628.6
Ph_2_P–NMeEt (**283**)	+629.0	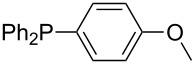 (**284**)	+629.8
PPh_2_*c*-Hex (**285**)	+630.2	PPh_2_*c*-Pen (**286**)	+630.5
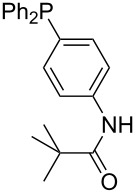 (**287**)	+630.6	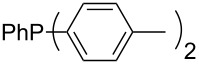 (**288**)	+630.8
PPh_2_*c*-Hep (**289**)	+631.8	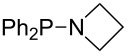 (**290**)	+632.3
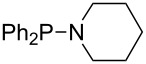 (**291**)	+632.3	PPh_2_*c*-Oct (**292**)	+633.1
Ph_2_P–NEt_2_ (**252**)	+634.3^c^	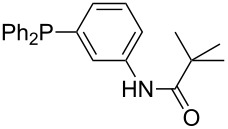 (**293**)	+634.4
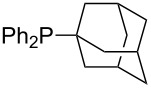 (**294**)	+636.4	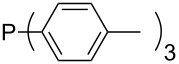 (**295**)	+636.9
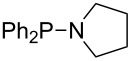 (**296**)	+638.6	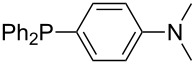 (**297**)	+646.7
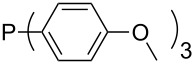 (**298**)	+651.0	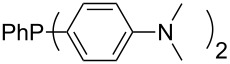 (**299**)	+673.1
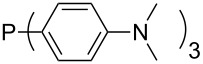 (**300**)	+694.9	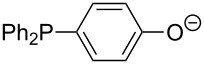 (**301**)	+923.4
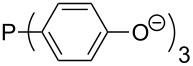 (**302**)	+1490.5		

^a^MCA value at oxygen; ^b^MCA value at nitrogen; ^c^MCA value at phosphorus.

In order to discuss inductive and mesomeric electron-donating effects the tri-*para*-substituted triphenylphosphanes (**295**, **298**, **300**) were chosen. The methyl group as the simplest example for an electron-donating substituent raises the MCA value by about 6 kJ/mol per group. The mesomeric effects of the methoxy- and dimethylamino-groups are significantly larger at about 11 and 25 kJ/mol per substituent. Beside the strong neutral dimethylamino group as electronic donating group also anionic substituents could be worthwhile. Phosphanes **301** and **302** have the highest MCA values of all Lewis bases considered here. One phenolate instead of a phenyl substituent increases the MCA value by approx. 300 kJ/mol.

Triarylphosphanes can be of interest for organocatalysis if at least one substituent in *ortho*-position of a phenyl group enables hydrogen bonds to the substrate as is, for example, the case in phosphanes **260**, **287,** and **293**. These latter three compounds differ only in the position of the pivaloylamido group (α, β, γ). The resulting MCA values vary by about 18 kJ/mol. Besides these amide-containing phosphanes (thio-)urea-containing phosphanes were also investigated. They possess lower MCA values than the previously discussed ones. In general, the ‘thiourea-phosphanes’ (**258**, **263**) show even lower MCA values than the ‘urea-phosphanes’ (**262**, **265**, **272**).

Can MCA values be increased through integration of the P-atom into a ring system? With respect to the results obtained for a small set of cyclic phosphanes (Table 7) it appears that there is at least no general trend for cyclic and acyclic phosphanes of otherwise comparable substitution pattern. The combination of phosphanes with unusually strained cyclic substituents such as diamandoids or cyclophanes also appears to have no particularly unusual effects. Phosphanes with strongly electron-withdrawing substituents such as PF_3_ (**303**) have the expected low MCA values, while exceedingly high cation affinties are found for phosphoranylideneamines. In the case of phosphanes with a second nucleophilic position (e.g., oxygen atoms) all MCA values are calculated for the phosphorus atom if not mentioned otherwise.

**Table 7:** MCA values of miscellaneous phosphanes, ordered by increasing MCA values.

system	MCA [kJ/mol]	system	MCA [kJ/mol]

PF_3_ (**303**)	+356.0	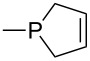 (**304**)	+584.0
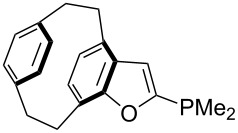 (**305**)	+591.3	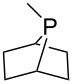 (**306**)	+598.5
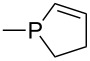 (**307**)	+599.6	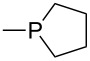 (**308**)	+602.0
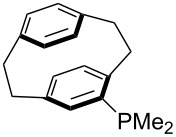 (**309**)	+614.0	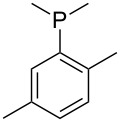 (**310**)	+614.3
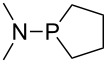 (**311**)	+616.3	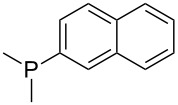 (**312**)	+616.4
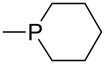 (**313**)	+616.8	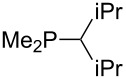 (**314**)	617.1
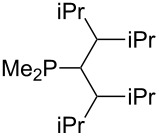 (**315**)	+620.2	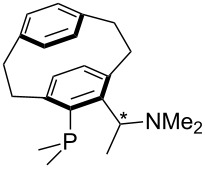 (**316**)	+620.8^a^ +642.0^b^ +586.0^c^
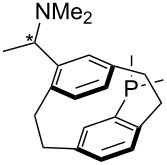 (**317**)	+621.3^a^ +619.7^b^ +586.6^c^	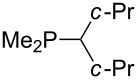 (**318**)	+627.3
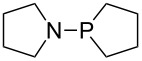 (**319**)	+629.9	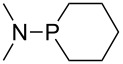 (**320**)	+631.2
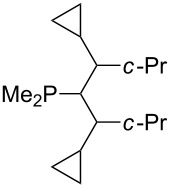 (**321**)	+631.6	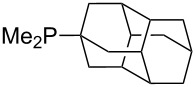 (**322**)	+634.8
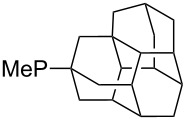 (**323**)	+637.3	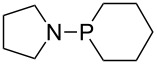 (**324**)	+644.8
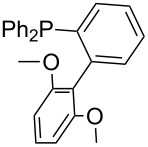 (**325**)	+647.6	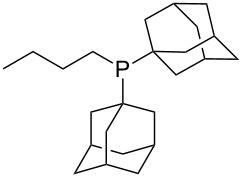 (**326**)	+662.2
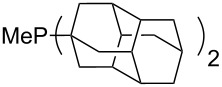 (**327**)	+663.2	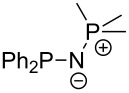 (**328**)	+702.3
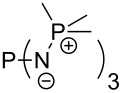 (**329**)	+848.1		

^a^(*S*)-configuration, phosphorus; ^b^(*R*)-configuration, phosphorus; ^c^(*S*)-configuration, nitrogen.

The chiral phosphanes **316** and **317** show that the (*R)*- and the (*S)*-enantiomer do not have to have the same affinity values. For the phosphane **317** the difference is below 2 kJ/mol, but for phosphane **316** the difference is 21 kJ/mol.

A last group of nucleophiles employed in Lewis-base catalysis concerns nucleophilic carbenes (Table 8) [14–17].

**Table 8:** MCA values of carbenes, ordered by increasing MCA values [18].

system	MCA [kJ/mol]	system	MCA [kJ/mol]

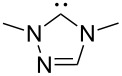 (**330**)	+674.4	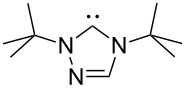 (**331**)	+676.8
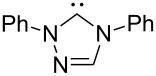 (**332**)	+694.4	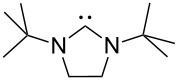 (**333**)	+699.4
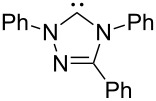 (**334**)	+712.2	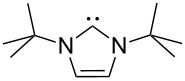 (**335**)	+714.3
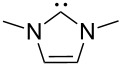 (**336**)	+718.0	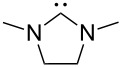 (**337**)	+719.3
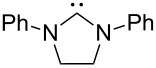 (**338**)	+722.9	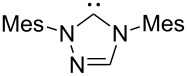 (**339**)	+728.4
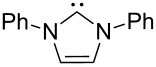 (**340**)	+742.4	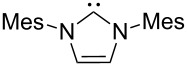 (**341**)	+767.2
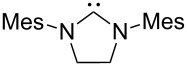 (**342**)	+768.9		

MCA values obtained for NHC-carbenes are significantly larger than those obtained for nitrogen- and phosphorus-based nucleophiles and depend on both the structure of the heterocyclic ring system as well as the substituents attached to the respective 2- and 5-positions. With respect to the dimethyl substituted carbenes, the lowest MCA value is found for the triazolyl carbene **330**, followed by imidazolyl carbene **336** and imidazolidinyl carbene **337**. It should be added that the MCA values for the latter two systems are closely similar (MCA(**336**) = +718.0 kJ/mol vs. MCA(**337**) = +719.3 kJ/mol, indicating a negligible influence of ring aromaticity. The influence of the ring substituents on MCA values is less systematic and depends on the ring system at hand. For all systems, however, the largest MCA values are obtained for mesityl substituents. Mayr and co-workers have determined nucleophilicity parameters *N* and slope parameters *s* of NHC-carbenes **334**, **341** and **342** in THF [18–20]. The slowest reactions were found for triazolyl carbene **334** with *N* = 14.07 (*s* = 0.84). The imidazolyl carbene **341** (*N* = 21.75, *s* = 0.45) and the imidazolidinyl carbene **342** (*N* = 23.35, *s* = 0.40) are, in contrast, 10^8^ to 10^9^ times more nucleophilic. This is in agreement with the MCA values for **341** and **342**, which are approx. 50 kJ/mol higher compared to the value of **334**. However, including other typical organocatalysts such as PPh_3_ (**89**), DMAP (**54**), and DBU (**60**) in the comparison of MCA- and N-values, Mayr et al. note that no general correlation appears to exist between reaction rates and reaction energies for the addition of these nucleophiles to cationic electrophiles. This has been interpreted as a reflection of much larger Marcus intrinsic barriers for carbene nucleophiles as compared to those of phosphanes or N-nucleophiles [21].

### Benzhydryl cation affinities (BHCA)

**Scheme 5:**
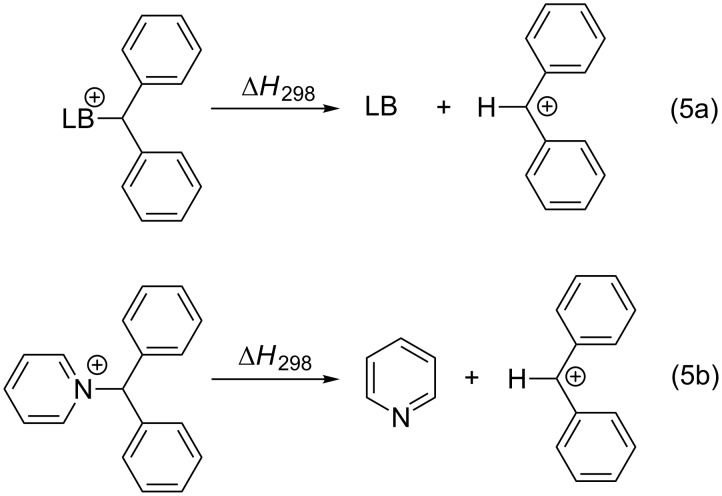
Reactions for the benzhydryl cation affinity (BHCA) of a Lewis base (5a) and pyridine (5b).

The carbon electrophiles involved in Lewis base-catalyzed reactions are typically much larger than the methyl cation. The substituents present in these systems do not only add, in part considerable, steric bulk to the systems, but also stabilize the cation through charge delocalization [22]. Affinity numbers obtained for larger carbocations such as the benzhydryl cation may thus more closely mimic the steric and electronic properties of synthetically used carbon electrophiles. The corresponding benzhydryl cation affinity (BHCA) of a neutral Lewis base (LB) is defined as the reaction enthalpy for the dissociation process shown in equation 5 in Scheme 5. For pyridine as the Lewis base the benzhydryl cation affinity (BHCA) amounts to BHCA(**1**) = 160.0 kJ/mol.

BHCA values of Lewis bases commonly used in organocatalysis and of selected phospanes have been collected in Table 9.

**Table 9:** BHCA values of Lewis base, ordered by increasing BHCA values.

system	BHCA [kJ/mol]	system	BHCA [kJ/mol]

PH_3_ (**343**)	+77.8	NH_3_ (**344**)	+84.3
Ph_2_P–NEt_2_ (**251**)	+137.7^a^	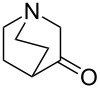 (**345**)	+148.1^b^
NEt_3_ (**45**)	+150.7	NMe_3_ (**20**)	+153.0
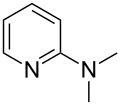 (**346**)	+157.8^b^	pyridine (**1**)	+160.0
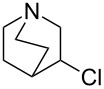 (**347**)	+164.4^b^	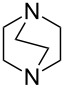 (**44**)	+167.8
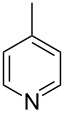 (**14**)	+171.7	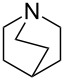 (**53**)	+184.6
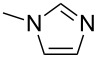 (**24**)	+186.4^c^	P(OPh)_3_ (**252**)	+193.0
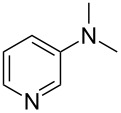 (**348**)	+200.5^b^	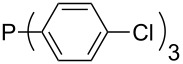 (**254**)	+207.7
P(OEt)_3_ (**255**)	+208.1	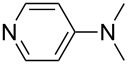 (**54**)	+213.0^b^
PMe_3_ (**70**)	+215.5	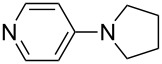 (**56**)	+221.4^b^
P(iPr)_3_ (**117**)	+224.8	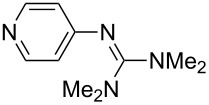 (**349**)	+225.8^b^
PPhMe_2_ (**256**)	+230.1	PPh(iPr)_2_ (**279**)	+230.4
PEt_3_ (**98**)	+230.6	PPh_2_*t*-Bu (**281**)	+232.7
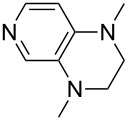 (**350**)	+233.3^b^	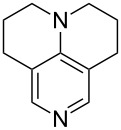 (**57**)	+233.3^b^
PPh_3_ (**89**)	+235.0	PPh_2_Me (**259**)	+236.0
PPhEt_2_ (**261**)	+237.6	PPh_2_Et (**264**)	+241.1
PPh_2_*c*-Pr (**267**)	+241.9	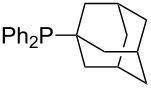 (**294**)	+242.1
PPh_2_*n*-Bu (**102**)	+243.2	PPh*n*-Pr_2_ (**269**)	+244.0
PPh_2_*n*-Pr (**270**)	+245.8	Ph_2_P–NEt_2_ (**251**)	+245.8^d^
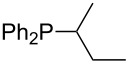 (**276**)	+246.0	PPh*n*-Bu_2_ (**280**)	+248.0
PPh_2_*c*-Bu (**278**)	+248.3	PPh_2_(iPr) (**268**)	+248.3
P*c*-Hex_3_ (**124**)	+249.8	PPh_2_*c*-Hex (**285**)	+250.5
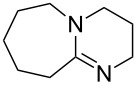 (**60**)	+250.6^e^	PPh_2_*c*-Pen (**286**)	+251.3
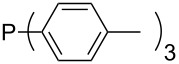 (**295**)	+252.4	PPh_2_*c*-Hep (**289**)	+254.5
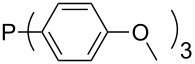 (**298**)	+266.5	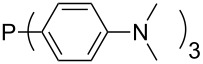 (**300**)	+306.2

^a^BHCA value on nitrogen; ^b^pyridine nitrogen; ^c^N3; ^d^BHCA value on phosphorus; ^e^N(sp^2^).

**Figure 8:**
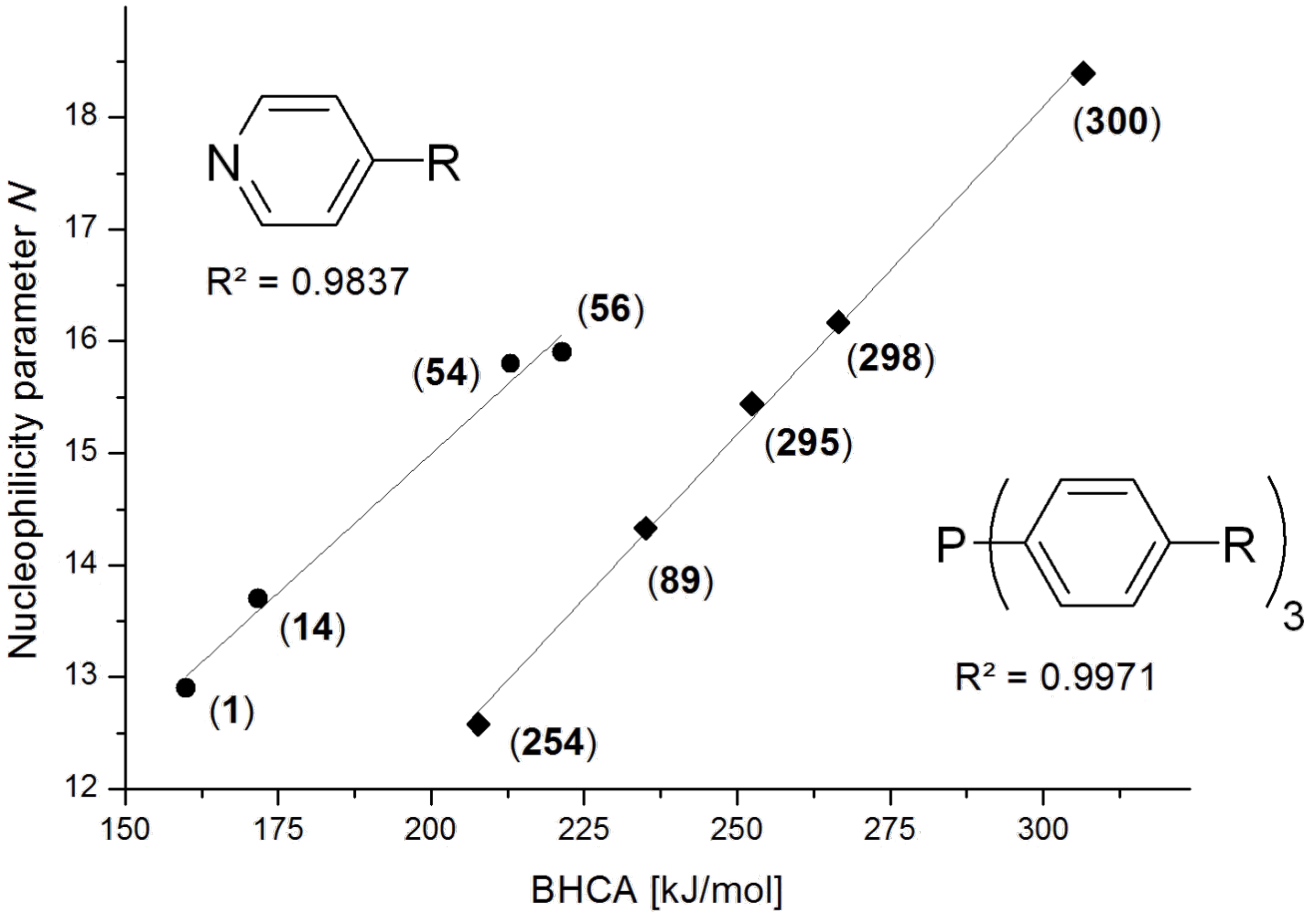
Comparison of BHCA values (kJ/mol) and nucleophilicity parameters *N* for sterically unbiased pyridines and phosphanes.

The benzhydryl cation affinities (BHCA) of weak nucleophiles like ammonia (**344**) and phosphane (**343**) are less than 100 kJ/mol and thus much smaller than the respective MCA values. In contrast, often used organocatalysts like 4-dimethylaminopyridine (**54**), PPY (**56**), and PPh_3_ (**89**) possess BHCA values above 200 kJ/mol. The strongest nucleophiles, the tris-*para-*methoxy- and tris-*para*-dimethylamino-substituted triphenylphosphanes (**298** and **300**) reach BHCA values of 267 and 306 kJ/mol, respectively. BHCA values of selected pyridine and phosphane bases correlate in a linear fashion with experimentally measured nucleophilicity parameters *N* of these systems (Figure 8) [23–24].

While correlation within each of the catalyst families is very good, it is also apparent that the pyridines and phosphanes form clearly separate correlation lines. This is commonly understood as a reflection of systematically different Marcus intrinsic barriers [21] for these two classes of nucleophiles.

### Trityl cation affinities (TCA)

**Scheme 6:**
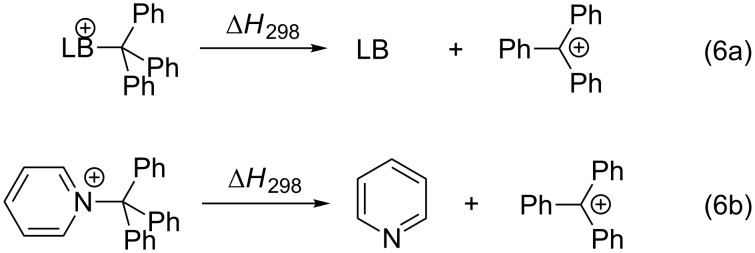
Reactions for the trityl cation affinity (THCA) of a Lewis base (6a) and pyridine (6b).

The benzhydrylium cation is attacked by the nucleophile on a secondary carbon atom. In order to cover also electrophiles with tertiary carbon atoms as the center of attack we chose the trityl cation (^+^CPh_3_) as the third reference electrophile. In this case the steric bulkiness is increased even more than in the benzhydrylium cation. The corresponding trityl cation affinity (TCA) of a neutral Lewis base (LB) is defined as the reaction enthalpy for the dissociation process shown in equation 6 in Scheme 6. For pyridine as the Lewis base the benzhydryl cation affinity (TCA) amounts to TCA(**1**) = 82.9 kJ/mol at the MP2-5 level of theory.

The TCA values of some Lewis bases commonly used in organocatalysis as well as various phosphanes and phosphites are shown in Table 10.

**Table 10:** TCA values of Lewis bases, ordered by increasing TCA values.

system	TCA [kJ/mol]	system	TCA [kJ/mol]

PH_3_ (**343**)	+18.2	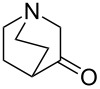 (**345**)	+28.0
NEt_3_ (**45**)	+31.6	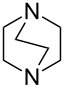 (**44**)	+34.1
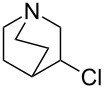 (**347**)	+34.9^a^	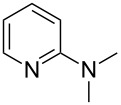 (**346**)	+54.1^a^
P(iPr)_3_ (**117**)	+66.6	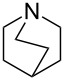 (**53**)	+70.8
pyridine (**1**)	+82.9	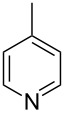 (**14**)	+94.4
PPh(iPr)_2_ (**279**)	+106.5	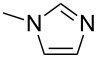 (**24**)	+113.4^b^
PPh_2_*t*-Bu (**281**)	+115.3	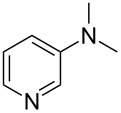 (**348**)	+121.4^a^
P(OPh)_3_ (**252**)	+121.5	P*c*-Hex_3_ (**124**)	+129.5
PMe_3_ (**70**)	+131.3	PEt_3_ (**98**)	+134.2
P(OEt)_3_ (**255**)	+134.3	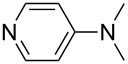 (**54**)	+134.7^a^
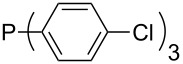 (**254**)	+135.5	PPh_2_(iPr) (**268**)	+137.5
PPhMe_2_ (**256**)	+141.9	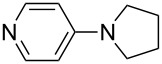 (**56**)	+142.6^a^
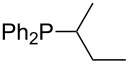 (**276**)	+142.8	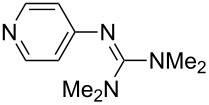 (**349**)	+145.9^a^
PPh_2_*c*-Hex (**285**)	+147.4	PPh*n*-Bu_2_ (**280**)	+148.5
PPh_2_Et (**264**)	+149.6	PPh_2_*n*-Bu (**102**)	+150.4
PPh_2_Me (**259**)	+150.6	PPh_2_*c*-Pen (**286**)	+152.5
PPh_2_*n*-Pr (**270**)	+154.2	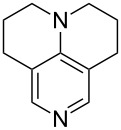 (**57**)	+154.8^a^
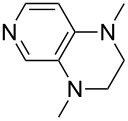 (**350**)	+155.6^a^	PPh_3_ (**89**)	+158.8
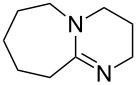 (**60**)	+160.0^c^	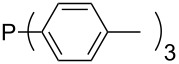 (**295**)	+176.1
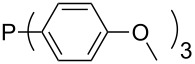 (**298**)	+189.9	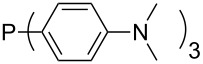 (**300**)	+229.7

^a^Pyridine nitrogen; ^b^N3; ^c^N(sp^2^).

In general, TCA values are about 70 to 80 kJ/mol smaller than the respective BHCA values (e.g., for pyridine (**1**) or triphenylphosphane (**89**)). Moreover, some of the weakest Lewis bases considered here such as DABCO (**44**) are not sufficently basic to form covalently bound adducts with trityl cations. The TCA values calculated for these systems thus represent the reaction enthalpies for the formation of ion-dipole complexes. Aside from DABCO this is the case for **345**, **45**, and **347**. The C–N bond distances of the energetically best conformations of these complexes range from 2.8 Å to 4.0 Å. As a reference bond length the C–N distance in pyridine-trityl adduct (**1TT**), which amounts to 1.57 Å, can be used. A slightly increased C–N bond length can be found for the TCA-adduct of quinuclidine **53** (1.76 Å), which is in distinct contrast to the structurally similar DABCO. It should be added that all other electrophiles considered here form covalent adducts even with weak Lewis bases, and that the formation of ion-dipole complexes between the trityl cation and weak nucleophiles are therefore true exceptions.

### General comparison

**Figure 9:**
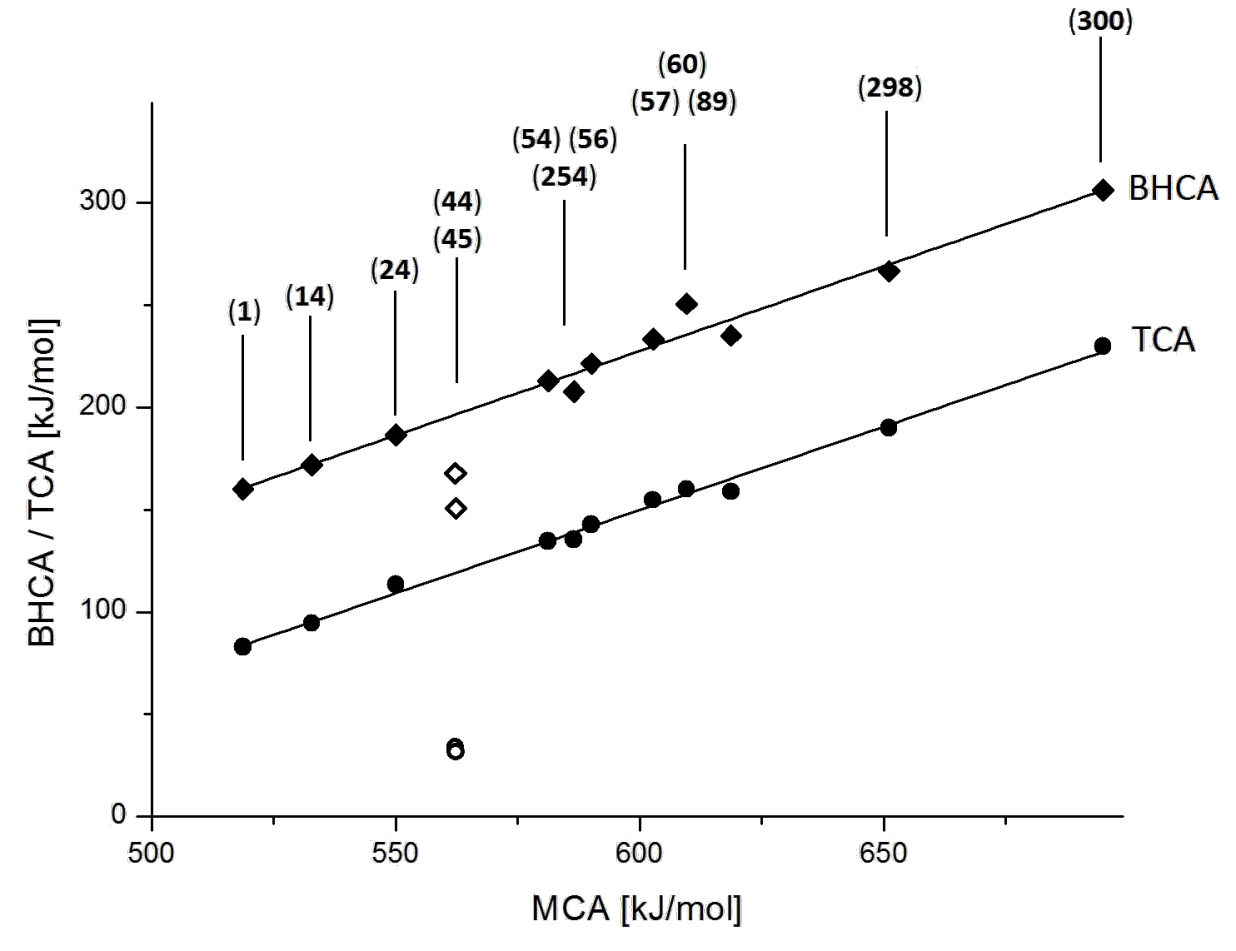
Comparison of MCA, BHCA, and TCA values of selected Lewis bases.

The affinity data for cationic electrophiles of varying stability presented in the previous section for a large range of different Lewis bases provides the basis for a more general analysis of Lewis base affinity data. Perusal of the results obtained for pyridine (**1**) with MCA(**1**) = +518.7 kJ/mol, BHCA(**1**) = +160.0 kJ/mol, and TCA(**1**) = +82.9 kJ/mol already indicates that cation affinity values towards different carbocations span an extraordinarily large energy range. In order to find out, whether different nucleophiles respond to changes in the electrophile in a systematically comparable manner, we have selected a small group of nucleophiles of different type for a direct comparison of affinity data (Figure 9).

**Scheme 7:**
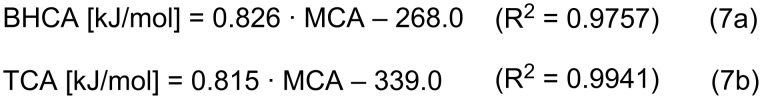
Correlations of BHCA/TCA values with the respective MCA data for sterically unbiased systems (excluding DABCO (**44**) and triethylamine (**45**)).

From Figure 9 it can be seen that most Lewis bases respond to the change from methyl cation (MCA) to benzhydryl cation (BHCA) and trityl cation (TCA) affinities in the same way, that is, with a large reduction of cation affinity. This is also reflected in the correlations of BHCA/TCA values with the respective MCA data for sterically unbiased systems (excluding DABCO (**44**) and triethylamine (**45**)), which can quantitatively be expressed by equations 7a and 7b given in Scheme 7.

The rather similar slope of both correlation lines (0.826 vs. 0.815) implies that the offset between both datasets of 268.0 − 339.0 = −71 kJ/mol is a reflection of the stability difference between the triphenylmethyl and the benzhydryl cation. This stability difference is slightly larger than that derived from theoretically calculated gas phase hydride ion affinities (63 kJ/mol) [25], or from experimentally determined hydride ion affinities in DMSO solution (38 kJ/mol) [26]. The only deviations from the general correlations in Figure 9 can be seen for bases unable to form covalently bound adducts, and for the sterically more demanding bases, which show much smaller BHCA values than should be expected on the basis of their MCA values. The much smaller Lewis basicity of DABCO (**44**) compared to that of DMAP (**54**) has also been cited in experimental studies as the prime reason for the different catalytic profile of these two catalysts [27]. The fact that no covalent adduct could be identified between the trityl cation and DABCO (**44**) also illustrates that this (kinetically very competent) nucleophile may not be able to form stable adducts with sterically demanding electrophilic substrates, thus limiting its catalytic potency for these types of substrates. This implies that for very strong Lewis bases any of the cation affinity scales can be used as a measure of Lewis basicity. For weak and sterically biased Lewis bases the reference cation has to be selected with the electrophilic substrate of the Lewis base-catalyzed process in mind.

**Figure 10:**
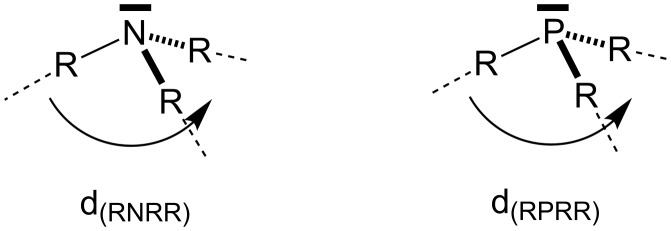
Scheme for the angle *d(RXRR)* measurements.

In order to identify further differences between reactions of amines and phosphanes the pyramidalization angle *d(RNRR/RPRR)* (Figure 10), the HOMO–LUMO gap (Δ_HOMO–LUMO_), and the s/p composition of the lone pair from NBO analysis has been compiled in Table 11 for selected systems.

**Table 11:** RXRR angle, Δ_HOMO-LUMO_ and character of the lone pair of Lewis bases.

system	angle (RXRR)^a^	Δ_HOMO–LUMO_ [a.u.]	lone pair character

NH_3_	112.1°	0.3463	25.3% s 75.6% p
PH_3_	93.8°	0.3154	54.2% s 45.8% p
NMe_3_	124.8°	0.3027	15.9% s 84.1% p
PMe_3_	101.2°	0.2914	53.5% s 46.5% p
NPh_3_	179.2°	0.1756	0.0% s 100.0% p
PPh_3_	106.1°	0.1976	48.9% s 51.1% p

^a^X = N or P; R = C or H.

From the data above it can easily be seen that the RXRR angle in phosphanes is systematically smaller than the one in amines. This implies that phosphanes have a more pyramidal structure than amines with a comparable substitution pattern. The least pyramidal structure is found here for triphenylamine, which is almost perfectly planar at the nitrogen atom. The degree of planarity correlates well with the character of the lone pair orbital. In amines the contribution of the s orbital is decreasing with increasing size of the substituents. This is different for phosphanes, where the lone pair orbital has a systematically larger s-character, which depends only marginally on the substitution pattern. The HOMO–LUMO gap, in contrast, shows no significant correlation with the degree of pyramidalization but depends largely on the substitution pattern.

### Mosher’s cation affinities (MOSCA)

**Scheme 8:**
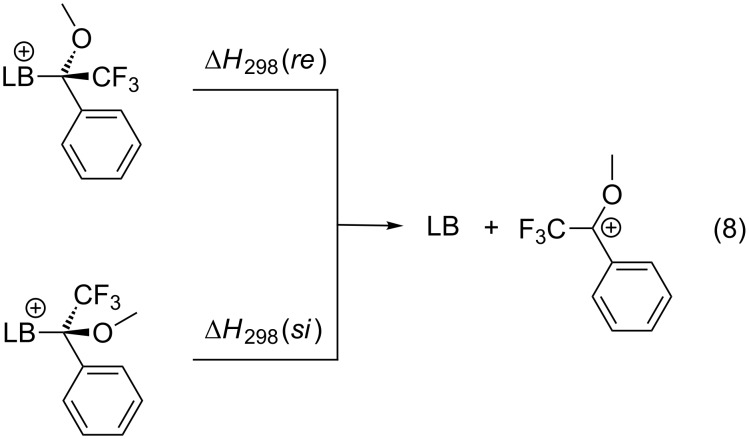
Reactions for the Mosher's cation affinity (MOSCA) of a Lewis base.

For the multitude of stereoselective organocatalytic transformations the affinity of chiral Lewis bases towards chiral or prochiral carbon electrophiles may constitute part of the overall stereodifferentiating process. The potential of differentiating the faces of a prochiral electrophile can be quantified for Lewis bases through affinity numbers to a prochiral reference cation. The potential of this approach has been explored using the 1-methoxy-1-trifluoromethylbenzyl cation shown in equation 8 (Scheme 8), whose substitution pattern resembles that of Mosher's acid [28] and has thus been named "Mosher's cation" [29]. The respective "Mosher's cation affinity" values (MOSCA) for the *re* and *si* face adducts of chiral Lewis bases will not be identical, but differ depending on how much of the chiral information is relayed to the reaction center (Scheme 8).

The results for selected systems relevant as Lewis base catalysts are shown in Table 12. In absolute terms it can readily be seen that MOSCA values are of similar magnitude like BHCA values.

**Table 12:** MOSCA values of chiral Lewis bases, ordered by increasing MCA values.

system	MOSCA [kJ/mol]	system	MOSCA [kJ/mol]

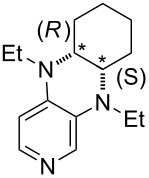 (**63**)	255.7(*si*) 257.5(*re*) 1.8^a^	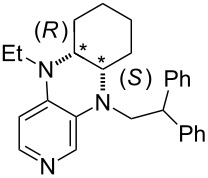 (**351**)	264.3(*si*) 264.2(*re*) −0.1^a^
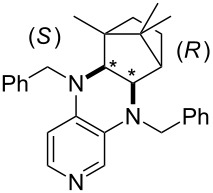 (**352**)	255.5(*si*) 248.8(*re*) −6.7^a^	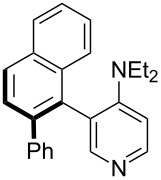 (**353**)	233.7(*si*) 235.6(*re*) 1.9^a^
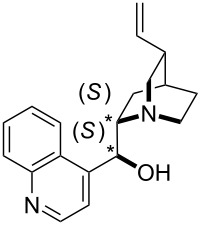 (**354**)	188.4(*si*)^b^ 197.6(*re*)^b^ 9.2^a^	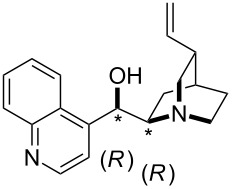 (**355**)	180.0(*si*)^b^ 183.7(*re*)^b^ 3.7^a^
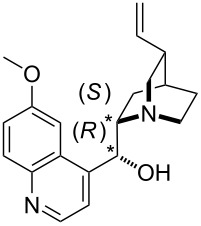 (**356**)	233.7(*si*)^b^ 227.3(*re*)^b^ −6.4^a^	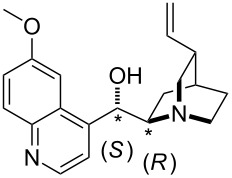 (**357**)	223.3(*si*)^b^ 231.2(*re*)^b^ 7.9^a^

^a^ΔMOSCA_re-si_; ^b^without PCM calculation.

It was recently shown that 3,4-diaminopyridines are catalytically active in a variety of group transfer reactions [9–10,30]. Chiral 3,4-diaminopyridines thus have the potential to act as catalysts in stereoselective transformations. In how far the chiral information present in Lewis bases **63**, **351**–**355** has the potential to reach the reaction center has therefore been elucidated through calculation of the respective MOSCA values. The very small difference between *re* and *si* face attack calculated for **351** indicates that stereoselectiove transformations may be difficult to achieve with this catalyst design.

In contrast, substantial *re*/*si* face differences have been obtained for Lewis bases **352** and **354**, indicating that these compounds may be useful catalysts for stereoselective transformations. This property is already well established for quinine (**356**) and quinidine (**357**), whose ΔMOSCA*_re-si_* values imply the clear potential of stereoselective Lewis base-catalyzed reactions [31–40].

### Acetyl cation affinities (ACA)

**Scheme 9:**
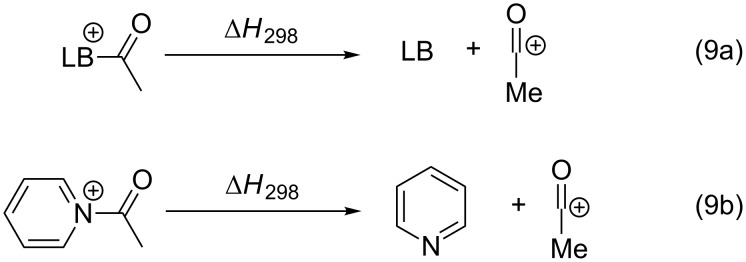
Reactions for the acetyl cation affinity (ACA) of a Lewis base (9a) and pyridine (9b).

Reactions between carbon electrophiles and Lewis bases may also lead to the formation of a new common π-system. This is, for example, the case in all acyl transfer reactions catalyzed by pyridine bases which involve acetylpyridinium cations as intermediates of the catalytic cycle [7,41–46]. The acetyl cation may be considered to be a representative cationic probe for this type of situation and the corresponding acetyl cation affinities (ACA) of neutral Lewis bases thus reflect the enthalpies for the reaction shown in equation 9 in Scheme 9. Using pyridine again as a typical example, the acetyl cation affinity amounts to ACA(**1**) = +156.1 kJ/mol. Additional ACA values can be found in Table 13.

**Table 13:** ACA values of pyridines and 4-aminopyridines, ordered by increasing ACA values.

system	ACA [kJ/mol]	system	ACA [kJ/mol]

pyridine (**1**)	+156.1	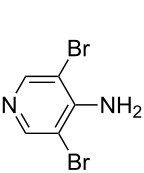 (**358**)	+164.7
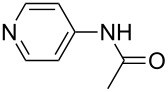 (**359**)	+175.0	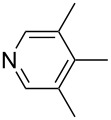 (**360**)	+182.4
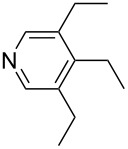 (**361**)	+183.8	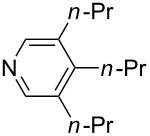 (**362**)	+183.5
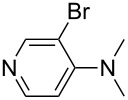 (**363**)	+184.1	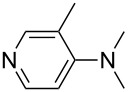 (**364**)	+206.4
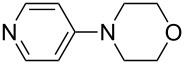 (**365**)	+207.2	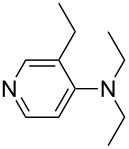 (**366**)	+210.7
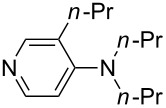 (**367**)	+211.4	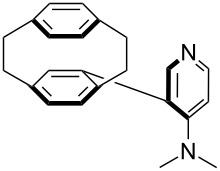 (**368**)	+211.5
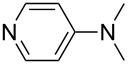 (**54**)	+217.3	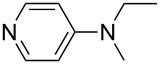 (**369**)	+219.4
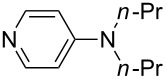 (**370**)	+222.2	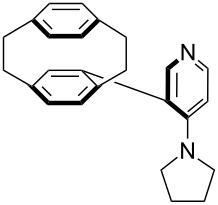 (**371**)	+222.8
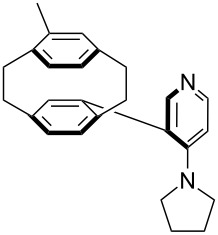 (**372**)	+223.0	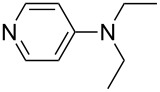 (**373**)	+223.2
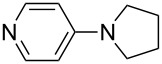 (**56**)	+223.7	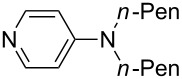 (**374**)	+223.9^a^
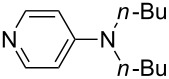 (**375**)	+224.0^a^	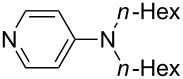 (**376**)	+224.2^a^
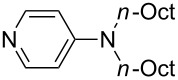 (**377**)	+224.6^a^	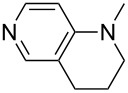 (**378**)	+227.8
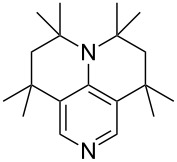 (**379**)	+230.0	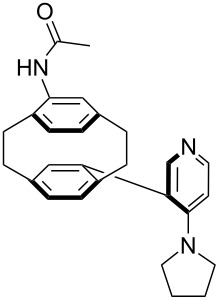 (**380**)	+236.6
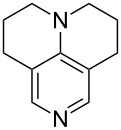 (**57**)	+238.3	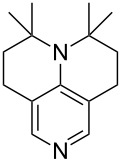 (**381**)	+238.5
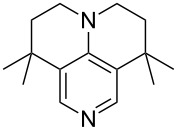 (**382**)	+239.1		

^a^The conformational space used is based on the minima found for system **370** and elongation of alkyl groups using all-*trans* conformations.

**Figure 11:**
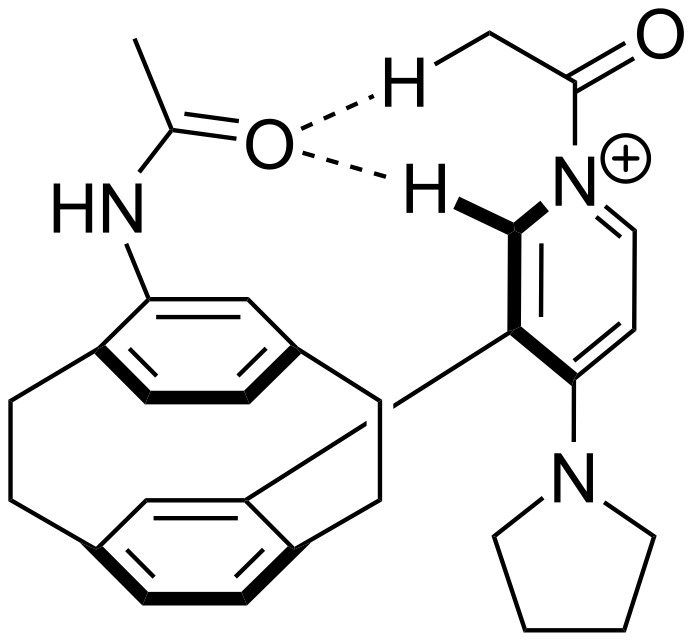
Structure of the acetylated pyridine **380** (**380Ac**).

For *N,N*-dialkyl-4-aminopyridines (**54**, **369**, **370**, **373**–**377**) it is interesting to see how elongation of the alkyl substituents leads to a rapid convergence of the ACA values. The two methyl groups in **54** lead to an ACA value just 7 kJ/mol or 3% below the two octyl groups (**377**). The group of pyridines derived from the tricyclic moiety (**57**) can just slightly be modified towards higher affinity to acetyl cation (**381**, **382**). Inclusion of too many methyl groups as in **379** leads to disfavorable interactions and therefore to a decrease of the ACA value. The 2,2’-paracyclophanes (**368**, **371**, **372**, **380**) are derived from DMAP (**54**) and PPY (**56**). In the first case (**368**) the paracyclophane substituent leads to a lower affinity towards the acetyl cation. The two Lewis bases **371** and **372** show almost no influence of the paracyclophane moiety on the ACA values. Inclusion of an amide substituent as in pyridine **380** leads to a surprisingly large increase in the ACA value. This is due to the formation of close contacts between the amide substituent and the acetylpyridinium moiety in the acetylated catalysts (Figure 11).

3,4-Diaminopyridines have been shown to be particularly effective as acyl transfer catalysts. This is also visible in the respective ACA values (Table 14).

**Table 14:** ACA values of 3,4-diaminopyridines, ordered by increasing ACA values.

system	ACA [kJ/mol]	system	ACA [kJ/mol]

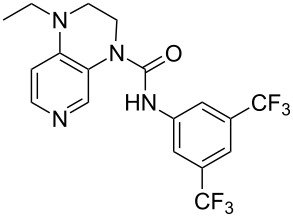 (**383**)	+207.3	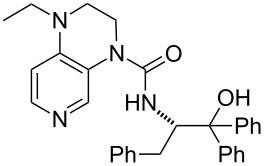 (**384**)	+211.1
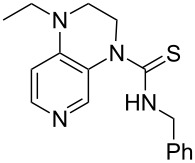 (**385**)	+214.0	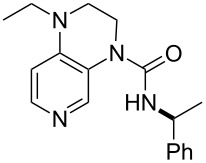 (**386**)	+215.2
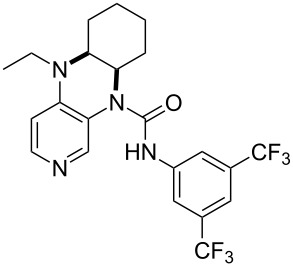 (**387**)	+215.6	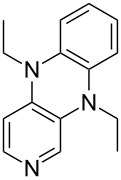 (**388**)	+216.2
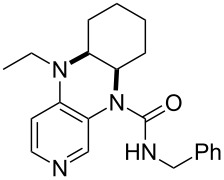 (**389**)	+217.3	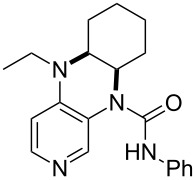 (**390**)	+219.1
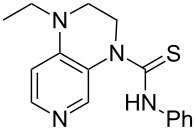 (**391**)	+219.3	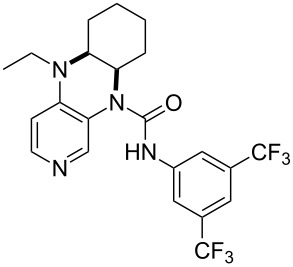 (**392**)	+219.8
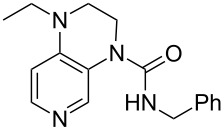 (**393**)	+220.4	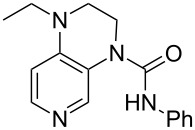 (**394**)	+220.4
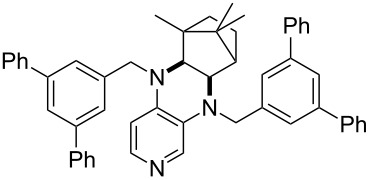 (**395**)	+225.4	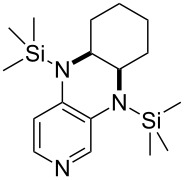 (**396**)	+225.9
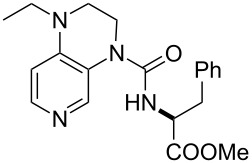 (**397**)	+226.7	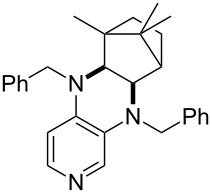 (**352**)	+231.5
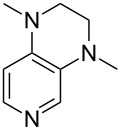 (**350**)	+233.8	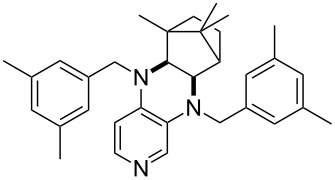 (**398**)	+233.9
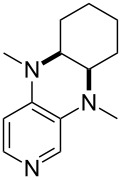 (**59**)	+235.5	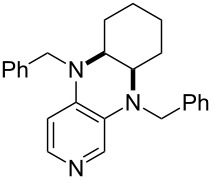 (**399**)	+236.0
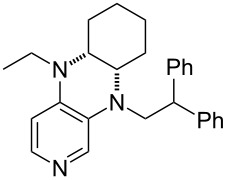 (**351**)	+236.2	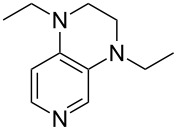 (**58**)	+237.5
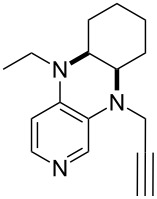 (**400**)	+237.9	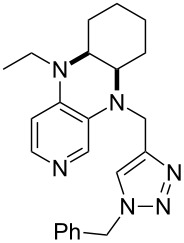 (**401**)	+238.2
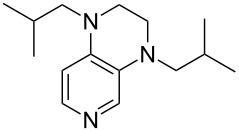 (**402**)	+238.8	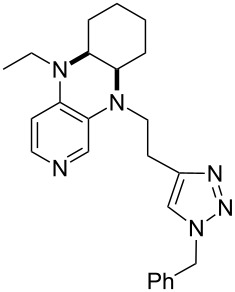 (**403**)	+239.3
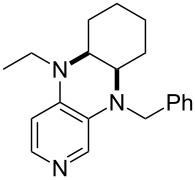 (**404**)	+240.2	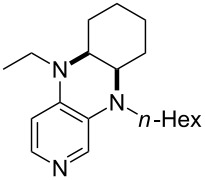 (**405**)	+240.9
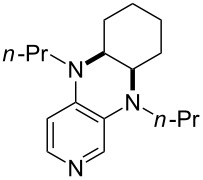 (**406**)	+241.2	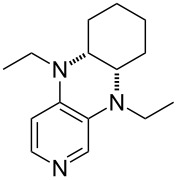 (**63**)	+241.3
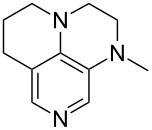 (**407**)	+242.9	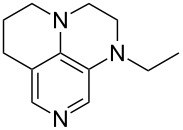 (**408**)	+243.6
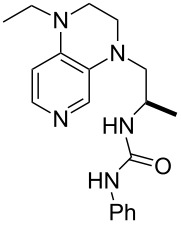 (**409**)	+245.0	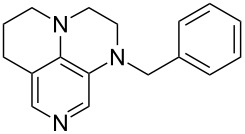 (**410**)	+246.3

Most of the 3,4-diaminopyridines (**58**, **59**, **63**, **399**–**408**) show ACA values which are roughly between 235 and 243 kJ/mol. However, the introduction of a (thio)urea moiety as in **383**–**387**, **389–394**, and **397** lowers the ACA value by 10 to 25 kJ/mol. Annelation of an additional six-membered ring to bicyclic 3,4-diaminopyridines leads to tricyclic diaminopyridines **407**, **408**, and **410** and is accompanied by an increase in acetyl cation affinities above 240 kJ/mol. Annelation of a carbocyclic ring thus has a comparabel effect as already observed for DMAP (**54**) and its ring-extended forms **378** and **57**. This is in remarkable contrast to DMAP derivatives such as **364** carrying non-annelated alkyl substituents in 3- and/or 5-position with clearly lower ACA values. Comparison of pyridines **63** and **388** furthermore shows that alkyl groups directly attached to the amine substituents in 3- and 5-position are significantly more effective than aryl substituents in stabilizing the pyridinium ions formed through acetyl cation addition.

Photo-switchable 3,4-diaminopyridines including a diazo moiety are potentially useful as special-purpose catalysts. The azobenzene substituent itself is electron-withdrawing in nature and the calculation of ACA values can thus be used to optimize the design of these Lewis bases (Table 15).

**Table 15:** ACA values of 3,4-diaminopyridines with diazo moiety, ordered by increasing ACA values.

system	ACA [kJ/mol]	system	ACA [kJ/mol]

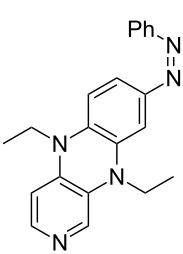 (**411**)	+205.2	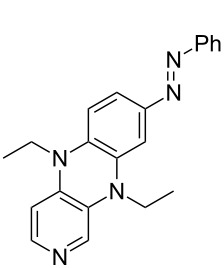 (**412**)	+209.0
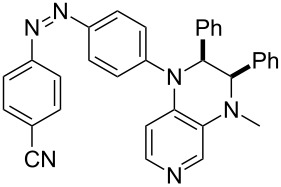 (**413**)	+214.3	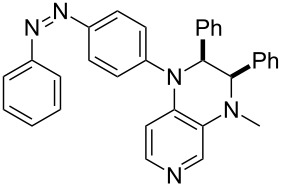 (**414**)	+217.0
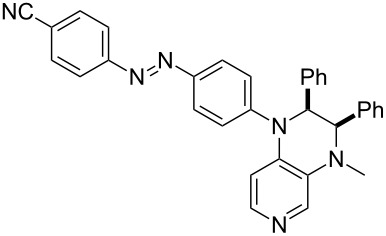 (**415**)	+217.2	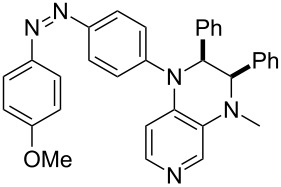 (**416**)	+218.0
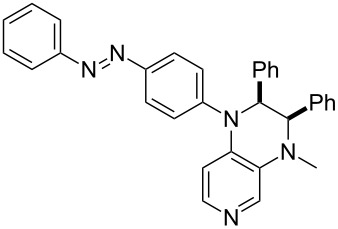 (**417**)	+219.1	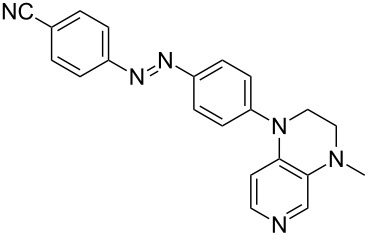 (**418**)	+220.1
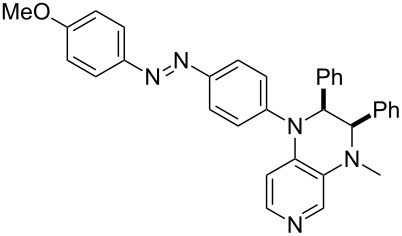 (**419**)	+221.2	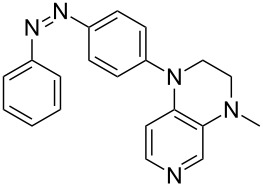 (**420**)	+223.1
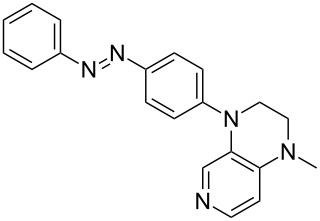 (**421**)	+223.2	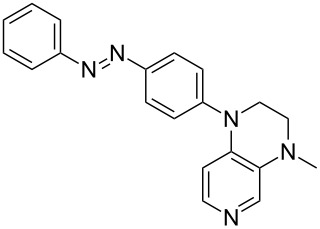 (**422**)	+223.7
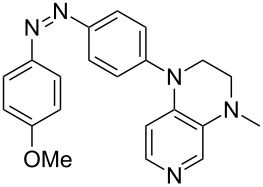 (**423**)	+223.7	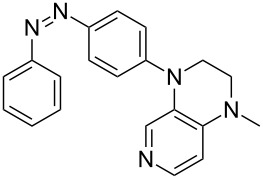 (**424**)	+225.5
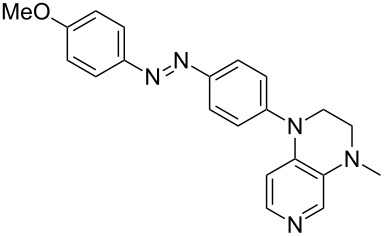 (**425**)	+225.6	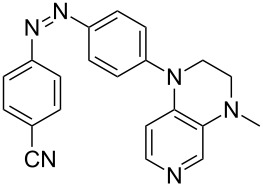 (**426**)	+225.9

In terms of their overall architecture the pyridine bases presented in Table 15 fall into two different categories: The first of these attaches the diazo bridge to the C8-position of the pyridoquinoxaline framework and leads to a significant drop in ACA values (e.g., in compounds **411**/**412**). In the second category, the diazo bridge connects to the 3,4-diaminopyridine amino nitrogen atoms through a phenyl spacer unit and leads to significantly larger ACA values as is best seen for compounds **418** and **426**. This latter system also displays a significant difference in ACA values for the *cis*- and *trans*-diazo isomers, indicating the potential for a photoswitchable Lewis base.

Pyridine-bases including a larger number of electron-donating substituents are highly interesting as Lewis base catalysts. ACA values for this class of compounds have been collected in Table 16.

**Table 16:** ACA values of 3,4,5-triaminopyridines and guanidines, ordered by increasing ACA values.

system	ACA [kJ/mol]	system	ACA [kJ/mol]

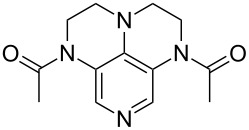 (**427**)	+204.3	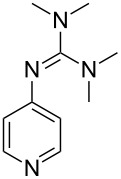 (**428**)	+223.6
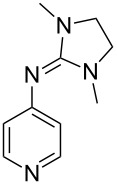 (**429**)	+226.2	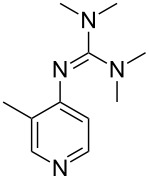 (**430**)	+229.1
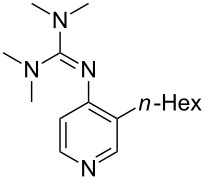 (**431**)	+229.9	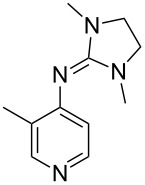 (**432**)	+231.5
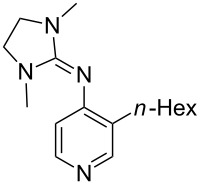 (**433**)	+231.6	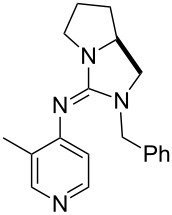 (**434**)	+232.8
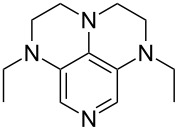 (**435**)	+243.9	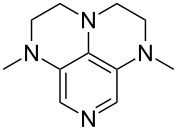 (**436**)	+245.3
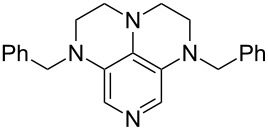 (**437**)	+254.4		

The 3,4,5-triaminopyridines (**435**–**437**) show the highest ACA values of all examined neutral Lewis bases with affinity values ranging from 244 to 255 kJ/mol. These values parallel the impressivley high nucleophilicity parameters *N* measured recently for these compounds and indicate that carbon basicities parallel the kinetics of base addition to carbocations for this class of compounds [47]. Guanidinyl pyridines such as **433** have, in contrast, surprisingly low ACA values around 230 kJ/mol. Structural changes in the guanidine motif have only a moderate influence on the affinity to the acetyl cation.

### Michael-acceptor affinities (MAA)

**Scheme 10:**
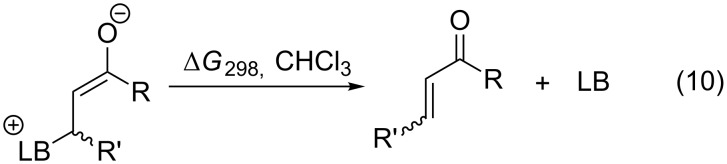
Reaction for the Michael-acceptor affinity (MAA) of a Lewis base.

A large number of reactions induced or catalyzed by Lewis bases involve initial or rate-limiting reaction with neutral electrophiles such as alkyl halides (substitution) or Michael acceptors (addition). Taking the (aza-)Morita–Baylis–Hillman reaction as an example the first step of the catalytic cycle involves the attack of N- or P-centered nucleophiles to a Michael acceptor (equation 10, Scheme 10). In contrast to the Lewis base additions to cationic electrophiles discussed above, in which a cationic substrate reacts to yield a cationic adduct, the reaction now leads from two neutral reactants to a zwitterionic adduct. Solvation energies for this latter type of species are typically significantly larger than for the neutral reactants, indicating a much larger role of solvent effects on this type of process than for the cation addition reactions considered initially. The use of this type of affinity data as the guiding principle in quantitative reactivity studies will thus be restricted to the comparison of structurally and electronically similar systems.

**Figure 12:**
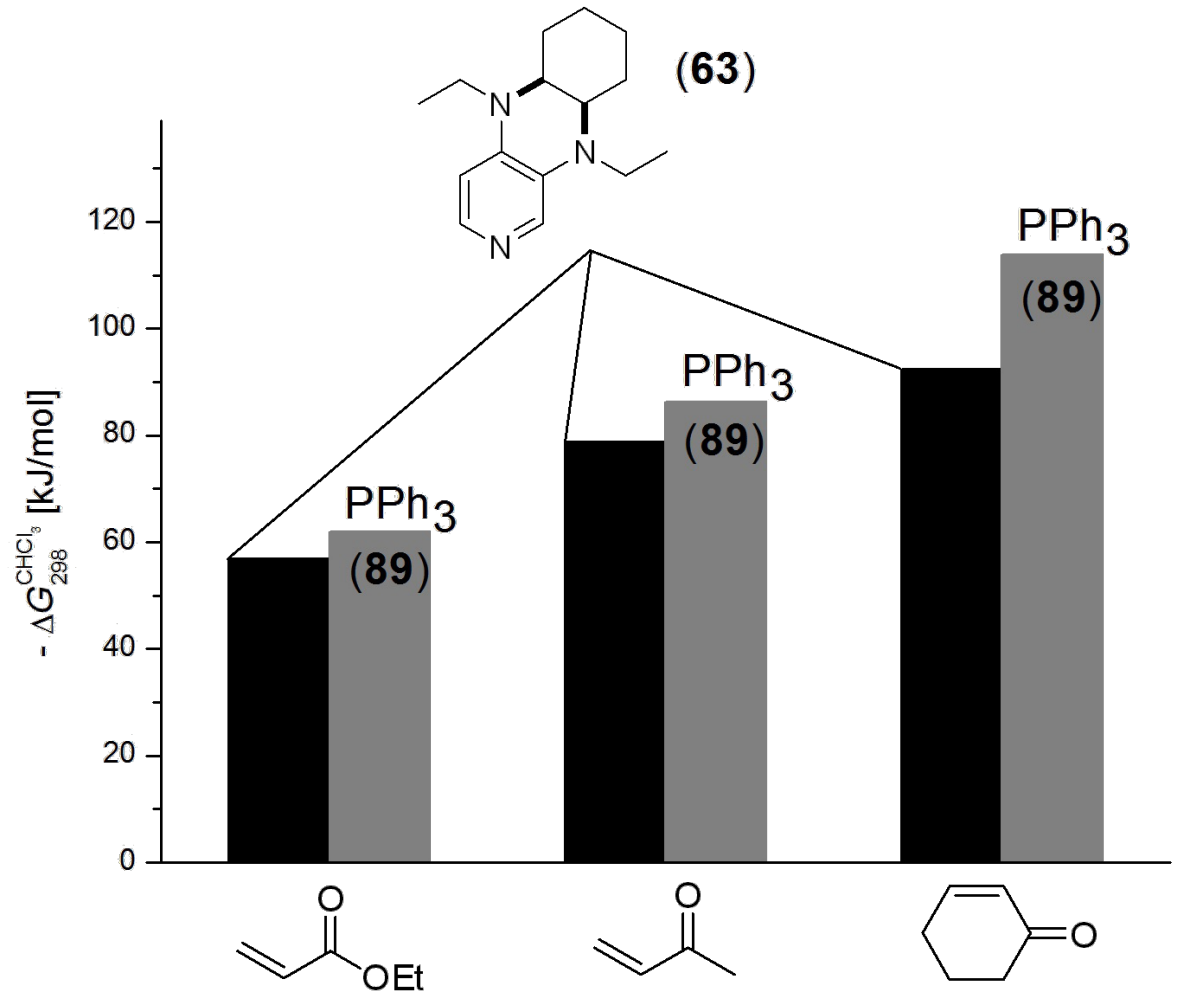
Inverted reaction free energies for the addition of N- and P-based Lewis bases to three different Michael acceptors.

For ethyl acrylate, methyl vinly ketone (MVK) and cyclohexenone as representative examples for synthetically useful Michael acceptors, the reaction with pyridine **63** and triphenylphosphane (**89**) is found to be significantly endergonic (Figure 12) [30].

**Figure 13:**
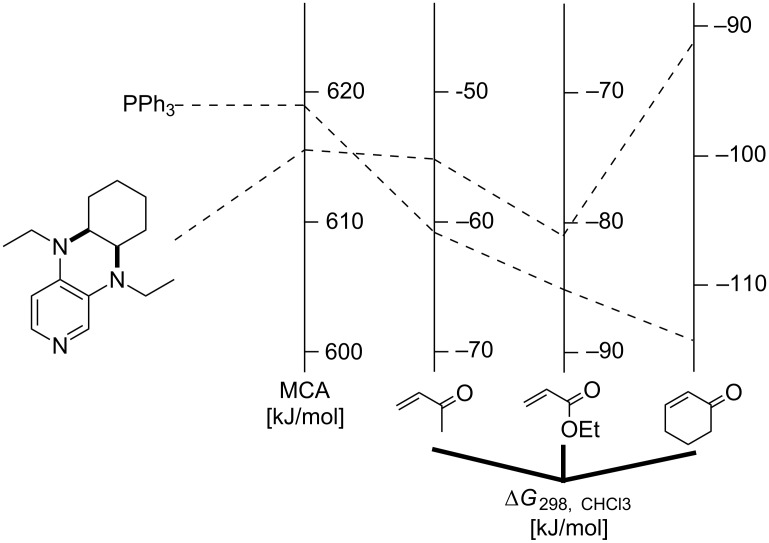
Correlation between MCA values and affinity values towards three different Michael acceptors.

In turn this implies that the free energy for dissociation of the zwitterionic adduct as defined in equation 10 is exergonic, which is in remarkable contrast to the energetics calculated for all cationic electrophiles above. Zwitterionic adducts formed by pyridine **63** are somewhat more stable than those formed by triphenylphosphane (**89**). These energetics parallel results obtained in azaMBH reactions of these three substrates with aromatic imines [30]. Matching the affinity data for Michael acceptors with MCA values we also find an inversion of Lewis basicity in that phosphane **89** has a larger MCA value but a lower binding affinity to the prototypical Michael acceptors selected here. The discussed results are depicted in Figure 13.

### Technical aspects

It was shown recently that MCA values can be calculated with high accuracy with methods like G2, G3 or W1 [5]. Beside these expensive methods some MP2 calculations can also afford, slightly less, accurate results. For the MP2 calculations different combinations of polarization functions and diffuse functions were tested. In contrast, DFT methods such as B3LYP seem to be unsuitable for predicting MCA values in an adequate manner. A good compromise between computational effort and predictive value was found for the MP2(FC)/6-31+G(2d,p)//B98/6-31G(d) level of theory. Therefore, all results described in this publication have been obtained using this approach.

**Scheme 11:**
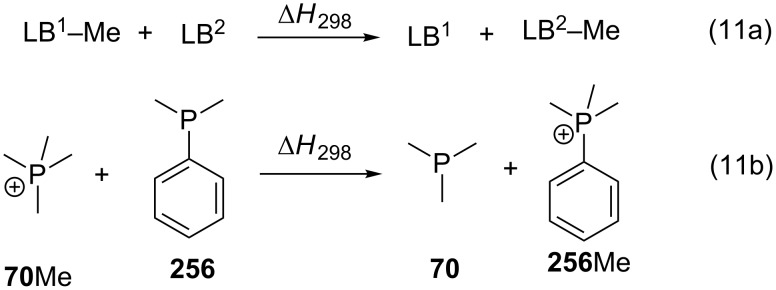
(a) General definition for a methyl cation transfer reaction between Lewis bases LB^1^ and LB^2^, and (b) methyl cation exchange between trimethylphosphane (**70**) and dimethylphenylphosphane (**256**).

Despite the fact that all affinity definitions in equations 1, 5, 6, 8–10 use the separate reactants as the thermochemical reference state, for most applications in synthesis and catalysis it is absolutely sufficient to consider differences in cation affinities between two different Lewis bases. These differences can most easily be expressed as cation transfer reactions between two Lewis bases as described by equation 11a (Scheme 11). Taking the methyl cation affinities of trimethylphosphane (**70**) with MCA(**70**) = 604.7 kJ/mol and dimethylphenylphosphane (**256**) with MCA(**256**) = 611.3 kJ/mol as an example we note that the latter is larger by 6.6 kJ/mol at the G3 level of theory (equation 11b, Scheme 11). A slightly lower value of 4.3 kJ/mol is obtained with the MP2-5 method used throughout this manuscript (Table 2 and Table 6).

**Figure 14:**
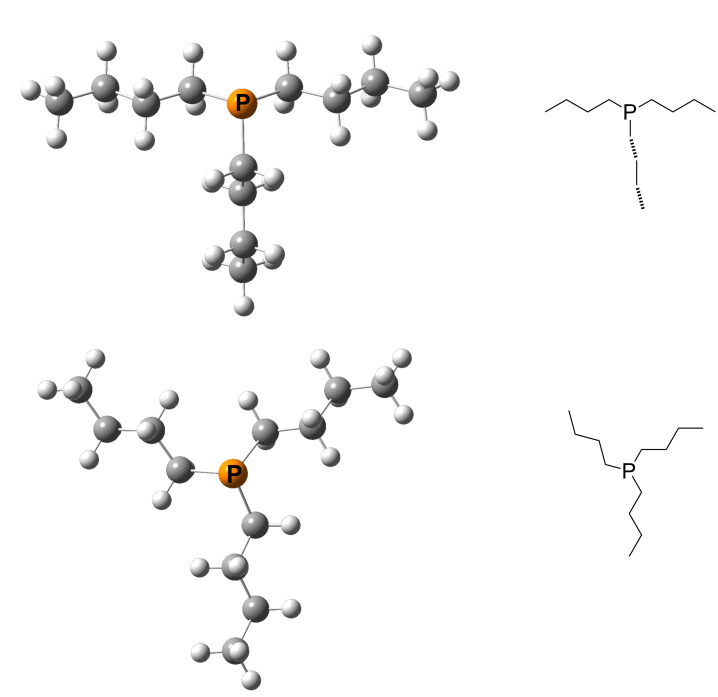
The energetically best conformations of P*n*-Bu_3_ (**120**_1, top) and (**120**_2, bottom).

The enthalpy for the methyl cation transfer reaction between these two species as expressed in equation 11b amounts to −6.6 kJ/mol, the negative sign indicating that the MCA of phosphane **256** is larger than that of phosphane **70**. Under the condition that the two Lewis bases involved in cation exchange are as similar as the two phosphanes **70** and **256**, the overall transformation represents an isodesmic reaction, in which the numbers of bonds of particular type are identical (at least formally) on both sides. The calculation of thermochemical data for isodesmic reactions is usually more accurate than for other defining equations due to the cancellation of numerous errors. Additional practical challenges in calculating accurate affinity numbers concern the often large conformational space of Lewis bases and their cationic adducts. This can easily be demonstrated for P*n*-Bu_3_ (**120**) and its methylated form P^+^Me(*n*-Bu)_3_ (**120**Me). Depending on the strategy and the programs used for conformational searches, both species will have hundreds of conformations. Using systematic searches in combination with specifically selected force fields leads to 665 (**120**) and 601 (**120**Me) conformations. Some of these conformations are eliminated on geometry optimizations at DFT level, but the final low-energy window of 10 kJ/mol for "good" structures still contains 139 (**120**) and 94 (**120**Me) structures (after the elimination of mirror-image conformers). A reliable calculation of Boltzmann-averaged thermochemical data and the identification of the best conformers thus requires frequency calculations and MP2 single point calculations for all of these structures. It should be added that the energetically best structure varies on moving from *E*_tot_(DFT) to *H*_298_(DFT) to *H*_298_(MP2). At this last level of theory two close-lying all-*trans* conformations can be found for P*n*-Bu_3_ (**120**) as depicted in Figure 14.

**Figure 15:**
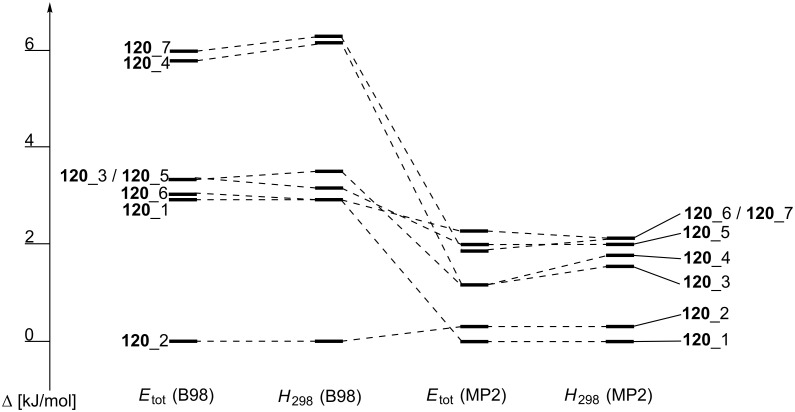
Relative order of the conformations **120**_1 to **120**_7 depending on the level of theory.

For the sake of clarity only the seven best conformations are shown in Figure 15.

**Figure 16:**
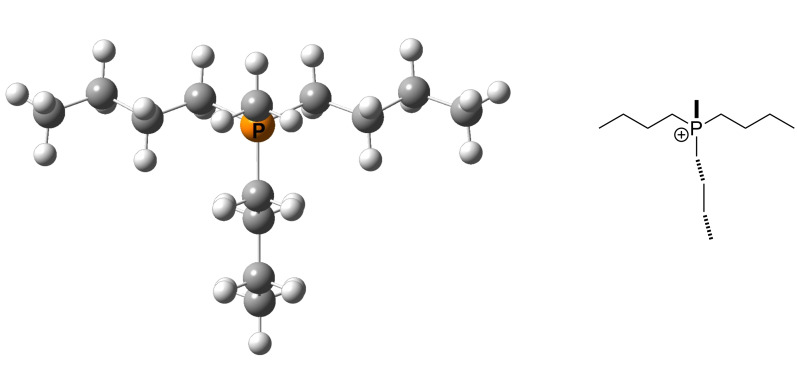
The structure of the energetically best conformations of **120**Me.

The eventually best conformation **120**_1 is less favorable by 3 kJ/mol as compared to conformation **120**_2 when using total energies (*E*_tot_) or enthalpies at 298 K (*H*_298_) obtained at B98/6-31G(d) level of theory. Moving to the MP2(FC)/6-31+G(2d,p)// B98/6-31G(d) energies or enthalpies the difference shrinks to 0.3 kJ/mol, now with **120**_1 as the more stable structure. The reduction of energy differences on moving from DFT to MP2 single point energies is a rather general phenomenon observed in these studies. This implies that the definition of, for example, an energy window of 10 kJ/mol for conformational selection has different implications at these different levels of theory. Conformational preferences can, of course, also be quite different for the neutral Lewis base and its methyl cation adduct. For phosphane **120** we find that conformation **120**Me_1 (Figure 16) has the lowest *E*_tot_ on both levels of theory as well as the lowest *H*_298_.

All calculated MCA values employ Boltzmann-averaging over all available conformations within a 15 kJ/mol (10 kJ/mol for P*n*-Bu_3_ (**120**)) energy window. The Boltzmann-averaged MCA value of P*n*-Bu_3_ (**120**) thus amounts to +639.5 kJ/mol. Taking only the energetically best conformations in each case (**120** and **120**Me) into account, the MCA value amounts to +639.2 kJ/mol. For this particular system the Boltzmann averaging procedure thus offers no notable benefit for the calculation of MCA values, but this can change depending on the systems under study. The most relevant role of extensive conformational searches is therefore that of the identification of the best conformation of the Lewis base as well as the cationic adduct (LB^+^-methyl, LB^+^-benzhydryl, LB^+^-trityl, LB^+^-MOSCA, LB^+^-acetyl). Unfortunately, the actual conformational rank depends on the used level of theory, especially if dispersion interactions play an important role. This problem will gain more relevance when the steric demand is large. In the present study it can be neglected due to the use of MP2 single point calculations and the fact that, even in the case of TCA (trityl cation affinity) values as the sterically most demanding electrophile, the important minima could be found at the B98 level of theory.

## Conclusion

Affinity data towards selected electrophiles provide the means to quantify Lewis bases with respect to their carbon basicity. This complements the limited amount of experimental affinity data and provides a quantitative guideline in catalyst development projects in which the addition of Lewis bases to carbon electrophiles represents the key step of the catalytic cycle.

## Supporting Information

File 1 File Typ: PDF.
Energies, enthalpies and geometries for all Lewis bases and their respective adducts with various electrophiles.
