# Supplementary material for: Cation affinity numbers of Lewis bases
Source: Beilstein J Org Chem. 2012 Aug 31;8:1406–42. doi: 10.3762/bjoc.8.163 (PMC3458768; doi:10.3762/bjoc.8.163)
Supplement: File 1 — File Typ: PDF. Energies, enthalpies and geometries for all Lewis bases and their respective adducts with various electrophiles. [file Beilstein_J_Org_Chem-08-1406-s001.pdf]

Supporting Information

for

Cation affinity numbers of Lewis bases

Christoph Lindner, Raman Tandon, Boris Maryasin, Evgeny Larionov and Hendrik Zipse*

Address: Department of Chemistry, Ludwigs-Maximilians-Universität München, Butenandstr. 5–13, D-81377 München, Germany

Email: Hendrik Zipse - zipse@cup.uni-muenchen.de

*Corresponding author

Energies, enthalpies and geometries for all Lewis bases and their respective adducts with various electrophiles

References

1. Larionov, E.; Zipse, H. *WIRES Comp. Mol. Sci.* **2011,** *1*, 601–619.
2. Denmark, S.E.; Beutner, G.L. *Angew. Chem. Int. Ed.* **2008,** *47*, 1560–1638.
3. de Rycke, N.; Couty, F.; David, O.R.P. *Chem. Eur. J.* **2011,** *17*, 12852–12871.
4. Wei, Y.; Sastry, G.N.; Zipse, H. *J. Am. Chem. Soc.* **2008,** *130*, 3473–3477.
5. Wei, Y.; Singer, T.; Mayr, H.; Sastry, G.N.; Zipse, H. *J. Comp. Chem*. **2008,** *29*, 291–297.
6. Wei, Y.; Sateesh, B.; Maryasin, B.; Sastry, G.N.; Zipse, H. *J. Comp. Chem.* **2009,** 2617–2624.
7. Müller, C.E.; Schreiner, P.R. *Angew. Chem. Int. Ed.* **2011,** *50*, 6012–6042.
8. Held, I.; Xu, S.; Zipse, H. *Synthesis* **2007,** 1185–1196.
9. Larionov, E.; Achrainer, F.; Humin, J.; Zipse, H. *ChemCatChem* **2012,** *4*, 559–566.
10. D'Elia, V.; Liu, Y.; Zipse, H. *Eur. J. Org. Chem.* **2011,** 1527–1533.
11. Held, I.; von den Hoff, P.; Stephenson, D.S.; Zipse, H. *Adv. Synth. Catal.* **2008,** *350*, 1891–1900.
12. Heinrich, M.R.; Klisa, H.S.; Mayr, H.; Steglich, W.; Zipse, H. *Angew. Chem.* **2003,** *115*, 4975–4977. *Angew. Chem. Int. Ed.* **2003,** *42*, 4826–4828.
13. Lindner, C.; Maryasin, B.; Richter, F.; Zipse, H. *J. Phys. Org. Chem.* **2010,** *23*, 1036–1042.
14. Enders, D.; Niemeier, O.; Henseler, A. *Chem. Rev.* **2007**, *107*, 5606–5655.
15. Marion, N.; Diez-Gonzalez, S.; Nolan, S. P. *Angew. Chem. Int. Ed. Engl.* **2007,** *46*, 2988–3000.
16. Nair, V.; Vellalath, S.; Babu, B. P. *Chem. Soc. Rev.* **2008**, *37*, 2691–2698.
17. Grossmann, A.; Enders, D. *Angew. Chem. Int. Ed. Engl.* **2012,** *51*, 314–325.
18. Maji, B.; Breugst, M.; Mayr, H. *Angew. Chem. Int. Ed.* **2011,** *50*, 6915–6919.
19. Bug, T.; Gotta, M.F.; Hering, N.; Irrgang, B.; Janker, B.; Kempf, B.; Loos, R.; Ofial, A.R.; Remennikov, G.; Schimmel, H.; Mayr, H. *J. Am. Chem. Soc.* **2001,** *123*, 9500–9512.
20. Patz, M.; Mayr, H. *Angew. Chem.* **1994,** *106*, 990–1010; *Angew. Chem. Int. Ed. Engl.* **1994,** *33*, 938–957.
21. Breugst, M.; Zipse, H.; Guthrie, J. P.; Mayr, H. *Angew. Chem. Int. Ed.* **2010**, *49*, 5165–5169.
22. Baidya, M.; Horn, M.; Zipse, H.; Mayr, H. *J. Org. Chem.* **2009,** 7157–7164.
23. Brotzel, F., Kempf, B.; Singer, T.; Zipse, H.; Mayr, H. *Chem. Eur. J.* **2007**, *13*, 336–345.
24. Kempf, B.; Mayr, H. *Chem. Eur. J.* **2005**, *11*, 917–927.
25. Zhu, X.-Q.; Wang, C.-H. *J. Phys. Chem. A* **2010**, *114*, 13244–13256.
26. Cheng, J.-P.; Handoo, K. L., Parker, V. D. *J. Am. Chem. Soc.* **1993**, *115*, 2655–2660.
27. Baidya, M.; Kobayashi, S.; Brotzel, F.; Schmidhammer, U.; Riedle, E.; Mayr, H. *Angew. Chem. Int. Ed.* **2007**, *46*, 6176–6179.
28. Seco, J. M.; Quinoa, E.; Riguera, R. *Chem. Rev.* **2004**, *104*, 17–118.
29. Wei, Y.; Sastry, G.N.; Zipse, H. *Org. Lett.* **2008,** *10*, 5413–5417.
30. Lindner, C.; Tandon, R.; Liu, Y.; Maryasin, B.; Zipse, H. *Org. Biomol. Chem.* **2012,** *10*, 3210–3218.
31. Hiratake, J.; Yamamoto, Y.; Oda, J. *J. Chem. Soc., Chem. Commun.* **1985**, 1717.
32. Hiratake, J.; Inagaki M.; Yamamoto, Y.; Oda, J. *J. Chem. Soc., Perkin Trans. 1* **1987**, 1053.
33. Aitken, R. A.; Gopal, J.; Hirst, J. A. *J. Chem. Soc., Chem. Commun.* **1988**, 632.
34. Aitken, R. A.; Gopal, J. *Tetrahedron: Asymmetry* **1990**, *1*, 513.
35. Bolm, C.; Gerlach A.; Dinter, C. L. *Synlett* **1999**, 195–196.
36. Bolm, C.; Schiffers, I.; Dinter, C. L.; Gerlach, A. *J. Org. Chem.* **2000**, *65,* 6984–6991.
37. Bolm, C.; Schiffers, I.; Atodiresei, I.; Ozcubukcu, S.; Raabe, G. *New J. Chem.* **2003**, *27*, 14.
38. Bolm, C.; Schiffers, I.; Atodiresei, I.; Hackenberger, C. P. R. *Tetrahedron: Asymmetry* **2003**, *14*, 3455–3467.
39. Bolm, C.; Atodiresei, I.; Schiffers, I. *Org. Synth.* **2005**, *82*, 120–124.
40. Atodiresei, I.; Schiffers, I.; Bolm, C. *Chem. Rev.* **2007**, *107*, 5683–5712.

41. Vedejs, E.; Jure, M. *Angew. Chem. Int. Ed.* **2005**, *44*, 3974–4001.
42. Wurz, R. *Chem. Rev.* **2007**, *107*, 5570–5595.
43. Spivey, A. C.; Arseniyadis, S. *Top. Curr. Chem.* **2010**, *291*, 233.
44. Held, I.; Villinger, A.; Zipse, H. *Synthesis* **2005,** 1425–1426.
45. Wei, Y.; Held, I; Zipse, H. *Org. Biomol. Chem*. **2006,** *4*, 4223–4230.
46. Held, I.; Larionov, E.; Bozler, C.; Wagner, F.; Zipse, H. *Synthesis* **2009,** 2267–2277.
47. De Rycke, N.; Berionni, G.; Couty, F.; Mayr, H.; Goumont, R.; David, O. R. P. *Org. Lett.* **2011**, *13*, 530–533.
48. Schrödinger, LLC., MacroModel 9.7, 2009.
49. M. J. Frisch, G. W. Trucks, H. B. Schlegel, G. E. Scuseria, M. A. Robb, J. R. Cheeseman, J. A. Montgomery, Jr, T. Vreven, K. N. Kudin, J. C. Burant, J. M. Millam, S. S. Iyengar, J. Tomasi, V. Barone, B. Mennucci, M. Cossi, G. Scalmani, N. Rega, G. A. Petersson, H. Nakatsuji, M. Hada, M. Ehara, K. Toyota, R. Fukuda, J. Hasegawa, M. Ishida, T. Nakajima, Y. Honda, O. Kitao, H. Nakai, M. Klene, X. Li, J. E. Knox, H. P. Hratchian, J. B. Cross, V. Bakken, C. Adamo, J. Jaramillo, R. Gomperts, R. E. Stratmann, O. Yazyev, A. J. Austin, R. Cammi, C. Pomelli, J. W. Ochterski, P. Y. Ayala, K. Morokuma, G. A. Voth, P. Salvador, J. J. Dannenberg, V. G. Zakrzewski, S. Dapprich, A. D. Daniels, M. C. Strain, O. Farkas, D. K. Malick, A. D. Rabuck, K. Raghavachari, J. B. Foresman, J. V. Ortiz, Q. Cui, A. G. Baboul, S. Clifford, J. Cioslowski, B. B. Stefanov, G. Liu, A. Liashenko, P. Piskorz, I. Komaromi, R. L. Martin, D. J. Fox, T. Keith, M. A. Al-Laham, C. Y. Peng, A. Nanayakkara, M. Challacombe, P. M. W. Gill, B. Johnson, W. Chen, M. W. Wong, C. Gonzalez, J. A. Pople, Gaussian 03, Revision D.01, Gaussian, Inc., Wallingford CT, 2004.

COMPUTATIONAL METHODS (MCA)

Methyl cation affinities of Lewis bases (LB) have been calculated as the reaction enthalpy at 298.15 K and 1 atm pressure for the methyl cation detachment reaction shown in equation (S1). This is in analogy to the mass spectrometric definition of proton affinities.

$$LB^{+}\text{-}CH_3 \;\; \text{-->} \;\; LB \; + \; CH_3^{+} \qquad \text{(S1)}$$

The geometries of all species in equation (S1) have been optimized at the B98/6-31G(d) level of theory. The conformational space of flexible Lewis bases and the corresponding cations have been searched using the MM3* force field and the systematic search routine implemented in MACROMODEL 9.7 [48]. All stationary points located at force field level have then been reoptimized at B98/6-31G(d) level as described before. Thermochemical corrections to 298.15 K have been calculated for all minima from unscaled vibrational frequencies obtained at this same level. The thermochemical corrections have been combined with single point energies calculated at the MP2(FC)/6-31+G(2d,p)//B98/6-31G(d) level to yield enthalpies H_{298} at 298.15 K. In conformationally flexible systems enthalpies have been calculated as Boltzmann-averaged values over all available conformers. This procedure has recently been found to reproduce G3 methyl cation affinity values of selected small and medium sized organocatalysts within 4.0 kJ/mol [5]. All quantum mechanical calculations have been performed with Gaussian 03 [49].

Table S1. Total energies and enthalpies (in Hartree) as calculated at the B98/6-31G(d) and MP2(FC)/6-31+G(2d,p)// B98/6-31G(d) level of theory for all systems (MCA). If more than one conformer exist at 298.15 K, the single values of each conformer are denoted as well as the Boltzmann-averaged values for H_{298} at MP2(FC)/6-31+G(2d,p)//B98/6-31G(d) level of theory. Only conformers are included with a Boltzmann-weighting of at least 1% (rounded) up to a maximum to ten conformers per system.

	B98/6-31G(d)		MP2(FC)/6-31+G(2d,p)// B98/6-31G(d)	
system	E_{tot}	H_{298}	E_{tot}	"H_{298}"
**CH_3^+**	-39.462922	-39.427481	-39.352370	-39.316929
**Ph_2CH^+**	-501.542782	-501.332153	-500.293523	-500.082894
**Ph_3C^+**	-732.51950	-732.22340	-730.722779	-730.426679
**1**	-248.181760	-248.087612	-247.5894387	-247.495291
**1-Me$^+$**	-287.856015	-287.71821	-287.147570	-287.009765
**2**	-56.524639	-56.486143	-56.408195	-56.369698
**2-Me$^+$**	-96.176646	-96.092230	-95.937308	-95.852893
**3**	-95.813081	-95.744235	-95.581368	-95.512522
**3-Me$^+$**	-135.479017	-135.364861	-135.129746	-135.015590
**4**	-557.72361	-557.48472	-556.36540	-556.126519
**4-Me$^+$**	-597.38371	-597.10052	-595.92255	-595.639365
**5**				-405.193721
5_1	-406.454167	-406.220014	-405.427963	-405.193810
5_2	-406.451066	-406.216817	-405.424634	-405.190385
**5-Me$^+$**				-444.709158
5-Me$^+$_1	-446.121673	-445.842894	-444.988334	-444.709555

5-Me$^+$_2	-446.120928	-445.841935	-444.987989	-444.708996
5-Me$^+$_3	-446.119721	-445.840829	-444.986595	-444.707702
5-Me$^+$_4	-446.118771	-445.840031	-444.985216	-444.706475
**6**	-645.957853	-645.474499	-644.269472	-643.786117
**6-Me$^+$**				-683.301613
6-Me$^+$_1	-685.626625	-685.097942	-683.830723	-683.302040
6-Me$^+$_2	-685.625711	-685.097135	-683.830174	-683.301598
6-Me$^+$_3	-685.624884	-685.096331	-683.829627	-683.301074
6-Me$^+$_4	-685.624256	-685.095664	-683.827763	-683.299171
6-Me$^+$_5	-685.622657	-685.094135	-683.827248	-683.298726
**7**	-135.105880	-135.007566	-134.762063	-134.663749
**7-Me$^+$**	-174.779435	-174.636053	-174.323314	-174.179932
**8**	-329.105074	-328.906539	-328.269758	-328.071223
**8-Me$^+$**				-367.587571
8-Me$^+$_1	-368.774595	-368.531311	-367.831115	-367.587832
8-Me$^+$_2	-368.773928	-368.530490	-367.831009	-367.587571
8-Me$^+$_3	-368.772970	-368.529666	-367.829774	-367.586470
**9**	-366.06253	-365.87871	-365.15454	-364.970725
**9-Me$^+$**	-405.72909	-405.50116	-404.71658	-404.488644
**10**				-526.294674
10_1	-528.080138	-527.685177	-526.690056	-526.295096
10_2	-528.076845	-527.681959	-526.687562	-526.292676
10_3	-528.077603	-527.682609	-526.687647	-526.292652
10_4	-528.076035	-527.680754	-526.686504	-526.291223
10_5	-528.076813	-527.681748	-526.685957	-526.290892
**10-Me$^+$**				-565.813255
10-Me$^+$_1	-567.751267	-567.310578	-566.254895	-565.814206
10-Me$^+$_2	-567.750546	-567.310031	-566.254531	-565.814016
10-Me$^+$_3	-567.751198	-567.310654	-566.254542	-565.813998
10-Me$^+$_4	-567.750720	-567.310224	-566.254463	-565.813966
10-Me$^+$_5	-567.750319	-567.309589	-566.254670	-565.813940
10-Me$^+$_6	-567.750439	-567.309658	-566.254538	-565.813757
10-Me$^+$_7	-567.750387	-567.309949	-566.254122	-565.813683
10-Me$^+$_8	-567.750486	-567.309752	-566.254334	-565.813600
10-Me$^+$_9	-567.749937	-567.309256	-566.254196	-565.813514
10-Me$^+$_10	-567.750426	-567.309904	-566.254013	-565.813491
**11**				-487.130603
11_1	-488.774201	-488.409658	-487.495342	-487.130799
11_2	-488.772538	-488.408274	-487.492873	-487.128608
**11-Me$^+$**				-526.649802
11-Me$^+$_1	-528.445506	-528.035950	-527.059775	-526.650218
11-Me$^+$_2	-528.445402	-528.035596	-527.059736	-526.649930
11-Me$^+$_3	-528.444760	-528.034724	-527.059410	-526.649374
11-Me$^+$_4	-528.445508	-528.035830	-527.058985	-526.649308
11-Me$^+$_5	-528.444122	-528.034431	-527.057396	-526.647704
**12**	-226.12498	-226.048948	-225.615954	-225.539920
**12-Me$^+$**	-265.806633	-265.686827	-265.179165	-265.059358
**13**	-251.753775	-251.590813	-251.109801	-250.946838

**13-Me$^+$**	-291.425967	-291.218266	-290.674089	-290.466388
**14**	-287.484718	-287.361209	-286.787285	-286.663777
**14-Me$^+$**	-327.165207	-326.998151	-326.350697	-326.183641
**15**	-331.588769	-331.343111	-330.722696	-330.477035
**15-Me$^+$**	-371.262513	-370.971721	-370.288870	-369.998078
**16**	-410.181157	-409.876229	-409.104874	-408.799946
**16-Me$^+$**				-448.321018
16-Me$^+$_1	-449.851719	-449.500274	-448.672679	-448.321235
16-Me$^+$_2	-449.849752	-449.498228	-448.670540	-448.319016
**17**				-408.802151
17_1	-410.187140	-409.881322	-409.108376	-408.802558
17_2	-410.187257	-409.881207	-409.108277	-408.802227
17_3	-410.184904	-409.879068	-409.106537	-408.800701
17_4	-410.183720	-409.877876	-409.105336	-408.799492
17_5	-410.183025	-409.876824	-409.105001	-408.798800
**17-Me$^+$**				-448.324083
17-Me$^+$_1	-449.861093	-449.509913	-448.676352	-448.325172
17-Me$^+$_2	-449.860903	-449.509725	-448.675519	-448.324340
17-Me$^+$_3	-449.860676	-449.509657	-448.675311	-448.324292
17-Me$^+$_4	-449.861008	-449.509982	-448.675310	-448.324284
17-Me$^+$_5	-449.860238	-449.509075	-448.675361	-448.324198
17-Me$^+$_6	-449.860458	-449.509353	-448.675263	-448.324158
17-Me$^+$_7	-449.860961	-449.509787	-448.675326	-448.324151
17-Me$^+$_8	-449.861060	-449.509979	-448.675227	-448.324146
17-Me$^+$_9	-449.861126	-449.510127	-448.675136	-448.324137
17-Me$^+$_10	-449.860524	-449.509392	-448.674938	-448.323806
**18**	-212.496424	-212.3601084	-211.9581467	-211.821913
**18-Me$^+$**	-252.178340	-251.997101	-251.525726	-251.344445
**19**				-291.311498
19_1	-292.294134	-292.077474	-291.528261	-291.311601
19_2	-292.290234	-292.073520	-291.524508	-291.307793
19_3	-292.289341	-292.072551	-291.523585	-291.306796
**19-Me$^+$**				-330.834689
19-Me$^+$_1	-331.969738	-331.708056	-331.096480	-330.834798
19-Me$^+$_2	-331.969804	-331.708036	-331.096345	-330.834577
**20**	-174.400713	-174.273290	-173.948653	-173.821230
**20-Me$^+$**	-214.077147	-213.904848	-213.517463	-213.517463
**21**				-526.241030
21_1	-528.007710	-527.612937	-526.635957	-526.241184
21_2	-528.004695	-527.609840	-526.633418	-526.238563
**21-Me$^+$**	-567.681960	-567.242068	-566.205611	-565.765719
**22**				-212.984070
22_1	-213.698589	-213.541330	-213.141523	-212.984264
22_2	-213.697397	-213.540150	-213.139297	-212.982050
**22-Me$^+$**	-253.377344	-253.174990	-252.712316	-252.509962
**23**	-410.163134	-409.858040	-409.096397	-408.791302
**23-Me$^+$**	-449.841249	-449.490532	-448.668201	-448.317484
**24**	-265.420879	-265.315183	-264.803119	-264.697423

**24-Me$^+$**	-305.108648	-304.959284	-304.373200	-304.223836
**25**				-290.113868
25_1	-291.059244	-290.865696	-290.307530	-290.113982
25_2	-291.055574	-290.862043	-290.303559	-290.110028
**25_3**	-291.056453	-290.862761	-290.303658	-290.109966
25-Me$^+$	-330.738860	-330.500298	-329.879303	-329.640741
**26**				-252.147704
26_1	-252.994007	-252.807082	-252.334954	-252.148029
26_2	-252.995120	-252.808395	-252.334284	-252.147559
26_3	-252.994629	-252.807839	-252.333770	-252.146979
**26-Me$^+$**	-292.674299	-292.442481	-291.906580	-291.674762
**27**				-252.145735
27_1	-252.995927	-252.808640	-252.333345	-252.146058
27_2	-252.995727	-252.808459	-252.332791	-252.145523
27_3	-252.994565	-252.807404	-252.330674	-252.143514
27_4	-252.991819	-252.804538	-252.329100	-252.141819
**27-Me$^+$**				-291.672929
27-Me$^+$_1	-292.675765	-292.443554	-291.905198	-291.672987
27-Me$^+$_2	-292.671494	-292.439496	-291.900997	-291.669000
**28**				-291.309110
28_1	-292.29233	-292.07563	-291.52637	-291.309674
28_2	-292.29001	-292.07297	-291.52608	-291.309038
28_3	-292.29109	-292.07444	-291.52496	-291.308307
28_4	-292.29094	-292.07416	-291.52486	-291.308076
28_5	-292.28870	-292.07192	-291.52391	-291.307138
**28-Me$^+$**				-330.836346
28-Me$^+$_1	-331.97094	-331.70910	-331.09833	-330.836493
28-Me$^+$_2	-331.96768	-331.70590	-331.09520	-330.833416
28-Me$^+$_3	-331.96658	-331.70443	-331.09405	-330.831903
**29**	-292.289289	-292.073448	-291.528877	-291.313036
**29-Me$^+$**	-331.969170	-331.708094	-331.101524	-330.840448
**30**				-291.307341
30_1	-292.293268	-292.076150	-291.524989	-291.307870
30_2	-292.292433	-292.075168	-291.525054	-291.307789
30_3	-292.293010	-292.075865	-291.524285	-291.307141
30_4	-292.291647	-292.074475	-291.523312	-291.306140
30_5	-292.291646	-292.074394	-291.523129	-291.305877
30_6	-292.291059	-292.073795	-291.522687	-291.305422
30_7	-292.291874	-292.074767	-291.522127	-291.305020
30_8	-292.291300	-292.074310	-291.521819	-291.304828
**30-Me$^+$**				-330.835192
30-Me$^+$_1	-331.974036	-331.711866	-331.097678	-330.835508
30-Me$^+$_2	-331.972716	-331.710593	-331.096728	-330.834604
30-Me$^+$_3	-331.969849	-331.707793	-331.093748	-330.831692
**31**				-329.303502
31_1	-330.381938	-330.157303	-329.528223	-329.303588
31_2	-330.379618	-330.154953	-329.524800	-329.300136
**31-Me$^+$**				-368.831571

31-Me$^+$_1	-370.062714	-369.793206	-369.101276	-368.831768
31-Me$^+$_2	-370.062896	-369.793025	-369.101090	-368.831218
**32**				-330.468969
31_1	-331.589781	-331.342595	-330.716844	-330.469658
32_2	-331.589775	-331.342605	-330.716821	-330.469651
32_3	-331.590641	-331.343640	-330.716626	-330.469624
32_4	-331.590397	-331.343413	-330.715857	-330.468873
32_5	-331.588659	-331.341341	-330.716129	-330.468811
32_6	-331.589355	-331.342295	-330.715700	-330.468640
32_7	-331.589483	-331.342425	-330.715668	-330.468610
32_8	-331.589260	-331.342143	-330.715135	-330.468017
32_9	-331.589140	-331.342092	-330.714992	-330.467944
32_10	-331.589099	-331.341872	-330.714994	-330.467767
**32-Me$^+$**				-369.997104
32-Me$^+$_1	-371.271966	-370.979944	-370.289784	-369.997762
32-Me$^+$_2	-371.270834	-370.978678	-370.289056	-369.996899
32-Me$^+$_3	-371.270627	-370.978555	-370.288947	-369.996875
32-Me$^+$_4	-371.269594	-370.977447	-370.288548	-369.996401
32-Me$^+$_5	-371.267752	-370.975781	-370.285886	-369.993915
32-Me$^+$_6	-371.267545	-370.975327	-370.286120	-369.993901
**33**				-446.778957
33_1	-448.261578	-447.945889	-447.095517	-446.779828
33_2	-448.261770	-447.946271	-447.095239	-446.779739
33_3	-448.263034	-447.947834	-447.094332	-446.779132
33_4	-448.261205	-447.945978	-447.094342	-446.779115
33_5	-448.262995	-447.947762	-447.094178	-446.778945
33_6	-448.262905	-447.947798	-447.094034	-446.778927
33_7	-448.262831	-447.947670	-447.094014	-446.778852
33_8	-448.262633	-447.947412	-447.093446	-446.778224
33_9	-448.261824	-447.946615	-447.093056	-446.777847
33_10	-448.262010	-447.946670	-447.093033	-446.777693
**33-Me$^+$**				-486.310923
33-Me$^+$_1	-487.946535	-487.586309	-486.671769	-486.311543
33-Me$^+$_2	-487.945042	-487.584876	-486.670045	-486.309879
33-Me$^+$_3	-487.944582	-487.584498	-486.669550	-486.309466
33-Me$^+$_4	-487.943351	-487.582950	-486.668347	-486.307946
33-Me$^+$_5	-487.943269	-487.583242	-486.667415	-486.307388
33-Me$^+$_6	-487.942795	-487.582486	-486.667497	-486.307187
33-Me$^+$_7	-487.942849	-487.582218	-486.667781	-486.307150
33-Me$^+$_8	-487.941923	-487.581738	-486.667191	-486.307006
33-Me$^+$_9	-487.943252	-487.582661	-486.667525	-486.306934
**34**				-408.797249
34_1	-410.18207	-409.87614	-409.10391	-408.797982
34_2	-410.17956	-409.87319	-409.10401	-408.797641
34_3	-410.17896	-409.87266	-409.10272	-408.796426
34_4	-410.18059	-409.87467	-409.10218	-408.796259
34_5	-410.18102	-409.87509	-409.10193	-408.796000
34_6	-410.17859	-409.87223	-409.10236	-408.795998

34_7	-410.17949	-409.87347	-409.10191	-408.795887
34_8	-410.17844	-409.87203	-409.10088	-408.794479
34_9	-410.17754	-409.87142	-409.10041	-408.794300
34_10	-410.17818	-409.87204	-409.10034	-408.794199
**34-Me$^+$**				-448.325652
34-Me$^+$_1	-449.86070	-449.50953	-448.67713	-448.325962
34-Me$^+$_2	-449.85804	-449.50650	-448.67453	-448.322983
34-Me$^+$_3	-449.85683	-449.50504	-448.67359	-448.321795
34-Me$^+$_4	-449.85617	-449.50441	-448.67338	-448.321623
34-Me$^+$_5	-449.85518	-449.50359	-448.67265	-448.321060
**35**				-252.146550
35_1	-252.996188	-252.808995	-252.334172	-252.146979
35_2	-252.995944	-252.808694	-252.333789	-252.146538
35_3	-252.995178	-252.807876	-252.332530	-252.145229
35_4	-252.995391	-252.808165	-252.332439	-252.145213
**35-Me$^+$**				
35-Me$^+$_1	-292.677368	-292.444963	-291.907694	-291.675289
35-Me$^+$_2	-292.676595	-292.444289	-291.906567	-291.674261
35-Me$^+$_3	-292.672945	-292.440724	-291.903148	-291.670927
**36**				-369.630240
36_1	-370.887224	-370.610055	-369.908558	-369.631388
36_2	-370.888032	-370.611101	-369.908194	-369.631263
36_3	-370.886016	-370.608930	-369.907872	-369.630786
36_4	-370.885881	-370.608859	-369.907781	-369.630759
36_5	-370.886832	-370.609913	-369.907440	-369.630521
36_6	-370.886817	-370.609886	-369.907375	-369.630444
36_7	-370.887793	-370.610791	-369.907395	-369.630393
36_8	-370.886736	-370.609735	-369.907358	-369.630357
36_9	-370.886830	-370.609880	-369.907278	-369.630328
36_10	-370.887238	-370.610566	-369.906909	-369.630237
**36-Me$^+$**				-409.158701
36-Me$^+$_1	-410.569638	-410.247619	-409.481631	-409.159612
36-Me$^+$_2	-410.568452	-410.246457	-409.481011	-409.159016
36-Me$^+$_3	-410.568361	-410.246329	-409.480963	-409.158931
36-Me$^+$_4	-410.568462	-410.246396	-409.480829	-409.158763
36-Me$^+$_5	-410.567219	-410.245158	-409.480622	-409.158561
36-Me$^+$_6	-410.567352	-410.245396	-409.480517	-409.158561
36-Me$^+$_7	-410.567158	-410.245137	-409.480179	-409.158157
36-Me$^+$_8	-410.566284	-410.244073	-409.480184	-409.157973
36-Me$^+$_9	-410.567021	-410.245014	-409.479865	-409.157858
36-Me$^+$_10	-410.565377	-410.243293	-409.478484	-409.156401
**37**				-408.791728
37_1	-410.184600	-409.877513	-409.100116	-408.793028
37_2	-410.185399	-409.878647	-409.099722	-408.792970
37_3	-410.183016	-409.875712	-409.100119	-408.792815
37_4	-410.183387	-409.876365	-409.099473	-408.792450
37_5	-410.183296	-409.876264	-409.099420	-408.792388
37_6	-410.183277	-409.876275	-409.099350	-408.792347

37_7	-410.184095	-409.877368	-409.099024	-408.792297
37_8	-410.183303	-409.876378	-409.099204	-408.792278
37_9	-410.184102	-409.877224	-409.099011	-408.792133
37_10	-410.184151	-409.877351	-409.098921	-408.792121
**37-Me$^+$**				-448.320327
37-Me$^+$_1	-449.867203	-449.515313	-448.673375	-448.321485
37-Me$^+$_2	-449.866092	-449.514148	-448.672895	-448.320951
37-Me$^+$_3	-449.865929	-449.514011	-448.672765	-448.320846
37-Me$^+$_4	-449.865958	-449.514004	-448.672604	-448.320651
37-Me$^+$_5	-449.864931	-449.512863	-448.672690	-448.320621
37-Me$^+$_6	-449.865954	-449.514014	-448.672549	-448.320610
37-Me$^+$_7	-449.864914	-449.512873	-448.672381	-448.320340
37-Me$^+$_8	-449.863819	-449.511849	-448.672210	-448.320240
37-Me$^+$_9	-449.864844	-449.512903	-448.672081	-448.320139
37-Me$^+$_10	-449.863778	-449.511798	-448.672108	-448.320128
**38**	-760.255685	-759.746185	-758.287841	-757.778341
**38-Me$^+$**				-797.307399
38-Me$^+$_1	-799.930742	-799.375100	-797.863291	-797.307649
38-Me$^+$_2	-799.928961	-799.373257	-797.861453	-797.305750
38-Me$^+$_3	-799.925739	-799.370019	-797.858622	-797.302902
**39**				-330.473659
39_1	-331.586946	-331.340769	-330.720248	-330.474071
39_2	-331.587872	-331.341896	-330.719121	-330.473146
39_3	-331.587581	-331.341457	-330.718312	-330.472188
**39-Me$^+$**				-370.002851
39-Me$^+$_1	-371.268167	-370.977269	-370.293797	-370.002899
39-Me$^+$_2	-371.264022	-370.972439	-370.289669	-369.998086
**40**				-407.623739
40_1	-408.971591	-408.686217	-407.910238	-407.624864
40_2	-408.972656	-408.687566	-407.908490	-407.623400
40_3	-408.971664	-408.686520	-407.908490	-407.623346
40_4	-408.970383	-408.684973	-407.908603	-407.623193
40_5	-408.972488	-408.687426	-407.908255	-407.623192
40_6	-408.970633	-408.685289	-407.908464	-407.623120
40_7	-408.972089	-408.687075	-407.908133	-407.623119
40_8	-408.972355	-408.687286	-407.908169	-407.623100
40_9	-408.972441	-408.687394	-407.908120	-407.623074
40_10	-408.972292	-408.687218	-407.907935	-407.622861
**40-Me$^+$**				-447.154063
40-Me$^+$_1	-448.655192	-448.324957	-447.484832	-447.154597
40-Me$^+$_2	-448.654008	-448.324028	-447.483726	-447.153746
40-Me$^+$_3	-448.654009	-448.323831	-447.483570	-447.153391
40-Me$^+$_4	-448.653982	-448.323733	-447.483280	-447.153031
**41**				-406.405932
41_1	-407.715774	-407.455967	-406.665927	-406.406120
41_2	-407.714775	-407.454776	-406.663384	-406.403385
41_3	-407.712277	-407.452211	-406.661601	-406.401535
**41-Me$^+$**				-445.936305

41-Me+_1	-447.399388	-447.094446	-446.241647	-445.936705
41-Me+_2	-447.398111	-447.093133	-446.240300	-445.935321
41-Me+_3	-447.397024	-447.092025	-446.239482	-445.934483
**42**				-368.472220
42_1	-369.684059	-369.428785	-368.727642	-368.472368
42_2	-369.684719	-369.429888	-368.727086	-368.472255
42_3	-369.686046	-369.431270	-368.726909	-368.472133
42_4	-369.682933	-369.428978	-368.723246	-368.469291
**42-Me+**	-409.368491	-409.068533	-408.302806	-408.002848
**43**				-330.470276
43_1	-331.590911	-331.343838	-330.718073	-330.471000
43_2	-331.590773	-331.343706	-330.718021	-330.470954
43_3	-331.590686	-331.343625	-330.717301	-330.470240
43_4	-331.590605	-331.343610	-330.717176	-330.470180
43_5	-331.590543	-331.343624	-330.717078	-330.470159
43_6	-331.590403	-331.343434	-330.716712	-330.469743
43_7	-331.590265	-331.343318	-330.716482	-330.469535
43_8	-331.590094	-331.343014	-330.716007	-330.468926
43_9	-331.589563	-331.342372	-330.716074	-330.468883
43_10	-331.588092	-331.341131	-330.715430	-330.468470
**43-Me+**				-370.001143
43-Me+_1	-371.273874	-370.981806	-370.293794	-370.001726
43-Me+_2	-371.273236	-370.981013	-370.292466	-370.000243
43-Me+_3	-371.273141	-370.980872	-370.292038	-369.999769
43-Me+_4	-371.269771	-370.977538	-370.289536	-369.997303
43-Me+_5	-371.269380	-370.977224	-370.289222	-369.997066
43-Me+_6	-371.269339	-370.977364	-370.289034	-369.997059
43-Me+_7	-371.269339	-370.977363	-370.289034	-369.997058
**44**	-345.1949681	-345.003513	-344.3461625	-344.1547074
**44-Me+**	-384.8798256	-384.643824	-383.9217601	-383.6857585
**45**				-291.308664
45_1	-292.293464	-292.076422	-291.526248	-291.309206
45_2	-292.292501	-292.075247	-291.525495	-291.308241
45_3	-292.292664	-292.075663	-291.525122	-291.308121
45_4	-292.291949	-292.074997	-291.524938	-291.307986
**45-Me+**				-330.839733
45-Me+_1	-331.976407	-331.714256	-331.102305	-330.840154
45-Me+_2	-331.975671	-331.713551	-331.101396	-330.839276
45-Me+_3	-331.974812	-331.712797	-331.100176	-330.838160
**46**				-408.793788
46_1	-410.184098	-409.876925	-409.102082	-408.794908
46_2	-410.184928	-409.877821	-409.101916	-408.794809
46_3	-410.184510	-409.877368	-409.101949	-408.794808
46_4	-410.184654	-409.877595	-409.101852	-408.794793
46_5	-410.185610	-409.878789	-409.101527	-408.794706
46_6	-410.185490	-409.878599	-409.101551	-408.794660
46_7	-410.183463	-409.876341	-409.101223	-408.794101
46_8	-410.184412	-409.877224	-409.100856	-408.793668

46_9	-410.185302	-409.878505	-409.100463	-408.793665
46_10	-410.184527	-409.877347	-409.100828	-408.793649
**46-Me$^+$**				-448.325569
46-Me$^+$_1	-449.870241	-449.518227	-448.678830	-448.326816
46-Me$^+$_2	-449.869066	-449.517027	-448.678061	-448.326023
46-Me$^+$_3	-449.868996	-449.516910	-448.677873	-448.325787
46-Me$^+$_4	-449.869588	-449.517561	-448.677444	-448.325417
46-Me$^+$_5	-449.867835	-449.515801	-448.677228	-448.325194
46-Me$^+$_6	-449.869532	-449.517485	-448.676977	-448.324930
46-Me$^+$_7	-449.867863	-449.515524	-448.677031	-448.324692
46-Me$^+$_8	-449.868233	-449.516193	-448.676494	-448.324454
46-Me$^+$_9	-449.868314	-449.516180	-448.676489	-448.324355
46-Me$^+$_10	-449.867850	-449.515301	-448.676903	-448.324354
**47**				-408.795631
47_1	-410.185566	-409.878883	-409.103662	-408.796979
47_2	-410.185704	-409.879031	-409.102767	-408.796094
47_3	-410.185678	-409.878914	-409.101817	-408.795053
47_4	-410.185686	-409.878910	-409.101811	-408.795035
47_5	-410.183956	-409.877272	-409.101416	-408.794732
47_6	-410.184805	-409.877978	-409.101500	-408.794673
47_7	-410.184127	-409.877117	-409.101577	-408.794566
47_8	-410.185389	-409.878732	-409.101005	-408.794348
47_9	-410.184164	-409.877022	-409.101410	-408.794269
47_10	-410.184149	-409.877354	-409.100760	-408.793964
**47-Me$^+$**				-448.328715
47-Me$^+$_1	-449.870978	-449.518782	-448.681516	-448.329320
47-Me$^+$_2	-449.870378	-449.518140	-448.680574	-448.328337
47-Me$^+$_3	-449.869446	-449.517336	-448.679143	-448.327033
47-Me$^+$_4	-449.866543	-449.514467	-448.677263	-448.325187
47-Me$^+$_5	-449.866338	-449.514332	-448.677139	-448.325133
47-Me$^+$_6	-449.866288	-449.514044	-448.677081	-448.324838
**48**				-640.261217
48_1	-642.338217	-641.919158	-640.681075	-640.262016
48_2	-642.337871	-641.919008	-640.680192	-640.261330
48_3	-642.337862	-641.918834	-640.679975	-640.260947
48_4	-642.337800	-641.918761	-640.679960	-640.260921
48_5	-642.337746	-641.918573	-640.680089	-640.260916
48_6	-642.334373	-641.915045	-640.679991	-640.260663
48_7	-642.334332	-641.914964	-640.679825	-640.260457
48_8	-642.334062	-641.914550	-640.679152	-640.259640
48_9	-642.333917	-641.914441	-640.679080	-640.259604
48_10	-642.332889	-641.913685	-640.676753	-640.257549
**48-Me$^+$**				-679.794586
48-Me$^+$_1	-682.018801	-681.553600	-680.260422	-679.795221
48-Me$^+$_2	-682.017798	-681.552843	-680.259826	-679.794870
48-Me$^+$_3	-682.018537	-681.553303	-680.260060	-679.794826
48-Me$^+$_4	-682.016995	-681.551770	-680.259021	-679.793796
48-Me$^+$_5	-682.016279	-681.551145	-680.258310	-679.793177

48-Me$^+$_6	-682.015586	-681.550819	-680.257623	-679.792856
48-Me$^+$_7	-682.015180	-681.550300	-680.257392	-679.792512
48-Me$^+$_8	-682.015540	-681.550414	-680.257233	-679.792107
48-Me$^+$_9	-682.015231	-681.550404	-680.256632	-679.791804
48-Me$^+$_10	-682.015185	-681.550158	-680.256573	-679.791546
**49**				-484.781877
49_1	-486.359540	-486.037627	-485.104463	-484.782550
49_2	-486.359685	-486.037716	-485.104124	-484.782155
49_3	-486.357126	-486.035235	-485.102881	-484.780990
49_4	-486.356960	-486.034845	-485.103070	-484.780955
49_5	-486.354822	-486.032768	-485.101692	-484.779638
49_6	-486.358179	-486.036287	-485.101393	-484.779501
49_7	-486.358312	-486.036416	-485.101302	-484.779406
49_8	-486.358583	-486.036823	-485.101145	-484.779385
49_9	-486.358501	-486.036450	-485.100901	-484.778850
49_10	-486.355784	-486.033529	-485.100684	-484.778429
**49-Me$^+$**				-524.316146
49-Me$^+$_1	-526.045644	-525.678413	-524.683880	-524.316649
49-Me$^+$_2	-526.042269	-525.675353	-524.681120	-524.314204
49-Me$^+$_3	-526.042077	-525.675112	-524.680828	-524.313863
49-Me$^+$_4	-526.042178	-525.674786	-524.681035	-524.313643
49-Me$^+$_5	-526.042141	-525.674560	-524.681039	-524.313459
49-Me$^+$_6	-526.041167	-525.673923	-524.679665	-524.312421
**50**				-522.695317
50_1	-524.368105	-524.041893	-523.021854	-522.695641
50_2	-524.366997	-524.040613	-523.021511	-522.695127
50_3	-524.367400	-524.041156	-523.020039	-522.693795
**50-Me$^+$**				-562.229704
50-Me$^+$_1	-564.054814	-563.683523	-562.601455	-562.230164
50-Me$^+$_2	-564.054215	-563.683160	-562.600734	-562.229679
50-Me$^+$_3	-564.054762	-563.683237	-562.600932	-562.229407
50-Me$^+$_4	-564.052764	-563.681497	-562.599189	-562.227921
50-Me$^+$_5	-564.051778	-563.680117	-562.598155	-562.226493
50-Me$^+$_6	-564.051049	-563.679423	-562.597890	-562.226264
**51**				-563.123794
51_1	-564.967778	-564.585188	-563.506872	-563.124282
51_2	-564.970305	-564.588216	-563.505944	-563.123855
51_3	-564.968346	-564.585893	-563.505785	-563.123332
51_4	-564.969986	-564.587792	-563.504543	-563.122349
51_5	-564.969096	-564.586536	-563.504694	-563.122134
**51-Me$^+$**				-602.658587
51-Me$^+$_1	-604.655480	-604.227524	-603.086652	-602.658696
51-Me$^+$_2	-604.651079	-604.223289	-603.082740	-602.654950
51-Me$^+$_3	-604.650524	-604.222596	-603.082208	-602.654280
**52**	-289.8746354	-289.701945	-289.1447154	-288.9720250
**52-Me$^+$**	-329.5659786	-329.349002	-328.7253074	-328.5083308
**53**	-329.1799428	-328.976697	-328.3433046	-328.1400588
**53-Me$^+$**	-368.8701586	-368.622359	-367.9259362	-367.6781366

**54**	-382.100964	-381.92897	-381.179961	-381.007963
**54-Me$^+$**	-421.801455	-421.585450	-420.762257	-420.546252
**55**	-749.387309	-749.093090	-747.581396	-747.287177
**55-Me$^+$**	-789.033425	-788.695362	-787.126335	-786.788272
**56**	-459.498999	-459.289442	-458.383909	-458.174353
**56-Me$^+$**	-499.203048	-498.949398	-497.969688	-497.716038
**57**	-536.905604	-536.658402	-535.602480	-535.355229
**57-Me$^+$**	-576.614364	-576.322975	-575.192996	-574.901595
**58**				-591.693361
58_1	-593.418065	-593.130882	-591.981253	-591.694070
58_2	-593.417089	-593.130032	-591.980205	-591.693148
58_3	-593.416648	-593.129503	-591.979900	-591.692755
58_4	-593.415838	-593.128722	-591.978867	-591.691751
58_5	-593.415488	-593.128356	-591.979018	-591.691886
58_6	-593.415277	-593.127873	-591.978972	-591.691568
58_7	-593.414082	-593.126927	-591.977705	-591.690550
**58-Me$^+$**				-631.242261
58-Me$^+$_1	-633.129214	-632.797785	-631.574118	-631.242689
58-Me$^+$_2	-633.129077	-632.797925	-631.573803	-631.242651
58-Me$^+$_3	-633.128021	-632.796615	-631.572771	-631.241365
58-Me$^+$_4	-633.127946	-632.796535	-631.572692	-631.241281
58-Me$^+$_5	-633.127843	-632.796281	-631.572934	-631.241371
58-Me$^+$_6	-633.126370	-632.794816	-631.571168	-631.239614
58-Me$^+$_7	-633.125330	-632.793715	-631.570008	-631.238393
**59**				-668.864138
59_1	-670.810555	-670.485932	-669.188397	-668.863774
59_2	-670.809955	-670.485240	-669.188192	-668.863477
59_3	-670.809509	-670.485967	-669.188063	-668.864521
**59-Me$^+$**				-708.4130556
59-Me$^+$_1	-710.523274	-710.154417	-708.782257	-708.413400
59-Me$^+$_2	-710.521930	-710.153123	-708.781454	-708.412647
59-Me$^+$_3	-710.521960	-710.153186	-708.781416	-708.412642
**60**	-461.902429	-461.644681	-460.754715	-460.497007
**60-Me$^+$**	-501.612755	-501.310409	-500.348461	-500.046115
**61**				-590.538821
61_1	-592.222726	-591.957797	-590.804005	-590.539076
61_2	-592.221494	-591.956591	-590.802484	-590.537581
**61-Me$^+$**				-630.088475
61-Me$^+$_1	-631.93496	-631.625782	-630.397667	-630.088489
61-Me$^+$_2	631.935067	-631.625863	-630.397664	-630.088461
**62**				-629.702332
62_1	-631.521285	-631.226469	-629.997804	-629.702988
62_2	-631.519929	-631.225072	-629.996450	-629.701594
62_3	-631.519580	-631.224611	-629.995589	-629.700620
62_4	-631.518936	-631.224066	-629.995691	-629.700821
62_5	-631.518089	-631.223278	-629.994812	-629.700001
62_6	-631.518347	-631.223563	-629.994767	-629.699983
**62-Me$^+$**				-669.252836

62-Me$^+$_1	-671.234101	-670.895069	-669.592430	-669.253399
62-Me$^+$_2	-671.232987	-670.893965	-669.591240	-669.252218
62-Me$^+$_3	-671.233860	-670.894876	-669.592079	-669.253094
62-Me$^+$_4	-671.232612	-670.893380	-669.590990	-669.251757
62-Me$^+$_5	-671.232695	-670.893444	-669.591128	-669.251876
62-Me$^+$_6	-671.232675	-670.893535	-669.590966	-669.251826
**63**				-747.191944
63_1	-749.405682	-749.021776	-747.576377	-747.192470
63_2	-749.407023	-749.022449	-747.576974	-747.192400
63_3	-749.404877	-749.020263	-747.576944	-747.192329
63_4	-749.405930	-749.021754	-747.576291	-747.192115
63_5	-749.406234	-749.021772	-747.576561	-747.192098
63_6	-749.404995	-749.020441	-747.576374	-747.191820
63_7	-749.404598	-749.020095	-747.576157	-747.191654
63_8	-749.404606	-749.020080	-747.576166	-747.191641
63_9	-749.404830	-749.020098	-747.576215	-747.191483
63_10	-749.406393	-749.021713	-747.576140	-747.191460
**63-Me$^+$**				-786.743479
63-Me$^+$_1	-789.121780	-788.692940	-787.173377	-786.744537
63-Me$^+$_2	-789.120016	-788.691557	-787.171915	-786.743456
63-Me$^+$_3	-789.120407	-788.691692	-787.172037	-786.743323
63-Me$^+$_4	-789.120407	-788.691688	-787.172038	-786.743319
63-Me$^+$_5	-789.119194	-788.690414	-787.171900	-786.743119
63-Me$^+$_6	-789.118810	-788.690096	-787.171405	-786.742691
63-Me$^+$_7	-789.118811	-788.690086	-787.171398	-786.742672
63-Me$^+$_8	-789.119475	-788.691090	-787.170972	-786.742587
63-Me$^+$_9	-789.119294	-788.690807	-787.170819	-786.742332
63-Me$^+$_10	-789.118385	-788.689728	-787.170896	-786.742240
**64**				-820.851865
64_1	-823.176622	-822.826137	-821.202780	-820.852295
64_2	-823.176434	-822.825873	-821.202409	-820.851848
64_3	-823.177128	-822.826607	-821.202518	-820.851997
64_4	-823.176637	-822.826010	-821.201939	-820.851312
64_5	-823.175198	-822.824733	-821.201087	-820.850621
64_6	-823.174700	-822.824147	-821.199884	-820.849331
**64-Me$^+$**				-860.405256
64-Me$^+$_1	-862.890937	-862.496224	-860.799004	-860.404291
64-Me$^+$_2	-862.891864	-862.497139	-860.800363	-860.405638
64-Me$^+$_3	-862.890446	-862.495612	-860.798210	-860.403376
64-Me$^+$_4	-862.891273	-862.496634	-860.798320	-860.403681
64-Me$^+$_5	-862.891448	-862.496815	-860.798564	-860.403931
64-Me$^+$_6	-862.891753	-862.497047	-860.800381	-860.405675
64-Me$^+$_7	-823.176622	-822.826137	-821.202780	-820.852295
64-Me$^+$_8	-823.176434	-822.825873	-821.202409	-820.851848
64-Me$^+$_9	-823.177128	-822.826607	-821.202518	-820.851997
64-Me$^+$_10	-823.176637	-822.826010	-821.201939	-820.851312
**65**	see reference 7			
**65-Me$^+$**	see reference 7			

**66**	-343.084654	-343.056497	-342.594262	-342.566105
**66-Me$^+$**	-382.720515	-382.649465	-382.124867	-382.053817
**67**	-382.386627	-382.326983	-381.789464	-381.729819
**67-Me$^+$**	-422.046080	-421.944312	-421.343927	-421.242159
**68**	-421.690601	-421.600096	-420.988339	-420.897834
**68-Me$^+$**	-461.369348	-461.236987	-460.562018	-460.429657
**69**	-932.554859	-932.077946	-930.512811	-930.035898
**69-Me$^+$**				-969.582606
69-Me$^+$_1	-972.251943	-971.732487	-970.102493	-969.583038
69-Me$^+$_2	-972.250674	-971.730952	-970.101315	-969.581593
69-Me$^+$_3	-972.249772	-971.730229	-970.100256	-969.580713
69-Me$^+$_4	-972.248294	-971.728588	-970.098738	-969.579032
**70**	-460.996338	-460.875398	-460.190881	-460.069941
**70-Me$^+$**	-500.690631	-500.527984	-499.779587	-499.616940
**71**				-773.379503
71_1	-775.368653	-775.010665	-773.737751	-773.379763
71_2	-775.368243	-775.010022	-773.736629	-773.378408
**71-Me$^+$**				-812.927595
71-Me$^+$_1	-815.066397	-814.665879	-813.328364	-812.927846
71-Me$^+$_2	-815.065766	-814.665403	-813.327304	-812.926940
**72**				-537.191893
72_1	-538.345989	-538.188633	-537.349319	-537.191963
72_2	-538.342312	-538.184924	-537.345605	-537.188217
**72-Me$^+$**	-578.042476	-577.843397	-576.939188	-576.740109
**73**	-618.182550	-617.942963	-616.963858	-616.724271
**73-Me$^+$**	-657.879352	-657.597861	-656.554230	-656.272739
**74**				-499.229506
74_1	-500.291789	-500.140620	-499.380925	-499.229756
74_2	-500.290437	-500.139265	-499.379363	-499.228192
**74-Me$^+$**	-539.988433	-539.795575	-538.971818	-538.778959
**75**				-614.313661
75_1	-615.695093	-615.501506	-614.507499	-614.313912
75_2	-615.692343	-615.498599	-614.505189	-614.311445
75_3	-615.692047	-615.498273	-614.504346	-614.310572
**75-Me$^+$**				-653.863612
75-Me$^+$_1	-655.394224	-655.158559	-654.099690	-653.864024
75-Me$^+$_2	-655.393933	-655.158173	-654.098930	-653.863170
75-Me$^+$_3	-655.393096	-655.157511	-654.098127	-653.862542
**76**				-577.556719
76_1	-578.886465	-578.676024	-577.767603	-577.557161
76_2	-578.885522	-578.675023	-577.766660	-577.556161
76_3	-578.884510	-578.673982	-577.765756	-577.555227
76_4	-578.882279	-578.671646	-577.763566	-577.552934
**76-Me$^+$**				-617.106709
76-Me$^+$_1	-618.583929	-618.331600	-617.359130	-617.106801
76-Me$^+$_2	-618.581109	-618.328607	-617.355924	-617.103422
**77**				-538.393636
77_1	-539.588244	-539.407327	-538.574773	-538.393856

77_2	-539.586615	-539.405739	-538.572794	-538.391918
**77-Me$^+$**	-579.286326	-579.063815	-578.166742	-577.944232
**78**				-538.391333
78_1	-539.589302	-539.408275	-538.572900	-538.391873
78_2	-539.588174	-539.407153	-538.572003	-538.390982
78_3	-539.587825	-539.406637	-538.571340	-538.390152
78_4	-539.587287	-539.406091	-538.571187	-538.389991
**78-Me$^+$**				-577.942235
78-Me$^+$_1	-579.287376	-579.064571	-578.165220	-577.942415
78-Me$^+$_2	-579.284837	-579.062040	-578.162866	-577.940069
**79**				-538.389368
79_1	-539.587192	-539.405864	-538.570888	-538.389560
79_2	-539.587129	-539.405732	-538.570864	-538.389467
79_3	-539.586011	-539.404407	-538.569936	-538.388332
79_4	-539.585712	-539.404324	-538.569356	-538.387969
79_5	-539.585741	-539.404392	-538.569142	-538.387794
**79-Me$^+$**				-577.940957
79-Me$^+$_1	-579.286127	-579.063048	-578.164383	-577.941304
79-Me$^+$_2	-579.285951	-579.062897	-578.163903	-577.940849
79-Me$^+$_3	-579.285941	-579.062757	-578.163895	-577.940711
79-Me$^+$_4	-579.284342	-579.061078	-578.162030	-577.938767
**80**				-577.552930
80_1	-578.886760	-578.675867	-577.764655	-577.553763
80_2	-578.885615	-578.674569	-577.763890	-577.552844
80_3	-578.885623	-578.674542	-577.763890	-577.552809
80_4	-578.885596	-578.674648	-577.763742	-577.552794
80_5	-578.884541	-578.673516	-577.763406	-577.552381
80_6	-578.884756	-578.673738	-577.763157	-577.552138
80_7	-578.885276	-578.674266	-577.763125	-577.552115
80_8	-578.883458	-578.672354	-577.762411	-577.551308
80_9	-578.884029	-578.672918	-577.762227	-577.551116
**80-Me$^+$**				-617.104612
80-Me$^+$_1	-618.585691	-618.332926	-617.357773	-617.105008
80-Me$^+$_2	-618.584535	-618.331912	-617.357103	-617.104480
80-Me$^+$_3	-618.583181	-618.330407	-617.355721	-617.102947
80-Me$^+$_4	-618.582031	-618.329248	-617.355107	-617.102323
**81**				-576.354625
81_1	-577.648530	-577.460788	-576.542706	-576.354964
81_2	-577.647685	-577.460060	-576.542283	-576.354658
81_3	-577.647391	-577.459517	-576.541616	-576.353742
81_4	-577.646116	-577.458234	-576.539961	-576.352079
**81-Me$^+$**				-615.906429
81-Me$^+$_1	-617.348441	-617.118870	-616.136226	-615.906656
81-Me$^+$_2	-617.346862	-617.117303	-616.134344	-615.904786
**82**				-616.714334
82_1	-618.184164	-617.943271	-616.956247	-616.715354
82_2	-618.182928	-617.942055	-616.955563	-616.714690
82_3	-618.182977	-617.942136	-616.955465	-616.714624

82_4	-618.182943	-617.942011	-616.955532	-616.714599
82_5	-618.182953	-617.942028	-616.955398	-616.714473
82_6	-618.182926	-617.942005	-616.955389	-616.714468
82_7	-618.181892	-617.941003	-616.955143	-616.714254
82_8	-618.181868	-617.940899	-616.955212	-616.714243
82_9	-618.181974	-617.940892	-616.955258	-616.714176
82_10	-618.182118	-617.941320	-616.954865	-616.714067
**82-Me$^+$**				-656.266368
82-Me$^+$_1	-657.883498	-657.600804	-656.549780	-656.267086
82-Me$^+$_2	-657.882355	-657.599641	-656.549179	-656.266464
82-Me$^+$_3	-657.882355	-657.599644	-656.549175	-656.266463
82-Me$^+$_4	-657.882290	-657.599642	-656.548983	-656.266336
82-Me$^+$_5	-657.881308	-657.598600	-656.548807	-656.266099
82-Me$^+$_6	-657.881098	-657.598463	-656.547916	-656.265282
82-Me$^+$_7	-657.879875	-657.597252	-656.547512	-656.264889
82-Me$^+$_8	-657.880049	-657.597323	-656.547302	-656.264576
82-Me$^+$_9	-657.878901	-657.596178	-656.546937	-656.264214
82-Me$^+$_10	-657.879787	-657.596989	-656.546839	-656.264042
**83**				-577.553925
83_1	-578.883871	-578.672968	-577.765547	-577.554644
83_2	-578.883036	-578.672148	-577.764909	-577.554020
83_3	-578.882644	-578.671852	-577.763876	-577.553084
83_4	-578.882365	-578.671603	-577.763649	-577.552887
83_5	-578.882496	-578.671729	-577.763638	-577.552872
83_6	-578.881982	-578.670986	-577.763758	-577.552762
83_7	-578.881272	-578.670394	-577.762826	-577.551948
**83-Me$^+$**				-617.105979
83-Me$^+$_1	-618.583606	-618.330943	-617.359038	-617.106375
83-Me$^+$_2	-618.581784	-618.329012	-617.357328	-617.104556
83-Me$^+$_3	-618.581637	-618.328859	-617.357119	-617.104341
**84**				-655.875754
84_1	-657.481573	-657.210795	-656.147812	-655.877035
84_2	-657.480303	-657.209548	-656.147156	-655.876401
84_3	-657.480346	-657.209510	-656.147166	-655.876330
84_4	-657.480376	-657.209644	-656.147039	-655.876307
84_5	-657.480275	-657.209460	-656.147051	-655.876236
84_6	-657.480303	-657.209471	-656.147063	-655.876231
84_7	-657.480310	-657.209546	-656.146962	-655.876198
84_8	-657.480330	-657.209522	-656.146950	-655.876142
84_9	-657.479279	-657.208494	-656.146891	-655.876106
84_10	-657.479270	-657.208358	-656.147002	-655.876090
**84-Me$^+$**				-695.427969
84-Me$^+$_1	-697.181147	-696.868538	-695.741606	-695.428997
84-Me$^+$_2	-697.180005	-696.867436	-695.741122	-695.428553
84-Me$^+$_3	-697.178870	-696.866356	-695.740825	-695.428311
84-Me$^+$_4	-697.179907	-696.867239	-695.740899	-695.428232
84-Me$^+$_5	-697.179947	-696.867302	-695.740785	-695.428140
84-Me$^+$_6	-697.178805	-696.866239	-695.740342	-695.427776

84-Me$^+$_7	-697.177904	-696.865225	-695.740361	-695.427681
84-Me$^+$_8	-697.178754	-696.866050	-695.740339	-695.427635
84-Me$^+$_9	-697.178747	-696.866248	-695.739910	-695.427411
84-Me$^+$_10	-697.178708	-696.865998	-695.740082	-695.427372
**85**				-695.037213
85_1	-696.778965	-696.478251	-695.339383	-695.038669
85_2	-696.777699	-696.476968	-695.338792	-695.038061
85_3	-696.777709	-696.476949	-695.338749	-695.037990
85_4	-696.776695	-696.475918	-695.338729	-695.037952
85_5	-696.777714	-696.476955	-695.338707	-695.037947
85_6	-696.777723	-696.476952	-695.338699	-695.037928
85_7	-696.776700	-696.475895	-695.338684	-695.037879
85_8	-696.777665	-696.476970	-695.338569	-695.037873
85_9	-696.777753	-696.477009	-695.338595	-695.037852
85_10	-696.777684	-696.476997	-695.338536	-695.037849
**85-Me$^+$**				-734.589493
85-Me$^+$_1	-736.478692	-736.136166	-734.933340	-734.590814
85-Me$^+$_2	-736.477587	-736.135027	-734.932890	-734.590329
85-Me$^+$_3	-736.477466	-736.134990	-734.932722	-734.590246
85-Me$^+$_4	-736.476534	-736.133915	-734.932786	-734.590167
85-Me$^+$_5	-736.477439	-736.134892	-734.932497	-734.589950
85-Me$^+$_6	-736.477434	-736.134789	-734.932559	-734.589914
85-Me$^+$_7	-736.475418	-736.132849	-734.932288	-734.589719
85-Me$^+$_8	-736.476285	-736.133631	-734.932237	-734.589583
85-Me$^+$_9	-736.475455	-736.132711	-734.932257	-734.589513
85-Me$^+$_10	-736.476407	-736.133736	-734.932146	-734.589474
**86**				-734.198790
86_1	-736.076339	-735.745771	-734.530938	-734.200370
86_2	-736.070760	-735.743263	-734.527756	-734.200259
86_3	-736.075078	-735.744549	-734.530294	-734.199766
86_4	-736.075087	-735.744518	-734.530269	-734.199699
86_5	-736.075049	-735.744570	-734.530153	-734.199674
86_6	-736.075087	-735.744417	-734.530309	-734.199639
86_7	-736.075112	-735.744430	-734.530313	-734.199631
86_8	-736.075058	-735.744505	-734.530161	-734.199608
86_9	-736.073965	-735.743426	-734.530115	-734.199576
86_10	-736.075100	-735.744430	-734.530234	-734.199564
**86-Me$^+$**				-773.751125
86-Me$^+$_1	-775.776172	-775.403730	-774.125007	-773.752565
86-Me$^+$_2	-775.775057	-775.402718	-774.124560	-773.752222
86-Me$^+$_3	-775.774984	-775.402565	-774.124459	-773.752040
86-Me$^+$_4	-775.774905	-775.402534	-774.124342	-773.751971
86-Me$^+$_5	-775.774088	-775.401530	-774.124508	-773.751950
86-Me$^+$_6	-775.772171	-775.399739	-774.124343	-773.751911
86-Me$^+$_7	-775.774763	-775.402445	-774.124189	-773.751871
86-Me$^+$_8	-775.773022	-775.400524	-774.124260	-773.751762
86-Me$^+$_9	-775.774884	-775.402466	-774.124141	-773.751723
86-Me$^+$_10	-775.772943	-775.400461	-774.124060	-773.751577

**87**				-695.039738
87_1	-696.774527	-696.474030	-695.341440	-695.040942
87_2	-696.775181	-696.474950	-695.340841	-695.040611
87_3	-696.775248	-696.474991	-695.340852	-695.040595
87_4	-696.774577	-696.474039	-695.341010	-695.040471
87_5	-696.774677	-696.474214	-695.340910	-695.040446
87_6	-696.774579	-696.474029	-695.340992	-695.040441
87_7	-696.774383	-696.473731	-695.341036	-695.040384
87_8	-696.774619	-696.474163	-695.340836	-695.040380
87_9	-696.774346	-696.473884	-695.340715	-695.040253
87_10	-696.773547	-696.473179	-695.340557	-695.040189
**87-Me$^+$**				-734.592142
87-Me$^+$_1	-736.474184	-736.131872	-734.935309	-734.592997
87-Me$^+$_2	-736.474009	-736.132044	-734.934816	-734.592851
87-Me$^+$_3	-736.474573	-736.132592	-734.934703	-734.592722
87-Me$^+$_4	-736.474515	-736.132576	-734.934652	-734.592713
87-Me$^+$_5	-736.474160	-736.131802	-734.934838	-734.592480
87-Me$^+$_6	-736.474258	-736.132131	-734.934501	-734.592374
87-Me$^+$_7	-736.473392	-736.131370	-734.934330	-734.592308
87-Me$^+$_8	-736.473749	-736.131520	-734.934499	-734.592270
87-Me$^+$_9	-736.473638	-736.131613	-734.934219	-734.592194
87-Me$^+$_10	-736.473168	-736.130717	-734.934640	-734.592189
**88**				-695.043972
88_1	-696.776563	-696.476650	-695.344690	-695.044777
88_2	-696.776633	-696.476686	-695.344523	-695.044576
88_3	-696.775424	-696.475499	-695.344040	-695.044115
88_4	-696.775787	-696.475855	-695.343936	-695.044004
88_5	-696.775611	-696.475659	-695.343800	-695.043848
88_6	-696.775546	-696.475518	-695.343603	-695.043576
88_7	-696.774817	-696.474825	-695.343164	-695.043173
88_8	-696.774639	-696.474559	-695.342872	-695.042792
88_9	-696.774720	-696.474593	-695.342910	-695.042784
88_10	-696.774564	-696.474616	-695.342699	-695.042750
**88-Me$^+$**				-734.596465
88-Me$^+$_1	-736.476703	-736.134674	-734.939499	-734.597470
88-Me$^+$_2	-736.476635	-736.134466	-734.939039	-734.596870
88-Me$^+$_3	-736.476555	-736.134548	-734.938738	-734.596731
88-Me$^+$_4	-736.476433	-736.134376	-734.938769	-734.596712
88-Me$^+$_5	-736.476517	-736.134645	-734.938568	-734.596695
88-Me$^+$_6	-736.476409	-736.134380	-734.938617	-734.596588
88-Me$^+$_7	-736.475762	-736.133699	-734.938612	-734.596549
88-Me$^+$_8	-736.476114	-736.134160	-734.938435	-734.596481
88-Me$^+$_9	-736.475926	-736.133960	-734.937814	-734.595848
88-Me$^+$_10	-736.475745	-736.133708	-734.937754	-734.595717
**89**	-1035.959965	-1035.669422	-1033.807304	-1033.516761
**89-Me$^+$**	-1075.664367	-1075.331813	-1073.401907	-1073.069353
**90**				-616.714499
90_1	-618.178677	-617.937839	-616.956274	-616.715436

90_2	-618.178085	-617.937532	-616.954657	-616.714104
90_3	-618.177047	-617.936184	-616.954391	-616.713528
90_4	-618.177077	-617.936166	-616.954231	-616.713320
90_5	-618.176987	-617.936145	-616.954070	-616.713228
90_6	-618.176742	-617.935685	-616.953926	-616.712870
90_7	-618.176563	-617.935467	-616.953930	-616.712834
90_8	-618.176051	-617.935127	-616.952779	-616.711855
90_9	-618.175857	-617.935047	-616.952637	-616.711827
90_10	-618.174729	-617.933832	-616.952051	-616.711154
**90-Me$^+$**				-656.267113
90-Me$^+$_1	-657.879057	-657.596525	-656.550197	-656.267665
90-Me$^+$_2	-657.878149	-657.595612	-656.549339	-656.266802
90-Me$^+$_3	-657.878017	-657.595163	-656.548873	-656.266019
90-Me$^+$_4	-657.877507	-657.594652	-656.548467	-656.265611
90-Me$^+$_5	-657.876095	-657.593317	-656.546820	-656.264042
**91**	-578.882974	-578.672922	-577.769460	-577.559408
**91-Me$^+$**	-618.584037	-618.332107	-617.364198	-617.112268
**92**				-734.201663
92_1	-736.071843	-735.741518	-734.533291	-734.202966
92_2	-736.072498	-735.742322	-734.532760	-734.202583
92_3	-736.071601	-735.741264	-734.532863	-734.202526
92_4	-736.070748	-735.740335	-734.532931	-734.202517
92_5	-736.071883	-735.741528	-734.532721	-734.202366
92_6	-736.070769	-735.740390	-734.532521	-734.202141
92_7	-736.071290	-735.741110	-734.532239	-734.202059
92_8	-736.070837	-735.740457	-734.532422	-734.202041
92_9	-736.071358	-735.741078	-734.532273	-734.201993
92_10	-736.070762	-735.740290	-734.532457	-734.201986
**92-Me$^+$**				-773.754529
92-Me$^+$_1	-775.771772	-775.399617	-774.127551	-773.755396
92-Me$^+$_2	-775.770755	-775.398657	-774.127405	-773.755307
92-Me$^+$_3	-775.772239	-775.400339	-774.127055	-773.755154
92-Me$^+$_4	-775.771461	-775.399523	-774.127049	-773.755111
92-Me$^+$_5	-775.771366	-775.399513	-774.126861	-773.755008
92-Me$^+$_6	-775.771764	-775.399620	-774.127085	-773.754941
92-Me$^+$_7	-775.770867	-775.398823	-774.126781	-773.754737
92-Me$^+$_8	-775.770673	-775.398506	-774.126821	-773.754655
92-Me$^+$_9	-775.771134	-775.399058	-774.126728	-773.754652
92-Me$^+$_10	-775.770185	-775.397956	-774.126820	-773.754591
**93**				-616.715725
93_1	-618.181148	-617.940393	-616.957332	-616.716577
93_2	-618.180143	-617.939416	-616.957095	-616.716368
93_3	-618.180226	-617.939613	-616.956806	-616.716193
93_4	-618.179953	-617.939216	-616.955684	-616.714947
93_5	-618.179408	-617.938466	-616.955821	-616.714879
93_6	-618.179745	-617.938975	-616.955589	-616.714819
93_7	-618.179864	-617.939042	-616.955485	-616.714663
93_8	-618.178983	-617.938170	-616.955474	-616.714662

93_9	-618.178653	-617.937828	-616.955134	-616.714309
93_10	-618.178574	-617.937802	-616.955054	-616.714282
**93-Me$^+$**				-656.268658
93-Me$^+$_1	-657.881697	-657.599141	-656.551645	-656.269089
93-Me$^+$_2	-657.880856	-657.598258	-656.551596	-656.268998
93-Me$^+$_3	-657.879792	-657.597229	-656.550126	-656.267563
93-Me$^+$_4	-657.879823	-657.597176	-656.550006	-656.267359
93-Me$^+$_5	-657.878495	-657.595816	-656.549367	-656.266689
93-Me$^+$_6	-657.878343	-657.595604	-656.548863	-656.266124
93-Me$^+$_7	-657.877581	-657.594944	-656.547671	-656.265034
**94**				-615.548621
94_1	-616.974756	-616.756123	-615.767613	-615.548980
94_2	-616.972323	-616.753756	-615.765989	-615.547422
94_3	-616.972428	-616.753513	-615.764823	-615.545908
**94-Me$^+$**	-656.675630	-656.415057	-655.362436	-655.101863
**95**				-655.876283
95_1	-657.476032	-657.205357	-656.148357	-655.877682
95_2	-657.476064	-657.205110	-656.148292	-655.877338
95_3	-657.475043	-657.204118	-656.147977	-655.877052
95_4	-657.475554	-657.205012	-656.146704	-655.876162
95_5	-657.475499	-657.204861	-656.146628	-655.875991
95_6	-657.474646	-657.203841	-656.146722	-655.875917
95_7	-657.474621	-657.203846	-656.146532	-655.875757
95_8	-657.474483	-657.203729	-656.146417	-655.875663
95_9	-657.474438	-657.203550	-656.146372	-655.875483
95_10	-657.474444	-657.203583	-656.146230	-655.875370
**95-Me$^+$**				-695.429682
95-Me$^+$_1	-697.177131	-696.864491	-695.743110	-695.430471
95-Me$^+$_2	-697.176293	-696.863576	-695.742789	-695.430072
95-Me$^+$_3	-697.177210	-696.864326	-695.742912	-695.430028
95-Me$^+$_4	-697.176771	-696.864264	-695.742455	-695.429948
95-Me$^+$_5	-697.176596	-696.863990	-695.742483	-695.429877
95-Me$^+$_6	-697.175653	-696.863057	-695.741995	-695.429399
95-Me$^+$_7	-697.175212	-696.862627	-695.741671	-695.429086
95-Me$^+$_8	-697.175592	-696.863036	-695.741469	-695.428912
95-Me$^+$_9	-697.175733	-696.863127	-695.741453	-695.428847
95-Me$^+$_10	-697.176070	-696.863221	-695.741555	-695.428707
**96**				-691.435173
96_1	-693.043842	-692.813807	-691.665471	-691.435436
96_2	-693.041564	-692.811529	-691.663637	-691.433602
96_3	-693.038571	-692.808464	-691.661113	-691.431006
**96-Me$^+$**				-730.988661
96-Me$^+$_1	-732.746080	-732.473852	-731.261227	-730.988999
96-Me$^+$_2	-732.746007	-732.473804	-731.261194	-730.988991
96-Me$^+$_3	-732.745680	-732.473451	-731.260386	-730.988156
96-Me$^+$_4	-732.744884	-732.472684	-731.259551	-730.987351
96-Me$^+$_5	-732.744521	-732.472362	-731.258799	-730.986640
96-Me$^+$_6	-732.742665	-732.471431	-731.256499	-730.985264

**97**				-654.719362
97_1	-656.279581	-656.030734	-654.968567	-654.719721
97_2	-656.278069	-656.029107	-654.966692	-654.717730
97_3	-656.276782	-656.027764	-654.966339	-654.717321
**97-Me$^+$**	-695.981191	-695.690430	-694.563909	-694.273148
**98**				-577.548752
98_1	-578.882317	-578.670863	-577.760698	-577.549243
98_2	-578.882307	-578.670812	-577.760706	-577.549211
98_3	-578.881414	-578.669729	-577.760384	-577.548699
98_4	-578.881118	-578.669561	-577.759884	-577.548327
98_5	-578.880980	-578.669528	-577.759134	-577.547682
98_6	-578.880980	-578.669524	-577.759129	-577.547673
98_7	-578.880891	-578.669360	-577.758980	-577.547449
98_8	-578.879750	-578.668302	-577.757930	-577.546482
**98-Me$^+$**				-617.102794
98-Me$^+$_1	-618.583673	-618.330318	-617.356777	-617.103423
98-Me$^+$_2	-618.583473	-618.329956	-617.356358	-617.102841
98-Me$^+$_3	-618.583197	-618.329644	-617.355828	-617.102274
98-Me$^+$_4	-618.583239	-618.329669	-617.355840	-617.102271
98-Me$^+$_5	-618.581787	-618.328153	-617.354398	-617.100764
98-Me$^+$_6	-618.581665	-618.328057	-617.354175	-617.100566
98-Me$^+$_7	-618.581633	-618.328095	-617.354046	-617.100508
**99**				-695.038191
99_1	-696.773316	-696.472594	-695.340422	-695.039700
99_2	-696.772416	-696.471526	-695.340153	-695.039263
99_3	-696.772928	-696.472449	-695.338743	-695.038264
99_4	-696.771988	-696.471459	-695.338784	-695.038255
99_5	-696.772008	-696.471410	-695.338766	-695.038168
99_6	-696.771830	-696.471078	-695.338445	-695.037693
99_7	-696.771784	-696.471044	-695.338392	-695.037652
99_8	-696.771652	-696.471173	-695.338011	-695.037532
99_9	-696.770798	-696.470068	-695.338236	-695.037506
99_10	-696.771351	-696.470530	-695.338270	-695.037449
**99-Me$^+$**				-734.592511
99-Me$^+$_1	-736.474253	-736.131777	-734.935785	-734.593309
99-Me$^+$_2	-736.475233	-736.132581	-734.935851	-734.593199
99-Me$^+$_3	-736.474662	-736.132244	-734.935309	-734.592890
99-Me$^+$_4	-736.474905	-736.132495	-734.935267	-734.592857
99-Me$^+$_5	-736.473553	-736.131120	-734.934865	-734.592432
99-Me$^+$_6	-736.473703	-736.131373	-734.934632	-734.592302
99-Me$^+$_7	-736.473773	-736.131166	-734.934894	-734.592287
99-Me$^+$_8	-736.472560	-736.130061	-734.934530	-734.592031
99-Me$^+$_9	-736.473404	-736.130579	-734.934654	-734.591828
99-Me$^+$_10	-736.472751	-736.130313	-734.934204	-734.591766
**100**				-616.712941
100_1	-618.182248	-617.941090	-616.955068	-616.713910
100_2	-618.182262	-617.941173	-616.954981	-616.713892
100_3	-618.181133	-617.940002	-616.954157	-616.713027

100_4	-618.180839	-617.939626	-616.954237	-616.713024
100_5	-618.181138	-617.940029	-616.954087	-616.712978
100_6	-618.181068	-617.939899	-616.954135	-616.712966
100_7	-618.181010	-617.939943	-616.953560	-616.712493
100_8	-618.180720	-617.939581	-616.953597	-616.712459
100_9	-618.179976	-617.938952	-616.953255	-616.712231
100_10	-618.180221	-617.939058	-616.953333	-616.712170
**100-Me$^+$**				-656.267583
100-Me$^+$_1	-657.883838	-657.600811	-656.551384	-656.268357
100-Me$^+$_2	-657.883653	-657.600694	-656.550788	-656.267829
100-Me$^+$_3	-657.883675	-657.600684	-656.550665	-656.267674
100-Me$^+$_4	-657.882065	-657.599070	-656.549224	-656.266229
100-Me$^+$_5	-657.881230	-657.598164	-656.548959	-656.265894
100-Me$^+$_6	-657.881204	-657.598155	-656.548639	-656.265590
100-Me$^+$_7	-657.881301	-657.598035	-656.548770	-656.265504
100-Me$^+$_8	-657.881205	-657.598070	-656.548557	-656.265422
100-Me$^+$_9	-657.881115	-657.598029	-656.548284	-656.265198
100-Me$^+$_10	-657.881245	-657.598096	-656.548343	-656.265194
**101**				-1038.181330
101_1	-1040.785315	-1040.402547	-1038.566062	-1038.183295
101_2	-1040.785057	-1040.402098	-1038.566199	-1038.183240
101_3	-1040.785297	-1040.402422	-1038.565498	-1038.182623
101_4	-1040.785032	-1040.402297	-1038.565183	-1038.182447
101_5	-1040.784865	-1040.402092	-1038.565106	-1038.182333
101_6	-1040.784365	-1040.402628	-1038.563976	-1038.182239
101_7	-1040.785243	-1040.402395	-1038.565010	-1038.182161
101_8	-1040.785115	-1040.402213	-1038.564863	-1038.181962
101_9	-1040.784569	-1040.402064	-1038.564433	-1038.181928
101_10	-1040.785069	-1040.402140	-1038.564823	-1038.181894
**101-Me$^+$**				-1077.736120
101-Me$^+$_1	-1080.487067	-1080.063179	-1078.161498	-1077.737610
101-Me$^+$_2	-1080.488145	-1080.063083	-1078.161343	-1077.736281
101-Me$^+$_3	-1080.486567	-1080.061660	-1078.160488	-1077.735581
101-Me$^+$_4	-1080.488105	-1080.063207	-1078.160460	-1077.735562
101-Me$^+$_5	-1080.487563	-1080.062618	-1078.160485	-1077.735540
101-Me$^+$_6	-1080.488093	-1080.063444	-1078.160128	-1077.735479
101-Me$^+$_7	-1080.487860	-1080.063003	-1078.160066	-1077.735209
101-Me$^+$_8	-1080.487443	-1080.062538	-1078.160095	-1077.735190
101-Me$^+$_9	-1080.487891	-1080.063205	-1078.159853	-1077.735167
101-Me$^+$_10	-1080.488032	-1080.063035	-1078.160139	-1077.735142
**102**				-959.850050
102_1	-962.195598	-961.871418	-960.174950	-959.850770
102_2	-962.193994	-961.870234	-960.174358	-959.850598
102_3	-962.195247	-961.871144	-960.174615	-959.850512
102_4	-962.194296	-961.870202	-960.174503	-959.850408
102_5	-962.194038	-961.869814	-960.174559	-959.850335
102_6	-962.194268	-961.870245	-960.174225	-959.850201
102_7	-962.194401	-961.870202	-960.174260	-959.850061

102_8	-962.193220	-961.869030	-960.174235	-959.850045
102_9	-962.193969	-961.869900	-960.173991	-959.849922
102_10	-962.194043	-961.869810	-960.174134	-959.849901
**102-Me$^+$**				-999.404875
102-Me$^+$_1	-1001.900316	-1001.534020	-999.771893	-999.405597
102-Me$^+$_2	-1001.900337	-1001.534304	-999.771514	-999.405481
102-Me$^+$_3	-1001.900386	-1001.534250	-999.771420	-999.405284
102-Me$^+$_4	-1001.899317	-1001.532986	-999.771319	-999.404988
102-Me$^+$_5	-1001.899207	-1001.532962	-999.771180	-999.404935
102-Me$^+$_6	-1001.899261	-1001.533228	-999.770875	-999.404841
102-Me$^+$_7	-1001.899287	-1001.532945	-999.770981	-999.404639
102-Me$^+$_8	-1001.897235	-1001.531198	-999.770264	-999.404227
102-Me$^+$_9	-1001.897748	-1001.531757	-999.770103	-999.404112
102-Me$^+$_10	-1001.897805	-1001.531752	-999.770129	-999.404076
**103**				-616.715989
103_1	-618.178225	-617.937456	-616.957188	-616.716419
103_2	-618.176618	-617.935883	-616.955395	-616.714660
103_3	-618.176410	-617.935504	-616.955300	-616.714394
**103-Me$^+$**				-656.270876
103-Me$^+$_1	-657.881357	-657.598580	-656.554033	-656.271256
103-Me$^+$_2	-657.879374	-657.596685	-656.551872	-656.269183
103-Me$^+$_3	-657.878696	-657.595912	-656.551172	-656.268388
103-Me$^+$_4	-657.877891	-657.595206	-656.550362	-656.267677
**104**				-693.870295
104_1	-695.565864	-695.286831	-694.150273	-693.871240
104_2	-695.565569	-695.286448	-694.149777	-693.870656
104_3	-695.565119	-695.285994	-694.149430	-693.870305
104_4	-695.565140	-695.285947	-694.149059	-693.869867
104_5	-695.563715	-695.284493	-694.148773	-693.869551
104_6	-695.564706	-695.285578	-694.148555	-693.869427
104_7	-695.564180	-695.285160	-694.147992	-693.868973
104_8	-695.564414	-695.285052	-694.148270	-693.868909
104_9	-695.564010	-695.284734	-694.147778	-693.868502
104_10	-695.563508	-695.284603	-694.147369	-693.868464
**104-Me$^+$**				-733.425226
104-Me$^+$_1	-735.268597	-734.947693	-733.746676	-733.425772
104-Me$^+$_2	-735.267731	-734.946821	-733.745726	-733.424816
104-Me$^+$_3	-735.267503	-734.946515	-733.745522	-733.424534
104-Me$^+$_4	-735.267672	-734.946690	-733.745341	-733.424358
**105**				-812.531519
105_1	-814.665707	-814.276335	-812.921906	-812.532534
105_2	-814.665956	-814.276581	-812.921774	-812.532399
105_3	-814.665462	-814.276121	-812.921337	-812.531997
105_4	-814.665061	-814.275638	-812.920948	-812.531524
105_5	-814.664644	-814.275127	-812.921029	-812.531513
105_6	-814.664961	-814.275466	-812.920998	-812.531503
105_7	-814.664958	-814.275423	-812.920993	-812.531458
105_8	-814.664945	-814.275457	-812.920902	-812.531414

105_9	-814.664124	-814.274512	-812.920558	-812.530947
105_10	-814.663582	-814.274153	-812.920194	-812.530764
**105-Me$^+$**				-852.086758
105-Me$^+$_1	-854.368709	-853.936905	-852.519824	-852.088021
105-Me$^+$_2	-854.368442	-853.936798	-852.519273	-852.087629
105-Me$^+$_3	-854.368456	-853.936803	-852.519235	-852.087582
105-Me$^+$_4	-854.368129	-853.936341	-852.519009	-852.087221
105-Me$^+$_5	-854.367735	-853.936060	-852.518840	-852.087165
105-Me$^+$_6	-854.368436	-853.936690	-852.518887	-852.087140
105-Me$^+$_7	-854.367974	-853.936375	-852.518634	-852.087035
105-Me$^+$_8	-854.368096	-853.936345	-852.518617	-852.086866
105-Me$^+$_9	-854.368033	-853.936100	-852.518735	-852.086801
105-Me$^+$_10	-854.368320	-853.936514	-852.518608	-852.086802
**106**				-733.026120
106_1	-734.856508	-734.547284	-733.336521	-733.027298
106_2	-734.855742	-734.546508	-733.335633	-733.026399
106_3	-734.855695	-734.546304	-733.335512	-733.026120
106_4	-734.855062	-734.545734	-733.334882	-733.025554
106_5	-734.854507	-734.545246	-733.334401	-733.025140
106_6	-734.855184	-734.545799	-733.334488	-733.025102
106_7	-734.854862	-734.545511	-733.334310	-733.024958
106_8	-734.854796	-734.545735	-733.333969	-733.024908
106_9	-734.854747	-734.545264	-733.334097	-733.024614
106_10	-734.854973	-734.545233	-733.334247	-733.024507
**106-Me$^+$**				-772.581520
106-Me$^+$_1	-774.559796	-774.208751	-772.933376	-772.582331
106-Me$^+$_2	-774.558999	-774.207760	-772.932402	-772.581162
106-Me$^+$_3	-774.558485	-774.207244	-772.931839	-772.580599
106-Me$^+$_4	-774.558108	-774.207029	-772.931544	-772.580465
106-Me$^+$_5	-774.557802	-774.206535	-772.931135	-772.579867
106-Me$^+$_6	-774.557925	-774.206545	-772.930876	-772.579495
106-Me$^+$_7	-774.557206	-774.205927	-772.930220	-772.578941
106-Me$^+$_8	-774.556197	-774.205019	-772.929484	-772.578307
106-Me$^+$_9	-774.556867	-774.206024	-772.928862	-772.578020
**107**				-772.184110
107_1	-774.148537	-773.809345	-772.524449	-772.185257
107_2	-774.147396	-773.808055	-772.522722	-772.183380
107_3	-774.147541	-773.807817	-772.522953	-772.183228
107_4	-774.147491	-773.808270	-772.522177	-772.182957
107_5	-774.147232	-773.807578	-772.522482	-772.182827
107_6	-774.146481	-773.807253	-772.521248	-772.182020
107_7	-774.146605	-773.807252	-772.521222	-772.181869
107_8	-774.146681	-773.807236	-772.521278	-772.181834
107_9	-774.146572	-773.807280	-772.521047	-772.181755
107_10	-774.146216	-773.806826	-772.520714	-772.181324
**107-Me$^+$**				-811.740055
107-Me$^+$_1	-813.852445	-813.471315	-812.121932	-811.740802
107-Me$^+$_2	-813.851074	-813.470063	-812.119388	-811.738377

107-Me$^+$_3	-813.850291	-813.469630	-812.118852	-811.738191
107-Me$^+$_4	-813.850400	-813.469156	-812.119377	-811.738133
107-Me$^+$_5	-813.850628	-813.469575	-812.118826	-811.737772
107-Me$^+$_6	-813.850166	-813.469072	-812.118299	-811.737205
107-Me$^+$_7	-813.849773	-813.468771	-812.117648	-811.736646
107-Me$^+$_8	-813.849772	-813.468764	-812.117650	-811.736643
107-Me$^+$_9	-813.849588	-813.468452	-812.117751	-811.736615
107-Me$^+$_10	-813.849605	-813.468344	-812.117648	-811.736388
**108**				-1038.183820
108_1	-1040.789226	-1040.406292	-1038.566984	-1038.184049
108_2	-1040.788995	-1040.406292	-1038.566682	-1038.183979
108_3	-1040.787773	-1040.404909	-1038.566701	-1038.183837
108_4	-1040.785091	-1040.402106	-1038.563734	-1038.180749
108_5	-1040.785280	-1040.402597	-1038.563322	-1038.180639
108_6	-1040.785254	-1040.402551	-1038.563189	-1038.180486
108_7	-1040.785093	-1040.402314	-1038.563254	-1038.180475
108_8	-1040.784935	-1040.402057	-1038.563050	-1038.180172
**108-Me$^+$**				-1077.739835
108-Me$^+$_1	-1080.494905	-1080.070130	-1078.164857	-1077.740082
108-Me$^+$_2	-1080.492994	-1080.068711	-1078.164186	-1077.739902
108-Me$^+$_3	-1080.494933	-1080.070257	-1078.164266	-1077.739590
108-Me$^+$_4	-1080.494823	-1080.070200	-1078.164186	-1077.739563
**109**				-695.036158
109_1	-696.777148	-696.476274	-695.338641	-695.037768
109_2	-696.777175	-696.476313	-695.338476	-695.037614
109_3	-696.775935	-696.475156	-695.337739	-695.036960
109_4	-696.775966	-696.474952	-695.337879	-695.036865
109_5	-696.775941	-696.474913	-695.337859	-695.036831
109_6	-696.776029	-696.475133	-695.337709	-695.036813
109_7	-696.775991	-696.474941	-695.337855	-695.036806
109_8	-696.776020	-696.475044	-695.337765	-695.036790
109_9	-696.775863	-696.474745	-695.337901	-695.036783
109_10	-696.776020	-696.475026	-695.337768	-695.036774
**109-Me$^+$**				-734.592214
109-Me$^+$_1	-736.480291	-736.137602	-734.936545	-734.593857
109-Me$^+$_2	-736.479259	-736.136327	-734.936061	-734.593129
109-Me$^+$_3	-736.480161	-736.137293	-734.935912	-734.593045
109-Me$^+$_4	-736.479238	-736.136291	-734.935899	-734.592953
109-Me$^+$_5	-736.480128	-736.137288	-734.935737	-734.592896
109-Me$^+$_6	-736.478291	-736.135371	-734.935489	-734.592569
109-Me$^+$_7	-736.478155	-736.135325	-734.935320	-734.592489
109-Me$^+$_8	-736.479060	-736.136198	-734.935219	-734.592357
109-Me$^+$_9	-736.479071	-736.136136	-734.935236	-734.592301
109-Me$^+$_10	-736.479083	-736.136105	-734.935251	-734.592273
**110**				-692.639671
110_1	-694.300547	-694.046038	-692.895067	-692.640558
110_2	-694.299433	-694.045029	-692.894530	-692.640127
110_3	-694.299517	-694.045025	-692.894137	-692.639646

110_4	-694.299094	-694.044469	-692.894077	-692.639452
110_5	-694.298681	-694.044150	-692.893776	-692.639245
110_6	-694.297931	-694.043425	-692.892881	-692.638374
110_7	-694.298167	-694.043693	-692.892735	-692.638260
110_8	-694.298163	-694.043566	-692.892847	-692.638250
110_9	-694.297543	-694.042879	-692.892741	-692.638077
110_10	-694.297826	-694.043058	-692.892832	-692.638064
**110-Me$^+$**				-732.196074
110-Me$^+$_1	-734.005503	-733.708938	-732.493156	-732.196592
110-Me$^+$_2	-734.004978	-733.708285	-732.492939	-732.196245
110-Me$^+$_3	-734.005286	-733.708632	-732.492784	-732.196130
110-Me$^+$_4	-734.003454	-733.706876	-732.491555	-732.194977
110-Me$^+$_5	-734.003879	-733.707420	-732.491316	-732.194857
110-Me$^+$_6	-734.003477	-733.706863	-732.490889	-732.194276
110-Me$^+$_7	-734.002346	-733.705804	-732.489462	-732.192920
**111**				-811.343906
111_1	-813.444134	-813.074459	-811.714672	-811.344997
111_2	-813.443692	-813.074281	-811.714320	-811.344908
111_3	-813.443446	-813.073843	-811.714442	-811.344838
111_4	-813.443464	-813.074163	-811.714115	-811.344814
111_5	-813.443512	-813.074100	-811.714181	-811.344769
111_6	-813.443359	-813.074075	-811.713993	-811.344709
111_7	-813.443535	-813.073944	-811.714031	-811.344439
111_8	-813.443263	-813.074065	-811.713544	-811.344346
111_9	-813.443010	-813.073580	-811.713204	-811.343774
111_10	-813.443261	-813.073753	-811.713240	-811.343732
**111-Me$^+$**				-850.900387
111-Me$^+$_1	-853.147827	-852.736571	-851.312573	-850.901317
111-Me$^+$_2	-853.147974	-852.736755	-851.312495	-850.901276
111-Me$^+$_3	-853.147664	-852.736508	-851.312067	-850.900911
111-Me$^+$_4	-853.147606	-852.736529	-851.311596	-850.900518
111-Me$^+$_5	-853.147636	-852.736304	-851.311812	-850.900480
111-Me$^+$_6	-853.147301	-852.735873	-851.311583	-850.900155
111-Me$^+$_7	-853.147212	-852.735918	-851.311100	-850.899806
111-Me$^+$_8	-853.145227	-852.734375	-851.310576	-850.899724
111-Me$^+$_9	-853.146428	-852.735308	-851.310844	-850.899724
111-Me$^+$_10	-853.145963	-852.735040	-851.310645	-850.899722
**112**				-695.037608
112_1	-696.769462	-696.468902	-695.339258	-695.038698
112_2	-696.769676	-696.469003	-695.339315	-695.038642
112_3	-696.768865	-696.468105	-695.338704	-695.037945
112_4	-696.768724	-696.468085	-695.338493	-695.037854
112_5	-696.769547	-696.468604	-695.338778	-695.037836
112_6	-696.768652	-696.467722	-695.338453	-695.037523
112_7	-696.767529	-696.466704	-695.337658	-695.036833
112_8	-696.767930	-696.467167	-695.337530	-695.036767
112_9	-696.768030	-696.467152	-695.337565	-695.036687
112_10	-696.768135	-696.467386	-695.337418	-695.036669

**112-Me$^+$**				-734.594689
112-Me$^+$_1	-736.475360	-736.132526	-734.938796	-734.595962
112-Me$^+$_2	-736.473285	-736.130519	-734.937123	-734.594357
112-Me$^+$_3	-736.473342	-736.130759	-734.936649	-734.594066
112-Me$^+$_4	-736.472913	-736.130122	-734.936560	-734.593768
112-Me$^+$_5	-736.473301	-736.130487	-734.936525	-734.593711
112-Me$^+$_6	-736.473289	-736.130547	-734.936394	-734.593652
112-Me$^+$_7	-736.473096	-736.130128	-734.936603	-734.593635
112-Me$^+$_8	-736.473265	-736.130472	-734.936380	-734.593587
112-Me$^+$_9	-736.472648	-736.129835	-734.936028	-734.593215
112-Me$^+$_10	-736.472653	-736.129812	-734.935768	-734.592926
**113**				-1038.176341
113_1	-1040.779623	-1040.396291	-1038.560595	-1038.177263
113_2	-1040.778992	-1040.395707	-1038.560149	-1038.176864
113_3	-1040.778859	-1040.395563	-1038.560122	-1038.176825
113_4	-1040.778649	-1040.396356	-1038.558901	-1038.176608
113_5	-1040.777678	-1040.394298	-1038.559923	-1038.176543
113_6	-1040.779189	-1040.395830	-1038.559796	-1038.176438
113_7	-1040.778632	-1040.395450	-1038.559536	-1038.176353
113_8	-1040.778565	-1040.395165	-1038.559751	-1038.176351
113_9	-1040.778663	-1040.395336	-1038.559570	-1038.176243
113_10	-1040.779538	-1040.396071	-1038.559499	-1038.176033
**113-Me$^+$**				-1077.733861
113-Me$^+$_1	-1080.486906	-1080.061453	-1078.160090	-1077.734637
113-Me$^+$_2	-1080.486242	-1080.060648	-1078.160028	-1077.734434
113-Me$^+$_3	-1080.486573	-1080.061027	-1078.159874	-1077.734329
113-Me$^+$_4	-1080.485697	-1080.060033	-1078.159823	-1077.734159
113-Me$^+$_5	-1080.486641	-1080.061138	-1078.159613	-1077.734111
113-Me$^+$_6	-1080.483600	-1080.058228	-1078.158831	-1077.733460
113-Me$^+$_7	-1080.486998	-1080.061129	-1078.159276	-1077.733407
113-Me$^+$_8	-1080.486695	-1080.060761	-1078.159322	-1077.733388
113-Me$^+$_9	-1080.484928	-1080.059193	-1078.158701	-1077.732966
113-Me$^+$_10	-1080.486487	-1080.060564	-1078.158732	-1077.732808
**114**				-773.359217
114_1	-775.359947	-774.999337	-773.721355	-773.360745
114_2	-775.359800	-774.999166	-773.720854	-773.360220
114_3	-775.359839	-774.999247	-773.720787	-773.360195
114_4	-775.358620	-774.997717	-773.719961	-773.359058
114_5	-775.358643	-774.997631	-773.719999	-773.358986
114_6	-775.358622	-774.997920	-773.719651	-773.358949
114_7	-775.358486	-774.997535	-773.719812	-773.358862
114_8	-775.357652	-774.996990	-773.719491	-773.358829
114_9	-775.358150	-774.997631	-773.719175	-773.358656
114_10	-775.358506	-774.997568	-773.719543	-773.358604
**114-Me$^+$**				-812.916776
114-Me$^+$_1	-815.065490	-814.662474	-813.321103	-812.918087
114-Me$^+$_2	-815.065391	-814.662205	-813.320871	-812.917685
114-Me$^+$_3	-815.065840	-814.662598	-813.320919	-812.917677

114-Me$^+$_4	-815.064875	-814.661925	-813.320434	-812.917483
114-Me$^+$_5	-815.064827	-814.661872	-813.320266	-812.917311
114-Me$^+$_6	-815.065044	-814.662293	-813.320048	-812.917296
114-Me$^+$_7	-815.064918	-814.661941	-813.320185	-812.917208
114-Me$^+$_8	-815.064271	-814.661731	-813.319682	-812.917142
114-Me$^+$_9	-815.064768	-814.661892	-813.319810	-812.916933
114-Me$^+$_10	-815.064338	-814.661584	-813.319640	-812.916886
**115**				-695.034802
115_1	-696.775018	-696.473798	-695.337264	-695.036044
115_2	-696.773868	-696.472765	-695.337118	-695.036016
115_3	-696.773942	-696.472760	-695.336405	-695.035223
115_4	-696.773798	-696.472344	-695.336597	-695.035143
115_5	-696.773832	-696.472713	-695.335952	-695.034834
115_6	-696.772606	-696.471280	-695.336067	-695.034741
115_7	-696.773726	-696.472593	-695.335715	-695.034582
115_8	-696.773083	-696.471843	-695.335677	-695.034438
115_9	-696.772825	-696.471643	-695.335565	-695.034383
115_10	-696.772897	-696.471754	-695.335420	-695.034277
**115-Me$^+$**				-734.593073
115-Me$^+$_1	-736.479952	-736.136800	-734.937424	-734.594272
115-Me$^+$_2	-736.479848	-736.136499	-734.936971	-734.593622
115-Me$^+$_3	-736.479510	-736.136260	-734.936263	-734.593012
115-Me$^+$_4	-736.478156	-736.134954	-734.935157	-734.591955
115-Me$^+$_5	-736.477305	-736.133987	-734.934995	-734.591677
115-Me$^+$_6	-736.478036	-736.134740	-734.934846	-734.591550
115-Me$^+$_7	-736.477364	-736.134031	-734.934838	-734.591506
115-Me$^+$_8	-736.478124	-736.134734	-734.934891	-734.591501
115-Me$^+$_9	-736.477348	-736.133942	-734.934777	-734.591372
115-Me$^+$_10	-736.477121	-736.133836	-734.934641	-734.591356
**116**	-696.762757	-696.463368	-695.342492	-695.043102
**116-Me$^+$**	-736.470129	-736.128730	-734.943311	-734.601912
**117**				-695.035243
117_1	-696.764415	-696.463812	-695.336484	-695.035881
117_2	-696.763341	-696.462502	-695.335357	-695.034518
117_3	-696.762638	-696.461873	-695.334389	-695.033624
117_4	-696.762440	-696.461212	-695.334289	-695.033061
117_5	-696.762039	-696.461116	-695.333609	-695.032686
117_6	-696.762066	-696.461454	-695.333293	-695.032681
117_7	-696.760293	-696.459351	-695.332179	-695.031238
**117-Me$^+$**				-734.594174
117-Me$^+$_1	-736.471871	-736.129126	-734.937546	-734.594802
117-Me$^+$_2	-736.471122	-736.128196	-734.936952	-734.594025
117-Me$^+$_3	-736.469445	-736.126517	-734.935041	-734.592113
117-Me$^+$_4	-736.469539	-736.126489	-734.935065	-734.592016
117-Me$^+$_5	-736.469481	-736.126460	-734.934993	-734.591972
117-Me$^+$_6	-736.468954	-736.125966	-734.934333	-734.591345
117-Me$^+$_7	-736.469182	-736.126158	-734.934256	-734.591233
117-Me$^+$_8	-736.468365	-736.125385	-734.933808	-734.590828

**118**				-771.026414
118_1	-772.951878	-772.635396	-771.343606	-771.027124
118_2	-772.950858	-772.634361	-771.342818	-771.026322
118_3	-772.949058	-772.632482	-771.342119	-771.025543
118_4	-772.949803	-772.633309	-771.341926	-771.025432
118_5	-772.950813	-772.634355	-771.340896	-771.024438
118_6	-772.948254	-772.631658	-771.340896	-771.024300
118_7	-772.946664	-772.630120	-771.340017	-771.023473
**118-Me$^+$**				-810.586007
118-Me$^+$_1	-812.659316	-812.300771	-810.945189	-810.586644
118-Me$^+$_2	-812.657985	-812.299400	-810.943854	-810.585269
118-Me$^+$_3	-812.658201	-812.299520	-810.943661	-810.584980
118-Me$^+$_4	-812.657911	-812.299181	-810.943708	-810.584977
118-Me$^+$_5	-812.655693	-812.296909	-810.942139	-810.583355
**119**				-808.925527
119_1	-810.952285	-810.630998	-809.247810	-808.926523
119_2	-810.950983	-810.629604	-809.247147	-808.925768
119_3	-810.950852	-810.629411	-809.247009	-808.925568
119_4	-810.949659	-810.628393	-809.246334	-808.925069
119_5	-810.949518	-810.628142	-809.245961	-808.924585
119_6	-810.949138	-810.627765	-809.245826	-808.924453
119_7	-810.949582	-810.628069	-809.245939	-808.924427
119_8	-810.949181	-810.627638	-809.245818	-808.924275
119_9	-810.948110	-810.626634	-809.245078	-808.923602
119_10	-810.948024	-810.626645	-809.244832	-808.923452
**119-Me$^+$**				-848.485656
119-Me$^+$_1	-850.660901	-850.297246	-848.849889	-848.486233
119-Me$^+$_2	-850.660825	-850.297065	-848.849980	-848.486220
119-Me$^+$_3	-850.660927	-850.297213	-848.849546	-848.485833
119-Me$^+$_4	-850.660715	-850.296892	-848.849535	-848.485712
119-Me$^+$_5	-850.659964	-850.296287	-848.849114	-848.485438
119-Me$^+$_6	-850.659263	-850.295624	-848.848703	-848.485065
119-Me$^+$_7	-850.659420	-850.295725	-848.848509	-848.484814
119-Me$^+$_8	-850.658729	-850.295072	-848.847943	-848.484286
119-Me$^+$_9	-850.658177	-850.294507	-848.847485	-848.483814
119-Me$^+$_10	-850.658818	-850.295069	-848.847535	-848.483786
**120**				-812.520264
120_1	-814.666280	-814.275333	-812.912789	-812.521842
120_2	-814.667375	-814.276445	-812.912691	-812.521761
120_3	-814.666120	-814.275086	-812.912277	-812.521243
120_4	-814.665189	-814.274055	-812.912295	-812.521161
120_5	-814.666141	-814.275205	-812.912010	-812.521074
120_6	-814.666247	-814.275380	-812.911927	-812.521059
120_7	-814.665089	-814.274045	-812.912063	-812.521019
120_8	-814.666185	-814.275235	-812.911940	-812.520990
120_9	-814.665077	-814.273919	-812.911925	-812.520767
120_10	-814.664967	-814.273871	-812.911814	-812.520718
**120-Me$^+$**				-852.080761

120-Me$^+$_1	-854.374538	-853.941503	-852.515266	-852.082231
120-Me$^+$_2	-854.374190	-853.941393	-852.514736	-852.081940
120-Me$^+$_3	-854.373488	-853.940383	-852.514723	-852.081618
120-Me$^+$_4	-854.373538	-853.940333	-852.514725	-852.081520
120-Me$^+$_5	-854.373439	-853.940354	-852.514587	-852.081501
120-Me$^+$_6	-854.372400	-853.939482	-852.514139	-852.081221
120-Me$^+$_7	-854.372327	-853.939451	-852.514043	-852.081167
120-Me$^+$_8	-854.373274	-853.940189	-852.514213	-852.081127
120-Me$^+$_9	-854.373179	-853.940210	-852.514058	-852.081088
120-Me$^+$_10	-854.372322	-853.939355	-852.514046	-852.081079
**121**				-1044.015602
121_1	-1046.839178	-1046.334212	-1044.521476	-1044.016511
121_2	-1046.837691	-1046.332564	-1044.519801	-1044.014674
121_3	-1046.837156	-1046.332032	-1044.519312	-1044.014188
121_4	-1046.836104	-1046.331159	-1044.518985	-1044.014040
121_5	-1046.836771	-1046.331715	-1044.518903	-1044.013847
121_6	-1046.836643	-1046.331853	-1044.518182	-1044.013392
121_7	-1046.835104	-1046.330102	-1044.518291	-1044.013289
121_8	-1046.835141	-1046.329905	-1044.518274	-1044.013038
121_9	-1046.834729	-1046.329608	-1044.517996	-1044.012876
121_10	-1046.836320	-1046.331111	-1044.517996	-1044.012787
**121-Me$^+$**				-1083.582284
121-Me$^+$_1	-1086.554290	-1086.006709	-1084.130727	-1083.583146
121-Me$^+$_2	-1086.553596	-1086.006194	-1084.130105	-1083.582703
121-Me$^+$_3	-1086.551723	-1086.004412	-1084.128335	-1083.581025
121-Me$^+$_4	-1086.551798	-1086.004257	-1084.128346	-1083.580805
121-Me$^+$_5	-1086.551906	-1086.004324	-1084.128156	-1083.580574
121-Me$^+$_6	-1086.551905	-1086.004303	-1084.128159	-1083.580557
121-Me$^+$_7	-1086.551906	-1086.004284	-1084.128172	-1083.580550
121-Me$^+$_8	-1086.551403	-1086.003945	-1084.127374	-1083.579916
121-Me$^+$_9	-1086.550794	-1086.003232	-1084.127234	-1083.579671
121-Me$^+$_10	-1086.550796	-1086.003243	-1084.127223	-1083.579670
**122**	-814.630252	-814.240491	-812.904321	-812.514560
**122-Me$^+$**	-854.343293	-853.911353	-852.510367	-852.078427
**123**				-926.504276
123_1	-928.926292	-928.511862	-926.919497	-926.505067
123_2	-928.926236	-928.511821	-926.919422	-926.505006
123_3	-928.925350	-928.510849	-926.918306	-926.503805
123_4	-928.922901	-928.508590	-926.917255	-926.502943
123_5	-928.924535	-928.510355	-926.917111	-926.502931
123_6	-928.924810	-928.510251	-926.917264	-926.502705
123_7	-928.924830	-928.510287	-926.917230	-926.502686
123_8	-928.924811	-928.510267	-926.917216	-926.502672
123_9	-928.924664	-928.510014	-926.917154	-926.502504
123_10	-928.922635	-928.508092	-926.916522	-926.501979
**123-Me$^+$**				-966.069098
123-Me$^+$_1	-968.639512	-968.182722	-966.526451	-966.069661
123-Me$^+$_2	-968.639326	-968.182479	-966.526277	-966.069430

123-Me$^+$_3	-968.638236	-968.181448	-966.525667	-966.068879
123-Me$^+$_4	-968.638674	-968.181748	-966.525099	-966.068173
123-Me$^+$_5	-968.638585	-968.181718	-966.524450	-966.067583
123-Me$^+$_6	-968.637786	-968.180797	-966.524186	-966.067196
123-Me$^+$_7	-968.634666	-968.178090	-966.522303	-966.065726
123-Me$^+$_8	-968.634770	-968.177865	-966.522539	-966.065634
123-Me$^+$_9	-968.635863	-968.178898	-966.522588	-966.065624
**124**				-1044.015602
124_1	-1046.839178	-1046.334212	-1044.521476	-1044.016511
124_2	-1046.837691	-1046.332564	-1044.519801	-1044.014674
124_3	-1046.837156	-1046.332032	-1044.519312	-1044.014188
124_4	-1046.836104	-1046.331159	-1044.518985	-1044.014040
124_5	-1046.836771	-1046.331715	-1044.518903	-1044.013847
124_6	-1046.836643	-1046.331853	-1044.518182	-1044.013392
124_7	-1046.835104	-1046.330102	-1044.518291	-1044.013289
124_8	-1046.835141	-1046.329905	-1044.518274	-1044.013038
124_9	-1046.834729	-1046.329608	-1044.517996	-1044.012876
124_10	-1046.836320	-1046.331111	-1044.517996	-1044.012787
**124-Me$^+$**				-1083.582284
124-Me$^+$_1	-1086.554290	-1086.006709	-1084.130727	-1083.583146
124-Me$^+$_2	-1086.553596	-1086.006194	-1084.130105	-1083.582703
124-Me$^+$_3	-1086.551723	-1086.004412	-1084.128335	-1083.581025
124-Me$^+$_4	-1086.551798	-1086.004257	-1084.128346	-1083.580805
124-Me$^+$_5	-1086.551906	-1086.004324	-1084.128156	-1083.580574
124-Me$^+$_6	-1086.551905	-1086.004303	-1084.128159	-1083.580557
124-Me$^+$_7	-1086.551906	-1086.004284	-1084.128172	-1083.580550
124-Me$^+$_8	-1086.551403	-1086.003945	-1084.127374	-1083.579916
124-Me$^+$_9	-1086.550794	-1086.003232	-1084.127234	-1083.579671
124-Me$^+$_10	-1086.550796	-1086.003243	-1084.127223	-1083.579670
**125**				-1063.765841
125_1	-1065.909798	-1065.748375	-1063.927405	-1063.765982
125_2	-1065.907487	-1065.746341	-1063.924380	-1063.763234
**125-Me$^+$**				-1103.242119
125-Me$^+$_1	-1105.538620	-1105.337537	-1103.443569	-1103.242486
125-Me$^+$_2	-1105.538413	-1105.337273	-1103.442927	-1103.241786
125-Me$^+$_3	-1105.538404	-1105.337220	-1103.442749	-1103.241565
**126**				-1015.748491
126_1	-1017.90124	-1017.70195	-1015.94844	-1015.749146
126_2	-1017.90136	-1017.70204	-1015.94786	-1015.748535
126_3	-1017.90111	-1017.70193	-1015.94767	-1015.748491
126_4	-1017.90068	-1017.70146	-1015.94750	-1015.748276
126_5	-1017.90072	-1017.70151	-1015.94733	-1015.748124
126_6	-1017.90044	-1017.70121	-1015.94711	-1015.747879
126_7	-1017.90012	-1017.70097	-1015.94680	-1015.747648
126_8	-1017.89962	-1017.70043	-1015.94592	-1015.746726
**126-Me$^+$**				-1055.237299
126-Me$^+$_1	-1057.54084	-1057.30146	-1055.47726	-1055.237881
126-Me$^+$_2	-1057.54039	-1057.30099	-1055.47685	-1055.237449

126-Me$^+$_3	-1057.53992	-1057.30047	-1055.47630	-1055.236842
126-Me$^+$_4	-1057.53986	-1057.30023	-1055.47635	-1055.236713
126-Me$^+$_5	-1057.53954	-1057.30000	-1055.47587	-1055.236330
126-Me$^+$_6	-1057.53863	-1057.29927	-1055.47519	-1055.235833
**127**				-862.541604
127_1	-864.279246	-864.131029	-862.689925	-862.541708
127_2	-864.276377	-864.128197	-862.686684	-862.538503
**127-Me$^+$**				-902.036063
127-Me$^+$_1	-903.925302	-903.736765	-902.224644	-902.036106
127-Me$^+$_2	-903.921105	-903.732767	-902.220104	-902.031766
**128**				-830.525147
128_1	-832.27063	-832.09720	-830.69891	-830.525474
128_2	-832.27006	-832.09676	-830.69825	-830.524953
128_3	-832.26921	-832.09581	-830.69703	-830.523630
**128-Me$^+$**				-870.027580
128-Me$^+$_1	-871.92216	-871.70839	-870.24173	-870.027961
128-Me$^+$_2	-871.92175	-871.70798	-870.24102	-870.027252
128-Me$^+$_3	-871.92110	-871.70738	-870.24013	-870.026410
**129**				-661.309283
129_1	-662.641182	-662.506340	-661.444157	-661.309315
129_2	-662.636622	-662.502003	-661.439242	-661.304623
**129-Me$^+$**	-702.306899	-702.131410	-701.000998	-700.825509
**130**				-967.621200
130_1	-969.772042	-969.537888	-967.855586	-967.621433
130_2	-969.771760	-969.537598	-967.854847	-967.620685
**130-Me$^+$**				-1007.137933
130-Me$^+$_1	-1009.439171	-1009.163974	-1007.413406	-1007.138210
130-Me$^+$_2	-1009.439249	-1009.163947	-1007.413387	-1007.138085
130-Me$^+$_3	-1009.439163	-1009.163921	-1007.412895	-1007.137653
130-Me$^+$_4	-1009.438955	-1009.163438	-1007.412305	-1007.136788
**131**				-645.299002
131_1	-646.63514	-646.48791	-645.44648	-645.299252
131_2	-646.63453	-646.48729	-645.44560	-645.298354
**131-Me$^+$**	-686.30489	-686.11664	-685.00816	-684.819911
**132**	-800.181735	-799.985236	-798.633392	-798.436893
**132-Me$^+$**	-839.853874	-839.616196	-838.197637	-837.959960
**133**				-629.253529
133_1	-630.589506	-630.430780	-629.412284	-629.253557
133_2	-630.589518	-630.430741	-629.412277	-629.253500
**133-Me$^+$**	-670.269955	-670.069850	-668.985319	-668.785213
**134**				-553.221541
134_1	-554.372595	-554.227219	-553.366992	-553.221616
134_2	-554.371092	-554.225822	-553.363240	-553.217969
**134-Me$^+$**	-594.070509	-593.883218	-592.956352	-592.769061
**135**				-646.375944
135_1	-647.753507	-647.583537	-646.546109	-646.376139
135_2	-647.748324	-647.578580	-646.542678	-646.372933
135_3	-647.752229	-647.582235	-646.542579	-646.372585

**135-Me⁺**				-685.925946
135-Me⁺_1	-687.454640	-687.242422	-686.138377	-685.926159
135-Me⁺_2	-687.451879	-687.239797	-686.135761	-685.923679
135-Me⁺_3	-687.452568	-687.240292	-686.134316	-685.922040
**136**	-555.613386	-555.444294	-554.587905	-554.418813
**136-Me⁺**	-595.312255	-595.100698	-594.181736	-593.970179
**137**	-859.157693	-858.868889	-857.442333	-857.153529
**137-Me⁺**	-898.858204	-898.526815	-897.038003	-896.706614
**138**				-593.582407
138_1	-594.912460	-594.713405	-593.781875	-593.582820
138_2	-594.911897	-594.712863	-593.781491	-593.582457
138_3	-594.911567	-594.712449	-593.780944	-593.581826
138_4	-594.910255	-594.711187	-593.780363	-593.581295
**138-Me⁺**				-633.135702
138-Me⁺_1	-634.612662	-634.371268	-633.377125	-633.135731
138-Me⁺_2	-634.612330	-634.371041	-633.376999	-633.135710
**139**	-672.310022	-672.073007	-670.987543	-670.750528
**139-Me⁺**				-710.305378
139-Me⁺_1	-712.012462	-711.733215	-710.584874	-710.305627
139-Me⁺_2	-712.011450	-711.732126	-710.583364	-710.304040
**140**				-592.386346
140_1	-593.680457	-593.504439	-592.562626	-592.386608
140_2	-593.676108	-593.500125	-592.561321	-592.385338
**140-Me⁺**	-633.383249	-633.165138	-632.159344	-631.941233
**141**				-739.532014
141_1	-741.136937	-740.941925	-739.727270	-739.532258
141_2	-741.133992	-740.939295	-739.725308	-739.530612
**141-Me⁺**				-779.087671
141-Me⁺_1	-780.841677	-780.604346	-779.325103	-779.087772
141-Me⁺_2	-780.838942	-780.601734	-779.321746	-779.084538
**142**				-632.745996
142_1	-634.210751	-633.981838	-632.975345	-632.746432
142_2	-634.208941	-633.979989	-632.974104	-632.745152
142_3	-634.209527	-633.980598	-632.974026	-632.745097
**142-Me⁺**				-672.301679
142-Me⁺_1	-673.913191	-673.642067	-672.573214	-672.302090
142-Me⁺_2	-673.911201	-673.640126	-672.571899	-672.300824
142-Me⁺_3	-673.911409	-673.640171	-672.571286	-672.300048
**143**	-947.357227	-946.914125	-945.337189	-944.894086
**143-Me⁺**	-987.060796	-986.575336	-984.935257	-984.449797
**144**				-671.908621
144_1	-673.507753	-673.248662	-672.168367	-671.909277
144_2	-673.507781	-673.248643	-672.168324	-671.909185
144_3	-673.508252	-673.249489	-672.167429	-671.908667
144_4	-673.507613	-673.248527	-672.167737	-671.908651
144_5	-673.508266	-673.249409	-672.167297	-671.908440
144_6	-673.505966	-673.247245	-672.166923	-671.908202
144_7	-673.506389	-673.247443	-672.167003	-671.908056

144_8	-673.506356	-673.247470	-672.166932	-671.908046
144_9	-673.506322	-673.247458	-672.166717	-671.907853
144_10	-673.506556	-673.247720	-672.166537	-671.907702
**144-Me$^+$**				-711.464638
144-Me$^+$_1	-713.211526	-712.910519	-711.766333	-711.465326
144-Me$^+$_2	-713.211454	-712.910493	-711.766103	-711.465142
144-Me$^+$_3	-713.210533	-712.909391	-711.766152	-711.465010
144-Me$^+$_4	-713.209222	-712.908141	-711.765480	-711.464399
144-Me$^+$_5	-713.209582	-712.908530	-711.765205	-711.464153
144-Me$^+$_6	-713.209840	-712.908604	-711.765328	-711.464092
144-Me$^+$_7	-713.209552	-712.908507	-711.765000	-711.463954
144-Me$^+$_8	-713.209447	-712.908339	-711.764956	-711.463848
144-Me$^+$_9	-713.209447	-712.908339	-711.764956	-711.463848
144-Me$^+$_10	-713.209837	-712.908734	-711.764513	-711.463410
**145**				-709.903441
145_1	-711.598412	-711.331145	-710.171009	-709.903742
145_2	-711.598900	-711.331649	-710.170942	-709.903691
145_3	-711.598595	-711.331358	-710.170683	-709.903446
145_4	-711.598519	-711.331291	-710.170524	-709.903296
145_5	-711.596458	-711.329277	-710.167980	-709.900799
145_6	-711.596364	-711.329123	-710.167846	-709.900605
145_7	-711.596380	-711.329152	-710.167786	-709.900558
**145-Me$^+$**				-749.460174
145-Me$^+$_1	-751.302468	-750.993027	-749.769987	-749.460545
145-Me$^+$_2	-751.302277	-750.992936	-749.769531	-749.460191
145-Me$^+$_3	-751.301946	-750.992403	-749.769521	-749.459977
145-Me$^+$_4	-751.301903	-750.992414	-749.769309	-749.459819
145-Me$^+$_5	-751.299604	-750.990069	-749.766463	-749.456928
**146**				-711.070871
146_1	-712.804267	-712.515264	-711.360842	-711.071840
146_2	-712.805097	-712.516206	-711.360442	-711.071551
146_3	-712.805729	-712.517083	-711.359617	-711.070971
146_4	-712.802954	-712.514195	-711.359596	-711.070837
146_5	-712.803010	-712.514253	-711.359541	-711.070784
146_6	-712.804943	-712.515934	-711.359677	-711.070669
146_7	-712.803934	-712.515137	-711.359350	-711.070553
146_8	-712.804036	-712.515164	-711.359305	-711.070433
146_9	-712.803502	-712.514769	-711.358797	-711.070064
146_10	-712.803462	-712.514684	-711.358651	-711.069874
**146-Me$^+$**				-750.627674
146-Me$^+$_1	-752.509783	-752.178879	-750.959273	-750.628369
146-Me$^+$_2	-752.508731	-752.177810	-750.959227	-750.628306
146-Me$^+$_3	-752.508881	-752.177669	-750.959290	-750.628077
146-Me$^+$_4	-752.507615	-752.176463	-750.958716	-750.627564
146-Me$^+$_5	-752.507884	-752.176808	-750.958230	-750.627155
146-Me$^+$_6	-752.507878	-752.176808	-750.958153	-750.627082
146-Me$^+$_7	-752.508065	-752.176832	-750.957945	-750.626713
146-Me$^+$_8	-752.507806	-752.176680	-750.957789	-750.626663

146-Me$^+$_9	-752.506573	-752.175343	-750.957739	-750.626509
146-Me$^+$_10	-752.508109	-752.176915	-750.957392	-750.626198
**147**				-648.772854
147_1	-650.235656	-650.017930	-648.990775	-648.773049
147_2	-650.231210	-650.013548	-648.988511	-648.770849
**147-Me$^+$**				-688.330440
147-Me$^+$_1	-689.940300	-689.679557	-688.591432	-688.330689
147-Me$^+$_2	-689.938489	-689.678007	-688.589575	-688.329092
**148**				-631.579114
148_1	-633.006312	-632.799344	-631.786516	-631.579548
148_2	-633.001009	-632.794068	-631.785296	-631.578356
148_3	-633.003977	-632.797002	-631.785182	-631.578207
**148-Me$^+$**	-672.711447	-672.462352	-671.386690	-671.137594
**149**	-744.858022	-744.591811	-743.397028	-743.130817
**149-Me$^+$**				-782.692371
149-Me$^+$_1	-784.567398	-784.257845	-783.002042	-782.692488
149-Me$^+$_2	-784.567461	-784.257561	-783.002353	-782.692453
149-Me$^+$_3	-784.567354	-784.257526	-783.002278	-782.692450
149-Me$^+$_4	-784.567138	-784.257714	-783.001232	-782.691809
**150**				-724.708040
150_1	-726.368916	-726.137529	-724.939848	-724.708461
150_2	-726.368880	-726.137503	-724.938817	-724.707440
150_3	-726.368364	-726.136892	-724.938767	-724.707295
**150-Me$^+$**				-764.269777
150-Me$^+$_1	-766.080145	-765.806026	-764.543952	-764.269833
150-Me$^+$_1	-766.080170	-765.806119	-764.543764	-764.269713
**151**				-727.100565
151_1	-728.833065	-728.555437	-727.379179	-727.101551
151_2	-728.832791	-728.555304	-727.378869	-727.101382
151_3	-728.832478	-728.554969	-727.378220	-727.100711
151_4	-728.832250	-728.554592	-727.378252	-727.100594
151_5	-728.831637	-728.554016	-727.377837	-727.100216
151_6	-728.831969	-728.554357	-727.377783	-727.100171
151_7	-728.828608	-728.551296	-727.376667	-727.099355
151_8	-728.831437	-728.553801	-727.376862	-727.099226
151_9	-728.827896	-728.550438	-727.376560	-727.099103
151_10	-728.831815	-728.554405	-727.376359	-727.098949
**151-Me$^+$**				-766.662301
151-Me$^+$_1	-768.541460	-768.221034	-766.983412	-766.662987
151-Me$^+$_2	-768.540662	-768.220508	-766.982710	-766.662556
151-Me$^+$_3	-768.540794	-768.220366	-766.982951	-766.662523
151-Me$^+$_4	-768.540307	-768.220030	-766.982738	-766.662461
151-Me$^+$_5	-768.540732	-768.220305	-766.982722	-766.662295
151-Me$^+$_6	-768.540169	-768.219778	-766.982535	-766.662144
151-Me$^+$_7	-768.539724	-768.219502	-766.982350	-766.662128
151-Me$^+$_8	-768.540215	-768.219795	-766.982508	-766.662088
151-Me$^+$_9	-768.540172	-768.219810	-766.982387	-766.662025
151-Me$^+$_10	-768.540013	-768.219994	-766.981941	-766.661922

**152**				-881.436754
152_1	-883.628797	-883.275305	-881.790791	-881.437299
152_2	-883.627848	-883.274197	-881.789733	-881.436081
152_3	-883.623433	-883.270213	-881.788157	-881.434937
152_4	-883.624317	-883.270830	-881.788259	-881.434771
152_5	-883.625521	-883.272045	-881.787316	-881.433839
**152-Me$^+$**				-921.000779
152-Me$^+$_1	-923.339657	-922.943315	-921.397651	-921.001309
152-Me$^+$_2	-923.339069	-922.942789	-921.396986	-921.000706
152-Me$^+$_3	-923.339117	-922.942770	-921.396878	-921.000531
152-Me$^+$_4	-923.338499	-922.942079	-921.396147	-920.999727
152-Me$^+$_5	-923.338378	-922.941924	-921.395916	-920.999462
**153**				-805.427349
153_1	-807.428712	-807.091308	-805.765937	-805.428534
153_2	-807.427351	-807.089865	-805.764986	-805.427500
153_3	-807.427480	-807.090254	-805.764719	-805.427494
153_4	-807.426949	-807.089693	-805.764649	-805.427392
153_5	-807.425977	-807.088800	-805.764118	-805.426941
153_6	-807.427533	-807.089925	-805.764377	-805.426770
153_7	-807.424096	-807.087036	-805.763419	-805.426359
153_8	-807.426288	-807.089186	-805.763300	-805.426198
153_9	-807.426870	-807.089581	-805.763443	-805.426154
153_10	-807.425861	-807.088804	-805.762917	-805.425860
**153-Me$^+$**				-844.993075
153-Me$^+$_1	-847.139670	-846.759504	-845.374073	-844.993907
153-Me$^+$_2	-847.139902	-846.759912	-845.373891	-844.993902
153-Me$^+$_3	-847.139992	-846.759971	-845.373760	-844.993738
153-Me$^+$_4	-847.139015	-846.759109	-845.373000	-844.993095
153-Me$^+$_5	-847.139025	-846.759046	-845.373031	-844.993052
153-Me$^+$_6	-847.138400	-846.758198	-845.373004	-844.992802
153-Me$^+$_7	-847.138378	-846.758401	-845.372566	-844.992588
153-Me$^+$_8	-847.138466	-846.758489	-845.372504	-844.992526
153-Me$^+$_9	-847.138220	-846.758347	-845.372252	-844.992380
153-Me$^+$_10	-847.137668	-846.757424	-845.372618	-844.992374
**154**				-803.095136
154_1	-805.020625	-804.727496	-803.388388	-803.095259
154_2	-805.016845	-804.723973	-803.387947	-803.095075
154_3	-805.016469	-804.723358	-803.388154	-803.095042
154_4	-805.020155	-804.727022	-803.387829	-803.094696
154_5	-805.019613	-804.726318	-803.387988	-803.094694
154_6	-805.019321	-804.726120	-803.386771	-803.093569
**154-Me$^+$**				-842.664064
154-Me$^+$_1	-844.736241	-844.400533	-842.999902	-842.664195
154-Me$^+$_2	-844.736241	-844.400515	-842.999740	-842.664015
154-Me$^+$_3	-844.736264	-844.400442	-842.999770	-842.663947
**155**				-857.031261
155_1	-859.055918	-858.769217	-857.318317	-857.031616
155_2	-859.055442	-858.768696	-857.317784	-857.031038

155_3	-859.055771	-858.769138	-857.317127	-857.030494
**155-Me⁺**				-896.601997
155-Me⁺_1	-898.778486	-898.448575	-896.931945	-896.602034
155-Me⁺_2	-898.778521	-898.448578	-896.931930	-896.601988
**156**				-1092.127843
156_1	-1094.946647	-1094.476804	-1092.597938	-1092.128095
156_2	-1094.947010	-1094.476956	-1092.598080	-1092.128026
156_3	-1094.944711	-1094.474939	-1092.595098	-1092.125326
156_4	-1094.944948	-1094.474994	-1092.595132	-1092.125178
156_5	-1094.944484	-1094.474610	-1092.595005	-1092.125131
**156-Me⁺**				-1131.698645
156-Me⁺_1	-1134.665730	-1134.152886	-1132.211887	-1131.699043
156-Me⁺_2	-1134.664879	-1134.151537	-1132.212336	-1131.698994
156-Me⁺_3	-1134.665273	-1134.152189	-1132.211957	-1131.698874
156-Me⁺_4	-1134.665134	-1134.152342	-1132.211345	-1131.698553
156-Me⁺_5	-1134.665096	-1134.152228	-1132.211392	-1131.698525
156-Me⁺_6	-1134.664606	-1134.151194	-1132.211702	-1131.698291
156-Me⁺_7	-1134.664050	-1134.151184	-1132.210230	-1131.697364
156-Me⁺_8	-1134.663970	-1134.150602	-1132.210732	-1131.697364
156-Me⁺_9	-1134.663939	-1134.150521	-1132.210586	-1131.697169
156-Me⁺_10	-1134.665730	-1134.152886	-1132.211887	-1131.699043
**157**				-974.613953
157_1	-977.032128	-976.652701	-974.994221	-974.614793
157_2	-977.032104	-976.652698	-974.993900	-974.614495
157_3	-977.032271	-976.652867	-974.993712	-974.614308
157_4	-977.032774	-976.653504	-974.993277	-974.614007
157_5	-977.032200	-976.652723	-974.993418	-974.613941
157_6	-977.032577	-976.653124	-974.993166	-974.613713
157_7	-977.032769	-976.653309	-974.993171	-974.613711
157_8	-977.032518	-976.653193	-974.992993	-974.613669
157_9	-977.032452	-976.653142	-974.992254	-974.612944
157_10	-977.032432	-976.652974	-974.992342	-974.612884
**157-Me⁺**				-1014.192381
157-Me⁺_1	-1016.760342	-1016.337951	-1014.615588	-1014.193198
157-Me⁺_2	-1016.760331	-1016.338110	-1014.614755	-1014.192535
157-Me⁺_3	-1016.759733	-1016.337332	-1014.614713	-1014.192312
157-Me⁺_4	-1016.759644	-1016.337256	-1014.614396	-1014.192008
157-Me⁺_5	-1016.759517	-1016.337144	-1014.614271	-1014.191898
157-Me⁺_6	-1016.759824	-1016.337551	-1014.614070	-1014.191798
157-Me⁺_7	-1016.759574	-1016.337067	-1014.614111	-1014.191604
157-Me⁺_8	-1016.759348	-1016.336838	-1014.613623	-1014.191113
157-Me⁺_9	-1016.759349	-1016.336828	-1014.613633	-1014.191112
157-Me⁺_10	-1016.758717	-1016.336451	-1014.612355	-1014.190089
**158**				-913.463189
158_1	-915.649231	-915.319699	-913.792946	-913.463414
158_2	-915.649013	-915.319484	-913.792249	-913.462720
**158-Me⁺**				-953.047150
158-Me⁺_1	-955.376968	-955.003849	-953.420380	-953.047261

158-Me$^+$_2	-955.377022	-955.003770	-953.420258	-953.047006
**159**				-735.926353
159_1	-737.407428	-737.285323	-736.048675	-735.926570
159_2	-737.405757	-737.283733	-736.046614	-735.924590
**159-Me$^+$**				-775.407539
159-Me$^+$_1	-777.037624	-776.874607	-775.570907	-775.407891
159-Me$^+$_2	-777.037371	-776.874343	-775.570713	-775.407686
159-Me$^+$_3	-777.037024	-776.873984	-775.570279	-775.407240
159-Me$^+$_4	-777.036847	-776.873802	-775.570189	-775.407144
159-Me$^+$_5	-777.035224	-776.872276	-775.568212	-775.405264
**160**				-1181.256025
160_1	-1183.799363	-1183.546196	-1181.509420	-1181.256254
160_2	-1183.800003	-1183.546851	-1181.509052	-1181.255900
160_3	-1183.797188	-1183.543799	-1181.506952	-1181.253563
**160-Me$^+$**				-1220.752062
160-Me$^+$_1	-1223.445910	-1223.152071	-1221.046509	-1220.752669
160-Me$^+$_2	-1223.444104	-1223.150195	-1221.045424	-1220.751515
160-Me$^+$_3	-1223.444104	-1223.150196	-1221.045423	-1220.751514
160-Me$^+$_4	-1223.445058	-1223.151426	-1221.044446	-1220.750814
160-Me$^+$_5	-1223.440906	-1223.147260	-1221.043463	-1220.749817
**161**				-643.979959
161_1	-645.276269	-645.154357	-644.102203	-643.980291
161_2	-645.275320	-645.153422	-644.101130	-643.979231
161_3	-645.271978	-645.150130	-644.098060	-643.976212
161_4	-645.272604	-645.150787	-644.097933	-643.976116
**161-Me$^+$**				-683.479754
161-Me$^+$_1	-684.924001	-684.761103	-683.642933	-683.480035
161-Me$^+$_2	-684.923686	-684.760800	-683.642496	-683.479610
161-Me$^+$_3	-684.923394	-684.760501	-683.642263	-683.479370
**162**				-940.861056
162_1	-942.867115	-942.657781	-941.070620	-940.861286
162_2	-942.867221	-942.657889	-941.070053	-940.860721
162_3	-942.863876	-942.654429	-941.066500	-940.857053
**162-Me$^+$**				-980.372421
162-Me$^+$_1	-982.527834	-982.277771	-980.622587	-980.372524
162-Me$^+$_2	-982.525488	-982.275453	-980.619333	-980.369298
**163**	-553.138493	-553.016941	-552.148634	-552.027082
**163-Me$^+$**	-592.808100	-592.645309	-591.712156	-591.549365
**164**				-700.465110
164_1	-701.932600	-701.767373	-700.630444	-700.465218
164_2	-701.932528	-701.767268	-700.630440	-700.465180
164_3	-701.929786	-701.764517	-700.627125	-700.461856
164_4	-701.929808	-701.764429	-700.627074	-700.461695
**164-Me$^+$**	-741.607452	-741.401061	-740.197470	-739.991080
**165**				-1133.231793
165_1	-1135.78944	-1135.49847	-1133.52307	-1133.232100
165_2	-1135.78902	-1135.49826	-1133.52196	-1133.231193
165_3	-1135.78696	-1135.49586	-1133.51968	-1133.228588

**165-Me$^+$**				-1172.760717
165-Me$^+$_1	-1175.47101	-1175.13855	-1173.09355	-1172.761088
165-Me$^+$_2	-1175.46995	-1175.13770	-1173.09111	-1172.758854
165-Me$^+$_3	-1175.46816	-1175.13559	-1173.09125	-1172.758678
165-Me$^+$_4	-1175.46576	-1175.13331	-1173.08911	-1172.756672
**166**	-741.229778	-741.029364	-739.817740	-739.617326
**166-Me$^+$**	-780.910438	-780.669165	-779.388643	-779.147370
**167**				-735.912197
167_1	-737.385753	-737.266187	-736.032404	-735.912839
167_2	-737.385701	-737.266006	-736.031987	-735.912292
167_3	-737.386366	-737.266807	-736.031714	-735.912155
167_4	-737.385580	-737.266022	-736.030933	-735.911375
167_5	-737.383229	-737.263635	-736.029830	-735.910236
167_6	-737.383926	-737.264367	-736.029761	-735.910202
167_7	-737.382842	-737.263243	-736.029667	-735.910068
167_8	-737.383939	-737.264397	-736.029389	-735.909848
**167-Me$^+$**				-775.444375
167-Me$^+$_1	-777.069066	-776.908413	-775.606065	-775.445412
167-Me$^+$_2	-777.067596	-776.907032	-775.604371	-775.443807
167-Me$^+$_3	-777.067360	-776.906736	-775.604316	-775.443693
167-Me$^+$_4	-777.067570	-776.906920	-775.603785	-775.443135
167-Me$^+$_5	-777.067355	-776.906761	-775.603603	-775.443009
167-Me$^+$_6	-777.067063	-776.906430	-775.603575	-775.442942
167-Me$^+$_7	-777.067109	-776.906501	-775.603387	-775.442779
167-Me$^+$_8	-777.066966	-776.906299	-775.603399	-775.442733
167-Me$^+$_9	-777.066866	-776.906263	-775.603015	-775.442412
167-Me$^+$_10	-777.066809	-776.906204	-775.602774	-775.442169
**168**				-908.843121
168_1	-910.85878	-910.62435	-909.07784	-908.843402
168_2	-910.85774	-910.62349	-909.07640	-908.842146
**168-Me$^+$**				-948.377524
168-Me$^+$_1	-950.54449	-950.26858	-948.65351	-948.377600
168-Me$^+$_2	-950.54177	-950.26604	-948.64970	-948.373967
**169**				-643.965118
169_1	-645.256894	-645.136804	-644.085871	-643.965781
169_2	-645.255151	-645.135066	-644.084356	-643.964272
169_3	-645.256132	-645.136031	-644.084300	-643.964199
169_4	-645.254456	-645.134337	-644.083468	-643.963348
169_5	-645.254865	-645.134960	-644.082547	-643.962641
169_6	-645.253627	-645.133649	-644.081497	-643.961519
169_7	-645.253193	-645.133301	-644.081164	-643.961272
**169-Me$^+$**				-683.499783
169-Me$^+$_1	-684.941269	-684.780040	-683.661417	-683.500189
169-Me$^+$_2	-684.941096	-684.779833	-683.661203	-683.499940
169-Me$^+$_3	-684.940989	-684.779683	-683.661005	-683.499698
169-Me$^+$_4	-684.940680	-684.779421	-683.660935	-683.499676
169-Me$^+$_5	-684.940266	-684.779000	-683.660474	-683.499208
169-Me$^+$_6	-684.939434	-684.778201	-683.659657	-683.498425

**170**				-684.454517
170_1	-685.92694	-685.74924	-684.63241	-684.454708
170_2	-685.92433	-685.74658	-684.62922	-684.451475
170_3	-685.92435	-685.74651	-684.62922	-684.451380
**170-Me$^+$**	-725.61549	-725.39630	-724.21231	-723.993120
**171**				-552.017157
171_1	-553.126411	-553.005814	-552.138095	-552.017498
171_2	-553.125988	-553.005509	-552.137080	-552.016601
171_3	-553.123857	-553.003341	-552.135213	-552.014697
**171-Me$^+$**	-592.815692	-592.653769	-591.720119	-591.558196
**172**	-685.92791	-685.75031	-684.63360	-684.455999
**172-Me$^+$**	-725.61791	-725.39898	-724.21711	-723.998172
**173**				-1133.231788
173_1	-1135.78667	-1135.49641	-1133.52213	-1133.231867
173_2	-1135.78454	-1135.49444	-1133.51840	-1133.228302
**173-Me$^+$**				-1172.775131
173-Me$^+$_1	-1175.47928	-1175.14741	-1173.10723	-1172.775359
173-Me$^+$_2	-1175.47878	-1175.14692	-1173.10696	-1172.775097
173-Me$^+$_3	-1175.47555	-1175.14347	-1173.10530	-1172.773222
**174**				-940.859402
174_1	-942.869663	-942.660697	-941.068597	-940.859631
174_2	-942.867203	-942.658258	-941.066410	-940.857465
174_3	-942.865563	-942.656687	-941.063895	-940.855019
**174-Me$^+$**				-980.403355
174-Me$^+$_1	-982.564711	-982.314422	-980.653900	-980.403611
174-Me$^+$_2	-982.562798	-982.312485	-980.652455	-980.402142
**175**				-700.464633
175_1	-701.933558	-701.768428	-700.629873	-700.464744
175_2	-701.933437	-701.768305	-700.629815	-700.464683
175_3	-701.930621	-701.765669	-700.627102	-700.462150
**175-Me$^+$**	-741.626834	-741.420320	-740.215335	-740.008821
**176**				-908.842240
176_1	-910.85715	-910.62311	-909.07652	-908.842482
176_2	-910.85727	-910.62319	-909.07630	-908.842218
176_3	-910.85513	-910.62145	-909.07430	-908.840615
**176-Me$^+$**				-948.388104
176-Me$^+$_1	-950.55100	-950.27543	-948.66390	-948.388327
176-Me$^+$_2	-950.54991	-950.27442	-948.66315	-948.387662
**177**				-1181.254931
177_1	-1183.806556	-1183.553586	-1181.508205	-1181.255234
177_2	-1183.802961	-1183.550221	-1181.505125	-1181.252385
177_3	-1183.802707	-1183.549856	-1181.505050	-1181.252200
177_4	-1183.802272	-1183.549489	-1181.504362	-1181.251578
**177-Me$^+$**	-1223.505632	-1223.211302	-1221.096066	-1220.801736
**178**				-668.408811
178_1	-669.880440	-669.691337	-668.597978	-668.408875
178_2	-669.877430	-669.688204	-668.594245	-668.405019
**178-Me$^+$**	-709.578106	-709.347102	-708.187398	-707.956394

**179**				-592.372915
179_1	-593.661460	-593.486307	-592.548410	-592.373257
179_2	-593.661188	-593.486335	-592.547505	-592.372652
179_3	-593.660088	-593.484972	-592.546410	-592.371294
**179-Me$^+$**	-633.359578	-633.142875	-632.139143	-631.922440
**180**				-709.896779
180_1	-711.588493	-711.321945	-710.164020	-709.897472
180_2	-711.587492	-711.320898	-710.163172	-709.896578
180_3	-711.588306	-711.321619	-710.162604	-709.895917
180_4	-711.588141	-711.321234	-710.162808	-709.895900
180_5	-711.587769	-711.321072	-710.162510	-709.895813
180_6	-711.585250	-711.318578	-710.160613	-709.893940
**180-Me$^+$**				-749.447922
180-Me$^+$_1	-751.287783	-750.979188	-749.757109	-749.448515
180-Me$^+$_2	-751.288404	-750.979681	-749.755614	-749.446891
180-Me$^+$_3	-751.288912	-750.980270	-749.755303	-749.446661
180-Me$^+$_4	-751.286710	-750.978236	-749.755062	-749.446588
180-Me$^+$_5	-751.285965	-750.977262	-749.752886	-749.444183
**181**				-631.540291
181 _1	-632.967340	-632.761890	-631.745996	-631.540546
181_2	-632.967284	-632.761682	-631.745959	-631.540357
181_3	-632.965821	-632.760499	-631.744247	-631.538925
181_4	-632.963458	-632.758023	-631.742173	-631.536737
**181-Me$^+$**	-672.667931	-672.420599	-671.338831	-671.091499
**182**				-724.676087
182_1	-726.325553	-726.096287	-724.905812	-724.676546
182_2	-726.326622	-726.097474	-724.905683	-724.676534
182_3	-726.325697	-726.096916	-724.903944	-724.675162
182_4	-726.324763	-726.095496	-724.903930	-724.674664
182_5	-726.324651	-726.095579	-724.902938	-724.673867
182_6	-726.323571	-726.094547	-724.902559	-724.673535
182_7	-726.324305	-726.095061	-724.902610	-724.673366
182_8	-726.323663	-726.094782	-724.902035	-724.673153
**182-Me$^+$**				-764.227722
182-Me$^+$_1	-766.028126	-765.757294	-764.498915	-764.228083
182-Me$^+$_2	-766.027590	-765.756836	-764.498695	-764.227941
182-Me$^+$_3	-766.027120	-765.756328	-764.497929	-764.227137
182-Me$^+$_4	-766.027222	-765.756379	-764.497918	-764.227075
182-Me$^+$_5	-766.025426	-765.754797	-764.495758	-764.225129
**183**				-876.751710
183_1	-878.765683	-878.508336	-877.009216	-876.751869
183_2	-878.765276	-878.508098	-877.008701	-876.751524
**183-Me$^+$**				-916.304076
183-Me$^+$_1	-918.468931	-918.169485	-916.603595	-916.304149
183-Me$^+$_2	-918.466123	-918.166874	-916.599706	-916.300457
**184**				-709.902674
184_1	-711.595079	-711.328443	-710.169652	-709.903016
184_2	-711.593638	-711.326946	-710.167957	-709.901264

184_3	-711.591910	-711.325111	-710.166799	-709.900000
**184-Me$^+$**	-751.296000	-750.987454	-749.763998	-749.455453
**185**				-1085.097189
185_1	-1087.651448	-1087.326397	-1085.422408	-1085.097357
185_2	-1087.650604	-1087.325348	-1085.422259	-1085.097003
185_3	-1087.647308	-1087.321959	-1085.418338	-1085.092989
**185-Me$^+$**				-1124.650349
185-Me$^+$_1	-1127.356064	-1126.988550	-1125.018320	-1124.650807
185-Me$^+$_2	-1127.355209	-1126.987951	-1125.015960	-1124.648702
185-Me$^+$_3	-1127.355292	-1126.987806	-1125.015989	-1124.648504
185-Me$^+$_4	-1127.352843	-1126.985125	-1125.015723	-1124.648006
**186**				-856.980018
186_1	-858.991018	-858.707683	-857.263774	-856.980438
186_2	-858.991347	-858.708315	-857.263468	-856.980436
186_3	-858.990710	-858.707392	-857.263704	-856.980386
186_4	-858.991302	-858.708205	-857.263378	-856.980280
186_5	-858.990057	-858.706953	-857.263001	-856.979897
186_6	-858.990087	-858.707017	-857.262778	-856.979708
186_7	-858.990064	-858.707060	-857.261297	-856.978293
186_8	-858.988214	-858.705064	-857.260960	-856.977810
186_9	-858.988818	-858.705760	-857.260750	-856.977693
186_10	-858.987638	-858.704268	-857.260697	-856.977327
**186-Me$^+$**				-896.533514
186-Me$^+$_1	-898.696192	-898.371266	-896.859367	-896.534441
186-Me$^+$_2	-898.695563	-898.370697	-896.859302	-896.534436
186-Me$^+$_3	-898.695792	-898.370769	-896.858500	-896.533478
186-Me$^+$_4	-898.695716	-898.370741	-896.858256	-896.533281
186-Me$^+$_5	-898.694977	-898.370069	-896.858085	-896.533177
186-Me$^+$_6	-898.694607	-898.369718	-896.857792	-896.532903
186-Me$^+$_7	-898.694733	-898.369919	-896.857674	-896.532860
186-Me$^+$_8	-898.694307	-898.369285	-896.857855	-896.532833
186-Me$^+$_9	-898.695080	-898.370232	-896.857545	-896.532697
186-Me$^+$_10	-898.693978	-898.369068	-896.857447	-896.532537
**187**				-631.538156
187_1	-632.966076	-632.760606	-631.743930	-631.538460
187_2	-632.965156	-632.759650	-631.743841	-631.538335
187_3	-632.964696	-632.759517	-631.742833	-631.537654
187_4	-632.964815	-632.759288	-631.742585	-631.537059
**187-Me$^+$**	-672.666030	-672.418865	-671.339332	-671.092167
**188**	-711.589794	-711.322907	-710.164583	-709.897696
188_1	-711.593061	-711.326256	-710.167407	-709.900601
188_2	-711.593289	-711.326475	-710.167551	-709.900737
188_3	-711.592069	-711.325422	-710.167196	-709.900549
**188-Me$^+$**				-749.456692
188-Me$^+$_1	-751.297006	-750.988208	-749.765647	-749.456849
188-Me$^+$_2	-751.294605	-750.986211	-749.762653	-749.454259
**189**				-670.732242
189_1	-672.291343	-672.055041	-670.969304	-670.733003

189_2	-672.290511	-672.054284	-670.968240	-670.732013
189_3	-672.289894	-672.053462	-670.967894	-670.731462
189_4	-672.289813	-672.053336	-670.967841	-670.731364
189_5	-672.289580	-672.053126	-670.967297	-670.730843
189_6	-672.289009	-672.052498	-670.966851	-670.730339
189_7	-672.287894	-672.051916	-670.965310	-670.729332
189_8	-672.286926	-672.050383	-670.965192	-670.728649
**189-Me$^+$**				-710.287548
189-Me$^+$_1	-711.993828	-711.715575	-710.565850	-710.287597
189-Me$^+$_2	-711.989968	-711.711827	-710.561551	-710.283410
**190**				-670.731418
190_1	-672.290368	-672.054036	-670.968454	-670.732122
190_2	-672.288744	-672.052317	-670.967540	-670.731113
190_3	-672.289244	-672.053027	-670.967292	-670.731076
190_4	-672.287797	-672.051446	-670.966940	-670.730589
190_5	-672.287159	-672.050813	-670.966083	-670.729737
190_6	-672.287333	-672.050983	-670.965833	-670.729484
190_7	-672.286259	-672.049823	-670.964270	-670.727834
**190-Me$^+$**				-710.286768
190-Me$^+$_1	-711.994134	-711.715938	-710.565225	-710.287029
190-Me$^+$_2	-711.992515	-711.714273	-710.563964	-710.285722
**191**				-803.010674
191_1	-804.938051	-804.648066	-803.301261	-803.011276
191_2	-804.937818	-804.647791	-803.301143	-803.011116
191_3	-804.936971	-804.647060	-803.300621	-803.010709
191_4	-804.937400	-804.647210	-803.300500	-803.010309
191_5	-804.936421	-804.646441	-803.299753	-803.009773
191_6	-804.936609	-804.646679	-803.299697	-803.009766
191_7	-804.936042	-804.646020	-803.299482	-803.009460
191_8	-804.934138	-804.643879	-803.298524	-803.008266
191_9	-804.934506	-804.644452	-803.298021	-803.007968
191_10	-804.934624	-804.644898	-803.297471	-803.007745
**191-Me$^+$**				-842.566086
191-Me$^+$_1	-844.644129	-844.312308	-842.898404	-842.566584
191-Me$^+$_2	-844.643517	-844.311420	-842.898167	-842.566070
191-Me$^+$_3	-844.643908	-844.311719	-842.898160	-842.565971
191-Me$^+$_4	-844.644083	-844.312111	-842.897899	-842.565927
191-Me$^+$_5	-844.642991	-844.311135	-842.897331	-842.565475
191-Me$^+$_6	-844.642567	-844.310513	-842.896369	-842.564315
**192**				-959.724336
192_1	-962.180082	-961.767392	-960.137814	-959.725124
192_2	-962.178472	-961.765894	-960.137612	-959.725033
192_3	-962.178092	-961.765978	-960.137005	-959.724891
192_4	-962.179024	-961.766467	-960.137255	-959.724698
192_5	-962.179277	-961.766895	-960.136947	-959.724565
192_6	-962.178541	-961.766144	-960.136817	-959.724420
192_7	-962.179513	-961.766904	-960.136772	-959.724163
192_8	-962.178763	-961.766217	-960.136307	-959.723762

192_9	-962.176918	-961.764663	-960.135719	-959.723464
192_10	-962.179285	-961.766514	-960.136232	-959.723461
**192-Me$^+$**				-999.280138
192-Me$^+$_1	-1001.884557	-1001.430174	-999.735721	-999.281338
192-Me$^+$_2	-1001.884009	-1001.429311	-999.734310	-999.279612
192-Me$^+$_3	-1001.884869	-1001.429907	-999.734546	-999.279584
192-Me$^+$_4	-1001.882955	-1001.428328	-999.733641	-999.279014
192-Me$^+$_5	-1001.884228	-1001.429431	-999.733799	-999.279002
192-Me$^+$_6	-1001.883195	-1001.428313	-999.733458	-999.278576
192-Me$^+$_7	-1001.883957	-1001.429341	-999.732618	-999.278002
192-Me$^+$_8	-1001.880793	-1001.426070	-999.732310	-999.277587
192-Me$^+$_9	-1001.883127	-1001.428516	-999.732188	-999.277577
192-Me$^+$_10	-1001.879805	-1001.425466	-999.731796	-999.277457
**193**				-749.052315
193_1	-750.879812	-750.583048	-749.349522	-749.052758
193_2	-750.879495	-750.582419	-749.349803	-749.052727
193_3	-750.879643	-750.582760	-749.349520	-749.052636
193_4	-750.879380	-750.582590	-749.349336	-749.052546
193_5	-750.879232	-750.582538	-749.349161	-749.052467
193_6	-750.878613	-750.581633	-749.348839	-749.051859
193_7	-750.878819	-750.582285	-749.348138	-749.051604
193_8	-750.878810	-750.582088	-749.347976	-749.051254
193_9	-750.878383	-750.581583	-749.347861	-749.051061
193_10	-750.878718	-750.581776	-749.347971	-749.051029
**193-Me$^+$**				-788.608365
193-Me$^+$_1	-790.583774	-790.245264	-788.947320	-788.608809
193-Me$^+$_2	-790.583835	-790.244935	-788.947689	-788.608789
193-Me$^+$_3	-790.584015	-790.245346	-788.947218	-788.608550
193-Me$^+$_4	-790.583272	-790.244328	-788.947198	-788.608253
193-Me$^+$_5	-790.583676	-790.244834	-788.947088	-788.608246
193-Me$^+$_6	-790.582154	-790.242673	-788.947557	-788.608076
193-Me$^+$_7	-790.583094	-790.243904	-788.946509	-788.607319
193-Me$^+$_8	-790.581708	-790.242792	-788.945238	-788.606323
193-Me$^+$_9	-790.581425	-790.242797	-788.944802	-788.606174
193-Me$^+$_10	-790.581442	-790.242931	-788.944673	-788.606162
**194**				-684.455319
194_1	-685.92811	-685.75061	-684.63294	-684.455430
194_2	-685.92488	-685.74757	-684.62963	-684.452317
**194-Me$^+$**	-725.63371	-725.41447	-724.23095	-724.011698
**195**				-974.482082
195_1	-976.907666	-976.533453	-974.857365	-974.483153
195_2	-976.907003	-976.532611	-974.856787	-974.482395
195_3	-976.906751	-976.532348	-974.856684	-974.482282
195_4	-976.907423	-976.533121	-974.856579	-974.482277
195_5	-976.906418	-976.532120	-974.855955	-974.481656
195_6	-976.906895	-976.532483	-974.855952	-974.481539
195_7	-976.906077	-976.531622	-974.855495	-974.481041
195_8	-976.905157	-976.530528	-974.855311	-974.480682

195_9	-976.905390	-976.530844	-974.855197	-974.480652
195_10	-976.906537	-976.531897	-974.855061	-974.480421
**195-Me$^+$**				-1014.039719
195-Me$^+$_1	-1016.617535	-1016.200870	-1014.457294	-1014.040628
195-Me$^+$_2	-1016.617830	-1016.201188	-1014.457168	-1014.040525
195-Me$^+$_3	-1016.617636	-1016.201007	-1014.456969	-1014.040340
195-Me$^+$_4	-1016.617667	-1016.200958	-1014.456949	-1014.040240
195-Me$^+$_5	-1016.618144	-1016.201272	-1014.457024	-1014.040152
195-Me$^+$_6	-1016.617087	-1016.200207	-1014.456561	-1014.039680
195-Me$^+$_7	-1016.617162	-1016.200392	-1014.456273	-1014.039502
195-Me$^+$_8	-1016.617823	-1016.201140	-1014.456106	-1014.039423
195-Me$^+$_9	-1016.616033	-1016.199244	-1014.455852	-1014.039063
195-Me$^+$_10	-1016.616999	-1016.200308	-1014.455648	-1014.038957
**196**				-749.051043
196_1	-750.879293	-750.582452	-749.348638	-749.051798
196_2	-750.878973	-750.582405	-749.347896	-749.051328
196_3	-750.877977	-750.581198	-749.347450	-749.050671
196_4	-750.876591	-750.580159	-749.346987	-749.050555
196_5	-750.877028	-750.580378	-749.345849	-749.049199
196_6	-750.876870	-750.579962	-749.346035	-749.049127
196_7	-750.876740	-750.580011	-749.345472	-749.048744
196_8	-750.876034	-750.579234	-749.345298	-749.048497
196_9	-750.875948	-750.579221	-749.345031	-749.048304
196_10	-750.875325	-750.578438	-749.345150	-749.048263
**196-Me$^+$**				-788.609058
196-Me$^+$_1	-790.586069	-790.247264	-788.947932	-788.609128
196-Me$^+$_2	-790.581738	-790.242825	-788.944299	-788.605387
**197**				-959.735337
197_1	-962.192106	-961.779794	-960.148195	-959.735883
197_2	-962.190692	-961.778298	-960.146792	-959.734399
197_3	-962.190495	-961.777933	-960.145988	-959.733426
197_4	-962.188737	-961.776084	-960.145021	-959.732369
197_5	-962.187691	-961.775291	-960.143896	-959.731497
197_6	-962.188580	-961.776092	-960.143744	-959.731256
197_7	-962.187110	-961.774662	-960.143666	-959.731218
**197-Me$^+$**				-999.293997
197-Me$^+$_1	-1001.900057	-1001.445361	-999.749135	-999.294439
197-Me$^+$_2	-1001.898165	-1001.443654	-999.747338	-999.292827
197-Me$^+$_3	-1001.897470	-1001.442883	-999.746329	-999.291742
197-Me$^+$_4	-1001.896581	-1001.442022	-999.745906	-999.291347
**198**				-803.007405
198_1	-804.935004	-804.644993	-803.298523	-803.008511
198_2	-804.934887	-804.644903	-803.297546	-803.007562
198_3	-804.935723	-804.645789	-803.297421	-803.007487
198_4	-804.933658	-804.643683	-803.297337	-803.007362
198_5	-804.933397	-804.643508	-803.297110	-803.007221
198_6	-804.933219	-804.643242	-803.296769	-803.006792
198_7	-804.934333	-804.644415	-803.296655	-803.006737

198_8	-804.934426	-804.644627	-803.296490	-803.006691
198_9	-804.933271	-804.643182	-803.296631	-803.006542
198_10	-804.933500	-804.643621	-803.296389	-803.006510
**198-Me$^+$**				-842.566450
198-Me$^+$_1	-844.640676	-844.308478	-842.898883	-842.566686
198-Me$^+$_2	-844.640077	-844.307960	-842.898694	-842.566577
198-Me$^+$_3	-844.639542	-844.307497	-842.897370	-842.565326
198-Me$^+$_4	-844.636740	-844.304886	-842.894321	-842.562467
**199**				-959.734395
199_1	-962.191327	-961.778804	-960.147798	-959.735275
199_2	-962.191335	-961.778940	-960.147498	-959.735104
199_3	-962.190259	-961.777837	-960.146913	-959.734491
199_4	-962.189749	-961.777204	-960.146153	-959.733608
199_5	-962.188372	-961.776180	-960.145734	-959.733543
199_6	-962.189252	-961.776600	-960.146165	-959.733513
199_7	-962.189981	-961.777349	-960.146080	-959.733448
199_8	-962.189583	-961.777176	-960.145686	-959.733279
199_9	-962.188869	-961.776638	-960.145157	-959.732926
199_10	-962.187837	-961.775327	-960.145086	-959.732576
**199-Me$^+$**				-999.293604
199-Me$^+$_1	-1001.899257	-1001.444792	-999.749058	-999.294592
199-Me$^+$_2	-1001.898584	-1001.443850	-999.748584	-999.293850
199-Me$^+$_3	-1001.897214	-1001.442243	-999.748128	-999.293157
199-Me$^+$_4	-1001.896471	-1001.441477	-999.748149	-999.293154
199-Me$^+$_5	-1001.897533	-1001.442810	-999.747252	-999.292529
199-Me$^+$_6	-1001.897338	-1001.443003	-999.746859	-999.292523
199-Me$^+$_7	-1001.897065	-1001.442499	-999.747019	-999.292452
199-Me$^+$_8	-1001.896899	-1001.442204	-999.747004	-999.292309
199-Me$^+$_9	-1001.896865	-1001.442087	-999.746383	-999.291605
199-Me$^+$_10	-1001.894886	-1001.440044	-999.746250	-999.291408
**200**				-749.052157
200_1	-750.879982	-750.583317	-749.350072	-749.053407
200_2	-750.879301	-750.582597	-749.349512	-749.052808
200_3	-750.878334	-750.581576	-749.349417	-749.052659
200_4	-750.879826	-750.583067	-749.349387	-749.052628
200_5	-750.878775	-750.581792	-749.349108	-749.052125
200_6	-750.879173	-750.582500	-749.348795	-749.052123
200_7	-750.878605	-750.581855	-749.348436	-749.051686
200_8	-750.878223	-750.581332	-749.348517	-749.051626
200_9	-750.877858	-750.581201	-749.348106	-749.051449
200_10	-750.877525	-750.580537	-749.348435	-749.051447
**200-Me$^+$**				-788.611597
200-Me$^+$_1	-790.586376	-790.247352	-788.950926	-788.611902
200-Me$^+$_2	-790.585510	-790.246507	-788.949583	-788.610580
**201**				-668.408329
201_1	-669.881255	-669.692197	-668.597519	-668.408461
201_2	-669.881260	-669.692198	-668.597520	-668.408459
201_3	-669.879439	-669.690407	-668.595814	-668.406782

**201-Me$^+$**	-709.590526	-709.359581	-708.199299	-707.968354
**202**				-881.394987
202_1	-883.585737	-883.233794	-881.748077	-881.396134
202_2	-883.584856	-883.232995	-881.747555	-881.395695
202_3	-883.584775	-883.232812	-881.747395	-881.395432
202_4	-883.584749	-883.232919	-881.747202	-881.395373
202_5	-883.584262	-883.232244	-881.747241	-881.395223
202_6	-883.584453	-883.232674	-881.746752	-881.394973
202_7	-883.584345	-883.232688	-881.746307	-881.394650
202_8	-883.583990	-883.232197	-881.746407	-881.394615
202_9	-883.583867	-883.232180	-881.746201	-881.394514
202_10	-883.584503	-883.232792	-881.746184	-881.394473
**202-Me$^+$**				-920.957361
202-Me$^+$_1	-923.295338	-922.901101	-921.351977	-920.957740
202-Me$^+$_2	-923.293756	-922.899514	-921.351956	-920.957714
202-Me$^+$_3	-923.294542	-922.900162	-921.352017	-920.957637
202-Me$^+$_4	-923.293739	-922.899360	-921.350976	-920.956598
202-Me$^+$_5	-923.293230	-922.898802	-921.350702	-920.956274
202-Me$^+$_6	-923.291320	-922.897165	-921.348612	-920.954457
202-Me$^+$_7	-923.291925	-922.898058	-921.348084	-920.954217
202-Me$^+$_8	-923.291691	-922.897882	-921.347900	-920.954091
202-Me$^+$_9	-923.291809	-922.897769	-921.348092	-920.954051
202-Me$^+$_10	-923.290878	-922.897048	-921.347792	-920.953963
**203**				-785.909843
203_1	-787.785022	-787.507152	-786.187910	-785.910040
203_2	-787.782395	-787.504689	-786.185575	-785.907869
**203-Me$^+$**				-825.472540
203-Me$^+$_1	-827.496105	-827.175913	-825.792808	-825.472616
203-Me$^+$_2	-827.495857	-827.175601	-825.792705	-825.472450
**204**				-881.393358
204_1	-883.582740	-883.231251	-881.745865	-881.394376
204_2	-883.582953	-883.231405	-881.745847	-881.394299
204_3	-883.579573	-883.228224	-881.745131	-881.393782
204_4	-883.581558	-883.229739	-881.745457	-881.393638
204_5	-883.581200	-883.229493	-881.745067	-881.393360
204_6	-883.580807	-883.229185	-881.744592	-881.392970
204_7	-883.580459	-883.228226	-881.745133	-881.392900
204_8	-883.581044	-883.229373	-881.744363	-881.392692
204_9	-883.581821	-883.230191	-881.744193	-881.392563
204_10	-883.580933	-883.229101	-881.744325	-881.392493
**204-Me$^+$**				-920.956260
204-Me$^+$_1	-923.295600	-922.901535	-921.350950	-920.956885
204-Me$^+$_2	-923.295117	-922.901271	-921.350573	-920.956727
204-Me$^+$_3	-923.293920	-922.900094	-921.349548	-920.955722
204-Me$^+$_4	-923.293617	-922.899868	-921.348953	-920.955203
204-Me$^+$_5	-923.292434	-922.898761	-921.348260	-920.954587
204-Me$^+$_6	-923.293005	-922.899190	-921.348296	-920.954481
204-Me$^+$_7	-923.292195	-922.898465	-921.347980	-920.954250

204-Me$^+$_8	-923.292370	-922.898494	-921.347528	-920.953652
204-Me$^+$_9	-923.293210	-922.899364	-921.347429	-920.953583
204-Me$^+$_10	-923.290847	-922.897209	-921.346552	-920.952914
**205**				-746.746366
205_1	-748.487292	-748.239016	-746.994686	-746.746410
205_2	-748.482831	-748.234650	-746.990281	-746.742100
**205-Me$^+$**	-788.199005	-787.908847	-786.599520	-786.309363
**206**				-974.477072
206_1	-976.904573	-976.530085	-974.852783	-974.478295
206_2	-976.903266	-976.528735	-974.852156	-974.477625
206_3	-976.905282	-976.530861	-974.851655	-974.477234
206_4	-976.902786	-976.528139	-974.851725	-974.477078
206_5	-976.902979	-976.528433	-974.851530	-974.476984
206_6	-976.902240	-976.527708	-974.851510	-974.476977
206_7	-976.902887	-976.528407	-974.851422	-974.476942
206_8	-976.903084	-976.528612	-974.851389	-974.476917
206_9	-976.902297	-976.527635	-974.851477	-974.476815
206_10	-976.903869	-976.529386	-974.850972	-974.476489
**206-Me$^+$**				-1014.041572
206-Me$^+$_1	-1016.612525	-1016.195523	-1014.459026	-1014.042024
206-Me$^+$_2	-1016.613321	-1016.196368	-1014.458827	-1014.041875
206-Me$^+$_3	-1016.613656	-1016.196661	-1014.456969	-1014.039974
206-Me$^+$_4	-1016.613739	-1016.196690	-1014.456924	-1014.039875
206-Me$^+$_5	-1016.611693	-1016.194660	-1014.456842	-1014.039809
206-Me$^+$_6	-1016.613112	-1016.196032	-1014.455940	-1014.038859
206-Me$^+$_7	-1016.610188	-1016.193715	-1014.454566	-1014.038093
**207**	-910.85680	-910.62306	-909.07253	-908.838795
**207-Me$^+$**				-948.405634
207-Me$^+$_1	-950.57519	-950.29944	-948.68182	-948.406069
207-Me$^+$_2	-950.57341	-950.29784	-948.68030	-948.404731
207-Me$^+$_3	-950.57318	-950.29742	-948.68028	-948.404525
**208**				-1133.227698
208_1	-1135.78888	-1135.49839	-1133.51873	-1133.228238
208_2	-1135.78711	-1135.49677	-1133.51791	-1133.227570
208_3	-1135.78853	-1135.49811	-1133.51727	-1133.226846
208_4	-1135.78718	-1135.49674	-1133.51603	-1133.225592
208_5	-1135.78539	-1135.49525	-1133.51553	-1133.225393
208_6	-1135.78661	-1135.49615	-1133.51448	-1133.224010
**208-Me$^+$**	-1175.51654	-1175.18430	-1173.13298	-1172.800734
**209**				-876.746362
209_1	-878.764077	-878.507098	-877.003763	-876.746785
209_2	-878.764060	-878.507079	-877.003705	-876.746724
209_3	-878.764436	-878.507421	-877.003417	-876.746402
209_4	-878.763381	-878.506410	-877.003091	-876.746120
209_5	-878.763365	-878.506454	-877.003030	-876.746119
209_6	-878.763346	-878.506380	-877.003023	-876.746057
209_7	-878.763322	-878.506335	-877.002853	-876.745867
209_8	-878.762660	-878.505646	-877.002567	-876.745552

**209-Me$^+$**				-916.319814
209-Me$^+$_1	-918.488591	-918.189391	-916.619263	-916.320063
209-Me$^+$_2	-918.488016	-918.188849	-916.618800	-916.319633
209-Me$^+$_3	-918.487880	-918.188663	-916.618811	-916.319594
**210**				-1111.752598
210_1	-1114.570072	-1114.135497	-1112.187889	-1111.753314
210_2	-1114.569510	-1114.134860	-1112.186856	-1111.752206
210_3	-1114.568590	-1114.133561	-1112.187139	-1111.752110
210_4	-1114.567687	-1114.133051	-1112.185994	-1111.751358
210_5	-1114.567793	-1114.132977	-1112.186124	-1111.751307
210_6	-1114.567735	-1114.133191	-1112.185725	-1111.751181
**210-Me$^+$**				-1151.331870
210-Me$^+$_1	-1154.298597	-1153.821302	-1151.809905	-1151.332610
210-Me$^+$_2	-1154.297814	-1153.820552	-1151.809070	-1151.331808
210-Me$^+$_3	-1154.297842	-1153.820523	-1151.809094	-1151.331775
210-Me$^+$_4	-1154.297758	-1153.820419	-1151.808970	-1151.331631
210-Me$^+$_5	-1154.296043	-1153.818609	-1151.808239	-1151.330805
210-Me$^+$_6	-1154.296078	-1153.818649	-1151.808213	-1151.330784
210-Me$^+$_7	-1154.296158	-1153.818741	-1151.807867	-1151.330450
210-Me$^+$_8	-1154.295882	-1153.818403	-1151.807707	-1151.330227
**211**				-1033.422644
211_1	-1035.976134	-1035.600796	-1033.798304	-1033.422967
211_2	-1035.973012	-1035.597738	-1033.797335	-1033.422061
211_3	-1035.971284	-1035.596027	-1033.795065	-1033.419808
**211-Me$^+$**				-1073.004001
211-Me$^+$_1	-1075.704953	-1075.287461	-1073.421648	-1073.004156
211-Me$^+$_2	-1075.702831	-1075.285417	-1073.418965	-1073.001551
**212**				-1085.087188
212_1	-1087.647052	-1087.322287	-1085.412149	-1085.087384
212_2	-1087.647505	-1087.322671	-1085.412207	-1085.087373
212_3	-1087.647593	-1087.322748	-1085.411991	-1085.087146
212_4	-1087.647572	-1087.322731	-1085.411908	-1085.087067
212_5	-1087.647491	-1087.322629	-1085.411436	-1085.086575
**212-Me$^+$**				-1124.671849
212-Me$^+$_1	-1127.385275	-1127.017893	-1125.039816	-1124.672434
212-Me$^+$_2	-1127.383817	-1127.016468	-1125.038629	-1124.671280
212-Me$^+$_3	-1127.382373	-1127.014992	-1125.037330	-1124.669948
212-Me$^+$_4	-1127.382618	-1127.015290	-1125.037198	-1124.669871
212-Me$^+$_5	-1127.381742	-1127.014436	-1125.036509	-1124.669202
212-Me$^+$_6	-1127.381453	-1127.014130	-1125.036116	-1124.668793
**213**				-1320.102859
213_1	-1323.465258	-1322.963024	-1320.605343	-1320.103109
213_2	-1323.461900	-1322.959695	-1320.603733	-1320.101528
**213-Me$^+$**				-1359.694886
213-Me$^+$_1	-1363.204865	-1362.659956	-1360.240553	-1359.695644
213-Me$^+$_2	-1363.203818	-1362.658968	-1360.239811	-1359.694961
213-Me$^+$_3	-1363.203967	-1362.658971	-1360.239374	-1359.694378
213-Me$^+$_4	-1363.203694	-1362.659039	-1360.238922	-1359.694266

213-Me$^+$_5	-1363.203800	-1362.658784	-1360.239095	-1359.694079
213-Me$^+$_6	-1363.202481	-1362.657432	-1360.238800	-1359.693751
213-Me$^+$_7	-1363.201957	-1362.657067	-1360.237819	-1359.692929
213-Me$^+$_8	-1363.202013	-1362.657008	-1360.237798	-1359.692793
**214**				-1437.601629
214_1	-1441.357421	-1440.766052	-1438.193734	-1437.602365
214_2	-1441.356059	-1440.765059	-1438.193031	-1437.602030
214_3	-1441.356719	-1440.765466	-1438.192635	-1437.601382
214_4	-1441.356944	-1440.765532	-1438.192687	-1437.601274
214_5	-1441.355857	-1440.764380	-1438.192676	-1437.601199
214_6	-1441.355800	-1440.765090	-1438.190917	-1437.600207
214_7	-1441.355777	-1440.764930	-1438.191011	-1437.600163
214_8	-1441.355832	-1440.764892	-1438.191045	-1437.600106
214_9	-1441.355873	-1440.764911	-1438.191046	-1437.600084
214_10	-1441.355735	-1440.764748	-1438.190932	-1437.599945
**214-Me$^+$**				-1477.195392
214-Me$^+$_1	-1481.098421	-1480.463879	-1477.830480	-1477.195938
214-Me$^+$_2	-1481.097181	-1480.462896	-1477.829466	-1477.195181
214-Me$^+$_3	-1481.095610	-1480.461470	-1477.828203	-1477.194063
214-Me$^+$_4	-1481.095523	-1480.461286	-1477.828180	-1477.193943
214-Me$^+$_5	-1481.093450	-1480.459122	-1477.827146	-1477.192818
**215**				-687.752649
215_1	-689.238515	-689.082586	-687.909043	-687.753114
215_2	-689.238584	-689.082655	-687.909039	-687.753110
215_3	-689.236910	-689.081005	-687.908186	-687.752281
215_4	-689.236799	-689.080889	-687.908176	-687.752266
215_5	-689.236920	-689.081010	-687.908126	-687.752216
215_6	-689.236580	-689.080661	-687.908058	-687.752139
215_7	-689.235052	-689.079162	-687.906931	-687.751042
215_8	-689.233410	-689.077525	-687.905746	-687.749861
**215-Me$^+$**				-727.282653
215-Me$^+$_1	-728.917726	-728.719924	-727.480599	-727.282796
215-Me$^+$_2	-728.917555	-728.719749	-727.480588	-727.282782
215-Me$^+$_3	-728.917460	-728.719629	-727.480589	-727.282758
215-Me$^+$_4	-728.917851	-728.720022	-727.480475	-727.282647
215-Me$^+$_5	-728.917514	-728.719713	-727.480244	-727.282443
215-Me$^+$_6	-728.917382	-728.719576	-727.480064	-727.282258
**216**				-611.860184
216_1	-613.159648	-613.015279	-612.004864	-611.860495
216_2	-613.159712	-613.015367	-612.004786	-611.860441
216_3	-613.159662	-613.015277	-612.004697	-611.860312
216_4	-613.158192	-613.013852	-612.003753	-611.859413
216_5	-613.157913	-613.013574	-612.003706	-611.859367
216_6	-613.156348	-613.012035	-612.002416	-611.858103
**216-Me$^+$**				-651.394392
216-Me$^+$_1	-652.842887	-652.656795	-651.580786	-651.394694
216-Me$^+$_2	-652.842830	-652.656746	-651.580416	-651.394332
216-Me$^+$_3	-652.842766	-652.656592	-651.580471	-651.394297

216-Me$^+$_4	-652.842599	-652.656460	-651.580102	-651.393962
**217**				-987.744789
217_1	-989.430968	-989.298817	-987.877313	-987.745162
217_2	-989.428049	-989.295924	-987.875193	-987.743067
217_3	-989.428418	-989.296282	-987.875201	-987.743065
**217-Me$^+$**	-1029.114327	-1028.940787	-1027.453166	-1027.279626
**218**				-1027.136829
218_1	-1029.308988	-1029.112590	-1027.333804	-1027.137406
218_2	-1029.310617	-1029.113051	-1027.334252	-1027.136685
218_3	-1029.310743	-1029.113213	-1027.334195	-1027.136664
218_4	-1029.310789	-1029.113213	-1027.334187	-1027.136612
218_5	-1029.310109	-1029.112588	-1027.333803	-1027.136282
218_6	-1029.309485	-1029.111935	-1027.333743	-1027.136193
**218-Me$^+$**				-1066.673383
218-Me$^+$_1	-1069.000397	-1068.761220	-1066.912830	-1066.673653
218-Me$^+$_2	-1069.000440	-1068.761179	-1066.912807	-1066.673546
218-Me$^+$_3	-1068.999364	-1068.760119	-1066.911571	-1066.672326
218-Me$^+$_4	-1068.997325	-1068.758049	-1066.909672	-1066.670397
218-Me$^+$_5	-1068.997323	-1068.758163	-1066.908999	-1066.669840
**219**				-838.115863
219_1	-839.875064	-839.702901	-838.288519	-838.116357
219_2	-839.873713	-839.701594	-838.286842	-838.114724
219_3	-839.873358	-839.701185	-838.286841	-838.114668
219_4	-839.871718	-839.699615	-838.285270	-838.113166
**219-Me$^+$**				-877.653021
219-Me$^+$_1	-879.563802	-879.350164	-877.867056	-877.653418
219-Me$^+$_2	-879.563514	-879.349790	-877.866589	-877.652865
219-Me$^+$_3	-879.563269	-879.349490	-877.866616	-877.652837
219-Me$^+$_4	-879.562134	-879.348337	-877.865438	-877.651642
**220**				-535.965713
220_1	-537.078986	-536.946253	-536.098709	-535.965976
220_2	-537.077356	-536.944629	-536.097494	-535.964767
**220-Me$^+$**	-576.767157	-576.592755	-575.680167	-575.505765
**221**				-987.745366
221_1	-989.430122	-989.298144	-987.877547	-987.745569
221_2	-989.430127	-989.298133	-987.877544	-987.745550
221_3	-989.430119	-989.298137	-987.877515	-987.745534
221_4	-989.429522	-989.297725	-987.876285	-987.744488
221_5	-989.429709	-989.297843	-987.875958	-987.744092
**221-Me$^+$**				-1027.285636
221-Me$^+$_1	-1029.117149	-1028.943909	-1027.458895	-1027.285655
221-Me$^+$_2	-1029.117210	-1028.943886	-1027.458940	-1027.285616
**222**				-649.093671
222_1	-650.436833	-650.290137	-649.240462	-649.093766
222_2	-650.436841	-650.290131	-649.240463	-649.093753
222_3	-650.434249	-650.287669	-649.237899	-649.091318
**222-Me$^+$**	-690.127367	-689.939179	-688.822813	-688.634625
**223**				-1483.338908

223_1	-1485.781710	-1485.615253	-1483.505921	-1483.339465
223_2	-1485.780358	-1485.613907	-1483.505368	-1483.338917
223_3	-1485.779965	-1485.613615	-1483.504460	-1483.338110
223_4	-1485.779695	-1485.613273	-1483.504410	-1483.337989
223_5	-1485.778398	-1485.612068	-1483.503905	-1483.337575
**223-Me$^+$**				-1522.882499
223-Me$^+$_1	-1525.473249	-1525.265216	-1523.090591	-1522.882559
223-Me$^+$_2	-1525.473162	-1525.265185	-1523.090468	-1522.882491
223-Me$^+$_3	-1525.473497	-1525.265531	-1523.090405	-1522.882439
**224**				-838.113383
224_1	-839.871536	-839.699104	-838.286041	-838.113609
224_2	-839.871663	-839.699227	-838.285873	-838.113437
224_3	-839.871851	-839.699377	-838.285855	-838.113381
224_4	-839.870446	-839.698087	-838.284625	-838.112266
**224-Me$^+$**				-877.657030
224-Me$^+$_1	-879.566451	-879.352404	-877.871481	-877.657434
224-Me$^+$_2	-879.565719	-879.351652	-877.871018	-877.656950
224-Me$^+$_3	-879.565327	-879.351315	-877.869985	-877.655973
224-Me$^+$_4	-879.564015	-879.349908	-877.868840	-877.654733
**225**				-971.704181
225_1	-973.390072	-973.246295	-971.848395	-971.704618
225_2	-973.387554	-973.243843	-971.846880	-971.703168
225_3	-973.387579	-973.243862	-971.846885	-971.703168
**225-Me$^+$**	-1013.081848	-1012.896498	-1011.433459	-1011.248109
**226**				-1994.975710
226_1	-1998.171442	-1997.982520	-1995.165176	-1994.976254
226_2	-1998.171449	-1997.982520	-1995.165173	-1994.976244
226_3	-1998.171198	-1997.982254	-1995.165025	-1994.976081
226_4	-1998.170687	-1997.981705	-1995.164578	-1994.975596
226_5	-1998.169923	-1997.980975	-1995.163968	-1994.975020
226_6	-1998.169885	-1997.980896	-1995.163988	-1994.974999
226_7	-1998.170305	-1997.981395	-1995.163901	-1994.974991
226_8	-1998.169897	-1997.980927	-1995.163957	-1994.974987
226_9	-1998.169752	-1997.980824	-1995.163885	-1994.974958
226_10	-1998.169842	-1997.980905	-1995.163894	-1994.974957
**226-Me$^+$**				-2034.519718
226-Me$^+$_1	-2037.863940	-2037.633344	-2034.750576	-2034.519980
226-Me$^+$_2	-2037.863020	-2037.632453	-2034.750479	-2034.519911
226-Me$^+$_3	-2037.864938	-2037.634270	-2034.750292	-2034.519624
226-Me$^+$_4	-2037.865770	-2037.635086	-2034.750293	-2034.519609
226-Me$^+$_5	-2037.865786	-2037.635096	-2034.750297	-2034.519607
226-Me$^+$_6	-2037.863853	-2037.633187	-2034.748849	-2034.518184
**227**				-1027.136629
227_1	-1029.309518	-1029.111434	-1027.335193	-1027.137108
227_2	-1029.309265	-1029.111253	-1027.334924	-1027.136913
227_3	-1029.308620	-1029.110635	-1027.334318	-1027.136333
227_4	-1029.308596	-1029.110600	-1027.334316	-1027.136320
227_5	-1029.308581	-1029.110593	-1027.334298	-1027.136310

227_6	-1029.308826	-1029.110780	-1027.334039	-1027.135994
227_7	-1029.307417	-1029.109407	-1027.332260	-1027.134250
**227-Me^{+}**				-1066.680834
227-Me^{+}_1	-1069.005879	-1068.766091	-1066.920950	-1066.681163
227-Me^{+}_2	-1069.005740	-1068.766004	-1066.920838	-1066.681102
227-Me^{+}_3	-1069.004635	-1068.764852	-1066.919881	-1066.680098
227-Me^{+}_4	-1069.003101	-1068.763293	-1066.918365	-1066.678557
227-Me^{+}_5	-1069.002661	-1068.762914	-1066.917769	-1066.678023
227-Me^{+}_6	-1069.002898	-1068.763162	-1066.917693	-1066.677957
**228**				-649.091734
228_1	-650.434660	-650.287908	-649.238613	-649.091861
228_2	-650.434663	-650.287896	-649.238600	-649.091833
228_3	-650.434672	-650.287895	-649.238599	-649.091823
228_4	-650.434675	-650.287894	-649.238598	-649.091817
228_5	-650.434680	-650.287856	-649.238595	-649.091772
228_6	-650.432992	-650.286215	-649.236896	-649.090120
228_7	-650.432444	-650.285704	-649.236086	-649.089345
**228-Me^{+}**	-690.128102	-689.939715	-688.824370	-688.635984
**229**				-1066.016166
229_1	-1067.974657	-1067.783664	-1066.207201	-1066.016209
229_2	-1067.971924	-1067.780738	-1066.203045	-1066.011859
**229-Me^{+}**	-1107.667765	-1107.435241	-1105.793406	-1105.560881
**230**				-987.744985
230_1	-989.429649	-989.297766	-987.877085	-987.745202
230_2	-989.427781	-989.295985	-987.874826	-987.743030
**230-Me^{+}**	-1029.122172	-1028.948846	-1027.463199	-1027.289873
**231**				-971.702515
231_1	-973.387444	-973.243801	-971.846587	-971.702944
231_2	-973.386465	-973.242770	-971.845533	-971.701838
231_3	-973.386048	-973.242438	-971.845291	-971.701680
**231-Me^{+}**	-1013.082283	-1012.897182	-1011.435375	-1011.250273
**232**				-613.182508
232_1	-614.520413	-614.350840	-613.352196	-613.182623
232_2	-614.520407	-614.350609	-613.352154	-613.182356
**232-Me^{+}**	-654.217279	-654.006037	-652.942159	-652.730917
**233**				-1483.337396
233_1	-1485.778286	-1485.612113	-1483.504016	-1483.337843
233_2	-1485.777789	-1485.611619	-1483.503375	-1483.337205
233_3	-1485.777502	-1485.611400	-1483.503201	-1483.337099
233_4	-1485.777250	-1485.611099	-1483.502555	-1483.336404
**233-Me^{+}**				-1522.886086
233-Me^{+}_1	-1525.474545	-1525.266974	-1523.094010	-1522.886439
233-Me^{+}_2	-1525.474218	-1525.266556	-1523.093841	-1522.886178
233-Me^{+}_3	-1525.473594	-1525.265920	-1523.093104	-1522.885431
233-Me^{+}_4	-1525.472540	-1525.264891	-1523.092098	-1522.884449
**234**				-766.296366
234_1	-768.044863	-767.826432	-766.515166	-766.296735
234_2	-768.044235	-767.825901	-766.514425	-766.296091

234_3	-768.043849	-767.825428	-766.513627	-766.295207
234_4	-768.042749	-767.824328	-766.512588	-766.294166
234_5	-768.042484	-767.824209	-766.512194	-766.293919
**234-Me⁺**				-805.845669
234-Me⁺_1	-807.743813	-807.483677	-806.106174	-805.846038
234-Me⁺_2	-807.742601	-807.482556	-806.105223	-805.845178
234-Me⁺_3	-807.741616	-807.481370	-806.104024	-805.843778
**235**				-919.410816
235_1	-921.568260	-921.301322	-919.678444	-919.411506
235_2	-921.566635	-921.299329	-919.676977	-919.409671
235_3	-921.567207	-921.300248	-919.676199	-919.409240
235_4	-921.567183	-921.300220	-919.676050	-919.409087
235_5	-921.567096	-921.300083	-919.675933	-919.408921
235_6	-921.565618	-921.298733	-919.675015	-919.408129
235_7	-921.564773	-921.297747	-919.674464	-919.407437
235_8	-921.568260	-921.301322	-919.678444	-919.411506
**235-Me⁺**				-958.960445
235-Me⁺_1	-961.267768	-960.958886	-959.269700	-958.960819
235-Me⁺_2	-961.267283	-960.958323	-959.269225	-958.960265
235-Me⁺_3	-961.266864	-960.957930	-959.269197	-958.960263
235-Me⁺_4	-961.264610	-960.955775	-959.266321	-958.957486
235-Me⁺_5	-961.262516	-960.953976	-959.265159	-958.956618
**236**				-919.412774
236_1	-921.577086	-921.310366	-919.679831	-919.413111
236_2	-921.577254	-921.310457	-919.679855	-919.413058
236_3	-921.576598	-921.309916	-919.679145	-919.412463
236_4	-921.576548	-921.309878	-919.679113	-919.412444
236_5	-921.575356	-921.308615	-919.677456	-919.410714
236_6	-921.575198	-921.308369	-919.677104	-919.410276
**236-Me⁺**				-958.968248
236-Me⁺_1	-961.285400	-960.977190	-959.276851	-958.968641
236-Me⁺_2	-961.285408	-960.977179	-959.276808	-958.968579
236-Me⁺_3	-961.284487	-960.976334	-959.276108	-958.967956
236-Me⁺_4	-961.283253	-960.975094	-959.275128	-958.966970
236-Me⁺_5	-961.282984	-960.974718	-959.274200	-958.965933
236-Me⁺_6	-961.282015	-960.973921	-959.273435	-958.965341
236-Me⁺_7	-961.281966	-960.973906	-959.273320	-958.965259
**237**				-1994.975053
237_1	-1998.169527	-1997.980839	-1995.164061	-1994.975373
237_2	-1998.169433	-1997.980792	-1995.163825	-1994.975184
237_3	-1998.169059	-1997.980362	-1995.163265	-1994.974568
237_4	-1998.168028	-1997.979372	-1995.162246	-1994.973590
**237-Me⁺**				-2034.525420
237-Me⁺_1	-2037.867664	-2037.637647	-2034.755787	-2034.525770
237-Me⁺_2	-2037.867745	-2037.637626	-2034.755766	-2034.525647
237-Me⁺_3	-2037.866807	-2037.636670	-2034.754951	-2034.524814
237-Me⁺_4	-2037.865672	-2037.635532	-2034.753903	-2034.523763
237-Me⁺_5	-2037.865287	-2037.635159	-2034.753186	-2034.523057

**238**				-613.183210
238_1	-614.524083	-614.354254	-613.353179	-613.183351
238_2	-614.524492	-614.354650	-613.352851	-613.183009
**238-Me$^+$**	-654.225148	-654.013527	-652.945572	-652.733951
**239**	-693.174735	-692.939912	-691.799023	-691.564200
**239-Me$^+$**	-732.873814	-732.597328	-731.391450	-731.114964
**240**				-1066.024884
240_1	-1067.985219	-1067.793848	-1066.216311	-1066.024940
240_2	-1067.980376	-1067.789156	-1066.212156	-1066.020936
**240-Me$^+$**	-1107.685965	-1107.452731	-1105.808940	-1105.575707
**241**				-766.297407
241_1	-768.050562	-767.832289	-766.516104	-766.297831
241_2	-768.050026	-767.831780	-766.515053	-766.296807
241_3	-768.049482	-767.831347	-766.514565	-766.296430
241_4	-768.049062	-767.830760	-766.513772	-766.295471
241_5	-768.048015	-767.829691	-766.512628	-766.294304
**241-Me$^+$**				-805.849374
241-Me$^+$_1	-807.753821	-807.494231	-806.109218	-805.849628
241-Me$^+$_2	-807.753251	-807.493637	-806.109090	-805.849476
241-Me$^+$_3	-807.751995	-807.492404	-806.107319	-805.847728
241-Me$^+$_4	-807.751456	-807.491935	-806.106217	-805.846697
**242**				-766.299179
242_1	-768.053509	-767.834952	-766.518158	-766.299601
242_2	-768.052623	-767.834045	-766.516844	-766.298266
242_3	-768.052013	-767.833510	-766.516236	-766.297733
242_4	-768.051868	-767.833245	-766.515799	-766.297175
242_5	-768.050627	-767.831984	-766.514289	-766.295646
**242-Me$^+$**				-805.851748
242-Me$^+$_1	-807.757550	-807.498431	-806.111394	-805.852275
242-Me$^+$_2	-807.758826	-807.497173	-806.113072	-805.851419
242-Me$^+$_3	-807.758961	-807.498548	-806.111110	-805.850697
242-Me$^+$_4	-807.756984	-807.496566	-806.111110	-805.850692
**243**				-535.956121
243_1	-537.067118	-536.935525	-536.087967	-535.956373
243_2	-537.065932	-536.934333	-536.087046	-535.955448
**243-Me$^+$**	-576.768239	-576.595088	-575.682105	-575.508954
**244**				-919.412774
244_1	-921.577086	-921.310366	-919.679831	-919.413111
244_2	-921.577254	-921.310457	-919.679855	-919.413058
244_3	-921.576598	-921.309916	-919.679145	-919.412463
244_4	-921.576548	-921.309878	-919.679113	-919.412444
244_5	-921.575356	-921.308615	-919.677456	-919.410714
244_6	-921.575198	-921.308369	-919.677104	-919.410276
**244-Me$^+$**				-958.968248
244-Me$^+$_1	-961.285400	-960.977190	-959.276851	-958.968641
244-Me$^+$_2	-961.285408	-960.977179	-959.276808	-958.968579
244-Me$^+$_3	-961.284487	-960.976334	-959.276108	-958.967956
244-Me$^+$_4	-961.283253	-960.975094	-959.275128	-958.966970

244-Me$^+$_5	-961.282984	-960.974718	-959.274200	-958.965933
244-Me$^+$_6	-961.282015	-960.973921	-959.273435	-958.965341
244-Me$^+$_7	-961.281966	-960.973906	-959.273320	-958.965259
**245**				-919.415257
245_1	-921.581169	-921.314033	-919.682696	-919.415560
245_2	-921.581236	-921.314038	-919.682692	-919.415494
245_3	-921.581255	-921.314024	-919.682697	-919.415467
245_4	-921.581204	-921.313849	-919.682376	-919.415020
245_5	-921.580728	-921.313355	-919.681875	-919.414502
245_6	-921.579818	-921.312522	-919.680523	-919.413227
245_7	-921.579237	-921.311913	-919.680483	-919.413159
**245-Me$^+$**				-958.973625
245-Me$^+$_1	-961.292979	-960.983677	-959.283226	-958.973924
245-Me$^+$_2	-961.292860	-960.983561	-959.283155	-958.973857
245-Me$^+$_3	-961.291557	-960.982206	-959.281682	-958.972331
245-Me$^+$_4	-961.290451	-960.981219	-959.280373	-958.971141
245-Me$^+$_5	-961.290182	-960.980837	-959.280342	-958.970997
**246**				-611.842008
246_1	-613.137201	-612.995130	-611.984567	-611.842497
246_2	-613.136683	-612.994535	-611.984395	-611.842247
246_3	-613.136047	-612.993870	-611.984071	-611.841894
246_4	-613.134812	-612.992536	-611.983461	-611.841185
246_5	-613.134951	-612.992770	-611.983336	-611.841155
246_6	-613.134951	-612.992843	-611.982946	-611.840838
**246-Me$^+$**				-651.400602
246-Me$^+$_1	-652.844643	-652.661546	-651.584145	-651.401048
246-Me$^+$_2	-652.845314	-652.660843	-651.584641	-651.400170
246-Me$^+$_3	-652.844526	-652.660700	-651.583686	-651.399860
246-Me$^+$_4	-652.842007	-652.658190	-651.581169	-651.397352
**247**				-687.727924
247_1	-689.206264	-689.053497	-687.881037	-687.728271
247_2	-689.206767	-689.054056	-687.880745	-687.728034
247_3	-689.205192	-689.052406	-687.880751	-687.727965
247_4	-689.204628	-689.051856	-687.880078	-687.727305
247_5	-689.203585	-689.050807	-687.879864	-687.727087
**247-Me$^+$**				-727.291390
247-Me$^+$_1	-728.920553	-728.726127	-727.486252	-727.291827
247-Me$^+$_2	-728.920447	-728.725989	-727.485903	-727.291445
247-Me$^+$_3	-728.919914	-728.725474	-727.485862	-727.291422
247-Me$^+$_4	-728.919805	-728.725330	-727.485408	-727.290933
247-Me$^+$_5	-728.919451	-728.724976	-727.484530	-727.290055
247-Me$^+$_6	-728.918393	-728.723912	-727.483407	-727.288926
**248**	-536.225681	-536.099230	-535.308221	-535.181770
**248-Me$^+$**	-575.882484	-575.713513	-574.844221	-574.675249
**249**	-2523.894841	-2523.713559	-2519.500822	-2519.319540
**249-Me$^+$**	-2563.554775	-2563.331554	-2559.047886	-2558.824665
**250**	-1649.24295	-1648.93677	-1646.03893	-1645.732746
**250-Me$^+$**				-1685.250266

250-Me$^+$_1	-1688.90583	-1688.55881	-1685.59743	-1685.250414
250-Me$^+$_2	-1688.90556	-1688.55848	-1685.59713	-1685.250048
**251**				-1015.047339
251_1	-1017.521315	-1017.178932	-1015.390461	-1015.048078
251_2	-1017.520917	-1017.178765	-1015.389683	-1015.047530
251_3	-1017.519673	-1017.177437	-1015.388717	-1015.046481
251_4	-1017.519945	-1017.177599	-1015.388561	-1015.046215
251_5	-1017.519444	-1017.177449	-1015.388163	-1015.046168
251_6	-1017.519492	-1017.177218	-1015.388381	-1015.046107
251_7	-1017.519407	-1017.177054	-1015.388397	-1015.046044
251_8	-1017.517992	-1017.175734	-1015.386126	-1015.043868
**251-Me$^+$ (N)**				-1054.572022
251-Me$^+$(N)_1	-1057.198020	-1056.811721	-1054.959205	-1054.572906
251-Me$^+$(N)_2	-1057.197833	-1056.811156	-1054.958759	-1054.572082
251-Me$^+$(N)_3	-1057.197066	-1056.810509	-1054.957803	-1054.571246
251-Me$^+$(N)_4	-1057.196345	-1056.809823	-1054.956904	-1054.570381
251-Me$^+$(N)_5	-1057.195209	-1056.808542	-1054.956660	-1054.569993
251-Me$^+$(N)_6	-1057.195073	-1056.808548	-1054.956477	-1054.569952
251-Me$^+$(N)_7	-1057.193872	-1056.807417	-1054.956091	-1054.569636
251-Me$^+$(N)_8	-1057.193835	-1056.807355	-1054.956008	-1054.569527
251-Me$^+$(N)_9	-1057.194774	-1056.808225	-1054.955414	-1054.568865
251-Me$^+$(N)_10	-1057.193638	-1056.807014	-1054.955385	-1054.568761
**251-Me$^+$ (P)**				-1054.605860
251-Me$^+$(P)_1	-1057.229043	-1056.844496	-1054.991000	-1054.606453
251-Me$^+$(P)_2	-1057.228821	-1056.844210	-1054.990974	-1054.606363
251-Me$^+$(P)_3	-1057.228287	-1056.843498	-1054.990710	-1054.605921
251-Me$^+$(P)_4	-1057.228265	-1056.843689	-1054.990425	-1054.605849
251-Me$^+$(P)_5	-1057.228087	-1056.843460	-1054.990254	-1054.605627
251-Me$^+$(P)_6	-1057.227581	-1056.843031	-1054.990125	-1054.605575
251-Me$^+$(P)_7	-1057.227284	-1056.842670	-1054.989639	-1054.605025
251-Me$^+$(P)_8	-1057.227247	-1056.842350	-1054.989758	-1054.604861
251-Me$^+$(P)_9	-1057.226926	-1056.842184	-1054.989100	-1054.604358
251-Me$^+$(P)_10	-1057.227102	-1056.842540	-1054.988605	-1054.604043
**252**				-1258.737910
252_1	-1261.589855	-1261.283840	-1259.045037	-1258.739022
252_2	-1261.590061	-1261.284158	-1259.043821	-1258.737918
252_3	-1261.589096	-1261.283254	-1259.043346	-1258.737503
252_4	-1261.589526	-1261.283606	-1259.043242	-1258.737322
252_5	-1261.589484	-1261.283582	-1259.043084	-1258.737183
252_6	-1261.589835	-1261.283768	-1259.042919	-1258.736852
252_7	-1261.589583	-1261.283609	-1259.042817	-1258.736843
252_8	-1261.589505	-1261.283596	-1259.042608	-1258.736699
252_9	-1261.589459	-1261.283527	-1259.042626	-1258.736694
252_10	-1261.588915	-1261.282976	-1259.042441	-1258.736502
**252-Me$^+$**				-1298.274109
252-Me$^+$_1	-1301.273026	-1300.925392	-1298.623044	-1298.275411
252-Me$^+$_2	-1301.273055	-1300.925446	-1298.621478	-1298.273868
252-Me$^+$_3	-1301.273057	-1300.925416	-1298.621459	-1298.273818

252-Me$^+$_4	-1301.273123	-1300.925543	-1298.621246	-1298.273666
252-Me$^+$_5	-1301.273019	-1300.925362	-1298.621302	-1298.273644
252-Me$^+$_6	-1301.273163	-1300.925492	-1298.621170	-1298.273499
252-Me$^+$_7	-1301.272945	-1300.925256	-1298.621155	-1298.273466
252-Me$^+$_8	-1301.273088	-1300.925520	-1298.621021	-1298.273452
252-Me$^+$_9	-1301.273083	-1300.925442	-1298.620870	-1298.273228
252-Me$^+$_10	-1301.273104	-1300.925465	-1298.620661	-1298.273022
**253**				-803.191765
253_1	-804.999895	-804.796479	-803.395397	-803.191981
253_2	-804.999347	-804.795976	-803.394709	-803.191337
**253-Me$^+$**				-842.728541
253-Me$^+$_1	-844.688201	-844.442655	-842.974398	-842.728852
253-Me$^+$_2	-844.686573	-844.442120	-842.972785	-842.728332
253-Me$^+$_3	-844.686094	-844.442022	-842.972207	-842.728135
**254**				-2410.678523
254_1	-2414.583096	-2414.317826	-2410.943810	-2410.678540
254_2	-2414.583097	-2414.317830	-2410.943772	-2410.678505
**254-Me$^+$**	-2454.272737	-2453.965773	-2450.525791	-2450.218827
**255**				-802.774620
255_1	-804.506279	-804.276401	-803.005409	-802.775530
255_2	-804.506089	-804.276195	-803.005153	-802.775259
255_3	-804.505590	-804.275775	-803.004587	-802.774772
255_4	-804.504538	-804.274753	-803.004186	-802.774402
255_5	-804.504601	-804.274758	-803.003909	-802.774066
255_6	-804.504835	-804.275009	-803.003592	-802.773766
255_7	-804.504054	-804.274249	-803.003157	-802.773352
255_8	-804.504420	-804.274682	-803.002933	-802.773195
255_9	-804.501447	-804.271655	-803.000362	-802.770570
255_10	-804.501353	-804.271584	-803.000291	-802.770522
**255-Me$^+$**				-842.320031
255-Me$^+$_1	-844.201364	-843.929492	-842.592470	-842.320599
255-Me$^+$_2	-844.200938	-843.929042	-842.592293	-842.320396
255-Me$^+$_3	-844.200725	-843.928862	-842.591959	-842.320096
255-Me$^+$_4	-844.200746	-843.928853	-842.591906	-842.320014
255-Me$^+$_5	-844.200858	-843.929055	-842.591750	-842.319948
255-Me$^+$_6	-844.200869	-843.929052	-842.591736	-842.319919
255-Me$^+$_7	-844.200593	-843.928645	-842.591660	-842.319712
255-Me$^+$_8	-844.200024	-843.928114	-842.590819	-842.318909
255-Me$^+$_9	-844.198477	-843.926761	-842.590468	-842.318752
255-Me$^+$_10	-844.197960	-843.926066	-842.590029	-842.318135
**256**				-651.216018
256_1	-652.650368	-652.472788	-651.393617	-651.216037
256_2	-652.650371	-652.472774	-651.393596	-651.215999
**256-Me$^+$**	-692.348328	-692.129003	-690.984052	-690.764727
**257**				-1151.023123
257_1	-1153.86154	-1153.48264	-1151.40213	-1151.023231
257_2	-1153.85706	-1153.47789	-1151.39925	-1151.020076
**257-Me$^+$**				-1190.572649

257-Me$^+$_1	-1193.56149	-1193.14013	-1190.99428	-1190.572912
257-Me$^+$_2	-1193.55958	-1193.13810	-1190.99324	-1190.571761
**258**				-1657.911400
258_1	-1661.462308	-1661.065583	-1658.309106	-1657.912381
258_2	-1661.462071	-1661.065240	-1658.308088	-1657.911256
258_3	-1661.463311	-1661.066127	-1658.308034	-1657.910850
258_4	-1661.461339	-1661.064207	-1658.30744	-1657.910313
258_5	-1661.459424	-1661.062480	-1658.306968	-1657.910024
258_6	-1661.460594	-1661.063415	-1658.306355	-1657.909175
258_7	-1661.458753	-1661.061795	-1658.305665	-1657.908707
258_8	-1661.459045	-1661.061944	-1658.305791	-1657.908689
258_9	-1661.458797	-1661.061967	-1658.305480	-1657.908650
258_10	-1661.460361	-1661.063382	-1658.305541	-1657.908562
**258-Me$^+$**				-1697.461550
258-Me$^+$_1	-1701.160693	-1700.722158	-1697.901221	-1697.462686
258-Me$^+$_2	-1701.160409	-1700.722024	-1697.900752	-1697.462367
258-Me$^+$_3	-1701.161681	-1700.723212	-1697.899661	-1697.461192
258-Me$^+$_4	-1701.161068	-1700.722710	-1697.899488	-1697.461130
258-Me$^+$_5	-1701.160985	-1700.722308	-1697.899598	-1697.460921
258-Me$^+$_6	-1701.160897	-1700.722238	-1697.899526	-1697.460868
258-Me$^+$_7	-1701.161398	-1700.722844	-1697.899394	-1697.460840
258-Me$^+$_8	-1701.161096	-1700.722895	-1697.898928	-1697.460727
258-Me$^+$_9	-1701.163479	-1700.724660	-1697.899423	-1697.460604
258-Me$^+$_10	-1701.161523	-1700.722483	-1697.899156	-1697.460116
**259**	-844.304704	-844.070628	-842.598952	-842.364875
**259-Me$^+$**	-884.006242	-883.730300	-882.191652	-881.915710
**260**				-1358.462628
260_1	-1361.789986	-1361.350525	-1358.902163	-1358.462703
260_2	-1361.792119	-1361.352635	-1358.898542	-1358.459058
**260-Me$^+$**				-1398.014396
260-Me$^+$_1	-1401.490580	-1401.009530	-1398.495510	-1398.014664
260-Me$^+$_2	-1401.490649	-1401.009803	-1398.491458	-1398.010408
**261**				-729.538558
261_1	-731.242508	-731.004591	-729.777017	-729.539100
261_2	-731.241901	-731.003984	-729.775727	-729.537810
261_3	-731.240559	-731.002514	-729.774730	-729.536684
261_4	-731.240369	-731.002389	-729.774510	-729.536530
261_5	-731.239942	-731.002029	-729.772997	-729.535084
**261-Me$^+$**				-769.090802
261-Me$^+$_1	-770.944211	-770.664324	-769.371333	-769.091446
261-Me$^+$_2	-770.943462	-770.663624	-769.370103	-769.090264
261-Me$^+$_3	-770.943685	-770.663679	-769.370037	-769.090031
261-Me$^+$_4	-770.943206	-770.663210	-769.369725	-769.089729
261-Me$^+$_5	-770.941262	-770.661285	-769.367689	-769.087712
261-Me$^+$_6	-770.941386	-770.661276	-769.367491	-769.087381
**262**				-1490.831178
262_1	-1494.525198	-1494.028325	-1491.328762	-1490.831889
262_2	-1494.525381	-1494.028383	-1491.328842	-1490.831844
262_3	-1494.525085	-1494.028297	-1491.328621	-1490.831833

262_4	-1494.528937	-1494.032300	-1491.328451	-1490.831814
262_5	-1494.526528	-1494.030016	-1491.327901	-1490.831390
262_6	-1494.527484	-1494.030993	-1491.327358	-1490.830867
262_7	-1494.527769	-1494.031225	-1491.327263	-1490.830719
262_8	-1494.527461	-1494.030734	-1491.327333	-1490.830606
262_9	-1494.522877	-1494.026103	-1491.327105	-1490.830331
262_10	-1494.526770	-1494.029977	-1491.326982	-1490.830189
**262-Me$^+$**				-1530.384133
262-Me$^+$_1	-1534.229258	-1533.691206	-1530.923030	-1530.384978
262-Me$^+$_2	-1534.230525	-1533.692400	-1530.922682	-1530.384557
262-Me$^+$_3	-1534.228669	-1533.690370	-1530.922703	-1530.384404
262-Me$^+$_4	-1534.229240	-1533.691216	-1530.920974	-1530.382950
262-Me$^+$_5	-1534.230569	-1533.692286	-1530.921081	-1530.382799
262-Me$^+$_6	-1534.227103	-1533.688709	-1530.920966	-1530.382572
262-Me$^+$_7	-1534.228368	-1533.690196	-1530.919888	-1530.381717
262-Me$^+$_8	-1534.228426	-1533.690097	-1530.919818	-1530.381489
262-Me$^+$_9	-1534.225880	-1533.687385	-1530.919936	-1530.381442
262-Me$^+$_10	-1534.227028	-1533.688689	-1530.919705	-1530.381366
**263**				-1809.900130
263_1	-1813.818159	-1813.395696	-1810.322954	-1809.900491
263_2	-1813.816309	-1813.393495	-1810.323251	-1809.900436
263_3	-1813.816942	-1813.394272	-1810.322602	-1809.899931
263_4	-1813.820087	-1813.397387	-1810.320767	-1809.898067
263_5	-1813.818264	-1813.395519	-1810.320474	-1809.897728
263_6	-1813.819013	-1813.396414	-1810.320161	-1809.897562
**263-Me$^+$**				-1849.453115
263-Me$^+$_1	-1853.522425	-1853.058195	-1849.917861	-1849.453631
263-Me$^+$_2	-1853.519519	-1853.055440	-1849.917166	-1849.453087
263-Me$^+$_3	-1853.523725	-1853.059560	-1849.916017	-1849.451851
263-Me$^+$_4	-1853.514162	-1853.049793	-1849.914729	-1849.450360
263-Me$^+$_5	-1853.521643	-1853.057513	-1849.914235	-1849.450104
263-Me$^+$_6	-1853.516234	-1853.052250	-1849.913988	-1849.450004
263-Me$^+$_7	-1853.521678	-1853.057443	-1849.913818	-1849.449484
263-Me$^+$_8	-1853.516234	-1853.051503	-1849.914042	-1849.449311
**264**				-881.526040
264_1	-883.600535	-883.336225	-881.790710	-881.526399
264_2	-883.600225	-883.335949	-881.790086	-881.525810
264_3	-883.599193	-883.334898	-881.789481	-881.525186
**264-Me$^+$**				-921.079123
264-Me$^+$_1	-923.303727	-922.997521	-921.385495	-921.079288
264-Me$^+$_2	-923.303724	-922.997522	-921.385283	-921.079081
264-Me$^+$_3	-923.303821	-922.997589	-921.385163	-921.078931
**265**				-1487.328628
265_1	-1490.895483	-1490.470560	-1487.754042	-1487.329108
265_2	-1490.893855	-1490.469421	-1487.752232	-1487.327798
265_3	-1490.893865	-1490.469485	-1487.752107	-1487.327727
**265-Me$^+$**				-1526.881927
265-Me$^+$_1	-1530.599755	-1530.133399	-1527.348652	-1523.882296

265-Me$^+$_2	-1530.599329	-1530.133042	-1527.346197	1523.879910
265-Me$^+$_3	-1530.597510	-1530.130908	-1527.345774	1523.879172
265-Me$^+$_4	-1530.598985	-1530.132687	-1527.344998	-1526.878700
**266**				-1683.413063
266_1	-1687.618353	-1687.030733	-1684.000867	-1683.413247
266_2	-1687.618739	-1687.030437	-1683.997496	-1683.409194
266_3	-1687.620564	-1687.032430	-1683.996926	-1683.408792
266_4	-1687.621353	-1687.033149	-1683.996221	-1683.408018
266_5	-1687.620660	-1687.032849	-1683.995701	-1683.407890
266_6	-1687.621037	-1687.032541	-1683.996206	-1683.407711
266_7	-1687.616720	-1687.028930	-1683.995411	-1683.40762
266_8	-1687.614953	-1687.026711	-1683.995558	-1683.407315
266_9	-1687.623195	-1687.035194	-1683.994486	-1683.406485
266_10	-1687.614429	-1687.025989	-1683.994682	-1683.406241
**266-Me$^+$**				-1722.966635
266-Me$^+$_1	-1727.322624	-1726.693228	-1723.596275	-1722.966879
266-Me$^+$_2	-1727.320829	-1726.691508	-1723.595372	-1722.966052
266-Me$^+$_3	-1727.315320	-1726.686036	-1723.586417	-1722.957133
266-Me$^+$_4	-1727.318242	-1726.688718	-1723.586598	-1722.957074
266-Me$^+$_5	-1727.315126	-1726.685868	-1723.585950	-1722.956831
266-Me$^+$_6	-1727.316674	-1726.686848	-1723.586551	-1722.956725
266-Me$^+$_7	-1727.315632	-1726.685761	-1723.582782	-1722.956079
266-Me$^+$_8	-1727.314321	-1726.686007	-1723.584004	-1722.955690
266-Me$^+$_9	-1727.311606	-1726.682233	-1723.585021	-1722.955648
266-Me$^+$_10	-1727.317185	-1726.688000	-1723.584637	-1722.955453
**267**				-919.488942
267_1	-921.654821	-921.384448	-919.759576	-919.489204
267_2	-921.653023	-921.382476	-919.758638	-919.488091
**267-Me$^+$**				-959.042917
267-Me$^+$_1	-961.359255	-961.046783	-959.355527	-959.043056
267-Me$^+$_2	-961.357189	-961.044744	-959.352717	-959.040272
**268**				-920.691196
268_1	-922.896755	-922.602898	-920.985314	-920.691457
268_2	-922.895678	-922.601783	-920.984493	-920.690598
**268-Me$^+$**				-960.245410
268-Me$^+$_1	-962.601255	-962.265188	-960.581779	-960.245712
268-Me$^+$_2	-962.600394	-962.264379	-960.580232	-960.244217
268-Me$^+$_3	-962.598264	-962.262182	-960.577869	-960.241787
**269**				-807.862770
269_1	-809.837632	-809.539939	-808.161715	-807.864022
269_2	-809.836474	-809.538685	-808.160865	-807.863076
269_3	-809.835628	-809.537926	-808.160489	-807.862787
269_4	-809.837026	-809.539258	-808.160423	-807.862655
269_5	-809.835262	-809.537569	-808.159993	-807.862300
269_6	-809.834441	-809.536783	-808.159711	-807.862053
269_7	-809.835553	-809.537715	-808.159795	-807.861958
269_8	-809.835828	-809.538099	-808.159384	-807.861655
269_9	-809.835812	-809.537988	-808.159435	-807.861611
269_10	-809.835355	-809.537544	-808.159325	-807.861514
**269-Me$^+$**				-847.417317
269-Me$^+$_1	-849.541369	-849.201709	-847.758269	-847.418608

269-Me$^+$_2	-849.540928	-849.201068	-847.757606	-847.417746
269-Me$^+$_3	-849.540902	-849.201282	-847.757115	-847.417495
269-Me$^+$_4	-849.540796	-849.200966	-847.757314	-847.417485
269-Me$^+$_5	-849.540456	-849.200775	-847.756921	-847.417241
269-Me$^+$_6	-849.538956	-849.199242	-847.756714	-847.417000
269-Me$^+$_7	-849.538882	-849.199229	-847.756525	-847.416873
269-Me$^+$_8	-849.538445	-849.198609	-847.756030	-847.416193
269-Me$^+$_9	-849.538322	-849.198550	-847.755673	-847.415902
269-Me$^+$_10	-849.538452	-849.198744	-847.755417	-847.415709
**270**				-920.687952
270_1	-922.898107	-922.603832	-920.983004	-920.688729
270_2	-922.897760	-922.603512	-920.982687	-920.688439
270_3	-922.897758	-922.603590	-920.982586	-920.688418
270_4	-922.896679	-922.602261	-920.982341	-920.687923
270_5	-922.896882	-922.602685	-920.982078	-920.687881
270_6	-922.896535	-922.602358	-920.981927	-920.687750
270_7	-922.896594	-922.602403	-920.981626	-920.687434
270_8	-922.895610	-922.601459	-920.981119	-920.686968
270_9	-922.895210	-922.600975	-920.980659	-920.686424
270_10	-922.893075	-922.598767	-920.980404	-920.686096
**270-Me$^+$**				-960.242397
270-Me$^+$_1	-962.602259	-962.266019	-960.579137	-960.242897
270-Me$^+$_2	-962.602260	-962.266204	-960.578759	-960.242703
270-Me$^+$_3	-962.602335	-962.266104	-960.578907	-960.242676
270-Me$^+$_4	-962.599348	-962.263605	-960.577145	-960.241402
270-Me$^+$_5	-962.598853	-962.262830	-960.577166	-960.241143
270-Me$^+$_6	-962.599740	-962.263703	-960.577079	-960.241042
270-Me$^+$_7	-962.599753	-962.263616	-960.577150	-960.241013
270-Me$^+$_8	-962.599333	-962.263123	-960.577069	-960.240859
**271**				-1108.581393
271_1	-1111.152070	-1110.856066	-1108.877413	-1108.581410
271_2	-1111.152118	-1110.856093	-1108.877402	-1108.581376
**271-Me$^+$**				-1148.136083
271-Me$^+$_1	-1150.859563	-1150.521644	-1148.474067	-1148.136147
271-Me$^+$_2	-1150.859403	-1150.521446	-1148.473965	-1148.136008
**272**				-1566.051226
272_1	-1530.196873	-1529.742469	-1526.951399	-1526.496994
272_2	-1530.195226	-1529.741392	-1526.949819	-1526.495985
272_3	-1530.195135	-1529.741201	-1526.949647	-1526.495713
**272-Me$^+$**				-1566.051226
272-Me$^+$_1	-1569.902003	-1569.406389	-1566.547243	-1566.051628
272-Me$^+$_2	-1569.902058	-1569.406323	-1566.546710	-1566.050976
272-Me$^+$_3	-1569.901792	-1569.406218	-1566.545124	-1566.049551
272-Me$^+$_4	-1569.899793	-1569.403753	-1566.544432	-1566.048391
**273**	-1075.26176	-1074.94184	-1073.00463	-1072.684702
**273-Me$^+$**	-1114.96868	-1114.60686	-1112.60145	-1112.239624
**274**	-938.924773	-938.642225	-937.001460	-936.718912
**274-Me$^+$**				-976.273851

274-Me$^+$_1	-978.629767	-978.304709	-976.599231	-976.274173
274-Me$^+$_2	-978.629123	-978.304371	-976.598314	-976.273562
274-Me$^+$_3	-978.629210	-978.304477	-976.598239	-976.273506
**275**				-999.011768
275_1	-1001.491033	-1001.136749	-999.367115	-999.012831
275_2	-1001.492955	-1001.138893	-999.366623	-999.012561
275_3	-1001.489623	-1001.135570	-999.366485	-999.012433
275_4	-1001.491647	-1001.137574	-999.366433	-999.012361
275_5	-1001.492603	-1001.138603	-999.366343	-999.012344
275_6	-1001.491640	-1001.137689	-999.366223	-999.012273
275_7	-1001.491467	-1001.137439	-999.366215	-999.012187
275_8	-1001.491755	-1001.137590	-999.366087	-999.011923
275_9	-1001.490627	-1001.136474	-999.366066	-999.011913
275_10	-1001.491721	-1001.137606	-999.366012	-999.011896
**275-Me$^+$**				-1038.566881
275-Me$^+$_1	-1041.198013	-1040.801912	-1038.963949	-1038.567847
275-Me$^+$_2	-1041.198060	-1040.802135	-1038.963545	-1038.567621
275-Me$^+$_3	-1041.198115	-1040.802055	-1038.963493	-1038.567434
275-Me$^+$_4	-1041.196743	-1040.800766	-1038.963373	-1038.567396
275-Me$^+$_5	-1041.196906	-1040.800759	-1038.963537	-1038.567390
275-Me$^+$_6	-1041.196667	-1040.800673	-1038.963344	-1038.567350
275-Me$^+$_7	-1041.196824	-1040.800690	-1038.963347	-1038.567213
275-Me$^+$_8	-1041.196757	-1040.800619	-1038.963346	-1038.567208
275-Me$^+$_9	-1041.196170	-1040.800005	-1038.963284	-1038.567119
275-Me$^+$_10	-1041.195882	-1040.799667	-1038.963331	-1038.567117
**276**				-959.852335
276_1	-962.192681	-961.868724	-960.176936	-959.852979
276_2	-962.192573	-961.868672	-960.176859	-959.852958
276_3	-962.191576	-961.867463	-960.176452	-959.852339
276_4	-962.191323	-961.867418	-960.175930	-959.852025
276_5	-962.191513	-961.867561	-960.175972	-959.852020
276_6	-962.191723	-961.867658	-960.176050	-959.851984
276_7	-962.191408	-961.867487	-960.175873	-959.851952
276_8	-962.190095	-961.866239	-960.175277	-959.851421
276_9	-962.190395	-961.866478	-960.175060	-959.851143
276_10	-962.189708	-961.865890	-960.174945	-959.851127
**276-Me$^+$**				-999.407630
276-Me$^+$_1	-1001.898044	-1001.532038	-999.774541	-999.408535
276-Me$^+$_2	-1001.897905	-1001.531873	-999.774223	-999.408191
276-Me$^+$_3	-1001.897455	-1001.531353	-999.773090	-999.406988
276-Me$^+$_4	-1001.897294	-1001.531243	-999.773014	-999.406964
276-Me$^+$_5	-1001.895218	-1001.529263	-999.772498	-999.406543
276-Me$^+$_6	-1001.894858	-1001.528841	-999.771751	-999.405734
276-Me$^+$_7	-1001.895214	-1001.528903	-999.771918	-999.405607
276-Me$^+$_8	-1001.895214	-1001.528892	-999.771916	-999.405593
276-Me$^+$_9	-1001.894876	-1001.528731	-999.771623	-999.405478
276-Me$^+$_10	-1001.894861	-1001.528884	-999.771446	-999.405469
**277**				-999.013287

277_1	-1001.487389	-1001.133246	-999.368183	-999.014040
277_2	-1001.487119	-1001.133203	-999.367870	-999.013955
277_3	-1001.487324	-1001.133181	-999.368027	-999.013885
277_4	-1001.485909	-1001.131930	-999.367468	-999.013489
277_5	-1001.485723	-1001.131765	-999.366507	-999.012550
277_6	-1001.485021	-1001.131242	-999.366181	-999.012402
277_7	-1001.485885	-1001.131762	-999.366511	-999.012387
277_8	-1001.484680	-1001.130751	-999.366072	-999.012143
277_9	-1001.484325	-1001.130494	-999.365880	-999.012050
277_10	-1001.484739	-1001.130552	-999.366051	-999.011864
**277-Me$^+$**				-1038.568563
277-Me$^+$_1	-1041.192023	-1040.796224	-1038.965397	-1038.569598
277-Me$^+$_2	-1041.192169	-1040.795939	-1038.965434	-1038.569204
277-Me$^+$_3	-1041.192383	-1040.796427	-1038.965060	-1038.569104
277-Me$^+$_4	-1041.191823	-1040.795785	-1038.964787	-1038.568749
277-Me$^+$_5	-1041.191896	-1040.795677	-1038.964814	-1038.568595
277-Me$^+$_6	-1041.191796	-1040.795612	-1038.964755	-1038.568571
277-Me$^+$_7	-1041.191664	-1040.795538	-1038.964483	-1038.568357
277-Me$^+$_8	-1041.191094	-1040.795182	-1038.963937	-1038.568025
277-Me$^+$_9	-1041.190818	-1040.794572	-1038.964251	-1038.568005
277-Me$^+$_10	-1041.190968	-1040.794844	-1038.964081	-1038.567957
**278**				-958.652892
278_1	-960.956852	-960.655882	-958.953942	-958.652971
278_2	-960.957277	-960.656363	-958.953841	-958.652928
278_3	-960.956545	-960.655629	-958.953665	-958.652748
**278-Me$^+$**				-998.208276
278-Me$^+$_1	-1000.663736	-1000.320743	-998.551883	-998.208890
278-Me$^+$_2	-1000.661939	-1000.319027	-998.550650	-998.207738
278-Me$^+$_3	-1000.662595	-1000.319632	-998.550274	-998.207311
278-Me$^+$_4	-1000.662288	-1000.319281	-998.549875	-998.206869
278-Me$^+$_5	-1000.661117	-1000.318225	-998.548686	-998.205794
**279**				-807.866673
279_1	-809.833365	-809.536072	-808.164298	-807.867006
279_2	-809.831810	-809.534543	-808.162657	-807.865390
279_3	-809.831065	-809.533774	-808.161158	-807.863866
**279-Me$^+$**				-847.422407
279-Me$^+$_1	-849.53881	-849.19923	-847.76223	-847.422654
279-Me$^+$_2	-849.53602	-849.19671	-847.75910	-847.419786
279-Me$^+$_3	-849.53614	-849.19652	-847.75872	-847.419105
**280**				-886.186548
280_1	-888.432530	-888.075013	-886.545569	-886.188052
280_2	-888.431276	-888.073723	-886.544955	-886.187402
280_3	-888.431363	-888.073813	-886.544846	-886.187296
280_4	-888.431287	-888.073852	-886.544724	-886.187289
280_5	-888.430522	-888.072992	-886.544761	-886.187231
280_6	-888.429549	-888.071917	-886.544561	-886.186928
280_7	-888.430270	-888.072575	-886.544473	-886.186779
280_8	-888.431983	-888.074369	-886.544256	-886.186642

280_9	-888.430083	-888.072709	-886.544007	-886.186633
280_10	-888.429302	-888.071820	-886.544089	-886.186606
**280-Me$^+$**				-925.742540
280-Me$^+$_1	-928.137526	-927.738045	-926.143459	-925.743978
280-Me$^+$_2	-928.136505	-927.736924	-926.142874	-925.743293
280-Me$^+$_3	-928.136420	-927.736837	-926.142866	-925.743283
280-Me$^+$_4	-928.135407	-927.736313	-926.142321	-925.743227
280-Me$^+$_5	-928.137083	-927.737380	-926.142927	-925.743224
280-Me$^+$_6	-928.137001	-927.737401	-926.142591	-925.742992
280-Me$^+$_7	-928.137130	-927.737635	-926.142320	-925.742824
280-Me$^+$_8	-928.135413	-927.735929	-926.142274	-925.742789
280-Me$^+$_9	-928.136044	-927.736379	-926.142417	-925.742752
280-Me$^+$_10	-928.135310	-927.735764	-926.142255	-925.742709
**281**				-959.853823
281_1	-962.187805	-961.864384	-960.177552	-959.854132
281_2	-962.187915	-961.864689	-960.176801	-959.853575
282_3	-962.188213	-961.864705	-960.176987	-959.853479
**281-Me$^+$**				-999.410135
281-Me$^+$_1	-1001.894289	-1001.528905	-999.775780	-999.410395
281-Me$^+$_2	-1001.894443	-1001.528555	-999.775216	-999.409328
**282**				-935.517694
282_1	-937.682723	-937.424105	-935.776326	-935.517708
282_2	-937.682088	-937.423537	-935.776231	-935.517680
**282-Me$^+$**	-977.389785	-977.088973	-975.374882	-975.074070
**283**				-975.883223
283_1	-978.223871	-977.911542	-976.196020	-975.883691
283_2	-978.222431	-977.909866	-976.195738	-975.883173
283_3	-978.223219	-977.910912	-976.195434	-975.883127
283_4	-978.222155	-977.909775	-976.195114	-975.882735
283_5	-978.222000	-977.909643	-976.194562	-975.882205
**283-Me$^+$**				-1015.439743
283-Me$^+$_1	-1017.929499	-1017.574624	-1015.795162	-1015.440287
283-Me$^+$_2	-1017.929166	-1017.574551	-1015.794866	-1015.440252
283-Me$^+$_3	-1017.929343	-1017.574704	-1015.794526	-1015.439887
283-Me$^+$_4	-1017.929163	-1017.574525	-1015.794404	-1015.439766
283-Me$^+$_5	-1017.928808	-1017.573889	-1015.794670	-1015.439751
283-Me$^+$_6	-1017.928826	-1017.574194	-1015.794059	-1015.439427
283-Me$^+$_7	-1017.928449	-1017.573544	-1015.794288	-1015.439383
283-Me$^+$_8	-1017.928512	-1017.573813	-1015.794019	-1015.439320
283-Me$^+$_9	-1017.928493	-1017.573908	-1015.793901	-1015.439316
283-Me$^+$_10	-1017.928752	-1017.573966	-1015.793951	-1015.439165
**284**				-1147.725361
284_1	-1150.441221	-1150.115249	-1148.051391	-1147.725419
284_2	-1150.441309	-1150.115218	-1148.051386	-1147.725295
**284-Me$^+$**				-1187.282182
284-Me$^+$_1	-1190.150502	-1189.782483	-1187.650272	-1187.282252
284-Me$^+$_2	-1190.150331	-1189.782345	-1187.650085	-1187.282099
**285**				-1037.018000

285_1	-1039.588309	-1039.226223	-1037.380450	-1037.018364
285_2	-1039.587282	-1039.225103	-1037.379820	-1037.017641
285_3	-1039.585366	-1039.223294	-1037.378189	-1037.016118
**285-Me$^+$**				-1076.574948
285-Me$^+$_1	-1079.295052	-1078.890870	-1076.979439	-1076.575256
285-Me$^+$_2	-1079.294427	-1078.890161	-1076.978010	-1076.573744
285-Me$^+$_3	-1079.292320	-1078.888110	-1076.975735	-1076.571526
**286**				-997.846414
286_1	-1000.282998	-999.951177	-998.178646	-997.846826
286_2	-1000.283075	-999.951244	-998.178488	-997.846657
286_3	-1000.281207	-999.949317	-998.178336	-997.846446
286_4	-1000.283029	-999.951109	-998.178278	-997.846358
286_5	-1000.281275	-999.949315	-998.177700	-997.845740
286_6	-1000.281528	-999.949499	-998.177318	-997.845289
286_7	-1000.278132	-999.946135	-998.175851	-997.843854
286_8	-1000.278888	-999.946925	-998.174911	-997.842948
**286-Me$^+$**				-1037.403503
286-Me$^+$_1	-1039.989807	-1039.615762	-1037.778133	-1037.404088
286-Me$^+$_2	-1039.989801	-1039.615729	-1037.777351	-1037.403279
286-Me$^+$_3	-1039.988159	-1039.614223	-1037.775247	-1037.401310
286-Me$^+$_4	-1039.987552	-1039.613459	-1037.775246	-1037.401153
286-Me$^+$_5	-1039.987364	-1039.613388	-1037.775033	-1037.401057
286-Me$^+$_6	-1039.988262	-1039.614123	-1037.774907	-1037.400768
286-Me$^+$_7	-1039.988241	-1039.613951	-1037.774675	-1037.400385
**287**				-1358.454170
287_1	-1361.791184	-1361.352028	-1358.893443	-1358.454288
287_2	-1361.791210	-1361.351997	-1358.893224	-1358.454011
**287-Me$^+$**				-1398.011278
287-Me$^+$_1	-1401.500985	-1401.019836	-1398.492454	-1398.011306
287-Me$^+$_2	-1401.500825	-1401.019698	-1398.492375	-1398.011248
**288**	-1114.56354	-1114.21429	-1112.20192	-1111.852666
**288-Me$^+$**	-1154.27297	-1153.88174	-1151.80110	-1151.409871
**289**				-1076.169764
289_1	-1078.874483	-1078.482152	-1076.563038	-1076.170707
289_2	-1078.874374	-1078.482067	-1076.562681	-1076.170374
289_3	-1078.874086	-1078.481968	-1076.562322	-1076.170205
289_4	-1078.874040	-1078.481869	-1076.562020	-1076.169850
289_5	-1078.874099	-1078.481940	-1076.561807	-1076.169648
289_6	-1078.873429	-1078.481074	-1076.561951	-1076.169596
289_7	-1078.872329	-1078.480147	-1076.561772	-1076.169590
289_8	-1078.873014	-1078.480656	-1076.561723	-1076.169365
289_9	-1078.873899	-1078.481683	-1076.561516	-1076.169300
289_10	-1078.873781	-1078.481661	-1076.561275	-1076.169155
**289-Me$^+$**				-1115.727347
289-Me$^+$_1	-1118.582290	-1118.148036	-1116.162709	-1115.728455
289-Me$^+$_2	-1118.580956	-1118.146485	-1116.162113	-1115.727642
289-Me$^+$_3	-1118.581576	-1118.147316	-1116.161750	-1115.727491
289-Me$^+$_4	-1118.581529	-1118.147027	-1116.161977	-1115.727475
289-Me$^+$_5	-1118.581347	-1118.147086	-1116.161629	-1115.727369

289-Me$^+$_6	-1118.581386	-1118.146917	-1116.161341	-1115.726872
289-Me$^+$_7	-1118.581473	-1118.146938	-1116.161311	-1115.726776
289-Me$^+$_8	-1118.581272	-1118.146923	-1116.161046	-1115.726697
289-Me$^+$_9	-1118.580247	-1118.145814	-1116.160994	-1115.726562
289-Me$^+$_10	-1118.581192	-1118.146758	-1116.160813	-1115.726380
**290**				-974.686749
290_1	-976.992280	-976.702806	-974.976450	-974.686976
290_2	-976.987292	-976.698118	-974.973311	-974.684138
290_3	-976.986951	-976.697792	-974.972900	-974.683742
**290-Me$^+$**				-1014.244506
290-Me$^+$_1	-1016.700458	-1016.368757	-1014.576814	-1014.245113
290-Me$^+$_2	-1016.698227	-1016.367267	-1014.574010	-1014.243050
290-Me$^+$_3	-1016.698794	-1016.367157	-1014.574553	-1014.242916
290-Me$^+$_4	-1016.699149	-1016.367535	-1014.574525	-1014.242911
290-Me$^+$_5	-1016.698709	-1016.367138	-1014.574171	-1014.242600
**291**				-1053.051872
291_1	-1055.621452	-1055.271120	-1053.402548	-1053.052216
291_2	-1055.620003	-1055.269774	-1053.401501	-1053.051272
291_3	-1055.618302	-1055.267827	-1053.399865	-1053.049390
**291-Me$^+$**				-1092.609629
291-Me$^+$_1	-1095.328990	-1094.936114	-1093.002839	-1092.609962
291-Me$^+$_2	-1095.328570	-1094.936068	-1093.002317	-1092.609815
291-Me$^+$_3	-1095.328652	-1094.936082	-1093.002301	-1092.609731
291-Me$^+$_4	-1095.328689	-1094.936005	-1093.002163	-1092.609478
291-Me$^+$_5	-1095.327363	-1094.934488	-1093.001682	-1092.608807
291-Me$^+$_6	-1095.327719	-1094.935060	-1093.001173	-1092.608514
**292**				-1115.325288
292_1	-1118.165046	-1117.742518	-1115.749097	-1115.326570
292_2	-1118.164803	-1117.742293	-1115.748746	-1115.326236
292_3	-1118.164544	-1117.741998	-1115.748425	-1115.325878
292_4	-1118.164721	-1117.742048	-1115.748527	-1115.325854
292_5	-1118.163922	-1117.741257	-1115.748014	-1115.325349
292_6	-1118.163370	-1117.740828	-1115.747618	-1115.325076
292_7	-1118.164019	-1117.741435	-1115.747635	-1115.325052
292_8	-1118.162878	-1117.740435	-1115.747400	-1115.324957
292_9	-1118.164099	-1117.741560	-1115.747481	-1115.324942
292_10	-1118.162988	-1117.740480	-1115.747435	-1115.324927
**292-Me$^+$**				-1154.883337
292-Me$^+$_1	-1157.872872	-1157.408261	-1155.349171	-1154.884560
292-Me$^+$_2	-1157.872566	-1157.408117	-1155.348500	-1154.884052
292-Me$^+$_3	-1157.872376	-1157.407906	-1155.348313	-1154.883842
292-Me$^+$_4	-1157.872120	-1157.407496	-1155.348411	-1154.883787
292-Me$^+$_5	-1157.872459	-1157.407839	-1155.348338	-1154.883718
292-Me$^+$_6	-1157.871795	-1157.407161	-1155.347978	-1154.883343
292-Me$^+$_7	-1157.872199	-1157.407566	-1155.347885	-1154.883253
292-Me$^+$_8	-1157.872017	-1157.407370	-1155.347812	-1154.883164
292-Me$^+$_9	-1157.870492	-1157.405943	-1155.347519	-1154.882970
292-Me$^+$_10	-1157.872009	-1157.407404	-1155.347537	-1154.882932
**293**				-1358.454816

293_1	-1361.791178	-1361.351917	-1358.894489	-1358.455228
293_2	-1361.790945	-1361.351692	-1358.894040	-1358.454787
293_3	-1361.789249	-1361.350216	-1358.893423	-1358.454390
293_4	-1361.789552	-1361.350493	-1358.892698	-1358.453639
**293-Me$^+$**				-1398.013377
293-Me$^+$_1	-1401.499840	-1401.018805	-1398.494450	-1398.013415
293-Me$^+$_2	-1401.501007	-1401.019852	-1398.494528	-1398.013373
293-Me$^+$_3	-1401.495836	-1401.014848	-1398.489210	-1398.008222
293-Me$^+$_4	-1401.495535	-1401.014540	-1398.488643	-1398.007648
**294**				-1191.356554
294_1	-1194.369712	-1193.933094	-1191.793339	-1191.356721
294_2	-1194.369666	-1193.933506	-1191.792839	-1191.356679
294_3	-1194.369752	-1193.933180	-1191.792306	-1191.355735
**294-Me$^+$**				-1230.915874
294-Me$^+$_1	-1234.079889	-1233.600807	-1231.395173	-1230.916091
294-Me$^+$_2	-1234.079739	-1233.600982	-1231.394578	-1230.915821
294-Me$^+$_3	-1234.079741	-1233.600730	-1231.394574	-1230.915563
**295**	-1153.86536	-1153.48674	-1151.39917	-1151.020539
**295-Me$^+$**	-1193.577178	-1193.156631	-1191.000593	-1190.580046
**296**				-1013.880489
296_1	-1016.317200	-1015.996926	-1014.201214	-1013.880940
296_2	-1016.317395	-1015.997004	-1014.200867	-1013.880475
296_3	-1016.317514	-1015.997298	-1014.200495	-1013.880279
296_4	-1016.315211	-1015.994895	-1014.200533	-1013.880217
296_5	-1016.312057	-1015.991906	-1014.198142	-1013.877992
296_6	-1016.312129	-1015.991854	-1014.198151	-1013.877876
**296-Me$^+$**				-1053.440662
296-Me$^+$_1	-1056.027301	-1055.664785	-1053.803509	-1053.440993
296-Me$^+$_2	-1056.027362	-1055.664890	-1053.803414	-1053.440942
296-Me$^+$_3	-1056.027119	-1055.664348	-1053.803063	-1053.440293
296-Me$^+$_4	-1056.026918	-1055.664152	-1053.802855	-1053.440089
296-Me$^+$_5	-1056.025453	-1055.663090	-1053.801346	-1053.438982
**297**				-1167.026583
297_1	-1169.875117	-1169.506795	-1167.395010	-1167.026688
297_2	-1169.875052	-1169.506757	-1167.394911	-1167.026617
297_3	-1169.874914	-1169.506658	-1167.394656	-1167.026400
**297-Me$^+$**	-1209.591455	-1209.180841	-1207.000432	-1206.589818
**298**				-1376.142423
298_1	-1379.403368	-1379.006497	-1376.539446	-1376.142574
298_2	-1379.403378	-1379.006406	-1376.539530	-1376.142557
298_3	-1379.403408	-1379.006460	-1376.539468	-1376.142520
298_4	-1379.403386	-1379.006524	-1376.539360	-1376.142497
298_5	-1379.403502	-1379.006618	-1376.539348	-1376.142464
298_6	-1379.403409	-1379.006481	-1376.539381	-1376.142453
298_7	-1379.403429	-1379.006643	-1376.539162	-1376.142376
298_8	-1379.403371	-1379.006555	-1376.539186	-1376.142370
298_9	-1379.403382	-1379.006546	-1376.539186	-1376.142350
298_10	-1379.403416	-1379.006513	-1376.539173	-1376.142270
**298-Me$^+$**				-1415.707295
298-Me$^+$_1	-1419.122130	-1418.683158	-1416.146484	-1415.707512

298-Me$^+$_2	-1419.122205	-1418.683155	-1416.146511	-1415.707461
298-Me$^+$_3	-1419.121974	-1418.682914	-1416.146302	-1415.707242
298-Me$^+$_4	-1419.121735	-1418.682791	-1416.146082	-1415.707138
298-Me$^+$_5	-1419.121543	-1418.682512	-1416.145847	-1415.706816
**299**				-1300.535398
299_1	-1303.789687	-1303.343133	-1300.982208	-1300.535654
299_2	-1303.789511	-1303.342934	-1300.982148	-1300.535571
299_3	-1303.789593	-1303.343085	-1300.982036	-1300.535528
299_4	-1303.789639	-1303.343134	-1300.982027	-1300.535522
299_5	-1303.789549	-1303.343059	-1300.981889	-1300.535399
299_6	-1303.788003	-1303.342008	-1300.981347	-1300.535351
299_7	-1303.789486	-1303.343027	-1300.981769	-1300.535310
299_8	-1303.789190	-1303.342929	-1300.981570	-1300.535308
299_9	-1303.789597	-1303.342988	-1300.981784	-1300.535175
299_10	-1303.789609	-1303.343082	-1300.981507	-1300.534980
**299-Me$^+$**	-1343.517145	-1343.028291	-1340.597545	-1340.108691
**300**				-1434.045160
300_1	-1437.702274	-1437.178430	-1434.569570	-1434.045726
300_2	-1437.703345	-1437.179556	-1434.569041	-1434.045252
300_3	-1437.703268	-1437.179348	-1434.569130	-1434.045210
300_4	-1437.703527	-1437.179576	-1434.569062	-1434.045111
300_5	-1437.703078	-1437.179003	-1434.569186	-1434.045111
300_6	-1437.703522	-1437.179320	-1434.569197	-1434.044995
300_7	-1437.703384	-1437.179351	-1434.569027	-1434.044994
300_8	-1437.703539	-1437.179450	-1434.569080	-1434.044990
300_9	-1437.703372	-1437.179357	-1434.568873	-1434.044858
300_10	-1437.703484	-1437.179209	-1434.569126	-1434.044850
**300-Me$^+$**	-1477.441483	-1476.874839	-1474.193401	-1473.626757
**301**	-1110.591829	-1110.310024	-1108.328816	-1108.047012
**301-Me$^+$**	-1150.416721	-1150.092460	-1148.039919	-1147.715659
**302**				-1256.867491
302_1	-1259.594005	-1259.330242	-1257.131512	-1256.867749
302_2	-1259.595544	-1259.331076	-1257.131200	-1256.866732
**302-Me$^+$**	-1299.651895	-1299.344552	-1297.059457	-1296.752114
**303**	-640.80902	-640.79546	-639.899396	-639.885832
**303-Me$^+$**	-680.41817	-680.36310	-679.39342	-679.338355
**304**	-537.154551	-537.019731	-536.173303	-536.038483
**304-Me$^+$**	-576.843873	-576.667442	-575.754277	-575.577846
**305**	-1190.926607	-1190.548768	-1188.432654	-1188.054815
**305-Me$^+$**	-1230.620488	-1230.201184	-1228.016255	-1227.596950
**306**	-615.760012	-615.563078	-614.574756	-614.377822
**306-Me$^+$**	-655.453918	-655.215172	-654.161473	-653.922727
**307**	-537.152192	-537.016891	-536.170342	-536.035041
**307-Me$^+$**	-576.8477738	-576.670837	-575.7572936	-575.580357
**308**	-538.383378	-538.223939	-537.384337	-537.224898
**308-Me$^+$**	-578.078258	-577.877210	-576.972147	-576.771099
**309**				-1036.997177
309_1	-1039.545644	-1039.186463	-1037.356361	-1036.997180

309_2	-1039.539604	-1039.180331	-1037.349044	-1036.989771
**309-Me$^+$**	-1079.246809	-1078.845968	-1076.948806	-1076.547966
**310**	-731.250368	-731.013907	-729.785867	-729.549406
**310-Me$^+$**	-770.950712	-770.672225	-769.378789	-769.100302
**311**				-631.573406
311_1	-633.000293	-632.792692	-631.781326	-631.573726
311_2	-633.000027	-632.792343	-631.780768	-631.573085
311_3	-632.995109	-632.787416	-631.778953	-631.571261
**311-Me$^+$**				-671.125064
311-Me$^+$_1	-672.700271	-672.450538	-671.374980	-671.125247
311-Me$^+$_2	-672.699929	-672.450082	-671.374999	-671.125152
311-Me$^+$_3	-672.699591	-672.449633	-671.374406	-671.124448
**312**				-804.382367
312_1	-806.232164	-806.005150	-804.609743	-804.382729
312_2	-806.231360	-806.004349	-804.609094	-804.382084
312_3	-806.231066	-806.004074	-804.608748	-804.381756
**312-Me$^+$**	-845.932988	-845.664347	-844.202728	-843.934087
**313**				-576.390286
313_1	-577.683936	-577.494343	-576.580136	-576.390543
313_2	-577.683137	-577.493444	-576.579239	-576.389546
**313-Me$^+$**	-617.383371	-617.151985	-616.173516	-615.942130
**314**				-695.038975
314_1	-696.765582	-696.465711	-695.339413	-695.039542
314_2	-696.764935	-696.464909	-695.338654	-695.038628
314_3	-696.763979	-696.464186	-695.337573	-695.037780
314_4	-696.763085	-696.462955	-695.336740	-695.036610
314_5	-696.761988	-696.461914	-695.335505	-695.035431
314_6	-696.761746	-696.461647	-695.335417	-695.035318
**314-Me$^+$**				-734.590927
314-Me$^+$_1	-736.466391	-736.124420	-734.933432	-734.591461
314-Me$^+$_2	-736.464216	-736.122156	-734.931510	-734.589450
314-Me$^+$_3	-736.464081	-736.121742	-734.931351	-734.589012
314-Me$^+$_4	-736.463781	-736.121403	-734.931047	-734.588668
314-Me$^+$_5	-736.463305	-736.121048	-734.930280	-734.588023
**315**				-1008.324439
315_1	-1011.108481	-1010.569706	-1008.864231	-1008.325457
315_2	-1011.107195	-1010.568146	-1008.862588	-1008.323539
315_3	-1011.106828	-1010.568496	-1008.861664	-1008.323331
315_4	-1011.106852	-1010.567818	-1008.862214	-1008.323180
315_5	-1011.107172	-1010.568946	-1008.861325	-1008.323099
315_6	-1011.106216	-1010.567614	-1008.861105	-1008.322502
315_7	-1011.106629	-1010.568349	-1008.860779	-1008.322499
315_8	-1011.107039	-1010.568033	-1008.861502	-1008.322496
315_9	-1011.105975	-1010.567540	-1008.859619	-1008.321183
315_10	-1011.105409	-1010.566642	-1008.859947	-1008.321179
**315-Me$^+$**				-1047.877591
315-Me$^+$_1	-1050.810644	-1050.229732	-1048.459213	-1047.878300
315-Me$^+$_2	-1050.809637	-1050.229164	-1048.458581	-1047.878108

315-Me[+]_3	-1050.810252	-1050.229075	-1048.458996	-1047.877819
315-Me[+]_4	-1050.810443	-1050.229353	-1048.458775	-1047.877684
315-Me[+]_5	-1050.809232	-1050.228808	-1048.457073	-1047.876648
315-Me[+]_6	-1050.808585	-1050.228132	-1048.457072	-1047.876619
315-Me[+]_7	-1050.809453	-1050.228756	-1048.456993	-1047.876296
315-Me[+]_8	-1050.807698	-1050.226254	-1048.457668	-1047.876223
315-Me[+]_9	-1050.809075	-1050.228281	-1048.456861	-1047.876067
315-Me[+]_10	-1050.808245	-1050.227313	-1048.456421	-1047.875489
**316[a]**				-1248.817662
316[a]_1	-1252.024828	-1251.528552	-1249.314207	-1248.817931
316[a]_2	-1252.022231	-1251.526230	-1249.311826	-1248.815825
316[a]_3	-1252.020194	-1251.523971	-1249.309504	-1248.813281
316[a]_4	-1252.020523	-1251.524494	-1249.308901	-1248.812873
316[a]_5	-1252.020274	-1251.523981	-1249.308455	-1248.812162
316[a]_6	-1252.019302	-1251.522886	-1249.307165	-1248.810749
316[a]_7	-1252.018808	-1251.522932	-1249.305855	-1248.809979
316[a]_8	-1252.012270	-1251.515854	-1249.300921	-1248.804505
**316[a]-Me[+]**				-1288.371039
316[a]-Me[+]_1	-1291.728781	-1291.190260	-1288.909837	-1288.371316
316[a]-Me[+]_2	-1291.725532	-1291.186154	-1288.910647	-1288.371269
316[a]-Me[+]_3	-1291.726811	-1291.188559	-1288.909095	-1288.370843
316[a]-Me[+]_4	-1291.725167	-1291.186790	-1288.907960	-1288.369583
316[a]-Me[+]_5	-1291.724655	-1291.186160	-1288.906406	-1288.367911
316[a]-Me[+]_6	-1291.723348	-1291.184589	-1288.906193	-1288.367434
316[a]-Me[+]_7	-1291.717558	-1291.179387	-1288.898425	-1288.360255
**316[b]**				-1248.819138
316[b]_1	-1252.027087	-1251.530889	-1249.315520	-1248.819322
316[b]_2	-1252.022562	-1251.526895	-1249.311943	-1248.816276
316[b]_3	-1252.022114	-1251.526258	-1249.311392	-1248.815536
316[b]_4	-1252.020381	-1251.523976	-1249.308756	-1248.812351
316[b]_5	-1252.018877	-1251.522316	-1249.307067	-1248.810506
316[b]_6	-1252.017360	-1251.520819	-1249.306582	-1248.810041
316[b]_7	-1252.005718	-1251.509452	-1249.294125	-1248.797859
316[b]_8	-1252.005243	-1251.508887	-1249.293315	-1248.796959
**316[b]-Me[+]**				-1288.380586
316[b]-Me[+]_1	-1291.734557	-1291.195702	-1288.919449	-1288.380594
316[b]-Me[+]_2	-1291.734558	-1291.195696	-1288.919441	-1288.380579
316[b]-Me[+]_3	-1291.728966	-1291.190884	-1288.909995	-1288.371913
316[b]-Me[+]_4	-1291.727849	-1291.189409	-1288.908330	-1288.369890
316[b]-Me[+]_5	-1291.725449	-1291.186512	-1288.905414	-1288.366478
316[b]-Me[+]_6	-1291.710568	-1291.171518	-1288.892786	-1288.353736
316[b]-Me[+]_7	-1291.710568	-1291.171518	-1288.892783	-1288.353733
316[b]-Me[+]_8	-1291.708227	-1291.169332	-1288.890636	-1288.351741
**316[c]-Me[+]**				-1288.357812
316[c]-Me[+]	-1291.716994	-1291.176087	-1288.898728	-1288.357822
316[c]-Me[+]_1	-1291.708348	-1291.167545	-1288.892433	-1288.351630
316[c]-Me[+]_2	-1291.705083	-1291.164392	-1288.885615	-1288.344924
316[c]-Me[+]_3	-1291.700807	-1291.159693	-1288.881613	-1288.340498

316c-Me+_4	-1291.682159	-1291.140915	-1288.863869	-1288.322626
**317a**				-1248.831509
317a_1	-1252.040646	-1251.544494	-1249.327996	-1248.831844
317a_2	-1252.035733	-1251.539723	-1249.325600	-1248.829590
317a_3	-1252.039019	-1251.542564	-1249.325809	-1248.829355
317a_4	-1252.034656	-1251.538717	-1249.321104	-1248.825165
**317a-Me+**				-1288.385140
317a-Me+_1	-1291.744499	-1291.206580	-1288.923228	-1288.385309
317a-Me+_2	-1291.744295	-1291.206393	-1288.922774	-1288.384872
**317b**				-1248.835489
317b_1	-1252.045199	-1251.548897	-1249.331805	-1248.835502
317b_2	-1252.039542	-1251.543407	-1249.325600	-1248.829466
317b_3	-1252.037006	-1251.540854	-1249.324624	-1248.828472
317b_4	-1252.032578	-1251.536349	-1249.319323	-1248.823094
**317b-Me+**				-1288.388438
317b-Me+_1	-1291.747962	-1291.210253	-1288.926170	-1288.388461
317b-Me+_2	-1291.741981	-1291.204054	-1288.921288	-1288.383361
**317c-Me+**				-1288.371877
317c-Me+_1	-1291.733531	-1291.192548	-1288.912870	-1288.371886
317c-Me+_2	-1291.727813	-1291.187020	-1288.906671	-1288.365878
317c-Me+_3	-1291.718765	-1291.177603	-1288.898199	-1288.357037
317c-Me+_4	-1291.714826	-1291.174208	-1288.893876	-1288.353259
**318**				-692.637532
318_1	-694.285044	-694.032573	-692.890680	-692.638209
318_2	-694.283448	-694.030860	-692.889809	-692.637221
318_3	-694.283871	-694.031416	-692.889585	-692.637130
318_4	-694.282158	-694.029483	-692.888395	-692.635721
318_5	-694.282327	-694.029536	-692.888461	-692.635670
318_6	-694.281885	-694.029242	-692.888024	-692.635382
318_7	-694.281523	-694.028886	-692.887279	-692.634642
**318-Me+**				-732.193379
318-Me+_1	-733.989179	-733.694834	-732.487983	-732.193638
318-Me+_2	-733.987761	-733.693093	-732.487275	-732.192606
**319**				-708.734680
319_1	-710.393437	-710.148162	-708.980170	-708.734895
319_2	-710.393415	-710.148044	-708.980106	-708.734735
319_3	-710.388774	-710.143179	-708.979610	-708.734015
**319-Me+**				-748.291511
319-Me+_1	-750.098726	-749.811231	-748.579567	-748.292072
319-Me+_2	-750.098799	-749.811192	-748.579493	-748.291886
319-Me+_3	-750.098483	-749.810993	-748.579135	-748.291646
319-Me+_4	-750.098287	-749.810724	-748.578933	-748.291370
319-Me+_5	-750.098275	-749.810813	-748.578619	-748.291158
319-Me+_6	-750.098187	-749.810728	-748.578602	-748.291143
319-Me+_7	-750.098354	-749.810784	-748.578701	-748.291130
319-Me+_8	-750.098321	-749.810782	-748.578631	-748.291093
319-Me+_9	-750.098156	-749.810679	-748.578497	-748.291020
319-Me+_10	-750.098258	-749.810774	-748.578476	-748.290992

**320**				-670.738952
320_1	-672.300969	-672.063265	-670.977029	-670.739324
320_2	-672.296090	-672.058166	-670.976236	-670.738312
320_3	-672.295660	-672.057792	-670.974569	-670.736702
**320-Me$^+$**				-710.296296
320-Me$^+$_1	-712.004598	-711.724419	-710.576705	-710.296526
320-Me$^+$_2	-712.004678	-711.724537	-710.576640	-710.296499
320-Me$^+$_3	-712.005050	-711.724951	-710.576084	-710.295985
320-Me$^+$_4	-712.004187	-711.723897	-710.575234	-710.294944
**321**				-1003.532041
321_1	-1006.158607	-1005.715297	-1003.976452	-1003.533142
321_2	-1006.157436	-1005.713681	-1003.975661	-1003.531906
321_3	-1006.156831	-1005.713789	-1003.973835	-1003.530793
321_4	-1006.156237	-1005.712454	-1003.974572	-1003.530789
321_5	-1006.156234	-1005.712626	-1003.974229	-1003.530621
321_6	-1006.156349	-1005.713004	-1003.973890	-1003.530545
321_7	-1006.155569	-1005.712258	-1003.973458	-1003.530147
321_8	-1006.155434	-1005.711944	-1003.973165	-1003.529676
321_9	-1006.155076	-1005.711822	-1003.972851	-1003.529597
321_10	-1006.154717	-1005.710983	-1003.973194	-1003.529460
**321-Me$^+$**				-1043.089538
321-Me$^+$_1	-1045.865751	-1045.380201	-1043.576066	-1043.090516
321-Me$^+$_2	-1045.864004	-1045.378300	-1043.575103	-1043.089400
321-Me$^+$_3	-1045.863348	-1045.377858	-1043.573306	-1043.087816
321-Me$^+$_4	-1045.860337	-1045.375669	-1043.572261	-1043.087592
321-Me$^+$_5	-1045.860626	-1045.375301	-1043.572820	-1043.087496
321-Me$^+$_6	-1045.863044	-1045.377732	-1043.572699	-1043.087386
321-Me$^+$_7	-1045.862520	-1045.376530	-1043.573354	-1043.087364
321-Me$^+$_8	-1045.861452	-1045.375680	-1043.573027	-1043.087255
321-Me$^+$_9	-1045.862076	-1045.376766	-1043.572222	-1043.086913
321-Me$^+$_10	-1045.861947	-1045.376679	-1043.572140	-1043.086872
**322**				-963.403404
322_1	-965.855789	-965.458553	-963.800758	-963.403522
322_2	-965.855885	-965.458382	-963.800749	-963.403246
**322-Me$^+$**				-1002.962126
322-Me$^+$_1	-1005.563760	-1005.124234	-1003.401760	-1002.962234
322-Me$^+$_2	-1005.563511	-1005.123806	-1003.401690	-1002.961985
**323**				-1117.752878
323_1	-1120.648472	-1120.177136	-1118.224258	-1117.752922
323_2	-1120.648477	-1120.177248	-1118.224059	-1117.752830
**323-Me$^+$**	-1160.356782	-1159.843296	-1157.826035	-1157.312549
**324**				-747.900952
324_1	-749.689884	-749.414222	-748.177148	-747.901487
324_2	-749.689849	-749.414331	-748.176667	-747.901149
324_3	-749.694038	-749.418460	-748.176011	-747.900434
324_4	-749.694057	-749.418463	-748.176006	-747.900413
324_5	-749.689136	-749.413491	-748.175009	-747.899363
324_6	-749.691767	-749.416126	-748.174752	-747.899111

**324-Me$^+$**				-787.463460
324-Me$^+$_1	-789.403837	-789.085960	-787.781904	-787.464027
324-Me$^+$_2	-789.403320	-789.085483	-787.780530	-787.462693
324-Me$^+$_3	-789.403258	-789.085373	-787.780432	-787.462548
324-Me$^+$_4	-789.403216	-789.085427	-787.780197	-787.462409
**325**				-1492.261365
325_1	-1495.874368	-1495.427604	-1492.708450	-1492.261685
325_2	-1495.870260	-1495.423915	-1492.705145	-1492.258801
325_3	-1495.869723	-1495.423573	-1492.704851	-1492.258701
325_4	-1495.870200	-1495.423815	-1492.704570	-1492.258185
**325-Me$^+$**				-1531.824964
325-Me$^+$_1	-1535.584680	-1535.095750	-1532.314445	-1531.825515
325-Me$^+$_2	-1535.583240	-1535.094462	-1532.312742	-1531.823964
325-Me$^+$_3	-1535.584274	-1535.095398	-1532.312507	-1531.823631
325-Me$^+$_4	-1535.588268	-1535.099424	-1532.312414	-1531.823570
**326**				-1275.529140
326_1	-1279.014012	-1278.398574	-1276.145177	-1275.529740
326_2	-1279.013065	-1278.397618	-1276.145079	-1275.529632
326_3	-1279.012870	-1278.397142	-1276.144761	-1275.529033
326_4	-1279.012890	-1278.397066	-1276.144552	-1275.528728
326_5	-1279.011875	-1278.396067	-1276.144045	-1275.528236
326_6	-1279.011922	-1278.395976	-1276.143513	-1275.527567
326_7	-1279.010776	-1278.394736	-1276.143043	-1275.527003
326_8	-1279.010480	-1278.394619	-1276.142836	-1275.526975
326_9	-1279.009784	-1278.393719	-1276.142623	-1275.526558
326_10	-1279.010478	-1278.394577	-1276.142456	-1275.526556
**326-Me$^+$**				-1315.098304
326-Me$^+$_1	-1318.731817	-1318.073559	-1315.757400	-1315.099142
326-Me$^+$_2	-1318.730638	-1318.072248	-1315.756880	-1315.098490
326-Me$^+$_3	-1318.730738	-1318.072207	-1315.756736	-1315.098205
326-Me$^+$_4	-1318.730590	-1318.072114	-1315.756309	-1315.097833
326-Me$^+$_5	-1318.729872	-1318.071447	-1315.755681	-1315.097256
326-Me$^+$_6	-1318.729626	-1318.070918	-1315.755733	-1315.097026
326-Me$^+$_7	-1318.729517	-1318.070903	-1315.755357	-1315.096743
326-Me$^+$_8	-1318.728785	-1318.070226	-1315.754948	-1315.096390
326-Me$^+$_9	-1318.728489	-1318.070240	-1315.754546	-1315.096297
326-Me$^+$_10	-1318.728951	-1318.070264	-1315.754871	-1315.096183
**327**	-1470.708484	-1470.034396	-1467.406898	-1466.732811
**327-Me$^+$**				-1506.302323
327-Me$^+$_1	-1510.426903	-1509.710633	-1507.018682	-1506.302412
327-Me$^+$_2	-1510.427077	-1509.710567	-1507.018723	-1506.302213
**328**				-1317.339430
328_1	-1320.145536	-1319.822671	-1317.662399	-1317.339535
328_2	-1320.141675	-1319.818609	-1317.659407	-1317.336340
**328-Me$^+$**	-1359.880458	-1359.514973	-1357.289341	-1356.923856
**329**	-1888.515548	-1888.128136	-1885.376896	-1884.989484
**329-Me$^+$**	-1928.307367	-1927.876246	-1925.060555	-1924.629435
**330**	see reference 18			

**330-Me$^+$**	see reference 18
**331**	see reference 18
**331-Me$^+$**	see reference 18
**332**	see reference 18
**332-Me$^+$**	see reference 18
**333**	see reference 18
**333-Me$^+$**	see reference 18
**334**	see reference 18
**334-Me$^+$**	see reference 18
**335**	see reference 18
**335-Me$^+$**	see reference 18
**336**	see reference 18
**336-Me$^+$**	see reference 18
**337**	see reference 18
**337-Me$^+$**	see reference 18
**338**	see reference 18
**338-Me$^+$**	see reference 18
**339**	see reference 18
**339-Me$^+$**	see reference 18
**340**	see reference 18
**340-Me$^+$**	see reference 18
**341**	see reference 18
**341-Me$^+$**	see reference 18
**342**	see reference 18
**342-Me$^+$**	see reference 18

[a] (*S*) configuration, phosphorus; [b] (*R*) configuration, phosphorus; [c] (*S*) configuration, nitrogen.

COMPUTATIONAL METHODS (BHCA, TCA)

In analogy to methyl cation affinities the benzhydryl and trityl cation affinities of Lewis bases have been calculated as the reaction enthalpy at 298.15 K and 1 atm pressure for the detachment reaction shown in equations (S2)/(S3). This is in analogy to the mass spectrometric definition of proton affinities.

$$LB^+\text{-}CHPh_2 \;\text{-->}\; LB + {}^+CHPh_2 \qquad (S2)$$

$$LB^+\text{-}CPh_3 \;\text{-->}\; LB + {}^+CPh_3 \qquad (S3)$$

The geometries of all species in equations (S2)/(S3) have been optimized at the B98/6-31G(d) level of theory. The conformational space of flexible Lewis bases and the corresponding cations have been searched using the MM3* force field and the systematic search routine implemented in MACROMODEL 9.7 [48]. All stationary points located at force field level have then been reoptimized at B98/6-31G(d) level as described before. Thermochemical corrections to 298.15 K have been calculated for all minima from unscaled vibrational frequencies obtained at this same level. The thermochemical corrections have been combined with single point energies calculated at the MP2(FC)/6-31+G(2d,p)//B98/6-31G(d) level to yield enthalpies H_{298} at 298.15 K. For each case the BSSE-corrected interaction energy E_{INT} of the best conformations (Lewis base and cationic adduct) was calculated as described below. All quantum mechanical calculations have been performed with

Gaussian03 [49].

In contrast to MCA calculations the BSSE cannot be neglected for BHCA and TCA calculations. In the reaction of two reactants A and B (equation (S4)) the interaction energy E_{INT}' is defined as in equation (S5)

$$A + B \dashrightarrow AB \qquad (S4)$$

$$E_{INT}' = E(AB) - E(A) - E(B) \qquad (S5)$$

Due to the BSSE the equation has to be changed slightly:

$$E_{INT}^* = E(AB)_c^{AB} - E(A)_r^{AB} - E(B)_r^{AB} \qquad (S6)$$

The subscripts c and r imply complex and reactant, the superscript AB represents the basis sets of the complex AB. As the energy of the reactants in the geometry of the equilibrium in the basis sets of the complex is not obvious, it is possible to extend and clarify the definition of the interaction energy through separate consideration of the deformation energies.

$$E_{INT} = E(AB)_c^{AB} - E(A)_c^{AB} - E(B)_c^{AB} + E_{DEF} \qquad (S7)$$

$$E_{DEF} = E(A)_c^{A} - E(A)_r^{A} + E(B)_c^{B} - E(B)_r^{B} \qquad (S8)$$

The interaction energy depends not any longer on the equilibrium geometry of the single reactants, but only on the complex geometry. For this the deformation energy E_{DEF} was adopted, which describes the difference of the geometries of the reactants comparing the equilibrium and the complex geometry. The calculations of the deformation energy are carried out in the basis sets of the reactants. Finally the thermal correction of the non-BSSE-corrected system was used to produce the BSSE-corrected 'H_{298}' from the interaction energy E_{INT} (equation (S7)). The thermal corrections for the benzhydryl- as well the trityl-cation and the Lewis bases can be extracted from Table S1, and the thermal corrections of the adducts can be found in Table S3.

Using the reaction of triphenylphosphane (**89**) with benzhydryl cation as an example, the following energy components are obtained:

non-BSSE corrected interaction energy: −292.33 kJ/mol
deformation energy (cation): +176.18 kJ/mol
deformation energy (Lewis base): +31.41 kJ/mol
sum of deformation energies: +207.59 kJ/mol
interaction energy in full basis: −451.40 kJ/mol
BSSE-corrected interaction energy: −243.81 kJ/mol

The combination with thermal corrections yields the BSSE-corrected interaction enthalpy of −235.0 kJ/mol.

Table S2. Total energies of the complex and the seperate reactants for each case in the geometry of the complex and in the full basis sets, total energies of the reactants in the seperate basis sets in the equilibrium geometry as well as in the geometry of the complex (cp. equations (S7)/(S8), LB = Lewis Base, BH = benzhydrl, TT = trityl); ordered by the sequence of Tables 9 and 10.

	**343**	**344**	**251 (N)**	**345**
$E(complex)_c^{AB}$	-842.931747	-556.748426	-1515.760683	-902.592591

$E(LB)_c^{AB}$	-342.591631	-56.412781	-1015.361850	-402.225777
$E(BH)_c^{AB}$	-500.244612	-500.250724	-500.229519	-500.239884
$E(LB)_c^{A}$	-342.583793	-56.407642	-1015.350245	-402.218042
$E(LB)_r^{A}$	-342.594262	-56.408191	-1015.390461	-402.222312
$E(BH)_c^{B}$	-500.241684	-500.246917	-500.220205	-500.232200
$E(BH)_r^{B}$	-500.293523	-500.293523	-500.293523	-500.293523
	**45**	**20**	**346**	**1**
$E(complex)_c^{AB}$	-791.899339	-674.320470	-881.559103	-747.960559
$E(LB)_c^{AB}$	-291.521529	-173.952281	-381.182417	-247.594057
$E(BH)_c^{AB}$	-500.230251	-500.240129	-500.224471	-500.237197
$E(LB)_c^{A}$	-291.512100	-173.944626	-381.174104	-247.587435
$E(LB)_r^{A}$	-291.526262	-173.948666	-381.185703	-247.589439
$E(BH)_c^{B}$	-500.222475	-500.233287	-500.216966	-500.230890
$E(BH)_r^{B}$	-500.293523	-500.293523	-500.293523	-500.293523
	**347**	**44**	**14**	**53**
$E(complex)_c^{AB}$	-1287.769727	-844.724435	-787.163095	-828.728177
$E(LB)_c^{AB}$	-787.397345	-344.350736	-286.791707	-328.347677
$E(BH)_c^{AB}$	-500.238695	-500.238680	-500.236650	-500.237337
$E(LB)_c^{A}$	-787.388957	-344.342680	-286.785106	-328.339584
$E(LB)_r^{A}$	-787.392562	-344.346163	-286.787285	-328.343305
$E(BH)_c^{B}$	-500.230971	-500.231192	-500.230171	-500.229751
$E(BH)_r^{B}$	-500.293523	-500.293523	-500.293523	-500.293523
	**24**	**252**	**348**	**254**
$E(complex)_c^{AB}$	-765.184519	-1759.435124	-881.563649	-2911.337791
$E(LB)_c^{AB}$	-264.805697	-1259.020326	-381.180067	-2410.942361
$E(BH)_c^{AB}$	-500.234915	-500.238950	-500.235422	-500.235428
$E(LB)_c^{A}$	-264.799041	-1259.008422	-381.172879	-2410.932009
$E(LB)_r^{A}$	-264.803119	-1259.045037	-391.176150	-2410.943810
$E(BH)_c^{B}$	-500.228610	-500.230603	-500.228703	-500.227584
$E(BH)_r^{B}$	-500.293523	-500.293523	-500.293523	-500.293523
	**255**	**54**	**70**	**56**
$E(complex)_c^{AB}$	-1303.400234	-881.572063	-960.583854	-958.779213
$E(LB)_c^{AB}$	-802.976747	-381.182550	-460.186092	-458.386428
$E(BH)_c^{AB}$	-500.231562	-500.232866	-500.236246	-500.232034
$E(LB)_c^{A}$	-802.965096	-381.175847	-460.176466	-458.379754
$E(LB)_r^{A}$	-803.005409	-381.179961	-460.190902	-458.383909
$E(BH)_c^{B}$	-500.234460	-500.226394	-500.231339	-500.225449
$E(BH)_r^{B}$	-500.293523	-500.293523	-500.293523	-500.293523
	**117**	**349**	**256**	**279**
$E(complex)_c^{AB}$	-1195.738067	-1108.388562	-1151.794274	-1308.567741
$E(LB)_c^{AB}$	-695.333489	-607.987681	-651.390861	-808.156619
$E(BH)_c^{AB}$	-500.229820	-500.231361	-500.237209	-500.230688
$E(LB)_c^{A}$	-695.322121	-607.980833	-651.380329	-808.145131
$E(LB)_r^{A}$	-695.336484	-607.990824	-651.393617	-808.164298
$E(BH)_c^{B}$	-500.222764	-500.224573	-500.231280	-500.223553
$E(BH)_r^{B}$	-500.293523	-500.293523	-500.293523	-500.293523
	**98**	**281**	**350**	**57**
$E(complex)_c^{AB}$	-1078.161683	-1460.582419	-1013.993578	-1036.002793

$E(LB)_c^{AB}$	-577.757045	-960.176907	-513.594853	-535.604024
$E(BH)_c^{AB}$	-500.233339	-500.232169	-500.231299	-500.231059
$E(LB)_c^{A}$	-577.746614	-960.165440	-513.587823	-535.597151
$E(LB)_r^{A}$	-577.760698	-960.177552	-513.593044	-535.602480
$E(BH)_c^{B}$	-500.227592	-500.224311	-500.224444	-500.224190
$E(BH)_r^{B}$	-500.293523	-500.293523	-500.293523	-500.293523
	**89**	**259**	**261**	**264**
$E(complex)_c^{AB}$	-1534.212169	-1343.003361	-1382.197360	-1230.182006
$E(LB)_c^{AB}$	-1033.806072	-842.596793	-881.788684	-729.773756
$E(BH)_c^{AB}$	-500.234169	-500.240662	-500.240106	-500.235853
$E(LB)_c^{A}$	-1033.795340	-842.585929	-881.777697	-729.762634
$E(LB)_r^{A}$	-1033.807304	-842.598952	-881.790710	-729.777017
$E(BH)_c^{B}$	-500.226420	-500.233858	-500.233088	-500.229311
$E(BH)_r^{B}$	-500.293523	-500.293523	-500.293523	-500.293523
	**267**	**294**	**102**	**269**
$E(complex)_c^{AB}$	-1420.167289	-1692.202695	-1460.584086	-1308.569956
$E(LB)_c^{AB}$	-919.747146	-1191.792959	-960.174950	-808.158887
$E(BH)_c^{AB}$	-500.239684	-500.231694	-500.240279	-500.236206
$E(LB)_c^{A}$	-919.746091	-1191.781566	-960.162422	-808.147488
$E(LB)_r^{A}$	-919.759576	-1191.793339	-960.174950	-808.161715
$E(BH)_c^{B}$	-500.232375	-500.223204	-500.232971	-500.229411
$E(BH)_r^{B}$	-500.293523	-500.293523	-500.293523	-500.293523
	**270**	**251 (P)**	**276**	**280**
$E(complex)_c^{AB}$	-1421.391237	-1515.571737	-1460.586782	-1386.955661
$E(LB)_c^{AB}$	-920.981562	-1015.386793	-960.176686	-886.543010
$E(BH)_c^{AB}$	-500.239620	-500.233631	-500.235926	-500.236332
$E(LB)_c^{A}$	-920.970523	-1015.374900	-960.165231	-886.531390
$E(LB)_r^{A}$	-920.983004	-1015.390461	-960.176936	-886.545569
$E(BH)_c^{B}$	-500.232432	-500.225527	-500.228313	-500.229353
$E(BH)_r^{B}$	-500.293523	-500.293523	-500.293523	-500.293523
	**278**	**268**	**124**	**285**
$E(complex)_c^{AB}$	-1459.364019	-1421.393800	-1544.934756	-1537.791930
$E(LB)_c^{AB}$	-958.953507	-920.985066	-1044.519484	-1037.380205
$E(BH)_c^{AB}$	-500.239039	-500.235190	-500.229936	-500.235080
$E(LB)_c^{A}$	-958.942406	-920.973576	-1044.507251	-1037.368862
$E(LB)_r^{A}$	-958.953942	-920.985314	-1044.521476	-1037.380450
$E(BH)_c^{B}$	-500.231626	-500.227758	-500.221621	-500.227367
$E(BH)_r^{B}$	-500.293523	-500.293523	-500.293523	-500.293523
	**60**	**286**	**295**	**289**
$E(complex)_c^{AB}$	-961.164553	-1498.590334	-1651.810409	-1576.976466
$E(LB)_c^{AB}$	-460.761887	-998.178008	-1151.397072	-1076.562026
$E(BH)_c^{AB}$	-500.220617	-500.236561	-500.236876	-500.237943
$E(LB)_c^{A}$	-460.753398	-998.166719	-1151.386813	-1076.550367
$E(LB)_r^{A}$	-460.754715	-998.178646	-1151.399165	-1076.563038
$E(BH)_c^{B}$	-500.212951	-500.228952	-500.228956	-500.229837
$E(BH)_r^{B}$	-500.293523	-500.293523	-500.293523	-500.293523
	**298**	**300**		
$E(complex)_c^{AB}$	-1876.956946	-1935.002933		

$E(LB)_c^{AB}$	-1376.534951	-1434.562048		
$E(BH)_c^{AB}$	-500.240021	-500.236119		
$E(LB)_c^{A}$	-1376.524037	-1434.551025		
$E(LB)_r^{A}$	-1376.539446	-1434.569570		
$E(BH)_c^{B}$	-500.231967	-500.228062		
$E(BH)_r^{B}$	-500.293523	-500.293523		
	**343**	**345**	**45**	**44**
$E(complex)_c^{AB}$	-1073.339973	-1132.964360	-1022.269428	-1075.093754
$E(LB)_c^{AB}$	-342.594287	-402.225284	-291.528533	-344.350344
$E(TT)_c^{AB}$	-730.673713	-730.725662	-730.725998	-730.720873
$E(LB)_c^{A}$	-342.585074	-402.222130	-291.525214	-344.345414
$E(LB)_r^{A}$	-342.594262	-402.2223122	-291.5262623	-344.3461625
$E(TT)_c^{B}$	-730.670191	-730.722318	-730.722807	-730.716202
$E(TT)_r^{B}$	-730.722779	-730.722779	-730.722779	-730.722779
	**347**	**346**	**117**	**53**
$E(complex)_c^{AB}$	-1518.137757	-1111.952050	-1426.107900	-1059.115265
$E(LB)_c^{AB}$	-787.396297	-381.179767	-695.332180	-328.347512
$E(TT)_c^{AB}$	-730.725763	-730.645988	-730.645861	-730.667349
$E(LB)_c^{A}$	-787.392801	-381.169398	-695.318070	-328.338104
$E(LB)_r^{A}$	-787.3925616	-381.1857028	-695.336484	-328.3433046
$E(TT)_c^{B}$	-730.722392	-730.636229	-730.637243	-730.658669
$E(TT)_r^{B}$	-730.722779	-730.722779	-730.722779	-730.722779
	**1**	**14**	**279**	**24**
$E(complex)_c^{AB}$	-978.363381	-1017.565636	-1538.954748	-978.363381
$E(LB)_c^{AB}$	-247.596077	-286.793763	-808.161343	-247.596077
$E(TT)_c^{AB}$	-730.653413	-730.652883	-730.653121	-730.653413
$E(LB)_c^{A}$	-247.587867	-286.785583	-808.147632	-247.587857
$E(LB)_r^{A}$	-247.5894387	-286.7872853	-808.164298	-264.8031187
$E(TT)_c^{B}$	-730.645523	-730.644904	-730.644375	-730.645523
$E(TT)_r^{B}$	-730.722779	-730.722779	-730.722779	-730.722779
	**281**	**348**	**252**	**124**
$E(complex)_c^{AB}$	-1690.970280	-1111.966016	-1989.839883	-1775.321716
$E(LB)_c^{AB}$	-960.180957	-381.182357	-1259.029056	-1044.520515
$E(TT)_c^{AB}$	-730.665629	-730.651836	-730.667275	-730.656372
$E(LB)_c^{A}$	-960.167869	-381.173546	-1259.015234	-1044.506509
$E(LB)_r^{A}$	-960.177552	-381.1761503	-1259.045037	-1044.521476
$E(TT)_c^{B}$	-730.656203	-730.643295	-730.657864	-730.646353
$E(TT)_r^{B}$	-730.722779	-730.722779	-730.722779	-730.722779
	**70**	**98**	**255**	**54**
$E(complex)_c^{AB}$	-1190.983199	-1308.558534	-1533.806050	-1111.974491
$E(LB)_c^{AB}$	-460.192708	-577.763428	-802.984976	-381.184764
$E(TT)_c^{AB}$	-730.664451	-730.662966	-730.665233	-730.650907
$E(LB)_c^{A}$	-460.181722	-577.750715	-802.970212	-381.176366
$E(LB)_r^{A}$	-460.190902	-577.760698	-803.005409	-381.179961
$E(TT)_c^{B}$	-730.658754	-730.655419	-730.656287	-730.642706
$E(TT)_r^{B}$	-730.722779	-730.722779	-730.722779	-730.722779
	**254**	**268**	**256**	**56**
$E(complex)_c^{AB}$	-3141.744561	-1651.787345	-1382.193335	-1189.181542

$E(LB)_c^{AB}$	-2410.947740	-920.985087	-651.396376	-458.388664
$E(TT)_c^{AB}$	-730.667971	-730.663379	-730.658180	-730.650584
$E(LB)_c^{A}$	-2410.934805	-920.971620	-651.383960	-458.380275
$E(LB)_r^{A}$	-2410.943810	-920.985314	-651.393617	-458.3839094
$E(TT)_c^{B}$	-730.657715	-730.653867	-730.650963	-730.064224
$E(TT)_r^{B}$	-730.722779	-730.722779	-730.722779	-730.722779
	**276**	**349**	**285**	**280**
$E(complex)_c^{AB}$	-1690.981178	-1338.790.4223	-1768.186917	-1617.352268
$E(LB)_c^{AB}$	-960.176781	-607.990152	-1037.380693	-886.548059
$E(TT)_c^{AB}$	-730.663018	-730.649605	-730.662188	-730.662188
$E(LB)_c^{A}$	-960.163273	-607.981575	-1037.366867	-1037.366867
$E(LB)_r^{A}$	-960.176936	-607.9908239	-1037.380450	-1037.380450
$E(TT)_c^{B}$	-730.653340	-730.641134	-730.652095	-730.652095
$E(TT)_r^{B}$	-730.722779	-730.722779	-730.722779	-730.722779
	**264**	**102**	**259**	**286**
$E(complex)_c^{AB}$	-1612.596041	-1690.983362	-1573.404738	-1728.987165
$E(LB)_c^{AB}$	-881.793388	-960.178752	-842.602288	-998.179848
$E(TT)_c^{AB}$	-730.657202	-730.657284	-730.659220	-730.661399
$E(LB)_c^{A}$	-881.780287	-960.165358	-842.589083	-998.166068
$E(LB)_r^{A}$	-881.790710	-960.176936	-842.598952	-998.178475
$E(TT)_c^{B}$	-730.648322	-730.648067	-730.650436	-730.651509
$E(TT)_r^{B}$	-730.722779	-730.722779	-730.722779	-1728.987165
	**270**	**57**	**350**	**89**
$E(complex)_c^{AB}$	-1651.596041	-1266.405582	-1244.396539	-1764.617433
$E(LB)_c^{AB}$	-881.793388	-535.606338	-513.597122	-1033.811231
$E(TT)_c^{AB}$	-730.657202	-730.650268	-730.650213	-730.666772
$E(LB)_c^{A}$	-881.780287	-535.597766	-513.588301	-1033.798076
$E(LB)_r^{A}$	-881.790710	-535.6024802	-513.593044	-1033.807304
$E(TT)_c^{B}$	-730.648322	-730.6413839	-730.641513	-730.656674
$E(TT)_r^{B}$	-730.722779	-730.722779	-730.722779	-730.722779
	**60**	**295**	**298**	**300**
$E(complex)_c^{AB}$	-1191.562910	-1882.215855	-2107.362099	-2165.408792
$E(LB)_c^{AB}$	-460.764772	-1151.402467	-1376.541389	-1434.568841
$E(TT)_c^{AB}$	-730.639311	-730.666015	-730.665673	-730.665316
$E(LB)_c^{A}$	-460.754007	-1151.389351	-1376.527834	-1434.554842
$E(LB)_r^{A}$	-460.754715	-1151.399165	-1376.539446	-1434.569570
$E(TT)_c^{B}$	-730.629330	-730.655689	-730.655220	-730.654605
$E(TT)_r^{B}$	-730.722779	-730.722779	-730.722779	-730.722779

Table S3. Thermal corrections of the adducts LB^+-BH and LB^+-TT (LB = Lewis Base, BH = benzhydrl, TT = trityl); ordered by the sequence of Table 9.

system	thermal correction LB^+-BH	thermal correction LB^+-TT
**343**	+0.242381	+0.327528
**344**	+0.254794	-
**251 (N)**	+0.557494	-

**345**	+0.399980	+0.482641
**45**	+0.433184	+0.515544
**20**	+0.343558	-
**346**	+0.386322	+0.470650
**1**	+0.308526	+0.393704
**347**	+0.410861	+0.493649
**44**	+0.407395	+0.489759
**14**	+0.337944	+0.423068
**53**	+0.419254	+0.503475
**24**	+0.320257	+0.405361
**252**	+0.519459	+0.604657
**348**	+0.386362	+0.471553
**254**	+0.479056	+0.564549
**255**	+0.443782	+0.528976
**54**	+0.386886	+0.471937
**70**	+0.334412	+0.419885
**56**	+0.424394	+0.509453
**117**	+0.515221	+0.596171
**349**	+0.488602	+0.573687
**256**	+0.448007	+0.476936
**279**	+0.507975	+0.597473
**98**	+0.511261	+0.511261
**281**	+0.537433	+0.623023
**350**	+0.442341	+0.527464
**57**	+0.462005	+0.547124
**89**	+0.504520	+0.590245
**259**	+0.448007	+0.533830
**261**	+0.451849	-
**264**	+0.478229	+0.564002
**267**	+0.484683	-
**294**	+0.651000	-
**102**	+0.537963	+0.623777
**269**	+0.511910	-
**270**	+0.507762	+0.593832
**251 (P)**	+0.556583	-
**276**	+0.538134	+0.623947
**280**	+0.571652	+0.657233
**278**	+0.515085	-
**268**	+0.507975	+0.593830
**124**	+0.719680	+0.805166
**285**	+0.576206	+0.661787
**60**	+0.473092	+0.557580
**286**	+0.545976	+0.632068
**295**	+0.592628	+0.678127
**289**	+0.606185	-
**298**	+0.611012	+0.696513
**300**	+0.738508	+0.824182

COMPUTATIONAL METHODS (MOSCA)

"Mosher's cation affinity" (MOSCA) of Lewis bases (LB) has been calculated as the reaction enthalpy at 298.15 K and 1 atm pressure including solvent effects ($CHCl_3$) for the detachment reaction shown in equation (S9). This is in analogy to the mass spectrometric definition of proton affinities.

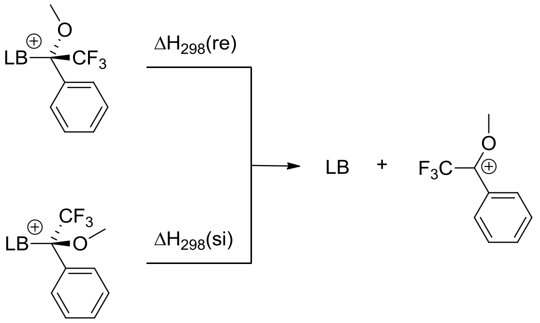

(S9)

The geometries of all species in equation (S9) have been optimized at the B98/6-31G(d) level of theory. The conformational space of flexible Lewis bases and the corresponding cations have been searched using the MM3* force field and the systematic search routine implemented in MACROMODEL 9.7 [48]. All stationary points located at force field level have then been reoptimized at B98/6-31G(d) level as described before. Thermochemical corrections to 298.15 K have been calculated for all minima from unscaled vibrational frequencies obtained at this same level. The thermochemical corrections have been combined with single point energies calculated at the MP2(FC)/6-31+G(2d,p)//B98/6-31G(d) level to yield enthalpies H_{298} at 298.15 K. In conformationally flexible systems enthalpies have been calculated as Boltzmann-averaged values over all available conformers. In order to consider solvent effects in chloroform the PCM/UAHF/RHF/6-31G(d) approach was used. All quantum mechanical calculations have been performed with Gaussian 03 [49].

Table S4. Calculated energies (in Hartree) of conformations of MOSC-adducts of pyridines **63**, **351**-**357**. Averaged enthalpies <H_{298}> were calculated at MP2(FC)/6-31+G(2d,p)//B98/6-31G(d) level of theory with inclusion of solvent effects in chloroform at PCM/UAHF/RHF/6-31G(d) level.

system	B98/6-31G(d)		MP2(FC)/6-31+G(2d,p)		PCM/ UAHF/ RHF/ 6-31G(d)	"H_{298}" + ΔG_{solv}
	**E_{tot}**	**H_{298}**	**E_{tot}**	**"H_{298}"**	**ΔG_{solv}** [kJ/mol]	**<H_{298}>**
**MOSC cation**						-720.373983
mosc_1	-721.9716283	-721.802591	-720.4962865	-720.3272492	-123.05	-720.374117
mosc_2	-721.9673426	-721.798547	-720.4944162	-720.3256206	-126.48	-720.373795
**63_si**						-1467.666415
**N_3ax N_4eq**						
63_si1_1	-1471.475458	-1470.917615	-1468.196958	-1467.639115	-73.18	-1467.666987
63_si1_2	-1471.475010	-1470.917501	-1468.195750	-1467.638241	-74.35	-1467.666559
63_si1_3	-1471.473966	-1470.916477	-1468.195487	-1467.637998	-75.14	-1467.666619
63_si1_4	-1471.472676	-1470.915430	-1468.194442	-1467.637196	-73.68	-1467.665259
63_si1_5	-1471.473741	-1470.916465	-1468.194444	-1467.637167	-76.15	-1467.666171
**N_3eq N_4ax**						
63_si2_1	-1471.473464	-1470.915918	-1468.195101	-1467.637555	-73.60	-1467.665586

63_si2_2	-1471.472778	-1470.915290	-1468.194385	-1467.636896	-74.64	-1467.665326
63_si2_3	-1471.472124	-1470.914649	-1468.194290	-1467.636816	-72.72	-1467.664513
**63_re**						-1467.667111
**N_3ax N_4eq**						
63_re1_1	-1471.475220	-1470.917544	-1468.197278	-1467.639602	-73.68	-1467.667665
63_re1_2	-1471.475527	-1470.918132	-1468.196113	-1467.638717	-75.27	-1467.667386
63_re1_2	-1471.473954	-1470.916603	-1468.195951	-1467.638600	-75.19	-1467.667237
63_re1_3	-1471.472200	-1470.914895	-1468.194804	-1467.637499	-73.47	-1467.665483
63_re1_4	-1471.474207	-1470.916467	-1468.194677	-1467.636936	-76.82	-1467.666195
**N_3eq N_4ax**						
63_re2_1	-1471.473284	-1470.915767	-1468.195508	-1467.637990	-74.18	-1467.666245
63_re2_2	-1471.472209	-1470.914659	-1468.194351	-1467.636800	-74.18	-1467.665055
63_re2_3	-1471.473512	-1470.916080	-1468.194134	-1467.636702	-75.60	-1467.665498
**351_si**						-1928.294158
351_si_1	-1933.383850	-1932.654873	-1929.009177	-1928.280201	-37.32	-1928.294416
351_si_2	-1933.383547	-1932.654586	-1929.007881	-1928.278920	-36.94	-1928.292991
**351_re**						-1928.294105
351_re_1	-1933.381012	-1932.651973	-1929.008539	-1928.279500	-38.66	-1928.294225
351_re_2	-1933.382643	-1932.653879	-1929.008349	-1928.279585	-37.70	-1928.293944
**352_si**						-2005.444708
352_si_1	-2010.739459	-2009.976436	-2006.190742	-2005.427719	-45.73	-2005.445137
352_si_2	-2010.737998	-2009.974494	-2006.189427	-2005.425924	-44.52	-2005.442880
352_si_3	-2010.738485	-2009.974889	-2006.189757	-2005.426161	-43.64	-2005.442782
352_si_3	-2010.740839	-2009.977367	-2006.191644	-2005.428173	-36.53	-2005.442085
**352_re**						-2005.442188
352_re_1	-2010.739950	-2009.976345	-2006.188553	-2005.424947	-46.53	-2005.442668
352_re_2	-2010.739609	-2009.975906	-2006.188746	-2005.425043	-45.27	-2005.442286
352_re_3	-2010.740263	-2009.977138	-2006.189781	-2005.426656	-38.20	-2005.441205
352_re_4	-2010.739382	-2009.975609	-2006.190313	-2005.426541	-37.40	-2005.440787
352_re_5	-2010.740118	-2009.976491	-2006.188897	-2005.425270	-39.25	-2005.440218
**353_si**						-1793.607925
353_si_1	-1798.248075	-1797.622995	-1794.217252	-1793.592172	-42.34	-1793.608299
353_si_2	-1798.248044	-1797.623118	-1794.217525	-1793.592599	-40.88	-1793.608168
353_si_3	-1798.249816	-1797.624807	-1794.216431	-1793.591422	-43.64	-1793.608043
353_si_4	-1798.247764	-1797.622829	-1794.217091	-1793.592156	-41.00	-1793.607774
353_si_5	-1798.249486	-1797.624509	-1794.217766	-1793.592789	-38.99	-1793.607642
353_si_6	-1798.246989	-1797.622103	-1794.216055	-1793.591169	-41.92	-1793.607137
353_si_7	-1798.247348	-1797.622564	-1794.215947	-1793.591163	-40.42	-1793.606557
**353_re**						-1793.608664
353_re_1	-1798.249822	-1797.624956	-1794.218403	-1793.593537	-41.17	-1793.609218
353_re_2	-1798.248135	-1797.623426	-1794.216952	-1793.592243	-43.60	-1793.608848
353_re_3	-1798.248103	-1797.623227	-1794.217291	-1793.592415	-41.88	-1793.608367
353_re_4	-1798.246678	-1797.622099	-1794.215778	-1793.591200	-43.05	-1793.607598
353_re_5	-1798.247486	-1797.622797	-1794.215951	-1793.591262	-42.51	-1793.607453
353_re_6	-1798.248874	-1797.623834	-1794.216305	-1793.591264	-42.09	-1793.607296
**354**	see reference 6					
**355**	see reference 6					
**356**	see reference 6					
**357**	see reference 6					

COMPUTATIONAL METHODS (ACA)

Acetyl cation affinity (ACA) of Lewis bases (LB) has been calculated as the reaction enthalpy at 298.15 K and 1 atm pressure including solvent effects ($CHCl_3$) for the detachment reaction shown in

equation (S10). This is in analogy to the mass spectrometric definition of proton affinities.

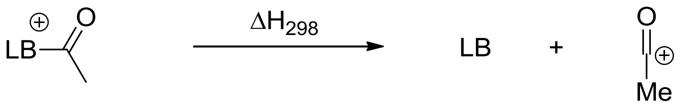

(S10)

The geometries of all species in equation (S10) have been optimized at the B98/6-31G(d) level of theory. The conformational space of flexible Lewis bases and the corresponding cations have been searched using the MM3* force field and the systematic search routine implemented in MACROMODEL 9.7 [48]. All stationary points located at force field level have then been reoptimized at B98/6-31G(d) level as described before. Thermochemical corrections to 298.15 K have been calculated for all minima from unscaled vibrational frequencies obtained at this same level. The thermochemical corrections have been combined with single point energies calculated at the MP2(FC)/6-31+G(2d,p)//B98/6-31G(d) level to yield enthalpies H_{298} at 298.15 K. In conformationally flexible systems enthalpies have been calculated as Boltzmann-averaged values over all available conformers. In order to consider solvent effects in chloroform the PCM/UAHF/RHF/6-31G(d) approach was used. All quantum mechanical calculations have been performed with Gaussian 03 [49].

Table S5. Total energies and enthalpies (in Hartree) as calculated at the B98/6-31G(d) and MP2(FC)/6-31+G(2d,p)// B98/6-31G(d) level of theory for all systems (ACA) as well as inclusion of solvent effects in chloroform at PCM/UAHF/RHF/6-31G(d). If more than one conformer exist at 298.15 K, the single values of each conformer are denoted as well as the Boltzmann-averaged values for H_{298} at MP2(FC)/6-31+G(2d,p)//B98/6-31G(d) level of theory. Only conformers are included with a Boltzmann-weighting of at least 1% (rounded) up to a maximum to ten conformers per system. The systems are ordered by the sequence of Tables 13-16.

system	B98/6-31G(d)		MP2(FC)/ 6-31+G(2d,p)	PCM/ UAHF/ RHF/ 6-31G(d)	MP2(FC)/ 6-31+G(2d,p)	"H_{298}" + ΔG_{solv}
	**E_{tot}**	**H_{298}**	**E_{tot}**	**ΔG_{solv}** [kJ/mol]	**"H_{298}" + ΔG_{solv}**	**<H_{298}>**
**1**	-248.181767	-248.087627	-247.589439	-9.00	-247.498727	-247.498727
**1-Ac$^+$**	-401.140004	-400.991691	-400.215516	-142.55	-400.121498	-400.121498
**358**	-5445.710391	-5445.615295	-5442.043341	-1.1	-5441.948244	-5441.949997
**358-Ac$^+$**	-5598.675003	-5598.525635	-5594.676214	-30.89	-5594.526846	-5594.576073
**359**						-454.947460
359_1	-456.123075	-455.969135	-455.094581	-4.29	-454.940642	-454.947478
359_2	-456.117560	-455.963631	-455.089897	-3.84	-454.935967	-454.942087
**359-Ac$^+$**						-607.577447
359-Ac$^+$_1	-609.097006	-608.889040	-607.733281	-32.79	-607.525315	-607.577570
359-Ac$^+$_2	-609.096181	-608.888102	-607.732549	-33.14	-607.524470	-607.577282
359-Ac$^+$_3	-609.084790	-608.876809	-607.721457	-34.48	-607.513476	-607.568424
359-Ac$^+$_4	-609.085031	-608.876864	-607.721499	-34.37	-607.513331	-607.568103
**360**	-366.086672	-365.904186	-365.181353	-7.11	-365.001576	-365.001576
**360-Ac$^+$**	-519.060128	-518.823483	-517.822649	-126.98	-517.634369	-517.634369
**361**						-482.485398
361_1	-483.975105	-483.702005	-482.758292	-1.67	-482.485830	
361_2	-483.973719	-483.700644	-482.756390	-1.92	-482.484048	

361_3	-483.972675	-483.699673	-482.755393	-2.64	-482.483395	
361_4	-483.972119	-483.699057	-482.753971	-3.77	-482.482344	
**361-Ac$^+$**						-635.118726
361-Ac$^+$_1	-636.951414	-636.624166	-635.402799	-115.06	-635.119375	
361-Ac$^+$_2	-636.949865	-636.622659	-635.400863	-115.14	-635.117513	
361-Ac$^+$_3	-636.949773	-636.622428	-635.400705	-114.77	-635.117072	
361-Ac$^+$_4	-636.949652	-636.622472	-635.399800	-115.27	-635.116523	
361-Ac$^+$_5	-636.949816	-636.622611	-635.399914	-114.68	-635.116389	
361-Ac$^+$_6	-636.948798	-636.621373	-635.399634	-114.89	-635.115970	
361-Ac$^+$_7	-636.948571	-636.621367	-635.399474	-113.68	-635.115569	
**362**						-599.969258
362_1	-601.867333	-601.504627	-600.335730	8.66	-599.969725	
362_2	-601.866425	-601.503546	-600.335656	8.54	-599.969526	
362_3	-601.865755	-601.503017	-600.335223	8.95	-599.969075	
362_4	-601.866606	-601.503817	-600.335327	9.12	-599.969064	
362_5	-601.866109	-601.503417	-600.334732	7.95	-599.969012	
362_6	-601.864819	-601.501971	-600.334716	8.33	-599.968696	
362_7	-601.864972	-601.502256	-600.334668	9.00	-599.968526	
362_8	-601.865940	-601.503323	-600.333754	8.12	-599.968046	
362_9	-601.866884	-601.504187	-600.333874	8.45	-599.967958	
**362-Ac$^+$**						-752.602459
362-Ac$^+$_1	-754.846894	-754.429794	-752.982879	-98.28	-752.603213	
362-Ac$^+$_2	-754.845981	-754.428934	-752.982842	-97.49	-752.602926	
362-Ac$^+$_3	-754.846060	-754.429106	-752.983008	-96.78	-752.602915	
362-Ac$^+$_4	-754.845109	-754.428275	-752.982213	-97.74	-752.602605	
362-Ac$^+$_5	-754.844513	-754.427544	-752.981423	-99.58	-752.602382	
362-Ac$^+$_6	-754.845179	-754.428012	-752.982244	-97.82	-752.602335	
362-Ac$^+$_7	-754.844582	-754.427449	-752.981463	-99.66	-752.602289	
362-Ac$^+$_8	-754.845169	-754.428013	-752.982907	-95.69	-752.602196	
362-Ac$^+$_9	-754.844386	-754.427448	-752.982380	-96.19	-752.602079	
**363**	-2953.188796	-2953.025593	-2950.788635	-1.79	-2950.625433	-2950.628285
**363-Ac$^+$**						-3103.261734
363-Ac$^+$_1	-3106.165812	-3105.947834	-3103.431149	-30.62	-3103.213171	-3103.261967
363-Ac$^+$_2	-3106.166084	-3105.947897	-3103.431489	-30.07	-3103.213302	-3103.261222
**364**	-421.396615	-421.195314	-420.375062	-2.24	-420.173761	-420.177330
**364-Ac$^+$**						-572.819276
364-Ac$^+$_1	-574.384977	-574.128494	-573.027529	-30.38	-572.771046	-572.819460
364-Ac$^+$_2	-574.384774	-574.128179	-573.027195	-30.35	-572.770601	-572.818966
**365**						-533.223855
365_1	-534.674094	-534.458445	-533.431574	-5.09	-533.215925	-533.224037
365_2	-534.671639	-534.456042	-533.428547	-4.90	-533.212950	-533.220759
365_3	-534.667855	-534.453746	-533.427916	-4.26	-533.213807	-533.220595
**365-Ac$^+$**						-685.866099
365-Ac$^+$_1	-687.661866	-687.391436	-686.082793	-33.72	-685.812363	-685.866099
365-Ac$^+$_2	-687.661866	-687.391436	-686.082792	-33.72	-685.812362	-685.866099
365-Ac$^+$_3	-687.661866	-687.391435	-686.082794	-33.72	-685.812363	-685.866099
365-Ac$^+$_4	-687.661866	-687.391435	-686.082793	-33.72	-685.812362	-685.866098
365-Ac$^+$_5	-687.661866	-687.391436	-686.082792	-33.72	-685.812362	-685.866098
365-Ac$^+$_6	-687.661866	-687.391436	-686.082793	-33.72	-685.812363	-685.866099
**366**						-537.664361
366_1	-539.288171	-538.997296	-537.955185	-0.43	-537.664310	-537.664995
366_2	-539.286526	-538.995985	-537.955218	-0.12	-537.664677	-537.664868
366_3	-539.287920	-538.996961	-537.954938	-0.33	-537.663979	-537.664504
366_4	-539.287884	-538.996914	-537.954329	-0.46	-537.663359	-537.664092
366_5	-539.287894	-538.996750	-537.953909	-0.78	-537.662765	-537.664008

366_6	-539.287382	-538.996484	-537.953801	-0.51	-537.662903	-537.663715
366_7	-539.287477	-538.996378	-537.953881	-0.43	-537.662782	-537.663467
366_8	-539.287388	-538.996260	-537.953389	-0.58	-537.662261	-537.663185
366_9	-539.287406	-538.996102	-537.952970	-0.93	-537.661667	-537.663149
366_10	-539.286388	-538.994735	-537.952295	-1.12	-537.660642	-537.662427
**366-Ac$^+$**						-690.307930
366-Ac$^+$_1	-692.279948	-691.933358	-690.611664	-27.47	-690.265074	-690.308850
366-Ac$^+$_2	-692.279758	-691.933333	-690.611476	-27.45	-690.265051	-690.308795
366-Ac$^+$_3	-692.280672	-691.933848	-690.611320	-27.29	-690.264496	-690.307986
366-Ac$^+$_4	-692.280672	-691.933849	-690.611318	-27.29	-690.264495	-690.307984
366-Ac$^+$_5	-692.280366	-691.933609	-690.610929	-27.12	-690.264173	-690.307391
366-Ac$^+$_6	-692.280366	-691.933609	-690.610925	-27.11	-690.264168	-690.307371
366-Ac$^+$_7	-692.279420	-691.932822	-690.610141	-27.42	-690.263543	-690.307240
366-Ac$^+$_8	-692.278087	-691.931753	-690.609443	-27.56	-690.263109	-690.307029
366-Ac$^+$_9	-692.278087	-691.931752	-690.609442	-27.56	-690.263107	-690.307027
366-Ac$^+$_10	-692.280012	-691.933306	-690.609490	-27.54	-690.262784	-690.306672
**367**						-655.147892
367_1	-657.179511	-656.799798	-655.531674	2.22	-655.151962	-655.148424
367_2	-657.179355	-656.798724	-655.532831	2.48	-655.152200	-655.148248
367_3	-657.179512	-656.798671	-655.531991	2.11	-655.151150	-655.147787
367_4	-657.179875	-656.799109	-655.532221	2.32	-655.151456	-655.147759
367_5	-657.180569	-656.799857	-655.532141	2.34	-655.151429	-655.147700
367_6	-657.180584	-656.799829	-655.532164	2.36	-655.151409	-655.147648
367_7	-657.180595	-656.799826	-655.532137	2.35	-655.151369	-655.147624
367_8	-657.179475	-656.798946	-655.531840	2.32	-655.151311	-655.147614
367_9	-657.177605	-656.796752	-655.531788	2.13	-655.150935	-655.147540
367_10	-657.180574	-656.799765	-655.532085	2.35	-655.151276	-655.147531
**367-Ac$^+$**						-807.791735
367-Ac$^+$_1	-810.174823	-809.738348	-808.191656	-23.18	-807.755181	-807.792120
367-Ac$^+$_2	-810.173846	-809.737642	-808.191449	-23.05	-807.755245	-807.791977
367-Ac$^+$_3	-810.172493	-809.736039	-808.191092	-23.28	-807.754638	-807.791737
367-Ac$^+$_4	-810.171537	-809.734888	-808.191329	-23.23	-807.754680	-807.791699
367-Ac$^+$_5	-810.172419	-809.736241	-808.190159	-23.65	-807.753981	-807.791670
367-Ac$^+$_6	-810.172419	-809.736241	-808.190159	-23.64	-807.753981	-807.791654
367-Ac$^+$_7	-810.174620	-809.738147	-808.191497	-22.95	-807.755023	-807.791596
367-Ac$^+$_8	-810.171541	-809.734866	-808.191352	-23.13	-807.754676	-807.791537
367-Ac$^+$_9	-810.174168	-809.737487	-808.191435	-23.01	-807.754754	-807.791422
367-Ac$^+$_10	-810.173784	-809.737454	-808.191607	-22.68	-807.755277	-807.791420
**368**						-997.111684
368_1	-999.950530	-999.511753	-997.549332	-3.01	-997.111702	
368_2	-999.945655	-999.507063	-997.543355	-4.02	-997.106292	
368_3	-999.939406	-999.501227	-997.538564	-2.01	-997.101150	
**368-Ac$^+$**						-997.111684
368-Ac$^+$_1	-1152.945152	-1152.451635	-1150.208832	-106.40	-1149.755840	
368-Ac$^+$_2	-1152.944895	-1152.451053	-1150.208192	-103.18	-1149.753648	
368-Ac$^+$_3	-1152.942366	-1152.448863	-1150.204785	-107.45	-1149.752206	
368-Ac$^+$_4	-1152.941373	-1152.447829	-1150.204162	-102.80	-1149.749772	
**54**	-382.100962	-381.928959	-381.179977	-13.68	-381.013184	-381.013184
**54-Ac$^+$**	-535.091159	-534.864305	-533.836116	-131.34	-533.659287	-533.659287
**369**	-421.400415	-421.198543	-420.374849	-2.84	-420.172978	-420.177503
**369-Ac$^+$**						-572.824386
369-Ac$^+$_1	-574.392288	-574.135480	-573.032902	-30.37	-572.776094	-572.824492
369-Ac$^+$_2	-574.392332	-574.135258	-573.032926	-30.37	-572.775852	-572.824249
**370**						-537.663074
370_1	-539.292811	-539.001043	-537.954451	-0.71	-537.662683	-537.663814

370_2	-539.293771	-539.002066	-537.954231	-0.65	-537.662525	-537.663561
370_3	-539.288540	-538.997719	-537.952842	-0.86	-537.662021	-537.663391
370_4	-539.294761	-539.003085	-537.953964	-0.67	-537.662288	-537.663356
370_5	-539.292832	-539.001072	-537.953309	-0.82	-537.661549	-537.662856
370_6	-539.292832	-539.001067	-537.953310	-0.82	-537.661545	-537.662852
370_7	-539.294028	-539.002224	-537.953368	-0.62	-537.661564	-537.662552
370_8	-539.292137	-539.000246	-537.953085	-0.66	-537.661193	-537.662245
370_9	-539.291362	-538.999336	-537.952841	-0.86	-537.660814	-537.662185
370_10	-539.290465	-538.998527	-537.952504	-0.91	-537.660566	-537.662017
**370-Ac$^+$**						-690.311051
370-Ac$^+$_1	-692.289974	-691.943339	-690.615729	-26.79	-690.269095	-690.311787
370-Ac$^+$_2	-692.289974	-691.943337	-690.615730	-26.76	-690.269093	-690.311738
370-Ac$^+$_3	-692.287303	-691.940567	-690.615189	-27.04	-690.268453	-690.311544
370-Ac$^+$_4	-692.287303	-691.940567	-690.615188	-26.99	-690.268451	-690.311463
370-Ac$^+$_5	-692.288154	-691.941357	-690.615007	-27.08	-690.268210	-690.311365
370-Ac$^+$_6	-692.288154	-691.941361	-690.615006	-27.06	-690.268213	-690.311336
370-Ac$^+$_7	-692.288154	-691.941360	-690.615006	-27.06	-690.268212	-690.311335
370-Ac$^+$_8	-692.286569	-691.939824	-690.614546	-27.18	-690.267802	-690.311116
370-Ac$^+$_9	-692.286568	-691.939820	-690.614543	-27.18	-690.267795	-690.311109
370-Ac$^+$_10	-692.288187	-691.941145	-690.615072	-27.03	-690.268030	-690.311105
**371**						-1074.278104
371_1	-1077.347015	-1076.870141	-1074.752619	-6.23	-1074.278120	
371_2	-1077.342715	-1076.866029	-1074.746456	-6.99	-1074.272431	
371_3	-1077.339728	-1076.863194	-1074.744949	-5.02	-1074.270328	
371_4	-1077.329692	-1076.853718	-1074.737255	-3.93	-1074.262779	
**371-Ac$^+$**						-1226.926262
371-Ac$^+$_1	-1230.347147	-1229.816244	-1227.417396	-105.14	-1226.926540	
371-Ac$^+$_2	-1230.346804	-1229.815727	-1227.416873	-101.84	-1226.924585	
371-Ac$^+$_3	-1230.344580	-1229.813377	-1227.413721	-105.27	-1226.922613	
371-Ac$^+$_4	-1230.344154	-1229.812748	-1227.413367	-101.42	-1226.920590	
371-Ac$^+$_5	-1230.334257	-1229.803052	-1227.403495	-104.85	-1226.912226	
371-Ac$^+$_6	-1230.331957	-1229.800557	-1227.400420	-107.49	-1226.909960	
**372**						-1113.447048
372_1	-1116.647618	-1116.141499	-1113.952361	-2.59	-1113.447230	
372_2	-1116.644983	-1116.139024	-1113.950626	-0.21	-1113.444746	
372_3	-1116.643679	-1116.137275	-1113.946626	-3.77	-1113.441656	
373_4	-1116.640671	-1116.134776	-1113.944643	-1.59	-1113.439353	
**372-Ac$^+$**						-1266.095295
372-Ac$^+$_1	-1269.648565	-1269.087791	-1266.617858	-101.04	-1266.095570	
372-Ac$^+$_2	-1269.647448	-1269.086953	-1266.616781	-96.65	-1266.093098	
372-Ac$^+$_3	-1269.646061	-1269.085151	-1266.614104	-100.75	-1266.091568	
372-Ac$^+$_4	-1269.646320	-1269.085355	-1266.614078	-100.37	-1266.091343	
372-Ac$^+$_5	-1269.645202	-1269.084814	-1266.613683	-96.02	-1266.089868	
372-Ac$^+$_6	-1269.645181	-1269.084299	-1266.612920	-95.48	-1266.088404	
**373**						-459.340887
373_1	-460.699626	-460.467717	-459.569493	-2.29	-459.337583	-459.341233
373_2	-460.698872	-460.467017	-459.568777	-2.39	-459.336921	-459.340730
373_3	-460.695706	-460.463514	-459.566589	-2.54	-459.334396	-459.338444
373_4	-460.695601	-460.463698	-459.566199	-2.53	-459.334296	-459.338328
373_5	-460.688598	-460.457621	-459.563361	-1.71	-459.332384	-459.335109
**373-Ac$^+$**						-611.989239
373-Ac$^+$_1	-613.693223	-613.406531	-612.229788	-29.32	-611.943096	-611.989821
373-Ac$^+$_2	-613.693223	-613.406527	-612.229789	-29.32	-611.943093	-611.989817
373-Ac$^+$_3	-613.691702	-613.404882	-612.228221	-29.54	-611.941402	-611.988477
373-Ac$^+$_4	-613.691702	-613.404881	-612.228221	-29.54	-611.941400	-611.988475

373-Ac$^+$_5	-613.691701	-613.404880	-612.228221	-29.54	-611.941399	-611.988474
373-Ac$^+$_6	-613.691702	-613.404879	-612.228221	-29.54	-611.941399	-611.988474
373-Ac$^+$_7	-613.691702	-613.404879	-612.228221	-29.54	-611.941399	-611.988474
373-Ac$^+$_8	-613.691701	-613.404879	-612.228221	-29.54	-611.941398	-611.988473
373-Ac$^+$_9	-613.688778	-613.401873	-612.225068	-29.50	-611.938163	-611.985174
**56**	-459.499042	-459.289458	-458.383907	-16.90	-458.1807599	-458.180760
**56-Ac$^+$**	-612.493384	-612.228939	-611.043991	-130.58	-610.8292813	-610.829281
**374**						-694.309437
374_1	-696.482340	-696.071133	-694.722029	0.33	-694.310821	-694.310295
374_2	-696.483260	-696.071916	-694.721441	0.15	-694.310098	-694.309858
374_3	-696.484181	-696.073018	-694.720709	-0.16	-694.309546	-694.309801
374_4	-696.482373	-696.071012	-694.720715	0.09	-694.309353	-694.309210
374_5	-696.482373	-696.071012	-694.720715	0.09	-694.309353	-694.309210
374_6	-696.483414	-696.072035	-694.720319	0.06	-694.308940	-694.308845
374_7	-696.480912	-696.069542	-694.720705	0.35	-694.309335	-694.308777
374_8	-696.480912	-696.069540	-694.720705	0.36	-694.309334	-694.308760
374_9	-696.481634	-696.070227	-694.720769	0.63	-694.309362	-694.308358
374_10	-696.480197	-696.068619	-694.721312	1.12	-694.309733	-694.307949
**374-Ac$^+$**						-846.958049
374-Ac$^+$_1	-849.480922	-849.014921	-847.384012	-25.62	-846.918011	-846.958839
374-Ac$^+$_2	-849.480922	-849.014921	-847.384012	-25.62	-846.918011	-846.958839
374-Ac$^+$_3	-849.478322	-849.012227	-847.384344	-25.35	-846.918249	-846.958646
374-Ac$^+$_4	-849.478322	-849.012228	-847.384346	-25.33	-846.918252	-846.958618
374-Ac$^+$_5	-849.478322	-849.012226	-847.384346	-25.33	-846.918250	-846.958616
374-Ac$^+$_6	-849.479604	-849.013299	-847.384208	-25.35	-846.917903	-846.958301
374-Ac$^+$_7	-849.479604	-849.013300	-847.384207	-25.35	-846.917902	-846.958300
374-Ac$^+$_8	-849.479582	-849.013234	-847.384164	-25.29	-846.917816	-846.958118
374-Ac$^+$_9	-849.479151	-849.012711	-847.383968	-25.46	-846.917528	-846.958102
374-Ac$^+$_10	-849.479151	-849.012711	-847.383968	-25.46	-846.917528	-846.958101
**375**						-615.985990
375_1	-617.887497	-617.535936	-616.338253	-0.06	-615.986692	-615.986788
375_2	-617.888418	-617.536743	-616.337923	-0.12	-615.986248	-615.986439
375_3	-617.889328	-617.537918	-616.337281	-0.31	-615.985872	-615.986366
375_4	-617.887503	-617.536024	-616.337133	-0.27	-615.985654	-615.986084
375_5	-617.887503	-617.536023	-616.337133	-0.27	-615.985653	-615.986083
375_6	-617.886098	-617.534293	-616.336942	-0.17	-615.985137	-615.985408
375_7	-617.886098	-617.534293	-616.336942	-0.16	-615.985137	-615.985392
375_8	-617.888537	-617.536890	-616.336840	-0.09	-615.985194	-615.985337
375_9	-617.886754	-617.535081	-616.337112	0.17	-615.985439	-615.985169
375_10	-617.885263	-617.533778	-616.337062	0.30	-615.985577	-615.985099
**375-Ac$^+$**						-768.634640
375-Ac$^+$_1	-770.882953	-770.476564	-769.000087	-26.00	-768.593698	-768.635132
375-Ac$^+$_2	-770.882953	-770.476564	-769.000087	-26.00	-768.593698	-768.635132
375-Ac$^+$_3	-770.883688	-770.477305	-768.999787	-26.18	-768.593405	-768.635125
375-Ac$^+$_4	-770.885523	-770.479189	-769.000042	-25.98	-768.593708	-768.635110
375-Ac$^+$_5	-770.885523	-770.479188	-769.000042	-25.98	-768.593707	-768.635109
375-Ac$^+$_6	-770.883688	-770.477305	-768.999788	-26.09	-768.593406	-768.634983
375-Ac$^+$_7	-770.883688	-770.477305	-768.999787	-26.09	-768.593404	-768.634981
375-Ac$^+$_8	-770.883729	-770.477103	-768.999863	-26.18	-768.593236	-768.634957
375-Ac$^+$_9	-770.884158	-770.477758	-769.000061	-25.89	-768.593661	-768.634919
375-Ac$^+$_10	-770.884158	-770.477752	-769.000064	-25.89	-768.593658	-768.634916
**376**						-772.632535
376_1	-775.077114	-774.606103	-773.105282	0.52	-772.634271	-772.633443
376_2	-775.078023	-774.606780	-773.104714	0.31	-772.633471	-772.632977
376_3	-775.078966	-774.607937	-773.103913	0.01	-772.632884	-772.632868

376_4	-775.077165	-774.605877	-773.103996	0.38	-772.632708	-772.632103
376_5	-775.077165	-774.605877	-773.103996	0.38	-772.632708	-772.632103
376_6	-775.078201	-774.607185	-773.103496	0.24	-772.632481	-772.632098
376_7	-775.075663	-774.604455	-773.103938	0.50	-772.632730	-772.631933
376_8	-775.075663	-774.604455	-773.103938	0.50	-772.632730	-772.631933
376_9	-775.076394	-774.605194	-773.104146	0.92	-772.632946	-772.631480
376_10	-775.075012	-774.604033	-773.105047	1.73	-772.634068	-772.631311
**376-Ac$^+$**						-925.281271
376-Ac$^+$_1	-928.073514	-927.547654	-925.767944	-24.99	-925.242084	-925.281908
376-Ac$^+$_2	-928.073508	-927.547619	-925.767969	-24.98	-925.242080	-925.281889
376-Ac$^+$_3	-928.073384	-927.547455	-925.767939	-24.99	-925.242010	-925.281834
376-Ac$^+$_4	-928.076042	-927.549910	-925.767587	-25.28	-925.241454	-925.281741
376-Ac$^+$_5	-928.076042	-927.549910	-925.767586	-25.28	-925.241454	-925.281740
376-Ac$^+$_6	-928.074230	-927.548259	-925.767633	-25.13	-925.241661	-925.281708
376-Ac$^+$_7	-928.074229	-927.548225	-925.767629	-25.09	-925.241625	-925.281609
376-Ac$^+$_8	-928.074229	-927.548115	-925.767627	-25.14	-925.241513	-925.281576
376-Ac$^+$_9	-928.074752	-927.548676	-925.767664	-25.02	-925.241588	-925.281460
376-Ac$^+$_10	-928.074718	-927.548335	-925.767783	-25.05	-925.241400	-925.281320
**377**						-929.278649
377_1	-932.266633	-931.675706	-929.871618	0.86	-929.280691	-929.279321
377_2	-932.267584	-931.676735	-929.871007	0.62	-929.280157	-929.279169
377_3	-932.268490	-931.677826	-929.870241	0.30	-929.279577	-929.279099
377_4	-932.266627	-931.675837	-929.870194	0.55	-929.279404	-929.278527
377_5	-932.267523	-931.676782	-929.869797	0.55	-929.279056	-929.278180
377_6	-932.265849	-931.675225	-929.870497	1.29	-929.279873	-929.277817
377_7	-932.265275	-931.674129	-929.870283	0.86	-929.279138	-929.277767
377_8	-932.266627	-931.675837	-929.870194	1.11	-929.279404	-929.277635
377_9	-932.265275	-931.674129	-929.870284	1.25	-929.279138	-929.277146
377_10	-932.265691	-931.675106	-929.869398	1.30	-929.278813	-929.276741
**377-Ac$^+$**						-1081.927528
377-Ac$^+$_1	-1085.265916	-1084.620141	-1082.534275	-24.83	-1081.888500	-1081.928069
377-Ac$^+$_2	-1085.265916	-1084.620140	-1082.534275	-24.83	-1081.888499	-1081.928068
377-Ac$^+$_3	-1085.264081	-1084.618509	-1082.534323	-24.59	-1081.888751	-1081.927938
377-Ac$^+$_4	-1085.264081	-1084.618507	-1082.534323	-24.59	-1081.888750	-1081.927937
377-Ac$^+$_5	-1085.264081	-1084.618503	-1082.534329	-24.58	-1081.888751	-1081.927922
377-Ac$^+$_6	-1085.264589	-1084.618904	-1082.534414	-24.52	-1081.888729	-1081.927804
377-Ac$^+$_7	-1085.263339	-1084.617439	-1082.534699	-24.45	-1081.888800	-1081.927763
377-Ac$^+$_8	-1085.263339	-1084.617439	-1082.534699	-24.45	-1081.888799	-1081.927763
377-Ac$^+$_9	-1085.263339	-1084.617439	-1082.534697	-24.45	-1081.888798	-1081.927761
377-Ac$^+$_10	-1085.264551	-1084.618863	-1082.534380	-24.49	-1081.888691	-1081.927718
**378**	-459.503374	-459.293804	-458.39138	-17.07	-458.1883116	-458.1883116
**378-Ac$^+$**						-610.838384
378-Ac$^+$_1	-612.49845	-612.234055	-611.052154	-132.51	-610.8382294	
378-Ac$^+$_2	-612.498722	-612.234281	-611.052535	-132.34	-610.8384997	
**379**						-848.669982
379_1	-851.254112	-850.771763	-849.151412	-0.62	-848.669063	-848.670051
379_2	-851.250657	-850.768052	-849.147394	-0.95	-848.664789	-848.666303
**379-Ac$^+$**						-1001.320907
379-Ac$^+$_1	-1004.257598	-1003.720341	-1001.821116	-23.35	-1001.283858	-1001.321069
379-Ac$^+$_2	-1004.257604	-1003.720441	-1001.820898	-23.37	-1001.283735	-1001.320978
379-Ac$^+$_3	-1004.256924	-1003.719222	-1001.820851	-23.64	-1001.283149	-1001.320822
379-Ac$^+$_4	-1004.256779	-1003.719155	-1001.820692	-23.59	-1001.283069	-1001.320662
**380**						-1281.725678
380_1	-1285.281471	-1284.745912	-1282.260154	-4.06	-1281.726141	
380_2	-1285.281982	-1284.745600	-1282.261284	-3.05	-1281.726065	

380_3	-1285.284861	-1284.748063	-1282.261928	-2.18	-1281.725959	
380_4	-1285.283215	-1284.746804	-1282.259371	-6.53	-1281.725447	
380_5	-1285.281273	-1284.745171	-1282.258752	-3.77	-1281.724084	
380_6	-1285.278318	-1284.743691	-1282.256444	-5.15	-1281.723776	
380_7	-1285.279574	-1284.743039	-1282.259638	-1.42	-1281.723644	
380_8	-1285.281750	-1284.745424	-1282.259941	0.08	-1281.723583	
380_9	-1285.282558	-1284.745726	-1282.258257	-3.93	-1281.722923	
380_10	-1285.279446	-1284.743044	-1282.258692	-1.46	-1281.722849	
**380-Ac$^+$**						-1434.379104
380-Ac$^+$_1	-1438.297571	-1437.705821	-1434.938889	-85.77	-1434.379807	
380-Ac$^+$_2	-1438.296478	-1437.705109	-1434.936891	-87.45	-1434.378828	
380-Ac$^+$_3	-1438.292381	-1437.700646	-1434.936752	-88.70	-1434.378802	
380-Ac$^+$_4	-1438.296438	-1437.704875	-1434.937393	-85.60	-1434.378435	
380-Ac$^+$_5	-1438.290944	-1437.699489	-1434.935021	-90.42	-1434.378003	
380-Ac$^+$_6	-1438.291622	-1437.700685	-1434.934609	-88.07	-1434.377217	
380-Ac$^+$_7	-1438.293252	-1437.702156	-1434.933042	-86.73	-1434.374981	
380-Ac$^+$_8	-1438.283480	-1437.692216	-1434.925131	-105.98	-1434.374233	
380-Ac$^+$_9	-1438.283807	-1437.693144	-1434.928622	-94.89	-1434.374102	
380-Ac$^+$_10	-1438.287670	-1437.696588	-1434.930812	-89.87	-1434.373960	
**57**						-535.362849
57_1	-536.905604	-536.658613	-535.602351	-20.33	-535.3631033	
57_2	-536.904889	-536.657992	-535.601147	-20.75	-535.3621533	
**57-Ac$^+$**						-688.016947
57-Ac$^+$_1	-689.905522	-689.603408	-688.268359	-133.80	-688.0172068	
57-Ac$^+$_2	-689.904728	-689.602521	-688.267441	-133.72	-688.0161653	
**381**						-692.015254
381_1	-694.077726	-693.713030	-692.375522	-2.88	-692.010827	-692.015416
381_2	-694.077773	-693.712773	-692.375016	-3.13	-692.010016	-692.015004
**381-Ac$^+$**						-844.669415
381-Ac$^+$_1	-847.078527	-846.660001	-845.043576	-28.10	-844.625051	-844.669831
381-Ac$^+$_2	-847.079945	-846.660004	-845.043578	-28.09	-844.623637	-844.668402
381-Ac$^+$_3	-847.078585	-846.658988	-845.042365	-27.91	-844.622767	-844.667245
381-Ac$^+$_4	-847.079945	-846.658938	-845.042332	-27.93	-844.621324	-844.665834
**382**						-692.027002
382_1	-694.092217	-693.727548	-692.387481	-2.76	-692.022812	-692.027211
382_2	-694.091422	-693.726710	-692.386808	-2.83	-692.022096	-692.026605
**382-Ac$^+$**						
382-Ac$^+$_1	-847.095804	-846.676012	-845.057557	-27.56	-844.637765	-844.681685
382-Ac$^+$_2	-847.095806	-846.676012	-845.057556	-27.55	-844.637763	-844.681666
382-Ac$^+$_3	-847.094841	-846.674985	-845.056326	-27.56	-844.636470	-844.680390
382-Ac$^+$_4	-847.094840	-846.674991	-845.056315	-27.54	-844.636466	-844.680354
**383**						-1584.721825
383_1	-1588.285390	-1587.924995	-1585.085573	7.57	-1584.722294	
383_2	-1588.294546	-1587.933999	-1585.083081	3.31	-1584.721275	
383_3	-1588.294453	-1587.933819	-1585.083140	3.64	-1584.721120	
383_4	-1588.280242	-1587.919944	-1585.080983	6.40	-1584.718247	
383_5	-1588.287273	-1587.926830	-1585.077348	1.00	-1584.716523	
383_6	-1588.286641	-1587.926165	-1585.077215	1.80	-1584.716053	
383_7	-1588.286486	-1587.925938	-1585.077096	1.34	-1584.716037	
383_8	-1588.284347	-1587.924094	-1585.071775	-9.25	-1584.715044	
**383-Ac$^+$**						-1737.364100
383-Ac$^+$_1	-1741.273285	-1740.858485	-1737.741174	-99.96	-1737.364445	
383-Ac$^+$_2	-1741.273221	-1740.858543	-1737.740388	-97.95	-1737.363017	
383-Ac$^+$_3	-1741.266890	-1740.852307	-1737.735856	-100.83	-1737.359679	
383-Ac$^+$_4	-1741.274732	-1740.859560	-1737.727750	-123.39	-1737.359573	

383-Ac$^+$_5	-1741.276775	-1740.861341	-1737.729497	-118.37	-1737.359146	
383-Ac$^+$_6	-1741.273378	-1740.858265	-1737.727391	-121.38	-1737.358508	
383-Ac$^+$_7	-1741.273409	-1740.858246	-1737.727364	-121.34	-1737.358416	
383-Ac$^+$_8	-1741.272771	-1740.857736	-1737.726798	-121.04	-1737.357866	
383-Ac$^+$_9	-1741.272773	-1740.857741	-1737.726768	-121.00	-1737.357823	
383-Ac$^+$_10	-1741.273007	-1740.858154	-1737.727087	-116.23	-1737.356505	
**384**						-1565.165014
384_1	-1569.423153	-1568.812959	-1565.785580	24.43	-1565.166079	
384_2	-1569.423897	-1568.813823	-1565.784863	24.06	-1565.165626	
384_3	-1569.416471	-1568.806523	-1565.781972	20.54	-1565.164200	
384_4	-1569.418389	-1568.808881	-1565.782299	23.10	-1565.163995	
384_5	-1569.417069	-1568.807055	-1565.781524	19.79	-1565.163972	
384_6	-1569.422489	-1568.812587	-1565.782267	22.55	-1565.163775	
384_7	-1569.419183	-1568.809427	-1565.783086	26.36	-1565.163290	
384_8	-1569.415513	-1568.805376	-1565.783279	26.02	-1565.163230	
384_9	-1569.415423	-1568.805615	-1565.783112	26.48	-1565.163216	
384_10	-1569.419540	-1568.809048	-1565.782558	23.85	-1565.162982	
**384-Ac$^+$**						-1717.808742
384-Ac$^+$_1	-1722.412842	-1721.747866	-1718.448540	-67.82	-1717.809397	
384-Ac$^+$_2	-1722.416753	-1721.752659	-1718.448210	-66.11	-1717.809296	
384-Ac$^+$_3	-1722.418811	-1721.753840	-1718.449764	-64.31	-1717.809286	
384-Ac$^+$_4	-1722.417503	-1721.752879	-1718.449044	-64.94	-1717.809153	
384-Ac$^+$_5	-1722.417503	-1721.752879	-1718.449043	-64.94	-1717.809152	
384-Ac$^+$_6	-1722.413553	-1721.748503	-1718.449947	-61.92	-1717.808482	
384-Ac$^+$_7	-1722.412895	-1721.748697	-1718.444820	-72.26	-1717.808143	
384-Ac$^+$_8	-1722.412477	-1721.748113	-1718.443559	-75.98	-1717.808134	
384-Ac$^+$_9	-1722.414067	-1721.749576	-1718.443841	-74.14	-1717.807589	
384-Ac$^+$_10	-1722.412250	-1721.748169	-1718.443646	-73.30	-1717.807485	
**385**						-1273.713229
385_1	-1276.646192	-1276.274479	-1274.083967	-4.18	-1273.713848	
385_2	-1276.646484	-1276.274391	-1274.082629	-4.64	-1273.712304	
385_3	-1276.652803	-1276.281307	-1274.079678	-7.61	-1273.711082	
385_4	-1276.642701	-1276.270715	-1274.081077	-4.73	-1273.710892	
385_5	-1276.652540	-1276.280945	-1274.079720	-6.95	-1273.710770	
385_6	-1276.652567	-1276.280910	-1274.079616	-6.15	-1273.710302	
385_7	-1276.652256	-1276.280459	-1274.079688	-4.98	-1273.709787	
385_8	-1276.648669	-1276.276966	-1274.075857	-9.00	-1273.707580	
385_9	-1276.648469	-1276.276796	-1274.075996	-7.45	-1273.707159	
**385-Ac$^+$**						-1426.358050
385-Ac$^+$_1	-1429.636696	-1429.210560	-1426.742103	-111.92	-1426.358595	
385-Ac$^+$_2	-1429.636549	-1429.210083	-1426.742158	-112.59	-1426.358576	
385-Ac$^+$_3	-1429.636834	-1429.210483	-1426.742368	-107.99	-1426.357148	
385-Ac$^+$_4	-1429.636578	-1429.210123	-1426.741964	-108.57	-1426.356862	
385-Ac$^+$_5	-1429.636578	-1429.210132	-1426.741950	-108.49	-1426.356826	
385-Ac$^+$_6	-1429.641606	-1429.215534	-1426.739869	-112.80	-1426.356761	
385-Ac$^+$_7	-1429.640949	-1429.214915	-1426.738920	-113.09	-1426.355961	
385-Ac$^+$_8	-1429.633008	-1429.206525	-1426.738490	-112.17	-1426.354732	
385-Ac$^+$_9	-1429.640289	-1429.214271	-1426.736434	-116.06	-1426.354622	
385-Ac$^+$_10	-1429.639696	-1429.213804	-1426.735780	-115.81	-1426.353999	
**386**						-990.305293
386_1	-993.016889	-992.613295	-990.709272	-0.33	-990.305805	
386_2	-993.026732	-992.623532	-990.707005	-3.89	-990.305287	
386_3	-993.026296	-992.623031	-990.705549	-5.19	-990.304261	
386_4	-993.026778	-992.623553	-990.706483	-1.92	-990.303991	
386_5	-993.023813	-992.620151	-990.703642	-3.60	-990.301350	

386_6	-993.022525	-992.619020	-990.702260	-6.49	-990.301226	
386_7	-993.023113	-992.619423	-990.703596	-2.97	-990.301037	
386_8	-993.009985	-992.606578	-990.702752	-3.31	-990.300603	
386_9	-993.010006	-992.606489	-990.701191	-3.10	-990.298853	
**386-Ac$^+$**						-1142.950561
386-Ac$^+$_1	-1146.011622	-1145.553539	-1143.369987	-103.55	-1142.951345	
386-Ac$^+$_2	-1146.020847	-1145.563102	-1143.368097	-105.65	-1142.950591	
386-Ac$^+$_3	-1146.011776	-1145.553691	-1143.369785	-100.54	-1142.949994	
386-Ac$^+$_4	-1146.017913	-1145.560112	-1143.364405	-112.93	-1142.949615	
386-Ac$^+$_5	-1146.018170	-1145.560290	-1143.364116	-112.88	-1142.949232	
386-Ac$^+$_6	-1146.017581	-1145.559823	-1143.363172	-112.38	-1142.948218	
386-Ac$^+$_7	-1146.006452	-1145.548454	-1143.364694	-108.16	-1142.947891	
386-Ac$^+$_8	-1146.006673	-1145.548746	-1143.364619	-107.61	-1142.947680	
386-Ac$^+$_9	-1146.006958	-1145.548815	-1143.365662	-104.18	-1142.947199	
**387**						-1740.218663
387_1	-1744.284109	-1743.826345	-1740.680601	10.38	-1740.218884	
387_2	-1744.283927	-1743.825878	-1740.680928	11.38	-1740.218544	
387_3	-1744.275711	-1743.818183	-1740.681784	15.10	-1740.218503	
387_4	-1744.269732	-1743.812360	-1740.677057	15.23	-1740.213884	
387_5	-1744.270097	-1743.813337	-1740.674869	14.31	-1740.212659	
**387-Ac$^+$**						-1892.865628
387-Ac$^+$_1	-1897.268588	-1896.756942	-1893.345766	-82.93	-1892.865705	
387-Ac$^+$_2	-1897.265973	-1896.75427	-1893.341298	-84.35	-1892.861722	
387-Ac$^+$_3	-1897.265161	-1896.752881	-1893.340591	-82.47	-1892.859721	
387-Ac$^+$_4	-1897.266552	-1896.754381	-1893.340332	-82.09	-1892.859427	
387-Ac$^+$_5	-1897.265901	-1896.753773	-1893.328081	-104.31	-1892.855681	
387-Ac$^+$_6	-1897.265527	-1896.753306	-1893.327164	-106.36	-1892.855452	
387-Ac$^+$_7	-1897.261455	-1896.749254	-1893.333955	-87.91	-1892.855235	
387-Ac$^+$_8	-1897.260641	-1896.749487	-1893.333560	-85.40	-1892.854931	
**388**						-743.697086
388_1	-745.789341	-745.476740	-744.007317	-7.32	-743.697505	
388_2	-745.786132	-745.473639	-744.004801	-6.61	-743.694826	
388_3	-745.786239	-745.473876	-744.005104	-6.78	-743.695323	
388_4	-745.784320	-745.472212	-744.004574	-5.52	-743.694569	
388-Ac$^+$_1						-896.342735
388-Ac$^+$_2	-898.783948	-898.416667	-896.667002	-108.07	-896.340884	
388-Ac$^+$_3	-898.780328	-898.413066	-896.663496	-107.49	-896.337173	
388-Ac$^+$_4	-898.782909	-898.415444	-896.665798	-107.78	-896.339384	
388-Ac$^+$_5	-898.785210	-898.417833	-896.668872	-109.54	-896.343215	
388-Ac$^+$_6	-898.781440	-898.414080	-896.665088	-109.24	-896.339337	
388-Ac$^+$_7	-898.784199	-898.416921	-896.667691	-109.29	-896.342037	
388-Ac$^+$_8	-1897.261455	-1896.749254	-1893.333955	-87.91	-1892.855235	
388-Ac$^+$_9	-1897.260641	-1896.749487	-1893.333560	-85.40	-1892.854931	
**389**						-1106.639006
389_1	-1109.710389	-1109.239257	-1107.111618	2.22	-1106.639641	
389_2	-1109.717647	-1109.246583	-1107.107426	-1.80	-1106.637047	
389_3	-1109.718429	-1109.247514	-1107.107708	-0.59	-1106.637016	
389_4	-1109.717447	-1109.246311	-1107.107703	-0.88	-1106.636901	
389_5	-1109.718298	-1109.247222	-1107.108049	0.92	-1106.636623	
389_6	-1109.717219	-1109.246398	-1107.105779	-3.85	-1106.636424	
389_7	-1109.717086	-1109.246146	-1107.105985	-3.10	-1106.636224	
389_8	-1109.709819	-1109.238618	-1107.102247	-3.01	-1106.632194	
389_9	-1109.711503	-1109.240266	-1107.100650	-7.07	-1106.632107	
389_10	-1109.709999	-1109.238952	-1107.101729	-3.22	-1106.631909	
**389-Ac$^+$**						-1259.285088

389-Ac$^+$_1	-1262.705793	-1262.180543	-1259.773476	-98.53	-1259.285755	
389-Ac$^+$_2	-1262.706096	-1262.180578	-1259.773460	-95.35	-1259.284260	
389-Ac$^+$_3	-1262.712980	-1262.187371	-1259.770484	-100.50	-1259.283154	
389-Ac$^+$_4	-1262.712980	-1262.187369	-1259.770481	-100.50	-1259.283149	
389-Ac$^+$_5	-1262.713320	-1262.187607	-1259.771098	-98.91	-1259.283058	
389-Ac$^+$_6	-1262.709628	-1262.184080	-1259.767444	-103.30	-1259.281242	
389-Ac$^+$_7	-1262.710557	-1262.185047	-1259.765669	-106.78	-1259.280828	
389-Ac$^+$_8	-1262.710557	-1262.185048	-1259.765666	-106.78	-1259.280826	
389-Ac$^+$_9	-1262.711127	-1262.185170	-1259.766543	-105.39	-1259.280729	
389-Ac$^+$_10	-1262.712327	-1262.186681	-1259.767835	-98.70	-1259.279782	
**390**						-1067.475665
390_1	-1070.423467	-1069.982556	-1067.915604	-3.64	-1067.476079	
390_2	-1070.423386	-1069.982330	-1067.915845	-2.55	-1067.475761	
390_3	-1070.413566	-1069.973034	-1067.914926	-1.46	-1067.474951	
390_4	-1070.413944	-1069.973160	-1067.914381	-2.80	-1067.474665	
390_5	-1070.416647	-1069.976280	-1067.909665	-2.85	-1067.470382	
390_6	-1070.415144	-1069.974447	-1067.907180	-9.20	-1067.469989	
390_7	-1070.415997	-1069.975389	-1067.909126	-2.38	-1067.469426	
390_8	-1070.414694	-1069.974084	-1067.906181	-9.67	-1067.469252	
390_9	-1070.415461	-1069.974210	-1067.908275	-5.06	-1067.468951	
390_10	-1070.417298	-1069.976682	-1067.908213	-3.10	-1067.468776	
**390-Ac$^+$**						-1220.122465
390-Ac$^+$_1	-1223.412017	-1222.916634	-1220.580324	-99.45	-1220.122821	
390-Ac$^+$_2	-1223.411551	-1222.916025	-1220.578576	-97.19	-1220.120070	
390-Ac$^+$_3	-1223.411550	-1222.916101	-1220.579484	-101.55	-1220.122711	
390-Ac$^+$_4	-1223.411260	-1222.916138	-1220.578013	-99.54	-1220.120803	
390-Ac$^+$_5	-1223.409480	-1222.914113	-1220.577280	-99.04	-1220.119633	
390-Ac$^+$_6	-1223.408020	-1222.912673	-1220.575804	-100.75	-1220.118831	
390-Ac$^+$_7	-1223.414189	-1222.918990	-1220.574269	-102.55	-1220.118130	
390-Ac$^+$_8	-1223.412727	-1222.917350	-1220.572754	-104.60	-1220.117217	
390-Ac$^+$_9	-1223.412252	-1222.917001	-1220.570616	-109.66	-1220.117133	
390-Ac$^+$_10	-1223.411813	-1222.916458	-1220.569769	-111.25	-1220.116788	
**391**						-1234.548690
391_1	-1237.349369	-1237.008059	-1234.887294	-8.08	-1234.549060	
391_2	-1237.353109	-1237.012007	-1234.884799	-9.29	-1234.547235	
391_3	-1237.352762	-1237.011295	-1234.884784	-8.54	-1234.546567	
391_4	-1237.344729	-1237.003506	-1234.878701	-11.17	-1234.541733	
391_5	-1237.345239	-1237.003885	-1234.878563	-11.72	-1234.541672	
391_6	-1237.344843	-1237.003367	-1234.878686	-10.79	-1234.541322	
391_7	-1237.342558	-1237.001353	-1234.874410	-18.07	-1234.540089	
**391-Ac$^+$**					-1387.195529	
391-Ac$^+$_1	-1390.341289	-1389.945337	-1387.548349	-113.64	-1387.195680	
391-Ac$^+$_2	-1390.340696	-1389.944628	-1387.546834	-110.67	-1387.192917	
391-Ac$^+$_3	-1390.338523	-1389.942546	-1387.539578	-117.32	-1387.188285	
391-Ac$^+$_4	-1390.340294	-1389.944269	-1387.539443	-116.73	-1387.187879	
391-Ac$^+$_5	-1390.340212	-1389.944253	-1387.539019	-117.65	-1387.187872	
391-Ac$^+$_6	-1390.337928	-1389.941812	-1387.538909	-117.57	-1387.187573	
391-Ac$^+$_7	-1390.339476	-1389.943424	-1387.541004	-111.04	-1387.187246	
391-Ac$^+$_8	-1390.338690	-1389.942862	-1387.539083	-115.27	-1387.187159	
391-Ac$^+$_9	-1390.338817	-1389.942724	-1387.540235	-110.88	-1387.186372	
391-Ac$^+$_10	-1429.633008	-1429.206525	-1426.738490	-112.17	-1426.354732	
**392**						-1740.218663
392_1	-1744.284109	-1743.826345	-1740.680601	10.38	-1740.218884	
392_2	-1744.283927	-1743.825878	-1740.680928	11.38	-1740.218544	
392_3	-1744.275711	-1743.818183	-1740.681784	15.10	-1740.218503	

392_4	-1744.269732	-1743.812360	-1740.677057	15.23	-1740.213884	
392_5	-1744.270097	-1743.813337	-1740.674869	14.31	-1740.212659	
**392-Ac$^+$**						-1892.865628
392-Ac$^+$_1	-1897.268588	-1896.756942	-1893.345766	-82.93	-1892.865705	
392-Ac$^+$_2	-1897.265973	-1896.75427	-1893.341298	-84.35	-1892.861722	
392-Ac$^+$_3	-1897.265161	-1896.752881	-1893.340591	-82.47	-1892.859721	
392-Ac$^+$_4	-1897.266552	-1896.754381	-1893.340332	-82.09	-1892.859427	
392-Ac$^+$_5	-1897.265901	-1896.753773	-1893.328081	-104.31	-1892.855681	
392-Ac$^+$_6	-1897.265527	-1896.753306	-1893.327164	-106.36	-1892.855452	
392-Ac$^+$_7	-1897.261455	-1896.749254	-1893.333955	-87.91	-1892.855235	
392-Ac$^+$_8	-1897.260641	-1896.749487	-1893.333560	-85.40	-1892.854931	
392-Ac$^+$_9	-1897.265260	-1896.753040	-1893.327202	-100.79	-1892.853372	
392-Ac$^+$_10	-1897.265518	-1896.753272	-1893.327986	-98.49	-1892.853254	
**393**						-951.139531
393_1	-953.720149	-953.346038	-951.512076	-6.32	-951.140372	
393_2	-953.728990	-953.354938	-951.510948	-6.65	-951.139429	
393_3	-953.729002	-953.354944	-951.510263	-8.03	-951.139264	
393_4	-953.727898	-953.354108	-951.508627	-10.50	-951.138837	
393_5	-953.720648	-953.346404	-951.511698	-3.51	-951.138792	
393_6	-953.727915	-953.354067	-951.508479	-10.79	-951.138742	
393_7	-953.716724	-953.342413	-951.509357	-6.78	-951.137627	
393_8	-953.725258	-953.351046	-951.507128	-9.41	-951.136501	
393_9	-953.724136	-953.350028	-951.505124	-12.09	-951.135621	
393_10	-953.721730	-953.347771	-951.502535	-18.33	-951.135556	
**393-Ac$^+$**						-1103.786798
393-Ac$^+$_1	-1106.714182	-1106.285660	-1104.172555	-114.35	-1103.787586	
393-Ac$^+$_2	-1106.714270	-1106.285678	-1104.172447	-113.76	-1103.787185	
393-Ac$^+$_3	-1106.714372	-1106.285911	-1104.172430	-111.34	-1103.786374	
393-Ac$^+$_4	-1106.714477	-1106.285912	-1104.172753	-110.00	-1103.786084	
393-Ac$^+$_5	-1106.721229	-1106.292731	-1104.171018	-114.01	-1103.785945	
393-Ac$^+$_6	-1106.721229	-1106.292733	-1104.171009	-113.97	-1103.785923	
393-Ac$^+$_7	-1106.720621	-1106.292151	-1104.170198	-113.97	-1103.785138	
393-Ac$^+$_8	-1106.717524	-1106.289247	-1104.167130	-118.41	-1103.783952	
393-Ac$^+$_9	-1106.718999	-1106.290627	-1104.166520	-120.21	-1103.783932	
393-Ac$^+$_10	-1106.720160	-1106.291615	-1104.166802	-119.24	-1103.783675	
**394**						-911.978235
394_1	-914.434079	-914.090468	-912.317918	-10.67	-911.978371	
394_2	-914.434029	-914.090433	-912.318133	-10.21	-911.978425	
394_3	-914.426811	-914.083226	-912.312367	-12.72	-911.973626	
394_4	-914.426240	-914.082568	-912.312192	-11.92	-911.973062	
394_5	-914.426045	-914.082395	-912.312277	-12.26	-911.973296	
394_6	-914.424404	-914.080903	-912.307462	-20.38	-911.971722	
394_7	-914.423689	-914.080334	-912.315623	-10.50	-911.976268	
394_8	-914.417843	-914.074596	-912.313649	-9.92	-911.974178	
**394-Ac$^+$**						-1064.625490
394-Ac$^+$_1	-1067.419733	-1067.021912	-1064.979632	-115.10	-1064.625651	
394-Ac$^+$_2	-1067.419506	-1067.021484	-1064.978040	-111.80	-1064.622599	
394-Ac$^+$_3	-1067.423787	-1067.025409	-1064.972701	-119.83	-1064.619964	
394-Ac$^+$_4	-1067.420543	-1067.022367	-1064.971265	-119.29	-1064.618522	
394-Ac$^+$_5	-1067.419955	-1067.021814	-1064.970518	-119.20	-1064.617779	
394-Ac$^+$_6	-1067.420060	-1067.021911	-1064.970339	-124.89	-1064.619759	
394-Ac$^+$_7	-1067.421393	-1067.023001	-1064.970543	-125.06	-1064.619783	
394-Ac$^+$_8	-1067.419465	-1067.021227	-1064.969697	-125.10	-1064.619108	
394-Ac$^+$_9	-1067.413825	-1067.015669	-1064.964263	-117.49	-1064.610856	
394-Ac$^+$_10	-1067.413409	-1067.015332	-1064.963629	-117.11	-1064.610157	

**395**						-2206.255203
395_1	-2212.521031	-2211.587771	-2207.214573	67.86	-2206.255465	
395_2	-2212.524033	-2211.590934	-2207.203380	42.55	-2206.254074	
395_3	-2212.521634	-2211.588352	-2207.196322	38.58	-2206.248347	
395_4	-2212.520811	-2211.587683	-2207.195645	45.81	-2206.245067	
**395-Ac$^+$**						-2358.904362
395-Ac$^+$_1	-2365.531860	-2364.543720	-2359.878651	-37.03	-2358.904614	
395-Ac$^+$_2	-2365.532457	-2364.544401	-2359.879792	-32.47	-2358.904103	
395-Ac$^+$_3	-2365.526447	-2364.539858	-2359.883327	-12.22	-2358.901391	
395-Ac$^+$_4	-2365.530534	-2364.542603	-2359.876213	-22.38	-2358.896808	
395-Ac$^+$_5	-2365.530981	-2364.543013	-2359.874073	-26.28	-2358.896113	
395-Ac$^+$_6	-2365.528125	-2364.539985	-2359.869431	-38.66	-2358.896016	
**396**						-1405.990947
396_1	-1409.421763	-1408.937279	-1406.486458	6.86	-1406.001973	-1405.991041
396_2	-1409.421317	-1408.936855	-1406.486333	6.93	-1406.001871	-1405.990828
**396-Ac$^+$**						-1558.640320
396-Ac$^+$_1	-1562.425032	-1561.885771	-1559.154996	-15.69	-1558.615736	-1558.640740
396-Ac$^+$_2	-1562.424062	-1561.884777	-1559.154521	-15.55	-1558.615235	-1558.640016
396-Ac$^+$_3	-1562.424044	-1561.886128	-1559.153583	-14.92	-1558.615667	-1558.639443
396-Ac$^+$_4	-1562.422706	-1561.883431	-1559.152478	-14.84	-1558.613202	-1558.636852
**397**						-1217.600993
397_1	-1220.818907	-1220.367388	-1218.055831	7.28	-1217.601540	
397_2	-1220.818626	-1220.367166	-1218.054727	4.73	-1217.601466	
397_3	-1220.817798	-1220.366737	-1218.052799	2.68	-1217.600718	
397_4	-1220.818592	-1220.367420	-1218.051629	-0.04	-1217.600473	
397_5	-1220.818546	-1220.367443	-1218.051845	0.79	-1217.600439	
397_6	-1220.817258	-1220.365975	-1218.052742	6.19	-1217.599101	
397_7	-1220.817017	-1220.365884	-1218.050281	2.64	-1217.598144	
397_8	-1220.816511	-1220.365417	-1218.051169	5.27	-1217.598067	
397_9	-1220.815538	-1220.364317	-1218.049583	1.09	-1217.597948	
397_10	-1220.817055	-1220.365854	-1218.050277	4.18	-1217.597483	
**397-Ac$^+$**						-1370.250656
397-Ac$^+$_1	-1373.819870	-1373.313895	-1370.723518	-88.53	-1370.251264	
397-Ac$^+$_2	-1373.819816	-1373.313527	-1370.721354	-90.46	-1370.249519	
397-Ac$^+$_3	-1373.819291	-1373.313231	-1370.720949	-90.17	-1370.249231	
397-Ac$^+$_4	-1373.812755	-1373.306715	-1370.721301	-86.73	-1370.248296	
397-Ac$^+$_5	-1373.812494	-1373.306331	-1370.721581	-85.73	-1370.248070	
397-Ac$^+$_6	-1373.812936	-1373.307616	-1370.715111	-96.48	-1370.246539	
397-Ac$^+$_7	-1373.813435	-1373.307616	-1370.715299	-96.99	-1370.246420	
397-Ac$^+$_8	-1373.812549	-1373.306560	-1370.718810	-87.15	-1370.246015	
397-Ac$^+$_9	-1373.812139	-1373.306133	-1370.714567	-92.63	-1370.243843	
397-Ac$^+$_10	-1373.812196	-1373.306219	-1370.710882	-97.95	-1370.242211	
**352**						-1284.973428
352_1	-1288.674630	-1288.083688	-1285.568297	9.71	-1284.973658	
352_2	-1288.673958	-1288.082890	-1285.567733	13.05	-1284.971692	
352_3	-1288.672207	-1288.081221	-1285.565655	20.92	-1284.966701	
352_4	-1288.672877	-1288.081718	-1285.564962	16.40	-1284.967556	
**352-Ac$^+$**						-1437.624915
352-Ac$^+$_1	-1441.681515	-1441.035695	-1438.241929	-75.90	-1437.625017	
352-Ac$^+$_2	-1441.680630	-1441.034903	-1438.240962	-78.78	-1437.625243	
352-Ac$^+$_3	-1441.680095	-1441.033915	-1438.240092	-75.14	-1437.622534	
352-Ac$^+$_4	-1441.679775	-1441.033834	-1438.240106	-77.19	-1437.623567	
352-Ac$^+$_5	-1441.680924	-1441.034925	-1438.240386	-67.24	-1437.619996	
352-Ac$^+$_6	-1441.677032	-1441.032019	-1438.235746	-69.58	-1437.617235	
352-Ac$^+$_7	-1441.679966	-1441.033950	-1438.238863	-70.33	-1437.619635	

352-Ac$^+$_8	-1441.678282	-1441.031988	-1438.236760	-75.65	-1437.619278	
**350**	-514.820620	-514.593193	-513.593048	-4.48	-513.365621	-513.372761
**350-Ac$^+$**						-666.025137
350-Ac$^+$_1	-667.820735	-667.538347	-666.258398	-30.97	-665.976010	-666.025364
350-Ac$^+$_2	-667.819559	-667.537128	-666.256714	-30.88	-665.974283	-666.023493
**398**						-1441.641383
398_1	-1445.880901	-1445.172693	-1442.357138	19.37	-1441.641552	
398_2	-1445.880375	-1445.171564	-1442.356300	22.34	-1441.638979	
398_3	-1445.878714	-1445.170331	-1442.355406	32.72	-1441.634561	
398_4	-1445.879184	-1445.170629	-1442.353600	25.73	-1441.635244	
**398-Ac$^+$**						-1594.293769
398-Ac$^+$_1	-1598.889925	-1598.126495	-1595.033168	-63.97	-1594.294104	
398-Ac$^+$_2	-1598.891052	-1598.127564	-1595.034445	-60.54	-1594.294016	
398-Ac$^+$_3	-1598.889015	-1598.125839	-1595.032196	-62.17	-1594.292701	
398-Ac$^+$_4	-1598.889646	-1598.126274	-1595.032726	-59.87	-1594.292159	
398-Ac$^+$_5	-1598.890016	-1598.126825	-1595.033191	-54.73	-1594.290844	
398-Ac$^+$_6	-1598.889550	-1598.126017	-1595.031496	-55.06	-1594.288935	
398-Ac$^+$_7	-1598.885235	-1598.121800	-1595.027104	-49.54	-1594.282537	
**59**						-668.869492
59_1	-670.809509	-670.485967	-669.188063	-3.32	-668.864521	-668.869812
59_2	-670.810555	-670.485932	-669.188397	-3.47	-668.863774	-668.869304
**59-Ac$^+$**						-821.522525
59-Ac$^+$_1	-823.814747	-823.434833	-821.857811	-28.39	-821.477897	-821.523139
59-Ac$^+$_2	-823.813008	-823.433445	-821.856756	-28.39	-821.477193	-821.522435
59-Ac$^+$_3	-823.812867	-823.433163	-821.856423	-28.36	-821.476719	-821.521913
59-Ac$^+$_4	-823.813532	-823.433843	-821.856130	-28.25	-821.476441	-821.521460
59-Ac$^+$_5	-823.811898	-823.432430	-821.855091	-28.21	-821.475623	-821.520579
59-Ac$^+$_6	-823.811762	-823.432069	-821.854807	-28.22	-821.475114	-821.520086
**399**						-1129.494665
399_1	-1132.719571	-1132.223683	-1129.991588	0.59	-1129.495476	
399_2	-1132.719743	-1132.224095	-1129.990748	1.21	-1129.494637	
399_3	-1132.717991	-1132.222095	-1129.989202	1.38	-1129.492781	
399_4	-1132.717803	-1132.222100	-1129.988772	2.05	-1129.492289	
399_5	-1132.718542	-1132.222495	-1129.990984	2.51	-1129.493981	
399_6	-1132.717516	-1132.222752	-1129.987748	3.39	-1129.491693	
399_7	-1132.720046	-1132.223977	-1129.992325	4.06	-1129.494711	
399_8	-1132.719620	-1132.223722	-1129.991243	4.18	-1129.493751	
399_9	-1132.718643	-1132.222572	-1129.990748	5.31	-1129.492652	
399_10	-1132.718590	-1132.222209	-1129.991153	5.98	-1129.492492	
**399-Ac$^+$**						-1282.147863
399-Ac$^+$_1	-1285.726811	-1285.175964	-1282.666285	-86.73	-1282.148474	
399-Ac$^+$_2	-1285.724706	-1285.173928	-1282.663656	-93.43	-1282.148463	
399-Ac$^+$_3	-1285.725949	-1285.175058	-1282.665334	-89.04	-1282.148355	
399-Ac$^+$_4	-1285.725437	-1285.174418	-1282.665419	-88.32	-1282.148040	
399-Ac$^+$_5	-1285.725512	-1285.174914	-1282.664508	-89.04	-1282.147821	
399-Ac$^+$_6	-1285.722401	-1285.173515	-1282.661184	-92.68	-1282.147596	
399-Ac$^+$_7	-1285.724411	-1285.173429	-1282.663035	-90.71	-1282.146602	
399-Ac$^+$_8	-1285.724972	-1285.173806	-1282.663722	-88.99	-1282.146452	
399-Ac$^+$_9	-1285.725819	-1285.174714	-1282.664002	-87.86	-1282.146363	
399-Ac$^+$_10	-1285.724837	-1285.173744	-1282.662904	-90.63	-1282.146328	
**351**						-1207.819503
351_1	-1211.312399	-1210.756414	-1208.381461	13.60	-1207.820297	
351_2	-1211.312697	-1210.756905	-1208.380677	12.47	-1207.820136	
351_3	-1211.311004	-1210.755073	-1208.380786	13.14	-1207.819851	
351_4	-1211.310918	-1210.754982	-1208.380161	12.43	-1207.819491	

351_5	-1211.310484	-1210.755045	-1208.378297	10.04	-1207.819033	
351_6	-1211.307244	-1210.751179	-1208.380018	13.93	-1207.818646	
351_7	-1211.306988	-1210.751164	-1208.379320	13.72	-1207.818269	
351_8	-1211.310772	-1210.754625	-1208.379032	12.97	-1207.817945	
351_9	-1211.309577	-1210.754309	-1208.376787	9.58	-1207.817869	
351_10	-1211.309836	-1210.753816	-1208.379027	13.72	-1207.817780	
**351-Ac$^+$**						-1360.472770
351-Ac$^+$_1	-1364.319820	-1363.708516	-1361.055082	-77.66	-1360.473355	
351-Ac$^+$_2	-1364.316651	-1363.705476	-1361.056803	-72.72	-1360.473325	
351-Ac$^+$_3	-1364.315092	-1363.703943	-1361.055270	-75.31	-1360.472806	
351-Ac$^+$_4	-1364.318656	-1363.707628	-1361.054256	-77.28	-1360.472662	
351-Ac$^+$_5	-1364.318791	-1363.707870	-1361.053435	-77.86	-1360.472172	
351-Ac$^+$_6	-1364.317774	-1363.706771	-1361.052658	-79.16	-1360.471806	
351-Ac$^+$_7	-1364.314227	-1363.703035	-1361.053552	-76.94	-1360.471666	
351-Ac$^+$_8	-1364.308866	-1363.697456	-1361.049916	-86.11	-1360.471303	
351-Ac$^+$_9	-1364.317938	-1363.706999	-1361.053512	-74.27	-1360.470859	
351-Ac$^+$_10	-1364.318127	-1363.707324	-1361.053333	-73.35	-1360.470466	
**58**						-591.698885
58_1	-593.418065	-593.130880	-591.981245	-14.56	-591.699606	
58_2	-593.417088	-593.130024	-591.980224	-14.52	-591.698690	
58_3	-593.416648	-593.129499	-591.979898	-14.18	-591.698150	
58_4	-593.415838	-593.128722	-591.978867	-14.14	-591.697137	
58_5	-593.415488	-593.128357	-591.979020	-15.06	-591.697625	
58_6	-593.415277	-593.127870	-591.978978	-15.19	-591.697357	
58_7	-593.414082	-593.126927	-591.977706	-14.56	-591.696097	
**58-Ac$^+$**						-744.352678
58-Ac$^+$_1	-746.420416	-746.078238	-744.649502	-121.08	-744.353441	
58-Ac$^+$_2	-746.420285	-746.077937	-744.649302	-121.29	-744.353151	
58-Ac$^+$_3	-746.419224	-746.076995	-744.648332	-121.13	-744.352239	
58-Ac$^+$_4	-746.41917	-746.07687	-744.648337	-121.50	-744.352314	
58-Ac$^+$_5	-746.419086	-746.076798	-744.648046	-121.17	-744.351909	
58-Ac$^+$_6	-746.419086	-746.076799	-744.648047	-121.17	-744.351911	
58-Ac$^+$_7	-746.419131	-746.076892	-744.64761	-120.12	-744.351122	
58-Ac$^+$_8	-746.419131	-746.076893	-744.64761	-120.08	-744.351108	
58-Ac$^+$_9	-746.418954	-746.076873	-744.647375	-120.33	-744.351125	
58-Ac$^+$_10	-746.417975	-746.075703	-744.646556	-120.16	-744.350051	
**400**						-783.954589
400_1	-786.211003	-785.845253	-784.319500	-4.35	-783.955407	
400_2	-786.210144	-785.844351	-784.318658	-5.82	-783.955080	
400_3	-786.209518	-785.843845	-784.317937	-5.90	-783.954510	
400_4	-786.208937	-785.843458	-784.317282	-5.82	-783.954018	
400_5	-786.207914	-785.842026	-784.317745	-4.85	-783.953705	
400_6	-786.208914	-785.843775	-784.317084	-4.39	-783.953619	
400_7	-786.208438	-785.843093	-784.317385	-3.89	-783.953522	
400_8	-786.208416	-785.842967	-784.317316	-4.02	-783.953397	
400_9	-786.206906	-785.841416	-784.315807	-7.49	-783.953169	
400_10	-786.207729	-785.842314	-784.316707	-3.89	-783.952773	
**400-Ac$^+$**						-936.608509
400-Ac$^+$_1	-939.215919	-938.795126	-936.990756	-103.34	-936.609326	
400-Ac$^+$_2	-939.214397	-938.793616	-936.989281	-105.90	-936.608835	
400-Ac$^+$_3	-939.215697	-938.794921	-936.990057	-101.00	-936.607751	
400-Ac$^+$_4	-939.212999	-938.792484	-936.987507	-106.40	-936.607518	
400-Ac$^+$_5	-939.214299	-938.793614	-936.988645	-103.60	-936.607417	
400-Ac$^+$_6	-939.213085	-938.792539	-936.987561	-105.39	-936.607158	
400-Ac$^+$_7	-939.211901	-938.791668	-936.986127	-107.53	-936.606849	

400-Ac$^+$_8	-939.212274	-938.791416	-936.987575	-103.97	-936.606317	
400-Ac$^+$_9	-939.213104	-938.792579	-936.987082	-104.01	-936.606174	
400-Ac$^+$_10	-939.213226	-938.792736	-936.987278	-103.09	-936.606055	
**401**						-1217.910363
401_1	-1221.300472	-1220.787113	-1218.426019	2.43	-1217.911735	
401_2	-1221.302906	-1220.789129	-1218.420923	-7.53	-1217.910014	
401_3	-1221.302230	-1220.788302	-1218.420066	-8.79	-1217.909485	
401_4	-1221.299409	-1220.786171	-1218.417271	-14.02	-1217.909371	
401_5	-1221.300922	-1220.787456	-1218.420254	-5.61	-1217.908924	
401_6	-1221.300735	-1220.786939	-1218.421235	-3.60	-1217.908809	
401_7	-1221.299636	-1220.786175	-1218.420075	-5.44	-1217.908686	
401_8	-1221.302848	-1220.789187	-1218.421419	-2.43	-1217.908683	
401_9	-1221.300369	-1220.786137	-1218.419442	-8.74	-1217.908541	
401_10	-1221.299675	-1220.786121	-1218.418254	-9.67	-1217.908382	
**401-Ac$^+$**						-1370.564416
401-Ac$^+$_1	-1374.313136	-1373.744397	-1371.099821	-90.63	-1370.565599	
401-Ac$^+$_2	-1374.312839	-1373.744081	-1371.097101	-96.73	-1370.565187	
401-Ac$^+$_3	-1374.312327	-1373.743559	-1371.097518	-93.18	-1370.564240	
401-Ac$^+$_4	-1374.312730	-1373.743987	-1371.095838	-97.40	-1370.564194	
401-Ac$^+$_5	-1374.314494	-1373.745936	-1371.097840	-90.96	-1370.563927	
401-Ac$^+$_6	-1374.311409	-1373.742739	-1371.095803	-96.40	-1370.563850	
401-Ac$^+$_7	-1374.310765	-1373.741953	-1371.095977	-95.81	-1370.563659	
401-Ac$^+$_8	-1374.310528	-1373.741791	-1371.095227	-96.90	-1370.563397	
401-Ac$^+$_9	-1374.310528	-1373.741791	-1371.095227	-96.90	-1370.563397	
401-Ac$^+$_10	-1374.315742	-1373.746520	-1371.099042	-88.07	-1370.563366	
**402**						-748.348429
402_1	-750.606553	-750.200824	-748.755443	1.00	-748.349332	
402_2	-750.607112	-750.201112	-748.755673	2.01	-748.348908	
402_3	-750.606375	-750.200488	-748.755172	1.13	-748.348854	
402_4	-750.605976	-750.200302	-748.754886	1.17	-748.348766	
402_5	-750.605854	-750.199821	-748.754930	0.46	-748.348722	
402_6	-750.605703	-750.199816	-748.754722	0.88	-748.348501	
402_7	-750.606566	-750.200615	-748.754755	1.72	-748.348151	
402_8	-750.605748	-750.200113	-748.754430	0.75	-748.348508	
402_9	-750.604419	-750.198622	-748.754286	0.54	-748.348282	
402_10	-750.604956	-750.199256	-748.754038	-0.29	-748.348450	
**402-Ac$^+$**						-901.002684
402-Ac$^+$_1	-903.612034	-903.151115	-901.427163	-97.91	-901.003535	
402-Ac$^+$_2	-903.611153	-903.150164	-901.426547	-99.87	-901.003598	
402-Ac$^+$_3	-903.611208	-903.150313	-901.426323	-98.66	-901.003006	
402-Ac$^+$_4	-903.611285	-903.150338	-901.426274	-100.58	-901.003637	
402-Ac$^+$_5	-903.610912	-903.149873	-901.425934	-99.66	-901.002854	
402-Ac$^+$_6	-903.611202	-903.150244	-901.425667	-98.66	-901.002287	
402-Ac$^+$_7	-903.610538	-903.149472	-901.425300	-101.55	-901.002910	
402-Ac$^+$_8	-903.610131	-903.149100	-901.425047	-101.09	-901.002517	
402-Ac$^+$_9	-903.609317	-903.148009	-901.425265	-100.12	-901.002091	
402-Ac$^+$_10	-903.609918	-903.149110	-901.424762	-97.82	-901.001212	
**403**						-1257.076545
403_1	-1260.602137	-1260.058365	-1257.620850	1.38	-1257.076552	
403_2	-1260.601213	-1260.057975	-1257.620677	1.09	-1257.077025	
403_3	-1260.600915	-1260.057214	-1257.618366	1.67	-1257.074027	
403_4	-1260.600412	-1260.057138	-1257.615224	-0.21	-1257.072030	
403_5	-1260.600078	-1260.056245	-1257.610102	-13.33	-1257.071352	
403_6	-1260.600056	-1260.056072	-1257.610029	-11.54	-1257.070443	
403_7	-1260.599983	-1260.056139	-1257.609818	-12.87	-1257.070882	

403_8	-1260.599965	-1260.056226	-1257.610036	-11.50	-1257.070680	
403_9	-1260.599808	-1260.056160	-1257.619214	3.43	-1257.074259	
403_10	-1260.599736	-1260.056515	-1257.620430	5.68	-1257.075041	
**403-Ac$^+$**						-1409.732015
403-Ac$^+$_1	-1413.606752	-1413.011180	-1410.297917	-79.55	-1409.732642	
403-Ac$^+$_2	-1413.609595	-1413.012340	-1410.295485	-86.74	-1409.731265	
403-Ac$^+$_3	-1413.608038	-1413.010206	-1410.293140	-91.58	-1409.730190	
403-Ac$^+$_4	-1413.609078	-1413.010161	-1410.294617	-89.87	-1409.729930	
403-Ac$^+$_5	-1413.608995	-1413.010886	-1410.294801	-86.02	-1409.729457	
403-Ac$^+$_6	-1413.608656	-1413.010277	-1410.294521	-85.02	-1409.728524	
403-Ac$^+$_7	-1413.606717	-1413.009431	-1410.289401	-93.55	-1409.727746	
403-Ac$^+$_8	-1413.609880	-1413.011223	-1410.293570	-85.65	-1409.727534	
403-Ac$^+$_9	-1413.615106	-1413.016425	-1410.294145	-82.85	-1409.727019	
403-Ac$^+$_10	-1413.608302	-1413.009669	-1410.293198	-84.69	-1409.726821	
**404**						-938.344926
404_1	-941.062462	-940.622577	-938.783610	-5.06	-938.345653	
404_2	-941.062845	-940.622744	-938.783815	-4.48	-938.345420	
404_3	-941.062964	-940.622926	-938.784047	-2.80	-938.345077	
404_4	-941.061682	-940.621588	-938.783857	-2.59	-938.344751	
404_5	-941.063112	-940.622726	-938.784121	-1.38	-938.344262	
404_6	-941.062892	-940.622604	-938.784221	-0.75	-938.344219	
404_7	-941.062444	-940.622379	-938.783124	-2.38	-938.343968	
404_8	-941.063065	-940.623052	-938.783669	-0.29	-938.343768	
404_9	-941.062076	-940.622092	-938.782384	-2.97	-938.343532	
404_10	-941.061804	-940.621404	-938.782401	-3.47	-938.343323	
**404-Ac$^+$**						-1090.999723
404-Ac$^+$_1	-1094.070524	-1093.575470	-1091.459203	-95.48	-1091.000515	
404-Ac$^+$_2	-1094.069510	-1093.574222	-1091.458027	-98.74	-1091.000348	
404-Ac$^+$_3	-1094.069039	-1093.573899	-1091.456561	-99.91	-1090.999477	
404-Ac$^+$_4	-1094.069156	-1093.573911	-1091.457714	-97.11	-1090.999457	
404-Ac$^+$_5	-1094.068112	-1093.572822	-1091.456644	-100.00	-1090.999441	
404-Ac$^+$_6	-1094.068051	-1093.573005	-1091.455343	-101.88	-1090.999100	
404-Ac$^+$_7	-1094.069887	-1093.574522	-1091.457509	-96.52	-1090.998909	
404-Ac$^+$_8	-1094.068873	-1093.573644	-1091.456366	-98.99	-1090.998842	
404-Ac$^+$_9	-1094.069398	-1093.573913	-1091.456641	-97.40	-1090.998255	
404-Ac$^+$_10	-1094.070036	-1093.574785	-1091.457152	-94.14	-1090.997757	
**405**						-903.840410
405_1	-906.595579	-906.091657	-904.345117	-1.42	-903.841737	
405_2	-906.595372	-906.091429	-904.344356	-3.43	-903.841719	
405_3	-906.595387	-906.090992	-904.345404	-0.29	-903.841120	
405_4	-906.594764	-906.090325	-904.344489	-1.97	-903.840800	
405_5	-906.594103	-906.090168	-904.343978	-1.80	-903.840729	
405_6	-906.594275	-906.090044	-904.343854	-2.22	-903.840467	
405_7	-906.594448	-906.090614	-904.343052	-2.68	-903.840238	
405_8	-906.594376	-906.089987	-904.343771	-2.09	-903.840179	
405_9	-906.593174	-906.088977	-904.345278	2.55	-903.840110	
405_10	-906.594709	-906.090505	-904.343251	-2.59	-903.840035	
**405-Ac$^+$**						-1056.495463
405-Ac$^+$_1	-1059.604369	-1059.044723	-1057.018841	-98.58	-1056.496740	
405-Ac$^+$_2	-1059.603285	-1059.044040	-1057.018766	-96.36	-1056.496222	
405-Ac$^+$_3	-1059.602986	-1059.043743	-1057.018176	-97.07	-1056.495905	
405-Ac$^+$_4	-1059.603144	-1059.043872	-1057.018425	-96.27	-1056.495821	
405-Ac$^+$_5	-1059.603134	-1059.043647	-1057.018231	-96.90	-1056.495652	
405-Ac$^+$_6	-1059.602284	-1059.042811	-1057.018814	-94.27	-1056.495245	
405-Ac$^+$_7	-1059.601250	-1059.042257	-1057.016767	-98.11	-1056.495144	

405-Ac$^+$_8	-1059.601422	-1059.042034	-1057.016358	-99.66	-1056.494931	
405-Ac$^+$_9	-1059.601853	-1059.042393	-1057.017464	-96.36	-1056.494705	
405-Ac$^+$_10	-1059.601652	-1059.042745	-1057.015841	-98.74	-1056.494542	
**406**						-825.516838
406_1	-828.001801	-827.557348	-825.963011	1.59	-825.517952	
406_2	-828.002033	-827.557927	-825.961857	-0.29	-825.517862	
406_3	-828.000917	-827.556782	-825.962331	1.59	-825.517591	
406_4	-828.000859	-827.556620	-825.961300	-0.50	-825.517252	
406_5	-827.998449	-827.554199	-825.961499	0.54	-825.517042	
406_6	-827.998623	-827.554291	-825.961033	-0.29	-825.516813	
406_7	-827.998803	-827.554666	-825.960348	-1.13	-825.516641	
406_8	-827.999580	-827.555168	-825.960747	-0.67	-825.516590	
406_9	-827.998462	-827.554037	-825.960445	-1.38	-825.516546	
406_10	-827.999940	-827.555453	-825.961145	0.38	-825.516515	
**406-Ac$^+$**						-978.172026
406-Ac$^+$_1	-981.009738	-980.510214	-978.635898	-97.11	-978.173362	
406-Ac$^+$_2	-981.008871	-980.509343	-978.636382	-95.14	-978.173092	
406-Ac$^+$_3	-981.007715	-980.508061	-978.635139	-96.73	-978.172330	
406-Ac$^+$_4	-981.008161	-980.508659	-978.634270	-98.37	-978.172234	
406-Ac$^+$_5	-981.006058	-980.506745	-978.633412	-97.57	-978.171261	
406-Ac$^+$_6	-981.005256	-980.505950	-978.632709	-99.29	-978.171219	
406-Ac$^+$_7	-981.007559	-980.508157	-978.633548	-97.07	-978.171118	
406-Ac$^+$_8	-981.005275	-980.505633	-978.633051	-98.62	-978.170970	
406-Ac$^+$_9	-981.008377	-980.508860	-978.633957	-94.98	-978.170614	
406-Ac$^+$_10	-981.005387	-980.505514	-978.634049	-95.56	-978.170573	
**63**						-747.195045
63_1	-749.405682	-749.021776	-747.576377	-1.97	-747.192470	-747.195610
63_2	-749.404877	-749.020263	-747.576944	-1.99	-747.192329	-747.195501
63_3	-749.407023	-749.022449	-747.576974	-1.92	-747.192400	-747.195459
63_4	-749.406234	-749.021772	-747.576561	-1.91	-747.192098	-747.195142
63_5	-749.405930	-749.021754	-747.576291	-1.88	-747.192115	-747.195111
63_6	-749.404995	-749.020441	-747.576374	-1.95	-747.191820	-747.194928
63_7	-749.406393	-749.021713	-747.576140	-2.10	-747.191460	-747.194807
63_8	-749.406385	-749.021639	-747.576067	-2.09	-747.191321	-747.194652
63_9	-749.404830	-749.020098	-747.576215	-1.96	-747.191483	-747.194607
63_10	-749.404828	-749.020084	-747.576198	-1.96	-747.191453	-747.194577
**63-Ac$^+$**						-899.850265
63-Ac$^+$_1	-902.413587	-901.973819	-900.249700	-25.92	-899.809932	-899.851239
63-Ac$^+$_2	-902.412031	-901.972378	-900.248054	-26.35	-899.808401	-899.850393
63-Ac$^+$_3	-902.411213	-901.971863	-900.247362	-26.04	-899.808012	-899.849509
63-Ac$^+$_4	-902.411402	-901.971872	-900.247304	-25.89	-899.807774	-899.849032
63-Ac$^+$_5	-902.410014	-901.970639	-900.246090	-26.40	-899.806715	-899.848786
63-Ac$^+$_6	-902.410906	-901.971372	-900.246750	-26.00	-899.807216	-899.848650
63-Ac$^+$_7	-902.410568	-901.970739	-900.247252	-25.80	-899.807423	-899.848538
63-Ac$^+$_8	-902.410654	-901.971044	-900.246195	-26.11	-899.806585	-899.848194
63-Ac$^+$_9	-902.410757	-901.971222	-900.246346	-25.88	-899.806810	-899.848053
63-Ac$^+$_10	-902.410757	-901.971222	-900.246346	-25.88	-899.806810	-899.848053
**407**						-590.547164
407_1	-592.222726	-591.957797	-590.804005	-5.24	-590.539076	-590.547426
407_2	-592.221494	-591.956591	-590.802484	-5.38	-590.537581	-590.546155
**407-Ac$^+$**						-743.202992
407-Ac$^+$_1	-745.226542	-744.906419	-743.473561	-31.37	-743.153438	-743.203429
407-Ac$^+$_2	-745.226382	-744.906245	-743.473132	-31.35	-743.152996	-743.202955
407-Ac$^+$_3	-745.225730	-744.905688	-743.472321	-31.24	-743.152280	-743.202064
407-Ac$^+$_4	-745.225508	-744.905414	-743.471922	-31.19	-743.151829	-743.201533

**408**						-629.709795
408_1	-631.521285	-631.226469	-629.997804	-4.71	-629.702988	-629.710494
408_2	-631.519929	-631.225072	-629.996450	-4.61	-629.701594	-629.708940
408_3	-631.518936	-631.224066	-629.995691	-4.90	-629.700821	-629.708630
408_4	-631.519580	-631.224611	-629.995589	-4.87	-629.700620	-629.708381
408_5	-631.518089	-631.223278	-629.994812	-4.96	-629.700001	-629.707905
408_6	-631.518347	-631.223563	-629.994767	-4.76	-629.699983	-629.707569
**408-Ac$^+$**						-782.365897
408-Ac$^+$_1	-784.525551	-784.175677	-782.668096	-30.39	-782.318222	-782.366652
408-Ac$^+$_2	-784.525570	-784.175622	-782.668009	-30.34	-782.318061	-782.366411
408-Ac$^+$_3	-784.524416	-784.174632	-782.666991	-30.42	-782.317207	-782.365684
408-Ac$^+$_4	-784.524425	-784.174211	-782.667069	-30.51	-782.316854	-782.365475
408-Ac$^+$_5	-784.524351	-784.174539	-782.666772	-30.33	-782.316960	-782.365294
408-Ac$^+$_6	-784.524123	-784.173948	-782.666624	-30.47	-782.316449	-782.365006
408-Ac$^+$_7	-784.524617	-784.174740	-782.666634	-30.16	-782.316757	-782.364820
408-Ac$^+$_8	-784.524536	-784.174700	-782.666386	-30.10	-782.316550	-782.364518
408-Ac$^+$_9	-784.523541	-784.173341	-782.665821	-30.37	-782.315621	-782.364019
408-Ac$^+$_10	-784.523397	-784.173384	-782.665597	-30.14	-782.315585	-782.363616
**409**						-1084.668315
409_1	-1087.652398	-1087.200677	-1085.121493	1.88	-1084.669055	
409_2	-1087.651791	-1087.200278	-1085.120114	4.52	-1084.666879	
409_3	-1087.649225	-1087.197328	-1085.119494	3.68	-1084.666195	
409_4	-1087.651631	-1087.199821	-1085.118574	-0.29	-1084.666876	
409_5	-1087.649246	-1087.197399	-1085.118472	1.67	-1084.665987	
409_6	-1087.647362	-1087.196005	-1085.116869	3.05	-1084.664349	
409_7	-1087.649716	-1087.198264	-1085.116692	2.80	-1084.664173	
409_8	-1087.648282	-1087.196410	-1085.116456	0.46	-1084.664409	
409_9	-1087.650401	-1087.199026	-1085.115575	-1.00	-1084.664582	
409_10	-1087.648878	-1087.197476	-1085.115318	0.59	-1084.663693	
**409-Ac$^+$**						-1237.324964
409-Ac$^+$_1	-1240.658226	-1240.151779	-1237.794427	-98.37	-1237.325445	
409-Ac$^+$_2	-1240.656011	-1240.149484	-1237.793683	-95.31	-1237.323459	
409-Ac$^+$_3	-1240.655066	-1240.148568	-1237.792044	-98.58	-1237.323091	
409-Ac$^+$_4	-1240.656859	-1240.150413	-1237.792807	-90.88	-1237.320974	
409-Ac$^+$_5	-1240.659746	-1240.153034	-1237.792123	-93.01	-1237.320836	
409-Ac$^+$_6	-1240.656875	-1240.150481	-1237.793081	-88.45	-1237.320375	
409-Ac$^+$_7	-1240.659973	-1240.153007	-1237.792199	-92.22	-1237.320356	
409-Ac$^+$_8	-1240.657664	-1240.151125	-1237.792033	-90.92	-1237.320123	
409-Ac$^+$_9	-1240.659296	-1240.152651	-1237.791337	-90.25	-1237.319065	
409-Ac$^+$_10	-1240.658992	-1240.152272	-1237.790361	-90.63	-1237.318158	
**410**						-820.858184
410_1	-823.176622	-822.826137	-821.202780	-4.06	-820.852295	-820.858765
410_2	-823.176434	-822.825873	-821.202409	-3.98	-820.851848	-820.858191
410_3	-823.177128	-822.826607	-821.202518	-3.73	-820.851997	-820.857941
410_4	-823.176637	-822.826010	-821.201939	-3.80	-820.851312	-820.857368
410_5	-823.175198	-822.824733	-821.201087	-4.15	-820.850621	-820.857235
410_6	-823.174700	-822.824147	-821.199884	-4.11	-820.849331	-820.855880
**410-Ac$^+$**						-973.515327
410-Ac$^+$_1	-976.183761	-975.778069	-973.877242	-27.91	-973.471551	-973.516028
410-Ac$^+$_2	-976.182553	-975.776936	-973.875645	-28.64	-973.470028	-973.515669
410-Ac$^+$_3	-976.183497	-975.777791	-973.876811	-27.86	-973.471105	-973.515503
410-Ac$^+$_4	-976.182300	-975.776758	-973.875174	-28.58	-973.469632	-973.515178
410-Ac$^+$_5	-976.183266	-975.777572	-973.874516	-28.61	-973.468821	-973.514414
410-Ac$^+$_6	-976.181375	-975.776644	-973.873542	-28.46	-973.468811	-973.514165
410-Ac$^+$_7	-976.182902	-975.777316	-973.874028	-28.56	-973.468442	-973.513955

410-Ac$^+$_8	-976.181392	-975.775636	-973.873555	-28.62	-973.467799	-973.513408
410-Ac$^+$_9	-976.182231	-975.776621	-973.874677	-27.78	-973.469067	-973.513337
410-Ac$^+$_10	-976.182048	-975.776520	-973.874454	-27.80	-973.468926	-973.513228
**411**						-825.516838
411_1	-828.001801	-827.557348	-825.963011	1.59	-825.517952	
411_2	-828.002033	-827.557927	-825.961857	-0.29	-825.517862	
411_3	-828.000917	-827.556782	-825.962331	1.59	-825.517591	
411_4	-828.000859	-827.556620	-825.961300	-0.50	-825.517252	
411_5	-827.998449	-827.554199	-825.961499	0.54	-825.517042	
411_6	-827.998623	-827.554291	-825.961033	-0.29	-825.516813	
411_7	-827.998803	-827.554666	-825.960348	-1.13	-825.516641	
411_8	-827.999580	-827.555168	-825.960747	-0.67	-825.516590	
411_9	-827.998462	-827.554037	-825.960445	-1.38	-825.516546	
411_10	-827.999940	-827.555453	-825.961145	0.38	-825.516515	
**411-Ac$^+$**						-978.172026
411-Ac$^+$_1	-981.009738	-980.510214	-978.635898	-97.11	-978.173362	
411-Ac$^+$_2	-981.008871	-980.509343	-978.636382	-95.14	-978.173092	
411-Ac$^+$_3	-981.007715	-980.508061	-978.635139	-96.73	-978.172330	
411-Ac$^+$_4	-981.008161	-980.508659	-978.634270	-98.37	-978.172234	
411-Ac$^+$_5	-981.006058	-980.506745	-978.633412	-97.57	-978.171261	
411-Ac$^+$_6	-981.005256	-980.505950	-978.632709	-99.29	-978.171219	
411-Ac$^+$_7	-981.007559	-980.508157	-978.633548	-97.07	-978.171118	
411-Ac$^+$_8	-981.005275	-980.505633	-978.633051	-98.62	-978.170970	
411-Ac$^+$_9	-981.008377	-980.508860	-978.633957	-94.98	-978.170614	
411-Ac$^+$_10	-981.005387	-980.505514	-978.634049	-95.56	-978.170573	
**412**						-1083.201763
412_1	-1086.167286	-1085.757767	-1083.608857	-4.69	-1083.201122	
412_2	-1086.169118	-1085.759289	-1083.611085	-3.26	-1083.202499	
412_3	-1086.165676	-1085.756122	-1083.606658	-4.27	-1083.198730	
412_4	-1086.167533	-1085.757840	-1083.608986	-2.97	-1083.200424	
412_5	-1086.165697	-1085.756307	-1083.608195	-2.93	-1083.199921	
412_6	-1086.163902	-1085.754685	-1083.605952	-4.85	-1083.198583	
412_7	-1086.165521	-1085.756162	-1083.607797	-3.81	-1083.199888	
412_8	-1086.161798	-1085.752904	-1083.605293	-4.14	-1083.197977	
412_9	-1086.163169	-1085.754080	-1083.606815	-3.05	-1083.198890	
**412-Ac$^+$**						-1235.844670
412-Ac$^+$_1	-1239.160699	-1238.696631	-1236.267209	-101.04	-1235.841627	
412-Ac$^+$_2	-1239.161682	-1238.697692	-1236.268697	-99.91	-1235.842762	
412-Ac$^+$_3	-1239.159800	-1238.695566	-1236.266017	-100.75	-1235.840157	
412-Ac$^+$_4	-1239.160696	-1238.696587	-1236.267525	-99.58	-1235.841343	
412-Ac$^+$_5	-1239.161830	-1238.697925	-1236.268971	-103.01	-1235.844301	
412-Ac$^+$_6	-1239.162820	-1238.698909	-1236.270428	-101.92	-1235.845337	
412-Ac$^+$_7	-1239.160967	-1238.696903	-1236.267827	-102.63	-1235.842855	
412-Ac$^+$_8	-1239.161880	-1238.697946	-1236.269305	-101.46	-1235.844015	
**413**						-1596.676531
413_1	-1600.943505	-1600.393752	-1597.233106	16.78	-1596.676962	
413_2	-1600.943019	-1600.392983	-1597.231793	16.90	-1596.675319	
413_3	-1600.941839	-1600.392107	-1597.227821	10.92	-1596.673929	
413_4	-1600.941870	-1600.391860	-1597.227872	11.51	-1596.673480	
413_5	-1600.945102	-1600.395076	-1597.225443	10.50	-1596.671417	
413_6	-1600.945447	-1600.394979	-1597.225799	11.34	-1596.671012	
413_7	-1600.945330	-1600.394774	-1597.225661	11.67	-1596.670658	
413_8	-1600.945404	-1600.395177	-1597.225864	13.43	-1596.670522	
**413-Ac$^+$**						-1749.321484
413-Ac$^+$_1	-1753.941715	-1753.337110	-1749.895734	-81.04	-1749.321998	

413-Ac$^+$_2	-1753.943862	-1753.339171	-1749.901724	-63.76	-1749.321319	
413-Ac$^+$_3	-1753.941461	-1753.336828	-1749.895471	-79.96	-1749.321292	
413-Ac$^+$_4	-1753.940520	-1753.335886	-1749.894078	-80.21	-1749.319993	
413-Ac$^+$_5	-1753.940298	-1753.335812	-1749.893874	-78.83	-1749.319412	
413-Ac$^+$_6	-1753.938627	-1753.334255	-1749.889320	-83.89	-1749.316900	
413-Ac$^+$_7	-1753.938925	-1753.334327	-1749.890192	-80.08	-1749.316096	
**414**						-1504.646119
414_1	-1508.737017	-1508.187430	-1505.202219	15.90	-1504.646576	
414_2	-1508.737030	-1508.187354	-1505.201915	15.86	-1504.646200	
414_3	-1508.736456	-1508.186795	-1505.199878	13.97	-1504.644895	
414_4	-1508.736311	-1508.186624	-1505.199552	13.60	-1504.644686	
414_5	-1508.739247	-1508.189284	-1505.197318	13.64	-1504.642160	
414_6	-1508.738758	-1508.188864	-1505.196532	12.55	-1504.641857	
414_7	-1508.739087	-1508.189058	-1505.197057	13.60	-1504.641849	
414_8	-1508.738807	-1508.188770	-1505.196897	14.77	-1504.641235	
**414-Ac$^+$**						-1657.292099
414-Ac$^+$_1	-1661.739770	-1661.135585	-1657.868256	-75.77	-1657.292931	
414-Ac$^+$_2	-1661.739713	-1661.135305	-1657.868442	-73.72	-1657.292114	
414-Ac$^+$_3	-1661.738235	-1661.134169	-1657.865534	-77.28	-1657.290901	
414-Ac$^+$_4	-1661.738459	-1661.134140	-1657.866495	-74.27	-1657.290463	
414-Ac$^+$_5	-1661.738415	-1661.134093	-1657.866680	-73.76	-1657.290453	
414-Ac$^+$_6	-1661.738125	-1661.134033	-1657.865666	-75.69	-1657.290402	
414-Ac$^+$_7	-1661.741785	-1661.137188	-1657.865667	-74.10	-1657.289293	
414-Ac$^+$_8	-1661.741977	-1661.137259	-1657.865439	-74.43	-1657.289071	
414-Ac$^+$_9	-1661.741049	-1661.136758	-1657.864756	-75.10	-1657.289069	
414-Ac$^+$_10	-1661.740984	-1661.136532	-1657.864698	-75.14	-1657.288867	
**415**						-1596.692162
415_1	-1600.966204	-1600.415847	-1597.246991	10.88	-1596.692490	
415_2	-1600.966115	-1600.415570	-1597.246901	10.84	-1596.692228	
415_3	-1600.970127	-1600.419255	-1597.245697	11.25	-1596.690538	
415_4	-1600.969969	-1600.419133	-1597.245337	11.30	-1596.690198	
**415-Ac$^+$**						-1749.338218
415-Ac$^+$_1	-1753.965349	-1753.360296	-1749.911629	-84.39	-1749.338719	
415-Ac$^+$_2	-1753.965287	-1753.360099	-1749.911621	-84.35	-1749.338560	
415-Ac$^+$_3	-1753.967892	-1753.362743	-1749.910628	-82.89	-1749.337049	
415-Ac$^+$_4	-1753.967977	-1753.362836	-1749.910648	-82.76	-1749.337028	
415-Ac$^+$_5	-1753.964239	-1753.359155	-1749.909777	-82.97	-1749.336293	
415-Ac$^+$_6	-1753.964166	-1753.359090	-1749.909957	-82.22	-1749.336196	
415-Ac$^+$_7	-1753.966777	-1753.361368	-1749.909059	-81.96	-1749.334869	
415-Ac$^+$_8	-1753.966850	-1753.361456	-1749.908857	-82.05	-1749.334714	
**416**						-1618.854935
416_1	-1623.218776	-1622.633783	-1619.446630	16.15	-1618.855486	
416_2	-1623.218781	-1622.633783	-1619.446121	15.40	-1618.855259	
416_3	-1623.218661	-1622.633560	-1619.445840	14.81	-1618.855098	
416_4	-1623.218347	-1622.633346	-1619.445917	16.32	-1618.854701	
416_5	-1623.217775	-1622.632594	-1619.443493	11.97	-1618.853755	
416_6	-1623.217768	-1622.632708	-1619.443964	13.72	-1618.853678	
416_7	-1623.217508	-1622.632487	-1619.442853	11.42	-1618.853482	
416_8	-1623.217651	-1622.632544	-1619.443244	15.02	-1618.852416	
416_9	-1623.220211	-1622.634857	-1619.441236	12.64	-1618.851069	
416_10	-1623.220326	-1622.634761	-1619.440915	11.46	-1618.850984	
**416-Ac$^+$**						-1771.501289
416-Ac$^+$_1	-1776.224056	-1775.584240	-1772.113812	-73.43	-1771.501964	
416-Ac$^+$_2	-1776.223817	-1775.583998	-1772.113735	-73.43	-1771.501884	
416-Ac$^+$_3	-1776.222414	-1775.582659	-1772.112641	-75.52	-1771.501650	

416-Ac+_4	-1776.222209	-1775.582524	-1772.112469	-74.56	-1771.501182	
416-Ac+_5	-1776.221865	-1775.582070	-1772.110647	-76.73	-1771.500078	
416-Ac+_6	-1776.222690	-1775.582887	-1772.111994	-72.97	-1771.499984	
416-Ac+_7	-1776.222483	-1775.582579	-1772.111984	-72.80	-1771.499808	
416-Ac+_8	-1776.220997	-1775.581292	-1772.110779	-74.98	-1771.499632	
416-Ac+_9	-1776.221988	-1775.582092	-1772.111038	-74.68	-1771.499588	
416-Ac+_10	-1776.220009	-1775.580385	-1772.108843	-78.91	-1771.499274	
**417**						-1504.662688
417_1	-1508.760339	-1508.210318	-1505.218216	13.64	-1504.663000	
417_	-1508.760247	-1508.210055	-1505.218110	13.68	-1504.662707	
417_	-1508.763667	-1508.213344	-1505.216447	13.72	-1504.660897	
417_	-1508.763555	-1508.212975	-1505.216075	13.72	-1504.660268	
**417-Ac+**						-1657.309450
417-Ac+_1	-1661.764638	-1661.159654	-1657.886974	-73.35	-1657.309925	
417-Ac+_2	-1661.764678	-1661.159820	-1657.886926	-73.14	-1657.309924	
417-Ac+_3	-1661.767432	-1661.162421	-1657.886049	-72.13	-1657.308512	
417-Ac+_4	-1661.767357	-1661.162365	-1657.886070	-71.76	-1657.308409	
417-Ac+_5	-1661.763456	-1661.158710	-1657.885301	-72.51	-1657.308172	
417-Ac+_6	-1661.763511	-1661.158765	-1657.885234	-72.38	-1657.308057	
417-Ac+_7	-1661.766250	-1661.161066	-1657.884425	-71.38	-1657.306428	
417-Ac+_8	-1661.766183	-1661.160950	-1657.884423	-71.04	-1657.306249	
**418**						-1136.056814
418_1	-1139.064106	-1138.684114	-1136.431421	-14.43	-1136.056927	
418_2	-1139.063984	-1138.683898	-1136.431237	-14.48	-1136.056666	
**418-Ac+**						-1288.703967
418-Ac+_1	-1292.058452	-1291.623979	-1289.092961	-120.04	-1288.704208	
418-Ac+_2	-1292.058464	-1291.624024	-1289.092943	-120.00	-1288.704207	
418-Ac+_3	-1292.057152	-1291.622859	-1289.091178	-119.96	-1288.702574	
418-Ac+_4	-1292.057158	-1291.622754	-1289.091143	-119.96	-1288.702428	
**419**						-1618.872669
419_1	-1623.242667	-1622.657151	-1619.463047	11.38	-1618.873196	
419_2	-1623.242764	-1622.656992	-1619.463134	11.46	-1618.872996	
419_3	-1623.242429	-1622.656825	-1619.462612	12.09	-1618.872403	
419_4	-1623.242362	-1622.656589	-1619.462488	11.38	-1618.872381	
419_5	-1623.245855	-1622.660184	-1619.461244	11.25	-1618.871285	
419_6	-1623.245720	-1622.659765	-1619.461113	11.25	-1618.870872	
419_7	-1623.245513	-1622.659546	-1619.460639	11.34	-1618.870353	
419_8	-1623.245407	-1622.659358	-1619.460595	11.34	-1618.870227	
**419-Ac+**						-1771.520233
419-Ac+_1	-1776.249635	-1775.609309	-1772.133535	-72.93	-1771.520985	
419-Ac+_2	-1776.249602	-1775.609200	-1772.133494	-72.97	-1771.520884	
419-Ac+_3	-1776.249220	-1775.608734	-1772.132863	-73.09	-1771.520217	
419-Ac+_4	-1776.249129	-1775.608637	-1772.132729	-73.14	-1771.520093	
419-Ac+_5	-1776.252470	-1775.612079	-1772.132731	-71.67	-1771.519638	
419-Ac+_6	-1776.252399	-1775.611956	-1772.132723	-71.71	-1771.519595	
419-Ac+_7	-1776.252002	-1775.611588	-1772.132032	-72.01	-1771.519043	
419-Ac+_8	-1776.248417	-1775.608026	-1772.131779	-72.17	-1771.518878	
419-Ac+_9	-1776.251937	-1775.611588	-1772.132013	-71.42	-1771.518867	
419-Ac+_10	-1776.248465	-1775.608150	-1772.131629	-71.80	-1771.518659	
**420**						-1044.008449
420_1	-1046.833133	-1046.454151	-1044.383069	-12.18	-1044.008724	
420_2	-1046.833002	-1046.453791	-1044.382675	-13.26	-1044.008515	
420_3	-1046.832807	-1046.453629	-1044.382354	-13.85	-1044.008451	
420_4	-1046.832796	-1046.453467	-1044.382303	-13.56	-1044.008137	
420_5	-1046.831620	-1046.45269	-1044.382978	-11.25	-1044.008335	

420_6	-1046.831344	-1046.452302	-1044.382393	-13.01	-1044.008307	
420_7	-1046.831558	-1046.452591	-1044.382727	-12.26	-1044.008430	
420_8	-1046.831275	-1046.452370	-1044.382436	-12.97	-1044.008471	
**420-Ac$^+$**						-1196.656734
420-Ac$^+$_1	-1199.832729	-1199.399018	-1197.047782	-113.30	-1196.657226	
420-Ac$^+$_2	-1199.832665	-1199.398953	-1197.047786	-113.22	-1196.657197	
420-Ac$^+$_3	-1199.831533	-1199.397899	-1197.046766	-113.97	-1196.656542	
420-Ac$^+$_4	-1199.831564	-1199.397960	-1197.047009	-112.80	-1196.656368	
420-Ac$^+$_5	-1199.831165	-1199.397592	-1197.045821	-113.51	-1196.655482	
420-Ac$^+$_6	-1199.831221	-1199.397592	-1197.045806	-113.47	-1196.655396	
420-Ac$^+$_7	-1199.830601	-1199.397022	-1197.045616	-112.68	-1196.654953	
420-Ac$^+$_8	-1199.830668	-1199.396959	-1197.045708	-112.51	-1196.654851	
**421**						-1044.026911
421_1	-1046.858831	-1046.479035	-1044.401895	-12.72	-1044.026943	
421_2	-1046.858788	-1046.479001	-1044.401914	-12.47	-1044.026876	
**421-Ac$^+$**						-1196.675234
421-Ac$^+$_1	-1199.856353	-1199.421957	-1197.066593	-113.93	-1196.675591	
421-Ac$^+$_2	-1199.856291	-1199.421917	-1197.066551	-113.43	-1196.675379	
421-Ac$^+$_3	-1199.856964	-1199.422639	-1197.067069	-109.20	-1196.674337	
421-Ac$^+$_4	-1199.856840	-1199.422570	-1197.066913	-108.95	-1196.674140	
**422**						-1044.022505
422_1	-1046.857690	-1046.478012	-1044.402223	-11.63	-1044.026975	
422_2	-1046.857580	-1046.477989	-1044.402053	-11.67	-1044.026908	
**422-Ac$^+$**						-1196.675477
422-Ac$^+$_1	-1199.858325	-1199.424128	-1197.068686	-108.28	-1196.675732	
422-Ac$^+$_2	-1199.858318	-1199.424100	-1197.068697	-108.24	-1196.675706	
422-Ac$^+$_3	-1199.856957	-1199.422878	-1197.066994	-108.20	-1196.674126	
422-Ac$^+$_4	-1199.856955	-1199.422802	-1197.066869	-108.28	-1196.673958	
**423**						-1158.217408
423_1	-1161.312756	-1160.898420	-1158.626372	-14.60	-1158.217598	
423_2	-1161.313891	-1160.899264	-1158.626205	-15.69	-1158.217554	
423_3	-1161.312626	-1160.898111	-1158.626250	-15.27	-1158.217551	
423_4	-1161.313736	-1160.899143	-1158.626025	-16.07	-1158.217551	
423_5	-1161.313722	-1160.899125	-1158.626041	-15.90	-1158.217500	
423_6	-1161.314173	-1160.899492	-1158.626777	-14.18	-1158.217498	
423_7	-1161.312806	-1160.898579	-1158.626613	-13.31	-1158.217454	
423_8	-1161.312893	-1160.898548	-1158.626689	-13.35	-1158.217427	
423_9	-1161.312577	-1160.898111	-1158.626108	-15.19	-1158.217427	
423_10	-1161.313883	-1160.899105	-1158.626425	-14.98	-1158.217352	
**423-Ac$^+$**						-1310.865945
423-Ac$^+$_1	-1314.316788	-1313.847750	-1311.292828	-112.59	-1310.866675	
423-Ac$^+$_2	-1314.316776	-1313.847540	-1311.292817	-112.68	-1310.866497	
423-Ac$^+$_3	-1314.315546	-1313.846437	-1311.291902	-114.06	-1310.866235	
423-Ac$^+$_4	-1314.315537	-1313.846448	-1311.291749	-114.18	-1310.866150	
423-Ac$^+$_5	-1314.315644	-1313.846431	-1311.292130	-112.34	-1310.865705	
423-Ac$^+$_6	-1314.315615	-1313.846476	-1311.291860	-112.21	-1310.865462	
423-Ac$^+$_7	-1314.313707	-1313.844687	-1311.290228	-115.10	-1310.865048	
423-Ac$^+$_8	-1314.313672	-1313.844656	-1311.290061	-115.27	-1310.864948	
423-Ac$^+$_9	-1314.315226	-1313.846079	-1311.290829	-112.84	-1310.864662	
423-Ac$^+$_10	-1314.315255	-1313.846004	-1311.290868	-112.84	-1310.864597	
**424**						-1044.008394
424_1	-1046.834230	-1046.455465	-1044.382535	-13.05	-1044.008742	
424_2	-1046.834105	-1046.454748	-1044.382233	-14.06	-1044.008230	
424_3	-1046.833798	-1046.454738	-1044.381630	-14.73	-1044.008179	
424_4	-1046.833785	-1046.454669	-1044.381789	-14.35	-1044.008139	

**424-Ac$^+$**						-1196.657594
424-Ac$^+$_1	-1199.830973	-1199.397156	-1197.047564	-116.69	-1196.658192	
424-Ac$^+$_2	-1199.830933	-1199.397049	-1197.047590	-116.27	-1196.657992	
424-Ac$^+$_3	-1199.830633	-1199.396905	-1197.046074	-117.28	-1196.657015	
424-Ac$^+$_4	-1199.830632	-1199.396894	-1197.047204	-113.55	-1196.656717	
424-Ac$^+$_5	-1199.830683	-1199.396877	-1197.045795	-117.40	-1196.656706	
424-Ac$^+$_6	-1199.830308	-1199.396600	-1197.046945	-111.55	-1196.655723	
424-Ac$^+$_7	-1199.831315	-1199.397614	-1197.046529	-112.42	-1196.655649	
424-Ac$^+$_8	-1199.831405	-1199.397645	-1197.046266	-112.80	-1196.655470	
**425**						-1158.237020
425_1	-1161.339892	-1160.924702	-1158.647079	-14.06	-1158.237244	
425_2	-1161.339746	-1160.924617	-1158.646918	-14.18	-1158.237192	
425_3	-1161.339527	-1160.924391	-1158.646518	-14.06	-1158.236736	
425_4	-1161.339460	-1160.924225	-1158.646432	-14.10	-1158.236568	
**425-Ac$^+$**						-1310.886271
425-Ac$^+$_1	-1314.343692	-1313.873929	-1311.315562	-107.40	-1310.886707	
425-Ac$^+$_2	-1314.343654	-1313.873816	-1311.315529	-107.36	-1310.886583	
425-Ac$^+$_3	-1314.343207	-1313.873467	-1311.315060	-107.74	-1310.886356	
425-Ac$^+$_4	-1314.343189	-1313.873439	-1311.315014	-107.61	-1310.886252	
425-Ac$^+$_5	-1314.342298	-1313.872577	-1311.313747	-107.49	-1310.884965	
425-Ac$^+$_6	-1314.342319	-1313.872598	-1311.313702	-107.40	-1310.884888	
425-Ac$^+$_7	-1314.341844	-1313.872277	-1311.313201	-107.74	-1310.884669	
425-Ac$^+$_8	-1314.341816	-1313.872144	-1311.313141	-107.57	-1310.884440	
**426**						-1136.037495
426_1	-1139.039005	-1138.659639	-1136.410931	-16.15	-1136.037716	
426_2	-1139.039185	-1138.659680	-1136.411005	-16.15	-1136.037651	
426_3	-1139.039209	-1138.659623	-1136.411170	-15.73	-1136.037576	
426_4	-1139.039202	-1138.659538	-1136.411299	-14.77	-1136.037261	
426_5	-1139.037014	-1138.657520	-1136.410914	-14.39	-1136.036901	
426_6	-1139.036955	-1138.657669	-1136.410733	-15.82	-1136.037471	
426_7	-1139.037093	-1138.657654	-1136.410975	-15.40	-1136.037400	
426_8	-1139.037144	-1138.657915	-1136.410933	-15.36	-1136.037552	
**426-Ac$^+$**						-1288.686840
426-Ac$^+$_1	-1292.036108	-1291.602523	-1289.082969	-100.08	-1288.687503	
426-Ac$^+$_2	-1292.033216	-1291.599308	-1289.072491	-123.22	-1288.685515	
426-Ac$^+$_3	-1292.033155	-1291.599259	-1289.072377	-123.34	-1288.685460	
426-Ac$^+$_4	-1292.033005	-1291.599221	-1289.073934	-116.19	-1288.684404	
426-Ac$^+$_5	-1292.032999	-1291.599160	-1289.073762	-116.57	-1288.684320	
426-Ac$^+$_6	-1292.031797	-1291.597913	-1289.070576	-123.22	-1288.683623	
426-Ac$^+$_7	-1292.031694	-1291.597709	-1289.070512	-123.39	-1288.683522	
426-Ac$^+$_8	-1292.031456	-1291.597746	-1289.070768	-120.62	-1288.683002	
426-Ac$^+$_9	-1292.031474	-1291.597804	-1289.070752	-120.50	-1288.682978	
**427**						-871.900890
427_1	-874.162557	-873.856387	-872.199497	-4.90	-871.893327	-871.901136
427_2	-874.162566	-873.856218	-872.199425	-4.97	-871.893077	-871.900997
427_3	-874.158168	-873.852116	-872.194565	-6.34	-871.888513	-871.898616
427_4	-874.157849	-873.851751	-872.194119	-6.39	-871.888021	-871.898204
427_5	-874.155463	-873.849018	-872.194489	-5.12	-871.888044	-871.896203
427_6	-874.152544	-873.846564	-872.188499	-8.23	-871.882520	-871.895635
427_7	-874.152349	-873.845984	-872.191219	-6.53	-871.884854	-871.895260
427_8	-874.152381	-873.846253	-872.188009	-8.17	-871.881881	-871.894900
**427-Ac$^+$**						-1024.542020
427-Ac$^+$_1	-1027.152078	-1026.791413	-1024.853242	-31.17	-1024.492577	-1024.542250
427-Ac$^+$_2	-1027.152078	-1026.791411	-1024.853244	-31.16	-1024.492576	-1024.542233
427-Ac$^+$_3	-1027.151914	-1026.791243	-1024.853053	-31.28	-1024.492382	-1024.542230

427-Ac$^+$_4	-1027.151914	-1026.791243	-1024.853053	-31.28	-1024.492382	-1024.542230
427-Ac$^+$_5	-1027.149460	-1026.788833	-1024.851236	-31.58	-1024.490609	-1024.540935
427-Ac$^+$_6	-1027.149354	-1026.788701	-1024.851159	-31.53	-1024.490506	-1024.540753
427-Ac$^+$_7	-1027.145691	-1026.785213	-1024.847850	-32.53	-1024.487372	-1024.539212
427-Ac$^+$_8	-1027.145689	-1026.785124	-1024.847908	-32.45	-1024.487343	-1024.539056
427-Ac$^+$_9	-1027.142172	-1026.781872	-1024.845140	-33.11	-1024.484840	-1024.537604
427-Ac$^+$_10	-1027.142172	-1026.781872	-1024.845140	-33.10	-1024.484840	-1024.537588
**428**	-609.437278	-609.163890	-607.990831	-9.50	-607.721061	-607.721061
**428-Ac$^+$**						-760.369530
428-Ac$^+$_1	-762.440075	-762.111592	-760.655492	-112.05	-760.369687	
428-Ac$^+$_2	-762.439141	-762.110580	-760.654585	-113.60	-760.369292	
**429**	-608.248922	-607.997703	-606.815152	-16.95	-606.5703889	-606.570389
**429-Ac$^+$**						-759.219846
429-Ac$^+$_1	-761.253972	-760.947690	-759.480966	-118.83	-759.2199440	
429-Ac$^+$_2	-761.253112	-760.946828	-759.480190	-120.29	-759.2197221	
**430**						-646.889338
430_1	-648.739813	-648.437021	-647.190473	-5.02	-646.889593	
430_2	-648.737139	-648.434075	-647.189498	-5.77	-646.888632	
**430-Ac$^+$**						-799.539929
430-Ac$^+$_1	-801.745225	-801.387184	-799.857844	-106.15	-799.5402334	
430-Ac$^+$_2	-801.744402	-801.386219	-799.857176	-106.15	-799.5394234	
430-Ac$^+$_3	-801.740146	-801.382030	-799.854065	-106.02	-799.5363299	
430-Ac$^+$_4	-801.739262	-801.381071	-799.853080	-106.94	-799.5356203	
**431**						-842.696676
431_1	-845.225804	-844.773120	-843.151656	4.35	-842.6973152	
431_2	-845.226266	-844.773664	-843.151150	3.89	-842.6970664	
431_3	-845.224920	-844.772280	-843.151380	5.52	-842.6966375	
431_4	-845.225141	-844.772591	-843.150273	3.43	-842.6964166	
431_5	-845.224944	-844.772307	-843.150164	3.51	-842.6961901	
431_6	-845.224639	-844.771780	-843.151589	6.82	-842.6961324	
431_7	-845.225043	-844.772409	-843.150398	4.77	-842.6959472	
431_8	-845.225399	-844.772609	-843.150866	5.69	-842.6959088	
431_9	-845.225251	-844.772595	-843.150973	6.57	-842.6958146	
**431-Ac$^+$**						-995.347569
431-Ac^{+1}_	-998.231706	-997.724119	-995.818900	-97.11	-995.3483003	
431-Ac$^+$_2	-998.232565	-997.724897	-995.819661	-94.89	-995.3481347	
431-Ac$^+$_3	-998.230948	-997.723126	-995.819330	-95.73	-995.3479697	
431-Ac$^+$_4	-998.231953	-997.724053	-995.820285	-92.38	-995.3475707	
431-Ac$^+$_5	-998.230527	-997.722839	-995.818092	-97.03	-995.3473608	
431-Ac$^+$_6	-998.230519	-997.722723	-995.818036	-96.27	-995.3469073	
431-Ac$^+$_7	-998.230476	-997.722694	-995.818050	-96.02	-995.3468401	
431-Ac$^+$_8	-998.231443	-997.723537	-995.818836	-94.14	-995.3467861	
431-Ac$^+$_9	-998.230740	-997.722574	-995.818674	-95.19	-995.346764	
431-Ac$^+$_10	-998.231579	-997.723492	-995.819356	-92.97	-995.3466794	
**432**						-645.739143
432_1	-647.550815	-647.269999	-646.015591	-12.09	-645.7393798	
432_2	-647.551603	-647.270936	-646.014767	-12.80	-645.7389753	
432_3	-647.549715	-647.268968	-646.012418	-12.76	-645.7365310	
**432-Ac$^+$**						-798.390625
432-Ac$^+$_1	-800.558357	-800.222515	-798.682759	-115.02	-798.3907258	
432-Ac$^+$_2	-800.559119	-800.223296	-798.683274	-113.39	-798.390639	
432-Ac$^+$_3	-800.554459	-800.218868	-798.679798	-112.34	-798.3869951	
432-Ac$^+$_4	-800.553670	-800.217939	-798.679003	-113.22	-798.3863953	
**433**						-841.545967
433_1	-844.036400	-843.605886	-841.976620	-1.92	-841.546837	

433_2	-844.036216	-843.605834	-841.977251	2.01	-841.546103	
433_3	-844.035767	-843.605382	-841.975710	-1.92	-841.546056	
433_4	-844.036125	-843.605687	-841.976356	-0.08	-841.545948	
433_5	-844.036255	-843.605695	-841.975393	-2.89	-841.545934	
433_6	-844.037044	-843.606530	-841.975303	-1.97	-841.545539	
433_7	-844.037044	-843.606530	-841.975303	-1.97	-841.545539	
433_8	-844.037144	-843.606699	-841.975565	-0.17	-841.545185	
433_9	-844.035455	-843.605001	-841.974594	-2.59	-841.545126	
433_10	-844.035456	-843.604966	-841.974249	-2.80	-841.544825	
**433-Ac$^+$**						-994.197488
433-Ac$^+$_1	-997.046466	-996.560962	-994.645085	-102.26	-994.198530	
433-Ac$^+$_2	-997.044593	-996.559081	-994.643719	-103.76	-994.197727	
433-Ac$^+$_3	-997.044744	-996.559383	-994.642986	-105.02	-994.197625	
433-Ac$^+$_4	-997.044342	-996.558805	-994.643316	-104.01	-994.197394	
433-Ac$^+$_5	-997.044580	-996.559039	-994.643666	-103.01	-994.197359	
433-Ac$^+$_6	-997.045386	-996.559941	-994.644275	-101.09	-994.197333	
433-Ac$^+$_7	-997.043785	-996.558338	-994.642910	-103.64	-994.196937	
433-Ac$^+$_8	-997.044277	-996.559610	-994.642851	-101.17	-994.196718	
433-Ac$^+$_9	-997.044780	-996.559270	-994.643901	-100.16	-994.196540	
433-Ac$^+$_10	-997.043032	-996.557678	-994.642475	-102.47	-994.196150	
**434**						-953.218523
434_1	-955.903158	-955.499018	-953.621331	-5.06	-953.219118	
434_2	-955.901028	-955.496897	-953.621346	-3.97	-953.218727	
434_3	-955.903126	-955.499183	-953.619806	-5.86	-953.218095	
434_4	-955.900586	-955.497080	-953.617966	-7.36	-953.217264	
434_5	-955.901229	-955.497343	-953.619450	-3.97	-953.217076	
434_6	-955.902205	-955.498497	-953.617240	-7.74	-953.216481	
434_7	-955.897733	-955.494139	-953.616828	-4.27	-953.214860	
434_8	-955.897976	-955.494064	-953.617214	-4.06	-953.214849	
434_9	-955.897697	-955.494087	-953.616685	-4.35	-953.214732	
**434-Ac$^+$**						-1105.870495
434-Ac$^+$_1	-1108.912657	-1108.454069	-1106.292039	-98.11	-1105.870819	
434-Ac$^+$_2	-1108.911987	-1108.453133	-1106.291541	-99.62	-1105.870630	
434-Ac$^+$_3	-1108.913135	-1108.454221	-1106.291777	-97.40	-1105.869961	
434-Ac$^+$_4	-1108.912567	-1108.453657	-1106.291288	-98.62	-1105.869940	
**435**						-724.056335
435_1	-726.136622	-725.794008	-724.392585	-4.50	-724.049971	-724.057142
435_2	-726.135261	-725.792675	-724.391022	-4.42	-724.048436	-724.055480
435_3	-726.134093	-725.791344	-724.390368	-4.72	-724.047619	-724.055140
435_4	-726.133773	-725.791423	-724.389136	-4.75	-724.046786	-724.054356
435_5	-726.133843	-725.791105	-724.389524	-4.33	-724.046786	-724.053687
435_6	-726.132311	-725.790069	-724.388635	-4.83	-724.046393	-724.054090
435_7	-726.132623	-725.790061	-724.388834	-4.62	-724.046272	-724.053634
435_8	-726.132578	-725.789756	-724.388529	-4.64	-724.045707	-724.053101
435_9	-726.131575	-725.788592	-724.388232	-4.92	-724.045249	-724.053090
435_10	-726.130711	-725.788734	-724.387694	-4.94	-724.045717	-724.053589
**435-Ac$^+$**						-876.712556
435-Ac$^+$_1	-879.143513	-878.745843	-877.065732	-28.52	-876.668062	-876.713511
435-Ac$^+$_2	-879.143513	-878.745843	-877.065732	-28.52	-876.668062	-876.713511
435-Ac$^+$_3	-879.142627	-878.744948	-877.064824	-28.63	-876.667145	-876.712770
435-Ac$^+$_4	-879.142627	-878.744947	-877.064824	-28.63	-876.667144	-876.712769
435-Ac$^+$_5	-879.142268	-878.744719	-877.064513	-28.48	-876.666963	-876.712349
435-Ac$^+$_6	-879.142094	-878.744165	-877.064457	-28.75	-876.666528	-876.712344
435-Ac$^+$_7	-879.141653	-878.743747	-877.064156	-28.87	-876.666250	-876.712258
435-Ac$^+$_8	-879.142238	-878.744608	-877.064431	-28.50	-876.666801	-876.712219

435-Ac+_9	-879.141397	-878.743848	-877.063798	-28.66	-876.666249	-876.711922
435-Ac+_10	-879.141959	-878.743942	-877.064138	-28.65	-876.666121	-876.711778
**436**						-645.730280
436_1	-647.539246	-647.256369	-646.004511	-5.59	-645.721634	-645.730542
436_2	-647.537517	-647.254755	-646.002984	-5.76	-645.720222	-645.729401
**436-Ac+**						-798.387055
436-Ac+_1	-800.544982	-800.207283	-798.676037	-30.69	-798.338338	-798.387246
436-Ac+_2	-800.544982	-800.207283	-798.676155	-30.59	-798.338457	-798.387205
436-Ac+_3	-800.545288	-800.207283	-798.676155	-30.58	-798.338150	-798.386883
436-Ac+_4	-800.545288	-800.206988	-798.676036	-30.69	-798.337736	-798.386644
**437**						-1106.352811
437_1	-1109.447043	-1108.993196	-1106.802424	-3.16	-1106.348577	-1106.353613
437_2	-1109.447132	-1108.993055	-1106.802354	-3.13	-1106.348278	-1106.353266
437_3	-1109.447628	-1108.993564	-1106.802303	-2.81	-1106.348239	-1106.352717
437_4	-1109.447585	-1108.993563	-1106.802088	-2.77	-1106.348066	-1106.352481
437_5	-1109.447026	-1108.992980	-1106.801891	-2.88	-1106.347844	-1106.352434
437_6	-1109.446406	-1108.992420	-1106.801637	-2.96	-1106.347651	-1106.352368
437_7	-1109.447786	-1108.993676	-1106.802326	-2.59	-1106.348216	-1106.352343
437_8	-1109.444983	-1108.991221	-1106.800827	-3.25	-1106.347065	-1106.352244
437_9	-1109.448248	-1108.994131	-1106.802136	-2.51	-1106.348019	-1106.352019
437_10	-1109.446099	-1108.991892	-1106.800767	-2.89	-1106.346560	-1106.351165
**437-Ac+**						-1259.013024
437-Ac+_1	-1262.458568	-1261.949602	-1259.483210	-24.72	-1258.974244	-1259.013638
437-Ac+_2	-1262.458568	-1261.949602	-1259.483208	-24.72	-1258.974242	-1259.013636
437-Ac+_3	-1262.459023	-1261.949814	-1259.483351	-24.48	-1258.974143	-1259.013154
437-Ac+_4	-1262.459022	-1261.949815	-1259.483347	-24.48	-1258.974140	-1259.013151
437-Ac+_5	-1262.459415	-1261.950243	-1259.481958	-24.77	-1258.972785	-1259.012259
437-Ac+_6	-1262.459504	-1261.950296	-1259.481941	-24.63	-1258.972733	-1259.011983
437-Ac+_7	-1262.458026	-1261.948997	-1259.481565	-24.51	-1258.972536	-1259.011595
437-Ac+_8	-1262.456839	-1261.947632	-1259.480614	-24.88	-1258.971407	-1259.011056
437-Ac+_9	-1262.457707	-1261.948591	-1259.481125	-24.47	-1258.972009	-1259.011005
437-Ac+_10	-1262.457476	-1261.948463	-1259.479556	-25.14	-1258.970543	-1259.010606

COMPUTATIONAL METHODS (MAA)

The Michael-acceptor affinity (MAA) of Lewis bases (LB) has been calculated as the reaction enthalpy at 298.15 K and 1 atm pressure including solvent effects ($CHCl_3$) for the detachment reaction shown in equation (S11). This is in analogy to the mass spectrometric definition of proton affinities.

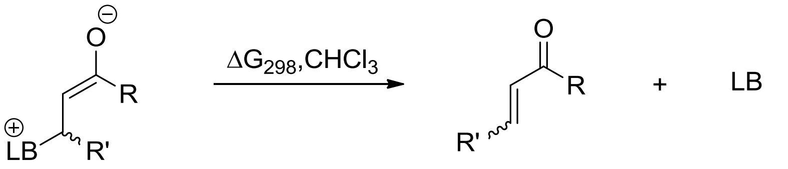

(S11)

The geometries of all species in equation (S11) have been optimized at the mPW1k/6-31+G(d) level of theory. The conformational space of flexible Lewis bases and the corresponding cations have been searched using the MM3* force field and the systematic search routine implemented in MACROMODEL 9.7.[48] All stationary points located at force field level have then been reoptimized at B98/6-31G(d) level as described before. Thermochemical corrections to 298.15 K have been calculated for all minima from unscaled vibrational frequencies obtained at this same level. The thermochemical corrections have been combined with single point energies calculated at the MP2(FC)/6-

31+G(2d,p)//mPW1k/6-31+G(d) level to yield enthalpies H_{298} at 298.15 K. In conformationally flexible systems enthalpies have been calculated as Boltzmann-averaged values over all available conformers. In order to consider solvent effects in chloroform the PCM/UAHF/RHF/6-31G(d) approach was used. All quantum mechanical calculations have been performed with Gaussian 03 [49].

For details of the calculations see reference [30].

Structures of all Systems (best conformation)
(Optimized at B98/6-31G(d) level)

CH_3^+
see reference 4

BH^+
1\1\GINC-NAUTILUS\SP\RMP2-FC\6-31+G(2d,p)\C13H11(1+)\CHRISTOPH\13-Jun-
2010\0\\#p MP2(FC)/6-31+g(2d,p) scf=tight\\refsp_1\\1,1\C\C,1,1.428550
92\C,2,1.42920585,1,118.68746509\C,3,1.38634613,2,120.6321887,1,-3.800
37237,0\C,4,1.40398116,3,119.49642002,2,2.42191193,0\C,1,1.38519191,2,
120.0828665,3,2.48966973,0\H,1,1.08482569,6,119.72597136,5,175.8255880
6,0\H,3,1.08763243,2,119.18202641,1,178.19618013,0\H,4,1.08599869,3,12
0.29450106,2,-178.45995578,0\H,5,1.08726138,4,119.57644712,3,178.72018
759,0\H,6,1.08616724,1,120.05405532,2,-178.59713794,0\C,2,5.3014062,1,
90.09915665,6,-169.9448705,0\C,12,1.40724778,2,51.1403505,1,35.8798864
6,0\C,13,1.38519122,12,120.0652973,2,4.99253905,0\C,14,1.42855076,13,1
20.08284583,12,0.14669542,0\C,15,1.42920588,14,118.68747664,13,2.48938
819,0\C,16,1.38634585,15,120.63218641,14,-3.80014983,0\H,12,1.08726159
,2,169.74071554,1,10.6038675,0\H,13,1.08616789,12,119.8687687,2,-176.2
614361,0\H,14,1.0848255,13,119.72598224,12,175.82558837,0\H,16,1.08763
253,15,119.18200706,14,178.19620924,0\H,17,1.08599892,16,120.29452479,
15,-178.46003708,0\H,15,2.11835463,14,150.03706758,13,163.77326093,0\C
,15,1.41947145,14,124.30511493,13,-179.32164014,0\\Version=AM64L-G03Re
vD.01\State=1-A\HF=-498.5040193\MP2=-500.293523\RMSD=1.281e-09\Thermal
=0.\PG=C01 [X(C13H11)]\\@

TT^+
1\1\GINC-CALYPSO\SP\RMP2-FC\6-31+G(2d,p)\C19H15(1+)\CHRISTOPH\17-Aug-2
011\0\\#p MP2(FC)/6-31+g(2d,p) scf=tight\\refttsp_9\\1,1\C\C,1,4.26386
79\C,2,1.40256448,1,60.24622706\C,3,1.38982466,2,120.00275497,1,0.7353
2798,0\C,4,1.42284032,3,120.41805581,2,-1.46647975,0\C,5,1.42268041,4,
118.65365583,3,0.71210391,0\C,6,1.38986775,5,120.41254653,4,0.78143296
,0\H,2,1.08711827,1,179.98632956,5,145.91113243,0\H,3,1.08636655,2,120
.10713222,1,-178.86737834,0\H,4,1.08596982,3,119.99611924,2,-178.01959
327,0\H,6,1.08599934,5,119.48263515,4,177.29705184,0\H,7,1.08636407,6,
119.88315619,5,178.09271382,0\C,1,4.26389838,5,119.9850297,4,-33.64900
215,0\C,13,1.4025899,1,60.24644952,5,145.8272488,0\C,14,1.3897937,13,1
20.0095702,1,0.72605507,0\C,15,1.42283453,14,120.41513196,13,-1.446446
31,0\C,16,1.42278149,15,118.64827949,14,0.70971723,0\C,17,1.38984725,1
6,120.42037646,15,0.76234516,0\H,13,1.0871139,1,179.98964286,16,123.74
050544,0\H,14,1.08636953,13,120.1033267,1,-178.84411777,0\H,15,1.08592
823,14,119.97205048,13,-177.92122168,0\H,17,1.08596857,16,119.48197291
,15,177.27090119,0\H,18,1.08636271,17,119.88825942,16,178.10589292,0\C
,1,4.26376155,16,120.06229012,15,-33.61501221,0\C,24,1.40256725,1,60.2
367564,16,145.70437702,0\C,25,1.38980441,24,120.00233665,1,0.71793758,
0\C,26,1.42280319,25,120.41898853,24,-1.47269031,0\C,27,1.42270161,26,
118.65314874,25,0.74017024,0\C,28,1.38985021,27,120.41276211,26,0.7505
5849,0\H,24,1.08711547,1,179.96654799,27,-170.24503545,0\H,25,1.086367
19,24,120.10641961,1,-178.8848862,0\H,26,1.08597744,25,120.00373522,24
,-178.01228569,0\H,28,1.08599354,27,119.48854231,26,177.24680461,0\H,2
9,1.08635721,28,119.88483644,27,178.10335246,0\\Version=AM64L-G03RevD.
01\State=1-A\HF=-728.0816324\MP2=-730.7227786\RMSD=9.270e-09\Thermal=0
.\PG=C01 [X(C19H15)]\\@
$MOSC^+$
see reference 29

1
see reference 4

1-Me$^+$
see reference 4

1-BH$^+$

1\1\GINC-CIPCLU08\SP\RMP2-FC\6-31+G(2d,p)\C18H16N1(1+)\C2175\14-Jan-20
11\0\\#p MP2(FC)/6-31+G(2d,p) scf=tight\\yin11bhsp_3\\1,1\C\H,1,1.0959
6552\C,1,4.32844458,2,107.81616865\C,3,1.39837712,1,59.18535099,2,26.9
2313902,0\C,4,1.39719633,3,120.10543358,1,-0.95282513,0\C,5,1.40313147
,4,120.28497339,3,0.41074843,0\C,6,1.40402273,5,119.40641557,4,-0.4944
7784,0\C,7,1.39799152,6,120.1743269,5,0.25059352,0\H,3,1.0867749,1,179
.0924846,6,175.78485776,0\H,4,1.08679585,3,120.21076633,1,179.41977711
,0\H,5,1.08864599,4,119.75908274,3,179.76481443,0\H,7,1.08877464,6,120
.60080693,5,-179.52354083,0\H,8,1.08694417,7,119.67357917,6,-179.89659
796,0\C,1,4.32739305,6,117.43697846,5,-92.07962252,0\C,14,1.3983125,1,
60.57348667,6,-28.22592968,0\C,15,1.39845313,14,120.27778088,1,0.18833
75,0\C,16,1.4042393,15,120.04669409,14,-0.27294732,0\C,17,1.40666764,1
6,119.34116006,15,0.58194483,0\C,18,1.39592463,17,120.46880422,16,-0.4
8211683,0\H,14,1.08691352,1,179.31813116,17,148.12445831,0\H,15,1.0868
3797,14,120.11450308,1,179.3956857,0\H,16,1.08667908,15,119.79060352,1
4,178.26277832,0\H,18,1.08929553,17,119.88915761,16,178.96800375,0\H,1
9,1.08680873,18,119.86719191,17,179.38644253,0\C,1,4.30414948,17,113.4
2664273,16,98.33166749,0\C,25,1.40051428,1,58.64845292,17,165.1965976,
0\C,26,1.38479129,25,119.13775986,1,0.10586299,0\C,27,2.35178528,26,91
.43457994,25,-0.34026391,0\C,28,1.39073111,27,90.9605484,26,0.47628093
,0\H,25,1.08644546,1,179.08157992,17,167.38091515,0\H,26,1.08518609,25
,121.59721157,1,-179.99430279,0\H,27,1.08489903,26,123.01956623,25,178
.68588982,0\H,28,1.08363677,27,145.87086522,26,-179.13035513,0\H,29,1.
08519896,28,118.99619543,27,179.77694841,0\N,28,1.34976428,27,29.69471
793,26,-179.47715442,0\\Version=AM64L-G03RevD.01\State=1-A\HF=-745.262
5265\MP2=-747.9605593\RMSD=6.553e-09\Thermal=0.\PG=C01 [X(C18H16N1)]\\
@

1-TT$^+$

1\1\GINC-EDDY\SP\RMP2-FC\6-31+G(2d,p)\C24H20N1(1+)\CHRISTOPH\04-Nov-20
11\0\\#p MP2(FC)/6-31+g(2d,p) scf=tight\\yin11ttsp_4\\1,1\C\C,1,4.3470
246\C,2,1.39912301,1,58.39793217\C,3,1.38563507,2,119.34115259,1,0.258
91764,0\C,4,2.34555895,3,91.37849952,2,-0.3088286,0\C,5,1.3908534,4,91
.0344134,3,0.34995694,0\H,2,1.08648998,1,178.95219474,4,12.59718336,0\
H,3,1.08520059,2,121.63595008,1,179.83312329,0\H,4,1.08310948,3,122.61
402112,2,178.8674897,0\H,5,1.08247527,4,146.5406697,3,179.28331886,0\H
,6,1.08519685,5,118.80322934,4,179.34594542,0\C,1,4.37246847,4,99.6989
6311,3,-114.63175501,0\C,12,1.40008275,1,58.31083843,4,-156.94925788,0
\C,13,1.39404564,12,120.32454147,1,-0.26930409,0\C,14,1.40972491,13,12
0.88737245,12,1.04986914,0\C,15,1.40186327,14,118.26457624,13,-1.93823
222,0\C,12,1.39342165,1,61.0048157,15,9.51338012,0\H,12,1.08669369,1,1
78.61347237,15,-175.85244694,0\H,13,1.08696573,12,120.23629739,1,-179.
61750871,0\H,14,1.08703412,13,119.41076663,12,-178.39199475,0\H,16,1.0
8560467,15,121.56204252,14,-177.04856255,0\H,17,1.08698205,12,120.2856
4096,1,179.35423266,0\C,1,4.37344136,15,106.96668228,14,56.58188602,0\
C,23,1.40005389,1,59.14345221,15,42.51123016,0\C,24,1.39460299,23,120.
1257761,1,-0.33568272,0\C,25,1.41277811,24,120.98197926,23,1.22752455,
0\C,26,1.4035421,25,118.24288577,24,-1.77241191,0\C,23,1.39495727,1,60
.30781277,26,18.26790276,0\H,23,1.086897,1,179.22742311,26,165.7054985
4,0\H,24,1.08704457,23,120.28286008,1,-179.23948858,0\H,25,1.08757099,
24,119.16049137,23,-177.89156435,0\H,27,1.08382252,26,120.6440181,25,-

177.32744519,0\H,28,1.08692088,23,120.21851452,1,179.0731803,0\C,1,4.3
7385293,26,113.21990085,25,169.98075145,0\C,34,1.39983431,1,59.2153471
,26,66.41130114,0\C,35,1.39477785,34,120.12600869,1,0.01430151,0\C,36,
1.41120448,35,120.98644499,34,-0.39289156,0\C,37,1.40224069,36,118.313
15966,35,1.27579572,0\C,34,1.39471433,1,60.19490948,26,-114.04208377,0
\H,34,1.08679814,1,179.42556133,26,64.01238282,0\H,35,1.08708148,34,12
0.26123034,1,179.91258902,0\H,36,1.08832367,35,118.85250396,34,179.612
33373,0\H,38,1.08415965,37,120.52171314,36,178.39935857,0\H,39,1.08693
371,34,120.2077187,1,-179.70500749,0\N,5,1.35040993,4,30.1042188,3,-17
9.41734751,0\\Version=AM64L-G03RevD.01\State=1-A\HF=-974.8012037\MP2=-
978.3633805\RMSD=4.057e-09\Thermal=0.\PG=C01 [X(C24H20N1)]\\@

1-Ac^{+}

1\1\GINC-GRETEL\SP\RMP2-FC\6-31+G(2d,p)\C5H5N1\RAMAN\08-Dec-2011\0\\#P
MP2/6-31+G(2d,p) scf=(direct,tight) int=finegrid\\sp of pyridin mp2-5
on 2p 900 MB\\0,1\C,0,1.143013,-0.723299,-0.000216\C,0,1.200465,0.674
231,0.000236\C,0,-0.000002,1.388111,-0.000579\C,0,-1.200466,0.674229,0
.000237\C,0,-1.143011,-0.723301,-0.000219\H,0,2.06059,-1.311561,0.0007
13\H,0,2.160886,1.183548,0.001142\H,0,-2.160889,1.183543,0.001147\H,0,
-2.060587,-1.311565,0.000716\N,0,0.000002,-1.422833,-0.00005\H,0,-0.00
0002,2.476038,-0.000125\\Version=IA32L-G03RevD.01\State=1-A\HF=-246.71
80605\MP2=-247.5894333\RMSD=7.831e-09\Thermal=0.\PG=C01 [X(C5H5N1)]\\@

2

1\1\GINC-MAX\SP\RMP2-FC\6-31+G(2d,p)\H3N1\CHRISTOPH\18-Nov-2010\0\\#p
MP2(FC)/6-31+g(2d,p) scf=tight\\nh3sp_1\\0,1\N\H,1,1.01925971\H,1,1.01
92602,2,105.8645454\H,1,1.0192602,2,105.8645454,3,112.09886098,0\\Vers
ion=IA32L-G03RevD.01\State=1-A\HF=-56.2029409\MP2=-56.4081946\RMSD=1.3
07e-09\Thermal=0.\PG=C03 [C3(N1),X(H3)]\\@

2-Me^{+}

1\1\GINC-MAX\SP\RMP2-FC\6-31+G(2d,p)\C1H6N1(1+)\CHRISTOPH\18-Nov-2010\
0\\#p MP2(FC)/6-31+g(2d,p) scf=tight\\nh3mesp_1\\1,1\H\H,1,1.65526101\
H,1,1.65526002,2,60.00001949\N,3,1.0276137,1,36.35204349,2,-38.3280072
8,0\C,4,1.515337,3,111.56757139,1,-122.48483995,0\H,5,1.09095244,4,108
.19399949,3,-179.98928221,0\H,5,1.09095198,4,108.19400732,3,-59.989284
97,0\H,5,1.09095225,4,108.19400269,3,60.01069354,0\\Version=IA32L-G03R
evD.01\State=1-A\HF=-95.5916116\MP2=-95.9373084\RMSD=3.276e-09\Thermal
=0.\PG=C03 [C3(C1N1),X(H6)]\\@

3

1\1\GINC-MAX\SP\RMP2-FC\6-31+G(2d,p)\C1H5N1\CHRISTOPH\18-Nov-2010\0\\#
p MP2(FC)/6-31+g(2d,p) scf=tight\\nh2sp_1\\0,1\N\H,1,1.01882617\H,1,1.
01882617,2,105.76972823\C,1,1.466426,2,109.56897077,3,118.01889506,0\H
,4,1.09665455,1,109.04543807,2,63.8059177,0\H,4,1.09665455,1,109.04543
807,2,-179.43065322,0\H,4,1.10554008,1,115.69232196,2,-57.81236776,0\\
Version=IA32L-G03RevD.01\State=1-A'\HF=-95.2290092\MP2=-95.5813684\RMS
D=9.381e-09\Thermal=0.\PG=CS [SG(C1H1N1),X(H4)]\\@

3-Me^{+}

1\1\GINC-PHOBOS\SP\RMP2-FC\6-31+G(2d,p)\C2H8N1(1+)\CHRISTOPH\20-Nov-20
10\0\\#p MP2(FC)/6-31+g(2d,p) scf=tight\\nh2mesp_1\\1,1\H\H,1,1.635932
\N,1,1.02700314,2,37.20671071\C,3,1.50850839,1,109.14086497,2,-117.203
34277,0\H,4,1.09208094,3,108.77927208,1,57.46551033,0\H,4,1.09176804,3
,108.33486491,1,177.59142217,0\H,4,1.09176804,3,108.33486491,1,-62.660
40151,0\C,3,1.50850839,1,109.14086497,4,-125.59331445,0\H,8,1.09176804
,3,108.33486491,1,-177.59142217,0\H,8,1.09208094,3,108.77927208,1,-57.
46551033,0\H,8,1.09176804,3,108.33486491,1,62.66040151,0\\Version=AM64

L-G03RevD.01\State=1-A1\HF=-134.6341346\MP2=-135.129746\RMSD=9.559e-09
\Thermal=0.\PG=C02V [C2(N1),SGV(C2H2),SGV'(H2),X(H4)]\\@

4

1\1\GINC-BORIX\SP\RMP2-FC\6-31+G(2d,p)\C13H13N1\CHRISTOPH\13-Feb-2012\
0\\#p MP2(FC)/6-31+g(2d,p) scf=tight\\nmeph2sp_1\\0,1\N\C,1,1.4588036\
H,2,1.10336173,1,112.54983123\H,2,1.09210811,1,109.01552789,3,119.6612
3438,0\H,2,1.09641628,1,110.20996042,4,119.01619495,0\C,1,4.24291896,2
,115.85140069,4,29.65627761,0\C,6,1.39893565,1,59.98164277,2,117.62822
039,0\C,7,1.39655918,6,120.53111924,1,0.00842033,0\C,8,1.40629841,7,12
0.3376155,6,1.01622528,0\C,9,1.40550661,8,118.88547997,7,-0.37686914,0
\C,10,1.39678265,9,120.49640431,8,-0.64625884,0\H,6,1.0873447,1,179.52
513435,9,176.89151168,0\H,7,1.08800021,6,120.07102639,1,-179.02475652,
0\H,8,1.08691778,7,120.32966465,6,-179.20678167,0\H,10,1.08774457,9,11
9.24099381,8,178.53009642,0\H,11,1.08790843,10,119.47316021,9,179.9756
5828,0\C,1,4.24013842,9,121.28966639,8,-44.81528205,0\C,17,1.40096451,
1,59.11597766,9,-30.6288894,0\C,18,1.39218933,17,120.96439711,1,0.5285
897,0\C,1,1.4055695,9,120.87030023,8,-43.06772695,0\C,20,1.41014973,1,
121.55879502,9,149.39370075,0\C,17,1.39512761,1,59.55486477,20,73.9170
8742,0\H,17,1.08701518,1,179.22411889,20,-177.53903064,0\H,18,1.088224
4,17,120.05002655,1,-178.63374494,0\H,19,1.0861369,18,119.88126969,17,
-178.6582092,0\H,21,1.0852631,20,120.4919836,1,-3.43589189,0\H,22,1.08
828477,17,120.09673246,1,179.40925051,0\\Version=AM64L-G03RevD.01\Stat
e=1-A\HF=-554.342572\MP2=-556.3654026\RMSD=6.151e-09\Thermal=0.\PG=C01
[X(C13H13N1)]\\@

4-Me$^+$

1\1\GINC-IBLIS\SP\RMP2-FC\6-31+G(2d,p)\C14H16N1(1+)\CHRISTOPH\15-Feb-2
012\0\\#p MP2(FC)/6-31+g(2d,p) scf=tight\\nmeph2mesp_1\\1,1\C\H,1,1.09
096856\H,1,1.0899279,2,109.83364007\H,1,1.09148669,3,110.96998538,2,-1
22.0908212,0\C,1,2.43704712,3,145.46081055,2,124.02694636,0\H,5,1.0914
8535,1,85.37859746,3,-142.62869383,0\H,5,1.09096716,1,91.09363725,3,10
7.19750939,0\H,5,1.08992695,1,145.45901281,3,-21.57677921,0\C,5,4.9103
4492,1,80.74854805,3,35.76034539,0\C,9,1.39951358,5,56.07302793,1,-125
.68527321,0\C,10,1.39535241,9,120.32989493,5,21.32415861,0\C,11,1.3989
8479,10,119.10154368,9,-0.0664921,0\C,12,1.39481296,11,121.40076313,10
,-0.05420508,0\C,9,1.39521123,5,66.86470543,1,34.22153647,0\H,9,1.0863
2963,5,161.70234247,1,150.6195534,0\H,10,1.0863569,9,120.36520391,5,-1
58.99859281,0\H,11,1.08652476,10,119.97774522,9,178.63113979,0\H,13,1.
08404657,12,122.3931921,11,179.97440523,0\H,14,1.08633999,9,120.374001
9,5,161.04806159,0\C,1,4.91033448,5,80.74920992,12,81.56307355,0\C,20,
1.39951303,1,56.06867545,5,-125.69872773,0\C,21,1.39535102,20,120.3299
4132,1,21.32402133,0\C,22,1.39898609,21,119.1015948,20,-0.0667067,0\C,
23,1.39481186,22,121.40065365,21,-0.05425185,0\C,20,1.39521026,1,66.86
864937,23,63.55397709,0\H,20,1.08632983,1,161.70211948,23,179.96430813
,0\H,21,1.08635834,20,120.36510936,1,-158.99875707,0\H,22,1.08652366,2
1,119.97790782,20,178.63146575,0\H,24,1.08404594,23,122.39348547,22,17
9.97400483,0\H,25,1.08634013,20,120.37411541,1,161.05009072,0\N,12,1.5
1541032,11,117.29770462,10,-177.14807779,0\\Version=AM64L-G03RevD.01\S
tate=1-A\HF=-593.7414035\MP2=-595.9225541\RMSD=4.915e-09\Thermal=0.\PG
=C01 [X(C14H16N1)]\\@

5

1\1\GINC-GRETEL\SP\RMP2-FC\6-31+G(2d,p)\C9H15N1\CHRISTOPH\11-Nov-2010\
0\\#p MP2(FC)/6-31+g(2d,p) scf=tight\\n15sp_1\\0,1\N\H,1,2.15985879\H,
1,2.16016211,2,74.98024788\C,1,1.4501057,2,96.05711614,3,95.84910686,0
\C,4,1.51358702,1,119.66680042,2,105.04178587,0\C,4,1.51388466,1,119.6
8554926,5,70.48058779,0\H,4,1.09829006,1,115.19526889,5,-144.71696497,

0\H,5,1.08761578,4,118.08213849,1,-141.62923993,0\H,5,1.08602232,4,117
.41554932,1,2.53173765,0\H,6,1.08762809,4,118.1737,1,141.66413765,0\H,
6,1.08607097,4,117.25707617,1,-2.53921129,0\C,1,1.45014119,4,110.95875
248,5,-154.90494929,0\C,12,1.51348464,1,119.62342043,4,81.307608,0\C,1
2,1.51384541,1,119.67509575,4,151.77760667,0\H,13,1.08586431,12,117.35
864346,1,2.49314687,0\H,13,1.08761555,12,118.08369295,1,-141.6224304,0
\H,14,1.08607578,12,117.24600136,1,-2.50704093,0\H,14,1.08762595,12,11
8.19584522,1,141.71018103,0\C,1,1.45009316,4,110.93820592,5,81.3004553
4,0\C,19,1.5135079,1,119.64874274,4,-154.85470188,0\C,19,1.51377802,1,
119.72168497,4,-84.36219764,0\H,20,1.08760444,19,118.08269048,1,-141.5
766901,0\H,20,1.08594918,19,117.38641646,1,2.54439336,0\H,21,1.0860956
3,19,117.28198388,1,-2.5247238,0\H,21,1.08763386,19,118.1872567,1,141.
70780632,0\\Version=IA32L-G03RevD.01\State=1-A\HF=-403.9119722\MP2=-40
5.4279626\RMSD=8.942e-10\Thermal=0.\PG=C01 [X(C9H15N1)]\\@

5-Me$^+$

1\1\GINC-MORITZ\SP\RMP2-FC\6-31+G(2d,p)\C10H18N1(1+)\CHRISTOPH\11-Nov-
2010\0\\#p MP2(FC)/6-31+g(2d,p) scf=tight\\n15mesp_7\\1,1\N\H,1,2.1203
3539\C,1,1.51013276,2,93.38687965\H,3,1.09005044,1,109.26520084,2,153.
40764868,0\H,3,1.0904463,1,109.6119632,2,31.418941,0\H,3,1.09112992,1,
107.5605778,2,-87.29619834,0\H,1,2.11717723,3,142.38458815,4,63.825882
73,0\C,1,1.51126563,3,106.3511581,4,-59.94045688,0\C,8,1.51031727,1,12
4.87556218,3,152.59948868,0\C,8,1.50975291,1,122.22011449,3,78.4703269
6,0\H,8,1.08498084,1,108.80612154,3,-61.88951537,0\H,9,1.08561356,8,12
0.15331605,1,-5.21551545,0\H,9,1.08723759,8,116.29299376,1,139.1193499
8,0\H,10,1.08533422,8,118.94472074,1,9.43295808,0\H,10,1.08668815,8,11
6.76444287,1,-134.95080185,0\C,1,1.5092958,3,113.29958888,8,123.430408
7,0\C,16,1.51048467,1,124.62245475,3,-34.42449554,0\C,16,1.51113506,1,
122.73974931,3,39.83059621,0\H,17,1.08717918,16,116.73668163,1,-138.38
757731,0\H,17,1.08638575,16,119.76625172,1,6.08645703,0\H,18,1.0867231
1,16,116.31603943,1,135.31573399,0\H,18,1.08634821,16,120.2353827,1,-8
.9653085,0\C,1,1.51633144,16,105.41732439,17,-151.58496998,0\C,23,1.50
844151,1,123.9211423,16,-160.07132078,0\C,23,1.50601801,1,123.03976884
,16,-85.57774192,0\H,24,1.08688078,23,116.21848977,1,-137.59634162,0\H
,24,1.08623288,23,120.57962626,1,6.88702516,0\H,25,1.08665311,23,116.9
8600452,1,136.35124011,0\H,25,1.08578201,23,118.83821266,1,-8.149638,0
\\Version=IA32L-G03RevD.01\State=1-A\HF=-443.3150335\MP2=-444.9883336\
RMSD=3.305e-09\Thermal=0.\PG=C01 [X(C10H18N1)]\\@

6

1\1\GINC-GOLEM\SP\RMP2-FC\6-31+G(2d,p)\C15H33N1\CHRISTOPH\05-Dec-2010\
0\\#p MP2(FC)/6-31+g(2d,p) scf=tight\\n41sp_18\\0,1\N\C,1,1.47909198\C
,1,1.4790085,2,110.89774245\C,1,1.4788683,3,110.91720905,2,-123.730251
4,0\C,2,1.56119426,1,118.78349226,4,-88.0787111,0\C,5,1.54119625,2,111
.07727831,1,-51.40751101,0\H,6,1.09804533,5,111.46034642,2,-60.3527940
2,0\H,6,1.09856627,5,110.46810632,2,-179.79223042,0\H,6,1.09508905,5,1
10.8682266,2,59.98529127,0\C,5,1.54754687,2,106.01470158,1,-170.047771
88,0\H,10,1.09785659,5,110.7018427,2,-179.5536666,0\H,10,1.09848412,5,
111.37441941,2,60.58026962,0\H,10,1.09889034,5,111.32323821,2,-59.8002
0137,0\H,2,1.09366313,1,107.01743946,4,151.89320681,0\H,2,1.10918775,1
,110.31226649,4,37.4835346,0\H,3,1.09383037,1,107.04246813,4,28.098221
3,0\H,3,1.10931578,1,110.32855092,4,-86.35667628,0\H,4,1.1093174,1,110
.31922516,3,37.35341481,0\H,4,1.09367568,1,107.02477102,3,151.77788254
,0\C,5,1.54516554,2,113.443223,1,71.88230931,0\H,20,1.09526796,5,112.1
6335941,2,-69.70251709,0\H,20,1.09900623,5,111.45502777,2,51.95479005,
0\H,20,1.09858024,5,109.97491144,2,171.1152393,0\C,3,1.56113466,1,118.
74259786,4,148.14015375,0\C,24,1.54503202,3,113.39986909,1,71.65933293
,0\H,25,1.09529915,24,112.11198593,3,-69.41686711,0\H,25,1.09860974,24

,109.9564701,3,171.45287417,0\H,25,1.0989131,24,111.48982036,3,52.2559
7448,0\C,24,1.54117832,3,111.16076254,1,-51.57135659,0\H,29,1.0951617,
24,110.8707737,3,60.27979689,0\H,29,1.09791121,24,111.4752165,3,-60.09
774064,0\H,29,1.09856281,24,110.44771707,3,-179.53425545,0\C,24,1.5476
8829,3,106.05440609,1,-170.28298256,0\H,33,1.09845606,24,111.3932406,3
,60.78641303,0\H,33,1.09785207,24,110.64446801,3,-179.35236086,0\H,33,
1.09892634,24,111.34168969,3,-59.63369045,0\C,4,1.56141274,1,118.74016
537,3,-88.09559858,0\C,37,1.54138408,4,111.13039438,1,-51.70732119,0\H
,38,1.09515265,37,110.89994306,4,60.08297904,0\H,38,1.09858679,37,110.
45550017,4,-179.71091118,0\H,38,1.0979218,37,111.44867107,4,-60.286617
19,0\C,37,1.54753637,4,106.07815316,1,-170.38782121,0\H,42,1.09843463,
37,111.34850371,4,60.5747892,0\H,42,1.09785233,37,110.67128048,4,-179.
57716388,0\H,42,1.09884923,37,111.31855047,4,-59.83254781,0\C,37,1.544
98965,4,113.36933309,1,71.5250089,0\H,46,1.09868253,37,109.99910642,4,
171.35942104,0\H,46,1.09524807,37,112.11410552,4,-69.465382,0\H,46,1.0
9896224,37,111.48567218,4,52.18077489,0\\Version=AM64L-G03RevD.01\Stat
e=1-A\HF=-641.7329521\MP2=-644.2694716\RMSD=7.340e-09\Thermal=0.\PG=C0
1 [X(C15H33N1)]\\@

6-Me$^+$

1\1\GINC-NAUTILUS\SP\RMP2-FC\6-31+G(2d,p)\C16H36N1(1+)\CHRISTOPH\06-De
c-2010\0\\#p MP2(FC)/6-31+g(2d,p) scf=tight\\n41mesp_14\\1,1\N\C,1,1.4
9464943\H,2,1.08840084,1,109.04704021\H,2,1.08868541,1,109.03983403,3,
119.99561554,0\H,2,1.08848246,1,109.02682728,3,-119.9999444,0\C,1,1.55
124125,2,111.93335548,3,68.43149285,0\C,1,1.55076421,2,111.88669891,6,
120.05999678,0\C,1,1.55107022,2,111.95745283,7,120.08912769,0\H,6,1.08
973494,1,104.85539461,2,83.23837499,0\H,7,1.08986033,1,104.78409354,2,
82.47551307,0\H,7,1.09524386,1,103.2832334,2,-165.03390281,0\H,8,1.089
94332,1,104.75152023,2,82.57946048,0\H,8,1.09522648,1,103.32948803,2,-
164.94542323,0\C,8,1.56299762,1,124.1921056,2,-40.66022443,0\C,14,1.54
395575,8,115.75199359,1,-54.52046008,0\H,15,1.09770398,14,112.07414835
,8,-54.26827251,0\H,15,1.09738282,14,113.17983059,8,69.10461049,0\H,15
,1.09646249,14,109.00734598,8,-172.49449107,0\C,14,1.54874747,8,113.31
135395,1,73.56900713,0\H,19,1.09614806,14,109.14203596,8,164.30938314,
0\H,19,1.09442496,14,113.48249313,8,-76.9217314,0\H,19,1.0981434,14,11
1.64222587,8,46.03807646,0\C,14,1.55612169,8,102.22630974,1,-171.35730
026,0\H,23,1.09758131,14,111.80603022,8,-58.34867622,0\H,23,1.09727425
,14,111.76183918,8,63.25331564,0\H,23,1.09561854,14,109.50180845,8,-17
7.43850209,0\H,6,1.09530503,1,103.24579601,2,-164.29334484,0\C,6,1.563
37981,1,124.09307018,2,-40.25624719,0\C,28,1.54874535,6,113.38652816,1
,73.22991826,0\H,29,1.09612291,28,109.11427768,6,164.31186712,0\H,29,1
.0944089,28,113.50487888,6,-76.92687567,0\H,29,1.09803769,28,111.61790
05,6,46.07162819,0\C,28,1.55607272,6,102.2131187,1,-171.66203353,0\H,3
3,1.09755224,28,111.78339634,6,-58.43340414,0\H,33,1.09565226,28,109.5
223399,6,-177.5250457,0\H,33,1.09723329,28,111.75732797,6,63.15402725,
0\C,28,1.54416316,6,115.64876301,1,-54.87884544,0\H,37,1.09782114,28,1
12.05835776,6,-54.35388489,0\H,37,1.09642518,28,109.01070616,6,-172.56
079429,0\H,37,1.09741923,28,113.21599197,6,68.98886107,0\C,7,1.5629838
,1,124.11192681,2,-40.83756548,0\C,41,1.54892351,7,113.31617212,1,73.7
9537035,0\H,42,1.09611042,41,109.13202079,7,163.71874945,0\H,42,1.0980
6499,41,111.63251983,7,45.44361048,0\H,42,1.09455353,41,113.47567171,7
,-77.50970209,0\C,41,1.54392597,7,115.70256305,1,-54.2382121,0\H,46,1.
09738183,41,113.18119195,7,68.74615346,0\H,46,1.09645602,41,109.000796
64,7,-172.81792235,0\H,46,1.09773508,41,112.03413704,7,-54.60186134,0\
C,41,1.55625944,7,102.24860473,1,-171.05271148,0\H,50,1.09756651,41,11
1.82753265,7,-58.49628306,0\H,50,1.09728816,41,111.75260694,7,63.11961
563,0\H,50,1.09560081,41,109.49248469,7,-177.59952868,0\\Version=AM64L
-G03RevD.01\State=1-A\HF=-681.1370586\MP2=-683.8307229\RMSD=3.610e-09\

Thermal=0.\PG=C01 [X(C16H36N1)]\\@

7

1\1\GINC-MAX\SP\RMP2-FC\6-31+G(2d,p)\C2H7N1\CHRISTOPH\18-Nov-2010\0\\#
p MP2(FC)/6-31+g(2d,p) scf=tight\\nh1sp_1\\0,1\N\H,1,1.01833777\C,1,1.
45816308,2,109.01726169\H,3,1.09634357,1,109.58889705,2,55.59510721,0\
H,3,1.09816832,1,109.19508897,2,173.32140549,0\H,3,1.10857545,1,114.39
580628,2,-66.27008283,0\C,1,1.45816308,3,112.55925054,4,176.68651469,0
\H,7,1.09816832,1,109.19508897,3,65.58718702,0\H,7,1.09634357,1,109.58
889705,3,-176.68651469,0\H,7,1.10857545,1,114.39580628,3,-54.82132466,
0\\Version=IA32L-G03RevD.01\State=1-A'\HF=-134.2591371\MP2=-134.762062
7\RMSD=7.382e-09\Thermal=0.\PG=CS [SG(H1N1),X(C2H6)]\\@

7-Me⁺

1\1\GINC-MAX\SP\RMP2-FC\6-31+G(2d,p)\C3H10N1(1+)\CHRISTOPH\18-Nov-2010
\0\\#p MP2(FC)/6-31+g(2d,p) scf=tight\\nh1mesp_1\\1,1\H\N,1,1.026353\C
,2,1.50679627,1,107.10107954\H,3,1.09217678,2,108.86206707,1,60.125397
04,0\H,3,1.09243007,2,108.34167672,1,180.,0\H,3,1.09217678,2,108.86206
707,1,-60.12539704,0\C,2,1.50679662,1,107.10107546,3,-120.0000146,0\H,
7,1.09243006,2,108.34163586,1,180.,0\H,7,1.09217637,2,108.86205788,1,-
60.12539487,0\H,7,1.09217672,2,108.86202432,1,60.12542161,0\C,2,1.5067
9662,1,107.10107546,3,120.0000146,0\H,11,1.09217672,2,108.86202432,1,-
60.12542161,0\H,11,1.09217637,2,108.86205788,1,60.12539487,0\H,11,1.09
243006,2,108.34163586,1,-180.,0\\Version=IA32L-G03RevD.01\State=1-A1\H
F=-173.6738606\MP2=-174.3233141\RMSD=1.245e-09\Thermal=0.\PG=C03V [C3(
N1H1),3SGV(C1H1),X(H6)]\\@

8

1\1\GINC-MAX\SP\RMP2-FC\6-31+G(2d,p)\C7H13N1\CHRISTOPH\11-Nov-2010\0\\
#p MP2(FC)/6-31+g(2d,p) scf=tight\\n14sp_1\\0,1\N\C,1,1.46429919\H,2,1
.09561332,1,109.42022556\H,2,1.10770391,1,112.98366344,3,120.48454587,
0\H,2,1.09561297,1,109.42035391,3,-119.03078755,0\H,1,2.16211135,2,98.
3037302,5,82.29420177,0\C,1,1.4435564,2,111.31666019,5,-176.50918382,0
\C,7,1.51321253,1,119.21481858,2,148.66333916,0\C,7,1.51448276,1,118.1
3790943,2,78.92067952,0\H,7,1.09909214,1,115.83581609,2,-65.19340681,0
\H,8,1.08602837,7,117.23714917,1,-0.49185666,0\H,8,1.08763376,7,118.07
006847,1,143.73146174,0\H,9,1.08759976,7,118.35145494,1,-141.91199541,
0\H,9,1.08747822,7,116.85891572,1,2.23196958,0\C,1,1.44355645,7,112.21
764071,8,-85.82280581,0\C,15,1.51321339,1,119.21428249,7,85.8236431,0\
C,15,1.51448118,1,118.13753469,7,155.5659549,0\H,16,1.08602765,15,117.
23696551,1,0.49130742,0\H,16,1.08763381,15,118.06957149,1,-143.7320168
4,0\H,17,1.08747959,15,116.85897267,1,-2.23126985,0\H,17,1.08759983,15
,118.35133742,1,141.91264315,0\\Version=IA32L-G03RevD.01\State=1-A\HF=
-327.0407851\MP2=-328.269758\RMSD=2.059e-09\Thermal=0.\PG=C01 [X(C7H13
N1)]\\@

8-Me⁺

1\1\GINC-PHOENIX\SP\RMP2-FC\6-31+G(2d,p)\C8H16N1(1+)\CHRISTOPH\11-Nov-
2010\0\\#p MP2(FC)/6-31+g(2d,p) scf=tight\\n14mesp_7\\1,1\N\C,1,1.5080
2261\H,2,1.09113532,1,109.66839952\H,2,1.09116511,1,107.71586262,3,-11
9.70905658,0\H,2,1.09031202,1,109.25999542,4,-118.83758231,0\C,1,1.508
01627,2,108.28955209,5,-178.00712477,0\H,6,1.09113731,1,109.67029485,2
,60.55068978,0\H,6,1.09116594,1,107.71624952,2,-59.15718494,0\H,6,1.09
030895,1,109.25933785,2,-177.99675314,0\H,1,2.11627537,6,93.96279765,2
,149.76396034,0\C,1,1.51166872,6,113.78303191,2,-119.18743131,0\C,11,1
.50797936,1,123.717478,6,-31.48897433,0\C,11,1.50768584,1,123.23551381
,6,42.97011673,0\H,11,1.08661315,1,107.95723545,6,-174.2784555,0\H,12,
1.08669328,11,116.51706344,1,-137.38287479,0\H,12,1.08646755,11,120.16

551399,1,7.21035184,0\H,13,1.08673153,11,116.70696309,1,136.55900919,0
\H,13,1.08705947,11,119.91714546,1,-7.84791724,0\C,1,1.51170548,6,107.
28433485,2,123.20710741,0\C,19,1.50793572,1,123.71259104,6,-151.271466
27,0\C,19,1.50769776,1,123.23449266,6,-76.81419307,0\H,20,1.08669738,1
9,116.52142715,1,-137.38393895,0\H,20,1.08646929,19,120.16589534,1,7.2
0413138,0\H,21,1.0870501,19,119.92006274,1,-7.83706458,0\H,21,1.086729
66,19,116.69907684,1,136.57115148,0\\Version=AM64L-G03RevD.01\State=1-
A\HF=-366.4482366\MP2=-367.8311151\RMSD=2.304e-09\Thermal=0.\PG=C01 [X
(C8H16N1)]\\@

9

1\1\GINC-BORIX\SP\RMP2-FC\6-31+G(2d,p)\C8H11N1\CHRISTOPH\13-Feb-2012\0
\\#p MP2(FC)/6-31+g(2d,p) scf=tight\\nme2phsp_1\\0,1\N\C,1,1.45309418\
H,2,1.09764712,1,110.86761298\H,2,1.10386102,1,113.01418252,3,121.4760
526,0\H,2,1.09341033,1,109.01578108,3,-118.02943405,0\C,1,1.45308151,2
,117.73953038,5,23.69777101,0\H,6,1.10387024,1,113.01803218,2,96.74592
056,0\H,6,1.0976384,1,110.8643648,2,-141.77718469,0\H,6,1.09341341,1,1
09.0154011,2,-23.75061731,0\C,1,4.24052044,6,119.18058991,2,-157.59738
049,0\C,10,1.39746352,1,59.19087242,6,168.17692781,0\C,11,1.39519643,1
0,121.2100078,1,-0.72164254,0\C,1,1.39428397,6,119.00894826,2,-156.645
82998,0\C,13,1.4159703,1,121.29860787,6,-12.1011219,0\C,14,1.39520195,
13,120.89429211,1,-178.28448271,0\H,10,1.08689506,1,179.51834612,13,-1
79.97155318,0\H,11,1.08843634,10,120.03820485,1,179.59750798,0\H,12,1.
08485761,11,118.71192486,10,-179.519262,0\H,14,1.08485562,13,120.39465
179,1,1.72341305,0\H,15,1.08843636,14,118.75090859,13,179.83834527,0\\
Version=AM64L-G03RevD.01\State=1-A\HF=-363.8155685\MP2=-365.1545373\RM
SD=6.899e-09\Thermal=0.\PG=C01 [X(C8H11N1)]\\@

9-Me$^+$

1\1\GINC-IBLIS\SP\RMP2-FC\6-31+G(2d,p)\C9H14N1(1+)\CHRISTOPH\13-Feb-20
12\0\\#p MP2(FC)/6-31+g(2d,p) scf=tight\\nme2phmesp_2\\1,1\C\H,1,1.091
0307\H,1,1.09158123,2,109.78819515\H,1,1.09103087,2,110.79276572,3,121
.43151499,0\C,1,2.4392156,2,89.39099515,3,-86.99464348,0\H,5,1.0927811
3,1,86.82964956,2,106.96180119,0\H,5,1.091566,1,90.5110286,2,-3.686702
91,0\H,5,1.0896606,1,145.44428971,2,-131.49377984,0\C,5,5.00912674,1,7
8.26044025,2,-81.54656867,0\C,9,1.39832101,5,52.25322739,1,-139.734346
29,0\C,10,1.39588713,9,120.37934604,5,18.23667617,0\C,11,1.40049008,10
,119.36387087,9,0.,0\C,12,1.39636551,11,120.87664694,10,0.0007669,0\C,
9,1.39489196,5,69.5500692,1,23.39660396,0\H,9,1.08624198,5,163.1104640
4,1,151.60006137,0\H,10,1.08627685,9,120.40901434,5,-161.76350144,0\H,
11,1.08626383,10,118.72948633,9,179.99961363,0\H,13,1.08334935,12,122.
48828644,11,179.99881244,0\H,14,1.08629396,9,120.38789539,5,164.686930
94,0\C,1,2.43921489,5,61.0791948,12,70.01471991,0\H,20,1.08966182,1,14
5.4442374,5,-30.21847291,0\H,20,1.09277984,1,86.83033172,5,91.32722679
,0\H,20,1.09156664,1,90.51026329,5,-158.02411198,0\N,1,1.50922455,20,3
6.3589585,12,-33.28149302,0\\Version=AM64L-G03RevD.01\State=1-A\HF=-40
3.2243741\MP2=-404.7165751\RMSD=4.831e-09\Thermal=0.\PG=C01 [X(C9H14N1
)]\\@

10

1\1\GINC-IBLIS\SP\RMP2-FC\6-31+G(2d,p)\C12H27N1\CHRISTOPH\24-Nov-2010\
0\\#p MP2(FC)/6-31+g(2d,p) scf=tight\\n38sp_13\\0,1\N\C,1,1.47134764\C
,1,1.47135693,2,111.30033229\C,1,1.47144048,2,111.24916925,3,-124.6541
6961,0\C,2,1.54395031,1,114.41342443,3,-154.79437799,0\H,5,1.101072,2,
108.46785847,1,-54.0101192,0\C,3,1.54349837,1,114.45313064,2,80.921996
46,0\H,7,1.10101278,3,108.50684528,1,-54.8005781,0\C,4,1.54357359,1,11
4.4107556,2,-154.8195173,0\H,9,1.10103915,4,108.46401712,1,-54.2724536
9,0\C,7,1.53710716,3,111.52944615,1,64.02036425,0\H,11,1.09502252,7,11

0.86716988,3,-56.49512677,0\H,11,1.09782179,7,111.05678205,3,-177.1072
5558,0\H,11,1.09981831,7,110.73759706,3,63.31757996,0\C,7,1.5393154,3,
109.79663449,1,-172.32183699,0\H,15,1.09934103,7,110.85541874,3,-61.49
123673,0\H,15,1.0980037,7,111.57531643,3,58.59819415,0\H,15,1.09731,7,
111.03641254,3,179.02494772,0\C,9,1.53920391,4,109.803728,1,-171.77061
978,0\H,19,1.09937282,9,110.91917435,4,-61.31186333,0\H,19,1.09800599,
9,111.57132757,4,58.80164039,0\H,19,1.09730431,9,111.00478833,4,179.19
189702,0\C,9,1.53710423,4,111.56838301,1,64.54592413,0\H,23,1.09976993
,9,110.71237893,4,63.48747561,0\H,23,1.09773759,9,111.02756873,4,-177.
00753701,0\H,23,1.09513217,9,110.93780374,4,-56.38717623,0\C,5,1.53693
723,2,111.5109428,1,64.77125255,0\H,27,1.09978013,5,110.70436645,2,63.
72735646,0\H,27,1.09775079,5,111.07733649,2,-176.72277249,0\H,27,1.095
07627,5,110.86955834,2,-56.08488103,0\C,5,1.53926527,2,109.85534167,1,
-171.51936606,0\H,31,1.09728962,5,111.00178935,2,178.95811927,0\H,31,1
.0993966,5,110.92902533,2,-61.54563417,0\H,31,1.09799939,5,111.5568573
9,2,58.57688591,0\H,2,1.10998574,1,110.64817205,3,80.94490543,0\H,2,1.
09830955,1,108.20973329,3,-34.84056804,0\H,3,1.09835241,1,108.16072605
,2,-159.10353194,0\H,3,1.10976025,1,110.63192052,2,-43.32475576,0\H,4,
1.09817074,1,108.18473475,2,-34.80842904,0\H,4,1.10992674,1,110.663535
83,2,80.96275307,0\\Version=AM64L-G03RevD.01\State=1-A\HF=-524.6388826
\MP2=-526.6900565\RMSD=6.708e-09\Thermal=0.\PG=C01 [X(C12H27N1)]\\@

10-Me$^+$

1\1\GINC-NODE24\SP\RMP2-FC\6-31+G(2d,p)\C13H30N1(1+)\ZIP07\02-Dec-2010
\0\\#p MP2(FC)/6-31+G(2d,p) scf=tight\\n38mesp_95\\1,1\N\C,1,1.5005146
1\H,2,1.09022519,1,109.19791842\H,2,1.09029677,1,109.22299843,3,119.99
696564,0\H,2,1.09028318,1,109.21977621,3,-120.0142197,0\C,1,1.5445724,
2,111.04113812,3,-52.27507482,0\C,1,1.54483026,2,111.07769088,6,119.99
494856,0\C,1,1.54446907,2,111.05285073,6,-119.99674663,0\H,6,1.0963037
8,1,105.01207608,2,-160.5603931,0\H,6,1.09025134,1,105.19392598,2,86.0
0984537,0\H,7,1.09027676,1,105.24464568,2,85.46437497,0\H,7,1.09631006
,1,104.99780077,2,-161.09098194,0\H,8,1.09635461,1,105.03469651,2,-160
.77665134,0\H,8,1.09019584,1,105.20323173,2,85.76798264,0\C,8,1.544061
41,1,119.50837596,2,-35.87308267,0\H,15,1.09887552,8,108.51724823,1,57
.84888127,0\C,15,1.54716231,8,105.94160758,1,172.49410459,0\H,17,1.096
77843,15,111.95837855,8,-56.87346155,0\H,17,1.09771681,15,111.24932633
,8,64.37550115,0\H,17,1.09515778,15,109.82308676,8,-176.50757086,0\C,1
5,1.54042811,8,116.54583333,1,-65.14421618,0\H,21,1.09845258,15,111.39
378499,8,-54.77921719,0\H,21,1.09712381,15,113.59448112,8,67.99071155,
0\H,21,1.09577942,15,109.33894194,8,-173.09074752,0\C,7,1.54417576,1,1
19.54652169,2,-36.25145128,0\H,25,1.09892879,7,108.52268247,1,57.64329
516,0\C,25,1.54072608,7,116.55871842,1,-65.37578112,0\H,27,1.0983361,2
5,111.40968898,7,-55.0389572,0\H,27,1.0970508,25,113.61764669,7,67.732
50647,0\H,27,1.095762,25,109.33290926,7,-173.35044933,0\C,25,1.5472773
6,7,105.93703249,1,172.25646216,0\H,31,1.09768654,25,111.27113314,7,64
.36848152,0\H,31,1.09516694,25,109.81578591,7,-176.52668197,0\H,31,1.0
9676057,25,111.95614398,7,-56.89311697,0\C,6,1.54425005,1,119.58625412
,2,-35.66403928,0\H,35,1.09892421,6,108.57318194,1,57.83932759,0\C,35,
1.54703408,6,105.90240797,1,172.44411833,0\H,37,1.0967363,35,111.95037
032,6,-56.80537386,0\H,37,1.09776808,35,111.26560742,6,64.46672867,0\H
,37,1.09511694,35,109.81188799,6,-176.43451535,0\C,35,1.54071377,6,116
.58060949,1,-65.24784995,0\H,41,1.09840801,35,111.37675993,6,-54.45864
182,0\H,41,1.09714452,35,113.64323935,6,68.29010141,0\H,41,1.09583213,
35,109.34376632,6,-172.76759106,0\\Version=AM64L-G03RevD.01\State=1-A\
HF=-564.0453683\MP2=-566.2548954\RMSD=7.626e-09\Thermal=0.\PG=C01 [X(C
13H30N1)]\\@

11

1\1\GINC-IBLIS\SP\RMP2-FC\6-31+G(2d,p)\C11H25N1\CHRISTOPH\05-Dec-2010\
0\\#p MP2(FC)/6-31+g(2d,p) scf=tight\\n40sp_11\\0,1\N\C,1,1.47309036\C
,1,1.47664058,2,114.27497516\C,1,1.46476089,2,111.46389468,3,126.07474
342,0\C,2,1.55918896,1,117.37640687,4,-92.22912147,0\C,5,1.54077793,2,
110.40140379,1,-59.22282719,0\H,6,1.09845913,5,110.68332395,2,-176.542
94333,0\H,6,1.09525019,5,110.61285606,2,63.48487129,0\H,6,1.09864468,5
,111.35095867,2,-56.64172355,0\C,5,1.54369185,2,113.10941844,1,63.1413
0907,0\H,10,1.09880923,5,111.62237556,2,53.33217129,0\H,10,1.09854651,
5,110.09208968,2,172.69029646,0\H,10,1.09639577,5,111.56724441,2,-68.2
7917548,0\H,2,1.09760549,1,107.8471446,4,148.13053023,0\H,2,1.10910923
,1,110.53516942,4,32.48149267,0\H,3,1.10627716,1,109.9799553,4,-22.684
79911,0\H,3,1.09852557,1,108.99095557,4,-138.37694224,0\H,4,1.09228256
,1,110.79330413,2,57.00043567,0\H,4,1.10924873,1,112.17886885,2,-63.62
621824,0\H,4,1.09376374,1,110.22722494,2,176.0568579,0\C,5,1.54612694,
2,106.42584566,1,-177.83017869,0\H,21,1.09872063,5,111.35356296,2,-61.
27054073,0\H,21,1.09868901,5,111.38934697,2,59.14015428,0\H,21,1.09779
303,5,110.6363322,2,178.92845988,0\C,3,1.55724939,1,116.2221964,4,100.
80080249,0\C,25,1.54191881,3,111.84933073,1,-56.20663112,0\H,26,1.0983
8914,25,110.35747298,3,-178.19086094,0\H,26,1.09645166,25,110.96341399
,3,62.35336977,0\H,26,1.09892997,25,111.51622546,3,-58.74307889,0\C,25
,1.54454959,3,107.16178537,1,-175.85849855,0\H,30,1.0989412,25,111.353
35606,3,-58.96842787,0\H,30,1.09795389,25,110.72984816,3,-178.7056349,
0\H,30,1.09877416,25,111.38753244,3,61.41476148,0\C,25,1.54144936,3,11
0.13585175,1,65.34966435,0\H,34,1.09918656,25,111.21122604,3,58.373562
17,0\H,34,1.09841893,25,110.77055706,3,178.13080324,0\H,34,1.09466358,
25,110.75610267,3,-61.75999537,0\\Version=AM64L-G03RevD.01\State=1-A\H
F=-485.5891532\MP2=-487.495342\RMSD=6.210e-09\Thermal=0.\PG=C01 [X(C11
H25N1)]\\@

11-Me$^+$

1\1\GINC-YIN\SP\RMP2-FC\6-31+G(2d,p)\C12H28N1(1+)\CHRISTOPH\05-Dec-201
0\0\\#p MP2(FC)/6-31+g(2d,p) scf=tight\\n40mesp_9\\1,1\N\C,1,1.5002602
3\H,2,1.0918042,1,108.51723702\H,2,1.08819328,1,109.59123586,3,120.360
32361,0\H,2,1.09014466,1,108.81556644,3,-119.21092652,0\C,1,1.55281341
,2,111.81683031,4,70.88021859,0\C,1,1.51102771,2,107.88307069,6,115.51
188819,0\C,1,1.54771868,2,113.47462342,7,121.53913584,0\H,6,1.09504806
,1,104.23412913,2,78.13301727,0\H,7,1.08822345,1,109.53752274,2,65.954
66336,0\H,7,1.09237186,1,108.90218229,2,-173.72027997,0\H,8,1.08956771
,1,104.86797001,2,81.35289696,0\H,8,1.09529216,1,103.21903365,2,-165.9
1784862,0\C,8,1.56114209,1,123.5017025,2,-42.10958578,0\C,14,1.5438456
2,8,115.37597261,1,-53.66335462,0\H,15,1.09762148,14,113.03985258,8,72
.32050597,0\H,15,1.09755749,14,112.12461385,8,-51.22312356,0\H,15,1.09
618624,14,109.08135906,8,-169.65115102,0\C,14,1.54825665,8,113.1110579
8,1,74.21940324,0\H,19,1.09603967,14,109.18305049,8,165.18365937,0\H,1
9,1.09805524,14,111.59156915,8,46.87796313,0\H,19,1.09463727,14,113.45
958472,8,-76.03812295,0\C,14,1.55588566,8,102.27991459,1,-170.55313941
,0\H,23,1.09753738,14,111.80544534,8,-58.03301832,0\H,23,1.09726209,14
,111.77223895,8,63.59720538,0\H,23,1.09544795,14,109.44610744,8,-177.1
006167,0\H,6,1.09512564,1,103.35994799,2,-169.40981327,0\H,7,1.0925338
7,1,108.73392196,2,-54.11391874,0\C,6,1.56158263,1,123.96330733,2,-44.
69275453,0\C,29,1.55575805,6,102.28370233,1,-171.10572123,0\H,30,1.095
42997,29,109.40906025,6,-177.51612567,0\H,30,1.09752277,29,111.7998373
3,6,-58.49588022,0\H,30,1.09724956,29,111.80616727,6,63.15868455,0\C,2
9,1.5438512,6,115.41530587,1,-54.03304623,0\H,34,1.09764843,29,112.108
8487,6,-54.04516202,0\H,34,1.0962291,29,108.99361377,6,-172.35928877,0
\H,34,1.09763701,29,113.00007571,6,69.36569454,0\C,29,1.54841291,6,113
.13530742,1,73.65220933,0\H,38,1.09599785,29,109.14516312,6,163.944790
69,0\H,38,1.09806339,29,111.67790655,6,45.6385743,0\H,38,1.09450285,29
,113.44966026,6,-77.34736505,0\\Version=AM64L-G03RevD.01\State=1-A\HF=

-524.9974928\MP2=-527.0597746\RMSD=2.835e-09\Thermal=0.\PG=C01 [X(C12H
28N1)]\\@

12
see reference 4

12-Me$^+$
see reference 4

13
1\1\GINC-PHOBOS\SP\RMP2-FC\6-31+G(2d,p)\C5H11N1\CHRISTOPH\11-Nov-2010\
0\\#p MP2(FC)/6-31+g(2d,p) scf=tight\\n13sp_1\\0,1\N\C,1,1.45889652\H,
2,1.09576841,1,109.47892916\H,2,1.09723578,1,109.59618505,3,118.769714
91,0\H,2,1.10910864,1,113.16601263,3,-120.69183897,0\C,1,1.45889495,2,
111.65992761,3,-177.82329516,0\H,6,1.09576614,1,109.47920007,2,177.823
06405,0\H,6,1.10910596,1,113.16578689,2,-61.48384352,0\H,6,1.09723515,
1,109.59424324,2,59.0538629,0\C,1,1.4397213,6,111.81242342,2,126.12757
799,0\C,10,1.5137443,1,117.90498912,6,151.58598024,0\C,10,1.51375636,1
,117.9059123,6,82.36145873,0\H,10,1.09996676,1,116.36014993,6,-63.0279
4687,0\H,11,1.08726403,10,116.76091099,1,-0.67847179,0\H,11,1.08759854
,10,118.40030205,1,143.66421099,0\H,12,1.0872671,10,116.75762222,1,0.6
7539322,0\H,12,1.08760342,10,118.40982892,1,-143.66426196,0\\Version=A
M64L-G03RevD.01\State=1-A\HF=-250.1669184\MP2=-251.1098008\RMSD=9.898e
-09\Thermal=0.\PG=C01 [X(C5H11N1)]\\@

13-Me$^+$
1\1\GINC-PHOBOS\SP\RMP2-FC\6-31+G(2d,p)\C6H14N1(1+)\CHRISTOPH\11-Nov-2
010\0\\#p MP2(FC)/6-31+g(2d,p) scf=tight\\n13mesp_1\\1,1\N\C,1,1.51081
641\H,2,1.0916732,1,108.53370547\H,2,1.09259918,1,108.96543242,3,-120.
38783327,0\H,2,1.09215804,1,108.80203313,3,119.64926979,0\C,1,1.506814
2,2,108.6634076,3,62.22044946,0\H,6,1.09050221,1,109.45285844,2,178.75
612502,0\H,6,1.09204401,1,107.9850623,2,59.50091175,0\H,6,1.09050538,1
,109.45739791,2,-59.75349648,0\C,1,1.51082479,6,108.65396437,2,-119.16
217726,0\H,10,1.09260069,1,108.96438149,6,58.08319769,0\H,10,1.0916825
8,1,108.53222752,6,-62.30136265,0\H,10,1.09216133,1,108.80398577,6,178
.04484205,0\C,1,1.50940633,6,113.79625747,10,-120.41582669,0\C,14,1.50
776218,1,123.51512255,6,37.12714021,0\C,14,1.50773766,1,123.50283467,6
,-37.36674558,0\H,14,1.08716922,1,108.21526062,6,179.89048832,0\H,15,1
.08670353,14,120.01093366,1,-7.43280808,0\H,15,1.08667097,14,116.55310
785,1,136.93504746,0\H,16,1.086725,14,120.00961867,1,7.44867106,0\H,16
,1.08667244,14,116.55885715,1,-136.9109633,0\\Version=AM64L-G03RevD.01
\State=1-A\HF=-289.5793162\MP2=-290.6740885\RMSD=1.699e-09\Thermal=0.\
PG=C01 [X(C6H14N1)]\\@

14
see reference 4

14-Me$^+$
see reference 4

14-BH$^+$
1\1\GINC-CIPCLU10\SP\RMP2-FC\6-31+G(2d,p)\C19H18N1(1+)\C2175\14-Jan-20
11\0\\#p MP2(FC)/6-31+G(2d,p) scf=tight\\yin12bhsp_2\\1,1\C\H,1,1.0960
2583\C,1,4.32958235,2,107.79797869\C,3,1.39831307,1,59.19837486,2,27.0
7512867,0\C,4,1.39720151,3,120.11235822,1,-0.94382738,0\C,5,1.40309456
,4,120.31534926,3,0.36582886,0\C,6,1.40390088,5,119.35485215,4,-0.4602
0282,0\C,7,1.39794013,6,120.20885433,5,0.25243428,0\H,3,1.08678383,1,1
79.09813526,6,175.63460872,0\H,4,1.08681672,3,120.21564193,1,179.39572

794,0\H,5,1.08866411,4,119.73935496,3,179.75452186,0\H,7,1.08868796,6,
120.56813673,5,-179.46455106,0\H,8,1.08695422,7,119.67665591,6,-179.90
320346,0\C,1,4.3295474,6,117.3555495,5,-91.78290055,0\C,14,1.39814562,
1,60.54852848,6,-27.67943743,0\C,15,1.3985931,14,120.28849739,1,0.2391
8136,0\C,16,1.40397317,15,120.09362415,14,-0.26533799,0\C,17,1.4065625
8,16,119.27888042,15,0.61957451,0\C,18,1.39590583,17,120.49724875,16,-
0.54486591,0\H,14,1.08693416,1,179.34945561,17,150.16018265,0\H,15,1.0
8690144,14,120.12550355,1,179.48371168,0\H,16,1.08671459,15,119.726471
46,14,178.29552004,0\H,18,1.08936629,17,119.90524983,16,178.93152739,0
\H,19,1.08687032,18,119.84248075,17,179.44825065,0\C,1,4.32994141,17,1
13.32166087,16,98.53194084,0\C,25,1.41009081,1,57.57742638,17,164.4927
8101,0\C,26,1.38060817,25,120.36641419,1,-0.07401826,0\C,27,2.34487031
,26,91.31084333,25,-0.23859735,0\C,28,1.388031,27,90.82438984,26,0.425
55923,0\H,26,1.08620248,25,120.86484601,1,179.75608097,0\H,27,1.084947
45,26,122.84966343,25,178.68956023,0\H,28,1.08376206,27,146.24301905,2
6,-179.39873869,0\H,29,1.08591622,28,118.49917785,27,179.67713933,0\N,
28,1.34947406,27,30.04902147,26,-179.46555403,0\C,25,1.50433978,1,178.
6531817,17,145.01996937,0\H,35,1.09344171,25,111.72053983,1,-171.96610
853,0\H,35,1.0985541,25,110.01041967,1,-51.90655712,0\H,35,1.09624734,
25,110.90689679,1,66.49455529,0\\Version=AM64L-G03RevD.01\State=1-A\HF
=-784.3113862\MP2=-787.1630948\RMSD=7.629e-09\Thermal=0.\PG=C01 [X(C19
H18N1)]\\@

14-TT+

1\1\GINC-STEAK\SP\RMP2-FC\6-31+G(2d,p)\C25H22N1(1+)\CHRISTOPH\09-Nov-2
011\0\\#p MP2(FC)/6-31+g(2d,p) scf=tight\\yin12ttsp_1\\1,1\C\C,1,4.372
43476\C,2,1.4074165,1,57.30212566\C,3,1.38231284,2,120.55579576,1,0.38
098887,0\C,4,2.33835644,3,91.2524166,2,-0.43610632,0\C,5,1.3873895,4,9
0.89719863,3,0.34167947,0\H,3,1.08615893,2,120.90838386,1,-179.9584211
4,0\H,4,1.08322219,3,122.40687906,2,178.96302678,0\H,5,1.08237221,4,14
6.94800858,3,179.63495663,0\H,6,1.08607799,5,118.33110483,4,179.608769
34,0\C,1,4.3720497,4,99.87563884,3,-113.98389951,0\C,11,1.3999374,1,58
.34938949,4,-156.87914316,0\C,12,1.39416709,11,120.29433528,1,-0.24256
715,0\C,13,1.40958766,12,120.92852751,11,1.04681994,0\C,14,1.4016842,1
3,118.25274267,12,-1.92920136,0\C,11,1.39370447,1,60.95307382,14,10.53
719605,0\H,11,1.08673698,1,178.66789119,14,-176.40594753,0\H,12,1.0869
8676,11,120.25533749,1,-179.58469964,0\H,13,1.08710255,12,119.41102859
,11,-178.38891688,0\H,15,1.08564361,14,121.53899762,13,-177.14599067,0
\H,16,1.08703619,11,120.2633134,1,179.36761369,0\C,1,4.37443207,14,106
.91504151,13,56.44815219,0\C,22,1.39985608,1,59.12974878,14,42.8930968
1,0\C,23,1.39481841,22,120.13458559,1,-0.29529946,0\C,24,1.41235704,23
,120.99424783,22,1.25536537,0\C,25,1.40349849,24,118.22066887,23,-1.86
041866,0\C,22,1.39505566,1,60.30324293,14,-136.7139393,0\H,22,1.086914
48,1,179.2102368,14,7.17376009,0\H,23,1.08706401,22,120.28247898,1,-17
9.16646708,0\H,24,1.0876395,23,119.10412027,22,-177.83727047,0\H,26,1.
08396563,25,120.56222653,24,-177.25104659,0\H,27,1.08696365,22,120.206
52207,1,179.05068213,0\C,1,4.37450813,14,114.65100435,13,-71.3439626,0
\C,33,1.39979983,1,59.18785837,14,-168.76057951,0\C,34,1.39478927,33,1
20.1132301,1,0.02418809,0\C,35,1.41095511,34,121.01756542,33,-0.415782
04,0\C,36,1.40228407,35,118.2862682,34,1.3034584,0\C,33,1.39477451,1,6
0.21382677,14,10.77642782,0\H,33,1.08684395,1,179.41832134,14,-171.130
27569,0\H,34,1.08711439,33,120.26816399,1,179.95552903,0\H,35,1.088305
85,34,118.88378615,33,179.61809054,0\H,37,1.08429749,36,120.50564111,3
5,178.30735569,0\H,38,1.08697166,33,120.20871218,1,-179.701815,0\N,5,1
.35089523,4,30.4428893,3,-179.47216832,0\C,2,1.50429767,1,178.39268247
,14,117.57257094,0\H,45,1.09492783,2,111.38371022,1,-78.55039469,0\H,4
5,1.09920331,2,109.74788374,1,40.29676828,0\H,45,1.09433732,2,111.5382
2913,1,159.50194495,0\\Version=AM64L-G03RevD.01\State=1-A\HF=-1013.849
8685\MP2=-1017.5656362\RMSD=9.578e-09\Thermal=0.\PG=C01 [X(C25H22N1)]\

\@

15

1\1\GINC-CALYPSO\SP\RMP2-FC\6-31+G(2d,p)\C7H17N1\CHRISTOPH\05-Dec-2010
\0\\#p MP2(FC)/6-31+g(2d,p) scf=tight\\n39sp_6\\0,1\N\C,1,1.47240059\C
,1,1.45724066,2,113.28261192\C,1,1.45724257,3,111.3687839,2,-129.08377
681,0\C,2,1.55397718,1,114.05806062,3,115.91664951,0\C,5,1.54186616,2,
109.90392472,1,60.22364197,0\H,6,1.09827543,5,110.85550434,2,-179.5329
043,0\H,6,1.09642392,5,110.43225177,2,-59.62739118,0\H,6,1.09931655,5,
111.22251284,2,60.71125944,0\C,5,1.54187215,2,109.90704489,1,-60.21402
532,0\H,10,1.09931562,5,111.22306946,2,-60.6994127,0\H,10,1.0964267,5,
110.43325232,2,59.64157801,0\H,10,1.09827491,5,110.85490457,2,179.5447
6198,0\H,2,1.10220864,1,109.80149266,3,-122.08520402,0\H,2,1.10222338,
1,109.80604063,3,-6.08980747,0\H,3,1.09569849,1,109.93775318,4,175.807
22151,0\H,3,1.0974873,1,109.58549616,4,57.51083489,0\H,4,1.09570319,1,
109.93715957,3,-175.79939182,0\H,4,1.09748601,1,109.58474568,3,-57.503
95491,0\H,4,1.1097115,1,113.41380972,3,63.07256418,0\H,3,1.10971448,1,
113.41266348,4,-63.06601595,0\C,5,1.54275258,2,108.11992458,1,-179.995
73335,0\H,22,1.09805183,5,110.85162184,2,-179.99807221,0\H,22,1.098972
75,5,111.32742375,2,60.17249152,0\H,22,1.09897313,5,111.3267879,2,-60.
16886473,0\\Version=AM64L-G03RevD.01\State=1-A\HF=-329.4424897\MP2=-33
0.7226961\RMSD=1.420e-09\Thermal=0.\PG=C01 [X(C7H17N1)]\\@

15-Me$^+$

1\1\GINC-GOLEM\SP\RMP2-FC\6-31+G(2d,p)\C8H20N1(1+)\CHRISTOPH\05-Dec-20
10\0\\#p MP2(FC)/6-31+g(2d,p) scf=tight\\n39mesp_1\\1,1\N\C,1,1.508703
36\H,2,1.0927317,1,108.48020568\H,2,1.09229846,1,108.79964788,3,119.41
827924,0\H,2,1.08809011,1,109.6447147,3,-120.11453709,0\C,1,1.51140474
,2,107.69701706,5,-179.37473407,0\C,1,1.50478037,2,109.29395497,6,117.
33522604,0\C,1,1.54866103,7,113.15093348,6,118.0150362,0\H,6,1.0925735
7,1,108.86769911,7,-62.80945513,0\H,6,1.09236177,1,108.95742593,7,177.
26233548,0\H,7,1.08939116,1,109.30094888,2,56.25788102,0\H,7,1.0915818
9,1,109.07934886,2,176.87898409,0\H,8,1.09510976,1,104.25920793,7,-75.
77937603,0\H,8,1.09525128,1,103.2554546,7,171.62158721,0\C,8,1.5589247
2,1,123.3187147,7,47.38301158,0\C,15,1.55580604,8,102.3731866,1,169.40
479102,0\H,16,1.09717028,15,111.74768219,8,-64.33741405,0\H,16,1.09746
538,15,111.82003123,8,57.37981256,0\H,16,1.09539137,15,109.41871786,8,
176.379271,0\C,15,1.54761587,8,112.98612371,1,-75.25497575,0\H,20,1.09
791503,15,111.59347252,8,-47.81598941,0\H,20,1.09493157,15,113.6141363
3,8,75.12188041,0\H,20,1.09582685,15,109.12512879,8,-166.09144016,0\C,
15,1.54382349,8,114.9851074,1,52.38492206,0\H,24,1.09598367,15,109.026
91202,8,169.02816823,0\H,24,1.09799347,15,112.96746026,8,-73.02845476,
0\H,24,1.09744372,15,112.16669002,8,50.54508612,0\H,6,1.09213804,1,109
.05371649,7,57.16923159,0\H,7,1.09292181,1,108.39769553,2,-63.35646863
,0\\Version=AM64L-G03RevD.01\State=1-A\HF=-368.8552887\MP2=-370.288869
5\RMSD=8.081e-09\Thermal=0.\PG=C01 [X(C8H20N1)]\\@

16

1\1\GINC-CIPCLU02\SP\RMP2-FC\6-31+G(2d,p)\C9H21N1\C2175\22-Nov-2010\0\
\#p MP2(FC)/6-31+G(2d,p) scf=tight\\n32sp_2\\0,1\N\C,1,1.46527027\H,2,
1.09793336,1,105.90305347\C,1,1.46525662,2,118.60380192,3,-16.43733782
,0\H,4,1.09812623,1,105.88638165,2,-172.26327461,0\C,1,1.46533944,4,11
8.57777884,2,156.58015353,0\H,6,1.09816792,1,105.98404055,4,-172.71509
591,0\C,6,1.54443914,1,111.33394791,4,71.89133686,0\H,8,1.09836259,6,1
11.46393386,1,-64.67003601,0\H,8,1.09618565,6,110.21698207,1,55.156234
45,0\H,8,1.09829719,6,110.87777582,1,175.65487685,0\C,6,1.54918197,1,1
15.24909751,4,-55.02352652,0\H,12,1.09833367,6,110.38491486,1,-176.464
6661,0\H,12,1.09820537,6,111.47469006,1,64.34821578,0\H,12,1.09625416,

6,111.31026896,1,-56.40959713,0\C,2,1.54897005,1,115.17886623,4,101.27
166727,0\H,16,1.09828083,2,110.38697482,1,-176.23127634,0\H,16,1.09617
584,2,111.26239315,1,-56.13368991,0\H,16,1.09796832,2,111.41446327,1,6
4.5703272,0\C,2,1.54443048,1,111.40310305,4,-131.77445285,0\H,20,1.098
31296,2,111.50789185,1,-65.43628654,0\H,20,1.09614353,2,110.22828592,1
,54.42637532,0\H,20,1.09828632,2,110.8895054,1,174.90345485,0\C,4,1.54
869026,1,115.18540411,2,-54.56422472,0\H,24,1.09620653,4,111.2670763,1
,-55.55907907,0\H,24,1.0980761,4,111.41644053,1,65.14373069,0\H,24,1.0
9830429,4,110.41695355,1,-175.66960912,0\C,4,1.54450792,1,111.37719652
,2,72.36826223,0\H,28,1.09824758,4,111.47674337,1,-65.21959826,0\H,28,
1.09622495,4,110.2423985,1,54.64773207,0\H,28,1.09831083,4,110.8827765
,1,175.12096504,0\\Version=AM64L-G03RevD.01\State=1-A\HF=-407.513861\M
P2=-409.1048737\RMSD=6.848e-09\Thermal=0.\PG=C01 [X(C9H21N1)]\\@

16-Me$^+$

1\1\GINC-PHOBOS\SP\RMP2-FC\6-31+G(2d,p)\C10H24N1(1+)\CHRISTOPH\03-Dec-
2010\0\\#p MP2(FC)/6-31+g(2d,p) scf=tight\\n32mesp_3\\1,1\N\C,1,1.5059
9106\H,2,1.09151649,1,108.85000164\H,2,1.08767026,1,109.82030197,3,120
.22037611,0\H,2,1.08749586,1,109.90801783,3,-119.6420495,0\C,1,1.57465
065,2,106.65988106,5,-65.23901725,0\H,6,1.09210756,1,102.70028402,2,-6
0.85501328,0\C,1,1.57005638,2,108.41090095,6,-118.08201724,0\H,8,1.089
20165,1,103.87824063,2,-172.08834159,0\C,1,1.58853872,2,110.35599058,6
,118.7546216,0\H,10,1.09116067,1,102.30129829,2,-93.23129348,0\C,10,1.
53908831,1,114.77499805,2,22.00466967,0\H,12,1.09268119,10,113.6416769
3,1,-58.90098967,0\H,12,1.0939758,10,113.63477032,1,66.4194547,0\H,12,
1.09582227,10,106.5266616,1,-176.01354603,0\C,10,1.53761467,1,114.3893
7041,2,150.54779344,0\H,16,1.09171938,10,114.06749479,1,51.04132721,0\
H,16,1.09432437,10,112.8623223,1,-74.07312916,0\H,16,1.09596559,10,106
.64851426,1,168.6869897,0\C,8,1.53765221,1,112.68371557,2,-54.8214067,
0\H,20,1.09567824,8,107.25213487,1,-165.11172479,0\H,20,1.09521017,8,1
12.31581895,1,77.62962138,0\H,20,1.09157705,8,113.95453561,1,-46.82357
893,0\C,8,1.53809703,1,113.75201184,2,70.55841997,0\H,24,1.09301895,8,
113.80083231,1,67.0042686,0\H,24,1.09576389,8,106.85867813,1,-175.4515
273,0\H,24,1.09565103,8,113.10408523,1,-58.35733939,0\C,6,1.5385329,1,
114.0197932,2,54.52956847,0\H,28,1.0958573,6,106.56030324,1,-176.58717
378,0\H,28,1.09263724,6,113.83066172,1,-59.42212939,0\H,28,1.09536796,
6,113.6639691,1,66.24768624,0\C,6,1.53382065,1,113.53211698,2,-178.222
05931,0\H,32,1.09565629,6,107.29064568,1,168.92144814,0\H,32,1.0949061
,6,112.2619026,1,-73.81857139,0\H,32,1.09366607,6,113.9022645,1,50.783
91105,0\\Version=AM64L-G03RevD.01\State=1-A\HF=-446.9173711\MP2=-448.6
726794\RMSD=2.291e-09\Thermal=0.\PG=C01 [X(C10H24N1)]\\@

17

1\1\GINC-CIPCLU02\SP\RMP2-FC\6-31+G(2d,p)\C9H21N1\C2175\22-Nov-2010\0\
\#p MP2(FC)/6-31+G(2d,p) scf=tight\\n37sp_1\\0,1\N\C,1,1.46707833\C,1,
1.46910989,2,111.87563704\C,1,1.4598257,2,111.5388851,3,125.31248854,0
\C,2,1.54403082,1,114.69890052,4,-73.85078696,0\H,5,1.1008144,2,108.71
080978,1,61.71921278,0\C,3,1.54380839,1,114.13671639,4,155.09450362,0\
H,7,1.10092267,3,108.57743259,1,56.26452065,0\C,7,1.53907159,3,109.841
36804,1,173.7310375,0\H,9,1.09938839,7,110.90031214,3,61.22408797,0\H,
9,1.09730058,7,110.99465738,3,-179.28807396,0\H,9,1.09810743,7,111.588
29817,3,-58.89410817,0\C,7,1.53711834,3,111.24436886,1,-62.48596757,0\
H,13,1.09977871,7,110.6459076,3,-64.83200719,0\H,13,1.09771718,7,111.1
0179181,3,175.61347984,0\H,13,1.09509488,7,110.64775922,3,54.80967117,
0\C,5,1.53693965,2,111.57951924,1,-57.46921601,0\H,17,1.0978656,5,110.
99067614,2,178.53699185,0\H,17,1.09518152,5,110.92760074,2,57.63115303
,0\H,17,1.09964239,5,110.65178605,2,-61.92189634,0\C,5,1.53916047,2,10
9.51545249,1,178.78719315,0\H,21,1.09727477,5,110.98688229,2,-177.8849

4507,0\H,21,1.09926082,5,110.89239534,2,62.55858411,0\H,21,1.09807931,
5,111.55054592,2,-57.57730427,0\H,2,1.11166688,1,110.73193942,4,50.251
12089,0\H,2,1.09823694,1,107.93505838,4,166.28660432,0\H,3,1.11018075,
1,111.17223234,4,-80.27825415,0\H,3,1.09954918,1,107.61108032,4,35.645
09446,0\H,4,1.09624941,1,109.62282886,2,176.85620354,0\H,4,1.10973285,
1,113.09542062,2,-62.28317238,0\H,4,1.09535798,1,110.27686393,2,58.543
13237,0\\Version=AM64L-G03RevD.01\State=1-A\HF=-407.5232409\MP2=-409.1
083765\RMSD=7.428e-09\Thermal=0.\PG=C01 [X(C9H21N1)]\\@

17-Me$^+$
1\1\GINC-YIN\SP\RMP2-FC\6-31+G(2d,p)\C10H24N1(1+)\CHRISTOPH\20-Jan-201
1\0\\#p MP2(FC)/6-31+g(2d,p) scf=tight\\n37mesp_8\\1,1\N\C,1,1.5073053
9\H,2,1.09292651,1,108.50334775\H,2,1.09256971,1,109.04645938,3,119.70
508981,0\H,2,1.08977524,1,109.50847388,3,-119.70193059,0\C,1,1.5099002
7,2,107.01746065,5,-179.18648987,0\C,1,1.54178202,2,107.11153123,6,118
.69761551,0\C,1,1.53384566,2,111.23683774,6,-117.58289902,0\H,6,1.0923
028,1,108.5668847,2,-58.69019681,0\H,6,1.08807633,1,109.74082963,2,-17
8.91386248,0\H,6,1.09239301,1,108.77943555,2,60.7164999,0\H,7,1.096307
75,1,104.88108479,2,-71.06057428,0\H,7,1.09322922,1,104.97177575,2,42.
0573139,0\H,8,1.09274102,1,104.55760603,2,177.99135292,0\H,8,1.0961097
8,1,105.39910128,2,64.7375156,0\C,8,1.54996423,1,117.57442402,2,-58.59
059992,0\H,16,1.09969889,8,109.43933516,1,-28.37891184,0\C,16,1.543245
34,8,113.00142747,1,93.33828837,0\H,18,1.09523766,16,113.79051771,8,-6
0.06469589,0\H,18,1.09783864,16,110.38789624,8,61.23700815,0\H,18,1.09
538301,16,109.89818535,8,179.58925883,0\C,16,1.54496049,8,108.22658953
,1,-145.32775078,0\H,22,1.09772831,16,110.94844013,8,-59.77312854,0\H,
22,1.09701794,16,112.03590302,8,61.13864622,0\H,22,1.09507415,16,110.1
6541613,8,-178.75708694,0\C,7,1.54305712,1,120.0252193,2,164.27398941,
0\H,26,1.09842757,7,108.66211195,1,-59.59078072,0\C,26,1.54743655,7,10
5.65161821,1,-174.27085396,0\H,28,1.09751152,26,111.24617622,7,-65.578
61415,0\H,28,1.09672348,26,112.0576496,7,55.81424827,0\H,28,1.09490797
,26,109.66472192,7,175.39607574,0\C,26,1.54119166,7,116.27035387,1,63.
62568041,0\H,32,1.09782538,26,111.60338897,7,49.37260106,0\H,32,1.0955
6331,26,109.33884381,7,167.92288354,0\H,32,1.0967602,26,113.41188195,7
,-73.62063627,0\\Version=AM64L-G03RevD.01\State=1-A\HF=-446.9355314\MP
2=-448.6763519\RMSD=2.066e-09\Thermal=0.\PG=C01 [X(C10H24N1)]\\@

18
see reference 4

18-Me$^+$
see reference 4

19
1\1\GINC-CIPCLU10\SP\RMP2-FC\6-31+G(2d,p)\C6H15N1\C2175\02-Dec-2010\0\
\#p MP2(FC)/6-31+G(2d,p) scf=tight\\n36sp_1\\0,1\N\C,1,1.46513305\C,1,
1.45785974,2,111.32520922\C,1,1.45818121,3,110.53911779,2,-125.8374846
2,0\C,2,1.54337342,1,114.41457133,3,163.46491072,0\H,5,1.10079767,2,10
8.65921077,1,61.4324702,0\C,5,1.53710955,2,111.36929888,1,-57.7180764,
0\H,7,1.09777248,5,111.04665287,2,176.56444348,0\H,7,1.09958066,5,110.
56315919,2,-63.91304628,0\H,7,1.09508249,5,110.86571276,2,55.45711605,
0\C,5,1.53903993,2,109.65124024,1,178.50144442,0\H,11,1.09805356,5,111
.56161384,2,-58.00631583,0\H,11,1.09922744,5,110.9070194,2,62.17858809
,0\H,11,1.09730056,5,110.94694394,2,-178.30074891,0\H,2,1.11203119,1,1
11.21674596,3,-72.14028522,0\H,2,1.10006627,1,107.27977911,3,43.901413
12,0\H,3,1.09662016,1,109.76049465,4,176.06797111,0\H,3,1.09707252,1,1
09.57396815,4,57.54860774,0\H,4,1.09501731,1,110.5000603,3,-175.041121
58,0\H,4,1.09669949,1,109.42403083,3,-56.62751868,0\H,4,1.1102428,1,11

2.9407126,3,63.76578671,0\H,3,1.10998864,1,113.36474242,4,-62.99435981
,0\\Version=AM64L-G03RevD.01\State=1-A\HF=-290.407448\MP2=-291.5282606
\RMSD=8.617e-09\Thermal=0.\PG=C01 [X(C6H15N1)]\\@

19-Me⁺

1\1\GINC-CIPCLU07\SP\RMP2-FC\6-31+G(2d,p)\C7H18N1(1+)\C2175\01-Dec-201
0\0\\#p MP2(FC)/6-31+G(2d,p) scf=tight\\n36mesp_10\\1,1\N\C,1,1.508774
65\H,2,1.08813919,1,109.68675459\H,2,1.09226032,1,108.66025851,3,120.3
4896798,0\H,2,1.09266095,1,108.54274164,4,119.43242391,0\C,1,1.5054971
7,2,109.72374779,3,62.68842087,0\C,1,1.51018322,6,108.66909036,2,-117.
66107384,0\C,1,1.54335254,6,111.34915694,7,-118.24175303,0\H,6,1.09248
772,1,108.97387949,2,-177.74749242,0\H,6,1.09041537,1,109.37796291,2,-
57.26550968,0\H,6,1.09264222,1,108.55183038,2,62.59391003,0\H,7,1.0926
0276,1,108.94099496,6,63.90510917,0\H,7,1.09251208,1,108.84388559,6,-1
76.15507696,0\H,7,1.09214024,1,109.05259047,6,-56.13775903,0\H,8,1.096
07315,1,105.15329982,6,-167.53938685,0\H,8,1.09479011,1,104.39310844,6
,78.93974679,0\C,8,1.54238472,1,119.46607192,6,-42.4022835,0\H,17,1.09
892357,8,108.57021807,1,59.78000609,0\C,17,1.54752681,8,105.66037952,1
,174.37002237,0\H,19,1.09482288,17,109.61264634,8,-175.43156153,0\H,19
,1.09668553,17,112.05779088,8,-55.8978308,0\H,19,1.09752543,17,111.268
8081,8,65.54431291,0\C,17,1.54075379,8,116.21807296,1,-63.39630649,0\H
,23,1.09710175,17,113.41678854,8,72.60316305,0\H,23,1.09793023,17,111.
59108242,8,-50.45681819,0\H,23,1.09538848,17,109.31409563,8,-168.98900
269,0\\Version=AM64L-G03RevD.01\State=1-A\HF=-329.8232259\MP2=-331.096
4802\RMSD=8.611e-09\Thermal=0.\PG=C01 [X(C7H18N1)]\\@

20

1\1\GINC-EDDY\SP\RMP2-FC\6-31+G(2d,p)\C3H9N1\CHRISTOPH\06-Nov-2010\0\\
#p MP2(FC)/6-31+g(2d,p) scf=tight\\n0sp_1\\0,1\N\C,1,1.45616012\H,2,1.
09694704,1,109.64215005\H,2,1.09696341,1,109.65636723,3,118.54949224,0
\H,2,1.11037026,1,113.25373212,3,-120.72752193,0\C,1,1.45624397,2,111.
35817785,3,58.13957839,0\H,6,1.09695151,1,109.65305907,2,-58.22115241,
0\H,6,1.11039476,1,113.24892143,2,62.50315254,0\H,6,1.09696494,1,109.6
6042723,2,-176.7795744,0\C,1,1.45629002,2,111.34569829,6,124.91128115,
0\H,10,1.09695497,1,109.66812685,2,176.82580421,0\H,10,1.11039495,1,11
3.24452314,2,-62.45428711,0\H,10,1.09698123,1,109.66028225,2,58.259722
94,0\\Version=AM64L-G03RevD.01\State=1-A\HF=-173.2910362\MP2=-173.9486
528\RMSD=4.275e-09\Thermal=0.\PG=C01 [X(C3H9N1)]\\@

20-Me⁺

1\1\GINC-EDDY\SP\RMP2-FC\6-31+G(2d,p)\C4H12N1(1+)\CHRISTOPH\06-Nov-201
0\0\\#p MP2(FC)/6-31+g(2d,p) scf=tight\\n0mesp_1\\1,1\C\H,1,1.09220899
\H,1,1.0922388,2,110.10921927\H,1,1.09216082,2,110.07128615,3,-121.550
04951,0\C,1,2.46436713,4,89.43750085,2,89.18959916,0\H,5,1.09221421,1,
89.44917976,4,-110.08798345,0\H,5,1.09217148,1,89.41549246,4,-0.012174
78,0\H,5,1.09220104,1,144.1680022,4,124.89458155,0\C,1,2.46467264,5,60
.01154978,7,-89.6867591,0\H,9,1.09225043,1,144.14686111,5,-35.25246778
,0\H,9,1.09220528,1,89.45894638,5,89.68545587,0\H,9,1.09227026,1,89.45
747155,5,-160.24361038,0\C,9,2.46494868,1,60.00725152,5,-70.54173455,0
\H,13,1.0924331,9,144.01892555,1,35.16240855,0\H,13,1.0924169,9,89.417
58273,1,160.10070369,0\H,13,1.09244309,9,89.3391295,1,-89.69439044,0\N
,1,1.50919264,5,35.2694752,9,-35.2452008,0\\Version=AM64L-G03RevD.01\S
tate=1-A\HF=-212.7099793\MP2=-213.5174631\RMSD=1.378e-09\Thermal=0.\PG
=C01 [X(C4H12N1)]\\@

20-BH⁺

1\1\GINC-NAUTILUS\SP\RMP2-FC\6-31+G(2d,p)\C16H20N1(1+)\CHRISTOPH\16-Ju
n-2010\0\\#p MP2(FC)/6-31+g(2d,p) scf=tight\\nme3bhsp_3\\1,1\C\C,1,1.4

0805883\C,2,1.40827356,1,118.13635319\C,3,1.39708575,2,120.62043788,1,
-0.87155592,0\C,4,1.39820916,3,120.47976243,2,0.66934848,0\C,1,1.39660
474,2,121.23812416,3,0.407518,0\H,1,1.08897406,6,119.02333752,5,179.31
543554,0\H,3,1.08479736,2,121.30673262,1,178.22224483,0\H,4,1.08682541
,3,119.40579434,2,179.97407992,0\H,5,1.08671886,4,120.14029738,3,179.4
9749099,0\H,6,1.08674094,1,119.71891442,2,-179.4599399,0\C,2,1.5228333
,1,116.82116906,6,178.41940407,0\H,12,1.09428947,2,106.24622054,1,-2.7
6639767,0\C,12,4.34349564,2,118.88622302,1,-118.67809403,0\C,14,1.3969
5996,12,58.44344681,2,121.16022944,0\C,15,1.39660884,14,119.9214546,12
,0.65952936,0\C,16,1.40805742,15,121.23832549,14,-0.26520203,0\C,17,1.
40827832,16,118.13573707,15,-0.41100885,0\C,18,1.39708535,17,120.62077
646,16,0.87505148,0\H,14,1.08671795,12,178.57081996,2,96.09440714,0\H,
15,1.08674091,14,120.35912956,12,-179.06409062,0\H,16,1.08897356,15,11
9.02309859,14,-179.31438633,0\H,18,1.08479476,17,121.30676675,16,-178.
22115308,0\H,19,1.08682587,18,119.40601593,17,-179.97507888,0\C,12,2.5
3170881,2,145.77549032,1,96.49745576,0\H,25,1.09017005,12,90.43818033,
2,137.28597272,0\H,25,1.09253047,12,87.33556701,2,-112.8647445,0\H,25,
1.09277691,12,145.20664304,2,8.93771617,0\C,25,2.44356802,12,61.145258
64,2,-21.36925557,0\H,29,1.09253266,25,90.03474968,12,-86.92885024,0\H
,29,1.09277607,25,87.97798561,12,163.27100644,0\H,29,1.09017147,25,145
.02623295,12,39.45368816,0\C,25,2.44100769,12,62.8828403,2,47.53843658
,0\H,33,1.09044857,25,144.77807801,12,-36.88063224,0\H,33,1.09311125,2
5,88.19147165,12,-159.65864716,0\H,33,1.09044786,25,89.55829219,12,90.
56868297,0\N,33,1.50160894,25,35.78535548,12,-34.04320481,0\\Version=A
M64L-G03RevD.01\State=1-A\HF=-671.8268765\MP2=-674.3204703\RMSD=4.762e
-09\Thermal=0.\PG=C01 [X(C16H20N1)]\\@

21

1\1\GINC-CIPCLU04\SP\RMP2-FC\6-31+G(2d,p)\C12H27N1\C2175\22-Nov-2010\0
\\#p MP2(FC)/6-31+G(2d,p) scf=tight\\n35sp_6\\0,1\N\C,1,1.52662311\C,1
,1.52647262,2,117.73085972\C,1,1.52639968,3,117.67848764,2,150.2527091
6,0\C,2,1.55892103,1,114.42962851,4,-93.42179802,0\H,5,1.09552156,2,11
1.04817184,1,65.32211742,0\H,5,1.09863067,2,109.56788209,1,-175.907058
27,0\H,5,1.09139738,2,113.70293267,1,-56.10963415,0\C,2,1.55896595,1,1
14.88618418,4,27.42729261,0\H,9,1.08956179,2,115.01478885,1,-60.631353
95,0\H,9,1.09634229,2,106.64248408,1,-178.42189152,0\H,9,1.09363044,2,
111.89860671,1,64.71467501,0\C,2,1.56971439,1,110.75636933,4,141.31742
56,0\H,13,1.0968172,2,109.59352259,1,-151.59051399,0\H,13,1.09548033,2
,109.45651287,1,-33.5346589,0\H,13,1.08972909,2,114.61808915,1,89.0354
3565,0\C,3,1.55906347,1,114.44728392,4,56.73928223,0\H,17,1.09864531,3
,109.56545167,1,-175.59299921,0\H,17,1.09139484,3,113.67102477,1,-55.7
9277021,0\H,17,1.09563004,3,111.07107105,1,65.61657434,0\C,3,1.5592395
7,1,114.92618564,4,177.63389542,0\H,21,1.08951664,3,115.01812048,1,-60
.55061558,0\H,21,1.09360731,3,111.91032242,1,64.86315418,0\H,21,1.0963
0217,3,106.61273506,1,-178.30674934,0\C,3,1.56931303,1,110.69647817,4,
-68.52722491,0\H,25,1.09680603,3,109.57442347,1,-152.06652104,0\H,25,1
.09544752,3,109.39796874,1,-34.05474006,0\H,25,1.08964523,3,114.662199
58,1,88.52450766,0\C,4,1.55937813,1,114.90123508,3,27.98610539,0\H,29,
1.09626115,4,106.59297325,1,-178.43390959,0\H,29,1.0895887,4,115.06206
998,1,-60.64664189,0\H,29,1.09376017,4,111.91379942,1,64.75743094,0\C,
4,1.55956475,1,114.45426717,3,-92.85124042,0\H,33,1.09863474,4,109.555
31891,1,-175.43342931,0\H,33,1.09160002,4,113.75375912,1,-55.62819218,
0\H,33,1.09571669,4,111.07654953,1,65.80946436,0\C,4,1.56967665,1,110.
74016983,3,141.84266486,0\H,37,1.09540289,4,109.41722943,1,-33.1623045
4,0\H,37,1.09686361,4,109.58925495,1,-151.19249516,0\H,37,1.08966183,4
,114.66871812,1,89.43678918,0\\Version=AM64L-G03RevD.01\State=1-A\HF=-
524.5473196\MP2=-526.6359571\RMSD=8.631e-09\Thermal=0.\PG=C01 [X(C12H2
7N1)]\\@

21-Me$^+$

1\1\GINC-PHOENIX\SP\RMP2-FC\6-31+G(2d,p)\C13H30N1(1+)\CHRISTOPH\02-Dec
-2010\0\\#p MP2(FC)/6-31+g(2d,p) scf=tight\\n35mesp_1\\1,1\N\C,1,1.506
57416\H,2,1.08852686,1,109.88923973\H,2,1.08844177,1,109.92178318,3,12
0.00965821,0\H,2,1.08840832,1,109.90517257,3,-119.95654588,0\C,1,1.673
50869,2,105.27745758,5,-47.0550874,0\C,1,1.67424932,2,105.38984125,6,1
19.94729959,0\C,1,1.67280471,2,105.38376355,6,-120.09210701,0\C,7,1.55
633805,1,111.46248839,2,-39.68201773,0\H,9,1.08475,7,116.06686523,1,-5
9.08490739,0\H,9,1.09337633,7,112.53471547,1,67.44119307,0\H,9,1.09554
793,7,105.77656062,1,-176.81640697,0\C,7,1.55009932,1,112.64380509,2,7
5.53209761,0\H,13,1.09331158,7,113.20440662,1,-59.75783688,0\H,13,1.09
518706,7,105.68579103,1,-175.3250099,0\H,13,1.08851358,7,115.12741876,
1,67.61908294,0\C,7,1.54345958,1,112.55697664,2,-162.76426765,0\H,17,1
.08955935,7,113.88542982,1,69.70571499,0\H,17,1.08957559,7,113.6066795
9,1,-55.84823825,0\H,17,1.09600457,7,106.71019383,1,-173.1983416,0\C,8
,1.55610103,1,111.49427786,2,-40.0603996,0\H,21,1.09332166,8,112.54876
266,1,66.95245881,0\H,21,1.08478723,8,116.05268523,1,-59.58746651,0\H,
21,1.09551545,8,105.79193373,1,-177.33132562,0\C,8,1.55055565,1,112.62
448079,2,75.15789623,0\H,25,1.09528457,8,105.71966036,1,-175.25302121,
0\H,25,1.0931107,8,113.23162271,1,-59.59181337,0\H,25,1.08848573,8,114
.97478293,1,67.73388232,0\C,8,1.54344373,1,112.66044896,2,-163.2804802
8,0\H,29,1.09590137,8,106.62817045,1,-173.20012528,0\H,29,1.08947765,8
,113.58203639,1,-55.92351121,0\H,29,1.0896955,8,114.01467281,1,69.6656
0337,0\C,6,1.5571287,1,111.48612504,2,-39.83346251,0\H,33,1.09544516,6
,105.75466059,1,-177.34514982,0\H,33,1.08465995,6,116.05109889,1,-59.6
4603563,0\H,33,1.0934866,6,112.58926271,1,66.93215423,0\C,6,1.54384855
,1,112.80833632,2,-163.06810232,0\H,37,1.09591718,6,106.59410098,1,-17
4.48786948,0\H,37,1.08954869,6,113.59172876,1,-57.25123568,0\H,37,1.08
966939,6,114.05866052,1,68.44743359,0\C,6,1.55037343,1,112.55707931,2,
75.26263518,0\H,41,1.09526977,6,105.69731134,1,-175.48240356,0\H,41,1.
09328487,6,113.2177939,1,-59.83614264,0\H,41,1.08866954,6,115.06063111
,1,67.4855442,0\\Version=AM64L-G03RevD.01\State=1-A\HF=-563.9530739\MP
2=-566.2056112\RMSD=4.783e-09\Thermal=0.\PG=C01 [X(C13H30N1)]\\@

22

1\1\GINC-EDDY\SP\RMP2-FC\6-31+G(2d,p)\C4H11N1\CHRISTOPH\06-Nov-2010\0\
\#p MP2(FC)/6-31+g(2d,p) scf=tight\\n1sp_1\\0,1\N\C,1,1.45800912\H,2,1
.09497052,1,110.52818235\H,2,1.09665995,1,109.3225084,3,118.29880118,0
\H,2,1.11054656,1,113.06042744,4,120.30800271,0\C,1,1.45769788,2,110.5
1928971,3,-174.33904545,0\H,6,1.09687118,1,109.73443117,2,176.5406838,
0\H,6,1.11021325,1,113.3650446,2,-62.59585751,0\H,6,1.09695886,1,109.6
1673839,2,58.01509385,0\C,1,1.46501771,6,110.93145406,2,125.70840661,0
\H,10,1.09843633,1,106.98464091,6,48.6911514,0\H,10,1.11170301,1,111.4
4328928,6,-67.30273022,0\C,10,1.53288891,1,113.46143491,6,168.35095782
,0\H,13,1.09634815,10,112.40993305,1,68.46158986,0\H,13,1.09697869,10,
109.91459174,1,-172.06268219,0\H,13,1.09634013,10,110.25226301,1,-52.4
7119007,0\\Version=AM64L-G03RevD.01\State=1-A\HF=-212.3300071\MP2=-213
.1415229\RMSD=5.815e-09\Thermal=0.\PG=C01 [X(C4H11N1)]\\@

22-Me$^+$

1\1\GINC-CIPCLU03\SP\RMP2-FC\6-31+G(2d,p)\C5H14N1(1+)\C2175\09-Nov-201
0\0\\#p MP2(FC)/6-31+G(2d,p) scf=tight\\n1mesp_1\\1,1\N\C,1,1.50732642
\H,2,1.0905384,1,109.4099551\H,2,1.09257716,1,108.88340408,3,120.49366
408,0\H,2,1.09239576,1,108.62111538,4,119.75721881,0\C,1,1.50732543,2,
109.44476331,3,57.77682309,0\H,6,1.0905393,1,109.409825,2,-57.77855221
,0\H,6,1.09239557,1,108.62123767,2,61.97060021,0\H,6,1.09257784,1,108.
88334011,2,-178.27206179,0\C,1,1.50911753,6,108.79981681,2,-118.763521

45,0\H,10,1.09236513,1,108.95749133,6,59.58324857,0\H,10,1.09240182,1,
108.94870914,6,179.57223283,0\H,10,1.09240069,1,108.94860368,6,-60.405
77746,0\C,1,1.5350424,6,110.91157287,10,-118.54587437,0\H,14,1.0947961
8,1,105.32352483,6,61.99557661,0\H,14,1.09479512,1,105.32389515,6,176.
17210967,0\C,14,1.5253356,1,115.82847677,6,-60.91589755,0\H,17,1.09596
922,14,106.99434691,1,179.99862983,0\H,17,1.09438935,14,113.02165,1,-6
2.51097949,0\H,17,1.09438854,14,113.02169296,1,62.50809111,0\\Version=
AM64L-G03RevD.01\State=1-A\HF=-251.7497849\MP2=-252.7123162\RMSD=3.819
e-09\Thermal=0.\PG=C01 [X(C5H14N1)]\\@

23

1\1\GINC-CIPCLU09\SP\RMP2-FC\6-31+G(2d,p)\C9H21N1\C2175\22-Nov-2010\0\
\#p MP2(FC)/6-31+G(2d,p) scf=tight\\n34sp_2\\0,1\N\C,1,1.50355656\C,1,
1.50641545,2,122.73132656\C,1,1.46752786,2,112.53543831,3,-136.7553022
4,0\H,4,1.10629525,1,114.47136252,2,54.7542893,0\C,2,1.5529841,1,111.6
1467087,4,153.47936468,0\H,6,1.09738441,2,109.38811964,1,-162.21456202
,0\H,6,1.09626538,2,109.84827743,1,-43.58647977,0\H,6,1.0908022,2,113.
91631959,1,78.47401288,0\C,2,1.55181729,1,114.34306493,4,-80.93022553,
0\H,10,1.09517019,2,112.24501119,1,-66.87294727,0\H,10,1.09837781,2,11
0.25176441,1,173.48352185,0\H,10,1.09756141,2,111.14231255,1,54.347079
34,0\H,4,1.09103909,1,109.97437976,2,175.16167893,0\H,4,1.09388681,1,1
10.91014254,2,-67.58447425,0\C,2,1.5542828,1,108.67094139,4,38.6914604
8,0\H,16,1.0959867,2,108.71144731,1,172.38762333,0\H,16,1.09480245,2,1
13.12542187,1,-68.74355544,0\H,16,1.0969903,2,110.94589854,1,53.843898
41,0\C,3,1.55533539,1,113.7724846,4,49.96949096,0\H,20,1.09799043,3,10
9.96488844,1,-176.20357906,0\H,20,1.09572693,3,112.10812573,1,-57.0068
7354,0\H,20,1.0955265,3,111.7037721,1,64.28712498,0\C,3,1.55457983,1,1
07.30806522,4,-70.49950406,0\H,24,1.09755992,3,110.03799572,1,-174.758
97736,0\H,24,1.09584748,3,109.72880848,1,-55.19579327,0\H,24,1.0953865
3,3,113.277232,1,65.61565815,0\C,3,1.54426606,1,114.16591294,4,173.575
50536,0\H,28,1.09688165,3,108.01289837,1,174.50526577,0\H,28,1.0939924
1,3,113.18678434,1,-67.31022465,0\H,28,1.09451664,3,111.76070577,1,56.
32462388,0\\Version=AM64L-G03RevD.01\State=1-A\HF=-407.491716\MP2=-409
.0963966\RMSD=2.142e-09\Thermal=0.\PG=C01 [X(C9H21N1)]\\@

23-Me$^+$

1\1\GINC-PHOBOS\SP\RMP2-FC\6-31+G(2d,p)\C10H24N1(1+)\CHRISTOPH\02-Dec-
2010\0\\#p MP2(FC)/6-31+g(2d,p) scf=tight\\n34mesp_1\\1,1\N\C,1,1.5102
5185\H,2,1.08911071,1,109.31913525\H,2,1.08908711,1,110.23609658,3,120
.46318249,0\H,2,1.08926041,1,109.21886096,3,-118.67921141,0\C,1,1.5102
4704,2,106.19082539,4,-167.24415703,0\H,6,1.08910981,1,109.31921826,2,
72.30525352,0\C,1,1.63311934,6,107.58578617,2,-115.63007068,0\C,1,1.63
310217,6,108.16174679,2,115.23590268,0\C,8,1.55003215,1,109.01797278,6
,73.44647808,0\H,10,1.09483944,8,114.2330283,1,-54.36350713,0\H,10,1.0
9340375,8,113.18546441,1,71.25001283,0\H,10,1.0951066,8,106.62327511,1
,-171.56662354,0\C,8,1.53877811,1,111.98649121,6,-168.29793423,0\H,14,
1.09445823,8,113.01511155,1,-56.36667265,0\H,14,1.09558708,8,106.81092
651,1,-173.16410397,0\H,14,1.09137773,8,113.78739392,1,69.19107703,0\H
,6,1.08908803,1,110.23571064,2,-167.23115609,0\H,6,1.08925803,1,109.21
885966,2,-46.37393679,0\C,8,1.54385029,1,111.08627484,6,-44.69105624,0
\H,20,1.08891979,8,114.32229202,1,-57.78866756,0\H,20,1.09537629,8,106
.81914508,1,-175.81962052,0\H,20,1.09377779,8,112.67628459,1,67.414569
91,0\C,9,1.54385568,1,111.08653964,6,-159.01072847,0\H,24,1.09537511,9
,106.81919342,1,-175.82338315,0\H,24,1.08891717,9,114.32240739,1,-57.7
9232304,0\H,24,1.09377913,9,112.67644066,1,67.41064197,0\C,9,1.5500277
1,1,109.0166755,6,-40.87358671,0\H,28,1.09484139,9,114.23245305,1,-54.
36255434,0\H,28,1.0951056,9,106.62325381,1,-171.56571597,0\H,28,1.0934
0454,9,113.18544174,1,71.25062989,0\C,9,1.53878093,1,111.98532431,6,77

.38106253,0\H,32,1.09558624,9,106.81119232,1,-173.1615303,0\H,32,1.094
45889,9,113.01477878,1,-56.36396953,0\H,32,1.09137855,9,113.78743386,1
,69.19303156,0\\Version=AM64L-G03RevD.01\State=1-A\HF=-446.9045645\MP2
=-448.6682008\RMSD=3.157e-09\Thermal=0.\PG=C01 [X(C10H24N1)]\\@

24
see reference 4

24-Me+
see reference 4

24-BH+
1\1\GINC-CIPCLU07\SP\RMP2-FC\6-31+G(2d,p)\C17H17N2(1+)\C2175\17-Jan-20
11\0\\#p MP2(FC)/6-31+G(2d,p) scf=tight\\yin13bhsp_1\\1,1\C\H,1,1.0964
1081\C,1,4.3296912,2,107.62892588\C,3,1.39838375,1,59.13927612,2,-27.2
6635984,0\C,4,1.39695667,3,120.10324713,1,0.98022126,0\C,5,1.4027184,4
,120.35418699,3,-0.38740512,0\C,6,1.40379333,5,119.32932748,4,0.435364
65,0\C,7,1.39811536,6,120.21729662,5,-0.17479029,0\H,3,1.08680468,1,17
9.06179013,6,-175.41518723,0\H,4,1.08681982,3,120.20353477,1,-179.4144
4902,0\H,5,1.08862169,4,119.72548197,3,-179.93128942,0\H,7,1.0886694,6
,120.48018579,5,179.4874958,0\H,8,1.0870325,7,119.68085255,6,179.84572
566,0\C,1,4.33309912,6,117.16793801,5,91.91046077,0\C,14,1.39884344,1,
59.45836895,6,-144.72065456,0\C,15,1.39727153,14,119.9216615,1,0.37780
58,0\C,16,1.40535748,15,120.46036944,14,0.0853468,0\C,17,1.40393806,16
,119.32165737,15,0.32040549,0\C,18,1.39800821,17,120.10665261,16,-0.51
802156,0\H,14,1.08696205,1,179.47767706,6,-135.51363282,0\H,15,1.08693
015,14,120.25568845,1,179.70591274,0\H,16,1.08932194,15,119.64416785,1
4,179.33538858,0\H,18,1.08685617,17,120.1093981,16,177.87410812,0\H,19
,1.08696599,18,119.61799838,17,179.4155354,0\C,1,2.54533227,6,123.5978
4863,5,-165.88674193,0\H,25,1.07959346,1,96.91388145,6,-115.66048698,0
\N,25,1.34054376,1,137.24902667,6,65.61230404,0\C,27,1.46793352,25,125
.76487791,1,178.80298939,0\H,28,1.09315987,27,109.46088912,25,-119.927
29749,0\H,28,1.09149329,27,108.91589688,25,-0.3073551,0\H,28,1.0931939
,27,109.53898782,25,119.38607609,0\N,25,1.337558,1,28.36492619,6,63.18
735306,0\C,32,1.38370969,25,108.4298352,1,178.23456576,0\H,33,1.079729
,32,121.73648133,25,179.06279046,0\C,33,1.3648186,32,107.21782988,25,-
0.03969652,0\H,35,1.0802829,33,130.97870078,32,179.98294977,0\\Version
=AM64L-G03RevD.01\State=1-A\HF=-762.4309126\MP2=-765.1845194\RMSD=5.63
6e-09\Thermal=0.\PG=C01 [X(C17H17N2)]\\@

24-TT+
1\1\GINC-EVGENIX\SP\RMP2-FC\6-31+G(2d,p)\C23H21N2(1+)\CHRISTOPH\10-Nov
-2011\0\\#p MP2(FC)/6-31+g(2d,p) scf=tight\\yin13ttsp_3\\1,1\C\C,1,4.3
6701868\C,2,1.39924903,1,58.56104803\C,3,1.3951806,2,120.26966174,1,0.
49992001,0\C,4,1.4079978,3,120.75570207,2,-0.79017261,0\C,5,1.40152055
,4,118.54293936,3,1.08461169,0\C,2,1.39482164,1,60.85656098,5,3.918048
36,0\H,2,1.08676163,1,178.83610582,5,-179.69715496,0\H,3,1.08700496,2,
120.25348746,1,179.92694962,0\H,4,1.08712875,3,119.46933083,2,178.8515
9596,0\H,6,1.08590786,5,121.15780828,4,178.72615656,0\H,7,1.08704314,2
,120.20324833,1,-179.8674885,0\C,1,4.37560977,5,107.65704025,4,-53.564
40557,0\C,13,1.3998249,1,59.07477905,5,-45.22534722,0\C,14,1.39577122,
13,120.06895325,1,0.6500972,0\C,15,1.41129587,14,120.94221032,13,-1.36
531202,0\C,16,1.40219001,15,118.39467022,14,2.14895877,0\C,13,1.395269
56,1,60.37470805,5,133.88553515,0\H,13,1.08686229,1,179.28990495,5,-29
.24288148,0\H,14,1.08717728,13,120.29241305,1,179.19483726,0\H,15,1.08
821217,14,119.24625906,13,176.76657042,0\H,17,1.08412288,16,120.522583
93,15,177.25932624,0\H,18,1.08699939,13,120.14887887,1,-179.35119166,0
\C,1,4.37275474,5,114.06309192,4,75.33035545,0\C,24,1.39959597,1,59.25

112569,5,169.1588901,0\C,25,1.39512086,24,120.06232277,1,0.02502814,0\
C,26,1.41011789,25,120.98855828,24,0.34511127,0\C,27,1.40174281,26,118
.41138775,25,-1.04119326,0\C,24,1.39506165,1,60.18460384,5,-10.5535103
5,0\H,24,1.08683117,1,179.49957687,5,164.21420122,0\H,25,1.0870856,24,
120.29651961,1,-179.92432115,0\H,26,1.08822195,25,118.94080314,24,-179
.86285447,0\H,28,1.0844028,27,120.44916922,26,-178.63210293,0\H,29,1.0
8692906,24,120.16710085,1,179.66250299,0\C,1,2.57693623,5,112.48724918
,4,-144.1727028,0\N,35,1.34007377,1,137.19120277,5,-84.79542587,0\C,36
,1.38387458,35,108.44738686,1,-0.75408987,0\C,37,1.36424806,36,106.892
73605,35,-0.07911918,0\C,36,1.46745343,35,125.70777876,1,-179.27368536
,0\H,39,1.0930637,36,109.40086798,35,-123.37700334,0\H,39,1.09141345,3
6,108.89949081,35,-3.86862119,0\H,39,1.09332068,36,109.6249471,35,115.
95072524,0\H,35,1.07907641,1,97.4933345,5,95.4163588,0\H,37,1.08017897
,36,122.14648201,35,-179.66985393,0\H,38,1.0788956,37,130.92362398,36,
-179.15528994,0\N,35,1.33846094,1,28.02944084,5,-86.06731766,0\\Versio
n=AM64L-G03RevD.01\State=1-A\HF=-991.9730347\MP2=-995.5888623\RMSD=8.5
17e-09\Thermal=0.\PG=C01 [X(C23H21N2)]\\@

25

1\1\GINC-PHOBOS\SP\RMP2-FC\6-31+G(2d,p)\C6H13N1\CHRISTOPH\11-Nov-2010\
0\\#p MP2(FC)/6-31+g(2d,p) scf=tight\\n16sp_1\\0,1\N\H,1,2.15204462\C,
1,1.45852825,2,96.06558254\H,3,1.09610365,1,109.83344168,2,-157.703232
72,0\C,1,1.45852591,3,111.03178973,4,177.48230218,0\C,1,1.45535349,5,1
11.32520671,3,-124.63664852,0\C,6,1.55548537,1,117.48826044,5,-65.9324
5754,0\C,7,1.55571106,6,88.29305236,1,-139.60558686,0\C,6,1.55548594,1
,117.48888297,5,-169.5942567,0\H,7,1.0967582,6,109.92953374,1,-27.4777
5938,0\H,7,1.09576837,6,118.24484096,1,99.12003819,0\H,8,1.09636021,7,
111.78557214,6,-93.70158173,0\H,8,1.09501329,7,117.54925343,6,139.6803
1185,0\H,9,1.09576805,6,118.24432388,1,-99.12169129,0\H,9,1.09675767,6
,109.93024705,1,27.47636224,0\H,3,1.10989924,1,113.13635399,6,63.01248
186,0\H,3,1.09696942,1,109.53040574,6,-176.42011251,0\H,5,1.09610346,1
,109.83420482,6,57.89541072,0\H,5,1.09696921,1,109.53015392,6,176.4344
6172,0\H,6,1.11121476,1,111.60065326,5,62.23635685,0\\Version=AM64L-G0
3RevD.01\State=1-A\HF=-289.2097601\MP2=-290.3075295\RMSD=7.999e-09\The
rmal=0.\PG=C01 [X(C6H13N1)]\\@

25-Me⁺

1\1\GINC-GRETEL\SP\RMP2-FC\6-31+G(2d,p)\C7H16N1(1+)\CHRISTOPH\11-Nov-2
010\0\\#p MP2(FC)/6-31+g(2d,p) scf=tight\\n16mesp_1\\1,1\N\H,1,2.12909
871\C,1,1.5050159,2,137.91387924\H,3,1.09045274,1,109.37407241,2,-178.
31572333,0\H,3,1.09045204,1,109.37450978,2,60.80820754,0\H,3,1.0924998
9,1,108.39394501,2,-58.7541478,0\H,1,2.12910644,3,137.91240043,5,178.3
3880266,0\H,1,2.09245736,3,143.16118224,5,-60.43337131,0\C,1,1.5072172
1,3,108.88294046,5,179.25445518,0\C,1,1.50721708,3,108.88393653,9,-119
.34788309,0\C,1,1.53639688,3,112.64015911,9,120.32610562,0\C,11,1.5470
5417,1,120.86226102,3,55.61893723,0\C,12,1.56082403,11,86.3696864,1,-1
47.9604504,0\C,11,1.54705641,1,120.86359213,3,-55.58434203,0\H,12,1.09
5417,11,113.32101723,1,-37.05756293,0\H,12,1.09355152,11,117.23608746,
1,91.68975698,0\H,13,1.09192679,12,116.58671853,11,140.48748617,0\H,13
,1.09374864,12,112.0701288,11,-92.63507866,0\H,14,1.09541502,11,113.32
075806,1,37.05732686,0\H,14,1.09355225,11,117.23641207,1,-91.69061318,
0\H,9,1.0919958,1,108.85555542,3,61.31394801,0\H,9,1.09274994,1,109.00
364344,3,-58.76679492,0\H,10,1.09199462,1,108.85606891,3,-61.29463035,
0\H,10,1.09275007,1,109.00344171,3,58.78638769,0\\Version=IA32L-G03Rev
D.01\State=1-A\HF=-328.6296572\MP2=-329.8793033\RMSD=2.154e-09\Thermal
=0.\PG=C01 [X(C7H16N1)]\\@

26

1\1\GINC-CIPCLU04\SP\RMP2-FC\6-31+G(2d,p)\C5H13N1\C2175\22-Nov-2010\0\
\#p MP2(FC)/6-31+G(2d,p) scf=tight\\n30sp_1\\0,1\N\C,1,1.47820709\H,2,
1.11284244,1,109.70048783\C,1,1.46138305,2,112.52930414,3,62.01303909,
0\H,4,1.09362918,1,110.89374054,2,63.85673112,0\C,1,1.46138301,4,109.2
9231258,2,125.78476189,0\H,6,1.11023799,1,113.23881886,4,-67.6999947,0
\C,2,1.5400879,1,111.1482744,6,57.25552145,0\H,8,1.09643386,2,109.7869
7441,1,173.14087203,0\H,8,1.09504042,2,112.79917283,1,-67.38629634,0\H
,8,1.09761859,2,110.12243526,1,53.92423384,0\C,2,1.54008803,1,111.1453
4944,6,178.75401298,0\H,12,1.0976185,2,110.12246868,1,-53.92157805,0\H
,12,1.09643332,2,109.78760247,1,-173.13815588,0\H,12,1.09504242,2,112.
79782014,1,67.38831584,0\H,4,1.09623329,1,109.02109531,6,-52.31503572,
0\H,4,1.11023793,1,113.2385457,6,67.70872486,0\H,6,1.096234,1,109.0208
8977,4,52.32419391,0\H,6,1.09362611,1,110.8941266,4,170.36713248,0\\Ve
rsion=AM64L-G03RevD.01\State=1-A\HF=-251.3655216\MP2=-252.3349541\RMSD
=7.321e-09\Thermal=0.\PG=C01 [X(C5H13N1)]\\@

26-Me$^+$

1\1\GINC-PHOENIX\SP\RMP2-FC\6-31+G(2d,p)\C6H16N1(1+)\CHRISTOPH\02-Dec-
2010\0\\#p MP2(FC)/6-31+g(2d,p) scf=tight\\n30mesp_1_2\\1,1\N\C,1,1.50
921593\H,2,1.08965238,1,109.68232133\H,2,1.0923817,1,108.50088992,3,11
9.5994798,0\H,2,1.09280411,1,108.99583581,4,119.56547092,0\C,1,1.50939
646,2,107.44272842,3,-171.03858777,0\H,6,1.09251138,1,108.93965766,2,-
62.34239072,0\H,6,1.09230181,1,108.58237749,2,57.2668377,0\H,6,1.08924
57,1,109.80886918,2,176.88918589,0\C,1,1.50617737,2,108.71453477,6,117
.50761418,0\H,10,1.09266622,1,108.39796534,2,-54.6279205,0\H,10,1.0908
658,1,109.39174294,2,64.85803979,0\H,10,1.09049035,1,109.45121206,2,-1
74.21220708,0\C,1,1.56877551,10,112.21393045,2,-121.84321336,0\H,14,1.
09662021,1,102.37298332,10,-173.2279303,0\C,14,1.53338888,1,112.255721
34,10,-55.99421294,0\H,16,1.09372129,14,114.07915306,1,-53.40076332,0\
H,16,1.09537864,14,112.18261766,1,71.53156794,0\H,16,1.09533152,14,107
.26025564,1,-171.24360633,0\C,14,1.53359415,1,112.35965085,10,69.85533
185,0\H,20,1.09602302,14,112.58345352,1,-63.23088158,0\H,20,1.09326323
,14,113.57723271,1,61.71711622,0\H,20,1.09533561,14,107.28993076,1,179
.52065984,0\\Version=AM64L-G03RevD.01\State=1-A\HF=-290.7856152\MP2=-2
91.9065801\RMSD=8.125e-09\Thermal=0.\PG=C01 [X(C6H16N1)]\\@

27

1\1\GINC-CIPCLU03\SP\RMP2-FC\6-31+G(2d,p)\C5H13N1\C2175\09-Nov-2010\0\
\#p MP2(FC)/6-31+G(2d,p) scf=tight\\n4sp_5\\0,1\N\C,1,1.4579788\H,2,1.
0951901,1,110.53623787\H,2,1.09667476,1,109.37431569,3,-118.34445856,0
\C,1,1.4575206,2,110.57947192,3,174.41515349,0\H,5,1.09705283,1,109.61
646878,2,-57.65686423,0\H,5,1.11005347,1,113.33629777,2,62.92591381,0\
C,1,1.4655259,5,111.1599101,2,-125.76123287,0\H,8,1.09938606,1,107.061
88164,5,-46.05195788,0\H,8,1.11156546,1,111.27708191,5,70.03721426,0\C
,8,1.53827255,1,113.97459044,5,-165.6754255,0\H,11,1.09961068,8,107.42
260796,1,-179.75379614,0\H,11,1.09899381,8,110.24150305,1,-64.99304502
,0\C,11,1.53483537,8,113.26209803,1,58.60601174,0\H,14,1.09717128,11,1
11.17177448,8,-179.32914733,0\H,14,1.09542038,11,110.77607544,8,-58.49
588732,0\H,14,1.09839986,11,110.84729476,8,60.75822857,0\H,2,1.1103454
1,1,113.0022261,5,-64.30515406,0\H,5,1.09671728,1,109.74228497,2,-176.
19772586,0\\Version=AM64L-G03RevD.01\State=1-A\HF=-251.368258\MP2=-252
.3333452\RMSD=6.202e-09\Thermal=0.\PG=C01 [X(C5H13N1)]\\@

27-Me$^+$

1\1\GINC-CIPCLU02\SP\RMP2-FC\6-31+G(2d,p)\C6H16N1(1+)\C2175\09-Nov-201
0\0\\#p MP2(FC)/6-31+G(2d,p) scf=tight\\n4mesp_1\\1,1\N\C,1,1.5072395\
H,2,1.09050315,1,109.49416616\H,2,1.09256308,1,108.62800874,3,-119.805
46291,0\C,1,1.53547314,2,110.95993717,3,-64.88452395,0\H,5,1.0956608,1

,105.33199866,2,-176.09718985,0\H,5,1.09565955,1,105.33036367,2,-61.98
563354,0\C,1,1.50883355,2,108.72797964,5,-118.54492327,0\H,8,1.0922362
6,1,108.90458366,2,60.4436605,0\H,8,1.09240524,1,108.96231431,2,-59.55
556937,0\H,8,1.09223599,1,108.90476095,2,-179.55491912,0\C,1,1.5072432
4,2,109.47314694,8,-118.65120051,0\H,12,1.09256329,1,108.62787832,2,61
.90037285,0\H,12,1.09050126,1,109.49443002,2,-57.9048003,0\C,5,1.53055
8,1,116.54431752,2,60.95752559,0\H,15,1.09683012,5,110.85971982,1,59.7
6935735,0\H,15,1.09683166,5,110.85997526,1,-59.73151816,0\C,15,1.54121
65,5,109.4339609,1,-179.98117195,0\H,18,1.09429344,15,109.78436023,5,1
79.9995457,0\H,18,1.09638519,15,111.50167125,5,-60.6120995,0\H,18,1.09
638628,15,111.50153894,5,60.61127623,0\H,2,1.09259275,1,108.85039752,1
2,178.40271449,0\H,12,1.09259282,1,108.85063882,2,-178.38854782,0\\Ver
sion=AM64L-G03RevD.01\State=1-A\HF=-290.7897657\MP2=-291.905198\RMSD=5
.908e-09\Thermal=0.\PG=C01 [X(C6H16N1)]\\@

28

1\1\GINC-ANGIE\SP\RMP2-FC\6-31+G(2d,p)\C6H15N1\CHRISTOPH\15-Feb-2012\0
\\#p MP2(FC)/6-31+g(2d,p) scf=tight\\n42sp_1\\0,1\C\H,1,1.09761038\C,1
,2.40204748,2,89.83807001\H,3,1.0968826,1,144.09747548,2,-118.60866432
,0\C,3,2.45070345,1,61.39721169,2,-153.38697425,0\H,5,1.10095204,3,80.
24073637,1,166.70850179,0\C,5,1.54612513,3,102.84401765,1,-87.36658088
,0\H,7,1.09736617,5,110.34430907,3,-138.50389374,0\H,7,1.09717852,5,11
1.65539414,3,-18.83179266,0\H,7,1.09775417,5,111.50991283,3,102.042615
38,0\C,5,1.54428503,3,141.01678603,1,62.2507982,0\H,11,1.09985359,5,10
7.58038711,3,159.3107606,0\H,11,1.10016313,5,109.84519327,3,-86.192143
44,0\C,11,1.53431726,5,113.78793895,3,37.46256451,0\H,14,1.09850664,11
,110.90691522,5,60.68550754,0\H,14,1.0950276,11,110.75774557,5,-58.627
00339,0\H,14,1.09729777,11,111.09123478,5,-179.51030256,0\H,1,1.107842
8,3,94.69348257,5,99.04283329,0\H,1,1.09538272,3,144.83805798,5,-32.05
64663,0\H,3,1.09785475,1,89.19722907,5,155.14003846,0\H,3,1.10909596,1
,95.86780611,5,-97.4853229,0\N,3,1.45551709,1,34.47819433,5,28.0538186
9,0\\Version=AM64L-G03RevD.01\State=1-A\HF=-290.4045298\MP2=-291.52637
17\RMSD=1.788e-09\Thermal=0.\PG=C01 [X(C6H15N1)]\\@

28-Me$^+$

1\1\GINC-IBLIS\SP\RMP2-FC\6-31+G(2d,p)\C7H18N1(1+)\CHRISTOPH\15-Feb-20
12\0\\#p MP2(FC)/6-31+g(2d,p) scf=tight\\n42mesp_1\\1,1\C\H,1,1.089441
78\C,1,2.51914214,2,90.17286169\H,3,1.09732568,1,78.84696397,2,-102.07
853783,0\C,1,2.42866518,3,61.46184169,4,96.6420951,0\H,5,1.09246697,1,
84.43921928,3,167.27487714,0\C,3,1.54408653,1,97.12219442,5,-155.05613
058,0\H,7,1.09793048,3,109.46997199,1,112.28263501,0\H,7,1.09631737,3,
111.26477471,1,-5.70446506,0\C,3,1.53291795,1,144.28358934,5,-10.84051
915,0\H,10,1.09628391,3,112.49637338,1,-75.15329447,0\H,10,1.09315231,
3,113.43674712,1,49.57083343,0\H,10,1.09427794,3,107.71856477,1,167.37
646188,0\C,7,1.54081484,3,111.90177359,1,-126.10552872,0\H,14,1.095105
79,7,112.43540095,3,-62.10987286,0\H,14,1.09673268,7,111.33201525,3,59
.59315431,0\H,14,1.09467508,7,109.49902383,3,178.67311992,0\C,5,2.4490
2768,1,60.42507339,3,71.937383,0\H,18,1.09099283,5,92.37177436,1,-157.
91906443,0\H,18,1.09270249,5,85.98396963,1,92.57110391,0\H,18,1.090437
54,5,144.87205918,1,-26.67915343,0\H,5,1.08981812,1,145.46197506,18,-2
1.53348153,0\H,5,1.09267132,1,92.9905062,18,-155.03643627,0\H,1,1.0924
4033,5,88.6609589,18,-88.20037662,0\H,1,1.09233661,5,88.45597891,18,16
1.89609032,0\N,18,1.50631058,5,35.73300995,1,-37.25392121,0\\Version=A
M64L-G03RevD.01\State=1-A\HF=-329.8230009\MP2=-331.0983318\RMSD=9.865e
-09\Thermal=0.\PG=C01 [X(C7H18N1)]\\@

29

1\1\GINC-CIPCLU04\SP\RMP2-FC\6-31+G(2d,p)\C6H15N1\C2175\22-Nov-2010\0\

\#p MP2(FC)/6-31+G(2d,p) scf=tight\\n33sp_2\\0,1\N\C,1,1.4924229\C,1,1
.46004261,2,115.26465508\H,3,1.0972842,1,108.4924194,2,-172.82078943,0
\C,1,1.46004697,3,109.3558794,2,-131.76323847,0\H,5,1.1082509,1,114.23
460723,3,64.17882406,0\C,2,1.54632968,1,108.77090039,3,173.81516725,0\
H,7,1.09600848,2,112.55475427,1,71.77944948,0\H,7,1.09661891,2,109.834
91409,1,-169.05989908,0\H,7,1.09678018,2,110.27153653,1,-49.56834287,0
\C,2,1.54632024,1,108.76943547,3,57.28390061,0\H,11,1.09677991,2,110.2
7134401,1,49.56791449,0\H,11,1.09600868,2,112.55558639,1,-71.78243423,
0\H,11,1.09661993,2,109.83347539,1,169.05817049,0\H,3,1.09330446,1,110
.99557961,5,173.08687135,0\H,3,1.10825916,1,114.23452704,5,-64.1760644
,0\H,5,1.09330732,1,110.99525854,3,-173.08230574,0\H,5,1.09728235,1,10
8.49160395,3,-55.41240761,0\C,2,1.55056521,1,113.53205974,3,-64.448777
89,0\H,19,1.09722334,2,111.57452039,1,60.52029488,0\H,19,1.09768262,2,
110.42280685,1,179.99850434,0\H,19,1.0972218,2,111.57527667,1,-60.5230
038,0\\Version=AM64L-G03RevD.01\State=1-A\HF=-290.400316\MP2=-291.5288
769\RMSD=1.185e-09\Thermal=0.\PG=C01 [X(C6H15N1)]\\@

29-Me$^+$

1\1\GINC-PHOBOS\SP\RMP2-FC\6-31+G(2d,p)\C7H18N1(1+)\CHRISTOPH\02-Dec-2
010\0\\#p MP2(FC)/6-31+g(2d,p) scf=tight\\n33mesp_1\\1,1\N\C,1,1.50964
753\H,2,1.09262925,1,108.14519366\H,2,1.08991244,1,109.64736994,3,119.
30684174,0\H,2,1.09036165,1,109.81053666,3,-119.35760129,0\C,1,1.50983
163,2,107.44449852,4,53.14521004,0\H,6,1.09029608,1,109.85806164,2,-70
.10764618,0\C,1,1.60585412,2,111.45987198,6,-122.37709102,0\C,1,1.5098
8923,2,107.47096932,6,115.27338233,0\H,9,1.08991094,1,109.68814162,2,5
2.71068689,0\C,8,1.54014516,1,109.27111164,2,46.13920228,0\H,11,1.0942
206,8,112.86724803,1,-70.11078929,0\H,11,1.09542165,8,113.34292305,1,5
4.66212418,0\H,11,1.09519504,8,107.43589176,1,172.19834686,0\C,8,1.540
09617,1,109.21795152,2,166.09907408,0\H,15,1.09554731,8,113.37388811,1
,54.65584232,0\H,15,1.09427172,8,112.78059458,1,-70.0391311,0\H,15,1.0
951904,8,107.45913624,1,172.25974083,0\H,6,1.09263038,1,108.12808103,2
,49.23018255,0\H,6,1.08998364,1,109.63949583,2,168.53367008,0\H,9,1.09
03355,1,109.76018107,2,174.02999639,0\H,9,1.09268565,1,108.17296835,2,
-66.59835742,0\C,8,1.54011351,1,109.23678025,2,-73.92614853,0\H,23,1.0
9426535,8,112.77976491,1,-69.9306694,0\H,23,1.09516361,8,107.43091572,
1,172.36766642,0\H,23,1.09537979,8,113.38331488,1,54.78468545,0\\Versi
on=AM64L-G03RevD.01\State=1-A\HF=-329.8189796\MP2=-331.1015243\RMSD=4.
126e-09\Thermal=0.\PG=C01 [X(C7H18N1)]\\@

30

1\1\GINC-MAX\SP\RMP2-FC\6-31+G(2d,p)\C6H15N1\CHRISTOPH\12-Nov-2010\0\\
#p MP2(FC)/6-31+g(2d,p) scf=tight\\n7sp_2\\0,1\N\C,1,1.4658535\H,2,1.1
1170045,1,111.19165273\C,1,1.45803612,2,112.50970981,3,-55.47249739,0\
H,4,1.09512677,1,110.5306382,2,-60.90567972,0\H,4,1.09665204,1,109.367
27045,2,-179.26626945,0\C,1,1.45761063,4,110.53722269,2,-124.74815522,
0\H,7,1.1100762,1,113.27953364,4,62.82367482,0\H,7,1.09669191,1,109.75
170951,4,-176.31974332,0\H,2,1.0992727,1,107.06186218,7,-46.99182348,0
\C,2,1.53774319,1,114.11490161,7,-166.79588904,0\H,11,1.10070915,2,107
.47365801,1,-179.98331169,0\H,11,1.10016787,2,110.3777609,1,-65.268431
89,0\C,11,1.53708349,2,113.77572671,1,58.44171869,0\H,14,1.10082487,11
,108.97132499,2,57.67778364,0\H,14,1.09754066,11,109.03869681,2,-57.62
114575,0\C,14,1.53528343,11,112.85523092,2,179.58625596,0\H,17,1.09727
39,14,111.33532504,11,-179.6866469,0\H,17,1.09853173,14,111.30394145,1
1,-59.50736967,0\H,17,1.09846501,14,111.1780284,11,60.29862943,0\H,4,1
.11037489,1,112.99242052,7,-64.36988719,0\H,7,1.09701075,1,109.6249995
1,4,-57.75762729,0\\Version=IA32L-G03RevD.01\State=1-A\HF=-290.4071752
\MP2=-291.5249889\RMSD=8.646e-09\Thermal=0.\PG=C01 [X(C6H15N1)]\\@

30-Me⁺

1\1\GINC-GRETEL\SP\RMP2-FC\6-31+G(2d,p)\C7H18N1(1+)\CHRISTOPH\12-Nov-2
010\0\\#p MP2(FC)/6-31+g(2d,p) scf=tight\\n7mesp_1\\1,1\N\C,1,1.506749
02\H,2,1.09039439,1,109.43566751\C,1,1.53659262,2,110.93530152,3,64.76
945687,0\H,4,1.09563075,1,105.26691528,2,62.03853181,0\H,4,1.09563064,
1,105.27042377,2,176.08494347,0\C,1,1.50675043,2,109.48064901,4,-122.7
6492366,0\H,7,1.09252851,1,108.63914868,2,-61.86119617,0\H,7,1.0925843
6,1,108.84271287,2,178.41055757,0\H,7,1.09038899,1,109.4366948,2,57.99
43105,0\C,1,1.50856141,2,108.72537297,7,-118.65020504,0\H,11,1.0922829
3,1,108.92215594,2,179.56216615,0\H,11,1.09253697,1,108.97522371,2,59.
56489994,0\C,4,1.52995971,1,116.48068145,2,-60.93499312,0\H,14,1.09774
541,4,110.93720473,1,59.74311773,0\H,14,1.097742,4,110.93966567,1,-59.
77387128,0\C,14,1.54585408,4,109.83592938,1,179.98399819,0\H,17,1.0991
7043,14,109.32854805,4,58.21913344,0\H,17,1.09916991,14,109.32825311,4
,-58.21374934,0\H,2,1.09252845,1,108.63982922,7,61.86058396,0\C,17,1.5
351939,14,111.9881714,4,-179.9969749,0\H,21,1.09510514,17,110.46766227
,14,179.99940104,0\H,21,1.09702198,17,111.31934264,14,-60.32543102,0\H
,21,1.09702191,17,111.31952781,14,60.32417394,0\H,2,1.09258415,1,108.8
4258194,7,-178.41059799,0\H,11,1.09228484,1,108.92190221,2,-60.4320338
7,0\\Version=IA32L-G03RevD.01\State=1-A\HF=-329.8296075\MP2=-331.09767
84\RMSD=4.861e-09\Thermal=0.\PG=C01 [X(C7H18N1)]\\@

31

1\1\GINC-GRETEL\SP\RMP2-FC\6-31+G(2d,p)\C7H15N1\CHRISTOPH\15-Nov-2010\
0\\#p MP2(FC)/6-31+g(2d,p) scf=tight\\n19sp_5\\0,1\N\H,1,2.12590541\H,
1,2.10869081,2,92.42402491\C,1,1.46009318,3,138.7361513,2,103.88661717
,0\C,1,1.46055005,4,109.78288953,2,-98.38729593,0\C,1,1.46767991,4,112
.19642587,5,124.74360786,0\C,6,1.55432911,1,112.66394958,4,174.9271881
7,0\C,7,1.55871746,6,106.43283381,1,-149.27100939,0\C,8,1.55087093,7,1
05.65949352,6,1.82742131,0\C,9,1.53852253,8,104.37891286,7,22.9936858,
0\H,7,1.09755763,6,108.11770519,1,-30.44286231,0\H,7,1.09596335,6,112.
15033905,1,87.84290302,0\H,8,1.09691898,7,110.15646784,6,121.10354891,
0\H,8,1.09626246,7,112.24810485,6,-120.17327676,0\H,9,1.09642634,8,112
.92491035,7,145.95607901,0\H,9,1.09898674,8,110.14037308,7,-94.3925420
8,0\H,10,1.09998399,9,110.72499992,8,76.81383058,0\H,10,1.09622304,9,1
12.44615064,8,-162.17954839,0\H,4,1.0944262,1,110.44290083,5,173.47114
88,0\H,5,1.11022285,1,113.06610801,4,63.76001166,0\H,5,1.09675599,1,10
9.30914718,4,-56.4788822,0\H,4,1.11019196,1,113.16698369,5,-65.0229553
8,0\H,4,1.09675565,1,109.17931554,5,55.14911436,0\\Version=IA32L-G03Re
vD.01\State=1-A\HF=-328.2747088\MP2=-329.5282232\RMSD=2.008e-09\Therma
l=0.\PG=C01 [X(C7H15N1)]\\@

31-Me⁺

1\1\GINC-IBLIS\SP\RMP2-FC\6-31+G(2d,p)\C8H18N1(1+)\CHRISTOPH\03-Dec-20
10\0\\#p MP2(FC)/6-31+g(2d,p) scf=tight\\n19mesp_2_5\\1,1\N\H,1,2.1290
4622\H,1,2.07839538,2,115.46085462\H,1,2.12034746,3,173.44478178,2,133
.11897552,0\C,1,1.5089696,3,89.92941392,2,14.38203414,0\C,1,1.50675949
,5,108.61532871,2,58.7128565,0\H,5,1.09244907,1,108.81878357,6,178.409
90682,0\H,5,1.09057178,1,109.44019339,6,-61.40546552,0\C,1,1.50972065,
6,108.71967721,5,117.36686869,0\C,1,1.55194901,6,113.2860441,5,-121.44
070761,0\C,10,1.54939666,1,114.22951737,6,-66.42844884,0\C,11,1.542178
83,10,103.18533534,1,152.87941733,0\C,12,1.5387544,11,103.07874917,10,
-40.72536755,0\C,13,1.54765458,12,103.89757125,11,41.01734335,0\H,11,1
.09472602,10,112.45754493,1,-84.73851495,0\H,11,1.0977502,10,111.49282
993,1,36.37744805,0\H,12,1.09463438,11,112.02041276,10,-163.14682766,0
\H,12,1.09815483,11,110.08735828,10,77.02799641,0\H,13,1.09428323,12,1
13.24710221,11,162.75920617,0\H,13,1.09816077,12,109.91914739,11,-77.2

7562913,0\H,14,1.09471488,13,112.25355922,12,-149.94705757,0\H,14,1.09
559005,13,110.49255836,12,91.69505204,0\H,6,1.09015715,1,109.72703624,
5,178.98803917,0\H,6,1.09002968,1,109.55473195,5,58.00655142,0\H,9,1.0
9258376,1,108.72686012,6,-61.45854052,0\H,9,1.09235902,1,108.84361707,
6,178.83086147,0\H,9,1.09033796,1,109.32862634,6,58.61124143,0\\Versio
n=AM64L-G03RevD.01\State=1-A\HF=-367.6954495\MP2=-369.1012762\RMSD=2.3
69e-09\Thermal=0.\PG=C01 [X(C8H18N1)]\\@

32

1\1\GINC-GRETEL\SP\RMP2-FC\6-31+G(2d,p)\C7H17N1\CHRISTOPH\13-Nov-2010\
0\\#p MP2(FC)/6-31+g(2d,p) scf=tight\\n9sp_7\\0,1\N\C,1,1.45761027\H,2
,1.10987286,1,113.30316404\C,1,1.4650863,2,111.11617503,3,-62.86686674
,0\H,4,1.11168749,1,111.31239012,2,68.40847528,0\C,1,1.45806597,2,110.
67135156,4,125.73057853,0\H,6,1.11028877,1,112.99979046,2,-64.56909076
,0\H,6,1.09666006,1,109.40270012,2,55.83109721,0\H,2,1.09682582,1,109.
73605529,6,-176.28482933,0\H,4,1.09808559,1,107.11190345,2,-47.4021564
,0\H,6,1.09520377,1,110.45465072,2,174.22301863,0\C,4,1.54023323,1,113
.99437385,2,-168.00037873,0\H,12,1.0991503,4,109.84735622,1,-71.033038
11,0\H,12,1.10072221,4,107.25284034,1,174.59856241,0\C,12,1.54030133,4
,114.85908113,1,52.51258634,0\H,15,1.10092138,12,108.38651493,4,-176.3
6122842,0\H,15,1.09864406,12,109.17336784,4,-60.42424988,0\C,15,1.5375
5722,12,114.48949115,4,61.89873854,0\H,18,1.09883651,15,109.48619787,1
2,-62.62841932,0\H,18,1.10133683,15,109.44937186,12,53.8234995,0\C,18,
1.53596769,15,112.61909995,12,175.7985106,0\H,21,1.09735554,18,111.486
37715,15,179.55998507,0\H,21,1.09844613,18,111.14849221,15,-60.2129696
2,0\H,21,1.09812893,18,110.98804952,15,59.45252721,0\H,2,1.09703525,1,
109.61676818,6,-57.74189173,0\\Version=IA32L-G03RevD.01\State=1-A\HF=-
329.444879\MP2=-330.7168439\RMSD=7.460e-09\Thermal=0.\PG=C01 [X(C7H17N
1)]\\@

32-Me$^+$

1\1\GINC-MORITZ\SP\RMP2-FC\6-31+G(2d,p)\C8H20N1(1+)\CHRISTOPH\13-Nov-2
010\0\\#p MP2(FC)/6-31+g(2d,p) scf=tight\\n9mesp_1\\1,1\N\C,1,1.506711
94\H,2,1.09259238,1,108.64531015\C,1,1.53686262,2,110.93778883,3,-175.
33251725,0\H,4,1.09562133,1,105.23782735,2,62.12166563,0\H,4,1.0956184
3,1,105.23595065,2,176.1439481,0\C,1,1.50671748,2,109.48098816,4,-122.
76100348,0\H,7,1.09045708,1,109.47122063,2,58.07749416,0\H,7,1.0925931
4,1,108.646709,2,-61.76295404,0\C,1,1.50841196,2,108.73645323,7,-118.6
667416,0\H,10,1.09223373,1,108.90517882,2,179.63259776,0\H,10,1.092495
51,1,108.9790782,2,59.62681709,0\C,4,1.52968972,1,116.57184215,2,-60.8
6335994,0\H,13,1.09769864,4,110.93186009,1,59.81587939,0\H,13,1.097707
25,4,110.94105074,1,-59.70638452,0\C,13,1.54543946,4,109.7845735,1,-17
9.94719104,0\H,16,1.10016643,13,109.45559536,4,-58.22951167,0\H,16,1.1
0016399,13,109.45466479,4,58.26698001,0\H,2,1.09258943,1,108.84162994,
7,-178.36831974,0\C,16,1.5388521,13,112.34597846,4,-179.98086357,0\H,2
0,1.09979928,16,109.24333374,13,58.05283358,0\H,20,1.09980041,16,109.2
4344379,13,-58.04680498,0\H,2,1.09045884,1,109.46712134,7,-57.93106276
,0\H,10,1.09223364,1,108.90402132,2,-60.37734662,0\C,20,1.53512861,16,
112.46036619,13,-179.99682877,0\H,25,1.09554298,20,110.78053926,16,179
.99967502,0\H,25,1.09731986,20,111.26816874,16,-60.17757545,0\H,25,1.0
9731993,20,111.26830164,16,60.17685258,0\H,7,1.0925876,1,108.84034489,
2,178.51312996,0\\Version=IA32L-G03RevD.01\State=1-A\HF=-368.8691566\M
P2=-370.2897843\RMSD=7.217e-09\Thermal=0.\PG=C01 [X(C8H20N1)]\\@

33

1\1\GINC-PHOBOS\SP\RMP2-FC\6-31+G(2d,p)\C10H21N1\CHRISTOPH\19-Nov-2010
\0\\#p MP2(FC)/6-31+g(2d,p) scf=tight\\n26sp_7\\0,1\N\C,1,1.46126305\H
,2,1.09400118,1,111.01609734\H,2,1.09619221,1,108.96741865,3,117.96233

905,0\H,2,1.10977578,1,113.28099652,4,119.99988797,0\C,1,1.46124884,2,
109.53944735,3,-168.27095183,0\H,6,1.10977595,1,113.28071468,2,-69.683
48069,0\H,6,1.09401213,1,111.01243307,2,168.28252118,0\H,6,1.09619166,
1,108.96930982,2,50.31855071,0\C,1,1.47933015,6,112.90502559,2,126.763
6348,0\C,10,1.55304984,1,111.42991945,6,-179.0717413,0\C,11,1.55225952
,10,115.70814134,1,-54.82177497,0\C,12,1.54781025,11,115.80246058,10,-
104.4003005,0\C,13,2.63892002,12,100.6478803,11,71.43287232,0\C,14,1.5
4781452,13,100.64615015,12,0.00458852,0\C,15,1.5522607,14,115.80408663
,13,-71.44146448,0\H,10,1.1131158,1,109.09570639,6,-62.49471409,0\H,11
,1.09668808,10,108.34860121,1,68.58791275,0\H,11,1.0986997,10,107.2715
8563,1,-178.82840029,0\H,12,1.09542314,11,108.62923926,10,20.56602721,
0\H,12,1.09929937,11,108.9838685,10,134.98003512,0\H,13,1.1009494,12,1
08.27359092,11,-77.52520424,0\H,15,1.09542319,14,110.47939394,13,52.56
816286,0\H,15,1.09929837,14,106.97934115,13,166.86206784,0\H,16,1.0966
9826,15,109.52973466,14,-132.82440762,0\H,16,1.09869978,15,110.6377508
,14,-17.84352532,0\H,13,1.10083572,12,108.39643634,11,168.08724902,0\C
,14,1.54044333,13,31.06912383,12,-126.95110252,0\H,28,1.09923742,14,10
9.11510471,13,125.11521307,0\H,28,1.10025724,14,107.47598347,13,-121.5
0333368,0\H,14,1.10095014,13,137.87180059,12,-133.11159969,0\H,14,1.10
083527,13,92.85260654,12,109.32630183,0\\Version=AM64L-G03RevD.01\Stat
e=1-A\HF=-445.3722424\MP2=-447.0955171\RMSD=2.277e-09\Thermal=0.\PG=C0
1 [X(C10H21N1)]\\@

33-Me$^+$

1\1\GINC-CIPCLU03\SP\RMP2-FC\6-31+G(2d,p)\C11H24N1(1+)\C2175\27-Nov-20
10\0\\#p MP2(FC)/6-31+G(2d,p) scf=tight\\n26mesp_1\\1,1\N\C,1,1.509079
42\H,2,1.09260761,1,108.6856396\H,2,1.09237605,1,108.69322873,3,119.55
911544,0\H,2,1.08965347,1,110.11026681,4,120.43117747,0\C,1,1.50647334
,2,109.06265008,5,-63.50961319,0\H,6,1.09282178,1,108.40472705,2,-60.2
9090308,0\H,6,1.09057432,1,109.45010064,2,-179.75524613,0\H,6,1.090168
14,1,109.74845848,2,59.43442988,0\C,1,1.50910259,6,108.30448035,2,116.
09081276,0\H,10,1.0926037,1,108.42172683,6,-65.83501241,0\H,10,1.08949
866,1,109.77297246,6,53.83864802,0\H,10,1.0925665,1,108.99203418,6,174
.67591297,0\C,1,1.58059153,6,112.73202362,10,122.43382688,0\C,14,1.550
59587,1,111.46378472,6,55.99546406,0\C,15,1.54951643,14,114.26370175,1
,129.20972471,0\C,16,1.54358559,15,118.96209798,14,67.5717593,0\C,17,2
.62178946,16,115.63400202,15,-28.67149503,0\C,18,1.53939443,17,92.7628
5762,16,44.05596418,0\C,14,1.54391297,1,110.87608835,6,-72.14359202,0\
H,14,1.09444013,1,101.16197392,6,172.05439306,0\H,15,1.09619048,14,110
.88062047,1,9.45160228,0\H,15,1.09653616,14,109.54578993,1,-106.214333
94,0\H,16,1.10165596,15,108.18989522,14,-56.83232862,0\H,16,1.09792163
,15,106.58357328,14,-170.33712393,0\H,17,1.09733809,16,105.9161638,15,
174.801256,0\H,19,1.09781692,18,107.76707112,17,153.14058942,0\H,19,1.
09670215,18,109.90644203,17,38.90056689,0\H,20,1.096628,14,109.9272756
9,1,-65.97764056,0\H,20,1.0994044,14,109.4012279,1,51.55451767,0\H,17,
1.09953773,16,109.85757095,15,61.43108683,0\C,18,1.54737033,17,32.1432
072,16,-99.11144875,0\H,32,1.09716969,18,106.78521655,17,-121.20729499
,0\H,32,1.10083981,18,110.8099471,17,125.5139942,0\H,18,1.09985098,17,
140.20921405,16,-81.33532872,0\H,18,1.09856488,17,98.69013026,16,150.9
6626985,0\\Version=AM64L-G03RevD.01\State=1-A\HF=-484.7979621\MP2=-486
.6717693\RMSD=3.493e-09\Thermal=0.\PG=C01 [X(C11H24N1)]\\@

34

1\1\GINC-TOFU\SP\RMP2-FC\6-31+G(2d,p)\C9H21N1\CHRISTOPH\15-Feb-2012\0\
\#p MP2(FC)/6-31+g(2d,p) scf=tight\\n43sp_24\\0,1\C\H,1,1.09628594\C,1
,2.47213684,2,86.17648489\H,3,1.10002962,1,135.79575113,2,-162.1648808
2,0\C,1,2.48102399,3,60.6780827,4,-2.72916141,0\H,5,1.10064895,1,136.8
939486,3,-27.71247793,0\C,5,1.54807116,1,99.32904034,3,100.59774233,0\

H,7,1.0980255,5,110.4025221,1,151.47066238,0\H,7,1.09606617,5,111.5777
4136,1,-88.99240498,0\H,7,1.09775354,5,111.52125978,1,32.04867447,0\C,
5,1.5471587,1,95.09561416,3,-148.20982776,0\H,11,1.09960134,5,107.0468
6562,1,-157.83649105,0\H,11,1.10024081,5,110.05093936,1,-43.67817049,0
\C,3,1.54854432,1,103.90526426,5,130.49954914,0\H,14,1.100279,3,108.25
891761,1,142.23820902,0\H,14,1.10044833,3,109.15350612,1,27.37882999,0
\C,3,1.54786072,1,90.65171555,5,-118.80611797,0\H,17,1.09767101,3,111.
47647322,1,-48.02174438,0\H,17,1.09564313,3,112.14079263,1,73.00556403
,0\H,17,1.09747074,3,109.94197632,1,-166.92643756,0\C,11,1.5338441,5,1
14.50737875,1,80.69856727,0\H,21,1.09819928,11,110.94831041,5,56.86066
012,0\H,21,1.09749451,11,110.83433692,5,176.5879146,0\H,21,1.09536231,
11,111.34072087,5,-62.76237507,0\C,14,1.53326503,3,113.78903602,1,-95.
55367229,0\H,25,1.09893276,14,111.02119062,3,-62.08801413,0\H,25,1.095
0563,14,110.53962845,3,57.60247193,0\H,25,1.09733776,14,111.19485062,3
,178.11192713,0\H,1,1.09611908,3,141.15317156,17,-165.8554731,0\H,1,1.
10587277,3,102.5105806,17,-25.26495704,0\N,1,1.45564889,3,32.80131473,
17,-142.16526706,0\\Version=AM64L-G03RevD.01\State=1-A\HF=-407.5153882
\MP2=-409.1039115\RMSD=9.486e-09\Thermal=0.\PG=C01 [X(C9H21N1)]\\@

34-Me$^+$

1\1\GINC-AZAZEL\SP\RMP2-FC\6-31+G(2d,p)\C10H24N1(1+)\CHRISTOPH\16-Feb-
2012\0\\#p MP2(FC)/6-31+g(2d,p) scf=tight\\n43mesp_10\\1,1\C\H,1,1.093
35033\C,1,2.59933661,2,90.71630394\H,3,1.0929999,1,83.47653579,2,-122.
93529052,0\C,3,2.50848182,1,59.18228474,2,147.00808988,0\H,5,1.0895898
6,3,145.32455548,1,-23.28655199,0\C,1,1.54856868,5,90.01326683,3,-168.
24774258,0\H,7,1.09774055,1,109.67493377,5,-57.87751751,0\H,7,1.095830
57,1,111.6872394,5,60.47465687,0\C,1,1.53364775,7,110.84256983,5,100.7
7616547,0\H,10,1.09330366,1,113.60534144,7,164.59742837,0\H,10,1.09626
995,1,112.42276177,7,-70.80087923,0\H,10,1.09436317,1,107.78004309,7,4
6.57972984,0\C,3,1.54434599,1,92.98801666,10,95.69967126,0\H,14,1.0978
6199,3,109.77414762,1,106.33008913,0\H,14,1.0953294,3,110.99338002,1,-
11.66747966,0\C,3,1.53592097,1,146.57243294,10,-126.23667206,0\H,17,1.
09538854,3,113.17126738,1,-71.91505341,0\H,17,1.09297508,3,113.6451948
5,1,53.51952632,0\H,17,1.09424266,3,107.23552516,1,170.62055537,0\C,7,
1.54067819,1,111.49472945,10,-78.42765755,0\H,21,1.09481241,7,109.5926
255,1,179.62049849,0\H,21,1.09683004,7,111.31417447,1,-61.23632893,0\H
,21,1.0955619,7,112.46925866,1,60.33488931,0\C,14,1.54135733,3,112.124
53547,1,-132.04710322,0\H,25,1.09734292,14,111.47196143,3,57.03995483,
0\H,25,1.09478911,14,109.49957738,3,176.07394173,0\H,25,1.09479246,14,
112.48957344,3,-64.6979329,0\C,5,2.44223434,3,61.18434129,1,-69.897312
96,0\H,29,1.09064717,5,144.98565999,3,46.065785,0\H,29,1.09024122,5,92
.15372085,3,-84.44149364,0\H,29,1.08968647,5,86.81716435,3,166.7853131
8,0\H,5,1.0894427,3,92.91187284,1,-153.4117381,0\H,5,1.09162419,3,86.0
5710213,1,96.91537615,0\N,5,1.50665426,3,36.39083715,1,-34.2276418,0\\
Version=AM64L-G03RevD.01\State=1-A\HF=-446.9308545\MP2=-448.6771281\RM
SD=2.583e-09\Thermal=0.\PG=C01 [X(C10H24N1)]\\@

35

1\1\GINC-CIPCLU07\SP\RMP2-FC\6-31+G(2d,p)\C5H13N1\C2175\08-Nov-2010\0\
\#p MP2(FC)/6-31+G(2d,p) scf=tight\\n2sp_1\\0,1\N\C,1,1.45956952\H,2,1
.0945231,1,110.22160116\H,2,1.09452371,1,110.22131708,3,-118.10241881,
0\H,2,1.11057724,1,112.78149115,4,-120.94860845,0\C,1,1.46716251,2,111
.66015357,3,-58.56749414,0\H,6,1.09811885,1,107.0993337,2,-172.2401152
8,0\H,6,1.11136928,1,111.57182345,2,-56.08829261,0\C,1,1.46716093,2,11
1.65954838,6,-124.76945551,0\H,9,1.0981197,1,107.09962166,2,172.235249
46,0\H,9,1.11136822,1,111.57173165,2,56.08335542,0\C,9,1.53351387,1,11
3.50422578,2,-68.32372823,0\H,12,1.09620102,9,112.59705745,1,68.792080
54,0\H,12,1.09711561,9,109.83809468,1,-171.7363808,0\H,12,1.09635975,9

,110.20369345,1,-52.21791057,0\C,6,1.53351462,1,113.50483121,2,68.3189
1241,0\H,16,1.09711458,6,109.83800321,1,171.73134841,0\H,16,1.09620036
,6,112.5971954,1,-68.79710956,0\H,16,1.09636009,6,110.20366379,1,52.21
326854,0\\Version=AM64L-G03RevD.01\State=1-A\HF=-251.3684556\MP2=-252.
3341716\RMSD=3.454e-09\Thermal=0.\PG=C01 [X(C5H13N1)]\\@

35-Me$^+$

1\1\GINC-CIPCLU06\SP\RMP2-FC\6-31+G(2d,p)\C6H16N1(1+)\C2175\08-Nov-201
0\0\\#p MP2(FC)/6-31+G(2d,p) scf=tight\\n2mesp_1\\1,1\N\C,1,1.5326031\
H,2,1.09485985,1,105.15868731\H,2,1.09325342,1,105.99274832,3,114.0134
8817,0\C,1,1.50802419,2,110.22707872,4,172.74269529,0\H,5,1.09260667,1
,108.95377765,2,-54.98705272,0\H,5,1.09068162,1,109.58594185,2,65.5598
2021,0\H,5,1.0925158,1,108.68158443,2,-174.66538992,0\C,1,1.50801915,5
,108.03088798,2,117.68901365,0\H,9,1.09068353,1,109.58498631,5,-176.76
18341,0\H,9,1.0925163,1,108.68188948,5,-56.98678044,0\H,9,1.0926065,1,
108.95368489,5,62.69205255,0\C,1,1.53259709,9,110.22449042,5,-117.6887
5279,0\H,13,1.09485938,1,105.15798647,9,58.7439761,0\H,13,1.09325309,1
,105.99356342,9,172.75823974,0\C,13,1.52526144,1,115.56667464,9,-63.88
659018,0\H,16,1.09456205,13,112.83826565,1,-63.85433998,0\H,16,1.09442
244,13,113.01161953,1,60.72581169,0\H,16,1.0958394,13,107.23024222,1,1
78.4141223,0\C,2,1.52526465,1,115.56815076,9,178.33518774,0\H,20,1.095
83818,2,107.23020921,1,178.41914618,0\H,20,1.09456143,2,112.838156,1,-
63.84949674,0\H,20,1.09442033,2,113.01216186,1,60.73111643,0\\Version=
AM64L-G03RevD.01\State=1-A\HF=-290.7893408\MP2=-291.9076937\RMSD=2.068
e-09\Thermal=0.\PG=C01 [X(C6H16N1)]\\@

36

1\1\GINC-CIPCLU02\SP\RMP2-FC\6-31+G(2d,p)\C8H19N1\C2175\23-Nov-2010\0\
\#p MP2(FC)/6-31+G(2d,p) scf=tight\\n10sp_9\\0,1\N\C,1,1.45766246\H,2,
1.11000206,1,113.27086945\C,1,1.46503467,2,111.04347509,3,-62.91791857
,0\H,4,1.11171732,1,111.34920563,2,68.03038258,0\C,1,1.45800296,2,110.
59879103,4,125.76341016,0\H,6,1.11024446,1,113.00578651,2,-64.27611533
,0\H,6,1.09672809,1,109.36851784,2,56.0723384,0\H,2,1.0967978,1,109.74
305994,6,-176.3517157,0\H,4,1.09806488,1,107.05648623,2,-47.78673273,0
\H,6,1.09512876,1,110.48401344,2,174.4411874,0\C,4,1.53972231,1,114.01
899689,2,-168.29855179,0\H,12,1.10062124,4,107.26808957,1,175.14146302
,0\H,12,1.0991946,4,109.87337084,1,-70.42423737,0\C,12,1.54066051,4,11
4.83656094,1,53.01094302,0\H,15,1.09868191,12,109.14205132,4,-60.94401
643,0\H,15,1.10083531,12,108.3787055,4,-176.80684857,0\C,15,1.53757031
,12,114.46229235,4,61.3585892,0\H,18,1.09985952,15,109.52690667,12,-62
.8435766,0\H,18,1.10243803,15,109.57545418,12,53.63028815,0\C,18,1.537
9655,15,113.00863495,12,175.63451805,0\H,21,1.10042719,18,109.10509031
,15,57.502287,0\H,21,1.10072608,18,109.27206851,15,-58.1528104,0\H,2,1
.09701303,1,109.64812635,6,-57.7794019,0\C,21,1.53552943,18,113.152698
4,15,179.62400601,0\H,25,1.09728734,21,111.42911734,18,-179.93244625,0
\H,25,1.09834711,21,111.1222761,18,59.91967881,0\H,25,1.09824582,21,11
1.0972261,18,-59.78685451,0\\Version=AM64L-G03RevD.01\State=1-A\HF=-36
8.4839178\MP2=-369.9085577\RMSD=7.587e-09\Thermal=0.\PG=C01 [X(C8H19N1
)]\\@

36-Me$^+$

1\1\GINC-CIPCLU09\SP\RMP2-FC\6-31+G(2d,p)\C9H22N1(1+)\C2175\17-Nov-201
0\0\\#p MP2(FC)/6-31+G(2d,p) scf=tight\\n10mesp_1\\1,1\N\C,1,1.5066119
9\H,2,1.09254812,1,108.64719248\C,1,1.5372046,2,110.92211137,3,-175.41
463409,0\H,4,1.09561582,1,105.24392163,2,62.04448939,0\H,4,1.09561619,
1,105.2453604,2,176.08163069,0\C,1,1.50661098,2,109.48049473,4,-122.73
607825,0\H,7,1.09038637,1,109.43046029,2,58.03286993,0\H,7,1.09254929,
1,108.64711924,2,-61.83625988,0\C,1,1.50837158,7,108.73358569,2,118.66

524545,0\H,10,1.09227722,1,108.91709126,7,60.44412009,0\H,10,1.0925582
6,1,108.98697761,7,-59.55988734,0\C,4,1.52980804,1,116.49730106,7,60.9
6166862,0\H,13,1.09770006,4,110.90942724,1,59.73586668,0\H,13,1.097699
03,4,110.91018599,1,-59.74394594,0\C,13,1.54550615,4,109.81241984,1,17
9.99590393,0\H,16,1.10009576,13,109.40869569,4,-58.22715411,0\H,16,1.1
0009479,13,109.40889972,4,58.22368188,0\H,2,1.09258338,1,108.83694658,
7,-178.41609062,0\C,16,1.53854223,13,112.33506617,4,179.99826724,0\H,2
0,1.10078989,16,109.35730595,13,58.0717366,0\H,20,1.10078908,16,109.35
709183,13,-58.07480973,0\H,2,1.09038896,1,109.43011315,7,-58.01997713,
0\H,10,1.09227802,1,108.91693569,7,-179.5636648,0\C,20,1.53831072,16,1
12.81726568,13,179.99851427,0\H,25,1.09999593,20,109.22245267,16,-57.9
5995648,0\H,25,1.09999691,20,109.22257362,16,57.95780002,0\H,7,1.09258
356,1,108.837043,2,178.42919195,0\C,25,1.53522692,20,112.67529509,16,1
79.99891235,0\H,29,1.09586425,25,110.98371944,20,179.9990035,0\H,29,1.
09759186,25,111.2145474,20,-60.0790572,0\H,29,1.09759169,25,111.214455
25,20,60.07719056,0\\Version=AM64L-G03RevD.01\State=1-A\HF=-407.908476
\MP2=-409.4816306\RMSD=7.739e-09\Thermal=0.\PG=C01 [X(C9H22N1)]\\@

37

1\1\GINC-AZAZEL\SP\RMP2-FC\6-31+G(2d,p)\C9H21N1\CHRISTOPH\08-Dec-2010\
0\\#p MP2(FC)/6-31+g(2d,p) scf=tight\\n11sp_10\\0,1\N\C,1,1.45801525\H
,2,1.11021531,1,112.99992329\C,1,1.46501718,2,112.567831,3,60.4405202,
0\H,4,1.11173442,1,111.35089908,2,-56.44508986,0\C,1,1.45774179,2,110.
62511416,4,-124.84995807,0\H,6,1.09680737,1,109.74182098,2,-176.171196
27,0\H,6,1.09697491,1,109.63609141,2,-57.60073663,0\H,2,1.09670653,1,1
09.38061618,6,55.96146529,0\H,4,1.09810551,1,107.07418101,6,-47.616857
59,0\H,6,1.10999217,1,113.27740219,2,63.01820455,0\C,4,1.53986468,1,11
4.00498486,6,-168.14556283,0\H,12,1.09919957,4,109.87613657,1,-70.2851
8964,0\H,12,1.10065558,4,107.26934034,1,175.32635032,0\C,12,1.5404129,
4,114.84624902,1,53.15050246,0\H,15,1.09864386,12,109.1213095,4,-60.43
787993,0\H,15,1.10081281,12,108.39005837,4,-176.30575322,0\C,15,1.5379
4591,12,114.47275776,4,61.85832072,0\H,18,1.10228223,15,109.51314656,1
2,53.95024747,0\H,18,1.09975351,15,109.50085254,12,-62.49721887,0\C,18
,1.5377647,15,113.0171789,12,175.98758458,0\H,21,1.10144203,18,109.197
65008,15,57.90445477,0\H,21,1.10175768,18,109.40018957,15,-57.82011215
,0\H,2,1.09514914,1,110.47113338,6,174.32934463,0\C,21,1.53767743,18,1
13.45625517,15,179.99151707,0\H,25,1.1005778,21,109.20442822,18,-57.41
82035,0\H,25,1.10068247,21,109.21137544,18,58.22893364,0\C,25,1.535625
16,21,113.16644799,18,-179.58676022,0\H,28,1.09731653,25,111.41922182,
21,-179.85463655,0\H,28,1.09818866,25,111.10075562,21,60.00568814,0\H,
28,1.09823817,25,111.12935594,21,-59.71260621,0\\Version=AM64L-G03RevD
.01\State=1-A\HF=-407.5229374\MP2=-409.1001157\RMSD=7.024e-09\Thermal=
0.\PG=C01 [X(C9H21N1)]\\@

37-Me^{+}

1\1\GINC-PHOBOS\SP\RMP2-FC\6-31+G(2d,p)\C10H24N1(1+)\CHRISTOPH\07-Dec-
2010\0\\#p MP2(FC)/6-31+g(2d,p) scf=tight\\n11mesp_1\\1,1\N\C,1,1.5065
6577\H,2,1.09258471,1,108.65015563\C,1,1.53722154,2,110.92288745,3,-17
5.42163719,0\H,4,1.0956255,1,105.23463746,2,62.05048052,0\H,4,1.095623
98,1,105.23036298,2,176.07414359,0\C,1,1.50656212,2,109.48365674,4,-12
2.73213578,0\H,7,1.09043433,1,109.45068148,2,58.07220932,0\H,7,1.09258
459,1,108.65156346,2,-61.78729072,0\C,1,1.50826585,7,108.73929731,2,11
8.67772402,0\H,10,1.09225101,1,108.90960639,7,60.45656072,0\H,10,1.092
53941,1,108.98694458,7,-59.54958983,0\C,4,1.52967628,1,116.54350647,7,
60.95809892,0\H,13,1.09769626,4,110.92831944,1,59.78110363,0\H,13,1.09
770104,4,110.92786693,1,-59.72738564,0\C,13,1.54562959,4,109.78619074,
1,-179.97290898,0\H,16,1.10009119,13,109.40295196,4,-58.22305929,0\H,1
6,1.10008975,13,109.40295332,4,58.21564651,0\H,2,1.09258773,1,108.8386

4293,7,-178.42311431,0\C,16,1.53859276,13,112.34478774,4,179.99617845,
0\H,20,1.10075052,16,109.31566224,13,58.04920428,0\H,20,1.10074971,16,
109.31519219,13,-58.05178096,0\H,2,1.09042873,1,109.4512065,7,-58.0120
7882,0\H,10,1.0922499,1,108.90942384,7,-179.5553238,0\C,20,1.53799606,
16,112.80651639,13,179.99895579,0\H,25,1.10102062,20,109.33754421,16,-
57.98466163,0\H,25,1.10102128,20,109.33757996,16,57.97869341,0\H,7,1.0
9258858,1,108.83840897,2,178.48130586,0\C,25,1.53808341,20,113.0453717
2,16,179.99691926,0\H,29,1.10020795,25,109.22013311,20,-57.91759504,0\
H,29,1.10020722,25,109.22027216,20,57.91634148,0\C,29,1.53525643,25,11
2.82748027,20,179.99939464,0\H,32,1.09614774,29,111.0993926,25,179.999
48381,0\H,32,1.09771744,29,111.19182832,25,-60.02088663,0\H,32,1.09771
701,29,111.19189601,25,60.01979903,0\\Version=AM64L-G03RevD.01\State=1
-A\HF=-446.947692\MP2=-448.673375\RMSD=8.366e-09\Thermal=0.\PG=C01 [X(
C10H24N1)]\\@

38

1\1\GINC-PHOENIX\SP\RMP2-FC\6-31+G(2d,p)\C18H33N1\CHRISTOPH\19-Nov-201
0\0\\#p MP2(FC)/6-31+g(2d,p) scf=tight\\n24sp_11\\0,1\N\H,1,2.06002886
\H,1,2.05985278,2,119.83885356\H,1,2.05954058,3,119.5974164,2,-169.717
85303,0\C,1,3.86268233,4,157.7600109,3,154.88025247,0\C,5,1.54042552,1
,21.95825968,4,-98.85196379,0\C,1,1.46302785,6,35.00681487,5,-6.486622
03,0\C,7,1.55301064,1,115.22652227,6,-127.06310147,0\C,8,1.54110625,7,
111.96260393,1,-177.56562049,0\C,9,1.53873269,8,111.55020752,7,-55.546
17303,0\H,5,1.10186254,1,97.76618158,7,-66.81035884,0\H,5,1.0989749,1,
131.97004625,7,172.71548678,0\H,6,1.09811027,5,110.76824082,1,117.7350
468,0\H,6,1.1013676,5,109.03836306,1,-125.17168498,0\H,8,1.10102299,7,
109.40161944,1,61.66672924,0\H,8,1.09804351,7,109.4693691,1,-54.981746
34,0\H,9,1.10190617,8,109.23062246,7,65.0761918,0\H,9,1.09904422,8,110
.02516352,7,-178.43147892,0\H,10,1.10159824,9,109.2160297,8,-65.087724
68,0\H,10,1.09888585,9,110.36861809,8,177.95292772,0\C,1,3.86252577,7,
129.86782968,6,47.46209671,0\C,21,1.54046259,1,21.90272808,7,-122.2742
1093,0\C,1,1.46285733,7,119.05478164,6,70.65417624,0\C,23,1.55326943,1
,115.22611835,7,103.28168311,0\C,24,1.54101201,23,111.89515494,1,-177.
54179312,0\C,25,1.53857504,24,111.51725912,23,-55.69945992,0\H,21,1.10
18764,1,97.92215389,23,-66.81256054,0\H,21,1.09894856,1,131.91078861,2
3,172.59831639,0\H,22,1.10128414,21,108.95849171,1,-125.10911415,0\H,2
2,1.09806448,21,110.77712006,1,117.82088377,0\H,24,1.10118791,23,109.4
0664092,1,61.69403807,0\H,24,1.09796365,23,109.50570514,1,-54.96455355
,0\H,25,1.09900518,24,110.05123175,23,-178.58713832,0\H,25,1.10189673,
24,109.20394632,23,64.89352141,0\H,26,1.10160699,25,109.19877123,24,-6
5.03607826,0\H,26,1.09889945,25,110.35753249,24,178.04191662,0\C,1,3.8
6186917,23,129.27185697,22,46.48850039,0\C,37,1.54056196,1,21.9295603,
23,-121.44026423,0\C,1,1.46276232,23,118.95805124,22,69.91275163,0\C,3
9,1.55289805,1,115.30902921,23,104.48979366,0\C,40,1.54118436,39,111.8
4400766,1,-177.39327936,0\C,41,1.53870477,40,111.56245389,39,-55.72674
011,0\H,37,1.10185419,1,97.72869123,39,-67.10247569,0\H,37,1.09896379,
1,131.96493438,39,172.46186176,0\H,38,1.10141689,37,109.04592459,1,-12
5.28917623,0\H,38,1.09815081,37,110.78692409,1,117.60615031,0\H,40,1.0
9807951,39,109.50261718,1,-54.84236709,0\H,40,1.10096911,39,109.420046
62,1,61.88147863,0\H,41,1.09905224,40,110.04394441,39,-178.64277194,0\
H,41,1.10188828,40,109.1889563,39,64.86837901,0\H,42,1.09890106,41,110
.34422633,40,177.96087016,0\H,42,1.10160125,41,109.21889401,40,-65.107
66833,0\\Version=AM64L-G03RevD.01\State=1-A\HF=-755.3739641\MP2=-758.2
878413\RMSD=1.707e-09\Thermal=0.\PG=C01 [X(C18H33N1)]\\@

38-Me$^+$

1\1\GINC-CIPCLU02\SP\RMP2-FC\6-31+G(2d,p)\C19H36N1(1+)\C2175\27-Nov-20
10\0\\#p MP2(FC)/6-31+G(2d,p) scf=tight\\n24mesp_2\\1,1\N\H,1,2.111075

52\C,1,1.50585179,2,109.15126882\H,3,1.08738825,1,110.16735519,2,22.49
469773,0\H,3,1.08719528,1,110.02762758,2,-97.71430938,0\H,3,1.09156051
,1,108.53330259,2,142.18826678,0\H,1,2.11775429,3,137.71455291,5,52.67
24194,0\C,1,3.95619204,3,124.72693597,5,168.8791251,0\C,8,1.54628473,1
,23.99899871,3,172.69571981,0\C,9,1.54040581,8,109.71129131,1,-10.0018
2791,0\C,10,1.54658353,9,109.35078206,8,-60.72006303,0\C,11,1.54818645
,10,109.0485791,9,60.63196493,0\C,12,1.53593421,11,111.88186751,10,-57
.80361858,0\H,8,1.10065226,1,94.45333707,3,-57.48624822,0\H,8,1.096586
74,1,132.26721888,3,-174.67953743,0\H,9,1.09784417,8,108.59195283,1,-1
30.08503725,0\H,9,1.09519779,8,108.96649648,1,113.00375554,0\H,10,1.09
34002,9,108.5999703,8,55.14463828,0\H,11,1.09412194,10,112.43837943,9,
-179.1172736,0\H,11,1.09823157,10,111.03274498,9,-58.68982537,0\H,12,1
.10018283,11,109.19168989,10,63.97543996,0\H,12,1.09654628,11,108.5030
318,10,179.76255527,0\H,13,1.10030218,12,109.46638556,11,-66.4636331,0
\H,13,1.09664819,12,110.12266295,11,176.57979031,0\C,1,3.94463818,3,99
.14096562,10,-137.01538216,0\C,25,1.54486213,1,23.38105131,3,-37.29059
166,0\C,26,1.54415187,25,109.84142119,1,9.08162872,0\C,27,1.54500511,2
6,109.11989693,25,61.04949647,0\C,28,1.54838242,27,109.61514112,26,-59
.18894089,0\C,29,1.53512504,28,112.56459669,27,56.24452621,0\H,25,1.09
663804,1,131.64777382,3,-50.08732692,0\H,25,1.10048133,1,94.97705257,3
,-167.58494966,0\H,26,1.09331129,25,109.48959859,1,-113.93312718,0\H,2
6,1.09787843,25,108.01071435,1,129.40844854,0\H,28,1.09895065,27,110.5
7219243,26,61.04891127,0\H,28,1.09377413,27,112.25324909,26,-178.80672
023,0\H,29,1.09663812,28,108.39452147,27,179.04917072,0\H,29,1.1004638
5,28,108.89669281,27,-65.50192712,0\H,30,1.09665963,29,110.03836823,28
,-175.84928044,0\H,30,1.10022274,29,109.60455755,28,67.13393304,0\C,1,
3.98107654,3,92.39727852,10,121.91163693,0\C,41,1.5467713,1,24.4310962
8,3,35.67096345,0\C,42,1.54545274,41,109.48133186,1,8.70790519,0\C,43,
1.54397261,42,110.18746506,41,58.53790253,0\C,44,1.54607228,43,109.741
99313,42,-59.28549829,0\C,45,1.5352196,44,111.5727004,43,57.58716321,0
\H,41,1.09666571,1,132.57048981,3,25.23269824,0\H,41,1.10054242,1,94.6
2344222,3,-92.2287402,0\H,42,1.09406149,41,108.00882169,1,-113.0770658
8,0\H,42,1.09727558,41,109.05320345,1,130.50827857,0\H,44,1.09724554,4
3,110.58107801,42,60.31680824,0\H,44,1.09377081,43,111.68776812,42,179
.88336403,0\H,45,1.09668702,44,108.53600206,43,179.9116235,0\H,45,1.10
065179,44,109.43754413,43,-64.14434469,0\H,46,1.09667682,45,110.251592
18,44,-177.05308295,0\H,46,1.1003581,45,109.46833609,44,65.88600297,0\
\Version=AM64L-G03RevD.01\State=1-A\HF=-794.7814706\MP2=-797.8632911\R
MSD=2.419e-09\Thermal=0.\PG=C01 [X(C19H36N1)]\\@

39

1\1\GINC-CIPCLU02\SP\RMP2-FC\6-31+G(2d,p)\C7H17N1\C2175\22-Nov-2010\0\
\#p MP2(FC)/6-31+G(2d,p) scf=tight\\n31sp_1\\0,1\N\C,1,1.47964553\H,2,
1.11114974,1,110.48446657\C,1,1.463189,2,111.46697199,3,58.42831626,0\
H,4,1.09465302,1,110.43415986,2,69.05991605,0\C,1,1.48131553,4,112.873
78433,2,131.39861197,0\H,6,1.09472313,1,105.71693326,4,-179.75871561,0
\C,6,1.54008377,1,111.10038432,4,66.23354746,0\H,8,1.09732867,6,112.81
789224,1,-75.38693239,0\H,8,1.09740421,6,109.74099009,1,165.41040704,0
\H,8,1.09594376,6,110.15602816,1,45.93043254,0\C,6,1.54599681,1,115.15
762574,4,-61.08607348,0\H,12,1.09772342,6,110.39069039,1,-175.33531918
,0\H,12,1.09765802,6,111.47448243,1,65.19779454,0\H,12,1.09746905,6,11
1.68264825,1,-55.56545133,0\C,2,1.54012667,1,112.43626923,4,178.803076
09,0\H,16,1.09546616,2,113.12336168,1,-70.3186227,0\H,16,1.09774414,2,
110.44781161,1,51.57148497,0\H,16,1.09645204,2,109.39922077,1,170.4964
3858,0\C,2,1.5423763,1,110.2844915,4,-59.94403865,0\H,20,1.09757949,2,
110.02603973,1,-55.59164423,0\H,20,1.09664134,2,109.85513837,1,-174.99
206905,0\H,20,1.0947945,2,112.76381757,1,65.53000986,0\H,4,1.09335425,
1,109.95664091,2,-173.31205721,0\H,4,1.10792867,1,113.65708078,2,-52.2

4762415,0\\Version=AM64L-G03RevD.01\State=1-A\HF=-329.4389687\MP2=-330
.720248\RMSD=9.781e-09\Thermal=0.\PG=C01 [X(C7H17N1)]\\@

39-Me⁺

1\1\GINC-IBLIS\SP\RMP2-FC\6-31+G(2d,p)\C8H20N1(1+)\CHRISTOPH\03-Dec-20
10\0\\#p MP2(FC)/6-31+g(2d,p) scf=tight\\n31mesp_1\\1,1\N\C,1,1.507286
34\H,2,1.08972716,1,109.22241853\H,2,1.09119369,1,109.65875487,3,120.3
8407407,0\H,2,1.08995672,1,109.10884495,3,-118.94512556,0\C,1,1.507290
36,2,107.96770395,3,48.50550022,0\H,6,1.08972906,1,109.22418534,2,48.5
0067286,0\H,6,1.09119304,1,109.65770957,2,168.88616291,0\H,6,1.0899574
2,1,109.1088686,2,-70.44423879,0\C,1,1.57039517,2,108.84324565,6,-119.
79873224,0\H,10,1.09283117,1,102.98887687,2,-59.5834958,0\C,1,1.570396
81,2,110.35093554,6,118.83917675,0\H,12,1.09282977,1,102.9874562,2,-17
7.86451077,0\C,12,1.53666304,1,112.98852268,2,-61.77588734,0\H,14,1.09
318816,12,113.93293545,1,-54.65141944,0\H,14,1.09509807,12,113.0012306
5,1,70.90393112,0\H,14,1.09558074,12,106.77428943,1,-172.02877143,0\C,
12,1.53348891,1,112.59593275,2,64.28858654,0\H,18,1.09567747,12,112.38
248352,1,-63.70469278,0\H,18,1.09537946,12,107.41734887,1,178.982232,0
\H,18,1.09344153,12,113.81623531,1,61.00217065,0\C,10,1.53666487,1,112
.99252486,2,56.50523426,0\H,22,1.09558182,10,106.77361689,1,-172.06135
593,0\H,22,1.09509786,10,113.00459773,1,70.87131693,0\H,22,1.09318265,
10,113.93120589,1,-54.68580155,0\C,10,1.53348313,1,112.59255282,2,-177
.43038673,0\H,26,1.09344084,10,113.8175417,1,60.97704634,0\H,26,1.0953
79,10,107.41770547,1,178.95834763,0\H,26,1.09567784,10,112.38022256,1,
-63.72814158,0\\Version=AM64L-G03RevD.01\State=1-A\HF=-368.8571977\MP2
=-370.2937975\RMSD=8.839e-09\Thermal=0.\PG=C01 [X(C8H20N1)]\\@

40

1\1\GINC-CIPCLU02\SP\RMP2-FC\6-31+G(2d,p)\C9H19N1\C2175\27-Nov-2010\0\
\#p MP2(FC)/6-31+G(2d,p) scf=tight\\n25sp_7\\0,1\N\C,1,1.46159201\H,2,
1.1098937,1,113.06947447\H,2,1.09637959,1,108.93471143,3,119.83466549,
0\H,2,1.09375955,1,111.05012716,4,118.22075483,0\C,1,1.46215639,2,108.
86776979,5,170.69716399,0\H,6,1.09340885,1,110.91823554,2,-173.9856450
7,0\H,6,1.11010197,1,112.85813106,2,64.55604295,0\H,6,1.09649096,1,109
.23510876,2,-55.49295202,0\C,1,1.47653045,2,112.77159599,6,124.3861254
2,0\C,10,1.55171186,1,112.47548126,2,178.13076715,0\C,11,1.5481137,10,
118.47889625,1,-45.93398756,0\C,12,1.5437888,11,117.67013468,10,-38.11
055022,0\C,13,1.54142458,12,116.00428719,11,-38.38113136,0\C,14,1.5413
8277,13,113.39459341,12,83.51993279,0\C,15,1.53898493,14,116.19019998,
13,-71.00050376,0\H,10,1.11440533,1,108.84953715,2,-64.88969462,0\H,11
,1.0987889,10,109.54732538,1,78.3329651,0\H,11,1.09872591,10,106.10225
684,1,-168.88276354,0\H,12,1.09854409,11,109.53427407,10,85.12709562,0
\H,12,1.10043773,11,106.69434529,10,-161.01886069,0\H,13,1.09973583,12
,107.69133393,11,-161.20992087,0\H,13,1.09732869,12,108.77592047,11,83
.62384995,0\H,14,1.09954501,13,108.60823409,12,-156.78272928,0\H,14,1.
10185683,13,110.62886583,12,-40.86708667,0\H,15,1.10039513,14,108.8923
3973,13,167.69907634,0\H,15,1.09973937,14,108.06293419,13,52.73808987,
0\H,16,1.09856183,15,108.58805314,14,-177.96131919,0\H,16,1.10014883,1
5,108.78534104,14,-63.95014584,0\\Version=AM64L-G03RevD.01\State=1-A\H
F=-406.3431779\MP2=-407.9102383\RMSD=1.868e-09\Thermal=0.\PG=C01 [X(C9
H19N1)]\\@

40-Me⁺

1\1\GINC-CIPCLU10\SP\RMP2-FC\6-31+G(2d,p)\C10H22N1(1+)\C2175\27-Nov-20
10\0\\#p MP2(FC)/6-31+G(2d,p) scf=tight\\n25mesp_1\\1,1\N\C,1,1.509561
03\H,2,1.09258406,1,108.37412823\H,2,1.08934219,1,109.86352483,3,119.7
351945,0\H,2,1.09259794,1,108.92953683,3,-119.43936178,0\C,1,1.5096080
1,2,106.80593423,4,172.66338906,0\H,6,1.09262641,1,108.60857397,2,-59.

33394534,0\H,6,1.08937687,1,110.21313345,2,-179.18312698,0\H,6,1.09239
092,1,108.75233799,2,60.17774439,0\C,1,1.50609001,2,108.6924784,6,-117
.41694482,0\H,10,1.09275586,1,108.4901031,2,55.3526275,0\H,10,1.090241
1,1,109.44611273,2,175.11811201,0\H,10,1.09077825,1,109.41932057,2,-64
.26486008,0\C,1,1.57713794,10,112.02738254,2,122.34598609,0\C,14,1.544
50916,1,111.91134026,10,53.98919328,0\C,15,1.54995673,14,111.73632144,
1,144.08192987,0\C,16,1.53791106,15,116.14724593,14,73.75123823,0\C,17
,1.54050271,16,116.07025604,15,-52.74825645,0\C,18,1.54380256,17,113.1
4669907,16,67.145866,0\C,14,1.54618463,1,110.84471409,10,-75.32860168,
0\H,14,1.09678034,1,101.54878327,10,169.30852234,0\H,15,1.09575555,14,
110.91874737,1,24.90960479,0\H,15,1.09778788,14,110.3038021,1,-92.5554
1435,0\H,16,1.10095765,15,108.34509537,14,-50.36096412,0\H,16,1.097202
92,15,107.63621705,14,-164.57356069,0\H,17,1.10012753,16,108.91354573,
15,69.58714455,0\H,17,1.09769085,16,107.84808768,15,-175.69289233,0\H,
18,1.101341,17,110.81906008,16,-57.96507629,0\H,18,1.09689545,17,107.8
8318805,16,-173.26613103,0\H,19,1.09679753,18,109.71022142,17,152.7834
4546,0\H,19,1.09823118,18,108.74658135,17,37.59823669,0\H,20,1.0963149
,14,108.62978111,1,-73.00817416,0\H,20,1.09877321,14,110.39745093,1,43
.81730741,0\\Version=AM64L-G03RevD.01\State=1-A\HF=-445.7676269\MP2=-4
47.4848322\RMSD=3.344e-09\Thermal=0.\PG=C01 [X(C10H22N1)]\\@

41

1\1\GINC-GRETEL\SP\RMP2-FC\6-31+G(2d,p)\C9H17N1\CHRISTOPH\12-Nov-2010\
0\\#p MP2(FC)/6-31+g(2d,p) scf=tight\\n17sp_1\\0,1\N\H,1,2.13004702\C,
1,1.46304471,2,96.73026474\H,3,1.10955819,1,113.11375559,2,35.57922432
,0\C,1,1.45868803,3,110.29300006,4,-62.1276528,0\C,5,1.55547655,1,117.
51418915,3,-61.0701928,0\C,6,1.5553237,5,88.22459404,1,-142.32310432,0
\C,7,1.55422671,6,88.20328141,5,20.2416152,0\H,6,1.09585657,5,118.2714
8947,1,96.15502323,0\H,6,1.09695868,5,110.01524728,1,-30.46542354,0\H,
7,1.09635019,6,111.80563196,5,-92.73721907,0\H,7,1.0950675,6,117.51720
271,5,140.64872865,0\H,8,1.09547314,7,118.30846948,6,-141.59328758,0\H
,8,1.09548024,7,111.08408814,6,90.72038846,0\H,3,1.09553062,1,109.7490
1503,5,177.11720031,0\H,3,1.09552662,1,109.75077386,5,58.62988672,0\H,
5,1.11086606,1,111.26472757,3,65.95965655,0\C,1,1.45868427,5,112.01550
79,6,175.67952697,0\C,18,1.55548069,1,117.51383727,5,-175.65975144,0\C
,19,1.55532312,18,88.22492991,1,142.32124681,0\C,20,1.5542335,19,88.20
452936,18,-20.23641601,0\H,19,1.09585787,18,118.27129809,1,-96.1582914
2,0\H,19,1.09695676,18,110.0141609,1,30.46161028,0\H,20,1.09506849,19,
117.51737334,18,-140.64324517,0\H,20,1.09634867,19,111.80540866,18,92.
74283081,0\H,21,1.09547709,20,111.08363425,19,-90.72923234,0\H,21,1.09
547544,20,118.30812353,19,141.58662174,0\\Version=IA32L-G03RevD.01\Sta
te=1-A\HF=-405.1259211\MP2=-406.6659271\RMSD=2.116e-09\Thermal=0.\PG=C
01 [X(C9H17N1)]\\@

41-Me$^+$

1\1\GINC-MORITZ\SP\RMP2-FC\6-31+G(2d,p)\C10H20N1(1+)\CHRISTOPH\12-Nov-
2010\0\\#p MP2(FC)/6-31+g(2d,p) scf=tight\\n17mesp_4\\1,1\N\H,1,2.1180
7462\H,1,2.0899571,2,172.56215713\H,1,2.11809825,3,90.60471237,2,-98.6
8521659,0\H,1,2.08995766,3,89.21569274,4,-172.55236851,0\C,1,1.5040725
7,3,91.85899915,5,143.26914258,0\C,1,1.50410728,6,108.28483415,4,-60.8
697798,0\C,1,1.53325879,6,112.73463649,7,-119.1904894,0\C,8,1.54799397
,1,120.99042396,6,56.67558406,0\C,9,1.56018285,8,86.42690963,1,-148.40
290894,0\C,8,1.54774614,1,121.15102112,6,-54.65990933,0\H,9,1.09545605
,8,113.24286174,1,-37.49775306,0\H,9,1.09361138,8,117.29008697,1,91.23
214004,0\H,10,1.09200608,9,116.63459312,8,140.67694577,0\H,10,1.093849
44,9,112.07053065,8,-92.46622048,0\H,11,1.09516475,8,113.31374511,1,37
.38807456,0\H,11,1.09370877,8,117.23554943,1,-91.34802687,0\H,6,1.0907
7301,1,109.49823655,7,179.64631539,0\H,6,1.09094402,1,109.52126997,7,5

8.71677073,0\H,7,1.09093625,1,109.52206427,6,58.70504936,0\H,7,1.09076
37,1,109.50013042,6,179.63761146,0\C,1,1.53324718,6,107.81589188,7,122
.26773761,0\C,22,1.54803442,1,120.98107765,6,-62.86631655,0\C,23,1.560
15732,22,86.43223515,1,-148.37972883,0\C,22,1.54780724,1,121.14136922,
6,-174.17593112,0\H,23,1.09545527,22,113.22832266,1,-37.48092433,0\H,2
3,1.09360207,22,117.29753085,1,91.24122776,0\H,24,1.09386229,23,112.06
495434,22,-92.45774312,0\H,24,1.09200268,23,116.63994283,22,140.682206
43,0\H,25,1.09516795,22,113.30245454,1,37.36824289,0\H,25,1.09369651,2
2,117.24063803,1,-91.36413631,0\\Version=IA32L-G03RevD.01\State=1-A\HF
=-444.5480921\MP2=-446.2416471\RMSD=3.778e-09\Thermal=0.\PG=C01 [X(C10
H20N1)]\\@

42

1\1\GINC-NODE22\SP\RMP2-FC\6-31+G(2d,p)\C8H17N1\ZIP07\24-Nov-2010\0\\#
p MP2(FC)/6-31+G(2d,p) scf=tight\\n22sp_3\\0,1\N\H,1,2.12576656\H,1,2.
09324418,2,122.20710709\C,1,1.46128226,3,89.63379912,2,-97.94214638,0\
C,1,1.4612802,4,109.40718752,3,-22.55950321,0\H,4,1.09400997,1,111.045
4161,5,-168.81524669,0\H,5,1.09401223,1,111.04479199,4,168.81594614,0\
H,5,1.10994343,1,113.21021785,4,-69.23718155,0\H,4,1.10994396,1,113.20
982526,5,69.23775143,0\H,4,1.09616998,1,109.03307076,5,-50.79994193,0\
C,1,3.05947909,5,100.5998903,4,-176.09552665,0\C,11,1.53992048,1,54.72
603847,5,-75.03610077,0\C,1,1.47934533,5,112.55643605,4,125.9260334,0\
C,13,1.54825963,1,111.84980561,5,178.04415358,0\C,14,1.53992139,13,111
.98077737,1,-63.97380997,0\C,15,1.54048736,14,111.06675615,13,-57.2711
7679,0\H,11,1.09864148,1,63.20876975,5,68.88244367,0\H,11,1.09907408,1
,150.26352967,5,-8.53514456,0\H,12,1.10046889,11,109.34201443,1,-148.1
7336775,0\H,12,1.09645477,11,110.84514913,1,95.60471469,0\H,14,1.09645
243,13,111.49729482,1,60.874125,0\H,14,1.10046978,13,107.12635102,1,17
6.15087208,0\H,15,1.09864147,14,109.18803148,13,63.42721434,0\H,15,1.0
990738,14,109.87536025,13,-179.32320526,0\H,16,1.10199396,15,108.97692
99,14,-67.32628944,0\H,16,1.09895857,15,110.28732472,14,176.01037006,0
\\Version=AM64L-G03RevD.01\State=1-A\HF=-367.3165798\MP2=-368.7276415\
RMSD=1.808e-09\Thermal=0.\PG=C01 [X(C8H17N1)]\\@

42-Me$^+$

1\1\GINC-PHOBOS\SP\RMP2-FC\6-31+G(2d,p)\C9H20N1(1+)\CHRISTOPH\01-Dec-2
010\0\\#p MP2(FC)/6-31+g(2d,p) scf=tight\\n22mesp_1_2\\1,1\N\C,1,1.509
08401\H,2,1.0926036,1,108.99526619\H,2,1.09239142,1,108.49209827,3,119
.5785187,0\H,2,1.08936073,1,109.79216257,4,119.57491356,0\C,1,1.506283
13,2,108.52687627,5,53.2963472,0\H,6,1.09285126,1,108.36277816,2,54.88
69423,0\H,6,1.09042623,1,109.49774397,2,174.40530183,0\H,6,1.09092732,
1,109.40893652,2,-64.63898152,0\C,1,1.5091183,6,108.66232789,2,-116.34
242308,0\H,10,1.09254617,1,108.90366839,6,-179.59383953,0\H,10,1.08916
457,1,109.8622838,6,-58.8479913,0\H,10,1.0924092,1,108.57622873,6,60.7
7773877,0\C,1,3.94347422,6,103.03438257,2,140.70311993,0\C,14,1.547185
86,1,22.96560878,6,43.62293611,0\C,15,1.53962453,14,109.80182231,1,-5.
83401738,0\C,16,1.53947116,15,110.70049014,14,-58.63781582,0\C,17,1.54
795592,16,109.77139798,15,58.14845521,0\C,18,1.53543618,17,111.9927066
6,16,-56.27592924,0\H,14,1.09992374,1,96.51989629,6,169.16450714,0\H,1
4,1.09613296,1,131.2912897,6,50.23529822,0\H,15,1.0986424,14,108.56772
647,1,-126.09581979,0\H,15,1.09545146,14,108.72957985,1,116.91891739,0
\H,16,1.09843974,15,109.07668412,14,61.05398622,0\H,17,1.09498026,16,1
11.73393647,15,178.51109621,0\H,17,1.09899903,16,110.16327111,15,-61.6
5850945,0\H,18,1.09998373,17,109.06749721,16,65.45515913,0\H,18,1.0961
5341,17,108.38135763,16,-178.88167119,0\H,19,1.10009104,18,109.6669214
8,17,-66.39050469,0\H,19,1.09636617,18,109.99613437,17,176.59094527,0\
\Version=AM64L-G03RevD.01\State=1-A\HF=-406.7417889\MP2=-408.3028063\R
MSD=3.230e-09\Thermal=0.\PG=C01 [X(C9H20N1)]\\@

43

1\1\GINC-CIPCLU02\SP\RMP2-FC\6-31+G(2d,p)\C7H17N1\C2175\09-Nov-2010\0\
\#p MP2(FC)/6-31+G(2d,p) scf=tight\\n5sp_7\\0,1\N\C,1,1.46737593\H,2,1
.09885684,1,107.24250746\C,1,1.45927181,2,111.61106702,3,169.66878812,
0\H,4,1.09478914,1,110.28277965,2,176.35108436,0\H,4,1.09478756,1,110.
28222822,2,58.08137169,0\C,1,1.46737394,4,111.6117183,2,125.55995012,0
\H,7,1.09885576,1,107.24206379,4,-169.67419777,0\H,7,1.11114616,1,111.
38150307,4,-53.42432755,0\C,7,1.53890007,1,114.04237648,4,70.84858845,
0\H,10,1.09900363,7,110.45298866,1,-65.45366323,0\H,10,1.09974207,7,10
7.3246854,1,179.83044701,0\C,10,1.53468771,7,113.3093399,1,58.26952273
,0\H,13,1.09717165,10,111.16439088,7,-179.87654532,0\H,13,1.09541177,1
0,110.77661684,7,-59.07876944,0\H,13,1.09841123,10,110.89686093,7,60.1
8191484,0\H,2,1.11114452,1,111.38111795,4,53.41921683,0\H,4,1.11019671
,1,112.64188309,7,62.7764783,0\C,2,1.53890283,1,114.04235538,4,-70.853
35732,0\H,19,1.09974257,2,107.32462331,1,-179.82958562,0\H,19,1.099003
94,2,110.45306335,1,65.45464437,0\C,19,1.53468815,2,113.30947762,1,-58
.26895432,0\H,22,1.09717074,19,111.16438564,2,179.86945294,0\H,22,1.09
841211,19,110.89691908,2,-60.18903953,0\H,22,1.09540983,19,110.7762408
6,2,59.07148551,0\\Version=AM64L-G03RevD.01\State=1-A\HF=-329.44485\MP
2=-330.7180729\RMSD=5.503e-09\Thermal=0.\PG=C01 [X(C7H17N1)]\\@

43-Me$^+$

1\1\GINC-CIPCLU03\SP\RMP2-FC\6-31+G(2d,p)\C8H20N1(1+)\C2175\09-Nov-201
0\0\\#p MP2(FC)/6-31+G(2d,p) scf=tight\\n5mesp_1\\1,1\N\C,1,1.5075733\
H,2,1.09043547,1,109.51445831\H,2,1.09245151,1,108.68352181,3,-119.781
48897,0\C,1,1.53310376,2,110.18430782,3,-65.54312665,0\H,5,1.0937197,1
,105.98402529,2,-172.46340721,0\H,5,1.09577846,1,105.22937488,2,-58.54
334867,0\C,1,1.50757986,2,107.96427197,5,-117.65657211,0\H,8,1.0924500
9,1,108.6836512,2,57.01829115,0\H,8,1.09267771,1,108.97557853,2,-62.71
643079,0\H,8,1.09043577,1,109.51480573,2,176.79963074,0\C,1,1.53312883
,2,107.93995896,8,-119.08863711,0\H,12,1.09577635,1,105.22741166,2,59.
20976367,0\H,12,1.09372209,1,105.98666242,2,-54.70997677,0\C,5,1.53059
584,1,116.33546797,2,64.15880079,0\H,15,1.09705508,5,110.65147895,1,60
.10757712,0\H,15,1.09686238,5,110.85368517,1,-59.04206889,0\C,15,1.540
80778,5,109.54616825,1,-179.50480925,0\H,18,1.09648138,15,111.4764801,
5,60.50824365,0\H,18,1.09437028,15,109.95156539,5,179.9925301,0\H,18,1
.09660118,15,111.45084309,5,-60.53794981,0\H,2,1.0926797,1,108.9757092
5,8,-62.71607943,0\C,12,1.53059962,1,116.33669429,2,-178.08628803,0\H,
23,1.09686738,12,110.85978127,1,-58.91412517,0\H,23,1.09704835,12,110.
64682103,1,60.23732603,0\C,23,1.54081116,12,109.54578808,1,-179.378649
5,0\H,26,1.09647683,23,111.4749999,12,60.55603649,0\H,26,1.09437002,23
,109.95090215,12,-179.9593214,0\H,26,1.09660254,23,111.45246867,12,-60
.49096598,0\\Version=AM64L-G03RevD.01\State=1-A\HF=-368.8691244\MP2=-3
70.2937937\RMSD=6.439e-09\Thermal=0.\PG=C01 [X(C8H20N1)]\\@

44

see reference 4

44-Me$^+$

see reference 4

44-BH$^+$

1\1\GINC-YANG\SP\RMP2-FC\6-31+G(2d,p)\C19H23N2(1+)\CHRISTOPH\10-Jan-20
11\0\\#p MP2(FC)/6-31+g(2d,p) scf=tight\\yin2bhsp_3\\1,1\C\H,1,1.09453
794\C,1,4.34625981,2,103.41876622\C,3,1.39702077,1,58.50964293,2,5.836
24219,0\C,4,1.3967051,3,119.92546754,1,0.63121126,0\C,5,1.40784293,4,1
21.25091636,3,-0.18731548,0\C,6,1.4077718,5,118.11240597,4,-0.62092761

,0\C,7,1.39709823,6,120.65424819,5,1.06027278,0\H,3,1.08675923,1,178.6
3517992,6,171.44631974,0\H,4,1.08678254,3,120.35679719,1,-179.02062803
,0\H,5,1.08900171,4,119.0240321,3,-179.22635825,0\H,7,1.08470299,6,121
.21028563,5,-177.6356175,0\H,8,1.08688699,7,119.41574447,6,-179.915194
63,0\C,1,4.34588823,6,117.6945628,5,119.7758707,0\C,14,1.39678668,1,58
.40345009,6,-118.19975628,0\C,15,1.39688667,14,119.94861844,1,-0.92513
47,0\C,16,1.4077334,15,121.25128512,14,0.35980692,0\C,17,1.40835727,16
,118.08514179,15,0.17285275,0\C,18,1.39699858,17,120.6727549,16,-0.660
83217,0\H,14,1.08673133,1,178.45129478,17,-171.85285186,0\H,15,1.08679
16,14,120.34611096,1,178.8820357,0\H,16,1.08883478,15,119.05775642,14,
179.44638771,0\H,18,1.08482447,17,121.28352194,16,178.69086279,0\H,19,
1.08689034,18,119.41521822,17,179.97977488,0\C,1,3.92597993,17,99.1045
4398,16,90.901819,0\C,25,1.56132843,1,21.44421352,17,-51.35947455,0\N,
26,1.52049162,25,109.17766641,1,-1.14072893,0\C,27,1.52124808,26,107.9
0931693,25,-62.32489774,0\C,28,1.56212485,27,109.16593981,26,54.343157
76,0\H,25,1.09484215,1,117.03829291,17,25.48530635,0\H,25,1.09484114,1
,122.3175552,17,-111.01177443,0\H,26,1.0947126,25,112.43246821,1,-118.
99563419,0\H,26,1.09164668,25,112.88216052,1,117.97245668,0\H,28,1.091
58742,27,106.79485336,26,176.57528623,0\H,28,1.09403503,27,107.0071425
6,26,-67.64186718,0\H,29,1.09489579,28,110.53844815,27,-113.61016856,0
\H,29,1.09478166,28,109.8442412,27,127.73914396,0\C,27,1.51553464,26,1
08.23109356,25,54.3257207,0\H,38,1.09150183,27,106.65721682,26,59.1583
1862,0\H,38,1.09183687,27,107.02134844,26,176.15673124,0\C,38,1.564332
46,27,109.01462042,26,-62.12661707,0\H,41,1.094819,38,109.7983358,27,1
27.72346687,0\H,41,1.09486719,38,110.42781709,27,-113.68559952,0\N,41,
1.46312683,38,111.25688614,27,6.96458201,0\\Version=AM64L-G03RevD.01\S
tate=1-A\HF=-841.6254789\MP2=-844.7244352\RMSD=3.959e-09\Thermal=0.\PG
=C01 [X(C19H23N2)]\\@

44-TT$^+$

1\1\GINC-PHOBOS\SP\RMP2-FC\6-31+G(2d,p)\C25H27N2(1+)\CHRISTOPH\27-Oct-
2011\0\\#p MP2(FC)/6-31+g(2d,p) scf=tight\\yin2ttsp_2\\1,1\C\C,1,4.293
57218\C,2,1.39905428,1,59.98400791\C,3,1.39315503,2,120.30286412,1,-0.
80856794,0\C,4,1.41940232,3,120.90488666,2,0.47071015,0\C,5,1.42003305
,4,117.71357909,3,0.55118263,0\C,6,1.39119412,5,121.09998433,4,-1.2283
1949,0\H,2,1.08723381,1,179.35542376,5,174.94053106,0\H,3,1.08695047,2
,120.1878754,1,178.42682384,0\H,4,1.08576482,3,119.07288755,2,176.8803
2752,0\H,6,1.08621163,5,119.4206207,4,179.96337482,0\H,7,1.08700368,6,
119.73955578,5,-179.25623266,0\N,1,5.36601346,5,94.71945128,4,120.4299
5471,0\C,13,1.46514809,1,70.48775854,5,-23.56272384,0\C,14,1.57853256,
13,109.6643256,1,-6.243012,0\C,15,2.40160775,14,86.13793415,13,39.4219
694,0\C,13,1.46520186,1,70.51077995,5,96.03380526,0\H,14,1.09616708,13
,108.47516718,1,115.05229064,0\H,15,1.09731208,14,111.6406322,13,132.7
6504469,0\H,16,1.0975965,15,144.06832696,14,-144.67244429,0\H,17,1.095
82225,13,108.47891044,1,113.73136329,0\C,1,4.29724308,5,117.4922842,4,
-135.93351137,0\C,22,1.40115837,1,60.51765681,5,-153.09900178,0\C,23,1
.39211463,22,120.21344575,1,0.87421571,0\C,24,1.41831593,23,121.040889
45,22,1.14708031,0\C,25,1.42018918,24,117.62769535,23,-1.30912915,0\C,
26,1.39225834,25,121.07707674,24,0.47766209,0\H,22,1.08719806,1,178.42
388348,5,95.73778235,0\H,23,1.08697699,22,120.1631464,1,-179.04598393,
0\H,24,1.08508678,23,119.37449074,22,-179.93811336,0\H,26,1.08463133,2
5,119.37244448,24,-176.3308189,0\H,27,1.08691034,26,119.5799914,25,-17
8.64887219,0\C,1,4.29647093,5,120.34003558,4,25.3581602,0\C,33,1.39937
056,1,59.92205885,5,-145.58016117,0\C,34,1.39271871,33,120.23873504,1,
-0.57140254,0\C,35,1.42005512,34,120.94479901,33,0.27450908,0\C,36,1.4
2149305,35,117.68148806,34,0.53214287,0\C,37,1.39056888,36,121.1003646
7,35,-0.99108765,0\H,33,1.08721015,1,179.38852126,5,-55.57488866,0\H,3
4,1.0869466,33,120.15033711,1,178.38005262,0\H,35,1.08634851,34,119.42

684573,33,176.42188038,0\H,37,1.08477622,36,118.96114298,35,179.762612
72,0\H,38,1.08712773,37,119.80591619,36,-179.50071008,0\H,14,1.0963569
6,13,109.41264489,1,-127.55721087,0\H,15,1.09703115,14,110.18980749,13
,-106.93852537,0\H,16,1.09616198,15,86.16383758,14,99.51346909,0\H,17,
1.09622978,13,109.4761633,1,-128.71347387,0\C,15,2.39478708,14,92.6471
6365,13,-20.36411519,0\C,13,1.46664847,1,69.36662383,5,-143.62137491,0
\H,48,1.09636237,15,143.41069415,14,109.49549895,0\H,48,1.09693846,15,
94.02360997,14,-122.45291285,0\H,49,1.09641777,13,108.2200833,1,114.57
163261,0\H,49,1.09606199,13,109.47661829,1,-128.14720802,0\N,48,1.4744
3275,15,35.82944508,14,120.14648184,0\\Version=AM64L-G03RevD.01\State=
1-A\HF=-1071.1540257\MP2=-1075.0937539\RMSD=7.043e-09\Thermal=0.\PG=C0
1 [X(C25H27N2)]\\@

45

1\1\GINC-CIPCLU02\SP\RMP2-FC\6-31+G(2d,p)\C6H15N1\C2175\08-Nov-2010\0\
\#p MP2(FC)/6-31+G(2d,p) scf=tight\\n3sp_1\\0,1\N\C,1,1.46927792\H,2,1
.10944742,1,111.32614166\H,2,1.09636555,1,107.91152093,3,-116.04287952
,0\C,1,1.46929926,2,111.79339068,4,-35.01172083,0\H,5,1.096326,1,107.9
3976161,2,-161.2007367,0\H,5,1.10943826,1,111.30247655,2,-45.12998806,
0\C,1,1.46921594,2,111.83746792,5,-126.25743331,0\H,8,1.10944541,1,111
.301574,2,80.62179007,0\H,8,1.09635972,1,107.94925271,2,-35.47121073,0
\C,8,1.53350694,1,112.81760289,2,-155.00541545,0\H,11,1.09601609,8,110
.12978678,1,58.4870406,0\H,11,1.09757659,8,110.3470582,1,178.52022986,
0\H,11,1.09686941,8,112.15688198,1,-61.65451923,0\C,5,1.53345899,1,112
.80914165,8,-154.467989,0\H,15,1.09761073,5,110.36321049,1,178.5783328
8,0\H,15,1.09690167,5,112.16024149,1,-61.58491406,0\H,15,1.0960195,5,1
10.12267971,1,58.55107117,0\C,2,1.53344701,1,112.87723511,8,79.2282734
6,0\H,19,1.09763971,2,110.35025444,1,178.20826616,0\H,19,1.09684696,2,
112.19135704,1,-61.97338689,0\H,19,1.09602912,2,110.13049494,1,58.1929
4739,0\\Version=AM64L-G03RevD.01\State=1-A\HF=-290.4069768\MP2=-291.52
62482\RMSD=2.774e-09\Thermal=0.\PG=C01 [X(C6H15N1)]\\@

45-Me$^+$

1\1\GINC-CIPCLU07\SP\RMP2-FC\6-31+G(2d,p)\C7H18N1(1+)\C2175\08-Nov-201
0\0\\#p MP2(FC)/6-31+G(2d,p) scf=tight\\n3mesp_1\\1,1\N\C,1,1.52888731
\H,2,1.09303749,1,105.76503403\H,2,1.09303131,1,105.76873122,3,-113.72
902218,0\C,1,1.53413228,2,111.74412731,4,63.48044442,0\H,5,1.09307895,
1,105.97173183,2,68.20736903,0\H,5,1.09483642,1,105.2085007,2,-177.850
35709,0\C,1,1.50653845,2,107.93105642,5,-120.34117584,0\H,8,1.09057932
,1,109.31210961,2,59.73086344,0\H,8,1.09283285,1,108.97836246,2,179.98
685505,0\H,8,1.09058108,1,109.31153009,2,-59.75788558,0\C,1,1.53412655
,8,109.40757823,2,121.78345731,0\H,12,1.09483468,1,105.20892836,8,58.3
5287291,0\H,12,1.09308072,1,105.96975116,8,172.29789043,0\C,12,1.52675
935,1,116.18021815,8,-64.45698972,0\H,15,1.09438026,12,113.25637985,1,
61.26502941,0\H,15,1.09607274,12,107.00122691,1,178.84239814,0\H,15,1.
09462639,12,112.95308423,1,-63.56883188,0\C,2,1.52594811,1,115.5404684
2,8,-179.99804356,0\H,19,1.09476433,2,112.93129785,1,-62.14829544,0\H,
19,1.09587954,2,107.33896571,1,-179.98391428,0\H,19,1.09476222,2,112.9
303518,1,62.1815208,0\C,5,1.52677112,1,116.184007,8,64.42787689,0\H,23
,1.09437686,5,113.25439939,1,-61.27483079,0\H,23,1.0946255,5,112.95466
588,1,63.55999795,0\H,23,1.0960716,5,107.00210464,1,-178.85262584,0\\V
ersion=AM64L-G03RevD.01\State=1-A\HF=-329.8278092\MP2=-331.1023051\RMS
D=3.853e-09\Thermal=0.\PG=C01 [X(C7H18N1)]\\@

45-BH$^+$

1\1\GINC-CIPCLU02\SP\RMP2-FC\6-31+G(2d,p)\C19H26N1(1+)\C2175\14-Jan-20
11\0\\#p MP2(FC)/6-31+G(2d,p) scf=tight\\yin15bhsp_4\\1,1\C\H,1,1.0924
1816\C,1,4.35346643,2,102.83508279\C,3,1.39674993,1,57.78688033,2,-15.

59114818,0\C,4,1.39667574,3,120.03338822,1,1.71246847,0\C,5,1.40763899
,4,121.41047729,3,-0.6494256,0\C,6,1.40801754,5,117.82415476,4,-0.0411
4851,0\C,3,1.3971925,1,61.60305996,6,29.7556901,0\H,3,1.08663283,1,177
.84761752,6,178.91531066,0\H,4,1.08679368,3,120.36522388,1,-178.531183
99,0\H,5,1.08902493,4,118.9789546,3,-179.52272495,0\H,7,1.08596894,6,1
21.73078221,5,-179.31822076,0\H,8,1.08695479,3,120.15501719,1,178.6278
9311,0\C,1,4.34314313,6,113.9735498,5,96.76978012,0\C,14,1.39903755,1,
60.33150771,6,41.52906751,0\C,15,1.39655012,14,120.3294927,1,0.0337446
2,0\C,16,1.40597177,15,120.57470153,14,1.01345143,0\C,17,1.40700001,16
,118.49708728,15,-2.64473517,0\C,18,1.39755168,17,120.90149226,16,2.47
791277,0\H,14,1.08682701,1,179.31463141,17,-173.59887656,0\H,15,1.0869
1966,14,120.09082503,1,-178.98841182,0\H,16,1.0857037,15,118.96580373,
14,-177.62375557,0\H,18,1.08876454,17,120.0097088,16,-176.27401207,0\H
,19,1.08687627,18,119.73640599,17,-179.76537456,0\N,1,1.60260694,17,11
3.26477246,16,-94.79175152,0\C,25,1.53415269,1,109.42602769,17,170.122
69469,0\H,26,1.09264952,25,105.65444566,1,-177.39773193,0\H,26,1.09147
808,25,105.94905846,1,-63.07409063,0\C,25,1.54208858,1,113.17123581,17
,51.28322876,0\H,29,1.08869197,25,105.83467742,1,39.11785775,0\H,29,1.
0935552,25,105.55739221,1,152.61508758,0\C,25,1.52756979,1,107.7903587
3,17,-70.74738162,0\H,32,1.09306743,25,106.46825273,1,-49.88123659,0\H
,32,1.09077424,25,106.05723926,1,64.3997126,0\C,29,1.53114057,25,118.3
0459081,1,-84.87306932,0\H,35,1.09693608,29,106.2529747,25,176.5357947
8,0\H,35,1.09351217,29,112.91446894,25,-66.50941673,0\H,35,1.09087992,
29,113.43033539,25,58.53991742,0\C,32,1.53020325,25,115.5025937,1,-172
.09462523,0\H,39,1.09427618,32,113.30975754,25,-53.91616474,0\H,39,1.0
9420432,32,112.70293146,25,69.91568998,0\H,39,1.09602634,32,107.404085
47,25,-172.19282136,0\C,26,1.52733683,25,116.31993072,1,60.26861312,0\
H,43,1.09293733,26,112.94611809,25,-62.72094033,0\H,43,1.09454091,26,1
13.09694415,25,62.22215509,0\H,43,1.09628964,26,106.91352146,25,179.81
752278,0\\Version=AM64L-G03RevD.01\State=1-A\HF=-788.9286628\MP2=-791.
8993385\RMSD=2.935e-09\Thermal=0.\PG=C01 [X(C19H26N1)]\\@

45-TT$^+$

1\1\GINC-PHOENIX\SP\RMP2-FC\6-31+G(2d,p)\C25H30N1(1+)\CHRISTOPH\08-Nov
-2011\0\\#p MP2(FC)/6-31+g(2d,p) scf=tight\\yin15ttsp_17_2\\1,1\C\C,1,
4.26249662\C,2,1.40242408,1,60.29385593\C,3,1.39016752,2,119.9946518,1
,0.78197867,0\C,4,1.42183967,3,120.38047852,2,-1.493367,0\C,5,1.421827
84,4,118.76286678,3,0.81694714,0\C,6,1.39027446,5,120.33423779,4,0.614
15112,0\H,2,1.08716984,1,179.91555768,5,-151.76936185,0\H,3,1.08642871
,2,120.10375567,1,-178.79167264,0\H,4,1.08608843,3,120.03631466,2,-178
.05041902,0\H,6,1.08547418,5,119.12118746,4,177.05784888,0\H,7,1.08658
69,6,119.87624532,5,178.33865941,0\C,1,4.26365829,5,119.78587282,4,-33
.51259315,0\C,13,1.40227593,1,60.25375022,5,145.58426736,0\C,14,1.3901
9658,13,120.03039718,1,0.73854927,0\C,15,1.42186737,14,120.39616075,13
,-1.38172265,0\C,16,1.42234093,15,118.66905638,14,0.61373115,0\C,17,1.
39022851,16,120.43229343,15,0.80475052,0\H,13,1.08716316,1,179.9655795
,5,57.00186293,0\H,14,1.086425,13,120.08091535,1,-178.81320148,0\H,15,
1.0858978,14,119.97928407,13,-177.98400214,0\H,17,1.08578099,16,119.29
236171,15,177.27974047,0\H,18,1.08642326,17,119.9120131,16,178.1790267
9,0\C,1,4.26279409,5,119.91949578,4,145.11521725,0\C,24,1.40225163,1,6
0.21660995,5,-33.53233303,0\C,25,1.39025111,24,119.96728572,1,0.699132
15,0\C,26,1.42219902,25,120.44681221,24,-1.3412325,0\C,27,1.42204956,2
6,118.67069214,25,0.67012051,0\C,28,1.3897147,27,120.38263474,26,0.654
90021,0\H,24,1.08716304,1,179.96480262,27,143.34113423,0\H,25,1.086430
37,24,120.13419095,1,-178.79333408,0\H,26,1.08595398,25,119.99399783,2
4,-177.89209686,0\H,28,1.08542813,27,119.47127456,26,176.85593604,0\H,
29,1.08648559,28,119.88267167,27,178.19358237,0\N,6,3.78519445,5,93.66
672845,4,129.51287302,0\C,35,1.47309897,6,85.13853327,5,166.71127191,0

\H,36,1.0991127,35,107.09677612,6,39.19161649,0\H,36,1.10651067,35,111
.94925264,6,155.19922333,0\C,36,1.53458874,35,113.93073598,6,-80.98900
606,0\H,39,1.09672807,36,109.6998832,35,173.51563966,0\H,39,1.09570245
,36,112.39960965,35,-67.4309495,0\H,39,1.09743151,36,111.16373574,35,5
3.61545473,0\C,35,1.47369578,6,83.17824986,5,-80.93478672,0\H,43,1.099
53402,35,106.7694804,6,-24.51374925,0\H,43,1.09820828,35,107.82256659,
6,89.23541751,0\C,35,1.47266129,6,147.65458703,5,43.68773687,0\H,46,1.
10718434,35,111.73794305,6,174.42254262,0\H,46,1.096654,35,107.1751473
1,6,58.63863918,0\C,43,1.54178693,35,117.5626923,6,-147.28489909,0\H,4
9,1.09614081,43,111.85393374,35,60.16895563,0\H,49,1.09706544,43,109.8
153095,35,179.53959107,0\H,49,1.09667668,43,111.94847143,35,-61.097717
25,0\C,46,1.53295861,35,114.20409568,6,-61.16140947,0\H,53,1.09606776,
46,112.58931632,35,-70.8436276,0\H,53,1.09794955,46,111.02392655,35,50
.32735699,0\H,53,1.09636978,46,109.61563029,35,169.91382286,0\\Version
=AM64L-G03RevD.01\State=1-A\HF=-1018.488198\MP2=-1022.2694284\RMSD=2.6
01e-09\Thermal=0.\PG=C01 [X(C25H30N1)]\\@

46

1\1\GINC-CIPCLU07\SP\RMP2-FC\6-31+G(2d,p)\C9H21N1\C2175\05-Dec-2010\0\
\#p MP2(FC)/6-31+G(2d,p) scf=tight\\n8sp_39\\0,1\N\C,1,1.46698545\H,2,
1.0979318,1,107.14075728\C,1,1.46701595,2,111.05665772,3,-46.84016476,
0\H,4,1.09793258,1,107.14313438,2,46.71339338,0\C,1,1.45963622,2,111.7
0815339,4,-125.43771109,0\H,6,1.09471096,1,110.24460594,2,-176.5789173
3,0\H,6,1.09471066,1,110.24518949,2,-58.36821861,0\H,2,1.11124528,1,11
1.37097188,6,-56.42849005,0\C,2,1.54034644,1,114.19161592,6,67.2289191
7,0\H,10,1.09904997,2,110.00362027,1,-69.9601905,0\H,10,1.1008102,2,10
7.19111378,1,175.62102404,0\C,10,1.54026434,2,114.8013868,1,53.6504038
7,0\H,13,1.09966371,10,108.36198678,2,-175.66739445,0\H,13,1.0978487,1
0,109.01290874,2,-59.76788022,0\C,13,1.5359794,10,114.11199479,2,62.53
074262,0\H,16,1.09898065,13,111.3232951,10,55.68723566,0\H,16,1.096951
45,13,111.44951741,10,-64.83070377,0\H,16,1.09723267,13,110.94112307,1
0,175.58183182,0\H,4,1.1112308,1,111.36527653,6,56.30505396,0\H,6,1.11
022497,1,112.69372206,2,62.52752853,0\C,4,1.54033338,1,114.18539769,6,
-67.35328891,0\H,22,1.0990389,4,109.99701345,1,69.76446137,0\H,22,1.10
081437,4,107.2001024,1,-175.81355807,0\C,22,1.54026555,4,114.78952104,
1,-53.84159825,0\H,25,1.09782592,22,109.00233311,4,59.49958051,0\H,25,
1.09965896,22,108.37237551,4,175.41013654,0\C,25,1.53602609,22,114.100
74008,4,-62.80467308,0\H,28,1.09697624,25,111.47094694,22,64.71952471,
0\H,28,1.09896713,25,111.31117046,22,-55.79127376,0\H,28,1.09723227,25
,110.94406054,22,-175.68168929,0\\Version=AM64L-G03RevD.01\State=1-A\H
F=-407.5199073\MP2=-409.1020816\RMSD=4.353e-09\Thermal=0.\PG=C01 [X(C9
H21N1)]\\@

46-Me$^+$

1\1\GINC-PHOENIX\SP\RMP2-FC\6-31+G(2d,p)\C10H24N1(1+)\CHRISTOPH\02-Dec
-2010\0\\#p MP2(FC)/6-31+g(2d,p) scf=tight\\n8mesp_1\\1,1\N\C,1,1.5070
6888\H,2,1.09033904,1,109.48547153\C,1,1.53287465,2,108.00666843,3,57.
60072986,0\H,4,1.09363056,1,105.77044851,2,-55.52367808,0\H,4,1.095910
06,1,105.19581786,2,58.36889063,0\C,1,1.5328682,2,110.19745269,4,-123.
10141314,0\H,7,1.09590865,1,105.19505994,2,-59.4109348,0\H,7,1.0936326
6,1,105.77016631,2,-173.30391659,0\C,1,1.50707103,2,107.97264702,4,119
.1489892,0\H,10,1.09034045,1,109.48582331,2,176.74758837,0\H,10,1.0924
5806,1,108.69365634,2,56.92447455,0\C,4,1.53080073,1,116.46324732,2,-1
78.80233706,0\H,13,1.09816682,4,110.80233067,1,60.32254734,0\H,13,1.09
779813,4,111.04967108,1,-58.9333135,0\C,13,1.54484939,4,109.95141578,1
,-179.3076611,0\H,16,1.09922626,13,109.34241119,4,58.22346217,0\H,16,1
.0993558,13,109.3504516,4,-58.16169065,0\H,2,1.09271517,1,108.9533555,
10,-62.84604711,0\C,16,1.53518766,13,112.07693113,4,-179.96891363,0\H,

20,1.09514244,16,110.52281815,13,179.9139966,0\H,20,1.09709752,16,111.
33354658,13,-60.39470255,0\H,20,1.09714951,16,111.33095456,13,60.20868
196,0\H,2,1.09245843,1,108.69371056,10,56.92609072,0\H,10,1.09271422,1
,108.95364037,2,-62.84788251,0\C,7,1.53080177,1,116.46229459,2,63.4161
7918,0\H,26,1.09779571,7,111.04667416,1,-59.0459009,0\H,26,1.09816532,
7,110.80457749,1,60.21000594,0\C,26,1.54484756,7,109.95240569,1,-179.4
1776142,0\H,29,1.09922726,26,109.34241325,7,58.1928969,0\H,29,1.099355
72,26,109.35053581,7,-58.19234556,0\C,29,1.53518913,26,112.07687184,7,
-180.,0\H,32,1.09709788,29,111.33365825,26,-60.38284399,0\H,32,1.09714
934,29,111.3306381,26,60.22047776,0\H,32,1.09514352,29,110.52305262,26
,179.92588229,0\\Version=AM64L-G03RevD.01\State=1-A\HF=-446.9486678\MP
2=-448.6788298\RMSD=5.255e-09\Thermal=0.\PG=C01 [X(C10H24N1)]\\@

47

1\1\GINC-CIPCLU07\SP\RMP2-FC\6-31+G(2d,p)\C9H21N1\C2175\11-Nov-2010\0\
\#p MP2(FC)/6-31+G(2d,p) scf=tight\\n6sp_4\\0,1\N\C,1,1.47034487\H,2,1
.10963669,1,110.82030625\C,1,1.47027669,2,111.33470244,3,-45.40414057,
0\H,4,1.1097003,1,110.78978878,2,79.58970109,0\H,4,1.09750731,1,107.95
904556,2,-36.30198653,0\C,1,1.47027958,4,111.314885,2,-124.85147766,0\
H,7,1.0975233,1,107.96312256,4,-36.64704711,0\H,7,1.10977789,1,110.817
79722,4,79.2483099,0\C,7,1.53923746,1,114.09474279,4,-156.48021859,0\H
,10,1.09949407,7,110.18886427,1,-59.75565211,0\H,10,1.10021125,7,107.5
171828,1,-174.76268176,0\C,10,1.53470408,7,113.52870973,1,63.53785224,
0\H,13,1.09883772,10,110.98099208,7,58.88577664,0\H,13,1.09552364,10,1
10.70230736,7,-60.78720381,0\H,13,1.09735631,10,111.1762534,7,178.8133
8896,0\H,2,1.09760601,1,107.97131115,4,-161.29373591,0\C,2,1.53920167,
1,114.04829235,4,78.84527751,0\H,18,1.09956564,2,110.20350798,1,-60.40
20589,0\H,18,1.1002008,2,107.53420279,1,-175.38464768,0\C,18,1.5347175
2,2,113.52650714,1,62.87643516,0\H,21,1.09734235,18,111.16925593,2,179
.37329497,0\H,21,1.09877037,18,111.03369589,2,59.42271755,0\H,21,1.095
43052,18,110.64922312,2,-60.22829332,0\C,4,1.53940843,1,114.10249765,7
,79.0414365,0\H,25,1.09939771,4,110.22300071,1,-59.73785977,0\H,25,1.1
001808,4,107.5228108,1,-174.73767922,0\C,25,1.53473663,4,113.50843885,
1,63.55312626,0\H,28,1.09876877,25,111.01104903,4,59.38109388,0\H,28,1
.09734453,25,111.17567392,4,179.32290371,0\H,28,1.09543709,25,110.6435
3783,4,-60.26273578,0\\Version=AM64L-G03RevD.01\State=1-A\HF=-407.5210
852\MP2=-409.1036624\RMSD=5.107e-09\Thermal=0.\PG=C01 [X(C9H21N1)]\\@

47-Me^{+}

1\1\GINC-CIPCLU07\SP\RMP2-FC\6-31+G(2d,p)\C10H24N1(1+)\C2175\11-Nov-20
10\0\\#p MP2(FC)/6-31+G(2d,p) scf=tight\\n6mesp_1\\1,1\N\C,1,1.5343436
9\H,2,1.09370657,1,106.02535942\C,1,1.53437803,2,106.49920358,3,-53.33
591419,0\H,4,1.09568281,1,105.17939293,2,-60.55828392,0\H,4,1.09371474
,1,106.02833647,2,53.26387497,0\C,1,1.50590704,2,109.33942886,4,-118.0
220563,0\H,7,1.0928464,1,108.99636449,2,58.06373779,0\H,7,1.09041242,1
,109.27004331,2,-62.13121198,0\H,7,1.09041863,1,109.26542113,2,178.253
7712,0\C,1,1.52890635,7,107.90606592,4,-121.8787679,0\H,11,1.09380477,
1,105.85101128,7,56.85840227,0\H,11,1.0938023,1,105.85501428,7,-56.875
07227,0\C,4,1.53178591,1,117.00710598,7,-65.45138584,0\H,14,1.0968682,
4,111.22554421,1,57.43550962,0\H,14,1.09687887,4,110.67066514,1,-61.93
265558,0\C,14,1.540868,4,109.42617958,1,177.89295242,0\H,17,1.09662601
,14,111.48288916,4,60.03112364,0\H,17,1.09442765,14,109.9256439,4,179.
47260656,0\H,17,1.09648538,14,111.48378353,4,-61.03891163,0\H,2,1.0956
8305,1,105.18227525,7,-57.53573695,0\C,11,1.53120459,1,116.17296601,7,
179.99148831,0\H,22,1.09718356,11,110.65862488,1,-59.3726345,0\H,22,1.
09718001,11,110.6553918,1,59.47001249,0\C,22,1.54014553,11,109.6645174
3,1,-179.95355736,0\H,25,1.09665237,22,111.43797496,11,60.46932494,0\H
,25,1.09442455,22,110.07786868,11,-179.98484028,0\H,25,1.09665343,22,1

11.43822295,11,-60.44046366,0\C,2,1.53178655,1,117.00740795,7,65.38732
652,0\H,29,1.09686185,2,111.22270036,1,-57.48489769,0\H,29,1.09688459,
2,110.67342231,1,61.88507097,0\C,29,1.54086979,2,109.42648847,1,-177.9
3952829,0\H,32,1.09662431,29,111.48327357,2,-60.04383062,0\H,32,1.0964
8718,29,111.48336712,2,61.02601733,0\H,32,1.09442723,29,109.92598339,2
,-179.48671638,0\\Version=AM64L-G03RevD.01\State=1-A\HF=-446.9471118\M
P2=-448.6815161\RMSD=6.044e-09\Thermal=0.\PG=C01 [X(C10H24N1)]\\@

48

1\1\GINC-YIN\SP\RMP2-FC\6-31+G(2d,p)\C15H27N1\CHRISTOPH\05-Dec-2010\0\
\#p MP2(FC)/6-31+g(2d,p) scf=tight\\n21sp_4\\0,1\N\H,1,2.05737015\H,1,
2.05826159,2,119.33438757\C,1,1.45896576,2,88.39778374,3,162.79802551,
0\C,4,1.55481017,1,119.17117886,2,-61.83742579,0\C,5,1.5374049,4,102.7
2831676,1,167.5039925,0\C,6,1.5541703,5,104.07138107,4,-40.96544261,0\
C,7,1.5565734,6,105.75942959,5,26.4635176,0\H,5,1.09690566,4,112.77960
158,1,-69.7612276,0\H,5,1.09957655,4,110.18704078,1,50.43656204,0\H,6,
1.09958744,5,109.51226646,4,76.67929637,0\H,6,1.09663605,5,113.4069865
,4,-163.76249893,0\H,7,1.09755765,6,109.79633294,5,-92.4792775,0\H,7,1
.09628605,6,111.77396969,5,149.30522118,0\H,8,1.09647792,7,112.8682832
9,6,119.45584072,0\H,8,1.09682659,7,110.92579446,6,-121.28841625,0\C,1
,1.45900859,4,117.67743346,5,98.4720146,0\C,17,1.55413819,1,119.232790
51,4,-50.516016,0\C,18,1.53769182,17,102.7224935,1,168.66663599,0\C,19
,1.55690798,18,104.40267198,17,-39.71218915,0\C,20,1.55683935,19,105.8
6171898,18,23.20041041,0\H,18,1.09946589,17,110.07268383,1,51.58725803
,0\H,18,1.09697096,17,112.87657586,1,-68.66373296,0\H,19,1.09933997,18
,109.4857585,17,78.06752194,0\H,19,1.09658361,18,113.28955785,17,-162.
59077755,0\H,20,1.09640455,19,111.48530227,18,145.67291105,0\H,20,1.09
726781,19,110.153433,18,-96.18915091,0\H,21,1.09716853,20,110.71199748
,19,-116.77918797,0\H,21,1.09637505,20,113.05599384,19,123.83555151,0\
C,1,1.45907475,4,117.5884987,5,-51.49920232,0\C,30,1.55388652,1,119.29
664413,4,99.37695519,0\C,31,1.53809127,30,102.70124208,1,168.76212185,
0\C,32,1.55720416,31,104.46094448,30,-39.57508865,0\C,33,1.55652788,32
,105.84307506,31,22.8778262,0\H,31,1.09958951,30,110.12392554,1,51.641
56626,0\H,31,1.09692211,30,112.87357954,1,-68.5864045,0\H,32,1.0993113
2,31,109.45302545,30,78.19955669,0\H,32,1.09656199,31,113.28935752,30,
-162.47638276,0\H,33,1.09632952,32,111.45829491,31,145.30393465,0\H,33
,1.09721604,32,110.18106727,31,-96.53412765,0\H,34,1.09723439,33,110.7
1688243,32,-116.27520824,0\H,34,1.09639607,33,113.10381766,32,124.3276
8531,0\H,4,1.09827967,1,106.22910588,17,-21.26217704,0\\Version=AM64L-
G03RevD.01\State=1-A\HF=-638.2327096\MP2=-640.681075\RMSD=6.781e-09\Th
ermal=0.\PG=C01 [X(C15H27N1)]\\@

48-Me$^+$

1\1\GINC-NODE3\SP\RMP2-FC\6-31+G(2d,p)\C16H30N1(1+)\ZIP07\24-Nov-2010\
0\\#p MP2(FC)/6-31+G(2d,p) scf=tight\\n21mesp_29\\1,1\N\H,1,2.14146016
\H,1,2.10474946,2,107.62303728\H,1,2.09128712,3,113.59749577,2,-128.44
193291,0\C,1,1.50847246,4,91.49315802,3,-107.8529245,0\H,5,1.09100727,
1,108.98999324,4,-26.24175671,0\H,5,1.08936948,1,108.97608971,4,93.086
7273,0\C,1,1.56252629,5,109.47889387,2,66.49406642,0\C,8,1.54531495,1,
118.09873034,5,-153.44435492,0\C,9,1.54353245,8,101.08459143,1,175.828
71282,0\C,10,1.55679362,9,105.11841682,8,-36.86226121,0\C,8,1.54939597
,1,116.34841548,5,-29.75323951,0\H,9,1.09295545,8,114.61887691,1,-62.7
4388029,0\H,9,1.09545608,8,110.98593871,1,59.96385729,0\H,10,1.0940255
8,9,111.53576175,8,-159.43155236,0\H,10,1.09692566,9,109.83872837,8,82
.05849143,0\H,11,1.09437168,10,111.46712854,9,-105.43097559,0\H,11,1.0
9434988,10,111.10005419,9,135.51514817,0\H,12,1.09406598,8,112.7565514
5,1,70.82119933,0\H,12,1.09507416,8,110.91852526,1,-49.94187044,0\H,1,
2.09698692,5,140.84057404,8,-119.75589892,0\C,1,1.55086028,5,110.54913

088,8,-123.40673967,0\C,22,1.55164731,1,115.90061016,5,-66.15947167,0\
C,23,1.56014455,22,104.16155662,1,162.59412741,0\C,24,1.55391062,23,10
6.28936916,22,-9.64145936,0\C,25,1.54208679,24,104.90139145,23,-17.743
63447,0\H,23,1.09653249,22,111.36289764,1,44.32982091,0\H,23,1.0943207
4,22,112.38063073,1,-76.31474262,0\H,24,1.09412698,23,111.22055471,22,
111.65861101,0\H,24,1.09449517,23,109.98223365,22,-130.11412617,0\H,25
,1.09710694,24,110.56439261,23,100.44525974,0\H,25,1.09403469,24,112.8
1233964,23,-139.63813878,0\H,26,1.09718971,25,109.39395042,24,-79.1895
4005,0\H,26,1.09345265,25,112.46362618,24,160.69479309,0\C,1,1.5554551
8,5,105.89766573,8,119.27526923,0\C,35,1.53880296,1,117.16531314,5,-17
7.3032494,0\C,36,1.54098728,35,100.92393735,1,172.06739015,0\C,37,1.54
850195,36,103.70129496,35,-43.27611097,0\C,35,1.55711064,1,115.8731241
4,5,-52.19761876,0\H,36,1.09579396,35,111.18260442,1,56.60212955,0\H,3
6,1.09544752,35,113.8063865,1,-66.23690179,0\H,37,1.09417701,36,112.21
358152,35,-165.40916022,0\H,37,1.0980515,36,109.95456777,35,75.1785104
2,0\H,38,1.09380432,37,112.26023797,36,151.19712927,0\H,38,1.09570845,
37,110.0595468,36,-89.66242811,0\H,39,1.09453454,35,110.83872265,1,84.
22814897,0\H,39,1.09595218,35,112.6751081,1,-35.40367405,0\\Version=AM
64L-G03RevD.01\State=1-A\HF=-677.6489898\MP2=-680.2604223\RMSD=4.420e-
09\Thermal=0.\PG=C01 [X(C16H30N1)]\\@

49

1\1\GINC-MORITZ\SP\RMP2-FC\6-31+G(2d,p)\C11H21N1\CHRISTOPH\16-Nov-2010
\0\\#p MP2(FC)/6-31+g(2d,p) scf=tight\\n20sp_3\\0,1\N\H,1,2.06064756\H
,1,2.1033731,2,171.05679105\C,1,1.47058414,2,90.39061596,3,163.9195018
9,0\C,4,1.55008952,1,113.95123394,2,36.84443499,0\C,5,1.55437233,4,105
.82351526,1,156.68111184,0\C,6,1.55756576,5,105.82137576,4,-13.6407581
7,0\C,7,1.53838121,6,105.08608863,5,-12.58592443,0\H,5,1.09902705,4,10
7.6878276,1,38.23275816,0\H,5,1.09526446,4,113.3265571,1,-80.54937626,
0\H,6,1.09624448,5,111.46342657,4,107.02511436,0\H,6,1.09644562,5,110.
60528582,4,-134.73405938,0\H,7,1.09637277,6,112.9587336,5,-135.4499965
9,0\H,7,1.09842323,6,110.10403825,5,105.33747298,0\H,8,1.09561835,7,11
2.09015991,6,157.45051471,0\H,8,1.1008022,7,111.04574898,6,-81.8152255
7,0\C,1,1.46218124,4,111.0930106,8,-57.04364884,0\C,1,1.47656464,17,11
3.54897743,4,129.58552019,0\C,18,1.5566142,1,112.8167449,17,71.5474581
3,0\C,19,1.53954807,18,104.98501651,1,-149.39470771,0\C,20,1.5417752,1
9,103.17825205,18,37.74567867,0\C,21,1.54432506,20,103.72336104,19,-41
.05240972,0\H,19,1.09831024,18,110.66683482,1,-31.27067045,0\H,19,1.09
571351,18,111.34594781,1,87.20386627,0\H,20,1.10016486,19,110.28537809
,18,-79.32501325,0\H,20,1.09713954,19,113.06572764,18,160.49273918,0\H
,21,1.09933231,20,109.33648597,19,76.45077979,0\H,21,1.09724277,20,113
.26333284,19,-164.06052509,0\H,22,1.09695094,21,109.9928522,20,-89.811
977,0\H,22,1.09686197,21,112.44032472,20,151.57279981,0\H,4,1.11346642
,1,110.93067766,17,64.00907985,0\H,17,1.09459627,1,110.28797227,4,-173
.93035844,0\H,17,1.10841582,1,113.73190788,4,-52.80075397,0\\Version=I
A32L-G03RevD.01\State=1-A\HF=-483.2532013\MP2=-485.1044632\RMSD=2.757e
-09\Thermal=0.\PG=C01 [X(C11H21N1)]\\@

49-Me$^+$

1\1\GINC-MAX\SP\RMP2-FC\6-31+G(2d,p)\C12H24N1(1+)\CHRISTOPH\18-Nov-201
0\0\\#p MP2(FC)/6-31+g(2d,p) scf=tight\\n20mesp_1\\1,1\N\H,1,2.1317534
9\H,1,2.09120965,2,116.05026902\H,1,2.13243053,3,121.34590713,2,-111.3
7249129,0\C,1,1.50644135,3,91.81272755,2,-16.29380751,0\C,1,1.50680106
,5,107.34489847,2,-64.20025778,0\H,5,1.09045783,1,108.55773628,6,55.13
792793,0\H,5,1.09068064,1,109.72872004,6,174.92824884,0\C,1,1.54952512
,5,108.39443442,6,-120.98458509,0\C,9,1.54328799,1,116.12376928,5,57.4
4514158,0\C,10,1.54131207,9,100.9005324,1,171.0625934,0\C,11,1.5503243
4,10,104.45155765,9,-41.34066008,0\C,9,1.55105927,1,115.41173823,5,-17

9.09350949,0\H,10,1.09760252,9,111.58606199,1,54.75803225,0\H,10,1.094
78138,9,113.79841438,1,-68.25014524,0\H,11,1.09757579,10,109.54445539,
9,77.24133762,0\H,11,1.09392866,10,112.0851301,9,-163.93421331,0\H,12,
1.09370347,11,111.8684473,10,147.37099761,0\H,12,1.0951008,11,110.6000
8155,10,-93.56717477,0\H,13,1.0953552,9,111.66827233,1,-37.29620706,0\
H,13,1.0945929,9,111.54368448,1,82.5673311,0\H,6,1.09049006,1,108.6021
712,5,55.40393405,0\H,6,1.09024467,1,109.75792318,5,175.24378231,0\H,1
,2.08989803,5,142.61550691,6,117.6804081,0\C,1,1.55087451,5,111.935897
99,6,118.76281605,0\C,25,1.54213216,1,116.11489801,5,-61.10365018,0\C,
26,1.54137991,25,100.82351738,1,170.98264648,0\C,27,1.55038848,26,104.
42692806,25,-41.39861642,0\C,25,1.55022768,1,115.34536291,5,62.6224982
5,0\H,26,1.09784257,25,111.59741739,1,54.71059999,0\H,26,1.09474137,25
,113.80013445,1,-68.314456,0\H,27,1.09762647,26,109.56831924,25,77.184
12118,0\H,27,1.09394596,26,112.08129881,25,-163.98837219,0\H,28,1.0937
3358,27,111.90059011,26,147.78246075,0\H,28,1.09514589,27,110.5662389,
26,-93.19793425,0\H,29,1.09575514,25,111.78016851,1,-37.00577389,0\H,2
9,1.09459341,25,111.47551736,1,82.85343549,0\\Version=IA32L-G03RevD.01
\State=1-A\HF=-522.6778541\MP2=-524.6838801\RMSD=4.559e-09\Thermal=0.\
PG=C01 [X(C12H24N1)]\\@

50

1\1\GINC-MORITZ\SP\RMP2-FC\6-31+G(2d,p)\C12H21N1\CHRISTOPH\15-Nov-2010
\0\\#p MP2(FC)/6-31+g(2d,p) scf=tight\\n18sp_1\\0,1\N\H,1,2.13066303\H
,1,2.13071323,2,70.83424422\C,1,1.4687829,2,93.17249794,3,94.38490362,
0\C,4,1.55911306,1,119.64221545,2,-165.97521889,0\C,5,1.55212235,4,88.
22530868,1,-144.07207949,0\C,6,1.55216267,5,88.00605605,4,21.21106067,
0\H,5,1.09569665,4,110.22891251,1,-32.48852394,0\H,5,1.09493527,4,118.
45164252,1,94.4541044,0\H,6,1.09514496,5,117.59339564,4,141.48798796,0
\H,6,1.09655985,5,111.93738553,4,-91.67813222,0\H,7,1.0951108,6,118.27
112268,5,-142.67173321,0\H,7,1.0954681,6,110.80320672,5,89.99464587,0\
H,4,1.11064921,1,110.60452672,7,-127.13504042,0\C,1,1.46901071,4,109.9
772216,7,64.12159822,0\C,15,1.55869312,1,119.59462043,4,-174.90172959,
0\C,16,1.55217976,15,88.23944035,1,144.19375402,0\C,17,1.55213735,16,8
7.98867413,15,-21.25571839,0\H,16,1.09510652,15,118.31518465,1,-94.323
70526,0\H,16,1.0954165,15,110.43409499,1,32.70742852,0\H,17,1.09514843
,16,117.56738152,15,-141.56943532,0\H,17,1.09653642,16,111.93792794,15
,91.60043736,0\H,18,1.09576097,17,110.8425008,16,-89.71795252,0\H,18,1
.09493861,17,118.26436211,16,142.90878775,0\C,1,1.46884155,4,109.98468
027,7,-174.48397767,0\C,25,1.55886992,1,119.53225121,4,64.07630439,0\C
,26,1.55204491,25,88.22639295,1,144.27290126,0\C,27,1.55200682,26,87.9
7902814,25,-21.35720998,0\H,26,1.0950842,25,118.28146112,1,-94.2938128
5,0\H,26,1.09546655,25,110.47014152,1,32.73119864,0\H,27,1.09655442,26
,111.9586768,25,91.52916209,0\H,27,1.09514715,26,117.55246712,25,-141.
6341374,0\H,28,1.09484652,27,118.21214349,26,142.96612549,0\H,28,1.095
78254,27,110.85636834,26,-89.66208155,0\\Version=IA32L-G03RevD.01\Stat
e=1-A\HF=-521.0363729\MP2=-523.0218539\RMSD=2.864e-09\Thermal=0.\PG=C0
1 [X(C12H21N1)]\\@

50-Me$^+$

1\1\GINC-MAX\SP\RMP2-FC\6-31+G(2d,p)\C13H24N1(1+)\CHRISTOPH\12-Nov-201
0\0\\#p MP2(FC)/6-31+g(2d,p) scf=tight\\n18mesp_2\\1,1\N\H,1,2.1242227
8\H,1,2.09559419,2,169.56425935\H,1,2.09726277,3,66.44706982,2,154.043
95254,0\H,1,2.09739001,3,66.41706485,4,73.4775606,0\C,1,1.49707196,3,1
40.85602756,5,143.26823342,0\C,1,1.5375265,6,111.26673582,2,-46.336782
95,0\C,7,1.55030023,1,120.69589264,6,68.08708141,0\C,8,1.5577401,7,86.
78209179,1,-149.01092539,0\C,7,1.55142028,1,121.96874847,6,-42.7890761
3,0\H,8,1.09505847,7,113.02287716,1,-37.85623079,0\H,8,1.09342423,7,11
7.23856885,1,90.36899705,0\H,9,1.09217552,8,116.78764821,7,141.0196865

7,0\H,9,1.0940966,8,112.05533412,7,-92.07143627,0\H,10,1.09474369,7,11
2.85717196,1,37.06106929,0\H,10,1.09259642,7,117.47572283,1,-91.459217
11,0\H,6,1.08999883,1,109.43095419,7,73.6158315,0\H,6,1.0900117,1,109.
42947576,7,-166.28469942,0\C,1,1.53746976,6,111.37206689,7,-120.006361
99,0\C,19,1.55008402,1,120.70499888,6,68.16011547,0\C,20,1.5577162,19,
86.71712416,1,-149.43725941,0\C,19,1.5518106,1,122.09758544,6,-42.7722
4408,0\H,20,1.0935531,19,117.20846291,1,90.05828851,0\H,20,1.09521913,
19,113.11724523,1,-38.24233306,0\H,21,1.09215825,20,116.77501032,19,14
1.28485676,0\H,21,1.0940648,20,112.06104545,19,-91.79316048,0\H,22,1.0
9268162,19,117.41594467,1,-91.19251072,0\H,22,1.09483557,19,113.019941
61,1,37.39853518,0\C,1,1.53734001,6,111.25659005,7,120.00148776,0\C,29
,1.55167202,1,122.0111999,6,-42.57295529,0\C,30,1.55783819,29,86.70039
974,1,148.10667494,0\C,29,1.54998563,1,120.68969123,6,68.30231831,0\H,
30,1.09252812,29,117.45691916,1,-91.34837974,0\H,30,1.09482076,29,112.
96823669,1,37.25230754,0\H,31,1.09409367,30,112.06763899,29,91.9798227
2,0\H,31,1.09217153,30,116.77342161,29,-141.10370792,0\H,32,1.09339347
,29,117.25253613,1,90.22102338,0\H,32,1.09522467,29,113.00787805,1,-38
.03118068,0\\Version=IA32L-G03RevD.01\State=1-A\HF=-560.4608024\MP2=-5
62.601455\RMSD=8.118e-09\Thermal=0.\PG=C01 [X(C13H24N1)]\\@

51

1\1\GINC-PHOENIX\SP\RMP2-FC\6-31+G(2d,p)\C13H25N1\CHRISTOPH\05-Dec-201
0\0\\#p MP2(FC)/6-31+g(2d,p) scf=tight\\n23sp_4\\0,1\N\H,1,2.07104045\
H,1,2.13709811,2,93.50130528\C,1,1.46379259,2,143.65471719,3,104.64447
164,0\H,4,1.0931207,1,110.07802182,2,40.0141349,0\H,4,1.10753642,1,113
.48213119,2,-80.85606013,0\H,4,1.095536,1,110.65766163,2,157.79479816,
0\C,1,3.90256486,4,103.48370982,5,54.89621317,0\C,8,1.54180894,1,23.57
904194,4,47.04463607,0\C,1,1.47899599,4,113.14584077,9,-27.68101532,0\
C,10,1.54439584,1,111.77379733,4,-69.12593667,0\C,11,1.54149245,10,111
.03495827,1,-173.32336135,0\C,8,1.53904662,1,98.25886561,4,-79.5762873
1,0\H,8,1.10169256,1,97.06309602,4,169.87458912,0\H,8,1.09892375,1,133
.66027525,4,49.02129874,0\H,9,1.09918262,8,109.88632744,1,118.90660286
,0\H,9,1.10071683,8,109.18602975,1,-124.41650773,0\H,11,1.10005751,10,
110.7550034,1,66.31166392,0\H,11,1.09797946,10,108.83808422,1,-50.9283
6307,0\H,12,1.10149189,11,109.37598445,10,64.3795607,0\H,12,1.09874955
,11,109.92819357,10,-179.01235082,0\H,13,1.10152247,8,109.17733716,1,8
6.07525174,0\H,13,1.09885067,8,110.26104382,1,-157.0992034,0\C,1,3.048
73868,4,99.79368874,10,-169.80855093,0\C,24,1.53954166,1,54.68854788,4
,-73.72136838,0\C,1,1.48128709,4,111.41985919,10,131.98221883,0\C,26,1
.54839468,1,113.25361278,4,177.54720727,0\C,27,1.54048726,26,112.27784
165,1,-63.1611694,0\C,24,1.54018586,1,100.38531496,4,177.77956453,0\H,
24,1.09858466,1,62.58671836,4,71.21868418,0\H,24,1.09900869,1,149.4408
5468,4,-5.94824812,0\H,25,1.10048584,24,109.39707242,1,-148.80256505,0
\H,25,1.09619588,24,110.76876904,1,94.91236767,0\H,27,1.09687491,26,11
1.94965974,1,62.40529031,0\H,27,1.10053761,26,106.67671978,1,177.34523
156,0\H,28,1.09881237,27,109.38973755,26,63.55520275,0\H,28,1.09909336
,27,109.82280074,26,-179.23250216,0\H,29,1.10200334,24,108.9948041,1,1
23.23346205,0\H,29,1.09893978,24,110.36618618,1,-120.03876605,0\\Versi
on=AM64L-G03RevD.01\State=1-A\HF=-561.342543\MP2=-563.5068716\RMSD=2.3
22e-09\Thermal=0.\PG=C01 [X(C13H25N1)]\\@

51-Me$^+$

1\1\GINC-PHOBOS\SP\RMP2-FC\6-31+G(2d,p)\C14H28N1(1+)\CHRISTOPH\19-Nov-
2010\0\\#p MP2(FC)/6-31+g(2d,p) scf=tight\\n23mesp_1\\1,1\N\C,1,1.5061
736\H,2,1.09056071,1,109.79808479\H,2,1.0901668,1,109.20843897,3,120.5
7354605,0\H,2,1.09104264,1,108.69196465,4,119.08802714,0\C,1,1.5061850
4,2,107.5007888,4,-46.47693291,0\H,6,1.09056096,1,109.79844551,2,-167.
03825792,0\H,6,1.09016529,1,109.2094585,2,-46.46285436,0\H,6,1.0910419

4,1,108.69188563,2,72.62468705,0\H,1,2.11229058,2,139.20837169,6,-127.
53889797,0\C,1,3.95395991,2,94.31931923,6,107.4949625,0\C,11,1.5481048
6,1,23.35887742,2,-50.24004915,0\C,12,1.54261745,11,109.6575075,1,-5.3
8675568,0\C,13,1.5416725,12,110.13898998,11,-58.42063809,0\C,14,1.5459
8895,13,109.9030508,12,59.42480205,0\C,11,1.53524364,1,99.97777048,2,-
174.39411091,0\H,11,1.10014373,1,96.37199426,2,74.24138206,0\H,11,1.09
637673,1,131.6772114,2,-44.63157859,0\H,12,1.09933615,11,108.86323448,
1,-126.52132799,0\H,12,1.09450987,11,108.24979761,1,116.73860887,0\H,1
3,1.09371724,12,107.80432337,11,60.41585269,0\H,14,1.09592046,13,112.1
0005307,12,-179.35327936,0\H,14,1.0982819,13,109.93407641,12,-59.96860
173,0\H,15,1.10008607,14,109.35837496,13,64.02465491,0\H,15,1.09635466
,14,108.60349048,13,-179.95795873,0\H,16,1.10021177,11,109.6480064,1,8
7.259597,0\H,16,1.09650262,11,110.01489067,1,-155.73451378,0\C,1,3.945
06448,2,98.76702899,6,-137.28754083,0\C,28,1.5459905,1,23.00271869,2,-
37.0824818,0\C,29,1.54166821,28,109.90307754,1,6.86843375,0\C,30,1.542
62522,29,110.138977,28,59.42294999,0\C,31,1.54810451,30,109.65908441,2
9,-58.41837079,0\C,32,1.53524354,31,112.22792193,30,56.30780675,0\H,28
,1.09635514,1,131.40483541,2,-45.72173281,0\H,28,1.10008744,1,96.03632
939,2,-164.23156967,0\H,29,1.09592187,28,108.87478035,1,-116.26742886,
0\H,29,1.0982783,28,108.5281099,1,127.11873727,0\H,31,1.09933769,30,11
0.60886839,29,61.65685353,0\H,31,1.09450443,30,111.73145045,29,-178.44
260588,0\H,32,1.09637626,31,108.40437737,30,179.02396963,0\H,32,1.1001
4383,31,108.96360214,30,-65.41195799,0\H,33,1.09650031,32,110.01534197
,31,-176.49368223,0\H,33,1.10021336,32,109.64804898,31,66.50028253,0\\
Version=AM64L-G03RevD.01\State=1-A\HF=-600.7684092\MP2=-603.0866521\RM
SD=1.163e-09\Thermal=0.\PG=C01 [X(C14H28N1)]\\@

52

1\1\GINC-LX64E19\SP\RMP2-FC\6-31+G(2d,p)\C6H11N1\UI271AB\26-Mar-2011\0
\\# MP2(FC)/6-31+G(2d,p) scf=tight geom=check guess=read\\z1_01 B98/6-
31G(d) opt 1-azabicyclo[2.2.1]heptane\\0,1\C,0,0.3614180437,-0.8333601
684,-1.1991680576\C,0,-0.1943157112,1.1781500898,0.\C,0,0.5950493008,0
.7217366869,-1.2499325623\H,0,1.2887921062,-1.4119683973,-1.1303322936
\H,0,-0.1841581745,-1.1806745191,-2.0845827241\H,0,1.6551928651,0.9975
097448,-1.1965386702\H,0,0.1815685406,1.1580638377,-2.168111333\C,0,-1
.3350417272,0.1272024211,0.\H,0,-1.9707205256,0.1569001974,0.893559267
8\H,0,-1.9707205256,0.1569001974,-0.8935592678\C,0,0.3614180437,-0.833
3601684,1.1991680576\H,0,-0.1841581745,-1.1806745191,2.0845827241\H,0,
1.2887921062,-1.4119683973,1.1303322936\C,0,0.5950493008,0.7217366869,
1.2499325623\H,0,0.1815685406,1.1580638377,2.168111333\H,0,1.655192865
1,0.9975097448,1.1965386702\H,0,-0.4850389612,2.233262129,0.\N,0,-0.48
27476534,-1.0879963642,0.\\Version=EM64L-G09RevB.01\State=1-A'\HF=-288
.0630245\MP2=-289.1447154\RMSD=2.913e-09\PG=CS [SG(C2H1N1),X(C4H10)]\\
@

52-Me$^+$

1\1\GINC-LX64E195\SP\RMP2-FC\6-31+G(2d,p)\C7H14N1(1+)\UI271AB\26-Mar-2
011\0\\# MP2(FC)/6-31+G(2d,p) scf=tight geom=check guess=read\\z1_03 B
98/6-31G(d) opt CH3+ + 1-azabicyclo[2.2.1]heptane\\1,1\C,0,0.397439347
5,-0.8329876095,-1.2371385987\C,0,-0.1964995214,1.1618761185,0.0000639
133\C,0,0.5857687925,0.7099628354,-1.2588907195\H,0,1.3174819467,-1.41
2439101,-1.1310183516\H,0,-0.160518249,-1.2077019866,-2.0994312516\H,0
,1.6399136132,0.9982314374,-1.2169334308\H,0,0.1550512504,1.1354172365
,-2.1705340799\C,0,-1.3598197662,0.1607767712,0.000124459\H,0,-1.98308
47808,0.1593617046,0.8991979663\H,0,-1.9832145644,0.1594181562,-0.8988
588393\C,0,0.3976154901,-0.8330739467,1.2370564987\H,0,-0.1602426565,-
1.2077935855,2.0994098596\H,0,1.3176255391,-1.4125523589,1.1308019886\
C,0,0.5859947644,0.7098724034,1.2588459036\H,0,0.1554664223,1.13528893

93,2.1705963262\H,0,1.6401392552,0.9981140242,1.2167004605\H,0,-0.4834
03524,2.2148106988,0.0001298528\N,0,-0.4691638178,-1.0725798194,0.0000
155147\C,0,-1.1628699936,-2.3907651213,-0.0000244221\H,0,-0.4141075409
,-3.1873339952,-0.0005862261\H,0,-1.7849349364,-2.4671376361,0.8951752
969\H,0,-1.7857494303,-2.466632095,-0.8947021877\\Version=EM64L-G09Rev
B.01\State=1-A\HF=-327.4983912\MP2=-328.7253074\RMSD=5.881e-09\PG=C01
[X(C7H14N1)]\\@

53
see reference 4

53-Me+
see reference 4

53-BH+
1\1\GINC-YANG\SP\RMP2-FC\6-31+G(2d,p)\C20H24N1(1+)\CHRISTOPH\10-Jan-20
11\0\\#p MP2(FC)/6-31+g(2d,p) scf=tight\\yin4bhsp_4\\1,1\C\H,1,1.09465
332\C,1,4.34738137,2,103.23859875\C,3,1.3966771,1,58.37975523,2,0.0700
3491,0\C,4,1.39702514,3,119.95346637,1,1.01692803,0\C,5,1.40766893,4,1
21.26503228,3,-0.35423154,0\C,6,1.4082444,5,118.06345485,4,-0.09468911
,0\C,7,1.39703816,6,120.68339513,5,0.55252366,0\H,3,1.08674024,1,178.4
107226,6,172.41534444,0\H,4,1.08678882,3,120.348426,1,-178.8384316,0\H
,5,1.08885154,4,119.06107241,3,-179.47514892,0\H,7,1.08477507,6,121.27
854765,5,-179.12610645,0\H,8,1.08692692,7,119.40341596,6,179.98708814,
0\C,1,4.34771438,6,117.72183825,5,113.47835322,0\C,14,1.39704263,1,58.
55321036,6,-123.53893678,0\C,15,1.39662991,14,119.92620191,1,-0.538148
43,0\C,16,1.40764732,15,121.26471112,14,0.10896884,0\C,17,1.40749215,1
6,118.09318956,15,0.95747121,0\C,18,1.39721429,17,120.6722826,16,-1.41
578894,0\H,14,1.08677199,1,178.71175757,6,-99.52776014,0\H,15,1.086818
35,14,120.35394138,1,179.06085862,0\H,16,1.08904316,15,118.99442485,14
,179.05784789,0\H,18,1.08477573,17,121.15813639,16,176.89587023,0\H,19
,1.08689598,18,119.41856419,17,179.93815651,0\C,1,3.91749142,6,97.9747
5135,5,-93.07120454,0\C,25,1.54844286,1,20.97565599,6,63.70474905,0\N,
26,1.52368673,25,110.45068039,1,7.48364054,0\C,27,1.52468121,26,107.97
423235,25,68.27137797,0\C,28,1.54826381,27,110.40158084,26,-48.5462318
1,0\H,25,1.09643866,1,111.48816103,6,-27.28307303,0\H,25,1.09560637,1,
125.69056661,6,105.03239133,0\H,26,1.09489615,25,112.14152823,1,125.30
545692,0\H,26,1.09132891,25,112.59288539,1,-112.24000412,0\H,28,1.0919
1889,27,106.29645651,26,-170.87366752,0\H,28,1.09323834,27,107.0888464
6,26,73.9514484,0\H,29,1.09648707,28,110.4824486,27,104.61682483,0\H,2
9,1.09553325,28,108.97559955,27,-137.86680641,0\C,27,1.51881234,26,108
.32862402,25,-48.51120639,0\H,38,1.09154656,27,106.17892139,26,-53.559
43141,0\H,38,1.09139248,27,106.96798691,26,-169.98073592,0\C,38,1.5499
847,27,110.33817674,26,67.99659791,0\H,41,1.09552231,38,108.95177733,2
7,-137.68865257,0\H,41,1.09641523,38,110.41455846,27,104.81154219,0\C,
41,1.54005258,38,109.01572998,27,-16.46676633,0\H,44,1.09445097,41,110
.63473585,38,-170.4959907,0\\Version=AM64L-G03RevD.01\State=1-A\HF=-82
5.6562391\MP2=-828.7281771\RMSD=7.671e-09\Thermal=0.\PG=C01 [X(C20H24N
1)]\\@

53-TT+
1\1\GINC-PHOENIX\SP\RMP2-FC\6-31+G(2d,p)\C26H28N1(1+)\CHRISTOPH\26-Oct
-2011\0\\#p MP2(FC)/6-31+g(2d,p) scf=tight\\yin4ttsp_1\\1,1\C\C,1,4.38
43372\C,2,1.39378311,1,59.31989313\C,3,1.40014264,2,120.62325126,1,0.8
9724726,0\C,4,1.40695249,3,121.33060219,2,0.17503662,0\C,5,1.41393273,
4,117.01003926,3,-2.121738,0\C,6,1.39426757,5,121.68571928,4,2.7324807
7,0\H,2,1.08675093,1,179.39905785,5,-175.17641485,0\H,3,1.08685675,2,1
20.26596435,1,-178.68834641,0\H,4,1.08301035,3,118.21309335,2,-178.406

36557,0\H,6,1.08499811,5,120.66560216,4,-175.66129384,0\H,7,1.08704045
,6,119.45276372,5,179.56939228,0\C,1,4.10305902,5,91.0434863,4,-113.93
714988,0\C,13,1.54839658,1,22.16811894,5,1.11261119,0\C,14,2.45051918,
13,82.60255385,1,9.03528448,0\C,15,1.54869988,14,95.78606084,13,-23.17
49117,0\H,13,1.09708497,1,106.64388123,5,106.31544492,0\H,14,1.0886716
4,13,112.42299935,1,106.61396128,0\H,15,1.0887093,14,143.33048715,13,-
164.39332246,0\H,16,1.09709865,15,111.07540817,14,-132.00392278,0\C,1,
4.38378474,5,110.47182885,4,-9.15739514,0\C,21,1.39385922,1,59.3247665
1,5,117.95958741,0\C,22,1.40017977,21,120.5993046,1,0.94514238,0\C,23,
1.40711334,22,121.33057573,21,0.17105986,0\C,24,1.41389354,23,117.0391
7326,22,-2.18585179,0\C,25,1.39438476,24,121.64851441,23,2.8153131,0\H
,21,1.08675947,1,179.36045639,24,-175.8866597,0\H,22,1.08687988,21,120
.27277934,1,-178.64306003,0\H,23,1.08309062,22,118.24595798,21,-178.37
065289,0\H,25,1.08530304,24,120.74316682,23,-175.55073387,0\H,26,1.087
03544,25,119.44477446,24,179.53551458,0\C,1,4.38383542,24,110.85356007
,23,-9.39939246,0\C,32,1.39382483,1,59.3498783,24,118.60123477,0\C,33,
1.40020201,32,120.5820612,1,0.79927686,0\C,34,1.40695529,33,121.350213
1,32,0.12480621,0\C,35,1.41412896,34,117.04096603,33,-2.19134081,0\C,3
6,1.39426269,35,121.62485197,34,2.87221731,0\H,32,1.08675671,1,179.495
75441,35,-176.63562247,0\H,33,1.08683824,32,120.28198156,1,-178.827077
46,0\H,34,1.08302719,33,118.27834511,32,-178.44612359,0\H,36,1.0851736
5,35,120.72242404,34,-175.55914428,0\H,37,1.08703798,36,119.44259623,3
5,179.50506891,0\H,13,1.09602768,1,128.96170664,35,95.10257512,0\H,14,
1.08906288,13,110.9559495,1,-133.37245396,0\H,15,1.08913127,14,82.0972
8103,13,87.25555697,0\H,16,1.096043,15,108.30655408,14,110.67208772,0\
C,15,2.45043843,14,60.00101217,13,-101.16096567,0\C,47,1.54847774,15,9
5.80437843,14,77.9443478,0\H,47,1.08917195,15,82.05126999,14,-171.6140
9813,0\H,47,1.08869924,15,143.36883453,14,-63.41926212,0\H,48,1.097078
8,47,111.06939655,15,-131.97793876,0\H,48,1.0960298,47,108.34268699,15
,110.66980871,0\C,16,1.53949148,15,108.31645755,14,-10.29081265,0\H,53
,1.09450324,16,111.27683883,15,166.21246336,0\N,47,1.52675183,15,36.64
287136,14,-39.06910066,0\\Version=AM64L-G03RevD.01\State=1-A\HF=-1055.
1693329\MP2=-1059.1152652\RMSD=6.406e-09\Thermal=0.\PG=C01 [X(C26H28N1
)]\\@

54
see reference 4

54-Me⁺
see reference 4

54-BH⁺

1\1\GINC-YANG\SP\RMP2-FC\6-31+G(2d,p)\C20H21N2(1+)\CHRISTOPH\09-Jan-20
11\0\\#p MP2(FC)/6-31+g(2d,p) scf=tight\\yin1bhsp_46\\1,1\C\H,1,1.0965
6439\C,1,4.33250763,2,107.56056883\C,3,1.39858657,1,59.1927506,2,-29.5
1875604,0\C,4,1.39692515,3,120.09853747,1,0.8207697,0\C,5,1.40314014,4
,120.39801591,3,-0.25616423,0\C,6,1.40318713,5,119.2683188,4,0.3644678
6,0\C,3,1.39793127,1,60.60629711,6,39.32106289,0\H,3,1.08687657,1,179.
14528378,6,-174.64787592,0\H,4,1.08692347,3,120.2051214,1,-179.5117271
9,0\H,5,1.08877046,4,119.74951923,3,-179.77805908,0\H,7,1.08845638,6,1
20.45919695,5,179.42488576,0\H,8,1.08710836,3,120.13128128,1,179.38501
48,0\C,1,4.33303173,6,116.76908258,5,88.4956373,0\C,14,1.39795733,1,60
.51611665,6,27.87907456,0\C,15,1.39882124,14,120.28174635,1,-0.3030234
7,0\C,16,1.40353992,15,120.14604505,14,0.26371355,0\C,17,1.40584751,16
,119.22677341,15,-0.53999726,0\C,18,1.39608047,17,120.53147944,16,0.43
547922,0\H,14,1.08699571,1,179.37123495,17,-148.52623364,0\H,15,1.0870
0155,14,120.12402791,1,-179.57567564,0\H,16,1.08687016,15,119.74500009
,14,-178.4496732,0\H,18,1.08927144,17,119.82715623,16,-179.11880514,0\

H,19,1.0869812,18,119.840062,17,-179.43456356,0\C,1,4.3434686,17,113.3
7831363,16,-98.41559676,0\C,25,1.43174071,1,56.97397105,17,-162.661174
22,0\C,26,1.37020845,25,120.36105701,1,0.2438617,0\C,27,2.34087672,26,
91.88529789,25,0.14954422,0\C,28,1.37418878,27,91.45111855,26,-0.34797
193,0\H,26,1.08272696,25,121.54433086,1,-179.65303531,0\H,27,1.0850062
5,26,121.78043395,25,-178.95821976,0\H,28,1.08358021,27,146.60227503,2
6,179.40463337,0\H,29,1.08272295,28,117.82165841,27,-179.91156417,0\N,
25,1.3465508,1,179.04458531,17,-169.24894765,0\C,34,1.46695394,25,120.
29120842,1,-173.22254732,0\H,35,1.09708947,34,111.05238251,25,-61.3273
9386,0\H,35,1.09697416,34,111.03219705,25,60.43880495,0\H,35,1.0901895
6,34,108.95590206,25,179.52528022,0\C,34,1.46719529,25,120.43196095,1,
6.87454786,0\H,39,1.09019062,34,108.91768829,25,178.98485081,0\H,39,1.
09714172,34,111.12783209,25,-61.89354041,0\H,39,1.09691829,34,111.0433
2023,25,59.97012409,0\N,28,1.35738006,27,30.63491875,26,179.36553795,0
\\Version=AM64L-G03RevD.01\State=1-A\HF=-878.3815984\MP2=-881.5720634\
RMSD=7.031e-09\Thermal=0.\PG=C01 [X(C20H21N2)]\\@

54-TT^{+}

1\1\GINC-CALYPSO\SP\RMP2-FC\6-31+G(2d,p)\C26H25N2(1+)\CHRISTOPH\26-Oct
-2011\0\\#p MP2(FC)/6-31+g(2d,p) scf=tight\\yin1ttsp_1\\1,1\C\C,1,4.37
708153\C,2,1.39973161,1,59.09158582\C,3,1.39481679,2,120.12986895,1,0.
02297139,0\C,4,1.41076451,3,121.04834574,2,-0.48403177,0\C,5,1.4019338
1,4,118.23487339,3,1.25262631,0\C,2,1.39476767,1,60.27471895,5,-14.607
97939,0\H,2,1.086924,1,179.34541896,5,167.64790564,0\H,3,1.08723407,2,
120.25333538,1,179.91120724,0\H,4,1.0881911,3,118.96798356,2,179.54424
121,0\H,6,1.08444247,5,120.44125679,4,178.39210819,0\H,7,1.08707995,2,
120.20851635,1,-179.64392013,0\C,1,4.38400542,5,102.99135017,4,-52.062
57431,0\C,13,1.42974492,1,56.69018656,5,-49.26039268,0\C,14,1.37102375
,13,120.55159594,1,0.26713276,0\C,15,2.33448045,14,91.84100014,13,-0.1
1844175,0\C,16,1.37468209,15,91.47303036,14,0.3483187,0\H,14,1.0828917
7,13,121.61215332,1,179.7411329,0\H,15,1.08315481,14,121.40570328,13,1
79.07809475,0\H,16,1.08201579,15,147.38253541,14,179.17681679,0\H,17,1
.08287564,16,117.63703591,15,179.16962237,0\C,1,4.37735009,5,114.19761
805,4,69.3857998,0\C,22,1.39988934,1,59.07880436,5,169.93986939,0\C,23
,1.39485972,22,120.14065049,1,-0.276553,0\C,24,1.41181145,23,121.02840
587,22,1.2580417,0\C,25,1.40294651,24,118.18588728,23,-1.79974204,0\C,
22,1.39486616,1,60.31430625,25,21.8589474,0\H,22,1.08699954,1,179.1685
1092,25,165.41730442,0\H,23,1.08716544,22,120.27274387,1,-179.15424438
,0\H,24,1.08767614,23,119.16855496,22,-177.88054466,0\H,26,1.08400981,
25,120.554574,24,-177.42980507,0\H,27,1.08706265,22,120.20298687,1,179
.04109032,0\C,1,4.37355089,25,106.24782597,24,44.94308929,0\C,33,1.399
85178,1,58.46073663,25,55.44137872,0\C,34,1.39421782,33,120.26978679,1
,-0.24614298,0\C,35,1.40955918,34,120.98500422,33,0.98315814,0\C,36,1.
40166193,35,118.20616508,34,-1.83059568,0\C,33,1.39381109,1,60.8303691
8,36,9.99622986,0\H,33,1.08681151,1,178.78032187,36,-176.84510627,0\H,
34,1.08706838,33,120.25031681,1,-179.59025408,0\H,35,1.08723131,34,119
.37174595,33,-178.39526366,0\H,37,1.08562624,36,121.34824012,35,-177.3
4336218,0\H,38,1.0871655,33,120.22864309,1,179.40424274,0\N,13,1.34710
266,1,178.92537541,36,65.39711698,0\C,44,1.46649986,13,120.27380408,1,
-175.09050824,0\H,45,1.09695901,44,111.01120531,13,60.09243076,0\H,45,
1.09028069,44,108.97512423,13,179.18198072,0\H,45,1.09719683,44,111.10
812353,13,-61.62563882,0\C,44,1.46662228,13,120.39736085,1,5.33493367,
0\H,49,1.09026606,44,108.93943423,13,-179.62198795,0\H,49,1.09702927,4
4,111.0697277,13,-60.56099348,0\H,49,1.09715722,44,111.12420603,13,61.
22770918,0\N,16,1.35814683,15,31.06199055,14,-179.17626623,0\\Version=
AM64L-G03RevD.01\State=1-A\HF=-1107.919868\MP2=-1111.9744912\RMSD=5.22
3e-09\Thermal=0.\PG=C01 [X(C26H25N2)]\\@

54-Ac+
see reference 9

55
1\1\GINC-EDDY\SP\RMP2-FC\6-31+G(2d,p)\C18H15N1\CHRISTOPH\05-Dec-2010\0
\\#p MP2(FC)/6-31+g(2d,p) scf=tight\\npsp_2\\0,1\N\C,1,4.23933386\C,2,
1.3988744,1,59.61904063\C,3,1.39603879,2,120.62032048,1,0.46366891,0\C
,4,1.4068975,3,120.2707172,2,-0.94888634,0\C,5,1.40691525,4,119.001634
78,3,0.48394674,0\C,6,1.39591192,5,120.25783324,4,0.44940496,0\H,2,1.0
8725528,1,179.97550434,5,-164.17158336,0\H,3,1.08795884,2,120.10661774
,1,179.53235104,0\H,4,1.08664122,3,120.33396654,2,179.14915222,0\H,6,1
.08666188,5,119.40072359,4,-179.65826341,0\H,7,1.08797547,6,119.266007
27,5,179.9739667,0\C,1,4.23912463,5,120.00729047,4,40.88806133,0\C,13,
1.39897046,1,59.5923236,5,41.27139368,0\C,14,1.39590077,13,120.6300722
8,1,0.47218657,0\C,15,1.40694121,14,120.25922667,13,-0.93268548,0\C,16
,1.40684936,15,119.00494754,14,0.46734519,0\C,17,1.39593428,16,120.266
94365,15,0.45706964,0\H,13,1.08723875,1,179.98797933,16,-167.14426363,
0\H,14,1.08796326,13,120.10181464,1,179.55937411,0\H,15,1.08668223,14,
120.35042537,13,179.16677187,0\H,17,1.08667308,16,119.40300445,15,-179
.61608872,0\H,18,1.08797397,17,119.2602554,16,179.98077897,0\C,1,4.239
26303,16,119.98194357,15,-139.06071317,0\C,24,1.39891563,1,59.59707492
,16,41.44945808,0\C,25,1.39592826,24,120.61634759,1,0.46061968,0\C,26,
1.40692826,25,120.26278908,24,-0.95038157,0\C,27,1.40676353,26,119.016
50926,25,0.48836869,0\C,28,1.39603288,27,120.25287345,26,0.44499059,0\
H,24,1.08724795,1,179.97715264,16,89.97961,0\H,25,1.08793956,24,120.09
610695,1,179.52612376,0\H,26,1.08667011,25,120.35234341,24,179.1622411
2,0\H,28,1.08665853,27,119.40119601,26,-179.68444025,0\H,29,1.08793162
,28,119.26786387,27,179.97956954,0\\Version=AM64L-G03RevD.01\State=1-A
\HF=-744.8706992\MP2=-747.581396\RMSD=3.336e-09\Thermal=0.\PG=C01 [X(C
18H15N1)]\\@

55-Me+
1\1\GINC-PHOBOS\SP\RMP2-FC\6-31+G(2d,p)\C19H18N1(1+)\CHRISTOPH\05-Dec-
2010\0\\#p MP2(FC)/6-31+g(2d,p) scf=tight\\npmesp_1\\1,1\C\H,1,1.08865
31\H,1,1.08866659,2,110.31708217\H,1,1.08856228,2,110.34719788,3,122.1
3801353,0\C,1,5.00971597,4,67.3863766,3,155.70050625,0\C,5,1.39560523,
1,72.9045251,4,-97.30678936,0\C,6,1.40040384,5,120.63977362,1,-11.7622
0231,0\C,7,1.39550357,6,118.98558532,5,0.31798709,0\C,8,1.39998061,7,1
21.05771364,6,-1.38273002,0\C,9,1.39649106,8,119.20225606,7,1.55791844
,0\H,5,1.08641428,1,162.92296307,8,179.09183921,0\H,6,1.08645918,5,120
.36444293,1,168.19013161,0\H,7,1.08359841,6,119.57760482,5,-179.818019
9,0\H,9,1.08482469,8,121.71616647,7,-177.37616547,0\H,10,1.08643493,9,
119.17519431,8,179.66149667,0\C,7,4.72582197,6,143.14349503,5,153.9608
013,0\C,16,1.39893953,7,60.51247867,6,-87.78561009,0\C,17,1.39613387,1
6,120.40829995,7,-37.30379279,0\C,18,1.40035761,17,119.19624581,16,-0.
60928594,0\C,19,1.39508909,18,121.10182529,17,1.51961874,0\C,16,1.3954
1335,7,68.83595591,6,126.21764997,0\H,16,1.08641774,7,147.82347193,6,1
2.8073715,0\H,17,1.08644041,16,120.3851967,7,142.3600326,0\H,18,1.0848
7929,17,119.15835646,16,178.26499392,0\H,20,1.08360425,19,121.39236401
,18,178.82663455,0\H,21,1.08644844,16,120.36759173,7,-145.41228536,0\C
,20,4.72753178,19,94.83067098,18,-166.86266853,0\C,27,1.3955737,20,68.
82211184,19,-75.96073815,0\C,28,1.40043878,27,120.62095932,20,34.60757
827,0\C,29,1.39532744,28,118.96574472,27,0.34805421,0\C,30,1.39995067,
29,121.10857881,28,-1.39585688,0\C,31,1.39627343,30,119.1749483,29,1.5
241128,0\H,27,1.08641574,20,147.88367666,19,170.62063064,0\H,28,1.0864
3247,27,120.3651831,20,-145.44667063,0\H,29,1.08356339,28,119.69594039
,27,-179.84791118,0\H,31,1.08485557,30,121.63941085,29,-177.47113704,0
\H,32,1.08643665,31,119.18292981,30,179.6529839,0\N,8,1.52259316,7,120

.34855719,6,-178.82202014,0\\Version=AM64L-G03RevD.01\State=1-A\HF=-78
4.2519872\MP2=-787.1263346\RMSD=3.122e-09\Thermal=0.\PG=C01 [X(C19H18N
1)]\\@

56
see reference 4

56-Me+
see reference 4

56-BH+
1\1\GINC-NAUTILUS\SP\RMP2-FC\6-31+G(2d,p)\C22H23N2(1+)\CHRISTOPH\14-Ja
n-2011\0\\#p MP2(FC)/6-31+g(2d,p) scf=tight\\yin7bhsp_44\\1,1\C\H,1,1.
0966302\C,1,4.33352173,2,106.20422375\C,3,1.3995864,1,59.35836585,2,-3
2.12214993,0\C,4,1.39594737,3,119.93641328,1,0.17847741,0\C,5,1.405755
81,4,120.53309235,3,-0.04354187,0\C,6,1.40331452,5,119.23159272,4,0.40
2169,0\C,3,1.39784219,1,60.5145364,6,11.1760663,0\H,3,1.08700457,1,179
.35695718,6,-147.72544062,0\H,4,1.08700658,3,120.21841979,1,179.559403
11,0\H,5,1.08926108,4,119.65009354,3,179.51412872,0\H,7,1.08692193,6,1
20.06983005,5,178.23880454,0\H,8,1.08701417,3,120.12637411,1,-179.5674
1103,0\C,1,4.33326602,6,114.37441627,5,-151.67741258,0\C,14,1.39773006
,1,60.60982041,6,-92.65002271,0\C,15,1.39858765,14,120.20037514,1,-0.6
7959681,0\C,16,1.40295737,15,120.26122212,14,0.0065846,0\C,17,1.403257
3,16,119.24407534,15,-0.22768615,0\C,18,1.39678288,17,120.41765164,16,
0.34599654,0\H,14,1.08689492,1,179.13920605,6,54.48234018,0\H,15,1.087
13656,14,120.13108908,1,179.39930373,0\H,16,1.08838194,15,119.24476338
,14,-179.64928482,0\H,18,1.08879374,17,119.82966625,16,179.87044113,0\
H,19,1.08694845,18,119.69817029,17,-179.91033234,0\C,1,4.33910302,6,11
3.39875571,5,80.5561213,0\C,25,1.42971402,1,58.69769946,6,19.86161628,
0\C,26,1.3730946,25,120.60356923,1,-0.7832247,0\C,27,2.34354891,26,91.
45232935,25,0.49507104,0\C,28,1.36930775,27,91.91469229,26,-0.33703075
,0\H,26,1.08329051,25,121.18456274,1,178.84405333,0\H,27,1.0835397,26,
121.98480844,25,-179.58108251,0\H,28,1.08502884,27,146.2755255,26,178.
63918783,0\H,29,1.08333697,28,118.45315529,27,-179.23127302,0\N,25,1.3
3895299,1,179.09449586,6,-168.41173364,0\C,34,1.47886021,25,123.946155
86,1,-175.12760094,0\C,35,1.53744251,34,103.64017716,25,-167.85542864,
0\C,36,1.53982185,35,103.39406317,34,-30.34109591,0\C,34,1.47900432,25
,124.11165407,1,5.25980416,0\H,35,1.09588323,34,110.38148597,25,-46.99
947075,0\H,35,1.09927842,34,109.75828941,25,72.59783823,0\H,36,1.09367
145,35,111.91085387,34,-152.37808079,0\H,36,1.09664144,35,109.93494139
,34,87.57041425,0\H,37,1.09368719,36,113.09614855,35,158.92367219,0\H,
37,1.09672234,36,110.46815664,35,-79.89438661,0\H,38,1.09588659,34,110
.44323437,25,-47.85150918,0\H,38,1.09931524,34,109.75742096,25,71.7975
1337,0\N,27,1.35890692,26,122.06266509,25,0.25168216,0\\Version=AM64L-
G03RevD.01\State=1-A\HF=-955.3051698\MP2=-958.7792133\RMSD=7.426e-09\T
hermal=0.\PG=C01 [X(C22H23N2)]\\@

56-TT+
1\1\GINC-BORIX\SP\RMP2-FC\6-31+G(2d,p)\C28H27N2(1+)\CHRISTOPH\28-Oct-2
011\0\\#p MP2(FC)/6-31+g(2d,p) scf=tight\\yin7ttsp_3\\1,1\C\C,1,4.3738
917\C,2,1.39985377,1,58.47201625\C,3,1.39422265,2,120.26378481,1,-0.23
552789,0\C,4,1.40950524,3,120.99581642,2,0.99482095,0\C,5,1.40163431,4
,118.20013495,3,-1.88846355,0\C,2,1.39385257,1,60.81445903,5,10.724490
36,0\H,2,1.08683708,1,178.79497112,5,-175.58196863,0\H,3,1.08708334,2,
120.25205878,1,-179.58473835,0\H,4,1.08724124,3,119.38517075,2,-178.38
132941,0\H,6,1.08563057,5,121.30073037,4,-177.29968137,0\H,7,1.0871882
7,2,120.22387563,1,179.40840333,0\C,1,4.37769735,5,114.48152028,4,-70.
91278363,0\C,13,1.39967081,1,59.0628204,5,-169.00039234,0\C,14,1.39480

272,13,120.12904878,1,0.03771204,0\C,15,1.41064709,14,121.06837987,13,
-0.5043327,0\C,16,1.40196087,15,118.21496203,14,1.28243487,0\C,13,1.39
477667,1,60.28871963,5,10.60847372,0\H,14,1.08726016,13,120.25371596,1
,179.91350347,0\H,15,1.08815258,14,118.96027121,13,179.49862206,0\H,17
,1.08445979,16,120.48034094,15,178.36596551,0\H,18,1.08709168,13,120.2
034602,1,-179.64220481,0\C,1,4.37745352,5,106.28075091,4,55.82969439,0
\C,23,1.39990893,1,59.06605818,5,44.76633748,0\C,24,1.39477033,23,120.
1365248,1,-0.22240531,0\C,25,1.41173045,24,121.03508647,23,1.25429403,
0\C,26,1.40283435,25,118.18855817,24,-1.76555834,0\C,23,1.39482395,1,6
0.32634067,5,-134.98392549,0\H,23,1.08701517,1,179.12691889,5,6.197193
83,0\H,24,1.08717846,23,120.27303048,1,-179.09948752,0\H,25,1.08765704
,24,119.19405494,23,-177.89784854,0\H,27,1.08401177,26,120.56147504,25
,-177.48124654,0\H,28,1.08707287,23,120.20235281,1,178.98995602,0\H,13
,1.08694959,1,179.3270385,5,-167.81746658,0\C,1,4.38028379,5,108.28138
669,4,176.27406464,0\C,35,1.42791732,1,58.72265487,5,-110.1711242,0\C,
36,1.37367973,35,120.72770641,1,0.15544693,0\C,37,2.3372009,36,91.4803
0027,35,-0.44341905,0\C,38,1.37005355,37,91.85380081,36,0.33827356,0\H
,36,1.08350868,35,121.2530981,1,-178.77756428,0\H,37,1.0819508,36,121.
15958971,35,-179.45420967,0\H,38,1.08317451,37,146.68896374,36,-178.73
937146,0\H,39,1.08352945,38,118.21629283,37,179.68053108,0\N,35,1.3395
4901,1,178.93273205,5,69.38175414,0\C,44,1.47811601,35,123.91424011,1,
-176.06579754,0\C,45,1.53756114,44,103.64231292,35,168.31247794,0\C,46
,1.53988368,45,103.41533691,44,30.21561795,0\C,44,1.47831447,35,124.07
312488,1,4.00506243,0\H,45,1.09591738,44,110.37788897,35,47.45471097,0
\H,45,1.09941397,44,109.81896355,35,-72.10054961,0\H,46,1.09371188,45,
111.91158985,44,152.24581503,0\H,46,1.09670356,45,109.94740507,44,-87.
74689301,0\H,47,1.09672408,46,110.44070355,45,80.01762255,0\H,47,1.093
71932,46,113.09904761,45,-158.83159314,0\H,48,1.09937864,44,109.821030
22,35,-72.11325747,0\H,48,1.0959048,44,110.43443647,35,47.5022652,0\N,
37,1.35949094,36,122.52674298,35,-0.10579523,0\\Version=AM64L-G03RevD.
01\State=1-A\HF=-1184.8432262\MP2=-1189.181542\RMSD=8.078e-09\Thermal=
0.\PG=C01 [X(C28H27N2)]\\@

56-Ac$^+$
see reference 9

57
1\1\GINC-MAX\SP\RMP2-FC\6-31+G(2d,p)\C11H14N2\EVGENY\15-Feb-2008\0\\#P
MP2/6-31+G(2D,P) SCF=TIGHT INT=FINEGRID\\Single point of TCAP_b MP2/6
-31+G(2d,p)\\0,1\N\N,1,4.22838464\C,1,1.45833541,2,119.82538393\C,3,1.
53235649,1,110.8184192,2,-38.79482405,0\C,4,1.53518041,3,109.84066001,
1,58.66282302,0\C,5,1.51730756,4,110.85178382,3,-49.07547597,0\C,2,1.3
397217,1,57.78757745,3,10.22770154,0\C,2,1.33972114,1,57.78753233,3,-1
68.28074803,0\C,8,1.39498999,2,125.51368698,1,1.3383167,0\C,9,1.517306
48,8,121.31667756,2,179.03829492,0\C,10,1.53518066,9,110.85183757,8,15
7.54361965,0\C,1,1.45833495,3,116.79020006,4,162.50447596,0\C,1,1.3884
336,12,119.49611478,11,40.66769462,0\H,4,1.09712233,3,109.53793908,1,-
179.37028482,0\H,12,1.10630849,1,111.12932786,13,-81.76265034,0\H,12,1
.09778683,1,107.66353296,13,161.35931988,0\H,10,1.10151954,9,109.87569
298,8,-81.80805505,0\H,8,1.09219833,2,115.79603958,1,-178.60948466,0\H
,7,1.09219777,2,115.79604838,1,178.60950633,0\H,3,1.1063085,1,111.1292
7602,13,81.7632637,0\H,5,1.09796751,4,110.5887283,3,-171.4482674,0\H,1
0,1.09796761,9,110.07360705,8,34.87148912,0\H,3,1.09778665,1,107.66348
281,13,-161.35874594,0\H,5,1.10151911,4,109.08592713,3,72.03991039,0\H
,4,1.09805413,3,108.84105851,1,-61.90628135,0\H,11,1.09805315,10,110.0
6868343,9,-70.74876351,0\H,11,1.09712299,10,110.84369116,9,170.2607098
,0\\Version=x86-Linux-G03RevB.03\State=1-A\HF=-533.6584082\MP2=-535.60
23514\RMSD=4.054e-09\PG=C01 [X(C11H14N2)]\\@

57-Me^{+}

1\1\GINC-STEAK\SP\RMP2-FC\6-31+G(2d,p)\C12H17N2(1+)\RAMAN\13-Oct-2011\
0\\#P MP2/6-31+G(2d,p) scf=(direct,tight) int=finegrid guess=read geom
=check\\sp of TCAP1.mca mp2-5 on 2p 900 MB\\1,1\C,0,-2.9926895797,-1.1
746782008,0.0017996958\C,0,-1.6213320467,-1.2299769711,-0.0259095567\C
,0,-0.8790187957,-0.0002624287,0.0029234331\C,0,-1.6207464558,1.229425
3798,0.0443227169\C,0,-2.992417968,1.1743992996,0.0380932426\N,0,-3.68
18280733,-0.0001298754,0.0286559873\N,0,0.4672779555,-0.0004373708,-0.
0048121705\C,0,1.2578714865,1.2421256571,-0.0766431407\C,0,0.455518767
3,2.4076934667,-0.6552699572\C,0,-0.8798679986,2.5482406475,0.09054577
84\C,0,-0.8815255465,-2.5493873946,-0.074447836\C,0,0.463303332,-2.406
2797956,0.6535603579\C,0,1.258251211,-1.2431535111,0.0599418678\H,0,-3
.5946982954,2.0774420509,0.0536051146\H,0,-3.5952930953,-2.0776431475,
-0.006466222\H,0,1.0471317542,3.3248048196,-0.5652363637\H,0,1.0534999
721,-3.3238932077,0.5594954492\H,0,0.2936137167,-2.2322263913,1.723813
7238\H,0,0.2719524587,2.2380055437,-1.7239345885\H,0,-0.6890779526,2.8
277123748,1.1374668711\H,0,-1.4922067347,3.3462669705,-0.3447129193\H,
0,-0.7040853504,-2.8338904126,-1.1223735305\H,0,-1.488801251,-3.344918
1145,0.3722409036\H,0,1.6275328041,1.48204408,0.9313023138\H,0,2.13192
7991,1.0325728941,-0.7046671723\H,0,2.14099728,-1.0320021487,0.6751091
911\H,0,1.6138025072,-1.486627553,-0.9522156275\C,0,-5.1551269941,-0.0
000700926,-0.0247521043\H,0,-5.4963524203,0.0073716993,-1.065100191\H,
0,-5.5329817442,-0.8920167562,0.479540874\H,0,-5.5336983338,0.88393910
89,0.4926502894\\Version=AM64L-G03RevD.01\State=1-A\HF=-573.1040818\MP
2=-575.1929934\RMSD=2.500e-09\Thermal=0.\PG=C01 [X(C12H17N2)]\\@

57-BH^{+}

1\1\GINC-NAUTILUS\SP\RMP2-FC\6-31+G(2d,p)\C24H25N2(1+)\CHRISTOPH\15-Ja
n-2011\0\\#p MP2(FC)/6-31+g(2d,p) scf=tight\\yin8bhsp_70\\1,1\C\C,1,1.
4329055\C,2,1.43594862,1,117.69707286\C,3,1.3722619,2,118.68091772,1,-
0.67315195,0\C,1,1.37603717,2,119.02585278,3,-0.42832304,0\H,4,1.08583
057,3,121.40097108,2,179.70593921,0\H,5,1.08422351,1,121.57988746,2,-1
79.84510587,0\N,2,1.34903393,1,121.24683618,5,179.61587948,0\C,3,1.513
07832,2,119.60113087,1,179.62662514,0\H,9,1.09602669,3,110.43553519,2,
153.36709122,0\H,9,1.10029823,3,109.61416363,2,-89.20675048,0\C,1,1.51
346466,5,121.42237721,4,-179.23980861,0\H,12,1.10027679,1,109.70294343
,5,90.75156874,0\H,12,1.09605485,1,110.40332855,5,-26.68212301,0\C,12,
1.53597267,1,109.34297814,5,-149.17546115,0\C,9,1.53587075,3,109.35614
3,2,30.82283396,0\C,8,1.47323314,2,122.42172423,1,175.12508982,0\C,8,1
.47355717,2,122.31247824,1,-4.94847041,0\H,16,1.09524355,9,110.7546241
1,3,-175.49723935,0\H,16,1.09766545,9,109.95037748,3,65.88458885,0\H,1
7,1.10041502,8,108.75213981,2,101.30598293,0\H,17,1.09681268,8,107.200
66853,2,-142.9412462,0\H,18,1.09680543,8,107.18160201,2,-142.80278057,
0\H,18,1.10036552,8,108.74301828,2,101.47696337,0\H,15,1.09524048,12,1
10.80076895,1,-175.47875918,0\H,15,1.09760282,12,109.92795342,1,65.881
02438,0\C,4,2.46537266,3,155.75681901,2,4.3369185,0\H,27,1.09674644,4,
83.28666919,3,-139.71693884,0\C,27,4.33425154,4,99.58875174,3,113.6906
6665,0\C,29,1.3977272,27,60.63125858,4,-61.99796488,0\C,30,1.39879446,
29,120.21180003,27,0.72334325,0\C,31,1.40272526,30,120.26163315,29,-0.
02625566,0\C,32,1.40314185,31,119.22560326,30,0.24168094,0\C,33,1.3966
5686,32,120.450275,31,-0.35196979,0\H,29,1.0869144,27,179.10282852,4,1
52.52781534,0\H,30,1.08719366,29,120.12477508,27,-179.34899416,0\H,31,
1.0883172,30,119.39259088,29,179.67230598,0\H,33,1.08877264,32,119.805
77636,31,-179.83674576,0\H,34,1.08698173,33,119.72351093,32,179.933712
97,0\C,27,4.33446264,4,138.29859493,3,-32.91430331,0\C,40,1.39954598,2
7,59.38430986,4,-65.89125011,0\C,41,1.39605939,40,119.94287686,27,-0.1
266592,0\C,42,1.40562316,41,120.53837621,40,0.06026485,0\C,43,1.403240

66,42,119.20863927,41,-0.40823786,0\C,40,1.39784748,27,60.48152808,4,1
14.41593833,0\H,40,1.08705019,27,179.3506075,4,-94.79575443,0\H,41,1.0
8702925,40,120.2027038,27,-179.45760525,0\H,42,1.0892474,41,119.669694
22,40,-179.49890293,0\H,44,1.08683293,43,119.99056105,42,-178.24527875
,0\H,45,1.08706467,40,120.12046683,27,179.47454262,0\N,5,1.35821536,1,
122.55215882,2,1.34213292,0\\Version=AM64L-G03RevD.01\State=1-A\HF=-10
32.2318752\MP2=-1036.0027931\RMSD=5.404e-09\Thermal=0.\PG=C01 [X(C24H2
5N2)]\\@

57-TT⁺

1\1\GINC-NAUTILUS\SP\RMP2-FC\6-31+G(2d,p)\C30H29N2(1+)\CHRISTOPH\01-No
v-2011\0\\#p MP2(FC)/6-31+g(2d,p) scf=tight\\yin8ttsp_12\\1,1\C\C,1,4.
37469901\C,2,1.39985838,1,58.50626293\C,3,1.39428005,2,120.27460562,1,
-0.06897977,0\C,4,1.40941198,3,121.0005684,2,1.07207061,0\C,5,1.401633
73,4,118.17380968,3,-2.05414425,0\C,2,1.39387468,1,60.76665169,5,18.89
937211,0\H,2,1.08686957,1,178.80544862,5,-175.23748277,0\H,3,1.0871055
4,2,120.2427837,1,-179.34955535,0\H,4,1.08725185,3,119.35028047,2,-178
.17696813,0\H,6,1.08560646,5,121.10369717,4,-177.16893767,0\H,7,1.0872
6195,2,120.21050799,1,179.20530281,0\C,1,4.37866443,5,114.7559326,4,-6
9.56574901,0\C,13,1.39975511,1,58.98925607,5,-169.92358145,0\C,14,1.39
476214,13,120.12979609,1,0.051015,0\C,15,1.41045003,14,121.10420313,13
,-0.61205011,0\C,16,1.40189122,15,118.16807788,14,1.45880153,0\C,13,1.
39460673,1,60.33587948,5,9.65121851,0\H,14,1.08730078,13,120.2453812,1
,179.9256375,0\H,15,1.08808814,14,118.99689607,13,179.42048946,0\H,17,
1.08428824,16,120.50112906,15,178.22901861,0\H,18,1.08713261,13,120.20
073163,1,-179.64208884,0\C,1,4.37824359,5,105.86396432,4,57.13491869,0
\C,23,1.39475202,1,60.3423702,5,-134.17300952,0\C,24,1.40103956,23,120
.5745049,1,-0.50213276,0\C,25,1.40270123,24,120.67493129,23,0.1838258,
0\C,26,1.41167909,25,118.15315415,24,1.10186229,0\C,27,1.39460129,26,1
21.08014816,25,-1.82972064,0\H,23,1.08702947,1,179.11237873,5,8.145976
23,0\H,24,1.08711199,23,120.19741834,1,179.01003416,0\H,25,1.08416433,
24,118.74087868,23,178.82051087,0\H,27,1.08766582,26,119.74629697,25,1
77.3232652,0\H,28,1.0872349,27,119.58991637,26,-179.87517172,0\H,13,1.
08696401,1,179.27693369,5,-171.17031003,0\C,1,4.35859695,5,108.3641855
4,4,177.53704521,0\C,35,1.43089361,1,59.69262355,5,-112.74904085,0\C,3
6,1.37632623,35,119.17701652,1,0.91791518,0\C,37,2.33862852,36,92.0440
922,35,-1.85986554,0\C,38,1.37294031,37,92.49694595,36,1.66531462,0\H,
37,1.08239595,36,120.79098958,35,179.40486083,0\H,38,1.08390539,37,146
.47213517,36,-177.24110565,0\N,35,1.34929251,1,178.90352519,5,48.51071
242,0\C,42,1.47310719,35,122.34676754,1,-156.15583242,0\H,43,1.0970309
,42,107.24742578,35,142.24518307,0\H,42,2.10424618,35,122.62526416,1,-
11.5612375,0\C,42,1.47268667,35,122.23245595,1,23.74676948,0\H,46,1.09
680249,42,107.22884268,35,143.79194967,0\C,39,1.51382729,38,121.456134
55,37,179.68694402,0\C,46,1.52952655,42,112.20585733,35,22.56619198,0\
C,43,1.5300072,42,112.33996313,35,20.91032333,0\C,36,1.51314969,35,119
.47982599,1,-179.26501102,0\H,50,1.09768401,43,109.69924518,42,70.1223
9825,0\H,50,1.09535227,43,108.85212498,42,-172.56362415,0\H,51,1.10036
145,36,109.81465847,35,88.4822733,0\H,51,1.09600063,36,110.43363386,35
,-153.95899218,0\H,49,1.09770108,46,109.66521487,42,69.10722717,0\H,49
,1.09532474,46,108.93403081,42,-173.54016896,0\H,48,1.10040205,39,109.
73853128,38,-90.31236678,0\H,48,1.0961038,39,110.32686186,38,27.128806
59,0\H,43,1.10046609,42,108.73234123,35,-102.05884268,0\N,37,1.3590896
5,36,122.9769549,35,-1.66849436,0\\Version=AM64L-G03RevD.01\State=1-A\
HF=-1261.769411\MP2=-1266.4055824\RMSD=9.713e-09\Thermal=0.\PG=C01 [X(
C30H29N2)]\\@

57-Ac⁺

see reference 15

58

1\1\GINC-CALYPSO\SP\RMP2-FC\6-31+G(2d,p)\C11H17N3\RAMAN\24-Oct-2011\0\
\#P MP2/6-31+G(2d,p) scf=(direct,tight) int=finegrid geom=check guess=
read\\opt of cat81et7 b98/6-31G(d) on 4p 900 MB\\0,1\C,0,-1.2541390147
,0.0294042914,0.8810025329\C,0,-0.5399591715,-1.2966797262,0.619729938
1\N,0,0.8789971733,-1.1380758963,0.8886173738\C,0,1.4986834817,-0.0239
127787,0.2998649794\C,0,0.708274021,1.1070697001,-0.0847401455\N,0,-0.
676810458,1.0679880838,0.04184771\C,0,1.3973699856,2.2131584353,-0.609
6962049\C,0,2.7860535249,2.1709553688,-0.7502910621\N,0,3.5349783195,1
.1224851163,-0.4067770575\C,0,2.8850614287,0.0611486677,0.096747727\C,
0,-1.4906651213,2.2553684924,-0.1901726424\C,0,-1.4873218478,3.2772512
442,0.9611905158\C,0,1.632138916,-2.3630489378,1.1331284602\C,0,1.9325
380061,-3.2157054492,-0.1141585489\H,0,-1.1865186965,0.271963577,1.954
8852556\H,0,-0.9371618193,-2.0637366162,1.2965905122\H,0,1.0460085372,
-2.9559372729,1.8485540035\H,0,2.56402404,-2.0996730844,1.6475831549\H
,0,1.0054689292,-3.5500504137,-0.5961530554\H,0,2.5136951945,-2.652053
459,-0.8526038337\H,0,2.5068382615,-4.1080587977,0.1655023863\H,0,-2.5
165043503,1.9077090257,-0.3711530111\H,0,-1.1609094616,2.7291923328,-1
.122420335\H,0,-1.8765690086,2.8307147453,1.8843261686\H,0,-0.47418941
89,3.6429457774,1.1633615207\H,0,-2.120185891,4.1376177856,0.709069612
\H,0,0.8659631514,3.1119213366,-0.9042172284\H,0,3.5191940523,-0.78250
57548,0.359452835\H,0,3.3131174366,3.032875839,-1.159299591\H,0,-0.746
0551688,-1.620204022,-0.4158375583\H,0,-2.3171047214,-0.0689528204,0.6
247984983\\Version=AM64L-G03RevD.01\State=1-A\HF=-589.827159\MP2=-591.
9812532\RMSD=7.365e-09\Thermal=0.\PG=C01 [X(C11H17N3)]\\@

58-Me⁺

1\1\GINC-EDDY\SP\RMP2-FC\6-31+G(2d,p)\C12H20N3(1+)\RAMAN\25-Oct-2011\0
\\#P MP2/6-31+G(2d,p) scf=(direct,tight) int=finegrid guess=read geom=
check\\sp of cat81et7.mca mp2-5 on 2p 900 MB\\1,1\C,0,-0.7404970248,-1
.986631002,0.1088414646\C,0,0.7412510273,-1.9522717444,-0.2522111809\N
,0,1.3611565644,-0.7695955677,0.3370464434\C,0,0.7114462452,0.43033846
32,0.1574269217\C,0,-0.7145387548,0.4381175093,-0.1382923316\N,0,-1.38
63370649,-0.7287489069,-0.2782935122\C,0,-1.3355849587,1.702422799,-0.
2670667968\C,0,-0.6213037541,2.8737418628,-0.1206239559\N,0,0.69865617
74,2.8505420409,0.1472417004\C,0,1.3551931142,1.6565017231,0.267362615
2\C,0,-2.8307692352,-0.7767420676,-0.5596568245\C,0,-3.7091251402,-0.6
691068973,0.6950784482\C,0,2.7989740066,-0.8324885407,0.6192943104\C,0
,3.7055615701,-0.6892170498,-0.6144286147\H,0,-0.8630654657,-2.1658537
924,1.1858244931\H,0,1.2322690697,-2.839416784,0.1605296734\H,0,2.9794
073132,-1.7997834017,1.1027304415\H,0,3.0322835739,-0.0711797371,1.373
7881153\H,0,3.5289723917,-1.500277084,-1.3301266799\H,0,3.5438734336,0
.2612896264,-1.1364931547\H,0,4.7575056562,-0.7357723106,-0.3103274284
\H,0,-3.0168422908,-1.7277051071,-1.0706899229\H,0,-3.0706590459,0.009
8861017,-1.2828157647\H,0,-3.5086775412,-1.4917204365,1.3908140318\H,0
,-3.5432544028,0.2752670282,1.2261996634\H,0,-4.7661299579,-0.71996930
77,0.4103860724\H,0,-2.3981498803,1.7840919531,-0.4546889658\H,0,2.417
917228,1.7335940744,0.4523928226\H,0,-1.0891957696,3.8476804803,-0.203
6247621\H,0,0.862313148,-1.9752378381,-1.3461799234\H,0,-1.2324494483,
-2.8021674448,-0.4311259229\C,0,1.468476857,4.0972386981,0.3059531366\
H,0,1.8990059195,4.1396522357,1.3107515424\H,0,2.2661701276,4.13521173
81,-0.4417660889\H,0,0.8016430919,4.9487063461,0.1630978837\\Version=A
M64L-G03RevD.01\State=1-A\HF=-629.2729568\MP2=-631.5741176\RMSD=5.988e
-09\Thermal=0.\PG=C01 [X(C12H20N3)]\\@

58-Ac⁺

see reference 9

59

1\1\GINC-AZAZEL\SP\RHF\6-31G(d)\C13H19N3\RAMAN\25-Jul-2011\0\\#P RHF/6
-31G(d) scf=tight int=finegrid SCRF=(PCM,Read,Solvent=Chloroform) geom
=check guess=read\\PCM of cat11me1 b98/6-31G(d) structures on 4p 900 M
B\\0,1\C,0,3.4729194535,0.840344011,-0.6790610748\C,0,2.772466803,-0.3
327174523,-0.3921984728\C,0,1.3916996575,-0.2779070905,-0.1376936539\C
,0,0.7596543834,1.0022322922,-0.2244021328\C,0,1.5862788687,2.10310479
54,-0.4969895549\N,0,2.9091573402,2.0472135917,-0.7292586409\N,0,-0.63
79674617,1.1143709828,-0.0588728965\C,0,-1.2484510422,0.0512635094,0.7
551660355\C,0,-0.7980836471,-1.3131169064,0.1790471746\N,0,0.656388947
8,-1.3973475462,0.2097153179\C,0,-1.3965529629,-1.5681115785,-1.223169
3918\C,0,-2.9336341676,-1.4871156332,-1.2065420521\C,0,-3.4142943994,-
0.1504884697,-0.6156634452\C,0,-2.7924151199,0.0837121333,0.7722165242
\C,0,-1.163143385,2.4652286208,0.0732093752\C,0,1.2694343111,-2.711543
2783,0.1729174999\H,0,-0.888618156,0.1112278507,1.7992315104\H,0,-1.17
47123529,-2.0850466768,0.8674541507\H,0,-1.0707084595,-2.554826874,-1.
5808042731\H,0,-0.9889444961,-0.8206231642,-1.9161139354\H,0,-3.327851
4888,-1.6217056237,-2.2225758766\H,0,-3.3369132617,-2.3149113139,-0.60
14939183\H,0,-4.5097447508,-0.1408961045,-0.535891176\H,0,-3.134666823
5,0.6687135943,-1.2924265752\H,0,-3.1621668914,1.0115912563,1.22441926
74\H,0,-3.1136192788,-0.721107587,1.4503740732\H,0,1.5909244562,-3.011
1988669,-0.8382998961\H,0,0.5436194095,-3.446473144,0.5349443444\H,0,-
2.25260287,2.440342277,0.1158816322\H,0,-0.8918713252,3.0539847687,-0.
8095509416\H,0,1.1622914296,3.1025946927,-0.5360863546\H,0,4.544359210
5,0.7954145973,-0.8748532912\H,0,3.3039596261,-1.2783190229,-0.3688494
167\H,0,2.1447738226,-2.7490609414,0.8344063525\H,0,-0.7869464794,2.99
27143111,0.9682135624\\Version=AM64L-G03RevD.01\State=1-A\HF=-666.6837
551\RMSD=5.414e-09\Thermal=0.\Dipole=-1.5492693,-1.4501222,0.5277208\P
G=C01 [X(C13H19N3)]\\@

59-Me$^+$

1\1\GINC-GOLEM\SP\RHF\6-31G(d)\C14H22N3(1+)\RAMAN\07-Mar-2012\0\\#P RH
F/6-31G(d) scf=tight int=finegrid SCRF=(PCM,Read,Solvent=Chloroform) g
eom=check guess=read\\PCM of cat11me1.mca b98/6-31G(d) structures on 2
p 512 MB\\1,1\C,0,-0.0581617785,-0.0708390203,-0.0002564762\C,0,-0.025
8983477,-0.0176156367,1.3771221843\C,0,1.2021168342,0.0470573635,2.079
3821892\C,0,2.4211202812,0.0560101836,1.2895384757\C,0,2.2952931989,-0
.0289738446,-0.0896281236\N,0,1.0804400852,-0.0729081295,-0.7211525402
\N,0,3.6448265739,0.1539368909,1.925141934\C,0,3.6744046252,-0.4037966
474,3.2983082594\C,0,2.5520121036,0.2719602826,4.1126929653\N,0,1.2615
148501,0.1029448958,3.4239336527\C,0,2.8516205709,1.7549050392,4.42027
51417\C,0,4.1970597754,1.9100657217,5.152486107\C,0,5.3386884045,1.261
2389122,4.3525177818\C,0,5.0137635282,-0.2096739225,4.0367059702\C,0,4
.840828551,0.0998457531,1.0912348666\C,0,0.0396962315,0.1282725936,4.2
28310701\H,0,3.4633256439,-1.4854220093,3.2491809181\H,0,2.4707293308,
-0.2829568234,5.0570670093\H,0,2.0346833238,2.1730563157,5.0231084243\
H,0,2.8734893066,2.3118144491,3.4738121396\H,0,4.401570534,2.974080093
,5.3212188905\H,0,4.1323231411,1.4414261251,6.1460564744\H,0,6.2764333
814,1.3112543303,4.9191216985\H,0,5.5002753914,1.8275814905,3.42409263
96\H,0,5.8286564634,-0.6992465952,3.4913285815\H,0,4.9220447445,-0.766
2458396,4.9804264812\H,0,-0.5670911412,1.017006904,4.0125756409\H,0,0.
3182111325,0.1494811137,5.2835592586\H,0,5.7212128414,0.3286129749,1.6
895949991\H,0,4.7748634716,0.8673752456,0.3117712456\H,0,3.1529344714,
-0.0639650877,-0.7469935556\H,0,-0.989298573,-0.1093453398,-0.55366914
22\H,0,-0.9694153533,-0.0116817966,1.9077144155\H,0,-0.5621049731,-0.7
712829256,4.0494502823\H,0,4.9842280212,-0.8838300414,0.6141369229\C,0
,1.0548837356,-0.1356618677,-2.1931165469\H,0,1.5303278452,0.757522170
5,-2.6091504839\H,0,1.58510832,-1.0303169172,-2.5330654242\H,0,0.01856

90319,-0.1827912095,-2.5307850507\\Version=AM64L-G03RevD.01\State=1-A\
HF=-706.1699643\RMSD=3.375e-09\Thermal=0.\Dipole=-1.5600252,-0.6310726
,-2.036478\PG=C01 [X(C14H22N3)]\\@

60
see reference 4

60-Me⁺
see reference 4

60-BH⁺
1\1\GINC-NODE4\SP\RMP2-FC\6-31+G(2d,p)\C22H27N2(1+)\ZIP07\15-Jan-2011\
0\\#p MP2(FC)/6-31+G(2d,p) scf=tight\\yin3bhsp_2\\1,1\C\C,1,1.54031719
\N,2,1.48547344,1,114.41490543\C,3,1.34023076,2,121.83931919,1,62.2161
6448,0\C,4,1.51580198,3,117.82542433,2,8.84284504,0\C,5,1.552736,4,113
.41879474,3,-75.38903687,0\C,6,1.53706723,5,113.11831747,4,81.41192691
,0\H,1,1.09947749,7,110.85904412,6,-64.53089635,0\H,1,1.09769789,7,108
.49123228,6,178.95540089,0\H,2,1.09781589,1,111.18303733,7,43.54089142
,0\H,2,1.09266428,1,109.36784019,7,160.92944427,0\H,5,1.09732309,4,107
.70268283,3,45.99646592,0\H,5,1.09010544,4,109.32813502,3,159.94971237
,0\H,6,1.09641756,5,107.7292584,4,-158.04634179,0\H,6,1.09965275,5,109
.73936323,4,-42.4753331,0\H,7,1.09695957,6,109.10323137,5,-179.3114420
2,0\H,7,1.10029718,6,108.59310402,5,65.2594264,0\C,3,1.47612905,2,116.
14599599,1,-112.76611725,0\H,18,1.09428824,3,107.48799662,2,-37.776055
65,0\H,18,1.09899029,3,109.36494458,2,79.06845425,0\C,4,2.46140223,3,9
1.23661909,2,-173.74654939,0\C,21,1.52241103,4,85.03230364,3,-26.70571
96,0\H,22,1.09749391,21,110.50445748,4,-79.76173054,0\H,22,1.09483009,
21,109.6033765,4,161.77754471,0\H,21,1.0963422,4,110.94068598,3,84.296
78119,0\H,21,1.09275492,4,128.90363264,3,-139.84660489,0\C,4,2.4868551
1,3,152.61206984,2,-163.1400735,0\H,27,1.09380803,4,82.31612205,3,135.
80594401,0\C,27,4.34214291,4,109.2389895,3,-118.68986172,0\C,29,1.3999
1866,27,58.87628625,4,-134.43987578,0\C,30,1.39544924,29,120.14508604,
27,0.09207697,0\C,31,1.40591088,30,120.57811497,29,0.11586051,0\C,32,1
.40096105,31,118.93258716,30,0.13599583,0\C,29,1.39608319,27,60.769495
98,4,45.29209366,0\H,29,1.08685866,27,179.03388677,4,-141.3498868,0\H,
30,1.08701876,29,120.17390825,27,-179.98932435,0\H,31,1.08877311,30,11
9.70430453,29,-179.32221295,0\H,33,1.08766072,32,120.51912296,31,179.2
6151529,0\H,34,1.08718705,29,120.14956752,27,179.81904425,0\C,27,4.342
56643,4,130.80680077,3,33.59565134,0\C,40,1.39776147,27,60.4721609,4,-
124.13229793,0\C,41,1.39862101,40,120.26037968,27,-0.1029557,0\C,42,1.
40245137,41,120.24750089,40,0.11912863,0\C,43,1.40484378,42,119.139920
31,41,-0.55200811,0\C,44,1.39648968,43,120.60473382,42,0.6284425,0\H,4
0,1.0869981,27,179.3675493,4,78.60817302,0\H,41,1.08704325,40,120.1098
6879,27,-179.64583028,0\H,42,1.0872851,41,119.78289531,40,-178.810382,
0\H,44,1.08987364,43,119.82078561,42,-178.8971136,0\H,45,1.08710983,44
,119.89121503,43,-179.55566527,0\N,4,1.33880561,3,121.94381002,2,-170.
89669067,0\\Version=AM64L-G03RevD.01\State=1-A\HF=-957.6361361\MP2=-96
1.1645526\RMSD=3.737e-09\Thermal=0.\PG=C01 [X(C22H27N2)]\\@

60-TT⁺
1\1\GINC-PHOBOS\SP\RMP2-FC\6-31+G(2d,p)\C28H31N2(1+)\CHRISTOPH\27-Oct-
2011\0\\#p MP2(FC)/6-31+g(2d,p) scf=tight\\yin3ttsp_1\\1,1\C\C,1,4.387
38254\C,2,1.39891676,1,58.68276862\C,3,1.39654298,2,120.11007339,1,-0.
57406617,0\C,4,1.40901206,3,121.4124031,2,0.55107254,0\C,5,1.40292326,
4,117.76618853,3,-0.85094052,0\C,2,1.39540597,1,60.47422728,5,-13.2862
0365,0\H,2,1.08686528,1,178.94363783,5,-165.69994344,0\H,3,1.08740201,
2,120.25713726,1,179.24361063,0\H,4,1.08786156,3,118.68301138,2,179.53
577962,0\H,6,1.08482556,5,120.50639957,4,-178.4449228,0\H,7,1.08723527

,2,120.17403416,1,-179.80770201,0\C,1,2.54681064,5,92.99817405,4,70.90
109263,0\N,13,1.34457277,1,152.29583054,5,-124.8165162,0\C,1,4.3833109
6,5,116.42991658,4,173.76377207,0\C,15,1.39904265,1,58.61565852,5,56.3
0823856,0\C,16,1.39501363,15,120.35561541,1,-0.72060761,0\C,17,1.40953
422,16,121.14676535,15,-1.56874902,0\C,18,1.4026323,17,117.74938012,16
,3.24374888,0\C,15,1.39438861,1,60.54307428,18,-52.40166431,0\H,15,1.0
8676492,1,178.58888111,18,174.35522288,0\H,16,1.08706159,15,120.229550
21,1,178.1559716,0\H,17,1.08667536,16,119.09720831,15,176.5368408,0\H,
19,1.08608622,18,120.81089668,17,175.84192588,0\H,20,1.08731924,15,120
.22706279,1,-178.16254082,0\C,1,4.39135538,18,102.609442,17,-60.855470
93,0\C,26,1.4003484,1,59.00815795,18,-48.23242247,0\C,27,1.39326271,26
,120.20894221,1,-0.02390274,0\C,28,1.41153879,27,121.14955793,26,-1.32
477699,0\C,29,1.40328544,28,118.02118231,27,1.95284887,0\C,26,1.393947
54,1,60.27140693,18,131.74022682,0\H,26,1.08697418,1,179.03284142,18,-
2.26327768,0\H,27,1.08719061,26,120.23187661,1,178.91924874,0\H,28,1.0
8712631,27,118.97191479,26,177.91796316,0\H,30,1.08397224,29,120.70521
181,28,177.72296454,0\H,31,1.08716102,26,120.23242088,1,-178.81814691,
0\C,13,2.4353322,1,63.06772818,18,122.56025833,0\C,37,1.52257774,13,85
.92469636,1,149.51422941,0\H,37,1.09812508,13,106.86330264,1,-99.97523
145,0\H,37,1.08826282,13,133.08315494,1,35.28289708,0\H,38,1.09750284,
37,110.12323175,13,-78.65427587,0\H,38,1.09490773,37,109.57672003,13,1
63.01998225,0\C,14,1.47448068,13,123.81279124,1,12.93830369,0\H,43,1.0
9592267,14,107.3629299,13,137.48193239,0\H,43,1.09856584,14,108.985780
09,13,-106.04919088,0\C,13,1.51817822,1,91.81221533,18,-60.34920581,0\
H,46,1.08353698,13,109.91899175,1,6.60939246,0\H,46,1.09768304,13,108.
45532012,1,121.55992529,0\C,14,1.48545433,13,122.48891729,1,-170.39119
771,0\H,49,1.09402807,14,105.19936933,13,162.22863143,0\H,49,1.0955056
,14,109.37725242,13,48.36084009,0\C,46,1.55551184,13,112.99210499,1,-1
16.14448472,0\C,49,1.53713389,14,113.99252573,13,-76.63092478,0\H,53,1
.09773606,49,107.8745087,14,-159.82208318,0\H,53,1.09975133,49,108.895
42422,14,-44.55452819,0\C,53,1.53637308,49,113.22574699,14,79.13622926
,0\H,52,1.0975228,46,109.54876944,13,39.07577806,0\H,52,1.09644214,46,
106.8452265,13,153.5139642,0\H,56,1.10054076,53,108.76190356,49,67.000
1241,0\H,56,1.09717533,53,109.1945426,49,-177.46616407,0\N,13,1.346585
67,1,30.73351091,18,122.16582711,0\\Version=AM64L-G03RevD.01\State=1-A
\HF=-1187.1607399\MP2=-1191.5629104\RMSD=8.767e-09\Thermal=0.\PG=C01 [
X(C28H31N2)]\\@

61

1\1\GINC-AZAZEL\SP\RHF\6-31G(d)\C11H15N3\RAMAN\18-Jul-2011\0\\#P RHF/6
-31G(d) scf=tight int=finegrid SCRF=(PCM,Read,Solvent=Chloroform) geom
=check guess=read\\PCM of rtcapme14 b98/6-31G(d) structures on 4p 900
MB\\0,1\C,0,-1.0568865323,1.8141250692,-0.2781054635\C,0,-0.7604108446
,0.4459871739,-0.2784248701\C,0,0.5917016568,0.0627001811,-0.039985799
3\C,0,1.5412870115,1.0851917064,0.1623051467\C,0,1.1091390923,2.413092
266,0.0963442635\N,0,-0.1529731097,2.7938279419,-0.1119768999\N,0,0.94
88921333,-1.2720788095,-0.0157995246\C,0,2.3368324573,-1.6984508313,0.
1256328928\C,0,3.3200129724,-0.6037721622,-0.3036561238\C,0,-1.4366283
955,-1.7971017972,0.2163215232\C,0,-0.0509377478,-2.3127739847,-0.1734
652979\H,0,1.8391751932,3.2142017632,0.2275202445\H,0,-2.0810632156,2.
1464406136,-0.4332250663\H,0,2.4835079007,-2.5943068896,-0.4948700774\
H,0,2.5380745061,-1.9960828978,1.1703425862\H,0,0.2130679256,-3.161356
4298,0.4759640718\H,0,-0.0747719506,-2.6829708538,-1.211855156\H,0,4.3
42118627,-0.9318067882,-0.0772590692\H,0,-2.1867051083,-2.5474520438,-
0.059001998\H,0,-1.487990688,-1.6537223708,1.3120232213\H,0,3.25049953
63,-0.4588513909,-1.390045444\N,0,-1.7160231027,-0.5648493936,-0.51112
2792\C,0,-3.115111422,-0.175617111,-0.5558821721\H,0,-3.2747569086,0.5
577017337,-1.3539829398\H,0,-3.7175052347,-1.0605703201,-0.7874877011\

C,0,2.987580609,0.7147781071,0.4125341902\H,0,3.6483591549,1.520623123
3,0.0679949685\H,0,3.1750564925,0.5963685461,1.4918776124\H,0,-3.48075
57184,0.2594902785,0.3924086841\\Version=AM64L-G03RevD.01\State=1-A\HF
=-588.6235353\RMSD=7.070e-09\Thermal=0.\Dipole=0.3128376,-2.1353997,0.
2504223\PG=C01 [X(C11H15N3)]\\@

61-Me$^+$

1\1\GINC-CALYPSO\SP\RHF\6-31G(d)\C12H18N3(1+)\RAMAN\26-Mar-2012\0\\#P
RHF/6-31G(d) scf=tight int=finegrid SCRF=(PCM,Read,Solvent=Chloroform)
geom=check guess=read\\PCM of rtcapme2.mca b98/6-31G(d) structures on
2p 512 MB\\1,1\N,0,0.0265489442,-0.1252374783,0.0416248692\N,0,-0.022
0529664,0.0312444104,4.1886170293\C,0,1.2941154087,-0.010076764,-0.679
657257\C,0,2.2398374124,0.9382469106,0.0532621172\C,0,1.2055862846,0.2
664394149,2.1289959245\C,0,1.1275250683,0.3263688755,3.513066624\C,0,-
1.1415265674,-0.3061910491,3.5136783912\C,0,-1.1702073366,-0.381388005
,2.1339671819\C,0,-2.4527784465,-0.1827264948,-0.0035740537\C,0,-1.155
5594295,-0.5293187132,-0.7362100306\C,0,0.0068655888,-0.0854817875,1.3
951444431\H,0,3.2250660512,0.8961046299,-0.4207995935\H,0,-1.102387912
4,-1.608899645,-0.94485223\H,0,-1.1065157512,-0.0061731134,-1.69788776
35\H,0,-2.0196377896,-0.5247825508,4.1117188455\H,0,1.9710425368,0.607
2033463,4.1293260713\H,0,1.7595755304,-0.9995954851,-0.7916349432\H,0,
1.0829095335,0.3816018956,-1.6806228773\H,0,1.8711947247,1.9740712164,
-0.0195728655\H,0,-2.5618981021,0.9076421112,0.0545767827\H,0,-3.30094
97194,-0.5686220471,-0.5787875393\N,0,2.377504303,0.5095203158,1.44245
7168\C,0,3.5611629494,0.9456966429,2.170804492\H,0,3.8189054938,0.2131
849089,2.94478236\H,0,4.4003858743,1.0006374801,1.4732203515\C,0,-2.43
73565644,-0.7875150539,1.4070416258\H,0,-2.4873480933,-1.8842111913,1.
3388991027\H,0,-3.3177068918,-0.4664279654,1.9760119938\H,0,3.43163635
45,1.9343223528,2.6406812775\C,0,-0.0101021326,0.0903546224,5.66117750
99\H,0,0.6756503386,-0.6650966956,6.0566361869\H,0,0.30711403,1.085407
2619,5.9864983616\H,0,-1.016351263,-0.1047278404,6.0346072798\\Version
=AM64L-G03RevD.01\State=1-A\HF=-628.1108822\RMSD=3.271e-09\Thermal=0.\
Dipole=-0.1375805,0.0803158,1.0637019\PG=C01 [X(C12H18N3)]\\@

62

1\1\GINC-GOLEM\SP\RHF\6-31G(d)\C12H17N3\RAMAN\05-Jul-2011\0\\#P RHF/6-
31G(d) scf=tight int=finegrid SCRF=(PCM,Read,Solvent=Chloroform) geom=
check guess=read\\PCM of rtcapet2 b98/6-31G(d) structures on 4p 900 MB
\\0,1\N,0,0.0396874564,1.5831121674,-0.0228410965\N,0,0.0019646878,-2.
6384204891,-0.1312161181\C,0,1.2253189391,2.2569324785,-0.5199811841\C
,0,2.4712515552,1.5387425036,-0.0015828335\C,0,1.2351199939,-0.5425063
419,-0.2432830487\C,0,1.1436282301,-1.9437219377,-0.245335725\C,0,-1.1
222301766,-1.934288415,0.0005443953\C,0,-1.1845285217,-0.5361773072,0.
0201499436\C,0,-2.3172391072,1.617304106,0.670567557\C,0,-1.2166094952
,2.3134807232,-0.1357421576\C,0,0.0177091718,0.1884101328,-0.091209315
6\H,0,3.3670654017,2.0108779497,-0.425010121\H,0,-1.5307369083,2.40883
93555,-1.1928792069\H,0,-1.0452366487,3.3298948315,0.2433606556\H,0,-2
.0446895846,-2.5094411726,0.0990908477\H,0,2.0429703752,-2.5470033012,
-0.3453146319\H,0,1.2516137471,2.2925243286,-1.6237758536\H,0,1.215782
5233,3.2910095883,-0.1509737449\H,0,2.5194134736,1.6495056192,1.096074
6867\H,0,-2.0240415121,1.5984750086,1.7286005134\H,0,-3.2489227054,2.1
914815599,0.593698078\N,0,2.443817242,0.148351326,-0.4260053905\C,0,3.
7168500256,-0.5628992476,-0.3845428026\H,0,3.651929792,-1.4322147832,-
1.0498615393\H,0,4.466232419,0.1055599087,-0.829727047\C,0,4.183923691
6,-0.9976911001,1.0172692998\H,0,5.1410808457,-1.5304122478,0.95081847
33\H,0,4.3266518052,-0.1297144098,1.6727883927\H,0,3.4537567995,-1.663
3195414,1.4910158008\C,0,-2.51327749,0.1856021302,0.1515776173\H,0,-3.
0171740848,0.2217931723,-0.8271244391\H,0,-3.1795588312,-0.3773600253,

0.8185017849\\Version=AM64L-G03RevD.01\State=1-A\HF=-627.6591355\RMSD=
6.211e-09\Thermal=0.\Dipole=0.310909,2.0282089,0.0256046\PG=C01 [X(C12
H17N3)]\\@

62-Me$^+$
1\1\GINC-AZAZEL\SP\RHF\6-31G(d)\C13H20N3(1+)\RAMAN\26-Mar-2012\0\\#P R
HF/6-31G(d) scf=tight int=finegrid SCRF=(PCM,Read,Solvent=Chloroform)
geom=check guess=read\\PCM of rtcapet2.mca b98/6-31G(d) structures on
2p 512 MB\\1,1\N,0,0.0258587823,-0.137100556,0.0436360693\N,0,-0.03569
82619,0.0264033427,4.1902889954\C,0,1.2915094214,0.0200425389,-0.67151
66356\C,0,2.170453884,1.0485622798,0.0349200403\C,0,1.1735499696,0.359
1977858,2.1296832502\C,0,1.0900918143,0.4020843841,3.5156228547\C,0,-1
.1330574196,-0.3768704327,3.5163503557\C,0,-1.1552267791,-0.4502999024
,2.1361753903\C,0,-2.4443964149,-0.3466332591,-0.0041466506\C,0,-1.127
3149854,-0.6196659424,-0.7319692246\C,0,0.0006775347,-0.0814908266,1.3
980065032\H,0,3.1627361681,1.0518689251,-0.427588824\H,0,-1.008903831,
-1.6960732092,-0.9310444824\H,0,-1.1082130515,-0.1031727516,-1.6984301
981\H,0,-1.9954002734,-0.6518882156,4.1138262707\H,0,1.9104426852,0.73
77833801,4.135140708\H,0,1.8139200352,-0.9448411889,-0.7408542367\H,0,
1.0694759303,0.3599839672,-1.6888932336\H,0,1.7388084056,2.0547065694,
-0.0827480038\H,0,-2.6189831358,0.7356200191,0.0462381253\H,0,-3.26701
16577,-0.7868232115,-0.5775803855\N,0,2.3222937329,0.6829406387,1.4399
214804\C,0,3.5204970558,1.1678661267,2.1327477503\H,0,3.6912414771,0.5
295488111,3.0084171854\H,0,4.3657243511,0.9848733777,1.4590203951\C,0,
3.4806661813,2.6536512768,2.5273358607\H,0,4.409164989,2.9280141099,3.
0410334761\H,0,3.3866093248,3.2934962896,1.6424787071\H,0,2.64195043,2
.8792122127,3.1964622641\C,0,-2.3934546289,-0.9394740656,1.4103009837\
H,0,-2.3756108972,-2.0376180246,1.3500995991\H,0,-3.2928007567,-0.6695
242142,1.9762545353\C,0,-0.0293776627,0.0919257452,5.6625361287\H,0,0.
7548609419,-0.5609907736,6.0567472383\H,0,0.1481481259,1.1219402274,5.
9860959512\H,0,-0.9975421369,-0.2412798198,6.0389253724\\Version=AM64L
-G03RevD.01\State=1-A\HF=-667.1449724\RMSD=3.346e-09\Thermal=0.\Dipole
=-0.4718511,-0.24971,0.9813392\PG=C01 [X(C13H20N3)]\\@

63
see reference 30

63-Me$^+$
1\1\GINC-EDDY\SP\RMP2-FC\6-31+G(2d,p)\C16H26N3(1+)\RAMAN\06-Aug-2011\0
\\#P MP2/6-31+G(2d,p) scf=(direct,tight) int=finegrid guess=read geom=
check\\sp of cat11_1.mca mp2-5 on 2p 900 MB\\1,1\C,0,3.5583861294,0.82
57649773,-0.6577534457\C,0,2.8402078358,-0.3164233271,-0.3797513001\C,
0,1.4362600595,-0.2783207889,-0.1875183622\C,0,0.7950662533,1.02857836
4,-0.2318302473\C,0,1.6025335414,2.1263981172,-0.4993969358\N,0,2.9483
366623,2.0250602972,-0.730919903\N,0,-0.5701111003,1.1300362122,-0.030
864497\C,0,-1.1749083392,0.0358902972,0.7641096446\C,0,-0.7630808133,-
1.3079440256,0.1316843711\N,0,0.7014301553,-1.3887721348,0.0201102668\
C,0,-1.4542576332,-1.5605119239,-1.225545936\C,0,-2.9857902615,-1.5075
202595,-1.0842934597\C,0,-3.4345311963,-0.168892497,-0.4762697449\C,0,
-2.7143293917,0.0950483021,0.8582236062\C,0,-1.184270947,2.4651138572,
0.0112846813\C,0,-0.9809403102,3.2365843214,1.3264884873\C,0,1.3038740
696,-2.7297805618,0.1119273249\C,0,1.8567640172,-3.0415131588,1.509524
0338\H,0,-0.7688402533,0.0693851468,1.7890271875\H,0,-1.061577843,-2.0
943972579,0.8386752512\H,0,-1.1372288735,-2.5346004018,-1.6205947943\H
,0,-1.1154121694,-0.7974638164,-1.9390628961\H,0,-3.4511970008,-1.6595
787182,-2.0655131432\H,0,-3.3253741733,-2.3352578749,-0.4434957425\H,0
,-4.5181803265,-0.171265224,-0.3066949128\H,0,-3.2295289349,0.64007664
53,-1.1917968918\H,0,-3.0374756822,1.0400575318,1.3096866506\H,0,-3.00

20420037,-0.6848682667,1.5779298069\H,0,2.0774575294,-2.8338410028,-0.
6572049891\H,0,0.5178611304,-3.4446268689,-0.1513279375\H,0,2.64845019
54,-2.3406603074,1.7976480953\H,0,1.0639021818,-2.9873722952,2.2646109
274\H,0,2.2735047715,-4.0548973423,1.5269820472\H,0,-2.2515572226,2.34
30836192,-0.1842666204\H,0,-0.8014725026,3.0342952809,-0.8440481104\H,
0,-1.4439179501,2.7100582799,2.1691232096\H,0,0.0790229611,3.380052284
,1.5663730157\H,0,-1.4493292875,4.2250026614,1.2530816551\H,0,1.213139
6914,3.133918516,-0.5370887145\H,0,4.628490128,0.8069518274,-0.8285441
627\H,0,3.3825356828,-1.2514110818,-0.3293730424\C,0,3.7059714687,3.25
33226192,-1.0255880368\H,0,3.3093293554,3.723316745,-1.9303473653\H,0,
3.6284446691,3.9475563979,-0.1832626968\H,0,4.7544417071,2.9968081354,
-1.1841408738\\Version=AM64L-G03RevD.01\State=1-A\HF=-784.2660631\MP2=
-787.1733766\RMSD=6.915e-09\Thermal=0.\PG=C01 [X(C16H26N3)]\\@

63-MOSC_si

1\1\GINC-NODE5\SP\RMP2-FC\6-31+G(2d,p)\C24H31F3N3O1(1+)\ZIP01\23-Dec-2
008\0\\#P MP2/6-31+G(2d,p) scf=(direct,tight) int=finegrid geom=check
guess=read\\sp of cat1_mosc_a.001.sp MP2/6-31+G(2d,p) on 4p 900 MB\\1,
1\C,-0.635377,2.095913,-0.664901\C,0.722962,2.302308,-0.712162\C,1.646
024,1.275523,-0.384547\C,1.091478,0.007364,0.056041\C,-0.287776,-0.118
184,0.073744\N,-1.135455,0.891405,-0.301409\N,1.942771,-1.030373,0.414
282\C,3.277696,-0.598706,0.886097\C,3.892969,0.324277,-0.184392\N,2.97
9832,1.439391,-0.479266\C,4.291683,-0.440586,-1.464766\C,5.275287,-1.5
8014,-1.143673\C,4.686342,-2.532882,-0.091036\C,4.25712,-1.757881,1.16
7903\C,1.354154,-2.252824,0.981438\C,0.879335,-2.135561,2.440592\C,3.5
93745,2.710994,-0.897315\C,3.725869,3.719773,0.252039\H,3.164516,-0.01
3873,1.814903\H,4.792183,0.772322,0.261164\H,4.735416,0.258534,-2.1858
56\H,3.382225,-0.847516,-1.926567\H,5.51921,-2.126012,-2.063072\H,6.21
9784,-1.156881,-0.769347\H,5.423584,-3.295491,0.188343\H,3.828593,-3.0
66197,-0.524658\H,3.850964,-2.428548,1.933847\H,5.14626,-1.298695,1.62
3597\H,3.0235,3.129973,-1.733882\H,4.582293,2.464413,-1.297377\H,2.748
794,3.992089,0.666644\H,4.337267,3.309428,1.064128\H,4.211069,4.633407
,-0.109828\H,2.099217,-3.046813,0.897274\H,0.533007,-2.563013,0.324509
\H,1.716457,-1.909563,3.111581\H,0.124463,-1.351834,2.572082\H,0.44051
4,-3.086663,2.764478\H,-0.776127,-1.029101,0.380202\H,-1.354236,2.8606
61,-0.920114\H,1.067787,3.281278,-1.018517\C,-2.629377,0.696942,-0.160
232\C,-2.9633,1.103457,1.320695\O,-3.164907,1.635381,-1.041466\F,-4.25
7255,0.904202,1.601567\F,-2.688689,2.398639,1.507881\F,-2.234132,0.392
377,2.202349\C,-3.01824,-0.738309,-0.530082\C,-3.466644,-1.697183,0.39
1448\C,-2.906926,-1.085347,-1.889042\C,-3.797871,-2.984926,-0.046391\H
,-3.570824,-1.460168,1.444271\C,-3.237147,-2.370212,-2.318129\H,-2.569
814,-0.340699,-2.605818\C,-3.683776,-3.324421,-1.395915\H,-4.151893,-3
.717825,0.673919\H,-3.151246,-2.625483,-3.371168\H,-3.945956,-4.324817
,-1.730587\C,-4.594659,1.744935,-1.14996\H,-4.765989,2.280542,-2.08537
8\H,-5.065228,0.757748,-1.197638\H,-5.007339,2.319877,-0.315555\\Versi
on=AM64L-G03RevD.01\State=1-A\HF=-1463.3370538\MP2=-1468.196958\RMSD=6
.194e-09\Thermal=0.\PG=C01 [X(C24H31F3N3O1)]\\@

64

1\1\GINC-AZAZEL\SP\RHF\6-31G(d)\C17H19N3\RAMAN\19-Jul-2011\0\\#P RHF/6
-31G(d) scf=tight int=finegrid SCRF=(PCM,Read,Solvent=Chloroform) geom
=check guess=read\\PCM of rtcapbn5 b98/6-31G(d) structures on 4p 900 M
B\\0,1\N,0,2.4030517794,-1.1320390627,0.0663092698\N,0,0.5868773626,2.
6453837852,-0.4501444789\C,0,3.7046885982,-1.2175148332,0.716231331\C,
0,4.6187331151,-0.0873030922,0.2319635581\C,0,2.524840139,1.3113178722
,0.1175869797\C,0,1.8683173335,2.5275822075,-0.1013775745\C,0,-0.10594
39331,1.5074012345,-0.6079571977\C,0,0.4261252744,0.2200930627,-0.4415
437922\C,0,0.3931796969,-2.1815064053,-0.8147070421\C,0,1.5288875085,-

2.2821688338,0.2025247257\C,0,1.7986405798,0.1188480165,-0.0688849848\
H,0,5.6016066539,-0.1773212039,0.7109698899\H,0,1.1070741006,-2.367145
0762,1.2204383595\H,0,2.1159035144,-3.1891665845,0.0070442874\H,0,-1.1
552756344,1.6299758442,-0.8639462086\H,0,2.4235825826,3.4595877971,0.0
200505407\H,0,3.6062978477,-1.1748512034,1.8178407315\H,0,4.1369433404
,-2.1957582033,0.4673020588\H,0,4.7653479612,-0.190923144,-0.851289504
2\H,0,0.8140462068,-2.246081611,-1.8342712018\H,0,-0.2908770237,-3.027
4628932,-0.6770606421\N,0,-0.349178738,-0.9463319278,-0.5988438854\C,0
,-1.6390787783,-0.8447939466,-1.2712850984\H,0,-1.8844938656,-1.850138
2589,-1.6396066133\H,0,-1.5710137611,-0.2000686762,-2.1645445752\C,0,3
.979189085,1.2721476007,0.5503940704\H,0,4.5366941053,2.0822664782,0.0
618977522\H,0,4.0500357514,1.460078277,1.6332802267\C,0,-2.7882570701,
-0.3660617239,-0.3864038264\C,0,-3.9080222766,0.240638023,-0.974083008
8\C,0,-2.7781828823,-0.5694562422,0.9994937042\C,0,-5.0010295541,0.628
9178386,-0.1942776251\H,0,-3.923483504,0.4142871656,-2.0497354222\C,0,
-3.8679817953,-0.1768940229,1.7821499684\H,0,-1.9030702138,-1.02214958
81,1.459186983\C,0,-4.9842287497,0.4205126131,1.188445967\H,0,-5.86030
07699,1.1014791704,-0.6654113283\H,0,-3.8429861317,-0.3345680213,2.858
4614599\H,0,-5.830541734,0.728313955,1.7985259404\\Version=AM64L-G03Re
vD.01\State=1-A\HF=-818.1692826\RMSD=1.809e-09\Thermal=0.\Dipole=0.763
5159,-1.9799971,0.1391064\PG=C01 [X(C17H19N3)]\\@

64-Me$^+$

1\1\GINC-AZAZEL\SP\RHF\6-31G(d)\C18H22N3(1+)\RAMAN\26-Mar-2012\0\\#P R
HF/6-31G(d) scf=tight int=finegrid SCRF=(PCM,Read,Solvent=Chloroform)
geom=check guess=read\\PCM of rtcapbn5.mca b98/6-31G(d) structures on
2p 512 MB\\1,1\N,0,0.0272598876,-0.125864816,0.0306128935\N,0,-0.11263
75294,-0.0186717027,4.1755546456\C,0,1.2893085951,0.0299078129,-0.7079
609847\C,0,2.2740634386,0.9183881065,0.0539837392\C,0,1.1519615588,0.1
596812233,2.1580342852\C,0,1.0574514549,0.1933179697,3.5356954109\C,0,
-1.2554373584,-0.2503825656,3.4678989043\C,0,-1.2662641821,-0.30270088
91,2.0811763933\C,0,-2.4230228721,-0.1246381223,-0.0315367066\C,0,-1.1
407227249,-0.5774533247,-0.723033693\C,0,-0.0152442747,-0.0885258833,1
.385067677\H,0,3.2222121711,0.9528286733,-0.492941873\H,0,-1.129676122
6,-1.6720523179,-0.8277191223\H,0,-1.0909050025,-0.140696446,-1.726436
847\H,0,-2.1440793502,-0.4293879935,4.0577182268\H,0,1.9244936719,0.37
95047685,4.1602493912\H,0,1.7277780574,-0.9620570606,-0.8979025214\H,0
,1.0449597929,0.4725948758,-1.6802295129\H,0,1.8826296654,1.9424481298
,0.1022230961\H,0,-2.5244717148,0.9700521738,-0.1071430927\H,0,-3.2797
510081,-0.5793939022,-0.5378139193\N,0,-2.4170518973,-0.5730348385,1.3
599276592\C,0,-3.7110954092,-0.6385290287,2.0483699641\H,0,-4.47511791
11,-0.6524194851,1.2619942482\H,0,-3.8870128834,0.2818857463,2.6314717
233\C,0,2.4875954932,0.366929563,1.4700417658\H,0,3.113787301,1.046372
5287,2.060165311\H,0,3.025328373,-0.5910302719,1.4149527963\C,0,-3.890
1117503,-1.8604852008,2.9429694581\C,0,-4.6365086287,-1.7466991719,4.1
254206454\C,0,-3.3708996196,-3.1115501976,2.5804029618\C,0,-4.86588510
58,-2.8672058468,4.9308611031\H,0,-5.0526167021,-0.7814320337,4.412849
828\C,0,-3.5945665613,-4.2297557073,3.388164388\H,0,-2.7891548207,-3.2
069059791,1.6661749891\C,0,-4.3432180111,-4.1108657806,4.5642917421\H,
0,-5.4518830339,-2.767249348,5.8412048446\H,0,-3.1890399831,-5.1956528
036,3.0965886178\H,0,-4.5197379598,-4.982709095,5.1891373928\C,0,-0.19
01208688,0.0019690933,5.6472698506\H,0,-0.8502007348,0.8121572156,5.97
11526197\H,0,-0.5762527756,-0.95610839,6.0071083763\H,0,0.8083919032,0
.1651531965,6.0553420107\\Version=AM64L-G03RevD.01\State=1-A\HF=-857.6
556802\RMSD=3.057e-09\Thermal=0.\Dipole=1.8713032,1.4359667,0.0938456\
PG=C01 [X(C18H22N3)]\\@

65

see reference 7

65-Me$^+$
see reference 7

66
1\1\GINC-CALYPSO\SP\RMP2-FC\6-31+G(2d,p)\H3P1\CHRISTOPH\02-Mar-2010\0\
\#p MP2(FC)/6-31+g(2d,p) scf=tight\\ph3sp\\0,1\P\H,1,1.42109953\H,1,1.
42109888,2,93.5201566\H,1,1.42109888,3,93.52010571,2,-93.75074791,0\\V
ersion=AM64L-G03RevD.01\State=1-A\HF=-342.4588847\MP2=-342.5942622\RMS
D=3.081e-09\Thermal=0.\PG=C03 [C3(P1),X(H3)]\\@

66-Me$^+$
1\1\GINC-CALYPSO\SP\RMP2-FC\6-31+G(2d,p)\C1H6P1(1+)\CHRISTOPH\02-Mar-2
010\0\\#p MP2(FC)/6-31+g(2d,p) scf=tight\\ph3mesp\\1,1\H\H,1,2.2439319
\H,1,2.2439319,2,60.00000297\P,2,1.39803701,1,36.62768202,3,-39.047949
72,0\C,4,1.813649,2,112.07675995,1,-123.06365336,0\H,5,1.09552398,4,10
9.34641901,2,180.,0\H,5,1.09552409,4,109.34641708,2,-60.00001425,0\H,5
,1.09552398,4,109.34641901,2,60.00002131,0\\Version=AM64L-G03RevD.01\S
tate=1-A\HF=-381.8456638\MP2=-382.1248671\RMSD=4.696e-09\Thermal=0.\PG
=C03 [C3(C1P1),X(H6)]\\@

67
1\1\GINC-CALYPSO\SP\RMP2-FC\6-31+G(2d,p)\C1H5P1\CHRISTOPH\02-Mar-2010\
0\\#p MP2(FC)/6-31+g(2d,p) scf=tight\\pme1sp\\0,1\P\H,1,1.42257127\H,1
,1.4225706,2,93.40083709\C,1,1.87380781,3,97.51755341,2,98.0493096,0\H
,4,1.09585819,1,108.85366587,3,74.33918969,0\H,4,1.09586005,1,108.8519
2962,3,-168.76025528,0\H,4,1.09435207,1,113.71388658,3,-47.21142992,0\
\Version=AM64L-G03RevD.01\State=1-A\HF=-381.5008963\MP2=-381.7894638\R
MSD=2.254e-09\Thermal=0.\PG=C01 [X(C1H5P1)]\\@

67-Me$^+$
1\1\GINC-CALYPSO\SP\RMP2-FC\6-31+G(2d,p)\C2H8P1(1+)\CHRISTOPH\02-Mar-2
010\0\\#p MP2(FC)/6-31+g(2d,p) scf=tight\\pme1mesp\\1,1\C\H,1,1.095643
68\H,1,1.09573179,2,109.26599564\H,1,1.09564368,2,109.33559359,3,-119.
54253071,0\P,1,1.81335646,4,109.6869194,3,-120.14408908,0\H,5,1.399032
28,1,109.33608637,4,177.18222324,0\H,5,1.39903228,1,109.33608637,4,62.
71823447,0\C,5,1.81335646,1,114.08675906,4,-60.04977115,0\H,8,1.095731
79,5,109.58398577,1,180.,0\H,8,1.09564368,5,109.6869194,1,-60.04977115
,0\H,8,1.09564368,5,109.6869194,1,60.04977115,0\\Version=AM64L-G03RevD
.01\State=1-A1\HF=-420.9110316\MP2=-421.343927\RMSD=6.555e-09\Thermal=
0.\PG=C02V [C2(P1),SGV(C2H2),SGV'(H2),X(H4)]\\@

68
1\1\GINC-CALYPSO\SP\RMP2-FC\6-31+G(2d,p)\C2H7P1\CHRISTOPH\02-Mar-2010\
0\\#p MP2(FC)/6-31+g(2d,p) scf=tight\\pme2sp\\0,1\P\H,1,1.42404723\C,1
,1.86912938,2,96.87887024\H,3,1.09659206,1,109.13858151,2,172.54166414
,0\H,3,1.09631201,1,109.36079203,2,-70.08441412,0\H,3,1.09656581,1,113
.15983025,2,51.42308944,0\C,1,1.86911629,3,100.13394072,5,-168.3827081
4,0\H,7,1.09630268,1,109.37338111,3,168.62921854,0\H,7,1.09660939,1,10
9.12513545,3,-73.99930778,0\H,7,1.09655662,1,113.16061439,3,47.1067555
6,0\\Version=AM64L-G03RevD.01\State=1-A\HF=-420.5447445\MP2=-420.98833
88\RMSD=4.799e-09\Thermal=0.\PG=C01 [X(C2H7P1)]\\@

68-Me$^+$
1\1\GINC-CALYPSO\SP\RMP2-FC\6-31+G(2d,p)\C3H10P1(1+)\CHRISTOPH\02-Mar-
2010\0\\#p MP2(FC)/6-31+g(2d,p) scf=tight\\pme2mesp\\1,1\C\H,1,1.09567
82\H,1,1.0956782,2,109.05123245\H,1,1.0960815,3,109.06770347,2,119.015

21383,0\C,1,2.99637623,3,144.22708134,2,-118.28087512,0\H,5,1.09567875
,1,144.22709971,3,0.,0\H,5,1.09567816,1,89.82613799,3,123.65347262,0\H
,5,1.0960815,1,91.91760889,3,-127.27378001,0\P,5,1.81425071,1,34.33146
43,3,-3.87742455,0\H,9,1.400238,5,107.53392101,1,117.55624647,0\C,9,1.
81425071,5,111.33709945,1,-124.88749134,0\H,11,1.09567816,9,109.941642
96,5,-177.58990611,0\H,11,1.09567875,9,109.94162514,5,-57.5225943,0\H,
11,1.0960815,9,109.74830926,5,62.44371231,0\\Version=AM64L-G03RevD.01\
State=1-A1\HF=-459.9740656\MP2=-460.562018\RMSD=6.730e-09\Thermal=0.\P
G=C03V [C3(P1H1),3SGV(C1H1),X(H6)]\\@

69

1\1\GINC-NODE-12\SP\RMP2-FC\6-31+G(2d,p)\C15H33P1\ZIP07\28-May-2009\0\
\#p MP2(FC)/6-31+g(2d,p) scf=tight\\tppspc001\\0,1\P\C,1,1.89039495\H,
2,1.09888739,1,106.42350163\H,2,1.10176785,1,108.87277892,3,-114.09496
776,0\C,1,1.88970378,2,98.98647918,3,137.90236354,0\H,5,1.09911845,1,1
06.21055431,2,38.58234572,0\H,5,1.10163791,1,108.94015567,2,-75.479670
81,0\C,1,1.89062093,5,99.1378185,2,100.80047615,0\H,8,1.0989988,1,106.
48476927,5,37.65935691,0\H,8,1.10171367,1,108.96776862,5,-76.51634361,
0\C,5,1.5562984,1,117.33395698,2,159.33724554,0\C,8,1.55619276,1,117.0
8500984,5,158.58288227,0\C,2,1.55638785,1,117.16186029,5,-101.19862667
,0\C,13,1.54396886,2,110.35195881,1,-62.86946,0\H,14,1.09690935,13,111
.28296848,2,61.19826052,0\H,14,1.09831169,13,110.63660792,2,-178.63162
842,0\H,14,1.0986845,13,110.98555903,2,-59.09057018,0\C,13,1.54367495,
2,111.40889839,1,58.75060113,0\H,18,1.09831511,13,110.51888089,2,177.2
9096865,0\H,18,1.09785817,13,111.49634903,2,-63.16395095,0\H,18,1.0989
2894,13,111.3194898,2,57.76820835,0\C,13,1.5457497,2,107.90601465,1,17
8.18311709,0\H,22,1.09860884,13,110.86239559,2,178.95534201,0\H,22,1.0
9844485,13,111.20101774,2,-61.1158585,0\H,22,1.0983341,13,111.15310415
,2,59.06728483,0\C,11,1.5459436,5,107.81460917,1,178.21605831,0\H,26,1
.09840217,11,111.10949432,5,59.13152871,0\H,26,1.09859109,11,110.84529
659,5,178.98415406,0\H,26,1.09838753,11,111.22942554,5,-61.05641689,0\
C,11,1.54411543,5,110.34068042,1,-62.84570576,0\H,30,1.09873464,11,110
.98557263,5,-59.24940589,0\H,30,1.09688996,11,111.25230694,5,61.038324
38,0\H,30,1.098378,11,110.62242396,5,-178.79995424,0\C,11,1.54346598,5
,111.44258584,1,58.8021008,0\H,34,1.09774258,11,111.54023768,5,-63.143
9673,0\H,34,1.09896049,11,111.31497771,5,57.80770735,0\H,34,1.09829412
,11,110.4565974,5,177.31331252,0\C,12,1.54390524,8,110.3878488,1,-63.1
936386,0\H,38,1.09872807,12,110.97698273,8,-59.24526013,0\H,38,1.09680
289,12,111.22392689,8,61.05416194,0\H,38,1.09839999,12,110.64756442,8,
-178.78072195,0\C,12,1.54593361,8,107.86344711,1,177.83627618,0\H,42,1
.09857944,12,110.83904832,8,179.05228043,0\H,42,1.09854138,12,111.2237
2826,8,-61.01741149,0\H,42,1.09837605,12,111.14014508,8,59.18261954,0\
C,12,1.54360158,8,111.39285754,1,58.46300614,0\H,46,1.09781019,12,111.
51390535,8,-62.79206526,0\H,46,1.09895348,12,111.31549475,8,58.1440579
2,0\H,46,1.09837836,12,110.49885213,8,177.62521161,0\\Version=IA32L-G0
3RevD.01\State=1-A\HF=-928.034924\MP2=-930.512811\RMSD=1.952e-09\Therm
al=0.\PG=C01 [X(C15H33P1)]\\@

69-Me$^+$

1\1\GINC-NODE-18\SP\RMP2-FC\6-31+G(2d,p)\C16H36P1(1+)\ZIP07\28-May-200
9\0\\#p mp2(FC)/6-31+g(2d,p) scf=tight\\tppmespc001\\1,1\C\H,1,1.09686
08\H,1,1.10128182,2,106.04140503\C,1,2.95372383,2,136.15905158,3,-83.5
3378903,0\H,4,1.09698686,1,76.14662367,2,175.62904874,0\H,4,1.10123787
,1,89.82819035,2,69.09739394,0\C,4,2.95640981,1,60.04643338,2,-2.73481
691,0\H,7,1.0968983,4,76.53360714,1,178.99826984,0\H,7,1.10127456,4,89
.44476414,1,72.39077581,0\C,4,1.56480282,1,157.51887398,7,-76.27794135
,0\C,7,1.56523885,4,157.68083976,1,-74.87654372,0\C,1,1.56518096,4,112
.63042399,10,79.76413495,0\C,12,1.54464496,1,111.24520245,4,-113.28370

637,0\H,13,1.0955033,12,112.8742089,1,70.1079988,0\H,13,1.09628654,12,
109.73060415,1,-170.78949192,0\H,13,1.09789311,12,111.37126782,1,-52.0
2381052,0\C,12,1.54261073,1,112.75991785,13,124.29278404,0\H,17,1.0962
5688,12,109.79329084,1,175.74288444,0\H,17,1.09867272,12,112.60091812,
1,-65.42000044,0\H,17,1.09808323,12,111.69897953,1,56.96723799,0\C,12,
1.54891988,1,105.43420384,13,-117.22614488,0\H,21,1.09633316,12,110.03
579188,1,178.69093098,0\H,21,1.09753598,12,111.53129986,1,-61.74981908
,0\H,21,1.09741666,12,111.46619482,1,59.27632708,0\C,10,1.54890476,4,1
05.45704545,1,-165.00646989,0\H,25,1.09739307,10,111.4892568,4,59.0769
0594,0\H,25,1.09634253,10,110.05235745,4,178.52647588,0\H,25,1.0975512
3,10,111.48801397,4,-61.93688384,0\C,10,1.54482591,4,111.25022788,1,-4
7.70687679,0\H,29,1.09792447,10,111.37662245,4,-52.50449183,0\H,29,1.0
9558327,10,112.86161268,4,69.61393079,0\H,29,1.09631151,10,109.7548446
8,4,-171.28530586,0\C,10,1.54235173,4,112.70480362,1,76.52977544,0\H,3
3,1.09878478,10,112.54378631,4,-65.77109996,0\H,33,1.09803384,10,111.6
6446672,4,56.57781581,0\H,33,1.0962431,10,109.81323584,4,175.37284487,
0\C,11,1.54476176,7,111.2778903,4,-48.31402283,0\H,37,1.09788334,11,11
1.37409467,7,-52.64261734,0\H,37,1.09556764,11,112.91407294,7,69.52244
899,0\H,37,1.09626323,11,109.6999264,7,-171.39001807,0\C,11,1.54906971
,7,105.40614542,4,-165.54723633,0\H,41,1.09637331,11,110.061645,7,178.
97921837,0\H,41,1.09747899,11,111.5234368,7,-61.46603067,0\H,41,1.0973
7812,11,111.46289694,7,59.55642681,0\C,11,1.54235003,7,112.76693007,4,
75.95892716,0\H,45,1.09878563,11,112.59808452,7,-65.42567311,0\H,45,1.
09808869,11,111.7004706,7,56.9619832,0\H,45,1.09624495,11,109.77623829
,7,175.73884207,0\P,7,1.84967265,4,36.95844149,1,-39.95853089,0\C,49,1
.82444176,7,112.62674419,4,-123.77277496,0\H,50,1.0937221,49,109.90069
996,7,53.46425962,0\H,50,1.09363705,49,109.89808426,7,173.50747639,0\H
,50,1.09369346,49,109.86749897,7,-66.51745543,0\\Version=IA32L-G03RevD
.01\State=1-A\HF=-967.4792026\MP2=-970.1024934\RMSD=2.564e-09\Thermal=
0.\PG=C01 [X(C16H36P1)]\\@

70

1\1\GINC-CIPCLU03\SP\RMP2-FC\6-31+G(2d,p)\C3H9P1\C2175\17-Nov-2009\0\\
#p MP2(FC)/6-31+G(2d,p) scf=tight\\pme3sp_1\\0,1\P\C,1,1.86497215\H,2,
1.09685206,1,109.61364171\H,2,1.09684723,1,109.6215538,3,117.80222963,
0\H,2,1.09886322,1,112.71356408,3,-121.07805684,0\C,1,1.86506174,2,99.
29371277,4,-171.62248987,0\H,6,1.09688092,1,109.61676987,2,-70.2057677
9,0\H,6,1.09891778,1,112.70385826,2,50.87148402,0\H,6,1.09683381,1,109
.62747355,2,171.97520625,0\C,1,1.86489918,2,99.40630136,6,101.16565665
,0\H,10,1.09688657,1,109.56739824,2,-171.68434261,0\H,10,1.0989131,1,1
12.7618532,2,-50.6052452,0\H,10,1.09692199,1,109.62811464,2,70.5568945
5,0\\Version=AM64L-G03RevD.01\State=1-A\HF=-459.5903732\MP2=-460.19088
11\RMSD=3.989e-09\Thermal=0.\PG=C01 [X(C3H9P1)]\\@

70-Me$^+$

1\1\GINC-CIPCLU08\SP\RMP2-FC\6-31+G(2d,p)\C4H12P1(1+)\C2175\17-Nov-200
9\0\\#p MP2(FC)/6-31+G(2d,p) scf=tight\\pme3mesp_1\\1,1\C\H,1,1.095865
94\H,1,1.09585111,2,108.89581276\H,1,1.09582994,3,108.89315529,2,118.6
4227629,0\C,1,2.96605495,4,90.45902368,3,-150.69790877,0\H,5,1.0958860
2,1,90.54710277,4,-108.96022288,0\H,5,1.0958657,1,90.42936704,4,-0.068
43832,0\H,5,1.09584855,1,145.31399137,4,125.37252239,0\C,5,2.96574131,
1,60.00953686,4,90.26006121,0\H,9,1.09586883,5,90.48583067,1,160.77745
375,0\H,9,1.09589374,5,90.45924552,1,-90.32126123,0\H,9,1.09586895,5,1
45.31968239,1,35.19819845,0\P,1,1.8162306,5,35.27139582,9,-35.28230538
,0\C,13,1.81637385,1,109.51391593,5,-120.03010925,0\H,14,1.09586037,13
,110.01863392,1,-179.94271598,0\H,14,1.09584524,13,110.05002534,1,-59.
95664847,0\H,14,1.09588397,13,110.03724252,1,60.06504024,0\\Version=AM
64L-G03RevD.01\State=1-A\HF=-499.0351154\MP2=-499.7795874\RMSD=2.002e-

09\Thermal=0.\PG=C01 [X(C4H12P1)]\\@

70-BH$^+$

1\1\GINC-CALYPSO\SP\RMP2-FC\6-31+G(2d,p)\C16H20P1(1+)\CHRISTOPH\09-Dec
-2010\0\\#p MP2(FC)/6-31+g(2d,p) scf=tight\\pme3bhsp_4\\1,1\C\H,1,1.10
076161\P,1,1.86496002,2,100.89710911\C,1,4.34710677,3,113.35394555,2,-
111.52819168,0\C,4,1.39782559,1,58.80398375,3,120.97728633,0\C,5,1.397
07454,4,120.13659319,1,-0.37857771,0\C,6,1.40507737,5,120.83611202,4,0
.51877617,0\C,7,1.40653764,6,118.52405505,5,-0.58814217,0\C,4,1.397593
54,1,60.81327922,7,-5.76037896,0\H,4,1.08668975,1,179.00257402,7,-171.
15586023,0\H,5,1.08686388,4,120.23455421,1,179.63463093,0\H,6,1.088630
78,5,119.38624737,4,179.61222063,0\H,8,1.08708891,7,120.91565647,6,179
.50095427,0\H,9,1.08697871,4,120.17280015,1,-179.605263,0\C,1,4.348403
38,7,119.28999544,6,-107.02854843,0\C,15,1.39786814,1,59.1857779,7,135
.93026819,0\C,16,1.39739101,15,120.00572374,1,-0.14221419,0\C,17,1.406
22883,16,120.76793324,15,0.02781223,0\C,18,1.40476029,17,118.77948943,
16,-0.47383569,0\C,19,1.39806979,18,120.37134518,17,0.46009922,0\H,15,
1.08673842,1,179.31483885,7,116.58475313,0\H,16,1.08692076,15,120.2767
2469,1,-179.32552877,0\H,17,1.08933208,16,119.31429539,15,-178.5230402
8,0\H,19,1.085847,18,120.52671832,17,-177.16160687,0\H,20,1.0869634,19
,119.49349685,18,-179.27305122,0\C,3,1.82072661,1,108.5806814,7,-163.6
348376,0\H,26,1.09568495,3,110.21145239,1,60.34341252,0\H,26,1.0958485
4,3,109.73021466,1,-179.80755655,0\H,26,1.095078,3,109.73460253,1,-59.
70339944,0\C,3,1.81942151,1,109.65897078,7,-45.11558437,0\H,30,1.09578
312,3,109.99172384,1,-177.20623539,0\H,30,1.09573997,3,109.80352439,1,
-57.15142817,0\H,30,1.09475448,3,109.68579701,1,62.2753717,0\C,3,1.820
25939,1,113.71259068,7,77.23555309,0\H,34,1.09536544,3,109.72509086,1,
49.64587378,0\H,34,1.09369736,3,110.82942726,1,-71.49055733,0\H,34,1.0
9613837,3,108.9018421,1,168.97409795,0\\Version=AM64L-G03RevD.01\State
=1-A\HF=-958.1567257\MP2=-960.5838562\RMSD=2.288e-09\Thermal=0.\PG=C01
[X(C16H20P1)]\\@

70-TT$^+$

1\1\GINC-BORIX\SP\RMP2-FC\6-31+G(2d,p)\C22H24P1(1+)\CHRISTOPH\19-Apr-2
012\0\\#p MP2(FC)/6-31+g(2d,p) scf=tight\\pme3ttsp_1\\1,1\C\C,1,4.3818
3451\C,2,1.39497759,1,59.67211757\C,3,1.40038104,2,120.66120395,1,-0.5
9614942,0\C,4,1.40352404,3,120.76987211,2,0.12047603,0\C,5,1.41028666,
4,117.89178888,3,1.40816694,0\C,6,1.39581911,5,121.32344966,4,-1.96875
538,0\H,2,1.08685663,1,179.68964167,5,172.42705783,0\H,3,1.08723426,2,
120.18943008,1,179.21012911,0\H,4,1.0844207,3,118.93302773,2,179.36691
961,0\H,6,1.0869432,5,120.27638955,4,175.27436773,0\H,7,1.08729924,6,1
19.5826563,5,-179.96551302,0\C,1,4.38232014,5,111.19866187,4,12.586304
99,0\C,13,1.39493302,1,59.65994565,5,-117.00145572,0\C,14,1.40029492,1
3,120.66216795,1,-0.6041492,0\C,15,1.40369086,14,120.77962863,13,0.164
58705,0\C,16,1.41026383,15,117.8798679,14,1.28487979,0\C,17,1.39589892
,16,121.32437356,15,-1.84090095,0\H,13,1.08684958,1,179.72287252,5,-6.
27037889,0\H,14,1.08721879,13,120.18393216,1,179.21121841,0\H,15,1.084
33463,14,118.89551328,13,179.37025913,0\H,17,1.08696745,16,120.2869436
9,15,175.43305505,0\H,18,1.08730477,17,119.57544972,16,-179.98748254,0
\C,1,4.38118317,5,112.52019412,4,-113.64358858,0\C,24,1.39498141,1,59.
66853967,5,9.3091723,0\C,25,1.40033318,24,120.65454175,1,-0.57397295,0
\C,26,1.40355189,25,120.76138897,24,0.18331766,0\C,27,1.4102628,26,117
.91860775,25,1.21094802,0\C,28,1.39585637,27,121.30193365,26,-1.767712
44,0\H,24,1.08684898,1,179.74883953,27,175.97154226,0\H,25,1.08722582,
24,120.19070304,1,179.25393821,0\H,26,1.08441154,25,118.97005297,24,17
9.44808144,0\H,28,1.08709602,27,120.33460797,26,175.55242116,0\H,29,1.
08729021,28,119.58036161,27,-179.97590495,0\P,1,1.94574399,27,106.6467
5439,26,126.93059192,0\C,35,1.82874445,1,112.98122137,27,89.87353324,0

\H,36,1.09342167,35,110.28251747,1,59.62792635,0\H,36,1.09658564,35,10
8.03430865,1,179.37092295,0\H,36,1.09284143,35,111.37119803,1,-61.3013
4208,0\C,35,1.82873957,1,113.01723479,27,-30.10865456,0\H,40,1.0933116
4,35,110.34617033,1,59.58118681,0\H,40,1.09654363,35,107.97684335,1,17
9.3069493,0\H,40,1.09279846,35,111.3733444,1,-61.40487587,0\C,35,1.828
95678,1,113.0905775,27,-150.04950613,0\H,44,1.09654394,35,107.95518598
,1,179.35829608,0\H,44,1.0926945,35,111.43024018,1,-61.32013549,0\H,44
,1.09327627,35,110.31879951,1,59.6711007,0\\Version=AM64L-G03RevD.01\S
tate=1-A\HF=-1187.6906642\MP2=-1190.9831988\RMSD=7.010e-09\Thermal=0.\
PG=C01 [X(C22H24P1)]\\@

71

1\1\GINC-NODE-01\SP\RMP2-FC\6-31+G(2d,p)\C11H25P1\ZIP07\27-May-2009\0\
\#p MP2(FC)/6-31+g(2d,p) scf=tight\\dpmpspc001\\0,1\P\C,1,1.86860283\H
,2,1.09700477,1,109.33227066\H,2,1.09576861,1,109.89317925,3,117.95437
961,0\H,2,1.09883201,1,112.66758627,3,-120.87892687,0\C,1,1.88753727,2
,97.75348893,4,-170.93247284,0\H,6,1.10057699,1,105.99276073,2,-37.060
09547,0\H,6,1.10167165,1,109.30509977,2,77.01605563,0\C,1,1.88846418,2
,100.09035796,6,101.51293319,0\H,9,1.09908456,1,106.64595441,2,-139.70
017468,0\H,9,1.1019584,1,108.72695671,2,-25.38985257,0\C,6,1.55586402,
1,117.06430122,2,-157.60221331,0\C,9,1.55566724,1,116.8653238,2,99.196
00496,0\C,13,1.54364832,9,111.13760585,1,-57.3216011,0\H,14,1.09808309
,13,111.39292676,9,63.92073457,0\H,14,1.09827182,13,110.59809374,9,-17
6.60346625,0\H,14,1.09878243,13,111.28306282,9,-56.94227914,0\C,13,1.5
4564868,9,107.99787782,1,-176.74455295,0\H,18,1.09856997,13,110.882038
96,9,-178.89889496,0\H,18,1.0984035,13,111.11832995,9,-59.02988642,0\H
,18,1.09847328,13,111.20208221,9,61.14057901,0\C,13,1.54389383,9,110.3
4544063,1,64.21870589,0\H,22,1.0987646,13,110.94710194,9,59.61097389,0
\H,22,1.09830539,13,110.67168294,9,179.12927498,0\H,22,1.09680804,13,1
11.28332483,9,-60.69110709,0\C,12,1.54364737,6,111.28752318,1,-58.6220
9576,0\H,26,1.09885127,12,111.28641358,6,-58.01274842,0\H,26,1.0980419
3,12,111.55765596,6,62.93006126,0\H,26,1.09831216,12,110.51032337,6,-1
77.49736678,0\C,12,1.54383098,6,110.28666424,1,62.96907309,0\H,30,1.09
876321,12,110.97303964,6,58.98626424,0\H,30,1.09829856,12,110.64358896
,6,178.55047406,0\H,30,1.09660827,12,111.2574593,6,-61.21655987,0\C,12
,1.54572358,6,107.96277021,1,-178.06360955,0\H,34,1.09849104,12,111.25
975814,6,60.97515965,0\H,34,1.09852315,12,110.86024174,6,-179.04630929
,0\H,34,1.09846847,12,111.11950842,6,-59.19745291,0\\Version=IA32L-G03
RevD.01\State=1-A\HF=-771.887443\MP2=-773.7377509\RMSD=3.984e-09\Therm
al=0.\PG=C01 [X(C11H25P1)]\\@

71-Me⁺

1\1\GINC-NODE-17\SP\RMP2-FC\6-31+G(2d,p)\C12H28P1(1+)\ZIP07\28-May-200
9\0\\#p MP2(FC)6-31+g(2d,p) scf=tight\\dpmpmespc001\\1,1\C\H,1,1.09592
942\H,1,1.09363412,2,109.05154494\H,1,1.09583054,3,108.83451888,2,118.
44027769,0\C,1,2.90364613,3,148.75602813,4,117.10245058,0\H,5,1.099684
14,1,74.52256142,3,149.28986128,0\H,5,1.10109751,1,91.46880659,3,-104.
47618986,0\C,5,2.98628122,1,61.03079388,3,-31.27592367,0\H,8,1.0970058
,5,78.50804336,1,177.22233337,0\H,8,1.1014359,5,88.3223945,1,-75.96251
728,0\C,5,1.56518577,1,156.34788516,8,79.01826148,0\C,8,1.56500257,5,1
57.24171324,1,65.60804921,0\C,12,1.54250123,8,112.47215138,5,-69.39062
106,0\H,13,1.09873997,12,112.44308895,8,68.26571908,0\H,13,1.09611332,
12,109.81168616,8,-173.2188568,0\H,13,1.09791771,12,111.756797,8,-54.2
278517,0\C,12,1.54844189,8,105.42497678,5,172.15171643,0\H,17,1.096281
76,12,110.00862528,8,-177.41951852,0\H,17,1.0974125,12,111.51277951,8,
-58.00841272,0\H,17,1.09748239,12,111.49984279,8,63.07235756,0\C,12,1.
54406286,8,111.00727449,5,54.71228754,0\H,21,1.09784466,12,111.3436059
7,8,53.94781839,0\H,21,1.09617857,12,109.81179295,8,172.78977317,0\H,2

1,1.09610767,12,112.77106788,8,-68.07057378,0\C,11,1.54248856,5,112.44
474954,1,-79.68129401,0\H,25,1.09806452,11,111.75311257,5,-56.12916869
,0\H,25,1.09925599,11,112.48596126,5,66.3434338,0\H,25,1.09614749,11,1
09.78652261,5,-174.96619108,0\C,11,1.54438684,5,111.10517045,1,44.2099
2445,0\H,29,1.09781756,11,111.39991067,5,53.19088846,0\H,29,1.09618362
,11,109.73012494,5,171.98288281,0\H,29,1.09580523,11,112.84068087,5,-6
8.94618751,0\C,11,1.54832554,5,105.58177372,1,161.67517976,0\H,33,1.09
746654,11,111.51096786,5,62.60668576,0\H,33,1.09626562,11,110.00880522
,5,-177.88097139,0\H,33,1.09733603,11,111.49318126,5,-58.46481368,0\P,
1,1.82523264,5,37.96923276,11,42.06770894,0\C,37,1.82272814,1,106.6322
349,5,-119.86712561,0\H,38,1.09548581,37,108.94960686,1,63.89942335,0\
H,38,1.09316067,37,111.29819607,1,-175.70582429,0\H,38,1.09482657,37,1
09.50455373,1,-55.05524032,0\\Version=IA32L-G03RevD.01\State=1-A\HF=-8
11.3333501\MP2=-813.3283641\RMSD=2.486e-09\Thermal=0.\PG=C01 [X(C12H28
P1)]\\@

72

1\1\GINC-NODE16\SP\RMP2-FC\6-31+G(2d,p)\C5H11P1\ZIP07\01-Sep-2009\0\\#
p MP2(FC)/6-31+g(2d,p) scf=tight\\pro1sp_1\\0,1\P\C,1,1.83967333\C,2,1
.52621088,1,119.03271862\C,3,1.508338,2,60.38575723,1,-108.3948909,0\H
,2,1.09281037,1,116.35617337,3,145.58116829,0\H,3,1.08835055,2,117.923
73729,1,-0.60123939,0\H,3,1.08773889,2,117.52397516,1,142.81962473,0\H
,4,1.08835985,3,117.78500218,2,108.01903661,0\H,4,1.0877438,3,118.4687
3662,2,-107.25623215,0\C,1,1.86546025,2,99.77445353,4,94.9013323,0\H,1
0,1.09649109,1,109.36767468,2,-69.73921371,0\H,10,1.09709784,1,109.637
49936,2,172.2443549,0\H,10,1.09872204,1,112.66857237,2,51.1915868,0\C,
1,1.86545282,2,99.77440857,4,-163.72829849,0\H,14,1.09648977,1,109.368
41844,2,69.74219815,0\H,14,1.09872067,1,112.66980384,2,-51.18999345,0\
H,14,1.09710124,1,109.6360066,2,-172.24208798,0\\Version=AM64L-G03RevD
.01\State=1-A\HF=-536.4627401\MP2=-537.3493187\RMSD=8.678e-09\Thermal=
0.\PG=C01 [X(C5H11P1)]\\@

72-Me$^+$

1\1\GINC-NODE28\SP\RMP2-FC\6-31+G(2d,p)\C6H14P1(1+)\ZIP07\01-Sep-2009\
0\\#p MP2(FC)/6-31+g(2d,p) scf=tight\\pro1mesp_2\\1,1\P\C,1,1.81829204
\H,2,1.0958511,1,109.86991237\H,2,1.09542499,1,109.78505266,3,119.6073
1702,0\H,2,1.09586843,1,110.28095448,4,120.36660994,0\C,1,1.81772904,2
,108.78187155,4,-61.74951167,0\H,6,1.09591875,1,109.10191576,2,-59.226
55431,0\H,6,1.09515197,1,110.61254359,2,60.14339402,0\H,6,1.09515335,1
,110.6108588,2,-178.59523522,0\C,1,1.8182943,6,108.77985232,2,118.4250
1926,0\H,10,1.09586769,1,110.28181142,6,-58.64083531,0\H,10,1.09542537
,1,109.78398329,6,61.72548332,0\H,10,1.09584976,1,109.87038919,6,-178.
66803462,0\C,1,1.79434036,6,111.24026244,10,120.78659813,0\C,14,1.5331
4471,1,122.5159299,6,35.47348667,0\C,15,1.50076,14,60.69657546,1,-110.
96287014,0\H,14,1.09066458,1,111.51152622,6,179.99453539,0\H,15,1.0874
5948,14,118.9212747,1,-3.21904421,0\H,15,1.08641391,14,115.89459058,1,
138.7699248,0\H,16,1.08745779,15,117.99061938,14,109.25486683,0\H,16,1
.08641475,15,119.04404002,14,-105.13688979,0\\Version=AM64L-G03RevD.01
\State=1-A\HF=-575.9082038\MP2=-576.9391879\RMSD=6.806e-09\Thermal=0.\
PG=C01 [X(C6H14P1)]\\@

73

1\1\GINC-NODE-17\SP\RMP2-FC\6-31+G(2d,p)\C7H17P1\ZIP07\27-May-2009\0\\
#p MP2(FC)/6-31+g(2d,p) scf=tight\\pdmpspc001\\0,1\P\C,1,1.86751217\H,
2,1.09573114,1,110.21032358\H,2,1.09719073,1,109.15002351,3,117.937681
76,0\H,2,1.09898538,1,112.60667063,4,120.61253289,0\C,1,1.86676165,2,9
8.45029018,3,173.05757982,0\H,6,1.09694242,1,109.38368387,2,66.8348597
2,0\H,6,1.0967323,1,109.68023243,2,-175.32904864,0\H,6,1.09871883,1,11

2.82259905,2,-54.06450487,0\C,1,1.88532953,6,98.32531276,2,102.5516260
8,0\H,10,1.10082086,1,106.06459773,6,40.64057498,0\H,10,1.10185471,1,1
09.0561586,6,-73.50601667,0\C,10,1.55555667,1,117.00050654,6,161.30234
831,0\C,13,1.54358574,10,111.05939688,1,57.54083726,0\H,14,1.09821884,
13,110.59567129,10,176.41224503,0\H,14,1.09805214,13,111.37285161,10,-
64.16633124,0\H,14,1.09871927,13,111.29735323,10,56.67720401,0\C,13,1.
54382755,10,110.28352645,1,-63.95546216,0\H,18,1.09826962,13,110.62052
682,10,-179.08967396,0\H,18,1.09871638,13,110.9360266,10,-59.55494957,
0\H,18,1.09663959,13,111.30123761,10,60.66545141,0\C,13,1.54552181,10,
108.01746493,1,177.00974234,0\H,22,1.09854145,13,110.87913073,10,178.6
284799,0\H,22,1.0985131,13,111.23334598,10,-61.39321142,0\H,22,1.09846
762,13,111.12844596,10,58.77492834,0\\Version=IA32L-G03RevD.01\State=1
-A\HF=-615.7392494\MP2=-616.9638578\RMSD=3.834e-09\Thermal=0.\PG=C01 [
X(C7H17P1)]\\@

73-Me$^+$

1\1\GINC-NODE-12\SP\RMP2-FC\6-31+G(2d,p)\C8H20P1(1+)\ZIP07\27-May-2009
\0\\#p MP2(FC)/6-31+g(2d,p) scf=tight\\pdmpmespc001\\1,1\C\H,1,1.09365
875\H,1,1.09602654,2,109.07370772\H,1,1.09580564,2,108.94597967,3,-118
.60860887,0\C,1,2.93478342,2,147.16019518,4,-123.79040283,0\H,5,1.0958
1263,1,88.30846397,2,-123.86519672,0\H,5,1.09571108,1,146.27878085,2,-
1.21596366,0\H,5,1.09585193,1,91.59625482,2,127.34751256,0\C,5,2.93141
718,1,62.15017699,2,40.72151907,0\H,9,1.09982479,5,76.89110971,1,-175.
51375989,0\H,9,1.10129088,5,89.4491447,1,77.79131534,0\C,9,1.56504137,
5,156.57944559,1,-67.18498194,0\C,12,1.54221208,9,112.01010232,5,70.60
186557,0\H,13,1.09590565,12,109.90488482,9,173.5080366,0\H,13,1.099190
2,12,112.13398968,9,-68.0779886,0\H,13,1.09796883,12,111.90223391,9,54
.34573668,0\C,12,1.54383242,9,110.88008172,5,-52.96715288,0\H,17,1.096
08819,12,109.76142279,9,-174.0096279,0\H,17,1.0975451,12,111.38891822,
9,-55.15506193,0\H,17,1.09631746,12,112.77379702,9,66.8673777,0\C,12,1
.54779316,9,105.7227332,5,-170.65158876,0\H,21,1.09616773,12,109.94164
526,9,177.53985614,0\H,21,1.0974826,12,111.53214965,9,-62.9758868,0\H,
21,1.09737104,12,111.54043442,9,58.16735112,0\P,1,1.8206545,5,36.32726
92,9,36.24136341,0\C,25,1.82050315,1,108.60611857,5,115.92555558,0\H,2
6,1.09434545,25,110.87280698,1,58.13094444,0\H,26,1.09510417,25,110.35
212951,1,179.16680843,0\H,26,1.09646095,25,108.7305109,1,-61.45280564,
0\\Version=IA32L-G03RevD.01\State=1-A\HF=-655.18536\MP2=-656.5542304\R
MSD=4.411e-09\Thermal=0.\PG=C01 [X(C8H20P1)]\\@

74

1\1\GINC-AZAZEL\SP\RMP2-FC\6-31+G(2d,p)\C4H11P1\CHRISTOPH\22-Sep-2009\
0\\#p MP2(FC)/6-31+g(2d,p) scf=tight\\et1sp_1\\0,1\P\C,1,1.87473466\H,
2,1.10087648,1,110.12782033\H,2,1.09953989,1,106.58713051,3,115.531324
02,0\C,1,1.86588604,2,99.02597506,4,64.22728603,0\H,5,1.09692999,1,109
.621272,2,-70.02941545,0\H,5,1.09892595,1,112.76132778,2,51.13106286,0
\H,5,1.0968843,1,109.56054864,2,172.16622852,0\C,1,1.86637929,5,99.044
27849,2,-101.82700775,0\H,9,1.09694195,1,109.33968854,5,-69.28943909,0
\H,9,1.09907875,1,112.63537334,5,51.52403874,0\H,9,1.09642156,1,110.01
142022,5,172.99703478,0\C,2,1.53805912,1,113.10675864,5,-175.36131676,
0\H,13,1.0970537,2,111.16428694,1,-56.42123347,0\H,13,1.09727652,2,111
.52731246,1,63.80977409,0\H,13,1.09749966,2,110.92099068,1,-176.481755
35,0\\Version=AM64L-G03RevD.01\State=1-A\HF=-498.6266327\MP2=-499.3809
249\RMSD=6.263e-09\Thermal=0.\PG=C01 [X(C4H11P1)]\\@

74-Me$^+$

1\1\GINC-AZAZEL\SP\RMP2-FC\6-31+G(2d,p)\C5H14P1(1+)\CHRISTOPH\22-Sep-2
009\0\\#p MP2(FC)/6-31+g(2d,p) scf=tight\\et1mesp_1\\1,1\P\C,1,1.81782

81\H,2,1.09588962,1,110.02717574\H,2,1.09529239,1,110.27953614,3,120.2
6598378,0\H,2,1.09581267,1,109.84633999,3,-119.85182176,0\C,1,1.817822
08,2,109.10764219,4,-59.64342403,0\H,6,1.09581232,1,109.84644349,2,-60
.1900979,0\H,6,1.09529313,1,110.27943008,2,59.69239063,0\H,6,1.0958875
6,1,110.02703661,2,179.95810118,0\C,1,1.81827374,6,109.01872516,2,118.
96159999,0\H,10,1.0958607,1,110.07292727,6,-59.46568598,0\H,10,1.09583
998,1,110.03722936,6,60.53410125,0\H,10,1.09583764,1,110.03777581,6,-1
79.46567251,0\C,1,1.82928173,6,110.31411088,10,119.70633914,0\H,14,1.0
9882666,1,106.57388012,6,-176.53645378,0\H,14,1.09882185,1,106.5738092
5,6,-62.87195617,0\C,14,1.54185051,1,114.80162114,6,60.29521877,0\H,17
,1.09534835,14,112.03624246,1,-61.30683071,0\H,17,1.09534751,14,112.03
674609,1,61.30353588,0\H,17,1.09461811,14,108.81632929,1,179.99806124,
0\\Version=AM64L-G03RevD.01\State=1-A\HF=-538.0733915\MP2=-538.9718177
\RMSD=8.189e-09\Thermal=0.\PG=C01 [X(C5H14P1)]\\@

75
1\1\GINC-NODE15\SP\RMP2-FC\6-31+G(2d,p)\C7H13P1\ZIP07\03-Sep-2009\0\\#
p MP2(FC)/6-31+g(2d,p) scf=tight\\pro2sp_1\\0,1\P\C,1,1.86568983\H,2,1
.09652018,1,109.4229198\H,2,1.09652109,1,109.4236529,3,-118.23339338,0
\H,2,1.09834847,1,112.69836092,3,120.88312737,0\C,1,1.83996413,2,99.66
813627,3,-69.51828807,0\C,6,1.52711366,1,118.86810674,2,93.42067746,0\
C,7,1.50855759,6,60.289059,1,108.79395927,0\H,6,1.09255707,1,116.34123
741,2,-51.70074871,0\H,7,1.08775763,6,117.57268244,1,-142.37572756,0\H
,7,1.08846,6,117.91551956,1,1.01666061,0\H,8,1.08820396,7,117.8266867,
6,-108.01887409,0\H,8,1.08777144,7,118.49583164,6,107.27384859,0\C,1,1
.83996673,6,100.72169981,8,-95.81800537,0\C,14,1.52711255,1,118.867221
33,6,164.73065095,0\C,15,1.50855785,14,60.28902026,1,-108.7955107,0\H,
14,1.09255587,1,116.3411648,6,-50.14890002,0\H,15,1.08846168,14,117.91
555998,1,-1.01871141,0\H,15,1.08775742,14,117.57293043,1,142.37368779,
0\H,16,1.08820447,15,117.82687792,14,108.0188827,0\H,16,1.08777042,15,
118.49559335,14,-107.27396742,0\\Version=AM64L-G03RevD.01\State=1-A\HF
=-613.3346082\MP2=-614.5074994\RMSD=3.566e-09\Thermal=0.\PG=C01 [X(C7H
13P1)]\\@

75-Me$^+$
1\1\GINC-NODE3\SP\RMP2-FC\6-31+G(2d,p)\C8H16P1(1+)\ZIP07\03-Sep-2009\0
\\#p MP2(FC)/6-31+g(2d,p) scf=tight\\pro2mesp_2\\1,1\P\C,1,1.79631008\
C,2,1.53310381,1,122.05109298\C,3,1.50078492,2,60.67258701,1,110.34150
435,0\H,2,1.09059306,1,111.99868331,4,144.92702047,0\H,3,1.0864384,2,1
15.88944713,1,-139.36084729,0\H,3,1.08733748,2,118.8790259,1,2.6673365
4,0\H,4,1.08644718,3,118.99545366,2,105.37210628,0\H,4,1.0870452,3,117
.70503839,2,-108.60403263,0\C,1,1.79691649,2,111.18184002,4,36.0874655
3,0\C,10,1.53335585,1,122.96606224,2,-86.51047755,0\C,11,1.50001858,10
,60.77540151,1,-109.57030845,0\H,10,1.08993621,1,112.05831098,2,60.034
91869,0\H,11,1.0874027,10,118.76829322,1,-1.76563364,0\H,11,1.08665149
,10,116.04532495,1,140.33936845,0\H,12,1.08731953,11,118.03166002,10,1
09.20560817,0\H,12,1.08642206,11,119.01848549,10,-105.11447142,0\C,1,1
.81986951,2,109.10875105,4,156.06130852,0\H,18,1.09542235,1,109.673978
07,2,-61.22342449,0\H,18,1.09583612,1,110.13313235,2,58.44333051,0\H,1
8,1.09552818,1,109.96058706,2,178.80449711,0\C,1,1.81958239,2,108.8381
461,4,-86.11301765,0\H,22,1.09510936,1,110.9277108,2,-177.64656147,0\H
,22,1.09604018,1,108.86448059,2,-58.34041241,0\H,22,1.09480719,1,110.4
6498483,2,60.60154534,0\\Version=AM64L-G03RevD.01\State=1-A\HF=-652.78
10415\MP2=-654.0996895\RMSD=2.379e-09\Thermal=0.\PG=C01 [X(C8H16P1)]\\
@

76
1\1\GINC-CALYPSO\SP\RMP2-FC\6-31+G(2d,p)\C6H15P1\CHRISTOPH\07-May-2009

\0\\#p MP2(FC)/6-31+g(2d,p) scf=tight\\ibdmpspc002\\0,1\P\C,1,1.865550
67\H,2,1.09691497,1,109.64176816\H,2,1.09689945,1,109.54266877,3,117.8
1401734,0\H,2,1.09896714,1,112.76586469,4,121.08007926,0\C,1,1.8663174
5,2,99.08961206,4,-70.09902892,0\H,6,1.09708377,1,109.30567102,2,68.57
06116,0\H,6,1.0965064,1,110.06772534,2,-173.71401457,0\H,6,1.09914315,
1,112.68334821,2,-52.27794779,0\C,1,1.87791225,2,98.44150002,6,-101.42
508949,0\H,10,1.1019066,1,108.96111161,2,52.56017043,0\H,10,1.10163114
,1,106.98272821,2,-62.35217786,0\C,10,1.54776899,1,115.30044584,2,176.
34135376,0\H,13,1.10186854,10,108.20701113,1,-55.51267273,0\C,13,1.540
33416,10,110.17371919,1,-172.70017152,0\H,15,1.09803103,13,111.0938574
6,10,177.55235253,0\H,15,1.09909909,13,110.74700438,10,-62.80995851,0\
H,15,1.09782734,13,111.43483576,10,57.14566951,0\C,13,1.53924024,10,11
2.17805753,1,63.61940693,0\H,19,1.09627316,13,111.76201792,10,-58.4198
3701,0\H,19,1.0993193,13,110.52424006,10,61.47317397,0\H,19,1.09776965
,13,110.8460398,10,-179.19062943,0\\Version=AM64L-G03RevD.01\State=1-A
\HF=-576.7032461\MP2=-577.7676027\RMSD=3.714e-09\Thermal=0.\PG=C01 [X(
C6H15P1)]\\@

76-Me⁺

1\1\GINC-AZAZEL\SP\RMP2-FC\6-31+G(2d,p)\C7H18P1(1+)\CHRISTOPH\07-May-2
009\0\\#p MP2(FC)/6-31+g(2d,p) scf=tight\\ibdmpmespc002\\1,1\C\H,1,1.0
9594454\H,1,1.09580867,2,108.83934462\H,1,1.09569914,3,108.91919178,2,
118.52487309,0\C,1,2.95189734,4,89.6634968,2,150.58837587,0\H,5,1.0958
6648,1,88.68529734,4,105.9461408,0\H,5,1.09494246,1,146.24302558,4,-13
0.73727621,0\H,5,1.09578027,1,91.12149266,4,-2.90540277,0\C,1,2.956094
64,5,61.23919243,7,-39.72794646,0\H,9,1.09978526,1,76.01018312,5,-108.
29405932,0\H,9,1.10040258,1,95.35275505,5,146.04085538,0\C,9,1.5546290
9,1,149.95893489,5,-3.53412781,0\H,12,1.10001443,9,107.89725703,1,-33.
21793121,0\C,12,1.54118004,9,108.1611987,1,-149.60136945,0\H,14,1.0956
0749,12,110.10529058,9,176.92047309,0\H,14,1.09776077,12,111.05898168,
9,-63.77672118,0\H,14,1.09663681,12,111.73768452,9,57.13569264,0\C,12,
1.538599,9,113.32611011,1,87.15430603,0\H,18,1.0981553,12,112.84316743
,9,-66.22295864,0\H,18,1.098143,12,111.2851748,9,55.76809244,0\H,18,1.
0954525,12,110.01289691,9,174.78415084,0\P,5,1.81856052,1,35.80111546,
9,-35.58641647,0\C,22,1.81991602,5,109.52411124,1,-117.07857235,0\H,23
,1.09594376,22,109.59778564,5,179.17030692,0\H,23,1.09373828,22,110.96
6888,5,-60.53362731,0\H,23,1.09607219,22,109.47466401,5,59.89603731,0\
\Version=AM64L-G03RevD.01\State=1-A\HF=-616.1502372\MP2=-617.3591301\R
MSD=4.413e-09\Thermal=0.\PG=C01 [X(C7H18P1)]\\@

77

1\1\GINC-CIPCLU06\SP\RMP2-FC\6-31+G(2d,p)\C5H13P1\C4371\28-Oct-2009\0\
\#P MP2(FC)/6-31+G(2d,p) scf=tight\\i1sp_1\\0,1\P\C,1,1.88701968\H,2,1
.1023286,1,108.10988122\C,1,1.86767357,2,100.25984846,3,-50.22113972,0
\H,4,1.09908572,1,112.77298443,2,47.35158714,0\C,1,1.86767397,4,98.212
21432,2,-102.05998254,0\H,6,1.09594541,1,110.3652333,4,176.57788079,0\
C,2,1.54063004,1,110.3776785,4,-169.04968022,0\H,8,1.09656325,2,112.18
219436,1,61.96368022,0\H,8,1.09872876,2,110.40113536,1,-178.13822624,0
\H,8,1.09830463,2,110.95221922,1,-58.83945905,0\C,2,1.54062766,1,110.3
8315527,4,68.61177208,0\H,12,1.0965585,2,112.1834988,1,-61.96287234,0\
H,12,1.09830765,2,110.95217129,1,58.84172194,0\H,12,1.09872724,2,110.4
0029448,1,178.13890689,0\H,4,1.09705545,1,108.94359738,6,65.75030869,0
\H,4,1.09594239,1,110.36617422,6,-176.58731328,0\H,6,1.09908337,1,112.
77233475,4,54.70088362,0\H,6,1.09705455,1,108.9450973,4,-65.75902559,0
\\Version=AM64L-G03RevD.01\State=1-A\HF=-537.6631186\MP2=-538.5747735\
RMSD=9.412e-09\Thermal=0.\PG=C01 [X(C5H13P1)]\\@

77-Me⁺

1\1\GINC-CIPCLU06\SP\RMP2-FC\6-31+G(2d,p)\C6H16P1(1+)\C4371\28-Oct-200
9\0\\#P MP2(FC)/6-31+G(2d,p) scf=tight\\i1mesp_1\\1,1\C\H,1,1.09489388
\C,1,2.94536701,2,146.38721313\H,3,1.09489371,1,146.38710876,2,0.00071
807,0\C,3,3.00311717,1,60.63424544,2,41.21141925,0\H,5,1.10108153,3,85
.14841533,1,87.26708105,0\C,5,1.5448515,3,91.06125724,1,-163.95964268,
0\H,7,1.09558576,5,108.57280598,3,-147.69576068,0\H,7,1.09649995,5,111
.72710488,3,94.13712158,0\H,7,1.09550296,5,112.84131536,3,-28.89109465
,0\C,5,1.54485077,3,146.19565688,1,-29.75955838,0\H,11,1.09558479,5,10
8.57284631,3,172.8114047,0\H,11,1.09650047,5,111.72725246,3,-69.021576
75,0\H,11,1.09550132,5,112.84125014,3,54.00670506,0\P,1,1.81995858,3,3
5.98363286,5,34.91780737,0\C,15,1.81979466,1,108.67341363,3,117.738668
55,0\H,16,1.09526498,15,110.33337491,1,-178.36056274,0\H,16,1.09594684
,15,109.56411344,1,-58.66135382,0\H,16,1.09526538,15,110.33387357,1,61
.03811958,0\H,3,1.09586072,1,88.08713858,15,-128.36347842,0\H,3,1.0959
8086,1,91.65766562,15,122.87622791,0\H,1,1.09598109,15,110.01180211,16
,-178.95256721,0\H,1,1.09586078,15,109.72162827,16,61.38457202,0\\Vers
ion=AM64L-G03RevD.01\State=1-A\HF=-577.1110763\MP2=-578.1667423\RMSD=2
.955e-09\Thermal=0.\PG=C01 [X(C6H16P1)]\\@

78

1\1\GINC-HAENSEL\SP\RMP2-FC\6-31+G(2d,p)\C5H13P1\CHRISTOPH\25-Sep-2009
\0\\#p MP2(FC)/6-31+g(2d,p) scf=tight\\pr1sp_2\\0,1\P\C,1,1.8658942\H,
2,1.09686937,1,109.62714418\H,2,1.09690918,1,109.52881792,3,117.823611
64,0\H,2,1.0988581,1,112.75217538,4,121.02737167,0\C,1,1.87408119,2,98
.92886327,3,70.96670068,0\H,6,1.10077701,1,106.56790586,2,-62.88438189
,0\H,6,1.10188298,1,110.22306053,2,52.54948633,0\C,1,1.86678406,2,99.0
2647601,6,101.98319949,0\H,9,1.0964929,1,110.00340713,2,-172.78297239,
0\H,9,1.09907996,1,112.68176051,2,-51.32133396,0\H,9,1.09696635,1,109.
32958625,2,69.5131561,0\C,6,1.53998934,1,113.6432214,2,176.81306356,0\
H,13,1.09925591,6,109.28721717,1,54.09070541,0\H,13,1.09963641,6,109.7
1052448,1,-62.08370709,0\C,13,1.53669567,6,112.59408324,1,176.10518149
,0\H,16,1.09735225,13,111.2782345,6,179.81864531,0\H,16,1.09808758,13,
111.16531031,6,59.64295199,0\H,16,1.09809382,13,111.00482109,6,-60.125
9627,0\\Version=IA32L-G03RevD.01\State=1-A\HF=-537.6657543\MP2=-538.57
28996\RMSD=6.610e-09\Thermal=0.\PG=C01 [X(C5H13P1)]\\@

78-Me$^+$

1\1\GINC-HAENSEL\SP\RMP2-FC\6-31+G(2d,p)\C6H16P1(1+)\CHRISTOPH\25-Sep-
2009\0\\#p MP2(FC)/6-31+g(2d,p) scf=tight\\pr1mesp_1\\1,1\P\C,1,1.8182
5148\H,2,1.09579027,1,110.11958397\H,2,1.09578306,1,110.00484907,3,-12
0.0453136,0\H,2,1.09578061,1,109.99894939,4,-119.91348862,0\C,1,1.8183
8221,2,108.89315923,5,60.63550889,0\H,6,1.09583703,1,109.87820678,2,58
.29878151,0\H,6,1.09588666,1,109.97553414,2,-61.53585228,0\H,6,1.09516
901,1,110.28919277,2,178.25178881,0\C,1,1.81838638,2,108.9004368,6,118
.83465082,0\H,10,1.09583786,1,109.878552,2,-58.26202859,0\H,10,1.09588
312,1,109.97828271,2,61.57524328,0\H,10,1.09516634,1,110.28398099,2,-1
78.21291901,0\C,1,1.82812521,2,109.19857998,6,-120.5793965,0\H,14,1.09
977288,1,106.6727476,2,-56.82444209,0\H,14,1.09977739,1,106.671208,2,5
6.84728368,0\C,14,1.54689855,1,115.19809049,2,-179.98793902,0\H,17,1.0
9814568,14,109.8415555,1,58.78503101,0\H,17,1.09814622,14,109.84810777
,1,-58.78129642,0\C,17,1.53694586,14,111.07784579,1,179.99953891,0\H,2
0,1.09653375,17,111.28885274,14,-60.38247612,0\H,20,1.09653517,17,111.
28962139,14,60.40416977,0\H,20,1.0950212,17,110.16683435,14,-179.98887
97,0\\Version=IA32L-G03RevD.01\State=1-A\HF=-577.1138323\MP2=-578.1652
198\RMSD=2.398e-09\Thermal=0.\PG=C01 [X(C6H16P1)]\\@

79

1\1\GINC-NODE15\SP\RMP2-FC\6-31+G(2d,p)\C5H13P1\ZIP07\21-Sep-2009\0\\#

p MP2(FC)/6-31+g(2d,p) scf=tight\\et2sp_1\\0,1\P\C,1,1.86686944\H,2,1.
09658496,1,109.71732279\H,2,1.09658436,1,109.71752689,3,117.4749251,0\
H,2,1.09930069,1,112.75969024,3,-121.26231308,0\C,1,1.87516791,2,99.97
955591,4,-70.90735382,0\H,6,1.09957175,1,106.5350013,2,-164.96320329,0
\H,6,1.10081321,1,110.22667364,2,-49.46381518,0\C,1,1.87516974,2,99.97
940211,6,-100.72047685,0\H,9,1.10081308,1,110.2266287,2,49.45920851,0\
H,9,1.0995718,1,106.53530292,2,164.95861792,0\C,6,1.53807515,1,113.200
49958,2,74.64488449,0\H,12,1.09727442,6,111.56919932,1,-63.70033969,0\
H,12,1.09756664,6,110.89175661,1,176.60517176,0\H,12,1.09700885,6,111.
15816598,1,56.5501688,0\C,9,1.53807558,1,113.20016708,2,-74.64955639,0
\H,16,1.09756728,9,110.8918857,1,-176.6062394,0\H,16,1.09700884,9,111.
15798613,1,-56.55094204,0\H,16,1.0972754,9,111.56927671,1,63.69932825,
0\\Version=AM64L-G03RevD.01\State=1-A\HF=-537.6627723\MP2=-538.5708878
\RMSD=5.200e-09\Thermal=0.\PG=C01 [X(C5H13P1)]\\@

79-Me$^+$

1\1\GINC-NODE26\SP\RMP2-FC\6-31+G(2d,p)\C6H16P1(1+)\ZIP07\21-Sep-2009\
0\\#p MP2(FC)/6-31+g(2d,p) scf=tight\\et2mesp_1\\1,1\P\C,1,1.81925784\
H,2,1.09526636,1,110.32978673\H,2,1.09593106,1,110.07787947,3,120.2502
6015,0\H,2,1.09583192,1,109.94880088,4,119.85646444,0\C,1,1.83076989,2
,108.85967738,3,59.67258668,0\H,6,1.09872589,1,106.56213943,2,57.59408
163,0\H,6,1.09824782,1,106.79290774,2,-56.03337846,0\C,1,1.81925287,2,
108.59918199,6,119.57131084,0\H,9,1.09582989,1,109.95126888,2,59.33113
865,0\H,9,1.09526597,1,110.32878604,2,179.22620818,0\H,9,1.0959336,1,1
10.07606727,2,-60.52601707,0\C,1,1.83076335,9,108.86153258,2,-119.5737
337,0\H,13,1.09824838,1,106.79470805,9,-56.00575882,0\H,13,1.09872849,
1,106.56088533,9,57.62166981,0\C,13,1.5413668,1,114.55998252,9,-179.27
501596,0\H,16,1.09466904,13,108.94150938,1,-179.5965362,0\H,16,1.09555
357,13,112.07658868,1,-60.84570167,0\H,16,1.09561117,13,112.00208945,1
,61.61944905,0\C,6,1.54136458,1,114.55924311,9,61.90598753,0\H,20,1.09
555307,6,112.07513134,1,-60.87931157,0\H,20,1.09466808,6,108.94212585,
1,-179.63068232,0\H,20,1.09561311,6,112.00304828,1,61.58516308,0\\Vers
ion=AM64L-G03RevD.01\State=1-A\HF=-577.1114892\MP2=-578.1643832\RMSD=6
.431e-09\Thermal=0.\PG=C01 [X(C6H16P1)]\\@

80

1\1\GINC-MAX\SP\RMP2-FC\6-31+G(2d,p)\C6H15P1\CHRISTOPH\28-Sep-2009\0\\
#p MP2(FC)/6-31+g(2d,p) scf=tight\\bu1sp_2\\0,1\P\C,1,1.86587316\H,2,1
.09687749,1,109.53931198\H,2,1.09689771,1,109.61678498,3,-117.79663972
,0\H,2,1.09882531,1,112.78668604,3,121.04981435,0\C,1,1.87493273,2,98.
99793941,3,-171.9038374,0\H,6,1.10063794,1,106.71915665,2,-63.94978719
,0\H,6,1.10193069,1,110.11323775,2,51.53744336,0\C,1,1.86615488,2,99.0
5501762,6,101.87757151,0\H,9,1.09902665,1,112.6855838,2,-52.09190188,0
\H,9,1.09638491,1,110.00401993,2,-173.5878223,0\H,9,1.09702153,1,109.2
8372604,2,68.74035517,0\C,6,1.53983699,1,113.4554796,2,175.57474078,0\
H,13,1.10028797,6,109.38600896,1,55.45515656,0\H,13,1.10069794,6,109.7
8062828,1,-60.71847009,0\C,13,1.53895916,6,113.04159613,1,177.58429666
,0\H,16,1.10047068,13,109.21537341,6,57.59130927,0\H,16,1.10039636,13,
109.18434325,6,-58.13162516,0\C,16,1.53571153,13,112.93758671,6,179.72
067725,0\H,19,1.09713754,16,111.3726536,13,-179.81790899,0\H,19,1.0980
606,16,111.10846205,13,60.03110607,0\H,19,1.09821911,16,111.10135589,1
3,-59.70398097,0\\Version=IA32L-G03RevD.01\State=1-A\HF=-576.7048365\M
P2=-577.7646553\RMSD=8.102e-09\Thermal=0.\PG=C01 [X(C6H15P1)]\\@

80-Me$^+$

1\1\GINC-AZAZEL\SP\RMP2-FC\6-31+G(2d,p)\C7H18P1(1+)\CHRISTOPH\28-Sep-2
009\0\\#p MP2(FC)/6-31+g(2d,p) scf=tight\\bu1mesp_1\\1,1\P\C,1,1.81891
298\H,2,1.09575912,1,109.96943331\H,2,1.0958256,1,110.19434252,3,120.0

6821408,0\H,2,1.09576153,1,109.96216037,3,-119.86995488,0\C,1,1.818134
56,2,108.90768319,3,-60.70514849,0\H,6,1.09585006,1,110.01670178,2,61.
49387206,0\H,6,1.09577202,1,109.88621456,2,-58.39063888,0\H,6,1.095125
98,1,110.18731615,2,-178.33746811,0\C,1,1.82751569,6,110.39912555,2,-1
19.89828669,0\H,10,1.0997372,1,106.63845954,6,176.56418171,0\H,10,1.09
973369,1,106.64113378,6,62.91441527,0\C,1,1.81815749,6,108.99734425,2,
118.68805739,0\H,13,1.09585234,1,110.01106178,6,179.88605553,0\H,13,1.
09512605,1,110.18754661,6,59.72123851,0\H,13,1.09577037,1,109.8902108,
6,-60.2292963,0\C,10,1.54621795,1,115.23043123,6,-60.2616938,0\H,17,1.
09908766,10,110.01419584,1,58.8655635,0\H,17,1.09909256,10,110.0183200
2,1,-58.83406267,0\C,17,1.54144705,10,111.30571414,1,-179.98405537,0\H
,20,1.09936555,17,109.13023138,10,58.04923439,0\H,20,1.09936598,17,109
.12954199,10,-58.04445937,0\C,20,1.53493008,17,112.25807856,10,-179.99
679581,0\H,23,1.09721817,20,111.32916403,17,60.30894196,0\H,23,1.09722
253,20,111.32897003,17,-60.29125182,0\H,23,1.09527196,20,110.56473092,
17,-179.99047983,0\\Version=AM64L-G03RevD.01\State=1-A\HF=-616.1537246
\MP2=-617.3577728\RMSD=3.619e-09\Thermal=0.\PG=C01 [X(C7H18P1)]\\@

81

1\1\GINC-NODE7\SP\RMP2-FC\6-31+G(2d,p)\C6H13P1\ZIP07\03-Sep-2009\0\\#p
MP2(FC)/6-31+g(2d,p) scf=tight\\bu1sp_1\\0,1\P\C,1,1.8667253\H,2,1.09
882835,1,112.69644935\H,2,1.09696006,1,109.65906384,3,-121.10878593,0\
H,2,1.09684095,1,109.51815203,4,-117.83451469,0\C,1,1.86672662,2,99.41
387949,5,170.92406916,0\H,6,1.09882782,1,112.69645456,2,-49.86467781,0
\H,6,1.09684139,1,109.51799659,2,-170.92126762,0\H,6,1.09695955,1,109.
65907642,2,71.24432433,0\C,1,1.8638889,2,99.57149282,6,-101.47187814,0
\C,10,1.56341119,1,117.98141394,2,-77.40579854,0\C,11,1.55536231,10,88
.70253848,1,-139.04096036,0\C,12,1.55536312,11,88.65425355,10,18.21647
322,0\H,10,1.10062532,1,110.54524176,2,50.67131482,0\H,11,1.09765352,1
0,111.54915326,1,-26.28782148,0\H,11,1.09599946,10,117.28570127,1,99.9
6346257,0\H,12,1.09659589,11,111.74405795,10,-94.7119374,0\H,12,1.0960
6029,11,117.51193247,10,138.79648511,0\H,13,1.09765432,12,111.58923717
,11,94.49846913,0\H,13,1.09599836,12,117.90038406,11,-138.67195616,0\\
Version=AM64L-G03RevD.01\State=1-A\HF=-575.5019296\MP2=-576.5427059\RM
SD=9.796e-09\Thermal=0.\PG=C01 [X(C6H13P1)]\\@

81-Me$^+$

1\1\GINC-AZAZEL\SP\RMP2-FC\6-31+G(2d,p)\C7H16P1(1+)\CHRISTOPH\04-Sep-2
009\0\\#p MP2(FC)/6-31+g(2d,p) scf=tight\\bu1mesp_1\\1,1\P\C,1,1.82236
257\C,2,1.56699714,1,119.95875572\C,3,1.55658838,2,87.75194246,1,142.8
2999596,0\C,4,1.55658884,3,89.58214788,2,-18.57124445,0\H,2,1.09870905
,1,106.29374703,5,126.12085641,0\H,3,1.09353452,2,116.36101561,1,-96.6
0342619,0\H,3,1.09636267,2,112.77818284,1,30.28861378,0\H,4,1.09298963
,3,116.89789364,2,-138.80951813,0\H,4,1.09443662,3,111.7380258,2,94.74
579954,0\H,5,1.09353492,4,118.21129437,3,137.46647732,0\H,5,1.09636233
,4,111.61505132,3,-95.0831463,0\C,1,1.81909686,2,109.61563285,5,173.97
209641,0\H,13,1.09573736,1,109.94624256,2,60.69574173,0\H,13,1.0957864
,1,110.31731664,2,-179.17123178,0\H,13,1.09570567,1,109.82846599,2,-59
.0551443,0\C,1,1.81909642,13,109.17953308,2,-120.08574013,0\H,17,1.095
73852,1,109.94623777,13,59.39339405,0\H,17,1.0957066,1,109.82855589,13
,179.14415902,0\H,17,1.09578535,1,110.31736019,13,-60.73961271,0\C,1,1
.81865297,17,108.64384446,13,118.33128513,0\H,21,1.09597699,1,109.5490
3018,17,-59.32816284,0\H,21,1.0952466,1,110.40883967,17,60.34747097,0\
H,21,1.09524629,1,110.40860658,17,-179.00368835,0\\Version=AM64L-G03Re
vD.01\State=1-A\HF=-614.9512211\MP2=-616.1362263\RMSD=4.911e-09\Therma
l=0.\PG=C01 [X(C7H16P1)]\\@

82

1\1\GINC-NODE19\SP\RMP2-FC\6-31+G(2d,p)\C7H17P1\ZIP07\26-Sep-2009\0\\#

p MP2(FC)/6-31+g(2d,p) scf=tight\\pex1sp_1\\0,1\P\C,1,1.86611631\H,2,1
.09640489,1,109.99683072\H,2,1.09702424,1,109.3185412,3,117.6913652,0\
H,2,1.09906998,1,112.65841378,4,120.85401584,0\C,1,1.87504131,2,100.04
628809,3,72.78635662,0\H,6,1.10191905,1,109.99027852,2,49.04856206,0\H
,6,1.10055515,1,106.78037544,2,164.49579654,0\C,1,1.86605047,2,99.0425
9575,6,100.7704096,0\H,9,1.09889053,1,112.73449575,2,-51.43040057,0\H,
9,1.09687654,1,109.57790963,2,69.6306935,0\H,9,1.09690418,1,109.614891
35,2,-172.5337295,0\C,6,1.53998532,1,113.53271982,9,-175.82251039,0\H,
13,1.10017253,6,109.35535024,1,-56.27320007,0\H,13,1.1006405,6,109.746
74093,1,59.87714869,0\C,13,1.53879497,6,112.97893835,1,-178.46965145,0
\H,16,1.10149238,13,109.31851113,6,58.10670729,0\H,16,1.10153433,13,10
9.27295428,6,-57.63148437,0\C,16,1.53783052,13,113.32952367,6,-179.709
40423,0\H,19,1.10055828,16,109.20913135,13,57.28192767,0\H,19,1.100375
79,16,109.21849341,13,-58.40095436,0\C,19,1.53561951,16,113.07632885,1
3,179.39976666,0\H,22,1.09815294,19,111.12177297,16,59.82075554,0\H,22
,1.0971672,19,111.38752854,16,179.95619046,0\H,22,1.09819979,19,111.10
538471,16,-59.91337398,0\\Version=AM64L-G03RevD.01\State=1-A\HF=-615.7
438409\MP2=-616.9562467\RMSD=9.083e-09\Thermal=0.\PG=C01 [X(C7H17P1)]\
\@

82-Me$^+$

1\1\GINC-YIN\SP\RMP2-FC\6-31+G(2d,p)\C8H20P1(1+)\CHRISTOPH\28-Sep-2009
\0\\#p MP2(FC)/6-31+g(2d,p) scf=tight\\pex1mesp_1\\1,1\P\C,1,1.8182589
7\H,2,1.09505703,1,110.19393118\H,2,1.09575582,1,109.90667479,3,119.97
244188,0\H,2,1.0958358,1,109.9813288,4,119.87998639,0\C,1,1.81872337,2
,108.90045134,3,-178.38136777,0\H,6,1.09572747,1,109.94925491,2,-60.70
573705,0\H,6,1.09574934,1,110.21369486,2,59.37583564,0\H,6,1.09572898,
1,109.95080667,2,179.45844471,0\C,1,1.82765737,2,110.37090398,6,-119.9
2236108,0\H,10,1.09970907,1,106.62591583,2,176.5323645,0\H,10,1.099710
32,1,106.63116914,2,62.90112797,0\C,1,1.81826024,2,109.01874665,6,118.
70099619,0\H,13,1.09583847,1,109.97926742,2,179.78483624,0\H,13,1.0957
5343,1,109.91032401,2,-60.33458956,0\H,13,1.09506118,1,110.19242519,2,
59.64000759,0\C,10,1.54632586,1,115.19596974,2,-60.28359562,0\H,17,1.0
9901046,10,109.96973755,1,58.82641151,0\H,17,1.09900225,10,109.9631149
1,1,-58.82254498,0\C,17,1.54084085,10,111.32396939,1,-179.99608139,0\H
,20,1.10033324,17,109.24560282,10,-58.05233809,0\H,20,1.10032545,17,10
9.24172113,10,58.08509524,0\C,20,1.53843947,17,112.6342795,10,-179.986
35864,0\H,23,1.09998743,20,109.26744927,17,58.04292917,0\H,23,1.099976
68,20,109.26646369,17,-58.02473126,0\C,23,1.53535875,20,112.54641304,1
7,-179.99023843,0\H,26,1.09731799,23,111.24420043,20,60.19169315,0\H,2
6,1.09731382,23,111.24223547,20,-60.09358839,0\H,26,1.09563959,23,110.
83465193,20,-179.95179312,0\\Version=AM64L-G03RevD.01\State=1-A\HF=-65
5.1931819\MP2=-656.5497796\RMSD=4.326e-09\Thermal=0.\PG=C01 [X(C8H20P1
)]\\@

83

1\1\GINC-MORITZ\SP\RMP2-FC\6-31+G(2d,p)\C6H15P1\CHRISTOPH\05-Dec-2009\
0\\#p MP2(FC)/6-31+g(2d,p) scf=tight\\aib1sp_1\\0,1\P\C,1,1.86793301\H
,2,1.09709919,1,108.73967936\C,1,1.86824196,2,97.89240684,3,64.0078651
8,0\H,4,1.0957323,1,110.6146516,2,177.42610737,0\C,1,1.89003669,2,100.
38930421,4,102.27379271,0\H,6,1.10355126,1,108.0146506,2,-51.79784058,
0\C,6,1.54176667,1,109.73477432,2,66.58360427,0\H,8,1.09689605,6,111.2
232256,1,-179.15467063,0\H,8,1.09636707,6,111.80229239,1,-59.5156614,0
\H,8,1.0985949,6,110.87564218,1,60.88576306,0\C,6,1.54801253,1,109.908
16011,2,-169.9135447,0\H,12,1.10036182,6,108.95030029,1,-62.10905037,0
\H,12,1.0987786,6,109.35870548,1,53.60026608,0\C,12,1.53863508,6,114.3
2496467,1,174.99541237,0\H,15,1.09629464,12,112.09383797,6,64.45756868
,0\H,15,1.09841454,12,111.08900665,6,-56.23122393,0\H,15,1.09727795,12

,110.5671135,6,-175.73684203,0\H,2,1.09912658,1,112.83339585,4,-56.317
21783,0\H,2,1.09559655,1,110.52299523,4,-178.42782348,0\H,4,1.09710516
,1,108.77308056,2,-64.96260277,0\H,4,1.09904089,1,112.77586464,2,55.35
729152,0\\Version=IA32L-G03RevD.01\State=1-A\HF=-576.6995139\MP2=-577.
7655473\RMSD=9.771e-09\Thermal=0.\PG=C01 [X(C6H15P1)]\\@

83-Me$^+$

1\1\GINC-MORITZ\SP\RMP2-FC\6-31+G(2d,p)\C7H18P1(1+)\CHRISTOPH\05-Dec-2
009\0\\#p MP2(FC)/6-31+g(2d,p) scf=tight\\aib1mesp_1\\1,1\C\H,1,1.0945
0358\C,1,3.01528534,2,93.25141321\H,3,1.10217695,1,83.89693356,2,-111.
85890497,0\C,1,2.93745325,3,60.79336908,4,89.90714469,0\H,5,1.09590752
,1,86.56145972,3,164.97053308,0\C,3,1.55433371,1,91.8713124,5,-161.678
59829,0\H,7,1.09912462,3,109.32298747,1,97.16843009,0\H,7,1.09850353,3
,109.83712702,1,-19.85899231,0\C,3,1.54481708,1,144.84587244,5,-24.425
8683,0\H,10,1.0967874,3,111.65888456,1,-73.66118339,0\H,10,1.09538327,
3,112.56435423,1,49.02830888,0\H,10,1.09408918,3,109.26372147,1,167.59
930385,0\C,7,1.53846612,3,112.87028808,1,-140.77466114,0\H,14,1.094850
27,7,112.15419401,3,-64.99418185,0\H,14,1.09698851,7,111.29980768,3,56
.62261826,0\H,14,1.09516451,7,109.64770707,3,175.7569616,0\P,1,1.82085
488,5,36.23602131,3,-34.44523479,0\C,18,1.81968763,1,108.63582938,5,-1
17.22901646,0\H,19,1.09531075,18,110.23803813,1,179.3225414,0\H,19,1.0
9583945,18,109.66409737,1,59.62367177,0\H,19,1.09510477,18,110.2873160
1,1,-60.2107836,0\H,5,1.09456999,1,146.51256196,18,11.10532068,0\H,5,1
.09592722,1,92.88759742,18,-120.81136762,0\H,1,1.0959337,18,109.529104
34,19,-61.88080686,0\H,1,1.09590169,18,110.01858601,19,178.63227217,0\
\Version=IA32L-G03RevD.01\State=1-A\HF=-616.1490437\MP2=-617.3590384\R
MSD=4.826e-09\Thermal=0.\PG=C01 [X(C7H18P1)]\\@

84

1\1\GINC-CIPCLU05\SP\RMP2-FC\6-31+G(2d,p)\C8H19P1\C2175\04-Feb-2010\0\
\#p MP2(FC)/6-31+G(2d,p) scf=tight\\hey1sp_1\\0,1\P\C,1,1.86629619\H,2
,1.09700403,1,109.33468543\H,2,1.09639849,1,109.98119763,3,-117.707763
95,0\H,2,1.09905898,1,112.65512069,3,120.86004538,0\C,1,1.87495956,2,1
00.0512362,4,72.65674167,0\H,6,1.10193099,1,110.09184292,2,49.02275382
,0\H,6,1.10059686,1,106.76689171,2,164.53693442,0\C,6,1.53997272,1,113
.43196088,2,-74.94810002,0\H,9,1.10013811,6,109.34714779,1,-55.8343194
6,0\H,9,1.10056667,6,109.7271164,1,60.292789,0\C,9,1.53895509,6,113.00
325602,1,-178.02221619,0\H,12,1.10137953,9,109.26150805,6,58.2116504,0
\H,12,1.10145314,9,109.25257646,6,-57.5037501,0\C,12,1.5376896,9,113.2
6967558,6,-179.59854995,0\H,15,1.10165424,12,109.28192339,9,57.3315087
1,0\H,15,1.10145491,12,109.31848821,9,-58.36146305,0\C,1,1.86583836,2,
99.06107195,6,100.90649024,0\H,18,1.09880599,1,112.78984653,2,-51.1715
6044,0\H,18,1.09690353,1,109.54208001,2,69.88807113,0\H,18,1.096888,1,
109.60902073,2,-172.32134248,0\C,15,1.53765919,12,113.4608104,9,179.42
519085,0\H,22,1.10050342,15,109.24281444,12,57.90135367,0\H,22,1.10056
895,15,109.20176327,12,-57.77430364,0\C,22,1.53566039,15,113.07552507,
12,-179.91546141,0\H,25,1.09717668,22,111.41373766,15,179.86737638,0\H
,25,1.09821677,22,111.09252466,15,59.71563964,0\H,25,1.098176,22,111.1
0142722,15,-59.98027061,0\\Version=AM64L-G03RevD.01\State=1-A\HF=-654.
7828759\MP2=-656.1478124\RMSD=9.322e-09\Thermal=0.\PG=C01 [X(C8H19P1)]
\\@

84-Me$^+$

1\1\GINC-CIPCLU05\SP\RMP2-FC\6-31+G(2d,p)\C9H22P1(1+)\C2175\04-Feb-201
0\0\\#p MP2(FC)/6-31+G(2d,p) scf=tight\\hey1mesp_1\\1,1\P\C,1,1.827333
77\H,2,1.09971639,1,106.64759637\H,2,1.09971601,1,106.64745482,3,-113.
65897477,0\C,2,1.54649114,1,115.22319619,4,-123.17045295,0\H,5,1.09904
364,2,109.97362309,1,58.82177864,0\H,5,1.09904354,2,109.97382352,1,-58

.82319641,0\C,1,1.81907665,2,109.2629364,5,-179.99965355,0\H,8,1.09576
888,1,109.95993116,2,-59.9293174,0\H,8,1.09576932,1,109.96000243,2,59.
92633375,0\H,8,1.09583599,1,110.21060823,2,179.99852636,0\C,1,1.818181
03,8,108.88452565,2,120.65433441,0\H,12,1.09584807,1,110.0153672,8,-61
.52465724,0\H,12,1.095756,1,109.90338201,8,58.37885218,0\H,12,1.095136
42,1,110.16422207,8,178.33562459,0\C,1,1.81818136,12,108.96814892,8,-1
18.6390032,0\H,16,1.09575578,1,109.90340876,12,60.26053993,0\H,16,1.09
584852,1,110.01533048,12,-179.83598284,0\H,16,1.09513685,1,110.1642387
6,12,-59.6963178,0\C,5,1.54098753,2,111.29198828,1,179.9992169,0\H,20,
1.10030659,5,109.20032092,2,58.04877225,0\H,20,1.10030674,5,109.200153
75,2,-58.04648478,0\C,20,1.53800675,5,112.62695045,2,-179.99866738,0\H
,23,1.10098304,20,109.39070974,5,58.06910406,0\H,23,1.10098323,20,109.
39070588,5,-58.07008244,0\C,23,1.53849736,20,112.87453387,5,179.999471
67,0\H,26,1.10002392,23,109.20355134,20,57.93379988,0\H,26,1.10002285,
23,109.20357003,20,-57.9327032,0\C,26,1.53516224,23,112.73655688,20,-1
79.99947073,0\H,29,1.09766367,26,111.20990106,23,60.06140951,0\H,29,1.
09594553,26,111.0212006,23,-180.,0\H,29,1.09766262,26,111.20997263,23,
-60.06115367,0\\Version=AM64L-G03RevD.01\State=1-A\HF=-694.2324625\MP2
=-695.7416058\RMSD=4.545e-09\Thermal=0.\PG=C01 [X(C9H22P1)]\\@

85

1\1\GINC-MAX\SP\RMP2-FC\6-31+G(2d,p)\C9H21P1\CHRISTOPH\11-Feb-2010\0\\
#p MP2(FC)/6-31+g(2d,p) scf=tight\\heo1sp_1\\0,1\P\C,1,1.86592306\H,2,
1.09687489,1,109.61415127\H,2,1.098822,1,112.74700614,3,121.12440816,0
\H,2,1.09688804,1,109.56208008,3,-117.82296945,0\C,1,1.87475721,2,98.9
535841,3,-70.67918666,0\H,6,1.1019158,1,110.06989919,2,-52.35004647,0\
H,6,1.1006029,1,106.73812862,2,63.1342092,0\C,1,1.86642351,2,99.039776
2,6,-101.78291545,0\H,9,1.09642655,1,109.98490911,2,173.3828178,0\H,9,
1.0970051,1,109.35310094,2,-68.89481881,0\H,9,1.09909535,1,112.6555887
9,2,51.96499434,0\C,6,1.53994346,1,113.47502826,2,-176.36056971,0\H,13
,1.10014998,6,109.34745476,1,-55.72084414,0\H,13,1.10061015,6,109.7410
2432,1,60.41740045,0\C,13,1.53894544,6,112.97612264,1,-177.89070741,0\
H,16,1.10136814,13,109.25501034,6,58.08387422,0\H,16,1.10141324,13,109
.24948846,6,-57.62172326,0\C,16,1.53784737,13,113.27874368,6,-179.7312
8917,0\H,19,1.10135129,16,109.2731103,13,-58.41830837,0\H,19,1.1015609
8,16,109.23835486,13,57.23971059,0\C,19,1.5374919,16,113.43496643,13,1
79.34265269,0\H,22,1.10159827,19,109.35606147,16,57.86941613,0\H,22,1.
10162774,19,109.29508556,16,-57.83962452,0\C,22,1.53773466,19,113.4391
5279,16,-179.9556193,0\H,25,1.10059677,22,109.19969547,19,57.48941069,
0\H,25,1.10050501,22,109.21220571,19,-58.15327917,0\C,25,1.53554549,22
,113.11909187,19,179.65648552,0\H,28,1.09822098,25,111.12473497,22,59.
87842002,0\H,28,1.09820792,25,111.10732118,22,-59.83602078,0\H,28,1.09
721326,25,111.41093784,22,-179.98018539,0\\Version=IA32L-G03RevD.01\St
ate=1-A\HF=-693.8219075\MP2=-695.3393826\RMSD=9.186e-09\Thermal=0.\PG=
C01 [X(C9H21P1)]\\@

85-Me$^+$

1\1\GINC-CIPCLU07\SP\RMP2-FC\6-31+G(2d,p)\C10H24P1(1+)\C2175\08-Feb-20
10\0\\#p MP2(FC)/6-31+G(2d,p) scf=tight\\heo1mesp_55\\1,1\C\H,1,1.0951
1694\H,1,1.09583679,2,108.95911197\H,1,1.09575371,2,108.87055389,3,-11
8.64920253,0\C,1,2.99342857,2,91.59612693,4,-150.85206212,0\H,5,1.0997
1719,1,141.25996554,2,129.48900333,0\H,5,1.09971483,1,89.25556611,2,-1
14.3158518,0\C,1,2.95934022,5,59.93668592,7,85.87386327,0\H,8,1.095754
95,1,145.48486891,5,35.92832161,0\H,8,1.0958109,1,90.17406712,5,161.13
212398,0\H,8,1.09575075,1,90.61395562,5,-89.94940293,0\C,5,1.54645052,
1,95.42635192,8,-163.47266323,0\H,12,1.09905132,5,109.97005459,1,88.62
392172,0\H,12,1.09905448,5,109.97131711,1,-29.01387594,0\C,12,1.540995

59,5,111.29829987,1,-150.19468036,0\H,15,1.10029342,12,109.19805143,5,
58.06348277,0\H,15,1.10029667,12,109.19938491,5,-58.02265054,0\C,15,1.
53810328,12,112.62945163,5,-179.9802765,0\H,18,1.10094204,15,109.34297
543,12,-58.04591753,0\H,18,1.10094188,15,109.34288213,12,58.0393947,0\
C,18,1.53818086,15,112.87293825,12,179.9967639,0\H,21,1.10104678,18,10
9.31874701,15,-57.95669227,0\H,21,1.10104621,18,109.31886696,15,57.956
01609,0\C,21,1.53793037,18,113.10294893,15,180.,0\H,24,1.10027021,21,1
09.22501857,18,57.90060652,0\H,24,1.10026994,21,109.22484012,18,-57.92
052289,0\C,24,1.53528239,21,112.86083366,18,179.99064712,0\H,27,1.0977
3852,24,111.19151497,21,-60.01660975,0\H,27,1.0962114,24,111.1202383,2
1,179.99448773,0\H,27,1.09773892,24,111.19127276,21,60.00577706,0\P,1,
1.818243,8,35.55741949,5,-34.94395982,0\C,31,1.81825558,1,108.96178642
,8,-118.66376554,0\H,32,1.09584047,31,110.01055079,1,-179.71133149,0\H
,32,1.09511732,31,110.14997587,1,-59.58388785,0\H,32,1.09574915,31,109
.91556411,1,60.37578582,0\\Version=AM64L-G03RevD.01\State=1-A\HF=-733.
2716549\MP2=-734.9333398\RMSD=4.655e-09\Thermal=0.\PG=C01 [X(C10H24P1)
]\\@

86

1\1\GINC-CIPCLU05\SP\RMP2-FC\6-31+G(2d,p)\C10H23P1\C2175\23-Feb-2010\0
\\#p MP2(FC)/6-31+G(2d,p) scf=tight\\oct1sp_1\\0,1\P\C,1,1.8658472\H,2
,1.09686803,1,109.60999628\H,2,1.09874857,1,112.77154289,3,121.1485874
8,0\H,2,1.0969009,1,109.54221485,3,-117.80304053,0\C,1,1.86648759,2,99
.06101221,3,-172.31026632,0\H,6,1.09700143,1,109.35259202,2,-69.017677
23,0\H,6,1.09908229,1,112.67133577,2,51.84815279,0\H,6,1.09646352,1,10
9.96510468,2,173.27152307,0\C,1,1.87455943,2,99.01739407,6,101.8802368
4,0\H,10,1.10065005,1,106.65066003,2,63.01912446,0\H,10,2.17434954,1,9
8.11613459,2,-151.59016212,0\H,10,2.16875915,1,95.49122688,2,160.29826
222,0\C,10,1.5399505,1,113.45893977,2,-176.59691861,0\C,14,1.53898809,
10,112.96049929,1,-176.75937451,0\H,15,1.10141082,14,109.30396865,10,-
57.61228479,0\H,15,1.10137152,14,109.18334311,10,58.08611792,0\C,15,1.
53782178,14,113.29657462,10,-179.80126194,0\H,18,1.10133922,15,109.257
51948,14,-57.69538043,0\H,18,1.10148247,15,109.27254227,14,57.98092298
,0\C,18,1.53774623,15,113.39858609,14,-179.86305742,0\H,21,1.10148932,
18,109.2645918,15,57.89938539,0\H,21,1.10153597,18,109.27617779,15,-57
.76828277,0\C,21,1.53758833,18,113.42622828,15,-179.93930415,0\H,24,1.
10162495,21,109.30758086,18,57.90968348,0\H,24,1.10158173,21,109.30458
975,18,-57.77393578,0\C,24,1.53767315,21,113.47177127,18,-179.92819962
,0\H,27,1.10056865,24,109.21916052,21,-57.82895471,0\H,27,1.10052269,2
4,109.21265751,21,57.83226137,0\C,27,1.53565396,24,113.09807372,21,-17
9.99817164,0\H,30,1.09820912,27,111.0981855,24,-59.85600721,0\H,30,1.0
9820106,27,111.0964923,24,59.83710915,0\H,30,1.09720952,27,111.4283084
8,24,179.99190387,0\H,10,1.10189638,1,110.18481968,2,-52.47551745,0\\V
ersion=AM64L-G03RevD.01\State=1-A\HF=-732.8609446\MP2=-734.5309376\RMS
D=9.718e-09\Thermal=0.\PG=C01 [X(C10H23P1)]\\@

86-Me$^+$

1\1\GINC-YIN\SP\RMP2-FC\6-31+G(2d,p)\C11H26P1(1+)\CHRISTOPH\25-Feb-201
0\0\\#p MP2(FC)/6-31+g(2d,p) scf=tight\\oct1mesp_1\\1,1\C\H,1,1.095772
66\H,1,1.09577037,2,108.86017641\H,1,1.0958397,3,108.90850954,2,118.61
964766,0\C,1,2.95875137,3,90.60431057,2,-151.00868178,0\H,5,1.09574958
,1,89.41989167,3,-108.26203598,0\H,5,1.09584369,1,91.0223938,3,0.63391
915,0\H,5,1.09513759,1,145.72027261,3,127.72035209,0\C,1,2.97376415,5,
60.60964222,8,37.73277728,0\H,9,1.09970709,1,86.05240522,5,-161.549167
97,0\H,9,2.1820039,1,130.2376017,5,-3.01660024,0\H,9,2.18197662,1,130.
23127775,5,-67.10124699,0\C,9,1.54652916,1,150.49304353,5,-35.06443183
,0\C,13,1.54101366,9,111.28740368,1,-179.98915487,0\H,14,1.10028837,13
,109.19980102,9,-58.03703807,0\H,14,1.10028766,13,109.1988357,9,58.055

2034,0\C,14,1.53815999,13,112.62298753,9,-179.99077294,0\H,17,1.100902
1,14,109.34269959,13,-58.04582253,0\H,17,1.1009057,14,109.34320905,13,
58.04180392,0\C,17,1.53832405,14,112.85768987,13,179.99825906,0\H,20,1
.10097614,17,109.2688292,14,57.93712151,0\H,20,1.10097691,17,109.26864
343,14,-57.92811532,0\C,20,1.53769979,17,113.09840287,14,-179.99534937
,0\H,23,1.10129326,20,109.33656723,17,57.93467176,0\H,23,1.1012941,20,
109.33721885,17,-57.93300929,0\C,23,1.53793572,20,113.20958422,17,-179
.99915745,0\H,26,1.10030518,23,109.20567467,20,-57.87079833,0\H,26,1.1
0030459,23,109.20603191,20,57.87802152,0\C,26,1.53528247,23,112.915818
39,20,-179.99586485,0\H,29,1.0978892,26,111.16288974,23,-59.96164357,0
\H,29,1.09788527,26,111.16323857,23,59.97094729,0\H,29,1.09639483,26,1
11.20799888,23,-179.99510511,0\H,9,1.09971035,1,86.06774578,5,91.44848
709,0\P,5,1.81817056,1,35.57759772,9,34.93049744,0\C,34,1.81816916,5,1
08.96817398,1,118.60756475,0\H,35,1.09584324,34,110.01050464,5,179.797
76702,0\H,35,1.09574674,34,109.90071964,5,-60.30036424,0\H,35,1.095136
52,34,110.16739006,5,59.66198407,0\\Version=AM64L-G03RevD.01\State=1-A
\HF=-772.3107859\MP2=-774.1250066\RMSD=5.403e-09\Thermal=0.\PG=C01 [X(
C11H26P1)]\\@

87

1\1\GINC-CIPCLU04\SP\RMP2-FC\6-31+G(2d,p)\C9H21P1\C2175\22-Dec-2009\0\
\#p MP2(FC)/6-31+G(2d,p) scf=tight\\maih1sp_3\\0,1\P\C,1,1.86584056\H,
2,1.09687836,1,109.49736215\H,2,1.09901847,1,112.81947299,3,-121.09047
413,0\H,2,1.09692085,1,109.60441568,3,117.77011399,0\C,1,1.86700899,2,
99.03666221,3,70.05729854,0\H,6,1.09700677,1,109.29397179,2,-68.837924
69,0\H,6,1.09911101,1,112.6072839,2,51.94827554,0\H,6,1.09663549,1,110
.11134401,2,173.41211817,0\C,1,1.87740835,2,98.28059892,6,101.63573039
,0\H,10,1.10182096,1,108.90415722,2,-50.04580475,0\H,10,1.1004416,1,10
6.50174673,2,64.19161254,0\C,10,1.54942705,1,116.06889231,2,-173.49400
874,0\H,13,1.10302285,10,107.416792,1,62.41827453,0\C,13,1.55034575,10
,113.40299117,1,-54.78857117,0\H,15,1.09984729,13,108.59253724,10,60.4
2400709,0\H,15,1.10000325,13,108.6230495,10,175.0478315,0\C,13,1.54934
411,10,109.19490641,1,177.57314301,0\H,18,1.10026017,13,107.98400414,1
0,-52.60970646,0\H,18,1.10009712,13,109.52048502,10,62.15916468,0\C,15
,1.53890263,13,116.23213574,10,-61.88205451,0\H,21,1.09975907,15,110.1
4926913,13,-57.56555488,0\H,21,1.0991596,15,109.75589166,13,59.3808192
9,0\C,18,1.53669338,13,114.90263638,10,-174.14482354,0\H,24,1.09856437
,18,111.13490165,13,54.67582287,0\H,24,1.09726491,18,110.74000382,13,1
74.27393099,0\H,24,1.09684428,18,112.02831915,13,-65.80725456,0\C,21,1
.53653071,15,112.11771231,13,-179.38063246,0\H,28,1.09841585,21,111.15
217023,15,59.87761946,0\H,28,1.09726357,21,111.39805015,15,-179.933797
26,0\H,28,1.09789898,21,110.94765756,15,-59.84267865,0\\Version=AM64L-
G03RevD.01\State=1-A\HF=-693.8142044\MP2=-695.3414398\RMSD=4.876e-09\T
hermal=0.\PG=C01 [X(C9H21P1)]\\@

87-Me$^+$

1\1\GINC-CIPCLU10\SP\RMP2-FC\6-31+G(2d,p)\C10H24P1(1+)\C2175\19-Dec-20
09\0\\#p MP2(FC)/6-31+G(2d,p) scf=tight\\maih1mesp_2\\1,1\C\H,1,1.0957
9921\H,1,1.09589395,2,108.85872054\H,1,1.09564739,2,108.92889925,3,-11
8.50342525,0\C,1,2.94966977,4,89.58795395,3,-150.6042412,0\H,5,1.09581
566,1,88.82436365,4,-106.40914361,0\H,5,1.09582315,1,90.90762555,4,2.4
7794754,0\H,5,1.09487372,1,146.25372906,4,129.90281402,0\C,1,2.9547796
6,5,61.30752899,8,38.91427548,0\H,9,1.09987842,1,75.52851422,5,108.389
30641,0\H,9,1.09916136,1,95.41359465,5,-146.16319954,0\C,9,1.55631272,
1,149.60285164,5,5.22742589,0\H,12,1.10149066,9,107.28806002,1,31.1483
0725,0\C,12,1.54864082,9,114.04314014,1,-87.34947436,0\H,14,1.10291032
,12,109.55336387,9,61.90756887,0\H,14,1.09769019,12,107.6375186,9,174.
96221598,0\C,12,1.55145561,9,107.53152836,1,145.7026311,0\H,17,1.09969

141,12,108.14396301,9,-54.32970527,0\H,17,1.09923676,12,109.57245277,9
,60.88026742,0\C,14,1.53985613,12,116.62446294,9,-62.6572712,0\H,20,1.
09798342,14,109.55139376,12,-59.12376007,0\H,20,1.10152075,14,110.6747
7477,12,57.38316643,0\C,17,1.53582742,12,114.21525955,9,-175.60159573,
0\H,23,1.09551894,17,110.02862832,12,173.3511407,0\H,23,1.09602327,17,
112.20675594,12,-67.24754713,0\H,23,1.09762694,17,111.40397244,12,54.0
7567983,0\C,20,1.53661774,14,112.22253431,12,179.39383753,0\H,27,1.096
90695,20,111.10966549,14,59.7785289,0\H,27,1.095599,20,111.0415251,14,
179.67733798,0\H,27,1.09869275,20,111.52852922,14,-60.1856551,0\P,5,1.
81900466,1,35.88136904,9,35.57021815,0\C,31,1.82052829,5,109.2957386,1
,116.64925257,0\H,32,1.09580517,31,109.69734749,5,-178.88331106,0\H,32
,1.09615478,31,109.36151265,5,-59.61052918,0\H,32,1.09393987,31,110.97
398359,5,60.74649832,0\\Version=AM64L-G03RevD.01\State=1-A\HF=-733.263
0659\MP2=-734.9353093\RMSD=1.948e-09\Thermal=0.\PG=C01 [X(C10H24P1)]\\
@

88

1\1\GINC-NODE-25\SP\RMP2-FC\6-31+G(2d,p)\C9H21P1\ZIP07\20-May-2009\0\\
#p MP2(FC)/6-31+g(2d,p) scf=tight\\dipmpspc002\\0,1\P\C,1,1.87885201\H
,2,1.10139211,1,106.94876513\H,2,1.10179238,1,109.14398776,3,-114.9034
5253,0\C,1,1.86667683,2,98.49336925,3,56.80847345,0\H,5,1.09663569,1,1
09.80494973,2,170.51823829,0\H,5,1.09691627,1,109.44803443,2,-71.79991
826,0\H,5,1.09910718,1,112.78548873,2,49.27776733,0\C,1,1.87946107,5,9
9.53133349,2,-100.64436558,0\H,9,1.10199307,1,108.9654934,5,44.5485060
8,0\H,9,1.10136127,1,107.33877179,5,159.60460092,0\C,9,1.54761156,1,11
4.9880977,5,-79.27485004,0\H,12,1.10199327,9,108.32304984,1,52.6511432
2,0\C,12,1.53923134,9,111.98979618,1,-66.38757018,0\H,14,1.09939138,12
,110.51453674,9,-62.5788395,0\H,14,1.09624271,12,111.60799659,9,57.345
66204,0\H,14,1.09773097,12,110.88071805,9,178.08155487,0\C,12,1.540337
58,9,110.2414003,1,169.98357303,0\H,18,1.09780397,12,111.43000504,9,-5
8.350447,0\H,18,1.09915468,12,110.74799918,9,61.60751394,0\H,18,1.0980
0225,12,111.09026745,9,-178.75884957,0\C,2,1.54737445,1,115.04658082,5
,177.93870939,0\H,22,1.10202579,2,108.31557985,1,52.36493852,0\C,22,1.
5403271,2,110.24601477,1,169.7354148,0\H,24,1.09798298,22,111.11640133
,2,-178.81504211,0\H,24,1.09784231,22,111.42722889,2,-58.3751961,0\H,2
4,1.09916828,22,110.73592658,2,61.55747093,0\C,22,1.53918307,2,112.001
85785,1,-66.57574563,0\H,28,1.09772196,22,110.87670685,2,178.4255879,0
\H,28,1.09941996,22,110.54930227,2,-62.19377433,0\H,28,1.09619111,22,1
11.5746331,2,57.72398359,0\\Version=IA32L-G03RevD.01\State=1-A\HF=-693
.8160254\MP2=-695.3446901\RMSD=9.740e-09\Thermal=0.\PG=C01 [X(C9H21P1)
]\\@

88-Me$^+$

1\1\GINC-NODE-08\SP\RMP2-FC\6-31+G(2d,p)\C10H24P1(1+)\ZIP07\22-May-200
9\0\\#p MP2(FC)/6-31+g(2d,p) scf=tight\\dipmpmespc014\\1,1\P\C,1,1.823
89563\H,2,1.09597714,1,109.72655233\H,2,1.09598349,1,109.6050679,3,119
.40738124,0\H,2,1.09390407,1,110.92388951,4,120.36278321,0\C,1,1.82235
975,2,106.57210659,5,177.84882779,0\H,6,1.09597376,1,109.51939822,2,59
.40112837,0\H,6,1.09485655,1,110.49864316,2,179.24798318,0\H,6,1.09575
5,1,110.17728054,2,-60.25866071,0\C,1,1.83698606,6,109.8356394,2,116.7
5513013,0\H,10,1.09916789,1,107.17726084,6,165.92639299,0\H,10,1.09981
166,1,104.72712812,6,-81.31586492,0\C,1,1.83931089,6,107.43283487,2,-1
17.99194646,0\H,13,1.10086765,1,105.47787508,6,75.55844267,0\H,13,1.09
829102,1,105.8906657,6,-37.02896471,0\C,13,1.55387192,1,118.72020605,6
,-160.52304292,0\H,16,1.09939564,13,107.83612873,1,60.29823622,0\C,10,
1.55436194,1,117.90983573,6,40.57327922,0\H,18,1.09978363,10,107.97541
888,1,-55.25011906,0\C,18,1.5412085,10,108.34017361,1,-171.60637449,0\
H,20,1.09563466,18,110.19464347,10,177.91688364,0\H,20,1.09778714,18,1

11.07526513,10,-62.69772772,0\H,20,1.09663469,18,111.65081191,10,58.11
992943,0\C,18,1.53802883,10,113.20905794,1,65.09465112,0\H,24,1.098475
99,18,112.95158139,10,-61.76820966,0\H,24,1.09797975,18,111.07741931,1
0,59.85594841,0\H,24,1.09558117,18,110.09212145,10,178.74395433,0\C,16
,1.54112263,13,108.15422837,1,176.57297428,0\H,28,1.09566436,16,110.16
521819,13,-176.55535557,0\H,28,1.09673542,16,111.68509376,13,-56.74319
949,0\H,28,1.09777387,16,111.05896677,13,64.10372011,0\C,16,1.5387643,
13,113.44714204,1,-60.05742918,0\H,32,1.0983758,16,112.77588917,13,65.
93492948,0\H,32,1.09554613,16,110.01273177,13,-175.07570754,0\H,32,1.0
9830383,16,111.36532779,13,-56.04509629,0\\Version=IA32L-G03RevD.01\St
ate=1-A\HF=-733.264377\MP2=-734.9394991\RMSD=5.694e-09\Thermal=0.\PG=C
01 [X(C10H24P1)]\\@

89

1\1\GINC-NAUTILUS\SP\RMP2-FC\6-31+G(2d,p)\C18H15P1\CHRISTOPH\15-Jun-20
10\0\\#p MP2(FC)/6-31+g(2d,p) scf=tight\\pph3sp_24\\0,1\P\C,1,4.669176
37\C,2,1.39889904,1,58.39835457\C,3,1.39711021,2,120.03404393,1,0.0731
2915,0\C,4,1.4078275,3,120.86573775,2,-0.90074517,0\C,5,1.40613305,4,1
18.52754336,3,1.01520813,0\C,2,1.39811597,1,61.29395672,5,-11.92393355
,0\H,2,1.08782689,1,178.46559567,5,-174.4705313,0\H,3,1.08791198,2,120
.13611998,1,179.40626729,0\H,4,1.08886424,3,119.6438903,2,178.83797575
,0\H,6,1.08715107,5,119.80867374,4,179.24351849,0\H,7,1.08811467,2,120
.05164401,1,-179.56503553,0\C,1,4.66759923,5,102.79294547,4,-161.16960
373,0\C,13,1.39899325,1,58.34285654,5,88.08101099,0\C,14,1.39699231,13
,120.01747891,1,0.16092582,0\C,15,1.40790244,14,120.84580135,13,-0.940
33665,0\C,16,1.40577377,15,118.58358895,14,1.08615587,0\C,13,1.3982075
7,1,61.36936355,16,-9.4420441,0\H,13,1.08783035,1,178.42795474,16,-174
.97501821,0\H,14,1.08790929,13,120.13808228,1,179.49477039,0\H,15,1.08
886588,14,119.66807053,13,178.83597793,0\H,17,1.08732569,16,119.797829
67,15,179.25960058,0\H,18,1.08810966,13,120.05975813,1,-179.64312327,0
\C,1,4.66856397,16,103.01046513,15,-161.85612879,0\C,24,1.39905748,1,5
8.34665971,16,87.0345034,0\C,25,1.39686331,24,120.01994036,1,-0.003546
64,0\C,26,1.40817789,25,120.88422236,24,-0.92025126,0\C,27,1.40580857,
26,118.52755415,25,0.94511419,0\C,24,1.39800658,1,61.33530279,16,-92.6
8200797,0\H,24,1.08781863,1,178.4050661,16,107.46009467,0\H,25,1.08792
536,24,120.14345756,1,179.30720453,0\H,26,1.08888881,25,119.61952007,2
4,178.8662804,0\H,28,1.08719633,27,119.86158696,26,179.37968377,0\H,29
,1.08813364,24,120.05777099,1,-179.49503835,0\\Version=AM64L-G03RevD.0
1\State=1-A\HF=-1031.152198\MP2=-1033.8073038\RMSD=3.162e-09\Thermal=0
.\PG=C01 [X(C18H15P1)]\\@

89-Me^{+}

1\1\GINC-CIPCLU04\SP\RMP2-FC\6-31+G(2d,p)\C19H18P1(1+)\C2175\18-Nov-20
10\0\\#p MP2(FC)/6-31+G(2d,p) scf=tight\\pph3mesp_14\\1,1\P\C,1,1.8263
4514\H,2,1.09468083,1,110.09519271\H,2,1.09467537,1,109.81417934,3,-12
0.05098936,0\H,2,1.09465709,1,109.81030113,4,-119.81389781,0\C,1,4.604
83596,2,108.65773713,5,58.54188599,0\C,6,1.3979893,1,60.31060999,2,-13
3.99138219,0\C,7,1.39750596,6,120.16250798,1,0.34638876,0\C,8,1.406607
78,7,119.66926518,6,0.00883656,0\C,9,1.40973574,8,120.05344476,7,-0.30
655184,0\C,10,1.39486017,9,119.75441021,8,0.3578895,0\H,6,1.08686592,1
,179.7564295,9,151.81750997,0\H,7,1.08657289,6,120.22442214,1,-179.817
13847,0\H,8,1.08655858,7,119.8478221,6,179.74968497,0\H,10,1.08762418,
9,120.95506978,8,-179.19938879,0\H,11,1.08659261,10,119.7100328,9,179.
80778784,0\C,1,4.60508301,9,110.1771647,8,-16.23460442,0\C,17,1.400660
14,1,59.91828951,9,-68.57193443,0\C,18,1.39469956,17,120.06814151,1,-0
.06315613,0\C,19,1.41016329,18,119.73318545,17,-0.05285141,0\C,20,1.40
624379,19,120.07581639,18,0.28034134,0\C,21,1.39769843,20,119.66412212
,19,-0.2870397,0\H,17,1.08686949,1,179.70569766,9,-82.24936965,0\H,18,

1.08658914,17,120.21835111,1,-179.97769289,0\H,19,1.08787278,18,119.35
747954,17,179.50157312,0\H,21,1.08646294,20,120.48649545,19,179.971730
27,0\H,22,1.08658631,21,119.61988035,20,-179.83927762,0\C,1,4.60462791
,9,110.73737951,8,106.31340037,0\C,28,1.3978858,1,60.26580244,9,-14.31
192615,0\C,29,1.39741046,28,120.16126529,1,0.33207762,0\C,30,1.4065610
8,29,119.68441993,28,0.01943062,0\C,31,1.41004,30,120.0450087,29,-0.20
170608,0\C,32,1.39492119,31,119.7431895,30,0.22272893,0\H,28,1.0868747
2,1,179.77256272,31,157.30357277,0\H,29,1.08656294,28,120.2270055,1,-1
79.81881523,0\H,30,1.08660953,29,119.81233638,28,179.7199043,0\H,32,1.
08764811,31,120.94611096,30,-179.33252035,0\H,33,1.08659108,32,119.709
93263,31,179.82997158,0\\Version=AM64L-G03RevD.01\State=1-A\HF=-1070.6
030109\MP2=-1073.4019066\RMSD=7.764e-09\Thermal=0.\PG=C01 [X(C19H18P1)
]\\@

89-BH$^+$

1\1\GINC-NODE6\SP\RMP2-FC\6-31+G(2d,p)\C31H26P1(1+)\ZIP07\30-Jun-2010\
0\\#p MP2(FC)/6-31+G(2d,p) scf=tight\\pph3bhsp_3\\1,1\C\C,1,1.4053327\
C,2,1.40651823,1,118.58284412\C,3,1.39675402,2,120.7947195,1,0.9868872
7,0\C,4,1.39801166,3,120.15502233,2,-0.27854661,0\C,5,1.39781961,4,119
.53645677,3,-0.46329209,0\H,1,1.0855487,6,119.18054757,5,-178.2257295,
0\H,3,1.08723614,2,119.96623358,1,-178.41171319,0\H,4,1.08707595,3,119
.60018936,2,-179.50406295,0\H,5,1.08699914,4,120.22986629,3,-179.49830
868,0\H,6,1.08721189,5,120.14646471,4,-178.67012861,0\C,2,1.53601731,1
,121.94607592,6,-178.48246257,0\H,12,1.09965382,2,106.91656911,1,151.5
3957196,0\C,12,4.34392845,2,114.18338111,1,35.1196821,0\C,14,1.3986388
6,12,58.8121953,2,99.54541503,0\C,15,1.3969679,14,120.10790788,12,0.65
838344,0\C,16,1.40555638,15,120.7886953,14,-0.26697195,0\C,17,1.406119
87,16,118.69330684,15,-0.04755097,0\C,14,1.39769925,12,60.7910397,2,-8
0.81908354,0\H,14,1.08690107,12,178.92592481,2,75.27305078,0\H,15,1.08
703024,14,120.22905977,12,-179.40224167,0\H,16,1.08852397,15,119.51040
064,14,-179.89026595,0\H,18,1.08668705,17,120.55094039,16,179.90986989
,0\H,19,1.08716814,14,120.13905771,12,179.50319427,0\P,12,1.89493537,2
,112.17607547,1,-99.61710391,0\C,25,4.62172991,12,105.7969063,2,-58.17
509439,0\C,26,1.40082468,25,59.2675369,12,-63.57484648,0\C,27,1.394476
22,26,120.04314976,25,-0.35749131,0\C,28,1.41095554,27,120.06249185,26
,0.22507544,0\C,29,1.40402724,28,119.64806657,27,-0.90707212,0\C,26,1.
39661259,25,60.81174277,12,117.26544735,0\H,26,1.08694315,25,179.12870
22,12,-46.57550104,0\H,27,1.08682306,26,120.24031591,25,179.9966627,0\
H,28,1.08834269,27,119.30115437,26,-178.73297009,0\H,30,1.08553124,29,
120.64032441,28,-178.49246517,0\H,31,1.08669931,26,120.21714626,25,-17
9.93033307,0\C,25,4.6178035,12,113.07977947,2,64.36719482,0\C,37,1.399
54674,25,60.35454837,12,-56.99635806,0\C,38,1.39621438,37,120.31871045
,25,-0.00610284,0\C,39,1.41182858,38,119.71925378,37,0.02540441,0\C,40
,1.40814402,39,119.73267745,38,-0.05161492,0\C,41,1.39682749,40,119.93
228841,39,0.03147239,0\H,37,1.08699097,25,179.71950883,12,134.61265134
,0\H,38,1.08681385,37,120.20346627,25,-179.9429243,0\H,39,1.08559512,3
8,119.45625183,37,179.67158611,0\H,41,1.08586274,40,120.36654411,39,17
9.89035104,0\H,42,1.08670406,41,119.54522792,40,-179.92892476,0\C,25,4
.6283503,12,113.80752154,2,-172.49623494,0\C,48,1.39765163,25,60.98253
03,12,-33.6472026,0\C,49,1.39754435,48,120.48447242,25,0.59340257,0\C,
50,1.40893316,49,119.80947318,48,0.63313257,0\C,51,1.41172362,50,119.4
3803608,49,-0.82336035,0\C,52,1.395448,51,120.15379942,50,0.39410086,0
\H,48,1.08700683,25,178.82714731,12,175.58471069,0\H,49,1.08674834,48,
120.20802645,25,-179.3216227,0\H,50,1.08337093,49,119.24400356,48,-179
.36106311,0\H,52,1.08621434,51,120.52498411,50,-179.88525347,0\H,53,1.
08675763,52,119.54981618,51,179.95024111,0\\Version=AM64L-G03RevD.01\S
tate=1-A\HF=-1529.7106044\MP2=-1534.2121684\RMSD=9.160e-09\Thermal=0.\
PG=C01 [X(C31H26P1)]\\@

89-TT$^+$

1\1\GINC-EDDY\SP\RMP2-FC\6-31+G(2d,p)\C37H30P1(1+)\CHRISTOPH\19-Aug-20
11\0\\#p MP2(FC)/6-31+g(2d,p) scf=tight\\pph3ttsp_1\\1,1\P\C,1,4.64296
938\C,2,1.39925811,1,60.21041579\C,3,1.39559838,2,120.46094513,1,0.287
54918,0\C,4,1.41260622,3,120.04346846,2,0.3915045,0\C,5,1.40881517,4,1
19.12036431,3,-1.01576561,0\C,2,1.39661168,1,59.58830603,5,128.5438027
5,0\H,2,1.08737146,1,179.62435311,5,175.03751749,0\H,3,1.08723913,2,12
0.21995254,1,-179.49534505,0\H,4,1.0846737,3,119.14804904,2,-179.34968
141,0\H,6,1.08510659,5,120.64791895,4,-179.00039833,0\H,7,1.08716301,2
,120.30404376,1,179.43104837,0\C,1,4.64156118,5,106.6184169,4,-179.458
07285,0\C,13,1.39933072,1,60.28939537,5,68.11479746,0\C,14,1.39558502,
13,120.46653057,1,0.22545393,0\C,15,1.41236444,14,119.99854365,13,0.33
898616,0\C,16,1.40847184,15,119.17579696,14,-0.92791044,0\C,13,1.39665
825,1,59.52910892,16,140.24952702,0\H,13,1.08735617,1,179.62227371,16,
169.1754491,0\H,14,1.08725969,13,120.21915121,1,-179.6165763,0\H,15,1.
08460782,14,119.18404862,13,-179.46203774,0\H,17,1.0851686,16,120.5412
2746,15,-179.1956672,0\H,18,1.08717592,13,120.32894852,1,179.55884606,
0\C,1,4.64358776,16,106.08669155,15,179.76147511,0\C,24,1.39928407,1,6
0.33268211,16,71.02822969,0\C,25,1.39571611,24,120.49448818,1,0.455889
56,0\C,26,1.41262529,25,120.00898574,24,0.4039609,0\C,27,1.40865776,26
,119.11524397,25,-1.02403239,0\C,24,1.39648835,1,59.45649864,16,-109.0
5099437,0\H,24,1.08737078,1,179.43458742,16,-61.88200135,0\H,25,1.0872
6608,24,120.20362875,1,-179.34244827,0\H,26,1.08454092,25,119.09871697
,24,-179.36472138,0\H,28,1.08516244,27,120.58692254,26,-178.99178449,0
\H,29,1.08718129,24,120.32011122,1,179.25992911,0\C,1,2.00379178,16,11
2.02843264,15,-55.94868904,0\C,15,4.38273341,14,112.27472293,13,160.80
692555,0\C,36,1.39475952,15,75.37751191,14,-91.23706438,0\C,37,1.39994
93,36,120.61029057,15,-50.68543024,0\C,38,1.40571459,37,121.13890828,3
6,0.07740786,0\C,39,1.41244866,38,117.47957232,37,-1.86463593,0\C,40,1
.39640311,39,121.30132333,38,2.33538331,0\H,36,1.08737956,15,130.82527
067,14,26.66255851,0\H,37,1.08750218,36,120.2191352,15,129.60193326,0\
H,38,1.0838707,37,118.42925858,36,-179.12817323,0\H,40,1.08405886,39,1
20.35360449,38,-176.16402732,0\H,41,1.08768799,40,119.38775106,39,179.
81699345,0\C,4,4.38155318,3,112.14114562,2,161.41985307,0\C,47,1.39473
611,4,75.58306566,3,-91.7041052,0\C,48,1.39992977,47,120.61726719,4,-5
0.47014638,0\C,49,1.40569964,48,121.11937679,47,0.21406945,0\C,50,1.41
249272,49,117.48770039,48,-2.11418494,0\C,51,1.39638723,50,121.3027148
1,49,2.52611893,0\H,47,1.08737706,4,130.94799352,3,26.36802676,0\H,48,
1.08750581,47,120.2118379,4,129.83006918,0\H,49,1.08380935,48,118.4599
2303,47,-179.19179351,0\H,51,1.08422095,50,120.36081476,49,-176.015936
,0\H,52,1.08767705,51,119.39281854,50,179.76409045,0\C,35,4.3883797,1,
107.88178268,16,-65.59110519,0\C,58,1.39487244,35,59.5181934,1,-126.18
469567,0\C,59,1.39992772,58,120.6090913,35,0.49777838,0\C,60,1.4058056
5,59,121.10967037,58,0.10175075,0\C,61,1.41235481,60,117.51221267,59,-
1.97316137,0\C,62,1.39640086,61,121.29037905,60,2.47087446,0\H,58,1.08
737955,35,179.80644885,1,138.41232601,0\H,59,1.08748812,58,120.2103612
6,35,-179.26129937,0\H,60,1.08393513,59,118.47922786,58,-179.18775998,
0\H,62,1.08407469,61,120.29067323,60,-176.17660608,0\H,63,1.08769682,6
2,119.40113645,61,179.74924901,0\\Version=AM64L-G03RevD.01\State=1-A\H
F=-1759.2373944\MP2=-1764.6174325\RMSD=7.041e-09\Thermal=0.\PG=C01 [X(
C37H30P1)]\\@

90

1\1\GINC-AZAZEL\SP\RMP2-FC\6-31+G(2d,p)\C7H17P1\CHRISTOPH\01-Dec-2009\
0\\#p MP2(FC)/6-31+g(2d,p) scf=tight\\ip1sp_1\\0,1\P\C,1,1.86769446\H,
2,1.09563694,1,110.53874915\C,1,1.89531575,2,100.10640767,3,-76.575560
9,0\H,4,1.10394921,1,106.96703545,2,-48.06056572,0\C,1,1.86795863,2,98

.0929621,4,-101.89964628,0\H,6,1.09916001,1,112.87976349,2,56.08540763
,0\C,4,1.54868718,1,109.93781951,2,68.71788793,0\H,8,1.09869637,4,108.
90204553,1,-55.52187558,0\H,8,1.09921045,4,109.68880229,1,60.002827,0\
C,4,1.55149282,1,111.47696529,2,-164.75993899,0\H,11,1.09876339,4,108.
8571592,1,58.04414742,0\H,11,1.09954833,4,108.35486058,1,172.07419109,
0\C,8,1.5383076,4,114.21912278,1,-176.58754912,0\H,14,1.09676273,8,112
.09575975,4,-65.28460625,0\H,14,1.09730445,8,110.60044439,4,174.836844
08,0\H,14,1.09844616,8,111.10764944,4,55.29375994,0\C,11,1.53802792,4,
115.73142307,1,-65.22707016,0\H,18,1.09723876,11,110.37395601,4,177.77
957152,0\H,18,1.0968182,11,111.61501401,4,57.69765076,0\H,18,1.0971165
9,11,111.72530253,4,-62.71354626,0\H,2,1.09716779,1,108.65875448,6,64.
06737574,0\H,2,1.09923787,1,112.94251552,6,-56.32819685,0\H,6,1.097148
15,1,108.76485188,2,-64.3505339,0\H,6,1.09588377,1,110.47196285,2,178.
09025802,0\\Version=AM64L-G03RevD.01\State=1-A\HF=-615.7346971\MP2=-61
6.9562738\RMSD=4.324e-09\Thermal=0.\PG=C01 [X(C7H17P1)]\\@

90-Me⁺

1\1\GINC-YANG\SP\RMP2-FC\6-31+G(2d,p)\C8H20P1(1+)\CHRISTOPH\01-Dec-200
9\0\\#p MP2(FC)/6-31+g(2d,p) scf=tight\\ip1mesp_1\\1,1\C\H,1,1.1028668
6\C,2,2.90557181,1,83.32430019\H,3,1.09518492,2,91.71252108,1,86.09572
958,0\C,3,2.93813814,2,68.65006065,1,-66.27703257,0\H,5,1.09596689,3,8
7.46510537,2,-173.74581424,0\C,1,1.55474789,3,97.80012094,2,106.355145
37,0\H,7,1.09758756,1,109.75228397,3,103.96689155,0\H,7,1.09980518,1,1
09.58008168,3,-12.59500506,0\C,1,1.55464881,7,114.91295488,3,-164.1855
98,0\H,10,1.09807714,1,109.13276317,7,-174.78369876,0\H,10,1.09669959,
1,106.12806677,7,-61.89883255,0\C,7,1.53786718,1,112.86222718,10,62.38
256408,0\H,13,1.09532953,7,112.0539591,1,-65.23076582,0\H,13,1.0969852
9,7,111.34750143,1,56.17295634,0\H,13,1.09519712,7,109.76988939,1,175.
46186785,0\C,10,1.53620049,1,116.85870039,7,59.93686869,0\H,17,1.09594
823,10,112.24741778,1,-56.29273672,0\H,17,1.09525972,10,109.60563469,1
,-175.38368335,0\H,17,1.09844133,10,112.71093404,1,65.93191775,0\P,5,1
.82056024,3,36.24967779,2,57.57859364,0\C,21,1.8213277,5,108.66379259,
3,116.10888401,0\H,22,1.09607033,21,109.27474432,5,-56.67162274,0\H,22
,1.09409608,21,110.73714992,5,63.43563797,0\H,22,1.09501781,21,110.048
48182,5,-176.05873709,0\H,5,1.09482031,3,146.70201205,2,64.76739707,0\
H,5,1.095781,3,91.78282448,2,-65.0226769,0\H,3,1.09585563,2,158.256726
08,1,-74.2891189,0\H,3,1.09567634,2,69.66666832,1,-164.36104353,0\\Ver
sion=AM64L-G03RevD.01\State=1-A\HF=-655.1844878\MP2=-656.5501974\RMSD=
6.725e-09\Thermal=0.\PG=C01 [X(C8H20P1)]\\@

91

1\1\GINC-EDDY\SP\RMP2-FC\6-31+G(2d,p)\C6H15P1\CHRISTOPH\06-May-2009\0\
\#p MP2(FC)/6-31+g(2d,p) scf=tight\\tbdmpspc001\\0,1\C\C,1,1.54609168\
H,2,1.09975949,1,110.00080626\H,2,1.0975159,1,111.91803502,3,119.28950
622,0\H,2,1.09761861,1,111.43929899,3,-119.58402319,0\C,1,1.5410616,2,
109.45185808,4,58.26505613,0\H,6,1.1003639,1,109.87884649,2,59.4776828
2,0\H,6,1.09697245,1,112.05024891,2,178.57539319,0\H,6,1.09697245,1,11
2.05024891,2,-59.62002755,0\C,1,1.54609168,6,109.45185808,2,-118.95536
564,0\H,10,1.09761858,1,111.43925416,6,-179.39155537,0\H,10,1.0975155,
1,111.9180695,6,-58.26502044,0\H,10,1.09975949,1,110.00080626,6,61.024
45009,0\P,1,1.90801038,6,115.1618428,2,120.52231718,0\C,14,1.86734635,
1,102.76169895,6,-51.01064878,0\H,15,1.09619837,14,110.24570373,1,-67.
98263218,0\H,15,1.09768428,14,113.78138349,1,54.63759165,0\H,15,1.0972
061,14,108.20471837,1,174.98944166,0\C,14,1.86734635,1,102.76169895,6,
51.01064878,0\H,19,1.09619837,14,110.24570373,1,67.98263218,0\H,19,1.0
972061,14,108.20471837,1,-174.98944166,0\H,19,1.09768428,14,113.781383
49,1,-54.63759165,0\\Version=AM64L-G03RevD.01\State=1-A\HF=-576.697056
2\MP2=-577.7694598\RMSD=4.632e-09\Thermal=0.\PG=C01 [X(C6H15P1)]\\@

91-Me$^+$

1\1\GINC-SOLARIS\SP\RMP2-FC\6-31+G(2d,p)\C7H18P1(1+)\CHRISTOPH\13-May-
2009\0\\#p MP2(FC)/6-31+g(2d,p) scf=tight\\tbdmpmespc001_3\\1,1\C\C,1,
1.54798461\H,2,1.09659497,1,108.30484415\H,2,1.09589943,1,112.32493138
,3,118.30286033,0\H,2,1.09691201,1,112.64892407,3,-118.28853577,0\C,1,
1.54806939,2,110.1891906,4,54.65187359,0\H,6,1.09662617,1,108.31420663
,2,58.28890978,0\H,6,1.09605789,1,112.36274167,2,176.60259803,0\H,6,1.
09679601,1,112.64132558,2,-60.00658968,0\C,1,1.54814452,2,110.16197359
,6,121.73258514,0\H,10,1.0960282,1,112.37962734,2,54.77842114,0\H,10,1
.09680384,1,112.62521912,2,178.18440793,0\H,10,1.09664198,1,108.288552
5,2,-63.53579875,0\C,1,3.04323473,2,87.58694841,10,146.09952122,0\H,14
,1.09477302,1,91.93938502,2,-5.32631453,0\H,14,1.09509009,1,90.7979568
6,2,103.81096371,0\H,14,1.09598626,1,144.08658313,2,-131.84169014,0\C,
14,2.94260239,1,61.08538104,2,-164.51807893,0\H,18,1.09513591,14,146.6
7396154,1,35.42307873,0\H,18,1.09598656,14,88.99754764,1,159.19549493,
0\H,18,1.09466463,14,90.47847935,1,-92.06753032,0\P,18,1.82196991,14,3
6.15098052,1,33.56852125,0\C,22,1.82186138,18,107.69478718,14,115.8557
56,0\H,23,1.09597071,22,109.24925597,18,-59.25770432,0\H,23,1.09468013
,22,110.52835021,18,60.3780105,0\H,23,1.0950614,22,110.48902281,18,-17
8.74724971,0\\Version=AM64L-G03RevD.01\State=1-A\HF=-616.1477681\MP2=-
617.3641982\RMSD=3.954e-09\Thermal=0.\PG=C01 [X(C7H18P1)]\\@

92

1\1\GINC-CIPCLU09\SP\RMP2-FC\6-31+G(2d,p)\C10H23P1\C2175\08-Jan-2010\0
\\#p MP2(FC)/6-31+G(2d,p) scf=tight\\mih1sp_2\\0,1\P\C,1,1.87839619\H,
2,1.10018763,1,106.63677001\H,2,1.10183927,1,108.94948608,3,114.369295
21,0\C,2,1.54984204,1,115.78491013,3,-122.36417508,0\H,5,1.10304412,2,
107.33804406,1,-60.71376013,0\C,1,1.86550731,2,98.4303122,5,172.947546
76,0\H,7,1.09693705,1,109.57373496,2,70.3473779,0\H,7,1.09688717,1,109
.49549186,2,-171.92749121,0\H,7,1.0988706,1,112.8690849,2,-50.80913909
,0\C,1,1.86638434,7,99.19070356,2,101.56058143,0\H,11,1.09698502,1,109
.31741861,7,68.8347304,0\H,11,1.09651271,1,109.98512047,7,-173.448114,
0\H,11,1.09910127,1,112.68337951,7,-52.04179466,0\C,5,1.54906692,2,109
.27037315,1,-175.826921,0\H,15,1.10106122,5,109.73140161,2,-61.6961048
3,0\H,15,1.10145414,5,107.97697678,2,53.08808995,0\C,5,1.5504555,2,113
.31641333,1,56.35058608,0\H,18,1.09991201,5,108.67366032,2,-174.371640
62,0\H,18,1.09994405,5,108.51317858,2,-59.74884253,0\C,15,1.53881856,5
,115.30644206,2,174.46920928,0\H,21,1.09892114,15,110.064097,5,65.0936
9002,0\H,21,1.10085544,15,109.28813326,5,-51.24663842,0\C,18,1.5391460
2,5,116.26938808,2,62.57371352,0\H,24,1.09907346,18,109.82367149,5,-59
.09695266,0\H,24,1.09994499,18,110.166708,5,57.84300168,0\C,24,1.53652
912,18,112.10880858,5,179.60287294,0\H,27,1.09726397,24,111.40080658,1
8,-179.8335302,0\H,27,1.09842219,24,111.12150256,18,-59.66678117,0\H,2
7,1.0979167,24,110.97729953,18,60.04387912,0\C,21,1.53633395,15,112.53
494928,5,-173.04257306,0\H,31,1.09822863,21,111.09870939,15,-59.588241
28,0\H,31,1.09721667,21,111.38667613,15,-179.68407818,0\H,31,1.0981906
,21,111.13523217,15,60.12437169,0\\Version=AM64L-G03RevD.01\State=1-A\
HF=-732.8530671\MP2=-734.5332913\RMSD=5.196e-09\Thermal=0.\PG=C01 [X(C
10H23P1)]\\@

92-Me$^+$

1\1\GINC-YIN\SP\RMP2-FC\6-31+G(2d,p)\C11H26P1(1+)\CHRISTOPH\25-Dec-200
9\0\\#p MP2(FC)/6-31+g(2d,p) scf=tight\\mih1mesp_2\\1,1\C\H,1,1.098914
5\H,1,1.09984737,2,106.31113119\C,1,1.55679172,2,111.24755702,3,-118.6
9892132,0\H,4,1.10152437,1,107.25887754,2,-179.37633697,0\C,1,3.009069
87,4,89.95542575,5,-35.03967958,0\H,6,1.0958556,1,144.32702371,4,-153.
02289171,0\H,6,1.09576412,1,88.97589955,4,85.00673976,0\H,6,1.09493496

,1,92.98430561,4,-23.90392726,0\C,6,2.96862316,1,61.20542016,4,-113.57
903124,0\H,10,1.09402568,6,91.82431154,1,92.93430097,0\H,10,1.09581666
,6,144.96597154,1,-34.59274182,0\H,10,1.09611981,6,89.59606937,1,-157.
98782438,0\C,4,1.5508323,1,107.73205607,6,-149.48798249,0\H,14,1.10010
216,4,109.86131692,1,-61.23766952,0\H,14,1.10085108,4,108.15188697,1,5
4.02489762,0\C,4,1.54867472,1,113.91071642,14,-127.21759385,0\H,17,1.0
9758501,4,107.64001579,1,-175.41427171,0\H,17,1.10322254,4,109.5218846
4,1,-62.38566532,0\C,14,1.53957677,4,114.49547231,1,175.0490334,0\H,20
,1.09855278,14,110.04518841,4,65.8861598,0\H,20,1.10043957,14,109.3527
5854,4,-50.74361814,0\C,17,1.53990559,4,116.63566575,1,62.13162644,0\H
,23,1.10155202,17,110.62301707,4,-57.77129617,0\H,23,1.09811608,17,109
.59605257,4,58.71502484,0\C,23,1.53655673,17,112.22686406,4,-179.77711
555,0\H,26,1.09867323,23,111.5410416,17,60.27901595,0\H,26,1.09559755,
23,111.03420454,17,-179.57827743,0\H,26,1.09687178,23,111.08574461,17,
-59.68511648,0\C,20,1.5361192,14,112.1768482,4,-172.45983138,0\H,30,1.
09740019,20,111.23737443,14,-59.62560611,0\H,30,1.09570602,20,110.8501
7098,14,-179.46334303,0\H,30,1.09743285,20,111.2551887,14,60.61957693,
0\P,6,1.81846969,1,34.70984814,4,-146.35579973,0\C,34,1.82075776,6,108
.34789216,1,-118.39377254,0\H,35,1.09595449,34,109.92514369,6,178.5968
9961,0\H,35,1.09567383,34,110.08110089,6,58.73435304,0\H,35,1.09576965
,34,110.19370883,6,-61.43678379,0\\Version=AM64L-G03RevD.01\State=1-A\
HF=-772.3022477\MP2=-774.1275509\RMSD=2.364e-09\Thermal=0.\PG=C01 [X(C
11H26P1)]\\@

93
1\1\GINC-AZAZEL\SP\RMP2-FC\6-31+G(2d,p)\C7H17P1\CHRISTOPH\06-Dec-2009\
0\\#p MP2(FC)/6-31+g(2d,p) scf=tight\\aip1sp_1\\0,1\P\C,1,1.86792734\H
,2,1.09711551,1,108.70428847\H,2,1.09577794,1,110.58177353,3,117.53001
575,0\H,2,1.09909455,1,112.88494691,3,-120.3747102,0\C,1,1.86794586,2,
97.97070052,4,-177.53516654,0\H,6,1.09708614,1,108.76329014,2,-64.1058
9044,0\H,6,1.09905841,1,112.81207456,2,56.25112848,0\H,6,1.09560863,1,
110.49732682,2,178.31029675,0\C,1,1.89058676,2,100.56529902,6,102.1972
5192,0\H,10,1.10330581,1,107.91952312,2,-48.22191999,0\C,10,1.54773668
,1,109.87066697,2,69.91491314,0\H,12,1.10131391,10,109.02661359,1,64.4
9677488,0\H,12,1.09999701,10,109.52038909,1,-51.19801111,0\C,10,1.5419
5209,1,109.75304654,2,-166.54715558,0\H,15,1.09845492,10,110.83510959,
1,-60.30411949,0\H,15,1.09635382,10,111.80927794,1,60.08883995,0\H,15,
1.09695276,10,111.27398852,1,179.76221427,0\C,12,1.5409073,10,114.7156
6115,1,-172.46953971,0\H,19,1.10073236,12,109.16045841,10,54.26662751,
0\H,19,1.09847171,12,110.2365345,10,-62.19249009,0\C,19,1.53625199,12,
112.4195225,10,175.93813588,0\H,22,1.09821942,19,111.16476488,12,-59.7
1355207,0\H,22,1.09811012,19,111.10725281,12,60.04688224,0\H,22,1.0971
7347,19,111.32876503,12,-179.87391396,0\\Version=AM64L-G03RevD.01\Stat
e=1-A\HF=-615.7384241\MP2=-616.9573316\RMSD=4.026e-09\Thermal=0.\PG=C0
1 [X(C7H17P1)]\\@

93-Me$^+$
1\1\GINC-YIN\SP\RMP2-FC\6-31+G(2d,p)\C8H20P1(1+)\CHRISTOPH\06-Dec-2009
\0\\#p MP2(FC)/6-31+g(2d,p) scf=tight\\aip1mesp_1\\1,1\C\H,1,1.1022522
9\C,1,3.01607052,2,88.34186962\H,3,1.09593895,1,88.26169414,2,-12.2840
4949,0\H,3,1.09463283,1,94.0282455,2,-121.23967069,0\H,3,1.09593381,1,
144.25984212,2,108.33691887,0\C,3,2.93842547,1,60.76050422,2,82.193687
86,0\H,7,1.09455296,3,146.84317988,1,40.38043148,0\H,7,1.09586864,3,87
.84995344,1,162.38615392,0\H,7,1.09582646,3,91.35948282,1,-88.79221142
,0\C,1,1.55391229,7,93.12297091,3,159.01074194,0\H,11,1.09990136,1,110
.17257371,7,15.13295795,0\H,11,1.0997645,1,109.3028682,7,-101.91426707
,0\C,1,1.54518109,11,113.35203468,7,155.95051539,0\H,14,1.0940495,1,10
9.20928008,11,58.34670263,0\H,14,1.09538209,1,112.47803709,11,176.9563

1423,0\H,14,1.09703435,1,111.77535935,11,-60.34211941,0\C,11,1.5428775
,1,113.18697952,14,-68.1265986,0\H,18,1.09737578,11,110.02412825,1,61.
84788346,0\H,18,1.09965595,11,109.11062154,1,-54.93891511,0\C,18,1.535
78485,11,111.8683806,1,-176.53561773,0\H,21,1.09737184,18,111.37728547
,11,60.39076132,0\H,21,1.09529743,18,110.56869314,11,-179.87810662,0\H
,21,1.09718613,18,111.32370086,11,-60.21298109,0\P,3,1.8208915,1,34.95
347651,14,-133.37507011,0\C,25,1.8196354,3,108.47770257,1,122.09778809
,0\H,26,1.09583354,25,109.65661261,3,56.69317904,0\H,26,1.09509474,25,
110.21308295,3,176.5090893,0\H,26,1.09535406,25,110.28946125,3,-63.052
13298,0\\Version=AM64L-G03RevD.01\State=1-A\HF=-655.1887066\MP2=-656.5
516454\RMSD=7.146e-09\Thermal=0.\PG=C01 [X(C8H20P1)]\\@

94
1\1\GINC-AZAZEL\SP\RMP2-FC\6-31+G(2d,p)\C7H15P1\CHRISTOPH\08-Oct-2009\
0\\#p MP2(FC)/6-31+g(2d,p) scf=tight\\pe1sp_6\\0,1\P\C,1,1.86684309\H,
2,1.09884597,1,112.69458684\H,2,1.09656673,1,109.75507777,3,121.242812
11,0\H,2,1.09698004,1,109.45895812,4,117.84864993,0\C,1,1.86638773,2,9
8.92964939,4,172.05108854,0\H,6,1.0990378,1,112.75003063,2,-52.4597280
6,0\H,6,1.09708838,1,109.38534779,2,68.37437865,0\H,6,1.09642363,1,109
.81533495,2,-173.82679837,0\C,1,2.8642973,6,130.06692474,2,96.01142713
,0\C,10,1.56008869,1,131.57880294,6,67.9030821,0\C,11,1.54448882,10,10
5.46257032,1,-41.38964744,0\C,12,1.54040616,11,103.4986078,10,31.64251
094,0\H,10,1.09641121,1,111.61285165,6,-84.5046989,0\H,10,1.09848292,1
,75.97092974,6,172.7628305,0\H,11,1.09641594,10,112.43562588,1,-164.36
682367,0\H,11,1.09778374,10,109.86242355,1,76.64321817,0\H,12,1.097062
,11,112.89004255,10,154.33710039,0\H,12,1.09954496,11,110.30770632,10,
-85.47843095,0\H,13,1.09686182,12,113.21795548,11,-163.88902788,0\H,13
,1.10070215,12,109.7427527,11,75.88465881,0\C,13,1.54885721,12,103.362
24698,11,-41.62901736,0\H,22,1.10192569,13,108.181103,12,-80.5733803,0
\\Version=AM64L-G03RevD.01\State=1-A\HF=-614.5707317\MP2=-615.7676134\
RMSD=9.911e-09\Thermal=0.\PG=C01 [X(C7H15P1)]\\@

94-Me$^+$
1\1\GINC-YIN\SP\RMP2-FC\6-31+G(2d,p)\C8H18P1(1+)\CHRISTOPH\08-Oct-2009
\0\\#p MP2(FC)/6-31+g(2d,p) scf=tight\\pe1mesp_1\\1,1\P\C,1,1.81968826
\H,2,1.09530222,1,110.04053243\H,2,1.09585811,1,109.98184701,3,119.959
73225,0\H,2,1.09582041,1,110.08442405,4,119.98447158,0\C,1,1.82000005,
2,108.43828478,3,60.59406104,0\H,6,1.09502589,1,110.44575421,2,-60.787
43747,0\H,6,1.09591451,1,109.30460404,2,58.82996655,0\H,6,1.09502536,1
,110.44658487,2,178.44621249,0\C,1,1.81968595,2,108.50732025,6,117.593
56407,0\H,10,1.0958207,1,110.08616169,2,-58.12475212,0\H,10,1.09585848
,1,109.98133348,2,61.86140061,0\H,10,1.09530438,1,110.03761028,2,-178.
18143101,0\C,1,1.82855662,10,109.84053274,6,122.32003972,0\C,14,1.5520
131,1,115.07969771,10,-60.17755235,0\C,15,1.55266437,14,103.16728981,1
,-166.42632571,0\C,16,1.55992894,15,106.42805393,14,24.25369238,0\C,14
,1.55189142,1,115.09305692,10,179.58454419,0\H,14,1.10187983,1,104.929
83064,10,59.70981843,0\H,15,1.09532966,14,113.00996276,1,71.54298872,0
\H,15,1.09863851,14,110.06893038,1,-49.04915094,0\H,16,1.09526376,15,1
09.94358969,14,-95.38756427,0\H,16,1.09415702,15,110.71979862,14,146.6
1780182,0\H,17,1.09533183,16,110.43987563,15,-118.82389749,0\H,17,1.09
415716,16,112.37461265,15,121.79934047,0\H,18,1.09534948,14,113.041861
59,1,-71.32222205,0\H,18,1.0986577,14,110.06816948,1,49.31705315,0\\Ve
rsion=AM64L-G03RevD.01\State=1-A\HF=-654.0206643\MP2=-655.3614964\RMSD
=4.774e-09\Thermal=0.\PG=C01 [X(C8H18P1)]\\@

95
1\1\GINC-GOLEM\SP\RMP2-FC\6-31+G(2d,p)\C8H19P1\CHRISTOPH\07-Dec-2009\0
\\#p MP2(FC)/6-31+g(2d,p) scf=tight\\aih1sp_1\\0,1\C\H,1,1.10396411\C,

1,2.88837636,2,82.28152886\H,3,1.09917789,1,82.98979557,2,10.09387427,
0\C,1,1.55068494,3,90.82865622,4,-96.97531676,0\H,5,1.10077461,1,108.4
8713953,3,137.67552807,0\H,5,1.09980784,1,108.88655493,3,23.67210572,0
\C,1,1.549194,5,113.40961653,3,159.0994868,0\H,8,1.09876714,1,108.9209
5614,5,178.9043011,0\H,8,1.09905715,1,109.62246015,5,-65.59381846,0\C,
5,1.54024488,1,116.21983623,8,59.52257404,0\H,11,1.09916381,5,109.8190
754,1,55.82358037,0\H,11,1.09932303,5,109.79592907,1,-60.42155007,0\C,
11,1.53599508,5,112.16554003,1,178.00657437,0\H,14,1.09839566,11,111.1
5551345,5,-60.44807591,0\H,14,1.09710445,11,111.35451357,5,179.4385789
6,0\H,14,1.09845061,11,111.17622807,5,59.28556989,0\P,3,1.86816352,1,4
0.22858899,8,-17.00670717,0\C,18,1.86797882,3,97.87853024,1,101.549947
59,0\H,19,1.09922552,18,112.74711598,3,-55.95593577,0\C,8,1.53835912,1
,114.29777661,5,57.74978913,0\H,21,1.09843669,8,111.1514758,1,55.07360
154,0\H,21,1.09672206,8,112.06513137,1,-65.53062055,0\H,21,1.09728936,
8,110.5837054,1,174.64447105,0\H,3,1.09712765,1,146.84847066,8,8.38681
547,0\H,3,1.09584008,1,97.45958101,8,-130.24104701,0\H,19,1.09713314,1
8,108.73310176,3,64.36775219,0\H,19,1.09566304,18,110.64901854,3,-178.
03901002,0\\Version=AM64L-G03RevD.01\State=1-A\HF=-654.7735012\MP2=-65
6.1483569\RMSD=4.507e-09\Thermal=0.\PG=C01 [X(C8H19P1)]\\@

95-Me$^+$

1\1\GINC-GOLEM\SP\RMP2-FC\6-31+G(2d,p)\C9H22P1(1+)\CHRISTOPH\07-Dec-20
09\0\\#p MP2(FC)/6-31+g(2d,p) scf=tight\\aih1mesp_2\\1,1\C\H,1,1.10293
802\C,1,3.06447517,2,132.59249079\H,3,1.09418245,1,92.1205698,2,154.34
852054,0\H,3,1.09501469,1,91.60165789,2,-96.60062121,0\H,3,1.09612556,
1,142.95208925,2,28.18780753,0\C,3,2.93761101,1,59.62454703,2,-7.78800
045,0\H,7,1.09570777,3,146.10089367,1,41.24071483,0\H,7,1.09583412,3,8
7.78881712,1,162.78685838,0\H,7,1.09518484,3,92.26433489,1,-88.5634806
1,0\C,1,1.55419808,7,143.58826148,3,76.00327061,0\H,11,1.09770473,1,10
6.23274804,7,145.29208924,0\H,11,1.09912473,1,109.15306242,7,32.412853
51,0\C,1,1.55491761,11,115.08269144,3,93.52478079,0\H,14,1.09988324,1,
109.58759784,11,-177.05890699,0\H,14,1.097309,1,109.80555938,11,-60.40
123445,0\C,11,1.53946023,1,117.29947966,14,60.56566165,0\H,17,1.101837
79,11,110.41848561,1,63.33400588,0\H,17,1.09850055,11,110.18877547,1,-
53.89909939,0\C,17,1.53682192,11,111.71766657,1,-175.51789988,0\H,20,1
.09678958,17,110.97310474,11,60.6241079,0\H,20,1.09530215,17,110.88400
275,11,-179.6232291,0\H,20,1.09727421,17,111.38430909,11,-59.50016017,
0\P,3,1.82143988,1,33.77053435,11,-106.36214697,0\C,24,1.82081241,3,10
8.58295403,1,124.06943762,0\H,25,1.0959431,24,109.43051245,3,60.130839
39,0\H,25,1.09576283,24,110.12995971,3,179.67502611,0\H,25,1.09477325,
24,110.57864947,3,-59.79735179,0\C,14,1.5378777,1,112.82177951,11,62.1
8312652,0\H,29,1.0952467,14,112.04682117,1,-65.54954194,0\H,29,1.09526
391,14,109.76668749,1,175.16304468,0\H,29,1.09699538,14,111.3552656,1,
55.87533436,0\\Version=AM64L-G03RevD.01\State=1-A\HF=-694.2240289\MP2=
-695.7431104\RMSD=7.764e-09\Thermal=0.\PG=C01 [X(C9H22P1)]\\@

96

1\1\GINC-NODE9\SP\RMP2-FC\6-31+G(2d,p)\C9H15P1\ZIP07\03-Sep-2009\0\\#p
MP2(FC)/6-31+g(2d,p) scf=tight\\pro3sp_20\\0,1\P\C,1,1.83956562\C,2,1
.52490896,1,119.36147283\C,3,1.50896922,2,60.36180235,1,108.34480582,0
\H,2,1.09229697,1,116.35940304,4,145.20380246,0\H,3,1.08770601,2,117.5
7034251,1,-142.6834758,0\H,3,1.08825351,2,117.95059624,1,0.6281742,0\H
,4,1.0877122,3,118.6955022,2,107.27170614,0\H,4,1.0882709,3,117.693650
4,2,-108.02283107,0\C,1,1.8402802,2,100.40660876,3,94.6234651,0\C,10,1
.52526914,1,119.09277922,2,-93.99482909,0\C,11,1.50901591,10,60.347804
16,1,-108.56822359,0\H,10,1.09214403,1,116.36200177,2,51.45172465,0\H,
11,1.08828257,10,117.90863511,1,-0.80607291,0\H,11,1.08774168,10,117.6
2084459,1,142.50050375,0\H,12,1.08824053,11,117.78045224,10,108.001817

51,0\H,12,1.08771591,11,118.61682942,10,-107.22303031,0\C,1,1.84077946
,2,100.18489152,3,-162.76587957,0\C,18,1.52549989,1,119.08742873,2,163
.5061851,0\C,19,1.50905636,18,60.33957212,1,-108.52796311,0\H,18,1.092
20924,1,116.37382421,2,-51.00864903,0\H,19,1.08824047,18,117.87886441,
1,-0.75606105,0\H,19,1.08774331,18,117.63196012,1,142.53862561,0\H,20,
1.08816492,19,117.78178145,18,108.02529547,0\H,20,1.08773762,19,118.59
46261,18,-107.23194273,0\\Version=AM64L-G03RevD.01\State=1-A\HF=-690.2
058404\MP2=-691.6654711\RMSD=4.744e-09\Thermal=0.\PG=C01 [X(C9H15P1)]\
\@

96-Me$^+$

1\1\GINC-MORITZ\SP\RMP2-FC\6-31+G(2d,p)\C10H18P1(1+)\CHRISTOPH\01-Sep-
2009\0\\#p MP2(FC)/6-31+g(2d,p) scf=tight\\pro3mesp_2\\1,1\P\C,1,1.820
74695\H,2,1.09559377,1,109.86979907\H,2,1.09555337,1,109.84913378,3,12
0.00718457,0\H,2,1.09559787,1,109.83497141,4,119.99249425,0\C,1,1.7990
8729,2,108.32545629,4,-60.41698207,0\C,6,1.53480079,1,121.23187705,2,-
84.08306014,0\C,7,1.50018102,6,60.64955475,1,-111.43287242,0\H,6,1.089
76652,1,112.5148605,2,58.69866752,0\H,7,1.08718605,6,118.89207569,1,-3
.77110851,0\H,7,1.08640272,6,115.86509791,1,138.31976629,0\H,8,1.08701
492,7,117.60528364,6,108.65228988,0\H,8,1.08670705,7,118.89941058,6,-1
05.66282196,0\C,1,1.79884924,6,110.56324843,8,86.98723333,0\C,14,1.534
57026,1,121.18449712,6,34.5905031,0\C,15,1.50034543,14,60.64707606,1,-
111.351208,0\H,14,1.08976977,1,112.57459291,6,177.35498809,0\H,15,1.08
722136,14,118.86539399,1,-3.63911945,0\H,15,1.08641115,14,115.85521594
,1,138.3831958,0\H,16,1.08694396,15,117.64423144,14,108.52388995,0\H,1
6,1.08672495,15,118.90754016,14,-105.66317012,0\C,1,1.799033,14,110.57
534871,16,87.09714788,0\C,22,1.53473477,1,121.22252226,14,34.73936027,
0\C,23,1.50015113,22,60.65642634,1,-111.36135209,0\H,22,1.08972741,1,1
12.55580653,14,177.4671509,0\H,23,1.08712637,22,118.92823627,1,-3.6648
8162,0\H,23,1.08641921,22,115.84332465,1,138.39748254,0\H,24,1.0868999
7,23,117.58134556,22,108.63119043,0\H,24,1.08669031,23,118.9048604,22,
-105.66092275,0\\Version=IA32L-G03RevD.01\State=1-A\HF=-729.6537124\MP
2=-731.2612273\RMSD=8.967e-09\Thermal=0.\PG=C01 [X(C10H18P1)]\\@

97

1\1\GINC-AZAZEL\SP\RMP2-FC\6-31+G(2d,p)\C8H17P1\CHRISTOPH\27-Aug-2009\
0\\#p MP2(FC)/6-31+g(2d,p) scf=tight\\hex1sp_1\\0,1\P\C,1,1.86779435\H
,2,1.09707402,1,108.88471076\H,2,1.09902418,1,112.81708719,3,120.48869
315,0\H,2,1.09595501,1,110.33922658,3,-117.62833301,0\C,1,1.86779769,2
,98.2612966,5,176.54474236,0\H,6,1.09595259,1,110.33968298,2,-176.5506
6628,0\H,6,1.09707284,1,108.88431028,2,65.8215648,0\H,6,1.09902554,1,1
12.81753194,2,-54.66684355,0\C,1,4.24031143,2,93.25907275,6,-121.56247
926,0\C,10,1.54169249,1,19.20949969,2,-61.48011383,0\C,11,1.5470191,10
,112.0052842,1,3.33026056,0\C,12,1.54702426,11,110.10765224,10,-54.584
02868,0\C,13,1.54169534,12,112.00513412,11,54.58272025,0\C,10,1.538028
17,1,99.17711118,2,166.59720577,0\H,10,1.1014332,1,101.3476637,2,54.73
683833,0\H,10,1.09900992,1,128.89238755,2,-67.70830158,0\H,11,1.098317
39,10,109.45465105,1,126.1627645,0\H,11,1.101576,10,109.2016859,1,-117
.43578216,0\H,12,1.10437835,11,108.41104663,10,63.86410537,0\H,13,1.09
831207,12,110.43857211,11,176.85522548,0\H,13,1.10157731,12,108.944777
7,11,-66.33232098,0\H,14,1.09901005,13,109.77503286,12,-178.18068923,0
\H,14,1.10143196,13,109.16611438,12,65.2956737,0\H,15,1.10138802,10,10
9.23519613,1,80.72075732,0\H,15,1.09882039,10,110.3275536,1,-162.33105
76,0\\Version=AM64L-G03RevD.01\State=1-A\HF=-653.6166878\MP2=-654.9685
672\RMSD=4.590e-09\Thermal=0.\PG=C01 [X(C8H17P1)]\\@

97-Me$^+$

1\1\GINC-NODE16\SP\RMP2-FC\6-31+G(2d,p)\C9H20P1(1+)\ZIP07\31-Aug-2009\

0\\#p MP2(FC)/6-31+g(2d,p) scf=tight\\hex1mesp_2\\1,1\P\C,1,1.82222275
\H,2,1.09586901,1,109.58094569\H,2,1.09489437,1,110.62585118,3,119.793
34994,0\H,2,1.09574362,1,110.15808597,3,-119.69755057,0\C,1,1.82220406
,2,107.45018231,4,175.09990098,0\H,6,1.09574387,1,110.1583846,2,64.452
81801,0\H,6,1.09489229,1,110.62527764,2,-175.03538955,0\H,6,1.09586843
,1,109.58012438,2,-55.24368025,0\C,1,1.82201172,6,107.45036861,2,115.3
565847,0\H,10,1.09417344,1,110.62839364,6,61.85396044,0\H,10,1.0941781
8,1,110.62610322,6,-177.13817405,0\H,10,1.09660889,1,108.70863867,6,-5
7.6443882,0\C,1,3.610165,10,78.44299769,2,143.43929645,0\C,14,1.540264
12,1,49.86137192,10,165.25822916,0\C,15,1.55565154,14,114.01850031,1,3
7.36178395,0\C,16,1.55566968,15,111.39397775,14,47.89589614,0\C,17,1.5
4028916,16,114.01883782,15,-47.8837558,0\C,18,1.53810136,17,111.591133
57,16,52.40565476,0\H,14,1.10037788,1,67.96792626,10,18.13114353,0\H,1
4,1.09679597,1,145.35891146,10,104.45682002,0\H,15,1.09911535,14,108.5
5255808,1,154.87585022,0\H,15,1.09784745,14,111.06209639,1,-89.3542049
2,0\H,16,1.1032931,15,107.78392825,14,165.94612725,0\H,17,1.09911154,1
6,105.69795242,15,71.26683478,0\H,17,1.09785913,16,111.30522045,15,-17
4.46857912,0\H,18,1.09679751,17,108.56141277,16,174.18323425,0\H,18,1.
10035497,17,110.69368129,16,-69.9174512,0\H,19,1.10005016,18,109.21808
754,17,65.11698314,0\H,19,1.09649851,18,110.09695877,17,-178.16983833,
0\\Version=AM64L-G03RevD.01\State=1-A\HF=-693.060709\MP2=-694.5578045\
RMSD=7.302e-09\Thermal=0.\PG=C01 [X(C9H20P1)]\\@

98

1\1\GINC-MORITZ\SP\RMP2-FC\6-31+G(2d,p)\C6H15P1\CHRISTOPH\23-Sep-2009\
0\\#p MP2(FC)/6-31+g(2d,p) scf=tight\\et3sp_2\\0,1\P\C,1,1.87718144\H,
2,1.09884109,1,106.77172005\H,2,1.10084149,1,110.15912105,3,-115.60492
38,0\C,1,1.87768173,2,99.48889056,3,61.59763688,0\H,5,1.09884687,1,106
.80113676,2,163.61909965,0\H,5,1.10080059,1,110.10420929,2,48.00998319
,0\C,1,1.87663699,2,99.60845806,5,101.4167659,0\H,8,1.0989024,1,106.77
852731,2,62.14060023,0\H,8,1.10077972,1,110.16649923,2,-53.51774926,0\
C,2,1.5387028,1,113.23166059,8,-76.81395564,0\H,11,1.09704716,2,111.68
802531,1,63.83504528,0\H,11,1.09747805,2,110.82226913,1,-176.42340073,
0\H,11,1.09690233,2,111.07513881,1,-56.43647618,0\C,5,1.53850825,1,113
.2499851,8,-177.65453049,0\H,15,1.09700078,5,111.66256277,1,63.7468882
3,0\H,15,1.09752647,5,110.80807263,1,-176.54534309,0\H,15,1.09684577,5
,111.11426192,1,-56.54137818,0\C,8,1.53863104,1,113.20012947,2,-177.69
933449,0\H,19,1.0974612,8,110.81829317,1,-176.05339044,0\H,19,1.097050
05,8,111.67758504,1,64.22532835,0\H,19,1.09687633,8,111.09582365,1,-56
.04816933,0\\Version=IA32L-G03RevD.01\State=1-A\HF=-576.6983479\MP2=-5
77.7606977\RMSD=9.819e-09\Thermal=0.\PG=C01 [X(C6H15P1)]\\@

98-Me$^+$

1\1\GINC-MORITZ\SP\RMP2-FC\6-31+G(2d,p)\C7H18P1(1+)\CHRISTOPH\23-Sep-2
009\0\\#p MP2(FC)/6-31+g(2d,p) scf=tight\\et3mesp_1\\1,1\P\C,1,1.83207
177\H,2,1.09875875,1,106.42920056\H,2,1.09829813,1,106.85712784,3,-113
.56423795,0\C,1,1.83119116,2,110.70928809,4,65.48100742,0\H,5,1.098234
53,1,106.73153163,2,63.23950159,0\H,5,1.09823411,1,106.73251784,2,176.
75946198,0\C,1,1.83206791,5,110.71002088,2,119.99568568,0\H,8,1.098298
04,1,106.8568965,5,-65.46432429,0\H,8,1.09875803,1,106.42965317,5,-179
.02863787,0\C,1,1.82101945,5,108.36178001,2,-120.00183345,0\H,11,1.095
22693,1,110.12102165,5,-59.85944574,0\H,11,1.09522715,1,110.12117963,5
,59.88064673,0\H,11,1.09595502,1,110.08784066,5,-179.98937785,0\C,5,1.
54154859,1,114.66002728,11,179.99810644,0\H,15,1.09564967,5,112.036295
83,1,-61.15455006,0\H,15,1.09565077,5,112.03663443,1,61.14485322,0\H,1
5,1.09471833,5,109.00014435,1,179.99518591,0\C,2,1.54147788,1,114.8921
5976,11,61.45127208,0\H,19,1.09553574,2,112.00862813,1,61.7911534,0\H,
19,1.09543561,2,112.06550622,1,-60.63436407,0\C,8,1.54147849,1,114.891

82916,11,-61.43540197,0\H,22,1.09543539,8,112.06478577,1,60.64475934,0
\H,22,1.09553702,8,112.00897119,1,-61.78048594,0\H,19,1.0947358,2,108.
96343805,1,-179.41285267,0\H,22,1.09473581,8,108.96353395,1,179.423455
4,0\\Version=IA32L-G03RevD.01\State=1-A\HF=-616.1493984\MP2=-617.35677
74\RMSD=1.736e-09\Thermal=0.\PG=C01 [X(C7H18P1)]\\@

98-BH$^+$

1\1\GINC-CIPCLU03\SP\RMP2-FC\6-31+G(2d,p)\C19H26P1(1+)\C2175\15-Sep-20
10\0\\#p MP2(FC)/6-31+G(2d,p) scf=tight\\k7bhsp_38\\1,1\C\H,1,1.099984
67\C,1,4.34797541,2,104.9950407\C,3,1.39791387,1,58.74935758,2,17.1632
5313,0\C,4,1.39720136,3,120.14963336,1,-0.66081614,0\C,5,1.4054171,4,1
20.85961683,3,0.38470457,0\C,6,1.40592312,5,118.44114045,4,-0.2743919,
0\C,3,1.39746848,1,60.85634185,6,-20.68817084,0\H,3,1.08670414,1,178.8
8682959,6,-175.2333333,0\H,4,1.08689072,3,120.22136388,1,179.34145529,
0\H,5,1.08877176,4,119.44452838,3,179.42057159,0\H,7,1.08787481,6,120.
8376531,5,179.48383837,0\H,8,1.08703615,3,120.19191675,1,-179.36383968
,0\P,1,1.87609341,6,113.29011961,5,128.63185291,0\C,1,4.34899727,6,117
.28655621,5,-100.3301163,0\C,15,1.39806809,1,59.38260805,6,142.4415693
7,0\C,16,1.39770111,15,120.01146883,1,-0.366885,0\C,17,1.40579031,16,1
20.7218386,15,0.30328641,0\C,18,1.40385795,17,118.86074513,16,-1.16299
698,0\C,19,1.39814646,18,120.34494802,17,1.07964087,0\H,15,1.08679914,
1,179.54264249,6,136.15219516,0\H,16,1.08701474,15,120.25841155,1,-179
.43788075,0\H,17,1.08933974,16,119.27835888,15,-178.13882761,0\H,19,1.
08623641,18,120.35111703,17,-177.1521181,0\H,20,1.0870773,19,119.51356
323,18,-179.45659126,0\C,14,1.84368519,1,114.26797916,6,85.2797478,0\C
,26,1.54241343,14,116.3085298,1,79.56436136,0\H,27,1.09498599,26,111.8
4745748,14,61.68981689,0\C,14,1.83702828,1,109.88859397,6,-35.99466495
,0\C,29,1.54086033,14,114.47195716,1,-59.02190531,0\H,30,1.09503113,29
,109.02264302,14,-178.35622461,0\C,14,1.83750995,1,107.84308074,6,-154
.08325543,0\C,32,1.54159004,14,114.33936659,1,173.71253674,0\H,33,1.09
535507,32,111.89349744,14,-63.43217835,0\H,26,1.09879527,14,105.412185
47,1,-157.94015833,0\H,26,1.09619029,14,107.44122199,1,-44.87106033,0\
H,27,1.09537693,26,108.50843856,14,-179.98606435,0\H,27,1.09301479,26,
112.10810634,14,-60.74973212,0\H,29,1.09688697,14,106.87429429,1,63.74
518695,0\H,29,1.09840358,14,106.48316057,1,178.00038315,0\H,30,1.09599
025,29,112.14727114,14,-59.38562459,0\H,30,1.09503124,29,111.52041557,
14,63.06176047,0\H,32,1.09708464,14,106.83762021,1,-62.91364565,0\H,32
,1.09805379,14,106.99771007,1,51.08587504,0\H,33,1.09551773,32,112.238
22981,14,58.6742788,0\H,33,1.09509107,32,109.08860672,14,177.72610199,
0\\Version=AM64L-G03RevD.01\State=1-A\HF=-1075.2641846\MP2=-1078.16168
28\RMSD=6.066e-09\Thermal=0.\PG=C01 [X(C19H26P1)]\\@

98-TT$^+$

1\1\GINC-NODE12\SP\RMP2-FC\6-31+G(2d,p)\C25H30P1(1+)\ZIP07\12-Sep-2011
\0\\#p MP2(FC)/6-31+G(2d,p) scf=tight\\k7ttsp_9\\1,1\C\P,1,1.97917476\
C,1,4.38561426,2,108.2674714\C,3,1.39528005,1,59.69087822,2,127.233337
62,0\C,4,1.40065334,3,120.65136945,1,-0.79334313,0\C,5,1.40498389,4,12
0.88855207,3,-0.24782014,0\C,6,1.40957046,5,117.6620952,4,1.80419949,0
\C,7,1.3957557,6,121.48158009,5,-2.10052025,0\H,3,1.08677996,1,179.463
62984,6,-179.21738613,0\H,4,1.08697009,3,120.1802736,1,178.84423149,0\
H,5,1.08392278,4,118.64506897,3,179.00458084,0\H,7,1.0864157,6,120.102
42952,5,175.58523495,0\H,8,1.08721321,7,119.62070775,6,-179.97161488,0
\C,1,4.38471444,6,109.84815168,5,11.25080961,0\C,14,1.39519754,1,59.72
854095,6,-116.19778977,0\C,15,1.40061367,14,120.64959382,1,-0.82742889
,0\C,16,1.40514172,15,120.87398873,14,-0.28158013,0\C,17,1.4093747,16,
117.68244147,15,1.79873182,0\C,18,1.3957663,17,121.47114196,16,-2.0574
2129,0\H,14,1.08676838,1,179.39072398,17,-179.26242696,0\H,15,1.086987
,14,120.17476437,1,178.76623399,0\H,16,1.08379654,15,118.66729069,14,1
78.88268643,0\H,18,1.08668823,17,120.05486438,16,175.54264221,0\H,19,1

.08719363,18,119.62613464,17,179.99612206,0\C,1,4.38514017,17,110.0288
5499,16,10.7194849,0\C,25,1.39524699,1,59.68126136,17,-115.84560787,0\
C,26,1.40056247,25,120.64379136,1,-0.81545972,0\C,27,1.40486386,26,120
.89057418,25,-0.26610723,0\C,28,1.40971966,27,117.68035331,26,1.808805
18,0\C,29,1.39573755,28,121.45788367,27,-2.08119301,0\H,25,1.08676939,
1,179.43271945,17,-10.92590514,0\H,26,1.08696943,25,120.18538141,1,178
.78086452,0\H,27,1.0839518,26,118.73906093,25,178.91257548,0\H,29,1.08
681272,28,120.1309753,27,175.59508867,0\H,30,1.08718978,29,119.6198304
5,28,-179.96920306,0\C,2,1.85368429,1,114.64737572,17,105.59990834,0\C
,36,1.53732738,2,118.68423184,1,-61.75948969,0\H,36,1.09997112,2,103.3
1082614,1,176.19746871,0\H,37,1.09546361,36,112.40061026,2,-57.1617656
9,0\H,37,1.09408292,36,111.69389046,2,66.05728161,0\C,2,1.85341925,1,1
14.77561315,17,-14.53443171,0\C,41,1.53768415,2,118.64228504,1,-62.172
8268,0\H,41,1.09986809,2,103.30366124,1,175.8278008,0\H,42,1.09563416,
41,112.41519633,2,-56.99299402,0\H,42,1.09402164,41,111.68880182,2,66.
2449536,0\C,2,1.85375746,1,114.70118016,17,-134.50600265,0\C,46,1.5373
2456,2,118.60768712,1,-62.69895819,0\H,46,1.09987846,2,103.35029492,1,
175.20294909,0\H,47,1.09554806,46,112.39649871,2,-57.12456844,0\H,47,1
.09405503,46,111.6148413,2,66.08952347,0\H,37,1.09557282,36,108.450328
78,2,-175.5561939,0\H,42,1.09558485,41,108.45040084,2,-175.42406218,0\
H,47,1.09552592,46,108.4840094,2,-175.56560514,0\H,36,1.09400106,2,107
.37691452,1,63.43819985,0\H,41,1.09391483,2,107.41388127,1,63.02733749
,0\H,46,1.09365193,2,107.3306094,1,62.39666013,0\\Version=AM64L-G03Rev
D.01\State=1-A\HF=-1304.7893556\MP2=-1308.558534\RMSD=2.065e-09\Therma
l=0.\PG=C01 [X(C25H30P1)]\\@

99

1\1\GINC-YANG\SP\RMP2-FC\6-31+G(2d,p)\C9H21P1\CHRISTOPH\14-Dec-2009\0\
\#p MP2(FC)/6-31+g(2d,p) scf=tight\\ih1sp_1\\0,1\C\H,1,1.10397098\C,1,
2.88773435,2,81.2394704\H,3,1.09714175,1,146.83701361,2,-104.1794593,0
\C,1,1.55138444,3,91.64176563,4,148.78218079,0\H,5,1.10075875,1,108.47
582772,3,135.97616382,0\H,5,1.09990315,1,108.944762,3,21.92183946,0\C,
1,1.54854159,5,113.52323795,3,159.86435913,0\H,8,1.09970975,1,108.8979
9752,5,178.30558476,0\H,8,1.1001214,1,109.83670998,5,-66.197652,0\C,5,
1.54004251,1,116.12918514,8,58.53902885,0\H,11,1.09908779,5,109.787261
49,1,55.69370345,0\H,11,1.0992645,5,109.77246202,1,-60.5764336,0\C,11,
1.53615528,5,112.24271757,1,177.85898053,0\H,14,1.09833968,11,111.1651
3571,5,-60.08382623,0\H,14,1.09713819,11,111.31874751,5,179.8442215,0\
H,14,1.09847517,11,111.22745809,5,59.70823531,0\P,3,1.86840364,1,40.26
07334,8,-20.52739402,0\C,18,1.86815242,3,97.91055886,1,101.65322588,0\
H,19,1.09568151,18,110.60067307,3,-177.89161886,0\C,8,1.5405627,1,114.
73351881,5,57.30132142,0\H,21,1.10079012,8,109.29620586,1,52.47165897,
0\H,21,1.09882396,8,110.10163057,1,-63.93839819,0\H,3,1.09579843,1,97.
63185369,8,-133.51076546,0\H,3,1.09914862,1,82.79887951,8,118.41613914
,0\H,19,1.09910797,18,112.75735735,3,-55.80141036,0\H,19,1.09710791,18
,108.72012759,3,64.51677711,0\C,21,1.53625836,8,112.40624555,1,174.250
74188,0\H,28,1.0981762,21,111.10517434,8,59.78268765,0\H,28,1.09719052
,21,111.34835304,8,179.85616067,0\H,28,1.09819486,21,111.15660442,8,-5
9.96555056,0\\Version=AM64L-G03RevD.01\State=1-A\HF=-693.812392\MP2=-6
95.340422\RMSD=4.846e-09\Thermal=0.\PG=C01 [X(C9H21P1)]\\@

99-Me$^+$

1\1\GINC-SOLARIS\SP\RMP2-FC\6-31+G(2d,p)\C10H24P1(1+)\CHRISTOPH\11-Dec
-2009\0\\#p MP2(FC)/6-31+g(2d,p) scf=tight\\ih1mesp_3\\1,1\C\H,1,1.101
9208\C,2,2.89878972,1,83.70459564\H,3,1.09583773,2,158.87830661,1,-71.
68510509,0\C,1,1.5536903,3,143.74523901,2,-98.11093578,0\H,5,1.0979372
,1,106.19695612,3,145.49888166,0\H,5,1.09916221,1,109.27335262,3,32.60
868072,0\C,1,1.55543,5,115.00708959,3,152.78895249,0\H,8,1.10097167,1,

109.4167703,5,-178.92369257,0\H,8,1.097683,1,109.57868765,5,-62.378828
81,0\C,5,1.5395269,1,117.25685785,8,60.09437245,0\H,11,1.09842356,5,11
0.08537488,1,-53.53877381,0\H,11,1.10181891,5,110.36690737,1,63.604580
54,0\C,11,1.53662969,5,111.78480653,1,-175.21444021,0\H,14,1.0973299,1
1,111.38298346,5,-59.54200236,0\H,14,1.09529495,11,110.9002781,5,-179.
67000735,0\H,14,1.09685254,11,110.95799091,5,60.55649574,0\C,3,2.93726
778,2,83.71528289,1,-5.66180058,0\H,18,1.09612701,3,88.63102233,2,-157
.52931962,0\C,8,1.54527894,1,114.10480478,5,59.99562161,0\H,20,1.09767
856,8,106.82136465,1,-178.08079107,0\H,20,1.09811693,8,109.86527194,1,
-63.75157063,0\H,3,1.09515099,2,90.85863421,1,86.81415258,0\H,3,1.0957
0898,2,70.35265202,1,-163.43174219,0\H,18,1.0943579,3,147.08927178,2,-
33.30400093,0\H,18,1.09499484,3,90.15232181,2,93.69056896,0\C,20,1.535
48261,8,114.61295697,1,60.78193611,0\H,27,1.09809468,20,111.35359151,8
,55.41890901,0\H,27,1.09538801,20,110.32945863,8,174.67302167,0\H,27,1
.09808467,20,112.46430985,8,-66.01276199,0\P,18,1.82156661,3,36.297667
67,2,-31.47554313,0\C,31,1.82078566,18,108.58201552,3,-115.86993629,0\
H,32,1.09574858,31,110.05000537,18,-179.93455859,0\H,32,1.09595686,31,
109.53207369,18,60.50865682,0\H,32,1.09476467,31,110.55922126,18,-59.4
9239148,0\\Version=AM64L-G03RevD.01\State=1-A\HF=-733.2621477\MP2=-734
.9357849\RMSD=9.484e-09\Thermal=0.\PG=C01 [X(C10H24P1)]\\@

100

1\1\GINC-NAUTILUS\SP\RMP2-FC\6-31+G(2d,p)\C7H17P1\CHRISTOPH\23-Sep-200
9\0\\#p MP2(FC)/6-31+g(2d,p) scf=tight\\pr2sp_3\\0,1\P\C,1,1.8674479\H
,2,1.09898979,1,112.71552647\H,2,1.09629713,1,110.00226858,3,121.41053
818,0\H,2,1.09695183,1,109.32479157,4,117.73277728,0\C,1,1.87545719,2,
98.66349113,4,173.599403,0\H,6,1.10190289,1,110.37852214,2,-54.7219548
1,0\H,6,1.1007157,1,106.49497028,2,60.66206929,0\C,1,1.87567065,2,99.8
7772502,6,-101.8120953,0\H,9,1.10017458,1,107.06086525,2,164.74279934,
0\H,9,1.10210231,1,110.16301661,2,48.97560969,0\C,9,1.54040792,1,113.3
8017954,2,-74.9840074,0\H,12,1.09925397,9,109.26573324,1,-54.77455406,
0\H,12,1.09957209,9,109.72476328,1,61.37257237,0\C,6,1.54027335,1,113.
60936147,2,-179.11124558,0\H,15,1.09921099,6,109.23091646,1,-54.567921
17,0\H,15,1.09967436,6,109.75279334,1,61.54348123,0\C,15,1.53649288,6,
112.65988412,1,-176.62236776,0\H,18,1.09811408,15,111.18447295,6,-59.9
1139396,0\H,18,1.09809991,15,111.03023921,6,59.88891995,0\H,18,1.09740
186,15,111.25787457,6,179.94086035,0\C,12,1.53663843,9,112.62855249,1,
-176.83695426,0\H,22,1.09738287,12,111.25474248,9,-179.87185659,0\H,22
,1.09808889,12,111.05191827,9,60.06168173,0\H,22,1.09808475,12,111.156
18464,9,-59.73841556,0\\Version=AM64L-G03RevD.01\State=1-A\HF=-615.740
8599\MP2=-616.9550677\RMSD=6.820e-09\Thermal=0.\PG=C01 [X(C7H17P1)]\\@

100-Me$^+$

1\1\GINC-NODE26\SP\RMP2-FC\6-31+G(2d,p)\C8H20P1(1+)\ZIP07\21-Sep-2009\
0\\#p MP2(FC)/6-31+g(2d,p) scf=tight\\pr2mesp_1\\1,1\P\C,1,1.82941757\
H,2,1.09893969,1,107.03208932\H,2,1.09971832,1,106.60859027,3,113.7313
1294,0\C,1,1.82942011,2,111.3032327,3,65.67251425,0\H,5,1.09971787,1,1
06.60754628,2,179.45229834,0\H,5,1.09893905,1,107.0337489,2,65.7210591
2,0\C,1,1.82014167,2,109.76124833,5,120.60261004,0\H,8,1.09508061,1,11
0.32113163,2,-62.48900982,0\H,8,1.09585121,1,109.97730231,2,57.7507314
,0\H,8,1.09577393,1,109.84702912,2,177.58207452,0\C,1,1.82014232,8,108
.20943866,5,119.24858347,0\H,12,1.09585193,1,109.97684565,8,-60.937373
26,0\H,12,1.0957736,1,109.84774315,8,58.89408544,0\H,12,1.09508191,1,1
10.32104923,8,178.82347342,0\C,2,1.5467295,1,115.04397492,8,62.9672222
4,0\H,16,1.09841893,2,109.8039199,1,59.13010873,0\H,16,1.09820878,2,10
9.89543285,1,-58.28970761,0\C,5,1.54672882,1,115.04334176,8,-178.71024
439,0\H,19,1.0982108,5,109.89624824,1,-58.27663721,0\H,19,1.09841559,5
,109.80257567,1,59.14307158,0\C,16,1.53689696,2,111.15236091,1,-179.58

823244,0\H,22,1.09511245,16,110.25512475,2,-179.98818545,0\H,22,1.0966
5531,16,111.27146124,2,-60.32901552,0\H,22,1.09661758,16,111.29313884,
2,60.36737656,0\C,19,1.53689691,5,111.15293495,1,-179.57608544,0\H,26,
1.09665513,19,111.27134019,5,-60.35488629,0\H,26,1.09661867,19,111.293
41396,5,60.34164875,0\H,26,1.09511262,19,110.2549251,5,179.98608623,0\
\Version=AM64L-G03RevD.01\State=1-A\HF=-655.192286\MP2=-656.5513837\RM
SD=3.267e-09\Thermal=0.\PG=C01 [X(C8H20P1)]\\@

101

1\1\GINC-NODE13\SP\RMP2-FC\6-31+G(2d,p)\C18H23P1\ZIP07\21-Apr-2010\0\\
#p MP2(FC)/6-31+G(2d,p) scf=tight\\bm22sp_17\\0,1\P\C,1,4.67098869\C,2
,1.40049421,1,61.19133041\C,3,1.39680031,2,120.0919499,1,0.63803855,0\
C,4,1.40905733,3,120.81568546,2,-0.01026686,0\C,5,1.40512924,4,118.356
23321,3,0.36288517,0\C,2,1.3967116,1,58.60333643,5,156.50598864,0\H,2,
1.0878455,1,178.58016461,5,-173.13413275,0\H,3,1.08809908,2,120.058006
37,1,-179.41772775,0\H,4,1.08840534,3,119.40960059,2,179.23852546,0\H,
6,1.08841816,5,119.21271279,4,179.83836465,0\H,7,1.08785614,2,120.1437
6668,1,179.20576908,0\C,1,4.67880672,5,105.26050532,4,-57.07098239,0\C
,13,1.3990601,1,58.7040627,5,161.97340379,0\C,14,1.39617482,13,120.178
99277,1,-1.70209293,0\C,15,1.4096223,14,121.14966444,13,-0.92149686,0\
C,16,1.40620301,15,117.98357155,14,1.7712776,0\C,13,1.3969083,1,60.660
15494,5,-16.63455599,0\H,13,1.08767663,1,178.11051072,5,-139.5632327,0
\H,14,1.08811884,13,120.15165631,1,177.89163236,0\H,15,1.0892479,14,11
8.95333232,13,178.56328079,0\H,17,1.08672875,16,119.93334345,15,178.84
170065,0\H,18,1.08822171,13,120.0664505,1,-178.14023285,0\C,1,1.894454
2,5,102.07962045,4,49.06539914,0\C,24,1.55739129,1,115.70938557,5,103.
61693826,0\C,25,1.55466871,24,112.33157272,1,-58.36896969,0\H,26,1.100
68297,25,108.3456981,24,60.12777751,0\H,26,1.10082904,25,107.62555652,
24,173.9945773,0\C,26,1.53726489,25,116.93373154,24,-63.49429997,0\H,2
9,1.09726336,26,109.98412161,25,178.90901942,0\H,29,1.09634125,26,112.
1255054,25,59.77118986,0\H,29,1.09686173,26,111.84262125,25,-61.902248
7,0\C,25,1.54537905,24,109.82595804,1,62.05995391,0\H,33,1.09685022,25
,111.52365858,24,-63.89887157,0\H,33,1.09832938,25,110.6132293,24,175.
93305547,0\H,33,1.09921146,25,111.00838921,24,56.52059912,0\C,25,1.546
70401,24,107.65555648,1,179.76691294,0\H,37,1.0969892,25,111.9609622,2
4,57.08738238,0\H,37,1.09830977,25,110.70137331,24,-62.92042634,0\H,37
,1.09879103,25,110.7860725,24,177.64918576,0\H,24,1.09843235,1,106.649
9153,5,-135.92501607,0\H,24,1.09815244,1,109.38105005,5,-21.47740617,0
\\Version=AM64L-G03RevD.01\State=1-A\HF=-1035.8122345\MP2=-1038.566062
4\RMSD=7.465e-09\Thermal=0.\PG=C01 [X(C18H23P1)]\\@

101-Me^{+}

1\1\GINC-NODE24\SP\RMP2-FC\6-31+G(2d,p)\C19H26P1(1+)\ZIP07\24-Apr-2010
\0\\#p MP2(FC)/6-31+G(2d,p) scf=tight\\bm22mesp_100\\1,1\C\C,1,1.39896
17\C,2,1.39639978,1,120.15760268\C,3,1.40871628,2,119.88408199,1,-0.01
113998,0\C,4,1.40836052,3,119.76728886,2,0.23175263,0\C,5,1.39665196,4
,119.8543904,3,-0.31835161,0\H,1,1.08646,6,119.88291494,5,-179.7976340
1,0\H,2,1.08627541,1,120.23758836,6,-179.76124298,0\H,3,1.08705067,2,1
19.58811672,1,-179.0576878,0\H,5,1.08598978,4,121.18298565,3,179.86027
077,0\H,6,1.08625006,5,119.58441942,4,-179.64901657,0\C,3,5.29023895,2
,141.12389659,1,-134.32846399,0\C,12,1.39783538,3,77.8896403,2,-145.96
608885,0\C,13,1.39794229,12,120.05877059,3,-27.73535312,0\C,14,1.40577
724,13,119.82411028,12,0.04667598,0\C,15,1.41036606,14,119.98609281,13
,0.57370973,0\C,16,1.39468624,15,119.71630261,14,-0.73263829,0\H,12,1.
08649399,3,148.72628481,2,-17.01288066,0\H,13,1.08617183,12,120.281632
8,3,152.19359977,0\H,14,1.08650008,13,119.25980091,12,-179.96096538,0\
H,16,1.08679106,15,120.63382662,14,177.98992109,0\H,17,1.08623833,16,1
19.72747502,15,179.8108978,0\C,4,2.96080023,3,151.83097651,2,-161.2137

3935,0\C,23,1.56740706,4,156.11994256,3,-7.23552453,0\C,24,1.55596074,
23,112.98156106,4,52.67509955,0\C,25,1.53691531,24,117.13812815,23,57.
06365233,0\H,26,1.09839709,25,112.89066244,24,-62.9697095,0\H,26,1.095
65264,25,109.72155532,24,178.06979247,0\H,26,1.09563563,25,112.1366958
6,24,59.00059946,0\H,23,1.0974351,4,77.48129747,3,103.8142929,0\H,23,1
.09946112,4,90.75863576,3,-149.55043729,0\P,15,1.8080479,14,120.963524
26,13,-177.69570616,0\C,32,1.8259884,15,109.4812494,14,-18.38946964,0\
H,33,1.09558407,32,108.13986114,15,-175.11791959,0\H,33,1.0925604,32,1
12.34815624,15,64.45013378,0\H,33,1.09497738,32,108.88135269,15,-57.52
467583,0\C,24,1.54301782,23,111.41658234,4,-69.92027646,0\H,37,1.09593
023,24,109.88795874,23,-170.79946912,0\H,37,1.09829437,24,111.87271121
,23,-51.76661858,0\H,37,1.09813819,24,112.03662021,23,70.71143298,0\C,
24,1.54915318,23,105.29904366,4,172.89678883,0\H,41,1.09635639,24,109.
95702926,23,-175.35949119,0\H,41,1.09590037,24,112.27484364,23,-55.364
71027,0\H,41,1.09766449,24,111.16496415,23,65.52945953,0\H,25,1.098765
87,24,106.42061506,23,178.44414044,0\H,25,1.097323,24,109.36058599,23,
-67.92362262,0\\Version=AM64L-G03RevD.01\State=1-A\HF=-1075.2622681\MP
2=-1078.1614978\RMSD=3.307e-09\Thermal=0.\PG=C01 [X(C19H26P1)]\\@

102

1\1\GINC-NODE10\SP\RMP2-FC\6-31+G(2d,p)\C16H19P1\ZIP07\11-Apr-2010\0\\
#p MP2(FC)/6-31+G(2d,p) scf=tight\\bm0sp_29\\0,1\P\C,1,4.6731981\C,2,1
.39993317,1,58.27249826\C,3,1.39528763,2,120.10836412,1,-0.71566543,0\
C,4,1.4097556,3,121.0976194,2,-0.91130616,0\C,5,1.40582965,4,118.12598
329,3,0.94864067,0\C,2,1.39643932,1,61.25126382,5,-32.38795599,0\H,2,1
.08772919,1,178.17170291,5,-177.76257593,0\H,3,1.08798897,2,120.119314
94,1,178.79569703,0\H,4,1.08900164,3,119.43834042,2,179.19932072,0\H,6
,1.08680351,5,120.36816165,4,179.69021609,0\H,7,1.08817341,2,120.08412
06,1,-178.93222527,0\C,1,4.6723687,5,103.37936446,4,72.94981028,0\C,13
,1.39729377,1,58.73726498,5,-146.91906293,0\C,14,1.39936211,13,119.973
80762,1,0.88794999,0\C,15,1.40594096,14,120.85483322,13,-0.60938419,0\
C,16,1.40733082,15,118.54441306,14,0.72336884,0\C,17,1.39696264,16,120
.65937761,15,-0.3443102,0\H,13,1.08791462,1,178.82464167,5,-165.718609
61,0\H,14,1.08800191,13,120.14720431,1,-179.61264432,0\H,15,1.08869142
,14,119.77734003,13,179.09365679,0\H,17,1.08821972,16,119.68767985,15,
178.94547637,0\H,18,1.08819454,17,119.79657991,16,179.78994387,0\C,1,1
.87530862,5,102.48963184,4,176.84487972,0\H,24,1.09991572,1,107.015157
84,5,63.24982974,0\H,24,1.09997217,1,110.79147443,5,-53.67121152,0\C,2
4,1.53997204,1,111.97599193,5,-176.92388839,0\H,27,1.1002948,24,109.44
232242,1,-56.16251171,0\H,27,1.09966496,24,109.52013373,1,60.13251595,
0\C,27,1.53887527,24,112.72587006,1,-178.10165248,0\H,30,1.10032006,27
,109.22546938,24,-57.83667857,0\H,30,1.10046174,27,109.28336044,24,58.
02868094,0\C,30,1.53575232,27,112.80399166,24,-179.88456185,0\H,33,1.0
9797331,30,111.07460344,27,59.85548551,0\H,33,1.09806127,30,111.100709
15,27,-59.91058684,0\H,33,1.09711469,30,111.33504054,27,179.97770122,0
\\Version=AM64L-G03RevD.01\State=1-A\HF=-957.7457288\MP2=-960.1749499\
RMSD=3.348e-09\Thermal=0.\PG=C01 [X(C16H19P1)]\\@

102-Me$^+$

1\1\GINC-NODE9\SP\RMP2-FC\6-31+G(2d,p)\C17H22P1(1+)\ZIP07\16-Apr-2010\
0\\#p MP2(FC)/6-31+G(2d,p) scf=tight\\bm0mesp_2\\1,1\C\C,1,1.39825927\
C,2,1.39673054,1,120.12561639\C,3,1.40783589,2,119.85404434,1,-0.13284
187,0\C,4,1.41015209,3,119.83003737,2,0.09330424,0\C,5,1.39558614,4,11
9.80775396,3,-0.02753575,0\H,1,1.08684236,2,119.8729003,3,179.89603249
,0\H,2,1.08655103,1,120.25401739,6,-179.69189482,0\H,3,1.08666711,2,11
9.09185602,1,-179.82334929,0\H,5,1.0873124,4,120.55048747,3,-179.19685
989,0\H,6,1.08660884,5,119.65164718,4,-179.76032735,0\C,5,5.46480955,4
,84.98936563,3,-149.34865731,0\C,12,1.39919141,5,44.74346425,4,86.1372

1646,0\C,13,1.39595565,12,120.13752089,5,-32.44046535,0\C,14,1.4088843
4,13,119.78468874,12,0.03693186,0\C,15,1.4082432,14,119.88878767,13,-0
.31341133,0\C,16,1.39633518,15,119.82840099,14,0.40737199,0\H,12,1.086
84757,5,150.21899352,4,156.36868397,0\H,13,1.08659017,12,120.21333971,
5,147.42711611,0\H,14,1.08726636,13,119.67248615,12,179.50837706,0\H,1
6,1.0869227,15,121.00745392,14,-179.68319472,0\H,17,1.08656174,16,119.
66110733,15,179.64242026,0\C,4,2.99297268,3,118.10872539,2,136.7123570
8,0\C,23,1.54315805,4,95.81501421,3,-127.41069911,0\C,24,1.54064057,23
,111.40675912,4,150.18649642,0\C,25,1.53505189,24,112.32922868,23,-179
.92195154,0\H,26,1.09741401,25,111.29350548,24,-60.07310915,0\H,26,1.0
9732189,25,111.27890029,24,60.35985875,0\H,26,1.09550514,25,110.707190
05,24,-179.86419115,0\P,4,1.80671051,3,120.5071101,2,177.04176619,0\C,
30,1.82524254,4,109.62236784,3,20.90750159,0\H,31,1.09518631,30,111.13
022676,4,-64.76836094,0\H,31,1.09552249,30,108.41264152,4,175.70479664
,0\H,31,1.09488515,30,110.05999645,4,56.90665394,0\H,25,1.09981111,24,
109.29197118,23,58.12679222,0\H,25,1.09948615,24,109.21104253,23,-57.9
7276811,0\H,24,1.09883887,23,109.98183144,4,-88.19295165,0\H,24,1.0983
3831,23,109.48868366,4,28.9837306,0\H,23,1.09945723,4,141.54102515,3,9
7.24649364,0\H,23,1.09968874,4,88.05635012,3,-17.0823996,0\\Version=AM
64L-G03RevD.01\State=1-A\HF=-997.1970822\MP2=-999.7718929\RMSD=4.432e-
09\Thermal=0.\PG=C01 [X(C17H22P1)]\\@

102-BH$^+$

1\1\GINC-NODE17\SP\RMP2-FC\6-31+G(2d,p)\C29H30P1(1+)\ZIP07\26-Jul-2010
\0\\#p MP2(FC)/6-31+G(2d,p) scf=tight\\xbubhsp_158\\1,1\C\H,1,1.099418
87\P,1,1.88865891,2,101.33454354\C,1,4.34372713,3,112.09865711,2,111.9
208975,0\C,4,1.39782377,1,58.96407712,3,-122.62585867,0\C,5,1.39731955
,4,120.09305291,1,0.39992805,0\C,6,1.40468383,5,120.82860115,4,-0.4838
3336,0\C,7,1.40695857,6,118.63369831,5,0.53991613,0\C,4,1.39809421,1,6
0.66873455,7,8.72082922,0\H,4,1.08685,1,179.11715511,7,168.58817084,0\
H,5,1.08699734,4,120.22601764,1,-179.60382799,0\H,6,1.08884937,5,119.4
5369887,4,-179.53127257,0\H,8,1.08769591,7,120.43129068,6,179.91864865
,0\H,9,1.08721731,4,120.10764752,1,179.588083,0\C,1,4.34914848,7,118.2
6916435,6,105.93146807,0\C,15,1.39869083,1,59.26284767,7,-150.92203142
,0\C,16,1.3970312,15,120.08852126,1,0.32184782,0\C,17,1.40727193,16,12
0.73942769,15,-0.48320599,0\C,18,1.40367236,17,118.72410219,16,1.06420
27,0\C,15,1.39749092,1,60.29605078,7,28.3537309,0\H,15,1.08701241,1,17
9.42050104,7,-129.20744184,0\H,16,1.08715139,15,120.24790394,1,179.363
07976,0\H,17,1.08784759,16,119.28836664,15,178.91303293,0\H,19,1.08515
908,18,120.33348189,17,177.63404227,0\H,20,1.08716143,15,120.11552755,
1,-179.45994378,0\C,3,4.61279016,1,111.28273893,7,-81.37673071,0\C,26,
1.39887716,3,59.91464307,1,-65.09762488,0\C,27,1.39621535,26,120.29075
631,3,-0.46356203,0\C,28,1.41149548,27,119.8083834,26,-0.15443658,0\C,
29,1.40906101,28,119.60135334,27,0.30208222,0\C,30,1.39638377,29,120.0
360492,28,-0.25900495,0\H,26,1.08695143,3,179.58820052,1,3.55900667,0\
H,27,1.08666257,26,120.22372238,3,179.36432836,0\H,28,1.08624233,27,11
9.53382271,26,179.7208321,0\H,30,1.08592977,29,120.9012788,28,179.5961
5523,0\H,31,1.08664931,30,119.61089174,29,-179.82762021,0\C,3,4.620135
02,1,109.19435944,7,154.48990182,0\C,37,1.400263,3,59.46000457,1,-50.9
3260755,0\C,38,1.39503975,37,120.03784064,3,1.02142911,0\C,39,1.410489
41,38,120.12817743,37,0.20741579,0\C,40,1.40529598,39,119.54761962,38,
-0.92348197,0\C,37,1.39695942,3,60.6091004,1,128.47346665,0\H,37,1.086
85238,3,178.96816479,1,-102.69473811,0\H,38,1.08673429,37,120.24896408
,3,-178.85176158,0\H,39,1.08845464,38,118.89367992,37,-179.46109444,0\
H,41,1.08578555,40,120.50807812,39,-178.84591565,0\H,42,1.08670708,37,
120.20888223,3,178.92125123,0\C,3,1.8384376,1,107.40233973,7,40.499733
95,0\H,48,1.09969382,3,105.46980911,1,42.48434261,0\H,48,1.09717775,3,
107.45971015,1,-69.84518452,0\C,48,1.54434163,3,115.37554303,1,164.552

05724,0\H,51,1.09775896,48,109.40933429,3,-60.97168718,0\H,51,1.098851
82,48,110.41079799,3,55.83648254,0\C,51,1.54045097,48,111.39703715,3,1
77.62323262,0\H,54,1.09990818,51,109.36682209,48,-58.95614545,0\H,54,1
.09976618,51,109.24215285,48,57.05755269,0\C,54,1.53514217,51,112.3062
2613,48,179.01388527,0\H,57,1.09747293,54,111.27511979,51,60.08963023,
0\H,57,1.09729243,54,111.23775432,51,-60.19978535,0\H,57,1.09567061,54
,110.80534401,51,179.93888373,0\\Version=AM64L-G03RevD.01\State=1-A\HF
=-1456.3112229\MP2=-1460.5840858\RMSD=9.795e-09\Thermal=0.\PG=C01 [X(C
29H30P1)]\\@

102-TT$^+$

1\1\GINC-CIPCLU07\SP\RMP2-FC\6-31+G(2d,p)\C35H34P1(1+)\LICCH\07-Sep-20
11\0\\#p MP2(FC)/6-31+G(2d,p) scf=tight\\xbuttsp_82\\1,1\C\P,1,1.97541
151\C,1,4.39237528,2,106.64062081\C,3,1.39943985,1,58.88562026,2,71.29
676533,0\C,4,1.39553472,3,120.18339143,1,0.56291954,0\C,5,1.4129479,4,
121.51199921,3,-1.45218063,0\C,6,1.40439702,5,117.37486118,4,3.5461521
6,0\C,3,1.39478604,1,60.1829426,6,-10.93605772,0\H,3,1.08693673,1,179.
29175985,6,162.06203472,0\H,4,1.08728367,3,120.29616436,1,179.03519171
,0\H,5,1.08595764,4,118.51788038,3,175.12651329,0\H,7,1.08281779,6,120
.62138593,5,175.45004516,0\H,8,1.08718122,3,120.18432713,1,-179.367069
38,0\C,1,4.38408128,6,107.90049451,5,-49.96038416,0\C,14,1.39845753,1,
58.33552032,6,-37.44395051,0\C,15,1.39572512,14,120.44032491,1,1.41111
396,0\C,16,1.4097863,15,121.1216807,14,-0.25025925,0\C,17,1.40329834,1
6,117.70700668,15,-1.34024737,0\C,14,1.3948082,1,60.78451635,17,41.514
0807,0\H,14,1.08687976,1,178.57522615,17,-169.29460151,0\H,15,1.087156
51,14,120.23575433,1,-178.67593408,0\H,16,1.08575019,15,118.93566945,1
4,-179.85981254,0\H,18,1.08341471,17,121.02485401,16,-175.81120353,0\H
,19,1.08727495,14,120.29433737,1,178.61657008,0\C,1,4.38782569,17,110.
67654453,16,86.59754722,0\C,25,1.395827,1,59.26383199,17,1.62438873,0\
C,26,1.39892698,25,120.60666699,1,-0.27490831,0\C,27,1.40629555,26,121
.10729876,25,-0.32884232,0\C,28,1.41268708,27,117.60104187,26,0.478652
52,0\C,29,1.39766203,28,121.19769889,27,-0.37546219,0\H,25,1.08702876,
1,179.74260276,28,130.80321828,0\H,26,1.08714246,25,120.19778329,1,179
.73978586,0\H,27,1.08385109,26,118.65867141,25,179.82645893,0\H,29,1.0
8445236,28,120.55320996,27,179.21497552,0\H,30,1.08737724,29,119.34388
474,28,-179.64119038,0\C,2,4.6341316,1,112.43796677,28,-71.78717343,0\
C,36,1.39727972,2,60.21988565,1,118.66195686,0\C,37,1.39723024,36,120.
29343671,2,-1.1316248,0\C,38,1.40757117,37,120.14212842,36,-0.32081428
,0\C,39,1.41156214,38,119.32313293,37,1.24933197,0\C,40,1.39611536,39,
119.98428996,38,-1.29780814,0\H,36,1.08699221,2,178.94090891,1,-133.78
535093,0\H,37,1.08683023,36,120.27057402,2,178.51697527,0\H,38,1.08489
885,37,119.17354711,36,179.11096928,0\H,40,1.08536275,39,120.96838751,
38,178.62620757,0\H,41,1.08682932,40,119.38550473,39,-179.82289214,0\C
,2,4.64754158,1,112.40216977,28,51.97742876,0\C,47,1.3972348,2,60.1740
0953,1,-60.78752063,0\C,48,1.39716395,47,120.51862184,2,0.11583034,0\C
,49,1.41043916,48,120.21772983,47,-0.23807053,0\C,50,1.41111179,49,118
.78383259,48,0.4286887,0\C,51,1.39599869,50,120.53407136,49,-0.3145692
,0\H,47,1.08690172,2,179.65221142,1,127.29455999,0\H,48,1.08680169,47,
120.25655034,2,179.87248325,0\H,49,1.08413221,48,118.95977152,47,179.8
4285176,0\H,51,1.08502503,50,121.03817618,49,178.99320937,0\H,52,1.086
77598,51,119.44968962,50,-179.94534942,0\C,2,1.84716401,1,111.65513033
,28,172.91745195,0\H,58,1.09718132,2,107.92128218,1,-67.12596594,0\H,5
8,1.09455894,2,105.89450427,1,46.47725672,0\C,58,1.54783063,2,115.3017
9212,1,166.79248539,0\H,61,1.09690092,58,109.07994768,2,-63.98912587,0
\H,61,1.0972444,58,111.24183137,2,52.97359854,0\C,61,1.54093318,58,110
.95755754,2,175.16033402,0\H,64,1.10016882,61,109.44846113,58,-59.7809
3199,0\H,64,1.09972874,61,109.29367971,58,56.27822837,0\C,64,1.5353643
3,61,112.26430203,58,178.19462211,0\H,67,1.09725823,64,111.21268461,61

,-60.41584174,0\H,67,1.09745208,64,111.2438356,61,59.80014873,0\H,67,1
.09575243,64,110.82866001,61,179.69038749,0\\Version=AM64L-G03RevD.01\
State=1-A\HF=-1685.8341312\MP2=-1690.9833618\RMSD=2.999e-10\Thermal=0.
\PG=C01 [X(C35H34P1)]\\@

103

1\1\GINC-CIPCLU06\SP\RMP2-FC\6-31+G(2d,p)\C7H17P1\C4371\28-Oct-2009\0\
\#P MP2(FC)/6-31+G(2d,p) scf=tight\\i2sp_1\\0,1\P\C,1,1.8942321\H,2,1.
100684,1,103.70402469\C,1,1.86867922,2,101.52860802,3,-171.09941984,0\
H,4,1.09659343,1,109.49944761,2,-62.18852994,0\C,1,1.88931012,4,100.08
672252,2,-105.47819817,0\H,6,1.10085521,1,109.26270772,4,47.37398999,0
\C,2,1.54285703,1,109.09490966,4,75.03278316,0\H,8,1.09674062,2,111.56
110629,1,52.02116446,0\H,8,1.09867108,2,110.47558741,1,172.28597111,0\
H,8,1.09818294,2,111.38741176,1,-68.6158245,0\C,2,1.53866155,1,117.091
14015,4,-51.98972533,0\H,12,1.09662645,2,112.36221718,1,-56.98831673,0
\H,12,1.09746184,2,111.49581281,1,64.3469321,0\H,12,1.09946822,2,110.2
4579006,1,-176.75894204,0\C,6,1.54028339,1,110.80144009,4,167.203326,0
\H,16,1.09882594,6,110.36575164,1,175.34620589,0\H,16,1.09839221,6,111
.02346142,1,56.06096171,0\H,16,1.09674439,6,112.22767365,1,-64.7935091
6,0\C,6,1.54211508,1,109.40692579,4,-70.92931661,0\H,20,1.09673114,6,1
12.17432256,1,61.36150296,0\H,20,1.09886698,6,110.31429996,1,-178.8095
233,0\H,20,1.09804134,6,110.99822336,1,-59.40154129,0\H,4,1.09596048,1
,109.18567509,6,75.44518856,0\H,4,1.09777921,1,113.78620852,6,-45.8960
3885,0\\Version=AM64L-G03RevD.01\State=1-A\HF=-615.7331274\MP2=-616.95
7188\RMSD=3.249e-09\Thermal=0.\PG=C01 [X(C7H17P1)]\\@

103-Me$^+$

1\1\GINC-CIPCLU10\SP\RMP2-FC\6-31+G(2d,p)\C8H20P1(1+)\C4371\28-Oct-200
9\0\\#P MP2(FC)/6-31+G(2d,p) scf=tight\\i2mesp_1\\1,1\C\H,1,1.09989617
\C,1,3.0039582,2,139.04953899\H,3,1.09477419,1,145.28814171,2,-3.94466
332,0\C,3,2.9940422,1,61.0964066,2,39.14165982,0\H,5,1.09989949,3,84.9
8878079,1,89.51534987,0\C,1,1.54464142,3,94.83100399,5,-87.21848522,0\
H,7,1.09570607,1,108.69423245,3,-150.72760479,0\H,7,1.09661408,1,111.7
7476943,3,-32.47164761,0\H,7,1.09558961,1,112.81050541,3,90.33559587,0
\C,1,1.54497632,7,111.7008017,5,153.40720528,0\H,11,1.09578981,1,108.5
3425156,7,58.41498483,0\H,11,1.09541298,1,112.83620991,7,177.14962408,
0\H,11,1.09632704,1,111.85568769,7,-59.76212281,0\C,5,1.54464445,3,146
.58717214,1,-27.67457849,0\H,15,1.09661462,5,111.77463447,3,-68.029347
48,0\H,15,1.09570604,5,108.69383758,3,173.7153495,0\H,15,1.09558768,5,
112.81175949,3,54.77899385,0\C,5,1.54497356,3,91.07816896,1,-162.11096
69,0\H,19,1.09632595,5,111.85567759,3,95.20391071,0\H,19,1.09541473,5,
112.8358255,3,-27.88416316,0\H,19,1.09579026,5,108.53434938,3,-146.618
91957,0\P,3,1.82288692,1,35.43062967,7,-121.72099709,0\C,23,1.82288834
,3,107.95927591,1,-118.92259503,0\H,24,1.09539987,23,110.3711104,3,176
.05316664,0\H,24,1.09508105,23,110.01474686,3,-63.66033719,0\H,24,1.09
477391,23,110.0835016,3,55.89049544,0\H,3,1.09508124,1,88.19258739,7,1
08.85519617,0\H,3,1.09540057,1,93.10787435,7,-0.05074457,0\\Version=AM
64L-G03RevD.01\State=1-A\HF=-655.1856897\MP2=-656.5540329\RMSD=3.340e-
09\Thermal=0.\PG=C01 [X(C8H20P1)]\\@

104

1\1\GINC-GOLEM\SP\RMP2-FC\6-31+G(2d,p)\C9H19P1\CHRISTOPH\07-Sep-2009\0
\\#p MP2(FC)/6-31+g(2d,p) scf=tight\\hep1sp_33\\0,1\P\H,1,2.45360139\C
,1,1.86848573,2,81.39104021\H,3,1.0962019,1,110.51049322,2,88.48172023
,0\H,3,1.09712953,1,108.96448005,2,-153.83027924,0\H,3,1.09902677,1,11
2.70164596,2,-33.31832925,0\C,1,1.86774085,3,97.99520424,4,173.7969003
8,0\H,7,1.09918726,1,112.73863362,3,-54.82388757,0\H,7,1.09713226,1,10
8.85506974,3,65.55258908,0\H,7,1.09564782,1,110.35682109,3,-176.684686

22,0\C,1,1.88982453,7,100.66267892,3,102.08971073,0\C,11,1.55071043,1,
109.84489137,7,-174.13113726,0\C,12,1.54454867,11,113.49784053,1,150.9
3799751,0\C,13,1.53874734,12,116.23871369,11,72.71160584,0\C,14,1.5415
7503,13,116.16224706,12,-52.81503852,0\C,15,1.54382961,14,113.39235899
,13,68.26117156,0\C,16,1.54624735,15,116.22136356,14,-86.26320852,0\H,
12,1.09991419,11,109.98430594,1,-84.56020566,0\H,12,1.09932074,11,109.
62844249,1,31.03117481,0\H,13,1.1001458,12,108.77468076,11,-165.151872
06,0\H,13,1.10154916,12,108.11199674,11,-50.52156475,0\H,14,1.10153956
,13,108.91123251,12,69.27672234,0\H,14,1.10002955,13,108.20279638,12,-
175.88802213,0\H,15,1.09933414,14,107.795337,13,-171.47764793,0\H,15,1
.10124372,14,110.35939999,13,-56.1442644,0\H,16,1.10002453,15,108.2278
4016,14,37.47341092,0\H,16,1.09933027,15,109.47505634,14,152.07069858,
0\H,17,1.09880161,16,108.58895681,15,-80.82213823,0\H,17,1.10173123,16
,107.51697738,15,164.92348961,0\\Version=AM64L-G03RevD.01\State=1-A\HF
=-692.6422042\MP2=-694.1502726\RMSD=5.073e-09\Thermal=0.\PG=C01 [X(C9H
19P1)]\\@

104-Me^{+}

1\1\GINC-GOLEM\SP\RMP2-FC\6-31+G(2d,p)\C10H22P1(1+)\CHRISTOPH\07-Sep-2
009\0\\#p MP2(FC)/6-31+g(2d,p) scf=tight\\hep1mesp_12\\1,1\P\C,1,1.843
23728\C,2,1.55798113,1,109.85070638\C,3,1.54682279,2,115.34258269,1,-1
63.72347805,0\C,4,1.5435118,3,116.33995987,2,-41.34626315,0\C,5,1.5414
1789,4,113.43493988,3,86.81919244,0\C,6,1.538216,5,116.31862297,4,-68.
9501931,0\C,7,1.54640471,6,116.07832192,5,53.53445766,0\H,2,1.10156002
,1,103.43706231,3,115.53534561,0\H,3,1.09802776,2,107.80332081,1,74.21
448182,0\H,3,1.10069896,2,110.41382692,1,-41.64964335,0\H,4,1.09858647
,3,109.39575529,2,82.26813468,0\H,4,1.09735412,3,106.33691569,2,-163.9
9264255,0\H,5,1.09710171,4,108.09442927,3,-153.72950249,0\H,5,1.101356
57,4,110.7341682,3,-38.36428512,0\H,6,1.10030868,5,108.22171536,4,54.0
7512814,0\H,6,1.09790412,5,109.10287949,4,169.03277066,0\H,7,1.097873,
6,108.43216358,5,175.09563808,0\H,7,1.10099857,6,109.73901075,5,-69.50
323658,0\H,8,1.09836576,7,110.76495666,6,50.92636971,0\H,8,1.09994723,
7,107.22260475,6,166.41050925,0\C,1,1.82131252,2,110.57064946,8,64.998
75081,0\H,22,1.09477681,1,110.74544034,2,-60.57336259,0\H,22,1.0957678
,1,109.90634312,2,59.8066615,0\H,22,1.09594856,1,109.67854514,2,179.42
75658,0\C,1,1.82078445,22,107.52490887,2,-121.04419495,0\H,26,1.095890
42,1,109.70756521,22,-55.79949528,0\H,26,1.09460096,1,110.4276414,22,-
175.83934318,0\H,26,1.09589594,1,110.03003228,22,63.87512647,0\C,1,1.8
1989455,26,108.53856806,22,117.08029577,0\H,30,1.09577347,1,109.724541
4,26,-57.93148838,0\H,30,1.09518456,1,110.37505728,26,61.89619288,0\H,
30,1.09516279,1,110.12382882,26,-177.78482897,0\\Version=AM64L-G03RevD
.01\State=1-A\HF=-732.0945907\MP2=-733.7466761\RMSD=7.300e-09\Thermal=
0.\PG=C01 [X(C10H22P1)]\\@

105

1\1\GINC-NODE-07\SP\RMP2-FC\6-31+G(2d,p)\C12H27P1\ZIP07\13-May-2009\0\
\#p MP2(FC)/6-31+g(2d,p) scf=tight\\tibpspc070\\0,1\P\C,1,1.88539318\H
,2,1.1012228,1,107.41805054\H,2,1.10141154,1,109.05734886,3,-114.81438
931,0\C,1,1.88042524,2,100.5131107,3,136.95993099,0\H,5,1.1015888,1,10
6.57736741,2,-165.94634107,0\H,5,1.10172694,1,109.08229977,2,-51.21488
358,0\C,1,1.88007338,5,97.44312822,2,100.90775765,0\H,8,1.10158373,1,1
09.33610364,5,-61.42626288,0\H,8,1.10115697,1,107.10611921,5,53.629386
79,0\C,5,1.54685582,1,115.95568383,8,173.72367245,0\H,11,1.10165475,5,
108.19238715,1,-56.50290749,0\C,2,1.54843378,1,115.00469073,8,158.4541
4728,0\H,13,1.10167944,2,108.47294569,1,50.4238024,0\C,8,1.54760421,1,
114.66877054,5,174.59296832,0\H,15,1.10239655,8,108.3621147,1,51.45365
662,0\C,11,1.54064184,5,110.03069767,1,-173.56191242,0\H,17,1.09788042
,11,111.4149222,5,58.02097023,0\H,17,1.09795798,11,111.09397458,5,178.

42101055,0\H,17,1.09912203,11,110.76100979,5,-61.9107623,0\C,11,1.5389
3885,5,112.25332133,1,62.76466301,0\H,21,1.09638276,11,111.7083618,5,-
58.22592466,0\H,21,1.09928434,11,110.54243102,5,61.55616197,0\H,21,1.0
9775741,11,110.86420098,5,-179.02295036,0\C,13,1.5385912,2,111.5674614
8,1,-68.21375429,0\H,25,1.09659455,13,111.34346127,2,56.87744294,0\H,2
5,1.09766647,13,111.00360633,2,177.42713092,0\H,25,1.09965474,13,110.5
4479338,2,-63.11930859,0\C,13,1.54005828,2,110.38989466,1,168.17097079
,0\H,29,1.09801519,13,111.1596781,2,-179.33901234,0\H,29,1.09798232,13
,111.41202976,2,-58.94633039,0\H,29,1.09920664,13,110.8059927,2,60.979
56652,0\C,15,1.53908625,8,111.88455798,1,-67.40948125,0\H,33,1.0976685
5,15,110.90640832,8,177.74796533,0\H,33,1.09623461,15,111.51192333,8,5
7.06088277,0\H,33,1.09946464,15,110.51594539,8,-62.86309133,0\C,15,1.5
4025688,8,110.31230776,1,168.91389162,0\H,37,1.0979828,15,111.10055045
,8,-178.96296171,0\H,37,1.09785114,15,111.43440878,8,-58.54198523,0\H,
37,1.09915307,15,110.74771107,8,61.4176782,0\\Version=IA32L-G03RevD.01
\State=1-A\HF=-810.9272678\MP2=-812.9219058\RMSD=5.048e-09\Thermal=0.\
PG=C01 [X(C12H27P1)]\\@

105-Me$^+$

1\1\GINC-NODE-06\SP\RMP2-FC\6-31+G(2d,p)\C13H30P1(1+)\ZIP07\18-May-200
9\0\\#p MP2(FC)/6-31+g(2d,p) scf=tight\\tibpmespc003\\1,1\C\H,1,1.1009
2038\H,1,1.09851199,2,106.31209649\C,1,3.06533572,3,101.11037228,2,146
.0528357,0\H,4,1.09965638,1,80.70150795,3,119.25092189,0\H,4,1.0984485
,1,134.9971764,3,-136.86815816,0\C,1,2.93687189,4,61.63068964,6,-69.80
010511,0\H,7,1.10006945,1,73.32111918,4,177.52706873,0\H,7,1.09916051,
1,97.05220672,4,-77.53574077,0\C,4,1.55395189,1,109.17274689,7,78.0754
6344,0\H,10,1.09923255,4,107.94884088,1,-92.5538983,0\C,1,1.55295306,7
,152.66141751,4,6.34100209,0\H,12,1.09934168,1,107.94591559,7,34.22679
595,0\C,7,1.55323654,1,149.54917524,12,84.13928234,0\H,14,1.09994221,7
,107.96166229,1,25.75057396,0\C,10,1.54125512,4,108.3789071,1,151.0988
4269,0\H,16,1.09670217,10,111.70817735,4,57.83624669,0\H,16,1.09582953
,10,110.18287382,4,177.67013344,0\H,16,1.09786603,10,111.09032473,4,-6
3.02016576,0\C,10,1.53844024,4,113.44199999,1,27.79754497,0\H,20,1.098
23845,10,112.96398038,4,-62.69246173,0\H,20,1.09794815,10,111.17707201
,4,59.12560187,0\H,20,1.09571045,10,109.98775961,4,177.94188751,0\C,12
,1.5388081,1,113.55474311,7,-86.20677683,0\H,24,1.09823976,12,111.2953
2283,1,-55.45230416,0\H,24,1.09800247,12,112.76595944,1,66.49209765,0\
H,24,1.09559255,12,110.00263127,1,-174.45788137,0\C,12,1.54152463,1,10
8.1693739,24,-123.30182273,0\H,28,1.095766,12,110.21347853,1,-177.0901
1207,0\H,28,1.09675682,12,111.67129477,1,-57.24590587,0\H,28,1.0979189
2,12,111.07850523,1,63.56089835,0\C,14,1.53842597,7,113.39785214,1,-94
.55073217,0\H,32,1.09798975,14,111.15208272,7,-59.72996373,0\H,32,1.09
826972,14,112.94440581,7,61.92873582,0\H,32,1.09570752,14,110.05560033
,7,-178.60951841,0\C,14,1.54118144,7,108.47261494,1,142.04686738,0\H,3
6,1.09578214,14,110.20843672,7,-177.31880375,0\H,36,1.09669078,14,111.
66016078,7,-57.52411275,0\H,36,1.09787198,14,111.11284146,7,63.3081277
3,0\P,7,1.84160642,1,37.16974898,12,37.78110775,0\C,40,1.82478225,7,10
8.19074024,1,116.88320821,0\H,41,1.09375917,40,110.43724476,7,-177.037
85494,0\H,41,1.09583494,40,109.67371903,7,-57.15522057,0\H,41,1.094853
96,40,110.0407915,7,62.58166486,0\\Version=IA32L-G03RevD.01\State=1-A\
HF=-850.377484\MP2=-852.5198243\RMSD=1.334e-09\Thermal=0.\PG=C01 [X(C1
3H30P1)]\\@

106

1\1\GINC-NAUTILUS\SP\RMP2-FC\6-31+G(2d,p)\C10H21P1\CHRISTOPH\05-Feb-20
10\0\\#p MP2(FC)/6-31+g(2d,p) scf=tight\\oc1sp_4\\0,1\P\C,1,1.89105185
\C,1,1.86803636,2,99.89876242\H,3,1.09635842,1,110.36263111,2,68.83490
85,0\H,3,1.09705133,1,109.11452685,2,-173.40775907,0\H,3,1.09891154,1,

112.72010825,2,-52.79532402,0\C,1,1.86752017,3,98.10933529,2,102.89363
128,0\H,7,1.09717352,1,108.80307388,3,64.64393718,0\H,7,1.09568558,1,1
10.38515093,3,-177.6481567,0\H,7,1.09921819,1,112.83439072,3,-55.69757
113,0\C,2,1.55181837,1,108.92556103,7,60.75110583,0\H,11,1.09971605,2,
108.86433568,1,-71.23881266,0\H,11,1.1020929,2,108.22569727,1,43.64580
696,0\C,2,1.55774731,1,109.11850107,7,-174.35700869,0\H,14,1.09922418,
2,109.61378262,1,-97.98551581,0\H,14,1.10033023,2,109.73551037,1,16.58
204417,0\C,11,1.54146274,2,117.44787945,1,164.93553379,0\H,17,1.098517
18,11,109.53960792,2,-60.14869371,0\H,17,1.10018021,11,107.1187042,2,-
173.80600573,0\C,14,1.54478272,2,115.56050772,1,136.83148617,0\H,20,1.
10055333,14,107.49863387,2,-170.27484044,0\H,20,1.10198777,14,108.1240
4178,2,-56.5051461,0\C,17,1.53980474,11,117.43894144,2,65.27545166,0\H
,23,1.10087509,17,109.24563295,11,59.60657981,0\H,23,1.10073454,17,106
.97702971,11,173.76625879,0\C,20,1.54459931,14,118.94943036,2,67.35217
159,0\C,23,1.54678659,17,117.49819387,11,-64.81808286,0\H,2,1.10007776
,1,106.81352266,7,-56.97770832,0\H,27,1.09816262,23,110.29443539,17,80
.67074083,0\H,27,1.09918318,23,107.00055342,17,-165.30671804,0\H,26,1.
09920552,20,106.48700499,14,174.12217396,0\H,26,1.10067652,20,109.7434
3011,14,60.44219578,0\\Version=AM64L-G03RevD.01\State=1-A\HF=-731.6721
122\MP2=-733.3365213\RMSD=4.930e-09\Thermal=0.\PG=C01 [X(C10H21P1)]\\@

106-Me⁺

1\1\GINC-NAUTILUS\SP\RMP2-FC\6-31+G(2d,p)\C11H24P1(1+)\CHRISTOPH\04-Fe
b-2010\0\\#p MP2(FC)/6-31+g(2d,p) scf=tight\\oc1mesp_34\\1,1\P\C,1,1.8
4379763\C,1,1.8208016,2,110.97576103\H,3,1.09588938,1,110.02648635,2,5
6.18453413,0\H,3,1.09591463,1,109.70814818,2,175.85444706,0\H,3,1.0947
0418,1,110.47285101,2,-64.13521309,0\C,1,1.82032005,3,108.33783505,2,1
22.231803,0\H,7,1.09575759,1,109.70832976,3,59.14762597,0\H,7,1.095013
7,1,110.26477349,3,179.13345651,0\H,7,1.09505588,1,110.14035338,3,-60.
60894682,0\C,1,1.82097383,7,108.66876394,3,-116.60811239,0\H,11,1.0958
4407,1,109.77997418,7,177.51485699,0\H,11,1.095859,1,109.87660788,7,57
.84453775,0\H,11,1.09481123,1,110.66946262,7,-62.31313312,0\C,2,1.5577
1521,1,109.80451723,7,-71.50954447,0\H,15,1.09893084,2,108.98257178,1,
-67.25938418,0\H,15,1.10090682,2,109.23489331,1,49.01955586,0\C,2,1.56
353578,1,110.45831687,7,56.46568493,0\H,18,1.10052363,2,110.86326844,1
,12.02495873,0\H,18,1.09718077,2,108.84951893,1,-103.2558672,0\C,15,1.
54243593,2,115.64060263,1,169.87144934,0\H,21,1.09835306,15,106.165945
03,2,-174.34456474,0\H,21,1.09744793,15,109.536312,2,-61.14818091,0\C,
18,1.54668564,2,114.4969743,1,132.27039866,0\H,24,1.10159763,18,108.18
368935,2,-57.40860715,0\H,24,1.09840483,18,106.73000611,2,-170.9488535
2,0\C,21,1.53918934,15,117.43999365,2,65.01337189,0\H,27,1.09991579,21
,109.41443743,15,59.32349973,0\H,27,1.09872926,21,106.31233626,15,173.
05094089,0\C,24,1.54363709,18,118.77903854,2,66.90653421,0\C,27,1.5477
5785,21,117.88217833,15,-65.863107,0\H,2,1.09916589,1,103.25127228,7,1
72.4950042,0\H,31,1.10052618,27,110.75865876,21,82.5599898,0\H,31,1.09
726703,27,106.69569338,21,-164.16804186,0\H,30,1.09743818,24,105.91781
725,18,173.99365425,0\H,30,1.09961861,24,109.84476283,18,60.66010677,0
\\Version=AM64L-G03RevD.01\State=1-A\HF=-771.1249757\MP2=-772.9333759\
RMSD=8.759e-09\Thermal=0.\PG=C01 [X(C11H24P1)]\\@

107

1\1\GINC-CIPCLU08\SP\RMP2-FC\6-31+G(2d,p)\C11H23P1\C2175\15-Feb-2010\0
\\#p MP2(FC)/6-31+G(2d,p) scf=tight\\no1sp_2\\0,1\P\C,1,1.86808419\H,2
,1.09720145,1,108.8806124\H,2,1.09583071,1,110.52035031,3,-117.7465867
9,0\H,2,1.0991699,1,112.70690477,3,120.33367316,0\C,1,1.86767077,2,98.
02994592,4,177.9175727,0\H,6,1.09715542,1,108.77706199,2,68.50928798,0
\H,6,1.09885083,1,112.9748428,2,-51.99339422,0\H,6,1.09614176,1,110.34
765801,2,-173.94845527,0\C,1,1.89050874,6,100.43068395,2,102.78971162,

0\H,10,1.1002885,1,106.84784934,6,-52.16538134,0\C,10,1.5589503,1,109.
94408373,6,66.03733952,0\H,12,1.0974075,10,109.37584332,1,123.78776692
,0\H,12,1.10073034,10,110.33203532,1,9.48041299,0\C,10,1.5524055,1,109
.00354694,6,-169.07394818,0\H,15,1.1007404,10,108.78506052,1,-54.93731
911,0\H,15,1.09981134,10,108.55990246,1,60.1607758,0\C,12,1.54700045,1
0,115.35595668,1,-112.77516442,0\H,18,1.1000831,12,109.08957669,10,66.
21867566,0\H,18,1.10088846,12,107.78252671,10,-179.09285406,0\C,15,1.5
4784467,10,116.57982691,1,-177.53344099,0\H,21,1.09759081,15,109.34274
047,10,67.18856668,0\H,21,1.09868435,15,107.27890121,10,-179.41116815,
0\C,21,1.55315955,15,115.16334591,10,-57.06863947,0\H,24,1.09865023,21
,109.67072506,15,-111.52678285,0\H,24,1.09816981,21,109.67281682,15,3.
42553609,0\C,18,1.54541918,12,116.59794749,10,-56.50208991,0\H,27,1.09
865107,18,107.58553042,12,-179.63967652,0\H,27,1.09808623,18,109.16112
356,12,66.80009978,0\C,27,1.55346978,18,115.0867245,12,-57.1269928,0\C
,24,1.54597964,21,115.02450147,15,126.7609789,0\H,30,1.09838874,27,109
.71111253,18,1.54281374,0\H,30,1.09861426,27,109.62475471,18,-113.3064
1274,0\H,31,1.10064786,24,108.96361207,21,66.68044931,0\H,31,1.1007200
4,24,108.12613067,21,-178.47885354,0\\Version=AM64L-G03RevD.01\State=1
-A\HF=-770.7034048\MP2=-772.5244486\RMSD=4.827e-09\Thermal=0.\PG=C01 [
X(C11H23P1)]\\@

107-Me$^+$

1\1\GINC-CIPCLU06\SP\RMP2-FC\6-31+G(2d,p)\C12H26P1(1+)\C2175\16-Feb-20
10\0\\#p MP2(FC)/6-31+G(2d,p) scf=tight\\no1mesp_1\\1,1\P\C,1,1.820850
77\H,2,1.09595421,1,109.62809646\H,2,1.09470942,1,110.55417645,3,-119.
99706231,0\H,2,1.09567353,1,110.04121362,3,119.67839208,0\C,1,1.821602
6,2,107.43171536,4,-178.23000843,0\H,6,1.09591412,1,109.6533044,2,54.6
7563124,0\H,6,1.09587887,1,109.96084678,2,-64.93466525,0\H,6,1.0946268
9,1,110.66412939,2,174.66281502,0\C,1,1.82051537,2,108.30690179,6,116.
70001044,0\H,10,1.09578586,1,109.5968989,2,-55.56305513,0\H,10,1.09521
892,1,110.38379483,2,64.27795654,0\H,10,1.09499244,1,110.22560229,2,-1
75.35640719,0\C,1,1.83985399,10,111.44356021,6,122.16859534,0\H,14,1.0
9966545,1,103.03337199,10,-176.76279761,0\C,14,1.56341957,1,111.152769
99,10,66.8782135,0\H,16,1.09461578,14,107.71098741,1,-131.14484132,0\H
,16,1.100393,14,111.56457239,1,-17.00658301,0\C,14,1.55809671,1,110.54
654809,10,-61.75986418,0\H,19,1.09947866,14,109.50161731,1,61.88794383
,0\H,19,1.09941749,14,109.00979244,1,-54.80359312,0\C,19,1.54863513,14
,114.59667906,1,-176.25103715,0\H,22,1.09699148,19,106.24792859,14,178
.41670324,0\H,22,1.09653815,19,109.23212823,14,-68.70331442,0\C,16,1.5
4681827,14,114.67654768,1,106.5438341,0\H,25,1.09871448,16,106.9911108
6,14,177.98370006,0\H,25,1.10074885,16,109.5195886,14,-67.67794874,0\C
,22,1.55306344,19,115.42756285,14,56.22560607,0\H,28,1.09980927,22,110
.30585167,19,-2.47030804,0\H,28,1.09704532,22,109.29866478,19,112.1299
4181,0\C,25,1.54567796,16,116.30268906,14,55.93782327,0\H,31,1.0970424
9,25,106.95293366,16,178.11152944,0\H,31,1.09947556,25,109.7397928,16,
-68.89805941,0\C,28,1.54628905,22,114.9315036,19,-126.46868472,0\H,34,
1.09913452,28,108.78944131,22,-66.64586777,0\H,34,1.09916442,28,107.87
523451,22,178.79051656,0\C,34,1.54555926,28,116.6182991,22,55.83034909
,0\H,37,1.09693877,34,107.54100853,28,178.08331527,0\H,37,1.09891516,3
4,109.44117234,28,-68.68584726,0\\Version=AM64L-G03RevD.01\State=1-A\H
F=-810.1568528\MP2=-812.1219316\RMSD=2.458e-09\Thermal=0.\PG=C01 [X(C1
2H26P1)]\\@

108

1\1\GINC-NODE13\SP\RMP2-FC\6-31+G(2d,p)\C18H23P1\ZIP07\18-Apr-2010\0\\
#p MP2(FC)/6-31+G(2d,p) scf=tight\\bm33sp_53\\0,1\P\C,1,4.6727486\C,2,
1.40012893,1,61.00133127\C,3,1.39687232,2,120.18069692,1,0.47769242,0\
C,4,1.40723565,3,120.65132308,2,0.12105574,0\C,5,1.40576198,4,118.5651

6662,3,0.32804108,0\C,2,1.39721025,1,58.79404478,5,157.71640998,0\H,2,
1.08792377,1,178.88619579,5,175.50680901,0\H,3,1.08819344,2,120.002864
42,1,-179.61344532,0\H,4,1.08825342,3,119.66436944,2,179.41721639,0\H,
6,1.08867067,5,119.34108063,4,179.05164737,0\H,7,1.08798074,2,120.1622
93,1,179.64642445,0\C,1,4.6745437,5,101.87632382,4,-37.6037931,0\C,13,
1.39992668,1,58.2552938,5,-71.7168706,0\C,14,1.39541367,13,120.1104570
2,1,0.62575953,0\C,15,1.40965704,14,121.10686497,13,0.90094547,0\C,16,
1.40584161,15,118.10337849,14,-0.96553177,0\C,13,1.3963775,1,61.260847
15,16,30.08946097,0\H,13,1.08774042,1,178.19975511,16,177.56046721,0\H
,14,1.08800843,13,120.11755007,1,-178.88158823,0\H,15,1.08901953,14,11
9.44417467,13,-179.21200705,0\H,17,1.08677501,16,120.38792084,15,-179.
6751812,0\H,18,1.08819415,13,120.08645233,1,179.01553468,0\C,1,1.87644
494,16,102.32063198,15,-177.96737043,0\C,24,1.54097885,1,111.22214421,
16,177.41890338,0\C,25,1.55697039,24,116.34316667,1,179.61972826,0\C,2
6,1.54379998,25,111.06527016,24,-59.60327267,0\H,27,1.09864471,26,111.
16500948,25,-55.49910647,0\H,27,1.09823357,26,110.59726319,25,-175.127
04693,0\H,27,1.09740265,26,111.86511471,25,65.22607305,0\H,24,1.098582
11,1,106.5060341,16,-62.57289367,0\H,24,1.09836311,1,110.1726957,16,53
.98911499,0\C,26,1.54422929,25,110.92777196,24,62.57820751,0\H,33,1.09
828071,26,110.55895835,25,175.28179183,0\H,33,1.09838336,26,111.073856
85,25,55.61821086,0\H,33,1.09732191,26,111.9402214,25,-65.0286936,0\C,
26,1.54519387,25,107.52490649,24,-178.59854937,0\H,37,1.09827015,26,11
0.9710192,25,-179.49763144,0\H,37,1.09840471,26,111.12064793,25,60.534
38632,0\H,37,1.09834441,26,111.12501646,25,-59.55089251,0\H,25,1.10019
707,24,108.91544628,1,-58.1460764,0\H,25,1.10068292,24,108.63120074,1,
57.18636276,0\\Version=AM64L-G03RevD.01\State=1-A\HF=-1035.8202967\MP2
=-1038.5669839\RMSD=3.009e-09\Thermal=0.\PG=C01 [X(C18H23P1)]\\@

108-Me$^+$

1\1\GINC-NAUTILUS\SP\RMP2-FC\6-31+G(2d,p)\C19H26P1(1+)\CHRISTOPH\22-Ap
r-2010\0\\#p MP2(FC)/6-31+g(2d,p) scf=tight\\bm33mesp_33\\1,1\C\C,1,1.
39967636\C,2,1.39552017,1,120.14351721\C,3,1.41019342,2,119.82336511,1
,-0.008171,0\C,4,1.40784126,3,119.8059869,2,0.04448168,0\C,5,1.3968629
8,4,119.86626134,3,-0.00142257,0\H,1,1.08683933,6,119.88223314,5,179.8
8643155,0\H,2,1.08661052,1,120.20629129,6,179.69611562,0\H,3,1.0872441
7,2,119.63348411,1,179.17201174,0\H,5,1.08663729,4,121.05721764,3,179.
66226356,0\H,6,1.0865434,5,119.61314339,4,179.70081257,0\C,3,5.4770107
8,2,141.38978438,1,122.4593862,0\C,12,1.39874061,3,81.1787877,2,161.20
131257,0\C,13,1.39638418,12,120.12534175,3,22.34861493,0\C,14,1.408344
42,13,119.82525332,12,-0.18348619,0\C,15,1.40871413,14,119.86420764,13
,0.32828115,0\C,16,1.39600747,15,119.81029463,14,-0.23939584,0\H,12,1.
08684673,3,150.26002417,2,23.09673164,0\H,13,1.08658436,12,120.2322368
2,3,-157.47817518,0\H,14,1.08710774,13,119.12387,12,-179.9124856,0\H,1
6,1.0872863,15,120.53500642,14,-179.71005477,0\H,17,1.08661677,16,119.
63770076,15,-179.84481043,0\C,4,2.99614589,3,108.06408363,2,-140.99662
174,0\C,23,1.54430085,4,94.80019928,3,12.71806045,0\C,24,1.55896451,23
,114.79885864,4,154.135704,0\C,25,1.54378124,24,110.92480784,23,-58.94
724857,0\H,26,1.09818347,25,112.34363114,24,66.61150846,0\H,26,1.09838
95,25,111.33819248,24,-54.98382234,0\H,26,1.09657918,25,110.24260508,2
4,-174.26284776,0\H,23,1.09845962,4,88.32215252,3,123.65559832,0\H,23,
1.09822753,4,141.10709639,3,-121.49191679,0\C,25,1.54524762,24,106.884
02098,23,-177.78630546,0\H,32,1.09807329,25,111.33861589,24,-59.628406
23,0\H,32,1.09666512,25,110.56025171,24,-179.31567561,0\H,32,1.0978961
4,25,111.29556235,24,61.02593579,0\C,25,1.54397867,24,110.87999428,23,
63.55372988,0\H,36,1.09881789,25,111.32149956,24,56.25836224,0\H,36,1.
0985013,25,112.42865881,24,-65.50409368,0\H,36,1.09657325,25,110.26920
976,24,175.42163754,0\H,24,1.09939823,23,109.4394868,4,-83.79038059,0\
H,24,1.09837689,23,108.60648222,4,32.54068447,0\P,4,1.80716068,3,119.5

7314692,2,-177.23986607,0\C,42,1.82527116,4,109.49300065,3,-163.529979
95,0\H,43,1.09554486,42,108.45275248,4,175.31180288,0\H,43,1.09525994,
42,111.05055625,4,-65.1604244,0\H,43,1.09489799,42,110.11638452,4,56.5
0331319,0\\Version=AM64L-G03RevD.01\State=1-A\HF=-1075.2723977\MP2=-10
78.1648567\RMSD=4.657e-09\Thermal=0.\PG=C01 [X(C19H26P1)]\\@

109

1\1\GINC-YANG\SP\RMP2-FC\6-31+G(2d,p)\C9H21P1\CHRISTOPH\23-Sep-2009\0\
\#p MP2(FC)/6-31+g(2d,p) scf=tight\\bu2sp_2\\0,1\P\C,1,1.87589008\H,2,
1.10189401,1,110.12780396\H,2,1.1006994,1,106.43166133,3,115.2015159,0
\C,1,1.86714491,2,98.46895356,4,60.93650616,0\H,5,1.09636543,1,110.026
60173,2,173.21389301,0\H,5,1.09905039,1,112.71258267,2,51.76017022,0\C
,1,1.87564596,5,99.92528272,2,-101.63310905,0\H,8,1.10212733,1,110.120
98739,5,49.26194356,0\H,8,1.1000746,1,106.96218065,5,164.94186359,0\C,
2,1.53963709,1,113.92477033,5,-178.68688919,0\H,11,1.10058275,2,109.89
8998,1,62.10058663,0\H,11,1.10024579,2,109.41833375,1,-54.17268206,0\C
,8,1.54010835,1,113.45766155,5,-74.85006944,0\H,14,1.10037224,8,109.38
060676,1,-54.37773445,0\H,14,1.10057315,8,109.85341689,1,61.85204688,0
\C,14,1.53908891,8,112.95604022,1,-176.35485907,0\H,17,1.1004386,14,10
9.12352663,8,58.04202924,0\H,17,1.10048517,14,109.2527426,8,-57.661886
36,0\C,11,1.53911462,2,112.82902003,1,-176.16941138,0\H,20,1.10042922,
11,109.09607956,2,58.20355531,0\H,20,1.10048659,11,109.22584374,2,-57.
45457801,0\C,20,1.53561491,11,113.02382347,2,-179.66365976,0\H,23,1.09
807824,20,111.12623613,11,-59.86893356,0\H,23,1.09816335,20,111.105760
99,11,59.88515373,0\H,23,1.09716436,20,111.35654147,11,179.99598629,0\
C,17,1.5356621,14,112.97913871,8,-179.86675176,0\H,27,1.09815259,17,11
1.12101347,14,60.02713623,0\H,27,1.09714897,17,111.36735498,14,-179.85
348616,0\H,27,1.09808658,17,111.09976204,14,-59.71911329,0\H,5,1.09696
86,1,109.30897773,8,-170.69328765,0\\Version=AM64L-G03RevD.01\State=1-
A\HF=-693.819055\MP2=-695.3386413\RMSD=9.059e-09\Thermal=0.\PG=C01 [X(
C9H21P1)]\\@

109-Me$^+$

1\1\GINC-NODE26\SP\RMP2-FC\6-31+G(2d,p)\C10H24P1(1+)\ZIP07\23-Sep-2009
\0\\#p MP2(FC)/6-31+g(2d,p) scf=tight\\bu2mesp_1\\1,1\P\C,1,1.82057953
\H,2,1.09537271,1,110.32815977\H,2,1.09583596,1,109.76978512,3,119.835
57763,0\H,2,1.09581313,1,110.16114705,4,119.86796495,0\C,1,1.82058062,
2,108.26939868,3,-179.15556176,0\H,6,1.09583497,1,109.7697174,2,-59.32
14913,0\H,6,1.09581244,1,110.16116174,2,60.54653765,0\H,6,1.09537377,1
,110.32809284,2,-179.15698874,0\C,1,1.82842572,2,108.70202649,6,119.37
543412,0\H,10,1.09972308,1,106.56236433,2,-57.74484635,0\H,10,1.099224
91,1,106.95050672,2,55.96770749,0\C,1,1.82842557,2,109.91886261,6,-118
.60565923,0\H,13,1.09922527,1,106.95060515,2,174.30597107,0\H,13,1.099
72315,1,106.56218308,2,60.59353103,0\C,13,1.54599195,1,115.09044938,2,
-62.42717235,0\H,16,1.09904021,13,110.05291517,1,57.78355945,0\H,16,1.
09926803,13,109.90727076,1,-59.71847721,0\C,10,1.54599181,1,115.090368
27,2,179.23438449,0\H,19,1.09926877,10,109.90730875,1,-59.71786169,0\H
,19,1.09904018,10,110.05294088,1,57.78426943,0\C,16,1.54069042,13,111.
36747834,1,179.04024961,0\H,22,1.09946395,16,109.13536759,13,57.998603
71,0\H,22,1.09944825,16,109.11306542,13,-58.03341457,0\C,19,1.54069063
,10,111.36747738,1,179.04090835,0\H,25,1.09944883,19,109.11307117,10,-
58.0333602,0\H,25,1.099464,19,109.13544579,10,57.99867631,0\C,22,1.535
02808,16,112.36841823,13,179.99301358,0\H,28,1.09726113,22,111.3492262
6,16,60.25571653,0\H,28,1.09535341,22,110.60039071,16,179.96222482,0\H
,28,1.09731932,22,111.35323039,16,-60.33096416,0\C,25,1.53502883,19,11
2.36841873,10,179.99312785,0\H,32,1.09731974,25,111.35317798,19,-60.33
106549,0\H,32,1.09726099,25,111.34925661,19,60.25562382,0\H,32,1.09535
276,25,110.60042739,19,179.9621455,0\\Version=AM64L-G03RevD.01\State=1

-A\HF=-733.2719513\MP2=-734.9365454\RMSD=1.818e-09\Thermal=0.\PG=C01 [X(C10H24P1)]\\@

110

1\1\GINC-NODE18\SP\RMP2-FC\6-31+G(2d,p)\C9H17P1\ZIP07\04-Sep-2009\0\\# p MP2(FC)/6-31+g(2d,p) scf=tight\\bu2sp_1\\0,1\P\C,1,1.86602712\C,2,1. 56403638,1,118.07404451\C,3,1.55536739,2,88.61868959,1,-140.00308336,0 \C,4,1.55481928,3,88.56350715,2,18.70276041,0\H,2,1.10060186,1,110.495 50944,3,127.74046162,0\H,3,1.09601793,2,117.33837841,1,98.89033457,0\H ,3,1.09768997,2,111.49579127,1,-27.39379563,0\H,4,1.09666467,3,111.683 67428,2,-94.1502062,0\H,4,1.09618434,3,117.56712539,2,139.35479874,0\H ,5,1.09734759,4,111.5518897,3,93.88436685,0\H,5,1.09602045,4,118.03119 866,3,-139.21596091,0\C,1,1.86602407,2,100.22677981,5,78.99372445,0\C, 13,1.56403757,1,118.07326212,2,176.94393386,0\C,14,1.55536588,13,88.61 87411,1,140.00407836,0\C,15,1.55481718,14,88.56336425,13,-18.70315013, 0\H,13,1.10060042,1,110.49690449,2,49.20393632,0\H,14,1.09769145,13,11 1.49421896,1,27.39676788,0\H,14,1.09601825,13,117.33990551,1,-98.88718 436,0\H,15,1.09666419,14,111.68303742,13,94.14879575,0\H,15,1.09618563 ,14,117.56771253,13,-139.35607423,0\H,16,1.09734816,15,111.55032319,14 ,-93.88199011,0\H,16,1.09602083,15,118.03267995,14,139.21838472,0\C,1, 1.86780091,13,99.5012089,16,179.45009799,0\H,24,1.09883398,1,112.88972 936,13,51.08071285,0\H,24,1.09688546,1,109.51841333,13,172.20071579,0\ H,24,1.09688472,1,109.52000987,13,-70.04053361,0\\Version=AM64L-G03Rev D.01\State=1-A\HF=-691.4129826\MP2=-692.8950668\RMSD=3.209e-09\Thermal =0.\PG=C01 [X(C9H17P1)]\\@

110-Me$^+$

1\1\GINC-NODE26\SP\RMP2-FC\6-31+G(2d,p)\C10H20P1(1+)\ZIP07\04-Sep-2009 \0\\#p MP2(FC)/6-31+g(2d,p) scf=tight\\bu2mesp_1\\1,1\P\C,1,1.82474691 \C,2,1.56629082,1,120.13536426\C,3,1.55669049,2,87.76590705,1,143.1303 2328,0\C,4,1.55647495,3,89.52652347,2,-18.64987982,0\H,2,1.09875842,1, 106.19300228,5,126.01603512,0\H,3,1.0936408,2,116.45144804,1,-96.28822 693,0\H,3,1.09637641,2,112.72290537,1,30.61618089,0\H,4,1.09452184,3,1 11.68640712,2,94.5610498,0\H,4,1.09304779,3,116.97201916,2,-138.945261 7,0\H,5,1.09367504,4,118.1926558,3,137.66522869,0\H,5,1.09642753,4,111 .58208166,3,-94.92597571,0\C,1,1.82474865,2,109.93933457,3,67.20588756 ,0\C,13,1.56668558,1,120.09365696,2,175.22185854,0\C,14,1.55647691,13, 87.75830571,1,-143.17476946,0\C,15,1.55669059,14,89.52541733,13,18.651 01867,0\H,13,1.09875786,1,106.19334202,2,-58.76460472,0\H,14,1.0936753 5,13,116.46951625,1,96.24880704,0\H,14,1.09643011,13,112.70238758,1,-3 0.67727765,0\H,15,1.094522,14,111.6696187,13,-94.57406912,0\H,15,1.093 04725,14,116.95764112,13,138.95984964,0\H,16,1.09364082,15,118.1984643 3,14,-137.66194579,0\H,16,1.09637472,15,111.59044584,14,94.93990817,0\ C,1,1.82097246,2,108.62701547,3,-171.29753207,0\H,24,1.09533831,1,110. 71599441,2,178.36064585,0\H,24,1.09513573,1,110.3426786,2,-60.81406523 ,0\H,24,1.09575772,1,109.2579453,2,58.5913079,0\C,1,1.82097299,24,107. 86618547,2,-120.25834028,0\H,28,1.09533724,1,110.71566296,24,58.074966 77,0\H,28,1.09513535,1,110.34305439,24,178.90042407,0\H,28,1.09575791, 1,109.25746548,24,-61.6942011,0\\Version=AM64L-G03RevD.01\State=1-A\HF =-730.8665802\MP2=-732.4931563\RMSD=2.262e-09\Thermal=0.\PG=C01 [X(C10 H20P1)]\\@

111

1\1\GINC-CIPCLU09\SP\RMP2-FC\6-31+G(2d,p)\C12H25P1\C2175\17-Feb-2010\0 \\#p MP2(FC)/6-31+G(2d,p) scf=tight\\dec1sp_1\\0,1\P\C,1,1.86775449\H, 2,1.09583055,1,110.50011362\H,2,1.09906116,1,112.9503708,3,122.1379621 1,0\H,2,1.09718172,1,108.63942048,3,-117.52869769,0\C,1,1.86823799,2,9 7.93837928,3,-178.75087607,0\H,6,1.09916549,1,112.71951348,2,55.780242

09,0\H,6,1.09711746,1,108.76986523,2,-64.58544318,0\H,6,1.0956329,1,11
0.57561448,2,177.79736579,0\C,1,1.89559295,2,100.68355205,6,101.820170
61,0\H,10,1.10404146,1,106.93688036,2,-54.65779986,0\C,10,1.55401226,1
,108.78368535,2,-170.74257571,0\H,12,1.09915684,10,108.58418225,1,57.7
0864882,0\H,12,1.09457645,10,108.62004652,1,-56.21130348,0\C,10,1.5568
8764,1,111.33592701,2,60.330001,0\H,15,1.09930643,10,106.99517075,1,-6
3.96660958,0\H,15,1.09836925,10,108.38474445,1,-175.89264838,0\C,15,1.
54979582,10,120.16500911,1,57.75793396,0\H,18,1.09956487,15,109.339548
97,10,-58.44389395,0\H,18,1.10134128,15,106.54454254,10,-172.48570555,
0\C,12,1.54827477,10,115.71789808,1,179.43698536,0\H,21,1.09997919,12,
108.9907035,10,-85.81922284,0\H,21,1.09368064,12,110.49544382,10,29.32
849783,0\C,18,1.54651597,15,118.00269831,10,65.42978493,0\H,24,1.09450
969,18,108.87332269,15,-66.97214925,0\H,24,1.09985217,18,107.91494426,
15,179.46792122,0\C,21,1.54796333,12,114.70346671,10,152.86711528,0\H,
27,1.10109658,21,108.06465064,12,-179.68239135,0\H,27,1.10114566,21,10
8.41879751,12,66.07299895,0\C,24,1.54715026,18,115.0318195,15,57.52771
289,0\H,30,1.09488017,24,110.102209,18,-27.60982838,0\C,27,1.54743889,
21,118.01882521,12,-55.84411766,0\H,32,1.10033853,27,106.59008762,21,1
73.65391965,0\H,32,1.09861913,27,109.63627638,21,60.77681313,0\C,30,1.
54731934,24,115.34214052,18,-151.31210803,0\H,35,1.10116314,30,108.122
06228,24,179.31277192,0\H,35,1.10108499,30,108.4265911,24,-66.42689669
,0\H,30,1.1000691,24,108.99690017,18,87.11798332,0\\Version=AM64L-G03R
evD.01\State=1-A\HF=-809.7387975\MP2=-811.7146721\RMSD=4.863e-09\Therm
al=0.\PG=C01 [X(C12H25P1)]\\@

111-Me⁺

1\1\GINC-CIPCLU02\SP\RMP2-FC\6-31+G(2d,p)\C13H28P1(1+)\C2175\18-Feb-20
10\0\\#p MP2(FC)/6-31+G(2d,p) scf=tight\\dec1mesp_2\\1,1\P\C,1,1.82285
145\H,2,1.09588483,1,109.63737521\H,2,1.09437458,1,110.83559725,3,120.
03994283,0\H,2,1.09592353,1,109.85886613,3,-119.51237063,0\C,1,1.82148
163,2,106.88792513,4,174.74896523,0\H,6,1.09619036,1,109.13210071,2,-5
3.03698138,0\H,6,1.09421966,1,110.53255886,2,-172.6563196,0\H,6,1.0952
8631,1,110.37987597,2,66.62847927,0\C,1,1.82141499,6,107.60815646,2,11
6.0913561,0\H,10,1.09547896,1,110.30437249,6,67.00388993,0\H,10,1.0958
7299,1,109.45151763,6,-52.62553822,0\H,10,1.09461757,1,110.45425745,6,
-172.45120973,0\C,1,1.8477972,10,111.44631029,6,-123.65128517,0\H,14,1
.09635111,1,103.09712687,10,-171.70560753,0\C,14,1.56324339,1,108.6783
8654,10,-56.84254145,0\H,16,1.09987023,14,109.11913969,1,-53.12330942,
0\H,16,1.09971925,14,108.57017241,1,62.78718945,0\C,14,1.55939916,1,11
2.66181179,10,70.81408219,0\H,19,1.09248187,14,106.55612599,1,-150.670
45168,0\H,19,1.10100454,14,110.36848771,1,-37.71000091,0\C,19,1.552705
33,14,117.30771667,1,87.07508087,0\H,22,1.09909831,19,106.46432986,14,
170.60243004,0\H,22,1.09880547,19,109.24706458,14,-76.37067984,0\C,16,
1.55021126,14,117.31244895,1,-175.85691386,0\H,25,1.09675024,16,109.84
947608,14,-61.85027611,0\H,25,1.09868939,16,105.0698668,14,-174.047320
36,0\C,22,1.54793365,19,119.18356159,14,47.36685541,0\H,28,1.09872865,
22,106.0902928,19,-168.98787999,0\H,28,1.10095271,22,110.24204092,19,-
56.40651566,0\C,25,1.54533284,16,118.60319704,14,66.29962514,0\H,31,1.
10032926,25,108.9622252,16,56.56658715,0\H,31,1.09917931,25,106.410724
97,16,170.09203081,0\C,28,1.54639739,22,118.30527277,19,71.33413057,0\
H,34,1.09953151,28,108.39807008,22,59.19899548,0\C,31,1.54592633,25,11
7.99621142,16,-67.94720238,0\H,36,1.09795106,31,109.47922194,25,70.605
4094,0\H,36,1.09837627,31,107.82615233,25,-176.33240369,0\C,34,1.54392
174,28,117.61648062,22,-64.04272942,0\H,39,1.09823744,34,108.15086247,
28,179.59397563,0\H,39,1.09640711,34,108.8991292,28,66.17719952,0\H,34
,1.0995521,28,107.13980448,22,173.05740812,0\\Version=AM64L-G03RevD.01
\State=1-A\HF=-849.1918016\MP2=-851.312573\RMSD=2.390e-09\Thermal=0.\P
G=C01 [X(C13H28P1)]\\@

112

1\1\GINC-CIPCLU03\SP\RMP2-FC\6-31+G(2d,p)\C9H21P1\C2175\10-Dec-2009\0\
\#p MP2(FC)/6-31+G(2d,p) scf=tight\\aib2sp_4\\0,1\P\C,1,1.87083579\H,2
,1.09587888,1,109.44688294\C,1,1.89179469,2,100.30236225,3,-75.6942630
7,0\H,4,1.10182998,1,109.22901471,2,-44.68108493,0\C,1,1.89698477,2,10
1.29723186,4,-106.04263258,0\H,6,1.10168852,1,103.72262243,2,170.44236
745,0\C,6,1.54437477,1,108.29403859,2,-76.0528608,0\H,8,1.09676662,6,1
11.16546704,1,-48.70715032,0\H,8,1.09858912,6,111.36479589,1,71.528515
37,0\H,8,1.09707037,6,111.27131447,1,-168.73528352,0\C,6,1.54520627,1,
116.53271458,2,51.83071501,0\H,12,1.09977818,6,109.35004534,1,-67.3803
6819,0\H,12,1.09872173,6,109.57923559,1,48.7389226,0\C,4,1.55003794,1,
108.81794413,2,72.92258763,0\H,15,1.10001271,4,108.93981468,1,64.17306
555,0\H,15,1.09891054,4,109.4551149,1,-51.47577231,0\C,4,1.54119172,1,
110.17760207,2,-164.20009023,0\H,18,1.09871364,4,110.91096268,1,-57.05
88006,0\H,18,1.09641589,4,111.81955221,1,63.34367691,0\H,18,1.09692598
,4,111.28301442,1,-176.99502922,0\C,12,1.53970622,6,114.03396263,1,170
.19494306,0\H,22,1.09859646,12,111.06923288,6,-56.26062835,0\H,22,1.09
646148,12,112.05769119,6,64.28722908,0\H,22,1.09747837,12,110.70396939
,6,-175.78739485,0\C,15,1.53869192,4,114.2406705,1,-172.83215812,0\H,2
6,1.09837391,15,111.0813501,4,57.0451725,0\H,26,1.09731094,15,110.5694
7832,4,176.55245218,0\H,26,1.09634047,15,112.13907806,4,-63.63838539,0
\H,2,1.09774125,1,113.72028278,4,45.78922035,0\H,2,1.09670588,1,109.34
808304,4,167.36867341,0\\Version=AM64L-G03RevD.01\State=1-A\HF=-693.80
58919\MP2=-695.3392578\RMSD=3.767e-09\Thermal=0.\PG=C01 [X(C9H21P1)]\\
@

112-Me$^+$

1\1\GINC-EDDY\SP\RMP2-FC\6-31+G(2d,p)\C10H24P1(1+)\CHRISTOPH\07-Dec-20
09\0\\#p MP2(FC)/6-31+g(2d,p) scf=tight\\aib2mesp_1\\1,1\C\H,1,1.10075
22\C,1,3.06270544,2,89.41656822\H,3,1.10088995,1,86.64495791,2,-121.26
065056,0\C,1,2.99786963,3,59.34250437,4,86.71496991,0\H,5,1.09495221,1
,88.56649769,3,162.14681776,0\C,1,1.55524833,5,89.65165464,3,-163.4940
0595,0\H,7,1.0988414,1,109.28642119,5,-42.65142619,0\H,7,1.09852369,1,
110.10219998,5,74.43678146,0\C,1,1.54525071,7,112.81557364,5,97.063664
48,0\H,10,1.09541108,1,112.62556388,7,175.6622455,0\H,10,1.09697478,1,
111.6834134,7,-61.81505135,0\H,10,1.09418398,1,109.29690037,7,56.88530
249,0\C,3,1.55404376,1,89.95965008,10,96.3394985,0\H,14,1.09921306,3,1
09.34237654,1,94.58323095,0\H,14,1.09835213,3,109.77038302,1,-22.27357
747,0\C,3,1.5455053,1,145.28400678,10,-130.37084549,0\H,17,1.09660315,
3,111.72409214,1,-70.38343138,0\H,17,1.09537296,3,112.67421842,1,52.34
782862,0\H,17,1.09419334,3,109.20427405,1,170.86782822,0\C,7,1.5385034
9,1,112.82952775,10,-67.6155991,0\H,21,1.09530449,7,109.7134808,1,-176
.51793299,0\H,21,1.09699532,7,111.28027922,1,-57.35318963,0\H,21,1.095
0319,7,112.23375144,1,64.21702449,0\C,14,1.53840272,3,112.97869263,1,-
143.29796023,0\H,25,1.09718584,14,111.27487093,3,55.96226856,0\H,25,1.
09522382,14,109.71685449,3,175.12061161,0\H,25,1.09490142,14,112.19260
157,3,-65.56982347,0\P,5,1.82357242,1,35.70354472,10,117.62968235,0\C,
29,1.8238414,5,107.82998657,1,118.8223274,0\H,30,1.09524797,29,110.250
09505,5,-176.85967801,0\H,30,1.0951657,29,109.89578939,5,63.0578127,0\
H,30,1.09449152,29,110.34896312,5,-56.56713307,0\H,5,1.09454471,1,145.
87746276,10,123.58409479,0\H,5,1.09542086,1,92.03704146,10,-5.37258065
,0\\Version=AM64L-G03RevD.01\State=1-A\HF=-733.2611297\MP2=-734.938795
7\RMSD=5.931e-09\Thermal=0.\PG=C01 [X(C10H24P1)]\\@

113

1\1\GINC-NODE15\SP\RMP2-FC\6-31+G(2d,p)\C18H23P1\ZIP07\08-May-2010\0\\
#p MP2(FC)/6-31+G(2d,p) scf=tight\\bm11sp_17\\0,1\P\C,1,4.6793262\C,2,

1.39831277,1,57.80959857\C,3,1.39700813,2,120.05845195,1,0.10571738,0\
C,4,1.41071459,3,121.28299596,2,1.46580728,0\C,5,1.40689419,4,117.8291
21,3,-2.17975772,0\C,2,1.3976017,1,61.61929864,5,19.47872935,0\H,2,1.0
8778723,1,177.9682742,5,178.45827302,0\H,3,1.08804934,2,120.18608594,1
,-178.80364042,0\H,4,1.08842952,3,119.28677904,2,-177.44387281,0\H,6,1
.08585575,5,120.0816222,4,-177.54060097,0\H,7,1.08824001,2,120.0425189
4,1,179.01948584,0\C,1,4.68095658,5,107.48269646,4,161.16774987,0\C,13
,1.39874957,1,62.36492999,5,60.03237036,0\C,14,1.39806838,13,120.31325
98,1,-0.42782618,0\C,15,1.41034448,14,121.07989846,13,0.06430262,0\C,1
6,1.40904128,15,117.61521597,14,-0.02779183,0\C,13,1.39679444,1,57.185
95232,5,-119.4814069,0\H,13,1.08783417,1,177.43354339,5,-128.90476325,
0\H,14,1.0881228,13,120.06024831,1,179.62459148,0\H,15,1.08533868,14,1
18.67055096,13,-179.47137966,0\H,17,1.08829435,16,118.96005614,15,179.
92748849,0\H,18,1.08790234,13,120.20246597,1,-179.57064667,0\C,1,1.929
45803,5,103.49844044,4,-84.87210419,0\C,24,1.55637617,1,108.66356674,5
,-164.87597081,0\H,25,1.10341365,24,106.72876335,1,179.40265884,0\H,25
,1.10007,24,108.64762853,1,65.63386365,0\C,25,1.53959277,24,117.921503
53,1,-58.56184518,0\H,28,1.09844591,25,110.10611899,24,59.0139403,0\H,
28,1.09867583,25,110.0484666,24,-57.75787776,0\C,28,1.53658607,25,111.
78059469,24,-179.28494828,0\H,31,1.09845899,28,111.19686638,25,59.3937
0941,0\H,31,1.09715957,28,111.24134182,25,179.48296053,0\H,31,1.098065
31,28,111.11561073,25,-60.4489077,0\C,24,1.5475615,1,105.93549725,5,76
.33796643,0\H,35,1.09992108,24,109.58715992,1,171.33315711,0\H,35,1.09
690135,24,111.13580779,1,-70.12810948,0\H,35,1.09610934,24,112.4858490
6,1,51.10425279,0\C,24,1.5433727,1,115.42038342,5,-43.01545143,0\H,39,
1.10049826,24,109.4921588,1,170.31628865,0\H,39,1.09532219,24,112.0538
2283,1,-71.26734328,0\H,39,1.09622039,24,111.88119367,1,50.94377351,0\
\Version=AM64L-G03RevD.01\State=1-A\HF=-1035.8048097\MP2=-1038.5605948
\RMSD=5.786e-09\Thermal=0.\PG=C01 [X(C18H23P1)]\\@

113-Me$^+$

1\1\GINC-NODE26\SP\RMP2-FC\6-31+G(2d,p)\C19H26P1(1+)\ZIP07\12-May-2010
\0\\#p MP2(FC)/6-31+G(2d,p) scf=tight\\bm11mesp_4\\1,1\P\C,1,1.829336\
H,2,1.09421251,1,111.79799854\H,2,1.09448277,1,108.88650077,3,120.4853
6646,0\H,2,1.09488519,1,109.13412368,4,118.47117296,0\C,1,1.88520915,2
,108.73028317,3,-51.20947277,0\C,6,1.55131986,1,106.74721296,2,69.3588
2835,0\H,7,1.09540765,6,108.51323439,1,-179.30753144,0\H,7,1.09685086,
6,112.68282079,1,-60.83282994,0\H,7,1.09478495,6,112.03895515,1,62.221
72338,0\C,6,1.54770067,1,108.91053707,2,-173.58738476,0\H,11,1.0953944
5,6,108.91231834,1,-172.73786502,0\H,11,1.09387998,6,113.25001928,1,67
.33540654,0\H,11,1.09626325,6,111.18763521,1,-55.01496204,0\C,6,1.5644
0182,1,108.68603019,2,-49.27756462,0\H,15,1.09840703,6,110.93614167,1,
-47.20532886,0\H,15,1.09945051,6,108.23157945,1,68.74688425,0\C,15,1.5
4385637,6,115.17546869,1,-169.95444887,0\H,18,1.09790874,15,110.344163
52,6,-53.25304183,0\H,18,1.09776977,15,109.8809596,6,64.12965371,0\C,1
8,1.53676788,15,111.33711932,6,-174.65781114,0\H,21,1.0975852,18,111.3
6387774,15,-59.33831407,0\H,21,1.09726903,18,111.32092304,15,61.110266
84,0\H,21,1.09548544,18,110.64489275,15,-179.0888562,0\C,1,4.62438844,
2,103.82473588,6,-126.70926961,0\C,25,1.39904359,1,58.53339718,2,-71.7
0746916,0\C,26,1.39507776,25,120.10128854,1,-1.45960612,0\C,27,1.41145
344,26,120.24176606,25,0.17666289,0\C,28,1.40730399,27,119.2900924,26,
0.02915466,0\C,25,1.39726292,1,61.47860452,28,-36.30887241,0\H,25,1.08
680776,1,178.17146769,28,179.17079942,0\H,26,1.08664099,25,120.2952288
5,1,178.78260319,0\H,27,1.08766466,26,119.2383008,25,-179.044705,0\H,2
9,1.08411918,28,121.29701105,27,179.84890248,0\H,30,1.08662533,25,120.
23162091,1,-178.84502941,0\C,1,4.61505762,28,107.9456165,27,47.1446756
4,0\C,36,1.39911015,1,59.86590763,28,39.10432329,0\C,37,1.39606846,36,
120.13633659,1,-1.03405077,0\C,38,1.40951121,37,119.938029,36,0.167382

39,0\C,39,1.40818442,38,119.66104098,37,-0.15148623,0\C,40,1.39670012,
39,119.94932117,38,-0.04927219,0\H,36,1.08684064,1,179.31346521,39,178
.34054136,0\H,37,1.08664154,36,120.21547347,1,179.35071418,0\H,38,1.08
720769,37,119.58695882,36,-178.65023266,0\H,40,1.08675376,39,120.93987
404,38,-179.82730867,0\H,41,1.0866193,40,119.62651583,39,-179.5963715,
0\\Version=AM64L-G03RevD.01\State=1-A\HF=-1075.259132\MP2=-1078.160090
3\RMSD=6.124e-09\Thermal=0.\PG=C01 [X(C19H26P1)]\\@

114

1\1\GINC-EDDY\SP\RMP2-FC\6-31+G(2d,p)\C11H25P1\CHRISTOPH\03-Dec-2009\0
\\#p MP2(FC)/6-31+g(2d,p) scf=tight\\ip2sp_1\\0,1\P\C,1,1.87021802\H,2
,1.09570278,1,109.54897674\C,1,1.899924,2,100.73161645,3,-176.90626353
,0\H,4,1.10191028,1,104.42201512,2,-163.47331286,0\C,1,1.89821864,2,99
.81908742,4,-105.39519635,0\H,6,1.10259886,1,108.29085116,2,44.0822598
9,0\C,4,1.54825931,1,115.76105681,2,-44.92351817,0\H,8,1.09849497,4,11
0.1134765,1,-38.10613623,0\H,8,1.09980759,4,109.13419335,1,77.98358517
,0\C,4,1.55270195,1,109.19185427,2,82.35621757,0\H,11,1.10107052,4,109
.62670725,1,-53.13777789,0\H,11,1.09971323,4,108.69291052,1,-168.07811
098,0\C,6,1.55039115,1,112.4181512,2,162.18561019,0\H,14,1.0994566,6,1
08.26462327,1,-175.62746026,0\H,14,1.09877263,6,108.98993496,1,-61.698
35139,0\C,6,1.55110443,1,108.46079172,2,-71.96179785,0\H,17,1.09887917
,6,109.67281755,1,-60.4215164,0\H,17,1.09871292,6,108.8707759,1,54.974
75902,0\C,8,1.5391904,4,113.68818048,1,-159.54925532,0\H,20,1.09699354
,8,112.12232626,4,-59.20165708,0\H,20,1.09831136,8,110.86043555,4,61.1
0220829,0\H,20,1.09755643,8,110.88167728,4,-179.32722474,0\C,11,1.5362
5112,4,114.35953682,1,70.35829754,0\H,24,1.09614773,11,111.40872049,4,
-59.47352377,0\H,24,1.09735256,11,110.87667643,4,-179.89392435,0\H,24,
1.09846184,11,110.8023398,4,60.55938819,0\C,14,1.53809023,6,115.831635
54,1,61.76550066,0\H,28,1.09725563,14,110.38384385,6,-179.15574468,0\H
,28,1.09694607,14,111.72901035,6,-59.03784472,0\H,28,1.09714606,14,111
.67014864,6,61.29291556,0\C,17,1.53860607,6,114.34195519,1,176.0732504
4,0\H,32,1.09733975,17,110.52490511,6,-174.53411672,0\H,32,1.09848382,
17,111.17997407,6,-55.00829323,0\H,32,1.09672369,17,112.12825942,6,65.
67965186,0\H,2,1.09701208,1,109.18468141,6,-165.37398943,0\H,2,1.09776
402,1,113.73352722,6,-44.047444,0\\Version=AM64L-G03RevD.01\State=1-A\
HF=-771.8769358\MP2=-773.7213549\RMSD=4.847e-09\Thermal=0.\PG=C01 [X(C
11H25P1)]\\@

114-Me$^+$

1\1\GINC-CIPCLU10\SP\RMP2-FC\6-31+G(2d,p)\C12H28P1(1+)\C2175\17-Dec-20
09\0\\#p mp2(FC)/6-31+G(2d,p) scf=tight\\ip2mesp_3\\1,1\C\H,1,1.101856
35\C,1,3.07014319,2,94.04361415\H,3,1.10011584,1,82.53350747,2,-122.11
561158,0\C,1,2.97826654,3,59.10466726,4,167.17816093,0\H,5,1.09549915,
1,146.51541456,3,-32.95775119,0\C,1,1.55420905,5,144.80424645,3,-4.447
53786,0\H,7,1.09766724,1,109.14256488,5,-34.6621057,0\H,7,1.09677021,1
,106.34573377,5,-147.64200146,0\C,1,1.55493418,7,114.56751747,3,-148.1
8616832,0\H,10,1.09742559,1,109.87788554,7,59.66483351,0\H,10,1.099504
71,1,109.59203365,7,176.30445692,0\C,3,1.55800191,1,92.97752897,7,92.5
9036523,0\H,13,1.09952574,3,110.98208947,1,-6.70966847,0\H,13,1.098533
72,3,108.79403535,1,110.16421888,0\C,3,1.5600688,1,145.77663454,7,-127
.48652709,0\H,16,1.09667534,3,107.13662714,1,-157.43751194,0\H,16,1.10
012551,3,110.92718529,1,-43.37943536,0\C,13,1.53776186,3,112.24416342,
1,-127.94329274,0\H,19,1.09533202,13,110.14231826,3,-178.14473349,0\H,
19,1.09599373,13,112.0874531,3,-58.48928939,0\H,19,1.09710989,13,111.0
6649084,3,62.42630959,0\C,16,1.536226,3,114.2943281,1,82.11659414,0\H,
23,1.096881,16,110.69446183,3,59.462111,0\H,23,1.09548323,16,110.39718
487,3,178.55296877,0\H,23,1.09666106,16,112.70519887,3,-61.26198725,0\
C,7,1.53640864,1,116.79154602,10,-61.5734883,0\H,27,1.09843013,7,112.5

5383906,1,-65.68761721,0\H,27,1.09606817,7,112.19637445,1,56.43962608,
0\H,27,1.09534186,7,109.69746401,1,175.53578536,0\C,10,1.53806997,1,11
2.89828271,7,-62.9474432,0\H,31,1.0970972,10,111.35185063,1,-55.848662
42,0\H,31,1.09535556,10,109.78413592,1,-175.1106952,0\H,31,1.09527301,
10,112.10905814,1,65.58170539,0\P,5,1.82545931,1,36.49172595,7,-39.187
58881,0\C,35,1.82598641,5,105.90539838,1,-118.81819194,0\H,36,1.094777
48,35,109.63065962,5,-60.18573469,0\H,36,1.09506849,35,109.1999573,5,5
8.70884497,0\H,36,1.09360143,35,111.24913236,5,178.89252572,0\H,5,1.09
506671,1,90.49831654,7,-163.49995483,0\H,5,1.09456961,1,89.48856967,7,
87.62646755,0\\Version=AM64L-G03RevD.01\State=1-A\HF=-811.3314689\MP2=
-813.3211032\RMSD=2.402e-09\Thermal=0.\PG=C01 [X(C12H28P1)]\\@

115
1\1\GINC-GOLEM\SP\RMP2-FC\6-31+G(2d,p)\C9H21P1\CHRISTOPH\20-Oct-2009\0
\\#p MP2(FC)/6-31+g(2d,p) scf=tight\\pr3sp_2\\0,1\P\C,1,1.87814893\H,2
,1.10017994,1,106.93894021\H,2,1.101692,1,110.14948768,3,115.63137494,
0\C,1,1.87788777,2,99.57690251,3,-59.48717717,0\H,5,1.1001114,1,106.81
996586,2,-159.80949514,0\H,5,1.10170042,1,110.23280067,2,-44.24281457,
0\C,1,1.8785793,5,99.46043486,2,101.36990097,0\H,8,1.10009915,1,106.91
384523,5,-161.44151951,0\H,8,1.10171435,1,110.12150618,5,-45.86797895,
0\C,2,1.54062671,1,113.46716423,5,-179.75125066,0\H,11,1.09903695,2,10
9.25838408,1,55.50921099,0\H,11,1.099539,2,109.73571318,1,-60.61820377
,0\C,5,1.54042479,1,113.53928126,2,80.04759406,0\H,14,1.09899232,5,109
.18814582,1,55.06733288,0\H,14,1.09964552,5,109.80647138,1,-61.0557169
9,0\C,8,1.54048192,1,113.54564634,5,78.30490412,0\H,17,1.09963805,8,10
9.7605696,1,-60.44471235,0\H,17,1.09901247,8,109.24570654,1,55.6666792
8,0\C,11,1.53708261,2,112.6339462,1,177.63180445,0\H,20,1.09798101,11,
110.99258589,2,-59.94959222,0\H,20,1.09802744,11,111.13123743,2,59.833
43291,0\H,20,1.0972848,11,111.22201359,2,179.98145184,0\C,17,1.5371909
6,8,112.61076361,1,177.78532802,0\H,24,1.09728854,17,111.23516593,8,17
9.9857996,0\H,24,1.09803514,17,111.00313818,8,-59.92901562,0\H,24,1.09
802294,17,111.10797182,8,59.83068095,0\C,14,1.53715743,5,112.61918707,
1,177.12386009,0\H,28,1.09803223,14,111.00157136,5,-59.96072042,0\H,28
,1.09801717,14,111.11309015,5,59.81081863,0\H,28,1.09731588,14,111.228
81219,5,179.96398328,0\\Version=AM64L-G03RevD.01\State=1-A\HF=-693.815
6612\MP2=-695.3372641\RMSD=3.416e-09\Thermal=0.\PG=C01 [X(C9H21P1)]\\@

115-Me$^+$
1\1\GINC-NAUTILUS\SP\RMP2-FC\6-31+G(2d,p)\C10H24P1(1+)\CHRISTOPH\20-Oc
t-2009\0\\#p MP2(FC)/6-31+g(2d,p) scf=tight\\pr3mesp_1\\1,1\P\C,1,1.82
163797\H,2,1.09523248,1,110.21540738\H,2,1.09601048,1,109.9726921,3,12
0.04634074,0\H,2,1.09523263,1,110.21544617,4,120.04639921,0\C,1,1.8318
6498,2,109.2407413,3,-179.21698932,0\H,6,1.09972136,1,106.50286345,2,6
0.22539394,0\H,6,1.09908649,1,106.98213534,2,173.8149802,0\C,1,1.83060
718,2,108.52479283,6,-120.82929668,0\H,9,1.09895182,1,106.93315903,2,5
6.88035971,0\H,9,1.09895246,1,106.9330959,2,-56.88044488,0\C,1,1.83186
521,2,109.24077503,9,-120.82935303,0\H,12,1.09908698,1,106.9821076,2,-
173.8148557,0\H,12,1.09972123,1,106.50282207,2,-60.2253352,0\C,12,1.54
642554,1,115.35283964,2,62.81589416,0\H,15,1.09819859,12,109.92238723,
1,-57.77483369,0\H,15,1.09831399,12,109.76314237,1,59.61014104,0\C,9,1
.54640939,1,114.92277009,2,-180.,0\H,18,1.09833796,9,109.81893509,1,58
.65110991,0\H,18,1.09833779,9,109.81890973,1,-58.65119514,0\C,6,1.5464
2641,1,115.35286841,2,-62.81583792,0\H,21,1.09831437,6,109.76317361,1,
-59.61006747,0\H,21,1.09819818,6,109.92235012,1,57.77486521,0\C,15,1.5
367931,12,111.16207814,1,-179.10163667,0\H,24,1.09670848,15,111.270717
64,12,-60.10869311,0\H,24,1.09662549,15,111.26747031,12,60.53935214,0\
H,24,1.09513062,15,110.29679944,12,-179.78558935,0\C,18,1.53673296,9,1
11.13129233,1,180.,0\H,28,1.09671545,18,111.24104431,9,60.28581763,0\H

,28,1.09512022,18,110.36144481,9,180.,0\H,28,1.09671631,18,111.2410211
1,9,-60.28594105,0\C,21,1.53679314,6,111.16209538,1,179.10169445,0\H,3
2,1.09662566,21,111.26747027,6,-60.53938714,0\H,32,1.0967072,21,111.27
073577,6,60.10872889,0\H,32,1.09513084,21,110.29677743,6,179.78566195,
0\\Version=AM64L-G03RevD.01\State=1-A\HF=-733.2702677\MP2=-734.9374244
\RMSD=4.040e-09\Thermal=0.\PG=C01 [X(C10H24P1)]\\@

116

1\1\GINC-GOLEM\SP\RMP2-FC\6-31+G(2d,p)\C9H21P1\CHRISTOPH\18-May-2009\0
\\#p MP2(FC)/6-31+g(2d,p) scf=tight\\btbmpspc001\\0,1\P\C,1,1.92314608
\C,1,1.92026772,2,110.69147031\C,3,1.54758419,1,107.98066743,2,83.3587
8615,0\H,4,1.10007335,3,109.71901325,1,172.94942207,0\H,4,1.0950858,3,
112.43609039,1,-67.47230662,0\H,4,1.09705302,3,111.14031967,1,53.61374
485,0\C,3,1.54114553,1,117.15957392,2,-40.90904823,0\H,8,1.10078974,3,
109.74308784,1,-173.15106948,0\H,8,1.09715908,3,112.15460803,1,-53.996
59049,0\H,8,1.09596843,3,112.03850234,1,67.93020131,0\C,3,1.54975675,1
,105.51246789,2,-161.99319191,0\H,12,1.09964543,3,109.42599412,1,-168.
34016409,0\H,12,1.09827068,3,111.74407162,1,-49.21023824,0\H,12,1.0962
0819,3,112.1370772,1,72.55496143,0\C,2,1.54412739,1,110.05811397,3,-56
.47180719,0\H,16,1.09774039,2,111.72926766,1,-48.03895578,0\H,16,1.096
24097,2,112.10887075,1,73.71184011,0\H,16,1.09999077,2,109.61573923,1,
-167.29262737,0\C,2,1.54351769,1,116.52917228,3,68.85494257,0\H,20,1.0
9653246,2,111.99255493,1,58.68334334,0\H,20,1.1006409,2,109.47663708,1
,177.36203934,0\H,20,1.09444932,2,112.51817182,1,-63.24459353,0\C,2,1.
55067102,1,104.16023297,3,-171.7571098,0\H,24,1.10008004,2,109.7050401
,1,179.17374841,0\H,24,1.09762266,2,112.16591089,1,-61.63690744,0\H,24
,1.09657398,2,111.44834016,1,59.4043295,0\C,1,1.86956552,3,101.5136405
1,8,65.02648469,0\H,28,1.09522074,1,109.27767868,3,74.95191563,0\H,28,
1.09637902,1,108.90121352,3,-168.45533934,0\H,28,1.09727547,1,114.5022
0525,3,-47.13405678,0\\Version=AM64L-G03RevD.01\State=1-A\HF=-693.7950
721\MP2=-695.3424916\RMSD=5.087e-09\Thermal=0.\PG=C01 [X(C9H21P1)]\\@

116-Me$^+$

1\1\GINC-SOLARIS\SP\RMP2-FC\6-31+G(2d,p)\C10H24P1(1+)\CHRISTOPH\18-May
-2009\0\\#p MP2(FC)/6-31+g(2d,p) scf=tight\\btbmpmespc001\\1,1\C\C,1,3
.23121795\C,2,1.54840511,1,100.30153504\H,3,1.0968775,2,108.10700436,1
,-149.02040093,0\H,3,1.0939154,2,113.0234733,1,-30.35375935,0\H,3,1.09
57711,2,112.2010858,1,92.98747436,0\C,2,1.54644961,1,88.47316977,3,-11
0.41117937,0\H,7,1.09697235,2,108.19011274,1,165.09696826,0\H,7,1.0966
4,2,112.592256,1,-76.7066635,0\H,7,1.09465061,2,112.56413165,1,46.7544
2824,0\C,2,1.55184124,1,137.15976634,7,-116.57750961,0\H,11,1.09685137
,2,107.8451806,1,174.49031685,0\H,11,1.09669339,2,113.11866483,1,-67.4
0009509,0\H,11,1.09519838,2,112.46041969,1,56.37111045,0\C,1,1.5464704
5,2,88.4737883,7,-84.24535008,0\H,15,1.09663829,1,112.59118566,2,-76.7
1165447,0\H,15,1.09464921,1,112.56346244,2,46.74923733,0\H,15,1.096971
05,1,108.18828892,2,165.09151853,0\C,1,1.54838013,15,110.35165927,3,-1
11.3789853,0\H,19,1.09577449,1,112.20327451,15,-174.81824161,0\H,19,1.
09687961,1,108.10744001,15,-56.82406556,0\H,19,1.09391913,1,113.024518
64,15,61.84124724,0\C,1,1.55184623,15,108.88396335,19,-119.57144242,0\
H,23,1.0968506,1,107.84551664,15,65.38187522,0\H,23,1.09669632,1,113.1
1842684,15,-176.5084062,0\H,23,1.09519674,1,112.45973654,15,-52.736028
57,0\C,1,3.00059607,15,145.54726614,23,-129.64856962,0\H,27,1.09439048
,1,144.78327821,15,43.11634737,0\H,27,1.09491926,1,83.22262063,15,155.
75549075,0\H,27,1.09455029,1,97.66510747,15,-96.1675808,0\P,27,1.82887
042,1,36.79209641,15,18.57452876,0\C,31,1.82886208,27,106.19571282,1,1
15.97521539,0\H,32,1.09455603,31,110.81812726,27,-169.73560699,0\H,32,
1.0943995,31,109.87753031,27,-49.31785517,0\H,32,1.09493295,31,109.974
85681,27,70.01541535,0\\Version=AM64L-G03RevD.01\State=1-A\HF=-733.250

9984\MP2=-734.943311\RMSD=9.055e-09\Thermal=0.\PG=C01 [X(C10H24P1)]\\@

117
1\1\GINC-CIPCLU03\SP\RMP2-FC\6-31+G(2d,p)\C9H21P1\C4371\29-Oct-2009\0\
\#P MP2(FC)/6-31+G(2d,p) scf=tight\\i3sp_5\\0,1\P\C,1,1.90688474\H,2,1
.10101814,1,103.5703462\C,1,1.89128098,2,104.33698111,3,140.20889375,0
\H,4,1.09927548,1,110.36899332,2,-43.61054617,0\C,1,1.89557934,4,102.9
1133888,2,106.92660337,0\H,6,1.10037097,1,103.00380864,4,62.65424922,0
\C,2,1.54374399,1,109.44879287,4,-105.84401288,0\H,8,1.09610403,2,111.
42454134,1,-53.32565,0\H,8,1.09800939,2,111.39172754,1,66.63807417,0\H
,8,1.09939482,2,110.61938403,1,-173.81857114,0\C,2,1.54115253,1,117.84
044628,4,21.59459873,0\H,12,1.09694891,2,111.65770887,1,-66.34979543,0
\H,12,1.0950133,2,112.61314369,1,55.08953666,0\H,12,1.09953229,2,109.8
4623294,1,175.05227745,0\C,4,1.54173857,1,110.61846072,6,-56.59502718,
0\H,16,1.09856539,4,111.21487322,1,-53.08101762,0\H,16,1.09616279,4,11
2.36104505,1,68.1424522,0\H,16,1.0989088,4,110.06161006,1,-172.1981026
2,0\C,4,1.54319752,1,109.16481039,6,-177.39790541,0\H,20,1.0978013,4,1
10.74991497,1,60.28390636,0\H,20,1.09911458,4,110.34195612,1,179.69362
903,0\H,20,1.09648847,4,112.52700354,1,-60.17518259,0\C,6,1.54283985,1
,109.34766255,4,175.91947186,0\H,24,1.09650496,6,111.46697191,1,-51.97
780022,0\H,24,1.09856022,6,111.66523603,1,68.67072197,0\H,24,1.0988677
1,6,110.41486229,1,-172.25163362,0\C,6,1.5393386,1,117.95022515,4,-56.
20464181,0\H,28,1.0973544,6,111.32917827,1,-68.13123981,0\H,28,1.09691
659,6,112.34430073,1,53.15652948,0\H,28,1.09974264,6,110.28836497,1,17
3.10322009,0\\Version=AM64L-G03RevD.01\State=1-A\HF=-693.7986747\MP2=-
695.3364836\RMSD=3.270e-09\Thermal=0.\PG=C01 [X(C9H21P1)]\\@

117-Me⁺
1\1\GINC-CIPCLU04\SP\RMP2-FC\6-31+G(2d,p)\C10H24P1(1+)\C4371\29-Oct-20
09\0\\#P MP2(FC)/6-31+G(2d,p) scf=tight\\i3mesp_2\\1,1\C\H,1,1.0989683
\C,1,3.0360084,2,90.35030206\H,3,1.10001583,1,84.16506517,2,121.568274
24,0\C,3,3.05570122,1,60.82803006,2,-82.40199748,0\H,5,1.09988626,3,72
.94437845,1,177.11571695,0\C,1,1.54527777,3,146.89165018,5,43.79374311
,0\H,7,1.09594459,1,108.59653074,3,178.08590896,0\H,7,1.0947272,1,112.
98899376,3,-62.88183006,0\H,7,1.09614235,1,111.74956341,3,59.92567467,
0\C,1,1.54519451,7,111.15317391,5,-155.82717084,0\H,11,1.09496319,1,11
2.81365272,7,-173.5922474,0\H,11,1.09671457,1,111.91004851,7,63.419696
56,0\H,11,1.09600046,1,108.5651366,7,-54.74979004,0\C,3,1.54461179,1,1
47.03994218,11,127.41445905,0\H,15,1.09524077,3,112.91682545,1,-50.870
03263,0\H,15,1.096423,3,112.03124116,1,72.24293384,0\H,15,1.09602789,3
,108.50758199,1,-169.63780357,0\C,3,1.54350239,1,92.21707456,11,-95.55
147302,0\H,19,1.0959071,3,113.00640708,1,23.73311209,0\H,19,1.09595948
,3,108.69045978,1,142.82332777,0\H,19,1.09595997,3,111.62702919,1,-99.
08563307,0\C,5,1.5449004,3,139.21408651,1,-85.21331197,0\H,23,1.094829
01,5,112.60094449,3,-28.95456075,0\H,23,1.09555321,5,112.22251375,3,93
.92129079,0\H,23,1.09605986,5,108.42081406,3,-147.60045091,0\C,5,1.545
50462,3,106.38369535,1,72.5778733,0\H,27,1.09572744,5,111.7136111,3,-1
06.98257348,0\H,27,1.09482178,5,113.11563665,3,15.71281106,0\H,27,1.09
587835,5,108.5278312,3,134.82599163,0\P,1,1.85656556,11,111.57554514,7
,-125.80753621,0\C,31,1.82666028,1,108.49423008,11,67.88055801,0\H,32,
1.09445733,31,110.40030602,1,176.51278456,0\H,32,1.09554132,31,109.601
56778,1,-63.84626591,0\H,32,1.09414034,31,110.59233947,1,56.33768928,0
\\Version=AM64L-G03RevD.01\State=1-A\HF=-733.2546946\MP2=-734.9375465\
RMSD=7.686e-09\Thermal=0.\PG=C01 [X(C10H24P1)]\\@

117-BH⁺
1\1\GINC-CIPCLU04\SP\RMP2-FC\6-31+G(2d,p)\C22H32P1(1+)\C2175\17-Sep-20
10\0\\#p MP2(FC)/6-31+G(2d,p) scf=tight\\k9bhsp_1\\1,1\C\H,1,1.0993378

3\C,1,4.35046583,2,104.2468667\C,3,1.39799918,1,58.70868199,2,12.88872
153,0\C,4,1.39702085,3,120.08989264,1,-0.53966091,0\C,5,1.40638747,4,1
21.01400675,3,-0.02817553,0\C,6,1.40581684,5,118.31022534,4,0.53939588
,0\C,3,1.39738416,1,60.82000588,6,-23.15596326,0\H,3,1.08671663,1,178.
87334098,6,179.76069636,0\H,4,1.08695924,3,120.25786137,1,179.19562388
,0\H,5,1.08882317,4,119.26137265,3,178.89827919,0\H,7,1.08643619,6,120
.79873827,5,178.76538247,0\H,8,1.08706957,3,120.12079573,1,-179.285269
97,0\P,1,1.89763314,6,113.63090238,5,120.77235632,0\C,1,4.35477321,6,1
16.06841539,5,-99.75959407,0\C,15,1.39752499,1,59.0277547,6,134.455182
8,0\C,16,1.39727364,15,119.99079533,1,0.15357949,0\C,17,1.40662497,16,
121.04709525,15,0.67522392,0\C,18,1.40415519,17,118.35046284,16,-2.309
33276,0\C,19,1.39793609,18,120.61987953,17,2.26975802,0\H,15,1.0867511
1,1,179.17790919,6,104.88900806,0\H,16,1.08701815,15,120.28907386,1,-1
78.87774469,0\H,17,1.08933793,16,119.02116141,15,-177.88875556,0\H,19,
1.08577521,18,120.37912587,17,-175.16619382,0\H,20,1.0870198,19,119.45
777657,18,-179.68706202,0\C,14,1.8672946,1,116.20874278,6,57.45143001,
0\C,26,1.54407992,14,116.28152241,1,55.90055594,0\H,27,1.0966487,26,10
8.10782594,14,-166.57332216,0\C,14,1.87153678,1,106.91571079,6,-61.170
96645,0\C,29,1.54541266,14,111.9659205,1,-48.86300598,0\H,30,1.0953533
6,29,111.19732787,14,75.72488432,0\C,14,1.87558161,1,106.47387906,6,-1
75.59783728,0\C,32,1.5456561,14,114.10027001,1,147.24898312,0\H,33,1.0
9653017,32,108.02061392,14,-178.76924515,0\H,26,1.09830833,14,102.1378
9584,1,-61.44678766,0\H,27,1.09242903,26,113.38469132,14,-46.89047852,
0\H,27,1.09308295,26,111.84995638,14,75.26342925,0\H,29,1.09842431,14,
102.85401888,1,-164.64896691,0\H,30,1.09640582,29,113.42492405,14,-47.
07718756,0\H,30,1.09607499,29,108.71253134,14,-166.37955817,0\H,32,1.0
9889863,14,102.63631472,1,30.75730274,0\H,33,1.09427162,32,113.1384066
6,14,-60.24978055,0\H,33,1.09543101,32,112.34892719,14,63.15024095,0\C
,29,1.54479295,14,114.72446093,1,78.55055175,0\H,44,1.09374916,29,112.
15530104,14,-65.00582606,0\H,44,1.09481923,29,112.78952805,14,58.06165
669,0\H,44,1.09679982,29,108.03739411,14,176.30147742,0\C,26,1.5471426
8,14,111.32443239,1,-176.43959631,0\H,48,1.09653858,26,108.25225876,14
,171.91411205,0\H,48,1.09526882,26,111.65713784,14,-70.18987128,0\H,48
,1.09486702,26,113.41421306,14,52.91755133,0\C,32,1.5456608,14,113.430
25645,1,-85.10326537,0\H,52,1.09510131,32,111.15874439,14,-75.42186697
,0\H,52,1.09319317,32,113.22246726,14,47.73589127,0\H,52,1.0962708,32,
108.21506024,14,166.94629789,0\\Version=AM64L-G03RevD.01\State=1-A\HF=
-1192.3579837\MP2=-1195.7380668\RMSD=5.369e-09\Thermal=0.\PG=C01 [X(C2
2H32P1)]\\@

117-TT$^+$

1\1\GINC-IBLIS\SP\RMP2-FC\6-31+G(2d,p)\C28H36P1(1+)\CHRISTOPH\05-Sep-2
011\0\\#p MP2(FC)/6-31+g(2d,p) scf=tight\\k9ttsp_3\\1,1\C\P,1,1.967499
75\C,1,4.37909958,2,110.50000996\C,3,1.39009974,1,59.41001589,2,-126.5
1003073,0\C,4,1.3946004,3,120.39999809,1,-0.27999129,0\C,5,1.41090003,
4,121.72996021,3,0.70996773,0\C,6,1.40849933,5,116.38005603,4,-1.94001
9,0\C,3,1.38999941,1,59.85000224,6,16.53851119,0\H,3,1.10379995,8,120.
14999591,7,-179.77863763,0\H,4,1.10430024,3,119.64000326,1,-179.770041
98,0\H,5,1.10270003,4,116.53001022,3,-178.98005746,0\H,7,1.09779911,6,
121.24006208,5,-176.49998383,0\H,8,1.10419988,3,119.49004252,1,179.430
02622,0\C,1,4.37240041,6,111.96349858,5,111.97969049,0\C,14,1.39049991
,1,59.92998588,6,5.3563192,0\C,15,1.3946997,14,120.39998721,1,0.060009
29,0\C,16,1.40869984,15,121.58003887,14,1.16997355,0\C,17,1.40889935,1
6,116.66997735,15,-2.37998771,0\C,14,1.3903006,1,59.40002207,17,120.91
361348,0\H,14,1.10389956,19,120.59995406,18,179.46652213,0\H,15,1.1042
9999,14,119.5899707,1,-178.82004336,0\H,16,1.10140089,15,116.59997853,
14,-177.32998552,0\H,18,1.09880089,17,121.13999506,16,-176.10004111,0\
H,19,1.10419944,14,119.58006186,1,178.84993627,0\C,1,4.37749725,17,107

.30032831,16,119.78404583,0\C,25,1.39010026,1,59.05181536,17,-21.73040
629,0\C,26,1.3942011,25,120.29998423,1,0.06914569,0\C,27,1.41149941,26
,121.76998679,25,1.41004116,0\C,28,1.40830041,27,116.43003658,26,-3.63
003914,0\C,25,1.3902002,1,60.23981851,17,159.27395782,0\H,25,1.1038998
,1,179.16336021,17,-56.48242198,0\H,26,1.10429957,25,119.7100203,1,-17
9.14081366,0\H,27,1.10369987,26,116.90996709,25,-177.81999298,0\H,29,1
.0961994,28,121.69998766,27,-174.92997322,0\H,30,1.10419998,25,119.419
94444,1,178.90440832,0\C,2,2.94609986,1,136.38999681,17,135.62114595,0
\C,36,1.54120061,2,35.530003,1,7.28002983,0\C,37,1.53969957,36,106.860
01356,2,130.36001177,0\H,36,1.1098997,2,99.36001749,1,122.71002056,0\H
,36,1.11029989,2,87.9100026,1,-129.17994759,0\H,37,1.1131001,36,105.50
998633,2,-115.95997758,0\H,38,1.10939929,37,112.65999499,36,171.449999
88,0\H,38,1.11200009,37,111.95998324,36,-66.48995878,0\C,2,2.91371351,
1,100.76762629,17,48.42794397,0\C,44,1.5383998,2,36.26548224,1,-128.38
814704,0\C,45,1.53790038,44,107.5099757,2,133.01857304,0\H,44,1.111299
98,2,95.75582243,1,-8.70868832,0\H,44,1.11140007,2,89.03258715,1,99.64
752569,0\H,45,1.11619901,44,106.47007635,2,-114.06141041,0\H,46,1.1091
0009,45,112.85997854,44,-177.26996033,0\H,46,1.10830052,45,112.1700070
3,44,-54.87998099,0\C,2,2.90898109,1,143.26967978,17,-92.50589438,0\C,
52,1.54299973,2,36.73956941,1,-16.81013796,0\C,53,1.54020016,52,106.47
998655,2,130.25710058,0\H,52,1.10670026,2,89.90729455,1,-148.26567769,
0\H,52,1.11299955,2,147.62825291,1,-27.3717647,0\H,53,1.11500068,52,10
6.74001191,2,-115.74293339,0\H,54,1.10870015,53,112.82999343,52,174.32
002564,0\H,54,1.10989989,53,112.54997161,52,-63.39007108,0\H,36,1.1133
0029,2,145.06997525,1,-13.63994644,0\H,38,1.11300024,37,111.20998566,3
6,52.65997015,0\H,44,1.11300078,2,146.91598958,1,-141.29172462,0\H,46,
1.11309928,45,110.98997856,44,63.49003612,0\H,52,1.11139991,2,94.85435
439,1,104.48511506,0\H,54,1.11299945,53,111.07000647,52,55.73999772,0\
\Version=AM64L-G03RevD.01\State=1-A\HF=-1421.8425438\MP2=-1426.1078998
\RMSD=7.934e-09\Thermal=0.\PG=C01 [X(C28H36P1)]\\@

118

1\1\GINC-YIN\SP\RMP2-FC\6-31+G(2d,p)\C11H21P1\CHRISTOPH\08-Oct-2009\0\
\#p MP2(FC)/6-31+g(2d,p) scf=tight\\pe2sp_26\\0,1\P\C,1,1.87767956\C,2
,1.54626521,1,114.9665188\C,3,1.54168297,2,103.3564681,1,-162.30532094
,0\C,4,1.55089029,3,104.49715657,2,38.76025186,0\C,2,1.55769401,1,111.
7962696,3,-117.04350211,0\H,2,1.10278778,1,109.33728635,6,-120.6100238
4,0\H,3,1.09587717,2,113.26506155,1,75.50801652,0\H,3,1.10017761,2,109
.78025691,1,-44.6862974,0\H,4,1.09898578,3,109.33559247,2,-79.14034523
,0\H,4,1.09686989,3,112.90636394,2,161.67277642,0\H,5,1.09636215,4,111
.62288866,3,-143.72424144,0\H,5,1.09694232,4,110.55639854,3,97.6086633
5,0\H,6,1.09933907,2,110.00262073,1,31.95577795,0\H,6,1.09590654,2,111
.51760337,1,-86.4100002,0\C,1,1.87781188,2,102.94794478,3,-79.60938718
,0\C,16,1.56269808,1,111.52864513,2,-165.33781612,0\C,17,1.56108826,16
,106.41592494,1,-144.60406466,0\C,18,1.5460447,17,105.64172971,16,-4.8
6388838,0\C,19,1.54084395,18,103.87655891,17,28.14218091,0\H,16,1.1021
8413,1,109.10907233,2,-45.18835976,0\H,17,1.0988763,16,110.73201831,1,
-25.07482014,0\H,17,1.09609579,16,110.85959586,1,93.2093711,0\H,18,1.0
9739927,17,109.94971563,16,113.68874498,0\H,18,1.09638397,17,112.26545
521,16,-127.4660812,0\H,19,1.09945285,18,110.24273729,17,-89.0032999,0
\H,19,1.09702854,18,112.86118394,17,151.01557178,0\H,20,1.09599031,19,
112.7266356,18,-163.30678227,0\H,20,1.10025206,19,110.0663926,18,76.57
843369,0\C,1,1.86959747,2,98.93407825,3,179.25525502,0\H,30,1.09621627
,1,109.58705307,2,172.51278835,0\H,30,1.09907591,1,112.74900087,2,51.4
7272506,0\H,30,1.09609248,1,109.66159431,2,-69.66706799,0\\Version=AM6
4L-G03RevD.01\State=1-A\HF=-769.5486748\MP2=-771.3436066\RMSD=3.406e-0
9\Thermal=0.\PG=C01 [X(C11H21P1)]\\@

118-Me$^+$

1\1\GINC-NAUTILUS\SP\RMP2-FC\6-31+G(2d,p)\C12H24P1(1+)\CHRISTOPH\08-Oc
t-2009\0\\#p MP2(FC)/6-31+g(2d,p) scf=tight\\pe2mesp_11\\1,1\P\C,1,1.8
2214641\H,2,1.09535333,1,109.28807983\H,2,1.095063,1,110.546355,3,119.
59631814,0\H,2,1.09512503,1,110.52690035,3,-119.65400091,0\C,1,1.82214
882,2,107.40865372,4,-179.52907651,0\H,6,1.09505871,1,110.54837897,2,-
179.65330415,0\H,6,1.09535038,1,109.28728524,2,60.7494385,0\H,6,1.0951
2732,1,110.52589439,2,-58.90297635,0\C,1,1.83057747,2,110.81025634,6,1
18.89142906,0\C,10,1.5705462,1,114.3068901,2,61.65015114,0\C,11,1.5582
4796,10,105.1354687,1,-140.32538391,0\C,12,1.54143227,11,105.56329084,
10,-13.66970626,0\C,13,1.54117422,12,103.63683272,11,35.15678362,0\H,1
0,1.09986267,1,104.45817604,2,-179.52483524,0\H,11,1.09499548,10,109.6
3079133,1,99.13520758,0\H,11,1.0973316,10,112.85761041,1,-19.59925427,
0\H,12,1.09681883,11,109.82671975,10,104.62776673,0\H,12,1.09433014,11
,111.76183044,10,-136.54273583,0\H,13,1.09480654,12,112.95394213,11,15
7.01607077,0\H,13,1.09811905,12,110.68668533,11,-82.2777087,0\H,14,1.0
9558907,13,113.53238463,12,-163.84458479,0\H,14,1.09918272,13,109.4377
7356,12,75.40775615,0\C,1,1.83057669,2,108.94488009,6,-120.08670802,0\
C,24,1.57057287,1,114.30232991,2,179.59212318,0\C,25,1.55823235,24,105
.13573025,1,-140.30241864,0\C,26,1.54142248,25,105.56088295,24,-13.694
2985,0\C,27,1.54116642,26,103.63541804,25,35.17277344,0\H,24,1.0998610
9,1,104.45806892,2,-61.58521928,0\H,25,1.09499592,24,109.62971528,1,99
.16247023,0\H,25,1.09732825,24,112.8610126,1,-19.57428183,0\H,26,1.096
81859,25,109.82699102,24,104.60075246,0\H,26,1.09432956,25,111.7620630
9,24,-136.56816103,0\H,27,1.09480911,26,112.95397415,25,157.03165477,0
\H,27,1.0981229,26,110.68706849,25,-82.26266059,0\H,28,1.09558749,27,1
13.53323161,26,-163.84479497,0\H,28,1.09918164,27,109.4366725,26,75.40
94301,0\\Version=AM64L-G03RevD.01\State=1-A\HF=-809.0055695\MP2=-810.9
451889\RMSD=2.406e-09\Thermal=0.\PG=C01 [X(C12H24P1)]\\@

119

1\1\GINC-NODE11\SP\RMP2-FC\6-31+G(2d,p)\C12H21P1\ZIP07\04-Sep-2009\0\\
#p MP2(FC)/6-31+g(2d,p) scf=tight\\bu3sp_5\\0,1\P\C,1,1.8688261\C,2,1.
56375858,1,118.33051005\C,3,1.55499795,2,88.6747245,1,-140.2506181,0\C
,4,1.55521433,3,88.51783181,2,18.81603636,0\H,2,1.10058457,1,110.44102
467,3,127.80972322,0\H,3,1.09732599,2,111.48838153,1,-27.63388328,0\H,
3,1.09597677,2,117.32558209,1,98.59664869,0\H,4,1.09621203,3,117.58892
426,2,139.41363753,0\H,4,1.09675724,3,111.71686902,2,-94.02829176,0\H,
5,1.09741472,4,111.51891965,3,93.83318327,0\H,5,1.09599143,4,118.04102
857,3,-139.29330901,0\C,1,1.86813103,2,99.74276662,3,-76.77426105,0\C,
13,1.56425999,1,118.40322882,2,76.91915493,0\C,14,1.5551802,13,88.6858
4115,1,139.94211629,0\C,15,1.55487952,14,88.55223991,13,-18.65871848,0
\H,13,1.10059873,1,110.47257794,2,-51.0109297,0\H,14,1.09739176,13,111
.52333303,1,27.25436872,0\H,14,1.09594336,13,117.27973634,1,-98.930694
14,0\H,15,1.09676469,14,111.72007775,13,94.20973247,0\H,15,1.09619049,
14,117.56020454,13,-139.2558688,0\H,16,1.09597272,15,118.0012025,14,13
9.16320674,0\H,16,1.09740362,15,111.49987618,14,-94.01415988,0\C,1,1.8
6825245,13,99.86438026,16,-77.00489392,0\C,24,1.56409128,1,118.4044768
7,13,77.18780659,0\C,25,1.55504046,24,88.67007891,1,140.06013526,0\C,2
6,1.55517837,25,88.52416705,24,-18.78098238,0\H,24,1.10061347,1,110.51
609147,13,-50.75477885,0\H,25,1.09600286,24,117.31742417,1,-98.7775862
5,0\H,25,1.09739473,24,111.49126583,1,27.43831013,0\H,26,1.09673673,25
,111.70975369,24,94.04669483,0\H,26,1.09618259,25,117.58686558,24,-139
.39058641,0\H,27,1.09599828,26,118.01889375,25,139.30027459,0\H,27,1.0
9739959,26,111.47162907,25,-93.87742787,0\\Version=AM64L-G03RevD.01\St
ate=1-A\HF=-807.3231966\MP2=-809.2478102\RMSD=6.025e-09\Thermal=0.\PG=
C01 [X(C12H21P1)]\\@

119-Me$^+$

1\1\GINC-AZAZEL\SP\RMP2-FC\6-31+G(2d,p)\C13H24P1(1+)\CHRISTOPH\07-Sep-2009\0\\#p MP2(FC)/6-31+g(2d,p) scf=tight\\bu3mesp_53\\1,1\P\C,1,1.829
33293\C,2,1.56682574,1,121.66425046\C,3,1.55577968,2,87.91721337,1,142
.18865283,0\C,4,1.55566835,3,89.35668818,2,-18.9318929,0\H,2,1.0981194
7,1,106.56840286,5,124.67756845,0\H,3,1.09396685,2,116.55859855,1,-97.
33283903,0\H,3,1.0959136,2,112.63060448,1,29.57215631,0\H,4,1.09468575
,3,111.71070503,2,94.24116237,0\H,4,1.09325929,3,117.03675483,2,-139.2
2668246,0\H,5,1.09388569,4,118.33557463,3,138.05056339,0\H,5,1.0962118
2,4,111.50542084,3,-94.6422763,0\C,1,1.82964469,2,109.11418427,3,-165.
4505551,0\C,13,1.56644841,1,121.27259966,2,74.29228969,0\C,14,1.556084
48,13,87.85260066,1,142.57243269,0\C,15,1.55629048,14,89.41770795,13,-
18.74070803,0\H,13,1.0984457,1,106.18533883,2,-52.56601792,0\H,14,1.09
375458,13,116.45812334,1,-96.77348553,0\H,14,1.09594229,13,112.6524704
9,1,30.04018409,0\H,15,1.09462606,14,111.77127149,13,94.45137154,0\H,1
5,1.09323878,14,116.99578027,13,-138.98601586,0\H,16,1.09392064,15,118
.24724974,14,137.8604747,0\H,16,1.09685157,15,111.53112876,14,-94.8627
894,0\C,1,1.8281247,2,111.87622687,3,74.89173643,0\C,24,1.56627185,1,1
20.77223042,2,-52.09639896,0\C,25,1.55667157,24,87.72608506,1,143.5295
3116,0\C,26,1.55640954,25,89.38854479,24,-19.0680583,0\H,24,1.09873895
,1,106.16636587,2,-178.39135717,0\H,25,1.09389205,24,116.57777459,1,-9
5.90577675,0\H,25,1.09565756,24,112.52848325,1,31.08455297,0\H,26,1.09
32495,25,117.06157388,24,-139.4189907,0\H,26,1.09469425,25,111.6727000
5,24,94.05718057,0\H,27,1.09382885,26,118.29648331,25,138.27597925,0\H
,27,1.09649436,26,111.46270929,25,-94.36336932,0\C,1,1.82216158,24,108
.04813586,27,176.80373197,0\H,35,1.0953175,1,109.85743923,24,65.374087
45,0\H,35,1.09477463,1,111.02490738,24,-174.14270516,0\H,35,1.09523458
,1,109.62019364,24,-53.74658989,0\\Version=AM64L-G03RevD.01\State=1-A\
HF=-846.7796052\MP2=-848.8498886\RMSD=2.789e-09\Thermal=0.\PG=C01 [X(C
13H24P1)]\\@

120

1\1\GINC-MORITZ\SP\RMP2-FC\6-31+G(2d,p)\C12H27P1\CHRISTOPH\15-Aug-2009
\0\\#p MP2(FC)/6-31+g(2d,p) scf=tight\\pbu3spc_3\\0,1\P\C,1,1.87589682
\H,2,1.10037949,1,111.34254893\H,2,1.10068699,1,105.87119652,3,-115.28
412809,0\C,1,1.87589668,2,98.87380057,3,49.71739482,0\H,5,1.10037841,1
,111.34098291,2,-49.71377641,0\H,5,1.10068606,1,105.87194577,2,65.5704
6542,0\C,1,1.88065132,5,102.26439042,2,104.71217883,0\H,8,1.10039056,1
,105.84259745,5,73.35297246,0\H,8,1.1003912,1,105.84235474,5,-175.3809
4536,0\C,2,1.53982341,1,113.4917343,5,174.65819745,0\H,11,1.10037646,2
,109.45248353,1,53.0571179,0\H,11,1.10090297,2,109.79081464,1,-63.1470
2084,0\C,5,1.53982343,1,113.49193655,2,-174.65362716,0\H,14,1.10037612
,5,109.45257874,1,-53.0611677,0\H,14,1.10090275,5,109.79089951,1,63.14
294344,0\C,8,1.5392272,1,119.67780413,5,-51.01362099,0\H,17,1.09990638
,8,110.00684869,1,-58.3803489,0\H,17,1.09990601,8,110.00558486,1,58.39
431251,0\C,11,1.53920391,2,112.97466963,1,175.13898186,0\H,20,1.100388
35,11,109.12745812,2,-57.94902578,0\H,20,1.10045983,11,109.24228804,2,
57.75637575,0\C,14,1.53920302,5,112.97431228,1,-175.14303937,0\H,23,1.
10038899,14,109.12754874,5,57.94944376,0\H,23,1.10046046,14,109.242230
05,5,-57.75582,0\C,17,1.54020246,8,112.62451928,1,-179.99381114,0\H,26
,1.10041603,17,109.19612652,8,-57.82816679,0\H,26,1.10041471,17,109.19
641505,8,57.84520816,0\C,20,1.53572157,11,112.9595771,2,179.95297287,0
\H,29,1.09803029,20,111.06853932,11,59.7864066,0\H,29,1.09716165,20,11
1.38463361,11,179.91705659,0\H,29,1.09821408,20,111.12465029,11,-59.94
34186,0\C,23,1.53572208,14,112.95970754,5,-179.95243204,0\H,33,1.09803
,23,111.06851749,14,-59.78661506,0\H,33,1.09821359,23,111.12466776,14,
59.94327594,0\H,33,1.09716172,23,111.38455393,14,-179.91720855,0\C,26,
1.53592333,17,112.98353784,8,-179.99075023,0\H,37,1.09816234,26,111.11

050787,17,-59.87954304,0\H,37,1.09816112,26,111.10940789,17,59.8800161
3,0\H,37,1.09717963,26,111.37979566,17,180.,0\\Version=IA32L-G03RevD.0
1\State=1-A\HF=-810.931277\MP2=-812.912789\RMSD=2.908e-09\Thermal=0.\P
G=C01 [X(C12H27P1)]\\@

120-Me⁺

1\1\GINC-NODE6\SP\RMP2-FC\6-31+G(2d,p)\C13H30P1(1+)\ZIP07\17-Aug-2009\
0\\#p MP2(FC)/6-31+g(2d,p) scf=tight\\pbu3mespc_1\\1,1\P\C,1,1.8318472
2\H,2,1.09970064,1,106.53990912\H,2,1.09892189,1,106.88865598,3,113.58
671221,0\C,1,1.82109927,2,109.2482893,4,174.8130558,0\H,5,1.09585112,1
,110.02976141,2,-59.24919902,0\H,5,1.09505028,1,110.10111951,2,60.8416
4158,0\H,5,1.09504962,1,110.09820631,2,-179.33782388,0\C,1,1.83184347,
5,109.24721651,2,118.51288373,0\H,9,1.09892346,1,106.88822017,5,-174.7
9655669,0\H,9,1.09969994,1,106.54063668,5,-61.20984067,0\C,1,1.8301458
4,5,108.56154679,9,120.74269778,0\H,12,1.09903502,1,106.86106763,5,-56
.78236214,0\H,12,1.0990373,1,106.85978876,5,56.92718435,0\C,12,1.54530
124,1,114.76147834,5,-179.9261447,0\H,15,1.0994761,12,109.94725832,1,-
58.63896317,0\H,15,1.09946824,12,109.94010732,1,58.70672157,0\C,9,1.54
554687,1,115.32146698,5,61.87574145,0\H,18,1.09916219,9,110.07790013,1
,-58.37221629,0\H,18,1.09929148,9,109.94856869,1,59.1544964,0\C,2,1.54
554802,1,115.3220903,5,-61.85763862,0\H,21,1.09928677,2,109.94914161,1
,-59.17400205,0\H,21,1.09916006,2,110.07763363,1,58.35398896,0\C,21,1.
54106312,2,111.49465402,1,179.56773114,0\H,24,1.09940726,21,109.138663
02,2,-58.19444156,0\H,24,1.09944716,21,109.1712982,2,57.86557711,0\C,1
8,1.54106163,9,111.49355438,1,-179.58680532,0\H,27,1.09940828,18,109.1
3871022,9,58.17441052,0\H,27,1.09944981,18,109.1703914,9,-57.88438067,
0\C,15,1.54079157,12,111.62072474,1,-179.96838017,0\H,30,1.09945501,15
,109.15173652,12,-57.95727338,0\H,30,1.09944868,15,109.15110602,12,58.
07585169,0\C,30,1.53490316,15,112.36001083,12,-179.94083061,0\H,33,1.0
9738835,30,111.34307146,15,-60.23805931,0\H,33,1.09738523,30,111.34148
293,15,60.27657649,0\H,33,1.09536336,30,110.65417913,15,-179.98093815,
0\C,27,1.5350341,18,112.30161872,9,-179.89459706,0\H,37,1.09731678,27,
111.32492801,18,60.34729191,0\H,37,1.09536585,27,110.65548291,18,-179.
89186376,0\H,37,1.09728042,27,111.28671305,18,-60.15981398,0\C,24,1.53
50383,21,112.30016993,2,179.87587618,0\H,41,1.09731681,24,111.32461798
,21,-60.36667778,0\H,41,1.09727944,24,111.28649193,21,60.14011926,0\H,
41,1.09536439,24,110.65589753,21,179.87206558,0\\Version=AM64L-G03RevD
.01\State=1-A\HF=-850.3896322\MP2=-852.5152665\RMSD=4.521e-09\Thermal=
0.\PG=C01 [X(C13H30P1)]\\@

121

1\1\GINC-AZAZEL\SP\RMP2-FC\6-31+G(2d,p)\C18H33P1\CHRISTOPH\31-Aug-2009
\0\\#p MP2(FC)/6-31+g(2d,p) scf=tight\\hex3sp_12\\0,1\P\C,1,4.2417529\
C,2,1.54059193,1,19.11118845\C,3,1.55020914,2,111.88945468,1,-9.409585
4,0\C,4,1.54688583,3,110.24163124,2,-54.888173,0\C,5,1.54323652,4,111.
47800047,3,55.19958827,0\C,6,1.53827409,5,111.60284981,4,-55.9826219,0
\H,2,1.10179241,1,97.80834883,4,-72.42941161,0\H,2,1.09912,1,128.89319
315,4,168.8788724,0\H,3,1.10137986,2,109.20575985,1,-130.77001872,0\H,
3,1.09794925,2,110.21795393,1,112.94794895,0\H,4,1.10338838,3,106.5499
9683,2,61.20755317,0\H,5,1.09697755,4,110.83727434,3,177.61546064,0\H,
5,1.10021119,4,109.71207709,3,-65.02291945,0\H,6,1.10165176,5,109.2012
0263,4,64.92194977,0\H,6,1.09935848,5,109.7757805,4,-178.60759123,0\H,
7,1.10157512,6,109.11606366,5,-65.31879347,0\H,7,1.09891277,6,110.3178
4755,5,177.87080609,0\C,1,4.23080891,4,99.71787834,3,-126.34665053,0\C
,19,1.54191547,1,19.65911421,4,55.9739063,0\C,20,1.54861718,19,111.508
45984,1,-10.52885278,0\C,21,1.54421392,20,110.20706034,19,-55.56942416
,0\C,22,1.54281145,21,111.39981094,20,56.16718154,0\C,23,1.53865597,22
,111.38495838,21,-56.20918517,0\H,19,1.10136601,1,97.18942373,21,-74.3

7631566,0\H,19,1.0990909,1,129.31489812,21,167.26762368,0\H,20,1.10163
428,19,108.77443435,1,-131.67100572,0\H,20,1.09838693,19,110.20531343,
1,111.8288355,0\H,21,1.10282251,20,106.17147631,19,60.75037174,0\H,22,
1.09867629,21,110.78712455,20,178.64957612,0\H,22,1.10051024,21,109.32
814229,20,-63.95605977,0\H,23,1.10155285,22,109.13374722,21,64.5510939
3,0\H,23,1.09924359,22,109.99297727,21,-178.83993602,0\H,24,1.10163021
,23,109.10607723,22,-65.76325965,0\H,24,1.09886131,23,110.32364768,22,
177.45634367,0\C,1,4.2334282,21,124.20285199,20,-174.28112186,0\C,36,1
.5410337,1,18.64785732,21,-178.88288119,0\C,37,1.54950272,36,112.05718
919,1,3.20794648,0\C,38,1.54780426,37,109.51438842,36,-55.56779759,0\C
,39,1.54193954,38,111.91960587,37,55.06333349,0\C,40,1.53770162,39,111
.85475017,38,-55.55292918,0\H,36,1.10156074,1,101.52129794,38,-56.4723
379,0\H,36,1.09911695,1,128.35969514,38,-178.66779901,0\H,37,1.1011514
6,36,109.28809279,1,-117.41509444,0\H,37,1.0981005,36,109.60505714,1,1
26.41355963,0\H,38,1.10109797,37,108.33436103,36,61.95543596,0\H,39,1.
0976278,38,110.60697161,37,176.82377671,0\H,39,1.10177597,38,109.16232
089,37,-66.12401824,0\H,40,1.1015324,39,109.04582186,38,65.31136466,0\
H,40,1.09912471,39,109.68326946,38,-178.34750518,0\H,41,1.10134372,40,
109.21809841,39,-65.81437525,0\H,41,1.09887027,40,110.3188155,39,177.2
3059029,0\\Version=AM64L-G03RevD.01\State=1-A\HF=-1041.6590398\MP2=-10
44.5214761\RMSD=4.717e-09\Thermal=0.\PG=C01 [X(C18H33P1)]\\@

121-Me$^+$

1\1\GINC-NODE1\SP\RMP2-FC\6-31+G(2d,p)\C19H36P1(1+)\ZIP07\01-Sep-2009\
0\\#p MP2(FC)/6-31+g(2d,p) scf=tight\\hex3mesp_6\\1,1\P\C,1,4.2392084\
C,2,1.542352,1,21.76734974\C,3,1.55224513,2,110.34972966,1,-5.32416313
,0\C,4,1.55321645,3,110.97195135,2,-56.16528437,0\C,5,1.54230204,4,110
.39308034,3,56.63551403,0\C,2,1.53749752,1,100.36477114,4,42.14601787,
0\H,2,1.10050384,1,97.47835264,4,-69.42148547,0\H,2,1.09703281,1,130.6
7047422,4,171.06341482,0\H,3,1.09957063,2,109.34564434,1,-126.28902733
,0\H,3,1.09695075,2,109.37900815,1,116.72742437,0\H,4,1.10204245,3,107
.40912739,2,61.45129667,0\H,5,1.0972172,4,111.08459635,3,178.73110031,
0\H,5,1.09934584,4,109.53027546,3,-63.29925288,0\H,6,1.10041546,5,109.
25756856,4,65.32352903,0\H,6,1.09706541,5,108.96346775,4,-178.53545914
,0\H,7,1.10054012,2,109.49370564,1,85.07496659,0\H,7,1.09672587,2,110.
00818161,1,-158.09398222,0\C,1,4.21858277,4,101.84511703,3,76.54664542
,0\C,19,1.54220026,1,20.97554079,4,-65.51557645,0\C,20,1.55303866,19,1
10.56371211,1,-3.85736266,0\C,21,1.55224098,20,110.70610027,19,-56.713
68525,0\C,22,1.54336761,21,110.50897885,20,56.07664102,0\C,19,1.537064
6,1,100.20113143,21,45.99741757,0\H,19,1.10040696,1,98.32472671,21,-65
.96177186,0\H,19,1.09705025,1,129.927506,21,174.02622095,0\H,20,1.0997
6379,19,108.87509435,1,-124.11659155,0\H,20,1.09720187,19,109.76442807
,1,118.90876434,0\H,21,1.10085119,20,108.20253479,19,61.65322389,0\H,2
2,1.09712098,21,110.94720666,20,177.40651827,0\H,22,1.10043738,21,109.
64381131,20,-64.3865779,0\H,23,1.10039868,22,108.93951353,21,65.966763
01,0\H,23,1.09698091,22,108.91062429,21,-178.08071434,0\H,24,1.1005284
4,19,109.43181553,1,84.10293932,0\H,24,1.09670991,19,110.07434761,1,-1
59.04607511,0\C,1,4.22396997,21,128.49719493,20,167.5863329,0\C,36,1.5
4349591,1,21.40791847,21,-178.14602046,0\C,37,1.55198242,36,110.346105
71,1,-4.89761623,0\C,38,1.5503531,37,110.91452367,36,-56.37341177,0\C,
39,1.54241653,38,110.45647463,37,56.79238658,0\C,36,1.53750532,1,100.3
4536647,21,57.00894965,0\H,36,1.10028183,1,97.83665441,21,-54.73419741
,0\H,36,1.09698925,1,130.30505908,21,-174.43784327,0\H,37,1.09999977,3
6,109.05026192,1,-125.62156705,0\H,37,1.09724344,36,109.37162066,1,117
.42648769,0\H,38,1.10184281,37,107.51130761,36,61.85370657,0\H,39,1.09
817116,38,111.02320908,37,178.93961253,0\H,39,1.09952655,38,109.298000
2,37,-63.10172289,0\H,40,1.1003883,39,109.16340451,38,65.28121922,0\H,
40,1.09696294,39,109.07336936,38,-178.53444346,0\H,41,1.10055232,36,10

9.4418395,1,84.75914808,0\H,41,1.09666934,36,109.9708895,1,-158.440225
73,0\C,1,1.82740502,21,108.32097471,20,55.85171616,0\H,53,1.09558833,1
,109.53965481,21,61.41540448,0\H,53,1.09379931,1,110.55695346,21,-58.6
6160234,0\H,53,1.09421022,1,110.42027884,21,-178.89222059,0\\Version=A
M64L-G03RevD.01\State=1-A\HF=-1081.1230883\MP2=-1084.130727\RMSD=9.570
e-09\Thermal=0.\PG=C01 [X(C19H36P1)]\\@

122

1\1\GINC-GOLEM\SP\RMP2-FC\6-31+G(2d,p)\C12H27P1\CHRISTOPH\20-May-2009\
0\\#p MP2(FC)/6-31+g(2d,p) scf=tight\\ttbpspc001\\0,1\P\C,1,1.94820396
\C,1,1.94787281,2,107.63971901\C,1,1.9484735,3,107.62455714,2,115.7301
3026,0\C,2,1.54890964,1,109.27538247,3,165.12771657,0\H,5,1.09976357,2
,108.6323437,1,168.02842712,0\H,5,1.09396477,2,112.98630201,1,-73.3226
8792,0\H,5,1.09676337,2,111.8270261,1,49.63517173,0\C,2,1.55344145,1,1
06.55768324,3,-81.88483883,0\H,9,1.09184346,2,113.41836412,1,69.149942
38,0\H,9,1.10027851,2,109.06140241,1,-171.39172315,0\H,9,1.09670994,2,
111.01536114,1,-52.61999607,0\C,2,1.54503692,1,117.88627755,3,41.26335
94,0\H,13,1.09509491,2,112.33583823,1,59.32152515,0\H,13,1.10116224,2,
109.35083903,1,178.4878042,0\H,13,1.09466067,2,112.49110256,1,-62.7804
1605,0\C,3,1.54875257,1,109.1671738,2,49.60140312,0\H,17,1.09979802,3,
108.65571908,1,168.05540471,0\H,17,1.09373915,3,113.01410985,1,-73.249
09872,0\H,17,1.09676837,3,111.79714296,1,49.67001439,0\C,3,1.54437001,
1,117.90875027,2,-74.31987618,0\H,21,1.09509282,3,112.33718276,1,59.66
014654,0\H,21,1.1010634,3,109.36257863,1,178.86202338,0\H,21,1.0945660
8,3,112.41461185,1,-62.42184072,0\C,3,1.55309326,1,106.53699677,2,162.
51443648,0\H,25,1.10025184,3,109.07423726,1,-171.36337444,0\H,25,1.096
67463,3,111.00414507,1,-52.58447658,0\H,25,1.09188089,3,113.40652383,1
,69.16261249,0\C,4,1.5489073,1,109.14108687,3,49.61984126,0\H,29,1.099
85772,4,108.66094516,1,168.00562188,0\H,29,1.09397604,4,112.97660465,1
,-73.33937787,0\H,29,1.09682929,4,111.83150999,1,49.59138862,0\C,4,1.5
448882,1,117.91054392,3,-74.23262531,0\H,33,1.09486799,4,112.34324174,
1,59.33769165,0\H,33,1.10114526,4,109.35852524,1,178.53374767,0\H,33,1
.09466752,4,112.45642299,1,-62.74749904,0\C,4,1.55321915,1,106.6045337
3,3,162.57515252,0\H,37,1.09672922,4,110.996562,1,-52.76370209,0\H,37,
1.09154768,4,113.42936539,1,69.00703,0\H,37,1.10024657,4,109.0502209,1
,-171.52053219,0\\Version=AM64L-G03RevD.01\State=1-A\HF=-810.8772444\M
P2=-812.904321\RMSD=8.548e-09\Thermal=0.\PG=C01 [X(C12H27P1)]\\@

122-Me$^+$

1\1\GINC-NAUTILUS\SP\RMP2-FC\6-31+G(2d,p)\C13H30P1(1+)\CHRISTOPH\23-Ma
y-2009\0\\#p MP2(FC)/6-31+g(2d,p) scf=tight\\ttbpmespc001_2\\1,1\C\C,1
,3.20707305\C,2,3.20846204,1,60.03520651\C,1,1.55318531,2,139.29924138
,3,63.42238707,0\H,4,1.09724135,1,107.26744325,2,160.2996496,0\H,4,1.0
9006568,1,114.01361818,2,-81.31568206,0\H,4,1.09546318,1,112.34072088,
2,43.06371396,0\C,1,1.54993044,4,106.36748498,3,-151.68260759,0\H,8,1.
09250831,1,113.23276669,4,-172.4966611,0\H,8,1.09711694,1,107.28898788
,4,-54.63189476,0\H,8,1.09550161,1,112.85822639,4,62.86948426,0\C,1,1.
54778007,8,108.77260656,4,118.13087381,0\H,12,1.09348962,1,112.8533308
5,8,-172.5959769,0\H,12,1.09730343,1,107.97656016,8,-54.50246403,0\H,1
2,1.09480526,1,112.82592713,8,63.85379602,0\C,2,1.552886,1,97.22813764
,12,135.7442345,0\H,16,1.09731085,2,107.31761573,1,-148.83002212,0\H,1
6,1.09069055,2,113.90556471,1,-30.43409498,0\H,16,1.09522319,2,112.376
73349,1,93.90958691,0\C,2,1.54690513,1,88.22659782,12,26.01667414,0\H,
20,1.09325928,2,112.75549136,1,40.87920624,0\H,20,1.09717686,2,107.999
72171,1,158.97619036,0\H,20,1.09443546,2,112.80029849,1,-82.61104802,0
\C,2,1.55042328,1,143.46814414,12,-93.77946004,0\H,24,1.09726909,2,107
.26889919,1,176.56357032,0\H,24,1.09558983,2,112.81339882,1,-65.957211
2,0\H,24,1.092612,2,113.27171901,1,58.6749198,0\C,3,1.55310992,2,97.25

412652,1,-144.00073701,0\H,28,1.09726653,3,107.30665966,2,-147.1300036
3,0\H,28,1.0898838,3,114.1027582,2,-28.63711162,0\H,28,1.09532247,3,11
2.19726235,2,95.66095382,0\C,3,1.54720285,2,88.25174391,1,106.196773,0
\H,32,1.09383173,3,112.76409275,2,41.70469556,0\H,32,1.09710464,3,108.
01957515,2,159.79881361,0\H,32,1.09405368,3,112.86870152,2,-81.8182192
7,0\C,3,1.55033814,2,143.40777566,1,-13.71416152,0\H,36,1.09538872,3,1
12.89415136,2,-65.15451587,0\H,36,1.0926496,3,113.22937462,2,59.467228
13,0\H,36,1.09712443,3,107.2659091,2,177.35430495,0\P,1,1.92222775,12,
111.60120382,8,-122.34106106,0\C,40,1.83402009,1,105.54964247,12,-163.
76272674,0\H,41,1.09397669,40,110.31506662,1,74.18653332,0\H,41,1.0939
9627,40,110.27128871,1,-165.85322817,0\H,41,1.09400154,40,110.36987508
,1,-45.86694973,0\\Version=AM64L-G03RevD.01\State=1-A\HF=-850.337462\M
P2=-852.5103675\RMSD=2.800e-09\Thermal=0.\PG=C01 [X(C13H30P1)]\\@

123

1\1\GINC-AZAZEL\SP\RMP2-FC\6-31+G(2d,p)\C15H27P1\CHRISTOPH\23-Oct-2009
\0\\#p MP2(FC)/6-31+g(2d,p) scf=tight\\pe3sp_2\\0,1\P\C,1,1.8854515\C,
2,1.5648617,1,113.81756401\C,3,1.55969523,2,105.898277,1,-149.99677342
,0\C,4,1.54644222,3,105.5811879,2,-9.29793634,0\C,5,1.54056339,4,103.5
2290187,3,31.67549934,0\H,2,1.10002511,1,103.30053785,3,115.43028645,0
\H,3,1.09632656,2,110.17201299,1,88.73418573,0\H,3,1.09544963,2,111.89
1809,1,-29.29993788,0\H,4,1.09639669,3,112.49697216,2,-132.29303058,0\
H,4,1.09854829,3,109.99013346,2,108.77396209,0\H,5,1.09731573,4,112.84
538391,3,154.51904351,0\H,5,1.09958437,4,110.23904198,3,-85.44605517,0
\H,6,1.09729509,5,113.27888773,4,-163.87611002,0\H,6,1.09903263,5,109.
41651934,4,76.22628219,0\C,1,1.88452546,2,100.52088153,6,64.09386686,0
\C,16,1.57006975,1,112.98187637,2,174.7826731,0\C,17,1.55480498,16,106
.42613766,1,-130.44299065,0\C,18,1.53982505,17,104.81066287,16,-18.312
01792,0\C,19,1.53948734,18,102.93980287,17,36.9018083,0\H,16,1.1002465
4,1,109.22288455,2,-64.09357795,0\H,17,1.09695435,16,110.00903568,1,10
9.33403988,0\H,17,1.09700408,16,111.98236426,1,-9.30119492,0\H,18,1.09
847743,17,109.85809003,16,99.0498597,0\H,18,1.09665179,17,112.57043986
,16,-141.6495728,0\H,19,1.09723839,18,113.1537458,17,159.26615748,0\H,
19,1.09984449,18,110.29583272,17,-80.22463382,0\H,20,1.0966589,19,113.
04071873,18,-164.02244818,0\H,20,1.1011501,19,109.28718788,18,76.33304
076,0\C,1,1.88168613,16,100.95711577,20,162.04484557,0\C,30,1.54955666
,1,114.26848285,16,-170.02193472,0\C,31,1.55391268,30,105.04832888,1,-
161.17826861,0\C,32,1.55875447,31,105.85070234,30,21.11386418,0\C,33,1
.54830994,32,105.62681029,31,4.54053946,0\H,30,1.10207718,1,109.739841
19,16,-47.51441791,0\H,31,1.09634426,30,112.97487614,1,76.05489877,0\H
,31,1.09961676,30,109.24492594,1,-43.19272383,0\H,32,1.09647421,31,111
.18424644,30,143.04907318,0\H,32,1.09699117,31,110.54532802,30,-98.637
98595,0\H,33,1.09647434,32,112.46085772,31,126.78294389,0\H,33,1.09758
358,32,110.23048793,31,-114.14292735,0\H,34,1.09547425,33,112.44641926
,32,-151.54553658,0\H,34,1.09984516,33,110.23815917,32,88.6792779,0\\V
ersion=AM64L-G03RevD.01\State=1-A\HF=-924.5231043\MP2=-926.9194972\RMS
D=4.338e-09\Thermal=0.\PG=C01 [X(C15H27P1)]\\@

123-Me$^+$

1\1\GINC-SOLARIS\SP\RMP2-FC\6-31+G(2d,p)\C16H30P1(1+)\CHRISTOPH\23-Oct
-2009\0\\#p MP2(FC)/6-31+g(2d,p) scf=tight\\pe3mesp_30\\1,1\P\C,1,1.82
61239\H,2,1.09434085,1,110.79638125\H,2,1.09499443,1,110.22386827,3,12
0.74690916,0\H,2,1.09535338,1,109.43030921,4,119.36711384,0\C,1,1.8370
3914,2,107.04744924,3,-177.64854993,0\C,6,1.56584521,1,114.89253012,2,
-55.31108377,0\C,7,1.56165083,6,105.08601174,1,-147.80995841,0\C,8,1.5
4476359,7,105.97313363,6,-7.01033792,0\C,9,1.54083665,8,104.0090941,7,
30.68876516,0\H,6,1.10013223,1,104.18818833,2,63.18340899,0\H,7,1.0974
6574,6,112.29316493,1,-27.92652511,0\H,7,1.09494996,6,110.24751757,1,9

0.95409499,0\H,8,1.09432559,7,111.70230623,6,-129.6262474,0\H,8,1.0962
3454,7,109.76855198,6,111.7748921,0\H,9,1.09812423,8,110.6244593,7,-86
.81755977,0\H,9,1.09487113,8,112.80060185,7,152.77749939,0\H,10,1.0960
8336,9,113.38250432,8,-163.591906,0\H,10,1.09804364,9,109.7808504,8,75
.47257554,0\C,1,1.84197586,2,107.98909482,6,-119.71259685,0\C,20,1.560
74318,1,114.28695759,2,-40.81777182,0\C,21,1.53996699,20,102.78677313,
1,-158.14402566,0\C,22,1.53914554,21,103.40526372,20,41.70670634,0\C,2
3,1.55361921,22,105.0094851,21,-38.39097146,0\H,20,1.0990539,1,104.085
01636,2,76.43890328,0\H,21,1.09544873,20,112.04248617,1,79.65255361,0\
H,21,1.09796165,20,111.3010714,1,-41.08403652,0\H,22,1.09504789,21,112
.33311194,20,163.95502301,0\H,22,1.09839359,21,109.89483107,20,-76.295
6819,0\H,23,1.09790343,22,109.75033502,21,79.84378287,0\H,23,1.0946204
2,22,113.03744542,21,-160.58662399,0\H,24,1.09523576,23,111.1415823,22
,-98.50690045,0\H,24,1.09532574,23,111.27534665,22,142.81196628,0\C,1,
1.83784581,2,109.52848587,6,117.36433068,0\C,34,1.55416941,1,116.51998
46,2,60.68900066,0\C,35,1.54072946,34,102.18701013,1,-162.29158586,0\C
,36,1.54233198,35,103.76681645,34,42.32004314,0\C,37,1.55926199,36,105
.77001824,35,-33.31332847,0\H,34,1.09888809,1,104.86597628,2,179.94488
519,0\H,35,1.0989526,34,111.10456822,1,-45.50937325,0\H,35,1.09555869,
34,112.62072579,1,75.77755191,0\H,36,1.09490361,35,112.37842349,34,164
.56314398,0\H,36,1.09819952,35,109.62860469,34,-75.93371511,0\H,37,1.0
9654916,36,109.84395365,35,85.05663107,0\H,37,1.0943218,36,112.5644101
7,35,-155.61940534,0\H,38,1.09695192,37,110.35471517,36,132.83293236,0
\H,38,1.09511628,37,112.12398491,36,-108.37527416,0\\Version=AM64L-G03
RevD.01\State=1-A\HF=-963.9849156\MP2=-966.5264513\RMSD=2.978e-09\Ther
mal=0.\PG=C01 [X(C16H30P1)]\\@

124

1\1\GINC-AZAZEL\SP\RMP2-FC\6-31+G(2d,p)\C18H33P1\CHRISTOPH\31-Aug-2009
\0\\#p MP2(FC)/6-31+g(2d,p) scf=tight\\hex3sp_12\\0,1\P\C,1,4.2417529\
C,2,1.54059193,1,19.11118845\C,3,1.55020914,2,111.88945468,1,-9.409585
4,0\C,4,1.54688583,3,110.24163124,2,-54.888173,0\C,5,1.54323652,4,111.
47800047,3,55.19958827,0\C,6,1.53827409,5,111.60284981,4,-55.9826219,0
\H,2,1.10179241,1,97.80834883,4,-72.42941161,0\H,2,1.09912,1,128.89319
315,4,168.8788724,0\H,3,1.10137986,2,109.20575985,1,-130.77001872,0\H,
3,1.09794925,2,110.21795393,1,112.94794895,0\H,4,1.10338838,3,106.5499
9683,2,61.20755317,0\H,5,1.09697755,4,110.83727434,3,177.61546064,0\H,
5,1.10021119,4,109.71207709,3,-65.02291945,0\H,6,1.10165176,5,109.2012
0263,4,64.92194977,0\H,6,1.09935848,5,109.7757805,4,-178.60759123,0\H,
7,1.10157512,6,109.11606366,5,-65.31879347,0\H,7,1.09891277,6,110.3178
4755,5,177.87080609,0\C,1,4.23080891,4,99.71787834,3,-126.34665053,0\C
,19,1.54191547,1,19.65911421,4,55.9739063,0\C,20,1.54861718,19,111.508
45984,1,-10.52885278,0\C,21,1.54421392,20,110.20706034,19,-55.56942416
,0\C,22,1.54281145,21,111.39981094,20,56.16718154,0\C,23,1.53865597,22
,111.38495838,21,-56.20918517,0\H,19,1.10136601,1,97.18942373,21,-74.3
7631566,0\H,19,1.0990909,1,129.31489812,21,167.26762368,0\H,20,1.10163
428,19,108.77443435,1,-131.67100572,0\H,20,1.09838693,19,110.20531343,
1,111.8288355,0\H,21,1.10282251,20,106.17147631,19,60.75037174,0\H,22,
1.09867629,21,110.78712455,20,178.64957612,0\H,22,1.10051024,21,109.32
814229,20,-63.95605977,0\H,23,1.10155285,22,109.13374722,21,64.5510939
3,0\H,23,1.09924359,22,109.99297727,21,-178.83993602,0\H,24,1.10163021
,23,109.10607723,22,-65.76325965,0\H,24,1.09886131,23,110.32364768,22,
177.45634367,0\C,1,4.2334282,21,124.20285199,20,-174.28112186,0\C,36,1
.5410337,1,18.64785732,21,-178.88288119,0\C,37,1.54950272,36,112.05718
919,1,3.20794648,0\C,38,1.54780426,37,109.51438842,36,-55.56779759,0\C
,39,1.54193954,38,111.91960587,37,55.06333349,0\C,40,1.53770162,39,111
.85475017,38,-55.55292918,0\H,36,1.10156074,1,101.52129794,38,-56.4723
379,0\H,36,1.09911695,1,128.35969514,38,-178.66779901,0\H,37,1.1011514

6,36,109.28809279,1,-117.41509444,0\H,37,1.0981005,36,109.60505714,1,1
26.41355963,0\H,38,1.10109797,37,108.33436103,36,61.95543596,0\H,39,1.
0976278,38,110.60697161,37,176.82377671,0\H,39,1.10177597,38,109.16232
089,37,-66.12401824,0\H,40,1.1015324,39,109.04582186,38,65.31136466,0\
H,40,1.09912471,39,109.68326946,38,-178.34750518,0\H,41,1.10134372,40,
109.21809841,39,-65.81437525,0\H,41,1.09887027,40,110.3188155,39,177.2
3059029,0\\Version=AM64L-G03RevD.01\State=1-A\HF=-1041.6590398\MP2=-10
44.5214761\RMSD=4.717e-09\Thermal=0.\PG=C01 [X(C18H33P1)]\\@

124-Me$^+$

1\1\GINC-NODE1\SP\RMP2-FC\6-31+G(2d,p)\C19H36P1(1+)\ZIP07\01-Sep-2009\
0\\#p MP2(FC)/6-31+g(2d,p) scf=tight\\hex3mesp_6\\1,1\P\C,1,4.2392084\
C,2,1.542352,1,21.76734974\C,3,1.55224513,2,110.34972966,1,-5.32416313
,0\C,4,1.55321645,3,110.97195135,2,-56.16528437,0\C,5,1.54230204,4,110
.39308034,3,56.63551403,0\C,2,1.53749752,1,100.36477114,4,42.14601787,
0\H,2,1.10050384,1,97.47835264,4,-69.42148547,0\H,2,1.09703281,1,130.6
7047422,4,171.06341482,0\H,3,1.09957063,2,109.34564434,1,-126.28902733
,0\H,3,1.09695075,2,109.37900815,1,116.72742437,0\H,4,1.10204245,3,107
.40912739,2,61.45129667,0\H,5,1.0972172,4,111.08459635,3,178.73110031,
0\H,5,1.09934584,4,109.53027546,3,-63.29925288,0\H,6,1.10041546,5,109.
25756856,4,65.32352903,0\H,6,1.09706541,5,108.96346775,4,-178.53545914
,0\H,7,1.10054012,2,109.49370564,1,85.07496659,0\H,7,1.09672587,2,110.
00818161,1,-158.09398222,0\C,1,4.21858277,4,101.84511703,3,76.54664542
,0\C,19,1.54220026,1,20.97554079,4,-65.51557645,0\C,20,1.55303866,19,1
10.56371211,1,-3.85736266,0\C,21,1.55224098,20,110.70610027,19,-56.713
68525,0\C,22,1.54336761,21,110.50897885,20,56.07664102,0\C,19,1.537064
6,1,100.20113143,21,45.99741757,0\H,19,1.10040696,1,98.32472671,21,-65
.96177186,0\H,19,1.09705025,1,129.927506,21,174.02622095,0\H,20,1.0997
6379,19,108.87509435,1,-124.11659155,0\H,20,1.09720187,19,109.76442807
,1,118.90876434,0\H,21,1.10085119,20,108.20253479,19,61.65322389,0\H,2
2,1.09712098,21,110.94720666,20,177.40651827,0\H,22,1.10043738,21,109.
64381131,20,-64.3865779,0\H,23,1.10039868,22,108.93951353,21,65.966763
01,0\H,23,1.09698091,22,108.91062429,21,-178.08071434,0\H,24,1.1005284
4,19,109.43181553,1,84.10293932,0\H,24,1.09670991,19,110.07434761,1,-1
59.04607511,0\C,1,4.22396997,21,128.49719493,20,167.5863329,0\C,36,1.5
4349591,1,21.40791847,21,-178.14602046,0\C,37,1.55198242,36,110.346105
71,1,-4.89761623,0\C,38,1.5503531,37,110.91452367,36,-56.37341177,0\C,
39,1.54241653,38,110.45647463,37,56.79238658,0\C,36,1.53750532,1,100.3
4536647,21,57.00894965,0\H,36,1.10028183,1,97.83665441,21,-54.73419741
,0\H,36,1.09698925,1,130.30505908,21,-174.43784327,0\H,37,1.09999977,3
6,109.05026192,1,-125.62156705,0\H,37,1.09724344,36,109.37162066,1,117
.42648769,0\H,38,1.10184281,37,107.51130761,36,61.85370657,0\H,39,1.09
817116,38,111.02320908,37,178.93961253,0\H,39,1.09952655,38,109.298000
2,37,-63.10172289,0\H,40,1.1003883,39,109.16340451,38,65.28121922,0\H,
40,1.09696294,39,109.07336936,38,-178.53444346,0\H,41,1.10055232,36,10
9.4418395,1,84.75914808,0\H,41,1.09666934,36,109.9708895,1,-158.440225
73,0\C,1,1.82740502,21,108.32097471,20,55.85171616,0\H,53,1.09558833,1
,109.53965481,21,61.41540448,0\H,53,1.09379931,1,110.55695346,21,-58.6
6160234,0\H,53,1.09421022,1,110.42027884,21,-178.89222059,0\\Version=A
M64L-G03RevD.01\State=1-A\HF=-1081.1230883\MP2=-1084.130727\RMSD=9.570
e-09\Thermal=0.\PG=C01 [X(C19H36P1)]\\@

124-BH$^+$

1\1\GINC-CIPCLU02\SP\RMP2-FC\6-31+G(2d,p)\C31H44P1(1+)\C2175\15-Sep-20
10\0\\#p MP2(FC)/6-31+G(2d,p) scf=tight\\k8bhsp_1\\1,1\C\H,1,1.0989866
6\C,1,4.35508003,2,103.30247862\C,3,1.39803897,1,60.46764848,2,160.299
81273,0\C,4,1.39789658,3,120.43815914,1,0.17740367,0\C,5,1.40404736,4,
120.64951065,3,0.64809258,0\C,6,1.40666571,5,118.31010091,4,-2.3709602

6,0\C,7,1.39735355,6,121.06461558,5,2.43202344,0\H,3,1.08683702,1,179.
19257664,6,-176.43805645,0\H,4,1.08711741,3,120.10041176,1,-178.917242
93,0\H,5,1.08575689,4,118.96410621,3,-177.00767453,0\H,7,1.08921246,6,
119.85866608,5,-176.25752671,0\H,8,1.08715394,7,119.72414787,6,-179.68
878928,0\P,1,1.89922144,6,116.18780035,5,-93.96468717,0\C,1,4.35124867
,6,114.64034695,5,45.1072021,0\C,15,1.39810072,1,58.72801382,6,100.288
91426,0\C,16,1.39712601,15,120.09898259,1,0.57045231,0\C,17,1.40633153
,16,121.01240472,15,-0.00248904,0\C,18,1.40552613,17,118.2822455,16,-0
.53247612,0\C,15,1.39731146,1,60.79353459,18,24.2806546,0\H,15,1.08679
186,1,178.90077687,18,-179.07250015,0\H,16,1.08703138,15,120.23185443,
1,-179.17403888,0\H,17,1.08875559,16,119.31186313,15,-178.97209448,0\H
,19,1.08615179,18,120.65851728,17,-178.84259192,0\H,20,1.08717774,15,1
20.12641,1,179.31131233,0\H,14,2.3593051,1,101.5952496,18,-33.17821363
,0\H,14,2.3725236,1,82.69727587,18,161.92517764,0\H,14,2.37085505,1,13
2.36724609,18,70.66195656,0\C,14,4.25695356,1,105.12643944,18,43.16262
047,0\C,29,1.54357745,14,22.96571339,1,-64.20492562,0\C,30,1.55190269,
29,109.81201931,14,6.743328,0\C,31,1.55157768,30,110.53324615,29,57.31
747732,0\C,32,1.54238972,31,110.44173363,30,-57.8548415,0\C,29,1.53750
308,14,100.37986358,1,58.68767059,0\H,29,1.09722516,14,131.82315979,1,
-71.0875701,0\H,29,1.10093124,14,96.34309113,1,169.91407426,0\H,30,1.0
9685583,29,109.16859396,14,-115.12418944,0\H,30,1.09701641,29,109.6883
5369,14,127.75778168,0\H,32,1.09869309,31,111.25517336,30,179.86748846
,0\H,32,1.09849968,31,109.31952688,30,61.56181049,0\H,33,1.09727284,32
,109.12960795,31,178.72884234,0\H,33,1.10062865,32,109.33028894,31,-64
.94020703,0\H,34,1.09698154,29,110.09538052,14,156.88061612,0\H,34,1.1
0050984,29,109.34251266,14,-86.21014143,0\C,7,4.07631051,6,99.43756328
,5,100.35568692,0\C,45,1.54125269,7,58.77476465,6,7.54049821,0\C,46,1.
552623,45,110.2078652,7,77.24606394,0\C,47,1.55276305,46,109.92834829,
45,58.77421181,0\C,48,1.54417672,47,110.11917387,46,-57.51242329,0\C,4
9,1.537014,48,112.36067632,47,55.57759991,0\H,45,1.09727413,7,110.5680
3461,6,-92.82521279,0\H,45,1.10061742,7,51.89671927,6,171.81698796,0\H
,46,1.09507903,45,110.02813567,7,-45.92693824,0\H,46,1.0986252,45,108.
66945919,7,-163.45361086,0\H,48,1.09896603,47,109.79499459,46,63.26345
36,0\H,48,1.09581323,47,111.41860935,46,-178.18787348,0\H,49,1.0973269
2,48,108.90369436,47,178.4079425,0\H,49,1.10082526,48,108.79926792,47,
-65.83900417,0\H,50,1.09701179,49,109.97189676,48,-176.43219499,0\H,50
,1.10060541,49,109.42204157,48,66.82470161,0\C,14,4.21458685,1,135.208
24094,18,-51.1813913,0\C,61,1.54349649,14,21.68510443,1,-170.41013294,
0\C,62,1.55472044,61,110.03948531,14,11.13260756,0\C,63,1.55051071,62,
110.08983699,61,58.17207628,0\C,64,1.54243308,63,109.808461,62,-58.963
75613,0\C,61,1.53788989,14,102.28440067,1,-51.30416419,0\H,61,1.097294
75,14,130.33615807,1,177.78029007,0\H,61,1.10058657,14,95.71897437,1,6
0.29543067,0\H,62,1.09706761,61,109.42634657,14,-111.7194999,0\H,62,1.
09901793,61,109.30795496,14,131.29727411,0\H,64,1.09566793,63,110.0229
6141,62,60.95714334,0\H,64,1.09431214,63,111.34958756,62,178.74484885,
0\H,65,1.09724825,64,109.05048464,63,179.55139045,0\H,65,1.10122466,64
,109.45866713,63,-64.17784183,0\H,66,1.09690933,61,109.9968351,14,156.
26747901,0\H,66,1.10058151,61,109.34040111,14,-86.99948816,0\\Version=
AM64L-G03RevD.01\State=1-A\HF=-1540.2235439\MP2=-1544.9347562\RMSD=9.5
28e-09\Thermal=0.\PG=C01 [X(C31H44P1)]\\@

124-TT⁺

1\1\GINC-IBLIS\SP\RMP2-FC\6-31+G(2d,p)\C37H48P1(1+)\CHRISTOPH\12-Sep-2
011\0\\#p MP2(FC)/6-31+g(2d,p) scf=tight\\k8ttsp_3\\1,1\C\P,1,2.073664
21\C,1,4.39874069,2,110.29244045\C,3,1.39845288,1,59.48701438,2,53.671
70041,0\C,4,1.3961634,3,120.20543712,1,-0.93680978,0\C,5,1.41090836,4,
121.79582498,3,-0.85724097,0\C,6,1.40680847,5,117.01331196,4,2.5031938
,0\C,3,1.3946946,1,59.45101909,6,-84.77599173,0\H,3,1.08676116,1,179.1

2628617,6,-177.60091237,0\H,4,1.08728808,3,120.35594091,1,178.1887027,
0\H,5,1.08534564,4,118.13101635,3,176.933241,0\H,7,1.08309449,6,120.46
012814,5,176.51006382,0\H,8,1.08705985,3,120.23970693,1,-178.29995875,
0\C,1,4.40072447,6,110.75936211,5,-63.78366453,0\C,14,1.39458852,1,59.
46432348,6,-4.94987547,0\C,15,1.40049499,14,120.74615974,1,0.97566566,
0\C,16,1.40709914,15,121.28625899,14,0.6014682,0\C,17,1.41118418,16,11
6.94017547,15,-2.4540701,0\C,18,1.39610035,17,121.85681238,16,2.615720
13,0\H,14,1.08676416,1,179.22429604,6,-99.34767682,0\H,15,1.08706939,1
4,120.2163068,1,-178.42283674,0\H,16,1.08302318,15,118.22801088,14,-17
8.33399022,0\H,18,1.08512843,17,120.07561189,16,-175.32176599,0\H,19,1
.08729481,18,119.43300572,17,179.9460903,0\C,1,4.39986926,6,108.326015
37,5,176.81840922,0\C,25,1.39836548,1,59.45190687,6,-63.80370441,0\C,2
6,1.39609097,25,120.19259027,1,-0.81254109,0\C,27,1.410979,26,121.8292
3107,25,-0.91645728,0\C,28,1.40694579,27,116.9813539,26,2.70923714,0\C
,25,1.39466875,1,59.47806119,6,115.87579544,0\H,25,1.08678007,1,179.26
442616,6,20.27301561,0\H,26,1.087283,25,120.3613664,1,178.32691893,0\H
,27,1.08531649,26,118.07371129,25,176.95507494,0\H,29,1.08299993,28,12
0.46680341,27,176.44299339,0\H,30,1.08706696,25,120.22058806,1,-178.46
611995,0\C,2,4.28534283,1,125.49030971,6,0.91624891,0\C,36,1.54446624,
2,22.24431805,1,-165.16766709,0\C,37,1.55516092,36,110.4860333,2,-4.87
517715,0\C,38,1.55166532,37,111.82157858,36,-53.73571743,0\C,39,1.5433
0008,38,111.13423067,37,53.48374543,0\C,40,1.53498203,39,112.04777866,
38,-54.76205025,0\H,36,1.1006283,2,97.52686605,1,-41.52055625,0\H,36,1
.09747971,2,130.77625543,1,-161.18161828,0\H,37,1.09545295,36,109.1092
6971,2,-127.40398005,0\H,37,1.09403931,36,108.56090979,2,116.24236573,
0\H,38,1.09547723,37,106.30979842,36,61.99933414,0\H,39,1.09461589,38,
109.93727406,37,173.27422235,0\H,39,1.09903285,38,110.40833435,37,-68.
00181058,0\H,40,1.10019174,39,108.78749264,38,66.54741615,0\H,40,1.097
34541,39,108.80790589,38,-177.64366096,0\H,41,1.10074519,40,109.707619
75,39,-64.83124519,0\H,41,1.09695439,40,110.20539642,39,178.01540719,0
\C,2,4.28702876,1,125.15216852,6,-119.2056208,0\C,53,1.5446071,2,22.32
703378,1,-165.95966933,0\C,54,1.55490336,53,110.47175661,2,-5.13912211
,0\C,55,1.55133096,54,111.73996619,53,-53.87540837,0\C,56,1.5433624,55
,111.09097563,54,53.75633968,0\C,57,1.53508389,56,111.99943394,55,-54.
96328512,0\H,53,1.10058084,2,97.39864474,1,-42.12998914,0\H,53,1.09748
555,2,130.83960416,1,-161.68104468,0\H,54,1.09553687,53,109.06939936,2
,-127.69666057,0\H,54,1.09377801,53,108.51592225,2,115.96144776,0\H,55
,1.09566591,54,106.28429049,53,61.77013369,0\H,56,1.09455758,55,109.90
029282,54,173.61066047,0\H,56,1.09897241,55,110.45196589,54,-67.620971
78,0\H,57,1.10017779,56,108.80870483,55,66.39080426,0\H,57,1.09734948,
56,108.80614169,55,-177.78747302,0\H,58,1.10077288,57,109.67170379,56,
-64.82464354,0\H,58,1.09697401,57,110.22080595,56,178.02937761,0\C,2,4
.28960552,1,124.9316634,6,121.3289965,0\C,70,1.54479483,2,22.56016752,
1,-167.4308181,0\C,71,1.55571723,70,110.3646862,2,-5.19478953,0\C,72,1
.55106478,71,111.77007683,70,-53.92533484,0\C,73,1.54259844,72,111.083
22191,71,53.85765425,0\C,74,1.53500783,73,112.00519505,72,-54.99078358
,0\H,70,1.10057491,2,97.20463565,1,-43.50336459,0\H,70,1.09747987,2,13
1.0659547,1,-163.00866592,0\H,71,1.09530759,70,109.09278261,2,-127.858
63003,0\H,71,1.0939537,70,108.56817394,2,115.78090966,0\H,72,1.0955573
5,71,106.12687142,70,61.70159055,0\H,73,1.09441027,72,109.9212768,71,1
73.61360818,0\H,73,1.09894055,72,110.44099381,71,-67.6003294,0\H,74,1.
10013918,73,108.78114892,72,66.34656814,0\H,74,1.09734224,73,108.82351
428,72,-177.82600324,0\H,75,1.100802,74,109.68959295,73,-64.89400237,0
\H,75,1.09697267,74,110.24586274,73,177.96097536,0\\Version=AM64L-G03R
evD.01\State=1-A\HF=-1769.7304096\MP2=-1775.321716\RMSD=6.794e-09\Ther
mal=0.\PG=C01 [X(C37H48P1)]\\@

125

1\1\GINC-MAX\SP\RMP2-FC\6-31+G(2d,p)\C6H6N9P1\CHRISTOPH\24-Nov-2009\0\
\#p MP2(FC)/6-31+g(2d,p) scf=tight\\tru3sp_8\\0,1\P\C,1,2.89580538\C,2
,1.3710948,1,128.51385285\N,3,2.17598263,2,75.18678192,1,1.46094072,0\
C,1,2.78125494,4,124.40361311,3,76.63593207,0\C,5,1.37362451,1,135.790
21382,4,-87.77111,0\N,6,2.17651106,5,74.56263237,1,-0.00408743,0\C,1,2
.89585334,4,111.35700599,3,-47.7613459,0\C,8,1.3710929,1,128.51232737,
4,-98.00353511,0\N,9,2.1759701,8,75.18672888,1,-1.46489263,0\H,9,1.080
86865,8,129.44954207,1,179.05993398,0\H,8,1.07902059,1,98.12298269,10,
-177.64334314,0\H,6,1.08037658,5,129.7873065,1,179.99799156,0\H,5,1.08
055335,1,91.19473777,10,-92.23296313,0\H,3,1.08086851,2,129.4493767,1,
-179.06222471,0\H,2,1.07901892,1,98.12219417,10,-82.75037887,0\N,8,1.3
7081932,1,24.30229804,10,-2.17372595,0\N,10,1.29044368,9,36.49174683,8
,179.87228765,0\N,2,1.37081363,1,24.303855,17,123.64061048,0\N,4,1.290
44434,3,36.49151608,2,-179.87181915,0\N,5,1.36812233,1,31.16326397,19,
-57.32873185,0\N,7,1.29625303,6,36.33307598,5,-179.99847469,0\\Version
=IA32L-G03RevD.01\State=1-A\HF=-1061.3511554\MP2=-1063.9274053\RMSD=7.
994e-09\Thermal=0.\PG=C01 [X(C6H6N9P1)]\\@

125-Me$^+$

1\1\GINC-GOLEM\SP\RMP2-FC\6-31+G(2d,p)\C7H9N9P1(1+)\CHRISTOPH\24-Nov-2
009\0\\#p MP2(FC)/6-31+g(2d,p) scf=tight\\tru3mesp_26\\1,1\C\C,1,1.367
67929\N,2,2.18796534,1,76.26839298\H,2,1.08071046,1,129.39383981,3,179
.66406034,0\H,1,1.08100142,2,132.74970087,3,179.1097003,0\P,3,2.664971
32,2,103.57723513,1,-4.79704561,0\C,6,1.78752518,3,104.43874392,2,-103
.8666915,0\H,7,1.09632413,6,108.54427654,3,-141.95064975,0\H,7,1.09623
438,6,108.49813807,3,-22.30093356,0\H,7,1.09628965,6,108.70731516,3,98
.0824746,0\N,6,2.62112582,3,110.33035876,2,162.90925688,0\C,11,2.18703
724,6,105.11805398,3,-40.26529245,0\C,12,1.36656124,11,76.45480652,6,2
.03202697,0\C,6,2.8175681,3,129.99045601,2,59.73972858,0\C,14,1.368577
07,6,129.52097319,3,-105.24364399,0\N,15,2.18540314,14,76.11112622,6,-
1.99962081,0\H,13,1.07984203,12,132.81716562,11,177.17609167,0\H,12,1.
08070805,11,154.18748354,6,179.94502975,0\H,15,1.08075109,14,129.39080
721,6,179.00278109,0\H,14,1.08068476,6,97.86011485,3,74.41762801,0\N,1
4,1.37915899,6,25.46544423,3,-110.46317965,0\N,16,1.27773792,15,36.082
20079,14,179.5106544,0\N,13,1.38241118,12,104.14094659,11,0.2486048,0\
N,11,1.27280352,6,141.13649509,3,-42.46768811,0\N,1,1.38215599,2,104.2
2613438,3,-0.93208341,0\N,3,1.27540355,2,35.95734778,1,179.78308672,0\
\Version=AM64L-G03RevD.01\State=1-A\HF=-1100.7317891\MP2=-1103.4435688
\RMSD=5.455e-09\Thermal=0.\PG=C01 [X(C7H9N9P1)]\\@

126

1\1\GINC-AZAZEL\SP\RMP2-FC\6-31+G(2d,p)\C9H9N6P1\CHRISTOPH\17-Feb-2012
\0\\#p MP2(FC)/6-31+g(2d,p) scf=tight\\imi13sp_10\\0,1\P\C,1,2.8161284
4\N,2,1.31007658,1,140.66552964\C,3,1.38117254,2,105.35662503,1,-10.76
805861,0\C,4,1.36972399,3,110.91837516,2,-0.16410597,0\H,2,1.08195288,
1,93.02658969,5,-177.32539738,0\H,4,1.08245963,3,121.24698632,2,-179.2
7182745,0\H,5,1.08045009,4,132.46799383,3,-178.98725251,0\C,1,2.737163
78,5,98.05981682,4,-168.47911587,0\N,9,1.30587268,1,145.12842447,5,60.
01252671,0\C,10,1.38596305,9,105.12379802,1,-2.64916166,0\C,11,1.36726
992,10,111.15097973,9,0.06496011,0\H,9,1.0834506,1,88.81086763,5,-122.
03197367,0\H,11,1.08256415,10,121.0158964,9,-179.60489236,0\H,12,1.079
41063,11,132.45898613,10,-177.92621332,0\C,1,2.87297956,9,108.06240038
,10,-46.7027066,0\C,16,1.36704713,1,131.83847502,9,-105.25307426,0\N,1
7,1.38619698,16,111.14877038,1,-1.74738396,0\C,18,1.30532708,17,105.14
537518,16,0.06124752,0\H,19,1.08342195,18,126.09070647,17,-179.9802511
5,0\H,16,1.07920076,1,95.78124105,19,-178.98780316,0\H,17,1.08252983,1
6,127.8225311,1,178.40071934,0\N,2,1.38812667,1,29.8169585,19,160.0037
3643,0\N,19,1.39223885,18,112.6435003,17,-0.30326323,0\N,9,1.39134895,

1,32.5530229,24,-73.10085181,0\\Version=AM64L-G03RevD.01\State=1-A\HF=
-1013.5010368\MP2=-1015.9484398\RMSD=7.046e-09\Thermal=0.\PG=C01 [X(C9
H9N6P1)]\\@

126-Me$^+$

1\1\GINC-TOFU\SP\RMP2-FC\6-31+G(2d,p)\C10H12N6P1(1+)\CHRISTOPH\17-Feb-
2012\0\\#p MP2(FC)/6-31+g(2d,p) scf=tight\\imi13mesp_1\\1,1\C\P,1,1.79
917674\H,1,1.09577439,2,109.77053846\H,1,1.09590666,2,109.70939118,3,1
19.98663655,0\H,1,1.09578921,2,109.71535218,4,119.9496735,0\C,2,2.7657
8605,1,126.5500401,3,-84.51920668,0\N,6,1.29240209,2,139.85031048,1,60
.20286566,0\C,7,1.38932393,6,106.77788056,2,-2.42926761,0\C,8,1.361524
07,7,111.21554346,6,-0.20619717,0\C,2,2.76207678,1,93.58162822,9,119.5
7141584,0\C,10,1.36134568,2,133.9245564,1,-141.77410421,0\N,11,1.38932
665,10,111.22317049,2,2.89731264,0\C,12,1.29228363,11,106.78790839,10,
-0.24014163,0\C,2,2.76267092,1,93.75604203,10,120.229094,0\C,14,1.3614
3133,2,133.92971906,1,-141.13198432,0\N,15,1.38929187,14,111.22341808,
2,2.90617534,0\C,16,1.29239038,15,106.77067079,14,-0.21167689,0\H,15,1
.08201966,14,127.71975801,2,-177.42459819,0\H,14,1.08075789,2,93.60325
495,1,41.32858737,0\H,17,1.082524,16,127.13608517,15,179.37270546,0\H,
13,1.08254991,12,127.16068447,11,179.3493804,0\H,10,1.08076076,2,93.63
349873,1,40.74302584,0\H,11,1.08201028,10,127.73116897,2,-177.45341966
,0\H,6,1.08252174,2,92.96864032,1,-121.21420039,0\H,8,1.08201285,7,121
.06697909,6,-179.85610278,0\H,9,1.0808212,8,132.38852241,7,179.5670746
4,0\N,14,1.41682513,2,28.72267053,1,-135.08696503,0\N,9,1.41662628,8,1
05.31625369,7,0.05212781,0\N,10,1.41691626,2,28.71420424,1,-135.733749
34,0\\Version=AM64L-G03RevD.01\State=1-A\HF=-1052.8927578\MP2=-1055.47
72603\RMSD=7.053e-09\Thermal=0.\PG=C01 [X(C10H12N6P1)]\\@

127

1\1\GINC-YIN\SP\RMP2-FC\6-31+G(2d,p)\C5H7N6P1\CHRISTOPH\25-Nov-2009\0\
\#p MP2(FC)/6-31+g(2d,p) scf=tight\\tru2sp_1\\0,1\P\C,1,2.80280854\C,2
,1.3738889,1,135.05431442\N,3,2.17734269,2,74.4405418,1,1.29646873,0\C
,1,2.87461134,4,71.02822048,3,-125.05890454,0\C,5,1.37286109,1,130.475
90097,4,151.7778627,0\N,6,2.17642415,5,74.84352639,1,-0.77502778,0\C,1
,1.84529362,7,115.76497182,6,58.34678255,0\H,8,1.09342376,1,113.114203
37,7,-78.65339994,0\H,6,1.08101292,5,129.54443669,1,179.8486696,0\H,5,
1.08001133,1,96.13241475,8,52.55483674,0\H,3,1.08062449,2,129.81257136
,1,-178.88197584,0\H,2,1.08038987,1,91.81404567,8,139.02880241,0\H,8,1
.09617027,1,107.86493777,7,43.33888016,0\H,8,1.09630708,1,108.31895217
,7,160.49137773,0\N,5,1.36833839,1,26.20153735,8,-130.36510954,0\N,7,1
.29595102,6,36.47278793,5,179.59770246,0\N,2,1.3666049,1,30.41087066,1
6,72.46930477,0\N,4,1.29846196,3,36.36727316,2,-179.87131532,0\\Versio
n=AM64L-G03RevD.01\State=1-A\HF=-860.7728726\MP2=-862.6899246\RMSD=6.4
39e-09\Thermal=0.\PG=C01 [X(C5H7N6P1)]\\@

127-Me$^+$

1\1\GINC-EDDY\SP\RMP2-FC\6-31+G(2d,p)\C6H10N6P1(1+)\CHRISTOPH\24-Nov-2
009\0\\#p MP2(FC)/6-31+g(2d,p) scf=tight\\tru2mesp_6\\1,1\C\H,1,1.0959
7926\P,1,1.80000004,2,108.7614322\C,3,1.80002798,1,114.0183358,2,-55.8
853475,0\H,4,1.09422334,3,110.36988216,1,-174.39345165,0\H,4,1.0956964
5,3,108.88372091,1,63.88604486,0\H,4,1.09598782,3,108.76456741,1,-55.8
087269,0\N,3,2.65605235,1,133.48291041,4,-101.70485892,0\C,8,2.1846134
7,3,104.52265439,1,-44.92165488,0\C,9,1.36761492,8,75.97010687,3,4.079
78492,0\C,3,2.81246964,1,126.72761191,4,113.26668427,0\C,11,1.367615,3
,130.11615238,1,32.54739703,0\N,12,2.18464529,11,75.9725328,3,-4.83477
487,0\H,10,1.08084482,9,132.13863319,8,-179.80485263,0\H,9,1.08069506,
8,154.50388862,3,-176.85931737,0\H,12,1.08069597,11,129.52178732,3,175
.6751395,0\H,11,1.08085448,3,97.61542595,1,-151.23471021,0\H,1,1.09421

068,3,110.38029162,4,-174.46590341,0\H,1,1.09569615,3,108.87549331,4,6
3.80120789,0\N,11,1.38012299,3,26.19508942,1,19.90731957,0\N,13,1.2760
5716,12,36.12598031,11,179.93257396,0\N,10,1.3801171,9,104.40542508,8,
0.89555197,0\N,8,1.27610757,3,140.51442747,1,-48.77472294,0\\Version=A
M64L-G03RevD.01\State=1-A\HF=-900.1682992\MP2=-902.2246435\RMSD=8.056e
-09\Thermal=0.\PG=C01 [X(C6H10N6P1)]\\@

128

1\1\GINC-TOFU\SP\RMP2-FC\6-31+G(2d,p)\C7H9N4P1\CHRISTOPH\17-Feb-2012\0
\\#p MP2(FC)/6-31+g(2d,p) scf=tight\\imi12sp_11\\0,1\P\C,1,1.84666685\
H,2,1.09598619,1,108.16538165\H,2,1.09503623,1,114.57420755,3,121.3624
7352,0\H,2,1.09598475,1,108.16338364,3,-117.27775782,0\C,1,2.75130435,
2,118.83048384,4,-70.92118084,0\N,6,1.30867332,1,145.32704427,2,46.507
49295,0\C,7,1.38368166,6,104.94005152,1,-0.06513996,0\C,8,1.36873204,7
,110.98577675,6,-0.20663758,0\H,6,1.08337663,1,88.69953678,2,-133.4735
6486,0\H,8,1.08278684,7,121.11038167,6,179.83777717,0\H,9,1.07972855,8
,131.87133264,7,179.7279832,0\C,1,2.75130037,2,118.83305383,9,110.2105
7365,0\N,13,1.30867319,1,145.32769457,2,-46.32777238,0\C,14,1.38368105
,13,104.94014428,1,-0.02277892,0\C,15,1.3687326,14,110.9857603,13,0.20
482514,0\H,13,1.08337728,1,88.69875951,2,133.58253862,0\H,15,1.0827856
4,14,121.110409,13,-179.84209686,0\H,16,1.07972854,15,131.87096805,14,
-179.72800138,0\N,13,1.38621787,1,32.42463027,2,-45.81088632,0\N,6,1.3
8621898,1,32.42460773,20,-71.30028887,0\\Version=AM64L-G03RevD.01\Stat
e=1-A\HF=-828.8693182\MP2=-830.6989077\RMSD=5.849e-09\Thermal=0.\PG=C0
1 [X(C7H9N4P1)]\\@

128-Me$^+$

1\1\GINC-TOFU\SP\RMP2-FC\6-31+G(2d,p)\C8H12N4P1(1+)\CHRISTOPH\17-Feb-2
012\0\\#p MP2(FC)/6-31+g(2d,p) scf=tight\\imi12mesp_5\\1,1\C\P,1,1.805
73996\H,2,2.41715267,1,135.78775631\H,1,1.09518547,2,110.45911619,3,17
9.74635763,0\H,1,1.09654833,2,108.00107844,3,-61.45558774,0\H,1,1.0960
5356,2,110.87475146,5,119.18103352,0\C,2,2.78459926,1,115.61493392,4,-
85.73352852,0\N,7,1.29356179,2,139.43894444,1,76.46632169,0\C,8,1.3884
0322,7,106.53854258,2,4.12805055,0\C,9,1.36175369,8,111.18029952,7,-0.
02912189,0\C,2,1.80574055,1,110.67022221,10,-143.08865465,0\C,2,2.7535
6823,1,132.13317088,11,-127.23233005,0\C,12,1.36175419,2,134.73988792,
1,19.43773265,0\N,13,1.38840248,12,111.18027795,2,-3.76630477,0\C,14,1
.29356113,13,106.53860229,12,-0.0275237,0\H,13,1.081883,12,127.7837163
8,2,176.89557554,0\H,12,1.08088683,2,92.66162779,1,-161.84291745,0\H,1
5,1.08242065,14,126.31185498,13,-179.9073396,0\H,7,1.08242085,2,94.174
62154,1,-100.27467457,0\H,9,1.08188407,8,121.03263331,7,179.36140773,0
\H,10,1.0808867,9,132.58471494,8,177.97175388,0\H,11,1.09654768,2,108.
00130903,1,-61.84728157,0\H,11,1.0960535,2,110.87472835,1,57.33372549,
0\N,7,1.41435656,2,28.09259994,1,85.456957,0\N,15,1.41435728,14,111.57
798043,13,-0.4116282,0\\Version=AM64L-G03RevD.01\State=1-A\HF=-868.270
8946\MP2=-870.241733\RMSD=5.689e-09\Thermal=0.\PG=C01 [X(C8H12N4P1)]\\
@

129

1\1\GINC-MORITZ\SP\RMP2-FC\6-31+G(2d,p)\C4H8N3P1\CHRISTOPH\24-Nov-2009
\0\\#p MP2(FC)/6-31+g(2d,p) scf=tight\\tru1sp_1\\0,1\P\C,1,2.81804359\
C,2,1.37577656,1,134.95303756\N,3,2.17689847,2,74.18670778,1,-0.002984
45,0\C,1,1.85439377,4,83.33051111,3,-129.45585021,0\H,5,1.09744155,1,1
08.45001894,4,-151.87796777,0\C,1,1.85440175,5,100.1417664,4,81.965015
43,0\H,7,1.09744198,1,108.45047178,5,70.01122982,0\H,3,1.08090692,2,12
9.82114681,1,179.99558247,0\H,2,1.08026544,1,91.85545614,5,-123.859647
8,0\H,7,1.09501211,1,111.94590599,5,-51.00727675,0\H,7,1.09566142,1,10
9.52685564,5,-172.34132986,0\H,5,1.09564163,1,109.54888603,7,172.42175

649,0\H,5,1.09499241,1,111.92673114,7,51.08400657,0\N,2,1.36377809,1,3
0.25538071,5,56.13769257,0\N,4,1.3031026,3,36.3704509,2,179.9996071,0\
\Version=IA32L-G03RevD.01\State=1-A\HF=-660.1853939\MP2=-661.4441569\R
MSD=6.748e-09\Thermal=0.\PG=C01 [X(C4H8N3P1)]\\@

129-Me$^+$

1\1\GINC-GRETEL\SP\RMP2-FC\6-31+G(2d,p)\C5H11N3P1(1+)\CHRISTOPH\24-Nov
-2009\0\\#p MP2(FC)/6-31+g(2d,p) scf=tight\\tru1mesp_1\\1,1\C\H,1,1.09
559824\H,1,3.91732534,2,94.87972196\P,1,1.80909761,2,110.77387941,3,-1
9.71361177,0\C,4,1.80606668,1,111.53655028,2,179.61658433,0\H,5,1.0963
5963,4,109.61586108,1,-66.57795487,0\H,5,1.09540053,4,109.73385513,1,5
3.8743099,0\H,5,1.09533577,4,109.00490716,1,173.21746717,0\C,4,1.80871
98,1,111.10643209,5,-124.28903678,0\C,4,2.83968823,1,88.3455913,9,-112
.48704989,0\C,10,1.37004395,4,129.33815343,1,-169.91189703,0\N,11,2.18
087483,10,75.66596035,4,3.05522601,0\H,11,1.08068895,10,129.43004696,4
,-177.49399135,0\H,10,1.08047232,4,98.43386664,1,11.89272856,0\H,1,1.0
9554235,4,110.22530243,5,-58.6488853,0\H,1,1.09694727,4,108.74537077,5
,60.53931414,0\H,9,1.09563329,4,109.99278521,1,-56.73196823,0\H,9,1.09
666548,4,109.89424587,1,63.77806669,0\N,10,1.37726598,4,25.35653555,1,
-161.77188397,0\N,12,1.28221848,11,36.21925887,10,-179.87338306,0\\Ver
sion=IA32L-G03RevD.01\State=1-A\HF=-699.6007933\MP2=-701.0009982\RMSD=
7.560e-09\Thermal=0.\PG=C01 [X(C5H11N3P1)]\\@

130

1\1\GINC-YIN\SP\RMP2-FC\6-31+G(2d,p)\C12H12N3P1\CHRISTOPH\29-Sep-2009\
0\\#p MP2(FC)/6-31+g(2d,p) scf=tight\\pyr3sp_12\\0,1\P\C,1,2.73317035\
C,2,1.37231295,1,141.35197169\C,3,1.432277,2,107.30717312,1,2.95735114
,0\C,4,1.37386001,3,107.74178957,2,-0.33987779,0\C,1,2.73417031,2,119.
34675556,3,-59.60590447,0\C,6,1.37240655,1,141.36872214,2,105.44578785
,0\C,7,1.43210023,6,107.30331517,1,2.71276725,0\C,8,1.37398036,7,107.7
4141084,6,-0.42171185,0\C,1,2.73296687,2,119.27768906,3,105.03686282,0
\C,10,1.37239422,1,141.40139974,2,-59.23194615,0\C,11,1.43213397,10,10
7.30464704,1,2.51150804,0\C,12,1.37398716,11,107.74348577,10,-0.389236
14,0\H,9,1.08093171,8,131.25429596,7,177.30988947,0\H,8,1.08292949,7,1
26.75397454,6,178.53112626,0\H,7,1.0825385,6,125.79176064,1,-178.12204
366,0\H,6,1.08209659,1,87.4946725,10,122.6737849,0\H,4,1.08293667,3,12
6.7416827,2,178.51708104,0\H,3,1.08253125,2,125.78810521,1,-177.900987
73,0\H,5,1.08099582,4,131.26924615,3,177.1591971,0\H,2,1.08208627,1,87
.47819112,10,-73.00090542,0\H,11,1.08253493,10,125.78402874,1,-178.285
29324,0\H,10,1.08209219,1,87.44861499,2,122.41969309,0\H,13,1.08095752
,12,131.26225971,11,177.26415526,0\H,12,1.08291924,11,126.75322732,10,
178.55830291,0\N,13,1.39407904,12,108.18524455,11,0.1730364,0\N,5,1.39
419072,4,108.19255708,3,0.21599513,0\N,9,1.39413601,8,108.19064731,7,0
.23545448,0\\Version=AM64L-G03RevD.01\State=1-A\HF=-965.4894456\MP2=-9
67.8555864\RMSD=9.955e-09\Thermal=0.\PG=C01 [X(C12H12N3P1)]\\@

130-Me$^+$

1\1\GINC-YIN\SP\RMP2-FC\6-31+G(2d,p)\C13H15N3P1(1+)\CHRISTOPH\29-Sep-2
009\0\\#p MP2(FC)/6-31+g(2d,p) scf=tight\\pyr3mesp_13\\1,1\P\C,1,1.799
79629\H,2,1.09524498,1,109.9248069\H,2,1.09529182,1,109.77022484,3,120
.09686929,0\H,2,1.0952843,1,109.72768653,4,119.82344678,0\C,1,2.737452
7,2,128.02937592,3,-158.79877376,0\C,6,1.36293874,1,136.84426368,2,-62
.09897905,0\C,7,1.43923989,6,108.30990247,1,6.08939522,0\C,8,1.3635544
4,7,108.19317585,6,0.17489142,0\C,1,2.73220528,2,129.22048968,9,157.82
39566,0\C,10,1.36308621,1,136.93834795,2,-58.91923588,0\C,11,1.4393430
1,10,108.29277786,1,6.80438014,0\C,12,1.36341634,11,108.20058317,10,0.
07719537,0\C,1,2.73336947,2,129.28014773,13,156.81058306,0\C,14,1.3628
7378,1,136.75012336,2,-60.56365194,0\C,15,1.43934625,14,108.31322691,1
,7.5179069,0\C,16,1.36335984,15,108.19045447,14,0.15397887,0\H,11,1.08

220655,10,125.30878905,1,-174.23782643,0\H,10,1.08106775,1,90.98887245
,2,125.28430188,0\H,13,1.08104832,12,131.14187322,11,-179.48221962,0\H
,12,1.0823258,11,126.49756715,10,179.26685398,0\H,15,1.08222017,14,125
.26944605,1,-173.48420469,0\H,14,1.0809637,1,91.19916248,2,124.1920253
,0\H,17,1.08113971,16,131.13451924,15,-179.40767802,0\H,16,1.08232745,
15,126.49018035,14,179.27982203,0\H,9,1.08116956,8,131.15469558,7,-179
.22112433,0\H,8,1.08231995,7,126.4938589,6,179.44429207,0\H,7,1.082220
46,6,125.26876085,1,-174.84873136,0\H,6,1.08091497,1,91.16901099,2,121
.65953749,0\N,13,1.41480162,12,107.72477591,11,0.47854903,0\N,9,1.4149
7155,8,107.72852642,7,0.28507166,0\N,17,1.41496005,16,107.7529928,15,0
.41389203,0\\Version=AM64L-G03RevD.01\State=1-A\HF=-1004.9111988\MP2=-
1007.4134063\RMSD=5.933e-09\Thermal=0.\PG=C01 [X(C13H15N3P1)]\\@

131
1\1\GINC-STEAK\SP\RMP2-FC\6-31+G(2d,p)\C5H9N2P1\CHRISTOPH\17-Feb-2012\
0\\#p MP2(FC)/6-31+g(2d,p) scf=tight\\imi11sp_2\\0,1\P\C,1,1.85714943\
H,2,1.09756049,1,108.42976012\H,2,1.09670455,1,113.46651843,3,120.7378
6542,0\H,2,1.09563012,1,109.34143982,3,-117.43007264,0\C,1,1.85755677,
2,99.86380388,5,170.46358887,0\H,6,1.09747335,1,108.42229318,2,72.0588
3356,0\H,6,1.09568106,1,109.3359163,2,-170.49523314,0\H,6,1.09696957,1
,113.48013363,2,-48.68687015,0\C,1,2.77249106,2,114.81110705,6,-123.67
568457,0\N,10,1.3125545,1,144.98249018,2,57.87972286,0\C,11,1.38193015
,10,104.78412172,1,-0.08339932,0\C,12,1.37084469,11,110.87768644,10,0.
19483597,0\H,10,1.08328783,1,89.13867518,2,-122.0584356,0\H,12,1.08314
334,11,121.176432,10,-179.99968347,0\H,13,1.0803247,12,131.97585629,11
,179.84428772,0\N,10,1.37985703,1,31.95763909,2,57.94582078,0\\Version
=AM64L-G03RevD.01\State=1-A\HF=-644.2317649\MP2=-645.4464793\RMSD=3.87
4e-09\Thermal=0.\PG=C01 [X(C5H9N2P1)]\\@

131-Me$^+$
1\1\GINC-IBLIS\SP\RMP2-FC\6-31+G(2d,p)\C6H12N2P1(1+)\CHRISTOPH\17-Feb-
2012\0\\#p MP2(FC)/6-31+g(2d,p) scf=tight\\imi11mesp_1\\1,1\C\P,1,1.81
143299\H,2,2.4105933,1,135.4273582\H,1,1.09662841,2,109.82931377,3,60.
15757547,0\H,1,1.09565878,2,109.56620414,3,-179.60307928,0\H,1,1.09563
221,2,110.24985134,3,-59.77666327,0\C,2,1.8091781,1,110.04731847,6,178
.70793263,0\H,7,1.09654817,2,108.34619821,1,60.78088546,0\C,2,1.811423
23,1,110.06929547,7,121.44868833,0\C,2,2.76240954,1,96.25565161,7,-139
.59817092,0\C,10,1.36328078,2,135.15258101,1,124.33023703,0\N,11,1.385
75799,10,111.02780753,2,0.04615411,0\C,12,1.29570341,11,106.44551938,1
0,-0.00106549,0\H,11,1.0818854,10,127.82465932,2,-179.96194002,0\H,10,
1.08118249,2,92.72430698,1,-55.64127188,0\H,13,1.08214762,12,126.18960
576,11,179.99255701,0\H,9,1.09663113,2,109.83058657,1,-59.86865009,0\H
,9,1.09562276,2,110.25128341,1,60.06513524,0\H,7,1.09531275,2,110.6598
2636,1,179.80736762,0\H,7,1.09531509,2,110.66872889,1,-58.24292304,0\N
,13,1.40914784,12,111.70759361,11,0.0072926,0\\Version=AM64L-G03RevD.0
1\State=1-A\HF=-683.6509583\MP2=-685.0081643\RMSD=7.216e-09\Thermal=0.
\PG=C01 [X(C6H12N2P1)]\\@

132
1\1\GINC-CALYPSO\SP\RMP2-FC\6-31+G(2d,p)\C9H11N2P1\CHRISTOPH\29-Sep-20
09\0\\#p MP2(FC)/6-31+g(2d,p) scf=tight\\pyr2sp_9\\0,1\P\C,1,1.8484482
9\H,2,1.09592988,1,108.14542843\H,2,1.09595061,1,108.12876932,3,117.36
487377,0\H,2,1.09509151,1,114.79991304,4,121.30675444,0\C,1,2.73801292
,2,119.84169271,5,71.32534509,0\C,6,1.37272089,1,141.83204521,2,-46.67
840188,0\C,7,1.43183161,6,107.19657631,1,1.13178592,0\C,8,1.37381675,7
,107.52001167,6,0.0988514,0\C,1,2.85686532,2,83.61602414,9,-79.1206423
9,0\C,10,1.37382475,1,136.02978019,2,-142.90968963,0\C,11,1.43181676,1
0,107.52055064,1,1.20778513,0\C,12,1.37272892,11,107.19611531,10,-0.10

42518,0\H,13,1.08175059,12,131.25904028,11,179.79216166,0\H,12,1.08254
997,11,126.88840134,10,179.71098355,0\H,11,1.08307381,10,125.65373359,
1,-178.87121354,0\H,10,1.08046468,1,93.68498866,2,38.34311999,0\H,9,1.
08046268,8,130.27315612,7,-179.5094107,0\H,8,1.08307601,7,126.82360009
,6,-179.97713936,0\H,7,1.08254895,6,125.9144743,1,-179.0726408,0\H,6,1
.08175735,1,86.90443763,2,134.00050917,0\N,9,1.3912722,8,108.61299926,
7,0.16684469,0\N,10,1.39125575,1,27.43877741,22,120.11183651,0\\Versio
n=AM64L-G03RevD.01\State=1-A\HF=-796.8583994\MP2=-798.6333923\RMSD=4.5
84e-09\Thermal=0.\PG=C01 [X(C9H11N2P1)]\\@

132-Me⁺

1\1\GINC-CALYPSO\SP\RMP2-FC\6-31+G(2d,p)\C10H14N2P1(1+)\CHRISTOPH\29-S
ep-2009\0\\#p MP2(FC)/6-31+g(2d,p) scf=tight\\pyr2mesp_1\\1,1\P\C,1,1.
80837383\H,2,1.09572257,1,111.13030592\H,2,1.09640714,1,107.97407054,3
,-119.31459749,0\H,2,1.09494965,1,110.101357,4,-118.69035126,0\C,1,1.8
0837384,2,109.80243604,5,176.46804555,0\H,6,1.09494911,1,110.10149564,
2,176.46633312,0\H,6,1.09640656,1,107.97401524,2,-64.84318265,0\H,6,1.
09572271,1,111.13018145,2,54.47139848,0\C,1,2.72618167,2,133.04175673,
6,-129.10112481,0\C,10,1.36386756,1,137.08598223,2,33.25896867,0\C,11,
1.43771941,10,108.18227188,1,-9.67388874,0\C,12,1.36386649,11,108.1696
6209,10,-0.03946762,0\C,1,2.72617823,2,101.68070629,6,144.60774334,0\C
,14,1.36386737,1,137.08535983,2,-98.52731831,0\C,15,1.43771938,14,108.
18228223,1,-9.67783437,0\C,16,1.36386626,15,108.16969316,14,-0.0397176
3,0\H,11,1.0822642,10,125.34422211,1,171.53801377,0\H,10,1.08112877,1,
91.11426663,2,-152.55455779,0\H,13,1.08049384,12,130.95566419,11,-178.
81485448,0\H,12,1.08226027,11,126.51386881,10,-178.84210614,0\H,17,1.0
8049393,16,130.95580374,15,-178.81429039,0\H,16,1.08226023,15,126.5138
3983,14,-178.8415594,0\H,15,1.08226393,14,125.34425584,1,171.53455911,
0\H,14,1.08112744,1,91.11450623,2,75.65637229,0\N,10,1.41490208,1,30.5
0422762,2,13.12160819,0\N,14,1.41490192,1,30.50424697,26,133.21172068,
0\\Version=AM64L-G03RevD.01\State=1-A\HF=-836.2828051\MP2=-838.1976373
\RMSD=5.007e-09\Thermal=0.\PG=C01 [X(C10H14N2P1)]\\@

133

1\1\GINC-YIN\SP\RMP2-FC\6-31+G(2d,p)\C6H10N1P1\CHRISTOPH\29-Sep-2009\0
\\#p MP2(FC)/6-31+g(2d,p) scf=tight\\pyr1sp_3\\0,1\P\C,1,1.85875428\H,
2,1.09767155,1,108.58412914\H,2,1.09696645,1,113.40935928,3,120.831261
07,0\H,2,1.09570756,1,109.26153332,3,-117.54513078,0\C,1,1.85865294,2,
99.54043532,5,170.76333642,0\H,6,1.09762531,1,108.56825015,2,71.513971
78,0\H,6,1.09566666,1,109.29533209,2,-170.92247319,0\H,6,1.09700794,1,
113.40889855,2,-49.2820259,0\C,1,2.84769274,6,86.48301641,2,86.2307506
,0\C,10,1.37623457,1,136.98720923,6,130.78033981,0\C,11,1.43017926,10,
107.38412656,1,-0.05865932,0\C,12,1.37557506,11,107.14175227,10,0.0055
9759,0\H,11,1.08320449,10,125.72636482,1,179.98337447,0\H,10,1.0814438
,1,92.60879836,6,-49.22555816,0\H,13,1.08179366,12,131.25541397,11,179
.98647078,0\H,12,1.0826773,11,126.94621323,10,-179.99664497,0\N,10,1.3
8833441,1,28.32939588,6,130.67281999,0\\Version=AM64L-G03RevD.01\State
=1-A\HF=-628.2250801\MP2=-629.4122838\RMSD=7.684e-09\Thermal=0.\PG=C01
[X(C6H10N1P1)]\\@

133-Me⁺

1\1\GINC-YIN\SP\RMP2-FC\6-31+G(2d,p)\C7H13N1P1(1+)\CHRISTOPH\29-Sep-20
09\0\\#p MP2(FC)/6-31+g(2d,p) scf=tight\\pyr1mesp_14\\1,1\P\C,1,1.8106
9962\H,2,1.09498016,1,110.89447355\H,2,1.09633122,1,108.9612341,3,119.
19926692,0\H,2,1.09524625,1,109.90443035,4,119.68937306,0\C,1,1.810695
4,2,109.67059965,3,-176.00011705,0\H,6,1.09632444,1,108.96309434,2,56.
84652725,0\H,6,1.0949823,1,110.89159813,2,176.0434523,0\H,6,1.09524494
,1,109.90579506,2,-62.84924137,0\C,1,1.81460155,6,109.67346152,2,-120.

49326311,0\H,10,1.09667494,1,110.27571959,6,60.29739686,0\H,10,1.09572
163,1,109.62301748,6,-179.35233986,0\H,10,1.09571935,1,109.62877171,6,
-60.05720385,0\C,1,2.74504394,6,88.5448078,2,133.31581257,0\C,14,1.364
87123,1,136.79922082,6,-162.58594873,0\C,15,1.436044,14,108.06222665,1
,9.58095641,0\C,16,1.36487269,15,108.0622122,14,-0.00038853,0\H,15,1.0
8220934,14,125.3830754,1,-171.70632805,0\H,14,1.08080338,1,92.05196221
,6,23.72505393,0\H,17,1.08080853,16,130.83464513,15,178.76262021,0\H,1
6,1.08220907,15,126.54078682,14,178.69287284,0\N,17,1.41230318,16,108.
02017848,15,0.99202067,0\\Version=AM64L-G03RevD.01\State=1-A\HF=-667.6
560831\MP2=-668.9853186\RMSD=1.310e-09\Thermal=0.\PG=C01 [X(C7H13N1P1)
]\\@

134
1\1\GINC-MORITZ\SP\RMP2-FC\6-31+G(2d,p)\C4H10N1P1\CHRISTOPH\08-Oct-200
9\0\\#p MP2(FC)/6-31+g(2d,p) scf=tight\\az1sp_1\\0,1\P\C,1,1.85754014\
H,2,1.09646581,1,109.72533396\H,2,1.09733306,1,109.5134769,3,118.15101
884,0\H,2,1.09699723,1,111.39610723,4,121.19319351,0\C,1,1.85754285,2,
99.16878453,3,171.91294231,0\H,6,1.09733386,1,109.51372104,2,69.935106
36,0\H,6,1.09646514,1,109.72546578,2,-171.91372252,0\H,6,1.09699758,1,
111.39559824,2,-51.25809758,0\C,1,2.75802907,2,99.19396644,6,-127.7354
7008,0\C,10,1.48959501,1,74.33412955,2,147.81705193,0\H,10,1.09220224,
1,83.50485221,2,-91.51521736,0\H,10,1.08888994,1,138.61662757,2,29.367
5758,0\H,11,1.09220927,10,117.22217641,1,73.96022954,0\H,11,1.08889743
,10,120.38723633,1,-137.64114587,0\N,11,1.47667081,10,59.71204403,1,-3
4.98100284,0\\Version=IA32L-G03RevD.01\State=1-A\HF=-552.4487341\MP2=-
553.3669918\RMSD=7.665e-09\Thermal=0.\PG=C01 [X(C4H10N1P1)]\\@

134-Me$^+$
1\1\GINC-HAENSEL\SP\RMP2-FC\6-31+G(2d,p)\C5H13N1P1(1+)\CHRISTOPH\07-Oc
t-2009\0\\#p MP2(FC)/6-31+g(2d,p) scf=tight\\az1mesp_2\\1,1\H\H,1,4.15
877206\C,1,3.90847281,2,65.12442191\C,3,1.49167901,1,100.84750731,2,17
.52552644,0\H,3,1.08693519,1,93.29785996,4,121.29609818,0\H,3,1.089545
46,1,101.97651078,4,-121.7473546,0\H,4,1.08694066,3,119.89907173,1,100
.23074183,0\H,4,1.08955133,3,117.81433061,1,-109.88330875,0\C,4,3.6389
3659,3,101.73387587,1,-16.4815291,0\C,9,2.97206704,4,78.26252554,3,0.0
0796422,0\P,9,1.80939993,4,49.29519998,3,-21.9026372,0\C,11,1.82252662
,9,108.78949927,4,-100.49730475,0\H,12,1.09590338,11,110.66617569,9,-1
79.5354202,0\H,12,1.09591286,11,110.66103165,9,59.32414353,0\H,12,1.09
695813,11,109.51973805,9,-60.10420036,0\H,10,1.09586008,9,91.40044694,
4,-154.35350704,0\H,10,1.0957277,9,144.80540544,4,-27.71016478,0\H,9,1
.09586591,4,152.61225881,3,-69.5575745,0\H,9,1.09504847,4,95.93067479,
3,88.57794187,0\N,4,1.48111976,3,59.76857314,1,-1.39068521,0\\Version=
IA32L-G03RevD.01\State=1-A\HF=-591.8973733\MP2=-592.9563518\RMSD=3.826
e-09\Thermal=0.\PG=C01 [X(C5H13N1P1)]\\@

135
1\1\GINC-AZAZEL\SP\RMP2-FC\6-31+G(2d,p)\C5H11N2P1\CHRISTOPH\08-Oct-200
9\0\\#p MP2(FC)/6-31+g(2d,p) scf=tight\\az2sp_3\\0,1\P\C,1,1.85206557\
H,2,1.09637447,1,109.85246276\H,2,1.09564195,1,108.93101803,3,118.6621
8339,0\H,2,1.09672294,1,111.18142551,4,120.55415265,0\C,1,2.83516509,2
,91.15943669,4,-89.58970138,0\C,6,1.50040742,1,73.33693573,2,161.24955
679,0\H,6,1.08927799,1,87.78232728,2,-79.32557789,0\H,6,1.08956955,1,1
34.35824465,2,45.83737123,0\H,7,1.09103218,6,117.11924758,1,80.136117,
0\H,7,1.08974978,6,120.15352475,1,-132.42671341,0\C,1,2.77451417,2,120
.95365376,6,-95.33538771,0\C,12,1.4891459,1,73.81984104,2,-25.37001459
,0\H,12,1.08921681,1,138.97192438,2,92.14994079,0\H,12,1.09218595,1,83
.73041502,2,-146.31388108,0\H,13,1.09279758,12,117.12956114,1,-74.1695
273,0\H,13,1.0891184,12,120.61451216,1,137.84000264,0\N,7,1.46254189,6

,59.42133472,1,-28.86130349,0\N,13,1.47584596,12,59.73368994,1,34.7613
2053,0\\Version=AM64L-G03RevD.01\State=1-A\HF=-645.3099287\MP2=-646.54
6109\RMSD=4.591e-09\Thermal=0.\PG=C01 [X(C5H11N2P1)]\\@

135-Me$^+$

1\1\GINC-CALYPSO\SP\RMP2-FC\6-31+G(2d,p)\C6H14N2P1(1+)\CHRISTOPH\08-Oc
t-2009\0\\#p MP2(FC)/6-31+g(2d,p) scf=tight\\az2mesp_1\\1,1\H\C,1,3.39
353734\C,2,1.4907691,1,119.40578842\H,2,1.08686122,1,101.47955165,3,-1
33.96631144,0\H,2,1.08849941,1,71.52234821,3,111.92300707,0\H,3,1.0868
4747,2,119.9398598,1,-125.7128069,0\H,3,1.08875691,2,117.60672281,1,84
.02095522,0\C,3,3.9340595,2,94.21217417,1,44.42218817,0\C,8,1.49011886
,3,114.71104899,2,-33.05302107,0\H,8,1.08949155,3,89.86455307,2,-153.9
3374,0\H,8,1.08720923,3,90.95636281,2,90.55476372,0\H,9,1.08974755,8,1
17.84094365,3,-103.95536183,0\H,9,1.0870373,8,119.96893702,3,106.13772
631,0\C,9,3.49214197,8,105.49360054,3,-16.59193199,0\P,14,1.80435981,9
,53.5304508,8,-9.30644632,0\C,15,1.81417512,14,110.51253088,9,-102.141
68886,0\H,16,1.09570975,15,110.31424423,14,-175.19676915,0\H,16,1.0954
9091,15,109.15321666,14,-56.1288196,0\H,16,1.09619418,15,110.66972011,
14,63.87874189,0\H,14,1.09543838,9,69.43719499,8,-146.98957957,0\H,14,
1.09491587,9,157.05724676,8,-55.74056647,0\N,2,1.48299679,1,63.8356893
5,14,-0.3882767,0\N,8,1.4818719,3,54.9958367,2,-28.49226815,0\\Version
=AM64L-G03RevD.01\State=1-A\HF=-684.7639786\MP2=-686.1383775\RMSD=6.04
3e-09\Thermal=0.\PG=C01 [X(C6H14N2P1)]\\@

136

1\1\GINC-NODE5\SP\RMP2-FC\6-31+G(2d,p)\C4H12N1P1\ZIP07\11-Sep-2009\0\\
#p MP2(FC)/6-31+g(2d,p) scf=tight\\dma1sp_2\\0,1\P\C,1,1.86056926\H,2,
1.09843271,1,114.16543254\H,2,1.09779274,1,108.51114,3,120.49289876,0\
H,2,1.09567295,1,109.12367119,4,117.54174739,0\C,1,1.87123327,2,98.373
89008,5,-173.27583326,0\H,6,1.09837833,1,113.6519067,2,51.22606719,0\H
,6,1.09693739,1,109.66287744,2,173.10302126,0\H,6,1.0980279,1,108.8469
2073,2,-69.48143947,0\C,1,2.79504263,2,88.32688721,6,84.94522169,0\H,1
0,1.10542902,1,106.90648125,2,-132.51412684,0\H,10,1.09461415,1,84.796
52129,2,-25.07048217,0\H,10,1.09990363,1,137.00453713,2,85.26222961,0\
C,10,2.42827548,1,62.14130483,2,140.57691348,0\H,14,1.09462993,10,143.
27268441,1,-20.51843911,0\H,14,1.10027544,10,91.07529817,1,-144.008219
75,0\H,14,1.10590546,10,94.29462727,1,108.31959976,0\N,10,1.45647957,1
,31.3316948,2,121.10794991,0\\Version=AM64L-G03RevD.01\State=1-A\HF=-5
53.6484405\MP2=-554.5879049\RMSD=8.162e-09\Thermal=0.\PG=C01 [X(C4H12N
1P1)]\\@

136-Me$^+$

1\1\GINC-NODE6\SP\RMP2-FC\6-31+G(2d,p)\C5H15N1P1(1+)\ZIP07\11-Sep-2009
\0\\#p MP2(FC)/6-31+g(2d,p) scf=tight\\dma1mesp_1\\1,1\P\C,1,1.8144472
7\H,2,1.09448808,1,111.49512061\H,2,1.09616451,1,108.63264541,3,119.07
75433,0\H,2,1.09516123,1,109.89041723,4,119.26758051,0\C,1,1.8144454,2
,108.4490035,3,-171.33306903,0\H,6,1.0951599,1,109.88989739,2,-67.0103
8242,0\H,6,1.09616568,1,108.63313272,2,52.25754273,0\H,6,1.09448942,1,
111.4949383,2,171.33557542,0\C,1,1.82363277,6,107.85797814,2,-116.5595
8793,0\H,10,1.0967841,1,110.43419739,6,58.47334358,0\H,10,1.09607159,1
,110.06085464,6,178.53843807,0\H,10,1.09607106,1,110.06120862,6,-61.59
198033,0\C,1,2.71825442,6,135.63568786,10,-135.39139034,0\H,14,1.09274
615,1,84.95675406,6,-124.55774887,0\H,14,1.0946865,1,136.03836283,6,-1
4.30291473,0\H,14,1.09883503,1,106.01098076,6,126.70732053,0\C,14,2.46
4964,1,63.03636076,6,38.57002406,0\H,18,1.09883459,14,95.57668715,1,10
5.14781733,0\H,18,1.0946862,14,87.30448123,1,-146.34793177,0\H,18,1.09
275127,14,144.07279129,1,-29.51198834,0\N,18,1.4777498,14,33.48568563,
1,-17.34136294,0\\Version=AM64L-G03RevD.01\State=1-A\HF=-593.0978904\M

P2=-594.1817357\RMSD=4.289e-09\Thermal=0.\PG=C01 [X(C5H15N1P1)]\\@

137

1\1\GINC-MAX\SP\RMP2-FC\6-31+G(2d,p)\C9H18N3P1\CHRISTOPH\28-Nov-2009\0
\\#p MP2(FC)/6-31+g(2d,p) scf=tight\\nadsp_1\\0,1\C\C,1,1.54460892\C,2
,1.54845886,1,109.99530381\H,1,1.10005345,2,109.09847254,3,-179.183819
53,0\H,2,1.09988212,1,109.50384096,3,120.79671166,0\H,2,1.09712445,1,1
09.3803303,3,-121.54205746,0\H,3,1.09993472,2,109.73880452,1,178.82867
572,0\C,1,1.54823016,2,109.86518836,3,-58.86808745,0\H,8,1.09991093,1,
109.80929086,2,-61.71161809,0\H,8,1.09706794,1,110.56874873,2,179.7240
1152,0\C,3,1.54515573,2,109.88640306,1,58.89555666,0\H,11,1.09709018,3
,109.41806088,2,179.73191803,0\H,11,1.09983445,3,109.44538191,2,62.046
96761,0\C,8,1.5446748,1,109.97324059,2,58.87495508,0\H,14,1.09995003,8
,109.10877475,1,-179.27584455,0\P,3,2.71564379,2,91.10690854,1,-32.735
44247,0\N,3,1.47974195,2,111.51921812,1,-62.23724641,0\N,1,1.47980726,
2,109.08884155,3,63.77789325,0\N,14,1.48031002,8,109.0607834,1,63.7029
0657,0\C,18,1.46115583,1,114.70727695,2,159.06890064,0\H,20,1.09910675
,18,109.5405904,1,-60.5063909,0\H,20,1.10360643,18,114.03319971,1,60.8
4214899,0\H,20,1.09639594,18,109.05610054,1,-178.59562952,0\C,19,1.460
42503,14,114.84097699,8,158.16667442,0\H,24,1.09638374,19,109.09882039
,14,-178.97508253,0\H,24,1.09918648,19,109.57463092,14,-60.82428472,0\
H,24,1.10373295,19,113.99134433,14,60.49592697,0\C,17,1.46051522,3,114
.78069071,2,-79.80302877,0\H,28,1.09641174,17,109.09770224,3,-178.3377
4162,0\H,28,1.09909099,17,109.50905327,3,-60.23010503,0\H,28,1.1038862
9,17,114.05870842,3,61.07945187,0\\Version=IA32L-G03RevD.01\State=1-A\
HF=-855.4146041\MP2=-857.4423326\RMSD=3.178e-09\Thermal=0.\PG=C01 [X(C
9H18N3P1)]\\@

137-Me⁺

1\1\GINC-MAX\SP\RMP2-FC\6-31+G(2d,p)\C10H21N3P1(1+)\CHRISTOPH\28-Nov-2
009\0\\#p MP2(FC)/6-31+g(2d,p) scf=tight\\nadmesp_1\\1,1\C\C,1,1.54555
363\C,2,1.54720414,1,110.53271236\H,1,1.09539992,2,109.5687885,3,-179.
46812611,0\H,2,1.09742012,1,108.96306784,3,120.46274292,0\H,2,1.095994
93,1,109.71484092,3,-122.40917434,0\H,3,1.09527063,2,110.66404255,1,17
9.1870072,0\C,1,1.54803488,2,110.56344855,3,-57.24074135,0\H,8,1.09733
405,1,109.49888188,2,-62.47112906,0\H,8,1.0960147,1,110.94912593,2,179
.28676535,0\C,3,1.54707941,2,110.54192454,1,57.35991329,0\H,11,1.09571
764,3,109.97346201,2,-179.94918551,0\H,11,1.09711095,3,108.94777026,2,
62.59302954,0\C,8,1.54515064,1,110.42299319,2,57.37419377,0\H,14,1.095
53076,8,109.54833915,1,-179.80099377,0\N,3,1.5100874,2,110.13674426,1,
-63.87230628,0\N,1,1.50809495,2,109.56728742,3,64.4080965,0\N,14,1.509
64528,8,109.5656549,1,64.26059566,0\C,17,1.476084,1,115.2108666,2,160.
37071971,0\H,19,1.09452624,17,108.02288571,1,-54.53505217,0\H,19,1.098
35403,17,113.3359633,1,66.39039483,0\H,19,1.09421293,17,109.69043343,1
,-171.47372604,0\C,18,1.47627728,14,115.13425392,8,160.2298228,0\H,23,
1.09413267,18,109.70423882,14,-173.03851597,0\H,23,1.09442895,18,107.9
8371855,14,-56.11335424,0\H,23,1.09833078,18,113.37157457,14,64.833402
34,0\C,16,1.47538226,3,115.1290958,2,-79.09318868,0\H,27,1.09409107,16
,109.80231284,3,-174.55102405,0\H,27,1.09451956,16,108.01177821,3,-57.
49634593,0\H,27,1.09832003,16,113.27007074,3,63.42105935,0\P,16,1.6647
1191,3,109.05223438,2,60.15387405,0\C,31,1.79885604,16,111.98732235,3,
-179.49428278,0\H,32,1.09503515,31,109.80028837,16,73.64409648,0\H,32,
1.09498898,31,109.71397861,16,-166.39072725,0\H,32,1.09490245,31,109.7
3263446,16,-46.42339582,0\\Version=IA32L-G03RevD.01\State=1-A\HF=-894.
8661981\MP2=-897.0380028\RMSD=2.855e-09\Thermal=0.\PG=C01 [X(C10H21N3P
1)]\\@

138

1\1\GINC-CIPCLU10\SP\RMP2-FC\6-31+G(2d,p)\C5H14N1P1\C2175\12-May-2010\
0\\#p MP2(FC)/6-31+G(2d,p) scf=tight\\nem1sp_7\\0,1\P\C,1,1.86319825\H
,2,1.09788624,1,108.64863752\H,2,1.09843584,1,114.02312291,3,120.49258
354,0\H,2,1.09574429,1,109.18726044,3,-117.63211613,0\C,1,1.86997455,2
,98.06012863,5,173.60520097,0\H,6,1.09679325,1,109.71626802,2,-174.446
73696,0\H,6,1.09843465,1,113.54603772,2,-52.63233778,0\H,6,1.09798519,
1,108.8451804,2,67.92128056,0\C,1,2.79764532,2,88.76964621,6,-85.06475
842,0\H,10,1.09531747,1,85.26523049,2,22.80370515,0\H,10,1.10335701,1,
106.82258899,2,129.98255744,0\H,10,1.09981931,1,136.99842785,2,-87.735
37173,0\C,10,2.46610839,1,61.97247741,2,-136.08973599,0\H,14,1.0966033
4,10,139.02012461,1,30.79181984,0\H,14,1.10194911,10,87.4908194,1,143.
99594029,0\C,14,1.53922375,10,101.08802048,1,-106.84543305,0\H,17,1.09
801688,14,110.41220456,10,-148.41310123,0\H,17,1.09763349,14,110.73530
917,10,91.99208312,0\H,17,1.09720881,14,111.72353217,10,-28.61937149,0
\N,10,1.45676895,1,31.01579942,2,-121.60773546,0\\Version=AM64L-G03Rev
D.01\State=1-A\HF=-592.6881652\MP2=-593.7818746\RMSD=8.529e-09\Thermal
=0.\PG=C01 [X(C5H14N1P1)]\\@

138-Me$^+$

1\1\GINC-CIPCLU09\SP\RMP2-FC\6-31+G(2d,p)\C6H17N1P1(1+)\C2175\14-May-2
010\0\\#p MP2(FC)/6-31+G(2d,p) scf=tight\\nem1mesp_3\\1,1\C\H,1,1.0955
0564\H,1,1.09616307,2,108.6930687\H,1,1.09676202,2,109.07789346,3,-118
.17521121,0\C,1,2.92866679,2,146.385316,3,124.81590773,0\H,5,1.0950457
4,1,146.15022287,2,-6.53689338,0\C,5,2.93936636,1,60.16742607,2,30.789
95085,0\H,7,1.09504882,5,91.92110946,1,-161.41318633,0\H,7,1.09459139,
5,147.03303292,1,-29.41829472,0\H,7,1.09615724,5,86.58251551,1,89.8427
9588,0\P,7,1.81284169,5,35.99166107,1,-38.04924467,0\C,11,2.75527279,7
,84.84726359,5,-131.42460349,0\H,12,1.0964351,11,132.57350071,7,121.15
509875,0\H,12,1.09442923,11,85.16232879,7,13.64909247,0\C,12,2.4993571
1,11,61.6212931,7,167.59992914,0\H,15,1.09725016,12,97.4500688,11,107.
35554648,0\H,15,1.09493692,12,87.40161375,11,-143.89116853,0\H,15,1.09
393353,12,142.9096359,11,-27.86674507,0\C,12,1.53260681,11,107.7956022
8,7,-96.54750241,0\H,19,1.09607148,12,109.49442432,11,151.42127867,0\H
,19,1.09790683,12,111.85978658,11,32.36531697,0\H,19,1.09565899,12,111
.95589545,11,-89.42875639,0\H,5,1.09470418,1,90.49042021,11,-126.98139
335,0\H,5,1.09635344,1,89.10913266,11,124.92065968,0\N,15,1.47717586,1
2,32.67986483,11,-13.06445959,0\\Version=AM64L-G03RevD.01\State=1-A\HF
=-632.1387414\MP2=-633.3771252\RMSD=7.859e-09\Thermal=0.\PG=C01 [X(C6H
17N1P1)]\\@

139

1\1\GINC-CIP-D-16\SP\RMP2-FC\6-31+G(2d,p)\C7H16N1P1\C2175\25-Mar-2010\
0\\#P MP2(FC)/6-31+G(2D,P) SCF=TIGHT\\dpia1sp_4\\0,1\P\C,1,1.86072301\
H,2,1.09571424,1,109.08265375\H,2,1.09774352,1,108.54949757,3,117.5579
704,0\H,2,1.09822424,1,114.15079805,4,120.54407171,0\C,1,1.87137302,2,
98.46123529,3,173.12440732,0\H,6,1.09693743,1,109.59941597,2,-173.7391
7216,0\H,6,1.09808654,1,108.77042447,2,68.93587868,0\H,6,1.09820572,1,
113.85371745,2,-51.82437542,0\C,1,4.01015817,2,112.16966448,6,-126.488
62821,0\C,10,1.53775023,1,26.45566685,2,171.22776226,0\C,11,2.43960514
,10,91.67696273,1,-53.47845391,0\C,12,1.53853904,11,91.57737714,10,-0.
12639337,0\C,13,1.53830846,12,110.85106535,11,-27.07905567,0\H,10,1.09
921974,1,87.83356973,2,-46.17269944,0\H,10,1.09899285,1,133.26004394,2
,-158.40195401,0\H,11,1.10878697,10,108.81690561,1,-147.3010037,0\H,11
,1.09592922,10,110.59536374,1,95.11253979,0\H,12,1.09578875,11,143.107
74889,10,126.6511699,0\H,12,1.1081023,11,92.9707088,10,-109.35396805,0
\H,13,1.09917213,12,108.58388093,11,93.45111687,0\H,13,1.09911653,12,1
09.49066634,11,-149.44070617,0\H,14,1.09829919,13,110.57071523,12,175.

90904538,0\H,14,1.10170735,13,109.17059022,12,-66.95839477,0\N,12,1.46
561838,11,33.77524196,10,126.54902724,0\\Version=x86-Linux-G03RevB.03\
State=1-A\HF=-669.6074955\MP2=-670.9875426\RMSD=4.067e-09\PG=C01 [X(C7
H16N1P1)]\\@

139-Me$^+$

1\1\GINC-CIPCLU09\SP\RMP2-FC\6-31+G(2d,p)\C8H19N1P1(1+)\C2175\25-Mar-2
010\0\\#p MP2(FC)/6-31+G(2d,p) scf=tight\\dpia1mesp_1\\1,1\P\C,1,1.815
59754\H,2,1.09504129,1,109.88481686\H,2,1.09623867,1,108.63862246,3,11
9.30117407,0\H,2,1.09421622,1,111.51938708,4,119.07530716,0\C,1,1.8249
467,2,107.60770101,5,53.98026807,0\H,6,1.09606694,1,109.96658195,2,-62
.04876022,0\H,6,1.09665421,1,110.56235176,2,58.12896137,0\H,6,1.096072
58,1,109.96680875,2,178.30764684,0\C,1,1.81559564,2,108.07351067,6,115
.94205146,0\H,10,1.09623414,1,108.63464872,2,-50.83227273,0\H,10,1.095
04477,1,109.8886657,2,68.46570361,0\H,10,1.09420932,1,111.51964764,2,-
169.90501196,0\C,1,3.99214135,10,91.08534877,6,119.71956549,0\C,14,1.5
3507121,1,27.48836489,10,-83.07691834,0\C,15,2.48331001,14,90.75244064
,1,-48.71979382,0\C,16,1.53506846,15,90.753984,14,0.00066799,0\C,17,1.
53693558,16,111.19773264,15,-27.6542324,0\H,14,1.09838423,1,88.7738982
9,10,54.8165938,0\H,14,1.09704165,1,133.9526993,10,-58.4197242,0\H,15,
1.10207153,14,110.17864676,1,-141.35008935,0\H,15,1.09445545,14,109.94
014066,1,99.84149361,0\H,16,1.0944777,15,142.88965343,14,125.64486448,
0\H,16,1.10207006,15,92.20672197,14,-110.22787097,0\H,17,1.09838389,16
,108.86792125,15,93.82585762,0\H,17,1.09704221,16,108.28332239,15,-149
.71987379,0\H,18,1.10020331,17,109.62029824,16,-66.46142344,0\H,18,1.0
9590255,17,110.19523189,16,176.31407711,0\N,16,1.49177907,15,33.662101
88,14,126.96514101,0\\Version=AM64L-G03RevD.01\State=1-A\HF=-709.06078
51\MP2=-710.5848741\RMSD=7.574e-09\Thermal=0.\PG=C01 [X(C8H19N1P1)]\\@

140

1\1\GINC-CIPCLU10\SP\RMP2-FC\6-31+G(2d,p)\C5H12N1P1\C2175\12-May-2010\
0\\#p MP2(FC)/6-31+G(2d,p) scf=tight\\nv1sp_4\\0,1\P\C,1,1.87258134\H,
2,1.09716674,1,109.7222951\H,2,1.09795847,1,108.89607351,3,117.3685959
,0\H,2,1.09845012,1,113.61660679,4,120.6748938,0\C,1,1.85949122,2,98.4
0990688,3,171.26181277,0\H,6,1.09561893,1,109.24171097,2,-175.47722863
,0\H,6,1.09852684,1,112.69220454,2,-54.49997014,0\H,6,1.09729907,1,109
.46903725,2,66.16688328,0\C,1,2.82920559,6,124.1139269,2,114.9532862,0
\C,10,1.55366616,1,116.48799001,6,-42.69192312,0\C,11,1.55255677,10,87
.02106002,1,-1.32570473,0\H,10,1.10230056,1,91.71201557,6,-159.2667892
3,0\H,10,1.09756796,1,106.88261378,6,90.71494345,0\H,11,1.09369935,10,
116.58449057,1,-119.63603013,0\H,11,1.09396615,10,112.92448508,1,112.2
9130713,0\H,12,1.09792814,11,117.12463744,10,129.86201559,0\H,12,1.102
31579,11,112.96393653,10,-102.70206161,0\N,10,1.47990142,1,29.99580678
,6,-18.61601117,0\\Version=AM64L-G03RevD.01\State=1-A\HF=-591.494742\M
P2=-592.5626262\RMSD=4.491e-09\Thermal=0.\PG=C01 [X(C5H12N1P1)]\\@

140-Me$^+$

1\1\GINC-CIPCLU10\SP\RMP2-FC\6-31+G(2d,p)\C6H15N1P1(1+)\C2175\12-May-2
010\0\\#p MP2(FC)/6-31+G(2d,p) scf=tight\\nv1mesp_1\\1,1\H\C,1,3.10635
731\H,2,1.09674028,1,69.83554269\C,2,3.74429775,1,80.67804983,3,177.75
008666,0\C,4,1.55292093,2,113.27873919,1,140.46878298,0\C,5,1.55292021
,4,88.47897024,2,-30.8377573,0\H,4,1.09421102,2,124.74547904,1,-13.576
4973,0\H,4,1.09773371,2,67.02380079,1,-112.37991179,0\H,5,1.09212037,4
,113.08007779,2,-145.35457056,0\H,5,1.09145667,4,115.67768099,2,87.079
24459,0\H,6,1.09773371,5,113.80760712,4,104.89696847,0\H,6,1.09421102,
5,116.96408969,4,-125.36602896,0\C,2,2.94249087,1,20.64667364,4,5.0127
5493,0\P,13,1.81195723,2,36.10898371,1,128.47107675,0\C,14,1.81195713,
13,110.61757643,2,-118.09458898,0\H,15,1.0959957,14,110.15491945,13,-1

78.72735638,0\H,15,1.09589452,14,109.88636233,13,61.77527899,0\H,15,1.
09489912,14,109.72775176,13,-58.58703794,0\H,2,1.09604752,1,153.847431
57,13,85.75227507,0\H,2,1.09604729,1,95.88439388,13,-75.00411115,0\H,1
3,1.09599639,2,91.64736983,1,-108.47121303,0\H,13,1.09489832,2,145.741
90697,1,122.91473994,0\N,4,1.49900117,2,47.00674144,1,76.53998496,0\\V
ersion=AM64L-G03RevD.01\State=1-A\HF=-630.9487983\MP2=-632.1593438\RMS
D=3.998e-09\Thermal=0.\PG=C01 [X(C6H15N1P1)]\\@

141

1\1\GINC-NAUTILUS\SP\RMP2-FC\6-31+G(2d,p)\C6H12N3P1\CHRISTOPH\12-Oct-2
009\0\\#p MP2(FC)/6-31+g(2d,p) scf=tight\\az3sp_61\\0,1\P\C,1,2.783464
29\C,2,1.49598417,1,77.28872173\H,2,1.09088046,1,86.12696661,3,118.803
76082,0\H,2,1.08961135,1,133.34404405,3,-119.72903356,0\H,3,1.0888463,
2,117.14914065,1,78.30800687,0\H,3,1.09005903,2,119.81814627,1,-132.77
375216,0\C,1,2.85687349,2,103.22506318,3,-41.44076528,0\C,8,1.49600435
,1,71.8647572,2,-119.81894112,0\H,8,1.08875386,1,88.45108664,9,-119.27
2178,0\H,8,1.08995661,1,135.51001154,9,114.39381086,0\H,9,1.08964053,8
,120.18197144,1,133.02935973,0\H,9,1.09086059,8,116.96864437,1,-78.955
806,0\C,1,2.8544426,9,102.92010294,8,-41.9411653,0\C,14,1.49592643,1,7
1.96130731,9,-120.2002009,0\H,14,1.08838012,1,88.24809632,9,120.444222
,0\H,14,1.09000216,1,135.61689732,9,-5.7524627,0\H,15,1.08963554,14,12
0.16892279,1,133.13924039,0\H,15,1.090868,14,117.0050581,1,-78.8299471
,0\N,2,1.46628678,1,32.47503343,9,-123.2755864,0\N,15,1.46648951,14,59
.54970264,1,29.52600373,0\N,9,1.46628454,8,59.55097297,1,29.4092587,0\
\Version=AM64L-G03RevD.01\State=1-A\HF=-738.1732036\MP2=-739.7272703\R
MSD=2.294e-09\Thermal=0.\PG=C01 [X(C6H12N3P1)]\\@

141-Me$^+$

1\1\GINC-YIN\SP\RMP2-FC\6-31+G(2d,p)\C7H15N3P1(1+)\CHRISTOPH\08-Oct-20
09\0\\#p MP2(FC)/6-31+g(2d,p) scf=tight\\az3mesp_1\\1,1\C\C,1,1.487304
04\H,1,1.08874015,2,117.46651013\H,1,1.08683356,2,119.85034403,3,-150.
76337302,0\H,2,1.08846268,1,117.5971398,4,-150.61541542,0\H,2,1.087022
25,1,119.8257268,4,-0.24807323,0\C,2,4.64778589,1,85.7852235,4,161.189
24718,0\C,7,1.48737117,2,48.20565262,1,156.91804091,0\H,7,1.08685656,2
,120.50314468,1,52.55051029,0\H,7,1.08874205,2,117.45647417,1,-99.5878
2728,0\H,8,1.08708549,7,119.84435749,2,-105.98727838,0\H,8,1.08834286,
7,117.58232396,2,103.67380186,0\C,2,4.36084361,1,96.83966134,8,-69.778
14137,0\C,13,1.48733361,2,59.55877213,1,158.16911985,0\H,13,1.08867787
,2,70.04294503,1,-60.27434511,0\H,13,1.08685053,2,169.54724057,1,68.43
342376,0\H,14,1.08848544,13,117.61821084,2,41.59739131,0\H,14,1.087083
24,13,119.83258584,2,-168.08727818,0\P,1,2.78179217,2,75.21626997,8,38
.57670982,0\C,19,1.79957457,1,97.6836932,2,169.29218573,0\H,20,1.09471
377,19,109.63285516,1,160.26916346,0\H,20,1.09482025,19,109.66226224,1
,-79.74582284,0\H,20,1.09475151,19,109.64985553,1,40.26414466,0\N,14,1
.48695127,13,60.02405256,2,-66.30900914,0\N,8,1.48682746,7,60.03219623
,2,-4.17785745,0\N,2,1.48689337,1,60.03222013,19,-27.66626359,0\\Versi
on=AM64L-G03RevD.01\State=1-A\HF=-777.6340935\MP2=-779.3251034\RMSD=6.
708e-09\Thermal=0.\PG=C01 [X(C7H15N3P1)]\\@

142

1\1\GINC-CIPCLU04\SP\RMP2-FC\6-31+G(2d,p)\C6H16N1P1\C2175\09-Mar-2010\
0\\#p MP2(FC)/6-31+G(2d,p) scf=tight\\dea1sp_5\\0,1\P\C,1,1.87054213\H
,2,1.09830043,1,108.84688726\H,2,1.09651683,1,109.40755979,3,117.55743
993,0\H,2,1.09878188,1,113.93927676,3,-120.64848966,0\C,1,1.86343843,2
,97.94933197,4,-173.90552487,0\H,6,1.09785897,1,108.84284161,2,-66.716
06488,0\H,6,1.09866551,1,113.52126387,2,53.71799107,0\H,6,1.09606467,1
,109.41195161,2,175.45133255,0\C,1,2.82137256,6,86.10698949,2,-88.3043
7677,0\H,10,1.09677758,1,79.56022267,6,58.91907841,0\H,10,1.1018264,1,

124.57611827,6,160.94868712,0\C,10,2.48531506,1,61.46852964,6,-138.958
53072,0\H,13,1.10034954,10,84.55879611,1,146.86833274,0\H,13,1.0968100
2,10,136.994455,1,38.44917655,0\C,10,1.53824144,1,120.81652115,6,-47.2
2531553,0\H,16,1.09838122,10,110.6180644,1,149.15122854,0\H,16,1.09693
053,10,111.30267355,1,-91.06245032,0\H,16,1.09622017,10,110.70461032,1
,28.85711018,0\C,13,1.53905268,10,105.37289899,1,-104.38767634,0\H,20,
1.09726109,13,110.56457041,10,95.47178521,0\H,20,1.0982931,13,110.6702
735,10,-144.77716482,0\H,20,1.09748603,13,111.58006331,10,-24.79165937
,0\N,10,1.46434993,1,29.9436582,6,-130.84909887,0\\Version=AM64L-G03Re
vD.01\State=1-A\HF=-631.7271026\MP2=-632.9753449\RMSD=3.270e-09\Therma
l=0.\PG=C01 [X(C6H16N1P1)]\\@

142-Me$^+$

1\1\GINC-CIPCLU05\SP\RMP2-FC\6-31+G(2d,p)\C7H19N1P1(1+)\C2175\09-Mar-2
010\0\\#p MP2(FC)/6-31+G(2d,p) scf=tight\\dea1mesp_5\\1,1\C\H,1,1.0946
8173\C,1,2.93914626,2,147.19650061\H,3,1.09496247,1,89.31808079,2,132.
57474536,0\H,3,1.09629617,1,90.06167256,2,-118.04018858,0\H,3,1.095187
61,1,146.93833321,2,7.26095723,0\C,1,3.162654,3,86.17047518,4,85.01277
449,0\H,7,1.095328,1,144.33030693,3,-67.45334683,0\H,7,1.09481949,1,53
.8745928,3,-131.553128,0\C,7,2.52116663,1,95.66636418,3,13.63617118,0\
H,10,1.09773163,7,136.74706089,1,36.9875995,0\H,10,1.09705782,7,82.088
7098,1,141.56729678,0\C,7,1.53245568,1,104.72081093,3,123.15382761,0\H
,13,1.09614047,7,111.68218751,1,-122.97801622,0\H,13,1.0961502,7,109.7
5516791,1,117.64371765,0\H,13,1.09785195,7,111.84274114,1,-1.57241553,
0\C,10,1.53097299,7,106.09983368,1,-108.9052926,0\H,17,1.09622185,10,1
09.69618333,7,-144.21247163,0\H,17,1.09576812,10,111.53418212,7,-24.97
525345,0\H,17,1.09580879,10,111.33083615,7,95.91773951,0\P,1,1.8134171
9,3,36.00270633,10,30.57442981,0\C,21,1.82444758,1,107.80048787,3,115.
40529415,0\H,22,1.09665682,21,110.37898011,1,-59.79518467,0\H,22,1.095
47053,21,109.94772195,1,60.64372661,0\H,22,1.09595767,21,110.14533898,
1,-179.68094783,0\H,1,1.09619511,21,108.32840298,3,-57.07954994,0\H,1,
1.09507249,21,110.41345661,3,61.98242683,0\N,7,1.48720946,1,63.5013411
5,21,-13.73274967,0\\Version=AM64L-G03RevD.01\State=1-A\HF=-671.180282
\MP2=-672.5732142\RMSD=6.095e-09\Thermal=0.\PG=C01 [X(C7H19N1P1)]\\@

143

1\1\GINC-MORITZ\SP\RMP2-FC\6-31+G(2d,p)\C14H30N1P1\CHRISTOPH\07-Jan-20
10\0\\#p MP2(FC)/6-31+g(2d,p) scf=tight\\yy1sp_2\\0,1\P\C,1,2.74634993
\C,2,1.60311242,1,138.81943364\C,3,1.59014827,2,103.8739179,1,12.14333
151,0\C,4,1.58512651,3,103.9233685,2,-30.07850641,0\C,5,1.55646682,4,1
13.57477654,3,-84.63367651,0\H,6,1.09159348,5,113.04252228,4,59.898598
53,0\H,6,1.0938032,5,109.47377641,4,179.26744081,0\H,6,1.09706965,5,11
1.48934714,4,-62.36342187,0\C,5,1.54589274,4,111.73503617,3,156.111463
55,0\H,10,1.09558689,5,110.93160775,4,173.426164,0\H,10,1.09182104,5,1
12.70026493,4,-65.08974783,0\H,10,1.09787337,5,109.69882558,4,55.72201
666,0\C,4,1.54418763,3,113.33203248,2,-153.15548087,0\H,14,1.09829186,
4,110.29725844,3,-63.07125512,0\H,14,1.09304084,4,113.68557493,3,57.96
550863,0\H,14,1.09837557,4,110.34187735,3,179.06920257,0\C,4,1.5518873
8,3,110.91013895,2,87.89284059,0\H,18,1.09525631,4,112.56196528,3,-60.
14029362,0\H,18,1.09481274,4,110.66929382,3,61.16999677,0\H,18,1.09539
315,4,110.61027031,3,179.12070358,0\C,3,1.55577872,2,112.87577299,1,-1
09.76480864,0\H,22,1.0961822,3,109.74836435,2,-177.74569876,0\H,22,1.0
9560323,3,110.25195772,2,-60.42346991,0\H,22,1.09157684,3,114.99216769
,2,61.16329706,0\C,3,1.55152255,2,112.52805837,1,134.05622974,0\H,26,1
.09832803,3,109.87959247,2,-176.2533766,0\H,26,1.09160129,3,114.270422
3,2,-55.09165078,0\H,26,1.09774039,3,110.18261047,2,66.15679661,0\C,2,
1.54985437,1,89.26829662,5,132.28051028,0\H,30,1.09588401,2,109.244650
39,1,33.4877858,0\H,30,1.09658792,2,111.38731247,1,152.14582898,0\H,30

,1.09477695,2,112.19026655,1,-85.9545324,0\C,2,1.54937081,1,91.5263606
,5,-121.36748414,0\H,34,1.09463683,2,112.29717783,1,86.74740924,0\H,34
,1.09798936,2,111.41663769,1,-151.47399969,0\H,34,1.09631389,2,109.159
08957,1,-32.85178123,0\C,1,1.86950274,2,122.43987323,34,167.35110223,0
\H,38,1.09642084,1,109.11700558,2,-28.21526735,0\H,38,1.09868312,1,107
.06180551,2,-144.2974124,0\H,38,1.09542558,1,115.63469786,2,95.0117345
8,0\C,1,1.86672977,38,98.4964297,10,-80.64073258,0\H,42,1.09855573,1,1
06.75545914,38,-68.3874749,0\H,42,1.09689345,1,115.36104256,38,51.3868
5622,0\H,42,1.09588025,1,109.9025783,38,175.18691501,0\N,5,1.48523345,
4,102.19395115,3,35.72513858,0\\Version=IA32L-G03RevD.01\State=1-A\HF=
-942.8244595\MP2=-945.3371887\RMSD=4.089e-09\Thermal=0.\PG=C01 [X(C14H
30N1P1)]\\@

143-Me$^+$

1\1\GINC-GRETEL\SP\RMP2-FC\6-31+G(2d,p)\C15H33N1P1(1+)\CHRISTOPH\07-Ja
n-2010\0\\#p MP2(FC)/6-31+g(2d,p) scf=tight\\yy1mesp_1\\1,1\C\C,1,1.58
938183\C,2,1.58687841,1,104.62516558\C,3,1.59338507,2,104.85766012,1,3
4.24979588,0\C,4,1.54577646,3,110.8884529,2,-146.72080013,0\H,5,1.0936
909,4,112.4574558,3,69.63238328,0\H,5,1.09184523,4,112.32928753,3,-167
.12256407,0\H,5,1.09525495,4,109.02940778,3,-50.21836952,0\C,4,1.54858
806,3,113.65494904,2,92.80803307,0\H,9,1.09720618,4,110.69548589,3,-17
6.95836749,0\H,9,1.09187494,4,113.44816885,3,-55.56787232,0\H,9,1.0945
2455,4,110.36591026,3,65.90280548,0\C,3,1.55669614,2,111.85790467,1,-8
7.16291934,0\H,13,1.09406559,3,110.69223631,2,-176.65237639,0\H,13,1.0
9266144,3,114.71877066,2,61.78553465,0\H,13,1.093587,3,109.78372298,2,
-59.6937654,0\C,3,1.54884553,2,112.9495391,1,155.61754267,0\H,17,1.091
66587,3,114.31359778,2,-59.42536615,0\H,17,1.09673082,3,109.71592616,2
,61.66114042,0\H,17,1.09743057,3,110.27836477,2,178.97751504,0\C,2,1.5
4817619,1,111.54656475,3,-122.50100665,0\H,21,1.09670623,2,109.7577726
5,1,179.53356011,0\H,21,1.09763899,2,110.30112927,1,-63.11795727,0\H,2
1,1.09147355,2,114.23304284,1,58.44915072,0\C,2,1.55501578,1,111.62044
142,3,121.01223865,0\H,25,1.09345442,2,109.90969612,1,-176.78462191,0\
H,25,1.09395601,2,114.50041901,1,-55.46756475,0\H,25,1.09444119,2,110.
60388278,1,66.09496259,0\C,1,1.54635846,2,111.02261105,21,88.44242224,
0\H,29,1.09562759,1,112.48931027,2,-174.60415389,0\H,29,1.09592154,1,1
08.77782971,2,-57.95651233,0\H,29,1.09239646,1,113.17514117,2,61.50561
756,0\C,1,1.54806382,29,106.66920068,2,124.53400001,0\H,33,1.09469358,
1,110.38172316,29,-59.88940905,0\H,33,1.09348174,1,110.72980425,29,57.
83712926,0\H,33,1.09086965,1,113.10756636,29,178.69266205,0\C,29,3.398
95489,1,90.75911104,33,-119.0770291,0\H,37,1.0929852,29,49.70768764,1,
-179.31671437,0\H,37,1.09671548,29,144.44978191,1,118.19398507,0\H,37,
1.09501145,29,105.41195551,1,-77.39295757,0\C,37,2.9224807,29,66.87290
358,1,68.32295118,0\H,41,1.09677622,37,84.9741134,29,152.14731978,0\H,
41,1.0943496,37,146.6702363,29,-90.71245219,0\H,41,1.09118486,37,93.66
878559,29,44.23118553,0\P,41,1.82728838,37,37.06473679,29,-80.63987114
,0\C,45,1.82494245,41,101.882399,37,-108.7424753,0\H,46,1.09677968,45,
105.97282405,41,52.71144165,0\H,46,1.091707,45,112.92177004,41,170.834
81048,0\H,46,1.0928866,45,111.10613299,41,-65.18979479,0\N,4,1.5324342
2,3,102.94960164,2,-26.46149824,0\\Version=IA32L-G03RevD.01\State=1-A\
HF=-982.2762634\MP2=-984.9352571\RMSD=6.638e-09\Thermal=0.\PG=C01 [X(C
15H33N1P1)]\\@

144

1\1\GINC-CIPCLU08\SP\RMP2-FC\6-31+G(2d,p)\C7H18N1P1\C2175\29-Jun-2010\
0\\#p MP2(FC)/6-31+G(2d,p) scf=tight\\na51sp_21\\0,1\P\C,1,1.86178588\
H,2,1.09845447,1,113.99817793\H,2,1.09799289,1,108.55183618,3,120.5322
4251,0\H,2,1.09572975,1,109.25714971,4,117.66500311,0\C,1,1.87076375,2
,98.15306555,5,-174.43037984,0\H,6,1.0984372,1,113.87725445,2,52.35307

919,0\H,6,1.09821505,1,108.72837502,2,-68.28683331,0\H,6,1.09669981,1,
109.57753053,2,174.24230537,0\C,1,2.80996321,2,88.45171142,6,86.082550
51,0\H,10,1.1039029,1,112.47748881,2,-142.58384278,0\H,10,1.09675222,1
,78.51039583,2,-39.70356705,0\C,10,2.49152357,1,61.17204308,2,138.1001
9333,0\H,13,1.09641893,10,138.23860901,1,-31.70274211,0\H,13,1.0995278
8,10,88.42922217,1,-144.95087323,0\C,13,1.53948363,10,101.65568749,1,1
05.58741962,0\H,16,1.09706168,13,111.92104062,10,28.94468911,0\H,16,1.
0980312,13,110.33292292,10,148.70491475,0\H,16,1.09754031,13,110.73452
334,10,-91.76119242,0\C,10,1.54333247,1,134.33838224,2,64.35743136,0\H
,20,1.09912139,10,110.02252473,1,94.16810039,0\H,20,1.10014577,10,107.
26517384,1,-151.19104838,0\C,20,1.53365317,10,113.70624275,1,-29.37169
332,0\H,23,1.0984504,20,111.0563903,10,-59.2347763,0\H,23,1.09603048,2
0,110.8413764,10,60.34505751,0\H,23,1.0970554,20,111.1263311,10,-179.2
2042133,0\N,10,1.46634807,1,30.85808941,2,123.41354959,0\\Version=AM64
L-G03RevD.01\State=1-A\HF=-670.7647424\MP2=-672.1683672\RMSD=4.042e-09
\Thermal=0.\PG=C01 [X(C7H18N1P1)]\\@

144-Me$^+$

1\1\GINC-CIPCLU05\SP\RMP2-FC\6-31+G(2d,p)\C8H21N1P1(1+)\C2175\28-Jun-2
010\0\\#p MP2(FC)/6-31+G(2d,p) scf=tight\\na51mesp_2\\1,1\C\H,1,1.0962
8719\C,1,2.92726366,2,87.10518845\H,3,1.0960517,1,91.9258177,2,-108.89
578372,0\C,3,3.85930955,1,74.49640294,2,147.81266441,0\H,5,1.09601856,
3,102.08479142,1,-73.31115894,0\H,5,1.09626474,3,143.64283086,1,66.298
18033,0\C,5,2.52215847,3,63.83641775,1,61.78768031,0\H,8,1.09773442,5,
136.77472887,3,-0.48663113,0\H,8,1.09702324,5,82.13901027,3,104.251607
68,0\C,8,1.53097174,5,106.01148753,3,-146.22508207,0\H,11,1.09583391,8
,111.34618212,5,95.99812066,0\H,11,1.0962253,8,109.70302693,5,-144.121
53571,0\H,11,1.09575481,8,111.47969725,5,-24.86747878,0\C,5,1.53688154
,3,80.56261005,1,177.50390488,0\H,15,1.09884357,5,109.53934453,3,-77.6
0669025,0\H,15,1.10134454,5,109.61540437,3,38.88383869,0\C,15,1.536428
61,5,111.68143689,3,160.4507829,0\H,18,1.0949218,15,110.69036657,5,-17
9.37067046,0\H,18,1.09687933,15,111.1488517,5,60.94885494,0\H,18,1.097
3356,15,111.47593981,5,-59.4520853,0\H,1,1.09515322,3,92.50211625,8,-6
3.75438664,0\H,1,1.09492635,3,146.05148672,8,66.69393685,0\P,1,1.81737
332,3,36.59007749,8,59.05653987,0\C,24,1.81351208,1,108.18202439,3,115
.99347716,0\H,25,1.0945402,24,111.16896389,1,-176.20451188,0\H,25,1.09
6186,24,108.4649807,1,-57.04451733,0\H,25,1.09504302,24,110.34700053,1
,62.10179093,0\H,3,1.09533394,1,146.16234177,24,-6.3827595,0\H,3,1.096
69616,1,88.15657006,24,-129.26738386,0\N,5,1.48697257,3,41.86944547,1,
31.70419817,0\\Version=AM64L-G03RevD.01\State=1-A\HF=-710.2202016\MP2=
-711.7663335\RMSD=7.205e-09\Thermal=0.\PG=C01 [X(C8H21N1P1)]\\@

145

1\1\GINC-MOKKORI\SP\RMP2-FC\6-31+G(2d,p)\C8H18N1P1\CHRISTOPH\07-Apr-20
10\0\\#p MP2(FC)/6-31+g(2d,p) scf=tight\\aze1sp_11\\0,1\P\C,1,2.705172
5\C,2,1.54493119,1,144.6283894\C,3,1.54244227,2,115.43487613,1,-13.634
35364,0\C,4,1.54261425,3,113.01340817,2,85.71998958,0\C,5,1.53895079,4
,115.70136808,3,-68.71637239,0\C,6,1.54313444,5,115.27363445,4,50.1527
2097,0\H,2,1.10670086,1,102.73892489,7,98.31734832,0\H,2,1.09702597,1,
77.61401151,7,-157.22602636,0\H,3,1.09926656,2,106.8403726,1,-136.4501
6312,0\H,3,1.10014272,2,109.87220122,1,109.4641625,0\H,4,1.09947696,3,
110.1170057,2,-38.09406921,0\H,4,1.09914848,3,108.62498939,2,-154.5237
0472,0\H,5,1.10185838,4,108.0384453,3,53.88290459,0\H,5,1.09969695,4,1
09.2260089,3,168.76787351,0\H,6,1.10132894,5,108.72165359,4,172.520224
84,0\H,6,1.09876566,5,109.44809801,4,-71.2455942,0\H,7,1.10255946,6,11
0.33248494,5,54.03227532,0\H,7,1.09749769,6,108.45265039,5,169.5102683
6,0\C,1,1.87205093,2,111.15881247,3,-123.48907626,0\H,20,1.0986518,1,1
14.07496172,2,83.9175557,0\H,20,1.0983173,1,108.79804327,2,-155.305135

29,0\H,20,1.09670804,1,109.39835625,2,-37.99340169,0\C,1,1.86117151,20
,98.08239654,7,-85.24214616,0\H,24,1.09780984,1,108.38212426,20,-66.60
5783,0\H,24,1.09833899,1,113.81390053,20,53.69706831,0\H,24,1.09596157
,1,109.56440165,20,175.77431594,0\N,7,1.46577457,6,112.59866435,5,-69.
41464055,0\\Version=IA32L-G03RevD.01\State=1-A\HF=-708.6347763\MP2=-71
0.1710091\RMSD=3.788e-09\Thermal=0.\PG=C01 [X(C8H18N1P1)]\\@

145-Me^{+}

1\1\GINC-GRETEL\SP\RMP2-FC\6-31+G(2d,p)\C9H21N1P1(1+)\CHRISTOPH\07-Apr
-2010\0\\#p MP2(FC)/6-31+g(2d,p) scf=tight\\aze1mesp_2\\1,1\C\C,1,1.53
833875\C,2,1.54321265,1,115.66401011\C,3,1.54074777,2,113.35161367,1,8
7.64565584,0\C,4,1.53817032,3,115.73843233,2,-66.46781142,0\C,5,1.5382
6811,4,114.97323239,3,51.9299175,0\H,1,1.10072541,2,109.0297161,3,-169
.38119202,0\H,1,1.09515681,2,108.24051577,3,73.51422669,0\H,2,1.098177
54,1,110.13885639,6,62.26620158,0\H,2,1.09796122,1,105.27080792,6,175.
59775273,0\H,3,1.10051079,2,110.23274564,1,-36.92785842,0\H,3,1.096896
35,2,108.25394414,1,-152.64972289,0\H,4,1.09750612,3,109.22694073,2,17
1.26454171,0\H,4,1.10028897,3,108.32326752,2,56.13982329,0\H,5,1.09897
619,4,108.87519932,3,173.02370685,0\H,5,1.09949762,4,110.05624971,3,-7
0.47493189,0\H,6,1.09702049,5,111.60984231,4,49.20225461,0\H,6,1.09787
999,5,109.48923521,4,165.79058161,0\C,6,3.26901058,5,96.30576302,4,-13
3.58592553,0\H,19,1.09496564,6,94.75779845,5,28.82022458,0\H,19,1.0963
3344,6,155.39576998,5,-163.2414634,0\H,19,1.09464241,6,66.75575175,5,-
79.83395144,0\C,19,2.9338701,6,85.80691728,5,118.23603787,0\H,23,1.096
23804,19,85.74544238,6,159.89858821,0\H,23,1.09511694,19,92.61822364,6
,-91.39352542,0\H,23,1.09446288,19,147.15704102,6,41.99694354,0\P,23,1
.81413077,19,36.14335021,6,30.47477614,0\C,27,1.82483685,23,107.383231
48,19,115.39895772,0\H,28,1.09661115,27,110.46013369,23,-58.44026196,0
\H,28,1.09587505,27,109.82042368,23,61.74781126,0\H,28,1.09590583,27,1
10.27314523,23,-178.5670432,0\N,6,1.49119996,5,112.38699611,4,-73.0410
3081,0\\Version=IA32L-G03RevD.01\State=1-A\HF=-748.089844\MP2=-749.769
9867\RMSD=6.513e-09\Thermal=0.\PG=C01 [X(C9H21N1P1)]\\@

146

1\1\GINC-AZAZEL\SP\RMP2-FC\6-31+G(2d,p)\C8H20N1P1\CHRISTOPH\20-Apr-201
0\0\\#p MP2(FC)/6-31+g(2d,p) scf=tight\\dpa1sp_47\\0,1\P\C,1,1.8654744
5\H,2,1.09578748,1,109.36464108\H,2,1.09859802,1,108.48558085,3,117.52
860205,0\H,2,1.0985235,1,114.07705044,4,120.5775483,0\C,1,1.86737826,2
,97.57269976,3,174.90567287,0\H,6,1.09634573,1,109.69866207,2,-176.892
52762,0\C,1,2.73521493,2,121.32689123,6,-131.66991075,0\H,8,1.10271667
,1,124.81157685,2,-14.86430807,0\H,8,1.09663569,1,73.81618911,2,-114.0
4955659,0\C,8,2.49559279,1,64.93012681,2,61.89785175,0\H,11,1.09682813
,8,139.64382986,1,-7.80711366,0\H,11,1.10382913,8,95.92692339,1,115.85
055773,0\C,11,1.54281949,8,95.61199863,1,-134.8171133,0\H,14,1.0991987
6,11,109.81926922,8,-38.79220393,0\H,14,1.10017938,11,107.27569269,8,-
153.38461956,0\C,8,1.54360274,1,123.79092796,2,143.5464466,0\H,17,1.09
985302,8,109.92258029,1,-101.60713014,0\H,17,1.10017156,8,107.29269856
,1,143.7365947,0\C,14,1.53318539,11,113.95634954,8,84.74994727,0\H,20,
1.09704252,14,111.04684354,11,179.6768028,0\H,20,1.09644405,14,111.030
20418,11,-59.97981566,0\H,20,1.09837089,14,111.12340692,11,59.72480231
,0\C,17,1.5337942,8,113.97396545,1,21.95570325,0\H,24,1.09712085,17,11
1.15794267,8,177.18473403,0\H,24,1.09663294,17,111.10222988,8,-62.7754
2957,0\H,24,1.09804517,17,111.08037881,8,57.02378703,0\H,6,1.0983915,1
,113.92936181,2,-54.72529371,0\H,6,1.09823941,1,108.57611822,2,65.6532
5584,0\N,11,1.46325623,8,31.60821704,1,-4.58741162,0\\Version=AM64L-G0
3RevD.01\State=1-A\HF=-709.801857\MP2=-711.3608423\RMSD=3.368e-09\Ther
mal=0.\PG=C01 [X(C8H20N1P1)]\\@

146-Me+

1\1\GINC-GOLEM\SP\RMP2-FC\6-31+G(2d,p)\C9H23N1P1(1+)\CHRISTOPH\20-Apr-2010\0\\#p MP2(FC)/6-31+g(2d,p) scf=tight\\dpa1mesp_2\\1,1\C\H,1,1.096 03678\H,1,1.09672411,2,108.6043959\H,1,1.09538611,2,108.61016124,3,118 .64032819,0\C,1,2.92539769,4,146.27168927,2,-124.56549606,0\H,5,1.0951 1151,1,92.41571967,4,129.37123473,0\C,1,3.8615407,5,74.48893649,6,103. 99287885,0\H,7,1.09607161,1,102.02342099,5,73.4655929,0\H,7,1.09620279 ,1,143.67855183,5,-66.01243216,0\C,7,2.52193175,1,63.80335609,5,-61.71 127616,0\H,10,1.0979382,7,81.98558477,1,-104.9490254,0\H,10,1.09890484 ,7,136.6787877,1,-0.5468979,0\C,10,1.53555403,7,106.64831578,1,145.848 10223,0\H,13,1.09833608,10,109.34675261,7,22.0946212,0\H,13,1.09873043 ,10,109.18273555,7,-94.00954635,0\C,7,1.53688732,1,80.58445021,5,-177. 33540578,0\H,16,1.09881037,7,109.49894266,1,77.95357856,0\H,16,1.10122 763,7,109.53074776,1,-38.49725507,0\C,13,1.53617221,10,111.69855681,7, 143.80572196,0\H,19,1.0949743,13,110.52308357,10,-179.96098056,0\H,19, 1.09705638,13,111.37481796,10,-60.31572786,0\H,19,1.09765823,13,111.56 069468,10,60.31167192,0\C,16,1.53624197,7,111.75830689,1,-160.08775182 ,0\H,23,1.0949493,16,110.66129409,7,179.44194201,0\H,23,1.09687254,16, 111.18208573,7,-60.89973119,0\H,23,1.09731958,16,111.47845517,7,59.539 83356,0\H,5,1.09489715,1,146.18327538,10,-66.55342022,0\H,5,1.09630425 ,1,87.07242116,10,172.09556455,0\P,5,1.81737461,1,36.65476519,10,-58.9 909736,0\C,29,1.81361039,5,108.12841103,1,-115.84160661,0\H,30,1.09483 006,29,111.26746999,5,175.56194164,0\H,30,1.09501185,29,110.31747161,5 ,-62.65097671,0\H,30,1.09611704,29,108.38372086,5,56.41351059,0\N,7,1. 48643489,1,41.81236925,29,-48.77438863,0\\Version=AM64L-G03RevD.01\Sta te=1-A\HF=-749.2600686\MP2=-750.9592731\RMSD=7.625e-09\Thermal=0.\PG=C 01 [X(C9H23N1P1)]\\@

147

1\1\GINC-YANG\SP\RMP2-FC\6-31+G(2d,p)\C5H15N2P1\CHRISTOPH\11-Sep-2009\ 0\\#p MP2(FC)/6-31+g(2d,p) scf=tight\\dma2sp_12\\0,1\P\C,1,1.85465536\ H,2,1.09578267,1,107.91368883\H,2,1.0957868,1,107.91055041,3,117.09820 281,0\H,2,1.09716262,1,116.21491185,4,121.449332,0\C,1,2.83455845,2,84 .31262392,3,159.73669674,0\H,6,1.09376453,1,85.26688964,2,-20.6591292, 0\H,6,1.10075929,1,134.70341879,2,89.44828671,0\H,6,1.10482803,1,109.0 9772554,2,-128.29981816,0\C,6,2.42208179,1,61.5183588,2,147.09748555,0 \H,10,1.10077626,6,91.2712609,1,-141.35067626,0\H,10,1.09499914,6,143. 50723596,1,-17.43029396,0\H,10,1.10535746,6,93.83478777,1,110.97945879 ,0\C,1,2.83464891,2,84.3173366,6,-76.55634231,0\H,14,1.10073363,1,134. 7012963,2,-89.48311837,0\H,14,1.09374415,1,85.26483105,2,20.6293773,0\ H,14,1.10483669,1,109.09490074,2,128.26992085,0\C,14,2.42211988,1,61.5 1590853,2,-147.1159388,0\H,18,1.10075055,14,91.26921275,1,141.3484155, 0\H,18,1.1053612,14,93.83600341,1,-110.97855787,0\H,18,1.09500389,14,1 43.50435075,1,17.42919699,0\N,6,1.45718176,1,29.82098968,2,129.4167933 ,0\N,14,1.45718309,1,29.8209673,22,131.06051472,0\\Version=AM64L-G03Re vD.01\State=1-A\HF=-647.7093669\MP2=-648.990775\RMSD=3.624e-09\Thermal =0.\PG=C01 [X(C5H15N2P1)]\\@

147-Me+

1\1\GINC-GOLEM\SP\RMP2-FC\6-31+G(2d,p)\C6H18N2P1(1+)\CHRISTOPH\11-Sep-2009\0\\#p MP2(FC)/6-31+g(2d,p) scf=tight\\dma2mesp_8\\1,1\P\C,1,1.818 36311\H,2,1.09515803,1,111.08657119\H,2,1.09657036,1,107.46820637,3,11 8.41595847,0\H,2,1.09528654,1,111.97231562,4,118.8764529,0\C,1,1.81835 436,2,106.49966676,3,178.97418721,0\H,6,1.09528803,1,111.97634844,2,56 .25810426,0\H,6,1.09515806,1,111.08423752,2,178.96584726,0\H,6,1.09657 102,1,107.46760065,2,-62.61874146,0\C,1,2.69541216,6,98.66242256,2,144 .07273979,0\H,10,1.09314751,1,80.34145984,6,77.57295088,0\H,10,1.09822 867,1,111.92439415,6,-29.63627245,0\H,10,1.09549874,1,132.38579696,6,-

176.6180185,0\C,1,2.69542595,6,136.45067762,2,-122.65992009,0\H,14,1.0
9823196,1,111.91931152,6,95.63030624,0\H,14,1.09549504,1,132.38946019,
6,-51.3460497,0\H,14,1.09314532,1,80.3440278,6,-157.1582271,0\C,10,2.4
7124417,1,64.43724007,6,-112.8321826,0\H,18,1.09843841,10,96.14746974,
1,108.87022463,0\H,18,1.09133845,10,143.8431127,1,-26.22347129,0\H,18,
1.09532694,10,86.98202308,1,-142.77131277,0\C,14,2.47123392,1,64.43733
735,6,12.42801533,0\H,22,1.09532685,14,86.98446737,1,-142.770349,0\H,2
2,1.09844244,14,96.14566811,1,108.87092487,0\H,22,1.09134225,14,143.84
462483,1,-26.21809126,0\N,18,1.47353643,10,33.13007151,1,-12.03096597,
0\N,22,1.47353279,14,33.13088549,1,-12.0336554,0\\Version=AM64L-G03Rev
D.01\State=1-A\HF=-687.1669109\MP2=-688.5914319\RMSD=4.499e-09\Thermal
=0.\PG=C01 [X(C6H18N2P1)]\\@

148

1\1\GINC-YANG\SP\RMP2-FC\6-31+G(2d,p)\C6H14N1P1\CHRISTOPH\21-Sep-2009\
0\\#p MP2(FC)/6-31+g(2d,p) scf=tight\\py1sp_3\\0,1\P\C,1,1.86296121\H,
2,1.09753784,1,108.8599852\H,2,1.09808831,1,113.51457893,3,120.5658831
6,0\H,2,1.09612668,1,109.35036167,3,-117.7611358,0\C,1,1.87135246,2,98
.16782772,5,174.4013616,0\H,6,1.09815486,1,108.74915794,2,69.90373349,
0\H,6,1.09676347,1,109.45989329,2,-172.79467635,0\H,6,1.09830579,1,113
.98401296,2,-50.78762651,0\C,1,2.84411966,2,85.86539915,6,-88.22161471
,0\C,10,1.54776191,1,132.62745078,2,-147.67408194,0\C,11,1.54154553,10
,103.815639,1,29.55120574,0\C,12,1.53489918,11,102.24956105,10,-36.220
60366,0\H,10,1.10077985,1,91.30089202,2,91.65011353,0\H,10,1.10060666,
1,100.04035054,2,-16.10084146,0\H,11,1.09581917,10,112.34716954,1,152.
32822493,0\H,11,1.09788896,10,109.97647904,1,-87.80893951,0\H,12,1.095
99932,11,113.10037408,10,-157.95225335,0\H,12,1.09773048,11,110.366573
22,10,80.48095242,0\H,13,1.0962639,12,113.29582498,11,157.27236297,0\H
,13,1.10572994,12,110.00521731,11,-82.31096793,0\N,13,1.46933908,12,10
2.99140546,11,37.92832072,0\\Version=AM64L-G03RevD.01\State=1-A\HF=-63
0.563075\MP2=-631.7865158\RMSD=3.163e-09\Thermal=0.\PG=C01 [X(C6H14N1P
1)]\\@

148-Me$^+$

1\1\GINC-GRETEL\SP\RMP2-FC\6-31+G(2d,p)\C7H17N1P1(1+)\CHRISTOPH\21-Sep
-2009\0\\#p MP2(FC)/6-31+g(2d,p) scf=tight\\py1mesp_2\\1,1\P\C,1,1.813
18504\H,2,1.09606682,1,109.21338383\H,2,1.09513828,1,110.70131618,3,11
9.32676809,0\H,2,1.09510067,1,110.01642768,3,-119.74605963,0\C,1,1.824
36942,2,107.62137285,5,-178.29616731,0\H,6,1.09668749,1,110.47659742,2
,59.64003441,0\H,6,1.09587809,1,110.10820357,2,179.71355735,0\H,6,1.09
593426,1,109.95454231,2,-60.54017348,0\C,1,1.81420951,2,109.39948408,6
,116.60212173,0\H,10,1.0954125,1,110.58299674,2,-178.98947779,0\H,10,1
.09599786,1,109.60447715,2,-59.45948903,0\H,10,1.09509701,1,109.772922
59,2,60.61879087,0\C,1,2.75427928,2,134.94608181,6,-134.7736613,0\C,14
,1.54033738,1,130.88307564,2,52.16088667,0\C,15,1.53819901,14,103.7323
4945,1,-42.64320861,0\C,16,1.53567536,15,103.17673541,14,39.36444822,0
\H,14,1.09732814,1,86.52047015,2,168.33551616,0\H,14,1.09670977,1,103.
62020906,2,-83.44825944,0\H,15,1.09667636,14,110.05474769,1,75.3363598
2,0\H,15,1.09385712,14,111.60045041,1,-164.76563052,0\H,16,1.09393499,
15,113.07029432,14,160.25221547,0\H,16,1.09648984,15,110.6622598,14,-7
8.08928677,0\H,17,1.09979438,16,110.95870748,15,83.15882504,0\H,17,1.0
9461902,16,112.75518102,15,-154.37691462,0\N,17,1.49356605,16,102.5738
1396,15,-35.65816947,0\\Version=IA32L-G03RevD.01\State=1-A\HF=-670.019
0479\MP2=-671.3866896\RMSD=4.132e-09\Thermal=0.\PG=C01 [X(C7H17N1P1)]\
\@

149

1\1\GINC-AZAZEL\SP\RMP2-FC\6-31+G(2d,p)\C6H18N3P1\CHRISTOPH\11-Sep-200

9\0\\#p MP2(FC)/6-31+g(2d,p) scf=tight\\dma3sp_51\\0,1\P\C,1,2.7152843
5\H,2,1.10642573,1,97.72286701\H,2,1.09242103,1,85.83679246,3,107.8883
152,0\H,2,1.098663,1,144.30210567,4,116.46413473,0\C,2,2.4008653,1,63.
76159715,4,162.97563602,0\H,6,1.10642317,2,92.6196151,1,97.34008198,0\
H,6,1.09242495,2,145.41144535,1,-30.89028831,0\H,6,1.09866028,2,89.954
13923,1,-154.92347175,0\C,1,2.82196475,6,106.8953095,2,-56.46286857,0\
H,10,1.10405041,1,111.48851885,6,-117.73170604,0\H,10,1.09333843,1,82.
54873456,6,-10.24730873,0\H,10,1.10123946,1,133.06049621,6,97.87878041
,0\C,10,2.44000519,1,61.59005766,6,159.0076884,0\H,14,1.10452628,10,93
.83061938,1,113.75692675,0\H,14,1.10106982,10,92.11422963,1,-138.44439
721,0\H,14,1.095597,10,142.90600944,1,-13.76689423,0\C,1,2.71386612,14
,103.76153499,10,104.37913387,0\H,18,1.10453441,1,113.23094175,14,-20.
7018849,0\H,18,1.10106832,1,134.34892566,14,-172.29611632,0\H,18,1.095
59535,1,77.69192562,14,84.08651472,0\C,18,2.43998883,1,66.14736155,14,
-104.36767052,0\H,22,1.10405492,18,94.48305242,1,112.03166006,0\H,22,1
.10124032,18,90.83784603,1,-140.22906767,0\H,22,1.09334022,18,142.6718
9626,1,-17.75536338,0\N,2,1.46610739,1,36.28759792,18,-10.34470854,0\N
,14,1.45625905,10,33.1346746,1,-13.02892514,0\N,18,1.45627515,1,34.345
63953,27,-89.04792124,0\\Version=AM64L-G03RevD.01\State=1-A\HF=-741.77
18653\MP2=-743.3970283\RMSD=6.150e-09\Thermal=0.\PG=C01 [X(C6H18N3P1)]
\\@

149-Me⁺

1\1\GINC-AZAZEL\SP\RMP2-FC\6-31+G(2d,p)\C7H21N3P1(1+)\CHRISTOPH\14-Sep
-2009\0\\#p MP2(FC)/6-31+g(2d,p) scf=tight\\dma3mesp_14\\1,1\P\C,1,1.8
2117975\H,2,1.09404801,1,112.8397311\H,2,1.09540095,1,108.26483826,3,1
20.96442493,0\H,2,1.09537693,1,109.66311271,4,117.51971698,0\C,1,2.712
74204,2,133.52419645,3,37.20182839,0\H,6,1.09256038,1,80.42162565,2,-1
40.20305547,0\H,6,1.09839487,1,114.70453539,2,113.41970156,0\H,6,1.096
00994,1,129.95439162,2,-34.37130273,0\C,6,2.46818105,1,64.37709738,2,2
9.9362514,0\H,10,1.09839575,6,93.87683492,1,116.47704343,0\H,10,1.0960
5275,6,89.44511269,1,-134.89033123,0\H,10,1.09144761,6,144.30560779,1,
-13.52096578,0\C,1,2.71840059,2,95.64853643,10,132.85377571,0\H,14,1.0
9318603,1,81.42216623,2,76.43818716,0\H,14,1.09609535,1,132.9042156,2,
-176.52090891,0\H,14,1.09887396,1,111.17630039,2,-30.75436702,0\C,14,2
.4671901,1,63.44972452,2,-112.79836559,0\H,18,1.0991679,14,94.32279397
,1,110.29311199,0\H,18,1.09576727,14,89.06716611,1,-141.22160844,0\H,1
8,1.09168954,14,143.69540337,1,-20.86235455,0\C,1,2.75706488,2,130.965
19013,10,-151.02482336,0\H,22,1.0913371,1,81.40809866,2,-134.11441166,
0\H,22,1.09823323,1,117.06660776,2,118.79811065,0\H,22,1.09657803,1,12
6.74491439,2,-26.83831444,0\C,22,2.46301818,1,62.4269263,2,44.87308548
,0\H,26,1.0957961,22,88.72675148,1,-137.93249375,0\H,26,1.09221106,22,
144.54255264,1,-17.69160909,0\H,26,1.09778524,22,94.23477671,1,113.247
14534,0\N,10,1.47236991,6,33.07429419,1,-7.57631965,0\N,18,1.47166552,
14,33.20225721,1,-13.74717501,0\N,26,1.47371002,22,33.32642514,1,-9.38
000058,0\\Version=AM64L-G03RevD.01\State=1-A\HF=-781.2353117\MP2=-783.
0020417\RMSD=9.275e-09\Thermal=0.\PG=C01 [X(C7H21N3P1)]\\@

150

1\1\GINC-CIPCLU08\SP\RMP2-FC\6-31+G(2d,p)\C7H15N2P1\C2175\12-May-2010\
0\\#p MP2(FC)/6-31+G(2d,p) scf=tight\\nv2sp_6\\0,1\P\C,1,1.857752\H,2,
1.098367,1,113.0110709\H,2,1.09541681,1,108.73290639,3,120.33824869,0\
H,2,1.09667622,1,109.70972565,4,118.4891687,0\C,1,2.80570765,2,123.934
07331,4,62.67702804,0\C,6,1.55229825,1,116.53295845,2,36.48557607,0\C,
7,1.5509476,6,86.72616817,1,-0.62971187,0\H,6,1.10148649,1,88.64962767
,2,151.04884872,0\H,6,1.09719997,1,108.92829673,2,-99.35940563,0\H,7,1
.09390769,6,116.77340635,1,117.81277316,0\H,7,1.09418873,6,112.8005084
1,1,-113.97846998,0\H,8,1.10149448,7,112.55718834,6,100.37979387,0\H,8

,1.09756766,7,117.41074617,6,-131.56133318,0\C,1,2.9449931,2,80.145968
78,8,83.4217104,0\C,15,1.55286134,1,112.08223407,2,143.49128293,0\C,16
,1.55498502,15,87.44809511,1,0.63710313,0\H,15,1.09878648,1,109.269470
04,2,-86.07093484,0\H,15,1.10157028,1,95.07867121,2,25.35555838,0\H,16
,1.09399997,15,113.34611432,1,115.06117579,0\H,16,1.09384081,15,116.07
049919,1,-117.29549142,0\H,17,1.10096801,16,113.85082977,15,-107.18792
593,0\H,17,1.09845431,16,116.16530601,15,125.81695049,0\N,6,1.48477083
,1,31.22994183,2,6.29961261,0\N,17,1.47892403,16,89.00852871,15,8.8875
8335,0\\Version=AM64L-G03RevD.01\State=1-A\HF=-723.4024479\MP2=-724.93
98479\RMSD=4.964e-09\Thermal=0.\PG=C01 [X(C7H15N2P1)]\\@

150-Me$^+$

1\1\GINC-CIPCLU10\SP\RMP2-FC\6-31+G(2d,p)\C8H18N2P1(1+)\C2175\12-May-2
010\0\\#p MP2(FC)/6-31+G(2d,p) scf=tight\\nv2mesp_11\\1,1\C\H,1,1.0944
7831\C,1,3.36841258,2,163.41175753\C,3,1.55346568,1,140.986605,2,86.23
054703,0\C,4,1.55346559,3,88.43189978,1,-23.10650069,0\H,3,1.09483166,
1,76.07618282,5,131.88016448,0\H,3,1.09715605,1,92.87716458,5,-118.597
0768,0\H,4,1.09162173,3,115.58719814,1,-140.87596601,0\H,4,1.0921661,3
,113.26600636,1,91.6242812,0\H,5,1.09483201,4,116.68734891,3,124.21200
773,0\H,5,1.09715624,4,113.96035317,3,-106.37149121,0\C,1,4.22995346,3
,76.69017712,4,69.23518097,0\C,12,1.55346498,1,96.93036769,3,99.291328
24,0\C,13,1.55346413,12,88.4316157,1,-11.42884422,0\H,12,1.09483191,1,
106.81654228,3,-140.08561279,0\H,12,1.09715701,1,111.96533921,3,-20.05
770588,0\H,13,1.09162207,12,115.58798775,1,-129.19923438,0\H,13,1.0921
6658,12,113.26527591,1,103.30088356,0\H,14,1.09715725,13,113.95971695,
12,-106.36853302,0\H,14,1.09483157,13,116.6879555,12,124.21493064,0\P,
1,1.81119363,3,58.08137387,4,40.82958333,0\C,21,1.81119313,1,113.02883
745,3,-132.12781424,0\H,22,1.09618295,21,110.31543225,1,179.99269943,0
\H,22,1.09447746,21,109.66842007,1,-60.35391125,0\H,22,1.09447879,21,1
09.66806864,1,60.34011167,0\H,1,1.09618354,21,110.31528702,22,179.9909
4096,0\H,1,1.0944781,21,109.66908334,22,-60.35533099,0\N,14,1.49597313
,13,88.6632642,12,8.90096555,0\N,3,1.49596823,1,53.70436006,21,23.1044
3676,0\\Version=AM64L-G03RevD.01\State=1-A\HF=-762.8652615\MP2=-764.54
39516\RMSD=5.347e-09\Thermal=0.\PG=C01 [X(C8H18N2P1)]\\@

151

1\1\GINC-CIPCLU08\SP\RMP2-FC\6-31+G(2d,p)\C7H19N2P1\C2175\17-May-2010\
0\\#p MP2(FC)/6-31+G(2d,p) scf=tight\\nem2sp_68\\0,1\P\C,1,1.85669858\
H,2,1.0957795,1,107.47499684\H,2,1.09696427,1,116.11185014,3,-120.7314
6027,0\H,2,1.09608604,1,108.41703812,3,117.12821964,0\C,1,2.83820774,2
,82.92142726,3,165.29297868,0\H,6,1.09403457,1,85.39688946,2,-13.32368
929,0\H,6,1.10059018,1,134.55245856,2,96.6706913,0\H,6,1.10258782,1,10
9.25825698,2,-120.68439295,0\C,6,2.45741474,1,61.3323115,2,147.7109993
,0\H,10,1.10242271,6,87.57945234,1,-141.27007879,0\H,10,1.09705662,6,1
39.36243433,1,-27.81773857,0\C,10,1.53917255,6,100.65982337,1,109.7199
2587,0\H,13,1.09804453,10,110.3202989,6,148.56690263,0\H,13,1.09713819
,10,111.74412281,6,28.93082444,0\H,13,1.09739154,10,110.89799237,6,-91
.70385342,0\C,1,2.81868501,2,85.52417987,6,-76.4311338,0\H,17,1.104935
68,1,108.62546928,2,133.61816285,0\H,17,1.0987355,1,136.05479887,2,-82
.1161301,0\H,17,1.09372814,1,84.07931052,2,26.48295913,0\C,17,2.447239
01,1,61.73063014,2,-142.0157531,0\H,21,1.10635754,17,87.26033916,1,-11
9.01044543,0\H,21,1.09700234,17,139.18931651,1,-6.109038,0\C,21,1.5362
8529,17,101.5344095,1,131.74566534,0\H,24,1.09630109,21,110.28405564,1
7,-91.11610053,0\H,24,1.09684884,21,112.13484911,17,29.45039711,0\H,24
,1.09782017,21,110.16742828,17,149.1023242,0\N,6,1.45817476,1,29.55646
463,2,134.9953409,0\N,17,1.45874908,1,30.66753235,28,135.68781308,0\\V
ersion=AM64L-G03RevD.01\State=1-A\HF=-725.7879949\MP2=-727.379179\RMSD
=5.565e-09\Thermal=0.\PG=C01 [X(C7H19N2P1)]\\@

151-Me$^+$

1\1\GINC-CIPCLU10\SP\RMP2-FC\6-31+G(2d,p)\C8H22N2P1(1+)\C2175\17-May-2
010\0\\#p MP2(FC)/6-31+G(2d,p) scf=tight\\nem2mesp_38\\1,1\C\H,1,1.096
12259\H,1,1.09528544,2,108.60622659\H,1,1.09518023,3,109.33097922,2,-1
18.00083428,0\C,1,2.91781583,4,147.55002131,2,-114.17209602,0\H,5,1.09
612244,1,86.75588236,4,-130.98056122,0\H,5,1.0951808,1,147.55007797,4,
-11.14882352,0\H,5,1.0952857,1,91.71307388,4,120.4758724,0\P,5,1.81883
008,1,36.66762841,4,-5.57503436,0\C,9,2.7735046,5,82.29883553,1,114.34
086488,0\H,10,1.09405115,9,84.60409049,5,17.94166723,0\H,10,1.09694497
,9,130.73730785,5,124.75136054,0\C,10,2.50201384,9,61.06161094,5,173.3
5898418,0\H,13,1.09366981,10,142.04998598,9,-21.95766848,0\H,13,1.0955
3372,10,88.36453405,9,-139.6180959,0\H,13,1.09702196,10,96.76379273,9,
111.51513338,0\C,10,1.5329329,9,110.0742569,5,-91.98618024,0\H,17,1.09
788475,10,111.69544101,9,32.20360384,0\H,17,1.09607554,10,109.66987844
,9,151.49020261,0\H,17,1.09557243,10,111.76713401,9,-89.26007593,0\C,9
,2.77350761,5,115.45296781,1,-89.28677755,0\H,21,1.09405168,9,84.60310
637,5,122.78383569,0\H,21,1.09694573,9,130.73533921,5,-130.40776332,0\
C,21,2.50201464,9,61.06181215,5,-81.79793714,0\H,24,1.09366862,21,142.
05076407,9,-21.9446023,0\H,24,1.09702099,21,96.7609023,9,111.52350864,
0\H,24,1.09553515,21,88.36702512,9,-139.60934202,0\C,21,1.53293014,9,1
10.07677899,5,12.85609312,0\H,28,1.09607556,21,109.67007371,9,151.4914
6008,0\H,28,1.097885,21,111.69549473,9,32.20460113,0\H,28,1.09557345,2
1,111.76692433,9,-89.25874912,0\N,13,1.47605552,10,32.412784,9,-9.3568
2386,0\N,24,1.4760536,21,32.41269924,9,-9.35228245,0\\Version=AM64L-G0
3RevD.01\State=1-A\HF=-765.2491214\MP2=-766.9834125\RMSD=8.113e-09\The
rmal=0.\PG=C01 [X(C8H22N2P1)]\\@

152

1\1\GINC-CIPCLU04\SP\RMP2-FC\6-31+G(2d,p)\C11H23N2P1\C2175\25-Mar-2010
\0\\#p MP2(FC)/6-31+G(2d,p) scf=tight\\dpia2sp_15\\0,1\P\C,1,1.8560906
8\H,2,1.09585396,1,107.96514927\H,2,1.09587613,1,107.74542496,3,117.01
426936,0\H,2,1.09698984,1,116.25666546,4,121.2765032,0\C,1,4.09057698,
2,84.62200912,3,170.6475984,0\C,6,1.53939594,1,28.96944053,2,-91.06911
612,0\C,7,2.43480343,6,91.62225904,1,-48.73701989,0\C,8,1.53756841,7,9
1.74662221,6,0.13280414,0\C,6,1.53824405,1,101.54203856,2,156.61593315
,0\H,6,1.09936518,1,87.23726744,2,47.1011868,0\H,6,1.09927215,1,136.53
198271,2,-65.62255818,0\H,7,1.09479212,6,109.6933727,1,101.02049154,0\
H,7,1.10764857,6,109.05776695,1,-142.01588152,0\H,8,1.09631327,7,142.1
588911,6,127.76123407,0\H,8,1.10833863,7,92.82530705,6,-108.91525476,0
\H,9,1.09909348,8,109.57107792,7,-149.6057006,0\H,9,1.09929414,8,108.6
0878716,7,93.21836027,0\H,10,1.09838975,6,110.64509902,1,-147.36416553
,0\H,10,1.10177895,6,109.13393418,1,95.48128158,0\C,1,3.99649655,2,113
.52137646,7,-113.62117976,0\C,21,1.53760943,1,26.80543221,2,159.907858
57,0\C,22,2.43736162,21,91.7062796,1,-55.84499342,0\C,23,1.53946534,22
,91.54112052,21,-0.04955211,0\C,24,1.53815367,23,110.79297504,22,-27.2
6436442,0\H,21,1.09909542,1,133.03000211,2,-166.4605132,0\H,21,1.09929
07,1,86.88894899,2,-55.26684473,0\H,22,1.10831157,21,108.91644176,1,-1
49.71897201,0\H,22,1.09641147,21,110.48168614,1,92.66117303,0\H,23,1.0
9491243,22,143.26600881,21,126.77418673,0\H,23,1.10762849,22,92.859971
06,21,-109.21046065,0\H,24,1.09924925,23,109.5246711,22,-149.71812802,
0\H,24,1.09941277,23,108.55792729,22,93.20345424,0\H,25,1.1017962,24,1
09.13064766,23,-66.55508081,0\H,25,1.09837816,24,110.66853543,23,176.2
7505157,0\N,23,1.46664564,22,33.91907195,21,126.86830507,0\N,7,1.46673
515,6,110.94288685,1,-20.00931139,0\\Version=AM64L-G03RevD.01\State=1-
A\HF=-879.6271835\MP2=-881.7907909\RMSD=6.502e-09\Thermal=0.\PG=C01 [X
(C11H23N2P1)]\\@

152-Me$^+$

1\1\GINC-CIPCLU07\SP\RMP2-FC\6-31+G(2d,p)\C12H26N2P1(1+)\C2175\25-Mar-
2010\0\\#p MP2(FC)/6-31+G(2d,p) scf=tight\\dpia2mesp_5\\1,1\P\C,1,1.81
972912\H,2,1.09651989,1,107.35283692\H,2,1.0950434,1,111.07688553,3,11
8.35416549,0\H,2,1.09515841,1,112.02997297,3,-118.93966169,0\C,1,1.819
72621,2,106.23899739,4,-178.04587787,0\H,6,1.0965189,1,107.35385672,2,
63.60707027,0\H,6,1.09516302,1,112.02353683,2,-55.32963453,0\H,6,1.095
04088,1,111.08166927,2,-178.0360795,0\C,1,3.98254336,6,89.8301228,2,12
7.7556668,0\C,10,1.53584373,1,30.79889755,6,-67.01335989,0\C,11,2.4847
4884,10,90.62431534,1,-50.20970063,0\C,12,1.53446811,11,90.96493655,10
,0.03385529,0\C,13,1.5376984,12,111.08717764,11,-27.50576849,0\H,10,1.
09918039,1,85.87627664,6,71.99233636,0\H,10,1.09719817,1,136.75693272,
6,-38.9776418,0\H,11,1.09260562,10,110.04321615,1,98.4411173,0\H,11,1.
10167449,10,110.06569454,1,-142.96439066,0\H,12,1.09500539,11,142.0507
0225,10,126.21571798,0\H,12,1.10173274,11,92.15195135,10,-110.12496528
,0\H,13,1.09720186,12,108.54597126,11,-149.54877489,0\H,13,1.09919792,
12,108.99689583,11,93.91940526,0\H,14,1.10027037,13,109.4680102,12,-66
.58598865,0\H,14,1.09612094,13,110.30756545,12,176.24722361,0\C,1,3.93
068593,6,113.73139574,2,-142.94469361,0\C,25,1.53446457,1,29.42861303,
6,-2.66801427,0\C,26,2.48470752,25,90.96657024,1,54.28029811,0\C,27,1.
53585418,26,90.62398917,25,0.03702544,0\C,25,1.53769897,1,104.35113993
,6,104.92662918,0\H,25,1.0991989,1,85.74174657,6,-145.44999081,0\H,25,
1.09720265,1,134.67931445,6,-35.26396699,0\H,26,1.09500674,25,110.4721
0721,1,-93.72944121,0\H,26,1.10173428,25,110.10258966,1,146.92208991,0
\H,27,1.10167263,26,92.37110826,25,110.15053502,0\H,27,1.09260191,26,1
42.9894313,25,-125.67977425,0\H,28,1.09719913,27,108.42640682,26,149.6
2936253,0\H,28,1.09917993,27,108.88524168,26,-93.96259511,0\H,29,1.096
12217,25,110.3084254,1,146.11622607,0\H,29,1.10026966,25,109.46724633,
1,-96.71698133,0\N,11,1.48537144,10,110.28480428,1,-22.61641524,0\N,27
,1.48536958,26,33.3831372,25,-127.80966652,0\\Version=AM64L-G03RevD.01
\State=1-A\HF=-919.0906326\MP2=-921.3976508\RMSD=7.278e-09\Thermal=0.\
PG=C01 [X(C12H26N2P1)]\\@

153

1\1\GINC-CIPCLU10\SP\RMP2-FC\6-31+G(2d,p)\C9H23N2P1\C2175\04-May-2010\
0\\#p MP2(FC)/6-31+G(2d,p) scf=tight\\dea2sp_106\\0,1\P\C,1,1.85908147
\H,2,1.09623668,1,108.19794121\H,2,1.09738731,1,115.87799041,3,121.281
12511,0\H,2,1.09606416,1,108.04937207,3,-117.25525243,0\C,1,2.8538972,
2,84.55077255,5,79.1190502,0\H,6,1.10108638,1,130.00621322,2,-82.22103
46,0\H,6,1.09642928,1,82.62867362,2,22.02180024,0\C,6,2.47984764,1,61.
00816018,2,-138.5614467,0\H,9,1.09676179,6,137.71672353,1,-21.65101695
,0\H,9,1.10195508,6,86.68354929,1,-132.58650931,0\C,6,1.54017249,1,114
.48440865,2,129.88253056,0\H,12,1.09666752,6,111.50154059,1,91.9883983
,0\H,12,1.09835699,6,110.30303297,1,-148.5426527,0\H,12,1.09566964,6,1
11.08497737,1,-28.33009061,0\C,9,1.53741076,6,103.58835939,1,118.06283
545,0\H,16,1.09824087,9,110.51191225,6,146.35450151,0\H,16,1.09665996,
9,110.31795989,6,-93.7037805,0\H,16,1.09726791,9,111.81159388,6,26.507
25393,0\C,1,2.71906269,2,124.99730548,6,112.9695442,0\H,20,1.10049548,
1,135.75697096,2,13.63882976,0\H,20,1.09719211,1,78.22390748,2,115.256
83418,0\C,20,1.53915554,1,110.11966912,2,-138.37621713,0\H,23,1.098374
73,20,110.46794929,1,-145.00376403,0\H,23,1.09683705,20,110.7222318,1,
-25.15398691,0\H,23,1.09751984,20,111.7292046,1,95.18114634,0\C,20,2.4
7770532,1,66.44215733,2,-40.27674764,0\H,27,1.09582742,20,137.71383563
,1,-27.95738836,0\H,27,1.10182142,20,84.27929108,1,-135.02165846,0\C,2
7,1.53811198,20,105.64792697,1,116.5111488,0\H,30,1.0968377,27,111.347
99922,20,25.35305876,0\H,30,1.0983554,27,110.60681461,20,145.16680871,
0\H,30,1.09615782,27,110.68948991,20,-94.59829512,0\N,6,1.46419769,1,2
8.95412463,2,-132.53851641,0\N,27,1.46572183,20,32.38662916,1,7.552062

33,0\\Version=AM64L-G03RevD.01\State=1-A\HF=-803.8643548\MP2=-805.7659
374\RMSD=4.787e-09\Thermal=0.\PG=C01 [X(C9H23N2P1)]\\@

153-Me$^+$

1\1\GINC-CIPCLU09\SP\RMP2-FC\6-31+G(2d,p)\C10H26N2P1(1+)\C2175\04-May-
2010\0\\#p MP2(FC)/6-31+G(2d,p) scf=tight\\dea2mesp_41\\1,1\C\H,1,1.09
52365\H,1,1.09494625,2,109.29849234\H,1,1.09624069,3,108.24687462,2,11
8.15501392,0\C,1,3.87910036,3,105.03188022,4,75.49603023,0\H,5,1.09404
39,1,100.10661165,3,160.76761486,0\H,5,1.09433617,1,143.7877293,3,25.5
0336903,0\C,5,2.52644316,1,60.87331203,3,21.52909968,0\H,8,1.09842744,
5,89.06182178,1,-89.60461242,0\H,8,1.09583477,5,139.12163947,1,25.8320
1906,0\C,5,1.53359274,1,83.13003406,8,-110.97630886,0\H,11,1.09579364,
5,111.79452147,1,82.16479725,0\H,11,1.0979152,5,111.89588048,1,-39.307
66884,0\H,11,1.09615421,5,109.67157703,1,-158.59064515,0\C,8,1.5318799
9,5,99.5011381,1,159.81104415,0\H,15,1.09617075,8,109.08102762,5,152.8
8015416,0\H,15,1.09531167,8,111.19748146,5,-87.71724963,0\H,15,1.09522
831,8,112.47642696,5,34.08451834,0\C,1,3.08567707,8,81.94027914,5,116.
87432432,0\H,19,1.09432301,1,142.8115322,8,44.49470573,0\H,19,1.093436
4,1,50.94225603,8,104.86436359,0\C,19,1.53349531,1,105.6437432,8,-151.
15087436,0\H,22,1.0958305,19,111.88742501,1,128.09235391,0\H,22,1.0979
5749,19,111.85484104,1,6.59731344,0\H,22,1.09614547,19,109.61567347,1,
-112.71472025,0\C,19,2.53140033,1,96.60971862,8,-46.00829704,0\H,26,1.
09888912,19,92.97804612,1,-114.02519868,0\H,26,1.0957458,19,138.988294
33,1,6.8255658,0\C,26,1.53203598,19,96.26292324,1,135.22981057,0\H,29,
1.09601327,26,108.90294886,19,159.24246075,0\H,29,1.09568213,26,111.27
132271,19,-81.41801466,0\H,29,1.09498646,26,112.54514765,19,40.6541520
4,0\P,1,1.82110026,19,62.29536723,22,-101.38118707,0\C,33,1.82085226,1
,106.25343796,19,113.68759525,0\H,34,1.09612064,33,107.47816403,1,-58.
78557446,0\H,34,1.09497029,33,111.04069847,1,-177.07442294,0\H,34,1.09
512356,33,112.00895934,1,60.44687283,0\N,5,1.48556628,1,40.04725944,33
,-51.4300052,0\N,19,1.48491367,1,65.28837122,33,9.20687488,0\\Version=
AM64L-G03RevD.01\State=1-A\HF=-843.3279031\MP2=-845.3740729\RMSD=3.214
e-09\Thermal=0.\PG=C01 [X(C10H26N2P1)]\\@

154

1\1\GINC-YANG\SP\RMP2-FC\6-31+G(2d,p)\C9H19N2P1\CHRISTOPH\21-Sep-2009\
0\\#p MP2(FC)/6-31+g(2d,p) scf=tight\\py2sp_30\\0,1\P\C,1,2.7578754\C,
2,1.53596634,1,133.07477532\C,3,1.54790341,2,102.84681739,1,-52.580107
81,0\C,4,1.55928384,3,104.87380948,2,25.00147611,0\H,2,1.1070176,1,103
.8772786,5,-97.20329912,0\H,2,1.09554039,1,84.29448149,5,155.77203569,
0\H,3,1.09673948,2,109.28271574,1,64.77705677,0\H,3,1.09624891,2,113.1
3920234,1,-174.48599478,0\H,4,1.09560545,3,112.18036375,2,146.89701127
,0\H,4,1.0967836,3,110.46841933,2,-93.5090927,0\H,5,1.09691229,4,112.2
2423962,3,117.48160841,0\H,5,1.10268382,4,109.94627829,3,-122.97012348
,0\C,1,2.87628039,2,114.42964576,3,91.89273954,0\C,14,1.54578192,1,131
.96636942,2,78.35804164,0\C,15,1.54054334,14,103.53819069,1,29.9925876
7,0\C,16,1.53550326,15,102.01873571,14,-37.63577862,0\H,14,1.09920123,
1,93.082303,2,-44.16973914,0\H,14,1.10112905,1,99.37652412,2,-152.1798
4513,0\H,15,1.09595851,14,112.43037414,1,152.90006693,0\H,15,1.0981378
5,14,109.94048769,1,-87.0640359,0\H,16,1.09795912,15,110.34356382,14,7
9.07621049,0\H,16,1.0960671,15,113.16025463,14,-159.34739419,0\H,17,1.
10526073,16,109.86003574,15,-82.45537541,0\H,17,1.09661813,16,113.3711
346,15,157.18459739,0\C,1,1.85788352,2,124.37145452,3,-1.97674034,0\H,
26,1.09660333,1,114.57309908,2,70.68093039,0\H,26,1.09637033,1,108.522
68571,2,-168.26102314,0\H,26,1.09578818,1,108.47641926,2,-50.6754339,0
\N,2,1.46600786,1,32.82832675,26,-27.18265622,0\N,17,1.47062115,16,103
.3271758,15,37.66369319,0\\Version=AM64L-G03RevD.01\State=1-A\HF=-801.
5381127\MP2=-803.3885064\RMSD=6.606e-09\Thermal=0.\PG=C01 [X(C9H19N2P1
)]\\@

154-Me$^+$

1\1\GINC-AZAZEL\SP\RMP2-FC\6-31+G(2d,p)\C10H22N2P1(1+)\CHRISTOPH\21-Se
p-2009\0\\#p MP2(FC)/6-31+g(2d,p) scf=tight\\py2mesp_3\\1,1\P\C,1,1.81
764606\H,2,1.09579481,1,108.38651107\H,2,1.09581493,1,111.33418428,3,1
19.63349803,0\H,2,1.09516285,1,110.6709948,3,-118.96702436,0\C,1,1.819
02007,2,107.11289775,5,-175.52647107,0\H,6,1.09588427,1,108.38433971,2
,-59.88435328,0\H,6,1.09560891,1,111.22761809,2,59.605182,0\H,6,1.0949
4896,1,110.93251039,2,-179.09084347,0\C,1,2.74048695,2,99.81780173,6,1
42.05946023,0\C,10,1.5356123,1,132.51184161,2,-140.49485935,0\C,11,1.5
3853227,10,102.9574593,1,43.73642668,0\C,12,1.54034941,11,103.8887761,
10,-39.27178486,0\H,10,1.09531629,1,88.01719597,2,99.26020279,0\H,10,1
.09927586,1,100.11320813,2,-9.22809689,0\H,11,1.09404711,10,111.958609
09,1,165.49880268,0\H,11,1.09706297,10,110.02662185,1,-74.17028045,0\H
,12,1.09673504,11,110.21868212,10,78.50274442,0\H,12,1.09404636,11,113
.17928806,10,-160.45845174,0\H,13,1.09746509,12,111.36764452,11,-89.71
645889,0\H,13,1.09520593,12,111.928099,11,148.5278729,0\C,1,2.73887537
,2,134.01816301,6,-124.03860843,0\C,22,1.53849753,1,132.40813251,2,28.
97944775,0\C,23,1.53856047,22,103.38809457,1,-42.24268856,0\C,24,1.537
0734,23,103.33126662,22,39.61522694,0\H,22,1.09786367,1,101.2749126,2,
-104.78155905,0\H,22,1.09663313,1,87.3313525,2,147.18232881,0\H,23,1.0
9682409,22,110.05410529,1,75.58646474,0\H,23,1.09403431,22,111.8063516
2,1,-164.34240006,0\H,24,1.09410051,23,113.15351681,22,160.62196987,0\
H,24,1.09688877,23,110.49530067,22,-77.9751858,0\H,25,1.09435021,24,11
2.54827319,23,-152.78492312,0\H,25,1.09895687,24,110.95627425,23,85.14
726438,0\N,25,1.49226584,24,102.90085271,23,-33.37105135,0\N,10,1.4907
1052,1,30.60562913,34,130.40544124,0\\Version=AM64L-G03RevD.01\State=1
-A\HF=-841.0065111\MP2=-842.9998634\RMSD=6.363e-09\Thermal=0.\PG=C01 [
X(C10H22N2P1)]\\@

155

1\1\GINC-CIPCLU09\SP\RMP2-FC\6-31+G(2d,p)\C9H18N3P1\C2175\13-May-2010\
0\\#p MP2(FC)/6-31+G(2d,p) scf=tight\\nv3sp_34\\0,1\P\C,1,2.8835953\C,
2,1.55190557,1,112.67481994\C,3,1.55334504,2,86.9994736,1,-0.60280128,
0\H,2,1.09989558,1,90.79826389,4,115.86127087,0\H,2,1.09664682,1,110.8
4526813,4,-133.45618546,0\H,3,1.09431848,2,112.94183405,1,-114.1934266
1,0\H,3,1.09393257,2,116.68414315,1,117.71912463,0\H,4,1.09735635,3,11
7.27596356,2,-130.24266093,0\H,4,1.10118837,3,112.73625647,2,102.14053
025,0\C,1,2.79765631,4,147.63184518,3,-46.10436267,0\C,11,1.55019525,1
,114.54959103,4,-25.29226148,0\C,12,1.55145817,11,86.40557138,1,6.3329
3024,0\H,11,1.09821527,1,114.52752941,13,134.93698437,0\H,11,1.1034234
6,1,84.26168604,13,-117.24512798,0\H,12,1.09402661,11,116.8337273,1,-1
12.1115422,0\H,12,1.09441054,11,112.86551608,1,119.745331,0\H,13,1.096
82709,12,117.73177478,11,132.04727587,0\H,13,1.10189887,12,112.6980366
9,11,-100.44539023,0\C,1,2.84293061,11,93.70960413,12,-173.00233306,0\
C,20,1.55623526,1,116.10945994,11,63.70832231,0\C,21,1.55417862,20,87.
82120387,1,6.32001504,0\H,20,1.09865977,1,107.69947519,11,-164.9501142
9,0\H,20,1.09988301,1,92.15681269,11,-54.92705048,0\H,21,1.0940378,20,
115.31826291,1,-110.9041144,0\H,21,1.09403485,20,114.02534373,1,121.81
452103,0\H,22,1.09797759,21,115.09813798,20,-111.40349479,0\H,22,1.098
32549,21,115.54808466,20,121.14442798,0\N,4,1.48292654,3,88.83114641,2
,-13.34678703,0\N,20,1.479533,1,28.97619279,29,-48.70135192,0\N,11,1.4
8743255,1,32.89810011,30,-105.60283349,0\\Version=AM64L-G03RevD.01\Sta
te=1-A\HF=-855.3104423\MP2=-857.3183167\RMSD=4.131e-09\Thermal=0.\PG=C
01 [X(C9H18N3P1)]\\@

155-Me$^+$

1\1\GINC-CIPCLU10\SP\RMP2-FC\6-31+G(2d,p)\C10H21N3P1(1+)\C2175\13-May-

2010\0\\#p MP2(FC)/6-31+G(2d,p) scf=tight\\nv3mesp_19\\1,1\C\C,1,1.553
39621\C,2,1.55410971,1,88.45748481\H,1,1.09635067,2,114.42270571,3,-10
7.78432227,0\H,1,1.09449431,2,116.44870436,3,122.9843852,0\H,2,1.09222
922,1,113.50474292,3,-114.94203796,0\H,2,1.09180681,1,115.52217042,3,1
17.5619837,0\H,3,1.09501082,2,116.48597237,1,-122.98912579,0\H,3,1.097
21929,2,114.14817819,1,107.84886356,0\C,1,4.70486611,2,143.5878978,3,1
9.95877925,0\C,10,1.55407531,1,90.25114236,2,97.29584234,0\C,11,1.5542
9928,10,88.58543141,1,-21.34499854,0\H,10,1.0962302,1,126.70536985,2,-
24.46496216,0\H,10,1.09453308,1,98.28378229,2,-146.15787579,0\H,11,1.0
9207403,10,113.82554712,1,94.16986032,0\H,11,1.09192799,10,115.0635181
7,1,-138.49844787,0\H,12,1.09618491,11,114.79600979,10,-109.99089807,0
\H,12,1.09443329,11,116.20342051,10,120.72805794,0\C,3,4.47492897,2,12
0.84487411,1,43.70114173,0\C,19,1.55405095,3,120.83706083,2,0.04954967
,0\C,20,1.55357277,19,88.50200885,3,-43.99137802,0\H,19,1.09723173,3,5
6.90929243,2,100.44374353,0\H,19,1.09504467,3,121.25878977,2,-165.5729
3371,0\H,20,1.09221163,19,113.55983758,3,-159.20609365,0\H,20,1.091865
69,19,115.27301177,3,73.50083652,0\H,21,1.09462426,20,116.41955991,19,
-122.2140792,0\H,21,1.09618555,20,114.49883876,19,108.5714209,0\P,12,2
.83639368,11,113.07077628,10,-5.1617167,0\C,28,1.81685238,12,108.76829
201,11,-94.38413122,0\H,29,1.09480307,28,109.57759469,12,83.38515909,0
\H,29,1.09488309,28,109.53858166,12,-36.3556169,0\H,29,1.0965029,28,11
1.31162873,12,-156.46189383,0\N,10,1.4929122,1,15.86846198,2,12.549540
97,0\N,21,1.49323277,20,88.79384428,19,-6.8746867,0\N,1,1.49334135,2,8
8.78271116,3,7.56646735,0\\Version=AM64L-G03RevD.01\State=1-A\HF=-894.
7851999\MP2=-896.9319448\RMSD=9.978e-09\Thermal=0.\PG=C01 [X(C10H21N3P
1)]\\@

156

1\1\GINC-NODE17\SP\RMP2-FC\6-31+G(2d,p)\C15H30N3P1\ZIP07\27-Mar-2010\0
\\#p MP2(FC)/6-31+G(2d,p) scf=tight\\dpia3sp_47\\0,1\P\C,1,4.08618808\
C,2,1.53930473,1,28.6523398\C,3,2.44454968,2,91.43643651,1,-47.8103741
5,0\C,4,1.5372475,3,91.63251932,2,0.55426187,0\C,2,1.53845339,1,101.28
946789,4,-3.02053008,0\H,2,1.09980874,1,88.39527468,4,-112.67946922,0\
H,2,1.09921953,1,136.34596071,4,133.78631105,0\H,3,1.10742875,2,108.74
851444,1,-141.60828074,0\H,3,1.09443255,2,110.40824604,1,100.32006443,
0\H,4,1.09702849,3,141.9433949,2,128.0089764,0\H,4,1.10855763,3,93.271
83408,2,-108.48960503,0\H,5,1.09951588,4,108.68385045,3,92.92747591,0\
H,5,1.09905221,4,109.55462354,3,-149.90957718,0\H,6,1.10181588,2,109.1
2574549,1,95.06302389,0\H,6,1.09844718,2,110.64525092,1,-147.80695585,
0\C,3,3.88099499,2,152.47696204,1,-151.16060953,0\C,17,1.53999863,3,64
.19536608,2,-160.19572377,0\C,18,2.44735556,17,91.60134169,3,-102.5097
2568,0\C,19,1.53978762,18,91.6380406,17,0.43227585,0\C,20,1.53942865,1
9,110.54059725,18,-27.39926684,0\H,17,1.09945519,3,114.58929145,2,-59.
3182212,0\H,17,1.09962144,3,45.65368975,2,31.20172045,0\H,18,1.1065081
1,17,108.54953379,3,164.30900571,0\H,18,1.09385493,17,111.57828708,3,4
6.80807893,0\H,19,1.09645748,18,141.91110371,17,128.35475717,0\H,19,1.
10609817,18,92.87357379,17,-108.59723194,0\H,20,1.09936127,19,109.7987
0545,18,-149.61976195,0\H,20,1.10032782,19,108.73972096,18,93.14630078
,0\H,21,1.10185023,20,109.03076091,19,-66.69518393,0\H,21,1.0984168,20
,110.63470794,19,176.33238991,0\C,1,4.08351759,4,109.16341346,3,-49.02
206003,0\C,32,1.53665229,1,22.57777167,4,-65.90483289,0\C,33,2.4156751
2,32,92.05343789,1,-43.86062287,0\C,34,1.53709595,33,91.95175661,32,0.
02085863,0\C,32,1.53757336,1,102.16628955,4,178.49510234,0\H,32,1.0997
536,1,93.64304659,4,67.38559433,0\H,32,1.09915237,1,131.25039691,4,-49
.56693761,0\H,33,1.1091361,32,109.04023885,1,-135.42708237,0\H,33,1.09
325886,32,110.5826307,1,107.01607369,0\H,34,1.09359207,33,143.93194234
,32,129.29048449,0\H,34,1.10916459,33,91.02810268,32,-109.13824545,0\H
,35,1.09960099,34,108.60013131,33,93.86532118,0\H,35,1.09910424,34,109

.37300447,33,-149.19018433,0\H,36,1.10144756,32,109.05805804,1,89.4178
3942,0\H,36,1.09852784,32,110.80330113,1,-153.3329137,0\N,19,1.4637442
5,18,33.31342851,17,129.67264409,0\N,34,1.47336166,33,35.0000351,32,12
3.95417168,0\N,4,1.46654193,3,33.54749803,2,127.80611902,0\\Version=AM
64L-G03RevD.01\State=1-A\HF=-1089.6457177\MP2=-1092.5979377\RMSD=1.300
e-09\Thermal=0.\PG=C01 [X(C15H30N3P1)]\\@

156-Me^{+}

1\1\GINC-NODE3\SP\RMP2-FC\6-31+G(2d,p)\C16H33N3P1(1+)\ZIP07\02-Apr-201
0\0\\#p MP2(FC)/6-31+G(2d,p) scf=tight\\dpia3mesp_15\\1,1\P\C,1,1.8253
6155\H,2,1.09432945,1,110.07051358\H,2,1.09431587,1,110.17822578,3,-11
9.90567481,0\H,2,1.09437498,1,110.24775124,3,120.01613853,0\C,1,3.9216
6795,2,111.64795876,4,18.6908026,0\C,6,1.5353528,1,32.70555356,2,168.3
4092152,0\C,7,2.48287237,6,90.86906807,1,-54.41449923,0\C,8,1.53577148
,7,90.99307992,6,-0.12476257,0\C,9,1.53857953,8,111.01770394,7,-27.235
01295,0\H,6,1.0992105,1,83.02171759,2,-48.91967329,0\H,6,1.0974517,1,1
37.58319946,2,-157.03068374,0\H,7,1.10212853,6,109.87622298,1,-147.222
94932,0\H,7,1.0936248,6,110.97014852,1,93.9175923,0\H,8,1.09473198,7,1
42.54289473,6,127.33778455,0\H,8,1.10173689,7,92.24029626,6,-110.31570
302,0\H,9,1.09921576,8,108.97532802,7,94.00660157,0\H,9,1.0972825,8,10
8.78586701,7,-149.33040136,0\H,10,1.1004701,9,109.40965473,8,-66.81533
215,0\H,10,1.09618995,9,110.20971586,8,176.17359517,0\C,1,3.92345071,2
,111.73188367,8,76.17856446,0\C,21,1.53530344,1,32.61493369,2,168.3691
0858,0\C,22,2.48462844,21,90.8099985,1,-54.23149703,0\C,23,1.53551807,
22,90.96732144,21,-0.15799298,0\C,24,1.53851619,23,111.01529603,22,-27
.26485696,0\H,21,1.09915867,1,83.14576316,2,-48.98671666,0\H,21,1.0974
3219,1,137.51355393,2,-157.21599915,0\H,22,1.10215354,21,109.87310432,
1,-147.22881512,0\H,22,1.09355419,21,110.94058092,1,93.87293213,0\H,23
,1.09479445,22,142.47599814,21,127.03148742,0\H,23,1.10180255,22,92.44
29534,21,-110.31526459,0\H,24,1.09915893,23,109.00338144,22,94.0149040
9,0\H,24,1.09729188,23,108.73994318,22,-149.33812171,0\H,25,1.10046753
,24,109.39234714,23,-66.67705147,0\H,25,1.09618903,24,110.23173548,23,
176.27787618,0\C,1,3.92726733,2,111.67348553,23,76.35009658,0\C,36,1.5
3530041,1,32.5548789,2,168.68916108,0\C,37,2.4834702,36,90.82166892,1,
-54.07781313,0\C,38,1.53571292,37,90.98030027,36,-0.14776701,0\C,39,1.
53832523,38,111.014759,37,-27.26786262,0\H,36,1.09915793,1,83.23784141
,2,-48.80977672,0\H,36,1.09742494,1,137.50197874,2,-157.14955794,0\H,3
7,1.10223508,36,109.85942771,1,-147.00522704,0\H,37,1.09353218,36,110.
91771478,1,94.14037214,0\H,38,1.09488502,37,142.5516098,36,127.1161836
7,0\H,38,1.10183615,37,92.36842709,36,-110.3226559,0\H,39,1.09918104,3
8,108.99551465,37,94.00627362,0\H,39,1.09729072,38,108.74409515,37,-14
9.35452335,0\H,40,1.1004348,39,109.39170803,38,-66.70939604,0\H,40,1.0
9619069,39,110.24084445,38,176.24578394,0\N,22,1.48215071,21,110.36603
715,1,-26.88141507,0\N,7,1.48193491,6,110.34692039,1,-26.93615915,0\N,
37,1.48222674,36,110.3731895,1,-26.66237036,0\\Version=AM64L-G03RevD.0
1\State=1-A\HF=-1129.1206744\MP2=-1132.2118871\RMSD=2.407e-09\Thermal=
0.\PG=C01 [X(C16H33N3P1)]\\@

157

1\1\GINC-NAUTILUS\SP\RMP2-FC\6-31+G(2d,p)\C12H24N3P1\CHRISTOPH\09-Oct-
2009\0\\#p MP2(FC)/6-31+g(2d,p) scf=tight\\py3sp_9\\0,1\P\C,1,2.752815
39\C,2,1.53810886,1,118.75997541\C,3,1.54481396,2,102.27763553,1,-69.8
6680718,0\C,4,1.55927639,3,104.72977731,2,29.21560826,0\H,2,1.10399194
,1,123.23485042,5,-91.13538204,0\H,2,1.0954109,1,78.00566074,5,165.522
92746,0\H,3,1.09662131,2,113.33650243,1,168.33399216,0\H,3,1.09820659,
2,109.49949143,1,47.41602901,0\H,4,1.09731961,3,110.03950242,2,-89.023
27028,0\H,4,1.0959667,3,112.62056841,2,151.36805097,0\H,5,1.10083391,4
,110.47189011,3,-129.91564493,0\H,5,1.09380903,4,112.53468764,3,110.65

818829,0\C,1,2.88442251,2,111.47957787,3,4.91921145,0\C,14,1.54734474,
1,127.5993049,2,-132.55358912,0\C,15,1.55873995,14,104.47972684,1,40.5
4323627,0\C,16,1.55326156,15,104.82659125,14,-5.01393459,0\H,14,1.1059
1506,1,106.93797338,2,0.12840402,0\H,14,1.09401025,1,87.77903298,2,108
.45471476,0\H,15,1.09622021,14,109.97786018,1,-77.87025537,0\H,15,1.09
615566,14,111.86274197,1,162.69651318,0\H,16,1.09546488,15,110.6138408
5,14,-124.30843651,0\H,16,1.09598238,15,112.2162547,14,115.73345192,0\
H,17,1.09557737,16,113.47823177,15,-139.46655814,0\H,17,1.10599868,16,
109.68233225,15,100.12223603,0\C,1,2.73523517,2,150.45879973,3,106.740
11472,0\C,26,1.54588153,1,138.37011584,2,-5.83474429,0\C,27,1.55685862
,26,104.36884417,1,18.57713713,0\C,28,1.54509507,27,104.41275649,26,0.
54763173,0\H,26,1.1076606,1,89.06136159,29,99.53328801,0\H,26,1.093860
74,1,94.08413995,29,-152.97839207,0\H,27,1.09595312,26,111.34418186,1,
140.4050526,0\H,27,1.09586561,26,110.45629306,1,-100.26716467,0\H,28,1
.09578971,27,110.50368605,26,-118.06383937,0\H,28,1.09598753,27,112.69
246128,26,121.62733977,0\H,29,1.09331986,28,113.28443437,27,-146.41055
848,0\H,29,1.10756555,28,109.96239987,27,93.14797638,0\N,29,1.47342167
,28,103.44510497,27,-26.37461879,0\N,17,1.46330688,16,104.03797138,15,
-19.34304618,0\N,2,1.46743805,1,32.71905426,39,-89.0820275,0\\Version=
AM64L-G03RevD.01\State=1-A\HF=-972.511018\MP2=-974.9928696\RMSD=1.419e
-09\Thermal=0.\PG=C01 [X(C12H24N3P1)]\\@

157-Me⁺

1\1\GINC-YANG\SP\RMP2-FC\6-31+G(2d,p)\C13H27N3P1(1+)\CHRISTOPH\13-Oct-
2009\0\\#p MP2(FC)/6-31+g(2d,p) scf=tight\\py3mesp_27\\1,1\P\C,1,1.816
80856\H,2,1.09510259,1,112.10607057\H,2,1.09537929,1,108.45413695,3,-1
20.14272002,0\H,2,1.09543555,1,110.030654,4,-118.65422631,0\C,1,2.7374
6085,2,133.17298085,3,-55.54947646,0\C,6,1.53842112,1,133.14843216,2,-
32.33039245,0\C,7,1.53856142,6,103.26740291,1,41.04211017,0\C,8,1.5375
4531,7,103.42085718,6,-39.33635328,0\H,6,1.09669542,1,87.82831193,2,-1
51.68924462,0\H,6,1.09852291,1,100.057735,2,100.10893006,0\H,7,1.09405
479,6,111.94304242,1,163.14525208,0\H,7,1.09728065,6,110.0760544,1,-76
.73397918,0\H,8,1.09688937,7,110.37619701,6,78.2016327,0\H,8,1.0941239
8,7,113.17898495,6,-160.4724559,0\H,9,1.09913941,8,111.03351099,7,-85.
94382492,0\H,9,1.09453971,8,112.3469899,7,152.30072006,0\C,1,2.7272533
9,2,86.84085539,9,110.4378283,0\C,18,1.53972094,1,133.99114932,2,156.0
1770737,0\C,19,1.5380399,18,103.47915317,1,20.10839163,0\C,20,1.535052
97,19,102.77736859,18,-39.86168418,0\H,18,1.09538228,1,99.26024259,2,2
2.10260487,0\H,18,1.0994766,1,87.9819897,2,-86.42881198,0\H,19,1.09407
355,18,111.70785998,1,142.4441909,0\H,19,1.09666357,18,109.9907433,1,-
97.64885962,0\H,20,1.09787577,19,110.49955651,18,77.77596237,0\H,20,1.
09435294,19,113.14437564,18,-160.84931863,0\H,21,1.09742297,20,110.859
57984,19,-83.24259893,0\H,21,1.09397194,20,113.50568037,19,154.7229450
8,0\C,1,2.77092817,2,103.08975136,9,-139.52763905,0\C,30,1.5376773,1,1
27.64757469,2,74.33474189,0\C,31,1.53832871,30,103.11422565,1,50.71339
838,0\C,32,1.53742761,31,103.24117566,30,-39.87206153,0\H,30,1.0957049
5,1,84.12201022,2,-40.18011525,0\H,30,1.09699285,1,108.9579491,2,-147.
29115091,0\H,31,1.09425895,30,111.916602,1,172.69549879,0\H,31,1.09735
688,30,110.24131144,1,-67.09777937,0\H,32,1.09413385,31,113.28557696,3
0,-160.98677423,0\H,32,1.09695087,31,110.39915857,30,77.64425295,0\H,3
3,1.09999712,32,110.62318607,31,-85.57583111,0\H,33,1.09471235,32,112.
87879165,31,152.8410469,0\N,33,1.49271163,32,103.22543304,31,33.319757
56,0\N,21,1.49487872,20,103.69007003,19,33.71290771,0\N,6,1.49074742,1
,30.93014881,43,-133.80274367,0\\Version=AM64L-G03RevD.01\State=1-A\HF
=-1011.9939518\MP2=-1014.6155883\RMSD=3.230e-09\Thermal=0.\PG=C01 [X(C
13H27N3P1)]\\@

158

1\1\GINC-EDDY\SP\RMP2-FC\6-31+G(2d,p)\C9H21N4P1\CHRISTOPH\21-Jan-2010\
0\\#p MP2(FC)/6-31+g(2d,p) scf=tight\\flosp_2\\0,1\C\P,1,2.80565198\H,
1,1.09950004,2,136.7440446\H,1,1.10279075,2,86.47789566,3,110.34002833
,0\C,2,2.82423131,1,78.93238518,3,-166.2951237,0\H,5,1.10042506,2,135.
61544443,1,116.52590883,0\H,5,1.09929226,2,86.37084086,1,-133.45259546
,0\C,2,2.81675811,1,78.16115358,5,-80.08939322,0\H,8,1.0982166,2,85.79
677054,1,-52.97540954,0\H,8,1.1006833,2,135.48063256,1,-163.12787844,0
\C,8,1.54655036,2,106.94906318,1,54.71360069,0\H,11,1.10621787,8,107.1
3925611,2,-149.66022343,0\N,11,1.45040818,8,115.62098601,2,-27.1254988
7,0\C,13,1.44874829,11,119.25145803,8,102.45043381,0\H,14,1.10609886,1
3,109.74897413,11,61.56144182,0\C,13,1.4482474,11,119.07501039,8,-59.1
0742919,0\H,16,1.09832309,13,107.6952063,11,-22.21717195,0\H,11,1.0984
5466,8,109.94938239,2,95.11512244,0\H,14,1.09848184,13,107.77330137,11
,177.19340009,0\H,16,1.10588171,13,109.46919698,11,-137.72149987,0\C,1
,2.46352201,16,133.55111842,13,-91.69702845,0\H,21,1.1045637,1,92.8651
0969,16,-163.47761355,0\H,21,1.09545519,1,142.20610883,16,72.22458048,
0\H,21,1.09933751,1,94.83456416,16,-55.1446598,0\C,8,2.49244075,2,61.1
9012401,1,168.49975451,0\H,25,1.09752571,8,139.56959707,2,16.32070726,
0\H,25,1.10118082,8,89.45275677,2,133.76277372,0\H,25,1.10344324,8,101
.07811755,2,-118.23026125,0\C,5,2.4897449,2,61.30727781,1,90.35606259,
0\H,29,1.09782184,5,139.58677535,2,13.37114178,0\H,29,1.1028683,5,100.
73941447,2,-120.67122251,0\H,29,1.10170965,5,89.83242168,2,131.2051699
,0\N,25,1.45455092,8,31.156621,2,-1.29946067,0\N,29,1.45488341,5,31.26
168957,2,-3.64567421,0\N,21,1.46308303,1,32.68682457,16,66.0631331,0\\
Version=AM64L-G03RevD.01\State=1-A\HF=-911.5586437\MP2=-913.7929459\RM
SD=3.890e-09\Thermal=0.\PG=C01 [X(C9H21N4P1)]\\@

158-Me$^+$

1\1\GINC-YANG\SP\RMP2-FC\6-31+G(2d,p)\C10H24N4P1(1+)\CHRISTOPH\21-Jan-
2010\0\\#p MP2(FC)/6-31+g(2d,p) scf=tight\\flomesp_1\\1,1\C\H,1,1.0970
3852\H,1,1.09537634,2,106.82876025\C,1,3.76295567,3,129.0697279,2,177.
73995417,0\H,4,1.0954344,1,170.87635905,3,6.77063158,0\H,4,1.09694355,
1,64.28591396,3,-5.48632029,0\C,4,3.76610992,1,60.04959209,3,178.32280
64,0\H,7,1.09703769,4,64.51800823,1,176.28729603,0\H,7,1.09544214,4,17
1.05565968,1,-171.57616684,0\C,7,1.54085254,4,71.66707233,1,-60.114584
86,0\H,10,1.09642425,7,109.83472589,4,135.69572719,0\N,10,1.45430947,7
,108.49084305,4,16.84083334,0\C,12,1.45437094,10,118.52164965,7,-78.41
957095,0\H,13,1.10129205,12,112.56021046,10,-112.2517352,0\C,12,1.4544
6198,10,118.51823559,7,125.75576785,0\H,15,1.1011459,12,112.54267335,1
0,43.5261511,0\H,10,1.10122513,7,110.07211602,4,-106.68217007,0\H,13,1
.09643315,12,108.76070477,10,6.20027354,0\H,15,1.09656104,12,108.77692
482,10,162.00131241,0\C,1,2.50269507,15,114.63736867,12,-86.18921347,0
\H,20,1.0960883,1,94.5392094,15,-146.64848033,0\H,20,1.09803196,1,91.6
0327491,15,-37.57594032,0\H,20,1.09267629,1,142.57913893,15,85.1743475
3,0\C,7,2.50129342,4,109.0423358,1,50.32349607,0\H,24,1.09806099,7,91.
84442686,4,-114.94224974,0\H,24,1.09259234,7,142.75937462,4,8.43702627
,0\H,24,1.09598844,7,94.08741863,4,136.03517547,0\C,4,2.50141385,1,109
.02015293,15,137.0718266,0\H,28,1.0959289,4,94.50729828,1,136.50766072
,0\H,28,1.09815296,4,91.54553357,1,-114.4312807,0\H,28,1.09264568,4,14
2.5807668,1,8.20465162,0\P,4,2.72862633,1,46.4102233,15,143.9965268,0\
C,32,1.83596953,4,127.14011832,1,136.26874233,0\H,33,1.09444607,32,110
.45235903,4,-22.32569725,0\H,33,1.09442454,32,110.51699495,4,97.681777
89,0\H,33,1.09455226,32,110.43233942,4,-142.23468178,0\N,24,1.47404488
,7,31.97237538,4,14.30435014,0\N,1,1.47388873,15,111.85179841,12,-51.5
1422022,0\N,4,1.4738608,1,78.10691968,38,-37.62880139,0\\Version=AM64L
-G03RevD.01\State=1-A\HF=-951.0381853\MP2=-953.4203797\RMSD=3.337e-09\
Thermal=0.\PG=C01 [X(C10H24N4P1)]\\@

159

1\1\GINC-YIN\SP\RMP2-FC\6-31+G(2d,p)\C6H6N3P1\CHRISTOPH\13-Oct-2009\0\
\#p MP2(FC)/6-31+g(2d,p) scf=tight\\oz3sp_12\\0,1\P\C,1,1.81046511\C,2
,1.46476209,1,150.01294838\N,2,1.26396329,1,140.92533895,3,167.4489876
9,0\H,3,1.08881172,2,119.64168001,1,-87.30970692,0\H,3,1.08941963,2,12
0.55920193,1,71.73586725,0\C,1,1.81049052,2,100.13369697,4,-35.5097373
5,0\C,7,1.4647246,1,150.04777332,2,54.67854236,0\N,7,1.26398103,1,140.
89031408,2,-137.8565263,0\H,8,1.08941958,7,120.56457653,1,71.74886443,
0\H,8,1.08882979,7,119.64771383,1,-87.34457708,0\C,1,1.8104849,2,100.1
0341615,4,-137.82201513,0\N,12,1.26394415,1,140.90101462,2,-35.5078906
8,0\C,12,1.46475445,1,150.03678749,2,157.0226509,0\H,14,1.08881294,12,
119.62542772,1,-87.30636241,0\H,14,1.08945316,12,120.56798055,1,71.750
3986,0\\Version=AM64L-G03RevD.01\State=1-A\HF=-734.5566455\MP2=-736.04
86751\RMSD=9.111e-09\Thermal=0.\PG=C01 [X(C6H6N3P1)]\\@

159-Me$^+$

1\1\GINC-CALYPSO\SP\RMP2-FC\6-31+G(2d,p)\C7H9N3P1(1+)\CHRISTOPH\13-Oct
-2009\0\\#p MP2(FC)/6-31+g(2d,p) scf=tight\\oz3mesp_16\\1,1\C\C,1,1.47
034688\N,1,1.26249312,2,68.49232984\H,2,1.08734362,1,119.8450597,3,99.
56032409,0\H,2,1.08737543,1,119.876064,3,-99.50638201,0\P,1,1.79243843
,3,134.86885089,2,179.84621536,0\C,6,1.81129686,1,111.74076234,3,-125.
94210156,0\H,7,1.09559257,6,109.18491284,1,63.33475246,0\H,7,1.0955672
2,6,109.26043994,1,-57.41197347,0\H,7,1.09590577,6,108.79823809,1,-176
.99557688,0\C,6,1.79210515,1,108.07044577,3,113.44211775,0\N,11,1.2633
156,6,133.38980686,1,119.2743845,0\C,11,1.46762273,6,157.93435337,1,-6
0.54969457,0\H,13,1.08726681,11,119.83660509,6,-80.68585992,0\H,13,1.0
8731029,11,119.96541118,6,80.44723686,0\C,6,1.78957632,1,107.19652254,
3,-4.39662691,0\N,16,1.26354017,6,133.32807758,1,-119.96017215,0\C,16,
1.46740702,6,157.90187189,1,54.40262426,0\H,18,1.08715004,16,119.69316
377,6,-75.95259548,0\H,18,1.08722722,16,119.78695441,6,84.92400393,0\\
Version=AM64L-G03RevD.01\State=1-A\HF=-773.9325174\MP2=-775.5709074\RM
SD=8.758e-09\Thermal=0.\PG=C01 [X(C7H9N3P1)]\\@

160

1\1\GINC-AZAZEL\SP\RMP2-FC\6-31+G(2d,p)\C9H12N9P1\CHRISTOPH\25-Nov-200
9\0\\#p MP2(FC)/6-31+g(2d,p) scf=tight\\tre3sp_15\\0,1\P\C,1,1.8243458
2\C,2,1.38984609,1,133.0495913\N,3,1.35888764,2,109.2613854,1,-171.859
96668,0\N,2,1.36793035,1,123.24586415,3,-169.59739055,0\C,1,1.82936488
,2,100.83365981,5,75.81142237,0\C,6,1.390529,1,134.39123334,2,2.053105
48,0\N,7,1.35883178,6,109.31949127,1,-173.87098344,0\N,6,1.36793995,1,
122.2909318,2,-170.34743013,0\C,1,1.81835566,2,102.13781976,5,-177.681
4892,0\N,10,1.37231631,1,131.75353474,2,-62.7803899,0\N,11,2.15545311,
10,75.40865251,1,-178.57366055,0\C,12,1.35620665,11,71.94335719,10,0.1
4730434,0\H,3,1.08141044,2,129.11369295,1,6.58729768,0\H,13,1.08203614
,12,121.88120868,11,-179.66983653,0\H,7,1.08188652,6,128.98082625,1,6.
31925684,0\C,5,1.45528163,2,129.61070837,1,-6.54134502,0\H,17,1.091302
32,5,106.84852145,2,174.04329184,0\H,17,1.094862,5,110.74582329,2,-66.
31672783,0\H,17,1.09536358,5,110.0212101,2,54.938725,0\C,9,1.45564944,
6,129.29257816,1,-6.33324918,0\H,21,1.0912872,9,106.91980095,6,176.078
02585,0\H,21,1.09514145,9,110.60705906,6,-64.41021929,0\H,21,1.0949609
3,9,110.10282497,6,56.69120003,0\C,11,1.45791406,10,130.82730906,1,-0.
75622361,0\H,25,1.09296369,11,110.27972122,10,53.46024445,0\H,25,1.091
30264,11,106.53934453,10,172.83708548,0\H,25,1.09380325,11,110.5970554
4,10,-67.72697315,0\N,12,1.31024825,11,36.4655835,10,179.82416002,0\N,
4,1.30696272,3,108.9138703,2,0.19513183,0\N,8,1.30869757,7,108.8483396
8,6,-0.13056487,0\\Version=AM64L-G03RevD.01\State=1-A\HF=-1178.4550706
\MP2=-1181.5094205\RMSD=2.522e-09\Thermal=0.\PG=C01 [X(C9H12N9P1)]\\@

160-Me^{+}
1\1\GINC-AZAZEL\SP\RMP2-FC\6-31+G(2d,p)\C10H15N9P1(1+)\CHRISTOPH\30-No
v-2009\0\\#p MP2(FC)/6-31+g(2d,p) scf=tight\\tre3mesp_1\\1,1\P\C,1,1.7
778297\C,2,1.39505548,1,128.53664587\N,3,1.35103557,2,108.65146745,1,-
179.88250778,0\N,2,1.37600136,1,127.64433878,3,-179.94145498,0\C,1,1.7
79729,2,108.8997557,5,72.32795355,0\C,6,1.39371937,1,127.80176593,2,-1
.50508805,0\N,7,1.35248751,6,108.62076791,1,179.55044337,0\N,6,1.37547
531,1,128.30781056,2,177.64089677,0\C,1,1.78269102,2,111.31428173,5,-4
6.65352127,0\N,10,1.3747652,1,127.42956683,2,-65.03237318,0\N,11,2.155
26006,10,74.80129014,1,175.85553221,0\C,12,1.35228089,11,72.76059637,1
0,-0.37525227,0\C,1,1.82201257,2,106.86782509,5,-168.55059898,0\H,14,1
.09511189,1,109.25926353,2,175.73416006,0\H,14,1.09566189,1,109.066513
52,2,-65.02741975,0\H,14,1.09448174,1,110.65313997,2,55.49828313,0\H,3
,1.08196704,2,129.91580189,1,0.49531396,0\H,7,1.08200723,6,129.5949286
,1,0.15539611,0\H,13,1.08224791,12,121.7349168,11,-178.89997145,0\C,9,
1.46293201,6,131.53591834,1,-3.72474199,0\H,21,1.09129074,9,106.585163
65,6,170.18943248,0\H,21,1.09500765,9,110.32511541,6,-71.44510676,0\H,
21,1.09321939,9,110.48503011,6,51.10387285,0\C,5,1.46425249,2,131.4312
9566,1,1.2354613,0\H,25,1.09110292,5,106.30341744,2,-176.17718349,0\H,
25,1.09372224,5,110.41335345,2,-57.25800056,0\H,25,1.09372036,5,110.41
948524,2,64.88202779,0\C,11,1.46253076,10,131.25705629,1,-4.79262684,0
\H,29,1.09441524,11,110.30519209,10,51.44222013,0\H,29,1.09125343,11,1
06.58398333,10,170.21493606,0\H,29,1.09461758,11,110.48774057,10,-71.2
4880106,0\N,12,1.30873834,11,36.15169934,10,179.59006188,0\N,4,1.30971
795,3,108.87861164,2,-0.03613988,0\N,8,1.30769197,7,108.93644367,6,-0.
34073499,0\\Version=AM64L-G03RevD.01\State=1-A\HF=-1217.8476095\MP2=-1
221.0465085\RMSD=6.180e-09\Thermal=0.\PG=C01 [X(C10H15N9P1)]\\@

161
1\1\GINC-CALYPSO\SP\RMP2-FC\6-31+G(2d,p)\C5H7N2P1\CHRISTOPH\13-Oct-200
9\0\\#p MP2(FC)/6-31+g(2d,p) scf=tight\\oz2sp_23\\0,1\P\C,1,1.85823758
\C,1,1.80813899,2,101.50859639\C,3,1.46652099,1,151.54480606,2,162.113
62586,0\N,3,1.26627467,1,139.43828303,2,-34.25887411,0\H,4,1.08984017,
3,119.90701715,1,-90.34397085,0\H,4,1.09000435,3,120.76204701,1,68.762
35748,0\C,1,1.80813521,3,98.54381103,5,-137.94047597,0\C,8,1.46652077,
1,151.54220177,3,-58.44411645,0\N,8,1.26627316,1,139.44038064,3,137.92
797667,0\H,9,1.08983955,8,119.90671785,1,90.34401028,0\H,9,1.09000419,
8,120.76240988,1,-68.76349003,0\H,2,1.09522522,1,108.37909661,8,171.71
64997,0\H,2,1.09522192,1,108.38169939,8,-70.46379749,0\H,2,1.09366216,
1,112.38615713,8,50.62750144,0\\Version=AM64L-G03RevD.01\State=1-A\HF=
-642.9081685\MP2=-644.1022033\RMSD=7.847e-09\Thermal=0.\PG=C01 [X(C5H7
N2P1)]\\@

161-Me^{+}
1\1\GINC-YANG\SP\RMP2-FC\6-31+G(2d,p)\C6H10N2P1(1+)\CHRISTOPH\13-Oct-2
009\0\\#p MP2(FC)/6-31+g(2d,p) scf=tight\\oz2mesp_1\\1,1\C\C,1,1.46844
546\N,1,1.26310477,2,68.60269823\H,2,1.08738891,1,119.99430998,3,99.46
149462,0\H,2,1.08738086,1,119.96858521,3,-99.39594562,0\P,1,1.79335875
,3,134.07393915,2,179.71141319,0\C,6,1.81284491,1,107.77387838,3,-2.30
248873,0\H,7,1.09564816,6,109.68762377,1,-179.95361848,0\H,7,1.0960229
7,6,109.16538611,1,-59.64934026,0\H,7,1.09546932,6,109.39726121,1,59.5
5669991,0\C,6,1.7933588,1,107.48730989,3,116.29671625,0\N,11,1.2631041
4,6,134.07424297,1,116.29923534,0\C,11,1.46844224,6,157.32154385,1,-64
.39899041,0\H,13,1.08738862,11,119.99287042,6,-79.99624409,0\H,13,1.08
738112,11,119.96877011,6,81.1431012,0\C,6,1.81284347,1,110.06198678,3,
-126.58782206,0\H,16,1.09564803,6,109.68773419,1,63.10714442,0\H,16,1.
09602304,6,109.16530457,1,-176.58865064,0\H,16,1.09546868,6,109.397274
57,1,-57.3826357,0\\Version=AM64L-G03RevD.01\State=1-A\HF=-682.3032797

\MP2=-683.6429327\RMSD=3.818e-09\Thermal=0.\PG=C01 [X(C6H10N2P1)]\\@

162

1\1\GINC-YIN\SP\RMP2-FC\6-31+G(2d,p)\C7H11N6P1\CHRISTOPH\25-Nov-2009\0
\\#p MP2(FC)/6-31+g(2d,p) scf=tight\\tre2sp_9\\0,1\P\C,1,1.82736224\C,
2,1.38995761,1,125.32661469\N,3,1.3574667,2,109.85434459,1,-179.161754
14,0\N,2,1.37177503,1,131.95401574,3,-178.71455215,0\C,1,1.82970089,2,
102.47736451,5,-48.24893112,0\C,6,1.39090533,1,133.48261584,2,113.1553
5849,0\N,7,1.35973859,6,109.45714749,1,173.5144531,0\N,6,1.36719543,1,
123.31822137,2,-75.045982,0\C,1,1.86066495,2,103.26950456,5,55.0763636
9,0\H,3,1.08209374,2,128.32195348,1,0.69374726,0\H,7,1.08150262,6,129.
23607994,1,-5.74686816,0\C,5,1.45553592,2,130.63110497,1,-2.51187546,0
\H,13,1.09148101,5,106.89670279,2,166.3581817,0\H,13,1.09292977,5,110.
22342512,2,46.70036442,0\H,13,1.09555017,5,110.59152821,2,-74.80922984
,0\C,9,1.45465596,6,129.16043278,1,6.12065093,0\H,17,1.09518429,9,110.
00466057,6,-56.02715542,0\H,17,1.09499987,9,110.6578599,6,64.95831124,
0\H,17,1.09136437,9,106.94111321,6,-175.37623943,0\H,10,1.09614603,1,1
08.46156814,2,-178.12729365,0\H,10,1.09501782,1,114.33272924,2,-56.435
57958,0\H,10,1.0954886,1,107.97715261,2,65.05374838,0\N,4,1.30970581,3
,108.3757124,2,0.07342178,0\N,8,1.30762391,7,108.79773644,6,-0.0053689
8,0\\Version=AM64L-G03RevD.01\State=1-A\HF=-938.8362601\MP2=-941.07061
99\RMSD=4.935e-09\Thermal=0.\PG=C01 [X(C7H11N6P1)]\\@

162-Me$^+$

1\1\GINC-GOLEM\SP\RMP2-FC\6-31+G(2d,p)\C8H14N6P1(1+)\CHRISTOPH\25-Nov-
2009\0\\#p MP2(FC)/6-31+g(2d,p) scf=tight\\tre2mesp_7\\1,1\C\C,1,1.394
43596\N,2,1.35217672,1,108.69266337\N,1,1.37499352,2,103.73687157,3,0.
07573348,0\C,1,2.94550274,4,103.98659481,3,-150.93004518,0\C,5,1.39443
284,1,140.00963516,4,-49.22934188,0\N,6,1.35217815,5,108.69262318,1,13
2.78657372,0\N,5,1.37500502,1,103.97580906,4,83.4164804,0\C,1,2.894472
27,4,164.11168436,3,-171.437641,0\H,2,1.08201194,1,129.88363719,4,-179
.87073594,0\H,6,1.08201217,5,129.88433895,1,-47.16284822,0\C,4,1.46240
283,1,131.19793008,2,-178.49906535,0\H,12,1.09481815,4,110.41956259,1,
69.30544331,0\H,12,1.09121674,4,106.52239964,1,-172.24308663,0\H,12,1.
09368293,4,110.43309077,1,-53.19237764,0\C,8,1.46239621,5,131.19666045
,1,30.61499062,0\H,16,1.09368341,8,110.4324574,5,-53.21006726,0\H,16,1
.09481534,8,110.42013445,5,69.28839177,0\H,16,1.09121793,8,106.5231396
8,5,-172.25946232,0\P,1,1.7800228,4,127.35818524,12,2.57092077,0\C,20,
1.81927412,1,111.45788807,4,-65.35818508,0\H,21,1.09576522,20,108.4981
4342,1,-56.36973752,0\H,21,1.0943871,20,110.83359192,1,63.32341988,0\H
,21,1.09587602,20,109.85930364,1,-175.72353008,0\H,9,1.09576377,1,144.
49347487,4,-7.96993634,0\H,9,1.09438239,1,90.3018301,4,116.7178516,0\H
,9,1.09588212,1,90.81693861,4,-133.92820039,0\N,3,1.30887896,2,108.891
46966,1,0.02868091,0\N,7,1.30888295,6,108.89179167,5,0.02788397,0\\Ver
sion=AM64L-G03RevD.01\State=1-A\HF=-978.2443654\MP2=-980.622587\RMSD=8
.887e-09\Thermal=0.\PG=C01 [X(C8H14N6P1)]\\@

163

1\1\GINC-YANG\SP\RMP2-FC\6-31+G(2d,p)\C4H8N1P1\CHRISTOPH\13-Oct-2009\0
\\#p MP2(FC)/6-31+g(2d,p) scf=tight\\oz1sp_9\\0,1\P\C,1,1.80932041\C,2
,1.46977179,1,150.25256127\N,2,1.26738208,1,141.12492491,3,165.5070516
4,0\H,3,1.090598,2,120.15889395,1,-88.88636544,0\H,3,1.09050452,2,120.
83870261,1,70.30306903,0\C,1,1.86142592,2,99.24808389,4,-31.49512227,0
\C,1,1.86485058,2,99.07320701,4,-135.29302799,0\H,7,1.09622393,1,109.0
6874216,2,-175.65603843,0\H,7,1.09589285,1,112.64590014,2,-54.03847214
,0\H,7,1.09564111,1,108.82042119,2,66.57497419,0\H,8,1.09580234,1,109.
14377897,2,-64.83778831,0\H,8,1.09616077,1,108.59039301,2,177.77058814
,0\H,8,1.09619913,1,113.03990086,2,56.72663048,0\\Version=AM64L-G03Rev

D.01\State=1-A\HF=-551.251732\MP2=-552.1486338\RMSD=3.987e-09\Thermal=
0.\PG=C01 [X(C4H8N1P1)]\\@

163-Me⁺

1\1\GINC-CALYPSO\SP\RMP2-FC\6-31+G(2d,p)\C5H11N1P1(1+)\CHRISTOPH\13-Oc
t-2009\0\\#p MP2(FC)/6-31+g(2d,p) scf=tight\\oz1mesp_11\\1,1\C\C,1,1.4
7000941\N,1,1.26308075,2,68.40615952\H,2,1.08760806,1,120.03931415,3,-
99.49027662,0\H,2,1.08760444,1,120.04517738,3,99.49637008,0\P,1,1.7935
371,3,134.77672936,2,179.98707876,0\C,6,1.81360416,1,107.33813203,3,-0
.10582499,0\H,7,1.09554567,6,109.63639762,1,-59.79891099,0\H,7,1.09555
86,6,109.63227307,1,59.57633997,0\H,7,1.09590267,6,109.77825621,1,179.
88798137,0\C,6,1.81594263,1,108.31855562,3,120.136481,0\C,6,1.81591725
,1,108.32638116,3,-120.36074639,0\H,12,1.09552878,6,109.85021703,1,61.
70727107,0\H,12,1.09636542,6,109.52380368,1,-178.38344467,0\H,12,1.095
60844,6,109.89709455,1,-58.49896774,0\H,11,1.0956087,6,109.89843033,1,
58.44037771,0\H,11,1.09552759,6,109.84257664,1,-61.76061758,0\H,11,1.0
9636253,6,109.52830255,1,178.32923107,0\\Version=AM64L-G03RevD.01\Stat
e=1-A\HF=-590.6704186\MP2=-591.7121563\RMSD=2.719e-09\Thermal=0.\PG=C0
1 [X(C5H11N1P1)]\\@

164

1\1\GINC-MORITZ\SP\RMP2-FC\6-31+G(2d,p)\C5H10N3P1\CHRISTOPH\25-Nov-200
9\0\\#p MP2(FC)/6-31+g(2d,p) scf=tight\\tre1sp_2\\0,1\P\C,1,1.83207934
\C,2,1.39019685,1,134.45081764\N,3,1.3588631,2,109.70511381,1,-177.095
30622,0\N,2,1.36844304,1,122.89658129,3,-176.30812384,0\C,1,1.86791983
,2,100.46248135,5,98.67665348,0\C,1,1.86078051,2,99.52646376,5,-159.33
68132,0\H,3,1.08143085,2,129.10822163,1,2.13619169,0\C,5,1.45406786,2,
129.32453504,1,-3.47882999,0\H,9,1.09400942,5,109.94439688,2,48.728035
77,0\H,9,1.0958727,5,110.85969775,2,-72.21248838,0\H,9,1.09155732,5,10
7.03517147,2,168.45901669,0\H,7,1.09655106,1,108.36055791,2,-174.56274
314,0\H,7,1.09701708,1,113.1790629,2,-54.22470175,0\H,7,1.0955296,1,10
9.66237545,2,67.79472342,0\H,6,1.09717483,1,108.79533629,2,172.4927854
6,0\H,6,1.09620111,1,109.70010649,2,-69.90397419,0\H,6,1.09656256,1,11
2.55560739,2,51.36474191,0\N,4,1.3094408,3,108.70239258,2,-0.15089851,
0\\Version=IA32L-G03RevD.01\State=1-A\HF=-699.2148224\MP2=-700.6304442
\RMSD=5.268e-09\Thermal=0.\PG=C01 [X(C5H10N3P1)]\\@

164-Me⁺

1\1\GINC-MAX\SP\RMP2-FC\6-31+G(2d,p)\C6H13N3P1(1+)\CHRISTOPH\25-Nov-20
09\0\\#p MP2(FC)/6-31+g(2d,p) scf=tight\\tre1mesp_9\\1,1\C\C,1,2.89326
554\C,2,1.39350444,1,91.33056823\N,3,1.3527785,2,108.77506259,1,178.79
88477,0\N,2,1.3745122,1,164.96041625,3,175.70426275,0\C,1,2.95457152,2
,61.21975808,5,28.35283217,0\H,3,1.08178018,2,129.86092415,1,-1.221927
44,0\C,5,1.46055654,2,131.50890753,1,3.77621307,0\H,8,1.09448017,5,110
.66112832,2,59.45595273,0\H,8,1.09453755,5,110.64792034,2,-63.57661007
,0\H,8,1.09133614,5,106.42302823,2,177.92970794,0\P,2,1.78061953,1,36.
8785887,6,34.91801309,0\C,12,1.81990207,2,111.50302395,1,118.90868425,
0\H,13,1.09648725,12,109.46732915,2,-176.18142411,0\H,13,1.09542851,12
,109.61460178,2,-56.29929252,0\H,13,1.09514202,12,110.69759939,2,64.03
27403,0\H,6,1.09651787,1,88.28100373,12,-127.61816736,0\H,6,1.09493523
,1,146.29435177,12,-5.55880549,0\H,6,1.09547102,1,91.30757591,12,123.1
6755924,0\H,1,1.09515542,12,110.35985434,2,60.59819219,0\H,1,1.0950917
,12,110.19747773,2,-60.47171741,0\H,1,1.09602475,12,108.93855409,2,-17
9.9546233,0\N,4,1.30679873,3,108.89949361,2,-0.00230899,0\\Version=IA3
2L-G03RevD.01\State=1-A\HF=-738.6378176\MP2=-740.1974705\RMSD=3.364e-0
9\Thermal=0.\PG=C01 [X(C6H13N3P1)]\\@

165

1\1\GINC-CALYPSO\SP\RMP2-FC\6-31+G(2d,p)\C12H15N6P1\CHRISTOPH\17-Feb-2
012\0\\#p MP2(FC)/6-31+g(2d,p) scf=tight\\imi53sp_3\\0,1\P\C,1,1.82968
47\C,2,1.38791813,1,133.82228342\N,3,1.37215942,2,111.47722311,1,173.7
6513431,0\C,4,1.31943966,3,104.65013146,2,0.00141691,0\N,5,1.36403468,
4,112.7689358,3,-0.52006972,0\H,3,1.08349768,2,127.08842772,1,-6.40024
212,0\H,5,1.08389912,4,125.61574385,3,179.11092817,0\C,6,1.45594074,5,
126.29725175,4,179.58245498,0\H,9,1.09538085,6,110.41957946,5,122.1315
9291,0\H,9,1.09551664,6,110.74381646,5,-117.25954819,0\H,9,1.09334493,
6,108.59859887,5,2.56730593,0\C,1,1.81163026,2,104.41584075,3,102.6528
3723,0\C,13,1.38837242,1,124.60901553,2,133.58097735,0\N,14,1.36767981
,13,111.81210002,1,-178.47795641,0\C,15,1.32114382,14,104.27245059,13,
0.31761575,0\N,16,1.3617279,15,113.33255521,14,-0.2396171,0\C,17,1.458
13859,16,125.0823375,15,177.35970494,0\H,18,1.09361931,17,108.17703312
,16,10.15510248,0\H,18,1.093181,17,110.41227422,16,129.48490912,0\H,18
,1.09422184,17,110.85775299,16,-109.84331511,0\H,16,1.08422548,15,125.
31032828,14,179.70028201,0\H,14,1.08372873,13,126.62971264,1,2.1893948
5,0\C,1,1.82339218,13,102.94663818,14,-121.01099759,0\N,24,2.27959932,
1,165.04266123,13,28.34506988,0\C,25,1.31787608,24,70.19543759,1,152.5
4538283,0\N,26,1.36532253,25,112.8065156,24,-0.56250429,0\C,27,1.45532
49,26,126.11873306,25,-178.78413563,0\H,28,1.09331251,27,108.56046003,
26,4.6118109,0\H,28,1.09592424,27,110.22022549,26,123.72472884,0\H,28,
1.09521972,27,110.83047909,26,-115.45892283,0\C,25,1.37184801,24,34.50
347745,1,-27.04975821,0\H,32,1.08283153,25,121.30464264,24,178.2696318
2,0\H,26,1.08382606,25,125.69140837,24,178.97988332,0\\Version=AM64L-G
03RevD.01\State=1-A\HF=-1130.5969884\MP2=-1133.5230684\RMSD=1.723e-09\
Thermal=0.\PG=C01 [X(C12H15N6P1)]\\@

165-Me^{+}

1\1\GINC-GOLEM\SP\RMP2-FC\6-31+G(2d,p)\C13H18N6P1(1+)\CHRISTOPH\19-Feb
-2012\0\\#p MP2(FC)/6-31+g(2d,p) scf=tight\\imi53mesp_1\\1,1\P\C,1,1.8
2686564\H,2,1.09477754,1,109.01924106\H,2,1.09520499,1,109.13149377,3,
119.05365706,0\H,2,1.09408527,1,111.0344135,3,-120.22560299,0\C,1,1.77
307974,2,105.86007713,5,55.7330137,0\N,6,1.39797582,1,127.08602642,2,-
170.45032095,0\C,7,1.35776762,6,105.74443127,1,179.85999103,0\N,8,1.32
434954,7,113.37149151,6,-0.00428542,0\C,9,1.35551241,8,105.07250059,7,
0.08285149,0\C,1,1.77904923,6,112.29583975,10,130.99379569,0\N,11,1.39
705145,1,127.18966871,6,-63.55417137,0\C,12,1.3593897,11,105.90385775,
1,174.67283841,0\N,13,1.32352009,12,113.23540287,11,-0.61356615,0\C,14
,1.35745755,13,105.06533365,12,0.11268065,0\C,1,1.77514148,6,109.23813
312,10,-109.0971507,0\C,16,1.3948256,1,126.47191265,6,-1.00999455,0\N,
17,1.35708736,16,110.87145097,1,179.45098136,0\C,18,1.32251676,17,105.
07990133,16,-0.43064533,0\N,19,1.3602969,18,113.35912433,17,0.42942989
,0\H,15,1.08364611,14,121.61824677,13,-178.66552009,0\H,17,1.08325529,
16,127.49632285,1,0.10227134,0\H,10,1.08337531,9,121.20113509,8,179.53
734628,0\H,19,1.08384199,18,125.18900154,17,-179.53266069,0\H,8,1.0836
7222,7,121.52252745,6,-179.94331618,0\H,13,1.08382375,12,121.57035167,
11,179.30435634,0\C,12,1.46195304,11,128.63981943,1,-6.53674418,0\H,27
,1.09519647,12,110.86839743,11,-70.96724288,0\H,27,1.09476899,12,110.4
3260609,11,51.26600159,0\H,27,1.09230867,12,108.35851711,11,169.959892
35,0\C,20,1.46256583,19,125.10019207,18,-175.01186489,0\H,31,1.0923913
4,20,108.32074993,19,-17.42870594,0\H,31,1.09581905,20,110.73406453,19
,101.59527234,0\H,31,1.09319809,20,110.51171752,19,-136.27394419,0\C,7
,1.46396225,6,128.87453695,1,1.37349113,0\H,35,1.09219655,7,108.040572
07,6,-176.12513591,0\H,35,1.09387149,7,110.57106041,6,-57.14959125,0\H
,35,1.09408475,7,110.7046865,6,64.47128418,0\\Version=AM64L-G03RevD.01
\State=1-A\HF=-1170.0268826\MP2=-1173.0935482\RMSD=4.328e-09\Thermal=0
.\PG=C01 [X(C13H18N6P1)]\\@

166
1\1\GINC-YIN\SP\RMP2-FC\6-31+G(2d,p)\C6H12N3P1\CHRISTOPH\26-Nov-2009\0
\\#p MP2(FC)/6-31+g(2d,p) scf=tight\\padsp_1\\0,1\N\C,1,1.4715416\N,2,
1.47134989,1,114.50818927\C,3,1.47205427,2,111.21267769,1,-67.07435552
,0\P,4,1.89903171,3,114.71440426,2,60.30837786,0\C,1,1.47218119,2,111.
22605897,3,67.02475749,0\H,2,1.09717239,1,109.02989729,6,-55.39481689,
0\H,2,1.0980793,1,107.8943078,6,-172.8369224,0\H,4,1.09864382,3,108.74
275592,2,-177.63948591,0\H,4,1.09864547,3,108.74457562,2,-61.77591817,
0\H,6,1.09864904,1,108.75935168,2,61.846457,0\H,6,1.0986498,1,108.7671
6213,2,177.69934906,0\C,3,1.47147196,2,108.11437148,1,55.29507172,0\H,
13,1.09715578,3,109.01952406,2,-177.68025158,0\H,13,1.09808242,3,107.8
912889,2,64.87560428,0\C,1,1.47165103,2,108.07005284,3,-55.32285017,0\
H,16,1.09716938,1,109.02672617,2,177.76091827,0\H,16,1.0980265,1,107.8
8551382,2,-64.80803593,0\C,5,1.89919974,4,94.71707991,3,47.49532785,0\
H,19,1.09864345,5,108.79987394,4,-169.53127293,0\H,19,1.09868651,5,108
.80303581,4,74.53757169,0\N,16,1.4713419,1,114.48737011,2,55.3258785,0
\\Version=AM64L-G03RevD.01\State=1-A\HF=-738.256728\MP2=-739.8177399\R
MSD=8.028e-09\Thermal=0.\PG=C01 [X(C6H12N3P1)]\\@

166-Me$^+$
1\1\GINC-CALYPSO\SP\RMP2-FC\6-31+G(2d,p)\C7H15N3P1(1+)\CHRISTOPH\26-No
v-2009\0\\#p MP2(FC)/6-31+g(2d,p) scf=tight\\padmesp_1\\1,1\N\C,1,1.47
58491\N,2,1.47589493,1,113.39854502\C,3,1.46361213,2,112.3511341,1,-69
.63128952,0\C,1,1.46344566,2,112.34971844,3,69.61562493,0\H,2,1.095515
5,1,109.62289591,5,-53.26463254,0\H,2,1.09445513,1,107.99839914,5,-170
.77967253,0\H,4,1.09719493,3,110.59768536,2,-177.88691216,0\H,4,1.0971
8716,3,110.52629433,2,-58.63666798,0\H,5,1.09719022,1,110.49997027,2,5
8.63119796,0\H,5,1.09716849,1,110.60666237,2,177.88753233,0\C,3,1.4760
0732,2,108.86815668,1,55.59403857,0\H,12,1.09551156,3,109.70052558,2,-
178.69243033,0\H,12,1.09445528,3,107.90706304,2,63.83846607,0\C,1,1.47
566142,5,112.47898952,2,123.29538051,0\H,15,1.09552514,1,109.67804791,
5,53.58781097,0\H,15,1.09447022,1,107.91755821,5,171.10013737,0\C,12,2
.442161,3,98.79793244,2,-87.34988996,0\H,18,1.09723416,12,144.49503518
,3,122.83346737,0\H,18,1.0971735,12,91.12399906,3,-113.60899647,0\N,18
,1.46340752,12,33.95728351,3,119.61672789,0\P,4,1.86484919,3,107.54274
716,2,61.67803991,0\C,22,1.82035364,4,117.01983272,3,179.96196387,0\H,
23,1.09624189,22,109.91644395,4,179.8957219,0\H,23,1.09624374,22,109.9
5144855,4,-60.12393214,0\H,23,1.09623469,22,109.95218518,4,59.91285579
,0\\Version=AM64L-G03RevD.01\State=1-A\HF=-777.6856717\MP2=-779.388642
8\RMSD=4.430e-09\Thermal=0.\PG=C01 [X(C7H15N3P1)]\\@

167
1\1\GINC-NODE-03\SP\RMP2-FC\6-31+G(2d,p)\C6H6N3P1\ZIP07\21-Oct-2009\0\
\#p MP2(FC)/6-31+g(2d,p) scf=tight\\uz3sp_22\\0,1\P\C,1,1.86121211\C,2
,1.46072564,1,124.70644349\N,3,1.25734075,2,70.00044996,1,-99.01697071
,0\H,2,1.09316622,1,114.14913734,4,-138.65849529,0\C,1,1.85185028,2,98
.21324196,3,-92.80072213,0\C,6,1.46284627,1,122.07229834,2,-123.546539
97,0\N,7,1.25331063,6,71.04400121,1,-98.28080709,0\H,6,1.09274927,1,11
7.75606454,2,40.59375303,0\C,1,1.85196019,6,99.19305453,7,135.47762904
,0\N,10,1.58551437,1,121.15540855,6,38.9239254,0\C,11,1.25556492,10,60
.44904904,1,-109.20149983,0\H,10,1.09185019,1,114.48028661,6,-177.6788
7687,0\H,12,1.08611879,11,139.38890607,10,178.3777407,0\H,7,1.08620775
,6,149.92180204,1,81.94040411,0\H,3,1.0855668,2,150.87725956,1,77.1600
7575,0\\Version=IA32L-G03RevD.01\State=1-A\HF=-734.537159\MP2=-736.032
4044\RMSD=4.640e-09\Thermal=0.\PG=C01 [X(C6H6N3P1)]\\@

167-Me$^+$
1\1\GINC-NODE-17\SP\RMP2-FC\6-31+G(2d,p)\C7H9N3P1(1+)\ZIP07\21-Oct-200

9\0\\#p MP2(FC)/6-31+g(2d,p) scf=tight\\uz3mesp_18\\1,1\C\C,1,1.468962
22\N,2,1.25130613,1,70.10067493\H,1,1.09087941,2,120.41720638,3,99.359
72369,0\H,1,3.25160984,2,121.12278756,3,-154.21740692,0\H,1,3.92362792
,2,129.6059236,3,-60.29854592,0\H,2,1.0855662,1,148.83309955,3,-176.61
62759,0\P,1,1.81422219,2,123.56245878,3,-95.14348765,0\C,8,1.81212557,
1,109.75129589,2,-124.62462147,0\N,9,1.56874819,8,115.28683294,1,-159.
62368157,0\C,10,1.25121463,9,61.59229574,8,111.4421047,0\C,8,1.8121503
7,1,108.27853778,2,117.8724748,0\C,12,1.46805815,8,121.99859856,1,-4.5
0109016,0\N,13,1.25175722,12,69.91244695,8,96.05389698,0\C,8,1.8162854
,1,110.77543883,2,-2.78332721,0\H,15,1.09598742,8,110.04588675,1,-64.1
0886383,0\H,15,1.09592921,8,108.90248033,1,176.90890852,0\H,15,1.09549
572,8,110.21566541,1,57.02564273,0\H,13,1.08486935,12,148.56208565,8,-
85.58925418,0\H,11,1.08484699,10,141.42822656,9,178.50941564,0\\Versio
n=IA32L-G03RevD.01\State=1-A\HF=-773.9705993\MP2=-775.6060648\RMSD=5.5
03e-09\Thermal=0.\PG=C01 [X(C7H9N3P1)]\\@

168

1\1\GINC-TOFU\SP\RMP2-FC\6-31+G(2d,p)\C9H13N4P1\CHRISTOPH\17-Feb-2012\
0\\#p MP2(FC)/6-31+g(2d,p) scf=tight\\imi52sp_5\\0,1\P\C,1,1.86226077\
H,2,1.09619098,1,108.64528763\H,2,1.0954887,1,114.47584973,3,121.65374
077,0\H,2,1.09553721,1,107.86485079,3,-117.03804796,0\C,1,1.81968886,2
,104.09433791,4,-57.47034181,0\C,6,1.38829299,1,124.59543546,2,-127.21
881969,0\N,7,1.36957234,6,111.96995606,1,-178.56622282,0\C,8,1.3203735
6,7,104.23938844,6,0.04884556,0\N,9,1.36251362,8,113.19065734,7,0.1692
7034,0\H,7,1.08377435,6,126.55116746,1,1.31243151,0\H,9,1.08439732,8,1
25.37862034,7,179.88697637,0\C,10,1.45553872,9,125.14242181,8,-178.342
37842,0\H,13,1.09654021,10,111.00478286,9,101.80582933,0\H,13,1.093292
5,10,110.13801227,9,-137.07459103,0\H,13,1.09380926,10,108.58943229,9,
-17.65732098,0\C,1,1.82846836,6,103.96099708,7,128.87159631,0\C,17,1.3
8825159,1,133.40582143,6,110.38683488,0\N,18,1.37288243,17,111.5705560
4,1,173.35130717,0\C,19,1.31852761,18,104.58174817,17,-0.1444353,0\N,2
0,1.36444238,19,112.77378455,18,-0.35243715,0\C,21,1.4555069,20,126.24
578035,19,-179.58234701,0\H,22,1.09527657,21,110.76792726,20,-118.0454
4836,0\H,22,1.09342115,21,108.58545803,20,1.89603593,0\H,22,1.0955762,
21,110.33573282,20,121.37498341,0\H,20,1.08385802,19,125.63268024,18,1
79.17187803,0\H,18,1.08310576,17,127.53287589,1,-5.67562565,0\\Version
=AM64L-G03RevD.01\State=1-A\HF=-906.9294248\MP2=-909.0778405\RMSD=7.25
0e-09\Thermal=0.\PG=C01 [X(C9H13N4P1)]\\@

168-Me$^+$

1\1\GINC-BORIX\SP\RMP2-FC\6-31+G(2d,p)\C10H16N4P1(1+)\CHRISTOPH\17-Feb
-2012\0\\#p MP2(FC)/6-31+g(2d,p) scf=tight\\imi52mesp_1\\1,1\P\C,1,1.8
2259366\H,2,1.09539695,1,108.2654507\H,2,1.09406109,1,110.95714545,3,1
19.55813264,0\H,2,1.09558327,1,110.09378493,3,-119.39454494,0\C,1,1.77
322609,2,106.22771107,4,-60.2566602,0\N,6,1.39736401,1,126.91819356,2,
177.8170946,0\C,7,1.35827279,6,105.84432129,1,-178.53053213,0\N,8,1.32
379384,7,113.27975638,6,0.42247564,0\C,9,1.35608828,8,105.1155322,7,-0
.3121489,0\C,1,1.82259457,6,112.06251763,10,116.41126821,0\C,1,1.77321
287,6,113.2900527,10,-123.40536513,0\C,12,1.39650457,1,128.15145699,6,
-123.34956305,0\N,13,1.3560917,12,110.85516891,1,178.27730605,0\C,14,1
.32379577,13,105.11570891,12,0.06890439,0\N,15,1.35827385,14,113.27921
248,13,-0.31471664,0\H,13,1.08346473,12,127.92841226,1,-1.5942587,0\H,
10,1.08346334,9,121.21652811,8,179.95245368,0\H,15,1.08372617,14,125.1
4116423,13,179.69541826,0\H,8,1.08372581,7,121.57975534,6,-179.5886735
1,0\C,16,1.46233398,15,125.5232733,14,178.08025095,0\H,21,1.09224479,1
6,108.31646765,15,11.11098746,0\H,21,1.0939789,16,110.55798348,15,130.
1369714,0\H,21,1.0954712,16,110.7702331,15,-107.81401093,0\C,7,1.46233
273,6,128.58569573,1,3.87250135,0\H,25,1.09224444,7,108.31496789,6,-17

1.83169968,0\H,25,1.09397495,7,110.56059888,6,-52.80185932,0\H,25,1.09
546567,7,110.7701961,6,69.24670482,0\H,11,1.0940589,1,110.95761631,12,
-60.26351953,0\H,11,1.09558456,1,110.09304546,12,60.78313752,0\H,11,1.
09539554,1,108.26546813,12,-179.82163203,0\\Version=AM64L-G03RevD.01\S
tate=1-A\HF=-946.363913\MP2=-948.6535105\RMSD=2.199e-09\Thermal=0.\PG=
C01 [X(C10H16N4P1)]\\@

169

1\1\GINC-NODE-31\SP\RMP2-FC\6-31+G(2d,p)\C5H7N2P1\ZIP07\21-Oct-2009\0\
\#p MP2(FC)/6-31+g(2d,p) scf=tight\\uz2sp_1\\0,1\P\C,1,1.85002384\C,2,
1.46235822,1,122.21152017\N,3,1.25368774,2,70.96337461,1,-98.6919107,0
\H,2,1.09370646,1,117.61318349,3,163.76973141,0\C,1,1.86671511,2,98.79
535934,3,-125.91860464,0\H,6,1.09655456,1,109.75512607,2,71.20034135,0
\C,1,1.85361863,2,99.06235751,3,132.98666494,0\N,8,1.5834069,1,121.506
74132,2,41.07172162,0\C,9,1.25611567,8,60.48448433,1,-109.28581902,0\H
,8,1.09319123,1,114.33208244,2,-175.26833793,0\H,10,1.08618702,9,139.3
6659234,8,178.18188842,0\H,3,1.08629586,2,150.06860227,1,81.27835624,0
\H,6,1.09641802,1,108.99981723,2,-170.83496724,0\H,6,1.09679126,1,112.
063272,2,-50.67722043,0\\Version=IA32L-G03RevD.01\State=1-A\HF=-642.88
9205\MP2=-644.0858712\RMSD=3.773e-09\Thermal=0.\PG=C01 [X(C5H7N2P1)]\\
@

169-Me⁺

1\1\GINC-NODE-03\SP\RMP2-FC\6-31+G(2d,p)\C6H10N2P1(1+)\ZIP07\21-Oct-20
09\0\\#p MP2(FC)/6-31+g(2d,p) scf=tight\\uz2mesp_8\\1,1\C\C,1,1.468667
86\N,2,1.25161686,1,69.9868645\H,1,1.09172968,2,120.09871568,3,99.5743
6479,0\H,1,3.27166383,2,106.39786306,3,-146.59932383,0\H,1,3.90948241,
2,140.74222626,3,-68.39648767,0\H,2,1.08554641,1,149.05654084,3,-176.2
5752475,0\P,1,1.81381577,2,123.83821871,3,-96.06888865,0\C,8,1.8138156
8,1,110.48174355,2,-102.98672413,0\N,9,1.57013434,8,115.76827654,1,-15
8.77117953,0\C,10,1.25161641,9,61.50912984,8,113.485422,0\C,8,1.814803
21,1,109.19527461,2,137.17626307,0\C,8,1.81480404,1,108.97303472,2,16.
98427112,0\H,13,1.09594155,8,110.23653259,1,-63.31866202,0\H,13,1.0957
7189,8,110.04165541,1,57.17195245,0\H,13,1.09558521,8,108.98451364,1,1
77.38790565,0\H,11,1.08554621,10,140.86291931,9,176.95124057,0\H,12,1.
09594109,8,110.23668567,1,57.44252226,0\H,12,1.09558539,8,108.9839419,
1,-61.85054848,0\\Version=IA32L-G03RevD.01\State=1-A\HF=-682.3234122\M
P2=-683.6614174\RMSD=2.337e-09\Thermal=0.\PG=C01 [X(C6H10N2P1)]\\@

170

1\1\GINC-EVGENIX\SP\RMP2-FC\6-31+G(2d,p)\C6H11N2P1\CHRISTOPH\17-Feb-20
12\0\\#p MP2(FC)/6-31+g(2d,p) scf=tight\\imi51sp_1\\0,1\P\C,1,1.866314
61\H,2,1.09734258,1,109.10335003\H,2,1.09691256,1,112.68446381,3,121.1
1133935,0\H,2,1.09580879,1,109.42409291,3,-117.94050728,0\C,1,1.864986
15,2,99.59932725,5,172.17399192,0\H,6,1.09720668,1,108.9316586,2,70.22
515573,0\H,6,1.09567361,1,109.40176258,2,-171.8842263,0\H,6,1.09699529
,1,112.96589478,2,-50.6973144,0\C,1,1.82747796,6,100.48404926,2,102.89
612667,0\C,10,1.38736637,1,133.6635333,6,-45.71023333,0\N,11,1.3702822
7,10,111.87083405,1,179.57736224,0\C,12,1.32068329,11,104.41928827,10,
0.04354439,0\N,13,1.36229218,12,112.82850835,11,-0.14677098,0\H,11,1.0
8377436,10,127.27396728,1,-0.17693478,0\H,13,1.08391069,12,125.5139270
8,11,179.92495783,0\C,14,1.45566897,13,125.6411423,12,179.25414523,0\H
,17,1.09412706,14,110.60747537,13,125.73486662,0\H,17,1.09541061,14,11
0.99547685,13,-113.64841038,0\H,17,1.09365755,14,108.40919115,13,6.074
71151,0\\Version=AM64L-G03RevD.01\State=1-A\HF=-683.2594589\MP2=-684.6
324082\RMSD=6.942e-09\Thermal=0.\PG=C01 [X(C6H11N2P1)]\\@

170-Me⁺

1\1\GINC-BORIX\SP\RMP2-FC\6-31+G(2d,p)\C7H14N2P1(1+)\CHRISTOPH\17-Feb-2012\0\\#p MP2(FC)/6-31+g(2d,p) scf=tight\\imi51mesp_1\\1,1\P\C,1,1.82270318\H,2,1.09508625,1,110.78944168\H,2,1.09528738,1,109.49434357,3,120.22789156,0\H,2,1.09647758,1,109.64754194,4,119.94164408,0\C,1,1.81757813,2,108.09944548,3,177.55151689,0\C,1,1.82261008,6,108.06717826,2,116.77752141,0\C,1,1.76982945,6,106.94598666,7,121.61087386,0\C,8,1.39652081,1,127.04740564,6,1.83356586,0\N,9,1.35549506,8,110.88726871,1,179.48234631,0\C,10,1.32232048,9,105.14682174,8,-0.01421972,0\N,11,1.35935178,10,113.29123771,9,-0.04964804,0\H,9,1.08325763,8,127.97021626,1,-0.49808476,0\H,11,1.08374535,10,125.1991474,9,179.93893105,0\C,12,1.4603693,11,125.26548357,10,179.3423168,0\H,15,1.09221189,12,108.30245847,11,2.73437307,0\H,15,1.09500471,12,110.90179111,11,121.25129022,0\H,15,1.09512523,12,110.93992607,11,-115.9667965,0\H,7,1.09530925,1,109.56987651,8,-55.18848742,0\H,7,1.09498918,1,110.81659917,8,65.17817416,0\H,7,1.09647938,1,109.55550097,8,-175.07243027,0\H,6,1.09477006,1,110.27616316,8,60.57125469,0\H,6,1.0957593,1,108.81890469,8,-179.96922899,0\H,6,1.09482047,1,110.39582467,8,-60.52903484,0\\Version=AM64L-G03RevD.01\State=1-A\HF=-722.6974135\MP2=-724.2123052\RMSD=5.143e-09\Thermal=0.\PG=C01 [X(C7H14N2P1)]\\@

171

1\1\GINC-MORITZ\SP\RMP2-FC\6-31+G(2d,p)\C4H8N1P1\CHRISTOPH\19-Oct-2009\0\\#p MP2(FC)/6-31+g(2d,p) scf=tight int=finegrid\\uz1sp_2\\0,1\P\C,1,1.85218169\C,2,1.45745636,1,125.5141692\N,3,1.25635383,2,71.26536766,1,-103.83106803,0\H,2,1.09361009,1,113.59986886,3,158.6560172,0\C,1,1.86197553,2,100.56410884,3,23.3667071,0\H,6,1.09646978,1,108.4917101,2,170.33585295,0\C,1,1.86593901,2,98.94345013,3,125.10535263,0\H,8,1.09684283,1,112.0533214,2,-51.0731384,0\H,3,1.08771835,2,150.18620838,1,75.96493736,0\H,6,1.09703508,1,110.06743124,2,-72.30640597,0\H,6,1.09685571,1,112.81293932,2,49.57047136,0\H,8,1.09692062,1,109.4667607,2,-172.52107491,0\H,8,1.09659643,1,109.45380536,2,69.70316529,0\\Version=IA32L-G03RevD.01\State=1-A\HF=-551.2386438\MP2=-552.1380955\RMSD=7.744e-09\Thermal=0.\PG=C01 [X(C4H8N1P1)]\\@

171-Me+

1\1\GINC-EDDY\SP\RMP2-FC\6-31+G(2d,p)\C5H11N1P1(1+)\CHRISTOPH\20-Oct-2009\0\\#p MP2(FC)/6-31+g(2d,p) scf=tight\\uz1mesp_3\\1,1\C\C,1,1.46803066\N,2,1.25208547,1,69.88421235\H,1,1.09213056,2,120.14247965,3,99.49822868,0\H,1,3.14629293,2,161.02344422,3,-31.17821997,0\H,1,3.90270738,2,98.72322744,3,-87.7867224,0\H,2,1.08555335,1,149.18326195,3,-176.29680196,0\P,1,1.81386467,2,123.9071219,3,-95.97149795,0\C,8,1.81671728,1,108.27712011,2,135.53991084,0\C,8,1.81429101,1,109.04326587,2,16.13046862,0\C,8,1.81802077,1,109.93045566,2,-104.95532919,0\H,11,1.09598529,8,109.9244699,1,-57.92754459,0\H,11,1.09541222,8,110.05180488,1,62.08098261,0\H,11,1.09610208,8,109.94722864,1,-177.64744903,0\H,10,1.09573752,8,109.76206324,1,56.15330067,0\H,10,1.0958272,8,110.32319365,1,-64.14831043,0\H,9,1.09568122,8,109.39935833,1,-58.78914946,0\H,9,1.0958848,8,110.24705625,1,-179.15937131,0\\Version=AM64L-G03RevD.01\State=1-A\HF=-590.6790873\MP2=-591.7201188\RMSD=8.180e-09\Thermal=0.\PG=C01 [X(C5H11N1P1)]\\@

172

1\1\GINC-GOLEM\SP\RMP2-FC\6-31+G(2d,p)\C6H11N2P1\CHRISTOPH\17-Feb-2012\0\\#p MP2(FC)/6-31+g(2d,p) scf=tight\\imi21sp_1\\0,1\P\C,1,1.86349348\H,2,1.09571922,1,111.15652839\H,2,1.09706692,1,109.1321206,3,121.18892218,0\H,2,1.09596418,1,109.67221768,4,118.21038723,0\C,1,1.86347508,2,99.46070867,3,-54.44598226,0\H,6,1.09710606,1,109.10918798,2,-66.78969385,0\H,6,1.09571784,1,111.1774683,2,54.38742388,0\H,6,1.09594456,1,1

09.67637333,2,175.01316603,0\C,1,1.84121487,6,98.76303843,2,-100.47214
189,0\N,10,1.38086817,1,123.95164675,6,-129.49260117,0\C,11,1.37606142
,10,106.99826,1,179.98277172,0\C,12,1.3768686,11,105.83294903,10,-0.00
150554,0\N,10,1.32906854,1,125.26767667,6,50.48298859,0\H,13,1.0830849
8,12,128.03828559,11,-179.98830537,0\H,12,1.08150646,11,121.65220245,1
0,179.97472823,0\C,11,1.45646359,10,128.0472214,1,-0.04919494,0\H,17,1
.0960103,11,110.33093611,10,-119.5134178,0\H,17,1.0922518,11,108.83903
755,10,0.17587336,0\H,17,1.09597456,11,110.31819917,10,119.8699731,0\\
Version=AM64L-G03RevD.01\State=1-A\HF=-683.2589548\MP2=-684.6335984\RM
SD=9.260e-09\Thermal=0.\PG=C01 [X(C6H11N2P1)]\\@

172-Me+

1\1\GINC-PHOENIX\SP\RMP2-FC\6-31+G(2d,p)\C7H14N2P1(1+)\CHRISTOPH\17-Fe
b-2012\0\\#p MP2(FC)/6-31+g(2d,p) scf=tight\\imi21mesp_1\\1,1\P\C,1,1.
81240127\H,2,1.0942075,1,109.68814171\H,2,1.09376093,1,109.39927578,3,
118.670646,0\H,2,1.09571583,1,109.16644049,4,120.74918862,0\C,1,1.8227
4184,2,109.03221351,4,-64.2526495,0\H,6,1.09645527,1,109.70147999,2,-5
9.64243939,0\H,6,1.09542437,1,109.24435067,2,60.08896751,0\H,6,1.09535
652,1,110.96396757,2,-179.78432746,0\C,1,1.82140294,2,109.34363911,6,-
118.38745147,0\H,10,1.09637599,1,109.40368645,2,57.98266383,0\H,10,1.0
9494035,1,110.99839171,2,177.78024725,0\H,10,1.09536959,1,109.55535261
,2,-61.55662214,0\C,1,1.78837251,2,105.9826109,6,120.59516926,0\N,14,1
.33060555,1,118.505064,2,-8.64224311,0\C,15,1.36203028,14,105.46134012
,1,-178.52762991,0\C,16,1.38280049,15,110.10037099,14,0.20473333,0\N,1
7,1.36994951,16,106.73692668,15,0.15790775,0\H,16,1.08173485,15,121.65
488896,14,179.97773736,0\H,17,1.08156946,16,131.86965642,15,-179.78876
674,0\C,18,1.46232036,17,125.24358755,16,-177.19165975,0\H,21,1.091911
37,18,108.72295295,17,-22.87367087,0\H,21,1.09534479,18,110.91816535,1
7,96.87583251,0\H,21,1.09417111,18,110.36505284,17,-141.04866861,0\\Ve
rsion=AM64L-G03RevD.01\State=1-A\HF=-722.696235\MP2=-724.2171073\RMSD=
6.119e-09\Thermal=0.\PG=C01 [X(C7H14N2P1)]\\@

173

1\1\GINC-STEAK\SP\RMP2-FC\6-31+G(2d,p)\C12H15N6P1\CHRISTOPH\17-Feb-201
2\0\\#p MP2(FC)/6-31+g(2d,p) scf=tight\\imi23sp_9\\0,1\P\C,1,1.8322114
7\N,2,1.37546828,1,122.77197502\C,3,1.37676687,2,106.76789306,1,-172.2
6802783,0\C,4,1.37522409,3,105.91048007,2,1.22842948,0\N,2,1.32773291,
1,125.21166788,3,-169.40131585,0\H,5,1.0826477,4,128.192655,3,179.0422
9221,0\H,4,1.08122831,3,121.4849035,2,-178.43874327,0\C,3,1.45580178,2
,127.05691677,1,12.06883445,0\H,9,1.09589069,3,109.79216119,2,-47.7014
0279,0\H,9,1.09462591,3,110.19422391,2,72.68144756,0\H,9,1.09316536,3,
108.83433252,2,-166.41559044,0\C,1,1.84430408,2,101.03812765,6,117.660
61395,0\N,13,1.33025848,1,127.64061879,2,0.80320063,0\C,14,1.37424152,
13,105.66866239,1,175.58363485,0\C,15,1.37521742,14,110.44999314,13,-0
.00593094,0\N,13,1.37760108,1,121.1085007,2,175.19554291,0\H,15,1.0827
3996,14,121.38012588,13,-179.84723072,0\H,16,1.08132928,15,132.5431226
9,14,179.17952859,0\C,17,1.45821147,13,126.9325641,1,7.42998045,0\H,20
,1.09342791,17,108.88815679,13,-159.075802,0\H,20,1.09420068,17,109.64
673585,13,-39.79743251,0\H,20,1.09562818,17,111.15569182,13,80.1684156
4,0\C,1,1.82090595,2,103.15329517,6,12.25722527,0\N,24,1.33304173,1,11
8.51469339,2,-132.65857573,0\C,25,1.36716649,24,105.71998947,1,-176.48
803571,0\C,26,1.3785782,25,110.18960471,24,0.46709967,0\N,27,1.3717760
2,26,106.38278067,25,0.26789145,0\H,26,1.08279515,25,121.62121963,24,-
179.82278578,0\H,27,1.08176311,26,132.30300018,25,-179.50601778,0\C,28
,1.46069847,27,124.68360366,26,-176.77625828,0\H,31,1.09292872,28,109.
90918378,27,91.04082644,0\H,31,1.09219772,28,109.93479573,27,-149.4751
441,0\H,31,1.09415631,28,108.40380255,27,-30.08613969,0\\Version=AM64L

-G03RevD.01\State=1-A\HF=-1130.5889983\MP2=-1133.5221272\RMSD=2.907e-0
9\Thermal=0.\PG=C01 [X(C12H15N6P1)]\\@

173-Me$^+$
1\1\GINC-TOFU\SP\RMP2-FC\6-31+G(2d,p)\C13H18N6P1(1+)\CHRISTOPH\17-Feb-
2012\0\\#p MP2(FC)/6-31+g(2d,p) scf=tight\\imi23mesp_2\\1,1\P\C,1,1.82
11838\H,2,1.09308893,1,110.27006034\H,2,1.09487094,1,109.17067985,3,12
1.25035018,0\H,2,1.09438709,1,108.39749544,4,120.18021511,0\C,1,1.7855
0227,2,105.46190624,3,55.60864274,0\N,6,1.33281972,1,118.73525493,2,-8
.51452294,0\C,7,1.36226579,6,105.15633514,1,-176.14754386,0\C,8,1.3841
3706,7,110.04685436,6,0.59609546,0\N,9,1.36745296,8,106.81767871,7,0.2
8643812,0\H,8,1.08179989,7,121.66230402,6,-179.61545781,0\H,9,1.081433
06,8,131.83023387,7,-179.46092903,0\C,10,1.46503818,9,125.53392148,8,-
177.30930384,0\H,13,1.09231892,10,109.78046332,9,95.16769882,0\H,13,1.
09369766,10,109.90364383,9,-144.73904035,0\H,13,1.09206598,10,108.3931
6221,9,-25.82864997,0\C,1,1.79951708,6,111.31982424,7,111.85670551,0\N
,17,1.33129718,1,121.2703444,6,118.33594865,0\C,18,1.36391021,17,105.4
4954787,1,-177.20185346,0\C,19,1.38237779,18,110.12060206,17,0.4322983
1,0\N,20,1.36933832,19,106.64453844,18,-0.04893093,0\H,19,1.08183776,1
8,121.64387262,17,179.98768564,0\H,20,1.08158407,19,131.89714432,18,17
9.85061015,0\C,21,1.4654675,20,125.2043785,19,-178.11513814,0\H,24,1.0
9532409,21,110.53159167,20,89.43059063,0\H,24,1.09331542,21,110.223878
53,20,-149.01117268,0\H,24,1.09229711,21,108.67919107,20,-30.24200213,
0\C,1,1.79042365,6,109.33322795,7,-127.98570897,0\N,28,1.33185975,1,11
8.51907088,6,4.16490064,0\C,29,1.36156133,28,105.39223127,1,175.792012
61,0\C,30,1.38268198,29,110.02921985,28,-0.36504681,0\N,31,1.36804087,
30,106.82849642,29,0.05055356,0\H,30,1.08173574,29,121.69750588,28,179
.80705518,0\H,31,1.08137806,30,131.86527643,29,179.93934041,0\C,32,1.4
6390564,31,125.31271572,30,179.19568881,0\H,35,1.0915114,32,108.269508
97,31,-3.72217863,0\H,35,1.09556379,32,110.54864291,31,114.87974787,0\
H,35,1.09246816,32,109.36497361,31,-123.01655498,0\\Version=AM64L-G03R
evD.01\State=1-A\HF=-1170.0220056\MP2=-1173.1072301\RMSD=3.104e-09\The
rmal=0.\PG=C01 [X(C13H18N6P1)]\\@

174
1\1\GINC-YIN\SP\RMP2-FC\6-31+G(2d,p)\C7H11N6P1\CHRISTOPH\27-Nov-2009\0
\\#p MP2(FC)/6-31+g(2d,p) scf=tight\\tro2sp_25\\0,1\P\C,1,1.86846782\C
,1,1.83541613,2,99.34354452\C,3,1.38488117,1,126.59683271,2,-127.10877
551,0\N,4,1.35376506,3,105.08985022,1,179.06012554,0\N,3,1.37556617,1,
126.13075301,2,51.69702765,0\C,1,1.8311419,3,100.26337602,6,-50.466227
25,0\C,7,1.38708194,1,132.4024516,3,28.0743513,0\N,8,1.35193779,7,104.
6257971,1,179.1388543,0\N,7,1.37318551,1,119.85447418,3,-152.97875011,
0\H,4,1.08075079,3,132.00413891,1,-1.04760831,0\H,8,1.08049271,7,131.5
1215798,1,-1.16777304,0\C,9,1.45273621,8,129.43975941,7,179.23863499,0
\H,13,1.09505882,9,110.50110029,8,-61.23552722,0\H,13,1.09174437,9,107
.05915745,8,179.40813566,0\H,13,1.09497866,9,110.44414428,8,60.0782903
7,0\C,5,1.45322812,4,129.60609303,3,-179.94673494,0\H,17,1.09492438,5,
110.4420072,4,-60.86677936,0\H,17,1.09159554,5,107.07978931,4,179.8002
3225,0\H,17,1.09491337,5,110.43878732,4,60.50703355,0\H,2,1.09520465,1
,112.21909685,7,55.25232023,0\H,2,1.09656652,1,109.13669026,7,176.4492
7662,0\H,2,1.09612053,1,108.45153355,7,-65.77487059,0\N,6,1.30439403,3
,109.69355925,1,-179.2097995,0\N,10,1.30412247,7,109.4933766,1,-179.07
976238,0\\Version=AM64L-G03RevD.01\State=1-A\HF=-938.8419521\MP2=-941.
0685966\RMSD=7.676e-09\Thermal=0.\PG=C01 [X(C7H11N6P1)]\\@

174-Me$^+$
1\1\GINC-YIN\SP\RMP2-FC\6-31+G(2d,p)\C8H14N6P1(1+)\CHRISTOPH\27-Nov-20
09\0\\#p MP2(FC)/6-31+g(2d,p) scf=tight\\tro2mesp_13\\1,1\C\N,1,1.3713

8972\N,2,2.14582213,1,71.78292666\C,3,1.34905918,2,75.87252771,1,-0.07
661323,0\C,1,2.89946364,2,91.35714696,3,-158.10654114,0\C,5,1.38803733
,1,99.3099169,2,18.27492719,0\N,6,1.34723187,5,103.572469,1,168.710438
56,0\N,5,1.37151991,1,149.81058551,2,177.3431138,0\C,5,2.9487351,1,60.
69499579,2,-109.0860233,0\H,6,1.08076674,5,132.14380045,1,-11.42968276
,0\H,4,1.08100806,3,122.78393768,2,179.84458011,0\C,3,1.4633951,2,154.
90323103,1,179.82789887,0\H,12,1.0932957,3,109.87885548,2,-120.2317111
5,0\H,12,1.09081981,3,106.93874956,2,-0.95005398,0\H,12,1.09337405,3,1
09.88686392,2,118.28764426,0\C,7,1.46259124,6,129.1579161,5,-179.18435
6,0\H,16,1.09311812,7,109.78097224,6,-56.9259849,0\H,16,1.09083412,7,1
06.97299316,6,-176.220004,0\H,16,1.09347446,7,109.97211105,6,64.449099
06,0\P,5,1.78060784,1,35.6914537,2,-143.9740367,0\C,20,1.81473671,5,10
8.20765375,1,-120.0066549,0\H,21,1.09527062,20,110.55989146,5,-172.406
77446,0\H,21,1.09463174,20,109.78667136,5,65.85241322,0\H,21,1.0956885
9,20,108.09205699,5,-52.47891128,0\H,9,1.09566908,5,144.66351636,1,-40
.71363602,0\H,9,1.09523838,5,91.62315648,1,87.16168928,0\H,9,1.0960308
,5,88.20072683,1,-163.54059092,0\N,8,1.29858582,5,108.53032514,1,-157.
70571173,0\N,2,1.29959548,1,108.78144998,4,0.23512591,0\\Version=AM64L
-G03RevD.01\State=1-A\HF=-978.2861234\MP2=-980.6538998\RMSD=7.589e-09\
Thermal=0.\PG=C01 [X(C8H14N6P1)]\\@

175

1\1\GINC-EDDY\SP\RMP2-FC\6-31+G(2d,p)\C5H10N3P1\CHRISTOPH\26-Nov-2009\
0\\#p MP2(FC)/6-31+g(2d,p) scf=tight\\tro1sp_4\\0,1\P\C,1,1.86550055\C
,1,1.83355081,2,99.41851672\C,3,1.38525595,1,127.76373591,2,-130.67672
811,0\N,4,1.35429145,3,105.05572627,1,179.85972588,0\N,3,1.37546619,1,
124.89986787,2,49.23040871,0\C,1,1.86618945,3,99.3143487,6,-51.9551271
8,0\H,4,1.08082884,3,132.19972807,1,0.59333595,0\C,5,1.45309577,4,129.
79960451,3,178.55658239,0\H,9,1.09468213,5,110.30945473,4,54.48689582,
0\H,9,1.09530385,5,110.61271768,4,-66.88959447,0\H,9,1.09170042,5,107.
07043112,4,173.77974093,0\H,2,1.09592557,1,111.90138859,3,-47.20386109
,0\H,2,1.09602727,1,109.55895503,3,73.79630129,0\H,2,1.09707215,1,108.
90831681,3,-168.32354924,0\H,7,1.09607271,1,109.4844875,3,-72.80542939
,0\H,7,1.0961021,1,111.97389805,3,48.11172049,0\H,7,1.0970448,1,108.99
699268,3,169.28731964,0\N,6,1.30307993,3,109.66656195,1,179.9871382,0\
\Version=AM64L-G03RevD.01\State=1-A\HF=-699.217277\MP2=-700.6298735\RM
SD=4.034e-09\Thermal=0.\PG=C01 [X(C5H10N3P1)]\\@

175-Me$^+$

1\1\GINC-GOLEM\SP\RMP2-FC\6-31+G(2d,p)\C6H13N3P1(1+)\CHRISTOPH\26-Nov-
2009\0\\#p MP2(FC)/6-31+g(2d,p) scf=tight\\tro1mesp_1\\1,1\C\N,1,1.371
66524\N,2,2.14671405,1,71.68312106\C,3,1.34867245,2,75.95060419,1,-0.0
0028501,0\C,1,2.944008,2,153.09861788,3,-179.9895281,0\C,1,2.92728929,
2,96.33465078,3,-149.4430093,0\H,4,1.08100028,3,122.76635492,2,179.997
31592,0\C,3,1.46353874,2,154.77933649,1,-179.980524,0\H,8,1.09082041,3
,106.88824624,2,-0.09579808,0\H,8,1.09332518,3,109.89697386,2,119.1402
1924,0\H,8,1.0933178,3,109.89436481,2,-119.3313885,0\P,1,1.78252878,2,
117.61096074,3,-179.99813863,0\C,12,1.81604959,1,108.86657936,2,-59.40
409727,0\H,13,1.0957897,12,108.73309597,1,54.6611966,0\H,13,1.09582785
,12,110.26694486,1,174.7940414,0\H,13,1.09523668,12,109.85553462,1,-64
.39477651,0\H,6,1.09523842,1,92.48093684,2,108.21044591,0\H,6,1.095789
75,1,86.95696017,2,-0.5709067,0\H,6,1.09582799,1,145.22917441,2,-121.3
1503952,0\H,5,1.09588675,1,144.14786869,2,-0.01973437,0\H,5,1.09550009
,1,91.24297163,2,-125.26474072,0\H,5,1.09550068,1,91.24627317,2,125.23
00123,0\N,2,1.29801645,1,108.72538251,4,0.,0\\Version=AM64L-G03RevD.01
\State=1-A\HF=-738.6600767\MP2=-740.215335\RMSD=9.819e-09\Thermal=0.\P
G=C01 [X(C6H13N3P1)]\\@

176

1\1\GINC-IBLIS\SP\RMP2-FC\6-31+G(2d,p)\C9H13N4P1\CHRISTOPH\17-Feb-2012
\0\\#p MP2(FC)/6-31+g(2d,p) scf=tight\\imi22sp_5\\0,1\P\C,1,1.86005952
\H,2,1.0959587,1,107.78928388\H,2,1.09286638,1,113.99132768,3,120.0516
7632,0\H,2,1.09617782,1,108.31765238,3,-116.83677646,0\C,1,1.8411048,2
,99.39403136,4,39.63416756,0\N,6,1.37648218,1,121.38783078,2,163.50953
733,0\C,7,1.37637156,6,107.05297105,1,-178.59931159,0\C,8,1.37623918,7
,105.87222919,6,0.42309931,0\N,6,1.33081569,1,127.66746374,2,-14.38604
045,0\H,9,1.08279104,8,128.13969068,7,179.53846661,0\H,8,1.08128329,7,
121.65444431,6,-179.54874707,0\C,7,1.459442,6,126.66708802,1,2.3637216
5,0\H,13,1.09477691,7,110.41436461,6,-62.32233809,0\H,13,1.09483307,7,
110.59709298,6,58.06269242,0\H,13,1.09285547,7,108.60323081,6,177.9268
8359,0\C,1,1.83507889,6,101.24024764,10,92.43495268,0\N,17,1.33306925,
1,118.80048703,6,121.6525566,0\C,18,1.36864447,17,106.04864662,1,-178.
64778044,0\C,19,1.37672822,18,110.24176293,17,0.1091206,0\N,20,1.37457
955,19,106.21075153,18,0.29419688,0\H,19,1.08285703,18,121.59128646,17
,179.95257352,0\H,20,1.08186368,19,132.41852813,18,-179.74504317,0\C,2
1,1.4593641,20,124.55926424,19,-178.77841067,0\H,24,1.09672653,21,110.
83260575,20,91.48223804,0\H,24,1.09301869,21,110.11071722,20,-147.1377
2921,0\H,24,1.09395018,21,108.56997148,20,-27.97647739,0\\Version=AM64
L-G03RevD.01\State=1-A\HF=-906.9240671\MP2=-909.0765227\RMSD=4.885e-09
\Thermal=0.\PG=C01 [X(C9H13N4P1)]\\@

176-Me$^+$

1\1\GINC-BORIX\SP\RMP2-FC\6-31+G(2d,p)\C10H16N4P1(1+)\CHRISTOPH\17-Feb
-2012\0\\#p MP2(FC)/6-31+g(2d,p) scf=tight\\imi22mesp_3\\1,1\P\C,1,1.8
1663675\H,2,1.09528021,1,108.73395488\H,2,1.0950356,1,108.87382946,3,1
19.83432338,0\H,2,1.0924182,1,110.60894822,3,-121.59483534,0\C,1,1.816
71924,2,108.90004847,5,174.93465153,0\H,6,1.09521875,1,108.7976343,2,-
64.87453866,0\H,6,1.09507935,1,108.84015506,2,54.94048004,0\H,6,1.0924
9508,1,110.53441594,2,173.41103856,0\C,1,1.7908747,2,112.72713842,6,11
6.61473975,0\N,10,1.33171261,1,119.85821068,2,-129.27243557,0\C,11,1.3
6312792,10,105.30040128,1,-177.21462462,0\C,12,1.3835571,11,110.138838
84,10,0.43265849,0\N,13,1.36902931,12,106.68241273,11,0.21292067,0\H,1
2,1.08181893,11,121.65839337,10,-179.72156324,0\H,13,1.08154353,12,131
.86175892,11,-179.66024496,0\C,14,1.46474533,13,125.69476836,12,-178.5
3191862,0\H,17,1.09403589,14,110.09098783,13,-145.34010914,0\H,17,1.09
211797,14,108.66553558,13,-26.77230255,0\H,17,1.09519199,14,110.651611
18,13,92.95344371,0\C,1,1.79076554,10,111.69889499,11,112.1360965,0\N,
21,1.33164117,1,119.90493238,10,111.31492705,0\C,22,1.36318948,21,105.
30280919,1,-176.89367441,0\C,23,1.3836044,22,110.13483766,21,0.4502588
6,0\N,24,1.3690353,23,106.68460989,22,0.20996365,0\H,23,1.08181495,22,
121.65705787,21,-179.68917425,0\H,24,1.08152307,23,131.8689151,22,-179
.58788245,0\C,25,1.46459813,24,125.755096,23,-178.56833655,0\H,28,1.09
415077,25,110.09558412,24,-143.23557482,0\H,28,1.09197011,25,108.65201
419,24,-24.63241685,0\H,28,1.09520681,25,110.67787936,24,95.02478832,0
\\Version=AM64L-G03RevD.01\State=1-A\HF=-946.362791\MP2=-948.6639001\R
MSD=4.600e-09\Thermal=0.\PG=C01 [X(C10H16N4P1)]\\@

177

1\1\GINC-GOLEM\SP\RMP2-FC\6-31+G(2d,p)\C9H12N9P1\CHRISTOPH\30-Nov-2009
\0\\#p MP2(FC)/6-31+g(2d,p) scf=tight\\tro3sp_7\\0,1\P\C,1,1.83597831\
N,2,1.3714601,1,121.63357472\N,3,2.14242231,2,72.33524631,1,-173.50241
106,0\C,4,1.3549454,3,75.41875676,2,0.47013384,0\C,1,1.83589089,2,100.
64946706,3,-49.90899677,0\C,6,1.38524624,1,129.96987429,2,35.89227084,
0\N,7,1.35478905,6,104.37845908,1,172.87607061,0\N,6,1.37140856,1,121.
68852826,2,-153.01262414,0\C,1,1.83592376,6,100.69690899,9,-49.8531449
2,0\C,10,1.38520948,1,129.92717089,6,36.06828334,0\N,11,1.35485882,10,

104.38024957,1,172.87358595,0\N,10,1.37127166,1,121.74173752,6,-152.82
903187,0\H,7,1.08004899,6,131.33058403,1,-7.74540319,0\H,11,1.08006094
,10,131.35251845,1,-7.76741388,0\H,5,1.08003876,4,124.26426673,3,-179.
96238569,0\C,4,1.45317795,3,155.520988,2,-179.86495967,0\H,17,1.094889
46,4,110.39058107,3,119.70736411,0\H,17,1.09479563,4,110.34172236,3,-1
19.09582662,0\H,17,1.09155596,4,107.13662578,3,0.27575747,0\C,12,1.453
22298,11,129.04736587,10,179.52299737,0\H,21,1.09154571,12,107.1441835
4,11,179.75754612,0\H,21,1.09477959,12,110.31879966,11,60.38554127,0\H
,21,1.09486608,12,110.39307109,11,-60.79800143,0\C,8,1.45320614,7,129.
05570573,6,179.6119233,0\H,25,1.09478125,8,110.33406644,7,60.64101488,
0\H,25,1.09154176,8,107.13032741,7,-179.98384191,0\H,25,1.09487039,8,1
10.38029906,7,-60.5532259,0\N,9,1.30815341,6,109.41360981,1,-173.97418
771,0\N,13,1.30809482,10,109.4366171,1,-173.9228396,0\N,3,1.3080052,2,
109.43602187,1,-174.05144389,0\\Version=AM64L-G03RevD.01\State=1-A\HF=
-1178.4629388\MP2=-1181.5082047\RMSD=5.266e-09\Thermal=0.\PG=C01 [X(C9
H12N9P1)]\\@

177-Me$^+$

1\1\GINC-GOLEM\SP\RMP2-FC\6-31+G(2d,p)\C10H15N9P1(1+)\CHRISTOPH\30-Nov
-2009\0\\#p MP2(FC)/6-31+g(2d,p) scf=tight\\tro3mesp_11\\1,1\C\N,1,1.3
7108609\N,2,2.14645076,1,71.53344214\C,3,1.34774149,2,76.0572205,1,0.1
7468196,0\C,1,2.90517263,2,90.61884855,3,160.41206936,0\C,5,1.38737323
,1,98.54235444,2,-15.14399309,0\N,6,1.34781902,5,103.63888125,1,-168.0
5459339,0\N,5,1.37122037,1,150.37967962,2,-172.51339942,0\C,5,2.904252
22,1,60.05852273,2,-177.89855918,0\C,9,1.38734221,5,98.59250262,1,163.
51656456,0\N,10,1.34792373,9,103.62206668,5,-167.74208575,0\N,9,1.3712
4421,5,150.19055869,1,6.75997666,0\H,6,1.0807277,5,131.95734997,1,11.7
5559395,0\H,10,1.08084374,9,131.96260951,5,12.01054281,0\H,4,1.0808113
4,3,124.47271441,2,179.99315641,0\C,3,1.46262124,2,154.87974564,1,-179
.50167443,0\H,16,1.09083406,3,107.07236815,2,-1.12949171,0\H,16,1.0932
9551,3,109.85105942,2,118.26727945,0\H,16,1.093238,3,109.80297802,2,-1
20.47011155,0\C,11,1.46264388,10,129.03903774,9,179.63940569,0\H,20,1.
09327285,11,109.84245122,10,-61.05340649,0\H,20,1.09329521,11,109.8149
6974,10,60.21710665,0\H,20,1.09087488,11,107.07070214,10,179.55701969,
0\C,7,1.46265808,6,129.06451844,5,179.50122739,0\H,24,1.09328031,7,109
.81728476,6,60.37148166,0\H,24,1.09081826,7,107.06396684,6,179.7094596
4,0\H,24,1.09327649,7,109.8396508,6,-60.90039611,0\P,1,1.78421933,2,11
9.41784297,3,-177.79079868,0\C,28,1.82017339,1,109.96927088,2,80.77724
614,0\H,29,1.09562378,28,109.17747711,1,65.13895725,0\H,29,1.0956124,2
8,109.1666214,1,-54.88918656,0\H,29,1.09569001,28,109.12373268,1,-174.
87574955,0\N,8,1.30017173,5,108.57839511,1,155.79430321,0\N,12,1.30002
444,9,108.59312272,5,155.1692372,0\N,2,1.30012004,1,108.61522727,4,-0.
39088199,0\\Version=AM64L-G03RevD.01\State=1-A\HF=-1217.9144682\MP2=-1
221.0960662\RMSD=8.171e-09\Thermal=0.\PG=C01 [X(C10H15N9P1)]\\@

178

1\1\GINC-YANG\SP\RMP2-FC\6-31+G(2d,p)\C7H12N1P1\CHRISTOPH\10-Oct-2009\
0\\#p MP2(FC)/6-31+g(2d,p) scf=tight\\y1sp_1\\0,1\P\C,1,1.86647119\C,1
,1.8266112,2,100.86074788\C,3,1.39390848,1,131.73531019,2,50.90879022,
0\C,4,1.4176974,3,108.3231753,1,179.99539867,0\C,5,1.38365745,4,106.73
631132,3,0.01274463,0\N,6,1.37059121,5,108.79268214,4,0.00765131,0\H,4
,1.0837468,3,125.2257618,1,0.02074244,0\H,5,1.08273297,4,127.33245328,
3,-179.97331755,0\H,6,1.08248285,5,130.76998033,4,179.99457454,0\C,1,1
.86646969,3,100.86343836,7,129.17909764,0\C,7,1.45610559,6,124.2191565
2,5,179.90449168,0\H,12,1.0951555,7,111.02963732,6,120.02072955,0\H,12
,1.09351084,7,108.37492212,6,0.37836021,0\H,12,1.09524609,7,111.056036
96,6,-119.27736333,0\H,11,1.09581849,1,109.39109912,3,-69.46171789,0\H
,11,1.09748089,1,109.18626339,3,172.49941698,0\H,11,1.09729404,1,112.7

0497785,3,51.43819652,0\H,2,1.09582129,1,109.39156265,3,69.46426222,0\
H,2,1.09747822,1,109.1859107,3,-172.49788217,0\H,2,1.09730177,1,112.70
420043,3,-51.43513424,0\\Version=AM64L-G03RevD.01\State=1-A\HF=-667.25
15517\MP2=-668.5979782\RMSD=3.958e-09\Thermal=0.\PG=C01 [X(C7H12N1P1)]
\\@

178-Me$^+$

1\1\GINC-CALYPSO\SP\RMP2-FC\6-31+G(2d,p)\C8H15N1P1(1+)\CHRISTOPH\10-Oc
t-2009\0\\#p MP2(FC)/6-31+g(2d,p) scf=tight\\y1mesp_7\\1,1\C\C,1,1.402
15422\C,2,1.4080771,1,107.58908532\C,3,1.38606586,2,106.91790782,1,-0.
0741947,0\N,4,1.36405748,3,109.69500449,2,-0.22998962,0\H,2,1.08229212
,1,126.13511083,5,-179.29191555,0\H,3,1.08138876,2,126.97268882,1,-179
.84813591,0\H,4,1.08243525,3,130.15967241,2,179.58049939,0\C,1,2.89176
823,5,163.40048759,4,175.22636315,0\C,9,2.93783486,1,61.96239156,5,43.
20789093,0\C,5,1.45918923,4,124.01631355,3,176.58072192,0\H,11,1.09606
806,5,111.21745267,4,-105.54893486,0\H,11,1.09513443,5,110.87821046,4,
131.83549627,0\H,11,1.09191448,5,108.52445466,4,13.66895395,0\P,1,1.76
808699,5,126.70061547,4,-178.3489267,0\C,15,1.82394615,1,113.12171316,
5,-58.26752573,0\H,16,1.09462396,15,110.84623651,1,65.50770639,0\H,16,
1.09523859,15,109.56658589,1,-54.88308072,0\H,16,1.09641637,15,109.554
65855,1,-174.75819734,0\H,9,1.09458705,1,90.36797578,5,-117.21569052,0
\H,9,1.0956711,1,144.41532008,5,7.50224372,0\H,9,1.09457071,1,91.22555
942,5,133.28072074,0\H,10,1.09497522,9,146.9901377,1,-39.04423438,0\H,
10,1.09652578,9,88.33852058,1,-161.75697038,0\H,10,1.09525705,9,90.820
85495,1,88.88328062,0\\Version=AM64L-G03RevD.01\State=1-A\HF=-706.6971
231\MP2=-708.1873981\RMSD=6.451e-09\Thermal=0.\PG=C01 [X(C8H15N1P1)]\\
@

179

1\1\GINC-YANG\SP\RMP2-FC\6-31+G(2d,p)\C5H12N1P1\CHRISTOPH\18-Oct-2009\
0\\#p MP2(FC)/6-31+g(2d,p) scf=tight\\ez1sp_4\\0,1\P\H,1,2.46186504\C,
2,1.09685596,1,45.60745196\H,1,2.49245239,3,92.84977439,2,164.49167137
,0\C,1,1.85868039,3,97.98909553,2,-171.97128261,0\N,5,1.47208296,1,120
.82969184,3,63.74222508,0\C,6,1.46343573,5,61.59974441,1,114.80727786,
0\H,7,1.09249104,6,118.07786524,5,106.42956399,0\H,7,1.08911258,6,115.
68312551,5,-112.21712837,0\C,1,1.86278036,5,100.43013781,6,-36.9988213
4,0\C,6,1.4598712,5,116.27307585,1,-138.26677326,0\H,11,1.09687218,6,1
09.12929751,5,-155.96168795,0\H,11,1.10621808,6,113.4551769,5,-35.0665
4836,0\H,11,1.0964537,6,109.13431127,5,85.80949129,0\H,10,1.09615623,1
,110.33615291,5,-80.86155709,0\H,10,1.09666921,1,111.54080875,5,41.092
73585,0\H,10,1.09679712,1,108.52486447,5,161.60984443,0\H,3,1.09737326
,2,109.11119097,1,-122.50238476,0\H,3,1.09691939,2,107.61413521,1,119.
32959094,0\\Version=AM64L-G03RevD.01\State=1-A\HF=-591.4748379\MP2=-59
2.5484098\RMSD=4.617e-09\Thermal=0.\PG=C01 [X(C5H12N1P1)]\\@

179-Me$^+$

1\1\GINC-NAUTILUS\SP\RMP2-FC\6-31+G(2d,p)\C6H15N1P1(1+)\CHRISTOPH\18-O
ct-2009\0\\#p MP2(FC)/6-31+g(2d,p) scf=tight\\ez1mesp_2\\1,1\C\P,1,1.8
1733714\H,2,2.42523433,1,133.7635177\H,2,2.42385658,1,96.21630612,3,-9
3.72408667,0\H,2,2.47705875,1,98.6010962,4,-120.0335263,0\C,2,1.817874
73,1,109.5555146,4,-21.90482869,0\C,2,1.81423014,1,109.10888683,6,120.
71444461,0\C,2,1.81227825,1,108.53051589,7,119.02526148,0\C,8,1.510990
33,2,122.87979684,1,-139.07528557,0\N,9,1.45198564,8,59.40139961,2,98.
43431842,0\H,9,1.09130998,8,115.05697386,2,-152.0133488,0\H,9,1.088383
42,8,121.41187394,2,-5.77208719,0\H,1,1.09585247,2,110.27748542,8,178.
53187519,0\H,1,1.09558703,2,109.08327344,8,58.58943536,0\H,1,1.0957105
7,2,110.13693894,8,-61.16320616,0\C,10,1.46857913,9,117.71130602,8,107
.57543678,0\H,16,1.10197595,10,113.00692152,9,-33.74926083,0\H,16,1.09

44133,10,109.05272082,9,-155.31548414,0\H,16,1.09400926,10,108.0475346
7,9,86.86127128,0\H,7,1.09558003,2,109.70633846,1,-51.2258686,0\H,7,1.
09567172,2,108.69280869,1,68.52449615,0\H,6,1.09559289,2,109.86269108,
1,-179.01828368,0\H,6,1.09586761,2,110.07865828,1,-59.07006834,0\\Vers
ion=AM64L-G03RevD.01\State=1-A\HF=-630.9235381\MP2=-632.1391432\RMSD=9
.605e-09\Thermal=0.\PG=C01 [X(C6H15N1P1)]\\@

180

1\1\GINC-GOLEM\SP\RMP2-FC\6-31+G(2d,p)\C8H18N1P1\CHRISTOPH\07-Jun-2010
\0\\#p MP2(FC)/6-31+g(2d,p) scf=tight\\pia1sp_1\\0,1\P\C,1,1.86472959\
H,2,1.09663392,1,108.40906489\C,1,4.29299458,2,89.31403823,3,71.477472
9,0\C,4,1.53677085,1,20.22890218,2,45.77637448,0\C,5,1.54284695,4,112.
23193031,1,-0.54248202,0\N,6,1.47862664,5,110.17188988,4,-55.60512744,
0\C,7,1.47175156,6,111.75449464,5,56.01662987,0\C,8,1.53031408,7,112.7
6711352,6,-58.20370778,0\H,4,1.09868457,1,130.51194146,2,43.36490128,0
\H,4,1.10110658,1,100.09126164,2,166.16147966,0\H,5,1.09794441,4,108.6
1338008,1,-121.04718549,0\H,5,1.09672202,4,109.98973481,1,121.59377059
,0\H,6,1.11495607,5,108.01602865,4,64.72811345,0\H,8,1.09803386,7,107.
47351416,6,-179.53053212,0\H,8,1.11249364,7,110.689867,6,64.24338561,0
\H,9,1.09850152,8,109.31565057,7,179.43175777,0\H,9,1.09918982,8,109.1
0697063,7,-63.30540811,0\C,1,1.86467165,2,100.97268312,6,103.35401758,
0\H,19,1.09684581,1,108.15159496,2,75.1931782,0\C,7,1.4638189,6,113.48
0305,5,179.02177753,0\H,21,1.10920631,7,112.77948213,6,-59.80970888,0\
H,21,1.09663823,7,108.7752981,6,-179.61837901,0\H,21,1.09292478,7,111.
03299185,6,61.316058,0\H,19,1.09714057,1,109.5877878,2,-168.07870514,0
\H,19,1.09710854,1,114.4527125,2,-45.55460997,0\H,2,1.09652481,1,108.9
4153727,19,-81.58922462,0\H,2,1.09717595,1,114.03088571,19,39.81090338
,0\\Version=AM64L-G03RevD.01\State=1-A\HF=-708.6235515\MP2=-710.164019
7\RMSD=6.359e-09\Thermal=0.\PG=C01 [X(C8H18N1P1)]\\@

180-Me$^+$

1\1\GINC-NAUTILUS\SP\RMP2-FC\6-31+G(2d,p)\C9H21N1P1(1+)\CHRISTOPH\08-J
un-2010\0\\#p MP2(FC)/6-31+g(2d,p) scf=tight\\pia1mesp_1\\1,1\P\C,1,1.
82646874\H,2,1.09227984,1,112.09649065\H,2,1.09620845,1,109.30250633,3
,-120.72237936,0\H,2,1.09625994,1,108.99395916,4,-118.57083156,0\C,1,1
.81803786,2,110.67831638,3,-56.13573044,0\H,6,1.09554678,1,109.8225816
6,2,-53.82501394,0\H,6,1.0932672,1,110.36389446,2,67.49918803,0\H,6,1.
0951587,1,109.25186508,2,-173.57079972,0\C,1,1.82394956,6,108.13460033
,2,114.21154634,0\H,10,1.09570489,1,110.49454077,6,177.19213557,0\H,10
,1.09616819,1,108.59465108,6,-63.8032517,0\H,10,1.09363733,1,111.10727
854,6,55.82958315,0\C,1,4.12182206,6,91.5148378,10,130.29751168,0\C,1,
1.88150935,6,109.53853191,10,120.06388077,0\C,15,1.55303856,1,107.3192
0696,6,-84.39067619,0\C,16,1.53590211,15,111.63255038,1,174.11182453,0
\C,14,1.52670225,1,99.18573067,6,-87.62844037,0\H,14,1.10752626,1,105.
67002381,6,159.0541427,0\H,14,1.09553711,1,124.25559934,6,34.74051994,
0\H,15,1.11208233,1,105.29600383,6,159.81140038,0\H,16,1.09608389,15,1
09.24648239,1,-63.96818638,0\H,16,1.10017906,15,109.41785701,1,53.9485
1084,0\H,17,1.09971241,16,109.70288512,15,65.96064753,0\H,17,1.0963067
9,16,109.13330088,15,-177.07073969,0\H,18,1.09850339,14,109.67905428,1
,76.67032198,0\H,18,1.0963162,14,108.96012084,1,-166.05634507,0\N,15,1
.46422256,1,110.18170664,6,38.15126957,0\C,28,1.46654313,15,113.175180
71,1,64.86674578,0\H,29,1.09385163,28,108.80309343,15,173.55465262,0\H
,29,1.09583177,28,111.37932512,15,-69.42590945,0\H,29,1.10597786,28,11
3.20320067,15,53.42165664,0\\Version=AM64L-G03RevD.01\State=1-A\HF=-74
8.0727491\MP2=-749.7571091\RMSD=3.000e-09\Thermal=0.\PG=C01 [X(C9H21N1
P1)]\\@

181

1\1\GINC-CIPCLU07\SP\RMP2-FC\6-31+G(2d,p)\C6H14N1P1\C2175\27-May-2010\
0\\#p MP2(FC)/6-31+G(2d,p) scf=tight\\maza1sp_2\\0,1\P\C,1,1.87530417\
C,2,1.5599282,1,124.90844627\C,3,1.54894281,2,85.30059637,1,136.449147
16,0\N,4,1.47805571,3,89.27524996,2,-18.26822284,0\H,2,1.10908124,1,10
6.99408538,5,124.24038881,0\H,3,1.09320055,2,116.63264547,1,-105.46151
417,0\H,3,1.09395956,2,113.61776607,1,23.63665165,0\H,4,1.10563219,3,1
11.54762561,2,96.0146013,0\H,4,1.09797018,3,118.51448717,2,-136.281338
98,0\C,1,1.86665485,2,99.18243036,5,171.93222718,0\H,11,1.09696308,1,1
09.69144376,2,69.40183295,0\H,11,1.09830669,1,112.92865106,2,-52.02716
604,0\H,11,1.09659965,1,109.37089938,2,-172.90104758,0\C,1,1.86116673,
11,100.76701409,2,102.98114901,0\H,15,1.09625822,1,109.4794537,11,83.8
0411248,0\H,15,1.09640408,1,108.00246755,11,-158.61006666,0\H,15,1.097
84898,1,113.55982958,11,-37.67652368,0\C,5,1.4499261,4,118.46585591,3,
142.21698128,0\H,19,1.09724525,5,109.6433493,4,68.91136944,0\H,19,1.09
635466,5,109.46385914,4,-172.6385704,0\H,19,1.10939845,5,112.89685185,
4,-51.8897338,0\\Version=AM64L-G03RevD.01\State=1-A\HF=-630.5202727\MP
2=-631.7459957\RMSD=5.996e-09\Thermal=0.\PG=C01 [X(C6H14N1P1)]\\@

181-Me$^+$

1\1\GINC-CIPCLU07\SP\RMP2-FC\6-31+G(2d,p)\C7H17N1P1(1+)\C2175\27-May-2
010\0\\#p MP2(FC)/6-31+G(2d,p) scf=tight\\maza1mesp_5\\1,1\C\C,1,1.560
77828\C,2,1.55165377,1,85.47356322\N,1,1.47153127,2,90.18641612,3,13.8
7550252,0\H,1,1.10470736,4,115.39293537,3,101.22215602,0\H,2,1.0915323
1,1,115.2102171,4,131.53608753,0\H,2,1.0938932,1,114.9265768,4,-100.07
962313,0\H,3,1.09461965,2,117.61045212,1,-131.08310541,0\H,3,1.0998796
5,2,112.26453488,1,100.73738696,0\H,1,3.19572716,4,101.89484884,3,175.
08884711,0\H,1,3.9564406,4,137.37758912,3,-129.95239603,0\C,4,1.459552
03,1,118.85381537,2,-136.9877815,0\H,12,1.1045277,4,112.74117818,1,53.
52135806,0\H,12,1.09593638,4,110.45991627,1,-68.18670541,0\H,12,1.0948
7196,4,108.67781802,1,173.90729154,0\C,1,3.00401639,4,146.34639108,12,
108.98299941,0\C,16,2.96382669,1,60.45197838,4,-44.77193626,0\P,17,1.8
1912655,16,35.46679669,1,34.88272179,0\C,18,1.81539986,17,108.51067116
,16,120.13575177,0\H,19,1.09570375,18,108.23204257,17,71.48685448,0\H,
19,1.0949394,18,110.74818706,17,-168.72396429,0\H,19,1.09542186,18,110
.05527569,17,-48.3370417,0\H,17,1.09518887,16,144.98758887,1,39.481039
,0\H,17,1.09604879,16,89.15737484,1,162.29763736,0\H,16,1.09585521,1,9
1.32898846,4,-133.78521612,0\H,16,1.09576278,1,89.45031958,4,117.32748
942,0\\Version=AM64L-G03RevD.01\State=1-A\HF=-669.9704254\MP2=-671.338
8308\RMSD=3.063e-09\Thermal=0.\PG=C01 [X(C7H17N1P1)]\\@

182

1\1\GINC-CALYPSO\SP\RMP2-FC\6-31+G(2d,p)\C7H15N2P1\CHRISTOPH\19-Oct-20
09\0\\#p MP2(FC)/6-31+g(2d,p) scf=tight\\ez2sp_36\\0,1\P\H,1,2.4976008
8\C,2,1.09562412,1,43.54856113\C,3,1.50530814,2,114.63970555,1,-153.64
975209,0\N,4,1.4646693,3,59.35391396,2,-106.21024381,0\H,4,1.09296923,
3,116.80322127,2,1.60114836,0\H,4,1.08728289,3,119.7889245,2,149.54542
281,0\H,1,2.47737603,3,93.42572669,2,145.35651286,0\C,5,1.45953182,4,1
16.31500226,3,106.97125042,0\H,9,1.10619057,5,113.50285824,4,-33.72161
299,0\H,9,1.09687273,5,109.03347761,4,87.138556,0\H,9,1.09644339,5,109
.13386552,4,-154.64098203,0\C,1,1.85334126,3,100.28632628,2,168.719750
98,0\N,13,1.47234501,1,122.43099672,3,68.74488128,0\C,14,1.46389879,13
,61.59619964,1,112.6354404,0\H,15,1.09221862,14,118.02740952,13,106.39
529139,0\H,15,1.08920118,14,115.65413703,13,-112.37265699,0\C,1,1.8638
7737,13,101.24711654,14,-32.87228346,0\C,14,1.46126295,13,115.36864749
,1,-140.19706281,0\H,19,1.09689684,14,109.11945873,13,-157.81840002,0\
H,19,1.10557989,14,113.28130863,13,-36.89814516,0\H,19,1.09588357,14,1
08.97868753,13,83.42772065,0\H,18,1.09622625,1,110.04035228,13,-81.793
55879,0\H,18,1.09621229,1,111.71612499,13,40.02776207,0\H,18,1.0967163

8,1,108.66135183,13,160.72317456,0\\Version=AM64L-G03RevD.01\State=1-A
\HF=-723.3581077\MP2=-724.9058119\RMSD=4.614e-09\Thermal=0.\PG=C01 [X(
C7H15N2P1)]\\@

182-Me^{+}

1\1\GINC-YANG\SP\RMP2-FC\6-31+G(2d,p)\C8H18N2P1(1+)\CHRISTOPH\19-Oct-2
009\0\\#p MP2(FC)/6-31+g(2d,p) scf=tight\\ez2mesp_12\\1,1\C\P,1,1.8148
3909\H,2,2.41511103,1,93.34825799\H,2,2.47082573,1,98.22335026,3,-161.
03405199,0\H,2,2.4760941,1,133.03894738,3,98.67402759,0\C,2,1.81374813
,1,108.10634879,3,-138.73611306,0\N,6,1.46942505,2,113.01517047,1,72.4
0338505,0\C,7,1.4527262,6,62.23303654,2,115.75428875,0\H,8,1.09126074,
7,118.37050445,6,104.02778554,0\H,8,1.08847402,7,116.39804677,6,-112.4
8704285,0\C,2,1.81542576,1,109.83133819,6,119.08454123,0\C,2,1.8120082
9,1,109.56362904,6,-118.98386696,0\C,12,1.51113109,2,122.95035223,1,-2
0.64589341,0\N,13,1.45190504,12,59.47657559,2,98.6704601,0\H,13,1.0913
143,12,115.08840548,2,-151.80424486,0\H,13,1.08840271,12,121.35374699,
2,-5.55504797,0\H,1,1.09540734,2,108.61638259,12,-169.5540971,0\H,1,1.
09514063,2,110.72288,12,70.70859343,0\H,1,1.09536727,2,108.98882072,12
,-49.88854087,0\C,14,1.46800523,13,117.58336258,12,107.39330763,0\H,20
,1.09413722,14,108.09796411,13,86.80526691,0\H,20,1.09457271,14,109.04
979819,13,-155.33341992,0\H,20,1.1021242,14,113.04137223,13,-33.828662
55,0\C,7,1.46826846,6,116.79769786,2,-135.53459366,0\H,24,1.10206044,7
,113.00604591,6,-37.35340536,0\H,24,1.09448116,7,109.05298699,6,84.113
06871,0\H,24,1.09413834,7,108.11209335,6,-158.01976976,0\H,11,1.095214
53,2,110.53102701,1,171.81200099,0\H,11,1.09571816,2,108.7829057,1,-68
.03835725,0\\Version=AM64L-G03RevD.01\State=1-A\HF=-762.8116397\MP2=-7
64.4989154\RMSD=5.788e-09\Thermal=0.\PG=C01 [X(C8H18N2P1)]\\@

183

1\1\GINC-AZAZEL\SP\RMP2-FC\6-31+G(2d,p)\C11H15N2P1\CHRISTOPH\09-Oct-20
09\0\\#p MP2(FC)/6-31+g(2d,p) scf=tight\\y2sp_14\\0,1\P\C,1,1.82241042
\C,2,1.39489052,1,122.75303463\C,3,1.41790491,2,108.460035,1,-179.5211
5978,0\C,4,1.38283066,3,106.52419986,2,0.16048795,0\N,5,1.37091101,4,1
09.15736178,3,0.16987592,0\H,3,1.08330162,2,124.39126247,1,0.0521077,0
\H,4,1.08246513,3,127.47156692,2,179.6260496,0\H,5,1.08308416,4,130.63
745696,3,179.96538578,0\C,1,1.83098271,2,105.05291856,6,-48.25416695,0
\C,10,1.39477271,1,131.63081104,2,111.75960001,0\C,11,1.42143897,10,10
8.08075564,1,173.44558143,0\C,12,1.38127769,11,106.86821908,10,-0.1003
3408,0\N,13,1.37306109,12,108.70395439,11,-0.48468094,0\H,11,1.0828452
8,10,125.50920395,1,-5.62264686,0\H,12,1.08262669,11,127.11983879,10,-
179.81125788,0\H,13,1.08243368,12,130.93540331,11,179.15394208,0\C,1,1
.8627987,2,104.11079105,6,56.2129039,0\C,6,1.45483833,5,123.70949664,4
,-176.97637811,0\H,19,1.09792992,6,111.30916042,5,93.94597832,0\H,19,1
.09425972,6,108.7762977,5,-25.68164179,0\H,19,1.0930479,6,110.02354783
,5,-144.8411048,0\C,14,1.4547664,13,124.81101556,12,179.14382822,0\H,2
3,1.09622685,14,111.26163373,13,-110.93289815,0\H,23,1.09346565,14,108
.64946378,13,9.3607534,0\H,23,1.09586984,14,110.23033955,13,128.583593
14,0\H,18,1.09633726,1,108.82441332,2,-179.67582252,0\H,18,1.09571377,
1,114.417824,2,-57.89453949,0\H,18,1.09563176,1,107.76418611,2,63.2016
3178,0\\Version=AM64L-G03RevD.01\State=1-A\HF=-874.9130259\MP2=-877.00
92161\RMSD=6.262e-09\Thermal=0.\PG=C01 [X(C11H15N2P1)]\\@

183-Me^{+}

1\1\GINC-EDDY\SP\RMP2-FC\6-31+G(2d,p)\C12H18N2P1(1+)\CHRISTOPH\09-Oct-
2009\0\\#p MP2(FC)/6-31+g(2d,p) scf=tight\\y2mesp_3\\1,1\C\C,1,1.40198
604\C,2,1.40884557,1,107.58005154\C,3,1.38689696,2,106.90665646,1,-0.0
1253937,0\N,4,1.36351609,3,109.63846006,2,-0.32552306,0\H,2,1.08238122
,1,126.05183309,5,-179.16700812,0\H,3,1.08150747,2,127.01101031,1,-179

.79237961,0\H,4,1.08249636,3,130.20484463,2,179.48471674,0\C,1,2.88009
116,5,162.97361439,4,-172.76554419,0\C,1,2.97679306,5,102.92583095,4,-
152.68962871,0\C,10,1.40201805,1,138.9668618,5,-53.77829238,0\C,11,1.4
0882695,10,107.58103145,1,136.39391397,0\C,12,1.38691578,11,106.905616
46,10,-0.01586623,0\N,13,1.36349762,12,109.63881667,11,-0.31926001,0\H
,11,1.08238368,10,126.05196338,1,-43.13351922,0\H,12,1.08150536,11,127
.0126781,10,-179.80830647,0\H,13,1.0824928,12,130.20266479,11,179.4818
8381,0\C,5,1.46137308,4,124.1066473,3,176.2328202,0\H,18,1.09393482,5,
110.54796937,4,138.86022094,0\H,18,1.09652674,5,111.00571528,4,-99.138
20992,0\H,18,1.09229704,5,108.49851762,4,20.05285066,0\C,14,1.46135588
,13,124.11928331,12,176.33987184,0\H,22,1.09226983,14,108.48868612,13,
19.29436789,0\H,22,1.09396701,14,110.56131844,13,138.1261684,0\H,22,1.
09650034,14,111.00389725,13,-99.86907591,0\P,1,1.77383979,5,125.674559
24,4,-179.18053199,0\C,26,1.82446463,1,111.9206633,5,-67.74053416,0\H,
27,1.09534809,26,110.30495384,1,-174.55344328,0\H,27,1.09528808,26,108
.09049069,1,-55.12801177,0\H,27,1.09384857,26,110.91697965,1,64.218663
42,0\H,9,1.09528708,1,144.32547281,5,-9.03747511,0\H,9,1.09384328,1,91
.1288656,5,116.67065691,0\H,9,1.09534623,1,90.29668006,5,-134.03346712
,0\\Version=AM64L-G03RevD.01\State=1-A\HF=-914.3625504\MP2=-916.603595
3\RMSD=6.134e-09\Thermal=0.\PG=C01 [X(C12H18N2P1)]\\@

184

1\1\GINC-NAUTILUS\SP\RMP2-FC\6-31+G(2d,p)\C8H18N1P1\CHRISTOPH\18-Jun-2
010\0\\#p MP2(FC)/6-31+g(2d,p) scf=tight\\pic1sp_1\\0,1\P\C,1,1.867595
11\H,2,1.09905134,1,112.80627886\H,2,1.09701357,1,108.97898515,3,120.5
8141256,0\H,2,1.09609126,1,110.23786307,4,117.62185262,0\C,1,1.8675918
5,2,98.43203915,5,-175.99280187,0\H,6,1.09609401,1,110.23758086,2,175.
98545457,0\H,6,1.09701387,1,108.97949083,2,-66.3919385,0\H,6,1.0990499
2,1,112.80556431,2,54.18982325,0\C,1,4.22988304,6,93.7073887,2,-120.85
292872,0\C,10,1.53601778,1,20.02213944,6,-59.79603301,0\C,11,1.5437827
5,10,111.22789005,1,4.77122764,0\C,12,1.54379121,11,108.76885115,10,-5
3.0639918,0\C,13,1.53602089,12,111.22781272,11,53.0622073,0\N,14,1.462
03175,13,111.28392164,12,-56.90440844,0\H,10,1.11303055,1,101.9633391,
6,54.23556555,0\H,10,1.0985077,1,129.48519172,6,-69.79626288,0\H,11,1.
09916842,10,108.79284414,1,-115.91356957,0\H,11,1.09808801,10,108.8096
5021,1,127.23569104,0\H,12,1.10430637,11,108.45696601,10,64.70778076,0
\H,13,1.09808121,12,110.91008681,11,174.30479932,0\H,13,1.09917139,12,
109.49336215,11,-67.20959747,0\H,14,1.11302966,13,109.33859753,12,66.4
7871064,0\H,14,1.09850887,13,109.70736771,12,-176.58462033,0\C,15,1.45
549127,14,111.49921924,13,-175.44340191,0\H,25,1.10990366,15,113.24297
493,14,-62.45171555,0\H,25,1.09701464,15,109.67422498,14,58.25629181,0
\H,25,1.09701497,15,109.67431171,14,176.84027209,0\\Version=AM64L-G03R
evD.01\State=1-A\HF=-708.6332954\MP2=-710.169652\RMSD=4.802e-09\Therma
l=0.\PG=C01 [X(C8H18N1P1)]\\@

184-Me$^+$

1\1\GINC-NAUTILUS\SP\RMP2-FC\6-31+G(2d,p)\C9H21N1P1(1+)\CHRISTOPH\18-J
un-2010\0\\#p MP2(FC)/6-31+g(2d,p) scf=tight\\pic1mesp_1\\1,1\H\H,1,4.
18125793\C,2,4.54710697,1,79.61308935\C,3,1.53914874,2,18.40633801,1,7
0.61296888,0\C,4,1.54966231,3,109.66996248,2,-106.12329446,0\C,5,1.549
66231,4,110.42929043,3,-53.12241859,0\C,6,1.53914853,5,109.66991841,4,
53.1225466,0\N,7,1.45769819,6,110.64708924,5,-57.32248694,0\H,3,1.1113
1189,2,94.76611957,1,-72.43639759,0\H,3,1.09620059,2,103.79261301,1,17
8.6572125,0\H,4,1.09787942,3,108.29780043,2,133.74120217,0\H,4,1.09741
631,3,109.16074902,2,15.98070853,0\H,5,1.10326988,4,108.50108179,3,65.
67497873,0\H,6,1.09741668,5,111.23800498,4,173.97832289,0\H,6,1.097878
34,5,110.06999269,4,-65.92707539,0\H,7,1.11131145,6,109.28715185,5,66.
72769793,0\H,7,1.09620235,6,108.73528815,5,-176.6980852,0\C,8,1.462520

61,7,111.92634817,6,-171.62108036,0\H,18,1.10625681,8,113.05863403,7,-
62.73608236,0\H,18,1.09544682,8,109.40660488,7,176.37508664,0\H,18,1.0
9544722,8,109.4065908,7,58.15262016,0\C,5,2.99802686,4,147.11659696,3,
177.09417174,0\C,22,2.947153,5,60.55969925,4,-30.22392455,0\P,23,1.820
38817,22,35.95441631,5,34.85682143,0\C,24,1.82004453,23,108.50483876,2
2,117.47911147,0\H,25,1.0958614,24,109.47551509,23,-58.60315121,0\H,25
,1.09513765,24,110.31913048,23,-178.37576543,0\H,25,1.09513723,24,110.
31936052,23,61.16956379,0\H,22,1.09582898,5,144.9385331,4,0.94065692,0
\H,22,1.09497721,5,92.14973404,4,128.87363815,0\H,23,1.09591565,22,91.
38121819,5,-88.44946675,0\H,23,1.09582946,22,88.52193841,5,162.7040498
9,0\\Version=AM64L-G03RevD.01\State=1-A\HF=-748.0839412\MP2=-749.76399
84\RMSD=2.156e-09\Thermal=0.\PG=C01 [X(C9H21N1P1)]\\@

185

1\1\GINC-YIN\SP\RMP2-FC\6-31+G(2d,p)\C15H18N3P1\CHRISTOPH\09-Oct-2009\
0\\#p MP2(FC)/6-31+g(2d,p) scf=tight\\y3sp_7\\0,1\P\C,1,1.82310776\C,2
,1.39231125,1,130.86919866\C,3,1.42059801,2,107.8737916,1,-175.3845011
3,0\C,4,1.38266058,3,106.9669672,2,-0.1027021,0\N,5,1.37211427,4,108.7
5740998,3,0.8087191,0\H,3,1.08251981,2,124.77790505,1,4.41540546,0\H,4
,1.08264109,3,127.14641238,2,179.55115315,0\H,5,1.08265477,4,130.91742
357,3,-178.81591726,0\C,1,1.82291053,2,101.63978938,6,-166.98183116,0\
C,10,1.39244816,1,130.70503485,2,-96.68990978,0\C,11,1.42036854,10,107
.85745303,1,-174.78735923,0\C,12,1.38296184,11,106.97785441,10,-0.0808
1263,0\N,13,1.37197873,12,108.74251435,11,0.76872253,0\H,11,1.08233601
,10,124.64803775,1,5.06171844,0\H,12,1.08266407,11,127.14142973,10,179
.64778397,0\H,13,1.08268302,12,130.91593691,11,-178.82296993,0\C,1,1.8
2383513,10,101.45518536,14,-165.7404734,0\C,18,1.39225727,1,130.772582
75,10,-96.57789145,0\C,19,1.42077082,18,107.84876001,1,-175.55424433,0
\C,20,1.38267029,19,106.97397444,18,-0.07808684,0\N,21,1.37220734,20,1
08.75036267,19,0.82566076,0\H,19,1.08251587,18,124.69547039,1,4.385921
79,0\H,20,1.08265475,19,127.14118236,18,179.6290798,0\H,21,1.08265972,
20,130.92850896,19,-178.98585312,0\C,6,1.45367666,5,124.75715873,4,-17
4.97659335,0\H,26,1.09720385,6,111.18645604,5,101.40672124,0\H,26,1.09
364771,6,108.83121461,5,-18.92553496,0\H,26,1.09515404,6,110.07584253,
5,-138.11052728,0\C,14,1.45346106,13,124.88420327,12,-175.15627789,0\H
,30,1.0953734,14,110.06111011,13,-137.21400949,0\H,30,1.09710173,14,11
1.13337204,13,102.34400222,0\H,30,1.09366704,14,108.84397156,13,-17.94
967727,0\C,22,1.45358263,21,124.83486152,20,-174.94509904,0\H,34,1.095
33728,22,110.04722684,21,-138.09389002,0\H,34,1.09708418,22,111.147660
12,21,101.4318787,0\H,34,1.09371862,22,108.85370182,21,-18.92889987,0\
\Version=AM64L-G03RevD.01\State=1-A\HF=-1082.5767022\MP2=-1085.422408\
RMSD=1.695e-09\Thermal=0.\PG=C01 [X(C15H18N3P1)]\\@

185-Me⁺

1\1\GINC-YIN\SP\RMP2-FC\6-31+G(2d,p)\C16H21N3P1(1+)\CHRISTOPH\09-Oct-2
009\0\\#p MP2(FC)/6-31+g(2d,p) scf=tight\\y3mesp_50\\1,1\C\C,1,1.39899
177\C,2,1.41087901,1,107.56904801\C,3,1.38508424,2,106.89422169,1,0.20
29719,0\N,4,1.3666045,3,109.66935914,2,-0.46790146,0\H,2,1.08248376,1,
125.40191355,5,179.69968975,0\H,3,1.08153602,2,127.04384406,1,-179.647
82141,0\H,4,1.08257956,3,130.26380284,2,179.0253438,0\C,1,2.90524192,5
,162.21099281,4,173.18776843,0\C,9,1.40137282,1,127.87495896,5,-93.374
6069,0\C,10,1.40889888,9,107.57767431,1,-134.51023345,0\C,11,1.3868963
7,10,106.86968232,9,0.1138173,0\N,12,1.36371285,11,109.69059125,10,0.0
8594114,0\H,10,1.08235535,9,126.04679075,1,44.71002903,0\H,11,1.081545
32,10,127.03692127,9,179.78761985,0\H,12,1.08242899,11,130.17521437,10
,-179.723477,0\C,1,2.88963376,5,105.0991509,4,-148.68930425,0\C,17,1.4
0037807,1,90.87805081,5,52.25396667,0\C,18,1.41034078,17,107.6250181,1
,179.64552286,0\C,19,1.38651651,18,106.89339598,17,-0.46987022,0\N,20,

1.36521744,19,109.55554576,18,-0.14662932,0\H,18,1.08282407,17,125.288
08883,1,1.0921409,0\H,19,1.08164559,18,127.10249832,17,179.85929636,0\
H,20,1.08257811,19,130.27704358,18,179.54089939,0\C,5,1.46105371,4,123
.65834187,3,173.10740977,0\H,25,1.09259744,5,108.52499364,4,24.7531637
4,0\H,25,1.0935755,5,110.47940579,4,143.28566038,0\H,25,1.09667774,5,1
11.01833033,4,-94.52573297,0\C,13,1.46268858,12,124.1588928,11,-177.59
818166,0\H,29,1.09398193,13,110.55336348,12,-129.69012021,0\H,29,1.094
72515,13,111.03826806,12,108.79726896,0\H,29,1.0922178,13,108.11572447
,12,-10.84682072,0\C,21,1.46055759,20,124.14773685,19,178.2592952,0\H,
33,1.09234388,21,108.53900979,20,18.37495216,0\H,33,1.0962038,21,111.1
1640903,20,-100.94391076,0\H,33,1.09485532,21,110.40781874,20,136.9417
5259,0\P,9,1.77564136,1,35.19640205,5,8.85214676,0\C,37,1.82968261,9,1
05.70945629,1,-118.10127356,0\H,38,1.09494258,37,109.22945351,9,65.270
92449,0\H,38,1.09376678,37,111.04930671,9,-55.54472512,0\H,38,1.094339
63,37,108.94551225,9,-175.69735796,0\\Version=AM64L-G03RevD.01\State=1
-A\HF=-1122.0235496\MP2=-1125.0183203\RMSD=4.003e-09\Thermal=0.\PG=C01
[X(C16H21N3P1)]\\@

186

1\1\GINC-NAUTILUS\SP\RMP2-FC\6-31+G(2d,p)\C9H18N3P1\CHRISTOPH\20-Oct-2
009\0\\#p MP2(FC)/6-31+g(2d,p) scf=tight\\ez3sp_7\\0,1\P\H,1,2.5299877
1\H,1,2.49392064,2,96.03954604\C,1,1.86335575,3,23.95329388,2,-72.2848
203,0\C,4,1.50532904,1,126.18357701,3,149.74883073,0\N,5,1.46448232,4,
59.37143598,1,104.31203268,0\H,5,1.09301411,4,116.71250287,1,-147.8350
4129,0\H,5,1.08753402,4,119.95526719,1,0.08778347,0\H,1,2.48104922,4,9
3.60263305,6,6.80174753,0\C,6,1.45984836,5,116.22404173,4,106.93720607
,0\H,10,1.10605349,6,113.46656097,5,-33.93339183,0\H,10,1.09687477,6,1
09.00786767,5,86.93329095,0\H,10,1.09639416,6,109.14460431,5,-154.8347
6664,0\C,1,1.85307109,4,100.50924041,6,30.06449006,0\N,14,1.47409253,1
,122.38855296,4,69.34099301,0\C,15,1.46477573,14,61.52867897,1,111.854
66386,0\H,16,1.09209262,15,118.01547575,14,106.61321034,0\H,16,1.08921
671,15,115.61772961,14,-111.91455298,0\C,1,1.84784229,14,100.31910634,
15,-31.46623929,0\N,19,1.47539081,1,114.47044433,14,178.92399901,0\C,2
0,1.45847793,19,61.78685388,1,113.61893954,0\H,21,1.09251575,20,118.00
930665,19,106.114728,0\H,21,1.08922326,20,115.68873484,19,-111.6203049
3,0\C,20,1.45975738,19,115.54699391,1,-138.85981043,0\H,24,1.09680958,
20,108.97274753,19,-157.39502415,0\H,24,1.10593096,20,113.58860438,19,
-36.37852775,0\H,24,1.09644111,20,108.98146036,19,84.45350656,0\C,15,1
.46140005,14,115.2516708,1,-141.0304355,0\H,28,1.09686916,15,109.13594
298,14,-158.04703542,0\H,28,1.10552892,15,113.26697142,14,-37.12080519
,0\H,28,1.09592469,15,108.96888409,14,83.19613232,0\\Version=AM64L-G03
RevD.01\State=1-A\HF=-855.2441703\MP2=-857.2637738\RMSD=5.976e-09\Ther
mal=0.\PG=C01 [X(C9H18N3P1)]\\@

186-Me⁺

1\1\GINC-MORITZ\SP\RMP2-FC\6-31+G(2d,p)\C10H21N3P1(1+)\CHRISTOPH\21-Oc
t-2009\0\\#p MP2(FC)/6-31+g(2d,p) scf=tight\\ez3mesp_43\\1,1\C\P,1,1.8
1605085\H,2,2.47213266,1,98.49078885\H,2,2.47159668,1,97.28059008,3,-1
20.20178283,0\H,2,2.46723073,1,133.26015527,4,-104.06728799,0\C,2,1.81
284268,1,107.6994532,4,21.94213042,0\N,6,1.47068028,2,112.72487471,1,7
4.04203638,0\C,7,1.45393977,6,62.04713495,2,115.55515457,0\H,8,1.09129
299,7,118.31125227,6,104.22374642,0\H,8,1.08787346,7,116.45763082,6,-1
12.00385828,0\C,2,1.81124984,1,109.85402373,6,119.78771048,0\C,11,1.51
079459,2,120.68289186,1,85.48675987,0\N,12,1.45342654,11,59.49152452,2
,101.85530846,0\H,12,1.09112158,11,115.32372587,2,-148.63146487,0\H,12
,1.0888616,11,121.06810654,2,-2.07823266,0\C,2,1.81093583,1,110.113809
13,6,-119.25640765,0\C,16,1.51074497,2,122.94070852,1,-14.40169938,0\N
,17,1.45215153,16,59.53069561,2,97.57479923,0\H,17,1.09136863,16,115.0

3878851,2,-152.98804212,0\H,17,1.08845207,16,121.46801321,2,-6.5924504
5,0\H,1,1.09533151,2,108.75482661,16,-170.2731052,0\H,1,1.09486028,2,1
10.43061226,16,70.03419359,0\H,1,1.09561688,2,109.14936351,16,-50.7375
6188,0\C,13,1.46778135,12,116.96111012,11,107.32801732,0\H,24,1.094265
37,13,108.06056272,12,85.64072137,0\H,24,1.09488808,13,109.24477956,12
,-156.66890402,0\H,24,1.10212731,13,113.18123612,12,-35.06012966,0\C,1
8,1.46796557,17,117.49563819,16,107.18688802,0\H,28,1.09425934,18,108.
14905201,17,86.93782367,0\H,28,1.09461392,18,109.02222004,17,-155.1576
1433,0\H,28,1.10199608,18,113.0223651,17,-33.77088613,0\C,7,1.46786147
,6,116.69899886,2,-135.87936181,0\H,32,1.10222668,7,113.03911271,6,-37
.1236966,0\H,32,1.09462852,7,109.07725768,6,84.29496093,0\H,32,1.09426
25,7,108.18501706,6,-157.80659182,0\\Version=IA32L-G03RevD.01\State=1-
A\HF=-894.699093\MP2=-896.8593674\RMSD=6.221e-09\Thermal=0.\PG=C01 [X(
C10H21N3P1)]\\@

187

1\1\GINC-GOLEM\SP\RMP2-FC\6-31+G(2d,p)\C6H14N1P1\CHRISTOPH\31-May-2010
\0\\#p MP2(FC)/6-31+g(2d,p) scf=tight\\mazb1sp_1\\0,1\H\H,1,2.62594725
\P,2,2.48315962,1,58.64108051\C,3,1.86624823,2,83.49013969,1,13.721510
34,0\C,3,1.86220599,2,24.31921838,1,143.58399479,0\C,5,1.55715166,3,11
8.29596745,2,-130.01061049,0\N,6,1.47810029,5,88.98851685,3,-138.32745
578,0\C,7,1.47809965,6,90.54571068,5,19.83992912,0\H,6,1.09803032,5,11
8.04663088,3,103.74514503,0\H,6,1.10656365,5,111.77775468,3,-23.848987
01,0\H,8,1.10656374,7,113.03319895,6,93.47026028,0\H,8,1.0980304,7,114
.93505516,6,-140.52994486,0\C,7,1.44937148,6,118.21552343,5,142.6378,0
\H,13,1.1092269,7,113.21615924,6,-53.73350212,0\H,13,1.09734505,7,109.
57567391,6,67.18668168,0\H,13,1.09734603,7,109.5756944,6,-174.6537351,
0\H,4,1.09690678,3,109.50180445,2,-152.72816064,0\H,4,1.09683797,3,109
.60779526,2,89.5242643,0\C,3,1.86624755,2,83.49040426,1,-86.55145055,0
\H,19,1.0968379,3,109.6082685,2,-89.53018834,0\H,19,1.09867101,3,112.7
3863647,2,31.65797322,0\H,19,1.09690713,3,109.50116574,2,152.72237198,
0\\Version=AM64L-G03RevD.01\State=1-A\HF=-630.5208975\MP2=-631.7439301
\RMSD=3.567e-09\Thermal=0.\PG=C01 [X(C6H14N1P1)]\\@

187-Me$^+$

1\1\GINC-GOLEM\SP\RMP2-FC\6-31+G(2d,p)\C7H17N1P1(1+)\CHRISTOPH\31-May-
2010\0\\#p MP2(FC)/6-31+g(2d,p) scf=tight\\mazb1mesp_1\\1,1\H\H,1,2.95
538211\H,2,4.18086484,1,69.64715831\C,3,1.09415187,2,68.47535551,1,-81
.4383652,0\C,4,2.96144321,3,90.88433441,2,-7.24186206,0\P,4,1.81640177
,3,110.09707274,2,23.2682973,0\C,6,1.81908622,4,109.09247636,3,-179.74
430061,0\H,7,1.09572505,6,110.11855266,4,178.20545245,0\H,7,1.09590142
,6,110.07193332,4,58.12100398,0\H,7,1.09562843,6,109.95741113,4,-61.76
820378,0\H,5,1.09562924,4,91.1829192,3,0.72188823,0\H,5,1.09590073,4,8
9.5404206,3,-108.10588146,0\H,4,1.0961393,3,109.52659775,2,-97.0127419
4,0\H,4,1.0941531,3,108.2186618,2,143.64055042,0\C,6,1.82294749,4,111.
20145223,3,59.63371889,0\C,15,1.56218247,6,114.47738002,4,-47.73214881
,0\N,16,1.47443245,15,88.4311082,6,94.64646706,0\C,17,1.47442932,16,91
.04059176,15,20.99749028,0\H,16,1.10350082,15,109.79810816,6,-150.0796
686,0\H,16,1.09871401,15,119.43848271,6,-23.7520918,0\H,18,1.10350093,
17,114.2574865,16,90.06046512,0\H,18,1.09871611,17,115.30880932,16,-14
3.0650975,0\C,17,1.45656398,16,118.58014738,15,144.69005667,0\H,23,1.0
9535506,17,109.31314527,16,-175.3160943,0\H,23,1.10498209,17,112.66476
712,16,-54.33485586,0\H,23,1.09535477,17,109.31405113,16,66.64737177,0
\\Version=AM64L-G03RevD.01\State=1-A\HF=-669.9699751\MP2=-671.3393318\
RMSD=9.402e-09\Thermal=0.\PG=C01 [X(C7H17N1P1)]\\@

188

1\1\GINC-CALYPSO\SP\RMP2-FC\6-31+G(2d,p)\C8H18N1P1\CHRISTOPH\11-Jun-20

10\0\\#p MP2(FC)/6-31+g(2d,p) scf=tight\\pib1sp_2\\0,1\P\C,1,1.8679275
\H,2,1.09725779,1,113.86144101\H,2,1.09666988,1,109.93715434,3,122.295
24121,0\H,2,1.09704681,1,108.38971295,4,117.01778805,0\C,1,1.86658148,
2,99.44251974,4,169.37040947,0\H,6,1.09699562,1,108.66268418,2,72.1654
5009,0\H,6,1.09691992,1,109.53038496,2,-170.72356194,0\H,6,1.09770191,
1,114.03840144,2,-48.6681477,0\N,1,4.22525791,6,90.84631125,2,92.69564
408,0\C,10,1.46716769,1,23.03821196,6,-61.44592838,0\C,11,1.53944831,1
0,111.025633,1,-4.48665927,0\C,12,1.54470249,11,109.68562764,10,-57.34
653018,0\C,13,1.53854775,12,110.31616281,11,53.28517906,0\C,10,1.46344
393,1,100.61478518,6,178.17500445,0\H,11,1.11139491,10,110.8939779,1,-
126.72586885,0\H,11,1.09759508,10,107.53770333,1,116.50768945,0\H,12,1
.10212846,11,107.07897831,10,59.0669247,0\H,13,1.09782639,12,110.35884
341,11,175.89152183,0\H,13,1.10195508,12,109.50882624,11,-66.80791226,
0\H,14,1.09876401,13,109.46378742,12,67.07360378,0\H,14,1.098616,13,11
0.53084213,12,-174.73335891,0\H,15,1.11306885,10,111.23629513,1,83.397
90571,0\H,15,1.09826805,10,108.17438096,1,-159.46007156,0\C,10,1.45610
723,1,133.82474178,6,-47.75305725,0\H,25,1.09702742,10,109.61958008,1,
171.11073335,0\H,25,1.10985153,10,113.32553336,1,-68.20735738,0\H,25,1
.09712738,10,109.69860472,1,52.58754621,0\\Version=AM64L-G03RevD.01\St
ate=1-A\HF=-708.6306626\MP2=-710.1674066\RMSD=4.945e-09\Thermal=0.\PG=
C01 [X(C8H18N1P1)]\\@

188-Me$^+$

1\1\GINC-NAUTILUS\SP\RMP2-FC\6-31+G(2d,p)\C9H21N1P1(1+)\CHRISTOPH\13-J
un-2010\0\\#p MP2(FC)/6-31+g(2d,p) scf=tight\\pib1mesp_1\\1,1\P\C,1,1.
82146573\H,2,1.09565702,1,110.47125303\H,2,1.09593895,1,109.44412011,3
,-119.75125397,0\H,2,1.09482157,1,110.24992798,4,-119.451879,0\C,1,1.8
1928154,2,107.57489017,5,-55.29734089,0\H,6,1.09439039,1,108.60673485,
2,72.99271057,0\N,1,3.29738311,6,77.09602507,2,-93.48410715,0\C,8,1.45
991935,1,58.47280645,6,177.983582,0\C,9,1.54609392,8,110.38727688,1,35
.24643802,0\C,10,1.55280023,9,109.82089087,8,57.54665144,0\C,11,1.5404
6983,10,112.70631681,9,-48.02004525,0\C,8,1.47325182,1,109.07261566,6,
-79.78620784,0\H,9,1.10964608,8,112.36479538,1,154.76055015,0\H,9,1.09
683001,8,109.17479741,1,-87.52371182,0\H,10,1.10205282,9,108.49377485,
8,176.41735655,0\H,11,1.09885085,10,105.78131401,9,71.18600583,0\H,11,
1.09788147,10,111.78622515,9,-173.96397898,0\H,12,1.09656153,11,109.04
867753,10,167.19381788,0\H,12,1.09666628,11,110.80590563,10,-75.976143
96,0\H,13,1.09582081,8,108.31835331,1,120.8300663,0\H,13,1.10828014,8,
110.49866338,1,-122.0776763,0\C,1,1.82189273,6,108.34312736,2,116.0848
3888,0\H,23,1.09530073,1,110.33594352,6,64.41108204,0\C,8,1.46713229,1
,139.40110755,6,91.56477156,0\H,25,1.09486336,8,109.28482051,1,-111.75
760971,0\H,25,1.1047445,8,112.77469027,1,127.68463751,0\H,25,1.0961886
2,8,109.76457806,1,6.74836767,0\H,23,1.09568974,1,110.25278893,6,-175.
29538436,0\H,23,1.09578782,1,109.81526494,6,-55.33018103,0\H,6,1.09651
15,1,108.98382807,2,-47.37484728,0\H,6,1.09441326,1,110.51517155,2,-16
6.95923483,0\\Version=AM64L-G03RevD.01\State=1-A\HF=-748.0823204\MP2=-
749.765647\RMSD=3.218e-09\Thermal=0.\PG=C01 [X(C9H21N1P1)]\\@

189

1\1\GINC-GOLEM\SP\RMP2-FC\6-31+G(2d,p)\C7H16N1P1\CHRISTOPH\22-Oct-2009
\0\\#p MP2(FC)/6-31+g(2d,p) scf=tight\\b1sp_10\\0,1\P\C,1,1.86663959\H
,2,1.09886159,1,112.75176229\C,1,1.86626653,2,99.05330978,3,-52.413279
32,0\H,4,1.09882267,1,112.70965787,2,50.14530189,0\C,1,1.87253225,4,99
.32415844,2,-101.81872237,0\C,6,1.56523835,1,112.98146739,4,-73.883953
19,0\C,7,1.54726133,6,104.54434259,1,-128.86673726,0\N,8,1.46284316,7,
104.21476669,6,-20.45603458,0\C,9,1.46193981,8,104.68889211,7,41.10596
532,0\H,6,1.09894406,1,109.37479059,4,47.86704412,0\H,7,1.09736782,6,1
12.47543791,1,-8.78235463,0\H,7,1.09520987,6,110.32989552,1,111.220420

48,0\H,8,1.09754167,7,113.26814174,6,-140.55838991,0\H,8,1.11004518,7,
109.96995631,6,99.09873586,0\H,10,1.09733956,9,110.54918473,8,-166.345
61967,0\H,10,1.11246559,9,111.67247495,8,73.70921928,0\C,9,1.45164475,
8,114.04266977,7,166.62903458,0\H,18,1.09703876,9,109.71645809,8,60.78
353794,0\H,18,1.09695667,9,109.7730127,8,179.26433342,0\H,18,1.1099986
4,9,113.03240594,8,-59.91321114,0\H,2,1.09669003,1,109.84340844,4,-173
.82024717,0\H,2,1.09702384,1,109.36358506,4,68.44326468,0\H,4,1.096709
48,1,109.74452137,2,171.35979147,0\H,4,1.09688651,1,109.44093559,2,-70
.83464053,0\\Version=AM64L-G03RevD.01\State=1-A\HF=-669.589308\MP2=-67
0.9693041\RMSD=3.464e-09\Thermal=0.\PG=C01 [X(C7H16N1P1)]\\@

189-Me$^+$

1\1\GINC-EDDY\SP\RMP2-FC\6-31+G(2d,p)\C8H19N1P1(1+)\CHRISTOPH\22-Oct-2
009\0\\#p MP2(FC)/6-31+g(2d,p) scf=tight\\b1mesp_1\\1,1\P\C,1,1.816877
27\H,2,1.09504256,1,108.94173395\H,2,1.09583679,1,109.82992017,3,121.5
238189,0\H,2,1.09498875,1,110.00671022,3,-118.50154523,0\C,1,1.8204876
4,2,108.9724638,5,166.80378125,0\H,6,1.09579608,1,110.10807519,2,176.0
577,0\H,6,1.09598142,1,109.68342895,2,-64.21590778,0\H,6,1.09479493,1,
110.53874353,2,55.55076856,0\C,1,1.81997474,2,109.31449471,6,-117.5390
5211,0\C,1,1.83653171,2,111.52442754,6,121.79410187,0\C,11,1.56991471,
1,112.19273973,2,53.40875441,0\C,12,1.55065004,11,104.09764898,1,-112.
34614744,0\N,13,1.4677099,12,104.32077961,11,18.59504743,0\C,14,1.4574
9919,13,104.42832461,12,-41.51643183,0\H,11,1.09783706,1,105.50211403,
2,175.69686366,0\H,12,1.0936958,11,110.19256122,1,127.66708587,0\H,12,
1.09669392,11,112.90951906,1,8.50853023,0\H,13,1.09522309,12,112.97079
162,11,138.67183594,0\H,13,1.10578347,12,109.77279713,11,-100.96540799
,0\H,15,1.10836492,14,113.17174762,13,-68.9070349,0\H,15,1.09748058,14
,110.98332076,13,170.70555243,0\C,14,1.46148987,13,113.94710234,12,-16
6.81024783,0\H,23,1.10520371,14,112.57651089,13,59.69178196,0\H,23,1.0
9563824,14,109.73293821,13,-179.28295907,0\H,23,1.09491214,14,109.3299
5285,13,-60.88861793,0\H,10,1.095752,1,110.08378294,2,175.57709767,0\H
,10,1.09530995,1,110.26639564,2,-64.2663169,0\H,10,1.09587244,1,109.90
253078,2,55.6568622,0\\Version=AM64L-G03RevD.01\State=1-A\HF=-709.0393
451\MP2=-710.5658501\RMSD=2.810e-09\Thermal=0.\PG=C01 [X(C8H19N1P1)]\\
@

190

1\1\GINC-GOLEM\SP\RMP2-FC\6-31+G(2d,p)\C7H16N1P1\CHRISTOPH\24-Oct-2009
\0\\#p MP2(FC)/6-31+g(2d,p) scf=tight\\a1sp_1\\0,1\P\C,1,1.86123042\H,
2,1.0959586,1,108.05931879\H,2,1.09654525,1,109.248217,3,-117.45141108
,0\H,2,1.09820787,1,113.89386393,3,121.17397261,0\C,1,1.86557015,2,100
.79103856,3,-164.75375955,0\H,6,1.0968269,1,109.03367851,2,-71.3777506
3,0\H,6,1.0983055,1,113.66074475,2,49.55373147,0\H,6,1.09720888,1,109.
58014176,2,171.29056191,0\C,1,1.89188878,2,101.02307633,6,103.04532202
,0\N,10,1.47908192,1,108.1296521,2,73.69151065,0\C,11,1.46207809,10,10
7.48520809,1,-151.25577437,0\C,12,1.53124509,11,102.8829754,10,40.3620
0259,0\C,13,1.54607001,12,102.41411081,11,-40.18786384,0\H,10,1.108885
74,1,105.39923111,2,-167.72404797,0\H,12,1.09786216,11,110.32266962,10
,161.98830946,0\H,12,1.11102454,11,112.04244998,10,-78.06972293,0\H,13
,1.09666627,12,109.65967556,11,77.29214069,0\H,13,1.09572138,12,112.98
595357,11,-161.90290972,0\H,14,1.09548543,13,111.96505025,12,149.21496
418,0\H,14,1.09681668,13,110.35326767,12,-91.9178069,0\C,11,1.45290426
,10,114.50363995,1,80.81658716,0\H,22,1.09695245,11,109.59159375,10,-1
76.2438445,0\H,22,1.11002337,11,112.68570965,10,63.27718453,0\H,22,1.0
9543148,11,109.81548611,10,-57.55552528,0\\Version=AM64L-G03RevD.01\St
ate=1-A\HF=-669.5856563\MP2=-670.968454\RMSD=6.099e-09\Thermal=0.\PG=C
01 [X(C7H16N1P1)]\\@

190-Me$^+$

1\1\GINC-NAUTILUS\SP\RMP2-FC\6-31+G(2d,p)\C8H19N1P1(1+)\CHRISTOPH\22-O
ct-2009\0\\#p MP2(FC)/6-31+g(2d,p) scf=tight\\a1mesp_1\\1,1\P\C,1,1.82
255165\H,2,1.09600364,1,109.88770375\H,2,1.09583949,1,110.16269133,3,1
19.79924647,0\H,2,1.09532693,1,110.44382779,3,-120.02706201,0\C,1,1.81
857014,2,108.21586983,5,178.94984082,0\H,6,1.09605101,1,109.55005881,2
,-59.44653207,0\H,6,1.09463855,1,109.42339897,2,-178.95472582,0\H,6,1.
09545073,1,110.51794932,2,60.44322498,0\C,1,1.81733679,6,107.90596177,
2,118.12232528,0\H,10,1.09488935,1,109.06447377,6,65.00996502,0\H,10,1
.09488998,1,110.58741861,6,-174.57493551,0\H,10,1.09577008,1,109.65529
207,6,-54.56380082,0\C,1,1.86650251,10,110.36341897,6,-120.84607236,0\
N,14,1.46541739,1,107.84700377,10,64.96389255,0\C,15,1.48074841,14,108
.59443142,1,-128.70499512,0\C,16,1.53022272,15,104.47612652,14,28.6332
2824,0\C,17,1.54243673,16,102.39302028,15,-37.69148926,0\H,14,1.103012
97,1,105.74947346,10,-173.76151368,0\H,16,1.09534934,15,109.87141827,1
4,150.77561058,0\H,16,1.10403758,15,110.67092624,14,-90.05813677,0\H,1
7,1.09386296,16,112.4892868,15,-158.24231902,0\H,17,1.09633825,16,110.
76383775,15,81.15460411,0\H,18,1.09567981,17,110.36991436,16,-83.05714
187,0\H,18,1.09677072,17,113.38866414,16,157.17114986,0\C,15,1.4644783
3,14,115.40882576,1,103.01428803,0\H,26,1.10442047,15,112.43896439,14,
63.38410027,0\H,26,1.09466434,15,108.95099534,14,-176.51786017,0\H,26,
1.09485842,15,110.95738889,14,-58.16829094,0\\Version=AM64L-G03RevD.01
\State=1-A\HF=-709.0387636\MP2=-710.5652253\RMSD=3.570e-09\Thermal=0.\
PG=C01 [X(C8H19N1P1)]\\@

191

1\1\GINC-CIPCLU07\SP\RMP2-FC\6-31+G(2d,p)\C9H19N2P1\C2175\27-May-2010\
0\\#p MP2(FC)/6-31+G(2d,p) scf=tight\\maza2sp_29\\0,1\P\C,1,1.87514806
\C,2,1.55903798,1,116.01315766\C,3,1.54703251,2,85.77097328,1,141.5911
8877,0\N,4,1.47958363,3,89.51171465,2,-16.87308434,0\H,2,1.10733791,1,
107.44102826,3,-123.74882384,0\H,3,1.09288786,2,116.65643504,1,-99.760
99073,0\H,3,1.09470233,2,113.0781223,1,28.43138279,0\H,4,1.0978781,3,1
18.30511775,2,-134.8603391,0\H,4,1.10515823,3,111.77123985,2,97.515853
9,0\C,1,1.85849775,2,101.41867022,5,36.13380931,0\H,11,1.09598028,1,10
9.89396841,2,85.03242177,0\H,11,1.096039,1,111.84668879,2,-36.15622531
,0\H,11,1.09633292,1,107.95122885,2,-157.62944545,0\H,1,2.45199068,11,
124.89966721,2,95.42665427,0\C,5,1.44951067,4,117.015494,3,140.3983096
2,0\H,16,1.09746553,5,109.46254769,4,66.84534315,0\H,16,1.10971327,5,1
13.00482209,4,-53.64573983,0\H,16,1.09536475,5,110.06037941,4,-174.792
12855,0\C,1,1.87596316,11,101.35334582,2,106.27807506,0\C,20,1.5610820
3,1,126.16926975,11,-26.51952194,0\C,21,1.54783909,20,85.5639448,1,133
.73538778,0\N,22,1.47662914,21,89.10603575,20,-18.38493589,0\H,21,1.09
32362,20,116.67100471,1,-108.46403977,0\H,21,1.09314911,20,113.4723878
5,1,20.45411459,0\H,22,1.10597949,21,111.58735839,20,95.90113459,0\H,2
2,1.098066,21,118.57678144,20,-136.33046013,0\C,23,1.44922445,22,118.7
3737327,21,142.68998859,0\H,28,1.10947779,23,112.89533344,22,-51.88473
45,0\H,28,1.097313,23,109.66922225,22,68.9457946,0\H,28,1.09636768,23,
109.31611393,22,-172.64778909,0\\Version=AM64L-G03RevD.01\State=1-A\HF
=-801.4485544\MP2=-803.3012613\RMSD=4.526e-09\Thermal=0.\PG=C01 [X(C9H
19N2P1)]\\@

191-Me$^+$

1\1\GINC-CIPCLU09\SP\RMP2-FC\6-31+G(2d,p)\C10H22N2P1(1+)\C2175\27-May-
2010\0\\#p MP2(FC)/6-31+G(2d,p) scf=tight\\maza2mesp_4\\1,1\C\C,1,1.56
151647\C,2,1.55086588,1,85.35792869\N,1,1.47234345,2,89.89323925,3,15.
03819403,0\H,1,1.10415608,4,115.4071532,3,99.30624192,0\H,2,1.0940416,
1,114.64558243,4,-98.56466596,0\H,2,1.09161294,1,115.44691542,4,132.86
322126,0\H,3,1.09482471,2,117.7585705,1,-132.3399731,0\H,3,1.10050801,

2,112.06639847,1,99.50909374,0\H,1,3.92140641,4,92.08420931,3,-117.646
61077,0\H,1,3.23984997,4,78.96196547,3,-170.39766906,0\C,4,1.45762046,
1,119.27057115,2,-138.52084404,0\H,12,1.09620686,4,110.41985037,1,-69.
54294212,0\H,12,1.09508107,4,108.64160695,1,172.64947502,0\H,12,1.1051
4425,4,112.88855363,1,52.33260251,0\C,1,3.06808424,4,98.71438127,12,69
.32116414,0\C,16,1.56153051,1,150.96299773,4,131.11647212,0\C,17,1.550
88425,16,85.3568362,1,123.06168783,0\N,16,1.47232712,1,98.73146008,4,-
123.05967547,0\H,17,1.09160689,16,115.44997551,1,-119.10974725,0\H,17,
1.09404798,16,114.63931761,1,9.46629498,0\H,18,1.09483246,17,117.75792
499,16,-132.34581499,0\H,18,1.10051154,17,112.06719939,16,99.50722379,
0\C,19,1.45762058,16,119.2714803,1,69.30133478,0\H,24,1.10514468,19,11
2.88787893,16,52.31786173,0\H,24,1.09507801,19,108.64228086,16,172.633
8934,0\H,24,1.09620364,19,110.41732453,16,-69.55859022,0\C,1,2.9731312
2,4,88.1514843,12,127.79709155,0\P,28,1.81859884,1,36.29435381,4,-135.
72029315,0\C,29,1.81858774,28,110.9182905,1,-119.34322037,0\H,30,1.095
07232,29,110.65575333,28,-47.19277034,0\H,30,1.09547195,29,108.4220663
8,28,-166.8635771,0\H,30,1.09540248,29,110.0398005,28,72.99479525,0\H,
28,1.09547517,1,82.84592145,4,-0.57552073,0\H,28,1.09507714,1,96.56603
083,4,107.98437047,0\\Version=AM64L-G03RevD.01\State=1-A\HF=-840.90405
09\MP2=-842.8984045\RMSD=3.110e-09\Thermal=0.\PG=C01 [X(C10H22N2P1)]\\
@

192

1\1\GINC-NAUTILUS\SP\RMP2-FC\6-31+G(2d,p)\C13H27N2P1\CHRISTOPH\11-Jun-
2010\0\\#p MP2(FC)/6-31+g(2d,p) scf=tight\\pia2sp_3\\0,1\P\C,1,4.29841
445\C,2,1.53686284,1,19.82677038\C,3,1.54529325,2,112.53117059,1,2.126
21699,0\N,4,1.48076087,3,110.14604025,2,-54.9350713,0\C,5,1.47209783,4
,111.94106695,3,55.30537342,0\C,6,1.52980736,5,112.81668298,4,-58.1051
7347,0\H,2,1.10122096,1,101.21763052,4,-57.35632347,0\H,2,1.09872008,1
,130.00531002,4,179.00191183,0\H,3,1.09768651,2,108.52773894,1,-118.47
659205,0\H,3,1.09667886,2,109.94866669,1,124.59885751,0\H,4,1.11199026
,3,108.45508455,2,65.05136203,0\H,6,1.11232508,5,110.6834265,4,64.3458
4977,0\H,6,1.09809218,5,107.39465425,4,-179.47116345,0\H,7,1.09926561,
6,109.12664364,5,-62.97138282,0\H,7,1.09850088,6,109.32915236,5,179.74
819445,0\C,1,3.42565554,4,93.54886756,3,-98.82606153,0\C,17,1.53805121
,1,57.53349822,4,67.61118552,0\C,18,1.54626584,17,112.23620931,1,35.59
904948,0\N,19,1.46710306,18,107.82859686,17,56.3972934,0\C,20,1.465829
21,19,114.68319188,18,-58.41475273,0\C,21,1.53493676,20,111.16778291,1
9,58.51242761,0\H,17,1.10000523,1,59.7140038,4,-79.49554501,0\H,17,1.0
9848581,1,148.34083012,4,-7.33482646,0\H,18,1.09601054,17,110.61482355
,1,-88.9286056,0\H,18,1.09990458,17,108.83900787,1,153.74060209,0\H,19
,1.10018744,18,108.66643603,17,171.32368117,0\H,21,1.10752084,20,111.7
0430491,19,-64.4362512,0\H,21,1.09841383,20,107.36647987,19,178.560401
07,0\H,22,1.09938344,21,108.77428858,20,66.42665757,0\H,22,1.09848854,
21,109.47370574,20,-176.39550704,0\C,1,1.8697553,4,98.83471561,3,52.98
979207,0\H,32,1.0949933,1,110.10864232,4,162.14277595,0\C,20,1.454035,
19,115.53902089,18,168.42645694,0\H,34,1.10653029,20,114.24161081,19,7
5.33378878,0\H,34,1.09794634,20,109.0958505,19,-164.298557,0\H,34,1.09
704799,20,109.66337597,19,-46.1413439,0\C,5,1.463752,4,113.29318608,3,
178.2825574,0\H,38,1.09666001,5,108.83190538,4,-179.96605891,0\H,38,1.
1091842,5,112.7352855,4,-60.12970194,0\H,38,1.09302786,5,110.82395078,
4,60.87679695,0\H,32,1.09718406,1,107.20022302,4,44.66167259,0\H,32,1.
09794002,1,114.10271598,4,-75.65288194,0\\Version=AM64L-G03RevD.01\Sta
te=1-A\HF=-957.6536194\MP2=-960.137814\RMSD=7.388e-09\Thermal=0.\PG=C0
1 [X(C13H27N2P1)]\\@

192-Me$^+$

1\1\GINC-AZAZEL\SP\RMP2-FC\6-31+G(2d,p)\C14H30N2P1(1+)\CHRISTOPH\25-Ma

y-2012\0\\#p MP2(FC)/6-31+g(2d,p) scf=tight\\pia2mesp_1\\1,1\P\C,1,1.8
3283174\H,2,1.09400347,1,110.64698739\H,2,1.09430978,1,110.83053836,3,
-121.03523793,0\H,2,1.09617499,1,107.37025377,3,118.2178712,0\C,1,1.82
567631,2,110.03921562,3,128.61289073,0\H,6,1.09412547,1,108.11378195,2
,-77.42207468,0\H,6,1.09357539,1,110.84564277,2,43.87638702,0\H,6,1.09
492978,1,110.14183662,2,164.87774377,0\H,1,2.4529837,6,129.01914398,2,
112.76693754,0\C,1,4.13385487,6,125.93888255,2,-101.31059982,0\C,1,1.8
9594253,6,108.80763564,2,-114.52037317,0\C,12,1.55275956,1,107.0148385
,6,34.67796401,0\C,13,1.53680471,12,111.44379441,1,174.26016955,0\C,11
,1.52705639,1,99.37294388,6,26.37333729,0\H,11,1.10778185,1,105.595422
19,6,-87.04664166,0\H,11,1.09577175,1,124.14347364,6,148.92721957,0\H,
12,1.10721009,1,106.75069343,6,-81.41643467,0\H,13,1.09548741,12,109.3
8419746,1,-63.97329982,0\H,13,1.09995985,12,109.57971993,1,53.98020496
,0\H,14,1.09992976,13,109.66255192,12,65.45926921,0\H,14,1.0964485,13,
109.17718957,12,-177.59616031,0\H,15,1.09862022,11,109.61256346,1,77.0
7414343,0\H,15,1.0964316,11,109.01780982,1,-165.6548048,0\N,12,1.46370
121,1,110.01997228,6,156.04479367,0\C,25,1.46412875,12,113.54740639,1,
63.75975982,0\H,26,1.09700954,25,111.18862049,12,-64.31332046,0\H,26,1
.10575096,25,113.25486785,12,58.314469,0\H,26,1.09434394,25,108.878247
76,12,178.4396347,0\C,1,4.12810368,6,86.86708972,2,111.25863619,0\N,30
,1.48502554,1,18.25985371,6,-58.38733709,0\C,31,1.46507277,30,112.5944
4665,1,-13.16934168,0\C,32,1.5517826,31,112.43167121,30,51.27073572,0\
C,33,1.53671829,32,112.07672466,31,-51.80362872,0\C,30,1.52607164,1,99
.05871938,6,82.80857341,0\H,30,1.09567297,1,123.85259025,6,-39.2535986
7,0\H,30,1.10767754,1,106.29566963,6,-163.79440122,0\H,33,1.0999937,32
,109.52285159,31,68.66300288,0\H,33,1.09559238,32,109.11209779,31,-173
.83153604,0\H,34,1.0964114,33,109.19663215,32,176.58503179,0\H,34,1.09
985727,33,109.70258765,32,-66.40565027,0\H,35,1.09641802,30,108.983720
52,1,167.14346143,0\H,35,1.0985524,30,109.68026005,1,-75.52712395,0\C,
31,1.46631236,30,108.59772197,1,-139.12258549,0\H,44,1.10641884,31,113
.00776845,30,71.5736158,0\H,44,1.09543414,31,111.47416988,30,-165.8809
3028,0\H,44,1.09401824,31,108.81952971,30,-48.4025445,0\\Version=AM64L
-G03RevD.01\State=1-A\HF=-997.106593\MP2=-999.7357208\RMSD=3.129e-09\T
hermal=0.\PG=C01 [X(C14H30N2P1)]\\@

193

1\1\GINC-CIPCLU05\SP\RMP2-FC\6-31+G(2d,p)\C9H20N1P1\C2175\28-Jun-2010\
0\\#p MP2(FC)/6-31+G(2d,p) scf=tight\\n7d1sp_2\\0,1\P\C,1,1.86868435\H
,2,1.09605028,1,110.70269986\H,2,1.09885006,1,112.71774382,3,121.95371
821,0\H,2,1.09706467,1,108.82498515,3,-117.62410956,0\H,1,2.46925176,2
,82.52313865,3,-90.1832699,0\C,1,1.86787119,2,97.76554943,3,-174.65825
96,0\H,7,1.09548166,1,110.66630721,2,179.52889543,0\H,7,1.09719459,1,1
08.64804675,2,-62.92023715,0\H,7,1.09898765,1,112.80682288,2,57.340799
68,0\C,1,1.8898909,7,100.90216897,2,-101.74108602,0\N,11,2.97336348,1,
165.28855993,7,140.57132418,0\C,12,1.46804165,11,82.05843144,1,139.103
87197,0\C,13,1.53962705,12,111.66672397,11,39.14133319,0\C,14,1.541697
77,13,116.69075257,12,-69.84737409,0\H,13,1.09750257,12,107.75185479,1
1,157.11757088,0\H,13,1.10686001,12,112.39041875,11,-86.58001983,0\H,1
4,1.09891916,13,106.79095303,12,52.64896262,0\H,14,1.10084388,13,108.6
8662541,12,167.66130141,0\H,15,1.09873112,14,107.18677403,13,173.46573
775,0\H,15,1.10117089,14,109.45808057,13,-71.93256588,0\C,11,1.5505238
5,1,109.34790111,7,172.60018588,0\H,22,1.09856294,11,108.19332384,1,84
.85913469,0\H,22,1.09891881,11,111.32551826,1,-30.27852955,0\C,12,1.46
151359,11,61.47858143,1,14.79096808,0\H,25,1.10050675,12,106.79924551,
11,-101.20359611,0\C,12,1.45580598,11,164.32560639,1,-47.3112737,0\H,2
7,1.09782062,12,109.38789352,11,0.59431187,0\H,27,1.10983399,12,113.66
081187,11,120.79211139,0\H,27,1.09644485,12,110.01959427,11,-117.66029
643,0\H,25,1.11197317,12,111.54643368,11,143.08579049,0\\Version=AM64L

-G03RevD.01\State=1-A\HF=-747.6570009\MP2=-749.349522\RMSD=5.407e-09\T
hermal=0.\PG=C01 [X(C9H20N1P1)]\\@

193-Me⁺

1\1\GINC-CIPCLU05\SP\RMP2-FC\6-31+G(2d,p)\C10H23N1P1(1+)\C2175\28-Jun-
2010\0\\#p MP2(FC)/6-31+G(2d,p) scf=tight\\n7d1mesp_4\\1,1\C\H,1,1.095
91225\H,1,1.09430873,2,108.97373205\H,1,1.09590794,3,108.68761798,2,11
8.49745541,0\H,1,3.15631509,3,103.10327484,4,170.4690993,0\C,1,2.95521
837,3,89.16648501,4,91.34937723,0\H,6,1.09538312,1,145.85127637,3,130.
20526581,0\H,6,1.09546316,1,90.55455589,3,3.93069793,0\H,6,1.0957077,1
,89.79410052,3,-104.91085021,0\C,6,3.01597597,1,60.6775525,3,95.415220
4,0\N,10,2.84237871,6,151.31739997,1,-176.8271965,0\C,11,1.47185222,10
,84.34224337,6,75.32303464,0\C,12,1.53895279,11,111.08535723,10,38.329
46838,0\C,13,1.54361249,12,116.278636,11,-66.63007263,0\H,12,1.0956423
5,11,108.53085967,10,156.31859731,0\H,12,1.10495498,11,112.14238453,10
,-87.05868112,0\H,13,1.09762543,12,107.27681453,11,55.48258923,0\H,13,
1.09857205,12,109.47552641,11,171.32955266,0\H,14,1.09862867,13,107.29
086825,12,171.03317923,0\H,14,1.09972626,13,109.73427561,12,-73.388617
17,0\C,10,1.55753551,6,92.21746922,1,160.29248974,0\H,21,1.09866026,10
,112.642269,6,-53.7783115,0\H,21,1.09700943,10,107.88880287,6,62.74396
611,0\C,11,1.45624628,10,64.8682969,6,-44.860932,0\H,24,1.09867774,11,
107.18504556,10,-102.37312216,0\C,11,1.46364664,10,160.65818329,6,-126
.72415812,0\H,26,1.0950154,11,109.60312692,10,-100.1461459,0\H,26,1.10
56768,11,113.30306749,10,138.5590409,0\H,26,1.09648028,11,109.44041295
,10,17.92619612,0\H,24,1.10865771,11,112.85445543,10,140.56732803,0\P,
6,1.81978408,1,35.76242248,10,33.50441641,0\C,31,1.82161889,6,108.7270
8165,1,116.05992444,0\H,32,1.09592417,31,109.83793506,6,179.74466232,0
\H,32,1.09588271,31,109.614646,6,-60.74992066,0\H,32,1.09443779,31,110
.96840917,6,59.27756287,0\\Version=AM64L-G03RevD.01\State=1-A\HF=-787.
1102329\MP2=-788.9473198\RMSD=2.410e-09\Thermal=0.\PG=C01 [X(C10H23N1P
1)]\\@

194

1\1\GINC-TOFU\SP\RMP2-FC\6-31+G(2d,p)\C6H11N2P1\CHRISTOPH\17-Feb-2012\
0\\#p MP2(FC)/6-31+g(2d,p) scf=tight\\imi41sp_2\\0,1\P\C,1,1.86648256\
H,2,1.09720739,1,109.34765398\H,2,1.09627801,1,111.53068789,3,121.2045
2384,0\H,2,1.09624607,1,109.54156572,3,-118.16332604,0\C,1,1.8664761,2
,98.93193345,5,175.24848736,0\H,6,1.09720718,1,109.34769019,2,66.56991
911,0\H,6,1.09624597,1,109.54322125,2,-175.26570708,0\H,6,1.09627586,1
,111.52918411,2,-54.63321638,0\C,1,1.83102282,6,99.40416627,2,101.1673
9595,0\C,10,1.38054062,1,126.12698395,6,129.62743831,0\N,11,1.37798278
,10,106.42851494,1,179.99040406,0\C,12,1.37033211,11,106.14656604,10,0
.00108114,0\N,13,1.31358369,12,112.65014289,11,-0.0022336,0\H,11,1.081
55334,10,131.61093301,1,-0.01978442,0\H,13,1.08435459,12,121.45483205,
11,-179.99744762,0\C,12,1.4529116,11,126.9656707,10,179.97602376,0\H,1
7,1.0935506,12,108.85805307,11,179.88414581,0\H,17,1.09592277,12,110.7
114334,11,-60.66103081,0\H,17,1.09590471,12,110.70145334,11,60.4381709
7,0\\Version=AM64L-G03RevD.01\State=1-A\HF=-683.2626271\MP2=-684.63293
54\RMSD=9.981e-09\Thermal=0.\PG=C01 [X(C6H11N2P1)]\\@

194-Me⁺

1\1\GINC-BORIX\SP\RMP2-FC\6-31+G(2d,p)\C7H14N2P1(1+)\CHRISTOPH\17-Feb-
2012\0\\#p MP2(FC)/6-31+g(2d,p) scf=tight\\imi41mesp_1\\1,1\C\C,1,2.93
007587\N,2,1.38158282,1,96.11231768\C,3,1.31418292,2,104.50453967,1,-1
49.58156628,0\N,4,1.37107389,3,112.47844916,2,-0.00130493,0\C,5,1.3678
7228,4,107.1092512,3,0.00258253,0\C,2,2.93020117,1,60.4662853,3,93.613
52611,0\P,2,1.77756087,1,35.84867022,7,36.2283044,0\C,8,1.81684249,2,1
10.17677588,1,120.71039226,0\H,9,1.09568913,8,109.56062327,2,-179.9733

5143,0\H,9,1.09538795,8,110.22463979,2,-60.39453972,0\H,9,1.09538211,8
,110.22273874,2,60.45043297,0\H,6,1.08140971,5,121.98024541,4,-179.992
35082,0\C,5,1.46475622,4,126.45636308,3,179.99373577,0\H,14,1.09192072
,5,108.57994054,4,0.0679542,0\H,14,1.09375134,5,110.10790127,4,119.423
94803,0\H,14,1.09375868,5,110.10913188,4,-119.28756805,0\H,4,1.0829021
,3,125.55908766,2,179.99786051,0\H,7,1.09517275,2,92.93508232,1,157.44
939387,0\H,7,1.09562293,2,86.45195075,1,-93.84852198,0\H,7,1.09567367,
2,145.17714424,1,26.11598395,0\H,1,1.09563828,8,108.5027301,2,-53.6222
1313,0\H,1,1.09516903,8,109.78743967,2,65.20198193,0\H,1,1.09566683,8,
110.53742845,2,-173.83032763,0\\Version=AM64L-G03RevD.01\State=1-A\HF=
-722.7170376\MP2=-724.230945\RMSD=5.474e-09\Thermal=0.\PG=C01 [X(C7H14
N2P1)]\\@

195

1\1\GINC-CIPCLU10\SP\RMP2-FC\6-31+G(2d,p)\C12H24N3P1\C2175\06-Jun-2010
\0\\#p MP2(FC)/6-31+G(2d,p) scf=tight\\maza3sp_29\\0,1\P\C,1,1.8772166
3\C,2,1.55579565,1,126.75058803\C,3,1.54856084,2,85.2914793,1,139.0736
2838,0\N,4,1.47975962,3,89.02490642,2,-19.27206743,0\H,2,1.10879978,1,
105.96339657,5,123.21859573,0\H,3,1.09141646,2,115.93981603,1,-101.803
83101,0\H,3,1.09375099,2,113.58806961,1,26.64635057,0\H,4,1.0979474,3,
118.75708495,2,-137.13791428,0\H,4,1.10578822,3,111.61885799,2,94.7505
4567,0\H,1,2.44446792,2,123.8842991,5,172.31613178,0\H,1,2.51569819,2,
85.04477934,5,91.96473371,0\C,5,1.45002852,4,118.71134,3,143.07480659,
0\H,13,1.09637022,5,109.35078442,4,-172.948181,0\H,13,1.09717441,5,109
.68149897,4,68.61078021,0\H,13,1.10937781,5,112.86230709,4,-52.2208018
,0\C,1,1.87666492,2,104.56305955,5,77.85828515,0\C,17,1.55879008,1,117
.24161351,2,165.25979801,0\C,18,1.54890513,17,85.30110717,1,136.727819
59,0\N,19,1.47517093,18,88.8308129,17,-19.22633738,0\H,18,1.09346292,1
7,116.9397903,1,-104.48678623,0\H,18,1.09529656,17,112.76019538,1,24.3
3610948,0\H,19,1.10674611,18,111.58396703,17,95.22222203,0\H,19,1.0980
0428,18,118.42244473,17,-137.15827905,0\C,20,1.44889448,19,119.1253796
6,18,143.95590727,0\H,25,1.09756812,20,109.49218543,19,68.36519908,0\H
,25,1.09666444,20,108.86590473,19,-172.90531134,0\H,25,1.11066114,20,1
12.91525235,19,-52.07869684,0\C,1,1.87696769,17,100.7947628,20,163.845
28154,0\N,29,1.49058017,1,121.46272037,17,75.19759542,0\C,30,1.4796061
7,29,90.50066621,1,140.92188353,0\C,31,1.54751882,30,89.25096818,29,-1
9.16202499,0\H,31,1.10555886,30,112.77001918,29,94.02448773,0\H,31,1.0
9781952,30,114.73133661,29,-140.4624334,0\H,32,1.09293242,31,117.60395
64,30,136.21883969,0\H,32,1.09482774,31,112.89311344,30,-94.87388891,0
\C,30,1.44971516,29,118.41779272,1,-96.89252735,0\H,37,1.09743974,30,1
09.50398147,29,-173.38554682,0\H,37,1.10969832,30,113.06788806,29,-52.
79346312,0\H,37,1.09665422,30,109.92693475,29,68.30143252,0\\Version=A
M64L-G03RevD.01\State=1-A\HF=-972.3757544\MP2=-974.8573652\RMSD=4.623e
-09\Thermal=0.\PG=C01 [X(C12H24N3P1)]\\@

195-Me$^+$

1\1\GINC-GOLEM\SP\RMP2-FC\6-31+G(2d,p)\C13H27N3P1(1+)\CHRISTOPH\11-Jun
-2010\0\\#p MP2(FC)/6-31+g(2d,p) scf=tight\\maza3mesp_157\\1,1\C\C,1,1
.56082322\C,2,1.55008404,1,84.92513322\N,1,1.46951566,2,89.68168175,3,
17.19325423,0\H,1,1.10526968,4,114.22912922,3,95.84986704,0\H,2,1.0918
9829,1,115.83719099,4,135.14037249,0\H,2,1.09336723,1,114.02188404,4,-
96.06010367,0\H,3,1.09554264,2,117.95921964,1,-134.68489493,0\H,3,1.10
141458,2,112.05800184,1,97.23773363,0\H,4,2.98261908,1,85.53683521,2,1
16.58697217,0\H,1,3.99446486,4,115.93720278,3,-165.86447771,0\C,4,1.45
799064,1,118.61918421,2,-140.85169465,0\H,12,1.09504456,4,109.50689394
,1,-63.39849607,0\H,12,1.09524994,4,108.95830589,1,177.50097361,0\H,12
,1.1055573,4,112.40993069,1,57.20914813,0\C,1,3.06758775,4,114.3797135
4,12,60.36001245,0\C,16,1.56006559,1,95.10286006,4,-123.12282157,0\C,1

7,1.55132977,16,85.28404986,1,107.15894116,0\N,16,1.47182528,1,91.6046
9367,4,-33.09231592,0\H,17,1.09166532,16,115.6111485,1,-134.88988738,0
\H,17,1.0939149,16,114.56359085,1,-6.32314538,0\H,18,1.10076822,17,112
.11626017,16,99.02336182,0\H,18,1.09484864,17,117.76736489,16,-132.789
85419,0\C,19,1.45783362,16,118.55952118,1,126.01681977,0\H,24,1.105190
03,19,112.78049092,16,52.10889438,0\H,24,1.09508013,19,108.88224833,16
,172.57681107,0\H,24,1.09671824,19,110.20647677,16,-69.54517543,0\C,16
,3.02511916,1,60.63167834,4,67.07493067,0\N,28,1.47362573,16,146.92153
085,1,60.07824826,0\C,29,1.48972924,28,91.08654356,16,114.38680461,0\C
,30,1.55055969,29,90.07723363,28,-13.2361396,0\H,30,1.10008417,29,112.
59442295,28,101.1619011,0\H,30,1.09506955,29,114.15948023,28,-133.6252
535,0\H,31,1.09211064,30,116.73910637,29,128.64742345,0\H,31,1.0944882
2,30,113.6753941,29,-103.06261462,0\C,29,1.45969049,28,118.24500898,16
,-123.8683876,0\H,36,1.10470206,29,112.48658542,28,-57.04275062,0\H,36
,1.0934709,29,109.9954069,28,63.58159689,0\H,36,1.09530687,29,108.8350
7502,28,-177.35832773,0\P,16,1.85416225,1,34.35032992,4,100.59678969,0
\C,40,1.82274415,16,108.13282987,1,118.80237366,0\H,41,1.09510743,40,1
10.53493881,16,41.44775671,0\H,41,1.09511908,40,110.27593428,16,-78.89
541949,0\H,41,1.09535923,40,108.27882051,16,160.99792467,0\\Version=AM
64L-G03RevD.01\State=1-A\HF=-1011.8337102\MP2=-1014.4572937\RMSD=2.938
e-09\Thermal=0.\PG=C01 [X(C13H27N3P1)]\\@

196

1\1\GINC-CIPCLU07\SP\RMP2-FC\6-31+G(2d,p)\C9H20N1P1\C2175\25-Jun-2010\
0\\#p MP2(FC)/6-31+G(2d,p) scf=tight\\n7b1sp_1\\0,1\P\C,1,1.86778577\H
,2,1.09585007,1,110.51675983\H,2,1.09721793,1,108.61611368,3,117.61081
902,0\H,2,1.09932065,1,113.03137546,4,120.13528646,0\H,1,2.45156177,2,
86.72504988,3,98.24660516,0\C,1,1.86258021,2,99.36541585,3,177.4062105
7,0\H,7,1.09536681,1,108.06381722,2,-164.1664852,0\H,7,1.09907543,1,11
2.80037715,2,-43.90459668,0\H,7,1.09658971,1,109.98204963,2,77.6233335
9,0\C,1,1.90223106,7,98.45002959,2,102.47851779,0\N,11,1.46670312,1,10
7.77532536,7,69.27286768,0\C,12,1.4619367,11,114.40975992,1,-147.45513
663,0\C,13,1.54469569,12,117.76568833,11,-70.09766191,0\C,14,1.5419728
6,13,115.25612599,12,55.06466802,0\C,15,1.54372214,14,113.72321831,13,
-70.92080904,0\C,11,1.55173684,1,111.18291098,7,-160.6326784,0\H,13,1.
1002842,12,107.14614463,11,52.69161004,0\H,13,1.1003727,12,107.5111956
8,11,166.8164866,0\H,14,1.10092481,13,107.40453709,12,177.31401538,0\H
,14,1.1007604,13,109.75930469,12,-67.86759117,0\H,15,1.09940065,14,108
.00674521,13,169.0936741,0\H,15,1.10151145,14,110.19982403,13,53.82907
858,0\H,16,1.09918108,15,109.03498111,14,-157.80506473,0\H,16,1.099516
13,15,107.76498614,14,-43.71322154,0\H,17,1.09902804,11,106.86337423,1
,69.89565868,0\H,17,1.10039734,11,109.87525205,1,-44.53505978,0\C,12,1
.45696248,11,116.46470557,1,74.45098978,0\H,28,1.10547345,12,114.91746
651,11,71.42099783,0\H,28,1.0978562,12,108.95428912,11,-167.70824663,0
\H,28,1.09580814,12,109.3351208,11,-49.93830355,0\\Version=AM64L-G03Re
vD.01\State=1-A\HF=-747.6532934\MP2=-749.3486382\RMSD=6.303e-09\Therma
l=0.\PG=C01 [X(C9H20N1P1)]\\@

196-Me$^+$

1\1\GINC-CIPCLU05\SP\RMP2-FC\6-31+G(2d,p)\C10H23N1P1(1+)\C2175\25-Jun-
2010\0\\#p MP2(FC)/6-31+G(2d,p) scf=tight\\n7b1mesp_4\\1,1\P\C,1,1.818
75812\H,2,1.09438617,1,109.09068664\H,2,1.09580629,1,109.82061165,3,-1
19.67678623,0\H,2,1.09563425,1,110.39698647,3,120.32278213,0\C,1,1.819
08368,2,107.91658513,3,63.86713144,0\H,6,1.0958193,1,109.54863441,2,56
.60110344,0\H,6,1.09501519,1,110.68969235,2,176.51776879,0\H,6,1.09459
932,1,109.27218819,2,-62.82165053,0\C,1,1.82554037,2,108.01660579,6,11
7.20025751,0\H,10,1.09505651,1,110.39350752,2,-174.80159386,0\H,10,1.0
9620971,1,110.10919259,2,-54.68588258,0\H,10,1.09611375,1,109.99257346

,2,65.02711672,0\C,1,1.88505064,2,109.06088421,6,-119.72703885,0\C,14,
1.54666288,1,110.95502171,2,-172.34923056,0\C,15,1.54714663,14,113.544
62315,1,-177.73678576,0\C,16,1.5387578,15,113.93978991,14,85.00476794,
0\C,17,1.53666587,16,115.35969956,15,-65.74566303,0\C,18,1.54307671,17
,114.86528917,16,54.85256453,0\N,14,1.45396726,1,105.57018855,2,57.203
15852,0\H,14,1.10072322,1,104.08377537,2,-57.53813269,0\H,15,1.0991775
1,14,111.11286393,1,-54.36032364,0\H,15,1.0989355,14,107.08351773,1,61
.54125422,0\H,16,1.09730436,15,107.63118723,14,-154.6514802,0\H,16,1.1
0013981,15,109.71401642,14,-39.57283297,0\H,17,1.10100033,16,108.45397
289,15,57.0138055,0\H,17,1.09758765,16,108.61474845,15,171.85004784,0\
H,18,1.09856622,17,108.69489017,16,175.40561554,0\H,18,1.09892773,17,1
10.13311566,16,-69.08127974,0\H,19,1.0982835,18,109.82257701,17,48.349
62038,0\H,19,1.09743791,18,109.18619814,17,164.34524473,0\C,20,1.46706
792,14,114.28317835,1,-111.56969537,0\H,32,1.10183243,20,113.67001914,
14,-66.49500515,0\H,32,1.09620483,20,110.16855466,14,54.80292049,0\H,3
2,1.09500372,20,109.00217587,14,172.65693994,0\\Version=AM64L-G03RevD.
01\State=1-A\HF=-787.1094779\MP2=-788.9479324\RMSD=4.404e-09\Thermal=0
.\PG=C01 [X(C10H23N1P1)]\\@

197

1\1\GINC-NAUTILUS\SP\RMP2-FC\6-31+G(2d,p)\C13H27N2P1\CHRISTOPH\22-Jun-
2010\0\\#p MP2(FC)/6-31+g(2d,p) scf=tight\\pic2sp_1\\0,1\P\H,1,2.48214
775\C,1,1.86891771,2,82.62063645\H,3,1.09760025,1,113.85191185,2,27.94
525196,0\H,3,1.09618093,1,109.08097139,2,-93.36364826,0\H,3,1.09644352
,1,109.51066948,5,-116.91024521,0\C,1,4.30184905,3,91.60834307,5,-168.
39833315,0\C,7,1.53735768,1,22.8711232,3,50.60616446,0\C,8,1.54145559,
7,110.92874255,1,0.37723277,0\C,9,1.54527211,8,109.23939857,7,-52.9383
182,0\C,10,1.53573903,9,111.06975523,8,52.89769077,0\N,7,1.46236062,1,
97.69592595,3,-77.64909216,0\H,7,1.11311467,1,99.26371477,3,169.150651
25,0\H,7,1.0986632,1,132.57854498,3,45.83158005,0\H,8,1.09813392,7,108
.28758872,1,-120.48986503,0\H,8,1.09821222,7,108.88034909,1,122.865644
58,0\H,9,1.10319062,8,107.96336021,7,62.93805467,0\H,10,1.09813833,9,1
10.42493236,8,174.62521039,0\H,10,1.09898705,9,109.99848928,8,-66.9532
6561,0\H,11,1.11281892,10,109.30347982,9,66.61071812,0\H,11,1.09848156
,10,109.80027651,9,-176.37380893,0\C,12,1.45582307,7,111.49200373,1,-1
65.94780093,0\H,22,1.10972948,12,113.24742026,7,62.45615902,0\H,22,1.0
9699367,12,109.65299893,7,-176.84392602,0\H,22,1.09704591,12,109.67302
051,7,-58.2694901,0\C,1,4.22021071,3,94.4612283,9,125.10588033,0\C,26,
1.53545881,1,19.53475617,3,62.66463191,0\C,27,1.54517471,26,111.250239
17,1,-5.00112156,0\C,28,1.54330609,27,108.73609461,26,53.1378889,0\C,2
9,1.53636789,28,111.2004436,27,-52.98212337,0\N,30,1.46172492,29,111.3
3699492,28,56.89177386,0\H,26,1.09855046,1,128.98719053,3,72.76641839,
0\H,26,1.11306388,1,102.26127806,3,-51.19966761,0\H,27,1.098954,26,108
.80621081,1,115.68713244,0\H,27,1.09813389,26,108.87362935,1,-127.4559
2081,0\H,29,1.09928872,28,109.57462071,27,67.39704112,0\H,29,1.0982331
1,28,110.95114837,27,-174.11527765,0\H,30,1.11317819,29,109.28307647,2
8,-66.4930974,0\H,30,1.09850989,29,109.68884845,28,176.63698932,0\C,31
,1.45530279,30,111.54298409,29,175.27747141,0\H,40,1.09704006,31,109.6
5687502,30,-58.16456407,0\H,40,1.10991277,31,113.26052126,30,62.547211
72,0\H,40,1.09700019,31,109.65768602,30,-176.73757203,0\\Version=AM64L
-G03RevD.01\State=1-A\HF=-957.6735894\MP2=-960.1481951\RMSD=4.715e-09\
Thermal=0.\PG=C01 [X(C13H27N2P1)]\\@

197-Me$^+$

1\1\GINC-NAUTILUS\SP\RMP2-FC\6-31+G(2d,p)\C14H30N2P1(1+)\CHRISTOPH\22-
Jun-2010\0\\#p MP2(FC)/6-31+g(2d,p) scf=tight\\pic2mesp_1\\1,1\H\H,1,4
.22160949\C,2,4.4869678,1,79.65407551\C,3,1.53888378,2,17.20379895,1,6
8.81705956,0\C,4,1.54896865,3,109.83141018,2,-109.03826417,0\C,5,1.549

54481,4,110.31868001,3,-53.05739592,0\C,6,1.53898069,5,109.69114344,4,
53.21135133,0\N,3,1.45775306,2,127.35270943,1,52.47314838,0\H,3,1.1115
9096,2,96.19260299,1,-71.55177777,0\H,3,1.09633644,2,103.47755413,1,17
9.20419167,0\H,4,1.09800192,3,108.37155137,2,130.65477718,0\H,4,1.0975
6914,3,109.12256074,2,13.10091576,0\H,5,1.10201569,4,108.54663256,3,65
.21308569,0\H,6,1.09718742,5,111.18237652,4,174.02243695,0\H,6,1.09762
223,5,110.22396333,4,-65.81680533,0\H,7,1.11145213,6,109.3367636,5,66.
54139031,0\H,7,1.09630511,6,108.79334859,5,-176.85052096,0\C,8,1.46200
919,3,111.91234597,2,177.0692096,0\H,18,1.10647973,8,113.07885561,3,62
.7315668,0\H,18,1.09552217,8,109.42856609,3,-58.13713327,0\H,18,1.0955
095,8,109.44315468,3,-176.381727,0\C,5,2.98795022,4,147.54207384,3,177
.76399067,0\C,5,4.86612552,4,87.70243333,3,126.39442478,0\C,23,1.53888
308,5,18.36554636,4,-176.88960968,0\C,24,1.54896894,23,109.83140694,5,
-66.69345861,0\C,25,1.54954348,24,110.31864515,23,-53.05753871,0\C,26,
1.53898144,25,109.69115576,24,53.21142343,0\N,23,1.45775231,5,119.9959
0869,4,119.31821905,0\H,23,1.09633639,5,116.13858171,4,-106.57256957,0
\H,23,1.11159075,5,90.87340153,4,2.96703481,0\H,24,1.09756929,23,109.1
2247522,5,55.44579338,0\H,24,1.0980021,23,108.37165636,5,172.99971486,
0\H,26,1.09762235,25,110.22399861,24,-65.81675985,0\H,26,1.09718779,25
,111.18242589,24,174.02235888,0\H,27,1.09630527,26,108.79322471,25,-17
6.85029078,0\H,27,1.11145101,26,109.33677224,25,66.54157459,0\C,28,1.4
620081,23,111.91237168,5,-170.44306723,0\H,37,1.09550986,28,109.443211
71,23,-176.38194672,0\H,37,1.09552305,28,109.42860448,23,-58.1373688,0
\H,37,1.10647984,28,113.07887873,23,62.73132158,0\P,22,1.82329261,5,35
.67695221,4,4.39349683,0\C,41,1.82329197,22,107.74473848,5,119.3565505
1,0\H,42,1.0948221,41,109.98173053,22,56.04576933,0\H,42,1.09495612,41
,110.05202082,22,-63.6415951,0\H,42,1.09522873,41,110.31545091,22,176.
19598858,0\H,22,1.09495589,5,145.65255247,4,-0.6094149,0\H,22,1.094821
54,5,91.80151279,4,127.24091379,0\\Version=AM64L-G03RevD.01\State=1-A\
HF=-997.1309029\MP2=-999.7491349\RMSD=2.650e-09\Thermal=0.\PG=C01 [X(C
14H30N2P1)]\\@

198

1\1\GINC-NAUTILUS\SP\RMP2-FC\6-31+G(2d,p)\C9H19N2P1\CHRISTOPH\02-Jun-2
010\0\\#p MP2(FC)/6-31+g(2d,p) scf=tight\\mazb2sp_17\\0,1\H\H,1,3.2942
6627\H,1,2.70816968,2,82.87829555\P,2,2.42031513,1,49.26625695,3,50.48
227556,0\C,4,1.86729362,2,88.99116628,1,23.19709115,0\C,4,1.87252916,2
,25.7748443,1,-91.10575635,0\C,6,1.55890872,4,115.39382241,2,-127.6650
8014,0\N,7,1.47790024,6,89.02189288,4,-104.10605651,0\C,8,1.47631371,7
,90.97332652,6,-18.65977847,0\H,7,1.10588387,6,111.63201831,4,141.1938
5852,0\H,7,1.09855477,6,118.52659533,4,13.80763322,0\H,9,1.09884493,8,
114.72414386,7,140.47430899,0\H,9,1.10652999,8,113.25121297,7,-94.2507
1783,0\C,8,1.44853472,7,118.38817385,6,-141.95581672,0\H,14,1.10956548
,8,113.11852117,7,54.25588584,0\H,14,1.0971534,8,109.64668768,7,-66.58
323563,0\H,14,1.09717114,8,109.64898645,7,175.11986564,0\C,4,1.8607695
9,2,128.29602596,1,-77.65259016,0\C,18,1.55526536,4,119.16244257,2,-90
.8736891,0\N,19,1.47920691,18,88.98939645,4,-136.56448075,0\C,20,1.477
2247,19,90.56440766,18,19.90775683,0\H,19,1.0961281,18,117.09787993,4,
105.20668982,0\H,19,1.10658577,18,112.0006518,4,-22.2940891,0\H,21,1.0
983129,20,115.02864936,19,-140.46446412,0\H,21,1.10709439,20,112.99571
281,19,93.59120249,0\C,20,1.44897475,19,118.24067069,18,142.62882523,0
\H,26,1.09749133,20,109.63573623,19,-174.54877673,0\H,26,1.10961197,20
,113.25616551,19,-53.60332773,0\H,26,1.09738692,20,109.56608449,19,67.
27238655,0\H,5,1.09682149,4,109.27922049,2,162.25709757,0\H,5,1.096877
26,4,109.5945306,2,44.65390634,0\\Version=AM64L-G03RevD.01\State=1-A\H
F=-801.4486493\MP2=-803.2985226\RMSD=6.171e-09\Thermal=0.\PG=C01 [X(C9
H19N2P1)]\\@

198-Me$^+$

1\1\GINC-EDDY\SP\RMP2-FC\6-31+G(2d,p)\C10H22N2P1(1+)\CHRISTOPH\02-Jun-2010\0\\#p MP2(FC)/6-31+g(2d,p) scf=tight\\mazb2mesp_1\\1,1\H\H,1,3.46235043\H,2,2.95904628,1,81.03910911\C,3,1.09402266,2,89.1248517,1,-64.0439644,0\P,4,1.8185617,3,110.28173043,2,30.44503794,0\C,5,1.81856206,4,108.7465297,3,178.67951637,0\H,6,1.09402272,5,110.28167474,4,178.68040948,0\H,6,1.09413066,5,110.03323209,4,59.32527645,0\H,6,1.09584957,5,109.28589849,4,-60.8104796,0\H,4,1.09584994,3,109.57194263,2,-89.89237478,0\H,4,1.09413201,3,108.25573858,2,150.87365866,0\C,5,1.82524162,4,110.49988211,3,59.21054901,0\C,12,1.56195977,5,114.90339322,4,-48.30628998,0\N,13,1.47447017,12,88.50411262,5,95.01886803,0\C,14,1.47450176,13,91.01062601,12,20.87969061,0\H,13,1.10367718,12,109.87150594,5,-149.76453395,0\H,13,1.09875495,12,119.50796842,5,-23.34462547,0\H,15,1.10363115,14,114.17755945,13,90.27052726,0\H,15,1.09859484,14,115.26021488,13,-142.99813479,0\C,14,1.45597111,13,118.6398782,12,144.5983838,0\H,20,1.09545867,14,109.33165352,13,-175.41460228,0\H,20,1.10524094,14,112.70913285,13,-54.42248909,0\H,20,1.0954686,14,109.32845848,13,66.56532267,0\C,5,1.82524208,4,108.89305817,3,-60.85342666,0\C,24,1.5619605,5,114.9033522,4,-167.68696649,0\N,25,1.47447075,24,88.5041549,5,95.0190727,0\C,26,1.47450298,25,91.01068169,24,20.87943162,0\H,25,1.10367693,24,109.87118404,5,-149.76458202,0\H,25,1.09875338,24,119.50818087,5,-23.3446128,0\H,27,1.10363175,26,114.17740184,25,90.2704513,0\H,27,1.0985946,26,115.26036546,25,-142.99810307,0\C,26,1.45597195,25,118.63993948,24,144.59825822,0\H,32,1.09545831,26,109.33167989,25,-175.41507129,0\H,32,1.10523992,26,112.70918176,25,-54.42291263,0\H,32,1.09546919,26,109.32839078,25,66.56491476,0\\Version=AM64L-G03RevD.01\State=1-A\HF=-840.9038546\MP2=-842.8988834\RMSD=1.826e-09\Thermal=0.\PG=C01 [X(C10H22N2P1)]\\@

199

1\1\GINC-NAUTILUS\SP\RMP2-FC\6-31+G(2d,p)\C13H27N2P1\CHRISTOPH\13-Jun-2010\0\\#p MP2(FC)/6-31+g(2d,p) scf=tight\\pib2sp_1\\0,1\P\H,1,2.41409356\C,1,1.87100874,2,127.96123644\H,3,1.0974198,1,114.02559134,2,-56.11087864,0\H,3,1.09642564,1,109.38883564,2,65.73111473,0\H,3,1.09599272,1,108.94083438,2,-177.46471291,0\N,1,4.16378612,3,120.06673213,6,-80.95941741,0\C,7,1.46562438,1,19.74951119,3,-165.11088013,0\C,8,1.54140239,7,111.59293996,1,2.47681597,0\C,9,1.54545304,8,109.42925081,7,-56.40121935,0\C,10,1.53797548,9,110.53903353,8,52.98853369,0\C,7,1.46326855,1,100.30124509,3,68.09749843,0\H,8,1.11252547,7,111.41122282,1,-119.80019582,0\H,8,1.09774622,7,107.40848538,1,123.40801149,0\H,9,1.10052999,8,107.89623569,7,61.15768974,0\H,10,1.09791003,9,110.81392166,8,175.13065531,0\H,10,1.10136623,9,109.155618,8,-67.40479064,0\H,11,1.09885123,10,109.68541313,9,66.56456597,0\H,11,1.09861647,10,110.35876256,9,-175.20511092,0\H,12,1.09830991,7,108.16824949,1,-163.56024881,0\H,12,1.11268573,7,111.32993059,1,79.20034846,0\C,7,1.4562207,1,130.87919804,3,-161.4415759,0\H,22,1.10959522,7,113.26978008,1,-63.90830691,0\H,22,1.09703045,7,109.71566216,1,56.85513318,0\H,22,1.09705594,7,109.58915302,1,175.41943142,0\C,1,4.21617603,3,97.86837104,9,-125.69440322,0\C,26,1.53823964,1,20.07469004,3,-64.90503712,0\C,27,1.54546854,26,110.35507315,1,4.92890643,0\C,28,1.53936447,27,109.86493085,26,53.08778555,0\N,29,1.4674497,28,111.02997285,27,-56.84952836,0\C,30,1.46330407,29,111.47595424,28,60.75991673,0\H,26,1.0986345,1,130.54135724,3,-64.50707415,0\H,26,1.09875918,1,99.39024829,3,172.67131285,0\H,27,1.09796871,26,110.66669754,1,-117.4721349,0\H,27,1.10144478,26,108.92386157,1,125.38349488,0\H,29,1.09756582,28,110.4481865,27,-175.98445033,0\H,29,1.11116191,28,109.77970838,27,66.19874003,0\H,31,1.09829723,30,108.16986242,29,179.17230916,0\H,31,1.11308475,30,111.30626609,29,62.02421957,0\C,30,1.45627414,29,111.24761401,28,-174.16622324,0\H,40,1.10982398,30,1

13.30304414,29,-62.76603667,0\H,40,1.09708523,30,109.62782268,29,176.5
6265618,0\H,40,1.09701451,30,109.68989021,29,58.00515109,0\\Version=AM
64L-G03RevD.01\State=1-A\HF=-957.6725741\MP2=-960.1477979\RMSD=4.191e-
09\Thermal=0.\PG=C01 [X(C13H27N2P1)]\\@

199-Me$^+$

1\1\GINC-NAUTILUS\SP\RMP2-FC\6-31+G(2d,p)\C14H30N2P1(1+)\CHRISTOPH\21-
Jun-2010\0\\#p MP2(FC)/6-31+g(2d,p) scf=tight\\pib2mesp_1\\1,1\P\C,1,1
.82271934\H,2,1.09483721,1,110.29884381\H,2,1.0953735,1,109.67535449,3
,-120.12509747,0\H,2,1.09542619,1,108.06008312,4,-121.03208546,0\C,1,3
.53650771,2,75.81448291,3,-55.26635371,0\C,6,1.5386543,1,52.65395348,2
,171.49888245,0\C,7,1.55156557,6,112.46785871,1,36.03183232,0\C,8,1.54
909816,7,109.71180142,6,48.91482792,0\N,9,1.46055548,8,111.32069954,7,
-55.98493773,0\C,10,1.47170698,9,110.61913838,8,62.92700402,0\H,6,1.09
782957,1,67.97846462,2,29.2277567,0\H,6,1.09655009,1,151.29348998,2,11
3.51987615,0\H,7,1.09927469,6,108.94033647,1,153.21376314,0\H,7,1.0972
1511,6,111.15049475,1,-90.26288185,0\H,8,1.10100555,7,108.70468015,6,1
66.40049038,0\H,9,1.10992557,8,106.76096667,7,66.6408266,0\H,9,1.09723
407,8,111.43849082,7,-177.74582491,0\H,11,1.09592598,10,108.18303996,9
,177.73304845,0\H,11,1.1087252,10,110.68780419,9,60.6217515,0\N,1,4.12
036367,2,127.70188597,8,120.55838568,0\C,21,1.45518901,1,22.66412252,2
,172.23527179,0\C,22,1.54975563,21,109.39618238,1,-5.81909004,0\C,23,1
.54981699,22,110.40010236,21,-58.59396404,0\C,24,1.53996279,23,109.162
22677,22,54.1719666,0\C,21,1.46626462,1,101.93882934,2,53.79071825,0\H
,22,1.09787806,21,107.77319937,1,114.49822574,0\H,22,1.11291366,21,112
.32297582,1,-127.55066966,0\H,23,1.10069959,22,107.33803764,21,59.2471
5128,0\H,24,1.09733617,23,111.18789532,22,175.85831045,0\H,24,1.099899
83,23,109.69461534,22,-65.3921997,0\H,25,1.09767897,24,109.5237347,23,
67.58569271,0\H,25,1.09653808,24,109.50341489,23,-174.68025743,0\H,26,
1.1104059,21,111.39093702,1,82.80074455,0\H,26,1.09590145,21,108.03930
823,1,-159.86239653,0\C,1,1.82561291,2,107.00576303,23,118.72348591,0\
H,36,1.09506267,1,110.67753908,2,175.11758064,0\C,21,1.46228659,1,133.
27853411,2,-169.80333766,0\H,38,1.09496563,21,109.15867405,1,168.76431
357,0\H,38,1.09628998,21,109.72124423,1,50.59714004,0\H,38,1.10657413,
21,113.1188495,1,-70.65116427,0\C,10,1.46625062,9,111.03065753,8,-173.
18221352,0\H,42,1.10514209,10,112.7903263,9,-61.96248795,0\H,42,1.0949
5992,10,109.30073829,9,177.50511465,0\H,42,1.09618859,10,109.78477438,
9,59.00254555,0\H,36,1.09518349,1,109.83831114,2,-64.65694655,0\H,36,1
.09494077,1,109.99380013,2,54.4814838,0\\Version=AM64L-G03RevD.01\Stat
e=1-A\HF=-997.1271294\MP2=-999.7490576\RMSD=3.212e-09\Thermal=0.\PG=C0
1 [X(C14H30N2P1)]\\@

200

1\1\GINC-CIPCLU07\SP\RMP2-FC\6-31+G(2d,p)\C9H20N1P1\C2175\25-Jun-2010\
0\\#p MP2(FC)/6-31+G(2d,p) scf=tight\\n7c1sp_2\\0,1\P\C,1,1.86738719\H
,2,1.0960294,1,110.4648528\H,2,1.09708541,1,108.9182998,3,117.70510302
,0\H,2,1.09878911,1,112.69547361,4,120.47825297,0\H,1,2.45529028,2,80.
98747665,3,87.38677741,0\C,1,1.868257,2,98.02023816,3,173.07067192,0\H
,7,1.09901503,1,112.80116849,2,-54.23706941,0\H,7,1.09581886,1,110.427
39457,2,-176.159498,0\H,7,1.09717854,1,108.74837767,2,66.11556203,0\C,
1,1.89042902,2,99.99505783,7,-102.58113187,0\N,11,2.54056544,1,136.695
61309,2,177.07425624,0\C,12,1.46983576,11,91.61563384,1,-169.42953899,
0\C,13,1.53853188,12,111.46824983,11,-70.39552633,0\C,14,1.53817201,13
,115.57276058,12,68.28688542,0\C,15,1.5431636,14,115.5016016,13,-49.06
686064,0\H,13,1.10664396,12,111.98947979,11,54.71103401,0\H,13,1.09761
721,12,108.06220566,11,171.07481557,0\H,14,1.09888001,13,106.91453448,
12,-53.66307999,0\H,14,1.10073385,13,108.93930511,12,-168.93209064,0\H
,15,1.1017187,14,109.38489149,13,73.22718656,0\H,15,1.09980806,14,108.

30838099,13,-171.654652,0\H,16,1.09957585,15,107.61832095,14,-168.7988
8138,0\H,16,1.09775365,15,110.51825859,14,-52.94342587,0\C,12,1.456813
76,11,143.74348038,1,-34.39990221,0\H,25,1.096344,12,109.89546478,11,1
74.94320274,0\H,25,1.09753995,12,109.45018597,11,56.72896277,0\H,25,1.
10988289,12,113.68095376,11,-63.63977573,0\C,12,1.4630883,11,33.486598
73,1,-33.22335094,0\H,29,1.110422,12,111.12358233,11,-123.52830157,0\H
,29,1.10192229,12,106.31908326,11,121.06762276,0\\Version=AM64L-G03Rev
D.01\State=1-A\HF=-747.6570322\MP2=-749.3500724\RMSD=6.316e-09\Thermal
=0.\PG=C01 [X(C9H20N1P1)]\\@

200-Me$^+$

1\1\GINC-CIPCLU06\SP\RMP2-FC\6-31+G(2d,p)\C10H23N1P1(1+)\C2175\25-Jun-
2010\0\\#p MP2(FC)/6-31+G(2d,p) scf=tight\\n7c1mesp_4\\1,1\C\H,1,1.093
12175\H,1,1.09652405,2,110.36703023\H,1,1.09450871,2,109.59890385,3,-1
19.6961805,0\H,1,3.93733327,2,100.28380571,4,-100.15303652,0\C,1,2.938
19717,2,144.90281447,4,-121.56157881,0\H,6,1.0957492,1,87.17701185,2,-
129.44234252,0\H,6,1.09544306,1,93.07004135,2,122.01149946,0\H,6,1.095
71255,1,146.056064,2,-9.05169818,0\C,6,2.95839827,1,63.3565672,2,34.30
747023,0\C,10,3.19257747,6,121.77811068,1,37.16908968,0\C,11,1.539688,
10,55.28712738,6,101.17044698,0\C,12,1.54023897,11,115.13621152,10,-27
.25268132,0\H,11,1.09950793,10,87.12524912,6,-15.87709754,0\H,11,1.097
93125,10,162.53689501,6,122.82121968,0\H,12,1.09871576,11,109.81261071
,10,97.78341772,0\H,12,1.09758808,11,109.03335451,10,-146.85848116,0\H
,13,1.0964225,12,108.69545285,11,179.9777782,0\H,13,1.10003488,12,107.
68555497,11,-66.48554199,0\C,10,1.54879749,6,143.50929356,1,-64.943175
96,0\H,20,1.10472191,10,108.47855459,6,-160.57267604,0\H,20,1.09657248
,10,107.77275524,6,-46.49167213,0\N,20,1.46794991,10,113.63343389,6,73
.27547869,0\P,1,1.81858855,6,36.26936113,10,33.43627765,0\C,24,1.82011
035,1,108.13438042,6,115.60690285,0\H,25,1.09395651,24,109.91143329,1,
57.62751336,0\H,25,1.09568684,24,110.4482941,1,178.50462075,0\H,25,1.0
960079,24,109.34874155,1,-61.92180667,0\C,23,1.47299736,20,110.8002346
5,10,-150.67586682,0\H,29,1.09636918,23,110.22148003,20,57.52944608,0\
H,29,1.10418215,23,112.86589329,20,-63.46822155,0\H,29,1.09482667,23,1
09.29258692,20,176.14792414,0\C,23,1.47767859,20,112.64535559,10,87.18
528469,0\H,33,1.09667975,23,106.92869774,20,175.50123673,0\H,33,1.1079
3595,23,109.80651497,20,60.13560578,0\\Version=AM64L-G03RevD.01\State=
1-A\HF=-787.109939\MP2=-788.9509259\RMSD=2.992e-09\Thermal=0.\PG=C01 [
X(C10H23N1P1)]\\@

201

1\1\GINC-EDDY\SP\RMP2-FC\6-31+G(2d,p)\C7H12N1P1\CHRISTOPH\12-Oct-2009\
0\\#p MP2(FC)/6-31+g(2d,p) scf=tight\\z1sp_2\\0,1\P\C,1,1.82378492\C,2
,1.43728118,1,131.02598657\C,3,1.37733251,2,107.63200552,1,179.1193240
2,0\N,4,1.37967896,3,108.43119093,2,-0.25908602,0\C,5,1.3717316,4,108.
7341965,3,0.51542004,0\C,1,1.86860451,2,101.10385823,6,125.86310611,0\
C,1,1.86805964,2,101.04319477,6,-132.72785909,0\H,6,1.08272119,5,120.8
5617842,4,179.57694641,0\H,4,1.08268143,3,131.13610646,2,179.99892923,
0\H,3,1.08317616,2,126.80150469,1,-0.00647547,0\C,5,1.45179658,4,125.3
9701823,3,175.24786747,0\H,12,1.09472483,5,109.6303381,4,36.54737851,0
\H,12,1.09419492,5,109.4095313,4,155.33366242,0\H,12,1.09788649,5,111.
5977325,4,-84.15201284,0\H,8,1.09742926,1,109.20692066,2,-171.60653433
,0\H,8,1.09788092,1,113.01901844,2,-50.61129293,0\H,8,1.09606571,1,109
.370846,2,70.44562852,0\H,7,1.09752008,1,109.32280926,2,171.59687652,0
\H,7,1.09606853,1,109.30457811,2,-70.42352231,0\H,7,1.09774694,1,112.8
6737895,2,50.46310357,0\\Version=AM64L-G03RevD.01\State=1-A\HF=-667.25
55061\MP2=-668.5975187\RMSD=8.906e-09\Thermal=0.\PG=C01 [X(C7H12N1P1)]
\\@

201-Me+

1\1\GINC-SOLARIS\SP\RMP2-FC\6-31+G(2d,p)\C8H15N1P1(1+)\CHRISTOPH\12-Oc
t-2009\0\\#p MP2(FC)/6-31+g(2d,p) scf=tight\\z1mesp_2\\1,1\P\C,1,1.820
77462\H,2,1.09597072,1,110.38587633\H,2,1.09554077,1,109.79369313,3,-1
20.16592758,0\H,2,1.09513302,1,109.54461882,4,-119.2060135,0\C,1,1.821
00213,2,107.75650229,5,177.55011941,0\C,1,1.7634632,2,110.92964698,6,1
21.90669264,0\C,7,1.4378741,1,126.87324401,2,-58.906222,0\C,8,1.374015
75,7,106.56101624,1,-179.52275607,0\N,9,1.37957138,8,108.85276741,7,0.
07301353,0\C,10,1.35754195,9,109.55625419,8,-0.11398021,0\C,1,1.818403
59,7,110.17421083,11,1.69742483,0\H,11,1.0816667,10,120.96087734,9,-17
9.86856254,0\H,9,1.08153351,8,130.45058664,7,-179.98684043,0\H,8,1.082
29945,7,127.64535475,1,0.2011408,0\C,10,1.46306433,9,124.79278114,8,-1
78.44825861,0\H,16,1.09203056,10,109.09198992,9,-169.87786534,0\H,16,1
.09466005,10,110.34278208,9,70.07388408,0\H,16,1.09351916,10,109.66624
855,9,-50.66720054,0\H,12,1.0951353,1,110.30002749,7,-60.61090973,0\H,
12,1.09557416,1,109.34030124,7,179.85370523,0\H,12,1.09510148,1,110.22
034784,7,60.30149767,0\H,6,1.09514764,1,109.53880804,7,60.67296084,0\H
,6,1.0959941,1,110.41852835,7,-178.69160185,0\H,6,1.09554718,1,109.759
73779,7,-58.48940843,0\\Version=AM64L-G03RevD.01\State=1-A\HF=-706.712
8879\MP2=-708.1992989\RMSD=7.842e-09\Thermal=0.\PG=C01 [X(C8H15N1P1)]\
\@

202

1\1\GINC-CALYPSO\SP\RMP2-FC\6-31+G(2d,p)\C11H23N2P1\CHRISTOPH\22-Dec-2
009\0\\#p MP2(FC)/6-31+g(2d,p) scf=tight\\b2sp_1\\0,1\P\C,1,1.86909777
\H,2,1.09885078,1,112.64417528\C,1,1.87503765,2,98.27506854,3,-53.3879
8065,0\C,4,1.56537151,1,112.407777,2,-68.74892339,0\C,5,1.54890399,4,1
04.69300671,1,-133.6330166,0\N,6,1.46336109,5,104.43868476,4,-17.25134
102,0\C,7,1.46206516,6,104.80107477,5,39.48020161,0\H,4,1.09908781,1,1
09.30710655,2,52.24329251,0\H,5,1.09753163,4,112.24814065,1,-13.873280
18,0\H,5,1.0950979,4,110.44151747,1,105.9458226,0\H,6,1.09756426,5,113
.24146853,4,-137.42504938,0\H,6,1.1099036,5,109.92621724,4,102.3546188
7,0\H,8,1.11188709,7,111.7178933,6,72.92846201,0\H,8,1.09716809,7,110.
49364513,6,-167.08515834,0\C,1,1.87667446,2,99.24270927,4,102.68277774
,0\C,16,1.56646286,1,113.79753425,2,178.52029091,0\C,17,1.54517176,16,
104.54762648,1,-125.54895821,0\N,18,1.46226858,17,103.97645965,16,-22.
85292315,0\C,19,1.46117222,18,104.58440146,17,42.30371604,0\H,16,1.098
94091,1,109.30387907,2,-59.03000349,0\H,17,1.09641513,16,112.65347323,
1,-5.13525835,0\H,17,1.09542075,16,110.18868941,1,115.0587893,0\H,18,1
.09747014,17,113.28384947,16,-142.90703195,0\H,18,1.11030705,17,110.02
073871,16,96.65180849,0\H,20,1.09715128,19,110.62279081,18,-165.948471
6,0\H,20,1.11293619,19,111.49264857,18,74.30649311,0\C,19,1.45173483,1
8,114.11295869,17,167.69297588,0\H,28,1.09697778,19,109.78078745,18,17
9.21492724,0\H,28,1.10994672,19,113.02074561,18,-59.95708066,0\H,28,1.
09693504,19,109.69698746,18,60.73510775,0\C,7,1.45147383,6,113.9479049
8,5,165.11131846,0\H,32,1.09706009,7,109.71117028,6,60.84259335,0\H,32
,1.09693065,7,109.79929523,6,179.32504907,0\H,32,1.11002832,7,113.0323
1498,6,-59.80077954,0\H,2,1.09651916,1,109.52318111,4,67.50483159,0\H,
2,1.0963615,1,109.80660154,4,-174.67754917,0\\Version=AM64L-G03RevD.01
\State=1-A\HF=-879.5867594\MP2=-881.7480769\RMSD=4.535e-09\Thermal=0.\
PG=C01 [X(C11H23N2P1)]\\@

202-Me+

1\1\GINC-MAX\SP\RMP2-FC\6-31+G(2d,p)\C12H26N2P1(1+)\CHRISTOPH\18-Dec-2
009\0\\#p MP2(FC)/6-31+g(2d,p) scf=tight\\b2mesp_3\\1,1\P\C,1,1.820512
95\H,2,1.09460751,1,109.251678\H,2,1.09477367,1,109.79854026,3,-118.22
049417,0\H,2,1.09473369,1,110.34433918,3,121.88781111,0\C,1,1.8207689,
2,109.11315978,3,167.81500715,0\H,6,1.09440492,1,109.49809655,2,70.355

1321,0\H,6,1.09479891,1,110.10550601,2,-171.17154557,0\H,6,1.09512248,
1,110.06208943,2,-50.90227696,0\C,1,1.83933652,2,109.43401217,6,119.53
614709,0\C,10,1.55713403,1,112.46094395,2,-64.14989848,0\N,11,1.456338
94,10,103.03814274,1,-93.83042902,0\C,12,1.46500212,11,104.3917756,10,
-45.65849324,0\C,13,1.54565114,12,103.65003806,11,44.72225028,0\H,10,1
.09650426,1,105.98256743,2,56.72348072,0\H,11,1.10839558,10,108.241467
2,1,146.2803654,0\H,11,1.09817475,10,115.03252025,1,26.77765997,0\H,13
,1.10690572,12,111.70291575,11,-73.70824418,0\H,13,1.09524702,12,110.6
7538632,11,166.01831916,0\H,14,1.09378536,13,112.28550292,12,-145.1174
831,0\H,14,1.09622607,13,110.2775989,12,95.58370644,0\C,1,1.84052444,2
,109.47923778,6,-119.2906616,0\C,22,1.57044473,1,112.31133973,2,56.934
5919,0\C,23,1.54784012,22,104.03020892,1,-116.50311907,0\N,24,1.466419
62,23,104.04240081,22,21.92853796,0\C,25,1.45701157,24,104.43497419,23
,-43.05811685,0\H,22,1.09662284,1,105.83765407,2,178.93647547,0\H,23,1
.09368354,22,110.59682561,1,123.08346239,0\H,23,1.09618971,22,112.6171
4231,1,3.63337806,0\H,24,1.09529606,23,112.93902836,22,141.87873029,0\
H,24,1.10649887,23,109.9362627,22,-97.60921807,0\H,26,1.1085017,25,112
.98894921,24,-70.23118985,0\H,26,1.09791,25,110.84107855,24,169.654827
2,0\C,12,1.46041674,11,114.05816885,10,-170.97055677,0\H,34,1.09495439
,12,109.35122089,11,-179.0203174,0\H,34,1.09580321,12,109.72357716,11,
-60.66452092,0\H,34,1.10566369,12,112.65124873,11,60.30420023,0\C,25,1
.46055717,24,114.04378963,23,-168.24256605,0\H,38,1.09581981,25,109.73
831811,24,-179.23083177,0\H,38,1.09496547,25,109.37724754,24,-60.82740
225,0\H,38,1.10560765,25,112.62545737,24,59.80877977,0\\Version=IA32L-
G03RevD.01\State=1-A\HF=-919.0422099\MP2=-921.351977\RMSD=2.554e-09\Th
ermal=0.\PG=C01 [X(C12H26N2P1)]\\@

203

1\1\GINC-SOLARIS\SP\RMP2-FC\6-31+G(2d,p)\C10H18N1P1\CHRISTOPH\04-Jan-2
010\0\\#p MP2(FC)/6-31+g(2d,p) scf=tight\\zz1sp_7\\0,1\P\C,1,1.8687908
8\C,1,1.86699444,2,99.78908748\C,1,1.82525116,3,103.80829048,2,-106.62
434009,0\C,4,1.39593022,1,120.6491918,3,-134.47062392,0\N,5,1.37797248
,4,108.15128858,1,-179.98091236,0\C,6,1.3899764,5,109.64399646,4,0.317
10917,0\C,7,1.38258742,6,108.12926315,5,-0.36763242,0\C,8,1.50640428,7
,125.16727226,6,-178.34572685,0\H,9,1.09628332,8,111.35300541,7,-14.08
062803,0\H,9,1.09661195,8,111.96697114,7,-133.80716992,0\H,9,1.0992002
6,8,112.1146648,7,105.78906857,0\C,7,1.49989948,6,120.85138264,5,-179.
95055255,0\H,13,1.10070226,7,112.46700203,6,64.40063729,0\H,13,1.09993
765,7,112.2725488,6,-56.54428669,0\H,13,1.0940145,7,110.24197684,6,-17
6.14987186,0\C,5,1.50124981,4,130.14856026,1,-0.19539908,0\H,17,1.0937
0418,5,109.10085086,4,1.70088963,0\H,17,1.09992153,5,112.21041125,4,12
0.97724543,0\H,17,1.09981129,5,112.33115242,4,-117.83286322,0\C,6,1.45
148128,5,126.03924344,4,177.0389952,0\H,21,1.09570072,6,110.43777517,5
,135.15225155,0\H,21,1.09182792,6,109.56626413,5,16.00280642,0\H,21,1.
09887787,6,111.56490704,5,-104.11304695,0\H,2,1.09613233,1,108.9992081
6,4,-66.41239994,0\H,2,1.09775382,1,108.72892303,4,176.29993972,0\H,2,
1.09656457,1,114.1196471,4,55.11022814,0\H,3,1.09614972,1,114.13716549
,4,-58.63929663,0\H,3,1.09629118,1,109.11913376,4,63.23369611,0\H,3,1.
09755348,1,108.49103275,4,-179.61485188,0\\Version=AM64L-G03RevD.01\St
ate=1-A\HF=-784.3742123\MP2=-786.1879101\RMSD=5.252e-09\Thermal=0.\PG=
C01 [X(C10H18N1P1)]\\@

203-Me^{+}

1\1\GINC-CALYPSO\SP\RMP2-FC\6-31+G(2d,p)\C11H21N1P1(1+)\CHRISTOPH\04-J
an-2010\0\\#p MP2(FC)/6-31+g(2d,p) scf=tight\\zz1mesp_1\\1,1\C\C,1,2.9
884333\C,2,1.45364359,1,137.12231074\C,3,1.38045576,2,106.10806262,1,1
33.41287902,0\N,4,1.39129415,3,108.52341763,2,-0.00097947,0\C,5,1.3625
3625,4,110.66100849,3,0.00218451,0\C,1,2.95423724,2,60.3790631,6,-97.4

8827797,0\C,3,1.50625029,2,128.96906784,1,-46.59154021,0\H,8,1.0933284
7,3,110.46725765,2,-179.97490611,0\H,8,1.09749731,3,112.49897544,2,-61
.05927203,0\H,8,1.0975008,3,112.50071121,2,61.10665008,0\C,4,1.4993347
9,3,130.93256721,2,-179.99849964,0\H,12,1.09248789,4,110.67355229,3,-0
.01580209,0\H,12,1.09838546,4,111.87816622,3,119.59495822,0\H,12,1.098
38267,4,111.87892666,3,-119.6282362,0\C,5,1.46139188,4,123.23964506,3,
179.99271051,0\H,16,1.09007799,5,109.94549969,4,179.95983989,0\H,16,1.
09481644,5,110.19907587,4,60.15197781,0\H,16,1.09482321,5,110.20314973
,4,-60.22508855,0\C,6,1.50280811,5,121.5773184,4,179.9899009,0\H,20,1.
09795336,6,111.80087385,5,60.43790119,0\H,20,1.09794811,6,111.79724598
,5,-60.37442354,0\H,20,1.09254388,6,111.47738997,5,-179.9641175,0\P,2,
1.77090607,1,34.44108686,7,33.98808121,0\C,24,1.81972579,2,112.9170272
2,1,119.07084413,0\H,25,1.09333163,24,110.98558976,2,-60.86972698,0\H,
25,1.09332426,24,110.99260642,2,60.88108712,0\H,25,1.09614247,24,107.8
4262068,2,-179.99259402,0\H,7,1.09627007,1,88.10630162,24,-128.7578676
1,0\H,7,1.09547434,1,144.84700204,24,-7.00520797,0\H,7,1.09403391,1,93
.34393601,24,122.44491481,0\H,1,1.09402868,24,111.24735808,2,-59.66412
695,0\H,1,1.09627147,24,109.96507404,2,179.64308089,0\H,1,1.09547396,2
4,108.95054496,2,59.92189748,0\\Version=AM64L-G03RevD.01\State=1-A\HF=
-823.8329357\MP2=-825.7928075\RMSD=8.283e-09\Thermal=0.\PG=C01 [X(C11H
21N1P1)]\\@

204

1\1\GINC-EDDY\SP\RMP2-FC\6-31+G(2d,p)\C11H23N2P1\CHRISTOPH\09-Dec-2009
\0\\#p MP2(FC)/6-31+g(2d,p) scf=tight\\a2sp_17\\0,1\P\C,1,1.86609795\H
,2,1.09632397,1,109.80552795\H,2,1.09616184,1,107.65553234,3,117.22499
363,0\H,2,1.09872992,1,114.19828503,4,120.92142969,0\C,1,1.89250109,2,
100.27820268,4,-57.83118498,0\N,6,1.48204631,1,107.3370642,2,74.850525
33,0\C,7,1.46154467,6,107.66114402,1,-149.72806536,0\C,8,1.53098108,7,
102.95826643,6,39.87958482,0\C,9,1.54592667,8,102.21610495,7,-40.54231
718,0\H,6,1.10567733,1,105.55864512,2,-166.90515471,0\H,8,1.09797264,7
,110.32231468,6,161.52584432,0\H,8,1.11112443,7,112.12791333,6,-78.521
24957,0\H,9,1.09673308,8,109.78179826,7,76.99523262,0\H,9,1.0957841,8,
112.99518777,7,-162.09615312,0\H,10,1.0975437,9,109.88641749,8,-90.733
42445,0\H,10,1.09535354,9,112.19436755,8,150.55122659,0\C,1,1.89295306
,2,100.67181234,6,-104.83810138,0\C,18,1.55364975,1,109.8920634,2,-77.
30362028,0\C,19,1.53958,18,103.27538586,1,-96.18557974,0\C,20,1.530898
71,19,101.62299354,18,-37.42137038,0\N,21,1.46944152,20,104.07772647,1
9,39.73799824,0\H,18,1.10376619,1,110.50453629,2,44.7975533,0\H,19,1.0
9856522,18,109.88420353,1,146.56923652,0\H,19,1.09656349,18,113.254382
12,1,26.32756486,0\H,20,1.09681185,19,110.32177828,18,79.47988155,0\H,
20,1.09571032,19,113.18312834,18,-158.69153941,0\H,21,1.09825125,20,11
3.52756437,19,159.81503047,0\H,21,1.10945376,20,109.80861944,19,-79.81
444872,0\C,7,1.45297614,6,114.39498418,1,82.26741082,0\H,30,1.09536798
,7,109.7559667,6,-57.27933226,0\H,30,1.0970638,7,109.59354069,6,-175.9
6756617,0\H,30,1.10980706,7,112.6762394,6,63.49651648,0\C,22,1.4522682
5,21,113.20621498,20,-157.02149944,0\H,34,1.09738544,22,109.39474807,2
1,-57.23675484,0\H,34,1.10994647,22,113.18795336,21,63.005982,0\H,34,1
.09514396,22,110.56429164,21,-175.57941037,0\\Version=AM64L-G03RevD.01
\State=1-A\HF=-879.579125\MP2=-881.745865\RMSD=5.933e-09\Thermal=0.\PG
=C01 [X(C11H23N2P1)]\\@

204-Me$^+$

1\1\GINC-NODE24\SP\RMP2-FC\6-31+G(2d,p)\C12H26N2P1(1+)\ZIP07\15-Dec-20
09\0\\#p MP2(FC)/6-31+g(2d,p) scf=tight\\a2mesp_173\\1,1\C\C,1,1.55721
105\C,2,1.54250454,1,103.81146844\C,3,1.53049536,2,102.36310677,1,-32.
58469261,0\N,1,1.46707515,2,106.55658877,3,16.60858019,0\H,1,1.1002354
4,5,112.63113547,4,-115.89266788,0\H,2,1.09624395,1,108.26717995,5,-10

0.44447235,0\H,2,1.09676936,1,114.32822257,5,140.53022277,0\H,3,1.0939
6013,2,112.19104015,1,-153.37153299,0\H,3,1.09607911,2,111.34889072,1,
85.85238692,0\H,4,1.09562989,3,113.53677657,2,157.20449407,0\H,4,1.104
38236,3,110.16962669,2,-81.6719818,0\C,1,3.00644249,5,90.47801305,4,97
.11936581,0\H,13,1.09533147,1,145.66107591,5,109.48978443,0\C,13,3.024
20365,1,61.87286748,5,155.27779229,0\N,15,1.4695768,13,142.34032793,1,
-29.13645971,0\C,16,1.47981191,15,108.51161791,13,-132.89122913,0\C,17
,1.52996526,16,104.41935624,15,29.49758784,0\C,18,1.54226711,17,102.38
171034,16,-38.05307101,0\H,15,1.10297097,13,85.68134127,1,93.36666444,
0\H,17,1.10459368,16,110.80680781,15,-89.18252475,0\H,17,1.09553692,16
,109.88132759,15,151.61097051,0\H,18,1.09622725,17,110.66917502,16,80.
74516921,0\H,18,1.09397684,17,112.51944615,16,-158.63658807,0\H,19,1.0
9642751,18,113.29921274,17,156.77932231,0\H,19,1.09580711,18,110.29985
065,17,-83.5244081,0\C,5,1.46409695,1,115.24533261,2,134.89746285,0\H,
27,1.09470287,5,110.89106659,1,57.49322924,0\H,27,1.09489617,5,109.019
22632,1,175.96963708,0\H,27,1.10469747,5,112.42520405,1,-63.92866734,0
\C,16,1.46284066,15,115.25338385,13,99.30002224,0\H,31,1.10495849,16,1
12.50334051,15,62.14469618,0\H,31,1.09483819,16,108.97820018,15,-177.7
3755349,0\H,31,1.09539414,16,110.9808056,15,-59.39343444,0\P,13,1.8224
8373,1,36.11917506,5,121.79730763,0\C,35,1.81805063,13,107.53086811,1,
-118.17668043,0\H,36,1.09519457,35,110.02069319,13,-57.12352434,0\H,36
,1.09443486,35,108.87325943,13,62.20250663,0\H,36,1.09440993,35,109.70
098963,13,-177.46778416,0\H,13,1.09504986,1,94.11570638,5,-117.9839784
1,0\H,13,1.09485533,1,85.88465163,5,-8.80118847,0\\Version=AM64L-G03Re
vD.01\State=1-A\HF=-919.0397585\MP2=-921.3509501\RMSD=3.875e-09\Therma
l=0.\PG=C01 [X(C12H26N2P1)]\\@

205

1\1\GINC-YANG\SP\RMP2-FC\6-31+G(2d,p)\C9H16N1P1\CHRISTOPH\20-Jan-2010\
0\\#p MP2(FC)/6-31+g(2d,p) scf=tight\\xx1sp_3\\0,1\P\C,1,1.82277086\C,
2,1.39271866,1,124.33361216\N,3,1.38309897,2,108.02478962,1,179.344620
79,0\C,4,1.38983138,3,109.66624499,2,0.65081833,0\C,5,1.37655479,4,107
.38439573,3,-0.62520697,0\C,1,1.86824554,2,101.16948006,3,-128.4595709
3,0\C,1,1.8680719,2,101.10832532,3,129.98574598,0\H,6,1.08386683,5,124
.88859874,4,-179.24991449,0\C,3,1.49999792,2,130.12770771,1,-0.6181127
7,0\H,10,1.09977884,3,112.33827356,2,-120.26233912,0\H,10,1.09418204,3
,109.03690438,2,-0.70511474,0\H,10,1.10022144,3,112.3540964,2,118.5854
9661,0\C,4,1.45188234,3,125.24382192,2,176.24204517,0\H,14,1.09916241,
4,111.78977009,3,-87.83338881,0\H,14,1.09339814,4,109.88526457,3,32.75
735586,0\H,14,1.09377634,4,109.83016498,3,151.62294152,0\C,5,1.4991632
1,4,122.55842229,3,179.53312015,0\H,18,1.1003545,5,112.66721221,4,61.3
9256883,0\H,18,1.09485281,5,109.17155666,4,-179.46287164,0\H,18,1.0999
2544,5,112.63945637,4,-60.14067779,0\H,7,1.09752198,1,109.35844608,2,-
172.10383547,0\H,7,1.09774782,1,112.89552357,2,-50.92483436,0\H,7,1.09
611571,1,109.27563264,2,69.90897226,0\H,8,1.09610926,1,109.28734193,2,
-69.95595649,0\H,8,1.09775527,1,112.92640384,2,50.90363968,0\H,8,1.097
49862,1,109.33816273,2,172.05973509,0\\Version=AM64L-G03RevD.01\State=
1-A\HF=-745.3394831\MP2=-746.9946859\RMSD=6.769e-09\Thermal=0.\PG=C01
[X(C9H16N1P1)]\\@

205-Me⁺

1\1\GINC-EDDY\SP\RMP2-FC\6-31+G(2d,p)\C10H19N1P1(1+)\CHRISTOPH\20-Jan-
2010\0\\#p MP2(FC)/6-31+g(2d,p) scf=tight\\xx1mesp_2\\1,1\C\C,1,2.9139
2074\C,2,1.40271195,1,164.04211548\N,3,1.36829674,2,106.92905828,1,178
.81017554,0\C,4,1.39141431,3,110.74895286,2,0.17358927,0\C,5,1.3743916
,4,107.46658708,3,-0.17817059,0\C,1,2.9325103,2,61.46363338,3,36.11272
596,0\H,6,1.08181642,5,125.11638482,4,-179.84949372,0\C,5,1.49871547,4
,123.09778706,3,179.82257164,0\H,9,1.09392042,5,109.19582616,4,-179.67

107621,0\H,9,1.09783507,5,112.20906542,4,-60.52289214,0\H,9,1.09804115
,5,112.21630704,4,61.26511209,0\C,4,1.46195646,3,123.91598241,2,179.10
840383,0\H,13,1.09024247,4,109.2321997,3,172.89847613,0\H,13,1.0957070
7,4,110.69611167,3,-67.36945511,0\H,13,1.09442575,4,110.32713527,3,53.
66509754,0\C,3,1.49997991,2,131.70789179,1,-1.13396655,0\H,17,1.094212
67,3,111.37560476,2,-2.62406605,0\H,17,1.09859467,3,111.79813946,2,117
.30844545,0\H,17,1.0981229,3,111.67638313,2,-122.41593286,0\P,2,1.7637
5796,1,36.20088829,7,34.88646897,0\C,21,1.82395994,2,112.68757136,1,11
8.87789082,0\H,22,1.09489354,21,110.54901665,2,61.05937349,0\H,22,1.09
61439,21,110.19646462,2,-178.52582101,0\H,22,1.09527908,21,109.2048101
,2,-58.37716977,0\H,7,1.09486383,1,146.90454539,21,-1.53024435,0\H,7,1
.09615858,1,89.36896709,21,-126.67443426,0\H,7,1.09526607,1,89.8631778
8,21,123.95811134,0\H,1,1.09549604,21,109.30852499,2,179.98352261,0\H,
1,1.09494248,21,110.19947208,2,-60.31675037,0\H,1,1.09492894,21,110.19
676864,2,60.27229977,0\\Version=AM64L-G03RevD.01\State=1-A\HF=-784.798
9165\MP2=-786.5995203\RMSD=3.802e-09\Thermal=0.\PG=C01 [X(C10H19N1P1)]
\\@

206

1\1\GINC-NAUTILUS\SP\RMP2-FC\6-31+G(2d,p)\C12H24N3P1\CHRISTOPH\23-Jun-
2010\0\\#p MP2(FC)/6-31+g(2d,p) scf=tight\\mazb3sp_15\\0,1\H\H,1,3.324
03674\H,2,2.65127006,1,83.3843708\P,1,2.42111199,2,48.31923178,3,-51.1
9837108,0\C,4,1.87372061,1,25.76792093,2,90.20408273,0\C,5,1.55948121,
4,115.43896942,1,127.5312339,0\N,6,1.47768416,5,89.02247592,4,104.2746
9674,0\C,7,1.47614829,6,90.99047443,5,18.64336594,0\H,6,1.10597209,5,1
11.52729684,4,-140.98830772,0\H,6,1.09845752,5,118.6031857,4,-13.70636
373,0\H,8,1.10652038,7,113.24007912,6,94.16996082,0\H,8,1.09852165,7,1
14.74114922,6,-140.54141744,0\C,7,1.44873771,6,118.35250606,5,141.9226
9528,0\H,13,1.10944889,7,113.10055043,6,-54.29379026,0\H,13,1.09711909
,7,109.6464224,6,66.55891262,0\H,13,1.09711569,7,109.63925008,6,-175.1
4571914,0\C,4,1.86436787,1,88.6119508,5,-114.11060942,0\C,17,1.5576148
1,4,117.75742494,1,-48.65773851,0\N,18,1.47745353,17,88.99820908,4,-13
8.47454068,0\C,19,1.47804721,18,90.57853549,17,19.88889824,0\H,18,1.09
815852,17,117.99705766,4,103.55182032,0\H,18,1.10688756,17,111.8395472
5,4,-24.00733904,0\H,20,1.09803771,19,114.93993826,18,-140.47200819,0\
H,20,1.10606037,19,113.02475156,18,93.49331122,0\C,19,1.44951188,18,11
8.20476287,17,142.73745306,0\H,25,1.10922173,19,113.23641623,18,-53.70
688115,0\H,25,1.09735219,19,109.57917239,18,67.222922,0\H,25,1.0972759
3,19,109.56785968,18,-174.62925798,0\C,4,1.86212235,1,128.22898027,5,-
12.90048165,0\C,29,1.55538146,4,118.96460965,1,88.9764889,0\N,30,1.479
50507,29,88.98876731,4,137.0191994,0\C,31,1.47743208,30,90.48807183,29
,-20.084732,0\H,30,1.09596074,29,117.07287436,4,-104.67049249,0\H,30,1
.10673238,29,111.98136999,4,22.83013315,0\H,32,1.09830799,31,115.05991
701,30,140.66183734,0\H,32,1.10677707,31,112.92844983,30,-93.38749046,
0\C,31,1.4491349,30,118.1727681,29,-142.69465515,0\H,37,1.09735755,31,
109.56171568,30,-67.30402684,0\H,37,1.10958794,31,113.21971517,30,53.5
5362871,0\H,37,1.09741869,31,109.64781335,30,174.50559744,0\\Version=A
M64L-G03RevD.01\State=1-A\HF=-972.3786219\MP2=-974.8527832\RMSD=6.851e
-09\Thermal=0.\PG=C01 [X(C12H24N3P1)]\\@

206-Me$^+$

1\1\GINC-GOLEM\SP\RMP2-FC\6-31+G(2d,p)\C13H27N3P1(1+)\CHRISTOPH\07-Jun
-2010\0\\#p MP2(FC)/6-31+g(2d,p) scf=tight\\mazb3mesp_1\\1,1\H\H,1,4.1
5370163\H,2,4.14300499,1,60.00097674\P,3,2.39856312,2,30.28070571,1,-6
.36006792,0\C,4,1.82548746,3,93.04095937,2,-95.70694728,0\H,5,1.095252
23,4,110.17909515,3,-158.64277801,0\H,5,1.09515175,4,110.08702574,3,81
.32720605,0\H,5,1.09523826,4,110.10943046,3,-38.61962444,0\C,4,1.82878
115,3,96.41825969,2,157.28735139,0\C,9,1.55692258,4,117.26277014,3,-70

.88165588,0\N,10,1.47607326,9,88.57188112,4,92.94860638,0\C,11,1.47467
329,10,90.56219152,9,21.8790887,0\H,10,1.10373343,9,109.19155181,4,-15
2.18418424,0\H,10,1.09455424,9,120.01971319,4,-26.06414969,0\H,12,1.10
42296,11,114.07584227,10,89.15665128,0\H,12,1.09899466,11,115.34828302
,10,-144.34016137,0\C,11,1.45588757,10,118.53404427,9,145.04953144,0\H
,17,1.09557309,11,109.44027152,10,-175.0068063,0\H,17,1.10546353,11,11
2.66712574,10,-54.00980759,0\H,17,1.09553288,11,109.33363862,10,66.876
11479,0\C,4,1.82918767,3,137.92248461,2,23.34154387,0\C,21,1.55703825,
4,117.30389186,3,80.41648501,0\N,22,1.47593014,21,88.55072483,4,92.832
98022,0\C,23,1.47459035,22,90.53356628,21,21.96158388,0\H,22,1.1037976
2,21,109.21838596,4,-152.29721487,0\H,22,1.09459764,21,120.01035115,4,
-26.15277546,0\H,24,1.10415365,23,114.06919594,22,89.06474026,0\H,24,1
.09882009,23,115.30999616,22,-144.46940452,0\C,23,1.45578543,22,118.54
4802,21,145.07962777,0\H,29,1.09556752,23,109.42024905,22,-174.9204348
4,0\H,29,1.10544318,23,112.67810015,22,-53.93113981,0\H,29,1.09550104,
23,109.33930083,22,66.96535277,0\C,4,1.82859385,3,25.7570223,2,27.2868
4876,0\C,33,1.55717745,4,117.40369481,3,133.076382,0\N,34,1.47601041,3
3,88.53666017,4,92.69060306,0\C,35,1.47459555,34,90.54825879,33,21.958
02098,0\H,34,1.10377201,33,109.18832785,4,-152.44876866,0\H,34,1.09459
388,33,120.03212222,4,-26.33945319,0\H,36,1.10430799,35,114.12641608,3
4,89.09633966,0\H,36,1.0989374,35,115.32683134,34,-144.3752917,0\C,35,
1.45579996,34,118.53442123,33,145.06492609,0\H,41,1.09554628,35,109.43
098638,34,-174.90748256,0\H,41,1.10545472,35,112.65283305,34,-53.92782
718,0\H,41,1.09552488,35,109.34630406,34,66.96443209,0\\Version=AM64L-
G03RevD.01\State=1-A\HF=-1011.8330217\MP2=-1014.4590263\RMSD=3.064e-09
\Thermal=0.\PG=C01 [X(C13H27N3P1)]\\@

207

1\1\GINC-GOLEM\SP\RMP2-FC\6-31+G(2d,p)\C9H13N4P1\CHRISTOPH\17-Feb-2012
\0\\#p MP2(FC)/6-31+g(2d,p) scf=tight\\imi42sp_11\\0,1\P\C,1,1.8697708
5\H,2,1.09684474,1,109.46790899\H,2,1.09544602,1,111.62541031,3,121.16
324912,0\H,2,1.09624951,1,108.50874799,3,-118.13217478,0\C,1,1.8323945
6,2,99.83998732,4,-57.47606986,0\C,6,1.38163012,1,130.97098149,2,82.50
892648,0\N,7,1.37783944,6,106.06714965,1,-179.56809806,0\C,8,1.3702112
9,7,106.44104788,6,0.23611261,0\N,9,1.31431509,8,112.50483309,7,-0.321
60394,0\H,7,1.08069534,6,131.11280638,1,-0.50794144,0\H,9,1.08406297,8
,121.60100484,7,-179.9763051,0\C,8,1.45228968,7,126.75537852,6,-178.88
191181,0\H,13,1.09631948,8,110.88114997,7,63.5192362,0\H,13,1.09374848
,8,108.90109439,7,-176.84335281,0\H,13,1.09581302,8,110.56551264,7,-57
.49404413,0\C,1,1.83275271,6,101.28401796,7,-19.42617506,0\N,17,1.3915
725,1,126.26685432,6,53.68877489,0\C,18,1.31452977,17,105.36525905,1,1
78.75456217,0\N,19,1.36921407,18,112.62156602,17,-0.18073735,0\C,20,1.
37771628,19,106.15850846,18,0.08913041,0\H,21,1.0814518,20,121.9482458
1,19,-179.95419483,0\H,19,1.08435837,18,125.88037319,17,179.96831258,0
\C,20,1.45296919,19,126.89341311,18,-179.42646049,0\H,24,1.09356392,20
,108.87312447,19,-1.53345794,0\H,24,1.09590317,20,110.73773867,19,117.
97281756,0\H,24,1.09576379,20,110.62556969,19,-120.96602617,0\\Version
=AM64L-G03RevD.01\State=1-A\HF=-906.930343\MP2=-909.0725283\RMSD=5.425
e-09\Thermal=0.\PG=C01 [X(C9H13N4P1)]\\@

207-Me$^+$

1\1\GINC-BORIX\SP\RMP2-FC\6-31+G(2d,p)\C10H16N4P1(1+)\CHRISTOPH\17-Feb
-2012\0\\#p MP2(FC)/6-31+g(2d,p) scf=tight\\imi42mesp_1\\1,1\C\C,1,1.3
8459839\N,2,1.36870875,1,105.26205039\C,3,1.3697458,2,107.00361799,1,0
.03431269,0\N,4,1.31532478,3,112.52494185,2,-0.12052499,0\C,1,2.920567
86,5,96.61283008,4,153.15932254,0\N,6,1.38219349,1,151.22548682,5,161.
67703471,0\C,7,1.31441047,6,104.35367978,1,164.90552764,0\N,8,1.372025
81,7,112.47729231,6,0.27766555,0\C,9,1.3680596,8,107.14910044,7,-0.057

90591,0\C,6,2.93815425,1,60.42427145,5,99.15036896,0\P,6,1.77923445,1,
34.92927307,5,134.31624181,0\C,12,1.81588659,6,108.24623822,1,120.5344
3119,0\H,13,1.09505046,12,110.77014374,6,171.38707246,0\H,13,1.0946801
9,12,109.85940474,6,-66.76432818,0\H,13,1.09540066,12,107.85620785,6,5
1.35965471,0\H,10,1.08075544,9,123.16473183,8,178.87351278,0\H,2,1.081
33483,1,132.73112512,5,-179.93279382,0\C,9,1.4630519,8,126.42388952,7,
-179.8026262,0\H,19,1.09378895,9,110.05369568,8,-122.13813018,0\H,19,1
.09214032,9,108.64400462,8,-2.83799506,0\H,19,1.09402189,9,110.2470139
,8,116.66409819,0\C,3,1.46381013,2,126.4645715,1,-179.7904343,0\H,23,1
.09201504,3,108.61332506,2,-179.60366041,0\H,23,1.09386578,3,110.14209
33,2,-60.21187953,0\H,23,1.09387736,3,110.14784692,2,61.03031657,0\H,4
,1.0829447,3,121.93275271,2,179.96889,0\H,8,1.08290688,7,125.57609562,
6,-179.53326135,0\H,11,1.09510484,6,92.87389685,1,-84.79710841,0\H,11,
1.09542973,6,144.76792937,1,45.56293648,0\H,11,1.09566041,6,86.4369493
9,1,165.90349225,0\\Version=AM64L-G03RevD.01\State=1-A\HF=-946.3971098
\MP2=-948.6818207\RMSD=3.720e-09\Thermal=0.\PG=C01 [X(C10H16N4P1)]\\@

208

1\1\GINC-LIEBIG\SP\RMP2-FC\6-31+G(2d,p)\C12H15N6P1\CHRISTOPH\17-Feb-20
12\0\\#p MP2(FC)/6-31+g(2d,p) scf=tight\\imi43sp_36\\0,1\P\C,1,1.83005
643\C,2,1.38149235,1,130.78238125\N,3,1.37964077,2,106.00358183,1,174.
37809091,0\C,4,1.36932346,3,106.40628951,2,0.08981988,0\N,5,1.31410452
,4,112.54238694,3,-0.39261348,0\H,3,1.08012403,2,131.63160087,1,-7.830
73662,0\H,5,1.08402962,4,121.6748871,3,-179.97719588,0\C,4,1.45234803,
3,126.70262971,2,-179.35631903,0\H,9,1.09362627,4,108.87727499,3,-178.
1824727,0\H,9,1.09599214,4,110.681768,3,-58.75224339,0\H,9,1.09620769,
4,110.79146309,3,62.28883857,0\C,1,1.8340536,2,101.27670742,3,5.285864
94,0\N,13,1.39164315,1,128.16574882,2,47.13093083,0\C,14,1.3151671,13,
105.44305659,1,178.27199419,0\N,15,1.36815363,14,112.53664469,13,0.016
51918,0\C,16,1.37631693,15,106.23507315,14,0.09859642,0\H,17,1.0813626
5,16,122.00225864,15,-179.91409657,0\H,15,1.08414773,14,125.83651348,1
3,-179.87931922,0\C,16,1.45396733,15,126.85780166,14,-179.44030293,0\H
,20,1.09555902,16,110.58206429,15,-120.70411394,0\H,20,1.09341339,16,1
08.86156752,15,-1.23465436,0\H,20,1.09555697,16,110.60647552,15,118.24
561432,0\C,1,1.81658031,2,104.2446033,3,113.41112891,0\N,24,1.39489428
,1,131.50044736,2,-43.88004155,0\C,25,1.36109621,24,106.54863286,1,-17
8.894313,0\N,26,1.32145467,25,113.289718,24,0.43447927,0\C,27,1.369660
8,26,104.10420222,25,-0.52687039,0\H,28,1.08357655,27,121.52761635,26,
-179.56859271,0\H,26,1.08449017,25,121.36747298,24,-179.56945522,0\C,2
5,1.45675813,24,128.03136256,1,4.67453345,0\H,31,1.09570293,25,110.464
73763,24,71.86828081,0\H,31,1.0946241,25,108.17870505,24,-168.71548201
,0\H,31,1.09346231,25,109.9144259,24,-48.99720053,0\\Version=AM64L-G03
RevD.01\State=1-A\HF=-1130.5996899\MP2=-1133.5187343\RMSD=4.898e-09\Th
ermal=0.\PG=C01 [X(C12H15N6P1)]\\@

208-Me$^+$

1\1\GINC-STEAK\SP\RMP2-FC\6-31+G(2d,p)\C13H18N6P1(1+)\CHRISTOPH\17-Feb
-2012\0\\#p MP2(FC)/6-31+g(2d,p) scf=tight\\imi43mesp_5\\1,1\C\C,1,1.3
8326952\N,2,1.36865219,1,105.14333661\C,3,1.37014982,2,107.12233439,1,
0.12521579,0\N,4,1.3153831,3,112.47019735,2,0.19333954,0\C,1,2.9163871
,2,148.72042443,3,137.33117197,0\N,6,1.38363333,1,152.95359072,2,45.35
075689,0\C,7,1.31547752,6,104.42921598,1,-162.75781537,0\N,8,1.3700168
,7,112.4739596,6,-0.38875162,0\C,9,1.36860728,8,107.13600139,7,0.15181
295,0\C,6,2.91638002,1,60.00294433,2,41.21238631,0\N,11,1.38379349,6,1
53.26010651,1,7.56189022,0\C,12,1.31529241,11,104.42858472,6,-164.6528
6793,0\N,13,1.37018402,12,112.47216426,11,-0.39778427,0\C,14,1.3686722
2,13,107.13547676,12,0.1621639,0\P,11,1.78441538,6,35.20944835,1,35.12
413679,0\C,16,1.82253156,11,109.26909896,6,119.96783371,0\H,17,1.09534

643,16,109.07721556,11,-67.31449458,0\H,17,1.09534457,16,109.0381621,1
1,52.65283314,0\H,17,1.095353,16,109.12929389,11,172.65930788,0\H,10,1
.08063925,9,123.55514473,8,-179.01131848,0\H,15,1.08066544,14,123.5331
2777,13,-178.81621722,0\H,2,1.08055826,1,131.34701232,5,178.56380921,0
\C,14,1.4622379,13,126.50710299,12,-179.43398073,0\H,24,1.09405769,14,
110.18860012,13,118.09258569,0\H,24,1.09396749,14,110.15765263,13,-120
.76061988,0\H,24,1.0921966,14,108.67158586,13,-1.34690206,0\C,9,1.4622
8814,8,126.51191156,7,-179.63531334,0\H,28,1.09393775,9,110.16382473,8
,-119.95214862,0\H,28,1.09216286,9,108.6641306,8,-0.51105587,0\H,28,1.
09400311,9,110.16967409,8,118.90935426,0\C,3,1.46227995,2,126.35284905
,1,-179.7808052,0\H,32,1.09221131,3,108.68845281,2,-177.5555276,0\H,32
,1.09385879,3,110.06638218,2,-58.24256693,0\H,32,1.09411411,3,110.2659
2583,2,62.88873759,0\H,4,1.08287633,3,121.9627936,2,-179.7601006,0\H,8
,1.08286524,7,125.56110599,6,179.52560454,0\H,13,1.08288439,12,125.557
7727,11,179.59005322,0\\Version=AM64L-G03RevD.01\State=1-A\HF=-1170.07
56287\MP2=-1173.1329751\RMSD=8.759e-09\Thermal=0.\PG=C01 [X(C13H18N6P1
)]\\@

209

1\1\GINC-CALYPSO\SP\RMP2-FC\6-31+G(2d,p)\C11H15N2P1\CHRISTOPH\20-Oct-2
009\0\\#p MP2(FC)/6-31+g(2d,p) scf=tight\\z2sp_9\\0,1\P\C,1,1.8242776\
C,2,1.43786643,1,131.31101676\C,3,1.37789589,2,107.63700726,1,-179.460
33029,0\N,4,1.37980517,3,108.41230041,2,-0.17478802,0\C,5,1.37176324,4
,108.73874863,3,0.50974898,0\C,1,1.83111244,2,102.34260679,6,122.68984
855,0\C,7,1.43632137,1,125.00498993,2,178.44580301,0\C,8,1.37844721,7,
107.73177299,1,176.16522843,0\N,9,1.37728449,8,108.29876523,7,-0.85207
788,0\C,10,1.37581019,9,108.88484223,8,0.88226037,0\C,1,1.87209367,2,1
00.73087163,6,-134.4243254,0\H,11,1.08180637,10,120.86700555,9,-179.77
766052,0\H,9,1.0825543,8,131.11124952,7,179.63622611,0\H,8,1.08343204,
7,126.52928121,1,-2.64426499,0\H,6,1.08268289,5,120.83996619,4,179.451
51821,0\H,4,1.08272044,3,131.16109056,2,179.78758826,0\H,3,1.08284444,
2,126.55076132,1,1.3689962,0\C,5,1.4517527,4,125.38996379,3,175.222227
2,0\H,19,1.09409464,5,109.36495523,4,156.66698203,0\H,19,1.09791201,5,
111.60725103,4,-82.85288225,0\H,19,1.09482315,5,109.67694383,4,37.8640
702,0\C,10,1.45052496,9,125.50124894,8,175.72003096,0\H,23,1.09842269,
10,111.76498334,9,-85.85891994,0\H,23,1.09488114,10,109.62480313,9,34.
82718387,0\H,23,1.09449634,10,109.44197998,9,153.58730038,0\H,12,1.096
79087,1,108.87828718,2,-171.41405377,0\H,12,1.09642298,1,112.81234653,
2,-50.90682917,0\H,12,1.09671204,1,109.19989437,2,70.64653383,0\\Versi
on=AM64L-G03RevD.01\State=1-A\HF=-874.9174164\MP2=-877.0037635\RMSD=1.
559e-09\Thermal=0.\PG=C01 [X(C11H15N2P1)]\\@

209-Me$^+$

1\1\GINC-YIN\SP\RMP2-FC\6-31+G(2d,p)\C12H18N2P1(1+)\CHRISTOPH\20-Oct-2
009\0\\#p MP2(FC)/6-31+g(2d,p) scf=tight\\z2mesp_10\\1,1\P\C,1,1.82409
428\H,2,1.09586349,1,110.21710012\H,2,1.09475672,1,109.42994637,3,119.
71078702,0\H,2,1.09525669,1,109.78720582,4,119.82577813,0\C,1,1.769133
02,2,110.81850939,4,-58.68660215,0\C,6,1.43739483,1,126.98076666,2,65.
33967496,0\C,7,1.37483531,6,106.66788115,1,-178.19206572,0\N,8,1.37959
052,7,108.78045355,6,0.07286134,0\C,9,1.35918357,8,109.48720758,7,0.04
363129,0\C,1,1.7685337,6,110.7165937,10,127.12095794,0\C,11,1.43772277
,1,127.66155223,6,166.00461399,0\C,12,1.37439799,11,106.62337133,1,177
.69174096,0\N,13,1.38027919,12,108.77343112,11,-0.05440407,0\C,14,1.35
84909,13,109.52314539,12,-0.01679076,0\C,1,1.82406041,11,111.18606532,
15,104.33092589,0\H,15,1.08145033,14,121.60319611,13,179.54891427,0\H,
13,1.0816436,12,130.52739822,11,179.54117408,0\H,12,1.08225643,11,127.
62723928,1,-2.23393801,0\H,10,1.08170385,9,120.90700542,8,-179.9776385
7,0\H,8,1.08167553,7,130.55801555,6,-179.95735168,0\H,7,1.08212338,6,1

27.15726305,1,2.09945962,0\C,9,1.4619532,8,124.82469734,7,179.38959071
,0\H,23,1.0921173,9,109.06966465,8,176.4436076,0\H,23,1.09451061,9,110
.20292679,8,-63.83075686,0\H,23,1.09417726,9,110.01689456,8,56.9644000
7,0\C,14,1.46156225,13,124.78126362,12,178.32187657,0\H,27,1.09478202,
14,110.36699847,13,-66.62226768,0\H,27,1.09399652,14,109.85404699,13,5
4.14335098,0\H,27,1.09209558,14,109.03788829,13,173.46206944,0\H,16,1.
09463244,1,109.8326776,11,-62.02927042,0\H,16,1.09537891,1,108.9892915
9,11,56.75372952,0\H,16,1.09571059,1,110.63064084,11,176.52947997,0\\V
ersion=AM64L-G03RevD.01\State=1-A\HF=-914.3891654\MP2=-916.619263\RMSD
=8.348e-09\Thermal=0.\PG=C01 [X(C12H18N2P1)]\\@

210

1\1\GINC-YIN\SP\RMP2-FC\6-31+G(2d,p)\C17H27N2P1\CHRISTOPH\04-Jan-2010\
0\\#p MP2(FC)/6-31+g(2d,p) scf=tight\\zz2sp_3\\0,1\P\C,1,1.87459049\C,
1,1.82613226,2,100.26726432\C,3,1.44692877,1,131.31958622,2,47.5865309
7,0\C,4,1.38179658,3,107.22280978,1,-176.3427244,0\N,5,1.39079773,4,10
8.15545634,3,0.24717984,0\C,6,1.3791451,5,109.63821887,4,-0.20105095,0
\C,1,1.84133951,3,106.85690715,7,125.4224912,0\C,8,1.39422705,1,130.98
008159,3,-24.02149102,0\N,9,1.38606385,8,107.91130329,1,-172.96868274,
0\C,10,1.38516618,9,109.89623654,8,1.58593821,0\C,11,1.38309195,10,107
.79633462,9,-0.60731781,0\C,12,1.50699379,11,125.63673843,10,178.26767
706,0\H,13,1.09855754,12,111.07764804,11,-138.10004365,0\H,13,1.096531
67,12,111.38033974,11,-18.87768148,0\H,13,1.09941583,12,112.73775905,1
1,101.57273123,0\C,11,1.50009409,10,121.91067618,9,179.78928246,0\H,17
,1.09415662,11,109.86066896,10,-177.98601085,0\H,17,1.10009471,11,112.
63053973,10,-58.4791931,0\H,17,1.10075351,11,112.56891583,10,62.984508
02,0\C,9,1.5015831,8,132.54361066,1,3.82065331,0\H,21,1.10119498,9,112
.46180359,8,115.66328567,0\H,21,1.10024115,9,111.38628813,8,-124.05805
333,0\H,21,1.09047073,9,110.23046254,8,-4.15370292,0\C,10,1.45064987,9
,124.52341689,8,177.25760726,0\H,25,1.0927317,10,109.60216119,9,162.70
995269,0\H,25,1.09970402,10,111.87417917,9,-77.00662867,0\H,25,1.09496
418,10,110.37380193,9,43.74833246,0\C,4,1.50660937,3,126.77947104,1,4.
23079989,0\H,29,1.0963167,4,110.78177757,3,34.89616041,0\H,29,1.097245
68,4,111.5470679,3,154.86831418,0\H,29,1.09976673,4,112.21214225,3,-84
.95952386,0\C,5,1.49946863,4,130.74716637,3,179.9274103,0\H,33,1.09422
44,5,109.97138178,4,3.88481175,0\H,33,1.10024729,5,112.44267326,4,123.
56652265,0\H,33,1.10065886,5,112.47037738,4,-115.42872825,0\C,6,1.4513
1461,5,124.10127404,4,-178.85756875,0\H,37,1.09159753,6,109.51887316,5
,-173.38522588,0\H,37,1.09670682,6,110.70517529,5,-54.00948575,0\H,37,
1.09842166,6,111.38491537,5,66.70062581,0\C,7,1.50081322,6,122.0691896
3,5,-179.79099326,0\H,41,1.09979853,7,112.4087002,6,61.91998328,0\H,41
,1.09399059,7,108.82321762,6,-178.61812628,0\H,41,1.09988549,7,112.288
24699,6,-59.46356259,0\H,2,1.09485657,1,110.03010073,3,170.9006074,0\H
,2,1.09487436,1,113.81960957,3,-66.81458787,0\H,2,1.09682423,1,107.361
45984,3,53.80626355,0\\Version=AM64L-G03RevD.01\State=1-A\HF=-1109.151
5639\MP2=-1112.1878892\RMSD=5.077e-09\Thermal=0.\PG=C01 [X(C17H27N2P1)
]\\@

210-Me$^+$

1\1\GINC-YANG\SP\RMP2-FC\6-31+G(2d,p)\C18H30N2P1(1+)\CHRISTOPH\04-Jan-
2010\0\\#p MP2(FC)/6-31+g(2d,p) scf=tight\\zz2mesp_1\\1,1\C\C,1,1.4523
4649\C,2,1.38166702,1,106.22882184\N,3,1.39033515,2,108.43486016,1,0.4
7475168,0\C,4,1.3660702,3,110.57538615,2,-0.32334787,0\C,1,2.96764058,
5,98.77033445,4,-157.5368958,0\C,6,1.45234017,1,145.60572718,5,-58.420
25612,0\C,7,1.3816735,6,106.2282221,1,138.14232936,0\N,8,1.39032917,7,
108.43537056,6,0.47084036,0\C,9,1.36607163,8,110.5750498,7,-0.3198142,
0\C,6,2.9227419,1,60.92104408,5,-173.41374161,0\C,2,1.50682092,1,128.8
6850095,5,178.94536238,0\H,12,1.09371586,2,110.61506175,1,178.31303222

,0\H,12,1.09829079,2,112.312054,1,-62.83928174,0\H,12,1.09716115,2,112
.69828742,1,59.01498505,0\C,5,1.50243043,4,121.70314657,3,179.21472763
,0\H,16,1.09800357,5,111.6897087,4,57.05874489,0\H,16,1.0925626,5,110.
97827387,4,176.09028697,0\H,16,1.0987608,5,111.69283994,4,-63.43790565
,0\C,4,1.45964935,3,123.45175969,2,-179.62535143,0\H,20,1.09441774,4,1
10.10786243,3,-53.68687671,0\H,20,1.09587889,4,110.63445954,3,66.75256
604,0\H,20,1.0903539,4,109.79921882,3,-173.11948057,0\C,3,1.49963636,2
,130.99665412,1,-179.66389051,0\H,24,1.09876258,3,111.95231693,2,-116.
96900657,0\H,24,1.09261337,3,110.66656964,2,2.65704533,0\H,24,1.098365
3,3,111.89090173,2,122.30091579,0\C,7,1.50681949,6,128.86930608,1,-42.
44153308,0\H,28,1.09371653,7,110.61514093,6,178.26565787,0\H,28,1.0982
9336,7,112.31324805,6,-62.88332541,0\H,28,1.09715594,7,112.69729774,6,
58.97082161,0\C,8,1.49963594,7,130.99622868,6,-179.66063336,0\H,32,1.0
9261201,8,110.66631813,7,2.59060387,0\H,32,1.09837129,8,111.89212799,7
,122.23672796,0\H,32,1.09875752,8,111.95141042,7,-117.03358364,0\C,9,1
.45965237,8,123.44945256,7,-179.64755995,0\H,36,1.09035031,9,109.79920
423,8,-173.21575948,0\H,36,1.0958685,9,110.63140472,8,66.65903283,0\H,
36,1.09443068,9,110.11085134,8,-53.78059451,0\C,10,1.50242985,9,121.70
692476,8,179.21045273,0\H,40,1.09799649,10,111.6893843,9,56.99843821,0
\H,40,1.09876554,10,111.69522773,9,-63.4991961,0\H,40,1.09256951,10,11
0.97559239,9,176.02544688,0\P,1,1.78239024,5,122.70975566,4,176.886488
9,0\C,44,1.82764483,1,108.11547985,5,-73.50535533,0\H,45,1.09366439,44
,110.56534087,1,63.0258596,0\H,45,1.09356847,44,111.94759943,1,-174.53
300035,0\H,45,1.09590488,44,107.04317923,1,-54.84713157,0\H,11,1.09590
378,6,85.38909731,1,160.12795663,0\H,11,1.09366532,6,92.30644562,1,-91
.59617035,0\H,11,1.09356742,6,147.1123432,1,41.16010792,0\\Version=AM6
4L-G03RevD.01\State=1-A\HF=-1148.6280936\MP2=-1151.8099047\RMSD=5.058e
-09\Thermal=0.\PG=C01 [X(C18H30N2P1)]\\@

211
1\1\GINC-YANG\SP\RMP2-FC\6-31+G(2d,p)\C15H23N2P1\CHRISTOPH\20-Jan-2010
\0\\#p MP2(FC)/6-31+g(2d,p) scf=tight\\xx2sp_4\\0,1\P\C,1,1.82767184\C
,2,1.3925343,1,123.89397053\N,3,1.3838459,2,108.05728306,1,178.5052187
1,0\C,4,1.39015371,3,109.58387382,2,0.95754761,0\C,5,1.37664212,4,107.
390932,3,-0.87439516,0\C,1,1.86878473,2,100.81891279,3,-113.74988052,0
\C,1,1.82585755,2,103.60704157,3,142.56079089,0\C,8,1.43607787,1,129.8
1383733,2,70.46334264,0\C,9,1.37726987,8,108.51454086,1,179.62967582,0
\N,10,1.38884412,9,107.33691598,8,0.36016662,0\C,11,1.38343689,10,109.
6452716,9,-0.88203861,0\H,9,1.08331265,8,126.72685467,1,0.00353925,0\H
,6,1.08300841,5,125.28589726,4,179.21364158,0\C,11,1.45151493,10,125.1
8273256,9,-176.46944036,0\H,15,1.09408723,11,109.98664477,10,-148.1304
5348,0\H,15,1.09347403,11,109.72823069,10,-29.28021211,0\H,15,1.099349
04,11,111.87334719,10,91.20157847,0\C,12,1.49941071,11,121.69903271,10
,-179.08292175,0\H,19,1.10051398,12,112.48294919,11,-66.09258276,0\H,1
9,1.09971068,12,112.23092878,11,55.13302027,0\H,19,1.09444772,12,108.9
9097327,11,174.58376068,0\C,10,1.4991138,9,129.95415844,8,-179.5490952
,0\H,23,1.10002597,10,112.70493384,9,119.76517747,0\H,23,1.10047784,10
,112.70456506,9,-118.62710426,0\H,23,1.09495391,10,109.12075834,9,0.47
551944,0\C,3,1.49980797,2,130.39875066,1,-1.46847953,0\H,27,1.10065156
,3,112.5260937,2,115.5222214,0\H,27,1.10002039,3,112.20186744,2,-123.4
2922153,0\H,27,1.09435801,3,109.10406393,2,-3.9054081,0\C,4,1.45112372
,3,124.89730574,2,176.43344296,0\H,31,1.0932145,4,109.68903295,3,158.5
6204657,0\H,31,1.09942249,4,111.85881819,3,-81.03309692,0\H,31,1.09450
686,4,110.09762887,3,39.65274894,0\C,5,1.49936916,4,122.69664601,3,179
.02359049,0\H,35,1.10049789,5,112.73132841,4,61.50044166,0\H,35,1.0949
2665,5,109.05227258,4,-179.43818076,0\H,35,1.10008034,5,112.74489703,4
,-60.18815199,0\H,7,1.09677587,1,113.10309519,8,55.70864492,0\H,7,1.09
64307,1,108.63745518,8,175.92040661,0\H,7,1.09672566,1,109.37914967,8,

-65.91490353,0\\Version=AM64L-G03RevD.01\State=1-A\HF=-1031.0855839\MP
2=-1033.7983045\RMSD=4.573e-09\Thermal=0.\PG=C01 [X(C15H23N2P1)]\\@

211-Me^{+}

1\1\GINC-EDDY\SP\RMP2-FC\6-31+G(2d,p)\C16H26N2P1(1+)\CHRISTOPH\21-Jan-
2010\0\\#p MP2(FC)/6-31+g(2d,p) scf=tight\\xx2mesp_17\\1,1\C\C,1,1.401
2804\N,2,1.37052334,1,107.01051289\C,3,1.39107828,2,110.66349881,1,0.2
1546735,0\C,4,1.37500552,3,107.43385529,2,-0.36789222,0\C,1,2.97598869
,2,102.75513977,3,-158.9803194,0\C,6,1.40091117,1,103.7055043,2,77.710
78557,0\N,7,1.37052012,6,107.02731966,1,-156.76049089,0\C,8,1.39114306
,7,110.63959942,6,0.24258452,0\C,9,1.37518611,8,107.4430148,7,-0.33992
107,0\C,6,2.8991139,1,60.5697894,2,-107.16795202,0\H,10,1.08205844,9,1
25.05873412,8,-179.68763689,0\H,5,1.08202376,4,125.01872637,3,-179.902
3395,0\C,9,1.49889019,8,123.06155782,7,179.7328241,0\H,14,1.09402153,9
,109.20185966,8,-179.38515555,0\H,14,1.09809614,9,112.33530832,8,-60.1
1447524,0\H,14,1.09839526,9,112.25423654,8,61.60258716,0\C,8,1.4605320
6,7,124.03651796,6,178.64390773,0\H,18,1.09068362,8,109.28842446,7,168
.22235441,0\H,18,1.09626976,8,110.90557681,7,-71.84383288,0\H,18,1.094
08381,8,110.21956591,7,49.09956416,0\C,7,1.49994478,6,131.30649798,1,2
3.59995718,0\H,22,1.0938506,7,110.82431317,6,-7.46308797,0\H,22,1.0991
4109,7,111.80130255,6,113.02792188,0\H,22,1.09815846,7,111.53247668,6,
-126.90827013,0\C,2,1.49988848,1,131.39221732,5,-179.47598223,0\H,26,1
.09357634,2,110.78591693,1,-8.1267374,0\H,26,1.09805917,2,111.51041432
,1,-127.63904061,0\H,26,1.09925854,2,111.76600985,1,112.3442656,0\C,3,
1.46054999,2,124.05809147,1,178.58511243,0\H,30,1.09070983,3,109.28998
406,2,167.29975399,0\H,30,1.09633138,3,110.93811901,2,-72.73727407,0\H
,30,1.09398366,3,110.19584153,2,48.21152928,0\C,4,1.49882449,3,123.043
60152,2,179.56942999,0\H,34,1.09403883,4,109.2246418,3,-179.1626769,0\
H,34,1.09846378,4,112.24516527,3,61.81075369,0\H,34,1.09810023,4,112.3
2032917,3,-59.8735501,0\P,1,1.77194987,2,127.19872303,3,176.40086521,0
\C,38,1.82593684,1,107.45212793,2,171.82326184,0\H,39,1.09512105,38,10
8.63110855,1,-179.9624764,0\H,39,1.09457065,38,110.16263017,1,-60.8144
5352,0\H,39,1.09545198,38,110.67380478,1,60.14687189,0\H,11,1.09461138
,6,90.73123694,1,93.52887182,0\H,11,1.09549558,6,90.83440687,1,-157.12
488317,0\H,11,1.09508717,6,144.30080503,1,-31.22744885,0\\Version=AM64
L-G03RevD.01\State=1-A\HF=-1070.5608253\MP2=-1073.4216484\RMSD=4.369e-
09\Thermal=0.\PG=C01 [X(C16H26N2P1)]\\@

212

1\1\GINC-YANG\SP\RMP2-FC\6-31+G(2d,p)\C15H18N3P1\CHRISTOPH\21-Oct-2009
\0\\#p MP2(FC)/6-31+g(2d,p) scf=tight\\z3sp_55\\0,1\P\C,1,1.83102823\C
,2,1.43564483,1,125.40693473\C,3,1.37815685,2,107.68902916,1,178.09915
886,0\N,4,1.3793393,3,108.31013747,2,-0.16239237,0\C,5,1.37462196,4,10
8.85773599,3,-0.15812031,0\C,1,1.83084399,2,101.61428424,6,-94.1954651
4,0\C,7,1.43571696,1,125.46094949,2,-166.75053485,0\C,8,1.37818003,7,1
07.68449941,1,178.10057473,0\N,9,1.37932314,8,108.3107073,7,-0.1720305
9,0\C,10,1.37459866,9,108.86100201,8,-0.13583744,0\C,1,1.83043203,7,10
1.57945478,11,-94.18245887,0\C,12,1.38719257,1,128.31921339,7,9.160174
3,0\N,13,1.37465177,12,109.00368073,1,-177.76611503,0\C,14,1.37929653,
13,108.86069393,12,0.42522011,0\C,15,1.37813658,14,108.30991495,13,-0.
14190877,0\H,11,1.08190921,10,121.08803633,9,-178.42551333,0\H,9,1.082
60136,8,131.16249232,7,-179.92445261,0\H,8,1.08307187,7,126.29831129,1
,-2.65695288,0\H,13,1.08190334,12,129.95181695,1,1.03247769,0\H,16,1.0
8310014,15,126.00200741,14,-179.3928405,0\H,15,1.08259063,14,120.51961
337,13,179.67206652,0\H,6,1.081934,5,121.03112522,4,-178.38959258,0\H,
4,1.08256905,3,131.18803302,2,-179.92074825,0\H,3,1.08306784,2,126.278
13454,1,-2.65986686,0\C,5,1.4509436,4,125.3329375,3,-173.43137483,0\H,
26,1.09430803,5,109.36790443,4,-157.14401376,0\H,26,1.09504167,5,109.7

0537599,4,-38.32135086,0\H,26,1.09820421,5,111.70024279,4,82.4139024,0
\C,10,1.45092831,9,125.31429021,8,-173.32767746,0\H,30,1.09438564,10,1
09.4150785,9,-156.50423558,0\H,30,1.0949639,10,109.67092231,9,-37.6730
6926,0\H,30,1.09820661,10,111.69644902,9,83.02969198,0\C,14,1.45088496
,13,125.42576833,12,173.69727329,0\H,34,1.09823746,14,111.6992006,13,-
90.58760302,0\H,34,1.09424421,14,109.33803316,13,29.8154779,0\H,34,1.0
9512907,14,109.75159721,13,148.65900358,0\\Version=AM64L-G03RevD.01\St
ate=1-A\HF=-1082.5793887\MP2=-1085.4121486\RMSD=8.202e-09\Thermal=0.\P
G=C01 [X(C15H18N3P1)]\\@

212-Me⁺

1\1\GINC-YANG\SP\RMP2-FC\6-31+G(2d,p)\C16H21N3P1(1+)\CHRISTOPH\21-Oct-
2009\0\\#p MP2(FC)/6-31+g(2d,p) scf=tight\\z3mesp_90\\1,1\P\C,1,1.8254
6487\H,2,1.09509105,1,109.68404719\H,2,1.09514003,1,109.7185421,3,120.
00276528,0\H,2,1.09515497,1,109.67337573,3,-120.00327009,0\C,1,1.77452
369,2,108.59468966,3,176.63977836,0\C,6,1.43736982,1,127.9642799,2,-54
.16810983,0\C,7,1.37542764,6,106.66259612,1,-179.92927612,0\N,8,1.3800
979,7,108.73608425,6,0.02992482,0\C,9,1.36041575,8,109.44856323,7,-0.0
9536502,0\C,1,1.77473208,6,110.36665153,10,7.04479494,0\C,11,1.4374089
7,1,127.83722158,6,65.28819063,0\C,12,1.37546552,11,106.64462054,1,-17
9.89747925,0\N,13,1.38007197,12,108.74259589,11,-0.0758594,0\C,14,1.36
058485,13,109.44576928,12,-0.0032266,0\C,1,1.77459036,6,110.36952085,1
0,-115.35180242,0\C,16,1.39411786,1,125.12337127,6,7.17910783,0\N,17,1
.36054532,16,108.2218846,1,-179.98157922,0\C,18,1.38001244,17,109.4483
7657,16,0.1044285,0\C,19,1.37546017,18,108.74765875,17,-0.07045673,0\H
,17,1.08149292,16,130.12963951,1,-0.34268492,0\H,20,1.08226364,19,126.
00032603,18,-179.57504722,0\H,19,1.08177108,18,120.69073682,17,179.710
65212,0\H,15,1.08149831,14,121.61155275,13,-179.68429834,0\H,13,1.0817
3899,12,130.58495196,11,-179.73602475,0\H,12,1.08224107,11,127.3608751
9,1,-0.34194191,0\H,10,1.08145414,9,121.62140145,8,-179.67185961,0\H,8
,1.08173211,7,130.58748876,6,-179.68668852,0\H,7,1.08230402,6,127.3488
466,1,-0.36015569,0\C,9,1.46052246,8,124.94213105,7,-177.32577122,0\H,
30,1.09387276,9,109.78176551,8,-48.69721682,0\H,30,1.09516901,9,110.59
204066,8,72.04698936,0\H,30,1.09228527,9,109.06427965,8,-167.86106713,
0\C,14,1.46053528,13,124.91239879,12,-177.15760336,0\H,34,1.09238034,1
4,109.10506317,13,-166.71300689,0\H,34,1.09382254,14,109.72063997,13,-
47.58214924,0\H,34,1.09521345,14,110.61683549,13,73.1260971,0\C,18,1.4
6044152,17,125.57913752,16,177.14407062,0\H,38,1.09381778,18,109.73142
063,17,135.6728144,0\H,38,1.09521859,18,110.60891332,17,-103.62796036,
0\H,38,1.09240447,18,109.11914056,17,16.52706417,0\\Version=AM64L-G03R
evD.01\State=1-A\HF=-1122.0647003\MP2=-1125.0398157\RMSD=5.419e-09\The
rmal=0.\PG=C01 [X(C16H21N3P1)]\\@

213

1\1\GINC-AZAZEL\SP\RMP2-FC\6-31+G(2d,p)\C21H30N3P1\CHRISTOPH\23-Jan-20
10\0\\#p MP2(FC)/6-31+g(2d,p) scf=tight\\xx3sp_9\\0,1\P\C,1,1.82787796
\C,2,1.39127434,1,124.17158265\N,3,1.3838706,2,108.09065644,1,-179.733
47777,0\C,4,1.39003598,3,109.57669255,2,-1.1194994,0\C,5,1.37788323,4,
107.36461835,3,0.9124166,0\C,1,1.82848809,2,102.27069749,3,-152.527579
92,0\C,7,1.39151435,1,124.17686404,2,100.15013032,0\N,8,1.38448365,7,1
08.02921372,1,-179.54671453,0\C,9,1.38950739,8,109.57891009,7,-1.27083
316,0\C,10,1.37794076,9,107.39548401,8,1.04229101,0\C,1,1.82834974,2,1
02.19757247,3,101.9121132,0\C,12,1.43425527,1,129.27685216,2,24.667614
54,0\C,13,1.37782961,12,108.37382595,1,-179.90270764,0\N,14,1.38957795
,13,107.41729511,12,-0.38654063,0\C,15,1.38439828,14,109.57441818,13,1
.03506961,0\H,13,1.08318497,12,125.89687898,1,-0.90851026,0\H,11,1.083
16992,10,125.69938561,9,-179.26918602,0\H,6,1.08320335,5,125.65348381,

4,-179.05117664,0\C,15,1.45081084,14,125.03526808,13,175.838429,0\H,20
,1.09975273,15,111.95813006,14,-87.31672383,0\H,20,1.09401585,15,109.8
3811197,14,33.21959867,0\H,20,1.0939123,15,109.90239797,14,152.0395090
8,0\C,16,1.49937944,15,121.92777656,14,178.98570388,0\H,24,1.09993105,
16,112.29263342,15,-53.8321998,0\H,24,1.0948926,16,108.97838012,15,-17
3.17046779,0\H,24,1.10061334,16,112.6455516,15,67.59639521,0\C,14,1.49
921256,13,130.02511721,12,179.40221246,0\H,28,1.095033,14,109.10387592
,13,-0.0329017,0\H,28,1.10071218,14,112.72964752,13,119.09047993,0\H,2
8,1.10035799,14,112.74524243,13,-119.35041191,0\C,10,1.49930159,9,122.
66610486,8,-178.60039877,0\H,32,1.09503701,10,109.05530104,9,179.96697
496,0\H,32,1.10067827,10,112.75997409,9,-60.93685332,0\H,32,1.10031585
,10,112.75625351,9,60.70234308,0\C,9,1.45076513,8,124.87469923,7,-176.
4545065,0\H,36,1.09448995,9,110.01213055,8,-38.43635396,0\H,36,1.09967
317,9,111.93626544,8,82.24272136,0\H,36,1.09357894,9,109.75165943,8,-1
57.28124658,0\C,8,1.4993154,7,130.13866486,1,-0.19321879,0\H,40,1.1007
6743,8,112.60444539,7,-110.18491976,0\H,40,1.0997887,8,112.26638259,7,
128.48604984,0\H,40,1.09499732,8,109.00945518,7,9.06235007,0\C,3,1.499
18172,2,130.26725666,1,-0.50658267,0\H,44,1.0999206,3,112.25199591,2,1
27.52606933,0\H,44,1.09479693,3,109.08224841,2,7.98952007,0\H,44,1.100
74966,3,112.51123978,2,-111.28875782,0\C,4,1.45088159,3,124.64326283,2
,-177.01777904,0\H,48,1.09940386,4,111.83674589,3,77.03082164,0\H,48,1
.09300237,4,109.66271428,3,-162.65474319,0\H,48,1.09526891,4,110.24866
199,3,-43.70849084,0\C,5,1.49938378,4,122.78764978,3,-178.84507144,0\H
,52,1.09504325,5,109.00745522,4,179.98712627,0\H,52,1.10029784,5,112.7
99085,4,60.75311293,0\H,52,1.10062504,5,112.75569618,4,-60.98137186,0\
\Version=AM64L-G03RevD.01\State=1-A\HF=-1316.8311117\MP2=-1320.6053426
\RMSD=6.425e-09\Thermal=0.\PG=C01 [X(C21H30N3P1)]\\@

213-Me$^+$

1\1\GINC-NAUTILUS\SP\RMP2-FC\6-31+G(2d,p)\C22H33N3P1(1+)\CHRISTOPH\29-
Jan-2010\0\\#p MP2(FC)/6-31+g(2d,p) scf=tight\\xx3mesp_38\\1,1\C\C,1,1
.40043697\N,2,1.37286739,1,106.98142332\C,3,1.3906651,2,110.54809988,1
,0.20866393,0\C,4,1.375371,3,107.4097949,2,-0.37276002,0\C,1,2.9241210
2,2,162.58539671,3,165.02432219,0\C,6,1.3996707,1,112.09480961,2,139.4
5164612,0\N,7,1.37274129,6,107.03870464,1,142.70155938,0\C,8,1.3903927
4,7,110.56936128,6,0.41987806,0\C,9,1.37525891,8,107.43549121,7,-0.543
16584,0\C,1,2.92138674,2,107.04130808,3,-151.974231,0\C,11,1.43654526,
1,90.97364678,2,48.13070524,0\C,12,1.37613095,11,107.73533583,1,-178.9
518887,0\N,13,1.39059877,12,107.40961942,11,0.03582647,0\C,14,1.372508
42,13,110.50649246,12,-0.53189663,0\H,12,1.08179445,11,126.38300863,1,
1.79625169,0\H,10,1.08195626,9,125.01172471,8,-179.29542625,0\H,5,1.08
169103,4,125.98104333,3,-178.77880948,0\C,14,1.4595734,13,125.28497037
,12,-178.76981445,0\H,19,1.09099613,14,109.3294982,13,-16.03190054,0\H
,19,1.0939868,14,110.20767472,13,-135.09358266,0\H,19,1.09665852,14,11
1.04121961,13,103.9515971,0\C,15,1.500356,14,121.60589427,13,-178.5362
8931,0\H,23,1.09961915,15,112.17483257,14,-72.93223155,0\H,23,1.097625
51,15,111.43129266,14,47.3329155,0\H,23,1.09397381,15,110.68919825,14,
166.26162897,0\C,13,1.49898067,12,129.62657195,11,-179.85953261,0\H,27
,1.09827054,13,112.36158317,12,120.47197831,0\H,27,1.09875701,13,112.3
917797,12,-117.84207823,0\H,27,1.0941812,13,109.19113452,12,1.25664233
,0\C,9,1.49885195,8,122.9030815,7,179.55441361,0\H,31,1.09412188,9,109
.25300618,8,-178.92603812,0\H,31,1.0982451,9,112.34000864,8,-59.565963
6,0\H,31,1.09870048,9,112.29762635,8,62.01101891,0\C,8,1.45928031,7,12
4.45732036,6,177.51654406,0\H,35,1.09153835,8,109.40834919,7,159.12057
673,0\H,35,1.09685038,8,111.13096427,7,-80.66961553,0\H,35,1.09316449,
8,110.01628387,7,40.1424984,0\C,7,1.49990768,6,131.19224044,1,-36.9098
4478,0\H,39,1.09288201,7,110.61922724,6,-4.33336266,0\H,39,1.0990359,7
,111.71100436,6,115.53457352,0\H,39,1.09838299,7,111.66306101,6,-124.1

344723,0\C,2,1.50079746,1,131.63224239,5,-178.96530197,0\H,43,1.093510
24,2,110.81260557,1,-10.2167616,0\H,43,1.09811917,2,111.68064924,1,-12
9.28363921,0\H,43,1.09952667,2,111.89585268,1,110.50716303,0\C,3,1.459
33386,2,124.15965066,1,178.55173951,0\H,47,1.09670793,3,111.06096751,2
,-73.88345257,0\H,47,1.09098372,3,109.31011121,2,166.13896691,0\H,47,1
.09407381,3,110.24841973,2,47.08507795,0\C,4,1.49899568,3,123.01513232
,2,179.71136034,0\H,51,1.09414858,4,109.12389017,3,-179.42203255,0\H,5
1,1.0983158,4,112.3943808,3,-60.17746737,0\H,51,1.09868508,4,112.39483
555,3,61.59071099,0\P,6,1.77870384,1,34.77795241,2,15.55186039,0\C,55,
1.83053668,6,106.15862671,1,-117.39051467,0\H,56,1.09451152,55,110.780
23681,6,-59.5709278,0\H,56,1.09487782,55,109.53881921,6,61.44900478,0\
H,56,1.09417317,55,109.03228014,6,-179.50955031,0\\Version=AM64L-G03Re
vD.01\State=1-A\HF=-1356.3153712\MP2=-1360.2405528\RMSD=4.778e-09\Ther
mal=0.\PG=C01 [X(C22H33N3P1)]\\@

214

1\1\GINC-GOLEM\SP\RMP2-FC\6-31+G(2d,p)\C24H36N3P1\CHRISTOPH\07-Jan-201
0\0\\#p Mp2(FC)/6-31+g(2d,p) scf=tight\\zz3sp_24\\0,1\P\C,1,1.82783342
\C,2,1.44845981,1,132.43743642\C,3,1.38299865,2,107.30609692,1,-176.20
209976,0\N,4,1.39168261,3,108.08225749,2,-0.26515551,0\C,5,1.37825939,
4,109.60795352,3,0.99896574,0\C,1,1.83417233,2,104.14907318,6,-138.223
33171,0\C,7,1.4468676,1,119.7218576,2,83.92905915,0\C,8,1.38158153,7,1
07.65972603,1,176.78640007,0\N,9,1.38931347,8,107.76342924,7,-0.035329
69,0\C,10,1.38478692,9,109.89269832,8,-0.32625924,0\C,1,1.83367557,2,1
08.82292627,6,108.71807106,0\C,12,1.39403164,1,133.29340179,2,-4.10699
62,0\N,13,1.38702463,12,107.765334,1,-172.61620972,0\C,14,1.38778728,1
3,109.92724905,12,1.04570238,0\C,15,1.38143374,14,107.80275029,13,-0.4
0681264,0\C,3,1.50436706,2,126.67076239,1,4.36488839,0\H,17,1.09651569
,3,111.48037238,2,172.83412161,0\H,17,1.09695944,3,111.51883804,2,-66.
26009679,0\H,17,1.09804716,3,111.37803256,2,52.87734272,0\C,6,1.500360
27,5,121.60549054,4,178.91586138,0\H,21,1.09953184,6,112.09973839,5,-5
4.07926984,0\H,21,1.10044838,6,112.42183802,5,67.06451738,0\H,21,1.094
22638,6,108.99417623,5,-173.59032378,0\C,5,1.45126296,4,124.84614079,3
,176.23851279,0\H,25,1.09311586,5,109.73748624,4,155.83851082,0\H,25,1
.09955707,5,111.87055993,4,-83.65090486,0\H,25,1.0941598,5,110.0289543
,4,36.96284151,0\C,4,1.50012327,3,130.78056998,2,179.4323869,0\H,29,1.
10019635,4,112.40330468,3,-119.92252127,0\H,29,1.09390525,4,110.026852
34,3,-0.53934505,0\H,29,1.10069437,4,112.56847336,3,118.92104167,0\C,1
6,1.50500523,15,126.7366852,14,-179.70908142,0\H,33,1.09651965,16,111.
72059417,15,-11.33631458,0\H,33,1.0989048,16,112.05485417,15,109.67918
323,0\H,33,1.09858978,16,110.83958059,15,-131.46032203,0\C,15,1.500002
91,14,121.721073,13,179.76988806,0\H,37,1.09413197,15,109.81459754,14,
-179.15718997,0\H,37,1.10020762,15,112.602868,14,-59.69065212,0\H,37,1
.10079884,15,112.58250931,14,61.673989,0\C,13,1.5008713,12,132.4424287
9,1,5.7919296,0\H,41,1.10122068,13,112.1311862,12,118.54289239,0\H,41,
1.10060226,13,111.7516797,12,-121.29400062,0\H,41,1.09088243,13,110.25
899591,12,-1.48076153,0\C,14,1.45046554,13,124.85485037,12,174.8963345
6,0\H,45,1.09340102,14,109.72935552,13,156.62571554,0\H,45,1.10007156,
14,111.97036379,13,-82.93260318,0\H,45,1.09398266,14,110.15574474,13,3
7.7633714,0\C,8,1.50511785,7,125.62443241,1,-4.21391219,0\H,49,1.09657
59,8,111.69741369,7,172.14240588,0\H,49,1.09933357,8,111.75570905,7,-6
7.29925955,0\H,49,1.09818599,8,111.02232844,7,51.73057611,0\C,9,1.4998
2645,8,130.64438886,7,179.96661059,0\H,53,1.10090469,9,112.63361341,8,
-116.85610869,0\H,53,1.09425372,9,109.84142459,8,2.37122356,0\H,53,1.1
002006,9,112.52925569,8,121.80741157,0\C,10,1.45060883,9,124.79179289,
8,-174.79062268,0\H,57,1.10001593,10,111.97259128,9,87.32235621,0\H,57
,1.09359762,10,110.01829287,9,-152.07185389,0\H,57,1.09381147,10,109.8
5213197,9,-33.21030479,0\C,11,1.50044219,10,120.41367452,9,179.463565,

0\H,61,1.10024165,11,112.30182165,10,59.14718454,0\H,61,1.09129078,11,
109.65107996,10,178.8322507,0\H,61,1.10122583,11,111.80594057,10,-61.4
9921184,0\\Version=AM64L-G03RevD.01\State=1-A\HF=-1433.9313855\MP2=-14
38.1937338\RMSD=6.798e-09\Thermal=0.\PG=C01 [X(C24H36N3P1)]\\@

214-Me$^+$

1\1\GINC-YIN\SP\RMP2-FC\6-31+G(2d,p)\C25H39N3P1(1+)\CHRISTOPH\15-Jan-2
010\0\\#p MP2(FC)/6-31+g(2d,p) scf=tight\\zz3mesp_20_3\\1,1\C\C,1,1.45
109395\C,2,1.38006457,1,106.61183349\N,3,1.39042259,2,108.16156073,1,0
.06043096,0\C,4,1.37074977,3,110.65378853,2,-0.68819644,0\C,1,2.972428
25,5,95.24073279,4,169.61671749,0\C,6,1.45140995,1,107.19843875,5,-66.
2008065,0\C,7,1.3794396,6,106.4966314,1,147.8327101,0\N,8,1.38973877,7
,108.28676318,6,-0.25449731,0\C,9,1.37101091,8,110.66708599,7,-0.27458
195,0\C,1,2.97483077,5,132.86191249,4,-137.11382518,0\C,11,1.40438614,
1,95.46341063,5,69.7999287,0\N,12,1.3709374,11,106.85728812,1,169.5727
4265,0\C,13,1.38996467,12,110.6630571,11,1.05921896,0\C,14,1.37957427,
13,108.2355348,12,-0.55721398,0\C,2,1.5075197,1,127.73160493,5,-178.68
080128,0\H,16,1.09862808,2,112.43631239,1,-80.94498679,0\H,16,1.097387
62,2,111.67063827,1,40.18293908,0\H,16,1.09490842,2,110.95362915,1,159
.4261893,0\C,5,1.5020404,4,120.14393896,3,-177.59644866,0\H,20,1.08988
156,5,110.94570656,4,169.60295185,0\H,20,1.09935011,5,111.78888413,4,-
70.45458072,0\H,20,1.09786846,5,110.93108178,4,49.45341779,0\C,4,1.459
35176,3,124.64009641,2,-178.75216304,0\H,24,1.09251932,4,110.01878052,
3,-146.08808411,0\H,24,1.09156345,4,109.4251085,3,-27.31426059,0\H,24,
1.09725415,4,111.31304819,3,92.97630211,0\C,3,1.4998514,2,130.29709286
,1,179.62587339,0\H,28,1.09914816,3,112.15417611,2,-114.00483626,0\H,2
8,1.09322834,3,110.16171018,2,5.08355114,0\H,28,1.09818336,3,112.16607
958,2,124.74948012,0\C,15,1.50732736,14,125.61210798,13,179.20450606,0
\H,32,1.09856201,15,112.43207088,14,100.32596057,0\H,32,1.09747167,15,
111.7151655,14,-138.52374535,0\H,32,1.0949159,15,110.90302875,14,-19.2
7023904,0\C,14,1.49942626,13,121.16443836,12,179.88146819,0\H,36,1.093
27079,14,110.32353875,13,-175.52967669,0\H,36,1.0983296,14,112.0816434
7,13,-55.70183945,0\H,36,1.09914784,14,112.06433564,13,65.22971774,0\C
,12,1.50238134,11,132.52222034,1,-12.08091416,0\H,40,1.09781973,12,111
.09377706,11,-126.99875183,0\H,40,1.09910666,12,111.84971032,11,112.74
840806,0\H,40,1.08980665,12,110.7037629,11,-7.02485113,0\C,13,1.459111
21,12,125.60666739,11,179.62070624,0\H,44,1.09339667,13,109.72716777,1
2,138.7388819,0\H,44,1.09081559,13,109.87675882,12,19.79610435,0\H,44,
1.09693741,13,111.11138448,12,-100.85638501,0\C,7,1.5074708,6,127.9380
5218,1,-31.37639543,0\H,48,1.09493911,7,110.87897274,6,159.74767103,0\
H,48,1.09859899,7,112.43603993,6,-80.67373418,0\H,48,1.09743567,7,111.
7351223,6,40.50249185,0\C,8,1.49935271,7,130.68544214,6,179.1415655,0\
H,52,1.09323521,8,110.34545522,7,4.57123659,0\H,52,1.09848522,8,112.11
426813,7,124.43488255,0\H,52,1.09909797,8,112.00987513,7,-114.75729767
,0\C,9,1.45875454,8,123.26014836,7,179.66281989,0\H,56,1.09609181,9,11
0.74528614,8,66.94512796,0\H,56,1.09493733,9,110.11606046,8,-53.484550
95,0\H,56,1.09019743,9,109.79856381,8,-172.83285872,0\C,10,1.50239518,
9,120.78296136,8,-177.89428457,0\H,60,1.09779477,10,111.18037061,9,51.
96906844,0\H,60,1.09888628,10,111.88186206,9,-68.45237096,0\H,60,1.089
79181,10,110.63273313,9,171.77453733,0\P,1,1.78928899,5,128.14354213,4
,178.37491471,0\C,64,1.84114168,1,106.29338047,5,-131.71835567,0\H,65,
1.0930456,64,109.7039778,1,157.99285719,0\H,65,1.09281693,64,109.65631
975,1,37.93830132,0\H,65,1.09304839,64,109.61413507,1,-82.01369131,0\\
Version=AM64L-G03RevD.01\State=1-A\HF=-1473.418964\MP2=-1477.8304797\R
MSD=5.126e-09\Thermal=0.\PG=C01 [X(C25H39N3P1)]\\@

215

1\1\GINC-CIPCLU09\SP\RMP2-FC\6-31+G(2d,p)\C9H9P1\C4371\28-Oct-2009\0\\
#P MP2(FC)/6-31+G(2d,p) scf=tight\\d3sp_88\\0,1\P\C,1,1.79174686\C,2,1
.30486533,1,151.82153109\C,3,1.50675865,2,65.73741889,1,163.77775832,0
\H,4,1.09414054,3,120.21095132,2,103.72628406,0\H,4,1.0952705,3,119.92
803193,2,-105.45770578,0\C,1,1.79113306,2,102.0202759,3,145.63864762,0
\C,7,1.53579463,1,143.53899807,2,57.59936792,0\C,7,1.30508066,1,151.61
105861,2,-145.20857255,0\H,8,1.09412175,7,118.49140955,1,-84.68469893,
0\H,8,1.09509995,7,119.58984983,1,62.02317058,0\C,1,1.79081053,7,103.2
4022744,9,-39.0712151,0\C,12,1.53657191,1,143.53313585,7,-162.92636353
,0\C,12,1.30492217,1,151.8894167,7,37.78001581,0\H,13,1.09505966,12,11
9.62708888,1,-63.20130322,0\H,13,1.09421568,12,118.54217553,1,83.73760
003,0\H,3,1.08009153,2,148.25108242,1,-11.75776059,0\H,9,1.08017303,7,
148.35117742,1,11.13356948,0\H,14,1.08016391,12,148.37503449,1,-10.030
92779,0\\Version=AM64L-G03RevD.01\State=1-A\HF=-686.5155569\MP2=-687.9
090433\RMSD=7.135e-09\Thermal=0.\PG=C01 [X(C9H9P1)]\\@

215-Me⁺

1\1\GINC-CIPCLU08\SP\RMP2-FC\6-31+G(2d,p)\C10H12P1(1+)\C4371\28-Oct-20
09\0\\#P MP2(FC)/6-31+G(2d,p) scf=tight\\d3mesp_82\\1,1\C\C,1,1.307906
86\C,2,1.50655812,1,65.78442563\H,3,1.09105129,2,119.08807993,1,105.34
917297,0\H,3,1.09089507,2,119.1140232,1,-104.97814325,0\H,2,1.08177776
,1,148.40802079,3,178.9278235,0\P,1,1.75873174,2,148.19335072,3,-177.6
3481305,0\C,7,1.81644054,1,109.57091036,2,-5.8671265,0\H,8,1.09523673,
7,109.27730061,1,-176.45286423,0\H,8,1.09504284,7,109.55214641,1,-56.4
1534804,0\H,8,1.09527225,7,109.28148931,1,63.84784593,0\C,7,1.76039242
,1,109.41065287,2,113.0711159,0\C,12,1.30632841,7,149.20523409,1,108.1
8450715,0\C,13,1.50641244,12,66.03422775,7,-178.1221064,0\H,14,1.09110
516,13,119.20609437,12,-105.04123061,0\H,14,1.09108498,13,119.05965616
,12,105.11304406,0\C,7,1.75727397,1,109.93282136,2,-126.67781599,0\C,1
7,1.30655864,7,149.07857269,1,-6.28018099,0\C,18,1.50563864,17,66.0556
3746,7,-179.76163732,0\H,19,1.09103094,18,119.16599515,17,105.05946509
,0\H,19,1.09109288,18,119.17801807,17,-105.03913593,0\H,13,1.08161455,
12,148.25567185,7,1.60198672,0\H,18,1.08157414,17,148.20230699,7,0.149
16775,0\\Version=AM64L-G03RevD.01\State=1-A\HF=-725.9445847\MP2=-727.4
805985\RMSD=3.410e-09\Thermal=0.\PG=C01 [X(C10H12P1)]\\@

216

1\1\GINC-CALYPSO\SP\RMP2-FC\6-31+G(2d,p)\C7H9P1\CHRISTOPH\24-Oct-2009\
0\\#p MP2(FC)/6-31+g(2d,p) scf=tight\\d2sp_1\\0,1\P\C,1,1.79286838\C,2
,1.30663391,1,151.22233645\C,3,1.50586452,2,65.72957122,1,-165.6345370
9,0\H,4,1.0943335,3,120.23446382,2,-103.85761729,0\H,4,1.09530324,3,11
9.96654484,2,105.36813289,0\C,1,1.79286559,2,100.91570807,3,-143.39065
332,0\C,7,1.53538846,1,144.04098956,2,-58.76732567,0\C,7,1.30663349,1,
151.22911069,2,143.46108728,0\H,8,1.09530165,7,119.55523945,1,-62.0523
1206,0\H,8,1.09433472,7,118.57189735,1,84.52206881,0\C,1,1.86323061,7,
101.74526602,9,38.88040169,0\H,3,1.08046112,2,148.41389491,1,10.087399
14,0\H,9,1.08046129,7,148.41291498,1,-10.13837417,0\H,12,1.09597801,1,
108.81089013,7,-69.22413048,0\H,12,1.09475666,1,112.27844166,7,51.9750
3351,0\H,12,1.09597906,1,108.80949948,7,173.17295868,0\\Version=AM64L-
G03RevD.01\State=1-A\HF=-610.8760968\MP2=-612.0048639\RMSD=5.274e-09\T
hermal=0.\PG=C01 [X(C7H9P1)]\\@

216-Me⁺

1\1\GINC-EDDY\SP\RMP2-FC\6-31+G(2d,p)\C8H12P1(1+)\CHRISTOPH\27-Oct-200
9\0\\#p MP2(FC)/6-31+g(2d,p) scf=tight\\d2mesp_14\\1,1\C\C,1,1.3076528
6\C,2,1.5059115,1,65.93340918\H,3,1.09094616,2,119.16743158,1,105.0238
7071,0\H,3,1.09101369,2,119.00365124,1,-105.28069141,0\H,2,1.08185227,
1,148.35078558,3,-179.28082831,0\P,1,1.75847089,2,148.92651529,3,178.0

9563301,0\C,7,1.81680136,1,109.18357189,2,128.86901892,0\H,8,1.095568,
7,109.64179731,1,-61.91586556,0\H,8,1.09552964,7,109.67236349,1,177.84
134045,0\H,8,1.09505666,7,109.46147271,1,57.82654891,0\C,7,1.75844533,
1,109.30986152,2,-111.60461098,0\C,12,1.30765452,7,148.92606082,1,-111
.58687265,0\C,13,1.50592559,12,65.93415017,7,178.06379333,0\H,14,1.091
01171,13,118.99976426,12,-105.28057788,0\H,14,1.09094149,13,119.170658
8,12,105.01687738,0\C,7,1.81681355,1,109.30064839,2,7.84873004,0\H,13,
1.0818521,12,148.34946156,7,-1.20687978,0\H,17,1.09553261,7,109.667530
55,1,58.29656493,0\H,17,1.09505786,7,109.46260607,1,-61.7140451,0\H,17
,1.09556528,7,109.64550656,1,178.53949069,0\\Version=AM64L-G03RevD.01\
State=1-A\HF=-650.3092252\MP2=-651.5807864\RMSD=5.008e-09\Thermal=0.\P
G=C01 [X(C8H12P1)]\\@

217

1\1\GINC-CIPCLU10\SP\RMP2-FC\6-31+G(2d,p)\C5H8N1P1S1\C2175\22-Dec-2009
\0\\#p MP2(FC)/6-31+G(2d,p) scf=tight\\ns51sp_5\\0,1\P\C,1,1.82763798\
S,2,1.76192077,1,127.49972776\C,3,1.74898042,2,89.06760557,1,179.99813
703,0\N,4,1.30214201,3,115.51582002,2,0.,0\C,5,1.37634009,4,110.072882
6,3,0.00042823,0\C,1,1.86538876,2,101.22733235,6,-128.81279419,0\H,7,1
.09575664,1,109.33075614,2,67.80448997,0\H,7,1.09743669,1,113.27176327
,2,-53.70552882,0\H,7,1.09716251,1,108.72673496,2,-174.60995452,0\C,1,
1.86539287,2,101.22685123,6,128.81482547,0\H,11,1.09575623,1,109.32963
053,2,-67.79630728,0\H,11,1.09716233,1,108.7268166,2,174.61877948,0\H,
11,1.097434,1,113.27195515,2,53.71355306,0\H,4,1.08560093,3,120.655979
22,2,179.99847841,0\H,6,1.08569438,5,118.86113813,4,179.9996576,0\\Ver
sion=AM64L-G03RevD.01\State=1-A\HF=-986.6992737\MP2=-987.8773133\RMSD=
2.721e-09\Thermal=0.\PG=C01 [X(C5H8N1P1S1)]\\@

217-Me^{+}

1\1\GINC-CIPCLU05\SP\RMP2-FC\6-31+G(2d,p)\C6H11N1P1S1(1+)\C2175\22-Dec
-2009\0\\#p MP2(FC)/6-31+G(2d,p) scf=tight\\ns51mesp_2\\1,1\C\S,1,1.75
206874\C,2,1.75039639,1,88.11237718\N,3,1.30242777,2,115.75272235,1,-0
.00214713,0\C,4,1.36425067,3,110.84396096,2,0.00387511,0\C,1,2.9576808
3,5,136.12683352,4,133.78935766,0\H,6,1.09535001,1,90.4360493,5,-33.81
920968,0\H,6,1.09626411,1,144.15332106,5,91.47690223,0\H,6,1.09576007,
1,91.27433994,5,-142.70133269,0\C,6,2.95740321,1,60.00209271,5,126.998
89771,0\H,10,1.09626363,6,89.56329277,1,-160.11644731,0\H,10,1.0953516
2,6,145.26136053,1,-35.22796731,0\H,10,1.09576077,6,90.90213769,1,90.9
4518732,0\H,3,1.08561826,2,120.00659645,1,-179.99688763,0\H,5,1.085483
77,4,118.64249566,3,-180.,0\P,1,1.77966017,5,127.14008559,4,179.991031
48,0\C,16,1.81692534,1,108.69388776,5,0.05868753,0\H,17,1.09609992,16,
108.84945009,1,179.99483411,0\H,17,1.09514394,16,110.38434967,1,-60.63
638948,0\H,17,1.09514214,16,110.38105757,1,60.62600308,0\\Version=AM64
L-G03RevD.01\State=1-A\HF=-1026.1318517\MP2=-1027.4531662\RMSD=9.340e-
09\Thermal=0.\PG=C01 [X(C6H11N1P1S1)]\\@

218

1\1\GINC-AZAZEL\SP\RMP2-FC\6-31+G(2d,p)\C12H9O3P1\CHRISTOPH\12-Sep-200
9\0\\#p MP2(FC)/6-31+g(2d,p) scf=tight\\fu3sp_37\\0,1\P\C,1,1.8279427\
C,2,1.37310208,1,136.79044082\C,3,1.43156789,2,106.82094592,1,178.7243
7605,0\C,4,1.36568708,3,105.83662554,2,0.09449924,0\O,5,1.35354825,4,1
10.76105959,3,-0.28257787,0\H,3,1.08118847,2,125.70994796,1,-1.2185809
3,0\H,4,1.08219181,3,127.52599161,2,-179.86820884,0\H,5,1.08082176,4,1
33.4911993,3,179.81429232,0\C,1,1.82796545,2,102.88441939,3,72.8515999
7,0\C,10,1.37310705,1,136.74601716,2,-71.95498202,0\C,11,1.43164905,10
,106.80684348,1,-178.82372539,0\C,12,1.36571102,11,105.83959792,10,-0.
12339109,0\O,13,1.35360278,12,110.7596138,11,0.29820792,0\H,11,1.08133
763,10,125.68212705,1,1.05625927,0\H,12,1.08217875,11,127.53019657,10,

179.85719008,0\H,13,1.08081402,12,133.4915217,11,-179.80859574,0\C,1,1
.81519623,2,101.14881014,3,-31.4511474,0\C,18,1.37132503,1,128.6058182
4,2,-126.50820169,0\C,19,1.43345757,18,107.05285908,1,179.72429305,0\C
,20,1.36352383,19,105.70997359,18,0.01173059,0\O,21,1.35803466,20,110.
85997333,19,-0.02902074,0\H,19,1.08217243,18,125.52986496,1,-0.3634161
,0\H,20,1.08182793,19,127.63513997,18,-179.98696363,0\H,21,1.0809909,2
0,133.42620402,19,179.91357852,0\\Version=AM64L-G03RevD.01\State=1-A\H
F=-1024.9183089\MP2=-1027.3318243\RMSD=6.602e-09\Thermal=0.\PG=C01 [X(
C12H9O3P1)]\\@

218-Me$^+$

1\1\GINC-AZAZEL\SP\RMP2-FC\6-31+G(2d,p)\C13H12O3P1(1+)\CHRISTOPH\12-Se
p-2009\0\\#p MP2(FC)/6-31+g(2d,p) scf=tight\\fu3mesp_6\\1,1\P\C,1,1.81
668793\H,2,1.09509213,1,109.14541327\H,2,1.0951041,1,109.14173985,3,11
9.98290749,0\H,2,1.09508909,1,109.15199118,4,120.0034227,0\C,1,1.77661
476,2,109.89359093,5,71.45431899,0\C,6,1.37550989,1,132.5426947,2,-134
.07958112,0\C,7,1.42716774,6,105.95182172,1,179.74011888,0\C,8,1.36809
601,7,106.11867322,6,-0.03543747,0\O,9,1.35396973,8,111.04838774,7,0.0
539586,0\H,7,1.08227231,6,126.59615026,1,0.08174513,0\H,8,1.08140072,7
,127.23274464,6,-179.6657059,0\H,9,1.08097488,8,133.40239113,7,-179.67
709559,0\C,1,1.77645745,6,109.0166478,10,166.18638902,0\C,14,1.3754172
3,1,132.59143462,6,105.394352,0\C,15,1.42721466,14,105.95747812,1,179.
91993032,0\C,16,1.36812144,15,106.1189558,14,-0.03755098,0\O,17,1.3538
8403,16,111.04490126,15,0.06742299,0\H,15,1.08224514,14,126.56297363,1
,0.27417029,0\H,16,1.08139912,15,127.23447486,14,-179.65003654,0\H,17,
1.08099183,16,133.39911136,15,-179.659643,0\C,1,1.77651498,14,109.0042
5917,18,166.37921184,0\C,22,1.37544512,1,132.62634238,14,106.07198436,
0\C,23,1.42716435,22,105.95852645,1,179.65412153,0\C,24,1.36805987,23,
106.11932834,22,-0.11566575,0\O,25,1.35395677,24,111.05082038,23,0.093
93873,0\H,23,1.08225746,22,126.58853404,1,-0.06363761,0\H,24,1.0814034
4,23,127.22979087,22,-179.71282157,0\H,25,1.08097972,24,133.39759552,2
3,-179.65885432,0\\Version=AM64L-G03RevD.01\State=1-A\HF=-1064.3589471
\MP2=-1066.91283\RMSD=2.025e-09\Thermal=0.\PG=C01 [X(C13H12O3P1)]\\@

219

1\1\GINC-GOLEM\SP\RMP2-FC\6-31+G(2d,p)\C9H9O2P1\CHRISTOPH\09-Sep-2009\
0\\#p MP2(FC)/6-31+g(2d,p) scf=tight\\fu2sp_2\\0,1\P\C,1,1.86476913\H,
2,1.09602359,1,108.77599079\H,2,1.09602405,1,108.77790069,3,117.887251
21,0\H,2,1.0943572,1,112.3576983,3,-121.05594171,0\C,1,1.82077308,2,10
0.39904538,5,52.48824702,0\C,6,1.3726951,1,129.64230493,2,135.33146017
,0\C,7,1.43312192,6,106.98512335,1,-179.56950309,0\C,8,1.36484318,7,10
5.6208507,6,0.12265268,0\O,9,1.35534245,8,110.93313656,7,-0.04724696,0
\H,7,1.08231436,6,125.591368,1,0.45095002,0\H,8,1.08192646,7,127.71735
708,6,-179.9784212,0\H,9,1.08109014,8,133.36374262,7,179.83814636,0\C,
1,1.82077865,6,102.53134229,7,-121.44046046,0\C,14,1.37269472,1,129.64
118192,6,121.53827731,0\C,15,1.43312414,14,106.98511098,1,179.56371372
,0\C,16,1.36484111,15,105.62087796,14,-0.11986828,0\O,17,1.35534653,16
,110.93306475,15,0.04487138,0\H,15,1.0823134,14,125.59131684,1,-0.4578
1463,0\H,16,1.08192822,15,127.71740073,14,179.9779043,0\H,17,1.0810897
7,16,133.36385027,15,-179.84405691,0\\Version=AM64L-G03RevD.01\State=1
-A\HF=-836.481876\MP2=-838.2885192\RMSD=6.627e-09\Thermal=0.\PG=C01 [X
(C9H9O2P1)]\\@

219-Me$^+$

1\1\GINC-AZAZEL\SP\RMP2-FC\6-31+G(2d,p)\C10H12O2P1(1+)\CHRISTOPH\09-Se
p-2009\0\\#p MP2(FC)/6-31+g(2d,p) scf=tight\\fu2mesp_4\\1,1\P\C,1,1.81
821359\H,2,1.09470867,1,110.01404767\H,2,1.09575078,1,108.54074646,3,-
118.78185223,0\H,2,1.09549763,1,110.30867292,4,-119.67561471,0\C,1,1.8

1823946,2,109.19905415,3,-179.40194003,0\H,6,1.09583421,1,109.38106038
,2,-68.12915349,0\H,6,1.09498126,1,109.09230377,2,172.53344968,0\H,6,1
.09532481,1,109.87352661,2,52.45881681,0\C,1,1.77398324,2,107.97860702
,6,120.70560508,0\C,10,1.37621524,1,133.11984068,2,6.62790248,0\C,11,1
.4270673,10,106.01759029,1,-178.9664475,0\C,12,1.3677335,11,106.100827
03,10,0.01879589,0\O,13,1.35390984,12,111.03204935,11,-0.0404184,0\H,1
1,1.08247596,10,127.10529467,1,0.97399774,0\H,12,1.08137971,11,127.170
89763,10,179.86338485,0\H,13,1.08094931,12,133.40781263,11,179.8063943
5,0\C,1,1.7776402,10,108.83375887,14,67.0473739,0\C,18,1.37459458,1,13
3.31824644,10,8.92634048,0\C,19,1.42785184,18,105.92136359,1,179.37081
004,0\C,20,1.36723398,19,106.20452542,18,-0.00779864,0\O,21,1.35566701
,20,110.98518725,19,0.04721488,0\H,19,1.08215383,18,126.70863221,1,-0.
95641714,0\H,20,1.0813972,19,127.16685318,18,179.76178014,0\H,21,1.080
89116,20,133.45799957,19,179.90077302,0\\Version=AM64L-G03RevD.01\Stat
e=1-A\HF=-875.9174931\MP2=-877.8670561\RMSD=4.350e-09\Thermal=0.\PG=C0
1 [X(C10H12O2P1)]\\@

220

1\1\GINC-NAUTILUS\SP\RMP2-FC\6-31+G(2d,p)\C5H9P1\CHRISTOPH\24-Oct-2009
\0\\#p MP2(FC)/6-31+g(2d,p) scf=tight\\d1sp_8\\0,1\P\C,1,1.79429001\C,
2,1.30694181,1,152.61984604\C,3,1.50477818,2,65.96353737,1,-166.624047
99,0\H,4,1.09560968,3,119.99144581,2,105.37421178,0\H,4,1.09505279,3,1
20.18942367,2,-104.24488377,0\C,1,1.86445072,2,100.47519295,3,-36.2604
072,0\C,1,1.86581995,2,99.93952804,3,-139.34987487,0\H,3,1.08062782,2,
148.37927041,1,8.85446661,0\H,8,1.09654708,1,108.90409852,2,177.234328
57,0\H,8,1.095869,1,109.05371584,2,-65.1622022,0\H,8,1.0966207,1,112.8
4709422,2,55.98482544,0\H,7,1.09663142,1,112.54352391,2,-52.11055458,0
\H,7,1.09606934,1,109.19464753,2,68.92999352,0\H,7,1.0967647,1,109.163
49837,2,-173.4182453,0\\Version=AM64L-G03RevD.01\State=1-A\HF=-535.234
3169\MP2=-536.0987086\RMSD=3.686e-09\Thermal=0.\PG=C01 [X(C5H9P1)]\\@

220-Me$^+$

1\1\GINC-SOLARIS\SP\RMP2-FC\6-31+G(2d,p)\C6H12P1(1+)\CHRISTOPH\26-Oct-
2009\0\\#p MP2(FC)/6-31+g(2d,p) scf=tight\\d1mesp_3\\1,1\C\P,1,1.81804
985\C,2,1.8180302,1,108.90225522\H,3,1.09526849,2,109.85886449,1,-178.
55242106,0\H,3,1.09547589,2,109.67977042,1,61.62673754,0\H,3,1.0961459
9,2,109.82339753,1,-58.3360888,0\C,2,1.75633743,1,109.17751858,3,119.1
3548799,0\C,7,1.30743941,2,149.20303433,1,120.50661261,0\C,8,1.5044729
7,7,66.13258234,2,-179.97868179,0\H,9,1.09099679,8,119.0929525,7,-105.
11973538,0\H,9,1.0909899,8,119.10388623,7,105.12202769,0\C,2,1.8166529
6,1,109.82727446,7,120.55926001,0\H,8,1.08189202,7,148.22894449,2,0.01
581903,0\H,12,1.09605107,2,109.77819702,1,59.89426309,0\H,12,1.0953141
1,2,109.82867003,1,-60.0862096,0\H,12,1.09531649,2,109.82937886,1,179.
87478141,0\H,1,1.09614679,2,109.829513,7,177.44325177,0\H,1,1.09547699
,2,109.67991326,7,57.47984624,0\H,1,1.09526623,2,109.85330622,7,-62.33
905096,0\\Version=AM64L-G03RevD.01\State=1-A\HF=-574.6726038\MP2=-575.
6801667\RMSD=2.641e-09\Thermal=0.\PG=C01 [X(C6H12P1)]\\@

221

1\1\GINC-MORITZ\SP\RMP2-FC\6-31+G(2d,p)\C5H8N1P1S1\CHRISTOPH\05-Jan-20
10\0\\#p MP2(FC)/6-31+g(2d,p) scf=tight\\ns531sp_2\\0,1\P\C,1,1.864733
59\H,2,1.09709111,1,108.8248277\H,2,1.09584402,1,109.56329416,3,-117.8
8229947,0\H,2,1.09565497,1,111.69708375,3,121.09617832,0\C,1,1.8650433
5,2,99.51449145,5,52.98788202,0\H,6,1.09710903,1,108.82131615,2,68.643
53242,0\H,6,1.09586409,1,109.54560141,2,-173.50021242,0\H,6,1.09576642
,1,111.72953638,2,-52.45357202,0\C,1,1.8519294,2,98.4031531,6,-100.046
13924,0\N,10,1.30854298,1,126.3729079,2,49.73166763,0\C,11,1.37431201,
10,111.62340033,1,179.94346405,0\C,12,1.3700028,11,116.01822476,10,-0.

01223899,0\S,13,1.7313377,12,109.65665028,11,0.02180824,0\H,13,1.08248
445,12,128.38391943,11,-179.99695395,0\H,12,1.08545252,11,119.0978203,
10,179.99113131,0\\Version=IA32L-G03RevD.01\State=1-A\HF=-986.6969497\
MP2=-987.8775474\RMSD=8.088e-09\Thermal=0.\PG=C01 [X(C5H8N1P1S1)]\\@

221-Me+

1\1\GINC-NAUTILUS\SP\RMP2-FC\6-31+G(2d,p)\C6H11N1P1S1(1+)\CHRISTOPH\04
-Jan-2010\0\\#p MP2(FC)/6-31+g(2d,p) scf=tight\\ns531mesp_9\\1,1\C\H,1
,1.09535946\H,1,1.09552402,2,109.01385346\H,1,1.09612102,2,109.4391342
9,3,-119.794069,0\C,1,2.97758827,2,89.10190474,3,147.82854826,0\H,5,1.
09599216,1,90.12413172,2,106.83696384,0\H,5,1.09572722,1,90.75836382,2
,-1.85038389,0\H,5,1.09560128,1,145.13660068,2,-128.56557587,0\C,1,2.9
4269921,5,59.8911869,8,-35.7008955,0\N,9,1.31149427,1,101.1193768,5,15
8.84521892,0\C,10,1.36789964,9,110.77851448,1,145.26461667,0\C,11,1.37
476339,10,115.37177615,9,0.00640083,0\S,12,1.72640758,11,110.64228121,
10,-0.11408869,0\H,12,1.08327768,11,128.54285818,10,179.76170193,0\H,1
1,1.08412717,10,119.51616415,9,179.8109848,0\P,9,1.79869886,1,35.73142
81,5,34.83094499,0\C,16,1.81481654,9,108.21864935,1,118.95616886,0\H,1
7,1.09596158,16,109.9483165,9,-176.31653126,0\H,17,1.09523973,16,109.7
6697453,9,63.11555115,0\H,17,1.09527283,16,108.86677438,9,-56.03437385
,0\\Version=AM64L-G03RevD.01\State=1-A\HF=-1026.1312277\MP2=-1027.4588
955\RMSD=6.731e-09\Thermal=0.\PG=C01 [X(C6H11N1P1S1)]\\@

222

1\1\GINC-NODE16\SP\RMP2-FC\6-31+G(2d,p)\C6H9O1P1\ZIP07\08-Sep-2009\0\\
#p MP2(FC)/6-31+g(2d,p) scf=tight\\fu1sp_1\\0,1\P\C,1,1.86726432\H,2,1
.09591916,1,109.43349315\H,2,1.09709764,1,108.88717377,3,-117.77334312
,0\H,2,1.09637049,1,112.57644573,4,-120.98616046,0\C,1,1.86705214,2,99
.29241334,3,-173.03864572,0\H,6,1.09631367,1,112.53939976,2,52.5333172
,0\H,6,1.09709712,1,108.88599901,2,-68.43826333,0\H,6,1.09592178,1,109
.4468457,2,173.7793541,0\C,1,1.8211144,6,100.35860486,2,-102.42244195,
0\C,10,1.37178853,1,130.49052673,6,-129.8657179,0\C,11,1.43404805,10,1
07.12583187,1,-179.92974015,0\C,12,1.36417807,11,105.64784773,10,0.004
35539,0\O,13,1.35664403,12,110.88168765,11,-0.00518512,0\H,11,1.082322
92,10,125.50808557,1,0.07448807,0\H,12,1.0819727,11,127.70906618,10,-1
79.99431885,0\H,13,1.08116725,12,133.3669846,11,-179.97072444,0\\Versi
on=AM64L-G03RevD.01\State=1-A\HF=-648.0374911\MP2=-649.240462\RMSD=5.6
06e-09\Thermal=0.\PG=C01 [X(C6H9O1P1)]\\@

222-Me+

1\1\GINC-NODE14\SP\RMP2-FC\6-31+G(2d,p)\C7H12O1P1(1+)\ZIP07\08-Sep-200
9\0\\#p MP2(FC)/6-31+g(2d,p) scf=tight\\fu1mesp_2\\1,1\P\C,1,1.8184313
4\H,2,1.09521301,1,109.71593256\H,2,1.09564463,1,109.41280006,3,119.39
680364,0\H,2,1.09612418,1,110.13072402,4,120.00235328,0\C,1,1.81844078
,2,108.62708287,3,177.08904031,0\H,6,1.09612361,1,110.13247849,2,-56.4
9777527,0\H,6,1.09564829,1,109.41098335,2,63.5049157,0\H,6,1.09521404,
1,109.71508475,2,-177.10035792,0\C,1,1.81509701,2,109.90620895,6,-120.
27860623,0\H,10,1.09578539,1,109.12632408,2,59.81231141,0\H,10,1.09515
098,1,110.21486094,2,179.33131471,0\H,10,1.09514481,1,110.20896844,2,-
59.70875671,0\C,1,1.77565681,10,107.88355799,2,120.24451094,0\C,14,1.3
7564919,1,133.88062123,10,0.06451822,0\C,15,1.42776693,14,105.98009363
,1,179.99255959,0\C,16,1.36709274,15,106.17490028,14,0.00174991,0\O,17
,1.35527915,16,110.96963336,15,-0.00126991,0\H,15,1.08255272,14,127.16
363901,1,-0.00687226,0\H,16,1.08138978,15,127.12147516,14,179.99965434
,0\H,17,1.08087461,16,133.46157144,15,179.99848134,0\\Version=AM64L-G0
3RevD.01\State=1-A\HF=-687.4764156\MP2=-688.8228129\RMSD=9.886e-09\The
rmal=0.\PG=C01 [X(C7H12O1P1)]\\@

223

1\1\GINC-YANG\SP\RMP2-FC\6-31+G(2d,p)\C9H9P1S2\CHRISTOPH\14-Sep-2009\0
\\#p MP2(FC)/6-31+g(2d,p) scf=tight\\thio2sp_30\\0,1\P\C,1,1.86401578\
H,2,1.09668882,1,113.87749275\H,2,1.0960961,1,108.60392606,3,-121.1395
6127,0\H,2,1.09609877,1,108.60377699,4,-117.72103834,0\C,1,1.82853729,
2,101.42251395,4,-175.01022721,0\C,6,1.37926943,1,123.45334661,2,-136.
16468081,0\C,7,1.42884965,6,114.12833513,1,179.16319138,0\C,8,1.370311
58,7,112.45521334,6,-0.19531583,0\S,9,1.73531757,8,111.73306332,7,0.14
607715,0\H,7,1.08625239,6,122.10951896,1,-0.6870616,0\H,8,1.08561132,7
,124.02428975,6,179.98689208,0\H,9,1.08354501,8,128.18290687,7,-179.79
68308,0\C,1,1.82857281,6,104.69224836,7,118.65587869,0\C,14,1.37926859
,1,123.45498792,6,-118.69330799,0\C,15,1.42884273,14,114.12794302,1,-1
79.1656932,0\C,16,1.37031578,15,112.45543827,14,0.19442077,0\S,17,1.73
531557,16,111.73243379,15,-0.1448623,0\H,15,1.08625399,14,122.11074381
,1,0.68586017,0\H,16,1.08561271,15,124.02376828,14,-179.98634684,0\H,1
7,1.08354442,16,128.18361249,15,179.80012319,0\\Version=AM64L-G03RevD.
01\State=1-A\HF=-1481.8068807\MP2=-1483.5059213\RMSD=4.564e-09\Thermal
=0.\PG=C01 [X(C9H9P1S2)]\\@

223-Me$^+$

1\1\GINC-AZAZEL\SP\RMP2-FC\6-31+G(2d,p)\C10H12P1S2(1+)\CHRISTOPH\14-Se
p-2009\0\\#p MP2(FC)/6-31+g(2d,p) scf=tight\\thio2mesp_7\\1,1\P\C,1,1.
82175755\H,2,1.09559039,1,110.56439518\H,2,1.09550762,1,108.47745143,3
,119.59451514,0\H,2,1.09475643,1,110.07640097,4,119.05577328,0\C,1,1.8
2175791,2,107.74455783,5,-179.95755133,0\H,6,1.09550608,1,108.48217907
,2,61.00229753,0\H,6,1.09475453,1,110.07870701,2,-179.93025148,0\H,6,1
.09559395,1,110.55978778,2,-58.58967983,0\C,1,1.77779713,2,108.0810694
4,6,120.19651585,0\C,10,1.38640617,1,127.14222057,2,-14.03106098,0\C,1
1,1.42113876,10,112.87670767,1,-176.56852927,0\C,12,1.37383358,11,112.
46494872,10,0.27234507,0\S,13,1.72766256,12,112.6203668,11,-0.31083773
,0\H,11,1.08564512,10,123.90970612,1,3.74278706,0\H,12,1.08455955,11,1
23.95979545,10,-179.36435147,0\H,13,1.08386928,12,128.04420628,11,-179
.48759699,0\C,1,1.77781186,10,110.61243752,11,-135.91939821,0\C,18,1.3
8640548,1,127.14372145,10,-136.22427778,0\C,19,1.42113307,18,112.87670
152,1,-176.52742948,0\C,20,1.37384439,19,112.46440815,18,0.27477756,0\
S,21,1.72766576,20,112.62041645,19,-0.30901169,0\H,19,1.08564835,18,12
3.91039571,1,3.78523715,0\H,20,1.08455931,19,123.95972723,18,-179.3550
2567,0\H,21,1.08386844,20,128.04367387,19,-179.47985924,0\\Version=AM6
4L-G03RevD.01\State=1-A\HF=-1521.2451672\MP2=-1523.0905912\RMSD=3.320e
-09\Thermal=0.\PG=C01 [X(C10H12P1S2)]\\@

224

1\1\GINC-EDDY\SP\RMP2-FC\6-31+G(2d,p)\C9H9O2P1\CHRISTOPH\30-Sep-2009\0
\\#p MP2(FC)/6-31+g(2d,p) scf=tight\\fa2sp_5\\0,1\P\C,1,1.82781769\C,2
,1.36930313,1,123.88117811\O,3,1.35665107,2,111.35893621,1,179.1077360
7,0\C,4,1.36514513,3,106.71067324,2,0.26717569,0\C,5,1.35976691,4,110.
6491977,3,-0.14466084,0\C,1,1.82972553,2,100.56693493,3,137.72393242,0
\C,7,1.44767533,1,125.80949387,2,-70.49667963,0\C,8,1.36024337,7,106.5
1172853,1,-175.82958256,0\O,9,1.36157595,8,110.63848681,7,0.49733647,0
\C,10,1.36113219,9,106.72785447,8,-0.40564803,0\H,11,1.08018261,10,115
.32028468,9,179.42456423,0\H,9,1.08046009,8,133.70232697,7,-179.412372
93,0\H,8,1.08226466,7,126.74988903,1,4.71378665,0\H,3,1.08105377,2,132
.73127766,1,-0.98233984,0\H,6,1.08207057,5,126.89493263,4,178.97008097
,0\H,5,1.08057961,4,115.48318381,3,179.56616049,0\C,1,1.865515,2,100.6
925584,3,-119.65758556,0\H,18,1.09672367,1,113.11885448,2,-51.99745384
,0\H,18,1.09683377,1,109.43435652,2,-174.01291996,0\H,18,1.09551657,1,
108.30151314,2,68.55589542,0\\Version=AM64L-G03RevD.01\State=1-A\HF=-8
36.4804051\MP2=-838.2860408\RMSD=3.595e-09\Thermal=0.\PG=C01 [X(C9H9O2

P1)]\\@

224-Me^{+}

1\1\GINC-GRETEL\SP\RMP2-FC\6-31+G(2d,p)\C10H12O2P1(1+)\CHRISTOPH\30-Se
p-2009\0\\#p MP2(FC)/6-31+g(2d,p) scf=tight\\fa2mesp_2\\1,1\C\C,1,1.37
673842\O,2,1.33979004,1,110.13324957\C,3,1.36402455,2,108.00049227,1,0
.14039808,0\C,4,1.35962049,3,110.55065578,2,-0.10079829,0\C,1,2.930062
23,2,142.2479924,3,135.49456774,0\H,2,1.08076076,1,133.1838475,5,-179.
85091454,0\H,5,1.08182791,4,126.38688295,3,-179.87188383,0\H,4,1.08004
944,3,115.79571026,2,-179.90328583,0\P,1,1.77618329,2,125.69433911,3,-
178.97929364,0\C,10,1.82015505,1,110.41324332,2,109.50273039,0\H,11,1.
0956839,10,109.0481873,1,58.21796185,0\H,11,1.09490057,10,109.97190239
,1,-60.60672198,0\H,11,1.09564664,10,110.42101888,1,177.88374639,0\C,1
0,1.77548476,1,109.81158861,2,-11.13107231,0\C,15,1.44920429,10,127.80
83556,1,-61.78888322,0\C,16,1.35974429,15,105.46489666,10,-179.2464703
7,0\O,17,1.36368709,16,110.52810932,15,0.05269223,0\C,18,1.33970118,17
,108.01853031,16,-0.00625125,0\H,19,1.08096213,18,116.15176929,17,179.
98823564,0\H,17,1.08007757,16,133.69325618,15,179.92599231,0\H,16,1.08
178044,15,127.88553025,10,0.82124794,0\H,6,1.09497198,1,91.23539037,2,
-42.97769571,0\H,6,1.09588463,1,89.68359469,2,-151.83571869,0\H,6,1.09
54407,1,144.58928516,2,83.8924282,0\\Version=IA32L-G03RevD.01\State=1-
A\HF=-875.9221554\MP2=-877.8714808\RMSD=6.128e-09\Thermal=0.\PG=C01 [X
(C10H12O2P1)]\\@

225

1\1\GINC-YANG\SP\RMP2-FC\6-31+G(2d,p)\C6H9P1S1\CHRISTOPH\09-Sep-2009\0
\\#p MP2(FC)/6-31+g(2d,p) scf=tight\\thio1sp_4\\0,1\P\C,1,1.86633245\H
,2,1.09742838,1,113.25342369\H,2,1.09579804,1,109.29573325,3,-121.3502
8162,0\H,2,1.09729815,1,108.82856907,4,-117.6735733,0\C,1,1.86631102,2
,99.44054386,4,-171.80863942,0\H,6,1.09579952,1,109.29720942,2,171.837
31507,0\H,6,1.09743053,1,113.25507255,2,50.48340258,0\H,6,1.09730286,1
,108.82728759,2,-70.49211907,0\C,1,1.8278865,6,101.49156493,2,-103.877
09025,0\C,10,1.37833035,1,124.61112695,6,-128.90540777,0\C,11,1.429789
32,10,114.22310552,1,179.99494227,0\C,12,1.36983745,11,112.51805313,10
,0.00277669,0\S,13,1.73634693,12,111.66933536,11,0.00186958,0\H,11,1.0
8627989,10,122.01034629,1,0.,0\H,12,1.08569729,11,123.99915585,10,-179
.99588203,0\H,13,1.0835197,12,128.19695041,11,-179.99947405,0\\Version
=AM64L-G03RevD.01\State=1-A\HF=-970.7000964\MP2=-971.8483946\RMSD=4.40
9e-09\Thermal=0.\PG=C01 [X(C6H9P1S1)]\\@

225-Me^{+}

1\1\GINC-GOLEM\SP\RMP2-FC\6-31+G(2d,p)\C7H12P1S1(1+)\CHRISTOPH\11-Sep-
2009\0\\#p MP2(FC)/6-31+g(2d,p) scf=tight\\thio1mesp_1\\1,1\P\C,1,1.81
693727\H,2,1.09545674,1,110.32312214\H,2,1.09545915,1,110.32571089,3,1
20.81198169,0\H,2,1.0958519,1,109.21045454,3,-119.59436537,0\C,1,1.819
92207,2,108.83367855,3,178.63582811,0\H,6,1.09628388,1,110.04543884,2,
-60.35473958,0\H,6,1.09523344,1,109.55777472,2,60.03702735,0\H,6,1.095
54608,1,109.97057528,2,179.55630378,0\C,1,1.81991903,2,108.8351584,6,-
118.08447065,0\H,10,1.09628278,1,110.04555235,2,60.37326278,0\H,10,1.0
9554734,1,109.97211969,2,-179.53989108,0\H,10,1.09523161,1,109.5575861
6,2,-60.01902724,0\C,1,1.7784779,2,109.61289936,6,120.96150736,0\C,14,
1.38620477,1,126.45049427,2,179.94087146,0\C,15,1.42074313,14,112.8868
4962,1,-179.99605518,0\C,16,1.37439667,15,112.50001123,14,0.0030601,0\
S,17,1.7258761,16,112.53278014,15,-0.03421384,0\H,15,1.08671177,14,123
.99864835,1,-0.00847411,0\H,16,1.08456093,15,123.99277845,14,179.98678
491,0\H,17,1.0838196,16,128.13165324,15,179.98919979,0\\Version=AM64L-
G03RevD.01\State=1-A\HF=-1010.1391136\MP2=-1011.4330668\RMSD=7.808e-09
\Thermal=0.\PG=C01 [X(C7H12P1S1)]\\@

226

1\1\GINC-AZAZEL\SP\RMP2-FC\6-31+G(2d,p)\C12H9P1S3\CHRISTOPH\14-Sep-200
9\0\\#p MP2(FC)/6-31+g(2d,p) scf=tight\\thio3sp_41\\0,1\P\C,1,1.826954
66\C,2,1.37987285,1,123.0679591\C,3,1.42779153,2,113.91941292,1,-179.6
9018017,0\C,4,1.37088649,3,112.42274212,2,-0.64150402,0\S,5,1.73472437
,4,111.85864333,3,0.17769869,0\H,3,1.08636501,2,122.3081111,1,0.549275
05,0\H,4,1.0856264,3,124.00322497,2,-179.85651512,0\H,5,1.0835619,4,12
8.12422579,3,-178.64888262,0\C,1,1.82699012,2,103.70241997,3,-148.8296
5525,0\C,10,1.37992197,1,123.04309785,2,102.58930296,0\C,11,1.42769744
,10,113.91331976,1,-179.70990378,0\C,12,1.3709102,11,112.42643246,10,-
0.61375206,0\S,13,1.73481273,12,111.8619354,11,0.14858805,0\H,11,1.086
34915,10,122.31992425,1,0.58197299,0\H,12,1.0856323,11,123.99358225,10
,-179.81421443,0\H,13,1.0835594,12,128.12105539,11,-178.62518624,0\C,1
,1.82717775,2,103.7141671,3,103.06290563,0\C,18,1.37993976,1,123.03282
126,2,-148.29490278,0\C,19,1.42784513,18,113.92710398,1,-179.76437296,
0\C,20,1.3708872,19,112.42455294,18,-0.6639843,0\S,21,1.73476624,20,11
1.85586709,19,0.16587569,0\H,19,1.08632225,18,122.3002682,1,0.46737049
,0\H,20,1.08560914,19,124.00554393,18,-179.86478296,0\H,21,1.08358595,
20,128.13120194,19,-178.66927602,0\\Version=AM64L-G03RevD.01\State=1-A
\HF=-1992.9106979\MP2=-1995.1651755\RMSD=2.592e-09\Thermal=0.\PG=C01 [
X(C12H9P1S3)]\\@

226-Me$^+$

1\1\GINC-YANG\SP\RMP2-FC\6-31+G(2d,p)\C13H12P1S3(1+)\CHRISTOPH\15-Sep-
2009\0\\#p MP2(FC)/6-31+g(2d,p) scf=tight\\thio3mesp_6\\1,1\P\C,1,1.82
209757\H,2,1.09541537,1,109.96978412\H,2,1.09484553,1,109.84530535,3,1
20.68090242,0\H,2,1.09525689,1,109.13830668,4,119.41884815,0\C,1,1.781
01194,2,108.44199891,4,58.44947911,0\C,6,1.38658563,1,127.46968109,2,-
36.19344929,0\C,7,1.42056297,6,112.80836305,1,-177.39566743,0\C,8,1.37
500558,7,112.41417157,6,0.17838617,0\S,9,1.72558465,8,112.68024578,7,-
0.14696482,0\H,7,1.08594151,6,123.95062512,1,2.59056848,0\H,8,1.084618
68,7,123.99231162,6,-179.37107052,0\H,9,1.08383185,8,127.89542148,7,-1
79.42909045,0\C,1,1.78265943,6,110.42301235,7,83.57352366,0\C,14,1.386
49168,1,127.05378259,6,-174.67025452,0\C,15,1.42077287,14,112.75301386
,1,179.9977501,0\C,16,1.37559918,15,112.36940191,14,0.0280301,0\S,17,1
.72475944,16,112.7033484,15,-0.05038874,0\H,15,1.08641246,14,123.76890
83,1,-0.31875197,0\H,16,1.0847224,15,124.03741351,14,-179.74347679,0\H
,17,1.08385008,16,127.8385999,15,-179.64630645,0\C,1,1.78144218,6,110.
14556164,7,-155.90682392,0\C,22,1.38542459,1,126.60570075,6,-107.72540
443,0\C,23,1.42205035,22,112.80725844,1,178.36134427,0\C,24,1.37337549
,23,112.55018914,22,-0.02049762,0\S,25,1.72860083,24,112.55318641,23,-
0.1085374,0\H,23,1.08526196,22,123.18353543,1,-1.63963496,0\H,24,1.084
58255,23,123.96721812,22,179.86566458,0\H,25,1.08386461,24,128.1134167
7,23,179.75043423,0\\Version=AM64L-G03RevD.01\State=1-A\HF=-2032.34927
48\MP2=-2034.7505758\RMSD=2.232e-09\Thermal=0.\PG=C01 [X(C13H12P1S3)]\
\@

227

1\1\GINC-NAUTILUS\SP\RMP2-FC\6-31+G(2d,p)\C12H9O3P1\CHRISTOPH\01-Oct-2
009\0\\#p MP2(FC)/6-31+g(2d,p) scf=tight\\fa3sp_22\\0,1\P\C,1,1.828611
36\C,2,1.36957397,1,123.52851482\O,3,1.35616329,2,111.32263026,1,-179.
78891589,0\C,4,1.36450524,3,106.76029203,2,-0.47715114,0\C,5,1.3602214
3,4,110.63535513,3,0.25349998,0\C,1,1.82950253,2,100.15654205,3,-146.5
2916804,0\C,7,1.4480345,1,125.83130215,2,64.77137469,0\C,8,1.36070401,
7,106.38633479,1,175.12339112,0\O,9,1.36119856,8,110.64655272,7,-0.480
68692,0\C,10,1.36092067,9,106.82076069,8,0.33876316,0\C,1,1.82640659,2
,102.58057641,3,109.72794885,0\C,12,1.36950522,1,122.74337398,2,-133.0

9003785,0\O,13,1.35565621,12,111.38739311,1,179.4091618,0\C,14,1.36472
599,13,106.73964998,12,-0.21533101,0\C,15,1.36021592,14,110.65868412,1
3,0.12991689,0\H,13,1.08096455,12,132.64137116,1,-0.64485289,0\H,16,1.
08163903,15,126.6915634,14,-179.43434311,0\H,15,1.08054689,14,115.5209
2655,13,-179.6334317,0\H,11,1.07996531,10,115.82207873,9,-179.51331732
,0\H,9,1.08051375,8,133.64825727,7,179.41043968,0\H,8,1.08222633,7,126
.91002023,1,-5.4444691,0\H,3,1.08096606,2,132.78163772,1,0.18699,0\H,6
,1.08193567,5,126.92550459,4,-178.56585893,0\H,5,1.0805377,4,115.50556
081,3,-179.46776651,0\\Version=AM64L-G03RevD.01\State=1-A\HF=-1024.925
2508\MP2=-1027.3351927\RMSD=7.138e-09\Thermal=0.\PG=C01 [X(C12H9O3P1)]
\\@

227-Me$^+$
1\1\GINC-CALYPSO\SP\RMP2-FC\6-31+G(2d,p)\C13H12O3P1(1+)\CHRISTOPH\01-O
ct-2009\0\\#p MP2(FC)/6-31+g(2d,p) scf=tight\\fa3mesp_26\\1,1\C\C,1,1.
37616686\O,2,1.34045987,1,110.13661784\C,3,1.36443864,2,107.9586997,1,
0.09933294,0\C,4,1.36000195,3,110.57056217,2,-0.09123441,0\C,1,2.90941
546,2,90.65279985,3,174.92909589,0\C,6,1.44883918,1,109.43842631,2,-56
.71549253,0\C,7,1.35995849,6,105.39734212,1,146.30049078,0\O,8,1.36430
524,7,110.57771641,6,0.20337931,0\C,9,1.34046129,8,107.95910999,7,-0.2
3949797,0\H,10,1.08072037,9,116.69278809,8,-179.95004383,0\H,8,1.08005
452,7,133.62178601,6,179.88766984,0\H,7,1.08181135,6,128.04554115,1,-3
3.54087904,0\H,2,1.08070745,1,133.15072964,5,-179.91016117,0\H,5,1.081
76008,4,126.55952306,3,-179.98809086,0\H,4,1.08005991,3,115.80618866,2
,-179.92208162,0\P,1,1.77771983,2,125.42678611,3,179.93650317,0\C,17,1
.82070605,1,109.13217524,2,-128.23283166,0\H,18,1.09520157,17,109.6702
2835,1,-177.69524795,0\H,18,1.09528194,17,109.72999372,1,-57.69258568,
0\H,18,1.09522872,17,109.66924814,1,62.31697203,0\C,17,1.77784701,1,10
9.8374132,2,112.18939728,0\C,22,1.44882338,17,128.62963256,1,171.71272
283,0\C,23,1.35997516,22,105.40045334,17,-179.81538299,0\O,24,1.364307
79,23,110.56334263,22,0.07949488,0\C,25,1.34050151,24,107.97664993,23,
-0.10397671,0\H,26,1.08068906,25,116.72402059,24,179.96660873,0\H,24,1
.08006474,23,133.62405392,22,179.84400886,0\H,23,1.08180568,22,128.035
59985,17,0.25215899,0\\Version=AM64L-G03RevD.01\State=1-A\HF=-1064.367
6946\MP2=-1066.9209505\RMSD=5.462e-09\Thermal=0.\PG=C01 [X(C13H12O3P1)
]\\@

228
1\1\GINC-GRETEL\SP\RMP2-FC\6-31+G(2d,p)\C6H9O1P1\CHRISTOPH\30-Sep-2009
\0\\#p MP2(FC)/6-31+g(2d,p) scf=tight\\fa1sp_2\\0,1\P\C,1,1.82816283\C
,2,1.36901274,1,123.70433656\O,3,1.3575248,2,111.52942654,1,-179.92543
102,0\C,4,1.36387943,3,106.66004205,2,0.06387908,0\C,5,1.36031243,4,11
0.62519347,3,-0.09758764,0\C,1,1.86701629,2,100.56714821,3,-130.593490
3,0\H,3,1.08087883,2,132.5176263,1,0.10304135,0\H,6,1.08242802,5,126.2
74929,4,-179.97266854,0\H,5,1.08061138,4,115.6037932,3,-179.98450127,0
\C,1,1.86677234,2,100.41578624,3,127.8158846,0\H,11,1.09745739,1,113.0
3983515,2,51.05385366,0\H,11,1.09711284,1,109.14089945,2,172.05819435,
0\H,11,1.09587516,1,109.43609323,2,-70.05995429,0\H,7,1.09722469,1,109
.00005184,2,-172.93165759,0\H,7,1.09799841,1,113.24254982,2,-51.999117
54,0\H,7,1.09597233,1,109.37809633,2,69.36518123,0\\Version=IA32L-G03R
evD.01\State=1-A\HF=-648.036579\MP2=-649.238613\RMSD=6.116e-09\Thermal
=0.\PG=C01 [X(C6H9O1P1)]\\@

228-Me$^+$
1\1\GINC-MAX\SP\RMP2-FC\6-31+G(2d,p)\C7H12O1P1(1+)\CHRISTOPH\30-Sep-20
09\0\\#p MP2(FC)/6-31+g(2d,p) scf=tight\\fa1mesp_2\\1,1\C\C,1,1.377152
2\O,2,1.33911754,1,110.15040169\C,3,1.36341392,2,108.04277934,1,0.0009
6485,0\C,4,1.35948461,3,110.52587394,2,-0.00064231,0\C,1,2.93797329,2,

90.83406092,3,179.96197571,0\H,2,1.08100327,1,133.69283514,5,-179.9981
2119,0\H,5,1.08188495,4,126.41453675,3,-180.,0\H,4,1.08003765,3,115.80
851877,2,-179.99934116,0\P,1,1.77442495,2,126.43483011,3,-179.9935546,
0\C,10,1.81868967,1,110.23188477,2,-120.11073798,0\H,11,1.0960072,10,1
10.10566177,1,-178.74504766,0\H,11,1.09562217,10,109.94814104,1,-58.68
877411,0\H,11,1.09530814,10,109.63766058,1,60.81581218,0\C,10,1.818702
08,1,110.23980117,2,119.95924643,0\H,6,1.09534281,1,91.3354631,2,-54.8
086149,0\H,6,1.09578934,1,143.86700427,2,179.95804324,0\H,6,1.09534669
,1,91.34825078,2,54.70587807,0\H,15,1.09530789,10,109.63627463,1,-60.8
2925472,0\H,15,1.09600859,10,110.10566576,1,178.73192804,0\H,15,1.0956
2232,10,109.94788198,1,58.67376819,0\\Version=IA32L-G03RevD.01\State=1
-A\HF=-687.4780929\MP2=-688.8243704\RMSD=5.742e-09\Thermal=0.\PG=C01 [
X(C7H12O1P1)]\\@

229

1\1\GINC-MAX\SP\RMP2-FC\6-31+G(2d,p)\C7H12N1P1S1\CHRISTOPH\04-Jan-2010
\0\\#p MP2(FC)/6-31+g(2d,p) scf=tight\\ns621sp_2\\0,1\C\H,1,1.09674668
\H,1,1.09565049,2,107.77580951\H,1,1.0971392,3,109.27496672,2,-117.675
50241,0\C,1,2.84887164,3,148.74463051,2,132.01186983,0\H,5,1.09731147,
1,91.95881958,3,-139.74152152,0\H,5,1.09604824,1,149.53115781,3,-8.729
93688,0\H,5,1.09687764,1,85.39844524,3,111.14924804,0\P,1,1.85817501,5
,40.31016916,7,13.41961825,0\S,9,4.52041446,1,88.49651067,5,-102.89795
569,0\C,10,1.79325443,9,15.57776824,1,7.22121722,0\C,11,1.34822153,10,
121.47896595,9,2.49501716,0\N,12,1.42203932,11,119.62648023,10,3.62702
03,0\C,13,1.40694263,12,117.34420159,11,38.05251909,0\C,14,1.33898993,
13,122.91204972,12,-38.75714522,0\H,11,1.08355105,10,115.63499705,9,17
8.49818308,0\H,15,1.08519819,14,122.51533991,13,172.46918093,0\H,14,1.
09066313,13,115.68257997,12,137.26735704,0\C,13,1.4579538,12,121.64151
703,11,-166.46073801,0\H,19,1.09488897,13,108.54895689,12,159.19773677
,0\H,19,1.1035411,13,113.41941726,12,-80.45782078,0\H,19,1.0925369,13,
109.96253161,12,41.09134578,0\\Version=IA32L-G03RevD.01\State=1-A\HF=-
1064.7235855\MP2=-1066.2072012\RMSD=6.019e-09\Thermal=0.\PG=C01 [X(C7H
12N1P1S1)]\\@

229-Me$^+$

1\1\GINC-MORITZ\SP\RMP2-FC\6-31+G(2d,p)\C8H15N1P1S1(1+)\CHRISTOPH\04-J
an-2010\0\\#p MP2(FC)/6-31+g(2d,p) scf=tight\\ns621mesp_21\\1,1\C\H,1,
1.09616224\H,1,1.0955311,2,108.57755579\H,1,1.09488811,3,109.50657228,
2,-118.3445403,0\C,1,2.96773221,4,89.67299562,2,-90.58468603,0\H,5,1.0
9508906,1,144.0339348,4,-121.62141433,0\H,5,1.09504118,1,88.66707657,4
,0.02047183,0\H,5,1.09593973,1,92.5495414,4,109.47272405,0\P,5,1.81999
493,1,35.38215224,4,-128.23806869,0\C,9,1.82069294,5,107.5497528,1,-11
6.67130741,0\H,10,1.09462749,9,109.9392225,5,-59.24897808,0\H,10,1.096
38985,9,109.18411775,5,59.94036463,0\H,10,1.09513851,9,110.41106951,5,
179.86199226,0\S,9,4.39867242,5,107.24638954,1,103.64886048,0\C,14,1.7
5246189,9,16.17952844,5,-135.37904284,0\C,15,1.35318685,14,122.4440616
,9,-22.39925772,0\N,16,1.43068031,15,124.7728138,14,-5.44346169,0\C,17
,1.43660241,16,113.63506219,15,36.06363939,0\C,18,1.33601012,17,123.67
539165,16,-34.83759965,0\H,15,1.08789085,14,114.49883657,9,152.5119337
4,0\H,19,1.0861005,18,122.66029233,17,179.69783846,0\H,18,1.08881485,1
7,115.39424437,16,145.91817451,0\C,17,1.48300398,16,114.97901572,15,-9
4.42470635,0\H,23,1.09406979,17,110.06931984,16,-53.67499241,0\H,23,1.
09473381,17,108.30839637,16,-171.94686401,0\H,23,1.09878751,17,112.093
23975,16,67.77781153,0\\Version=IA32L-G03RevD.01\State=1-A\HF=-1104.16
84939\MP2=-1105.7934055\RMSD=9.104e-09\Thermal=0.\PG=C01 [X(C8H15N1P1S
1)]\\@

230

1\1\GINC-MAX\SP\RMP2-FC\6-31+G(2d,p)\C5H8N1P1S1\CHRISTOPH\23-Dec-2009\
0\\#p MP2(FC)/6-31+g(2d,p) scf=tight\\ns521sp_4\\0,1\P\C,1,1.86551834\
H,2,1.09708531,1,108.98584245\H,2,1.09608422,1,109.58743691,3,-117.906
2244,0\H,2,1.09619092,1,111.87205797,3,121.04872242,0\C,1,1.86552239,2
,99.22932792,4,174.45411153,0\H,6,1.09608353,1,109.5884484,2,-174.4523
5452,0\H,6,1.09708452,1,108.98545264,2,67.6408548,0\H,6,1.09619129,1,1
11.87182549,2,-53.40755987,0\C,1,1.84572304,2,99.09315155,6,-100.84502
895,0\N,10,1.39019477,1,122.25370119,2,50.47121503,0\C,11,1.29822765,1
0,111.13462776,1,-180.,0\S,12,1.75575497,11,115.33299238,10,-0.0015734
8,0\C,10,1.37230318,1,123.11036835,2,-129.53149635,0\H,14,1.08244886,1
0,127.8905844,1,0.00168032,0\H,12,1.08545491,11,124.14772729,10,-179.9
9925265,0\\Version=IA32L-G03RevD.01\State=1-A\HF=-986.6982868\MP2=-987
.877085\RMSD=8.490e-09\Thermal=0.\PG=C01 [X(C5H8N1P1S1)]\\@

230-Me+

1\1\GINC-MAX\SP\RMP2-FC\6-31+G(2d,p)\C6H11N1P1S1(1+)\CHRISTOPH\23-Dec-
2009\0\\#p MP2(FC)/6-31+g(2d,p) scf=tight\\ns521mesp_2\\1,1\C\H,1,1.09
545204\H,1,1.09528327,2,108.97409562\H,1,1.09587788,3,109.46294596,2,-
119.78424059,0\C,1,2.95998008,3,145.19975742,2,122.18760717,0\H,5,1.09
528197,1,145.19689309,3,0.25839776,0\H,5,1.09544621,1,91.24958542,3,-1
26.70137159,0\H,5,1.0958791,1,88.73464034,3,123.77087365,0\C,1,2.93759
476,5,59.74806465,6,41.34831164,0\N,9,1.38103728,1,94.26521355,5,92.41
295034,0\C,10,1.30213989,9,109.99026601,1,149.6081661,0\S,11,1.7502241
4,10,115.26118077,9,0.,0\C,9,1.375624,1,137.48094939,5,-129.67771509,0
\H,13,1.08298457,9,129.52693917,1,48.29727031,0\H,11,1.08513695,10,123
.90961375,9,179.9946181,0\P,9,1.79629872,1,35.82754024,5,-36.10992386,
0\C,16,1.81587794,9,110.12294463,1,-120.60603067,0\H,17,1.09545108,16,
110.35465744,9,60.59362714,0\H,17,1.09584222,16,109.25304267,9,-179.97
025287,0\H,17,1.09545527,16,110.34233418,9,-60.54033615,0\\Version=IA3
2L-G03RevD.01\State=1-A\HF=-1026.1399851\MP2=-1027.463199\RMSD=9.573e-
09\Thermal=0.\PG=C01 [X(C6H11N1P1S1)]\\@

231

1\1\GINC-NODE26\SP\RMP2-FC\6-31+G(2d,p)\C6H9P1S1\ZIP07\16-Sep-2009\0\\
#p MP2(FC)/6-31+g(2d,p) scf=tight\\thia1sp_3\\0,1\P\C,1,1.86719234\H,2
,1.09725031,1,108.89270131\H,2,1.09762987,1,113.35384988,3,120.9514271
,0\H,2,1.09597423,1,109.35751968,3,-117.65545858,0\C,1,1.86719074,2,99
.29664634,5,172.24130709,0\H,6,1.0972488,1,108.89399257,2,70.12498686,
0\H,6,1.09597401,1,109.35688841,2,-172.218766,0\H,6,1.09762743,1,113.3
5284237,2,-50.82753295,0\C,1,1.8407788,6,100.67369291,2,102.81755705,0
\C,10,1.44310507,1,128.11198559,6,-50.84873354,0\C,11,1.36684984,10,11
3.40752706,1,-179.99861046,0\S,12,1.74160125,11,111.56568538,10,0.,0\C
,10,1.3753419,1,120.57960657,6,129.15333753,0\H,14,1.08340329,10,127.4
1690667,1,-0.00232447,0\H,12,1.08322827,11,128.37859795,10,179.9992633
1,0\H,11,1.08610898,10,123.83965575,1,0.00220312,0\\Version=AM64L-G03R
evD.01\State=1-A\HF=-970.6989738\MP2=-971.8465872\RMSD=3.165e-09\Therm
al=0.\PG=C01 [X(C6H9P1S1)]\\@

231-Me+

1\1\GINC-NODE3\SP\RMP2-FC\6-31+G(2d,p)\C7H12P1S1(1+)\ZIP07\16-Sep-2009
\0\\#p MP2(FC)/6-31+g(2d,p) scf=tight\\thia1mesp_3\\1,1\P\C,1,1.819859
42\H,2,1.09604934,1,110.12946396\H,2,1.09556814,1,109.99987258,3,-120.
11176791,0\H,2,1.09532988,1,109.60877141,4,-119.48068033,0\C,1,1.81985
426,2,108.48361053,5,178.12923009,0\H,6,1.09604753,1,110.12843349,2,-5
7.72203291,0\H,6,1.09556782,1,110.00212321,2,62.38795188,0\H,6,1.09532
926,1,109.60860574,2,-178.12896266,0\C,1,1.8168349,6,108.75501806,2,11
8.13514756,0\H,10,1.09527664,1,110.36029925,6,-178.40844302,0\H,10,1.0
9527607,1,110.35628083,6,60.46186877,0\H,10,1.09581708,1,109.18188672,

6,-58.97320375,0\C,1,1.785918,10,110.19074452,6,-121.0149189,0\C,14,1.
44135204,1,123.68235465,10,179.93985726,0\C,15,1.36718097,14,112.00467
593,1,179.99206789,0\S,16,1.7357402,15,112.04696231,14,-0.00063123,0\C
,14,1.38356992,1,123.75683033,10,-0.07044691,0\H,18,1.083093,14,128.63
511197,1,0.01143812,0\H,16,1.08316604,15,127.91509027,14,179.99857068,
0\H,15,1.08565768,14,124.91658152,1,-0.00981395,0\\Version=AM64L-G03Re
vD.01\State=1-A\HF=-1010.1415999\MP2=-1011.4353746\RMSD=7.184e-09\Ther
mal=0.\PG=C01 [X(C7H12P1S1)]\\@

232

1\1\GINC-NAUTILUS\SP\RMP2-FC\6-31+G(2d,p)\C7H11P1\CHRISTOPH\28-Sep-200
9\0\\#p MP2(FC)/6-31+g(2d,p) scf=tight\\cpi1sp_4\\0,1\P\C,1,1.86845292
\H,2,1.0973545,1,108.80352066\H,2,1.09866456,1,113.13347499,3,120.6117
1426,0\H,2,1.09616653,1,109.97245755,3,-117.44994793,0\C,1,1.86844892,
2,98.12957714,5,174.24994694,0\H,6,1.09736031,1,108.79604716,2,68.2465
0774,0\H,6,1.09615834,1,109.98848899,2,-174.30191797,0\H,6,1.09867404,
1,113.12940783,2,-52.35548314,0\C,1,1.9035285,6,99.29124059,2,100.8692
7581,0\C,10,1.50528577,1,109.41823604,6,-173.85259471,0\C,11,1.3548407
1,10,109.24186558,1,-113.52720211,0\C,12,1.46534801,11,109.20386634,10
,-1.82478003,0\C,13,1.35483289,12,109.2031531,11,0.00636272,0\H,10,1.1
0132494,1,108.19191985,6,-49.93307809,0\H,12,1.08604343,11,126.2165165
3,10,178.27604679,0\H,11,1.08488827,10,123.94549802,1,64.98214168,0\H,
14,1.08488547,13,126.79955172,12,-179.74437758,0\H,13,1.08604304,12,12
4.57863823,11,-179.91257822,0\\Version=AM64L-G03RevD.01\State=1-A\HF=-
612.1994869\MP2=-613.3521964\RMSD=3.027e-09\Thermal=0.\PG=C01 [X(C7H11
P1)]\\@

232-Me^{+}

1\1\GINC-YIN\SP\RMP2-FC\6-31+G(2d,p)\C8H14P1(1+)\CHRISTOPH\28-Sep-2009
\0\\#p MP2(FC)/6-31+g(2d,p) scf=tight\\cpi1mesp_4\\1,1\P\C,1,1.8162310
5\H,2,1.09436075,1,109.8101359\H,2,1.09570929,1,109.45399516,3,-120.08
755135,0\H,2,1.09435919,1,109.8112676,4,-120.08821313,0\C,1,1.81978781
,2,109.27812742,5,-61.01365986,0\H,6,1.09502748,1,110.1560627,2,58.319
26502,0\H,6,1.09584158,1,110.11095153,2,178.6508619,0\H,6,1.09602143,1
,109.8182633,2,-61.53025165,0\C,1,1.81978834,2,109.2777371,6,-118.1236
8003,0\H,10,1.09602141,1,109.81816586,2,61.54236069,0\H,10,1.09502717,
1,110.15597248,2,-58.30713208,0\H,10,1.09584074,1,110.11095754,2,-178.
6388591,0\C,1,1.84094542,2,110.36435203,10,-120.93805636,0\C,14,1.5164
5433,1,110.27863505,2,56.90479719,0\C,15,1.35224671,14,108.46226631,1,
-117.15905425,0\C,16,1.469839,15,109.73928724,14,-0.55205892,0\C,17,1.
35224597,16,109.73931623,15,0.00157016,0\H,14,1.10168232,1,106.9283735
4,2,-179.99559497,0\H,16,1.08510439,15,125.84228462,14,-178.7836398,0\
H,15,1.08433536,14,124.16230955,1,64.80390641,0\H,18,1.08433453,17,127
.34210921,16,-177.40678444,0\H,17,1.08510398,16,124.39323406,15,-178.2
634614,0\\Version=AM64L-G03RevD.01\State=1-A\HF=-651.6461594\MP2=-652.
9421591\RMSD=9.277e-09\Thermal=0.\PG=C01 [X(C8H14P1)]\\@

233

1\1\GINC-NODE21\SP\RMP2-FC\6-31+G(2d,p)\C9H9P1S2\ZIP07\16-Sep-2009\0\\
#p MP2(FC)/6-31+g(2d,p) scf=tight\\thia2sp_14\\0,1\P\C,1,1.8647854\H,2
,1.09565028,1,108.05823463\H,2,1.09678016,1,109.54503849,3,117.2638098
3,0\H,2,1.0968248,1,113.2650984,3,-120.46920882,0\C,1,1.83991131,2,101
.00781267,3,170.57440868,0\C,6,1.44315253,1,121.82610583,2,-170.742502
5,0\C,7,1.36619924,6,113.43187183,1,-176.10891805,0\S,8,1.73978756,7,1
11.58226003,6,0.61541698,0\C,6,1.37522845,1,126.58658184,2,14.28875026
,0\C,1,1.84054495,6,100.43712831,10,117.28326911,0\C,11,1.44205368,1,1
27.4941303,6,-41.14430019,0\C,12,1.36645019,11,113.20888644,1,-178.600
90201,0\S,13,1.74279312,12,111.60177428,11,-0.08155932,0\C,11,1.375333

43,1,120.85390957,6,140.01089311,0\H,15,1.08349324,11,127.59869504,1,-
1.47320277,0\H,13,1.08320536,12,128.42031987,11,-179.64515695,0\H,12,1
.08580601,11,123.30282726,1,2.3439992,0\H,10,1.08250211,6,128.47584765
,1,-3.9564411,0\H,8,1.08299162,7,128.2642296,6,-179.54514606,0\H,7,1.0
8614275,6,123.32091476,1,4.3817221,0\\Version=AM64L-G03RevD.01\State=1
-A\HF=-1481.8064859\MP2=-1483.5040157\RMSD=6.994e-09\Thermal=0.\PG=C01
 [X(C9H9P1S2)]\\@

233-Me$^+$
1\1\GINC-NODE1\SP\RMP2-FC\6-31+G(2d,p)\C10H12P1S2(1+)\ZIP07\17-Sep-200
9\0\\#p MP2(FC)/6-31+g(2d,p) scf=tight\\thia2mesp_2\\1,1\P\C,1,1.82236
506\H,2,1.09490591,1,109.76625841\H,2,1.0955206,1,109.51292671,3,119.8
4542173,0\H,2,1.09571102,1,110.27043067,4,120.20563492,0\C,1,1.8207134
5,2,107.67559689,3,-178.89241657,0\H,6,1.09568693,1,108.88500048,2,-60
.92800206,0\H,6,1.09558198,1,110.53791893,2,58.73018677,0\H,6,1.094824
44,1,109.99656832,2,-179.62881672,0\C,1,1.78747322,6,109.67398073,2,-1
19.35296629,0\C,10,1.38315443,1,123.68988774,6,-1.76342219,0\S,11,1.72
211181,10,111.51056465,1,178.88157051,0\C,12,1.73674701,11,91.87149112
,10,0.03982424,0\C,13,1.36736266,12,112.019707,11,-0.07568635,0\C,1,1.
78955763,10,110.38271194,11,119.80919105,0\C,15,1.38293751,1,122.97906
733,10,-14.71276042,0\S,16,1.72164143,15,111.4687263,1,-178.1565876,0\
C,17,1.73674048,16,91.90311239,15,0.12851439,0\C,18,1.36728755,17,112.
00333603,16,-0.08091626,0\H,11,1.08308926,10,128.64131316,1,-1.1272300
9,0\H,14,1.08538359,13,123.35978825,12,179.71232147,0\H,13,1.08315319,
12,120.00855574,11,-179.94407109,0\H,16,1.08316131,15,128.04030475,1,2
.03525379,0\H,19,1.08548079,18,123.03323402,17,-179.83805015,0\H,18,1.
08314568,17,120.06466496,16,-179.75845403,0\\Version=AM64L-G03RevD.01\
State=1-A\HF=-1521.2488401\MP2=-1523.0940101\RMSD=8.867e-09\Thermal=0.
\PG=C01 [X(C10H12P1S2)]\\@

234
1\1\GINC-CALYPSO\SP\RMP2-FC\6-31+G(2d,p)\C11H13P1\CHRISTOPH\28-Sep-200
9\0\\#p MP2(FC)/6-31+g(2d,p) scf=tight\\cpi2sp_2\\0,1\P\C,1,1.91430088
\C,2,1.50848723,1,108.81998323\C,3,1.3540061,2,109.18404569,1,-122.495
72814,0\C,4,1.46861962,3,109.15249675,2,-1.65060715,0\C,5,1.35471767,4
,109.19596382,3,0.13059687,0\H,2,1.10102421,1,103.78349121,3,119.15616
45,0\C,1,1.90530808,2,100.73776514,6,54.00818961,0\C,8,1.50591191,1,10
7.63449209,2,-179.59597402,0\C,9,1.35524284,8,109.21598028,1,-112.4571
0157,0\C,10,1.46457713,9,109.19762988,8,-1.99853333,0\C,11,1.35521697,
10,109.22099865,9,0.00486775,0\H,8,1.09933899,1,109.12532507,2,-56.126
36938,0\C,1,1.86546288,8,99.62999735,12,171.92921491,0\H,14,1.09627423
,1,108.81445693,8,72.6052178,0\H,14,1.09570562,1,108.62799575,8,-170.1
3819871,0\H,14,1.09588456,1,113.53328086,8,-49.42475314,0\H,12,1.08495
284,11,126.70623731,10,-179.57971458,0\H,11,1.08599063,10,124.59498496
,9,-179.8515981,0\H,10,1.08594848,9,126.20477725,8,178.12594177,0\H,9,
1.08477597,8,123.94311041,1,65.91089197,0\H,6,1.08491237,5,126.5503591
7,4,-178.94823126,0\H,5,1.08630931,4,124.54788897,3,-179.36539184,0\H,
4,1.08605764,3,126.2577975,2,178.94086159,0\H,3,1.08506845,2,123.56754
64,1,56.168445,0\\Version=AM64L-G03RevD.01\State=1-A\HF=-764.8074475\M
P2=-766.5151658\RMSD=7.691e-09\Thermal=0.\PG=C01 [X(C11H13P1)]\\@

234-Me$^+$
1\1\GINC-GRETEL\SP\RMP2-FC\6-31+G(2d,p)\C12H16P1(1+)\CHRISTOPH\28-Sep-
2009\0\\#p MP2(FC)/6-31+g(2d,p) scf=tight\\cpi2mesp_1\\1,1\P\C,1,1.819
9537\H,2,1.0949395,1,109.49056753\H,2,1.0941568,1,110.18273865,3,-120.
49403755,0\H,2,1.09472198,1,109.41363495,3,119.78974028,0\C,1,1.819952
86,2,109.03439339,4,-171.89635093,0\H,6,1.09472099,1,109.41358582,2,68
.36905693,0\H,6,1.09415714,1,110.18234286,2,-171.91503719,0\H,6,1.0949

3952,1,109.49081675,2,-51.42099532,0\C,1,1.84492848,6,109.20352909,2,-
119.68722113,0\C,10,1.51577811,1,110.34227674,6,57.16412661,0\C,11,1.3
5249252,10,108.51433831,1,-117.23803223,0\C,12,1.46963335,11,109.71680
613,10,-0.48106353,0\C,13,1.3522014,12,109.69518576,11,-0.1302193,0\H,
10,1.10077091,1,106.82058308,6,179.97287607,0\C,1,1.84492999,6,109.564
56783,2,119.46409606,0\C,16,1.51577535,1,110.34312639,6,-62.1934735,0\
C,17,1.35249209,16,108.51440107,1,-117.23684653,0\C,18,1.46963242,17,1
09.71690839,16,-0.48113305,0\C,19,1.35220235,18,109.69507163,17,-0.130
65783,0\H,16,1.10077036,1,106.82097032,6,60.61580394,0\H,20,1.08446595
,19,127.23801627,18,-177.56516816,0\H,19,1.0851239,18,124.41399155,17,
-178.46579118,0\H,18,1.08516839,17,125.86132562,16,-178.71129422,0\H,1
7,1.08428703,16,124.12162748,1,64.74545113,0\H,14,1.08446612,13,127.23
801738,12,-177.5648567,0\H,13,1.08512454,12,124.41389335,11,-178.46531
264,0\H,12,1.08516804,11,125.86134066,10,-178.71108608,0\H,11,1.084286
23,10,124.12160857,1,64.74423376,0\\Version=IA32L-G03RevD.01\State=1-A
\HF=-804.2565688\MP2=-806.1061739\RMSD=5.514e-09\Thermal=0.\PG=C01 [X(
C12H16P1)]\\@

235

1\1\GINC-NAUTILUS\SP\RMP2-FC\6-31+G(2d,p)\C15H15P1\CHRISTOPH\28-Sep-20
09\0\\#p MP2(FC)/6-31+g(2d,p) scf=tight\\cpi3sp_5\\0,1\P\C,1,1.9009044
6\C,2,1.50627446,1,107.12868679\C,3,1.35517138,2,109.0871542,1,-110.85
586671,0\C,4,1.46485464,3,109.26363984,2,-2.11356548,0\C,5,1.35551711,
4,109.30511103,3,0.16274217,0\H,2,1.09730158,1,110.0392747,3,123.80120
173,0\C,1,1.90808988,2,102.93624569,6,174.28043308,0\C,8,1.51010201,1,
110.56688152,2,93.86924393,0\C,9,1.35346293,8,109.31620818,1,-128.5462
4514,0\C,10,1.46971037,9,109.11066394,8,-0.40097719,0\C,11,1.35421635,
10,109.2873518,9,0.10304852,0\H,8,1.10396283,1,102.19131818,2,-148.061
34663,0\C,1,1.91420133,2,100.91690868,6,70.44081195,0\C,14,1.50712361,
1,109.46591609,2,174.66834765,0\C,15,1.35460116,14,109.17915672,1,-124
.86974581,0\C,16,1.46850666,15,109.1814436,14,-1.43079034,0\C,17,1.355
11805,16,109.17569603,15,0.24360955,0\H,14,1.10176315,1,102.79980862,2
,-67.05250287,0\H,16,1.08603298,15,126.25411052,14,179.30367391,0\H,15
,1.08500763,14,123.57976426,1,54.24908877,0\H,18,1.08501953,17,126.718
91307,16,-178.74492544,0\H,17,1.08615746,16,124.54121451,15,-179.47621
156,0\H,6,1.08496875,5,126.66787501,4,-179.29517328,0\H,5,1.08611652,4
,124.59331264,3,-179.45884097,0\H,4,1.08592698,3,126.18325231,2,178.10
606423,0\H,3,1.08358441,2,123.32114466,1,66.85098142,0\H,10,1.0860976,
9,126.28013881,8,-179.88946236,0\H,9,1.08478123,8,123.47927824,1,50.94
492765,0\H,12,1.08355276,11,127.12916233,10,-177.84498772,0\H,11,1.086
30457,10,124.55422829,9,-179.1181586,0\\Version=AM64L-G03RevD.01\State
=1-A\HF=-917.4136879\MP2=-919.6784443\RMSD=7.290e-09\Thermal=0.\PG=C01
[X(C15H15P1)]\\@

235-Me^{+}

1\1\GINC-CALYPSO\SP\RMP2-FC\6-31+G(2d,p)\C16H18P1(1+)\CHRISTOPH\28-Sep
-2009\0\\#p MP2(FC)/6-31+g(2d,p) scf=tight\\cpi3mesp_1\\1,1\P\C,1,1.82
31182\H,2,1.09449939,1,109.78103429\H,2,1.09400104,1,109.56184688,3,11
9.6365888,0\H,2,1.09500195,1,109.65612945,4,120.22036588,0\C,1,1.84834
753,2,109.80981465,4,-73.63032511,0\C,6,1.51664225,1,113.56301384,2,15
8.82602516,0\C,7,1.35147992,6,108.55648363,1,-123.44684499,0\C,8,1.470
52046,7,109.71019571,6,0.52319627,0\C,9,1.35167719,8,109.69805878,7,0.
30801074,0\H,6,1.10316338,1,104.66028756,2,-79.7273521,0\C,1,1.8466039
7,2,108.97696198,6,122.95618918,0\C,12,1.51644613,1,109.67840677,2,58.
13279008,0\C,13,1.35244523,12,108.46968298,1,-117.44909891,0\C,14,1.46
968978,13,109.72665597,12,-0.54021999,0\C,15,1.35218429,14,109.7283494
2,13,-0.08039772,0\H,12,1.09964543,1,106.82612301,2,-178.49313521,0\C,
1,1.84985673,2,107.35239684,12,118.72470599,0\C,18,1.5151416,1,112.020

76147,2,177.72543614,0\C,19,1.35244436,18,108.58551758,1,-119.22832049
,0\C,20,1.46983215,19,109.63927354,18,-0.33461427,0\C,21,1.35284995,20
,109.68998246,19,0.0123904,0\H,18,1.10134558,1,105.916493,2,-59.278317
83,0\H,16,1.08264901,15,127.87910838,14,-178.47805261,0\H,15,1.0851533
7,14,124.37226683,13,-178.28730741,0\H,14,1.08520485,13,125.81991267,1
2,-178.62692383,0\H,13,1.08442279,12,124.33381453,1,64.68258594,0\H,20
,1.08513307,19,125.93842238,18,-178.6436515,0\H,19,1.08454093,18,124.1
1175173,1,62.80518824,0\H,22,1.08424538,21,127.23130312,20,-177.227432
54,0\H,21,1.08517073,20,124.4024291,19,-178.13626747,0\H,10,1.08401909
,9,127.22289759,8,-177.78658047,0\H,9,1.08522921,8,124.45648561,7,-178
.06039365,0\H,8,1.08512258,7,125.83354494,6,-177.75815564,0\H,7,1.0831
5483,6,123.61570426,1,59.74625749,0\\Version=AM64L-G03RevD.01\State=1-
A\HF=-956.8633588\MP2=-959.2697004\RMSD=4.101e-09\Thermal=0.\PG=C01 [X
(C16H18P1)]\\@

236

1\1\GINC-NODE23\SP\RMP2-FC\6-31+G(2d,p)\C7H11P1\ZIP07\17-Sep-2009\0\\#
p MP2(FC)/6-31+g(2d,p) scf=tight\\cpe1sp_7\\0,1\P\C,1,1.86885015\H,2,1
.09721705,1,108.96243786\H,2,1.09609533,1,109.36933555,3,-117.65501973
,0\H,2,1.09770641,1,113.34058667,3,120.97959209,0\C,1,1.86885131,2,99.
04144109,4,172.76280164,0\H,6,1.0960966,1,109.36759996,2,-172.75312732
,0\H,6,1.09721335,1,108.96543139,2,69.59037594,0\H,6,1.09770968,1,113.
33997867,2,-51.39139963,0\C,1,1.83479615,2,100.46860524,6,-102.5012672
9,0\C,10,1.48462455,1,128.52761333,2,50.67243803,0\C,11,1.34936929,10,
109.81736973,1,179.99656805,0\C,12,1.50900861,11,109.2304432,10,0.0020
3234,0\C,10,1.35702125,1,123.54057099,2,-129.32977477,0\H,13,1.1022635
3,12,112.19948678,11,120.44953358,0\H,13,1.10226171,12,112.19983584,11
,-120.45447352,0\H,14,1.08566684,10,125.89042466,1,0.00411163,0\H,12,1
.08551575,11,126.82681355,10,-180.,0\H,11,1.08641821,10,124.28849158,1
,-0.0014954,0\\Version=AM64L-G03RevD.01\State=1-A\HF=-612.2034888\MP2=
-613.3523678\RMSD=9.942e-09\Thermal=0.\PG=C01 [X(C7H11P1)]\\@

236-Me^{+}

1\1\GINC-MAX\SP\RMP2-FC\6-31+G(2d,p)\C8H14P1(1+)\CHRISTOPH\17-Sep-2009
\0\\#p MP2(FC)/6-31+g(2d,p) scf=tight\\cpe1mesp_2\\1,1\P\C,1,1.8168520
4\H,2,1.0951284,1,110.24921786\H,2,1.09580528,1,109.27370228,3,-119.58
142468,0\H,2,1.09513042,1,110.24529045,4,-119.57745793,0\C,1,1.8199533
1,2,108.89840049,3,178.61013122,0\H,6,1.09591809,1,110.17418804,2,-60.
6989685,0\H,6,1.09558072,1,109.94399238,2,179.17405699,0\H,6,1.0953397
6,1,109.63533145,2,59.70856715,0\C,1,1.81995284,2,108.90166692,6,-118.
08457483,0\H,10,1.09558129,1,109.94346946,2,-179.22431512,0\H,10,1.095
91954,1,110.17438279,2,60.64960649,0\H,10,1.09533744,1,109.63461598,2,
-59.75785751,0\C,1,1.78566862,2,110.52768644,6,120.95873076,0\C,14,1.4
816809,1,124.74167795,2,-179.99855,0\C,15,1.34961956,14,108.33481716,1
,-179.99235889,0\C,16,1.50454129,15,109.82082475,14,-0.00044309,0\C,14
,1.36069304,1,125.59517379,2,0.01023543,0\H,17,1.10203459,16,112.50134
348,15,-120.45828567,0\H,17,1.10203792,16,112.49962455,15,120.45574563
,0\H,18,1.0848027,14,127.48709909,1,-0.00848429,0\H,16,1.08425352,15,1
26.33797024,14,179.99973486,0\H,15,1.085403,14,125.14169424,1,0.007594
96,0\\Version=IA32L-G03RevD.01\State=1-A\HF=-651.6504598\MP2=-652.9435
883\RMSD=7.274e-09\Thermal=0.\PG=C01 [X(C8H14P1)]\\@

237

1\1\GINC-HAENSEL\SP\RMP2-FC\6-31+G(2d,p)\C12H9P1S3\CHRISTOPH\17-Sep-20
09\0\\#p MP2(FC)/6-31+g(2d,p) scf=tight\\thia3sp_45\\0,1\P\C,1,1.84010
834\C,2,1.44203737,1,121.74931693\C,3,1.36690048,2,113.27431162,1,177.
92412195,0\S,4,1.74098773,3,111.47387796,2,-0.64255493,0\C,2,1.3752456
,1,126.48135507,3,177.04477119,0\C,1,1.8400794,2,101.60804897,6,-94.01

509586,0\C,7,1.44213316,1,121.73866749,2,-167.61929406,0\C,8,1.3668694
3,7,113.27354807,1,177.74362796,0\S,9,1.74104531,8,111.47877581,7,-0.7
1247936,0\C,7,1.37517806,1,126.48408769,2,9.20998051,0\C,1,1.83997782,
7,101.61089921,11,-95.38901813,0\C,12,1.37526119,1,126.49187253,7,10.1
6277,0\S,13,1.73865546,12,112.10232297,1,-177.33497675,0\C,14,1.741070
89,13,91.4308805,12,-0.25724046,0\C,15,1.36686205,14,111.47573956,13,0
.54079684,0\H,11,1.08284896,7,127.72559509,1,2.24221379,0\H,9,1.083017
45,8,128.33825265,7,-179.96809517,0\H,8,1.0861715,7,123.35312814,1,-2.
14768694,0\H,13,1.08285596,12,127.73064319,1,2.05726557,0\H,16,1.08617
205,15,123.37300466,14,179.18300289,0\H,15,1.08302302,14,120.1699861,1
3,179.8666042,0\H,6,1.08286618,2,127.72716975,1,1.96860141,0\H,4,1.083
00982,3,128.34782476,2,-179.92671111,0\H,3,1.08618296,2,123.3583853,1,
-1.90472032,0\\Version=IA32L-G03RevD.01\State=1-A\HF=-1992.9133906\MP2
=-1995.1640606\RMSD=2.583e-09\Thermal=0.\PG=C01 [X(C12H9P1S3)]\\@

237-Me^{+}

1\1\GINC-GOLEM\SP\RMP2-FC\6-31+G(2d,p)\C13H12P1S3(1+)\CHRISTOPH\21-Sep
-2009\0\\#p MP2(FC)/6-31+g(2d,p) scf=tight\\thia3mesp_41\\1,1\P\C,1,1.
82403291\H,2,1.09503107,1,109.81201107\H,2,1.09500692,1,109.76140549,3
,-120.05774026,0\H,2,1.09486491,1,109.72429745,4,-119.92385875,0\C,1,1
.79070101,2,108.72442608,5,179.44520829,0\C,6,1.38257565,1,122.7357498
,2,-132.26846995,0\S,7,1.72201881,6,111.48712956,1,-179.56424722,0\C,8
,1.73750322,7,91.88451926,6,0.13459455,0\C,9,1.36765939,8,112.00557035
,7,-0.09011818,0\C,1,1.79128333,6,110.28585263,7,108.6021092,0\C,11,1.
44207869,1,124.5614503,6,169.4106393,0\C,12,1.36750075,11,111.99541464
,1,179.82852718,0\S,13,1.73781105,12,111.97825106,11,-0.02461087,0\C,1
1,1.38218165,1,122.78168688,6,-10.8694078,0\C,1,1.79117196,6,110.08755
113,7,-13.17582752,0\C,16,1.38251244,1,122.94818278,6,113.53325775,0\S
,17,1.72215992,16,111.47310135,1,179.40892889,0\C,18,1.73766887,17,91.
88046668,16,0.00667893,0\C,19,1.36736844,18,112.00720181,17,-0.1133797
7,0\H,7,1.0830444,6,127.91661965,1,0.77372633,0\H,10,1.08521369,9,123.
27717376,8,-179.98475167,0\H,9,1.08305017,8,120.03475696,7,-179.741129
52,0\H,15,1.0831307,11,127.92458613,1,0.24802745,0\H,13,1.08303032,12,
127.95815151,11,179.70724046,0\H,12,1.08517569,11,124.73997929,1,-0.09
266995,0\H,17,1.0830179,16,127.95092265,1,-0.5721309,0\H,20,1.08512279
,19,123.32449824,18,179.63241264,0\H,19,1.08310441,18,120.03258952,17,
-179.88389854,0\\Version=AM64L-G03RevD.01\State=1-A\HF=-2032.3575011\M
P2=-2034.755787\RMSD=4.575e-09\Thermal=0.\PG=C01 [X(C13H12P1S3)]\\@

238

1\1\GINC-HAENSEL\SP\RMP2-FC\6-31+G(2d,p)\C7H11P1\CHRISTOPH\09-Sep-2009
\0\\#p MP2(FC)/6-31+g(2d,p) scf=tight\\cpd1sp_5\\0,1\P\C,1,1.82359082\
C,2,1.36042978,1,129.40558169\C,3,1.46921154,2,110.16548514,1,174.0392
8394,0\C,4,1.35066718,3,109.3643045,2,-0.4844141,0\C,5,1.50873012,4,10
9.00317084,3,-0.31622956,0\H,3,1.08619317,2,126.05948387,1,-5.74096535
,0\H,4,1.08642131,3,124.24498293,2,179.17979458,0\H,5,1.08511064,4,127
.08414868,3,179.07512657,0\H,6,1.10202897,5,111.79951278,4,122.1118505
,0\H,6,1.10128581,5,111.88653231,4,-119.04106281,0\C,1,1.86173914,2,10
1.06713026,3,15.7273952,0\H,12,1.09659488,1,108.67085885,2,170.3438773
5,0\H,12,1.09616329,1,109.8769349,2,-72.11155731,0\H,12,1.09770337,1,1
12.67754637,2,49.61273012,0\C,1,1.87068683,2,100.76073097,3,117.799334
91,0\H,16,1.09725355,1,112.62831547,2,-52.76373635,0\H,16,1.09638417,1
,109.65380512,2,68.61750531,0\H,16,1.09716706,1,108.97280517,2,-173.94
502526,0\\Version=IA32L-G03RevD.01\State=1-A\HF=-612.2025886\MP2=-613.
3528507\RMSD=6.380e-09\Thermal=0.\PG=C01 [X(C7H11P1)]\\@

238-Me^{+}

1\1\GINC-GOLEM\SP\RMP2-FC\6-31+G(2d,p)\C8H14P1(1+)\CHRISTOPH\09-Sep-20

09\0\\#p MP2(FC)/6-31+g(2d,p) scf=tight\\cpd1mesp_2\\1,1\P\C,1,1.77101
052\C,2,1.36661354,1,125.85603263\C,3,1.4585973,2,109.93817724,1,-179.
99887497,0\C,4,1.3536179,3,108.81724846,2,0.00266977,0\C,5,1.50486558,
4,110.22388249,3,-0.00495381,0\H,3,1.08645953,2,126.25938068,1,0.00732
342,0\H,4,1.0840942,3,124.28179029,2,-179.99767485,0\H,5,1.08472307,4,
126.80853256,3,-179.99936974,0\H,6,1.10133324,5,111.25032943,4,120.746
74139,0\H,6,1.10134532,5,111.24268907,4,-120.72475597,0\C,1,1.81787481
,2,110.22094434,3,0.02873272,0\H,12,1.09508223,1,110.32465999,2,-60.49
125346,0\H,12,1.0950482,1,110.3230357,2,60.48370267,0\H,12,1.09576389,
1,109.17552604,2,179.99694265,0\C,1,1.82213017,2,111.06785923,3,120.17
234449,0\H,16,1.09530148,1,109.69024646,2,-60.48884319,0\H,16,1.095665
13,1,110.09004557,2,59.27737619,0\H,16,1.09609501,1,110.11200093,2,179
.1715571,0\C,1,1.82212004,2,111.07671901,3,-120.10441442,0\H,20,1.0961
0479,1,110.10822972,2,-179.1745668,0\H,20,1.09566135,1,110.09187241,2,
-59.27913995,0\H,20,1.09528695,1,109.68802221,2,60.48582972,0\\Version
=AM64L-G03RevD.01\State=1-A\HF=-651.6527424\MP2=-652.9455719\RMSD=7.63
5e-09\Thermal=0.\PG=C01 [X(C8H14P1)]\\@

239

1\1\GINC-NAUTILUS\SP\RMP2-FC\6-31+G(2d,p)\C9H15P1\CHRISTOPH\27-Nov-200
9\0\\#p MP2(FC)/6-31+g(2d,p) scf=tight\\adsp_1\\0,1\P\C,1,1.87541458\C
,2,1.54680968,1,114.02172175\C,3,1.54615573,2,111.02424931,1,-61.09491
781,0\C,4,1.5465244,3,111.74226808,2,66.11418761,0\C,5,1.54671044,4,11
1.03531039,3,-66.12661594,0\H,2,1.09976953,1,107.75858691,6,-73.446394
45,0\H,2,1.09975826,1,107.76846064,6,172.26080787,0\H,3,1.10080635,2,1
08.09746419,1,-179.96108761,0\H,4,1.10029817,3,109.12816153,2,-173.098
62565,0\H,4,1.10039669,3,110.00150727,2,-56.3942929,0\H,5,1.1008407,4,
108.5106826,3,175.24298405,0\H,6,1.09973817,5,110.35127052,4,-60.32909
566,0\H,6,1.09972092,5,110.36427124,4,-177.49205982,0\C,3,1.54635886,2
,111.07670616,1,61.0945717,0\H,15,1.1003033,3,109.10149163,2,173.25726
024,0\H,15,1.100334,3,110.02403917,2,56.55214915,0\C,1,1.87547868,6,97
.65513328,5,49.38904224,0\H,18,1.09978444,1,107.75935836,6,73.53009874
,0\H,18,1.09983028,1,107.7575117,6,-172.20467017,0\C,5,1.54651941,4,10
9.61063187,3,56.91688563,0\H,21,1.10030336,5,110.03028966,4,-179.43672
607,0\H,21,1.10031009,5,109.12805006,4,63.83465209,0\C,21,1.54618624,5
,111.73950341,4,-56.93044122,0\H,24,1.10088376,21,108.5008456,5,175.25
223566,0\\Version=AM64L-G03RevD.01\State=1-A\HF=-690.3283201\MP2=-691.
7990233\RMSD=5.086e-09\Thermal=0.\PG=C01 [X(C9H15P1)]\\@

239-Me$^+$

1\1\GINC-YIN\SP\RMP2-FC\6-31+G(2d,p)\C10H18P1(1+)\CHRISTOPH\27-Nov-200
9\0\\#p MP2(FC)/6-31+g(2d,p) scf=tight\\admesp_1\\1,1\C\C,1,1.56241222
\C,2,1.54847576,1,111.05273503\C,3,1.54920731,2,112.1110915,1,67.73662
893,0\C,4,1.56244607,3,111.07450046,2,-67.7166493,0\H,1,1.09798112,2,1
11.41897285,3,58.22478131,0\H,1,1.09797713,2,111.42322543,3,178.772972
84,0\H,2,1.09696261,1,106.52892585,3,-118.50108238,0\H,3,1.09752319,2,
108.62519183,1,-172.1963868,0\H,3,1.0990426,2,110.37113556,1,-55.74514
289,0\H,4,1.09702598,3,108.98737406,2,175.2242891,0\H,5,1.09792838,4,1
11.45469123,3,-58.23464107,0\H,5,1.09791892,4,111.45297557,3,-178.8468
8784,0\C,2,1.54863975,1,111.12290569,3,122.90227205,0\H,14,1.09755068,
2,108.58444559,1,172.31380822,0\H,14,1.09900053,2,110.37282244,1,55.87
891982,0\C,14,2.56441372,2,96.30299114,1,-35.71855682,0\H,17,1.0979458
4,14,145.74488308,2,-122.57073168,0\H,17,1.09804906,14,91.42216725,2,1
11.87687117,0\C,4,1.54889409,3,110.10152691,2,55.76175447,0\H,20,1.098
92576,4,110.40764493,3,-179.26254205,0\H,20,1.09754823,4,108.62695175,
3,64.24881245,0\C,20,1.54864155,4,112.08984806,3,-55.7767001,0\H,23,1.
09701854,20,108.96757717,4,175.22573641,0\P,5,1.82103759,4,107.2129668
8,3,61.44538525,0\C,25,1.81633013,5,113.94992463,4,179.90200245,0\H,26

,1.09605218,25,109.91327527,5,-60.08356663,0\H,26,1.0960298,25,110.014
30073,5,59.9328437,0\H,26,1.09607033,25,109.93726663,5,179.95703989,0\
\Version=AM64L-G03RevD.01\State=1-A\HF=-729.7770703\MP2=-731.3914502\R
MSD=4.279e-09\Thermal=0.\PG=C01 [X(C10H18P1)]\\@

240

1\1\GINC-CIPCLU08\SP\RMP2-FC\6-31+G(2d,p)\C7H12N1P1S1\C2175\22-Dec-200
9\0\\#p MP2(FC)/6-31+G(2d,p) scf=tight\\ns61sp_15\\0,1\C\H,1,1.0958600
9\H,1,1.09740849,2,108.04619636\H,1,1.09693974,2,108.89857868,3,118.29
822929,0\P,1,1.86537753,2,109.40695653,3,-118.4567863,0\C,5,1.86534943
,1,99.09035196,2,-176.03481597,0\H,6,1.09782047,5,113.12951491,1,50.59
716665,0\H,6,1.09729312,5,108.78166135,1,-69.99719936,0\H,6,1.09601818
,5,109.55167864,1,172.24600011,0\N,5,4.09169456,1,108.4106491,6,122.12
845591,0\C,10,1.39900419,5,13.46501566,1,133.12634165,0\C,11,1.3500601
5,10,124.90234841,5,13.00331338,0\S,12,1.81791279,11,118.15131746,10,5
.45308328,0\C,13,1.7875501,12,97.37100713,11,-32.81864755,0\C,14,1.338
94456,13,120.72216252,12,32.69538733,0\H,11,1.08960517,10,115.10185571
,5,-163.62036133,0\H,15,1.08870831,14,121.4344742,13,-179.70079302,0\H
,14,1.08505696,13,117.52158095,12,-144.83144287,0\C,10,1.45805487,5,13
0.93202193,1,118.85609797,0\H,19,1.09387698,10,109.30474193,5,46.50946
392,0\H,19,1.10265675,10,112.75226054,5,-74.54397147,0\H,19,1.09464158
,10,109.36484785,5,164.37569708,0\\Version=AM64L-G03RevD.01\State=1-A\
HF=-1064.736835\MP2=-1066.216311\RMSD=6.584e-09\Thermal=0.\PG=C01 [X(C
7H12N1P1S1)]\\@

240-Me$^+$

1\1\GINC-CIPCLU09\SP\RMP2-FC\6-31+G(2d,p)\C8H15N1P1S1(1+)\C2175\22-Dec
-2009\0\\#p MP2(FC)/6-31+G(2d,p) scf=tight\\ns61mesp_2\\1,1\C\H,1,1.09
606844\H,1,1.09542918,2,109.1683292\H,1,1.09499525,3,108.93061424,2,-1
19.59835449,0\C,1,2.9604366,4,88.59936384,3,148.75475568,0\H,5,1.09607
429,1,89.52107736,4,107.6527916,0\H,5,1.09481738,1,90.3401958,4,-0.912
45709,0\H,5,1.09525666,1,146.00449697,4,-128.57496664,0\P,5,1.82093035
,1,35.63566073,4,-127.83276922,0\C,9,1.82077009,5,107.9426454,1,-116.3
277473,0\H,10,1.09596199,9,109.7836912,5,-179.77250575,0\H,10,1.095979
72,9,110.14758731,5,60.43452928,0\H,10,1.09529515,9,109.81727278,5,-60
.20948716,0\N,9,4.03653877,5,95.62234129,1,115.69494108,0\C,14,1.36348
999,9,17.69515859,5,18.01884457,0\C,15,1.36629313,14,123.35731936,9,17
.7795177,0\S,16,1.80981815,15,119.76398494,14,11.21676054,0\C,17,1.791
27429,16,96.07028903,15,-35.84952679,0\C,18,1.33627112,17,120.2962092,
16,33.8837412,0\H,15,1.0897782,14,114.99516695,9,-158.93612443,0\H,19,
1.08726111,18,121.60090184,17,178.36815993,0\H,18,1.08469442,17,117.50
345944,16,-145.56817198,0\C,14,1.46680867,9,138.22943819,5,5.73713186,
0\H,23,1.09685851,14,110.72308865,9,-108.14043012,0\H,23,1.09259305,14
,109.47122063,9,12.11975081,0\H,23,1.09470834,14,109.88050645,9,131.12
486288,0\\Version=AM64L-G03RevD.01\State=1-A\HF=-1104.1848206\MP2=-110
5.8089401\RMSD=3.512e-09\Thermal=0.\PG=C01 [X(C8H15N1P1S1)]\\@

241

1\1\GINC-NODE12\SP\RMP2-FC\6-31+G(2d,p)\C11H13P1\ZIP07\17-Sep-2009\0\\
#p MP2(FC)/6-31+g(2d,p) scf=tight\\cpe2sp_22\\0,1\P\C,1,1.86542864\H,2
,1.09657704,1,109.28505393\H,2,1.09572401,1,108.5245494,3,117.45533438
,0\H,2,1.09668267,1,113.03886749,4,121.01142015,0\C,1,1.83580995,2,100
.66859362,4,-67.99547385,0\C,6,1.48318548,1,127.82527878,2,-58.0255877
9,0\C,7,1.34921372,6,109.64502822,1,179.01793707,0\C,8,1.5097311,7,109
.27524873,6,0.10441153,0\C,6,1.35681697,1,123.92577402,2,121.21049888,
0\H,9,1.10225092,8,112.21146315,7,120.59023136,0\H,9,1.10227842,8,112.
23600388,7,-120.28083269,0\C,1,1.83264478,6,100.11851216,10,-135.82363
414,0\C,13,1.35816356,1,129.2412081,6,-122.00347115,0\C,14,1.50973945,

13,109.82917523,1,-174.84333511,0\C,15,1.50826015,14,103.04892816,13,-
0.15155226,0\C,16,1.34874129,15,109.20928812,14,0.39449798,0\H,15,1.10
26228,14,111.9707158,13,120.33135997,0\H,15,1.10243034,14,111.96400621
,13,-120.88438093,0\H,14,1.08504479,13,126.82582664,1,4.72144062,0\H,1
7,1.08632276,16,126.4641919,15,-179.91784788,0\H,16,1.08528963,15,124.
01547728,14,-179.56941399,0\H,10,1.08569952,6,126.06613327,1,0.9252749
8,0\H,8,1.08550174,7,126.86717813,6,179.71227846,0\H,7,1.0859483,6,123
.72133223,1,-1.75496487,0\\Version=AM64L-G03RevD.01\State=1-A\HF=-764.
8161506\MP2=-766.5161042\RMSD=7.444e-09\Thermal=0.\PG=C01 [X(C11H13P1)
]\\@

241-Me$^+$

1\1\GINC-NODE1\SP\RMP2-FC\6-31+G(2d,p)\C12H16P1(1+)\ZIP07\17-Sep-2009\
0\\#p MP2(FC)/6-31+g(2d,p) scf=tight\\cpe2mesp_15\\1,1\P\C,1,1.820501\
H,2,1.09467235,1,109.91339293\H,2,1.09560602,1,109.14130613,3,-118.974
22211,0\H,2,1.09530301,1,110.37235051,4,-119.78880006,0\C,1,1.82282984
,2,107.83660171,3,179.86111652,0\H,6,1.09563505,1,110.05427576,2,-62.0
4983956,0\H,6,1.09499419,1,109.70351103,2,178.25908696,0\H,6,1.0954044
1,1,109.75066595,2,58.3128143,0\C,1,1.78899363,2,109.74319346,6,-118.6
6150089,0\C,10,1.36032713,1,124.98090299,2,-114.40348232,0\C,11,1.5030
1392,10,108.55033909,1,179.30844494,0\C,12,1.50506455,11,103.64596869,
10,0.00378597,0\C,13,1.3496255,12,109.78037224,11,0.02190988,0\H,12,1.
10194281,11,111.50983478,10,121.26595707,0\H,12,1.10201856,11,111.3374
6086,10,-121.09387939,0\C,1,1.78728508,10,110.64320139,11,7.12147673,0
\C,17,1.36036883,1,125.30599065,10,-118.61672455,0\C,18,1.50304419,17,
108.61194244,1,-179.113654,0\C,19,1.50489254,18,103.61351734,17,-0.089
09601,0\C,20,1.34984238,19,109.79083883,18,0.16940117,0\H,19,1.1020649
5,18,111.36953825,17,120.96070319,0\H,19,1.10194874,18,111.4675725,17,
-121.43018107,0\H,18,1.08469591,17,127.43166635,1,0.83512644,0\H,21,1.
08511874,20,126.73551338,19,-179.96420037,0\H,20,1.08429474,19,123.835
00633,18,179.92944791,0\H,11,1.08461243,10,126.94670292,1,-0.64358134,
0\H,14,1.08540829,13,126.44210512,12,179.96418934,0\H,13,1.08431084,12
,123.88216366,11,179.92299704,0\\Version=AM64L-G03RevD.01\State=1-A\HF
=-804.2656904\MP2=-806.1092178\RMSD=4.942e-09\Thermal=0.\PG=C01 [X(C12
H16P1)]\\@

242

1\1\GINC-YANG\SP\RMP2-FC\6-31+G(2d,p)\C11H13P1\CHRISTOPH\15-Sep-2009\0
\\#p MP2(FC)/6-31+g(2d,p) scf=tight\\cpd2sp_23\\0,1\P\C,1,1.82708653\C
,2,1.36051352,1,129.82177579\C,3,1.46953999,2,110.0838967,1,174.472107
81,0\C,4,1.35095048,3,109.34845356,2,-0.33748843,0\C,5,1.50840354,4,10
9.03205139,3,-0.50988442,0\H,3,1.08627682,2,126.11147188,1,-5.36662067
,0\H,4,1.08651069,3,124.23899275,2,179.32155315,0\H,5,1.08517267,4,127
.01751384,3,179.1833628,0\H,6,1.10156732,5,112.37176308,4,122.44369216
,0\H,6,1.10145619,5,111.77685558,4,-118.62057652,0\C,1,1.8284078,2,101
.24870381,3,118.10144358,0\C,12,1.359786,1,123.88476507,2,127.66618573
,0\C,13,1.46836548,12,110.27310924,1,-179.22429652,0\C,14,1.35182547,1
3,109.03391395,12,0.1614053,0\C,15,1.50804377,14,109.29506237,13,-0.12
636815,0\H,13,1.08684579,12,125.22815277,1,0.64090793,0\H,14,1.0861378
9,13,124.5174941,12,-179.82847144,0\H,15,1.08566666,14,126.78401067,13
,-179.86167602,0\H,16,1.10111375,15,112.36766231,14,120.79713321,0\H,1
6,1.10170531,15,111.57942812,14,-120.44824997,0\C,1,1.86547871,2,100.6
7232577,3,13.90504597,0\H,22,1.09588215,1,108.48187168,2,170.33546839,
0\H,22,1.09688983,1,113.28127507,2,49.18439824,0\H,22,1.09655075,1,109
.25571691,2,-72.25738704,0\\Version=AM64L-G03RevD.01\State=1-A\HF=-764
.8171616\MP2=-766.5181583\RMSD=2.818e-09\Thermal=0.\PG=C01 [X(C11H13P1
)]\\@

242-Me⁺

1\1\GINC-YANG\SP\RMP2-FC\6-31+G(2d,p)\C12H16P1(1+)\CHRISTOPH\11-Sep-20
09\0\\#p MP2(FC)/6-31+g(2d,p) scf=tight\\cpd2mesp_4\\1,1\P\C,1,1.77660
921\C,2,1.3663664,1,126.25615327\C,3,1.45900236,2,109.93200744,1,176.6
4518082,0\C,4,1.35385594,3,108.85483781,2,-0.34912996,0\C,5,1.50395852
,4,110.13399086,3,0.06286195,0\H,3,1.08630356,2,125.85444438,1,-3.4714
325,0\H,4,1.0842744,3,124.30017487,2,179.54810819,0\H,5,1.08474675,4,1
26.80849039,3,179.78856037,0\H,6,1.1012458,5,111.40636036,4,120.870308
92,0\H,6,1.10191772,5,111.15046698,4,-120.75295796,0\C,1,1.77661023,2,
111.46201004,3,32.48386033,0\C,12,1.36636689,1,126.25567196,2,32.52800
174,0\C,13,1.45900095,12,109.93209606,1,176.6429434,0\C,14,1.3538563,1
3,108.85476363,12,-0.34594057,0\C,15,1.50395862,14,110.13402619,13,0.0
5886571,0\H,13,1.08630468,12,125.85448967,1,-3.47273845,0\H,14,1.08427
495,13,124.30019515,12,179.54880791,0\H,15,1.08474731,14,126.80847056,
13,179.78740223,0\H,16,1.1019175,15,111.15194832,14,-120.75230471,0\H,
16,1.10124701,15,111.4047333,14,120.87124067,0\C,1,1.82684026,2,111.24
071276,3,-88.57610881,0\H,22,1.09485614,1,109.70624139,2,60.76286361,0
\H,22,1.09621625,1,110.43081483,2,-178.5477444,0\H,22,1.09554054,1,109
.60121245,2,-58.70525011,0\C,1,1.82684194,2,108.36022225,3,155.2109039
8,0\H,26,1.09485599,1,109.70582257,2,-62.10028067,0\H,26,1.09621621,1,
110.43091682,2,58.58868408,0\H,26,1.09554157,1,109.60124717,2,178.4316
7773,0\\Version=AM64L-G03RevD.01\State=1-A\HF=-804.2672936\MP2=-806.11
1394\RMSD=3.646e-09\Thermal=0.\PG=C01 [X(C12H16P1)]\\@

243

1\1\GINC-NAUTILUS\SP\RMP2-FC\6-31+G(2d,p)\C5H9P1\CHRISTOPH\23-Oct-2009
\0\\#p MP2(FC)/6-31+g(2d,p) scf=tight\\c1sp_1\\0,1\P\C,1,1.8589874\C,2
,1.52861797,1,121.54481754\C,3,1.29418713,2,64.9554453,1,-106.67347582
,0\H,2,1.10136047,1,113.98277188,3,150.21188833,0\C,1,1.86787322,2,98.
92568794,4,-159.57085641,0\H,6,1.09654723,1,109.54671273,2,72.82117875
,0\C,1,1.86786492,2,98.92765985,4,100.08565923,0\H,8,1.09705859,1,109.
75644774,2,169.05576456,0\H,4,1.08010166,3,150.12613898,2,179.61561223
,0\H,3,1.08010221,2,144.91842953,1,72.99395947,0\H,8,1.0990672,1,112.5
5166522,2,48.06275276,0\H,8,1.09654699,1,109.54818774,2,-72.82789689,0
\H,6,1.09705862,1,109.75761689,2,-169.06253718,0\H,6,1.09906581,1,112.
55115593,2,-48.06823003,0\\Version=AM64L-G03RevD.01\State=1-A\HF=-535.
2241329\MP2=-536.0879668\RMSD=6.361e-09\Thermal=0.\PG=C01 [X(C5H9P1)]\
\@

243-Me⁺

1\1\GINC-NAUTILUS\SP\RMP2-FC\6-31+G(2d,p)\C6H12P1(1+)\CHRISTOPH\23-Oct
-2009\0\\#p MP2(FC)/6-31+g(2d,p) scf=tight\\c1mesp_1\\1,1\C\H,1,1.0954
8382\H,1,3.20299686,2,97.17485139\P,1,1.81945377,2,109.83556556,3,-49.
20273441,0\C,4,1.81944777,1,108.56285084,2,-178.92662393,0\H,5,1.09599
608,4,109.82343564,1,-61.47204999,0\H,5,1.09583618,4,110.39336669,1,58
.56267175,0\H,5,1.09548185,4,109.83542542,1,178.93665635,0\C,4,1.82192
767,1,109.87074129,5,-120.16731474,0\C,9,1.52672838,4,121.60214735,1,-
150.07668784,0\C,10,1.29275303,9,64.95185258,4,106.70810479,0\C,4,1.81
588506,1,109.41568551,5,119.3569424,0\H,11,1.08022829,10,151.23937537,
9,172.36366495,0\H,10,1.08022788,9,143.43349076,4,-79.45521672,0\H,1,1
.0959957,4,109.82389783,12,-179.16158572,0\H,1,1.09583632,4,110.392469
08,12,60.80297907,0\H,12,1.09545799,4,110.1135543,1,-179.43457995,0\H,
12,1.09547383,4,109.76180607,1,-59.38804626,0\H,12,1.09545608,4,110.11
243618,1,60.6582563,0\\Version=AM64L-G03RevD.01\State=1-A\HF=-574.6745
786\MP2=-575.6821054\RMSD=6.173e-09\Thermal=0.\PG=C01 [X(C6H12P1)]\\@

244

1\1\GINC-NODE4\SP\RMP2-FC\6-31+G(2d,p)\C15H15P1\ZIP07\19-Sep-2009\0\\#

p MP2(FC)/6-31+g(2d,p) scf=tight\\cpe3sp_23\\0,1\P\C,1,1.83403393\C,2,
1.48450662,1,129.11496717\C,3,1.34940278,2,109.61828534,1,-179.7795861
9,0\C,4,1.50945602,3,109.34012461,2,0.02176747,0\C,2,1.35682908,1,122.
73186008,3,-179.61101934,0\H,5,1.10210908,4,112.36232703,3,-120.442427
36,0\H,5,1.10217924,4,112.16465705,3,120.32724165,0\C,1,1.83891774,2,1
02.41725722,6,-133.52690181,0\C,9,1.4838617,1,128.88804407,2,-71.25223
633,0\C,10,1.34934067,9,109.68205431,1,179.30087651,0\C,11,1.50919686,
10,109.26541659,9,-0.02299213,0\C,9,1.35737354,1,122.9737237,2,108.526
80614,0\H,12,1.10210482,11,112.29880956,10,121.18048741,0\H,12,1.10236
17,11,112.11505351,10,-119.6926072,0\C,1,1.83322174,2,101.05969651,6,1
23.10193522,0\C,16,1.48392477,1,122.8663593,2,163.51332676,0\C,17,1.34
931703,16,109.71235256,1,174.24580976,0\C,18,1.50851686,17,109.2328806
1,16,-0.6359419,0\C,16,1.3576161,1,128.59767761,2,-23.93396251,0\H,19,
1.1026831,18,111.83601116,17,-119.67968631,0\H,19,1.10224991,18,112.23
570117,17,121.48299453,0\H,20,1.08506071,16,126.3522522,1,6.08130166,0
\H,18,1.0853374,17,126.72114775,16,179.45829664,0\H,17,1.08632397,16,1
23.87205571,1,-6.26964647,0\H,6,1.08546798,2,125.95002991,1,-0.1452985
,0\H,4,1.08543868,3,126.79236705,2,179.86596351,0\H,3,1.08496604,2,124
.02584376,1,0.32563241,0\H,13,1.0856296,9,126.05143726,1,0.71122197,0\
H,11,1.0853935,10,126.8270046,9,179.79725673,0\H,10,1.08568558,9,123.7
8610842,1,-1.87860034,0\\Version=AM64L-G03RevD.01\State=1-A\HF=-917.42
77757\MP2=-919.6798309\RMSD=7.874e-09\Thermal=0.\PG=C01 [X(C15H15P1)]\
\@

244-Me$^+$

1\1\GINC-NODE25\SP\RMP2-FC\6-31+G(2d,p)\C16H18P1(1+)\ZIP07\21-Sep-2009
\0\\#p MP2(FC)/6-31+g(2d,p) scf=tight\\cpe3mesp_57\\1,1\P\C,1,1.825362
41\H,2,1.09513209,1,109.76736614\H,2,1.09512775,1,109.75452271,3,119.9
8969837,0\H,2,1.09508813,1,109.76515916,4,119.99131975,0\C,1,1.7905170
1,2,108.56877176,5,61.53261861,0\C,6,1.48197304,1,125.64281205,2,53.09
515599,0\C,7,1.34988045,6,108.40346173,1,-179.53072588,0\C,8,1.5056184
3,7,109.7583102,6,0.21558485,0\C,6,1.35989822,1,124.73162959,2,-126.20
000895,0\H,9,1.10193087,8,112.53913111,7,-120.69886298,0\H,9,1.1019362
5,8,112.40910475,7,120.24341719,0\C,1,1.79032335,6,110.37704236,10,114
.91057931,0\C,13,1.4820382,1,125.57215688,6,172.40073402,0\C,14,1.3498
8319,13,108.40740154,1,-179.56146667,0\C,15,1.50562613,14,109.75706438
,13,0.19570012,0\C,13,1.35984914,1,124.80725716,6,-6.9460361,0\H,16,1.
10196164,15,112.4133191,14,120.24735268,0\H,16,1.10191641,15,112.54343
708,14,-120.68741591,0\C,1,1.79038679,13,110.32400233,17,115.30522614,
0\C,20,1.3598302,1,124.72703299,13,-8.07023519,0\C,21,1.5032757,20,108
.60816838,1,179.54986992,0\C,22,1.50565143,21,103.61151692,20,0.127492
49,0\C,23,1.34994428,22,109.75978664,21,-0.20608515,0\H,22,1.101886,21
,111.45902422,20,121.42234803,0\H,22,1.1019863,21,111.41396909,20,-120
.92382494,0\H,21,1.08464275,20,126.88227397,1,-0.31160572,0\H,24,1.085
1546,23,126.66548344,22,179.91723745,0\H,23,1.08437395,22,123.86277074
,21,-179.93587798,0\H,17,1.08462787,13,126.88802037,1,-0.49222504,0\H,
15,1.08437172,14,126.3810736,13,179.97173688,0\H,14,1.08514049,13,124.
92503412,1,0.76930681,0\H,10,1.08463379,6,126.89167625,1,-0.45661183,0
\H,8,1.08437255,7,126.37668294,6,179.95773578,0\H,7,1.08513028,6,124.9
3484686,1,0.76490765,0\\Version=AM64L-G03RevD.01\State=1-A\HF=-956.881
3366\MP2=-959.276851\RMSD=6.333e-09\Thermal=0.\PG=C01 [X(C16H18P1)]\\@

245

1\1\GINC-AZAZEL\SP\RMP2-FC\6-31+G(2d,p)\C15H15P1\CHRISTOPH\15-Sep-2009
\0\\#p MP2(FC)/6-31+g(2d,p) scf=tight\\cpd3sp_15\\0,1\P\C,1,1.82781096
\C,2,1.36003762,1,129.59072854\C,3,1.46871695,2,109.95614711,1,-176.99
072994,0\C,4,1.35224922,3,109.31473558,2,0.25509611,0\C,5,1.50684697,4
,109.08417315,3,0.5604904,0\H,3,1.08601633,2,125.35744886,1,3.14844022

,0\H,4,1.08626908,3,124.30144402,2,-179.31613075,0\H,5,1.08522938,4,12
6.94553408,3,-179.32571321,0\H,6,1.1012616,5,111.98789431,4,118.888011
78,0\H,6,1.10195196,5,112.24660355,4,-122.26935751,0\C,1,1.82665064,2,
102.14157749,3,-95.76285636,0\C,12,1.35987668,1,129.59507934,2,6.94646
33,0\C,13,1.46887393,12,109.96071435,1,-175.99966286,0\C,14,1.35194695
,13,109.33713249,12,0.30803003,0\C,15,1.50737827,14,109.09011473,13,0.
58488735,0\H,13,1.08605773,12,125.42526304,1,4.26787365,0\H,14,1.08629
684,13,124.26429322,12,-179.21372057,0\H,15,1.08523313,14,126.9622607,
13,-179.19207845,0\H,16,1.1012276,15,111.9430354,14,118.70445347,0\H,1
6,1.10173146,15,112.27641471,14,-122.37195498,0\C,1,1.82737611,12,102.
18119648,13,-98.58868531,0\C,22,1.36022062,1,129.63727729,12,7.5328720
7,0\C,23,1.46884573,22,109.94952505,1,-176.4599875,0\C,24,1.3520436,23
,109.32658284,22,0.33997299,0\C,25,1.50710512,24,109.08827319,23,0.542
16315,0\H,23,1.08608585,22,125.43457053,1,3.89117176,0\H,24,1.08626857
,23,124.28053736,22,-179.28588411,0\H,25,1.08522004,24,126.95807757,23
,-179.33339623,0\H,26,1.10125115,25,111.9733564,24,118.88728448,0\H,26
,1.10179378,25,112.24342055,24,-122.20756572,0\\Version=AM64L-G03RevD.
01\State=1-A\HF=-917.4279201\MP2=-919.6826958\RMSD=1.635e-09\Thermal=0
.\PG=C01 [X(C15H15P1)]\\@

245-Me$^+$

1\1\GINC-AZAZEL\SP\RMP2-FC\6-31+G(2d,p)\C16H18P1(1+)\CHRISTOPH\15-Sep-
2009\0\\#p MP2(FC)/6-31+g(2d,p) scf=tight\\cpd3mesp_54\\1,1\P\C,1,1.82
644019\H,2,1.09535406,1,109.9437512\H,2,1.09536264,1,109.87809055,3,12
0.01262915,0\H,2,1.09538182,1,109.87578601,4,119.97474247,0\C,1,1.7811
4699,2,108.2674738,3,178.68139342,0\C,6,1.36478645,1,125.2615834,2,138
.56335428,0\C,7,1.46077175,6,109.86426846,1,178.22256955,0\C,8,1.35370
841,7,108.85639466,6,-0.11339758,0\C,9,1.50529587,8,110.10067057,7,0.1
4741828,0\H,7,1.08656822,6,125.63255063,1,-1.70226531,0\H,8,1.08435811
,7,124.34978783,6,179.67486147,0\H,9,1.08484315,8,126.78013888,7,179.5
6406971,0\H,10,1.10113658,9,111.81094657,8,-121.13356706,0\H,10,1.1014
9739,9,111.13288951,8,120.29154985,0\C,1,1.78070397,6,110.61956882,7,1
9.97222413,0\C,16,1.36497301,1,125.23216214,6,-103.42166827,0\C,17,1.4
6057358,16,109.87369475,1,178.16366366,0\C,18,1.35364808,17,108.851744
88,16,-0.15461014,0\C,19,1.50539134,18,110.1079313,17,0.09511099,0\H,1
7,1.08663481,16,125.65428739,1,-1.79179409,0\H,18,1.08436248,17,124.34
385933,16,179.64674509,0\H,19,1.08486343,18,126.77641454,17,179.574519
71,0\H,20,1.10143225,19,111.18920226,18,120.48978951,0\H,20,1.10121362
,19,111.74512553,18,-120.94320128,0\C,1,1.78084957,16,110.53296857,17,
19.3820232,0\C,26,1.36475056,1,125.21814763,16,-103.74030285,0\C,27,1.
46073121,26,109.86332581,1,178.18094129,0\C,28,1.3536671,27,108.855564
55,26,-0.12171403,0\C,29,1.50545601,28,110.11315332,27,0.15781011,0\H,
27,1.08659623,26,125.64313245,1,-1.73449985,0\H,28,1.08437004,27,124.3
3451883,26,179.66932779,0\H,29,1.08486003,28,126.75960026,27,179.57783
383,0\H,30,1.1015924,29,111.09471844,28,120.20016575,0\H,30,1.10106564
,29,111.87215708,28,-121.19197667,0\\Version=AM64L-G03RevD.01\State=1-
A\HF=-956.8872066\MP2=-959.2832262\RMSD=9.756e-09\Thermal=0.\PG=C01 [X
(C16H18P1)]\\@

246

1\1\GINC-NAUTILUS\SP\RMP2-FC\6-31+G(2d,p)\C7H9P1\CHRISTOPH\23-Oct-2009
\0\\#p MP2(FC)/6-31+g(2d,p) scf=tight\\c2sp_2\\0,1\P\C,1,1.86108637\C,
2,1.52829413,1,121.53740776\C,3,1.2944144,2,65.00455594,1,106.58762974
,0\H,2,1.1009902,1,114.21100663,3,-150.35137163,0\C,1,1.86109636,2,99.
08008613,3,101.66724676,0\C,6,1.52950735,1,121.47886572,2,-161.4024973
9,0\C,7,1.29441717,6,64.90717943,1,106.70691323,0\H,6,1.10098402,1,114
.21111291,2,48.495672,0\C,1,1.87001276,2,98.36746037,3,-158.39978443,0
\H,10,1.09906083,1,112.45251467,2,-50.26578635,0\H,4,1.08019731,3,150.

11208704,2,-179.31010082,0\H,3,1.08010779,2,144.97170604,1,-72.9186917
5,0\H,8,1.08010816,7,150.01937831,6,-179.43894388,0\H,7,1.08020006,6,1
44.97893376,1,-72.68908194,0\H,10,1.09672145,1,109.67811358,2,70.53477
816,0\H,10,1.09671959,1,109.67697019,2,-171.06502418,0\\Version=AM64L-
G03RevD.01\State=1-A\HF=-610.8572562\MP2=-611.9845674\RMSD=8.209e-09\T
hermal=0.\PG=C01 [X(C7H9P1)]\\@

246-Me+

1\1\GINC-NAUTILUS\SP\RMP2-FC\6-31+G(2d,p)\C8H12P1(1+)\CHRISTOPH\24-Oct
-2009\0\\#p MP2(FC)/6-31+g(2d,p) scf=tight\\c2mesp_23\\1,1\C\H,1,1.095
56223\H,1,3.93398817,2,96.32145345\H,1,3.24312808,2,150.46380988,3,-61
.8969365,0\P,1,1.81874255,2,110.45588441,3,-23.09457832,0\C,5,1.821947
28,1,108.52175667,2,-59.17938175,0\H,6,1.09604046,5,110.13663699,1,-63
.25728886,0\H,6,1.09563154,5,109.66727493,1,177.16268863,0\H,6,1.09548
883,5,110.24830216,1,57.11745155,0\C,5,1.82113258,1,109.47601578,6,119
.85731124,0\C,10,1.52751583,5,121.40092376,1,-30.74710827,0\C,11,1.292
54516,10,64.92218282,5,107.16997213,0\C,5,1.82432901,1,109.93566175,6,
-118.62181933,0\C,13,1.52618958,5,121.64940983,1,-86.63266151,0\C,14,1
.2923299,13,65.02241267,5,-106.70122371,0\H,15,1.08003266,14,151.30910
75,13,-173.23522356,0\H,14,1.07995629,13,143.50669861,5,79.03529928,0\
H,12,1.07994658,11,151.10808557,10,173.0272169,0\H,11,1.08012229,10,14
3.48559011,5,-78.44302797,0\H,1,1.09516162,5,109.90208627,10,-59.57813
817,0\H,1,1.09564405,5,109.59982398,10,-179.21867541,0\\Version=AM64L-
G03RevD.01\State=1-A\HF=-650.3128823\MP2=-651.5841453\RMSD=6.862e-09\T
hermal=0.\PG=C01 [X(C8H12P1)]\\@

247

1\1\GINC-NAUTILUS\SP\RMP2-FC\6-31+G(2d,p)\C9H9P1\CHRISTOPH\24-Oct-2009
\0\\#p MP2(FC)/6-31+g(2d,p) scf=tight\\c3sp_97\\0,1\P\C,1,1.85811757\C
,2,1.52948889,1,121.6581251\C,3,1.29417799,2,64.95159139,1,-105.956076
24,0\H,2,1.09978264,1,114.53819497,4,-149.86170127,0\C,1,1.87259661,2,
100.05421075,4,-157.03536294,0\C,6,1.5217326,1,123.51011806,2,44.11416
084,0\C,7,1.29804824,6,65.18551752,1,-111.10970315,0\H,6,1.09869519,1,
110.46241601,2,-169.27868063,0\C,1,1.86278698,2,99.14316161,4,102.9438
6549,0\C,10,1.53003809,1,121.38405107,2,-161.31419323,0\C,11,1.2943141
1,10,64.931056,1,106.7902105,0\H,10,1.09999968,1,114.27808525,2,48.788
72912,0\H,4,1.08011376,3,150.10628196,2,179.16833783,0\H,3,1.08005725,
2,144.94293092,1,73.09684466,0\H,8,1.0807454,7,149.15469613,6,178.9594
9577,0\H,7,1.08103083,6,145.36004697,1,68.12956788,0\H,12,1.08015675,1
1,149.98515732,10,-179.2654581,0\H,11,1.08020745,10,144.9864434,1,-72.
50146525,0\\Version=AM64L-G03RevD.01\State=1-A\HF=-686.4878606\MP2=-68
7.8810375\RMSD=6.760e-09\Thermal=0.\PG=C01 [X(C9H9P1)]\\@

247-Me+

1\1\GINC-EDDY\SP\RMP2-FC\6-31+G(2d,p)\C10H12P1(1+)\CHRISTOPH\24-Oct-20
09\0\\#p MP2(FC)/6-31+g(2d,p) scf=tight\\c3mesp_39\\1,1\C\C,1,1.526674
53\C,2,1.29241944,1,65.01417134\H,1,1.09670022,2,119.66751433,3,105.38
094478,0\H,3,3.04283689,2,94.76480765,1,76.53478961,0\H,1,3.22458374,2
,95.87060631,3,-151.50561193,0\H,3,1.08001226,2,151.23350275,1,-173.74
193283,0\H,2,1.0798632,1,143.6198174,3,-174.67330208,0\P,1,1.82625611,
2,121.71783095,3,-107.12440844,0\C,9,1.82128525,1,108.64848865,2,151.9
0787246,0\H,10,1.09522197,9,110.34997336,1,176.61860259,0\H,10,1.09532
9,9,109.36488939,1,-63.32517991,0\H,10,1.09575998,9,110.297615,1,56.33
389065,0\C,9,1.8235477,1,109.28433849,2,32.11309633,0\C,14,1.52681216,
9,121.39029872,1,-155.83940263,0\C,15,1.29224327,14,65.00738494,9,107.
10130974,0\C,9,1.82321592,1,110.11940348,2,-89.15881945,0\C,17,1.52692
704,9,122.19626234,1,-148.10557332,0\C,18,1.29258843,17,65.00630382,9,
106.12873564,0\H,19,1.08006323,18,151.22632874,17,173.49793966,0\H,18,

1.07992553,17,143.67988881,9,-79.2534142,0\H,16,1.07981454,15,151.1777
3607,14,173.87968508,0\H,15,1.07990386,14,143.56084278,9,-78.16821616,
0\\Version=AM64L-G03RevD.01\State=1-A\HF=-725.950565\MP2=-727.4862523\
RMSD=4.828e-09\Thermal=0.\PG=C01 [X(C10H12P1)]\\@

248

1\1\GINC-STEAK\SP\RMP2-FC\6-31+G(2d,p)\C3H9O1P1\CHRISTOPH\24-Feb-2012\
0\\#p MP2(FC)/6-31+g(2d,p) scf=tight\\pme3osp_1\\0,1\P\C,1,1.83323316\
H,2,1.09605571,1,109.23748223\H,2,1.09605571,1,109.23748223,3,117.9767
3602,0\H,2,1.09678479,1,112.11323572,3,-121.01163199,0\C,1,1.83323294,
2,104.27025933,3,175.55904979,0\H,6,1.09605615,1,109.23748583,2,66.464
2073,0\H,6,1.09605584,1,109.23750535,2,-175.55906804,0\H,6,1.09678479,
1,112.11322655,2,-54.5473951,0\C,1,1.83323294,6,104.27024675,2,109.094
84483,0\H,10,1.09605584,1,109.23750535,6,66.46422321,0\H,10,1.09605615
,1,109.23748583,6,-175.55905213,0\H,10,1.09678479,1,112.11322655,6,-54
.54744973,0\O,1,1.499363,10,114.27391747,6,-125.45257495,0\\Version=AM
64L-G03RevD.01\State=1-A\HF=-534.4991141\MP2=-535.3082206\RMSD=2.055e-
09\Thermal=0.\PG=C01 [X(C3H9O1P1)]\\@

248-Me$^+$

1\1\GINC-STEAK\SP\RMP2-FC\6-31+G(2d,p)\C4H12O1P1(1+)\CHRISTOPH\24-Feb-
2012\0\\#p MP2(FC)/6-31+g(2d,p) scf=tight\\pme3omesp_1\\1,1\P\C,1,1.80
109916\H,2,1.0954318,1,109.57856181\H,2,1.0954318,1,109.57856181,3,119
.48520583,0\H,2,1.09592805,1,110.08539869,4,120.25739709,0\C,1,1.81018
437,2,110.50972092,4,-59.59629765,0\H,6,1.09587314,1,109.76494779,2,59
.13694306,0\H,6,1.09585888,1,110.88608967,2,179.82305021,0\H,6,1.09681
407,1,109.67430575,2,-60.52145388,0\C,1,1.81018437,2,110.50972092,6,-1
21.32219888,0\H,10,1.09585888,1,110.88608967,2,-179.82305021,0\H,10,1.
09587314,1,109.76494779,2,-59.13694306,0\H,10,1.09681407,1,109.6743057
5,2,60.52145388,0\O,1,1.59137548,2,103.82083799,6,119.33890056,0\C,14,
1.4543564,1,124.65945498,2,180.,0\H,15,1.0939215,14,110.31801474,1,61.
52820843,0\H,15,1.08979539,14,105.13896942,1,180.,0\H,15,1.0939215,14,
110.31801474,1,-61.52820843,0\\Version=AM64L-G03RevD.01\State=1-A'\HF=
-573.9011671\MP2=-574.8442206\RMSD=2.607e-09\Thermal=0.\PG=CS [SG(C2H2
O1P1),X(C2H10)]\\@

249

1\1\GINC-EVGENIX\SP\RMP2-FC\6-31+G(2d,p)\C18F15P1\CHRISTOPH\16-Nov-201
1\0\\#p MP2(FC)/6-31+g(2d,p) scf=tight\\f15sp_1\\0,1\P\C,1,4.67376517\
C,2,1.39504834,1,57.63099575\C,3,1.39484468,2,119.21498162,1,-2.183598
28,0\C,4,1.40545103,3,122.67705576,2,-0.31262641,0\C,5,1.40325643,4,11
6.31349279,3,-0.39728535,0\C,6,1.3948822,5,122.21768365,4,0.96095455,0
\C,1,4.67567282,5,107.22825441,4,-145.65608072,0\C,8,1.39520551,1,57.5
8782879,5,93.52572961,0\C,9,1.39464816,8,119.21212727,1,-2.05609901,0\
C,10,1.40515482,9,122.715136,8,-0.31701986,0\C,11,1.40310249,10,116.29
600338,9,-0.47675289,0\C,12,1.39521457,11,122.20757142,10,1.12128752,0
\C,1,4.67548265,5,107.46987969,4,97.50916381,0\C,14,1.39525451,1,57.49
447631,5,-157.19778252,0\C,15,1.39449782,14,119.19393929,1,-1.82093969
,0\C,16,1.40537188,15,122.751429,14,-0.42985792,0\C,17,1.40266408,16,1
16.26564026,15,-0.33675745,0\C,18,1.39535237,17,122.22033479,16,1.0694
54,0\F,16,1.33687658,15,117.36260465,14,179.98295055,0\F,15,1.33172309
,14,120.11254876,1,178.07741165,0\F,14,1.32805746,1,177.09280548,5,-12
1.37409167,0\F,19,1.3322713,18,120.44262344,17,179.92908484,0\F,18,1.3
3391747,17,121.08166919,16,-178.28724057,0\F,4,1.33631755,3,117.308818
02,2,179.84301284,0\F,6,1.33374652,5,121.02594074,4,-178.29639935,0\F,
7,1.33226951,6,120.48615544,5,179.91223329,0\F,2,1.32787473,1,176.9785
3994,5,-177.70833033,0\F,3,1.33179782,2,120.10905251,1,177.6402134,0\F
,10,1.3366204,9,117.37036678,8,179.92950934,0\F,9,1.33175352,8,120.114

27582,1,177.79997207,0\F,8,1.32803768,1,177.07105543,5,132.47885245,0\
F,13,1.33230307,12,120.4461884,11,179.88944008,0\F,12,1.33396124,11,12
1.09046231,10,-178.27362893,0\\Version=AM64L-G03RevD.01\State=1-A\HF=-
2513.8625432\MP2=-2519.5008217\RMSD=2.890e-09\Thermal=0.\PG=C01 [X(C18
F15P1)]\\@

249-Me⁺

1\1\GINC-EVGENIX\SP\RMP2-FC\6-31+G(2d,p)\C19H3F15P1(1+)\CHRISTOPH\17-N
ov-2011\0\\#p MP2(FC)/6-31+g(2d,p) scf=tight\\f15mesp_1\\1,1\P\C,1,1.8
2754442\H,2,1.09243705,1,109.12889448\H,2,1.09234649,1,109.03391975,3,
-120.09592272,0\H,2,1.09260984,1,108.95104513,4,-119.93766212,0\C,1,4.
63102293,2,106.32555149,4,171.94075406,0\C,6,1.39816595,1,61.15872011,
2,-131.99986251,0\C,7,1.39533337,6,119.78735089,1,-1.02841196,0\C,8,1.
40720531,7,121.42485654,6,0.96537591,0\C,9,1.40821322,8,117.17812998,7
,-1.95283625,0\C,10,1.3899474,9,122.35118058,8,1.79484882,0\C,1,4.6314
9845,9,110.79795,8,-15.1296692,0\C,12,1.40128066,1,59.02287384,9,-65.7
5133232,0\C,13,1.38972537,12,119.02079542,1,0.86478654,0\C,14,1.408579
09,13,122.37704533,12,-0.46622006,0\C,15,1.40737,14,117.1666382,13,1.4
6445635,0\C,16,1.39565985,15,121.41215659,14,-1.65646868,0\C,1,4.63076
473,9,112.15526742,8,111.86871032,0\C,18,1.39803347,1,61.12557918,9,-1
3.60324892,0\C,19,1.39542257,18,119.80025786,1,-1.09460295,0\C,20,1.40
73079,19,121.41142771,18,0.88027959,0\C,21,1.40858801,20,117.17764053,
19,-1.71735908,0\C,22,1.38982243,21,122.36145585,20,1.54723715,0\F,20,
1.33074922,19,117.36352425,18,-178.84963822,0\F,19,1.32223435,18,120.1
5215564,1,179.28927209,0\F,18,1.31559002,1,178.7104291,21,172.22978941
,0\F,23,1.32170082,22,120.64039707,21,-179.9383631,0\F,22,1.3379433,21
,119.33170591,20,-177.04601428,0\F,10,1.33800272,9,119.35994601,8,-176
.75558824,0\F,11,1.32176759,10,120.61941597,9,179.99005732,0\F,6,1.315
63403,1,178.71325794,21,-99.58815907,0\F,7,1.32221234,6,120.16492374,1
,179.42618524,0\F,8,1.33086767,7,117.37772487,6,-178.56476958,0\F,14,1
.33810776,13,118.29552931,12,178.08954318,0\F,13,1.32168981,12,120.330
20426,1,-179.67357483,0\F,12,1.3156104,1,178.66926087,21,138.56847409,
0\F,17,1.32223262,16,120.03054736,15,-179.49213033,0\F,16,1.33072192,1
5,121.26403417,14,178.0032282,0\\Version=AM64L-G03RevD.01\State=1-A\HF
=-2553.25774\MP2=-2559.0478864\RMSD=4.074e-09\Thermal=0.\PG=C01 [X(C19
H3F15P1)]\\@

250

1\1\GINC-TOFU\SP\RMP2-FC\6-31+G(2d,p)\C18H12N3O6P1\CHRISTOPH\11-Feb-20
12\0\\#p MP2(FC)/6-31+g(2d,p) scf=tight\\no23sp_2\\0,1\C\C,1,1.3954628
5\C,2,1.39401308,1,118.49903317\C,3,1.40927452,2,121.0264203,1,-0.9144
0345,0\C,4,1.40727062,3,118.88029151,2,0.94864829,0\C,1,1.39473699,2,1
22.08608544,3,0.28373611,0\H,2,1.08428618,1,119.75874395,6,179.6074397
,0\H,3,1.08790918,2,119.16494582,1,178.7141682,0\H,5,1.08627059,4,120.
21754267,3,179.5537028,0\H,6,1.08442347,1,119.64334016,2,-179.92916705
,0\C,5,5.02300373,4,96.14870202,3,163.86939248,0\C,11,1.39430198,5,67.
49476373,4,82.18068856,0\C,12,1.39638518,11,118.78632748,5,-37.2009591
8,0\C,13,1.40641549,12,120.72323189,11,-0.24909009,0\C,14,1.41008632,1
3,118.89816918,12,-0.49097801,0\C,15,1.3933069,14,121.01767487,13,1.10
231981,0\H,12,1.08453891,11,119.65287562,5,142.50804637,0\H,13,1.08634
577,12,119.03391935,11,179.78437359,0\H,15,1.08792883,14,119.83577544,
13,-178.52935056,0\H,16,1.08422356,15,121.75730759,14,179.72473664,0\C
,13,5.01842271,12,140.76717308,11,-159.80137026,0\C,21,1.39583299,13,6
5.82097944,12,98.12239562,0\C,22,1.39371418,21,118.50240087,13,38.4282
0099,0\C,23,1.40975189,22,121.01068286,21,-0.9559636,0\C,24,1.40673296
,23,118.89479836,22,1.09841537,0\C,21,1.39455493,13,67.06785553,12,-11
6.58865968,0\H,22,1.084273,21,119.73357025,13,-142.24678431,0\H,23,1.0
8791189,22,119.18003108,21,178.63733498,0\H,25,1.08629538,24,120.17716

806,23,179.4796148,0\H,26,1.08442594,21,119.65427594,13,142.34256819,0
\P,24,1.85033255,23,116.52389885,22,179.28032324,0\N,1,1.47313569,6,11
8.94667249,5,-179.89945497,0\O,32,1.22948897,1,117.52076509,6,-179.881
98237,0\O,32,1.22951845,1,117.5400288,6,0.19714333,0\N,21,1.47300328,1
3,145.79776221,12,-8.31977925,0\O,35,1.22956545,21,117.51819726,13,88.
73248999,0\O,35,1.22951817,21,117.55466696,13,-91.2455363,0\N,11,1.472
57094,5,145.82066552,4,-168.98686678,0\O,38,1.22959746,11,117.53032902
,5,-91.9036366,0\O,38,1.22964349,11,117.54429843,5,88.04291795,0\\Vers
ion=AM64L-G03RevD.01\State=1-A\HF=-1641.5938549\MP2=-1646.0389274\RMSD
=5.785e-10\Thermal=0.\PG=C01 [X(C18H12N3O6P1)]\\@

250-Me^{+}

1\1\GINC-TOFU\SP\RMP2-FC\6-31+G(2d,p)\C19H15N3O6P1(1+)\CHRISTOPH\11-Fe
b-2012\0\\#p MP2(FC)/6-31+g(2d,p) scf=tight\\no23mesp_2\\1,1\C\C,1,1.3
9528212\C,2,1.39352728,1,118.57786204\C,3,1.41059236,2,119.87547952,1,
-0.00494609,0\C,4,1.40615548,3,120.37052506,2,-0.13091625,0\C,1,1.3923
6547,2,122.69606848,3,0.12960099,0\H,2,1.08475806,1,119.69231802,6,-17
9.96244507,0\H,3,1.08729631,2,118.89883872,1,-179.6583286,0\H,5,1.0860
4473,4,120.8533228,3,179.90085664,0\H,6,1.08461702,1,119.80139341,2,17
9.95227683,0\C,5,5.12338298,4,96.31435168,3,172.02342893,0\C,11,1.3932
6102,5,68.35694755,4,75.21998435,0\C,12,1.39565497,11,118.61344286,5,-
36.34029363,0\C,13,1.40790796,12,119.86613827,11,0.08021829,0\C,14,1.4
0925222,13,120.33893197,12,0.03926514,0\C,11,1.39438071,5,64.43581103,
4,-71.30228509,0\H,12,1.08471868,11,119.71546746,5,143.93806553,0\H,13
,1.08636724,12,119.31561072,11,-179.41515988,0\H,15,1.08664671,14,121.
31501564,13,179.78842322,0\H,16,1.08467772,11,119.7654735,5,-142.61539
488,0\C,13,5.17857953,12,141.70198282,11,-147.67782615,0\C,21,1.395227
79,13,61.16552024,12,85.04590586,0\C,22,1.39346599,21,118.56335051,13,
37.44810285,0\C,23,1.41018562,22,119.90739156,21,0.14451787,0\C,24,1.4
0656948,23,120.36541472,22,-0.37843162,0\C,21,1.39242723,13,70.9782295
4,12,-127.595991,0\H,22,1.08465799,21,119.7421128,13,-142.58180004,0\H
,23,1.08727049,22,118.88038177,21,-179.52071966,0\H,25,1.08601268,24,1
20.87582628,23,-179.87911157,0\H,26,1.08469068,21,119.73985103,13,145.
73364533,0\P,14,1.8123159,13,119.79261355,12,178.50412475,0\C,31,1.824
35528,14,109.12651177,13,143.75339843,0\H,32,1.09492836,31,109.5061488
6,14,-174.87027815,0\H,32,1.09492275,31,110.18479563,14,-54.99277319,0
\H,32,1.09484651,31,110.08369079,14,65.56546825,0\N,1,1.4849554,6,118.
73480677,5,179.93460009,0\O,36,1.22519407,1,116.80331163,6,179.9283423
1,0\O,36,1.22488409,1,116.87511092,6,-0.05559428,0\N,21,1.48496979,13,
147.34713923,12,-13.83523712,0\O,39,1.22518003,21,116.83763021,13,80.4
6515026,0\O,39,1.22498593,21,116.85288254,13,-99.54930134,0\N,11,1.485
07922,5,146.64526644,4,-175.16408226,0\O,42,1.22507588,11,116.84384789
,5,86.08950871,0\O,42,1.22498289,11,116.8345157,5,-93.86156472,0\\Vers
ion=AM64L-G03RevD.01\State=1-A\HF=-1681.0001115\MP2=-1685.5974297\RMSD
=6.608e-09\Thermal=0.\PG=C01 [X(C19H15N3O6P1)]\\@

251

1\1\GINC-SOLARIS\SP\RMP2-FC\6-31+G(2d,p)\C16H20N1P1\CHRISTOPH\29-May-2
010\0\\#p MP2(FC)/6-31+g(2d,p) scf=tight\\xnetsp_36\\0,1\P\C,1,4.68233
697\C,2,1.39773355,1,58.05386452\C,3,1.39864934,2,119.90360175,1,1.188
70544,0\C,4,1.40744068,3,121.1542267,2,-0.31762898,0\C,5,1.40796953,4,
118.15976547,3,0.70177622,0\C,6,1.39773182,5,120.84231546,4,-0.5863796
3,0\H,2,1.08794833,1,178.0332972,5,179.86545527,0\H,3,1.08801629,2,120
.16363173,1,-179.29900048,0\H,4,1.08923769,3,119.6442351,2,179.2349738
1,0\H,6,1.08762364,5,119.81500924,4,178.34763787,0\H,7,1.08824989,6,11
9.77850629,5,-179.98407015,0\C,1,4.66649303,5,104.2530518,4,-146.25865
25,0\C,13,1.39799386,1,59.76933849,5,-136.20309726,0\C,14,1.3982007,13
,120.28359022,1,2.54957082,0\C,15,1.4064793,14,120.84317383,13,0.74582
741,0\C,16,1.40827346,15,118.28922746,14,-1.34302552,0\C,17,1.39741811
,16,120.82790423,15,1.08409939,0\H,13,1.08779531,1,177.67479371,16,179
.62013574,0\H,14,1.0882741,13,120.06110521,1,-177.28327944,0\H,15,1.08
761296,14,120.08040722,13,-179.19767274,0\H,17,1.08820013,16,119.86769
182,15,-179.32197473,0\H,18,1.08815581,17,119.6383741,16,179.63708503,
0\C,1,2.78596629,16,89.10253383,15,-54.92259376,0\H,24,1.10319752,1,10
2.35158741,16,-130.01026642,0\H,24,1.09572854,1,81.16596511,16,-24.875
14134,0\C,24,2.50410596,1,61.19620479,16,136.99124576,0\H,27,1.0989266
6,24,91.55729368,1,-141.97608696,0\H,27,1.09625053,24,138.50061509,1,-
24.65705223,0\C,27,1.53799581,24,98.57744323,1,108.03952812,0\H,30,1.0
9715831,27,111.87315988,24,32.8897413,0\H,30,1.09785409,27,110.1486321
,24,152.58791162,0\H,30,1.09697498,27,110.9365956,24,-87.58557826,0\C,
24,1.53499584,1,141.88875168,16,83.57998597,0\H,34,1.09717947,24,109.4
0661269,1,-153.45437653,0\H,34,1.09701731,24,110.97952258,1,-34.075609
67,0\H,34,1.09638046,24,112.44526921,1,87.29865476,0\N,24,1.4711914,1,
31.98067729,16,119.22558367,0\\Version=AM64L-G03RevD.01\State=1-A\HF=-
1012.767337\MP2=-1015.3904611\RMSD=4.289e-09\Thermal=0.\PG=C01 [X(C16H
20N1P1)]\\@

251-Me$^+$ (N)

1\1\GINC-SOLARIS\SP\RMP2-FC\6-31+G(2d,p)\C17H23N1P1(1+)\CHRISTOPH\21-J
ul-2010\0\\#p MP2(FC)/6-31+g(2d,p) scf=tight\\lcet2mesp_8\\1,1\C\C,1,1
.41404141\C,2,1.41288731,1,118.28563973\C,3,1.39793165,2,120.98809677,
1,-0.63040012,0\C,4,1.39617984,3,119.89624971,2,0.76777832,0\C,1,1.395
07953,2,120.51037834,3,0.01729482,0\H,1,1.08528536,6,118.35545257,5,-1
79.40843851,0\H,3,1.08820452,2,119.65815407,1,179.86193998,0\H,4,1.086
70279,3,119.76163458,2,-179.44013933,0\H,5,1.08694593,4,120.13937045,3
,179.73901715,0\H,6,1.08703569,1,119.59428866,2,-179.90365978,0\C,2,5.
58553335,1,90.98601185,6,-142.99945124,0\C,12,1.39946104,2,45.37751457
,1,16.78523782,0\C,13,1.39611921,12,120.37146911,2,-7.76254999,0\C,14,
1.41111061,13,120.43554248,12,-0.22384767,0\C,15,1.4151447,14,118.4134
8137,13,2.09734597,0\C,16,1.39589032,15,120.89842345,14,-2.66389531,0\
H,12,1.08698334,2,164.06086347,1,42.7034973,0\H,13,1.08694781,12,120.0
5563315,2,171.55449625,0\H,14,1.08415956,13,118.85671512,12,177.502886
46,0\H,16,1.08866452,15,119.93812708,14,175.80001303,0\H,17,1.0867794,
16,119.86319356,15,-179.89561672,0\P,15,1.82825696,14,126.92725869,13,
175.67153377,0\C,23,2.81094488,15,132.07702215,14,89.58963987,0\H,24,1
.09425085,23,77.92875086,15,143.13007003,0\H,24,1.09356822,23,84.60314
376,15,-107.63144156,0\C,24,1.53009926,23,158.14044402,15,27.01050934,
0\H,27,1.09487796,24,112.71496552,23,-72.52855843,0\H,27,1.09481693,24
,112.981284,23,51.45067462,0\H,27,1.09585233,24,107.47280011,23,169.61
227528,0\C,24,2.47985115,23,67.11205171,15,-18.23850192,0\H,31,1.09040
994,24,85.08934008,23,-96.07149761,0\H,31,1.09257987,24,143.28250977,2
3,21.76357951,0\H,31,1.09367487,24,95.5749224,23,155.04819111,0\C,31,2
.43596143,24,61.77218143,23,73.10997805,0\H,35,1.09538079,31,82.660834
81,24,97.80273725,0\H,35,1.09449113,31,87.57483761,24,-154.55104546,0\

C,35,1.52797961,31,151.73674308,24,-21.78228431,0\H,38,1.09408554,35,1
12.78241471,31,-64.7911727,0\H,38,1.09470055,35,112.53252135,31,58.394
8441,0\H,38,1.09611534,35,107.52790664,31,176.2887412,0\N,31,1.4965188
8,24,35.00800278,23,38.35911143,0\\Version=AM64L-G03RevD.01\State=1-A\
HF=-1052.1762161\MP2=-1054.9592051\RMSD=3.366e-09\Thermal=0.\PG=C01 [X
(C17H23N1P1)]\\@

251-Me^{+} (P)

1\1\GINC-AZAZEL\SP\RMP2-FC\6-31+G(2d,p)\C17H23N1P1(1+)\CHRISTOPH\31-Ma
y-2010\0\\#p MP2(FC)/6-31+g(2d,p) scf=tight\\xnetmesp_3\\1,1\C\C,1,1.3
9761638\C,2,1.39779941,1,120.0625068\C,3,1.4057009,2,119.95936279,1,0.
12051589,0\C,4,1.41007922,3,119.76635869,2,-0.09016924,0\C,5,1.3949994
9,4,119.88212137,3,-0.10298244,0\H,1,1.08689229,2,119.94019566,3,-179.
96607339,0\H,2,1.08667933,1,120.21916411,6,179.97957297,0\H,3,1.086343
9,2,119.80810834,1,-179.93870568,0\H,5,1.08792089,4,120.88224939,3,-17
9.231167,0\H,6,1.08668559,5,119.71952405,4,-179.81296401,0\C,4,5.49781
868,3,124.97188191,2,-118.15444876,0\C,12,1.39792825,4,43.28998276,3,5
4.53551948,0\C,13,1.39728307,12,120.12705203,4,-7.50697162,0\C,14,1.40
710091,13,119.74316671,12,-0.09628322,0\C,15,1.41040393,14,119.9655574
3,13,0.10991592,0\C,16,1.39466532,15,119.79109678,14,-0.08630918,0\H,1
2,1.08692472,4,162.15864836,3,76.81703531,0\H,13,1.08658148,12,120.219
23031,4,172.32263943,0\H,14,1.08662127,13,119.82625161,12,179.49422253
,0\H,16,1.08764525,15,120.84124318,14,-179.62670069,0\H,17,1.08660385,
16,119.74313644,15,179.87157225,0\C,4,3.54173691,3,78.78502554,2,152.4
2755454,0\H,23,1.09707715,4,58.3454366,3,-85.48081672,0\H,23,1.0974273
3,4,106.66917516,3,13.09550447,0\C,23,2.52538031,4,78.75479188,3,92.16
783554,0\H,26,1.0934879,23,135.74633855,4,70.81931661,0\H,26,1.0959672
7,23,80.71252607,4,174.91699901,0\C,26,1.53171432,23,107.42904078,4,-7
6.52401634,0\H,29,1.09647445,26,109.58131729,23,-142.53468329,0\H,29,1
.09693737,26,111.87558127,23,-23.41653697,0\H,29,1.09671859,26,111.346
70962,23,98.25220399,0\C,23,1.53228526,4,142.94318419,3,-167.55586112,
0\H,33,1.09639656,23,109.68674038,4,124.21984326,0\H,33,1.0957211,23,1
11.56918843,4,-116.54770444,0\H,33,1.09586455,23,111.47372475,4,4.4648
507,0\P,15,1.80384532,14,120.39124091,13,-179.43565839,0\C,37,1.824486
12,15,108.59834533,14,-134.07482351,0\H,38,1.09431791,37,110.1704278,1
5,-59.6312919,0\H,38,1.0950522,37,109.63755367,15,60.41886446,0\H,38,1
.09487696,37,110.24214598,15,179.80174514,0\N,26,1.48606692,23,31.8920
6749,4,32.11295707,0\\Version=AM64L-G03RevD.01\State=1-A\HF=-1052.2246
997\MP2=-1054.9910001\RMSD=4.730e-09\Thermal=0.\PG=C01 [X(C17H23N1P1)]
\\@

251-BH^{+} (N)

1\1\GINC-PHOENIX\SP\RMP2-FC\6-31+G(2d,p)\C29H31N1P1(1+)\CHRISTOPH\02-A
ug-2011\0\\#p MP2(FC)/6-31+g(2d,p) scf=tight\\lcbhnsp_24\\1,1\C\H,1,1.
09389952\C,1,4.35049813,2,102.84404446\C,3,1.39740065,1,61.34858231,2,
-163.85797493,0\C,4,1.39822737,3,120.52925793,1,0.72598502,0\C,5,1.405
87826,4,120.70282473,3,0.90128616,0\C,6,1.40706958,5,118.06524657,4,-0
.88659936,0\C,7,1.39673277,6,121.26457877,5,0.16939419,0\H,3,1.0868322
,1,178.11563006,6,-179.05593803,0\H,4,1.08725488,3,120.05969214,1,-178
.73537359,0\H,5,1.08552677,4,118.92332078,3,-178.93390156,0\H,7,1.0891
7706,6,119.6649693,5,-178.79306825,0\H,8,1.08694348,7,119.64685858,6,-
179.63540789,0\C,1,4.3490352,6,113.41208618,5,80.5428902,0\C,14,1.3982
9417,1,59.20270712,6,135.32100993,0\C,15,1.39740922,14,119.93072376,1,
0.54585394,0\C,16,1.40779738,15,121.08051558,14,0.83098526,0\C,17,1.40
698613,16,118.30602681,15,-2.32456717,0\C,18,1.39755686,17,120.6156393
7,16,2.29936032,0\H,14,1.08706203,1,178.85373224,17,170.72446362,0\H,1
5,1.08703689,14,120.30963982,1,-178.42592213,0\H,16,1.08848014,15,119.
0247868,14,-178.26039927,0\H,18,1.08436609,17,120.19568179,16,-176.682

81126,0\H,19,1.08696489,18,119.47884259,17,-179.98407665,0\C,1,2.49836
903,17,96.21503932,16,-58.88468935,0\H,25,1.09057838,1,90.3488613,17,-
5.20102193,0\H,25,1.09538948,1,79.75023868,17,102.24510709,0\C,25,2.50
061096,1,60.91161217,17,-157.97585565,0\H,28,1.09138491,25,141.4773365
8,1,39.45062321,0\H,28,1.08879754,25,90.04540658,1,157.91219957,0\C,25
,1.52739663,1,153.16855896,17,-145.16375525,0\H,31,1.09245271,25,112.4
9567076,1,77.72883272,0\H,31,1.09651852,25,106.9750487,1,-165.06647227
,0\H,31,1.09439536,25,113.20862166,1,-47.20831858,0\C,28,1.53233961,25
,95.06456549,1,-92.38184551,0\H,35,1.09495644,28,113.13675433,25,-30.6
3672464,0\H,35,1.09291156,28,112.96281612,25,94.13181717,0\H,35,1.0964
527,28,106.90961524,25,-148.37294653,0\P,28,2.92580032,25,65.98846405,
1,73.71087218,0\C,39,4.64817398,28,125.94148399,25,31.03659806,0\C,40,
1.39860771,39,58.73603257,28,-93.65063843,0\C,41,1.39559604,40,120.221
38581,39,2.50055517,0\C,42,1.41418029,41,120.72008002,40,1.85442055,0\
C,43,1.41178176,42,118.31299368,41,-3.16047524,0\C,44,1.39691773,43,12
0.57476344,42,2.2230375,0\H,40,1.08704483,39,176.95371677,28,-163.2638
5742,0\H,41,1.08701854,40,120.25459653,39,-176.33320846,0\H,42,1.08603
099,41,118.96440033,40,-177.23129451,0\H,44,1.08442918,43,120.58042604
,42,-175.99863394,0\H,45,1.0870915,44,119.43773042,43,-179.61216758,0\
C,39,4.64371461,28,79.02129374,25,136.4345111,0\C,51,1.4013838,39,63.0
9039311,28,-66.69548248,0\C,52,1.39472182,51,120.28551163,39,0.7579910
6,0\C,53,1.41324521,52,120.60435636,51,0.5196263,0\C,54,1.41098608,53,
118.28107164,52,-0.05676926,0\C,51,1.39571782,39,56.8524306,28,112.229
53489,0\H,51,1.08702356,39,176.83814671,28,129.9451356,0\H,52,1.087145
96,51,120.0426312,39,-178.93449471,0\H,53,1.08663122,52,118.76071749,5
1,-179.50759025,0\H,55,1.08815492,54,119.80149799,53,-179.70972963,0\H
,56,1.08680112,51,120.30974498,39,178.69789202,0\N,28,1.52206957,25,34
.77576757,1,37.82688641,0\\Version=AM64L-G03RevD.01\State=1-A\HF=-1511
.268219\MP2=-1515.7606829\RMSD=6.701e-09\Thermal=0.\PG=C01 [X(C29H31N1
P1)]\\@

251-BH$^+$ (P)

1\1\GINC-NODE4\SP\RMP2-FC\6-31+G(2d,p)\C29H31N1P1(1+)\ZIP07\23-Jan-201
1\0\\#p MP2(FC)/6-31+G(2d,p) scf=tight\\lcbhsp_20\\1,1\C\H,1,1.0984764
3\P,1,1.88464885,2,102.50970183\C,1,4.35561136,3,110.11633259,2,111.81
508842,0\C,4,1.39803874,1,59.40925727,3,-84.28898811,0\C,5,1.39721512,
4,120.11473911,1,0.18031583,0\C,6,1.40615667,5,120.75072763,4,0.327663
2,0\C,7,1.4045711,6,118.67969771,5,-0.8518616,0\C,8,1.3976129,7,120.49
102478,6,0.67756474,0\H,4,1.08704117,1,179.27573986,7,155.17452656,0\H
,5,1.08709026,4,120.25917448,1,-179.00227868,0\H,6,1.08734886,5,119.16
332318,4,-179.10487274,0\H,8,1.0853392,7,120.26951536,6,-177.79281551,
0\H,9,1.08715433,8,119.44227665,7,-179.36339593,0\C,1,4.3436603,7,113.
6930322,6,140.96069014,0\C,15,1.39758368,1,60.77510926,7,81.44344679,0
\C,16,1.39906741,15,120.37252152,1,0.85804124,0\C,17,1.40471748,16,120
.47408504,15,0.06948993,0\C,18,1.40521012,17,118.65765392,16,-0.139816
59,0\C,19,1.3968339,18,120.84189988,17,0.07718284,0\H,15,1.08685597,1,
178.71506286,18,-176.63600837,0\H,16,1.08722358,15,120.11718386,1,-179
.01922384,0\H,17,1.08630558,16,119.02253885,15,179.92186056,0\H,19,1.0
8907937,18,119.72372071,17,-179.14996797,0\H,20,1.08701226,19,119.6818
2458,18,-179.78263786,0\C,3,4.62861407,1,108.51813009,18,-161.23767378
,0\C,26,1.40012891,3,59.07232343,1,50.31769655,0\C,27,1.39482539,26,12
0.01709126,3,-1.90695771,0\C,28,1.41136236,27,120.37897414,26,-0.53554
428,0\C,29,1.40578751,28,119.1556514,27,1.73232738,0\C,26,1.39682743,3
,60.92386147,1,-128.39905561,0\H,26,1.08690699,3,178.13197997,1,109.08
647518,0\H,27,1.08683633,26,120.23490706,3,177.90375427,0\H,28,1.08845
242,27,118.81361083,26,179.19785345,0\H,30,1.08572623,29,120.54461406,
28,177.93718227,0\H,31,1.08680231,26,120.19110045,3,-177.91015559,0\C,
3,4.61515717,1,110.24524443,18,75.07418019,0\C,37,1.3989941,3,59.82652

016,1,-109.28283767,0\C,38,1.39612776,37,120.13426692,3,-0.61487004,0\
C,39,1.40862687,38,120.02410687,37,-0.41077691,0\C,40,1.41048086,39,11
9.63674947,38,0.85619735,0\C,41,1.39683295,40,119.80511145,39,-0.69300
148,0\H,37,1.08701219,3,179.44649885,1,-41.9826188,0\H,38,1.08680094,3
7,120.21574196,3,179.29590698,0\H,39,1.08613746,38,120.11439829,37,178
.8952816,0\H,41,1.08599169,40,120.80457133,39,178.95038205,0\H,42,1.08
670144,41,119.48483387,40,179.97768783,0\C,3,2.65670559,1,141.80087288
,18,-54.39716098,0\H,48,1.0960305,3,76.98071029,1,168.10280939,0\H,48,
1.10069239,3,102.80730786,1,-87.26010641,0\C,48,2.51365379,3,64.733885
99,1,-2.03585108,0\H,51,1.09905779,48,77.66706893,3,-133.31939431,0\H,
51,1.09398544,48,132.91580806,3,-32.46695944,0\C,48,1.53041501,3,142.7
4334792,1,64.26563796,0\H,54,1.09633071,48,108.62751725,3,147.12659367
,0\H,54,1.09450726,48,112.2268145,3,-94.28471597,0\H,54,1.09580761,48,
111.63248271,3,27.88197152,0\C,51,1.53216594,48,113.45493427,3,119.209
14785,0\H,58,1.09708371,51,109.03041362,48,143.44492873,0\H,58,1.09380
19,51,110.9914151,48,-97.44910098,0\H,58,1.09356298,51,112.01106169,48
,24.03817962,0\N,51,1.49288497,48,32.76229528,3,21.09553941,0\\Version
=AM64L-G03RevD.01\State=1-A\HF=-1511.3283494\MP2=-1515.8004401\RMSD=8.
383e-09\Thermal=0.\PG=C01 [X(C29H31N1P1)]\\@

252

1\1\GINC-CIPCLU07\SP\RMP2-FC\6-31+G(2d,p)\C18H15O3P1\C2175\26-Aug-2010
\0\\#p MP2(FC)/6-31+G(2d,p) scf=tight\\k1sp_80\\0,1\P\O,1,1.65960987\O
,1,1.66294389,2,102.127239\O,1,1.66292497,2,102.12995381,3,94.17506199
,0\C,2,4.18279133,1,126.88815908,4,47.08619433,0\C,5,1.39858801,2,59.8
5622361,1,89.44045473,0\C,6,1.39787068,5,120.43351127,2,-1.08715825,0\
C,2,1.39632807,1,125.03038958,4,47.08531255,0\C,8,1.39710275,2,119.431
69994,1,-91.54965303,0\C,9,1.39787676,8,119.18209767,2,-176.78734406,0
\H,5,1.08730396,2,179.00709961,1,179.99347056,0\H,6,1.08760233,5,120.1
6344452,2,178.90838401,0\H,7,1.0862025,6,121.10452482,5,179.85238494,0
\H,9,1.08620216,8,119.7139737,2,3.0444077,0\H,10,1.08760138,9,119.4026
7084,8,179.97320773,0\C,3,4.17606288,1,122.92019918,2,-93.38575544,0\C
,16,1.39672532,3,60.79830948,1,36.59653314,0\C,17,1.39975543,16,120.64
329408,3,-0.03873017,0\C,3,1.38124225,1,120.80426909,2,-92.69253363,0\
C,19,1.39987684,3,116.69087156,1,-144.21222659,0\C,20,1.39540008,19,11
9.51310591,3,-178.48321962,0\H,16,1.08705972,3,178.89251365,1,-161.412
63179,0\H,17,1.08762005,16,120.19271109,3,179.43763901,0\H,18,1.086987
19,17,120.39013076,16,178.50520179,0\H,20,1.08629192,19,118.85633208,3
,1.50096249,0\H,21,1.08765913,20,119.3817225,19,179.89920379,0\C,4,4.1
7606147,1,122.92522555,2,93.36605433,0\C,27,1.40032809,4,58.70501794,1
,143.20961818,0\C,28,1.39539812,27,120.52705715,4,-0.65345073,0\C,4,1.
38123615,1,120.80843034,2,92.67245467,0\C,30,1.40129082,4,122.72267427
,1,-37.57682078,0\C,27,1.39672575,4,60.79878451,1,-36.57824307,0\H,27,
1.08705943,4,178.89156658,1,161.4123812,0\H,28,1.08765888,27,120.09054
854,4,179.64823312,0\H,29,1.08629268,28,121.62993504,27,-179.61595424,
0\H,31,1.08698628,30,120.36335494,4,0.33939519,0\H,32,1.08761907,27,12
0.19297823,4,-179.43765194,0\\Version=AM64L-G03RevD.01\State=1-A\HF=-1
255.7988128\MP2=-1259.0450366\RMSD=5.731e-09\Thermal=0.\PG=C01 [X(C18H
15O3P1)]\\@

252-Me$^+$

1\1\GINC-CIPCLU08\SP\RMP2-FC\6-31+G(2d,p)\C19H18O3P1(1+)\C2175\03-Sep-
2010\0\\#p MP2(FC)/6-31+G(2d,p) scf=tight\\k1mesp_98\\1,1\O\O,1,2.6076
0161\O,2,2.60266305,1,60.0848788\C,1,4.18649519,2,127.02446057,3,-76.2
6362049,0\C,4,1.3989797,1,60.18107705,2,-51.46841529,0\C,5,1.39957215,
4,120.48928603,1,0.86387796,0\C,6,1.39113724,5,117.81779724,4,0.127549
89,0\C,7,1.39201507,6,123.2198778,5,-0.86993081,0\C,8,1.3993235,7,118.
0207256,6,1.00486644,0\H,4,1.08649616,1,179.05322136,7,-176.21907979,0

\H,5,1.08658867,4,120.22436733,1,-178.75719093,0\H,6,1.08558615,5,121.
50014121,4,-179.13887691,0\H,8,1.08621033,7,120.66231048,6,-178.397605
86,0\H,9,1.08655761,8,119.39655383,7,-179.87143955,0\C,2,4.1864108,1,9
7.46878529,7,54.32733299,0\C,15,1.39942851,2,60.046046,1,69.54512116,0
\C,16,1.39929114,15,120.26362263,2,-0.64096323,0\C,17,1.39193384,16,11
8.03146714,15,-0.43326614,0\C,18,1.39108058,17,123.21680957,16,0.94884
67,0\C,15,1.39897599,2,60.12184722,1,-110.03135202,0\H,15,1.08649906,2
,179.12107587,1,155.43523533,0\H,16,1.08654961,15,120.33577084,2,178.8
1683982,0\H,17,1.08622761,16,121.31601138,15,179.06480325,0\H,19,1.085
64139,18,120.66825189,17,178.5478299,0\H,20,1.08659496,15,120.2168704,
2,-178.81348614,0\C,3,4.18671935,2,97.24573994,1,128.61114295,0\C,26,1
.39941576,3,59.983251,2,70.60027202,0\C,27,1.39925313,26,120.25514621,
3,-0.7402309,0\C,28,1.39187846,27,118.03404158,26,-0.36614128,0\C,29,1
.39093861,28,123.22585523,27,0.9830672,0\C,26,1.39890313,3,60.1915101,
2,-108.97025606,0\H,26,1.08650262,3,179.05498383,2,152.84218075,0\H,27
,1.08655931,26,120.33604646,3,178.75873685,0\H,28,1.0861768,27,121.335
87791,26,179.04844992,0\H,30,1.08557577,29,120.67843874,28,178.4257723
7,0\H,31,1.08657412,26,120.22486765,3,-178.74471674,0\P,1,1.57460462,7
,128.05925767,6,-96.72936302,0\C,37,1.78440156,1,107.06050829,7,-143.3
5900973,0\H,38,1.09479958,37,109.40872439,1,-169.59413228,0\H,38,1.094
80428,37,109.40472189,1,-49.58432598,0\H,38,1.09481212,37,109.40384357
,1,70.40660152,0\\Version=AM64L-G03RevD.01\State=1-A\HF=-1295.2336366\
MP2=-1298.6230444\RMSD=6.783e-09\Thermal=0.\PG=C01 [X(C19H18O3P1)]\\@

252-BH$^+$

1\1\GINC-IBLIS\SP\RMP2-FC\6-31+G(2d,p)\C31H26O3P1(1+)\CHRISTOPH\05-Oct
-2010\0\\#p MP2(FC)/6-31+g(2d,p) scf=tight\\k1bhsp_1\\1,1\C\H,1,1.0997
7651\C,1,4.3469254,2,106.92336854\C,3,1.3982554,1,60.25353926,2,152.05
003992,0\C,4,1.39880851,3,120.34443083,1,0.13287448,0\C,5,1.40299072,4
,120.15178903,3,-0.1230724,0\C,6,1.405429,5,119.25507792,4,-0.22420148
,0\C,7,1.39684732,6,120.43913879,5,0.33110387,0\H,3,1.08700427,1,179.3
3914905,6,-134.29722797,0\H,4,1.08709819,3,120.16418506,1,-179.2350939
2,0\H,5,1.08597922,4,119.59503315,3,-178.59356979,0\H,7,1.08821122,6,1
19.95006984,5,-179.07977258,0\H,8,1.08709801,7,119.70504509,6,-179.223
92491,0\C,1,4.34012119,6,116.27752998,5,32.61695042,0\C,14,1.39853483,
1,58.95771577,6,98.86483325,0\C,15,1.39638223,14,120.13040414,1,0.6963
7944,0\C,16,1.40379417,15,120.51709157,14,-0.45100808,0\C,17,1.4053854
7,16,119.09186181,15,0.28106253,0\C,14,1.39758608,1,60.77993804,17,23.
79632013,0\H,14,1.08694444,1,178.97870575,17,175.15940668,0\H,15,1.086
93957,14,120.22229258,1,-179.44859171,0\H,16,1.0882866,15,119.65494465
,14,179.83328948,0\H,18,1.0877595,17,120.73045407,16,-179.99900148,0\H
,19,1.08707514,14,120.2042159,1,179.40381993,0\P,1,1.83059914,17,111.8
4128429,16,-133.71837656,0\O,25,1.57875248,1,104.90907204,17,163.77883
941,0\O,25,1.5725164,1,111.91392949,17,-73.3959536,0\O,25,1.58507775,1
,113.06858597,17,42.1120546,0\C,27,4.18202528,25,130.7015751,1,-121.53
595481,0\C,29,1.39912378,27,59.91090136,25,-96.69245482,0\C,30,1.39827
168,29,120.30180218,27,0.84624295,0\C,31,1.39086652,30,117.93871037,29
,0.34883375,0\C,32,1.39060388,31,123.36014541,30,-1.04898854,0\C,29,1.
39868982,27,60.28607378,25,82.76092225,0\H,29,1.08661203,27,179.020477
31,25,-175.01035412,0\H,30,1.08656053,29,120.28947037,27,-178.81005556
,0\H,31,1.08597933,30,121.67584924,29,-179.37519432,0\H,33,1.08613741,
32,120.67860395,31,-178.21834386,0\H,34,1.0867579,29,120.22163869,27,1
78.64872404,0\C,26,4.18815327,25,131.38934542,1,175.42818267,0\C,40,1.
39930334,26,59.16035648,25,-125.75050131,0\C,41,1.39749512,40,120.3567
195,26,0.91959173,0\C,42,1.39119188,41,118.18137155,40,0.12608037,0\C,
43,1.39264874,42,122.98796884,41,-0.73095832,0\C,40,1.39753333,26,60.8
8759039,25,53.60941929,0\H,40,1.08647661,26,178.77499088,25,-172.21012
421,0\H,41,1.0866148,40,120.27570424,26,-178.98854785,0\H,42,1.0856610

3,41,121.82402385,40,179.72502408,0\H,44,1.08528408,43,121.46553642,42
,-178.7365975,0\H,45,1.08670102,40,120.26302014,26,178.78377245,0\C,28
,4.18686479,25,126.99304033,1,40.59067575,0\C,51,1.3998017,28,59.19782
342,25,-125.67353982,0\C,52,1.39730199,51,120.43608011,28,0.9920538,0\
C,53,1.39192981,52,118.2050229,51,-0.19983089,0\C,54,1.39410637,53,122
.77987086,52,-0.24786309,0\C,51,1.3974148,28,60.88526221,25,53.6032142
9,0\H,51,1.08646493,28,178.78079288,25,-173.541146,0\H,52,1.08656768,5
1,120.21915098,28,-179.08320741,0\H,53,1.08550052,52,121.83739088,51,1
79.36106477,0\H,55,1.08650858,54,121.26224164,53,-178.29291887,0\H,56,
1.08668906,51,120.30751831,28,178.72562355,0\\Version=AM64L-G03RevD.01
\State=1-A\HF=-1754.3524913\MP2=-1759.435124\RMSD=9.336e-09\Thermal=0.
\PG=C01 [X(C31H26O3P1)]\\@

252-TT$^+$

1\1\GINC-CIPCLU06\SP\RMP2-FC\6-31+G(2d,p)\C37H30O3P1(1+)\LICCH\14-Sep-
2011\0\\#p MP2(FC)/6-31+G(2d,p) scf=tight\\k1ttsp_56\\1,1\C\P,1,1.8800
7131\C,1,4.38527046,2,106.33588962\C,3,1.39539457,1,59.65972922,2,121.
77500477,0\C,4,1.39977466,3,120.57282656,1,-0.52749161,0\C,5,1.4025006
,4,120.70586496,3,0.01619383,0\C,6,1.41109997,5,118.28719668,4,1.07238
577,0\C,7,1.3964999,6,120.82398552,5,-1.34504638,0\H,3,1.08708444,1,17
9.47620825,6,165.84089017,0\H,4,1.08717557,3,120.20321937,1,179.308688
38,0\H,5,1.08418882,4,119.05206758,3,179.28541116,0\H,7,1.08548459,6,1
20.29113644,5,176.88296523,0\H,8,1.08765853,7,119.3724692,6,179.552546
3,0\C,1,4.38538611,6,111.23411595,5,4.74328558,0\C,14,1.39565229,1,59.
30652787,6,-112.84526474,0\C,15,1.39902832,14,120.62545987,1,-1.049151
81,0\C,16,1.40448851,15,120.80736082,14,0.10332409,0\C,17,1.40927094,1
6,118.07535194,15,1.19925736,0\C,18,1.39795203,17,120.95010122,16,-1.6
5795246,0\H,14,1.08697803,1,179.21636836,17,169.55884229,0\H,15,1.0873
0211,14,120.19714638,1,178.7311764,0\H,16,1.08424315,15,118.77652949,1
4,179.29447478,0\H,18,1.0866406,17,120.53679612,16,176.26453991,0\H,19
,1.08762922,18,119.27750121,17,179.71900563,0\C,1,4.38115243,17,109.54
393544,16,14.92582589,0\C,25,1.39862684,1,59.73341275,17,67.44151628,0
\C,26,1.39747918,25,120.30060167,1,0.83084408,0\C,27,1.40892075,26,120
.88195705,25,0.78803234,0\C,28,1.40375504,27,118.27549516,26,-1.548857
54,0\C,25,1.39595107,1,59.50548742,28,90.09119737,0\H,25,1.0870351,1,1
78.99982433,28,172.24958554,0\H,26,1.08763722,25,120.34581445,1,-178.0
9710761,0\H,27,1.08690978,26,118.67331375,25,-176.91799979,0\H,29,1.08
444043,28,120.49722727,27,-178.19985033,0\H,30,1.08733516,25,120.21456
389,1,178.57422625,0\O,2,1.58354731,1,109.105991,28,-49.59481847,0\O,2
,1.58452842,1,108.80585722,28,-168.7884517,0\O,2,1.58388509,1,109.3465
828,28,71.18213805,0\C,38,4.18847659,2,129.4909675,1,142.53520071,0\C,
39,1.39964092,38,60.141922,2,95.84647217,0\C,40,1.39851652,39,120.5391
9036,38,-0.69622297,0\C,41,1.39299327,40,117.96432919,39,-0.2116898,0\
C,42,1.39210042,41,122.95274135,40,1.28688117,0\C,39,1.39871903,38,59.
89104156,2,-84.15971093,0\H,39,1.08677888,38,179.43682863,2,-158.89636
241,0\H,40,1.08684751,39,120.19984241,38,179.09931681,0\H,41,1.0853939
4,40,121.43564112,39,179.35047318,0\H,43,1.08609182,42,120.25595146,41
,178.0486088,0\H,44,1.08703768,39,120.30696748,38,-178.97667087,0\C,37
,4.18532785,2,129.58344606,1,138.39202279,0\C,50,1.39875029,37,60.1267
5804,2,93.55728389,0\C,51,1.3998819,50,120.50845455,37,-0.99100076,0\C
,52,1.39156341,51,118.02529432,50,-0.18498298,0\C,53,1.39304547,52,122
.88353909,51,1.02628289,0\C,54,1.39842285,53,118.18543835,52,-1.183153
89,0\H,50,1.08682403,37,179.01456553,2,-169.40019794,0\H,51,1.08696172
,50,120.30105835,37,178.59035981,0\H,52,1.08575671,51,121.26569989,50,
179.1828952,0\H,54,1.08615244,53,120.16849929,52,178.48429969,0\H,55,1
.08696772,54,119.34156209,53,-179.94681326,0\C,36,4.1842665,2,130.7215
1531,1,140.59897846,0\C,61,1.39915568,36,60.04177722,2,95.95695924,0\C
,62,1.39859348,61,120.44483784,36,-0.93430922,0\C,63,1.39260834,62,118

.04330705,61,-0.30193497,0\C,64,1.39203685,63,122.94634085,62,1.299732
64,0\C,65,1.39919224,64,118.10070358,63,-1.48509007,0\H,61,1.08681987,
36,178.9770562,2,-174.68905711,0\H,62,1.08693337,61,120.2267926,36,178
.62888517,0\H,63,1.0856902,62,121.61960146,61,179.47757638,0\H,65,1.08
641,64,120.37607862,63,178.03818219,0\H,66,1.08693008,65,119.34610682,
64,-180.,0\\Version=AM64L-G03RevD.01\State=1-A\HF=-1983.8921149\MP2=-1
989.8398828\RMSD=9.552e-09\Thermal=0.\PG=C01 [X(C37H30O3P1)]\\@

253

1\1\GINC-CIPCLU04\SP\RMP2-FC\6-31+G(2d,p)\C12H11P1\C2175\11-Aug-2010\0
\\#p MP2(FC)/6-31+G(2d,p) scf=tight\\xhsp_12\\0,1\P\H,1,1.42173804\C,1
,4.66401977,2,97.44950258\C,3,1.39745671,1,60.67950761,2,0.00672703,0\
C,4,1.39914883,3,120.16412271,1,1.15606399,0\C,5,1.40640727,4,120.7360
9999,3,-0.47957868,0\C,6,1.40791746,5,118.45220203,4,-0.21520624,0\C,7
,1.3962755,6,120.82102021,5,0.95542009,0\H,3,1.08768847,1,178.8414388,
6,179.9846095,0\H,4,1.08802984,3,120.12288304,1,-179.18865658,0\H,5,1.
08837505,4,119.45241267,3,179.53759893,0\H,7,1.08881298,6,119.63896862
,5,-179.06361892,0\H,8,1.08790102,7,119.79615871,6,179.56320558,0\C,1,
4.66983185,6,104.49587965,5,-102.4188505,0\C,14,1.39683608,1,58.656371
02,6,-145.89434623,0\C,15,1.39963973,14,119.95382391,1,0.49761906,0\C,
16,1.40472538,15,120.75702096,14,-0.52939891,0\C,17,1.40632406,16,118.
74963428,15,0.7181502,0\C,18,1.39663557,17,120.53064489,16,-0.42362218
,0\H,14,1.08786941,1,178.78408518,6,-152.67674964,0\H,15,1.08790913,14
,120.18541009,1,-179.91376133,0\H,16,1.08858625,15,119.78099591,14,179
.34797872,0\H,18,1.08792569,17,119.74440983,16,179.01910419,0\H,19,1.0
8811044,18,119.78210416,17,179.96983528,0\\Version=AM64L-G03RevD.01\St
ate=1-A\HF=-801.5881275\MP2=-803.3953969\RMSD=5.556e-09\Thermal=0.\PG=
C01 [X(C12H11P1)]\\@

253-Me$^+$

1\1\GINC-CIPCLU06\SP\RMP2-FC\6-31+G(2d,p)\C13H14P1(1+)\C2175\11-Aug-20
10\0\\#p MP2(FC)/6-31+G(2d,p) scf=tight\\xhmesp_6\\1,1\C\C,1,1.3997752
8\C,2,1.3950134,1,120.10783162\C,3,1.4094851,2,119.52524699,1,-0.10214
528,0\C,4,1.40829769,3,120.31944307,2,0.03423247,0\C,5,1.39592122,4,11
9.52212408,3,0.09939666,0\H,1,1.08680671,6,119.78091552,5,179.95775194
,0\H,2,1.08644917,1,120.2346697,6,179.83479566,0\H,3,1.08726909,2,119.
82102628,1,179.38151677,0\H,5,1.08665205,4,121.213871,3,179.90552487,0
\H,6,1.08642162,5,119.62854023,4,179.73265832,0\C,3,5.42413678,2,143.8
2713493,1,134.72168018,0\C,12,1.40085637,3,52.50480392,2,-65.57988823,
0\C,13,1.39458113,12,120.09695033,3,-34.85102793,0\C,14,1.40936497,13,
119.38954806,12,-0.1038241,0\C,15,1.40782661,14,120.51374304,13,0.2397
836,0\C,16,1.39653606,15,119.46543637,14,-0.15665426,0\H,12,1.08682609
,3,150.0266302,2,19.86268571,0\H,13,1.08646329,12,120.17019133,3,144.9
604956,0\H,14,1.08799823,13,119.79526537,12,179.17976332,0\H,16,1.0879
9104,15,120.84526267,14,179.54604397,0\H,17,1.0863951,16,119.6998327,1
5,179.72735068,0\P,15,1.79460636,14,119.974861,13,-177.45685482,0\C,23
,1.82055062,15,111.81367787,14,73.79695495,0\H,24,1.09511823,23,110.86
731332,15,171.56041669,0\H,24,1.09531096,23,108.51824316,15,51.9335349
1,0\H,24,1.09511092,23,109.83611168,15,-67.18989722,0\H,23,1.40314563,
15,104.87735661,14,-172.37122715,0\\Version=AM64L-G03RevD.01\State=1-A
\HF=-841.0232601\MP2=-842.9743977\RMSD=4.265e-09\Thermal=0.\PG=C01 [X(
C13H14P1)]\\@

254

1\1\GINC-YANG\SP\RMP2-FC\6-31+G(2d,p)\C18H12Cl3P1\CHRISTOPH\25-Aug-201
0\0\\#p MP2(FC)/6-31+g(2d,p) scf=tight\\k5sp_2\\0,1\C\C,1,1.39681424\C
,2,1.39617418,1,118.90485019\C,3,1.40741106,2,121.32573116,1,0.8877605
7,0\C,4,1.40566653,3,118.34955844,2,-1.07397877,0\C,1,1.39630441,2,121

.21054823,3,-0.11438379,0\H,2,1.08593872,1,120.20227675,6,-179.4428103
4,0\H,3,1.08856694,2,118.98249902,1,-178.87503034,0\H,5,1.0871182,4,12
0.01581685,3,-179.19254098,0\H,6,1.08613593,1,120.09933662,2,179.60803
929,0\C,4,5.42063582,3,104.50835308,2,121.17737159,0\C,11,1.39595366,4
,43.58111921,3,-85.93522181,0\C,12,1.39812434,11,119.15787633,4,-7.497
46214,0\C,13,1.40562193,12,121.06913057,11,0.2472462,0\C,14,1.40771975
,13,118.32044186,12,0.51200359,0\C,15,1.39599832,14,121.32610605,13,-1
.12291046,0\H,12,1.08610548,11,120.11398768,4,172.54098866,0\H,13,1.08
71531,12,118.88433116,11,179.88106688,0\H,15,1.08860362,14,119.7119738
3,13,178.62062847,0\H,16,1.08592869,15,120.88312619,14,-179.76488807,0
\C,5,5.05055424,4,95.65094759,3,-162.3513875,0\C,21,1.3971495,5,67.837
89985,4,57.93396287,0\C,22,1.39594928,21,118.91436477,5,-37.45868224,0
\C,23,1.40776652,22,121.32231361,21,0.92087887,0\C,24,1.40557026,23,11
8.33712509,22,-1.12593531,0\C,21,1.39593991,5,64.25517197,4,-86.896776
99,0\H,22,1.0859622,21,120.17522338,5,143.25094808,0\H,23,1.08860617,2
2,118.98100331,21,-178.80061423,0\H,25,1.08715811,24,120.06177739,23,-
179.07791398,0\H,26,1.08614475,21,120.1226545,5,-141.82539066,0\P,4,1.
85054358,3,116.90000311,2,-179.71875859,0\Cl,1,1.76024844,6,119.396873
54,5,179.93444104,0\Cl,11,1.7603698,4,162.02613308,3,-64.43276597,0\Cl
,21,1.7603272,5,146.08763824,4,168.02506582,0\\Version=AM64L-G03RevD.0
1\State=1-A\HF=-2407.8485078\MP2=-2410.9438102\RMSD=9.591e-09\Thermal=
0.\PG=C01 [X(C18H12Cl3P1)]\\@

254-Me+

1\1\GINC-YIN\SP\RMP2-FC\6-31+G(2d,p)\C19H15Cl3P1(1+)\CHRISTOPH\25-Aug-
2010\0\\#p MP2(FC)/6-31+g(2d,p) scf=tight\\k5mesp_13\\1,1\C\C,1,1.4018
5969\C,2,1.39255632,1,119.34250155\C,3,1.41027561,2,120.28205157,1,0.0
9438319,0\C,4,1.40718604,3,119.63191247,2,-0.22062702,0\C,5,1.39511914
,4,120.240116,3,0.17684494,0\H,2,1.08546322,1,120.06105473,6,179.98518
547,0\H,3,1.08740422,2,118.65136221,1,-179.53415011,0\H,5,1.08642551,4
,120.62146899,3,179.88613482,0\H,6,1.08544714,5,120.52680159,4,179.851
56411,0\C,5,5.17165645,4,95.17455802,3,168.19685292,0\C,11,1.39906535,
5,72.06010002,4,71.48512749,0\C,12,1.3952686,11,119.41061605,5,-33.812
41669,0\C,13,1.40686226,12,120.24003376,11,-0.00438404,0\C,14,1.410554
02,13,119.62584912,12,0.20139102,0\C,15,1.3924747,14,120.28976815,13,-
0.22885029,0\H,12,1.08548598,11,120.08817666,5,146.30135149,0\H,13,1.0
8646353,12,119.14620786,11,-179.82899975,0\H,15,1.08745855,14,121.0703
6663,13,179.24129705,0\H,16,1.0854824,15,120.6264394,14,-179.8953208,0
\C,13,5.17943718,12,143.1313384,11,-162.10556485,0\C,21,1.40189812,13,
59.21981995,12,89.39788323,0\C,22,1.39263968,21,119.32821342,13,37.999
33964,0\C,23,1.41028406,22,120.27174767,21,0.07263063,0\C,24,1.4069918
4,23,119.66097173,22,-0.29258563,0\C,25,1.39511653,24,120.22081225,23,
0.26745977,0\H,22,1.08551086,21,120.06279458,13,-142.1310082,0\H,23,1.
08749102,22,118.65136124,21,-179.62498651,0\H,25,1.08639257,24,120.574
22729,23,179.91231439,0\H,26,1.08545408,25,120.52856487,24,179.7767899
7,0\P,4,1.80623004,3,119.76749698,2,-179.10470148,0\C,31,1.82609906,4,
108.58600341,3,-47.01288217,0\H,32,1.0946674,31,109.9115274,4,-177.570
80838,0\H,32,1.09468038,31,109.96498397,4,62.47409961,0\H,32,1.0947297
3,31,109.94600882,4,-57.62447573,0\Cl,1,1.74095918,6,119.53149065,5,17
9.9021588,0\Cl,11,1.74108216,5,147.16729943,4,-171.50241033,0\Cl,21,1.
74096081,13,147.39542143,12,-7.93448676,0\\Version=AM64L-G03RevD.01\St
ate=1-A\HF=-2447.2845084\MP2=-2450.5257915\RMSD=6.270e-09\Thermal=0.\P
G=C01 [X(C19H15Cl3P1)]\\@

254-BH+

1\1\GINC-PHOBOS\SP\RMP2-FC\6-31+G(2d,p)\C31H23Cl3P1(1+)\CHRISTOPH\22-S
ep-2010\0\\#p MP2(FC)/6-31+g(2d,p) scf=tight\\k5bhsp_43\\1,1\C\H,1,1.0
9959252\C,1,4.34376885,2,105.33990994\C,3,1.39839407,1,58.8101536,2,15

.72858311,0\C,4,1.39701197,3,120.11579403,1,-0.62270945,0\C,5,1.405124
77,4,120.80302629,3,0.36517198,0\C,6,1.40665886,5,118.67351294,4,-0.10
260119,0\C,3,1.39801902,1,60.80166884,6,-20.24834541,0\H,3,1.08682043,
1,178.9332927,6,-176.77998417,0\H,4,1.0869556,3,120.2312233,1,179.4797
3125,0\H,5,1.08841239,4,119.45305393,3,179.92085864,0\H,7,1.08673739,6
,120.57413549,5,-179.57954724,0\H,8,1.08711457,3,120.14590209,1,-179.5
1915459,0\P,1,1.89721068,6,113.82961212,5,126.33256591,0\C,1,4.3538586
9,6,115.4180851,5,-100.61268384,0\C,15,1.39792524,1,59.3095795,6,142.4
3282649,0\C,16,1.39667817,15,120.11284168,1,0.3965855,0\C,17,1.4064869
6,16,120.86475951,15,0.26442199,0\C,18,1.40556921,17,118.53041389,16,-
1.12469404,0\C,15,1.39793987,1,60.23410235,6,-37.37531594,0\H,15,1.086
91453,1,179.12132524,6,84.29449582,0\H,16,1.08701384,15,120.25355158,1
,-178.80746477,0\H,17,1.08725429,16,119.14025636,15,-178.92885792,0\H,
19,1.08535111,18,120.32800284,17,-177.1451061,0\H,20,1.08715514,15,120
.14132388,1,178.68914895,0\C,14,4.60807724,1,112.87301303,6,68.8934698
2,0\C,26,1.39865321,14,60.24655858,1,-121.76472897,0\C,27,1.39465595,2
6,119.39032717,14,-0.17782265,0\C,28,1.40820965,27,120.48656396,26,0.0
8986429,0\C,29,1.41224167,28,119.34306718,27,-0.33852578,0\C,30,1.3940
9513,29,120.25176323,28,0.3002613,0\H,27,1.08551741,26,120.12231623,14
,179.9496206,0\H,28,1.08586084,27,118.95961438,26,-179.97040801,0\H,30
,1.08570628,29,120.92759385,28,-179.95932465,0\H,31,1.08566212,30,120.
41898272,29,-179.94814534,0\C,14,4.62155448,1,112.96412374,6,-53.89705
949,0\C,36,1.39902279,14,61.14179458,1,42.19199061,0\C,37,1.39459345,3
6,119.66835356,14,-0.6379234,0\C,38,1.41078374,37,120.39769196,36,-0.4
6999052,0\C,39,1.41118393,38,118.96899088,37,0.98879633,0\C,40,1.39391
523,39,120.7228087,38,-0.75442595,0\H,37,1.08558421,36,120.07541501,14
,179.30225483,0\H,38,1.08355672,37,118.55315784,36,179.2310162,0\H,40,
1.08561099,39,120.68320447,38,179.30554624,0\H,41,1.08553235,40,120.44
406885,39,-179.93964188,0\C,14,4.61258489,1,106.70177886,6,-168.671579
,0\C,46,1.39736616,14,61.14403526,1,-122.24810617,0\C,47,1.39674092,46
,119.47392963,14,0.12119185,0\C,48,1.40471623,47,120.45128128,46,0.258
90842,0\C,49,1.41105216,48,119.20283952,47,-1.00256245,0\C,50,1.392270
99,49,120.63504259,48,0.95106941,0\H,47,1.08555636,46,120.11694638,14,
-179.63468552,0\H,48,1.08554749,47,118.85731996,46,-179.25146849,0\H,5
0,1.0881751,49,120.86911639,48,-177.97710426,0\H,51,1.08560587,50,120.
66900072,49,-179.85333508,0\Cl,46,1.74324789,14,179.20934149,1,60.5078
5634,0\Cl,36,1.74323678,14,179.1082536,1,-172.77525269,0\Cl,26,1.74327
192,14,179.71360906,1,-81.78645666,0\\Version=AM64L-G03RevD.01\State=1
-A\HF=-2906.39387\MP2=-2911.337791\RMSD=3.733e-09\Thermal=0.\PG=C01 [X
(C31H23Cl3P1)]\\@

254-TT^{+}

1\1\GINC-BORIX\SP\RMP2-FC\6-31+G(2d,p)\C37H27Cl3P1(1+)\CHRISTOPH\03-Se
p-2011\0\\#p MP2(FC)/6-31+g(2d,p) scf=tight\\k5ttsp_1\\1,1\C\P,1,2.001
31277\C,1,4.38766024,2,107.39508819\C,3,1.39507315,1,59.52034308,2,125
.49520107,0\C,4,1.39979921,3,120.60569076,1,-0.4447031,0\C,5,1.4058055
,4,121.12227621,3,-0.13094859,0\C,6,1.41211355,5,117.50139538,4,2.0369
2018,0\C,7,1.3964593,6,121.31254008,5,-2.51527732,0\H,3,1.08722477,1,1
79.82171993,6,177.25237906,0\H,4,1.08736022,3,120.21296006,1,179.27495
867,0\H,5,1.0839595,4,118.44067874,3,179.03676071,0\H,7,1.08422188,6,1
20.37399579,5,175.97775519,0\H,8,1.08754785,7,119.41322732,6,-179.7818
6791,0\C,1,4.38727344,6,111.20024408,5,9.49862276,0\C,14,1.39526009,1,
59.56332076,6,-118.12313149,0\C,15,1.39991524,14,120.60521651,1,-0.398
57005,0\C,16,1.40595693,15,121.08826366,14,-0.0198897,0\C,17,1.4121382
6,16,117.54044414,15,1.86111365,0\C,18,1.3963852,17,121.30029494,16,-2
.38365914,0\H,14,1.08723783,1,179.88536359,17,-167.15682165,0\H,15,1.0
8733514,14,120.21839268,1,179.38454317,0\H,16,1.08391385,15,118.446532
78,14,179.22166553,0\H,18,1.08429396,17,120.31111383,16,176.2988962,0\

H,19,1.08757277,18,119.41857463,17,-179.77933264,0\C,1,4.38780178,17,1
11.2091179,16,8.72173242,0\C,25,1.39508192,1,59.48803655,17,-117.68109
712,0\C,26,1.39976314,25,120.60563222,1,-0.42062715,0\C,27,1.40594134,
26,121.12482357,25,-0.17116727,0\C,28,1.41255523,27,117.49311189,26,2.
11190776,0\C,29,1.39653573,28,121.29700596,27,-2.55415038,0\H,25,1.087
22826,1,179.83252736,17,-35.23737022,0\H,26,1.08735447,25,120.22000335
,1,179.33771781,0\H,27,1.08389222,26,118.42211948,25,179.1766746,0\H,2
9,1.0845432,28,120.50299375,27,175.85681623,0\H,30,1.08757018,29,119.4
0269458,28,-179.81674456,0\C,2,4.63345916,1,112.76215298,17,66.1288373
9,0\C,36,1.3998202,2,60.78075523,1,55.00822558,0\C,37,1.39393026,36,11
9.64278763,2,-0.15446221,0\C,38,1.41256431,37,120.50971255,36,-0.29099
132,0\C,39,1.40830278,38,118.82195706,37,0.95952937,0\C,40,1.39550771,
39,120.80129712,38,-0.86837187,0\Cl,36,1.74422066,2,179.67212539,1,-13
2.48905448,0\H,37,1.08595089,36,120.08582446,2,179.70101799,0\H,38,1.0
8469149,37,118.47567177,36,179.50292063,0\H,40,1.08515554,39,120.64817
486,38,179.1122081,0\H,41,1.08588487,40,120.36421939,39,-179.99369785,
0\C,2,4.63469477,1,113.51362214,17,-53.49040593,0\C,47,1.39969122,2,60
.77651818,1,53.18383295,0\C,48,1.39403958,47,119.66820741,2,-0.4616045
4,0\C,49,1.41253713,48,120.53201902,47,-0.32553762,0\C,50,1.40868883,4
9,118.75923196,48,0.98899303,0\C,51,1.3954797,50,120.83670515,49,-0.89
604549,0\Cl,47,1.74431239,2,179.52136621,1,-172.04143593,0\H,48,1.0859
2629,47,120.07747751,2,179.30888508,0\H,49,1.08456745,48,118.42679802,
47,179.20208647,0\H,51,1.08503609,50,120.78405428,49,178.89871923,0\H,
52,1.08593827,51,120.36364886,50,179.9596089,0\C,2,4.63359036,1,112.91
565124,17,-173.47291086,0\C,58,1.39980754,2,60.67848568,1,53.83559932,
0\C,59,1.39395577,58,119.62676985,2,-0.27422864,0\C,60,1.41233512,59,1
20.5496396,58,-0.33334949,0\C,61,1.40875904,60,118.80098183,59,1.03998
249,0\C,62,1.39559697,61,120.77746288,60,-0.91610498,0\Cl,58,1.7442632
9,2,179.73749253,1,-146.84690254,0\H,59,1.08594189,58,120.08794037,2,1
79.55250738,0\H,60,1.08477307,59,118.52598432,58,179.44012397,0\H,62,1
.0850321,61,120.81476143,60,178.81033432,0\H,63,1.08584047,62,120.3463
4792,61,179.96481509,0\\Version=AM64L-G03RevD.01\State=1-A\HF=-3135.92
1806\MP2=-3141.7445612\RMSD=8.594e-09\Thermal=0.\PG=C01 [X(C37H27Cl3P1
)]\\@

255

1\1\GINC-YANG\SP\RMP2-FC\6-31+G(2d,p)\C6H15O3P1\CHRISTOPH\25-Aug-2010\
0\\#p MP2(FC)/6-31+g(2d,p) scf=tight\\k2sp_26\\0,1\P\O,1,1.6676716\O,1
,1.65304404,2,96.33818775\O,1,1.63263523,3,97.86363136,2,-104.97544061
,0\C,2,1.43306765,1,117.45782226,4,-97.5204357,0\H,5,1.10129858,2,110.
17342402,1,-28.75767376,0\H,5,1.09653484,2,106.18217332,1,-145.0959796
7,0\C,5,1.52744957,2,111.59028671,1,94.24086189,0\H,8,1.09638998,5,110
.7283838,2,-62.91545441,0\H,8,1.09769644,5,110.38463461,2,176.92457335
,0\H,8,1.0963664,5,110.39396583,2,57.06178397,0\C,3,1.43211908,1,117.6
5501037,4,-178.10843526,0\H,12,1.10197051,3,110.07352361,1,-44.3761111
4,0\H,12,1.09827215,3,109.20013524,1,73.78675429,0\C,12,1.52286659,3,1
08.2848576,1,-165.25803629,0\H,15,1.09575888,12,110.57454668,3,60.7981
3332,0\H,15,1.09596719,12,110.40326967,3,-59.13204282,0\H,15,1.0970247
3,12,110.26242866,3,-179.02539994,0\C,4,1.44422331,1,122.51993169,3,45
.91618246,0\H,19,1.09664307,4,109.34925756,1,42.53016779,0\H,19,1.0958
6463,4,108.50135979,1,-75.3683676,0\C,19,1.52211661,4,108.0283598,1,16
3.78163004,0\H,22,1.09735164,19,110.07828369,4,178.33188429,0\H,22,1.0
9592657,19,110.63300071,4,58.45297488,0\H,22,1.09623563,19,110.7924638
8,4,-61.68008799,0\\Version=AM64L-G03RevD.01\State=1-A\HF=-801.3441162
\MP2=-803.0054088\RMSD=4.243e-09\Thermal=0.\PG=C01 [X(C6H15O3P1)]\\@

255-Me^{+}

1\1\GINC-CIPCLU06\SP\RMP2-FC\6-31+G(2d,p)\C7H18O3P1(1+)\C2175\27-Aug-2

010\0\\#p MP2(FC)/6-31+G(2d,p) scf=tight\\k2mesp_9\\1,1\O\O,1,2.528691
23\O,1,2.50243718,2,62.91364153\C,1,1.48131648,3,96.29358604,2,-179.81
924608,0\H,4,1.09512933,1,107.24600937,3,91.28285205,0\H,4,1.09316382,
1,107.42856474,3,-26.73889026,0\C,4,1.51488138,1,107.3956349,3,-148.11
171292,0\H,7,1.09623015,4,108.74976654,1,-179.3760039,0\H,7,1.09457669
,4,111.12164657,1,-60.16032106,0\H,7,1.09473381,4,111.00291071,1,61.52
632167,0\C,2,1.4848539,1,97.7932304,4,106.0355506,0\H,11,1.09308094,2,
106.91569051,1,33.25125733,0\H,11,1.09464733,2,107.49391592,1,-84.4819
7404,0\C,11,1.5146751,2,107.35642245,1,154.34035455,0\H,14,1.09453719,
11,110.98228349,2,-60.50064686,0\H,14,1.09622456,11,108.71935467,2,-17
9.58426856,0\H,14,1.09472259,11,111.23172506,2,61.17627028,0\C,3,1.478
64301,1,161.46616936,4,-143.88803988,0\H,18,1.09503327,3,107.33149466,
1,69.22576576,0\H,18,1.09516069,3,107.9944652,1,-48.88660312,0\C,18,1.
51571458,3,107.56970566,1,-170.11972547,0\H,21,1.09607822,18,108.88478
93,3,-179.10787567,0\H,21,1.09466043,18,111.07062144,3,61.57033903,0\H
,21,1.09465978,18,110.93233543,3,-59.95188937,0\P,2,1.56360502,1,36.10
558443,4,-34.94962034,0\C,25,1.79340174,2,105.06194179,1,-120.84712481
,0\H,26,1.09547407,25,110.34589118,2,-58.9095066,0\H,26,1.09502121,25,
109.14238624,2,60.50902365,0\H,26,1.09563735,25,110.00198771,2,-179.40
657272,0\\Version=AM64L-G03RevD.01\State=1-A\HF=-840.7953628\MP2=-842.
5924704\RMSD=9.532e-09\Thermal=0.\PG=C01 [X(C7H18O3P1)]\\@

255-BH$^+$

1\1\GINC-CIPCLU08\SP\RMP2-FC\6-31+G(2d,p)\C19H26O3P1(1+)\C2175\07-Sep-
2010\0\\#p MP2(FC)/6-31+G(2d,p) scf=tight\\k2bhsp_22\\1,1\C\H,1,1.0997
5001\C,1,4.35374397,2,106.70335951\C,3,1.39894975,1,59.26842183,2,-35.
14522809,0\C,4,1.39686426,3,120.15299997,1,0.17021965,0\C,5,1.40684006
,4,120.55017215,3,-0.30538128,0\C,6,1.40296826,5,118.9714728,4,0.99881
333,0\C,3,1.39718322,1,60.33148853,6,0.79858563,0\H,3,1.08689191,1,179
.39301512,6,-155.30054549,0\H,4,1.08710125,3,120.19979593,1,179.297098
81,0\H,5,1.08780769,4,119.53580298,3,179.33285058,0\H,7,1.08553777,6,1
20.33330123,5,177.0600461,0\H,8,1.08713263,3,120.13948713,1,-179.23582
944,0\P,1,1.8334867,6,111.18074726,5,75.52960738,0\O,14,1.56662984,1,1
11.72977936,6,175.90862159,0\O,14,1.57418417,1,105.0834645,6,-68.03233
526,0\O,14,1.57885516,1,113.65966649,6,55.20104919,0\C,15,2.42383946,1
4,150.02813659,1,-51.81291429,0\C,15,1.47902466,14,128.88408526,1,-99.
76555314,0\H,18,1.09476375,15,93.08652097,14,46.34708136,0\C,17,1.4818
8657,14,125.29051744,1,-80.42395943,0\C,21,1.51511429,17,108.24264141,
14,145.50950102,0\H,22,1.09641413,21,108.82201206,17,175.5179813,0\H,1
8,1.09661636,15,144.10243414,14,-81.706471,0\H,19,1.09332705,15,105.50
15356,14,-97.98602462,0\H,21,1.09297996,17,108.93559704,14,23.26880436
,0\H,21,1.09413971,17,105.57833421,14,-94.40958781,0\H,22,1.09490605,2
1,111.05411398,17,56.39099086,0\H,22,1.0949977,21,110.99790368,17,-65.
56508216,0\H,18,1.09489752,15,90.22881308,14,155.61164117,0\H,19,1.092
02858,15,108.64978204,14,19.30133964,0\C,1,4.34402481,6,117.96365057,5
,-155.14403941,0\C,32,1.39841634,1,59.06114454,6,107.21737204,0\C,33,1
.3976678,32,120.1552103,1,0.2289208,0\C,34,1.40375824,33,120.6496667,3
2,-0.444445,0\C,35,1.40693457,34,118.83037121,33,0.36824869,0\C,32,1.3
9808335,1,60.62327842,35,0.55660342,0\H,32,1.08682772,1,179.21401503,3
5,166.49429724,0\H,33,1.08691179,32,120.21065845,1,-179.75562158,0\H,3
4,1.08826714,33,119.56072057,32,-179.87961566,0\H,36,1.08806972,35,120
.44629571,34,-179.79881188,0\H,37,1.08717774,32,120.14807965,1,179.683
33697,0\C,16,1.4763964,14,123.70644205,1,-167.4889106,0\H,43,1.0937295
9,16,107.3649766,14,56.9784299,0\H,43,1.09534477,16,107.83373519,14,-6
0.90207996,0\C,43,1.51590308,16,107.46686138,14,178.09423967,0\H,46,1.
09615951,43,108.98679525,16,179.92325946,0\H,46,1.09477753,43,110.8830
31,16,-60.90418858,0\H,46,1.09486552,43,111.0757309,16,60.54816468,0\\
Version=AM64L-G03RevD.01\State=1-A\HF=-1299.9143248\MP2=-1303.4002341\

RMSD=6.450e-09\Thermal=0.\PG=C01 [X(C19H26O3P1)]\\@

255-TT+

1\1\GINC-EVGENIX\SP\RMP2-FC\6-31+G(2d,p)\C25H30O3P1(1+)\CHRISTOPH\11-S
ep-2011\0\\#p MP2(FC)/6-31+g(2d,p) scf=tight\\k2ttsp_11_2\\1,1\C\P,1,1
.87525636\C,1,4.38697867,2,104.60182749\C,3,1.39825384,1,59.78858672,2
,-56.63826655,0\C,4,1.39732887,3,120.23719856,1,-0.37405616,0\C,5,1.41
096916,4,121.05043821,3,0.55745395,0\C,6,1.40427709,5,118.05375699,4,-
0.80191708,0\C,3,1.39634641,1,59.47905769,6,-96.7680301,0\H,3,1.086921
31,1,179.88425855,6,-84.51581448,0\H,4,1.08735896,3,120.25432695,1,-17
9.54270521,0\H,5,1.0862077,4,118.53963819,3,-178.18457012,0\H,7,1.0841
84,6,120.18521117,5,-179.22806878,0\H,8,1.08709628,3,120.17135602,1,17
9.76348265,0\C,1,4.37470079,6,109.75017036,5,-176.25687633,0\C,14,1.39
652681,1,59.14075677,6,-100.64178283,0\C,15,1.39845962,14,120.43358727
,1,-1.45208472,0\C,16,1.40441164,15,120.70608665,14,0.22977735,0\C,17,
1.40677144,16,118.38297332,15,0.95771105,0\C,18,1.39704895,17,120.7870
5038,16,-1.56685244,0\H,14,1.08691588,1,178.98276324,17,173.33694317,0
\H,15,1.087066,14,120.23843962,1,178.60459178,0\H,16,1.08534109,15,119
.1660355,14,179.87356364,0\H,18,1.08653132,17,120.73854979,16,177.1272
6549,0\H,19,1.08727416,18,119.4592678,17,-179.56168037,0\C,1,4.3811397
5,17,109.82269972,16,29.74509897,0\C,25,1.39609864,1,60.09716297,17,-1
21.45266676,0\C,26,1.40075014,25,120.5465338,1,-0.30878902,0\C,27,1.40
347758,26,120.61693796,25,-0.18254721,0\C,28,1.41029131,27,118.3383760
2,26,1.62420726,0\C,29,1.39610179,28,120.87098022,27,-1.91436327,0\H,2
5,1.08696543,1,179.47805189,17,27.05063904,0\H,26,1.08713738,25,120.19
189289,1,179.1304563,0\H,27,1.08442413,26,118.933603,25,178.83581867,0
\H,29,1.08645623,28,119.95554877,27,176.51093521,0\H,30,1.08731923,29,
119.52598043,28,179.8517421,0\C,2,2.68691427,1,133.45272762,17,-33.905
73533,0\H,36,1.09376119,2,91.10898365,1,103.61917943,0\H,36,1.09565695
,2,91.66269668,1,-146.96961656,0\C,2,2.74296214,1,105.7725634,17,-141.
9400348,0\H,39,1.09277114,2,102.66025415,1,-10.32138884,0\H,39,1.09397
103,2,84.98095905,1,98.02912821,0\C,2,2.74951047,1,116.05086702,17,100
.9270597,0\H,42,1.09158702,2,83.90854118,1,72.35856955,0\H,42,1.093707
83,2,106.38410738,1,-179.04840577,0\C,39,1.51665516,2,131.2156212,1,-1
46.42311583,0\H,45,1.09639879,39,108.90503949,2,-166.28653628,0\H,45,1
.09533326,39,111.36702007,2,-46.8699314,0\H,45,1.09506539,39,111.04675
029,2,74.6394039,0\C,42,1.51580731,2,128.32442089,1,-41.17342223,0\H,4
9,1.09662206,42,108.78565226,2,163.46098911,0\H,49,1.09508875,42,111.1
0149358,2,-77.51011867,0\H,49,1.09475894,42,110.90733462,2,44.21538595
,0\C,36,1.5162305,2,136.71915412,1,-21.33201983,0\H,53,1.09621455,36,1
09.03486895,2,178.68619473,0\H,53,1.0950525,36,111.1937393,2,-61.90378
084,0\H,53,1.09470697,36,110.69336533,2,59.39893922,0\O,42,1.47714313,
2,26.49432348,1,-86.50198703,0\O,36,1.47413583,2,29.28515413,1,-21.405
26995,0\O,39,1.47581302,2,27.20489797,1,-112.85553096,0\\Version=AM64L
-G03RevD.01\State=1-A\HF=-1529.4559372\MP2=-1533.8060499\RMSD=8.082e-0
9\Thermal=0.\PG=C01 [X(C25H30O3P1)]\\@

256

1\1\GINC-CIPCLU04\SP\RMP2-FC\6-31+G(2d,p)\C8H11P1\C2175\11-Aug-2010\0\
\#p MP2(FC)/6-31+G(2d,p) scf=tight\\ymesp_1\\0,1\P\C,1,4.67589748\C,2,
1.39995617,1,61.06303609\C,3,1.39683538,2,120.08101157,1,0.,0\C,4,1.40
699033,3,120.92744055,2,0.,0\C,5,1.40534576,4,118.26865928,3,0.,0\C,2,
1.39697197,1,58.66997498,5,-179.99529456,0\H,2,1.08791779,1,178.852748
62,5,-179.97937778,0\H,3,1.08818829,2,120.04108323,1,-180.,0\H,4,1.088
72777,3,119.00164462,2,179.99941735,0\H,6,1.08856342,5,119.15872795,4,
-179.99942442,0\H,7,1.08798064,2,120.13196539,1,-179.99931226,0\C,1,1.
86690851,5,101.01604838,4,50.96806668,0\H,13,1.097627,1,113.48518674,5
,-52.28361629,0\H,13,1.09732623,1,108.86024047,5,-173.27736288,0\H,13,

1.09609986,1,109.35138568,5,69.14130031,0\C,1,1.86690303,5,101.0172226
5,4,-50.9712919,0\H,17,1.09732633,1,108.85783524,5,173.25900689,0\H,17
,1.09609983,1,109.35301647,5,-69.15981778,0\H,17,1.09762868,1,113.4851
8245,5,52.26725265,0\\Version=AM64L-G03RevD.01\State=1-A\HF=-650.11124
51\MP2=-651.3936174\RMSD=6.786e-09\Thermal=0.\PG=C01 [X(C8H11P1)]\\@

256-Me^{+}

1\1\GINC-CIPCLU04\SP\RMP2-FC\6-31+G(2d,p)\C9H14P1(1+)\C2175\11-Aug-201
0\0\\#p MP2(FC)/6-31+G(2d,p) scf=tight\\ymemesp_1\\1,1\C\C,1,1.4003662
4\C,2,1.39430427,1,120.0551156\C,3,1.41031766,2,119.78101668,1,0.,0\C,
4,1.40714906,3,120.00011105,2,0.00036304,0\C,5,1.39707961,4,119.692049
71,3,-0.00054597,0\H,1,1.08672743,6,119.87069298,5,-180.,0\H,2,1.08643
87,1,120.22629021,6,-179.99970535,0\H,3,1.08860903,2,119.12503238,1,18
0.,0\H,5,1.08655308,4,121.13755908,3,-179.99921991,0\H,6,1.0864214,5,1
19.58722156,4,-179.99928058,0\C,4,2.97231266,3,98.2299719,2,149.853398
81,0\H,12,1.09599008,4,144.7118683,3,127.9566093,0\H,12,1.095301,4,90.
3016666,3,3.28095532,0\C,12,2.95385115,4,60.20511618,3,94.73326755,0\H
,15,1.09599073,12,89.21035433,4,161.54296915,0\H,15,1.09529965,12,91.4
1070645,4,-89.54419302,0\P,4,1.80084048,3,118.85480479,2,179.99575618,
0\C,18,1.81719229,4,111.02741962,3,179.9816064,0\H,19,1.09514461,18,11
0.54426891,4,60.64214017,0\H,19,1.09592077,18,108.87778869,4,-179.9974
153,0\H,19,1.09514418,18,110.54136736,4,-60.63768359,0\H,12,1.0953873,
4,90.89544471,3,-105.41492037,0\H,15,1.09538697,12,145.32302722,4,37.2
5058132,0\\Version=AM64L-G03RevD.01\State=1-A\HF=-689.557616\MP2=-690.
984052\RMSD=5.374e-09\Thermal=0.\PG=C01 [X(C9H14P1)]\\@

256-BH^{+}

1\1\GINC-CIPCLU09\SP\RMP2-FC\6-31+G(2d,p)\C21H22P1(1+)\C2175\18-Feb-20
11\0\\#p MP2(FC)/6-31+G(2d,p) scf=tight\\ymebhsp_5\\1,1\C\H,1,1.101157
32\C,1,4.34341711,2,105.35436287\C,3,1.39812083,1,58.89594697,2,14.776
61005,0\C,4,1.39688313,3,120.10700985,1,-0.50761833,0\C,5,1.40507259,4
,120.76989115,3,0.51956874,0\C,6,1.40651522,5,118.71391702,4,-0.606670
96,0\C,3,1.39757668,1,60.74661634,6,-12.42995677,0\H,3,1.08681014,1,17
9.00902418,6,-169.87163305,0\H,4,1.08692314,3,120.23630167,1,179.52714
802,0\H,5,1.08868697,4,119.44517615,3,179.61472979,0\H,7,1.08700403,6,
120.67301198,5,-179.75440777,0\H,8,1.08711548,3,120.13146118,1,-179.51
762039,0\C,1,4.34627238,6,118.52258386,5,-103.81100148,0\C,14,1.398199
83,1,60.38800133,6,-40.03703895,0\C,15,1.39805439,14,120.44923568,1,-0
.02082286,0\C,16,1.40406704,15,120.31399648,14,0.07070125,0\C,17,1.406
68549,16,118.82202861,15,0.52348228,0\C,18,1.39728667,17,120.78938584,
16,-0.68222059,0\H,14,1.08687796,1,179.33298178,6,107.54406437,0\H,15,
1.08707992,14,120.07771867,1,179.3456758,0\H,16,1.08548332,15,119.3486
1673,14,178.44412025,0\H,18,1.08934003,17,119.88971783,16,177.75678036
,0\H,19,1.08699901,18,119.75186976,17,179.26243128,0\P,1,1.87342297,6,
111.81344951,5,124.56859201,0\C,25,4.60454327,1,115.60750158,6,75.8190
6061,0\C,26,1.39875553,25,60.3682152,1,-108.65974058,0\C,27,1.39617084
,26,120.11847093,25,0.42438613,0\C,28,1.40881824,27,119.85986311,26,-0
.07878182,0\C,29,1.41019046,28,119.83515697,27,0.0632838,0\C,30,1.3957
2023,29,119.75204927,28,0.01825027,0\H,26,1.08688784,25,179.42290183,1
,135.56501026,0\H,27,1.08657741,26,120.25896497,25,-179.33085025,0\H,2
8,1.08645858,27,119.21989706,26,-179.98610702,0\H,30,1.086507,29,120.8
9530594,28,179.68482189,0\H,31,1.0865656,30,119.58426056,29,-179.89501
002,0\C,25,1.82106439,1,107.87601329,6,-48.35154994,0\H,37,1.09399863,
25,110.4701233,1,61.16965902,0\H,37,1.09510393,25,110.31997157,1,-176.
9673204,0\H,37,1.09592872,25,108.64114897,1,-57.51736264,0\C,25,1.8234
7911,1,107.77458575,6,-163.93600437,0\H,41,1.09526244,25,109.35871961,
1,-179.96016362,0\H,41,1.09428125,25,110.30392238,1,-59.77070921,0\H,4
1,1.09592583,25,109.83919153,1,60.13559845,0\\Version=AM64L-G03RevD.01

\State=1-A\HF=-1148.6781338\MP2=-1151.7942739\RMSD=5.328e-09\Thermal=0
.\PG=C01 [X(C21H22P1)]\\@

256-TT⁺

1\1\GINC-IBLIS\SP\RMP2-FC\6-31+G(2d,p)\C27H26P1(1+)\CHRISTOPH\11-Sep-2
011\0\\#p MP2(FC)/6-31+g(2d,p) scf=tight\\ymettsp_9\\1,1\C\P,1,1.94039
87\C,1,4.38310322,2,115.22129237\C,3,1.39817173,1,58.21372505,2,-154.9
4041716,0\C,4,1.39587211,3,120.46939933,1,1.59970215,0\C,5,1.40881676,
4,121.14680015,3,-0.31942167,0\C,6,1.40408579,5,117.59780157,4,-0.8466
9997,0\C,3,1.39505384,1,60.95247158,6,42.4783201,0\H,3,1.08673042,1,17
8.39070604,6,-171.89117415,0\H,4,1.08702148,3,120.21902843,1,-178.5276
7228,0\H,5,1.08569638,4,119.09080522,3,-179.97104219,0\H,7,1.08420303,
6,121.32025312,5,-177.60896636,0\H,8,1.08708202,3,120.33827723,1,178.6
4286528,0\C,1,4.3857336,6,111.03687159,5,88.23627039,0\C,14,1.39772849
,1,59.79220494,6,-177.39522863,0\C,15,1.39753063,14,120.33898206,1,0.7
8453624,0\C,16,1.41201543,15,121.11757861,14,-0.07572429,0\C,17,1.4065
8011,16,117.7795142,15,-0.17766623,0\C,14,1.39592907,1,59.40908896,17,
96.50124446,0\H,14,1.08688909,1,179.58679684,17,163.37244936,0\H,15,1.
08731837,14,120.28854822,1,-179.79189392,0\H,16,1.08501837,15,118.3936
3427,14,179.15875182,0\H,18,1.08414908,17,120.34276257,16,179.83787047
,0\H,19,1.08707299,14,120.17445277,1,179.59030322,0\C,1,4.382363,17,11
7.0255211,16,-55.04050234,0\C,25,1.39979342,1,58.89737795,17,-177.4594
9257,0\C,26,1.39559473,25,120.07339186,1,0.16049681,0\C,27,1.41132216,
26,121.2454729,25,-0.98183977,0\C,28,1.40237279,27,117.98301122,26,2.5
4368692,0\C,25,1.39509468,1,60.36480979,17,1.33623439,0\H,25,1.0868903
1,1,179.17334379,17,-158.99261651,0\H,26,1.08734054,25,120.2824814,1,1
78.80454832,0\H,27,1.08689147,26,118.79699904,25,175.88450644,0\H,29,1
.08372606,28,120.4916472,27,176.23502312,0\H,30,1.08711027,25,120.1486
0766,1,-179.13773987,0\C,2,4.626781,1,114.45082879,17,-76.46606647,0\C
,36,1.39813415,2,60.00910319,1,107.34823017,0\C,37,1.39613221,36,120.1
7061273,2,-1.11022559,0\C,38,1.40956579,37,120.17193623,36,0.14281305,
0\C,39,1.40980112,38,119.3378335,37,-0.24166707,0\C,40,1.39598264,39,1
20.00174494,38,0.13473212,0\H,36,1.08689881,2,178.93773902,1,-162.0205
4171,0\H,37,1.08669567,36,120.29937093,2,178.78167684,0\H,38,1.0855225
2,37,118.80462235,36,-179.38197116,0\H,40,1.0858005,39,121.0124537,38,
-179.05882165,0\H,41,1.08662381,40,119.42846061,39,-179.9965578,0\C,2,
1.83046928,1,111.03789251,17,43.44971255,0\H,47,1.09356171,2,110.15225
883,1,-52.43662429,0\H,47,1.0920841,2,111.07427783,1,67.6198913,0\H,47
,1.09529983,2,108.75214855,1,-172.80306423,0\C,2,1.8315674,1,113.44828
352,17,163.61737384,0\H,51,1.09610524,2,107.7306409,1,-168.97926279,0\
H,51,1.09317669,2,111.18607856,1,-48.70754937,0\H,51,1.09258564,2,110.
33136709,1,72.72351238,0\\Version=AM64L-G03RevD.01\State=1-A\HF=-1378.
2081543\MP2=-1382.1933345\RMSD=9.550e-09\Thermal=0.\PG=C01 [X(C27H26P1
)]\\@

257

1\1\GINC-EDDY\SP\RMP2-FC\6-31+G(2d,p)\C21H21P1\CHRISTOPH\16-Oct-2011\0
\\#p MP2(FC)/6-31+g(2d,p) scf=tight\\otol3sp_4\\0,1\P\C,1,4.67071617\C
,2,1.39766614,1,59.04678464\C,3,1.40229374,2,121.63722595,1,-1.2293127
9,0\C,4,1.42032727,3,118.71878411,2,-0.48405216,0\C,5,1.40639789,4,119
.09398991,3,0.22586817,0\C,2,1.39553925,1,60.60318871,5,-69.42610906,0
\H,2,1.08794936,1,178.24538772,5,163.76495264,0\H,3,1.08892059,2,119.5
4591119,1,178.46637257,0\H,6,1.08708277,5,119.40157806,4,-179.7794642,
0\H,7,1.08791265,2,120.48767367,1,-178.63823255,0\C,1,4.67066497,5,103
.57876687,4,81.25170282,0\C,12,1.39586746,1,60.59024707,5,9.64381753,0
\C,13,1.39794544,12,119.61514326,1,1.41941488,0\C,14,1.4063216,13,121.
27801013,12,-0.25505308,0\C,15,1.41976154,14,119.12701216,13,0.1150279
3,0\C,12,1.39744985,1,59.06797138,5,-171.72202417,0\H,12,1.08797689,1,

178.3662417,5,-119.180696,0\H,13,1.0878995,12,120.49046278,1,-178.8097
1289,0\H,14,1.08714424,13,119.45075577,12,179.80855116,0\H,17,1.088886
57,12,119.53667199,1,178.60129192,0\C,1,4.67082252,5,104.83013489,4,-1
69.03986953,0\C,22,1.3957051,1,60.59674258,5,-97.19119509,0\C,23,1.398
00611,22,119.62531015,1,1.51127674,0\C,24,1.40647998,23,121.29335175,2
2,-0.31316819,0\C,25,1.41986365,24,119.0886391,23,0.30478888,0\C,22,1.
39746615,1,59.05114886,25,109.82379531,0\H,22,1.08795953,1,178.2871643
,25,163.52781084,0\H,23,1.08789796,22,120.48268952,1,-178.72848996,0\H
,24,1.0870586,23,119.34337396,22,179.65849607,0\H,27,1.08891585,22,119
.54072462,1,178.53086097,0\C,4,1.51469484,3,119.51625094,2,-179.578359
58,0\H,32,1.09789749,4,111.39393883,3,-127.19584982,0\H,32,1.09578301,
4,110.86155002,3,-6.52445611,0\H,32,1.09846482,4,111.44494003,3,113.55
876653,0\C,16,1.51482247,15,121.7966411,14,179.14562503,0\H,36,1.09580
124,16,110.89045419,15,172.79073855,0\H,36,1.09857411,16,111.35780295,
15,-67.20947208,0\H,36,1.09766228,16,111.45866799,15,52.0125567,0\C,26
,1.51486351,25,121.79207671,24,179.03049666,0\H,40,1.09574449,26,110.8
4936023,25,174.23958797,0\H,40,1.09857698,26,111.39665799,25,-65.73827
039,0\H,40,1.09773076,26,111.43369594,25,53.48361663,0\\Version=AM64L-
G03RevD.01\State=1-A\HF=-1148.266021\MP2=-1151.4021269\RMSD=3.304e-09\
Thermal=0.\PG=C01 [X(C21H21P1)]\\@

257-Me$^+$

1\1\GINC-EVGENIX\SP\RMP2-FC\6-31+G(2d,p)\C22H24P1(1+)\CHRISTOPH\17-Oct
-2011\0\\#p MP2(FC)/6-31+g(2d,p) scf=tight\\otol3mesp_6\\1,1\C\C,1,1.3
9697884\C,2,1.40274736,1,122.06474851\C,3,1.42189805,2,117.44317692,1,
0.13378888,0\C,4,1.41012927,3,120.30209333,2,-0.85797987,0\C,5,1.39482
677,4,120.72878299,3,0.93297453,0\H,1,1.0869499,6,120.28144696,5,-179.
99614918,0\H,2,1.08769969,1,119.39575258,6,-179.22461774,0\H,5,1.08612
26,4,119.98760351,3,-179.10519392,0\H,6,1.0863598,5,119.87455981,4,179
.88317562,0\C,4,5.4527073,3,95.97560478,2,-128.99901601,0\C,11,1.39576
076,4,41.00125468,3,68.47761648,0\C,12,1.39486515,11,119.40495939,4,5.
32471603,0\C,13,1.40992389,12,120.7321889,11,-0.22796724,0\C,14,1.4218
5821,13,120.30743763,12,0.9106399,0\C,11,1.39699964,4,79.21076779,3,-1
16.63147658,0\H,11,1.08694846,4,160.84253153,3,54.26595653,0\H,12,1.08
636866,11,120.71953706,4,-174.82939136,0\H,13,1.0860308,12,119.3261362
6,11,179.79258572,0\H,16,1.08767047,11,119.40524829,4,176.84720698,0\C
,5,5.09915943,4,99.61015958,3,172.54824315,0\C,21,1.39574992,5,70.7968
8925,4,68.01692865,0\C,22,1.39481832,21,119.40615949,5,-33.86471336,0\
C,23,1.41006124,22,120.73029256,21,-0.26783625,0\C,24,1.42185907,23,12
0.30210805,22,1.01817984,0\C,21,1.39694283,5,58.7852234,4,-78.18007885
,0\H,21,1.08694096,5,147.46482429,4,-175.45693488,0\H,22,1.08635951,21
,120.7189003,5,145.97022891,0\H,23,1.0860445,22,119.30474095,21,179.75
746367,0\H,26,1.08768187,21,119.4039721,5,-141.82242496,0\C,3,1.516295
32,2,118.42845543,1,-179.79534663,0\H,31,1.09429772,3,110.0383177,2,18
.68781387,0\H,31,1.09617018,3,112.63228937,2,138.28787962,0\H,31,1.097
99743,3,111.55176674,2,-99.84780742,0\C,15,1.51621147,14,124.08445001,
13,179.05617024,0\H,35,1.09621868,15,112.55987853,14,-42.00937126,0\H,
35,1.09790699,15,111.59185515,14,79.81006473,0\H,35,1.09429882,15,110.
06140044,14,-161.60500088,0\C,25,1.51641266,24,124.121461,23,178.91939
629,0\H,39,1.09625183,25,112.60932801,24,-41.61813849,0\H,39,1.0979865
3,25,111.57292721,24,80.23919736,0\H,39,1.09429524,25,110.03976083,24,
-161.21158388,0\P,24,1.82225218,23,117.14539145,22,179.63629231,0\C,43
,1.83745114,24,109.4865603,23,132.27199598,0\H,44,1.0930277,43,109.714
84659,24,88.97835093,0\H,44,1.09304351,43,109.7702752,24,-151.05342792
,0\H,44,1.09299312,43,109.80701507,24,-31.02606178,0\\Version=AM64L-G0
3RevD.01\State=1-A\HF=-1187.7115776\MP2=-1190.9942801\RMSD=1.935e-09\T
hermal=0.\PG=C01 [X(C22H24P1)]\\@

258

1\1\GINC-YIN\SP\RMP2-FC\6-31+G(2d,p)\C21H21N2P1S1\CHRISTOPH\18-Dec-200
8\0\\#p MP2(FC)/6-31+g(2d,p) scf=tight\\etsspc051\\0,1\C\C,1,1.4005312
1\C,2,1.39775356,1,120.0797305\C,3,1.40865707,2,120.5872889,1,0.378092
27,0\C,4,1.40496875,3,118.71762638,2,-0.60578313,0\C,1,1.39723301,2,11
9.84010606,3,-0.02763397,0\P,4,1.85110943,3,123.76476258,2,-178.289982
13,0\C,7,1.84895514,4,104.5331265,3,-69.66747387,0\C,8,1.40542122,7,12
4.87090501,4,4.7585345,0\C,9,1.39924086,8,120.56600955,7,-177.27519512
,0\C,10,1.39751613,9,120.37188956,8,-0.37301443,0\C,11,1.39920866,10,1
19.64152841,9,0.60478279,0\C,12,1.39661214,11,120.02414453,10,0.213087
39,0\C,7,1.85756823,4,99.97537442,3,35.8430572,0\C,14,1.40406104,7,124
.67333968,4,-106.68654138,0\C,15,1.39907095,14,121.09750586,7,179.2015
666,0\C,16,1.39718407,15,120.08742031,14,0.47491488,0\C,17,1.39721105,
16,119.82233303,15,1.19672312,0\C,18,1.40270507,17,120.19262811,16,-1.
22465679,0\N,19,1.42380081,18,119.80325399,17,178.19067422,0\C,20,1.37
686182,19,128.03047054,18,62.89258688,0\N,21,1.35378442,20,115.9377381
8,19,1.28215227,0\H,17,1.0874257,16,120.40370145,15,179.75753952,0\H,1
6,1.08761835,15,119.70787335,14,-179.16269944,0\H,15,1.08694753,14,119
.34120018,7,0.3040249,0\H,18,1.08715765,17,120.86235908,16,177.4915941
6,0\H,20,1.01365853,19,118.05436493,18,-126.24220422,0\H,22,1.01400757
,21,117.19226096,20,13.13091828,0\H,9,1.08671934,8,119.98625372,7,2.28
2687,0\H,13,1.0890826,12,119.49160993,11,178.48484212,0\H,10,1.0880186
5,9,119.5659481,8,179.69778049,0\H,12,1.08781953,11,120.18321543,10,17
9.37038594,0\H,11,1.08769958,10,120.19342446,9,-179.92191575,0\H,3,1.0
8834541,2,119.68219506,1,-179.44974204,0\H,5,1.08774952,4,119.55840181
,3,-178.24585256,0\H,2,1.08840378,1,120.08534548,6,179.9320287,0\H,6,1
.08733671,1,120.20001471,2,179.10319774,0\H,1,1.08782632,6,120.1101440
8,5,-179.77355751,0\C,22,1.46086099,21,123.30946942,20,172.92715588,0\
H,39,1.09560575,22,108.42591832,21,59.374256,0\H,39,1.09878108,22,109.
67470921,21,-56.91593156,0\C,39,1.52972167,22,109.92741737,21,-179.001
48006,0\H,42,1.09555869,39,109.92848238,22,-179.18777606,0\H,42,1.0971
8154,39,111.38732471,22,-59.44133253,0\H,42,1.09851305,39,111.16755382
,22,61.67835049,0\S,21,1.68654013,20,120.12563087,19,-178.75609979,0\\
Version=AM64L-G03RevD.01\State=1-A\HF=-1654.6956502\MP2=-1658.3091063\
RMSD=3.648e-09\Thermal=0.\PG=C01 [X(C21H21N2P1S1)]\\@

258-Me$^+$

1\1\GINC-YIN\SP\RMP2-FC\6-31+G(2d,p)\C22H24N2P1S1(1+)\CHRISTOPH\15-Jan
-2009\0\\#p MP2(FC)/6-31+g(2d,p) scf=tight\\etsmespc044\\1,1\C\C,1,1.4
0070697\C,2,1.39454921,1,120.05903102\C,3,1.41051475,2,119.86287653,1,
0.06028624,0\C,4,1.40545725,3,119.92698753,2,-0.17925684,0\C,1,1.39724
853,2,120.20878514,3,0.0123302,0\C,4,2.96806918,3,153.65074913,2,-174.
95968119,0\C,7,1.4060178,4,122.96591174,3,94.17876421,0\C,8,1.39790758
,7,119.61021475,4,136.64492267,0\C,9,1.39764986,8,120.21952055,7,-0.18
036412,0\C,10,1.4010437,9,120.27373752,8,0.38814662,0\C,11,1.39494456,
10,120.01045517,9,-0.06240943,0\C,4,2.9406902,3,98.37648472,2,150.5992
4883,0\C,13,1.40591679,4,85.34049952,3,-69.57050647,0\C,14,1.39886076,
13,120.3464898,4,169.24862742,0\C,15,1.39599953,14,120.12211089,13,-0.
34032523,0\C,16,1.4001275,15,120.04439763,14,0.5988142,0\C,17,1.398894
99,16,120.47999136,15,0.02412522,0\N,18,1.42657181,17,120.51045157,16,
-178.95501374,0\C,19,1.38896355,18,127.41928035,17,-93.30243143,0\N,20
,1.34745274,19,115.57035803,18,9.71988755,0\H,16,1.08677874,15,120.217
86854,14,-179.28956927,0\H,15,1.08651991,14,119.5883462,13,-179.768908
1,0\H,14,1.08637989,13,120.21287082,4,-9.86799404,0\H,17,1.08719167,16
,120.82556384,15,-179.61209591,0\H,19,1.01296788,18,118.69133098,17,78
.88723389,0\H,21,1.01397253,20,117.59436199,19,12.99544727,0\H,8,1.085
64252,7,121.12739212,4,-44.3268717,0\H,12,1.08808147,11,119.66222095,1
0,179.42614494,0\H,9,1.0864523,8,119.48263708,7,179.59094645,0\H,11,1.

08685477,10,120.22703248,9,179.84679257,0\H,10,1.08700657,9,119.882308
57,8,-179.80759896,0\H,3,1.08824051,2,119.33498184,1,179.59013603,0\H,
5,1.08628806,4,120.54298074,3,-179.48216195,0\H,2,1.08668886,1,120.229
65183,6,-179.90011713,0\H,6,1.08662989,1,120.21717661,2,179.84457448,0
\H,1,1.0868826,6,119.95491788,5,-179.86481928,0\C,21,1.46661301,20,124
.72582677,19,179.62016174,0\H,38,1.09675389,21,106.75611342,20,149.126
05565,0\H,38,1.09323278,21,107.11185314,20,33.48056419,0\C,38,1.532617
53,21,112.9014823,20,-88.5485511,0\H,41,1.09637216,38,109.80768498,21,
-179.00173154,0\H,41,1.09712521,38,111.43348403,21,-59.44232244,0\H,41
,1.09501329,38,110.41289844,21,61.62654586,0\S,20,1.68409211,19,118.61
602874,18,-167.85429029,0\P,7,1.80746679,4,35.06195835,3,-3.78800452,0
\C,46,1.82451671,7,109.92731387,4,117.18052093,0\H,47,1.09536711,46,10
8.96922212,7,169.87080046,0\H,47,1.09566935,46,108.00823497,7,-72.7293
0268,0\H,47,1.09659672,46,111.56954601,7,48.51761837,0\\Version=AM64L-
G03RevD.01\State=1-A\HF=-1694.138702\MP2=-1697.9012206\RMSD=3.763e-09\
Thermal=0.\PG=C01 [X(C22H24N2P1S1)]\\@

259

1\1\GINC-NODE8\SP\RMP2-FC\6-31+G(2d,p)\C13H13P1\ZIP07\06-Apr-2010\0\\#
p MP2(FC)/6-31+G(2d,p) scf=tight\\xmesp_10\\0,1\P\C,1,4.6718572\C,2,1.
39628778,1,60.94409687\C,3,1.40036957,2,120.33409749,1,-1.60475314,0\C
,4,1.40559134,3,120.82029347,2,0.24858828,0\C,5,1.40977489,4,118.14414
088,3,0.39296451,0\C,6,1.39517586,5,121.04792441,4,-0.95749969,0\H,2,1
.08768767,1,178.24045531,5,179.664338,0\H,3,1.08816822,2,120.09599272,
1,178.65377549,0\H,4,1.08703898,3,118.81701519,2,-179.67669028,0\H,6,1
.08892624,5,119.53880697,4,179.45745678,0\H,7,1.08799775,6,119.7322061
9,5,-179.47534617,0\C,1,4.67034987,5,103.60816691,4,118.00527222,0\C,1
3,1.39733257,1,58.74167541,5,145.59853587,0\C,14,1.39935871,13,119.968
82879,1,-0.96188993,0\C,15,1.40533578,14,120.82054993,13,0.54353209,0\
C,16,1.40705378,15,118.613235,14,-0.68127784,0\C,17,1.39685962,16,120.
63162096,15,0.34953469,0\H,13,1.08792382,1,178.77948304,5,169.05562926
,0\H,14,1.08797897,13,120.16089665,1,179.51243606,0\H,15,1.08878786,14
,119.78478051,13,-179.18097024,0\H,17,1.08817199,16,119.68143149,15,-1
78.95269842,0\H,18,1.08817556,17,119.81215468,16,-179.83593176,0\C,1,1
.86494287,5,102.23421841,4,14.8327346,0\H,24,1.09654507,1,110.02918861
,5,-72.66800222,0\H,24,1.09707704,1,113.37074596,5,50.17165878,0\H,24,
1.09598742,1,107.65051912,5,170.20003019,0\\Version=AM64L-G03RevD.01\S
tate=1-A\HF=-840.6313243\MP2=-842.5989515\RMSD=9.216e-09\Thermal=0.\PG
=C01 [X(C13H13P1)]\\@

259-Me$^+$

1\1\GINC-NODE8\SP\RMP2-FC\6-31+G(2d,p)\C14H16P1(1+)\ZIP07\06-Apr-2010\
0\\#p MP2(FC)/6-31+G(2d,p) scf=tight\\xmemesp_11\\1,1\C\C,1,1.3983476\
C,2,1.39683253,1,120.10650133\C,3,1.40739154,2,119.7104477,1,-0.178891
45,0\C,4,1.40961071,3,120.04783992,2,0.05304424,0\C,5,1.39505402,4,119
.71518805,3,0.10506057,0\H,1,1.08683128,2,119.84300743,3,179.95215169,
0\H,2,1.08651728,1,120.25537542,6,-179.68248932,0\H,3,1.0866746,2,119.
19690223,1,-179.8872696,0\H,5,1.08774396,4,120.59258388,3,-179.0259931
3,0\H,6,1.08655579,5,119.69710013,4,-179.84936124,0\C,5,5.43026412,4,8
5.4953845,3,-148.22734661,0\C,12,1.39996434,5,49.14988615,4,76.2954843
2,0\C,13,1.39505474,12,120.09541309,5,-34.58172659,0\C,14,1.40960725,1
3,119.71515902,12,-0.13670945,0\C,15,1.40739309,14,120.04796097,13,0.1
0363652,0\C,16,1.39683148,15,119.71039215,14,0.0541965,0\H,12,1.086831
75,5,149.80031645,4,156.02417781,0\H,13,1.08655646,12,120.2068482,5,14
5.12862736,0\H,14,1.08774333,13,119.68682428,12,179.00346967,0\H,16,1.
08667527,15,121.09193592,14,179.75580931,0\H,17,1.0865162,16,119.63802
595,15,179.65228561,0\C,15,2.96466597,14,106.46815932,13,-140.99173043
,0\H,23,1.09560123,15,87.36049994,14,114.72814028,0\H,23,1.09531322,15

,145.82522948,14,-124.69413382,0\H,23,1.09489909,15,92.17308688,14,6.0
7435401,0\P,4,1.80472861,3,121.00754073,2,177.20516525,0\C,27,1.822817
52,4,109.62382495,3,-99.74960238,0\H,28,1.09560052,27,108.49540126,4,5
5.7570424,0\H,28,1.09489828,27,110.09553312,4,-63.1140347,0\H,28,1.095
31306,27,111.01591301,4,175.36010716,0\\Version=AM64L-G03RevD.01\State
=1-A\HF=-880.0804427\MP2=-882.1916521\RMSD=5.060e-09\Thermal=0.\PG=C01
[X(C14H16P1)]\\@

259-BH+

1\1\GINC-NAUTILUS\SP\RMP2-FC\6-31+G(2d,p)\C26H24P1(1+)\CHRISTOPH\03-Ju
l-2010\0\\#p MP2(FC)/6-31+g(2d,p) scf=tight\\xmebhsp_33\\1,1\C\H,1,1.1
0000503\P,1,1.88547712,2,101.25715212\C,1,4.3498032,3,111.73343381,2,1
12.52675696,0\C,4,1.39748353,1,60.29884299,3,104.56538997,0\C,5,1.3985
3444,4,120.4536286,1,-0.01135685,0\C,6,1.40371169,5,120.39992935,4,0.0
772144,0\C,7,1.40724,6,118.76893703,5,0.81640128,0\C,8,1.39698663,7,12
0.70480997,6,-1.09350893,0\H,4,1.08698321,1,179.39928574,7,153.0063779
,0\H,5,1.0871413,4,120.1276633,1,179.4100113,0\H,6,1.08523851,5,119.23
823308,4,178.56331833,0\H,8,1.08793981,7,120.01995806,6,178.20709504,0
\H,9,1.0871148,8,119.64472165,7,179.54510443,0\C,1,4.34245915,7,117.66
669965,6,-27.24569958,0\C,15,1.39784886,1,59.01839865,7,-106.6989142,0
\C,16,1.39741946,15,120.11982935,1,-0.33995256,0\C,17,1.40456654,16,12
0.76472523,15,0.56032955,0\C,18,1.40708784,17,118.68595363,16,-0.61461
063,0\C,15,1.39813861,1,60.63587966,18,-3.60216884,0\H,15,1.08684089,1
,179.17398588,18,-168.2783379,0\H,16,1.08697799,15,120.20947515,1,179.
68423725,0\H,17,1.08880907,16,119.48008465,15,179.59687084,0\H,19,1.08
762132,18,120.42711001,17,-179.94219944,0\H,20,1.08715036,15,120.13905
457,1,-179.69174918,0\C,3,4.61649842,1,109.81318466,18,-155.30333262,0
\C,26,1.40040806,3,59.54291716,1,53.7953653,0\C,27,1.3947105,26,120.01
982062,3,-0.73972172,0\C,28,1.41039451,27,120.04500042,26,-0.15356225,
0\C,29,1.4052619,28,119.69614501,27,0.53505846,0\C,26,1.39699063,3,60.
59038925,1,-125.7010351,0\H,26,1.08685898,3,179.11052951,1,102.0693574
9,0\H,27,1.08668234,26,120.24787067,3,179.0866516,0\H,28,1.08856182,27
,118.95236786,26,179.65964505,0\H,30,1.08580708,29,120.52213216,28,179
.21720727,0\H,31,1.08666493,26,120.20676837,3,-179.13380005,0\C,3,4.60
908516,1,113.12913111,18,79.48591345,0\C,37,1.39831167,3,60.06665342,1
,-120.08086183,0\C,38,1.3963461,37,120.13823523,3,-0.01183359,0\C,39,1
.40888245,38,119.97331984,37,0.01591374,0\C,40,1.41176523,39,119.70366
401,38,-0.13818467,0\C,41,1.39613039,40,119.7232333,39,0.12145183,0\H,
37,1.08692624,3,179.97554784,1,-165.257715,0\H,38,1.08662455,37,120.27
289738,3,-179.90246168,0\H,39,1.08604217,38,119.06391063,37,179.965667
86,0\H,41,1.08597937,40,120.79483098,39,-179.7203517,0\H,42,1.08664364
,41,119.46595339,40,179.99331156,0\C,3,1.82505933,1,107.16036207,18,-4
1.5262935,0\H,48,1.09357052,3,110.84320041,1,63.68570673,0\H,48,1.0955
4388,3,108.48387013,1,-55.30280972,0\H,48,1.09477078,3,109.77762547,1,
-173.83988259,0\\Version=AM64L-G03RevD.01\State=1-A\HF=-1339.196245\MP
2=-1343.003361\RMSD=6.146e-09\Thermal=0.\PG=C01 [X(C26H24P1)]\\@

259-TT+

1\1\GINC-PHOBOS\SP\RMP2-FC\6-31+G(2d,p)\C32H28P1(1+)\CHRISTOPH\22-Aug-
2011\0\\#p MP2(FC)/6-31+g(2d,p) scf=tight\\xmettsp_12\\1,1\P\C,1,4.642
35866\C,2,1.39771986,1,60.32747113\C,3,1.39687981,2,120.55154745,1,0.4
2891164,0\C,4,1.41094001,3,120.04440042,2,-0.09692743,0\C,5,1.41073751
,4,119.00495709,3,-0.10423443,0\C,6,1.39629713,5,120.40581617,4,0.2114
9246,0\H,2,1.08684307,1,179.45359114,5,177.9194523,0\H,3,1.08672325,2,
120.22288877,1,-179.6635759,0\H,4,1.08395541,3,119.00401145,2,-179.724
13968,0\H,6,1.08485783,5,121.17895417,4,179.59971157,0\H,7,1.08669438,
6,119.43829269,5,179.94389841,0\C,1,1.83007646,5,107.15384007,4,-177.0
1366677,0\C,1,4.62977048,5,108.03899092,4,72.62865449,0\C,14,1.3988653

4,1,59.88182022,5,170.52732069,0\C,15,1.39586977,14,120.36077202,1,0.9
6935285,0\C,16,1.41100632,15,119.89518792,14,0.25579402,0\C,17,1.40813
455,16,119.48274729,15,-0.63378983,0\C,18,1.39677191,17,120.05613294,1
6,0.51934346,0\H,14,1.08694337,1,178.99954098,17,-178.21232852,0\H,15,
1.08670931,14,120.23774237,1,-178.80537019,0\H,16,1.08563928,15,119.05
32246,14,-179.97690378,0\H,18,1.08512795,17,120.59552987,16,-178.91234
62,0\H,19,1.08672826,18,119.47990352,17,-179.7548218,0\C,1,1.96170197,
17,112.63141844,16,-64.66019236,0\C,4,4.22199746,3,111.62941903,2,162.
1217543,0\C,26,1.39849685,4,69.34255385,3,137.61957854,0\C,27,1.397567
12,26,120.31015119,4,55.86220738,0\C,28,1.41221468,27,121.14746323,26,
-0.12031692,0\C,29,1.40585732,28,117.76366924,27,0.14789552,0\C,26,1.3
9613065,4,73.43474351,3,-91.40560264,0\H,26,1.08695339,4,129.37769632,
3,24.69236151,0\H,27,1.08726697,26,120.3076492,4,-124.43398005,0\H,28,
1.0848007,27,118.3788292,26,179.94285895,0\H,30,1.08396061,29,120.2156
6024,28,-179.99038627,0\H,31,1.08707078,26,120.17926451,4,126.2882857,
0\C,16,4.28308821,15,109.69605466,14,150.87783422,0\C,37,1.39974492,16
,73.16113323,15,143.36801962,0\C,38,1.39550825,37,120.17610041,16,53.9
9546113,0\C,39,1.41238464,38,121.39948936,37,-1.269468,0\C,40,1.403978
57,39,117.57235069,38,3.11484896,0\C,37,1.39484943,16,70.35914177,15,-
86.26262645,0\H,37,1.08688384,16,129.53381051,15,27.56959293,0\H,38,1.
08726637,37,120.27632023,16,-127.43380655,0\H,39,1.08575386,38,118.597
03434,37,175.57940515,0\H,41,1.08305296,40,120.55843289,39,175.8909749
2,0\H,42,1.0870841,37,120.19207852,16,125.69582437,0\C,25,4.38350156,1
,112.96493389,17,169.8570925,0\C,48,1.3982069,25,58.4984605,1,-152.176
09292,0\C,49,1.39620113,48,120.46441187,25,1.48169694,0\C,50,1.4090030
6,49,121.06813529,48,-0.26251827,0\C,51,1.40466144,50,117.76826756,49,
-1.31998441,0\C,48,1.39526878,25,60.64404048,1,27.46146581,0\H,48,1.08
682108,25,178.66601163,1,171.36682033,0\H,49,1.08707986,48,120.2249303
5,25,-178.59850672,0\H,50,1.08550597,49,118.94149283,48,-179.76019355,
0\H,52,1.08411027,51,121.00675939,50,-176.14052184,0\H,53,1.08720483,4
8,120.29441484,25,178.57147749,0\H,13,1.09176518,1,108.92855995,17,-56
.5898976,0\H,13,1.09448946,1,108.58749963,17,60.63395095,0\H,13,1.0939
0102,1,111.54027419,17,-177.50069858,0\\Version=AM64L-G03RevD.01\State
=1-A\HF=-1568.7235168\MP2=-1573.4047376\RMSD=8.383e-09\Thermal=0.\PG=C
01 [X(C32H28P1)]\\@

260

1\1\GINC-GOLEM\SP\RMP2-FC\6-31+G(2d,p)\C23H24N1O1P1\RAMAN\03-Dec-2008\
0\\#p MP2(FC)/6-31+g(2d,p) scf=tight int=finegrid\\orthokatsp003\\0,1\
C\C,1,1.40540596\C,2,1.40783269,1,118.75190254\C,3,1.39629306,2,120.72
364304,1,-1.07611363,0\C,4,1.39933534,3,120.05822282,2,0.96494986,0\C,
5,1.39769989,4,119.71691616,3,-0.23471598,0\P,2,1.84554832,1,124.01774
919,6,178.51990785,0\C,7,1.84837634,2,103.62171871,1,-10.37389083,0\C,
8,1.40551369,7,124.335761,2,95.26845698,0\C,9,1.39898548,8,120.4680059
3,7,178.64781977,0\C,10,1.39807813,9,120.3179428,8,0.28956193,0\C,11,1
.39911922,10,119.74347648,9,-0.43631836,0\C,12,1.39686928,11,119.99354
475,10,-0.20846858,0\C,7,1.85195206,2,102.83681992,1,97.23398553,0\C,1
4,1.40475726,7,122.3741463,2,-23.50175005,0\C,15,1.3959823,14,121.5043
4278,7,179.71853941,0\C,16,1.39739266,15,119.15486675,14,-0.13415139,0
\C,17,1.39494754,16,120.99693849,15,-0.56478923,0\C,18,1.40716475,17,1
19.91584063,16,0.35373442,0\N,19,1.40792906,18,122.05068627,17,-179.01
785678,0\C,20,1.38106539,19,129.76327805,18,-8.21633238,0\C,21,1.55401
568,20,114.09179791,19,-178.46480916,0\C,22,1.54008808,21,108.08964829
,20,-175.70313346,0\O,21,1.22266883,20,123.5453457,19,0.44341437,0\C,2
2,1.54997179,21,109.15546567,20,65.26913668,0\C,22,1.54894429,21,110.7
549245,20,-55.95609667,0\H,17,1.08796315,16,120.11639682,15,179.853673
11,0\H,18,1.08214073,17,121.20863268,16,-179.8944147,0\H,3,1.08873539,
2,119.62575346,1,178.86631125,0\H,1,1.08715149,6,119.67736705,5,-179.9

3947612,0\H,4,1.08781649,3,119.79093359,2,-179.653286,0\H,6,1.08800715
,5,120.08082714,4,179.78427937,0\H,5,1.08771221,4,120.12859529,3,179.4
0600068,0\H,9,1.08689129,8,119.79096427,7,-1.00812593,0\H,13,1.0890138
4,12,119.63193614,11,-179.01128039,0\H,10,1.0879615,9,119.61590696,8,-
179.7604667,0\H,12,1.08782362,11,120.1695114,10,-179.55547537,0\H,11,1
.08778024,10,120.13391239,9,179.99314924,0\H,16,1.08732192,15,120.1555
0438,14,179.8267273,0\H,15,1.08738441,14,118.92935597,7,-0.13743372,0\
H,26,1.09704493,22,111.17096393,21,-55.90597748,0\H,26,1.09788283,22,1
12.90746573,21,65.69041537,0\H,26,1.09763956,22,109.50586219,21,-175.0
759855,0\H,23,1.09552248,22,111.11015532,21,61.23676351,0\H,23,1.09743
084,22,109.69152798,21,-178.69662985,0\H,23,1.09448538,22,111.10249778
,21,-58.37414013,0\H,25,1.09698769,22,110.91624256,21,57.88003577,0\H,
25,1.09776724,22,109.75185489,21,177.16013496,0\H,25,1.09745044,22,112
.47712921,21,-63.01772104,0\H,20,1.01490547,19,113.83840177,18,170.711
9904,0\\Version=AM64L-G03RevD.01\State=1-A\HF=-1355.0986717\MP2=-1358.
8985422\RMSD=5.347e-09\Thermal=0.\PG=C01 [X(C23H24N1O1P1)]\\@

260-Me$^+$

1\1\GINC-CALYPSO\SP\RMP2-FC\6-31+G(2d,p)\C24H27N1O1P1(1+)\RAMAN\21-Dec
-2008\0\\#p MP2(FC)/6-31+g(2d,p) scf=tight int=finegrid\\metorthokatsp
005\\1,1\C\C,1,1.40662\C,2,1.411627,1,120.04\C,3,1.394883,2,119.805,1,
-0.461,0\C,4,1.400289,3,120.05,2,0.216,0\C,5,1.39738,4,120.2,3,0.02,0\
P,2,1.809811,3,119.018,4,-179.145,0\C,7,1.809554,2,111.703,3,165.182,0
\C,8,1.409897,7,119.33,2,-104.517,0\C,9,1.394108,8,120.424,7,175.021,0
\C,10,1.398073,9,119.729,8,-0.216,0\C,11,1.395502,10,120.409,9,1.217,0
\C,12,1.399958,11,120.595,10,-0.499,0\N,13,1.423513,8,120.772,7,5.578,
0\C,14,1.393507,13,122.056,8,-126.427,0\O,15,1.220911,14,120.403,13,12
.857,0\C,7,1.817559,8,111.094,9,129.49,0\C,17,1.409418,7,118.405,8,-47
.341,0\C,18,1.39629,17,119.78,7,-177.336,0\C,19,1.400199,18,120.075,17
,0.184,0\C,20,1.398464,19,120.199,18,-0.264,0\C,21,1.398401,20,120.236
,19,0.038,0\C,7,1.827743,17,105.972,18,70.002,0\C,15,1.54534,14,117.46
2,13,-169.817,0\C,24,1.547348,15,106.969,14,133.846,0\C,24,1.54286,15,
114.187,14,12.498,0\C,24,1.549728,15,106.537,14,-109.028,0\H,11,1.0867
23,10,120.076,9,-179.432,0\H,12,1.085127,11,120.556,10,179.663,0\H,22,
1.08655,17,120.514,18,179.195,0\H,18,1.088651,17,120.629,22,-179.392,0
\H,21,1.086775,22,119.576,17,-179.906,0\H,19,1.086729,18,119.706,17,-1
79.907,0\H,20,1.086856,21,119.939,22,179.908,0\H,3,1.087845,2,121.115,
7,0.98,0\H,1,1.085761,2,121.457,7,-1.211,0\H,4,1.086697,3,119.66,2,179
.897,0\H,6,1.086524,5,120.423,4,179.888,0\H,5,1.08696,4,119.807,3,179.
793,0\H,10,1.08624,9,119.786,8,-179.733,0\H,9,1.085575,8,120.69,7,-4.2
73,0\H,25,1.097504,24,111.26,15,-66.569,0\H,25,1.093673,24,110.676,15,
53.875,0\H,25,1.096526,24,109.711,15,174.03,0\H,26,1.099039,24,112.596
,15,-65.01,0\H,26,1.098382,24,111.992,15,58.389,0\H,26,1.096272,24,109
.261,15,176.734,0\H,27,1.096692,24,109.818,15,-176.178,0\H,27,1.094954
,24,110.566,15,-56.421,0\H,27,1.098354,24,111.39,15,64.144,0\H,14,1.01
1861,13,117.536,8,68.962,0\H,23,1.09442,7,110.793,17,174.451,0\H,23,1.
09493,7,110.203,17,-63.41,0\H,23,1.094663,7,108.546,17,55.004,0\\Versi
on=AM64L-G03RevD.01\State=1-A\HF=-1394.5437745\MP2=-1398.4955268\RMSD=
4.439e-09\Thermal=0.\PG=C01 [X(C24H27N1O1P1)]\\@

261

1\1\GINC-CIPCLU04\SP\RMP2-FC\6-31+G(2d,p)\C10H15P1\C2175\11-Aug-2010\0
\\#p MP2(FC)/6-31+G(2d,p) scf=tight\\yetsp_1\\0,1\P\C,1,4.67882178\C,2
,1.39992077,1,61.027504\C,3,1.39700118,2,120.08443872,1,-0.00040411,0\
C,4,1.40783739,3,120.97245611,2,0.00036283,0\C,5,1.40572947,4,118.1708
0315,3,-0.00136136,0\C,2,1.39698358,1,58.69256164,5,-179.98976491,0\H,
2,1.08791743,1,178.88942592,5,179.98456588,0\H,3,1.08821279,2,120.0419
7702,1,180.,0\H,4,1.08872602,3,118.96478731,2,-179.99965549,0\H,6,1.08

84614,5,119.12135711,4,-179.99914815,0\H,7,1.08799612,2,120.13012289,1
,-179.99899487,0\C,1,1.87660844,5,101.21102148,4,50.92897122,0\H,13,1.
0997976,1,110.8606675,5,-52.81211726,0\H,13,1.09971327,1,106.17764161,
5,-168.38650097,0\C,1,1.87663472,5,101.2090313,4,-50.93056296,0\H,16,1
.09979477,1,110.85741058,5,52.81282448,0\H,16,1.09971083,1,106.1801158
,5,168.38766406,0\C,16,1.53719279,1,112.62063021,5,-71.28748698,0\H,19
,1.0974884,16,110.86516138,1,-177.4041738,0\H,19,1.09704963,16,111.169
49329,1,-57.33265436,0\H,19,1.0968229,16,111.09973507,1,62.71885338,0\
C,13,1.53719508,1,112.62052019,5,71.28940793,0\H,23,1.09748776,13,110.
86520729,1,177.4072456,0\H,23,1.09682574,13,111.09974064,1,-62.7158333
3,0\H,23,1.09704946,13,111.16949928,1,57.33546382,0\\Version=AM64L-G03
RevD.01\State=1-A\HF=-728.1846863\MP2=-729.7770168\RMSD=6.504e-09\Ther
mal=0.\PG=C01 [X(C10H15P1)]\\@

261-Me⁺

1\1\GINC-CIPCLU04\SP\RMP2-FC\6-31+G(2d,p)\C11H18P1(1+)\C2175\11-Aug-20
10\0\\#p MP2(FC)/6-31+G(2d,p) scf=tight\\yetmesp_1\\1,1\C\C,1,1.400254
46\C,2,1.39469192,1,120.05447169\C,3,1.4104444,2,119.87375572,1,-0.000
40568,0\C,4,1.40700545,3,119.84631342,2,0.0015705,0\C,5,1.39724014,4,1
19.80512417,3,-0.00194255,0\H,1,1.08676452,6,119.89143004,5,-179.99959
186,0\H,2,1.08651059,1,120.22051174,6,-179.99925006,0\H,3,1.08859982,2
,119.13838074,1,-179.99959203,0\H,5,1.08652065,4,121.02504732,3,-179.9
9927933,0\H,6,1.08650103,5,119.60308696,4,-179.99847907,0\C,4,2.985843
45,3,97.89081965,2,149.85829753,0\H,12,1.09883206,4,141.31738419,3,125
.41108802,0\H,12,1.09844151,4,89.38637994,3,8.9117973,0\C,12,2.9677544
9,4,60.20019036,3,94.49914557,0\H,15,1.09883235,12,85.55096578,4,161.2
2574085,0\H,15,1.09843843,12,85.87915041,4,-91.65288716,0\C,15,1.54037
869,12,150.31260929,4,34.43813151,0\H,18,1.09476561,15,109.06508041,12
,-177.74125997,0\H,18,1.09552122,15,111.93259513,12,63.43198244,0\C,12
,1.54037368,4,94.42449015,3,-101.80001913,0\H,21,1.09520052,12,111.550
47651,4,29.09214428,0\H,21,1.0947658,12,109.06471911,4,148.22928359,0\
P,4,1.80413224,3,118.71347041,2,179.99049029,0\C,24,1.81922306,4,110.4
7635312,3,179.94826581,0\H,25,1.09465584,24,110.6455404,4,60.63226511,
0\H,25,1.09605034,24,108.81549621,4,-179.92831464,0\H,25,1.09465155,24
,110.65219483,4,-60.4823639,0\H,18,1.09520007,15,111.54926429,12,-58.6
0403835,0\H,21,1.09552159,12,111.9322612,4,-92.945172,0\\Version=AM64L
-G03RevD.01\State=1-A\HF=-767.6343441\MP2=-769.371333\RMSD=4.671e-09\T
hermal=0.\PG=C01 [X(C11H18P1)]\\@

261-BH⁺

1\1\GINC-NODE11\SP\RMP2-FC\6-31+G(2d,p)\C23H26P1(1+)\ZIP07\18-Feb-2011
\0\\#p MP2(FC)/6-31+G(2d,p) scf=tight\\yetbhsp_1\\1,1\C\H,1,1.10017341
\C,1,4.3435875,2,105.57790247\C,3,1.39809545,1,58.91358806,2,16.794854
54,0\C,4,1.39705907,3,120.12478773,1,-0.54582726,0\C,5,1.40537063,4,12
0.75084459,3,0.43751938,0\C,6,1.40629242,5,118.68716589,4,-0.45023872,
0\C,3,1.39744965,1,60.74012162,6,-16.59442819,0\H,3,1.08702971,1,178.9
9110196,6,-171.51173345,0\H,4,1.08717793,3,120.19414691,1,179.43676119
,0\H,5,1.08898947,4,119.49434231,3,179.35029406,0\H,7,1.08779763,6,120
.55104448,5,-179.85770252,0\H,8,1.08736913,3,120.13714434,1,-179.46716
365,0\C,1,4.34759838,6,117.66191196,5,-101.08600438,0\C,14,1.39823807,
1,59.33912792,6,140.44719097,0\C,15,1.39755027,14,119.98425732,1,-0.14
885812,0\C,16,1.40662445,15,120.75180649,14,0.32701425,0\C,17,1.403835
32,16,118.80639779,15,-0.76562314,0\C,18,1.39791272,17,120.36962796,16
,0.54408312,0\H,14,1.08710862,1,179.36300666,6,104.26464939,0\H,15,1.0
8725359,14,120.26151728,1,-179.11153653,0\H,16,1.08945251,15,119.32180
978,14,-178.16416926,0\H,18,1.08569374,17,120.27007426,16,-177.9304174
9,0\H,19,1.08730102,18,119.48197763,17,-179.31863588,0\P,1,1.8792422,6
,112.12202644,5,127.58630678,0\C,25,4.61306385,1,114.46848936,6,80.272

37508,0\C,26,1.39885835,25,59.69626822,1,68.80206557,0\C,27,1.39562992
,26,120.19577341,25,-0.50515201,0\C,28,1.41070488,27,119.92874553,26,-
0.02503334,0\C,29,1.40881308,28,119.58593393,27,0.0655133,0\C,30,1.396
43226,29,119.98029978,28,-0.09712229,0\H,26,1.08709145,25,179.31032106
,1,127.88946621,0\H,27,1.08683872,26,120.23184504,25,179.4235019,0\H,2
8,1.08663372,27,119.22879844,26,-179.59450949,0\H,30,1.08656446,29,120
.92106063,28,179.77934124,0\H,31,1.08684941,30,119.57942289,29,179.800
30998,0\C,25,1.83611027,1,108.82403034,6,-43.37486807,0\H,37,1.0967651
1,25,106.83352532,1,61.76417576,0\H,37,1.09768873,25,107.08808043,1,17
6.95201183,0\C,25,1.83894872,1,107.44720106,6,-160.84536582,0\H,40,1.0
9699466,25,106.92185499,1,-59.6930407,0\H,40,1.09848844,25,106.8985746
1,1,54.1072933,0\C,40,1.53983488,25,113.91616161,1,177.12363241,0\H,43
,1.09526849,40,109.19156549,25,177.20004383,0\H,43,1.09588329,40,111.8
2742793,25,-63.80584166,0\H,43,1.09515782,40,111.54746485,25,57.941656
2,0\C,37,1.54172549,25,114.0201529,1,-59.99977313,0\H,47,1.0951792,37,
108.83169819,25,-179.48420252,0\H,47,1.095751,37,112.27585411,25,-60.5
9267152,0\H,47,1.09530427,37,111.58563055,25,62.12769002,0\\Version=AM
64L-G03RevD.01\State=1-A\HF=-1226.7513056\MP2=-1230.182006\RMSD=4.942e
-09\Thermal=0.\PG=C01 [X(C23H26P1)]\\@

262

1\1\GINC-YIN\SP\RMP2-FC\6-31+G(2d,p)\C25H27N2O1P1\CHRISTOPH\12-Dec-200
8\0\\#p MP2(FC)/6-31+g(2d,p) scf=tight\\hexospc016\\0,1\N\H,1,1.014956
97\C,1,1.38941879,2,108.52087186\O,3,1.22463234,1,121.16115887,2,-5.14
940634,0\N,3,1.39298889,1,114.05829255,4,-177.65395091,0\H,5,1.0115015
9,3,115.40136277,1,15.38112573,0\C,5,1.40425074,3,127.8899395,1,178.54
060488,0\C,7,1.42728801,5,119.20051845,3,-170.8947514,0\C,7,1.40945563
,5,121.54704276,3,10.90980157,0\C,8,1.4094959,7,118.29611353,5,-178.67
291079,0\C,9,1.39413318,7,120.52221417,5,177.97926932,0\H,9,1.08183123
,7,118.63035349,5,-2.71587767,0\C,10,1.39383151,8,122.25722234,7,0.945
68927,0\H,10,1.08823408,8,118.10821899,7,-178.99171377,0\H,11,1.088061
99,9,118.71268438,7,-179.92991117,0\H,13,1.08693649,10,120.41533515,8,
179.70897727,0\P,8,1.84744078,7,128.97659152,5,1.46837576,0\C,17,1.859
29329,8,104.50538869,7,40.34887466,0\C,18,1.40716768,17,123.7151644,8,
-133.14819081,0\C,18,1.41040038,17,117.68621308,8,53.41554424,0\C,19,1
.40066104,18,120.69092869,17,-173.80282982,0\H,19,1.08751715,18,119.98
426414,17,6.33458459,0\C,20,1.39538086,18,120.87882743,17,174.70422213
,0\H,20,1.08848515,18,119.64959206,17,-6.09327889,0\C,21,1.39683278,19
,120.39229022,18,-0.18527135,0\H,21,1.08831651,19,119.47764803,18,179.
89705786,0\H,23,1.08774801,20,119.58992091,18,179.23099544,0\H,25,1.08
747244,21,120.309734,19,-179.82236608,0\C,17,1.85128817,8,105.31003601
,7,-68.63007175,0\C,29,1.40984593,17,116.94042421,8,-170.28107069,0\C,
29,1.40618123,17,124.49193389,8,4.12780967,0\C,30,1.39725126,29,120.99
939641,17,176.94901874,0\H,30,1.08915984,29,119.58824968,17,-2.3166054
7,0\C,31,1.39940004,29,120.59791888,17,-175.58977984,0\H,31,1.08641442
,29,120.01713946,17,3.42559964,0\C,34,1.39837437,31,120.43742691,29,-0
.30255681,0\H,32,1.08787225,30,119.77035918,29,179.58058375,0\H,34,1.0
8807642,31,119.52431526,29,179.58523698,0\H,36,1.08767843,34,120.21208
912,31,-179.97843418,0\C,1,1.46904533,3,126.72342181,4,-146.58018377,0
\C,40,1.54455525,1,112.46303567,3,71.85582626,0\C,40,1.53992092,1,109.
13837599,3,-164.24647888,0\H,40,1.09773859,1,108.32123875,3,-47.927782
57,0\C,41,1.54010663,40,111.7649692,1,177.2066456,0\H,41,1.10176823,40
,108.42168493,1,56.20101064,0\H,41,1.09837574,40,109.32802781,1,-60.02
511493,0\C,42,1.54007481,40,111.46992877,1,-178.98493632,0\H,42,1.1025
5589,40,108.85799761,1,-58.34601664,0\H,42,1.09749005,40,109.03468089,
1,58.11179573,0\C,44,1.53909176,41,111.38624843,40,-54.86349461,0\H,44
,1.09853531,41,109.79279793,40,-177.40067793,0\H,44,1.10066675,41,109.
22113398,40,66.17633521,0\H,47,1.09837893,42,109.7570882,40,177.882669

15,0\H,47,1.10163025,42,109.07139924,40,-65.7257025,0\H,50,1.09847175,
44,110.2773573,41,177.36100731,0\H,50,1.10137396,44,109.3027331,41,-65
.70182499,0\\Version=AM64L-G03RevD.01\State=1-A\HF=-1487.0541256\MP2=-
1491.3287622\RMSD=3.075e-09\Thermal=0.\PG=C01 [X(C25H27N2O1P1)]\\@

262-Me$^+$

1\1\GINC-CALYPSO\SP\RMP2-FC\6-31+G(2d,p)\C26H30N2O1P1(1+)\CHRISTOPH\22
-Jan-2009\0\\#p MP2(FC)/6-31+g(2d,p) scf=tight\\hexomespc004\\1,1\N\H,
1,1.01331879\C,1,1.36836691,2,111.66799249\O,3,1.22459444,1,123.478548
45,2,-5.14116705,0\N,3,1.40537008,1,115.38542588,4,-176.38659895,0\H,5
,1.01144617,3,119.4787532,1,0.07310489,0\C,5,1.41426198,3,121.85029814
,1,-167.25594057,0\C,7,1.41717432,5,120.10670277,3,-132.88722023,0\C,7
,1.40166779,5,120.86764465,3,47.34123223,0\C,8,1.41042859,7,119.788025
78,5,-176.83900165,0\C,9,1.39449263,7,120.50480053,5,178.18954126,0\H,
9,1.08476447,7,118.86582659,5,-2.10930263,0\C,10,1.39301765,8,120.3584
1071,7,-1.98207563,0\H,10,1.08565011,8,120.64561809,7,178.98960167,0\H
,11,1.08682629,9,119.38467547,7,-179.83517522,0\H,13,1.0861833,10,119.
85445338,8,-179.67925331,0\C,8,2.99223563,7,105.08309565,5,41.55833692
,0\C,17,1.40691536,8,87.98467272,7,43.43483728,0\C,17,1.411564,8,150.3
7069369,7,-154.48948213,0\C,18,1.39761856,17,119.46472935,8,170.062585
55,0\H,18,1.08607076,17,121.61210743,8,-10.3754183,0\C,19,1.39496287,1
7,119.81816922,8,-159.3292025,0\H,19,1.08772844,17,121.14324666,8,20.6
6976043,0\C,20,1.39736349,18,120.44336122,17,-0.19235713,0\H,20,1.0864
8575,18,119.11272808,17,179.86282884,0\H,22,1.08671906,19,119.6564471,
17,179.78655223,0\H,24,1.08699887,20,119.99090108,18,-179.71023105,0\C
,8,2.9921133,7,97.87717588,5,-19.72076366,0\C,28,1.4056635,8,139.58843
599,7,61.68160221,0\C,28,1.40944992,8,93.57819823,7,-86.21491277,0\C,2
9,1.39852046,28,119.65190226,8,-142.5904645,0\H,29,1.08657338,28,120.4
1686695,8,36.87592677,0\C,30,1.39649363,28,119.8255929,8,156.73197585,
0\H,30,1.08862507,28,120.65122877,8,-22.70023007,0\C,31,1.39890896,29,
120.24929199,28,0.26436459,0\H,31,1.0868977,29,119.55971872,28,-179.80
262303,0\H,33,1.08678153,30,119.72402927,28,179.97097635,0\H,35,1.0870
0982,31,119.93687502,29,-179.92699698,0\C,1,1.46777218,3,128.1695998,4
,-164.09401943,0\C,39,1.5450038,1,112.96359907,3,68.65274594,0\C,39,1.
54031971,1,109.30927451,3,-167.53178321,0\H,39,1.10262869,1,107.923136
03,3,-51.95196206,0\C,40,1.53963374,39,111.24411354,1,179.18350073,0\H
,40,1.1001935,39,108.25564987,1,58.33088499,0\H,40,1.09904688,39,110.1
0309598,1,-57.86723139,0\C,41,1.54017812,39,111.15151849,1,179.2371186
5,0\H,41,1.10185229,39,108.7345575,1,-60.28017265,0\H,41,1.09797382,39
,109.54861283,1,56.41698127,0\C,43,1.53904849,40,111.31658257,39,-55.7
6066176,0\H,43,1.0973414,40,109.76176995,39,-178.37063864,0\H,43,1.102
33519,40,109.25269303,39,65.14721023,0\H,46,1.09721168,41,109.51243327
,39,177.88306722,0\H,46,1.10174373,41,109.03585571,39,-65.8870316,0\H,
49,1.09757548,43,110.25191416,40,177.08717509,0\H,49,1.10049425,43,109
.23114399,40,-66.10718329,0\P,8,1.80682735,7,120.50730496,5,8.19088052
,0\C,56,1.82744133,8,108.32090188,7,-170.87445502,0\H,57,1.09442295,56
,108.71916636,8,174.45230836,0\H,57,1.0943379,56,110.80058442,8,-65.91
675511,0\H,57,1.09496614,56,110.09247349,8,56.03346806,0\\Version=AM64
L-G03RevD.01\State=1-A\HF=-1526.5093751\MP2=-1530.9230303\RMSD=5.227e-
09\Thermal=0.\PG=C01 [X(C26H30N2O1P1)]\\@

263

1\1\GINC-GOLEM\SP\RMP2-FC\6-31+G(2d,p)\C25H21N2P1S1\CHRISTOPH\13-Nov-2
008\0\\#p MP2(FC)/6-31+g(2d,p) scf=tight\\phsspc22\\0,1\C\C,1,1.397103
39\C,2,1.39927948,1,120.46618668\C,3,1.40460608,2,120.46670863,1,0.092
61795,0\C,4,1.41046004,3,118.56226748,2,-1.39107246,0\C,5,1.39639565,4
,120.83540055,3,1.99872615,0\P,4,1.84571371,3,124.52483739,2,-175.5323
606,0\C,7,1.85857621,4,104.22493009,3,-110.72885874,0\C,8,1.40704917,7

,124.71739155,4,-8.90123301,0\C,9,1.40001232,8,120.62982689,7,-174.233
09651,0\C,10,1.39654598,9,120.38431235,8,-0.28615807,0\C,11,1.39975925
,10,119.58196033,9,0.05426822,0\C,12,1.39560937,11,120.15598667,10,0.4
5908494,0\C,7,1.85222496,4,105.71233979,3,-2.28504787,0\C,14,1.4098040
8,7,113.66723738,4,118.3452074,0\C,15,1.39512586,14,122.04703426,7,179
.98970626,0\C,16,1.39689179,15,119.2543954,14,-1.10947345,0\C,17,1.393
84748,16,120.14601881,15,0.1231805,0\C,18,1.4030178,17,120.64786731,16
,1.10562563,0\N,19,1.41972561,18,118.98992507,17,-178.82843548,0\C,20,
1.36714818,19,126.72264195,18,-50.28380529,0\N,21,1.37469737,20,114.98
332821,19,172.08986269,0\C,22,1.4173093,21,131.13589291,20,-22.5606011
,0\C,23,1.40562042,22,117.66414137,21,153.8878708,0\C,24,1.39530893,23
,120.26747259,22,175.59343583,0\C,25,1.39884584,24,120.35780688,23,0.8
9021659,0\C,26,1.39805736,25,119.32883391,24,-0.53203808,0\C,27,1.3966
9422,26,120.86931712,25,-0.43064367,0\H,17,1.08744402,16,120.45221919,
15,-179.18306175,0\H,16,1.08717628,15,120.14699394,14,179.32161873,0\H
,15,1.08789725,14,118.26080055,7,-0.38616172,0\H,18,1.08492186,17,120.
33636082,16,-179.55101214,0\H,20,1.01737194,19,115.90145365,18,139.548
49046,0\H,22,1.01248915,21,111.86974654,20,168.07096637,0\H,24,1.08817
387,23,119.32260177,22,-4.25700472,0\H,28,1.08569544,27,120.10799259,2
6,-177.73643176,0\H,25,1.08769127,24,119.39738414,23,-179.40506332,0\H
,27,1.08748359,26,120.08359496,25,-179.38456489,0\H,26,1.08714804,25,1
20.33136381,24,179.78355016,0\H,9,1.08729043,8,119.84091064,7,5.668755
45,0\H,13,1.08901628,12,119.5010014,11,178.89263274,0\H,10,1.08757167,
9,119.50749701,8,-179.28287421,0\H,12,1.08805017,11,120.15919494,10,17
9.86788616,0\H,11,1.08743477,10,120.17557015,9,-179.74436687,0\H,3,1.0
861213,2,118.79454983,1,179.98360674,0\H,5,1.08933837,4,119.76660222,3
,-177.79071026,0\H,2,1.08764356,1,120.40782936,6,-179.85394963,0\H,6,1
.08800679,5,119.73967228,4,179.51844266,0\H,1,1.08784666,2,120.2426100
8,3,-179.92243528,0\S,21,1.68451955,20,125.87529839,19,-10.85545796,0\
\Version=AM64L-G03RevD.01\State=1-A\HF=-1806.1687064\MP2=-1810.3229538
\RMSD=4.002e-09\Thermal=0.\PG=C01 [X(C25H21N2P1S1)]\\@

263-Me⁺

1\1\GINC-EDDY\SP\RMP2-FC\6-31+G(2d,p)\C26H24N2P1S1(1+)\CHRISTOPH\15-No
v-2008\0\\#p MP2(FC)/6-31+g(2d,p) scf=tight\\phsmespc22\\1,1\C\C,1,1.3
9676042\C,2,1.39822973,1,120.3511144\C,3,1.4048521,2,119.44847483,1,0.
43333031,0\C,4,1.41203509,3,120.20644939,2,-0.47094544,0\C,5,1.3939971
4,4,119.70414259,3,0.30418271,0\C,4,2.99675644,3,134.65486612,2,142.31
582014,0\C,7,1.40661392,4,88.01558444,3,-90.470434,0\C,8,1.3975862,7,1
19.69682512,4,-174.61820798,0\C,9,1.39780017,8,120.21824909,7,-0.03750
735,0\C,10,1.3997423,9,120.24514205,8,-0.23638508,0\C,11,1.39526593,10
,120.04073668,9,0.0810987,0\C,7,2.98512174,4,60.47658769,3,49.42843869
,0\C,13,1.41030515,7,131.59102743,4,113.3073555,0\C,14,1.39316712,13,1
20.61110904,7,141.0507698,0\C,15,1.39831178,14,119.64257352,13,-0.3948
6917,0\C,16,1.39454342,15,120.47400018,14,-0.49335865,0\C,17,1.4005617
7,16,120.58201257,15,0.66724573,0\N,18,1.41999223,17,120.50972651,16,-
178.16965073,0\C,19,1.38135617,18,124.7960788,17,-49.56200364,0\N,20,1
.36475511,19,115.23663862,18,163.8543727,0\C,21,1.42417793,20,130.1579
6411,19,-11.89634892,0\C,22,1.40416479,21,118.41076121,20,137.53263377
,0\C,23,1.39630585,22,119.93360219,21,176.58624135,0\C,24,1.39968793,2
3,120.33173738,22,1.20559799,0\C,25,1.39823081,24,119.66488756,23,-1.0
9732578,0\C,26,1.39796009,25,120.49218345,24,-0.26115281,0\H,16,1.0866
4359,15,120.1123393,14,-179.81176712,0\H,15,1.086195,14,119.82319699,1
3,179.51299181,0\H,14,1.08523124,13,120.48582711,7,-39.58701158,0\H,17
,1.08480932,16,120.46708129,15,-179.7771607,0\H,19,1.01668805,18,116.8
050615,17,116.81768544,0\H,21,1.01355122,20,112.72655179,19,169.441850
08,0\H,23,1.08781861,22,119.53536018,21,-2.70536126,0\H,27,1.08712525,
26,120.14076715,25,-175.59263378,0\H,24,1.08727327,23,119.48857183,22,

-178.67126106,0\H,26,1.08712366,25,120.15723506,24,-178.14420279,0\H,2
5,1.08686337,24,120.16166883,23,-179.78332696,0\H,8,1.0866272,7,120.43
75165,4,5.57076008,0\H,12,1.08818637,11,119.30894236,10,-179.19346873,
0\H,9,1.08611175,8,119.53926737,7,-179.25961928,0\H,11,1.08668169,10,1
20.26174369,9,-179.80250589,0\H,10,1.08683099,9,119.89825676,8,-179.97
65197,0\H,3,1.08612624,2,119.21647155,1,179.26602121,0\H,5,1.08836281,
4,120.95843742,3,179.85877602,0\H,2,1.08642822,1,120.50724526,6,179.43
904259,0\H,6,1.0866994,5,119.70024018,4,-179.98833217,0\H,1,1.08698592
,2,119.95537731,3,179.78127116,0\P,4,1.80640083,3,121.19003874,2,-177.
02799135,0\C,49,1.82777113,4,106.75447029,3,119.42284593,0\H,50,1.0946
2951,49,110.22212129,4,-58.11380505,0\H,50,1.09507857,49,107.91381005,
4,60.31984993,0\H,50,1.09421767,49,111.42002513,4,179.52287796,0\S,20,
1.67847295,19,123.97806565,18,-19.34659329,0\\Version=AM64L-G03RevD.01
\State=1-A\HF=-1845.6212291\MP2=-1849.9178611\RMSD=7.946e-09\Thermal=0
.\PG=C01 [X(C26H24N2P1S1)]\\@

264

1\1\GINC-NODE5\SP\RMP2-FC\6-31+G(2d,p)\C14H15P1\ZIP07\06-Apr-2010\0\\#
p MP2(FC)/6-31+G(2d,p) scf=tight\\xetsp_3\\0,1\P\C,1,4.67316659\C,2,1.
39627539,1,61.06685361\C,3,1.40043493,2,120.32552779,1,-1.47177745,0\C
,4,1.40567073,3,120.83930475,2,0.21753065,0\C,5,1.40986977,4,118.12038
403,3,0.40403554,0\C,6,1.39520827,5,121.06023331,4,-0.9315412,0\H,2,1.
08772488,1,178.21338074,5,179.58888062,0\H,3,1.08819083,2,120.10959889
,1,178.77677406,0\H,4,1.08678398,3,118.80360789,2,-179.65460096,0\H,6,
1.08893668,5,119.50601628,4,179.43964054,0\H,7,1.08802916,6,119.740704
73,5,-179.52719505,0\C,1,4.67182867,5,103.18445735,4,116.96743687,0\C,
13,1.40017044,1,61.0101165,5,-34.47597903,0\C,14,1.39677219,13,120.167
56367,1,0.53527354,0\C,15,1.40758352,14,120.66060259,13,0.15512198,0\C
,16,1.40574299,15,118.55841646,14,0.23649723,0\C,13,1.39713453,1,58.79
339421,5,144.74940877,0\H,13,1.08792541,1,178.85355705,5,165.42125608,
0\H,14,1.08815851,13,120.01026712,1,-179.53883645,0\H,15,1.08823065,14
,119.65479255,13,179.42796686,0\H,17,1.08863946,16,119.34488024,15,179
.18437452,0\H,18,1.08797616,13,120.15554043,1,179.58513353,0\C,1,1.876
69457,5,102.4394883,4,13.19249513,0\H,24,1.09902928,1,110.68675007,5,5
0.39726247,0\H,24,1.09887727,1,106.97344071,5,-66.47217905,0\C,24,1.53
766554,1,111.62761127,5,173.7306329,0\H,27,1.09706735,24,111.23821303,
1,57.66253331,0\H,27,1.09639915,24,111.25564164,1,-62.69418405,0\H,27,
1.09725275,24,110.52450142,1,177.53471121,0\\Version=AM64L-G03RevD.01\
State=1-A\HF=-879.6674328\MP2=-881.7907096\RMSD=9.864e-09\Thermal=0.\P
G=C01 [X(C14H15P1)]\\@

264-Me^{+}

1\1\GINC-NODE5\SP\RMP2-FC\6-31+G(2d,p)\C15H18P1(1+)\ZIP07\06-Apr-2010\
0\\#p MP2(FC)/6-31+G(2d,p) scf=tight\\xetmesp_21\\1,1\C\C,1,1.39867831
\C,2,1.3964101,1,120.11923665\C,3,1.40838651,2,119.81585561,1,0.262501
93,0\C,4,1.40918204,3,119.87742063,2,-0.45274194,0\C,5,1.39582066,4,11
9.79628114,3,0.31732674,0\H,1,1.08681224,2,119.86078082,3,-179.9122812
9,0\H,2,1.08654439,1,120.24308278,6,179.94425546,0\H,3,1.08694939,2,11
9.09539702,1,-179.82457848,0\H,5,1.08731893,4,120.57289048,3,179.80640
297,0\H,6,1.08658682,5,119.64335647,4,179.85462927,0\C,5,5.37859999,4,
88.20659291,3,154.62425325,0\C,12,1.39838792,5,79.7206081,4,66.8194086
,0\C,13,1.39667218,12,120.12929775,5,-25.08128738,0\C,14,1.40796798,13
,119.82307156,12,0.15217276,0\C,15,1.41021036,14,119.8581752,13,-0.065
04121,0\C,16,1.39554123,15,119.80248991,14,-0.02616572,0\H,12,1.086838
82,5,149.44583841,4,-159.6929715,0\H,13,1.08652085,12,120.25038932,5,1
54.72911211,0\H,14,1.08667275,13,119.13116222,12,179.80154132,0\H,16,1
.08723646,15,120.54004439,14,178.99522452,0\H,17,1.08659828,16,119.664
78868,15,179.77283539,0\C,4,2.95457966,3,115.44416492,2,-135.87568261,

0\H,23,1.09530153,4,146.25629014,3,-98.36934435,0\H,23,1.09557778,4,86
.73626238,3,21.38615584,0\H,23,1.09491869,4,92.27699568,3,129.99963908
,0\P,15,1.80622619,14,120.55865856,13,-177.02091448,0\C,27,1.83682383,
15,110.38492562,14,96.55275259,0\H,28,1.09843068,27,107.29425762,15,-1
76.27068607,0\H,28,1.09871266,27,105.14104675,15,-63.16358603,0\C,28,1
.53911966,27,115.28809371,15,58.92036471,0\H,31,1.09488706,28,111.3703
2045,27,-61.04612172,0\H,31,1.09522041,28,111.9766578,27,60.78026447,0
\H,31,1.09494415,28,109.01157669,27,-179.87539584,0\\Version=AM64L-G03
RevD.01\State=1-A\HF=-919.1173867\MP2=-921.3854947\RMSD=3.640e-09\Ther
mal=0.\PG=C01 [X(C15H18P1)]\\@

264-BH$^+$

1\1\GINC-CIPCLU03\SP\RMP2-FC\6-31+G(2d,p)\C27H26P1(1+)\C2175\23-Nov-20
10\0\\#p MP2(FC)/6-31+G(2d,p) scf=tight\\xetbhsp_13\\1,1\C\H,1,1.09930
45\P,1,1.88805101,2,101.50171086\C,1,4.34402184,3,112.07139587,2,112.1
3689511,0\C,4,1.3977451,1,58.98139245,3,-122.52742331,0\C,5,1.39747977
,4,120.10368221,1,0.44530568,0\C,6,1.4046345,5,120.81606524,4,-0.48056
446,0\C,7,1.40706177,6,118.63373205,5,0.5277468,0\C,4,1.39804644,1,60.
65312104,7,11.53530045,0\H,4,1.08682979,1,179.10588437,7,167.75509357,
0\H,5,1.08698329,4,120.22679049,1,-179.51730634,0\H,6,1.08886853,5,119
.46224965,4,-179.47910105,0\H,8,1.08771666,7,120.45569676,6,179.955963
41,0\H,9,1.08719853,4,120.11150862,1,179.53266158,0\C,1,4.34966141,7,1
18.30604869,6,106.46995064,0\C,15,1.39861415,1,59.265767,7,-150.279970
22,0\C,16,1.3971316,15,120.11839,1,0.44219429,0\C,17,1.4072129,16,120.
72236356,15,-0.50582384,0\C,18,1.40385594,17,118.70479034,16,1.1308526
8,0\C,15,1.39745558,1,60.28398124,7,28.83964567,0\H,15,1.0869981,1,179
.4539938,7,-141.22271523,0\H,16,1.08713039,15,120.23481412,1,179.49100
544,0\H,17,1.087946,16,119.19455509,15,178.75887375,0\H,19,1.08516204,
18,120.34778705,17,177.67658972,0\H,20,1.08714234,15,120.13180625,1,-1
79.62470439,0\C,3,4.61167802,1,111.19902339,7,-81.95531102,0\C,26,1.39
853098,3,60.26425881,1,113.98339977,0\C,27,1.39628018,26,120.10199364,
3,0.52513349,0\C,28,1.40906335,27,120.02338118,26,0.11222892,0\C,29,1.
41139134,28,119.64013855,27,-0.35126384,0\C,30,1.39610824,29,119.78378
434,28,0.38549807,0\H,26,1.08694234,3,179.59033885,1,-1.38333511,0\H,2
7,1.08662554,26,120.28199565,3,-179.58574893,0\H,28,1.08589759,27,119.
08760291,26,-179.61953425,0\H,30,1.08621411,29,120.64905689,28,-179.54
996708,0\H,31,1.08663075,30,119.49465244,29,179.98498512,0\C,3,4.61915
392,1,109.69491789,7,153.71773775,0\C,37,1.40021556,3,59.53136083,1,-4
8.66333655,0\C,38,1.39509273,37,120.04209778,3,1.18856751,0\C,39,1.410
51776,38,120.10074914,37,0.18995898,0\C,40,1.4055797,39,119.56741306,3
8,-0.89302663,0\C,37,1.39702995,3,60.56259636,1,130.5596149,0\H,37,1.0
868568,3,178.89659376,1,-107.10757697,0\H,38,1.08671206,37,120.2434663
3,3,-178.74117122,0\H,39,1.0884502,38,118.91951556,37,-179.55398663,0\
H,41,1.08574685,40,120.47682653,39,-178.86536389,0\H,42,1.0866906,37,1
20.21201563,3,178.75295939,0\C,3,1.84040876,1,107.35670421,7,40.000057
58,0\H,48,1.09889923,3,105.1837588,1,43.19050956,0\H,48,1.09615552,3,1
07.51947397,1,-69.22116489,0\C,48,1.54006838,3,114.95666898,1,165.0197
7223,0\H,51,1.09534601,48,108.95567557,3,179.89781468,0\H,51,1.0945257
6,48,111.40270558,3,-60.98185416,0\H,51,1.09513362,48,112.26926344,3,6
0.42206068,0\\Version=AM64L-G03RevD.01\State=1-A\HF=-1378.231858\MP2=-
1382.1973603\RMSD=8.850e-09\Thermal=0.\PG=C01 [X(C27H26P1)]\\@

264-TT$^+$

1\1\GINC-CALYPSO\SP\RMP2-FC\6-31+G(2d,p)\C33H30P1(1+)\CHRISTOPH\25-Aug
-2011\0\\#p MP2(FC)/6-31+g(2d,p) scf=tight\\xetttsp_13\\1,1\P\C,1,4.63
21654\C,2,1.39894742,1,59.72682673\C,3,1.39615536,2,120.3779526,1,-1.2
2439701,0\C,4,1.41181444,3,119.9162658,2,-0.41628658,0\C,5,1.40761514,
4,119.39877945,3,1.25070054,0\C,6,1.39719305,5,120.11335565,4,-1.19372

729,0\H,2,1.08700389,1,178.83145472,5,178.24089785,0\H,3,1.08682786,2,
120.23537363,1,178.5288344,0\H,4,1.08549834,3,119.07900717,2,179.59374
873,0\H,6,1.08500059,5,120.59944761,4,178.27498493,0\H,7,1.08684132,6,
119.4617417,5,179.95254213,0\C,1,1.84893846,5,104.55222393,4,-57.55249
291,0\C,1,4.64601749,5,108.94684983,4,-172.26148806,0\C,14,1.3979041,1
,59.57548502,5,117.37651003,0\C,15,1.39596283,14,120.20389717,1,0.0549
1686,0\C,16,1.41103218,15,120.51355599,14,-0.01756784,0\C,17,1.4104242
6,16,118.84138311,15,0.34035186,0\C,18,1.39714635,17,120.17614986,16,-
0.44046611,0\H,14,1.08690492,1,179.6429606,5,99.07733498,0\H,15,1.0868
0365,14,120.33093421,1,-179.89617169,0\H,16,1.08487064,15,118.49322255
,14,179.24470968,0\H,18,1.08435031,17,120.82106257,16,179.61138009,0\H
,19,1.08678955,18,119.23721731,17,179.98586656,0\C,1,1.97414676,5,111.
45817837,4,62.98460651,0\C,25,4.38364049,1,113.87918599,5,-170.3001788
8,0\C,26,1.39498415,25,60.79825564,1,-24.02670825,0\C,27,1.40032744,26
,120.42576816,25,0.72183798,0\C,28,1.40345548,27,121.14532965,26,1.233
07526,0\C,29,1.40955917,28,117.71871587,27,-1.99820484,0\C,30,1.395712
24,29,121.1371106,28,1.26904499,0\H,26,1.08686302,25,178.52160028,1,-1
71.09781151,0\H,27,1.08727377,26,120.28082355,25,-178.58263023,0\H,28,
1.08354141,27,117.77306931,26,-176.81967436,0\H,30,1.08577261,29,119.8
5794646,28,-178.38549954,0\H,31,1.08714074,30,119.32205912,29,-179.834
24143,0\C,4,4.28024889,3,109.23263611,2,-153.67104726,0\C,37,1.3947838
2,4,68.7292794,3,85.77919059,0\C,38,1.40068809,37,120.71992039,4,54.89
383641,0\C,39,1.40451585,38,121.05431853,37,-0.22023592,0\C,40,1.41302
924,39,117.37515838,38,3.0059329,0\C,41,1.3955169,40,121.52683889,39,-
3.65707799,0\H,37,1.08693506,4,130.04827522,3,-26.83376674,0\H,38,1.08
718378,37,120.18986082,4,-125.64310998,0\H,39,1.08286353,38,118.283880
45,37,178.19998256,0\H,41,1.08600791,40,119.88189392,39,172.86431006,0
\H,42,1.08728422,41,119.51363738,40,179.98820997,0\C,18,4.15372913,17,
124.9216033,16,148.35767984,0\C,48,1.39831547,18,69.65078015,17,67.486
96463,0\C,49,1.39759628,48,120.34854271,18,-56.42843225,0\C,50,1.41266
717,49,121.17784452,48,-0.10110633,0\C,51,1.40601015,50,117.64637248,4
9,0.30510178,0\C,48,1.39594606,18,73.52491209,17,-63.15997921,0\H,48,1
.08702375,18,128.77966013,17,-179.30803056,0\H,49,1.08739673,48,120.29
401511,18,123.81872416,0\H,50,1.08448672,49,118.26822021,48,179.628165
2,0\H,52,1.08399453,51,120.12552864,50,179.76389029,0\H,53,1.08713821,
48,120.19940309,18,-125.56040535,0\H,13,1.09606251,1,107.99276012,5,-1
72.85489255,0\C,13,1.54357308,1,114.92619494,5,-46.57657435,0\H,60,1.0
9390448,13,113.10395357,1,-56.88038673,0\H,60,1.09391494,13,111.054404
67,1,64.66388404,0\H,13,1.0938431,1,105.57163244,5,73.52720352,0\H,60,
1.09585982,13,108.51202134,1,-176.74025834,0\\Version=AM64L-G03RevD.01
\State=1-A\HF=-1607.7549494\MP2=-1612.5960413\RMSD=8.156e-09\Thermal=0
.\PG=C01 [X(C33H30P1)]\\@

265

1\1\GINC-EDDY\SP\RMP2-FC\6-31+G(2d,p)\C25H21N2O1P1\CHRISTOPH\08-Oct-20
08\0\\#p MP2(FC)/6-31+g(2d,p) scf=tight int=finegrid\\Ph_Ospc010\\0,1\
C\C,1,1.3959567\C,2,1.40073338,1,120.43043288\C,3,1.40521466,2,120.567
07279,1,0.10798861,0\C,4,1.41042873,3,118.44042685,2,0.35413597,0\C,5,
1.39483109,4,120.85280653,3,-0.80944078,0\P,4,1.85744964,3,125.1110953
9,2,174.59780898,0\C,7,1.84999218,4,105.16210067,3,14.58994355,0\C,8,1
.41021151,7,118.00684881,4,-70.74947609,0\C,9,1.39661401,8,120.9561074
3,7,-174.44595299,0\C,10,1.39971007,9,120.15554383,8,1.55869013,0\C,11
,1.39819115,10,119.49979325,9,0.00188764,0\C,12,1.39961657,11,120.3658
1502,10,-0.84826107,0\C,7,1.84795539,4,104.19469263,3,124.54803755,0\C
,14,1.4097302,7,112.74381324,4,137.65622781,0\C,15,1.39376807,14,122.3
2981878,7,-179.89573662,0\C,16,1.3960568,15,118.59213402,14,0.42795178
,0\C,17,1.3940359,16,120.97668219,15,-0.1977911,0\C,18,1.40921045,17,1
20.64472963,16,0.02983564,0\N,19,1.40849853,18,120.96365613,17,-177.77

478703,0\C,20,1.38847374,19,126.32679697,18,-19.93656691,0\N,21,1.3924
2289,20,115.41157264,19,-179.55553782,0\C,22,1.41172647,21,132.8248403
,20,-13.29851428,0\C,23,1.40634088,22,118.26215989,21,148.04143447,0\C
,24,1.39506084,23,120.32429014,22,177.70153752,0\C,25,1.39984977,24,12
0.42030828,23,1.15258986,0\C,26,1.39782025,25,119.29515664,24,-1.32249
483,0\C,27,1.39793396,26,120.71217352,25,-0.19099049,0\O,21,1.22297172
,20,125.18809069,19,-0.74077478,0\H,17,1.08797207,16,120.26518472,15,1
79.74456725,0\H,16,1.08696858,15,120.41889033,14,179.99433829,0\H,15,1
.08815112,14,118.02703058,7,-0.22405188,0\H,18,1.08151993,17,120.44747
121,16,179.12166626,0\H,20,1.01316869,19,115.66904496,18,177.6565987,0
\H,22,1.0117966,21,110.27697017,20,168.61553448,0\H,24,1.08814327,23,1
19.29460463,22,-2.97821432,0\H,28,1.08526773,27,119.83525268,26,-177.3
6636077,0\H,25,1.08789407,24,119.39402861,23,-179.57977873,0\H,27,1.08
734204,26,120.40367519,25,-179.41145247,0\H,26,1.08718175,25,120.32583
65,24,179.24878299,0\H,9,1.08884463,8,119.74810075,7,4.94568901,0\H,13
,1.08714433,12,119.39384838,11,179.40986256,0\H,10,1.0879401,9,119.692
05361,8,-179.52389768,0\H,12,1.08812932,11,120.09610012,10,178.9448151
8,0\H,11,1.08780636,10,120.22489995,9,178.94422305,0\H,3,1.08650897,2,
119.0917219,1,-178.96516198,0\H,5,1.08868262,4,119.66459738,3,179.5786
6525,0\H,2,1.08755655,1,120.31860131,6,-179.29457534,0\H,6,1.08788286,
5,119.65381209,4,-179.4454573,0\H,1,1.08770357,2,120.30013177,3,179.91
723606,0\\Version=AM64L-G03RevD.01\State=1-A\HF=-1483.5433303\MP2=-148
7.7540417\RMSD=5.240e-09\Thermal=0.\PG=C01 [X(C25H21N2O1P1)]\\@

265-Me⁺

1\1\GINC-CALYPSO\SP\RMP2-FC\6-31+G(2d,p)\C26H24N2O1P1(1+)\CHRISTOPH\06
-Oct-2008\0\\#p MP2(FC)/6-31+g(2d,p) scf=tight int=finegrid\\Ph_OMespc
021\\1,1\C\C,1,1.39799742\C,2,1.39757723,1,120.19910834\C,3,1.40612701
,2,119.66441554,1,-0.02249798,0\C,4,1.40931484,3,120.01618318,2,-0.428
26195,0\C,5,1.39539121,4,119.81900112,3,0.62310144,0\C,4,3.00163284,3,
87.83855505,2,175.96895195,0\C,7,1.40598109,4,132.30307477,3,88.935028
52,0\C,8,1.39794583,7,119.46039436,4,-141.53484072,0\C,9,1.39740369,8,
120.38643131,7,-0.18702439,0\C,10,1.40042262,9,120.23026053,8,0.228197
63,0\C,11,1.39476321,10,120.01439755,9,-0.19117596,0\C,4,2.99229254,3,
131.73868575,2,-138.70758109,0\C,13,1.40964574,4,133.07508388,3,-167.5
6067661,0\C,14,1.39368929,13,120.37489818,4,-144.5304677,0\C,15,1.3987
0529,14,119.65828798,13,-0.22753156,0\C,16,1.39498557,15,120.56507914,
14,1.53530721,0\C,17,1.4010424,16,120.50888644,15,-0.64179596,0\N,18,1
.41646995,17,120.73319337,16,179.05529703,0\C,19,1.39867485,18,121.097
66334,17,48.39064002,0\N,20,1.37800214,19,115.82049643,18,-166.5352775
6,0\C,21,1.42106262,20,130.21619605,19,7.47886037,0\C,22,1.40427078,21
,118.89962679,20,-133.36137294,0\C,23,1.39664813,22,120.05676274,21,-1
77.18720477,0\C,24,1.39970594,23,120.34653994,22,-1.3833632,0\C,25,1.3
9812207,24,119.59852763,23,1.18861822,0\C,26,1.39831417,25,120.4639930
6,24,0.41973552,0\O,20,1.2218344,19,122.00654821,18,16.38695642,0\H,16
,1.08678301,15,120.02095742,14,-179.25038728,0\H,15,1.08619871,14,119.
84838846,13,-179.53745726,0\H,14,1.08561021,13,120.66428747,4,36.47673
778,0\H,17,1.08495177,16,120.52483092,15,179.8008592,0\H,19,1.01406724
,18,118.62898421,17,-117.53410695,0\H,21,1.01276638,20,111.9025369,19,
-169.15533521,0\H,23,1.08789264,22,119.48247606,21,2.17506578,0\H,27,1
.087348,26,120.18477562,25,175.43935639,0\H,24,1.08742251,23,119.50024
591,22,178.66735322,0\H,26,1.08725996,25,120.14648646,24,178.15241285,
0\H,25,1.08696169,24,120.19428621,23,179.88673355,0\H,8,1.0858049,7,12
1.35613558,4,37.49392871,0\H,12,1.08815946,11,119.24310916,10,179.6958
1508,0\H,9,1.08650192,8,119.19465029,7,179.71119141,0\H,11,1.0867181,1
0,120.29381721,9,179.99439024,0\H,10,1.0870035,9,119.9719882,8,-179.77
498331,0\H,3,1.08657744,2,119.91987282,1,-179.58955502,0\H,5,1.0882510
8,4,120.78759132,3,-178.80963244,0\H,2,1.08607219,1,120.26000396,6,-17

8.92673122,0\H,6,1.08672841,5,119.71436093,4,179.80945642,0\H,1,1.0869
1532,2,119.89801164,3,-179.98321199,0\P,13,1.80803225,4,34.46786429,3,
-86.64208648,0\C,50,1.82728482,13,108.29335936,4,-116.52563059,0\H,51,
1.09447692,50,110.32976051,13,-63.74409828,0\H,51,1.09455422,50,111.00
32343,13,58.38784271,0\H,51,1.09472647,50,108.2707275,13,177.36676927,
0\\Version=AM64L-G03RevD.01\State=1-A\HF=-1523.0000833\MP2=-1527.34865
22\RMSD=5.218e-09\Thermal=0.\PG=C01 [X(C26H24N2O1P1)]\\@

266

1\1\GINC-EDDY\SP\RMP2-FC\6-31+G(2d,p)\C28H33N2O2P1\RAMAN\23-Nov-2008\0
\\#p MP2(FC)/6-31+g(2d,p) scf=tight int=finegrid\\diorthokatsp059\\0,1
\C\C,1,1.397987\C,2,1.394423,1,119.328\C,3,1.413003,2,122.039,1,-0.888
,0\C,4,1.413252,3,117.953,2,2.746,0\C,5,1.405686,4,119.609,3,-2.666,0\
P,4,1.855614,3,111.5,2,-173.204,0\C,7,1.847644,4,102.19,3,94.208,0\C,8
,1.407775,7,124.269,4,33.318,0\C,9,1.398,8,120.125,7,-179.611,0\C,10,1
.399336,9,120.468,8,-0.169,0\C,11,1.397937,10,119.833,9,0.326,0\C,12,1
.398433,11,119.879,10,0.114,0\N,5,1.427121,4,122.319,7,-9.743,0\C,14,1
.379335,5,122.081,4,129.375,0\C,15,1.549752,14,115.881,5,159.96,0\C,16
,1.540068,15,108.298,14,170.678,0\C,7,1.850183,8,102.672,9,-80.626,0\C
,18,1.407319,7,122.114,8,-15.184,0\C,19,1.396982,18,121.188,7,-171.008
,0\C,20,1.397851,19,120.284,18,-0.097,0\C,21,1.395654,20,119.484,19,0.
907,0\C,22,1.398976,21,120.188,20,-0.201,0\N,23,1.431551,18,120.172,19
,-177.791,0\C,24,1.370571,23,122.664,18,-109.856,0\O,25,1.231642,24,12
1.388,23,1.328,0\C,25,1.54694,24,117.558,23,-175.82,0\C,27,1.544874,25
,107.845,24,-137.618,0\C,27,1.54278,25,113.937,24,-15.766,0\C,27,1.550
292,25,106.044,24,104.999,0\O,15,1.225914,14,121.653,5,-18.535,0\C,16,
1.545554,15,111.753,14,49.781,0\C,16,1.551229,15,107.76,14,-70.663,0\H
,19,1.086101,20,119.456,21,179.33,0\H,21,1.08724,20,120.541,19,-179.64
,0\H,22,1.086683,21,120.955,20,179.574,0\H,24,1.011979,23,117.617,18,6
0.678,0\H,28,1.096891,27,111.24,25,67.052,0\H,28,1.093958,27,110.472,2
5,-53.151,0\H,28,1.097471,27,109.767,25,-173.192,0\H,29,1.09695,27,109
.366,25,-177.245,0\H,29,1.098756,27,112.359,25,64.455,0\H,29,1.098198,
27,111.712,25,-58.56,0\H,30,1.097989,27,111.233,25,-63.895,0\H,30,1.09
5291,27,110.523,25,56.595,0\H,30,1.097525,27,109.919,25,176.467,0\H,9,
1.087438,8,120.925,7,-0.087,0\H,13,1.088737,8,119.499,7,0.385,0\H,10,1
.087755,9,119.334,8,179.855,0\H,12,1.087932,13,119.868,8,179.913,0\H,1
1,1.087986,10,120.096,9,179.937,0\H,3,1.08882,4,118.59,7,5.489,0\H,2,1
.087463,3,120.067,4,179.022,0\H,6,1.086026,5,118.378,4,-179.141,0\H,1,
1.08757,2,120.526,3,179.9,0\H,14,1.018879,5,115.439,4,-72.265,0\H,32,1
.098187,16,109.298,15,176.387,0\H,32,1.09584,16,112.707,15,-64.426,0\H
,32,1.0964,16,111.318,15,57.187,0\H,33,1.097919,16,110.954,15,-57.718,
0\H,33,1.096806,16,111.812,15,63.552,0\H,33,1.098011,16,109.696,15,-17
6.751,0\H,17,1.096071,16,111.075,15,-62.086,0\H,17,1.094427,16,111.134
,15,57.559,0\H,17,1.097676,16,109.751,15,178.004,0\H,20,1.087792,21,12
0.158,22,-179.601,0\\Version=AM64L-G03RevD.01\State=1-A\HF=-1679.03971
89\MP2=-1684.0008669\RMSD=6.787e-09\Thermal=0.\PG=C01 [X(C28H33N2O2P1)
]\\@

266-Me^{+}

1\1\GINC-EDDY\SP\RMP2-FC\6-31+G(2d,p)\C28H33N2O2P1\RAMAN\23-Nov-2008\0
\\#p MP2(FC)/6-31+g(2d,p) scf=tight int=finegrid\\diorthokatsp059\\0,1
\C\C,1,1.397987\C,2,1.394423,1,119.328\C,3,1.413003,2,122.039,1,-0.888
,0\C,4,1.413252,3,117.953,2,2.746,0\C,5,1.405686,4,119.609,3,-2.666,0\
P,4,1.855614,3,111.5,2,-173.204,0\C,7,1.847644,4,102.19,3,94.208,0\C,8
,1.407775,7,124.269,4,33.318,0\C,9,1.398,8,120.125,7,-179.611,0\C,10,1
.399336,9,120.468,8,-0.169,0\C,11,1.397937,10,119.833,9,0.326,0\C,12,1
.398433,11,119.879,10,0.114,0\N,5,1.427121,4,122.319,7,-9.743,0\C,14,1
.379335,5,122.081,4,129.375,0\C,15,1.549752,14,115.881,5,159.96,0\C,16

,1.540068,15,108.298,14,170.678,0\C,7,1.850183,8,102.672,9,-80.626,0\C
,18,1.407319,7,122.114,8,-15.184,0\C,19,1.396982,18,121.188,7,-171.008
,0\C,20,1.397851,19,120.284,18,-0.097,0\C,21,1.395654,20,119.484,19,0.
907,0\C,22,1.398976,21,120.188,20,-0.201,0\N,23,1.431551,18,120.172,19
,-177.791,0\C,24,1.370571,23,122.664,18,-109.856,0\O,25,1.231642,24,12
1.388,23,1.328,0\C,25,1.54694,24,117.558,23,-175.82,0\C,27,1.544874,25
,107.845,24,-137.618,0\C,27,1.54278,25,113.937,24,-15.766,0\C,27,1.550
292,25,106.044,24,104.999,0\O,15,1.225914,14,121.653,5,-18.535,0\C,16,
1.545554,15,111.753,14,49.781,0\C,16,1.551229,15,107.76,14,-70.663,0\H
,19,1.086101,20,119.456,21,179.33,0\H,21,1.08724,20,120.541,19,-179.64
,0\H,22,1.086683,21,120.955,20,179.574,0\H,24,1.011979,23,117.617,18,6
0.678,0\H,28,1.096891,27,111.24,25,67.052,0\H,28,1.093958,27,110.472,2
5,-53.151,0\H,28,1.097471,27,109.767,25,-173.192,0\H,29,1.09695,27,109
.366,25,-177.245,0\H,29,1.098756,27,112.359,25,64.455,0\H,29,1.098198,
27,111.712,25,-58.56,0\H,30,1.097989,27,111.233,25,-63.895,0\H,30,1.09
5291,27,110.523,25,56.595,0\H,30,1.097525,27,109.919,25,176.467,0\H,9,
1.087438,8,120.925,7,-0.087,0\H,13,1.088737,8,119.499,7,0.385,0\H,10,1
.087755,9,119.334,8,179.855,0\H,12,1.087932,13,119.868,8,179.913,0\H,1
1,1.087986,10,120.096,9,179.937,0\H,3,1.08882,4,118.59,7,5.489,0\H,2,1
.087463,3,120.067,4,179.022,0\H,6,1.086026,5,118.378,4,-179.141,0\H,1,
1.08757,2,120.526,3,179.9,0\H,14,1.018879,5,115.439,4,-72.265,0\H,32,1
.098187,16,109.298,15,176.387,0\H,32,1.09584,16,112.707,15,-64.426,0\H
,32,1.0964,16,111.318,15,57.187,0\H,33,1.097919,16,110.954,15,-57.718,
0\H,33,1.096806,16,111.812,15,63.552,0\H,33,1.098011,16,109.696,15,-17
6.751,0\H,17,1.096071,16,111.075,15,-62.086,0\H,17,1.094427,16,111.134
,15,57.559,0\H,17,1.097676,16,109.751,15,178.004,0\H,20,1.087792,21,12
0.158,22,-179.601,0\\Version=AM64L-G03RevD.01\State=1-A\HF=-1679.03971
89\MP2=-1684.0008669\RMSD=6.787e-09\Thermal=0.\PG=C01 [X(C28H33N2O2P1)
]\\@

267

1\1\GINC-CIPCLU09\SP\RMP2-FC\6-31+G(2d,p)\C15H15P1\C2175\26-May-2010\0
\\#p MP2(FC)/6-31+G(2d,p) scf=tight\\x3sp_16\\0,1\P\C,1,4.66962762\C,2
,1.39715213,1,58.73976378\C,3,1.3994076,2,119.96124584,1,-1.03469992,0
\C,4,1.40529801,3,120.8461045,2,0.40021171,0\C,5,1.40736877,4,118.5836
071,3,-0.54656724,0\C,6,1.39677711,5,120.64073374,4,0.31214277,0\H,2,1
.08794395,1,178.72672268,5,178.07519088,0\H,3,1.08799684,2,120.1568967
7,1,179.38318607,0\H,4,1.08866979,3,119.77789293,2,-179.30788411,0\H,6
,1.08807777,5,119.69033024,4,-178.99099875,0\H,7,1.08819489,6,119.8108
7664,5,-179.90933817,0\C,1,4.67084254,5,103.0513002,4,143.22428089,0\C
,13,1.39690048,1,60.42485669,5,124.76197434,0\C,14,1.39960865,13,120.3
3319031,1,-1.83839496,0\C,15,1.40599226,14,120.76385959,13,-0.01565287
,0\C,16,1.40906479,15,118.280488,14,0.5656032,0\C,17,1.39591974,16,120
.91961226,15,-0.86640855,0\H,13,1.08775299,1,178.3471634,16,-178.61258
58,0\H,14,1.08823609,13,120.07517756,1,178.35972101,0\H,15,1.08706113,
14,119.38808453,13,179.89983833,0\H,17,1.08868053,16,119.68823094,15,1
79.74868328,0\H,18,1.08808608,17,119.69222096,16,-179.49015657,0\C,1,1
.83959146,16,102.03120036,15,24.0344241,0\C,24,1.52892364,1,119.006654
03,16,-96.89462161,0\C,25,1.50706636,24,60.29095463,1,-106.77949738,0\
H,24,1.09110708,1,116.73292399,16,50.57229316,0\H,25,1.08816455,24,117
.84724474,1,1.10178277,0\H,25,1.088024,24,117.44757159,1,144.33529757,
0\H,26,1.08813244,25,117.91425966,24,108.12342137,0\H,26,1.0873899,25,
118.5847708,24,-106.81218941,0\\Version=AM64L-G03RevD.01\State=1-A\HF=
-917.5035208\MP2=-919.7595762\RMSD=4.778e-09\Thermal=0.\PG=C01 [X(C15H
15P1)]\\@

267-Me$^+$

1\1\GINC-CIPCLU06\SP\RMP2-FC\6-31+G(2d,p)\C16H18P1(1+)\C2175\26-May-20

10\0\\#p MP2(FC)/6-31+G(2d,p) scf=tight\\x3mesp_27\\1,1\C\C,1,1.398977
9\C,2,1.39630147,1,120.11786089\C,3,1.40875201,2,119.7722354,1,-0.2311
3992,0\C,4,1.40915343,3,119.93268461,2,0.31842417,0\C,5,1.3961175,4,11
9.77987218,3,-0.15424341,0\H,1,1.08685166,2,119.87896657,3,-179.698859
29,0\H,2,1.08658745,1,120.21613572,6,179.55544408,0\H,3,1.08724578,2,1
19.8416914,1,178.9092856,0\H,5,1.08714533,4,120.99383051,3,179.3898371
6,0\H,6,1.0865856,5,119.67763629,4,179.62615111,0\C,3,5.26900265,2,141
.84280266,1,141.49894469,0\C,12,1.40053632,3,58.59752573,2,-80.8311025
8,0\C,13,1.3945387,12,120.06729882,3,-36.95869779,0\C,14,1.40985533,13
,119.76554457,12,-0.04924606,0\C,15,1.40658832,14,120.02789868,13,0.32
320793,0\C,16,1.39765303,15,119.70096399,14,-0.3490272,0\H,12,1.086862
31,3,148.37772714,2,16.04300167,0\H,13,1.08658061,12,120.19723901,3,14
3.05437029,0\H,14,1.08810343,13,119.39168156,12,179.45456589,0\H,16,1.
08643708,15,120.60140281,14,179.46514804,0\H,17,1.0865872,16,119.64253
149,15,-179.89431242,0\P,15,1.80599212,14,119.16225783,13,-179.3539091
8,0\C,23,1.82354245,15,108.69974834,14,54.4611115,0\H,24,1.09476725,23
,110.37486318,15,175.08714959,0\H,24,1.09545782,23,108.69566996,15,55.
88322861,0\H,24,1.09469229,23,110.5872443,15,-63.76448404,0\C,23,1.801
23802,15,109.97671474,14,171.81506897,0\C,28,1.53285145,23,120.9659320
4,15,155.70290704,0\C,29,1.50031061,28,60.63795783,23,-110.88420715,0\
H,29,1.08650754,28,115.87321759,23,138.84610945,0\H,29,1.08737681,28,1
18.50748254,23,-3.54458779,0\H,30,1.08673175,29,119.0382834,28,-105.57
119426,0\H,30,1.08753101,29,117.5200278,28,108.47493516,0\H,28,1.09023
866,23,112.28244437,15,-60.64441755,0\\Version=AM64L-G03RevD.01\State=
1-A\HF=-956.9538678\MP2=-959.3555273\RMSD=8.299e-09\Thermal=0.\PG=C01
[X(C16H18P1)]\\@

267-BH$^+$

1\1\GINC-NODE19\SP\RMP2-FC\6-31+G(2d,p)\C28H26P1(1+)\ZIP07\27-Jul-2010
\0\\#p MP2(FC)/6-31+G(2d,p) scf=tight\\x3bhsp_68\\1,1\C\H,1,1.0986848\
C,1,4.34474026,2,105.23875595\C,3,1.39785847,1,58.96407547,2,12.815591
11,0\C,4,1.3973392,3,120.11183073,1,-0.46740352,0\C,5,1.40431864,4,120
.82918526,3,0.55599588,0\C,6,1.407284,5,118.62424148,4,-0.6234837,0\C,
3,1.39802615,1,60.66023771,6,-10.65634602,0\H,3,1.08687156,1,179.09654
819,6,-166.51186769,0\H,4,1.08701856,3,120.21826172,1,179.54688696,0\H
,5,1.08869616,4,119.46447597,3,179.75242975,0\H,7,1.08767433,6,120.472
40502,5,-179.73499678,0\H,8,1.08721104,3,120.12508462,1,-179.57446425,
0\C,1,4.35262396,6,117.85285957,5,-106.39363845,0\C,14,1.39848192,1,59
.27119063,6,150.94301272,0\C,15,1.39710641,14,120.11936504,1,-0.350832
77,0\C,16,1.40709351,15,120.80787022,14,0.5067422,0\C,17,1.40397266,16
,118.59244346,15,-1.15517861,0\C,14,1.39738871,1,60.22635812,6,-28.258
50795,0\H,14,1.08698199,1,179.47640092,6,135.53801666,0\H,15,1.0871465
1,14,120.24623936,1,-179.44503178,0\H,16,1.08762755,15,119.06585394,14
,-178.89938556,0\H,18,1.08497962,17,120.35154449,16,-177.71570352,0\H,
19,1.0871561,14,120.13563404,1,179.55060148,0\P,1,1.88884839,6,110.622
59486,5,123.06127679,0\C,25,4.62200521,1,111.05188915,6,-155.49616837,
0\C,26,1.39731165,25,59.95049466,1,-138.50610405,0\C,27,1.39788211,26,
120.25849541,25,1.24214971,0\C,28,1.40751602,27,120.02267096,26,0.0901
2634,0\C,29,1.41061881,28,119.39361534,27,-0.36867817,0\C,30,1.3956154
8,29,120.17388715,28,0.40445304,0\H,27,1.08669341,26,120.21596087,25,-
178.87784789,0\H,28,1.0861084,27,119.5932049,26,179.724427,0\H,30,1.08
749316,29,120.95711353,28,-179.73173264,0\H,31,1.08672965,30,119.67183
939,29,179.78086309,0\C,25,4.61157167,1,111.65559266,6,81.6591313,0\C,
36,1.39843746,25,60.09455315,1,-117.26475741,0\C,37,1.39659886,36,120.
10403435,25,-0.3232977,0\C,38,1.40835428,37,119.93676818,36,0.17569454
,0\C,39,1.41112362,38,119.78952309,37,-0.40858784,0\C,40,1.395915,39,1
19.69907679,38,0.28971689,0\H,36,1.0870086,25,179.82252115,1,-24.17593
803,0\H,37,1.08670659,36,120.2525482,25,179.95391233,0\H,38,1.08588778

,37,119.55652578,36,-179.45394832,0\H,40,1.08626608,39,120.69232961,38
,-179.35449827,0\H,41,1.08668996,40,119.50367428,39,-179.9440907,0\H,2
5,2.43251449,1,92.74246932,6,-18.57332534,0\H,26,1.08686258,25,179.047
39841,1,132.81273436,0\C,25,1.80755055,1,107.82448065,6,-38.3089184,0\
C,49,1.53121549,25,121.67845227,1,-160.34762446,0\C,50,1.50101013,49,6
0.66250542,25,111.32202253,0\H,50,1.08761728,49,118.09200566,25,3.8813
786,0\H,50,1.08695933,49,116.3472543,25,-138.47095179,0\H,51,1.0864919
6,50,117.39080718,49,-109.54885811,0\H,51,1.08654511,50,119.04626242,4
9,104.71720764,0\\Version=AM64L-G03RevD.01\State=1-A\HF=-1416.0666014\
MP2=-1420.1672887\RMSD=6.604e-09\Thermal=0.\PG=C01 [X(C28H26P1)]\\@

268

1\1\GINC-YANG\SP\RMP2-FC\6-31+G(2d,p)\C15H17P1\CHRISTOPH\03-Aug-2010\0
\\#p MP2(FC)/6-31+g(2d,p) scf=tight\\xiprsp_3\\0,1\P\C,1,4.67622161\C,
2,1.39829075,1,57.98035129\C,3,1.39746163,2,120.03620482,1,-0.35598719
,0\C,4,1.407939,3,121.16164524,2,0.91270399,0\C,5,1.40755527,4,118.043
50453,3,-0.95794942,0\C,2,1.39808638,1,61.62550763,5,2.62256024,0\H,2,
1.0878012,1,178.19468356,5,176.36200249,0\H,3,1.08792735,2,120.1408973
1,1,-179.72876917,0\H,4,1.08870879,3,119.62061437,2,-178.8488974,0\H,6
,1.08757232,5,120.23898636,4,-179.47652261,0\H,7,1.08816609,2,120.0681
3302,1,179.84994257,0\C,1,4.67665191,5,104.26997883,4,-95.68626572,0\C
,13,1.39921794,1,61.26921061,5,-29.4101254,0\C,14,1.39766707,13,120.26
576992,1,0.32107443,0\C,15,1.4066385,14,120.73601341,13,0.10951805,0\C
,16,1.40722669,15,118.3416343,14,0.6026757,0\C,13,1.39763964,1,58.4099
6897,5,149.86125475,0\H,13,1.08790486,1,178.58136822,5,159.39385486,0\
H,14,1.08822672,13,120.02021472,1,-179.78333972,0\H,15,1.08763659,14,1
19.35042817,13,179.22535313,0\H,17,1.08875408,16,119.32465859,15,178.6
9349246,0\H,18,1.08802946,13,120.1703749,1,179.81508897,0\C,1,1.890564
43,5,103.36207232,4,158.8436211,0\H,24,1.10023041,1,109.47826059,5,52.
00012879,0\C,24,1.54079353,1,109.74357605,5,-68.13889543,0\H,26,1.0960
2108,24,112.24828723,1,60.9052048,0\H,26,1.09864609,24,110.19372481,1,
-178.70758803,0\H,26,1.09802432,24,110.68417052,1,-59.6273435,0\C,24,1
.54048185,1,109.47860662,5,170.25699131,0\H,30,1.09575312,24,111.95597
501,1,-61.67415417,0\H,30,1.09854569,24,109.98507532,1,178.30916762,0\
H,30,1.09839701,24,111.00622429,1,59.23602191,0\\Version=AM64L-G03RevD
.01\State=1-A\HF=-918.7031093\MP2=-920.9853142\RMSD=7.067e-09\Thermal=
0.\PG=C01 [X(C15H17P1)]\\@

268-Me$^+$

1\1\GINC-YIN\SP\RMP2-FC\6-31+G(2d,p)\C16H20P1(1+)\CHRISTOPH\03-Aug-201
0\0\\#p MP2(FC)/6-31+g(2d,p) scf=tight\\xiprmesp_1\\1,1\C\C,1,1.398976
15\C,2,1.39601662,1,120.1545061\C,3,1.40901317,2,119.85779695,1,0.0548
0077,0\C,4,1.40840063,3,119.7726723,2,-0.4131196,0\C,5,1.39650971,4,11
9.87983788,3,0.52579364,0\H,1,1.08682976,6,119.88983641,5,179.93580899
,0\H,2,1.0866221,1,120.22180199,6,-179.92827895,0\H,3,1.08719512,2,119
.58570122,1,179.61726028,0\H,5,1.08695259,4,121.0982076,3,-179.6020379
3,0\H,6,1.08656016,5,119.62114867,4,179.63178458,0\C,3,5.36315699,2,14
2.81120765,1,135.87342536,0\C,12,1.39956667,3,46.85827481,2,-59.062424
91,0\C,13,1.39554594,12,120.14217621,3,-34.66800668,0\C,14,1.41035727,
13,119.88857426,12,0.00414759,0\C,15,1.40823265,14,119.71707339,13,-0.
02231471,0\C,16,1.39689559,15,119.90225291,14,0.09677129,0\H,12,1.0868
3653,3,149.31587918,2,17.01362803,0\H,13,1.0866371,12,120.22073613,3,1
45.07312838,0\H,14,1.08714097,13,119.6070808,12,178.99713348,0\H,16,1.
08660737,15,121.05915992,14,179.69593071,0\H,17,1.08656917,16,119.6030
024,15,179.64588586,0\C,15,3.00941503,14,108.48663595,13,-140.05034534
,0\H,23,1.10019808,15,139.087616,14,-114.35862797,0\C,23,1.54438677,15
,93.42867187,14,121.91935493,0\H,25,1.09640045,23,111.48228179,15,-30.
47548809,0\H,25,1.09534651,23,112.75890168,15,92.37334868,0\H,25,1.095

86284,23,108.55629551,15,-148.85116841,0\C,23,1.54291897,15,92.9667240
6,14,10.14759615,0\H,29,1.09592748,23,108.68177734,15,155.40609437,0\H
,29,1.09593562,23,111.09713892,15,37.11739922,0\H,29,1.09533875,23,112
.74344952,15,-85.1018277,0\P,15,1.80851241,14,119.50320123,13,-176.928
50975,0\C,33,1.82601737,15,109.56735417,14,-160.39026286,0\H,34,1.0937
4528,33,111.97237731,15,-61.04924403,0\H,34,1.09499564,33,109.29919744
,15,60.4221053,0\H,34,1.09577888,33,108.47426214,15,178.6983731,0\\Ver
sion=AM64L-G03RevD.01\State=1-A\HF=-958.1539443\MP2=-960.581779\RMSD=6
.627e-09\Thermal=0.\PG=C01 [X(C16H20P1)]\\@

268-BH$^+$

1\1\GINC-CALYPSO\SP\RMP2-FC\6-31+G(2d,p)\C28H28P1(1+)\CHRISTOPH\07-Aug
-2010\0\\#p MP2(FC)/6-31+g(2d,p) scf=tight\\xiprbhsp_2\\1,1\C\C,1,1.40
562331\C,2,1.40569286,1,118.56635846\C,3,1.39908422,2,120.56679161,1,-
0.25478795,0\C,4,1.39730102,3,120.30700138,2,0.26352476,0\C,1,1.396885
63,2,120.83546572,3,-0.01817661,0\H,1,1.08890573,6,119.45758377,5,179.
38680618,0\H,3,1.08737033,2,120.45314262,1,178.98888528,0\H,4,1.087192
68,3,119.55453307,2,179.8514567,0\H,5,1.08680149,4,120.19640729,3,179.
79454742,0\H,6,1.08699677,1,119.66287391,2,-179.65325923,0\C,2,1.52847
956,1,117.94567672,6,178.18501751,0\H,12,1.09778088,2,106.79675084,1,1
9.03269607,0\C,12,4.35062729,2,116.49029336,1,-99.42093313,0\C,14,1.39
783212,12,60.17541112,2,-34.27973319,0\C,15,1.39763966,14,120.39887094
,12,-0.05660876,0\C,16,1.40408503,15,120.47782723,14,0.1157392,0\C,17,
1.40635403,16,118.72032492,15,0.51046413,0\C,18,1.39737902,17,120.7287
8779,16,-0.74519732,0\H,14,1.08700688,12,179.47079603,2,106.94539063,0
\H,15,1.08712616,14,120.13263342,12,179.43263952,0\H,16,1.08538879,15,
119.27754913,14,178.81722362,0\H,18,1.08755022,17,120.07060819,16,178.
70232233,0\H,19,1.08711666,18,119.65154376,17,179.49447684,0\P,12,1.89
260512,2,112.70165735,1,130.73902903,0\C,25,4.62633243,12,110.65068088
,2,-154.9466227,0\C,26,1.39896452,25,60.12040274,12,37.34306762,0\C,27
,1.39617632,26,120.12223294,25,-1.72339661,0\C,28,1.40999922,27,120.23
41733,26,-0.32919998,0\C,29,1.40807384,28,119.25464802,27,0.97318016,0
\C,30,1.39725944,29,120.11777302,28,-0.98519158,0\H,26,1.08686213,25,1
78.62687306,12,131.18934089,0\H,27,1.08673267,26,120.25941773,25,178.2
7242831,0\H,28,1.0868035,27,118.74789284,26,179.70615867,0\H,30,1.0859
4712,29,120.45799533,28,179.27894093,0\H,31,1.08673023,30,119.50781506
,29,-179.64768982,0\C,25,4.61985414,12,109.45977122,2,83.65750457,0\C,
37,1.39847313,25,59.6320319,12,71.90869765,0\C,38,1.39632323,37,120.26
51341,25,0.60257665,0\C,39,1.41051508,38,119.9555828,37,0.27290372,0\C
,40,1.40924528,39,119.45069394,38,-0.60017076,0\C,41,1.39649717,40,120
.10475117,39,0.52097942,0\H,37,1.0869338,25,179.43306914,12,24.2924683
8,0\H,38,1.0866434,37,120.2573136,25,-179.19744203,0\H,39,1.08635522,3
8,119.51437023,37,-179.71145227,0\H,41,1.08616552,40,120.95698615,39,-
179.43457682,0\H,42,1.08667105,41,119.58633807,40,179.66041071,0\C,25,
1.86207133,12,110.51372832,2,-38.95722187,0\H,48,1.0982193,25,104.8791
7205,12,66.43639994,0\C,48,1.54348538,25,111.35523617,12,-50.4024917,0
\H,50,1.09600265,48,111.46179749,25,-63.46051594,0\H,50,1.09444757,48,
112.50923489,25,59.97490609,0\H,50,1.09621604,48,108.32161187,25,178.5
7444457,0\C,48,1.54460736,25,111.19099339,12,-174.53699,0\H,54,1.09641
225,48,108.51399998,25,-174.75883546,0\H,54,1.09567864,48,111.00279625
,25,66.93652098,0\H,54,1.09483632,48,113.21571845,25,-55.04513054,0\\V
ersion=AM64L-G03RevD.01\State=1-A\HF=-1417.2646993\MP2=-1421.3937999\R
MSD=7.076e-09\Thermal=0.\PG=C01 [X(C28H28P1)]\\@

268-TT$^+$

1\1\GINC-EDDY\SP\RMP2-FC\6-31+G(2d,p)\C34H32P1(1+)\CHRISTOPH\20-Aug-20
11\0\\#p MP2(FC)/6-31+g(2d,p) scf=tight\\xiprttsp_18\\1,1\P\C,1,4.6507
7384\C,2,1.3974227,1,59.84377959\C,3,1.39617324,2,120.25281019,1,0.190

55411,0\C,4,1.41016613,3,120.65192121,2,0.09702023,0\C,5,1.41200167,4,
118.61448036,3,-0.127096,0\C,6,1.39676302,5,120.30026327,4,0.14176472,
0\H,2,1.08696785,1,179.80510447,5,171.69114039,0\H,3,1.08685071,2,120.
34407725,1,-179.768105,0\H,4,1.08232016,3,118.38371376,2,-179.1727522,
0\H,6,1.08414835,5,120.81286803,4,179.94396319,0\H,7,1.08685875,6,119.
21548997,5,179.93911499,0\C,1,1.88529214,5,111.23652671,4,-9.65603314,
0\C,1,4.64532706,5,107.55553311,4,-118.43099169,0\C,14,1.39855665,1,59
.73970825,5,-179.3089573,0\C,15,1.39629513,14,120.43295591,1,1.2061247
2,0\C,16,1.41166523,15,120.09846507,14,0.49621615,0\C,17,1.40808711,16
,119.09504325,15,-1.30953199,0\C,14,1.39712906,1,60.02402332,5,-0.0582
6439,0\H,14,1.08702566,1,178.8583375,5,98.83007364,0\H,15,1.08690139,1
4,120.23428652,1,-178.56444759,0\H,16,1.08475302,15,118.83247767,14,-1
79.74567978,0\H,18,1.08467892,17,120.62498679,16,-178.20648718,0\H,19,
1.08693094,14,120.28101563,1,178.3630257,0\C,1,2.00743747,5,111.096646
71,4,118.25242442,0\C,6,4.21990119,5,125.35523581,4,-147.28618822,0\C,
26,1.3952803,6,74.84155627,5,62.26174508,0\C,27,1.39952075,26,120.6229
3174,6,-52.65088295,0\C,28,1.4060355,27,121.12625373,26,-0.00297688,0\
C,29,1.41301865,28,117.51994179,27,-0.99315403,0\C,30,1.39679644,29,12
1.27668139,28,1.22835706,0\H,26,1.08706188,6,129.54084826,5,179.531834
96,0\H,27,1.08716342,26,120.2051204,6,127.5424054,0\H,28,1.0835764,27,
118.57211251,26,-179.43089163,0\H,30,1.08431581,29,120.43995208,28,-17
8.1673675,0\H,31,1.08742242,30,119.35229957,29,-179.95140973,0\C,16,4.
32715065,15,110.17506501,14,158.02961475,0\C,37,1.39932258,16,70.67792
212,15,140.0434745,0\C,38,1.39564476,37,120.18406976,16,53.9324639,0\C
,39,1.41207132,38,121.62324792,37,-1.23353101,0\C,40,1.40505544,39,117
.23543061,38,3.3128613,0\C,37,1.39469714,16,71.97623407,15,-89.0921037
8,0\H,37,1.08693076,16,130.41988171,15,26.0636291,0\H,38,1.08734782,37
,120.29763778,16,-127.26570984,0\H,39,1.08452502,38,118.28791096,37,17
5.83306118,0\H,41,1.08313562,40,120.45207152,39,175.99741853,0\H,42,1.
08719391,37,120.19834551,16,127.56463027,0\C,25,4.38883486,1,111.18161
272,5,-66.88345709,0\C,48,1.39723244,25,59.93330008,1,42.33641938,0\C,
49,1.39721075,48,120.40546279,25,-0.65700661,0\C,50,1.40862755,49,121.
30038052,48,-1.28919958,0\C,51,1.40761721,50,117.49774746,49,2.8799461
1,0\C,48,1.39602665,25,59.11029749,1,-137.88712981,0\H,48,1.08694871,2
5,179.20109052,1,162.70350391,0\H,49,1.0873833,48,120.26948819,25,178.
53277985,0\H,50,1.08316034,49,118.1552377,48,176.89774885,0\H,52,1.084
20948,51,120.30901983,50,177.00083377,0\H,53,1.08717549,48,120.2562467
7,25,-178.55233685,0\H,13,1.09781525,1,100.3061083,5,-172.64003866,0\C
,13,1.54838104,1,111.73919132,5,-60.4638288,0\H,60,1.09687229,13,107.6
6107619,1,-168.80037643,0\H,60,1.09377455,13,112.3663197,1,-50.3187086
1,0\H,60,1.09358931,13,112.92763344,1,72.56620081,0\C,13,1.54327225,1,
117.69437864,5,70.97993169,0\H,64,1.09706267,13,107.80268243,1,170.788
34012,0\H,64,1.09407783,13,112.86432612,1,-70.88777118,0\H,64,1.092712
93,13,113.20961023,1,51.22848876,0\\Version=AM64L-G03RevD.01\State=1-A
\HF=-1646.7817064\MP2=-1651.7873449\RMSD=8.758e-09\Thermal=0.\PG=C01 [
X(C34H32P1)]\\@

269

1\1\GINC-SOLARIS\SP\RMP2-FC\6-31+G(2d,p)\C12H19P1\CHRISTOPH\23-Aug-201
0\0\\#p MP2(FC)/6-31+g(2d,p) scf=tight\\yprsp_1\\0,1\P\C,1,4.6784802\C
,2,1.40004489,1,61.049808\C,3,1.39701846,2,120.09512419,1,0.,0\C,4,1.4
0781413,3,120.94350867,2,0.00049039,0\C,5,1.40584297,4,118.1913049,3,-
0.00059247,0\C,2,1.39696465,1,58.68402787,5,179.99545962,0\H,2,1.08793
008,1,178.88158566,5,179.98203193,0\H,3,1.08823827,2,120.01614288,1,18
0.,0\H,4,1.08880268,3,119.03122246,2,180.,0\H,6,1.08850723,5,119.11864
465,4,179.99957767,0\H,7,1.08797812,2,120.14599516,1,-179.99946337,0\C
,1,1.87648256,5,101.29048844,4,50.8901232,0\H,13,1.10075099,1,110.9611
0847,5,-51.9628462,0\H,13,1.10083723,1,106.2454271,5,-167.50511937,0\C

,1,1.87647504,5,101.2900133,4,-50.85083069,0\H,16,1.10075017,1,110.960
81768,5,51.92211713,0\H,16,1.10083577,1,106.24795966,5,167.46614647,0\
C,16,1.53939028,1,113.06082596,5,-72.25498495,0\H,19,1.09927562,16,109
.2952732,1,-55.77062985,0\H,19,1.09904195,16,109.31211904,1,60.2819552
4,0\C,13,1.53938387,1,113.06347174,5,72.21683012,0\H,22,1.09904188,13,
109.31236088,1,-60.2978021,0\H,22,1.09927789,13,109.29599036,1,55.7549
5625,0\C,19,1.53640881,16,112.57119365,1,-177.83887624,0\H,25,1.098225
39,19,111.15840753,16,59.85552142,0\H,25,1.09737378,19,111.20069076,16
,179.94825119,0\H,25,1.09815605,19,111.13755582,16,-60.02065043,0\C,22
,1.53640931,13,112.57088263,1,177.82298058,0\H,29,1.09822609,22,111.15
86267,13,-59.86038185,0\H,29,1.0981559,22,111.13747451,13,60.01585851,
0\H,29,1.09737445,22,111.20062054,13,-179.95322294,0\\Version=AM64L-G0
3RevD.01\State=1-A\HF=-806.2629253\MP2=-808.1617154\RMSD=6.535e-09\The
rmal=0.\PG=C01 [X(C12H19P1)]\\@

269-Me$^+$

1\1\GINC-YANG\SP\RMP2-FC\6-31+G(2d,p)\C13H22P1(1+)\CHRISTOPH\24-Aug-20
10\0\\#p MP2(FC)/6-31+g(2d,p) scf=tight\\yprmesp_12\\1,1\C\C,1,1.39785
877\C,2,1.39732719,1,120.15342987\C,3,1.40649937,2,119.78226252,1,-0.0
0281595,0\C,4,1.41005307,3,119.86833409,2,0.,0\C,5,1.39469058,4,119.89
891697,3,0.00166615,0\H,1,1.08678073,2,119.88981391,3,-179.99680155,0\
H,2,1.08652073,1,120.23043155,6,-179.99926967,0\H,3,1.08651607,2,119.2
5936728,1,179.99160746,0\H,5,1.08856598,4,120.86787309,3,179.9965063,0
\H,6,1.08652612,5,119.7398289,4,179.99917651,0\C,4,2.9860977,3,132.479
46738,2,137.58036961,0\H,12,1.09979658,4,141.35013704,3,90.68627069,0\
H,12,1.09946399,4,89.54343824,3,-152.71379928,0\C,12,2.96936296,4,60.1
8271086,3,121.64968094,0\H,15,1.09978215,12,85.52149211,4,-161.4091801
9,0\H,15,1.09945541,12,86.09831671,4,91.56731223,0\C,15,1.54518355,12,
150.8574658,4,-34.80330096,0\H,18,1.09770599,15,109.49235149,12,56.692
01793,0\H,18,1.09818509,15,109.80451263,12,-60.54609809,0\C,12,1.54517
246,4,94.92086843,3,-42.33596383,0\H,21,1.09770004,12,109.4931745,4,-2
7.0953586,0\H,21,1.09819569,12,109.81019865,4,90.14267215,0\C,18,1.536
67147,15,111.16277102,12,178.23227089,0\H,24,1.09679272,18,111.3663694
8,15,60.13620016,0\H,24,1.09683797,18,111.24863542,15,-60.54431362,0\C
,21,1.53667336,12,111.15639727,4,-148.63468974,0\H,27,1.09523766,21,11
0.28887741,12,-179.89595224,0\H,27,1.09680007,21,111.36792461,12,-60.1
9222928,0\P,4,1.80517933,3,121.51555197,2,179.96803401,0\C,30,1.819715
1,4,110.39942655,3,-0.00533974,0\H,31,1.09606263,30,108.76917386,4,179
.98651421,0\H,31,1.09453872,30,110.71479673,4,-60.64952576,0\H,31,1.09
452988,30,110.72102742,4,60.62173231,0\H,24,1.09523998,18,110.28816081
,15,179.83827006,0\H,27,1.09683991,21,111.24772202,12,60.48745439,0\\V
ersion=AM64L-G03RevD.01\State=1-A\HF=-845.7145941\MP2=-847.7582685\RMS
D=4.653e-09\Thermal=0.\PG=C01 [X(C13H22P1)]\\@

269-BH$^+$

1\1\GINC-NODE20\SP\RMP2-FC\6-31+G(2d,p)\C25H30P1(1+)\ZIP07\23-Feb-2011
\0\\#p MP2(FC)/6-31+G(2d,p) scf=tight\\yprbhsp_2\\1,1\C\H,1,1.09998957
\C,1,4.34468891,2,105.44636283\C,3,1.39808339,1,58.90950206,2,16.12160
805,0\C,4,1.39713615,3,120.09944956,1,-0.5516472,0\C,5,1.40533407,4,12
0.80534874,3,0.40680484,0\C,6,1.40645304,5,118.6501987,4,-0.35211248,0
\C,3,1.39756819,1,60.72363756,6,-17.49476619,0\H,3,1.08684642,1,179.00
725581,6,-171.4942027,0\H,4,1.08699131,3,120.22916024,1,179.38198732,0
\H,5,1.08886798,4,119.46052272,3,179.33808195,0\H,7,1.08727996,6,120.5
7891903,5,179.88421943,0\H,8,1.08718477,3,120.12277964,1,-179.425008,0
\C,1,4.34792133,6,117.69440614,5,-101.63414887,0\C,14,1.39812629,1,60.
32475917,6,-38.95477996,0\C,15,1.39804933,14,120.43973522,1,-0.0493765
6,0\C,16,1.4039775,15,120.38608455,14,0.10507381,0\C,17,1.40662686,16,
118.74921579,15,0.60541547,0\C,18,1.39738791,17,120.80452947,16,-0.837

79325,0\H,14,1.08689753,1,179.37396719,6,109.2140517,0\H,15,1.08710808
,14,120.0970314,1,179.38044799,0\H,16,1.08553241,15,119.34520348,14,17
8.58802578,0\H,18,1.08926128,17,119.91531598,16,177.76341667,0\H,19,1.
08708168,18,119.73908748,17,179.35187888,0\P,1,1.87963849,6,112.208680
36,5,126.83486088,0\C,25,4.61386079,1,114.07395508,6,78.23322702,0\C,2
6,1.39894441,25,59.75128288,1,69.27201217,0\C,27,1.39594579,26,120.217
66882,25,-0.31937837,0\C,28,1.41050689,27,119.9009414,26,-0.08178163,0
\C,29,1.40874524,28,119.59631838,27,0.13307407,0\C,30,1.39652738,29,12
0.00102008,28,-0.0992539,0\H,26,1.08690338,25,179.46423318,1,123.36143
168,0\H,27,1.08664136,26,120.22316006,25,179.58675938,0\H,28,1.0864987
2,27,119.25336949,26,-179.61943273,0\H,30,1.08624963,29,120.91752107,2
8,179.92285072,0\H,31,1.08664999,30,119.60611072,29,179.78914577,0\C,2
5,1.83510036,1,108.67869972,6,-44.91452198,0\H,37,1.09737476,25,106.97
239093,1,61.50426962,0\H,37,1.09838845,25,107.17867365,1,176.62641928,
0\C,25,1.83644295,1,107.51954608,6,-162.83792671,0\H,40,1.09793807,25,
106.96602498,1,-60.66025125,0\H,40,1.09885484,25,107.14196886,1,53.140
35581,0\C,40,1.54476218,25,114.51196,1,176.26383478,0\H,43,1.09843788,
40,109.7976389,25,-60.4776393,0\H,43,1.09756378,40,109.57116574,25,56.
6580524,0\C,37,1.54591416,25,114.55527967,1,-60.47115235,0\H,46,1.0983
7195,37,110.09627565,25,-59.05200024,0\H,46,1.09768339,37,109.71431911
,25,58.81996972,0\C,43,1.53668338,40,111.18640097,25,178.20879878,0\H,
49,1.095416,43,110.37982573,40,179.93597611,0\H,49,1.09687367,43,111.2
1770874,40,-60.40920437,0\H,49,1.09698883,43,111.37673797,40,60.185513
46,0\C,46,1.5368562,37,110.94451039,25,179.66166317,0\H,53,1.09540268,
46,110.39783609,37,179.73991966,0\H,53,1.09665177,46,111.13425787,37,-
60.59125652,0\H,53,1.09693954,46,111.2834367,37,60.00626663,0\\Version
=AM64L-G03RevD.01\State=1-A\HF=-1304.8315231\MP2=-1308.5699559\RMSD=8.
615e-09\Thermal=0.\PG=C01 [X(C25H30P1)]\\@

270

1\1\GINC-NODE8\SP\RMP2-FC\6-31+G(2d,p)\C15H17P1\ZIP07\07-Apr-2010\0\\#
p MP2(FC)/6-31+G(2d,p) scf=tight\\xprsp_43\\0,1\P\C,1,4.67269616\C,2,1
.39724394,1,58.74675566\C,3,1.39945136,2,119.97853447,1,0.93658808,0\C
,4,1.40585845,3,120.85431947,2,-0.61203819,0\C,5,1.40723366,4,118.5402
7672,3,0.75531508,0\C,6,1.3970041,5,120.66556239,4,-0.37754786,0\H,2,1
.08791491,1,178.82039638,5,-176.81668338,0\H,3,1.08800033,2,120.147545
97,1,-179.5611569,0\H,4,1.08868685,3,119.77975379,2,179.10177833,0\H,6
,1.08821624,5,119.67789685,4,178.9571384,0\H,7,1.08819952,6,119.791475
63,5,179.79672839,0\C,1,4.67333185,5,102.104198,4,-144.81238897,0\C,13
,1.39646492,1,61.26080406,5,-107.60436246,0\C,14,1.40037245,13,120.339
80573,1,1.22419388,0\C,15,1.40580058,14,120.81514016,13,-0.30220174,0\
C,16,1.40973581,15,118.11460564,14,-0.37459639,0\C,17,1.39536562,16,12
1.10299776,15,0.99083642,0\H,13,1.08773932,1,178.19584785,16,-177.7699
4275,0\H,14,1.08818854,13,120.08949966,1,-178.98120389,0\H,15,1.086811
13,14,118.80709315,13,179.69556039,0\H,17,1.08903261,16,119.45980195,1
5,-179.10458146,0\H,18,1.08800803,17,119.76976836,16,179.56860418,0\C,
1,1.87546647,16,102.48678315,15,-5.52047588,0\H,24,1.1000791,1,110.787
7709,16,-53.60833093,0\H,24,1.10002014,1,107.05529825,16,63.29423113,0
\C,24,1.54030249,1,112.06605436,16,-176.8302135,0\H,27,1.09870515,24,1
09.44314658,1,59.8361394,0\H,27,1.09922924,24,109.34417185,1,-56.43429
531,0\C,27,1.53635683,24,112.32680257,1,-178.39339078,0\H,30,1.0979795
2,27,111.11528929,24,-60.04646174,0\H,30,1.09811534,27,111.20157884,24
,59.92007932,0\H,30,1.09725749,27,111.10643176,24,179.97146316,0\\Vers
ion=AM64L-G03RevD.01\State=1-A\HF=-918.7066188\MP2=-920.983004\RMSD=2.
587e-09\Thermal=0.\PG=C01 [X(C15H17P1)]\\@

270-Me$^+$

1\1\GINC-MORITZ\SP\RMP2-FC\6-31+G(2d,p)\C16H20P1(1+)\CHRISTOPH\08-Apr-

2010\0\\#p MP2(FC)/6-31+g(2d,p) scf=tight\\xprmesp_41\\1,1\C\C,1,1.399
72256\C,2,1.39543597,1,120.13283279\C,3,1.41043752,2,119.81779115,1,-0
.02477674,0\C,4,1.40779702,3,119.82144281,2,0.05104803,0\C,5,1.396852,
4,119.84937857,3,0.01111744,0\H,1,1.08684179,6,119.87949503,5,179.8976
446,0\H,2,1.08660216,1,120.21135836,6,179.68271159,0\H,3,1.08732264,2,
119.59205995,1,179.0697434,0\H,5,1.08658704,4,121.05450467,3,179.67594
904,0\H,6,1.08652439,5,119.61353947,4,179.69737118,0\C,3,5.48002099,2,
141.308535,1,121.96546635,0\C,12,1.39907294,3,44.42652771,2,-46.104969
16,0\C,13,1.39602135,12,120.15236004,3,-32.17647977,0\C,14,1.40873007,
13,119.77638604,12,-0.0036191,0\C,15,1.40839606,14,119.88752508,13,-0.
2413396,0\C,16,1.39627809,15,119.83588526,14,0.34371749,0\H,12,1.08683
101,3,150.27074126,2,23.35248512,0\H,13,1.08659354,12,120.20592033,3,1
47.65791693,0\H,14,1.08721921,13,119.66308956,12,179.47822237,0\H,16,1
.08703399,15,121.00395409,14,-179.80974922,0\H,17,1.0865502,16,119.653
14112,15,179.64019311,0\C,15,2.9529098,14,111.09063298,13,-137.3012017
8,0\H,23,1.09554775,15,86.5782705,14,119.27044593,0\H,23,1.09524842,15
,146.27772048,14,-121.21464536,0\H,23,1.09493444,15,92.34310605,14,10.
69740452,0\P,4,1.80656244,3,119.54354066,2,-177.28201701,0\C,27,1.8359
7862,4,110.70145441,3,78.52023346,0\H,28,1.09955126,27,105.33896004,4,
62.87264369,0\H,28,1.09945876,27,107.26185581,4,176.00480773,0\C,28,1.
54345797,27,115.76452621,4,-59.38784458,0\H,31,1.09727857,28,109.27527
251,27,59.1623805,0\H,31,1.09792366,28,109.82950515,27,-57.9220029,0\C
,31,1.5368134,28,111.16414699,27,-179.65863234,0\H,34,1.09531388,31,11
0.29208887,28,-179.51087144,0\H,34,1.09682431,31,111.30048929,28,-59.8
9819354,0\H,34,1.0970119,31,111.43397315,28,60.79505294,0\\Version=IA3
2L-G03RevD.01\State=1-A\HF=-958.1573577\MP2=-960.5791373\RMSD=5.225e-0
9\Thermal=0.\PG=C01 [X(C16H20P1)]\\@

270-BH$^+$

1\1\GINC-NAUTILUS\SP\RMP2-FC\6-31+G(2d,p)\C28H28P1(1+)\CHRISTOPH\09-Ju
l-2010\0\\#p MP2(FC)/6-31+g(2d,p) scf=tight\\xprbhsp_2\\1,1\C\H,1,1.09
919429\P,1,1.88816467,2,101.44888457\C,1,4.34922305,3,111.50270433,2,1
12.4848703,0\C,4,1.39854859,1,59.26806964,3,-80.10388319,0\C,5,1.39718
378,4,120.0855852,1,-0.32414713,0\C,6,1.40700718,5,120.73631536,4,0.43
253358,0\C,7,1.40382651,6,118.73096626,5,-0.96307228,0\C,4,1.39761208,
1,60.29986056,7,-7.8472383,0\H,4,1.08702522,1,179.42062421,7,149.25818
894,0\H,5,1.08716413,4,120.2521204,1,-179.41387117,0\H,6,1.08784212,5,
119.27408963,4,-179.0113867,0\H,8,1.08508134,7,120.29660384,6,-177.725
76775,0\H,9,1.08716433,4,120.11546049,1,179.49839471,0\C,1,4.34410048,
7,117.29362989,6,147.67134338,0\C,15,1.39782075,1,58.95256123,7,-105.0
5075234,0\C,16,1.39729231,15,120.10773682,1,-0.4067663,0\C,17,1.404630
08,16,120.81084663,15,0.48125231,0\C,18,1.40700109,17,118.63642338,16,
-0.48799198,0\C,15,1.39795894,1,60.68732081,18,-9.14521735,0\H,15,1.08
685829,1,179.09410009,18,-169.0109644,0\H,16,1.08699535,15,120.2126666
1,1,179.60100851,0\H,17,1.08876556,16,119.4495646,15,179.57171862,0\H,
19,1.08765963,18,120.44952106,17,-179.99480735,0\H,20,1.08721684,15,12
0.11984066,1,-179.57824335,0\C,3,4.6205794,1,109.66613859,18,-154.1942
4754,0\C,26,1.4000438,3,59.54685844,1,47.85316729,0\C,27,1.39508723,26
,120.04229221,3,-1.26536169,0\C,28,1.41042891,27,120.16344941,26,-0.28
118494,0\C,29,1.4058791,28,119.49009334,27,1.06661715,0\C,26,1.3970489
2,3,60.50949239,1,-131.3272482,0\H,26,1.08687484,3,178.84431682,1,110.
43274238,0\H,27,1.08674692,26,120.26022544,3,178.59789941,0\H,28,1.088
29173,27,118.84631165,26,179.33785507,0\H,30,1.08578618,29,120.5333124
3,28,178.76326558,0\H,31,1.08672717,26,120.19984656,3,-178.65404453,0\
C,3,4.61238368,1,111.13076038,18,81.77870016,0\C,37,1.39884993,3,59.85
660968,1,66.72336905,0\C,38,1.39617895,37,120.27003176,3,0.4874373,0\C
,39,1.41126118,38,119.83818509,37,0.16051957,0\C,40,1.40888503,39,119.
59523944,38,-0.34930083,0\C,41,1.39635067,40,120.02883726,39,0.3251148

2,0\H,37,1.08695331,3,179.58657713,1,4.83275597,0\H,38,1.08665529,37,1
20.24316384,3,-179.3611662,0\H,39,1.08631993,38,119.54830561,37,-179.8
04413,0\H,41,1.08593377,40,120.89293433,39,-179.49510141,0\H,42,1.0866
7175,41,119.60907221,40,179.77467408,0\C,3,1.83890666,1,107.37306311,1
8,-40.37777213,0\H,48,1.09722896,3,107.57251158,1,68.17756709,0\H,48,1
.10002977,3,105.25045525,1,-44.17915541,0\C,48,1.54473591,3,115.594506
22,1,-165.9826677,0\H,51,1.09776202,48,110.24070108,3,-57.74789298,0\H
,51,1.09694167,48,109.38377326,3,59.07720452,0\C,51,1.53680242,48,110.
98013698,3,-179.54282081,0\H,54,1.09551737,51,110.368005,48,-179.39173
945,0\H,54,1.09704723,51,111.26496422,48,-59.73799188,0\H,54,1.0971461
1,51,111.46387244,48,60.78383512,0\\Version=AM64L-G03RevD.01\State=1-A
\HF=-1417.2716564\MP2=-1421.3912372\RMSD=9.965e-09\Thermal=0.\PG=C01 [
X(C28H28P1)]\\@

270-TT$^+$

1\1\GINC-CIPCLU09\SP\RMP2-FC\6-31+G(2d,p)\C34H32P1(1+)\LICCH\25-Aug-20
11\0\\#p MP2(FC)/6-31+G(2d,p) scf=tight\\xprttsp_19\\1,1\P\C,1,4.63306
742\C,2,1.39900063,1,59.68670311\C,3,1.39608728,2,120.37343324,1,-1.11
714713,0\C,4,1.41157735,3,119.95068912,2,-0.41868768,0\C,5,1.40749441,
4,119.36348066,3,1.28014185,0\C,6,1.39720422,5,120.13238825,4,-1.23461
773,0\H,2,1.08699914,1,178.9097314,5,177.8039105,0\H,3,1.08681685,2,12
0.23519069,1,178.63066484,0\H,4,1.08540985,3,119.1074941,2,179.5148605
2,0\H,6,1.08484485,5,120.61696182,4,178.26113044,0\H,7,1.08684113,6,11
9.44915871,5,179.97652102,0\C,1,1.84753314,5,104.82049803,4,-57.461211
35,0\C,1,4.64776524,5,108.93063531,4,-172.21073431,0\C,14,1.39782039,1
,59.55429541,5,117.13777316,0\C,15,1.39598726,14,120.21468398,1,0.0017
5831,0\C,16,1.411301,15,120.55263622,14,-0.0276037,0\C,17,1.4106749,16
,118.76702914,15,0.30628232,0\C,18,1.39711438,17,120.21108712,16,-0.40
699237,0\H,14,1.08689403,1,179.66297351,5,110.85002047,0\H,15,1.086772
99,14,120.33195363,1,-179.9621058,0\H,16,1.0849744,15,118.38086021,14,
179.22739849,0\H,18,1.08426407,17,120.85341935,16,179.69111152,0\H,19,
1.08678223,18,119.22143145,17,179.9885203,0\C,1,1.97590001,5,111.32945
993,4,63.26350618,0\C,18,4.1483441,17,125.16206238,16,148.73973856,0\C
,26,1.39834894,18,69.71280524,17,67.13095241,0\C,27,1.39763721,26,120.
34869713,18,-56.40194318,0\C,28,1.41267912,27,121.18372153,26,-0.10377
565,0\C,29,1.40618442,28,117.63498702,27,0.28646003,0\C,26,1.39591592,
18,73.4750214,17,-63.50481259,0\H,26,1.08702788,18,128.78470952,17,-17
9.62609373,0\H,27,1.08739431,26,120.292108,18,123.84846408,0\H,28,1.08
462844,27,118.2774149,26,179.60909369,0\H,30,1.08400479,29,120.1963929
6,28,179.73916453,0\H,31,1.08713122,26,120.19783081,18,-125.52682848,0
\C,25,4.38410042,1,113.67666379,5,-170.29539894,0\C,37,1.39832007,25,5
8.36338852,1,155.19320235,0\C,38,1.39581292,37,120.43229151,25,-1.4216
0573,0\C,39,1.4097122,38,121.14103335,37,0.21232287,0\C,40,1.40359685,
39,117.69049179,38,1.38172343,0\C,37,1.39488875,25,60.75166099,1,-24.5
0308099,0\H,37,1.08687728,25,178.59285329,1,-172.84792276,0\H,38,1.087
14213,37,120.24495024,25,178.63951291,0\H,39,1.08564616,38,118.9688032
8,37,179.82226179,0\H,41,1.08349909,40,121.02365122,39,175.79968037,0\
H,42,1.08727795,37,120.2787538,25,-178.59677321,0\C,4,4.28163922,3,108
.93668515,2,-153.04683758,0\C,48,1.39473544,4,68.80369328,3,85.5545141
5,0\C,49,1.40065965,48,120.69081302,4,54.85136196,0\C,50,1.40440468,49
,121.09655693,48,-0.25156103,0\C,51,1.41295407,50,117.36384149,49,3.00
223817,0\C,52,1.3954993,51,121.50299992,50,-3.615843,0\H,48,1.08694089
,4,130.06996069,3,-27.13405596,0\H,49,1.08716124,48,120.19902017,4,-12
5.70310529,0\H,50,1.08274982,49,118.28076622,48,178.11974411,0\H,52,1.
08592356,51,119.88665518,50,172.90683561,0\H,53,1.08729195,52,119.5038
4204,51,179.9530057,0\H,13,1.09726481,1,107.89085376,5,-172.04483944,0
\C,13,1.54844516,1,115.34911739,5,-45.98001672,0\H,60,1.09635765,13,11
1.08607215,1,-52.9461585,0\H,60,1.09608225,13,108.95820914,1,63.881008

42,0\C,60,1.53701801,13,110.67350928,1,-175.24287784,0\H,63,1.09714413
,60,111.3382467,13,-58.53904533,0\H,63,1.09739836,60,111.55903558,13,6
2.03664391,0\H,63,1.09563501,60,110.31791705,13,-178.16044089,0\H,13,1
.09446407,1,105.89500584,5,74.3374198,0\\Version=AM64L-G03RevD.01\Stat
e=1-A\HF=-1646.7945445\MP2=-1651.7903556\RMSD=8.875e-09\Thermal=0.\PG=
C01 [X(C34H32P1)]\\@

271

1\1\GINC-STEAK\SP\RMP2-FC\6-31+G(2d,p)\C18H15O1P1\CHRISTOPH\08-Feb-201
2\0\\#p MP2(FC)/6-31+g(2d,p) scf=tight\\oh1sp_9\\0,1\P\C,1,4.67023696\
C,2,1.39929676,1,58.37074707\C,3,1.39662496,2,120.04232249,1,-0.094263
79,0\C,4,1.40855846,3,120.88827598,2,-1.02878355,0\C,5,1.40515807,4,11
8.49035337,3,1.17543083,0\C,2,1.39768199,1,61.27588553,5,-18.53162193,
0\H,2,1.08784488,1,178.39092405,5,-175.02367486,0\H,3,1.08799079,2,120
.14628693,1,179.22549922,0\H,4,1.08905894,3,119.58230474,2,178.8385023
9,0\H,6,1.08705537,5,119.75629235,4,179.21461385,0\H,7,1.08819715,2,12
0.04221823,1,-179.34871588,0\C,1,4.67085464,5,104.50123197,4,83.565215
28,0\C,13,1.39793456,1,61.34818671,5,13.63873948,0\C,14,1.39936935,13,
120.31181831,1,0.39581842,0\C,15,1.40599243,14,120.58608896,13,-0.2291
5747,0\C,16,1.40803815,15,118.49435256,14,-0.45564571,0\C,17,1.3967699
7,16,120.90504399,15,1.02541282,0\H,13,1.08786584,1,178.42827592,5,-15
1.87068406,0\H,14,1.0882088,13,120.05314948,1,-179.65939615,0\H,15,1.0
8732498,14,119.58539578,13,-179.91158209,0\H,17,1.08892642,16,119.4813
2238,15,-178.75669831,0\H,18,1.08800847,17,119.81898503,16,179.7744337
3,0\C,1,4.66789189,5,103.6217619,4,-167.46223919,0\C,24,1.40204113,1,6
1.28775636,5,-83.97634202,0\C,25,1.39656435,24,119.9816368,1,0.2451067
9,0\C,26,1.40563121,25,121.14040281,24,-0.18027634,0\C,27,1.40930397,2
6,117.85050422,25,-0.46523306,0\C,28,1.39316773,27,121.6285285,26,0.97
71333,0\H,25,1.09015476,24,119.98238161,1,-179.65020654,0\H,26,1.08744
391,25,118.97444777,24,-179.7777329,0\H,28,1.08864882,27,119.35573416,
26,-178.79854954,0\H,29,1.08653328,28,121.40640743,27,179.89943429,0\O
,24,1.36273608,1,175.99946973,27,177.88396678,0\H,34,0.96802645,24,108
.99468989,1,176.50341803,0\\Version=AM64L-G03RevD.01\State=1-A\HF=-110
6.018645\MP2=-1108.8774133\RMSD=3.537e-09\Thermal=0.\PG=C01 [X(C18H15O
1P1)]\\@

271-Me$^+$

1\1\GINC-STEAK\SP\RMP2-FC\6-31+G(2d,p)\C19H18O1P1(1+)\CHRISTOPH\08-Feb
-2012\0\\#p MP2(FC)/6-31+g(2d,p) scf=tight\\oh1mesp_2\\1,1\P\C,1,1.826
9277\H,2,1.09488095,1,109.71457537\H,2,1.09459299,1,109.95239726,3,119
.76974487,0\H,2,1.09474851,1,110.13143389,3,-119.92477845,0\C,1,4.6070
7271,2,108.10474133,4,178.01156555,0\C,6,1.39769105,1,60.27821265,2,-1
31.1975796,0\C,7,1.39770464,6,120.14619942,1,0.18379605,0\C,8,1.406315
75,7,119.7247273,6,0.05336446,0\C,9,1.40996477,8,119.99758359,7,-0.263
56416,0\C,10,1.39475079,9,119.77529639,8,0.26078606,0\H,6,1.08690129,1
,179.79995083,9,139.61262351,0\H,7,1.0866211,6,120.22508234,1,-179.933
12609,0\H,8,1.08654486,7,119.91163719,6,179.78269751,0\H,10,1.08776513
,9,120.91576715,8,-179.22945461,0\H,11,1.08664087,10,119.7121344,9,179
.86115468,0\C,1,4.60647548,9,110.5852811,8,-12.61691008,0\C,17,1.40545
011,1,60.15979857,9,110.3050673,0\C,18,1.3923985,17,119.97634881,1,0.1
6111056,0\C,19,1.4077302,18,120.48182409,17,0.08484705,0\C,20,1.414668
89,19,119.07206616,18,-0.28196356,0\C,21,1.38630618,20,120.63152436,19
,0.23868378,0\H,18,1.08827095,17,120.1882526,1,-179.99264696,0\H,19,1.
08658874,18,119.06484161,17,179.82497954,0\H,21,1.08782159,20,120.7532
0231,19,-179.30465551,0\H,22,1.08580279,21,121.28071846,20,179.8279198
1,0\C,1,4.6064074,20,109.8287328,19,-12.56488501,0\C,27,1.39796043,1,6
0.23647249,20,105.78139674,0\C,28,1.39739962,27,120.15287898,1,0.44899
031,0\C,29,1.40679588,28,119.72130559,27,-0.04504857,0\C,30,1.4095347,

29,119.9931762,28,-0.22713573,0\C,31,1.39514619,30,119.77987409,29,0.3
2096443,0\H,27,1.08691627,1,179.78244189,20,-26.50785703,0\H,28,1.0866
3291,27,120.22534565,1,-179.77058462,0\H,29,1.08661438,28,119.83302112
,27,179.62821792,0\H,31,1.08754816,30,120.90601589,29,-179.34979697,0\
H,32,1.08665255,31,119.7011066,30,179.72134094,0\O,17,1.34453041,1,176
.53307843,20,142.32922703,0\H,38,0.96944324,17,110.78981237,1,179.5830
8822,0\\Version=AM64L-G03RevD.01\State=1-A\HF=-1145.4722902\MP2=-1148.
4740667\RMSD=4.710e-09\Thermal=0.\PG=C01 [X(C19H18O1P1)]\\@

272

1\1\GINC-GOLEM\SP\RMP2-FC\6-31+G(2d,p)\C26H23N2O1P1\CHRISTOPH\18-Nov-2
008\0\\#p MP2(FC)/6-31+g(2d,p) scf=tight\\tolospc012\\0,1\C\C,1,1.3945
7785\C,2,1.40490955,1,120.4396284\C,3,1.40376285,2,118.77395479,1,0.38
142377,0\C,4,1.39682915,3,120.09420598,2,-1.88411128,0\C,5,1.40264491,
4,121.67831076,3,2.00036372,0\H,1,1.08904924,2,119.0910264,3,-179.7834
4946,0\H,2,1.08820089,1,120.16455577,6,-178.06619582,0\H,4,1.0854573,3
,120.22446162,2,177.8424255,0\H,5,1.0883561,4,118.57027192,3,-178.7119
4767,0\C,6,1.51422692,5,121.13223853,4,177.95878656,0\H,11,1.09941609,
6,111.28579487,5,-86.86440214,0\H,11,1.09648651,6,111.31445363,5,32.76
308308,0\H,11,1.09650665,6,111.44259085,5,153.4234094,0\N,3,1.41323366
,2,118.56967795,1,177.7155961,0\H,15,1.01175181,3,116.94983501,2,-36.2
0817932,0\C,15,1.39111547,3,132.59828191,2,147.42730243,0\O,17,1.22331
641,15,119.49493848,3,167.0434227,0\N,17,1.38923215,15,115.3333003,3,-
14.08043902,0\H,19,1.01312313,17,115.76997738,15,-16.96024141,0\C,19,1
.40803482,17,126.42855055,15,-179.93692263,0\C,21,1.42610191,19,119.82
5013,17,163.37621882,0\C,21,1.40941703,19,120.97576403,17,-18.9251625,
0\C,22,1.40972536,21,118.25961106,19,178.03945812,0\C,23,1.39397525,21
,120.67458467,19,-177.74302528,0\H,23,1.0815828,21,118.85758205,19,3.1
232477,0\C,24,1.39373477,22,122.35373909,21,-0.52510523,0\H,24,1.08817
61,22,118.0189292,21,179.17170708,0\H,25,1.08799144,23,118.73961468,21
,-179.95263596,0\H,27,1.08697191,24,120.43600931,22,-179.98711434,0\P,
22,1.84788166,21,129.03002066,19,-2.53655717,0\C,31,1.84957149,22,104.
66381739,21,68.70241967,0\C,32,1.4065439,31,123.13993044,22,7.84116044
,0\C,32,1.41022425,31,118.11598984,22,179.46956825,0\C,33,1.39952766,3
2,120.74064178,31,172.99156445,0\H,33,1.08723196,32,119.82755449,31,-6
.23608934,0\C,34,1.39663678,32,120.97113287,31,-174.2572246,0\H,34,1.0
8888435,32,119.77072213,31,5.07436017,0\C,35,1.39814865,33,120.3843020
5,32,0.11591783,0\H,35,1.08813407,33,119.53267611,32,-179.64540458,0\H
,37,1.08800454,34,119.67622252,32,-179.59221647,0\H,39,1.08780069,35,1
20.26064269,33,-179.73045419,0\C,31,1.85704634,22,104.30387155,21,-41.
61325298,0\C,43,1.41052743,31,116.24235334,22,-61.48237239,0\C,43,1.40
527366,31,125.08425668,22,124.21296011,0\C,44,1.39481829,43,120.847169
15,31,-175.50970852,0\H,44,1.08871495,43,119.68400294,31,4.8401491,0\C
,45,1.4007195,43,120.56941533,31,174.49841489,0\H,45,1.0866501,43,120.
33779724,31,-6.50560189,0\C,48,1.39593496,45,120.43164881,43,0.1541899
3,0\H,46,1.08787679,44,119.64940038,43,-179.43318583,0\H,48,1.08749524
,45,119.24248927,43,179.29905249,0\H,50,1.08770031,48,120.30185729,45,
179.88903461,0\\Version=AM64L-G03RevD.01\State=1-A\HF=-1522.5839753\MP
2=-1526.9513987\RMSD=4.390e-09\Thermal=0.\PG=C01 [X(C26H23N2O1P1)]\\@

272-Me$^+$

1\1\GINC-EDDY\SP\RMP2-FC\6-31+G(2d,p)\C27H26N2O1P1(1+)\CHRISTOPH\06-No
v-2008\0\\#p MP2(FC)/6-31+g(2d,p) scf=tight\\tolomespc19\\1,1\C\C,1,1.
39834359\C,2,1.40310805,1,120.01229179\C,3,1.40428653,2,119.12087682,1
,-1.53332006,0\C,4,1.39459253,3,120.18819974,2,-0.1389921,0\C,1,1.4021
7253,2,121.49560995,3,2.10350826,0\H,1,1.08819084,2,118.97366704,3,179
.7973478,0\H,2,1.08753895,1,119.94962355,6,-175.50133132,0\H,4,1.08801
499,3,119.53175953,2,-179.56010915,0\H,5,1.08873825,4,119.09899774,3,-

178.75598746,0\C,6,1.51368674,1,121.35981292,2,177.43816563,0\H,11,1.0
9814903,6,110.95504081,1,-107.46357689,0\H,11,1.09532232,6,111.466836,
1,12.31824672,0\H,11,1.09740333,6,111.55113102,1,133.33772226,0\N,3,1.
42223811,2,121.62440243,1,-178.65576296,0\H,15,1.012737,3,117.91970103
,2,132.43382564,0\C,15,1.37660936,3,130.0428124,2,-50.43444207,0\O,17,
1.222159,15,122.27633168,3,175.26461083,0\N,17,1.39958771,15,115.70723
012,3,-7.54916654,0\H,19,1.01417202,17,118.63068979,15,0.46982494,0\C,
19,1.4157315,17,121.16474005,15,165.68622658,0\C,21,1.41684919,19,120.
09674972,17,131.95358447,0\C,21,1.40130341,19,120.83616024,17,-47.4979
8426,0\C,22,1.40952476,21,119.77124204,19,177.66918538,0\C,23,1.394914
38,21,120.49944032,19,-178.93686764,0\H,23,1.08491579,21,118.95644953,
19,1.50056854,0\C,24,1.39352375,22,120.36883831,21,1.94662961,0\H,24,1
.08552328,22,120.64836998,21,-179.12481107,0\H,25,1.08679326,23,119.40
284802,21,179.82372931,0\H,27,1.08620178,24,119.85402739,22,179.556197
15,0\C,22,2.99063205,21,99.11303441,19,23.93672593,0\C,31,1.40592828,2
2,132.90441964,21,-46.98001017,0\C,31,1.40942322,22,97.72545179,21,97.
63841908,0\C,32,1.39746699,31,119.65880695,22,138.9531397,0\H,32,1.086
43244,31,120.43900877,22,-40.51450912,0\C,33,1.39538934,31,119.8142351
3,22,-151.21570497,0\H,33,1.08838371,31,120.77683335,22,28.15914279,0\
C,34,1.39780113,32,120.21705147,31,-0.10040836,0\H,34,1.08610277,32,11
9.50913905,31,-179.31232195,0\H,36,1.08671834,33,119.70547406,31,-179.
88007324,0\H,38,1.08695101,34,119.89983436,32,-179.90929886,0\C,22,2.9
9842984,21,103.33305827,19,-37.39139054,0\C,42,1.40616274,22,87.523217
09,21,-49.18447239,0\C,42,1.41110221,22,151.77828235,21,142.0500007,0\
C,43,1.39783697,42,119.46331439,22,-174.02963086,0\H,43,1.08584381,42,
121.38651927,22,6.92799404,0\C,44,1.39475791,42,119.80750612,22,167.10
755911,0\H,44,1.0880804,42,120.97742487,22,-13.30933155,0\C,45,1.39741
546,43,120.39880346,42,0.19111132,0\H,45,1.08649158,43,119.18409289,42
,-179.70981781,0\H,47,1.0867192,44,119.69105779,42,-179.93116393,0\H,4
9,1.08701654,45,119.97994171,43,179.79009566,0\P,22,1.80748486,21,120.
23325854,19,-5.30961683,0\C,53,1.82740964,22,108.34761469,21,174.90484
006,0\H,54,1.09444637,53,110.40034528,22,63.5405189,0\H,54,1.09472646,
53,108.33545373,22,-177.48635058,0\H,54,1.09464189,53,110.88501518,22,
-58.59747048,0\\Version=AM64L-G03RevD.01\State=1-A\HF=-1562.0416561\MP
2=-1566.5472425\RMSD=6.217e-09\Thermal=0.\PG=C01 [X(C27H26N2O1P1)]\\@

273

1\1\GINC-AZAZEL\SP\RMP2-FC\6-31+G(2d,p)\C19H17P1\CHRISTOPH\12-Aug-2011
\0\\#p MP2(FC)/6-31+g(2d,p) scf=tight\\tol1sp_8\\0,1\P\C,1,4.66936217\
C,2,1.3980344,1,61.35141464\C,3,1.39919371,2,120.30803693,1,-0.5319983
8,0\C,4,1.40579208,3,120.5681717,2,0.27479177,0\C,5,1.40809183,4,118.5
2843866,3,0.41930282,0\C,6,1.3968338,5,120.88870354,4,-1.03896013,0\H,
3,1.08817296,2,120.0506285,1,179.54237426,0\H,4,1.08722761,3,119.62369
61,2,-179.9640028,0\H,6,1.08893768,5,119.4890553,4,178.74528047,0\H,7,
1.0879797,6,119.8316622,5,-179.73657587,0\C,1,4.69135465,5,104.5461447
1,4,94.16993621,0\C,12,1.40224261,1,60.44809376,5,-15.53280878,0\C,13,
1.39844428,12,121.28124367,1,-0.30144635,0\C,14,1.40463032,13,120.7135
1551,12,0.13612486,0\C,15,1.40788546,14,118.03177276,13,0.39257397,0\C
,16,1.3950287,15,121.00704328,14,-0.97953333,0\H,13,1.08925816,12,119.
3812786,1,179.83156061,0\H,14,1.08736934,13,119.44424563,12,-179.92054
967,0\H,16,1.08900498,15,119.54317172,14,179.0378839,0\H,17,1.08922254
,16,119.49567725,15,-179.53169408,0\C,1,4.66922104,15,104.67939311,14,
92.21718834,0\C,22,1.39826342,1,61.3193716,15,-15.97164747,0\C,23,1.39
894745,22,120.2831749,1,-0.47089302,0\C,24,1.4059475,23,120.5825751,22
,0.19808124,0\C,25,1.40780543,24,118.54178093,23,0.4638128,0\C,26,1.39
717869,25,120.87149687,24,-1.00383991,0\H,23,1.0881779,22,120.04195034
,1,179.54966558,0\H,24,1.08730537,23,119.63545756,22,179.89665313,0\H,
26,1.08891413,25,119.46898597,24,178.72314877,0\H,27,1.08797417,26,119

.82793232,25,-179.78589836,0\C,12,1.51387509,1,177.93117954,15,149.983
58793,0\H,32,1.09920912,12,111.11848059,1,-40.92683401,0\H,32,1.097052
64,12,111.42379448,1,78.51803488,0\H,32,1.09596459,12,111.39579888,1,-
160.72492866,0\H,2,1.08786743,1,178.40795777,15,-105.0790857,0\H,22,1.
08787514,1,178.44500208,15,145.98117535,0\\Version=AM64L-G03RevD.01\St
ate=1-A\HF=-1070.1936038\MP2=-1073.0046306\RMSD=7.080e-09\Thermal=0.\P
G=C01 [X(C19H17P1)]\\@

273-Me+

1\1\GINC-AZAZEL\SP\RMP2-FC\6-31+G(2d,p)\C20H20P1(1+)\CHRISTOPH\12-Aug-
2011\0\\#p MP2(FC)/6-31+g(2d,p) scf=tight\\tol1mesp_7\\1,1\C\C,1,1.397
79289\C,2,1.39756682,1,120.15376022\C,3,1.40650471,2,119.70437525,1,-0
.01458411,0\C,4,1.40988356,3,120.01876052,2,0.24290656,0\C,5,1.3948266
7,4,119.76200061,3,-0.27926756,0\H,2,1.08661127,1,120.22184407,6,179.9
5705869,0\H,3,1.08654153,2,119.89975963,1,-179.72708961,0\H,5,1.087706
42,4,120.93340764,3,179.27744154,0\H,6,1.08662906,5,119.7035628,4,-179
.82622198,0\C,3,5.20646335,2,143.17509183,1,-161.41659097,0\C,11,1.408
00859,3,56.91246368,2,88.27493141,0\C,12,1.39192244,11,121.21418786,3,
38.61545473,0\C,13,1.41062147,12,119.94047376,11,-0.08264062,0\C,14,1.
4060481,13,119.40748729,12,-0.27192753,0\C,15,1.39578041,14,119.885715
06,13,0.27824837,0\H,12,1.08777514,11,119.52535843,3,-141.44108552,0\H
,13,1.08797179,12,119.13362181,11,-179.4415237,0\H,15,1.08670323,14,12
0.51736786,13,-179.81997244,0\H,16,1.08756725,15,119.16699245,14,-179.
99485637,0\C,15,5.17340267,14,95.45229717,13,170.01605088,0\C,21,1.397
91697,15,70.88416506,14,71.15623403,0\C,22,1.39738809,21,120.15549048,
15,-34.44336236,0\C,23,1.40673485,22,119.70486299,21,0.01497674,0\C,24
,1.40960905,23,120.01412938,22,0.24999117,0\C,25,1.39502983,24,119.767
93368,23,-0.32101012,0\H,22,1.08661688,21,120.22600603,15,145.72843669
,0\H,23,1.08658273,22,119.83515485,21,-179.70476555,0\H,25,1.08758061,
24,120.92013189,23,179.26891044,0\H,26,1.086635,25,119.70161999,24,-17
9.7834243,0\C,11,1.51035012,3,148.41727919,2,-7.46433521,0\H,31,1.0946
4592,11,111.55579927,3,-84.72793521,0\H,31,1.09584805,11,111.33108367,
3,36.55069483,0\H,31,1.09869732,11,110.43821238,3,155.61470221,0\H,1,1
.08689946,2,119.91884238,3,179.82269228,0\H,21,1.08690662,15,147.18660
531,14,-172.97928052,0\P,14,1.80405775,13,119.78582352,12,-179.3550553
6,0\C,37,1.82655329,14,108.51642227,13,-49.09422432,0\H,38,1.09477614,
37,109.76548964,14,62.87285109,0\H,38,1.09470182,37,110.10454521,14,-1
77.08306426,0\H,38,1.09463872,37,109.88572157,14,-56.91714672,0\\Versi
on=AM64L-G03RevD.01\State=1-A\HF=-1109.647251\MP2=-1112.6014492\RMSD=5
.424e-09\Thermal=0.\PG=C01 [X(C20H20P1)]\\@

274

1\1\GINC-CIPCLU07\SP\RMP2-FC\6-31+G(2d,p)\C14H16N1P1\C2175\18-May-2010
\0\\#p MP2(FC)/6-31+G(2d,p) scf=tight\\xnmesp_18\\0,1\P\C,1,4.66370954
\C,2,1.39817278,1,59.71583493\C,3,1.39805161,2,120.30316565,1,-2.51632
188,0\C,4,1.40694429,3,120.77862931,2,-0.72302025,0\C,5,1.40803451,4,1
18.35124017,3,1.32352525,0\C,6,1.39753131,5,120.79754994,4,-1.08673348
,0\H,2,1.08779277,1,177.72426284,5,-179.81049655,0\H,3,1.0882362,2,120
.06456372,1,177.33881586,0\H,4,1.08752035,3,120.11654284,2,179.3755911
9,0\H,6,1.08813804,5,119.89489906,4,179.34014731,0\H,7,1.08812821,6,11
9.62714341,5,-179.60895624,0\C,1,4.67896736,5,105.50905379,4,142.22625
397,0\C,13,1.39971648,1,61.70682284,5,-31.04341112,0\C,14,1.39741569,1
3,120.20124686,1,1.31394841,0\C,15,1.40851635,14,120.80877483,13,0.044
0459,0\C,16,1.40735414,15,118.20579326,14,0.24718238,0\C,13,1.39750489
,1,58.07431436,5,147.37516628,0\H,13,1.08796002,1,177.92374042,5,179.7
8212668,0\H,14,1.08825001,13,120.01180574,1,-178.71283377,0\H,15,1.087
54714,14,119.33998613,13,179.09323221,0\H,17,1.08906433,16,119.2140398
8,15,179.16041445,0\H,18,1.08799709,13,120.16053648,1,178.90896386,0\C

,1,2.785921,5,88.47056804,4,56.39926056,0\H,24,1.09899802,1,139.339931
81,5,-95.52621694,0\H,24,1.1034507,1,102.10596377,5,126.76332058,0\H,2
4,1.09450155,1,86.68993639,5,18.49082134,0\C,24,2.43484779,1,61.785105
04,5,-141.5801526,0\H,28,1.1045224,24,94.645463,1,-104.6948637,0\H,28,
1.09467508,24,143.22554701,1,24.92138799,0\H,28,1.09921323,24,90.34022
034,1,147.3997398,0\N,24,1.46099004,1,31.8997235,5,-119.39453644,0\\Ve
rsion=AM64L-G03RevD.01\State=1-A\HF=-934.6901973\MP2=-937.0014596\RMSD
=4.744e-09\Thermal=0.\PG=C01 [X(C14H16N1P1)]\\@

276-Me$^+$

1\1\GINC-CIPCLU06\SP\RMP2-FC\6-31+G(2d,p)\C15H19N1P1(1+)\C2175\18-May-
2010\0\\#p MP2(FC)/6-31+G(2d,p) scf=tight\\xnmemesp_2\\1,1\C\C,1,1.399
86866\C,2,1.39500352,1,120.07877232\C,3,1.41193015,2,120.04189458,1,-0
.08885244,0\C,4,1.40848136,3,119.50183916,2,0.2577256,0\C,5,1.39683598
,4,120.02260151,3,-0.40022558,0\H,1,1.08685905,6,119.89449264,5,179.98
200798,0\H,2,1.08664654,1,120.19691003,6,-179.71278838,0\H,3,1.0875776
,2,119.37940162,1,-179.01513646,0\H,5,1.08632229,4,120.97444054,3,-179
.78869179,0\H,6,1.08658531,5,119.63802446,4,-179.50810547,0\C,4,5.5227
0421,3,84.53657915,2,-145.84446807,0\C,12,1.39848878,4,44.08075402,3,7
0.79401961,0\C,13,1.39680404,12,120.14136061,4,6.80884951,0\C,14,1.407
93678,13,119.66259938,12,-0.03982698,0\C,15,1.40973573,14,120.08180544
,13,0.58073603,0\C,16,1.3952421,15,119.68606636,14,-0.82393042,0\H,12,
1.08692311,4,163.0282704,3,49.58848218,0\H,13,1.0865811,12,120.2210368
2,4,-173.02174968,0\H,14,1.08664148,13,119.70431201,12,-179.84418103,0
\H,16,1.08715061,15,120.22347641,14,179.72923407,0\H,17,1.08663898,16,
119.69914466,15,-179.52808279,0\C,4,3.66255322,3,115.96556826,2,123.89
029456,0\H,23,1.09234438,4,98.01285729,3,176.02881204,0\H,23,1.0995342
4,4,73.01660423,3,-75.89689311,0\C,23,2.45576858,4,66.10848649,3,30.06
03429,0\H,26,1.09500046,23,88.80161733,4,178.68135844,0\H,26,1.0993715
2,23,93.78735931,4,-72.52333369,0\P,15,1.80347122,14,120.53746535,13,1
74.76380604,0\C,29,1.81877426,15,108.36401597,14,-94.35250448,0\H,30,1
.09514462,29,108.42872534,15,-61.37197735,0\H,30,1.09530909,29,108.190
44416,15,56.25501925,0\H,30,1.09355175,29,112.93131661,15,177.16203474
,0\H,23,1.09467882,4,151.54986857,3,21.87270662,0\H,26,1.09338374,23,1
44.10422075,4,58.6733485,0\N,23,1.47639232,4,49.74694795,3,65.3654849,
0\\Version=AM64L-G03RevD.01\State=1-A\HF=-974.1436841\MP2=-976.5992307
\RMSD=7.647e-09\Thermal=0.\PG=C01 [X(C15H19N1P1)]\\@

275

1\1\GINC-NODE8\SP\RMP2-FC\6-31+G(2d,p)\C17H21P1\ZIP07\27-May-2010\0\\#
p MP2(FC)/6-31+G(2d,p) scf=tight\\xpesp_107\\0,1\P\C,1,4.6755601\C,2,1
.39632589,1,60.8016224\C,3,1.40015634,2,120.35521024,1,1.70020145,0\C,
4,1.40600635,3,120.85672621,2,-0.07786978,0\C,5,1.40976669,4,118.08183
377,3,-0.47506288,0\C,6,1.3953605,5,121.06182077,4,0.85168354,0\H,2,1.
08771124,1,178.25539701,5,179.1766473,0\H,3,1.08820808,2,120.10015704,
1,-178.55185552,0\H,4,1.08693726,3,118.7645575,2,179.72605016,0\H,6,1.
08876874,5,119.55464558,4,-179.75416263,0\H,7,1.08805193,6,119.6935105
2,5,179.48110786,0\C,1,4.67216004,5,102.95534849,4,-125.41628584,0\C,1
3,1.40051724,1,60.97893273,5,39.6690801,0\C,14,1.39686329,13,120.12624
335,1,-0.75205982,0\C,15,1.40801415,14,120.68498501,13,0.04431322,0\C,
16,1.40514949,15,118.55562963,14,-0.31421291,0\C,13,1.39693991,1,58.84
061436,5,-139.42307569,0\H,13,1.08790014,1,178.83821535,5,-168.1992372
8,0\H,14,1.08813764,13,120.02828395,1,179.42980594,0\H,15,1.08824221,1
4,119.63831625,13,-179.07090997,0\H,17,1.08853328,16,119.31013377,15,-
179.44177954,0\H,18,1.08795347,13,120.15100875,1,-179.40416381,0\C,1,1
.8766674,5,102.00131341,4,-21.91172236,0\H,24,1.09999926,1,107.1897412
8,5,69.31230786,0\H,24,1.09885203,1,110.07141279,5,-47.14850836,0\C,24
,1.54041816,1,111.90806381,5,-170.97584537,0\H,27,1.09822938,24,109.44

506199,1,60.65826478,0\H,27,1.09962188,24,109.03659427,1,-55.01568776,
0\C,27,1.54321845,24,113.74692572,1,-176.26538798,0\H,30,1.10076235,27
,108.97078394,24,61.76414411,0\H,30,1.10086765,27,108.12486738,24,176.
78307999,0\C,30,1.54242231,27,115.69652168,24,-61.1557,0\H,33,1.099771
62,30,109.84233167,27,64.2574332,0\H,33,1.09960371,30,108.11591877,27,
179.25174607,0\C,33,1.53681226,30,114.25000597,27,-59.07532614,0\H,36,
1.09861447,33,111.07253891,30,-55.60493386,0\H,36,1.09749704,33,111.91
133459,30,64.65555558,0\H,36,1.09728097,33,110.91000554,30,-175.314338
06,0\\Version=AM64L-G03RevD.01\State=1-A\HF=-996.7811293\MP2=-999.3671
151\RMSD=8.830e-09\Thermal=0.\PG=C01 [X(C17H21P1)]\\@

275-Me$^+$

1\1\GINC-NODE5\SP\RMP2-FC\6-31+G(2d,p)\C18H24P1(1+)\ZIP07\31-May-2010\
0\\#p MP2(FC)/6-31+G(2d,p) scf=tight\\xpemesp_1\\1,1\C\C,1,1.3982115\C
,2,1.39682234,1,120.12768951\C,3,1.40778141,2,119.85378105,1,-0.104306
39,0\C,4,1.41026903,3,119.82251766,2,0.02047792,0\C,5,1.39544804,4,119
.8180883,3,0.0370539,0\H,1,1.08685001,2,119.87714667,3,179.90342364,0\
H,2,1.08653386,1,120.25614835,6,-179.66598116,0\H,3,1.08659998,2,119.0
8736909,1,-179.79074586,0\H,5,1.08721065,4,120.53574932,3,-179.0975898
8,0\H,6,1.08661366,5,119.65490243,4,-179.7756649,0\C,5,5.48062326,4,84
.24760201,3,-147.99349798,0\C,12,1.39909528,5,44.49708207,4,86.0214732
1,0\C,13,1.3960442,12,120.14599393,5,-32.18623524,0\C,14,1.40879474,13
,119.81152895,12,-0.00746033,0\C,15,1.40840888,14,119.85346977,13,-0.2
1911056,0\C,16,1.39633479,15,119.83894799,14,0.32642393,0\H,12,1.08682
898,5,150.30390257,4,155.60627866,0\H,13,1.0866024,12,120.22082507,5,1
47.64309862,0\H,14,1.0872198,13,119.64818917,12,179.45170427,0\H,16,1.
08711525,15,121.05462544,14,-179.84398685,0\H,17,1.08657185,16,119.639
58553,15,179.65451766,0\C,4,2.99505444,3,119.26662391,2,136.56522758,0
\H,23,1.09949995,4,87.9608806,3,-18.83890787,0\H,23,1.09947441,4,141.3
7780079,3,95.27467661,0\C,23,1.54327844,4,96.10670793,3,-129.14824182,
0\H,26,1.09876341,23,109.96760279,4,-87.18925573,0\H,26,1.09827523,23,
109.41056767,4,29.9684353,0\C,26,1.54048643,23,111.43408676,4,151.1643
8833,0\H,29,1.1004402,26,109.35459938,23,-57.84556584,0\H,29,1.1008394
6,26,109.39596344,23,58.32010736,0\C,29,1.53821496,26,112.63992847,23,
-179.76758944,0\H,32,1.10001187,29,109.21833326,26,58.2310992,0\H,32,1
.10004481,29,109.23440061,26,-57.74556385,0\C,32,1.5353414,29,112.6447
8183,26,-179.78213825,0\H,35,1.09586625,32,110.96097131,29,-179.711170
07,0\H,35,1.09751584,32,111.24491462,29,60.35993585,0\H,35,1.09748456,
32,111.20800229,29,-59.80854447,0\P,4,1.80659154,3,120.58831914,2,177.
12914628,0\C,39,1.82549431,4,109.60467543,3,18.92488829,0\H,40,1.09555
143,39,108.34111401,4,175.65047867,0\H,40,1.09523813,39,111.09237775,4
,-64.88117188,0\H,40,1.09493157,39,110.17683748,4,56.86043272,0\\Versi
on=AM64L-G03RevD.01\State=1-A\HF=-1036.2364551\MP2=-1038.9639487\RMSD=
5.023e-09\Thermal=0.\PG=C01 [X(C18H24P1)]\\@

276

1\1\GINC-LX64I158\SP\RMP2-FC\6-31+G(2d,p)\C16H19P1\UI271AC\06-Jul-2010
\0\\#MP2(FC)/6-31+g(2d,p) scf=tight\\xsbusp_6\\0,1\C\C,1,1.39807148\C,
2,1.3988145,1,120.24417459\C,3,1.40768688,2,120.93528569,1,0.28011447,
0\C,4,1.40798129,3,118.00048458,2,0.42400966,0\C,5,1.39749787,4,121.18
290083,3,-1.01794605,0\H,1,1.08778285,2,120.17167043,3,179.97241042,0\
H,2,1.08819309,1,120.06592073,6,179.67579215,0\H,3,1.08759201,2,118.77
02707,1,-179.97457465,0\H,5,1.08872221,4,119.21485558,3,178.78266306,0
\H,6,1.08792955,5,119.81023871,4,-179.6826152,0\C,4,5.43205249,3,105.2
7914531,2,-120.4453573,0\C,12,1.39951565,4,45.92752043,3,14.34348621,0
\C,13,1.39739727,12,120.23945174,4,-14.47931019,0\C,14,1.40681131,13,1
20.75963693,12,0.05808129,0\C,15,1.40688953,14,118.3424538,13,0.542349
22,0\C,12,1.3974609,4,74.88229673,3,-152.95119731,0\H,12,1.08791104,4,

161.9043835,3,58.81184695,0\H,13,1.08821206,12,120.01949815,4,165.3782
5536,0\H,14,1.08768248,13,119.33709565,12,179.14719867,0\H,16,1.088668
07,15,119.31375096,14,178.87300953,0\H,17,1.08801426,12,120.17096495,4
,-169.02262683,0\C,15,2.90000042,14,105.85157264,13,-139.71938343,0\C,
23,1.54158695,15,89.25666033,14,112.39271109,0\H,24,1.09571047,23,111.
54472084,15,-25.16597875,0\H,24,1.0968487,23,110.81937255,15,-144.9479
4006,0\H,24,1.09855057,23,110.92569157,15,95.29394487,0\C,23,1.5481568
2,15,147.67576169,14,-114.3699322,0\H,28,1.10013078,23,108.69640356,15
,-71.54369619,0\H,28,1.09826904,23,109.50617693,15,44.0496285,0\P,4,1.
85546517,3,125.7154388,2,179.89626732,0\C,28,1.53817058,23,114.0515382
9,15,165.81201759,0\H,32,1.0985395,28,111.11138734,23,-57.48305767,0\H
,32,1.09719662,28,110.51087908,23,-176.90519066,0\H,32,1.09641167,28,1
12.17066211,23,63.21037783,0\H,23,1.10139335,15,85.07715085,14,4.24467
101,0\\Version=IA64L-G03RevE.01\State=1-A\HF=-957.7398525\MP2=-960.176
9359\RMSD=7.437e-09\Thermal=0.\PG=C01 [X(C16H19P1)]\\@

276-Me$^+$

1\1\GINC-LX64I158\SP\RMP2-FC\6-31+G(2d,p)\C17H22P1(1+)\UI271AC\07-Jul-
2010\0\\#MP2(FC)/6-31+g(2d,p) scf=tight\\xsbumesp_2\\1,1\C\C,1,1.39969
176\C,2,1.3954421,1,120.14488363\C,3,1.41046172,2,119.8776318,1,-0.006
30388,0\C,4,1.40796471,3,119.71825004,2,-0.03805498,0\C,5,1.39699461,4
,119.9164383,3,-0.01630512,0\H,1,1.08684181,6,119.89547917,5,-179.8933
4784,0\H,2,1.08662731,1,120.2088194,6,-179.63895497,0\H,3,1.08702588,2
,119.57883648,1,-179.04287179,0\H,5,1.08636529,4,120.99693298,3,-179.6
0701358,0\H,6,1.0865557,5,119.60928345,4,-179.69223455,0\C,3,5.4817370
7,2,141.1319327,1,-121.72187412,0\C,12,1.39877718,3,81.83986644,2,-162
.86487262,0\C,13,1.39628495,12,120.13192689,3,-21.55204937,0\C,14,1.40
856024,13,119.89289758,12,0.27355342,0\C,15,1.40875658,14,119.75843737
,13,-0.49463345,0\C,16,1.39626235,15,119.86489638,14,0.37796028,0\H,12
,1.08682989,3,150.3000481,2,-23.00908646,0\H,13,1.08657203,12,120.2404
5223,3,158.34949126,0\H,14,1.08702411,13,119.04084235,12,-179.93704398
,0\H,16,1.08708676,15,120.52554577,14,179.91532027,0\H,17,1.08662517,1
6,119.61821902,15,179.82436798,0\C,4,3.01519834,3,107.59152684,2,140.9
512918,0\C,23,1.54461066,4,93.11495896,3,-121.08750938,0\H,24,1.096537
8,23,111.41650571,4,31.64795842,0\H,24,1.09532367,23,112.47531286,4,-9
0.81423056,0\H,24,1.09443526,23,109.24787061,4,150.59421925,0\C,23,1.5
5236406,4,93.18793885,3,-7.87610118,0\H,28,1.09835832,23,108.85705275,
4,-41.27253026,0\H,28,1.09823044,23,109.87439996,4,75.40759257,0\P,4,1
.80862161,3,119.59626295,2,177.21021995,0\C,31,1.82662167,4,109.428103
48,3,162.22992498,0\H,32,1.09374381,31,112.03697076,4,61.32941145,0\H,
32,1.09577565,31,108.38027743,4,-178.4731525,0\H,32,1.09506968,31,109.
34846292,4,-60.25252139,0\C,28,1.53830918,23,112.82638183,4,-163.40076
815,0\H,36,1.09545273,28,109.77883181,23,-176.93145458,0\H,36,1.095226
68,28,112.32822499,23,63.71317357,0\H,36,1.09742311,28,111.33684826,23
,-57.76624063,0\H,23,1.10129684,4,138.74985227,3,115.91053103,0\\Versi
on=IA64L-G03RevE.01\State=1-A\HF=-997.1915726\MP2=-999.7745409\RMSD=5.
910e-09\Thermal=0.\PG=C01 [X(C17H22P1)]\\@

276-BH$^+$

1\1\GINC-YIN\SP\RMP2-FC\6-31+G(2d,p)\C29H30P1(1+)\CHRISTOPH\25-Jul-201
0\0\\#p MP2(FC)/6-31+g(2d,p) scf=tight\\xsbubhsp_42\\1,1\C\H,1,1.09766
577\C,1,4.35092514,2,106.15811085\C,3,1.3982887,1,59.37452365,2,-27.86
340478,0\C,4,1.39725097,3,120.09310619,1,0.2286676,0\C,5,1.40663925,4,
120.74432161,3,-0.4367992,0\C,6,1.4039507,5,118.70402696,4,0.85788653,
0\C,3,1.39772117,1,60.19037235,6,0.61709925,0\H,3,1.08701789,1,179.468
89852,6,-143.72113466,0\H,4,1.0871418,3,120.25272358,1,179.33545471,0\
H,5,1.08745893,4,119.18728364,3,179.04739024,0\H,7,1.08539652,6,120.26
658373,5,178.0693576,0\H,8,1.08714618,3,120.13100832,1,-179.43158282,0

\C,1,4.3465786,6,115.41697471,5,-144.65610548,0\C,14,1.39734879,1,60.7
3532267,6,-79.81624942,0\C,15,1.39904516,14,120.32923906,1,-0.39519118
,0\C,16,1.40576959,15,120.56630853,14,-0.25769491,0\C,17,1.40568984,16
,118.53951804,15,0.28241005,0\C,18,1.39695343,17,120.86597208,16,-0.01
842721,0\H,14,1.08683356,1,178.92888017,17,175.52572639,0\H,15,1.08722
036,14,120.11878763,1,179.18559535,0\H,16,1.08730109,15,118.975613,14,
178.99565956,0\H,18,1.08894988,17,119.6816734,16,179.14287194,0\H,19,1
.08698519,18,119.66140026,17,179.65926072,0\P,1,1.89337642,17,112.6006
1063,16,51.64190614,0\C,25,1.86499535,1,110.99145278,17,39.08743471,0\
C,26,1.55253134,25,111.34354162,1,49.53258159,0\H,27,1.09837786,26,109
.13688718,25,67.52887776,0\H,27,1.09660201,26,109.81870861,25,-50.1630
7829,0\C,26,1.54524762,25,110.21242496,1,174.63036223,0\H,30,1.0958378
5,26,110.79613928,25,-70.25343431,0\H,30,1.094863,26,109.36032192,25,1
70.79129135,0\H,30,1.09463391,26,112.99913333,25,51.21745,0\C,25,4.623
13999,1,109.03717914,17,-83.33570509,0\C,34,1.3982746,25,59.64771101,1
,-71.99187645,0\C,35,1.3964089,34,120.28325064,25,-0.77117341,0\C,36,1
.4107562,35,120.00319598,34,-0.23406873,0\C,37,1.40936,36,119.35864128
,35,0.4405902,0\C,38,1.39641505,37,120.15436376,36,-0.3691819,0\H,34,1
.08694288,25,179.30991981,1,-11.15777111,0\H,35,1.08664172,34,120.2632
3319,25,179.04871117,0\H,36,1.08638638,35,119.39575193,34,179.73796242
,0\H,38,1.0860181,37,120.97471006,36,179.49783872,0\H,39,1.08670557,38
,119.56973356,37,-179.7136699,0\C,25,4.62742856,1,110.47392878,17,155.
22764054,0\C,45,1.39900791,25,60.00360745,1,-38.63874137,0\C,46,1.3959
8627,45,120.13846695,25,1.74215167,0\C,47,1.40998959,46,120.27083773,4
5,0.36050244,0\C,48,1.40776358,47,119.20294306,46,-0.9789836,0\C,45,1.
39742074,25,59.96558395,1,139.89916159,0\H,45,1.08684214,25,178.584378
09,1,-128.52138658,0\H,46,1.08675215,45,120.26151725,25,-178.23272313,
0\H,47,1.0869069,46,118.65373541,45,-179.58857431,0\H,49,1.08589738,48
,120.46005311,47,-179.23148371,0\H,50,1.08673216,45,120.23299978,25,17
8.22883611,0\C,27,1.53869955,26,112.58600528,25,-170.58391799,0\H,56,1
.09566362,27,109.75383615,26,176.48847959,0\H,56,1.09516205,27,112.306
16337,26,-64.09255093,0\H,56,1.09713609,27,111.10654193,26,57.48045798
,0\H,26,1.0992833,25,104.66660281,1,-66.88591713,0\\Version=AM64L-G03R
evD.01\State=1-A\HF=-1456.3019187\MP2=-1460.5867825\RMSD=9.258e-09\The
rmal=0.\PG=C01 [X(C29H30P1)]\\@

276-TT$^+$

1\1\GINC-BORIX\SP\RMP2-FC\6-31+G(2d,p)\C35H34P1(1+)\CHRISTOPH\31-Aug-2
011\0\\#p MP2(FC)/6-31+g(2d,p) scf=tight\\xibuttsp_13\\1,1\C\C,1,4.387
36425\C,2,1.39718569,1,59.93680637\C,3,1.39732289,2,120.37439679,1,0.6
9636194,0\C,4,1.40803452,3,121.285328,2,1.21060886,0\C,5,1.40743054,4,
117.58515426,3,-2.69024955,0\C,2,1.39623543,1,59.1273593,5,108.2446694
2,0\H,2,1.08689772,1,179.20725939,5,167.47110638,0\H,3,1.08732737,2,12
0.27924802,1,-178.55710571,0\H,4,1.08350747,3,118.18846256,2,-177.0483
7843,0\H,6,1.08436649,5,120.28469516,4,-177.17650132,0\H,7,1.08715726,
2,120.23659878,1,178.55566389,0\C,1,4.38898753,5,111.02577619,4,71.421
38263,0\C,13,1.39537469,1,59.40150192,5,2.34669775,0\C,14,1.39948758,1
3,120.61356752,1,0.0059963,0\C,15,1.40585896,14,121.13183081,13,0.0327
1534,0\C,16,1.4128174,15,117.53841098,14,0.99112221,0\C,17,1.39683526,
16,121.25588649,15,-1.2566581,0\H,13,1.08700786,1,179.88078281,16,167.
32175644,0\H,14,1.08711976,13,120.22298521,1,179.84733662,0\H,15,1.083
56704,14,118.58315232,13,179.6125528,0\H,17,1.08422301,16,120.38574548
,15,178.27046028,0\H,18,1.087374,17,119.34381767,16,-179.98627677,0\C,
1,4.39414506,16,113.61844393,15,-122.03212078,0\C,24,1.3992233,1,59.18
969463,16,179.42340462,0\C,25,1.39570794,24,120.19698509,1,-0.30862339
,0\C,26,1.41213798,25,121.64903957,24,1.19037183,0\C,27,1.4051366,26,1
17.17914651,25,-3.25185865,0\C,24,1.394623,1,59.80524928,16,1.12845578
,0\H,24,1.08689847,1,179.62235113,16,-164.94074445,0\H,25,1.08731665,2

4,120.30296656,1,-179.09883172,0\H,26,1.08421685,25,118.22806149,24,-1
75.8067631,0\H,28,1.08304007,27,120.48577879,26,-176.02139147,0\H,29,1
.08713609,24,120.20782061,1,179.42333994,0\P,1,2.00124089,16,105.87836
894,15,120.72898844,0\C,35,4.64491623,1,111.91842787,16,68.92180544,0\
C,36,1.39856139,35,59.77130203,1,59.10588104,0\C,37,1.39655243,36,120.
415952,35,-0.95867084,0\C,38,1.41143199,37,120.07322352,36,-0.46195183
,0\C,39,1.40818554,38,119.14696103,37,1.07614858,0\C,36,1.39728742,35,
60.02522409,1,-120.27727139,0\H,36,1.0869684,35,179.066515,1,140.12652
849,0\H,37,1.08687096,36,120.22627424,35,178.9100308,0\H,38,1.08505835
,37,118.92746513,36,-179.86877763,0\H,40,1.0848529,39,120.57731909,38,
178.63505495,0\H,41,1.08688617,36,120.27143033,35,-178.63374934,0\C,35
,4.65113908,1,111.76989257,16,-51.77306479,0\C,47,1.39782246,35,59.981
35079,1,57.56123343,0\C,48,1.39658985,47,120.54102412,35,0.01985739,0\
C,49,1.4123525,48,120.25534797,47,0.04799541,0\C,50,1.40979436,49,118.
66618434,48,0.11014946,0\C,51,1.39649768,50,120.62228567,49,-0.1052897
5,0\H,47,1.08690897,35,179.80955377,1,-92.61604071,0\H,48,1.08681619,4
7,120.25790896,35,-179.94120778,0\H,49,1.08372878,48,118.88114439,47,1
79.78400273,0\H,51,1.0820208,50,120.88550635,49,-179.78394426,0\H,52,1
.08678983,51,119.41066455,50,179.94303737,0\C,35,2.86370745,1,143.8383
2939,16,176.52908924,0\C,58,1.56391208,35,37.57701939,1,10.29649215,0\
C,59,1.54323944,58,111.88338322,35,134.16389066,0\C,58,1.53778854,35,1
36.58019267,1,-49.28438556,0\H,58,1.09665605,35,110.27531821,1,105.639
07601,0\H,58,1.09595772,35,75.74909498,1,-151.65404994,0\H,59,1.098017
62,58,106.26458488,35,-108.27632423,0\H,60,1.09276679,59,113.16781994,
58,169.08964855,0\H,60,1.0940859,59,112.70879117,58,-69.11849553,0\H,6
1,1.09578443,58,110.29764791,35,-149.92790925,0\H,61,1.09708125,58,110
.91441402,35,-30.52632098,0\H,60,1.09665679,59,108.06598567,58,49.6722
8857,0\H,61,1.09686858,58,111.92139021,35,90.35788427,0\\Version=AM64L
-G03RevD.01\State=1-A\HF=-1685.8192763\MP2=-1690.9811784\RMSD=7.180e-0
9\Thermal=0.\PG=C01 [X(C35H34P1)]\\@

277

1\1\GINC-NODE21\SP\RMP2-FC\6-31+G(2d,p)\C17H21P1\ZIP07\14-Aug-2010\0\\
#p MP2(FC)/6-31+G(2d,p) scf=tight\\xi5sp_15\\0,1\C\C,1,1.39747031\C,2,
1.39900709,1,119.97911397\C,3,1.40679599,2,120.97617889,1,-0.64348375,
0\C,4,1.40673167,3,118.35088114,2,0.86137178,0\C,5,1.39744792,4,120.76
794168,3,-0.49110818,0\H,1,1.0879028,2,120.1667687,3,-179.55026574,0\H
,2,1.08798301,1,120.16964017,6,179.50484317,0\H,3,1.08868267,2,119.690
50936,1,179.02542698,0\H,5,1.08756234,4,119.84444221,3,178.58863017,0\
H,6,1.08817586,5,119.78279424,4,179.75635629,0\C,5,5.18185379,4,90.082
10233,3,147.87133761,0\C,12,1.39794527,5,56.23929639,4,107.23859598,0\
C,13,1.39912785,12,120.24561171,5,-39.05424814,0\C,14,1.40748901,13,12
0.92090484,12,-0.2562075,0\C,15,1.40803947,14,118.0103603,13,-0.485105
15,0\C,16,1.39747808,15,121.19095918,14,1.06776681,0\H,12,1.08777987,5
,146.81520816,4,-158.05496377,0\H,13,1.08817953,12,120.06673097,5,140.
84705618,0\H,14,1.08760276,13,118.77252518,12,179.98852015,0\H,16,1.08
864439,15,119.18484165,14,-178.75220306,0\H,17,1.08789642,16,119.82890
818,15,179.66653958,0\C,4,2.90013366,3,121.71015698,2,-132.81515476,0\
H,23,1.10184289,4,84.18042037,3,135.16991513,0\C,23,1.55181601,4,89.77
436016,3,28.76826074,0\H,25,1.09942037,23,107.96088932,4,137.06309572,
0\H,25,1.09800977,23,108.63122369,4,22.98139586,0\C,23,1.54845945,4,14
7.65727419,3,-108.94693986,0\H,28,1.09896475,23,109.43052369,4,69.1282
9096,0\H,28,1.09791159,23,109.10941918,4,-46.24852068,0\P,15,1.8554449
4,14,125.98821396,13,179.57831532,0\C,25,1.53782171,23,115.99782742,4,
-100.4680536,0\H,32,1.09718622,25,110.20300183,23,177.20636221,0\H,32,
1.09722164,25,111.92421357,23,-63.31685857,0\H,32,1.09690027,25,111.77
003072,23,57.30874024,0\C,28,1.53829072,23,114.00491926,4,-167.8140274
,0\H,36,1.09855152,28,111.17813662,23,56.15028852,0\H,36,1.09719455,28

,110.53033164,23,175.65903656,0\H,36,1.0967368,28,112.08711407,23,-64.
44475667,0\\Version=AM64L-G03RevD.01\State=1-A\HF=-996.7745646\MP2=-99
9.3681831\RMSD=6.983e-09\Thermal=0.\PG=C01 [X(C17H21P1)]\\@

277-Me⁺

1\1\GINC-CIPCLU06\SP\RMP2-FC\6-31+G(2d,p)\C18H24P1(1+)\C2175\17-Aug-20
10\0\\#p MP2(FC)/6-31+G(2d,p) scf=tight\\xi5mesp_35\\1,1\C\C,1,1.39767
541\C,2,1.39768319,1,120.15365795\C,3,1.40684225,2,119.90748369,1,0.03
254716,0\C,4,1.40962656,3,119.68453869,2,-0.38744927,0\C,5,1.39494346,
4,119.98428709,3,0.48460733,0\H,1,1.08683494,2,119.92466159,3,-179.881
01879,0\H,2,1.08659264,1,120.24999073,6,-179.87984153,0\H,3,1.08628102
,2,119.08406491,1,179.82773014,0\H,5,1.08773278,4,120.77395292,3,-178.
90181817,0\H,6,1.08659361,5,119.68725403,4,179.85724332,0\C,4,5.476349
9,3,122.20713807,2,-115.58499591,0\C,12,1.39886947,4,75.80985877,3,-13
8.69684376,0\C,13,1.39650086,12,120.12079133,4,9.65162997,0\C,14,1.408
06504,13,119.81697061,12,-0.04393031,0\C,15,1.40844785,14,119.88038774
,13,-0.37118993,0\C,16,1.39579332,15,119.82824418,14,0.51629359,0\H,12
,1.08685902,4,161.92969058,3,69.51122576,0\H,13,1.0866382,12,120.22850
133,4,-170.10316451,0\H,14,1.08705719,13,119.12675404,12,-179.76257606
,0\H,16,1.08738785,15,120.4578077,14,-178.7736281,0\H,17,1.08663907,16
,119.67186461,15,-179.82635638,0\C,4,3.01760786,3,87.69658421,2,173.32
131264,0\H,23,1.1004467,4,82.20369748,3,36.0676446,0\C,23,1.55727658,4
,94.60000527,3,-72.2545574,0\H,25,1.09866754,23,108.71500478,4,-109.75
909504,0\H,25,1.09867849,23,110.86966542,4,7.22870019,0\C,23,1.5606334
7,4,144.31624838,3,146.29293884,0\H,28,1.09685648,23,107.10122077,4,15
8.25573252,0\H,28,1.09970495,23,110.89802856,4,43.96824306,0\P,15,1.81
032123,14,120.92540307,13,177.37072943,0\C,31,1.8258059,15,108.5679972
9,14,28.62441885,0\H,32,1.0943369,31,110.21241088,15,50.59432216,0\H,3
2,1.09500625,31,110.58900477,15,-69.84278247,0\H,32,1.09432485,31,109.
20450657,15,170.4188145,0\C,25,1.53778469,23,112.10651504,4,128.616591
69,0\H,36,1.09739704,25,111.1321075,23,-62.70013604,0\H,36,1.09605886,
25,112.1359573,23,58.19083185,0\H,36,1.09538783,25,110.10867982,23,177
.93167967,0\C,28,1.53561529,23,114.21586099,4,-80.90111193,0\H,40,1.09
569796,28,110.28403751,23,-178.47869536,0\H,40,1.09719811,28,110.67144
317,23,-59.46611839,0\H,40,1.09559143,28,112.18695623,23,61.36621422,0
\\Version=AM64L-G03RevD.01\State=1-A\HF=-1036.2262185\MP2=-1038.965397
3\RMSD=9.702e-09\Thermal=0.\PG=C01 [X(C18H24P1)]\\@

278

1\1\GINC-CIPCLU06\SP\RMP2-FC\6-31+G(2d,p)\C16H17P1\C2175\26-May-2010\0
\\#p MP2(FC)/6-31+G(2d,p) scf=tight\\x4sp_20\\0,1\P\C,1,4.67220569\C,2
,1.39761961,1,58.41314936\C,3,1.39883609,2,119.98746509,1,0.69293871,0
\C,4,1.40751063,3,120.99335764,2,-0.85105778,0\C,5,1.40801738,4,118.33
706243,3,0.9888756,0\C,6,1.39782345,5,120.70440487,4,-0.41983246,0\H,2
,1.08787513,1,178.58891845,5,-174.6271182,0\H,3,1.0879912,2,120.167846
25,1,179.92081534,0\H,4,1.08882251,3,119.67540597,2,178.41069698,0\H,6
,1.08754501,5,119.6871259,4,178.77706938,0\H,7,1.08818664,6,119.703049
1,5,179.78501449,0\C,1,4.67096513,5,103.51249986,4,-151.56037222,0\C,1
3,1.39645598,1,60.92548698,5,-117.89418588,0\C,14,1.40008747,13,120.36
224587,1,2.03233758,0\C,15,1.40604481,14,120.87481839,13,-0.27547706,0
\C,16,1.41019469,15,118.03668593,14,-0.38945532,0\C,17,1.39545433,16,1
21.1019915,15,0.96925611,0\H,13,1.08770395,1,177.96296487,16,-179.3527
3009,0\H,14,1.08817028,13,120.09120601,1,-178.28682686,0\H,15,1.086569
92,14,118.89364438,13,179.62249223,0\H,17,1.08884075,16,119.52921359,1
5,-179.27204326,0\H,18,1.08805679,17,119.70578469,16,179.5836173,0\H,1
,2.41701327,16,92.29614275,15,8.91279885,0\C,1,1.8720657,16,103.479128
85,15,-14.75665875,0\C,25,1.56260026,1,116.76551013,16,-172.01081186,0
\C,26,1.55520635,25,88.3371471,1,148.06449319,0\C,27,1.55520275,26,88.

88397375,25,-18.7091985,0\H,26,1.09547978,25,117.18952858,1,-90.705625
77,0\H,26,1.0966392,25,111.64551982,1,35.84341732,0\H,27,1.09653166,26
,111.69901355,25,94.33808158,0\H,27,1.09582493,26,117.29593534,25,-139
.18868527,0\H,28,1.09592292,27,117.84089445,26,139.07324457,0\H,28,1.0
9559793,27,111.60598776,26,-93.98609635,0\\Version=AM64L-G03RevD.01\St
ate=1-A\HF=-956.5400824\MP2=-958.9539415\RMSD=4.251e-09\Thermal=0.\PG=
C01 [X(C16H17P1)]\\@

278-Me+

1\1\GINC-AZAZEL\SP\RMP2-FC\6-31+G(2d,p)\C17H20P1(1+)\CHRISTOPH\28-May-
2010\0\\#p MP2(FC)/6-31+g(2d,p) scf=tight\\x4mesp_23\\1,1\C\C,1,1.3992
8396\C,2,1.39594489,1,120.12547793\C,3,1.40956639,2,119.8398153,1,-0.1
0772585,0\C,4,1.40837298,3,119.81114572,2,0.1307511,0\C,5,1.39664012,4
,119.860743,3,-0.00162331,0\H,1,1.08686392,6,119.85366272,5,179.858219
64,0\H,2,1.08663169,1,120.20388312,6,179.57291229,0\H,3,1.08733433,2,1
19.69148136,1,178.83804915,0\H,5,1.08682076,4,120.9470633,3,179.437735
02,0\H,6,1.08659694,5,119.64108218,4,179.58270471,0\C,3,5.35527392,2,1
41.39563884,1,131.50104295,0\C,12,1.39794394,3,77.22875418,2,147.55961
999,0\C,13,1.39734646,12,120.09443236,3,27.61912859,0\C,14,1.40691516,
13,119.78800492,12,-0.15827118,0\C,15,1.40971924,14,119.94476351,13,-0
.02767846,0\C,16,1.39490055,15,119.78458609,14,0.17936863,0\H,12,1.086
86297,3,149.26935846,2,19.37603541,0\H,13,1.0865572,12,120.24556955,3,
-152.27270091,0\H,14,1.08639745,13,119.31838586,12,-179.84175152,0\H,1
6,1.08791707,15,120.58889706,14,-179.16270779,0\H,17,1.08662092,16,119
.71690533,15,-179.98841032,0\P,15,1.80770736,14,121.02014222,13,177.27
339239,0\C,23,1.82563308,15,108.94361562,14,-106.72762902,0\H,24,1.095
45013,23,108.5554929,15,55.2658519,0\H,24,1.09490739,23,110.26318241,1
5,-63.93283471,0\H,24,1.09512628,23,110.76334828,15,174.68907599,0\H,2
3,2.39525273,15,96.41110636,14,-11.33797179,0\C,23,1.83092245,15,111.0
1763546,14,10.87628113,0\C,29,1.5639429,23,120.4575178,15,69.41718198,
0\C,30,1.55598575,29,87.95243554,23,140.66563758,0\C,31,1.55618385,30,
89.34991203,29,-18.72664936,0\H,30,1.09568585,29,112.0724017,23,28.111
02178,0\H,30,1.09393574,29,116.64853577,23,-98.54282861,0\H,31,1.09321
688,30,116.93248956,29,-138.76887323,0\H,31,1.09466322,30,111.86297595
,29,94.56489748,0\H,32,1.09603271,31,111.41092327,30,-94.40538555,0\H,
32,1.09390236,31,118.22357546,30,137.75767013,0\\Version=AM64L-G03RevD
.01\State=1-A\HF=-995.9952307\MP2=-998.5518833\RMSD=4.100e-09\Thermal=
0.\PG=C01 [X(C17H20P1)]\\@

278-BH+

1\1\GINC-YIN\SP\RMP2-FC\6-31+G(2d,p)\C29H28P1(1+)\CHRISTOPH\04-Aug-201
0\0\\#p MP2(FC)/6-31+g(2d,p) scf=tight\\x4bhsp_4\\1,1\C\H,1,1.09843651
\C,1,4.35273367,2,106.23960483\C,3,1.39743736,1,60.22312363,2,148.7635
1338,0\C,4,1.39825456,3,120.47074869,1,-0.0117498,0\C,5,1.40381397,4,1
20.49692514,3,-0.12369606,0\C,6,1.40701883,5,118.6084234,4,-0.72430356
,0\C,7,1.39710554,6,120.81137454,5,1.03506408,0\H,3,1.08698644,1,179.4
6938782,6,-152.30528678,0\H,4,1.08715816,3,120.13100366,1,-179.5149246
4,0\H,5,1.08510702,4,119.12297611,3,-178.6738969,0\H,7,1.08752868,6,12
0.08714095,5,-178.48427845,0\H,8,1.08715404,7,119.6296077,6,-179.60030
325,0\C,1,4.34537693,6,116.77965431,5,31.14721679,0\C,14,1.39793355,1,
58.9712083,6,105.25766512,0\C,15,1.39753786,14,120.10600971,1,0.451047
81,0\C,16,1.40482569,15,120.83628307,14,-0.39851789,0\C,17,1.4069244,1
6,118.59226806,15,0.37689707,0\C,14,1.39790893,1,60.65014339,17,14.036
21667,0\H,14,1.08686556,1,179.08855131,17,168.2303174,0\H,15,1.0870349
3,14,120.21964815,1,-179.43871112,0\H,16,1.08885272,15,119.45901866,14
,-179.47288665,0\H,18,1.08766854,17,120.42531206,16,-179.94558874,0\H,
19,1.08721845,14,120.11747972,1,179.50930601,0\P,1,1.8896698,17,110.94
924006,16,-124.28793691,0\C,25,4.61460903,1,111.33812567,17,-83.047271

91,0\C,26,1.39917041,25,59.90232149,1,-65.6814219,0\C,27,1.39586479,26
,120.26657372,25,-0.29319433,0\C,28,1.41111318,27,119.83092043,26,-0.0
0726237,0\C,29,1.40876619,28,119.62576446,27,-0.12692168,0\C,30,1.3966
7624,29,120.00693466,28,0.13851309,0\H,27,1.08667549,26,120.22829667,2
5,179.6651503,0\H,28,1.08636212,27,119.58727445,26,-179.81347018,0\H,3
0,1.08581089,29,120.8594885,28,-179.69321602,0\H,31,1.08669583,30,119.
61693277,29,-179.79605045,0\C,25,4.62481376,1,111.17196702,17,154.5165
6449,0\C,36,1.39913558,25,60.1839991,1,-37.84405633,0\C,37,1.39592853,
36,120.11933312,25,1.60949001,0\C,38,1.41013583,37,120.24439972,36,0.2
2435524,0\C,39,1.40797573,38,119.25742695,37,-0.57464065,0\C,36,1.3974
5653,25,59.8344675,1,140.69678384,0\H,36,1.08686374,25,178.71335922,1,
-134.25399453,0\H,37,1.08674616,36,120.25274646,25,-178.47516436,0\H,3
8,1.0869733,37,118.70512154,36,179.90041643,0\H,40,1.08610265,39,120.3
8732514,38,-179.78822529,0\H,41,1.08672733,36,120.22237566,25,178.4741
6731,0\H,26,1.08698555,25,179.74812258,1,-13.87823592,0\C,25,1.8381915
3,1,108.37067415,17,38.06959206,0\C,48,1.56559707,25,119.41792218,1,60
.51411324,0\C,49,1.55630854,48,87.73893213,25,143.41209183,0\C,50,1.55
573497,49,89.24379198,48,-19.30610718,0\H,49,1.09349403,48,116.0982760
8,25,-95.6712134,0\H,49,1.09516578,48,112.60900964,25,31.61965551,0\H,
50,1.09479836,49,111.75935953,48,93.97841942,0\H,50,1.09351753,49,116.
93403145,48,-139.43391338,0\H,51,1.09560874,50,111.67938735,49,-93.633
5723,0\H,51,1.09427211,50,118.19706554,49,138.73956031,0\H,48,1.096017
35,25,106.77296311,1,-64.38741664,0\\Version=AM64L-G03RevD.01\State=1-
A\HF=-1455.107337\MP2=-1459.3640188\RMSD=6.913e-09\Thermal=0.\PG=C01 [
X(C29H28P1)]\\@

279

1\1\GINC-PHOBOS\SP\RMP2-FC\6-31+G(2d,p)\C12H19P1\CHRISTOPH\28-Jun-2011
\0\\#p MP2(FC)/6-31+g(2d,p) scf=tight\\yiprsp_1\\0,1\P\C,1,1.89959987\
H,2,1.10090407,1,103.17052166\C,1,1.88629487,2,103.1064135,3,69.661757
18,0\H,4,1.09973328,1,110.55334812,2,54.66286442,0\C,4,1.54173307,1,10
9.70874638,2,-64.4889381,0\H,6,1.09828544,4,111.07272993,1,-56.1973051
8,0\H,6,1.09669249,4,112.27666035,1,64.79637734,0\H,6,1.0986397,4,110.
15456469,1,-175.39833537,0\C,4,1.54142987,1,109.30726726,2,174.1225371
7,0\H,10,1.09791262,4,110.85297211,1,59.55046613,0\H,10,1.09880214,4,1
10.30719491,1,178.91363366,0\H,10,1.09614818,4,111.87277263,1,-60.8745
0803,0\C,2,1.54151912,1,109.01024127,4,-176.37376281,0\H,14,1.09702545
,2,111.58006109,1,-52.89963622,0\H,14,1.09763579,2,111.11401796,1,67.6
6284492,0\H,14,1.09861063,2,110.32880376,1,-173.01128098,0\C,2,1.53730
216,1,117.03347977,4,-49.39742962,0\H,18,1.09954051,2,110.13175485,1,1
77.46592636,0\H,18,1.09715806,2,111.16469485,1,-63.48367906,0\H,18,1.0
9692516,2,112.32304964,1,57.86220913,0\C,1,4.68157652,4,105.36713946,1
0,66.88839708,0\C,22,1.39867085,1,61.50019851,4,28.31639198,0\C,23,1.3
9827022,22,120.24501915,1,0.20525844,0\C,24,1.40858292,23,121.03150674
,22,-0.42977763,0\C,25,1.40836782,24,117.84211575,23,-0.26230268,0\C,2
2,1.39763675,1,58.0786996,25,-179.54010677,0\H,22,1.08785234,1,178.329
457,25,-175.69187235,0\H,23,1.08822623,22,120.04295823,1,-179.94552563
,0\H,24,1.08738433,23,118.80374477,22,179.90570755,0\H,26,1.08868175,2
5,119.10720469,24,-178.5388079,0\H,27,1.08801296,22,120.15276655,1,179
.75468935,0\\Version=AM64L-G03RevD.01\State=1-A\HF=-806.2535684\MP2=-8
08.1642982\RMSD=4.482e-09\Thermal=0.\PG=C01 [X(C12H19P1)]\\@

279-Me$^+$

1\1\GINC-EDDY\SP\RMP2-FC\6-31+G(2d,p)\C13H22P1(1+)\CHRISTOPH\19-Feb-20
10\0\\#p MP2(FC)/6-31+g(2d,p) scf=tight\\yiprmesp_9\\1,1\C\H,1,1.09913
215\C,1,3.0502802,2,88.86940122\H,3,1.10023748,1,87.10392887,2,-120.59
679316,0\C,3,1.54365323,1,145.70370552,2,119.34481604,0\H,5,1.09611273
,3,111.44653929,1,-63.14414382,0\H,5,1.09567535,3,112.73552238,1,59.48

023713,0\H,5,1.09594935,3,108.6746109,1,178.22767352,0\C,3,1.54282245,
1,90.05158056,5,-130.78532055,0\H,9,1.0960432,3,112.80145297,1,-30.762
38399,0\H,9,1.09621636,3,111.32624264,1,91.89874888,0\H,9,1.09584941,3
,108.77602835,1,-149.77819914,0\C,1,1.54417956,3,88.78857975,9,97.0451
9575,0\H,13,1.09545108,1,112.79061608,3,-32.73769164,0\H,13,1.0965358,
1,111.85924464,3,90.12016538,0\H,13,1.09591167,1,108.65361099,3,-151.7
3729604,0\C,1,1.54423629,13,111.9672564,3,-151.84391209,0\H,17,1.09632
351,1,111.55974661,13,56.95891422,0\H,17,1.09526978,1,112.4207759,13,1
79.38376994,0\H,17,1.09586734,1,108.75683311,13,-61.28185744,0\C,5,5.4
9794986,3,82.99841537,1,-43.27730843,0\C,21,1.39812321,5,56.48462919,3
,117.07555806,0\C,22,1.39677608,21,120.16273484,5,-36.57962803,0\C,23,
1.40831098,22,120.00143817,21,-0.02288344,0\C,24,1.41038916,23,119.535
57312,22,0.02558694,0\C,25,1.39569618,24,120.01938075,23,-0.04337668,0
\H,21,1.08677303,5,148.95199455,3,-149.46203699,0\H,22,1.08655222,21,1
20.24620824,5,143.6148059,0\H,23,1.08646737,22,119.0236926,21,-179.717
2823,0\H,25,1.08716588,24,120.93375932,23,-179.62318076,0\H,26,1.08656
96,25,119.64236135,24,-179.74713531,0\P,24,1.81012616,23,120.59386762,
22,178.39999962,0\C,32,1.82397399,24,109.37908333,23,20.68229257,0\H,3
3,1.09534915,32,109.20932477,24,176.20036118,0\H,33,1.09366888,32,111.
00753178,24,-64.45781117,0\H,33,1.09453441,32,110.19911979,24,56.40357
972,0\\Version=AM64L-G03RevD.01\State=1-A\HF=-845.7068486\MP2=-847.762
2352\RMSD=4.346e-09\Thermal=0.\PG=C01 [X(C13H22P1)]\\@

279-BH$^+$

1\1\GINC-CALYPSO\SP\RMP2-FC\6-31+G(2d,p)\C25H30P1(1+)\CHRISTOPH\04-Jul
-2011\0\\#p MP2(FC)/6-31+g(2d,p) scf=tight\\yiprbhsp_4\\1,1\C\H,1,1.09
903284\P,1,1.89135299,2,101.31614569\C,1,4.34953563,3,112.42990285,2,1
12.3999613,0\C,4,1.39836693,1,60.22299474,3,89.96380876,0\C,5,1.397772
82,4,120.4436214,1,-0.1808339,0\C,6,1.40485324,5,120.41614703,4,-0.087
62407,0\C,7,1.4067117,6,118.68076968,5,0.83341147,0\C,8,1.39773805,7,1
20.84440403,6,-0.94687714,0\H,4,1.08690083,1,179.35308087,7,150.728763
5,0\H,5,1.08713097,4,120.08574966,1,179.17512338,0\H,6,1.08591318,5,11
9.34920664,4,178.4535695,0\H,8,1.089201,7,119.9289114,6,177.46450085,0
\H,9,1.08703414,8,119.74349235,7,179.35411275,0\C,1,4.34744873,7,113.8
9890757,6,-44.0828312,0\C,15,1.39689177,1,60.83391214,7,83.97817124,0\
C,16,1.3995098,15,120.37369634,1,0.65372891,0\C,17,1.40494873,16,120.5
2062838,15,0.28723661,0\C,18,1.40624662,17,118.57199498,16,-0.33298426
,0\C,19,1.39652221,18,120.85589774,17,0.07124113,0\H,15,1.08677631,1,1
78.72300691,18,-177.55382946,0\H,16,1.08719939,15,120.12456303,1,-178.
98776758,0\H,17,1.08677177,16,118.90773066,15,-179.11502278,0\H,19,1.0
8897029,18,119.6848748,17,-179.0458877,0\H,20,1.08697228,19,119.654907
96,18,-179.68736687,0\C,3,1.86374171,1,108.50282923,18,-44.28511705,0\
H,26,1.0989809,3,102.69629048,1,71.40557849,0\C,3,1.86834316,1,106.462
00922,18,-167.29783604,0\H,28,1.10002758,3,101.97321163,1,-47.68081737
,0\C,28,1.54463631,3,113.44610072,1,67.9836732,0\H,30,1.09623288,28,10
8.38814368,3,-168.17763097,0\H,30,1.09559991,28,113.6051138,3,-48.9757
4568,0\H,30,1.09520367,28,111.52097395,3,73.9131016,0\C,28,1.54465933,
3,113.80848566,1,-164.01856434,0\H,34,1.09436885,28,112.01285679,3,-69
.35970418,0\H,34,1.09346622,28,112.68029907,3,53.24732079,0\H,34,1.096
53391,28,108.16591835,3,172.33359469,0\C,26,1.545189,3,112.86778972,1,
-171.2365325,0\H,38,1.09524305,26,111.23050194,3,74.32150517,0\H,38,1.
09640287,26,108.47031652,3,-167.91701102,0\H,38,1.09486393,26,113.3646
7441,3,-48.15071033,0\C,26,1.54520035,3,113.5058899,1,-43.67091222,0\H
,42,1.09399776,26,112.35876117,3,-66.36301051,0\H,42,1.09366486,26,112
.40905281,3,57.38047419,0\H,42,1.09647568,26,107.91841167,3,175.452091
26,0\C,3,4.62979783,1,111.3188138,18,76.73922769,0\C,46,1.39802787,3,6
0.44766723,1,-108.84198872,0\C,47,1.39669109,46,120.19736154,3,-0.0063
1309,0\C,48,1.40927959,47,120.26744826,46,-0.02167408,0\C,49,1.4107954

2,48,119.13503262,47,0.05939246,0\C,50,1.39630832,49,120.17518246,48,-
0.02091105,0\H,46,1.08684105,3,179.51525622,1,88.99206503,0\H,47,1.086
67077,46,120.27833569,3,-179.78831085,0\H,48,1.08577481,47,118.7043474
1,46,179.78496727,0\H,50,1.08601395,49,120.85440367,48,179.7803304,0\H
,51,1.08666955,50,119.49278665,49,-179.98497225,0\\Version=AM64L-G03Re
vD.01\State=1-A\HF=-1304.8136844\MP2=-1308.5677405\RMSD=9.073e-09\Ther
mal=0.\PG=C01 [X(C25H30P1)]\\@

279-TT$^+$

1\1\GINC-NODE17\SP\RMP2-FC\6-31+G(2d,p)\C31H34P1(1+)\ZIP07\12-Sep-2011
\0\\#p MP2(FC)/6-31+G(2d,p) scf=tight\\yiprttsp_8\\1,1\C\P,1,2.0216036
2\C,1,4.39471825,2,104.55623973\C,3,1.39541578,1,59.33656152,2,-120.79
418894,0\C,4,1.39919685,3,120.644098,1,0.19240664,0\C,5,1.40766042,4,1
21.21679421,3,-0.36578489,0\C,6,1.4118447,5,117.30538844,4,0.30223315,
0\C,7,1.39727976,6,121.44508845,5,-0.04296924,0\H,3,1.08687406,1,179.6
631583,6,123.48058849,0\H,4,1.08712241,3,120.20899574,1,-179.65161993,
0\H,5,1.08413744,4,118.45505532,3,-179.77040914,0\H,7,1.08474107,6,120
.26645746,5,-179.32539955,0\H,8,1.08743889,7,119.39060977,6,-179.54493
551,0\C,1,4.39128293,6,115.17330083,5,121.66742262,0\C,14,1.39442576,1
,60.27350107,6,8.26734747,0\C,15,1.40079857,14,120.76613209,1,0.388937
44,0\C,16,1.40428856,15,120.99359824,14,0.52837994,0\C,17,1.4103416,16
,117.40337048,15,-2.44248797,0\C,18,1.39491389,17,121.63050421,16,2.60
446662,0\H,14,1.08697636,1,179.04762944,6,-137.7359041,0\H,15,1.087177
45,14,120.17912956,1,-178.75313839,0\H,16,1.08273191,15,118.1211381,14
,-177.52768403,0\H,18,1.08367148,17,119.75059059,16,-174.85424122,0\H,
19,1.08749547,18,119.530194,17,-179.62087743,0\C,1,4.37966732,6,107.86
891616,5,3.467272,0\C,25,1.39466709,1,60.97844198,6,-98.7414868,0\C,26
,1.40080487,25,120.22314791,1,-1.05085411,0\C,27,1.40110706,26,121.449
00678,25,-0.73452396,0\C,28,1.41045659,27,117.57230437,26,0.91293617,0
\C,29,1.39531626,28,121.15057885,27,-0.42850496,0\H,25,1.08677288,1,17
8.32577556,28,-173.35577533,0\H,26,1.08717701,25,120.34809882,1,178.86
621297,0\H,27,1.08357229,26,117.76479508,25,179.37906517,0\H,29,1.0859
6782,28,119.71611096,27,179.16591187,0\H,30,1.08708976,29,119.33770991
,28,179.90296833,0\C,2,4.64712216,1,107.59735579,28,153.6115295,0\C,36
,1.39778699,2,59.4227328,1,-76.83431909,0\C,37,1.39595032,36,120.26160
791,2,0.24490033,0\C,38,1.40963511,37,120.52427646,36,0.14612739,0\C,3
9,1.40946021,38,118.69517633,37,-0.12351021,0\C,40,1.39694612,39,120.4
8606341,38,0.04029572,0\H,36,1.08687935,2,179.47053634,1,-111.44330293
,0\H,37,1.0867046,36,120.29305282,2,-179.88776479,0\H,38,1.08496448,37
,118.80352322,36,179.07666914,0\H,40,1.08517687,39,121.02056198,38,179
.53188699,0\H,41,1.08684641,40,119.46357649,39,-179.90451495,0\C,2,1.8
8856519,1,110.05023081,28,-89.83620958,0\H,47,1.09641023,2,102.7036787
3,1,-59.2699692,0\C,2,1.90633207,1,119.72743061,28,32.34885295,0\H,49,
1.09912903,2,101.25099622,1,-91.73995163,0\C,47,1.54683836,2,113.78725
861,1,-176.76949925,0\H,51,1.09692143,47,107.70294689,2,169.00442695,0
\H,51,1.0956009,47,111.49412475,2,-73.79465038,0\H,51,1.09326752,47,11
3.97029314,2,49.68495138,0\C,47,1.54254224,2,114.85068636,1,56.7115943
3,0\H,55,1.09663928,47,107.66580898,2,-173.54961892,0\H,55,1.09138794,
47,113.1308251,2,-54.81599666,0\H,55,1.09695717,47,112.03045408,2,69.2
5203151,0\C,49,1.54869408,2,111.20396058,1,153.61773868,0\H,59,1.09706
121,49,107.59417612,2,164.08718785,0\H,59,1.09396222,49,111.17206239,2
,-78.01524818,0\H,59,1.09103831,49,114.19956903,2,44.41673531,0\C,49,1
.54025668,2,118.8336223,1,26.17028151,0\H,63,1.09716919,49,107.5257836
,2,-174.55030597,0\H,63,1.09182443,49,113.31538712,2,-55.75669895,0\H,
63,1.09469278,49,111.65555764,2,68.23035057,0\\Version=AM64L-G03RevD.0
1\State=1-A\HF=-1534.3231781\MP2=-1538.9547482\RMSD=7.376e-09\Thermal=
0.\PG=C01 [X(C31H34P1)]\\@

280

1\1\GINC-CIPCLU04\SP\RMP2-FC\6-31+G(2d,p)\C14H23P1\C2175\23-Dec-2010\0
\\#p MP2(FC)/6-31+G(2d,p) scf=tight\\ybusp_1\\0,1\P\C,1,4.67869806\C,2
,1.40002956,1,61.04042197\C,3,1.39701078,2,120.09373563,1,0.000552,0\C
,4,1.40778652,3,120.94955401,2,0.,0\C,5,1.40581282,4,118.18682394,3,0.
0002753,0\C,2,1.39695779,1,58.69005138,5,179.97364118,0\H,2,1.08793425
,1,178.88830008,5,-179.99542984,0\H,3,1.08824405,2,120.01785551,1,-179
.99953544,0\H,4,1.08879586,3,119.02680478,2,180.,0\H,6,1.08850493,5,11
9.11827114,4,179.99917918,0\H,7,1.08798495,2,120.14506263,1,179.999381
5,0\C,1,1.87652803,5,101.2535967,4,50.88076239,0\H,13,1.10066929,1,110
.89680514,5,-51.95381469,0\H,13,1.10075986,1,106.22112913,5,-167.46347
057,0\C,1,1.87652102,5,101.25550747,4,-50.85625107,0\H,16,1.10066883,1
,110.89855173,5,51.95806786,0\H,16,1.10075976,1,106.22015876,5,167.467
53615,0\C,16,1.539084,1,113.03379107,5,-72.22646172,0\H,19,1.10032774,
16,109.39235363,1,-55.94432809,0\H,19,1.10011089,16,109.41620583,1,60.
15156363,0\C,13,1.53908197,1,113.03400837,5,72.22957024,0\H,22,1.10011
115,13,109.4162316,1,-60.1492986,0\H,22,1.10032729,13,109.39253438,1,5
5.94663517,0\C,19,1.5387844,16,112.92674725,1,-177.99851848,0\H,25,1.1
0059856,19,109.2656942,16,57.7652334,0\H,25,1.10048635,19,109.22416599
,16,-58.02383219,0\C,22,1.53878522,13,112.92670118,1,178.00083539,0\H,
28,1.10059836,22,109.26575714,13,-57.76619336,0\H,28,1.10048623,22,109
.22404993,13,58.02291626,0\C,28,1.53572039,22,112.89799529,13,-179.909
82021,0\H,31,1.09719194,28,111.37374748,22,-179.83976823,0\H,31,1.0980
6635,28,111.07673747,22,-59.69776714,0\H,31,1.09806701,28,111.09241664
,22,60.02127385,0\C,25,1.53572001,19,112.89797868,16,179.90882515,0\H,
35,1.0980662,25,111.07681874,19,59.69785717,0\H,35,1.09806609,25,111.0
9241618,19,-60.02127569,0\H,35,1.09719244,25,111.37374719,19,179.83982
364,0\\Version=AM64L-G03RevD.01\State=1-A\HF=-884.3411517\MP2=-886.545
5693\RMSD=6.459e-09\Thermal=0.\PG=C01 [X(C14H23P1)]\\@

280-Me⁺

1\1\GINC-CIPCLU07\SP\RMP2-FC\6-31+G(2d,p)\C15H26P1(1+)\C2175\03-Jan-20
11\0\\#p MP2(FC)/6-31+G(2d,p) scf=tight\\ybumesp_1\\1,1\C\C,1,1.400220
4\C,2,1.39471521,1,120.02007226\C,3,1.40995498,2,119.9101732,1,0.00073
44,0\C,4,1.40639915,3,119.8547705,2,-0.00249309,0\C,5,1.39735195,4,119
.79199618,3,0.00240794,0\H,1,1.08679019,6,119.89402663,5,179.99863542,
0\H,2,1.08653999,1,120.24014785,6,-180.,0\H,3,1.08854974,2,119.2337386
1,1,179.99930297,0\H,5,1.08651438,4,120.95190676,3,179.99910058,0\H,6,
1.08653262,5,119.61570896,4,179.99797836,0\C,4,2.98557355,3,97.8859762
9,2,-149.79058501,0\H,12,1.09946926,4,89.38277227,3,-8.59024859,0\H,12
,1.09977578,4,141.31561062,3,-124.93194019,0\C,12,2.97083606,4,60.1627
3444,3,-94.42894243,0\H,15,1.09946334,12,86.0758084,4,91.57023206,0\H,
15,1.09977829,12,85.48954596,4,-161.41497082,0\C,15,1.54445082,12,150.
83094807,4,-34.831955,0\H,18,1.09915605,15,109.94624217,12,-60.6596678
7,0\H,18,1.09864769,15,109.63924377,12,56.6770093,0\C,12,1.5444481,4,9
5.02354878,3,101.81763492,0\H,21,1.09865125,12,109.64008069,4,-26.8211
1129,0\H,21,1.09915597,12,109.94636275,4,90.5176665,0\C,18,1.54070645,
15,111.48108312,12,178.15654871,0\H,24,1.0995494,18,109.28344558,15,57
.79539568,0\H,24,1.09958278,18,109.13539408,15,-58.3126611,0\C,21,1.54
070947,12,111.48130652,4,-148.29990534,0\H,27,1.09954863,21,109.283309
02,12,-57.7901107,0\H,27,1.09958023,21,109.1350361,12,58.31824596,0\C,
27,1.53504532,21,112.30418934,12,-179.79535099,0\H,30,1.09546237,27,11
0.68124622,21,-179.77954871,0\H,30,1.09729743,27,111.27920387,21,-59.9
9160511,0\H,30,1.09732039,27,111.30038675,21,60.47009261,0\C,24,1.5350
4538,18,112.30426816,15,179.80092652,0\H,34,1.09729818,24,111.27909926
,18,59.99283784,0\H,34,1.09732092,24,111.30017869,18,-60.46833998,0\H,
34,1.0954613,24,110.68140338,18,179.7809814,0\P,4,1.80548024,3,118.608
19091,2,-179.9840333,0\C,38,1.81963797,4,110.3657543,3,-179.87198981,0

\H,39,1.09605675,38,108.75024737,4,179.94938421,0\H,39,1.09450317,38,1
10.70689882,4,-60.70686269,0\H,39,1.09450558,38,110.70263367,4,60.6027
4601,0\\Version=AM64L-G03RevD.01\State=1-A\HF=-923.7940772\MP2=-926.14
34586\RMSD=4.442e-09\Thermal=0.\PG=C01 [X(C15H26P1)]\\@

280-BH$^+$

1\1\GINC-NODE25\SP\RMP2-FC\6-31+G(2d,p)\C27H34P1(1+)\ZIP07\13-Mar-2011
\0\\#p MP2(FC)/6-31+G(2d,p) scf=tight\\ybubhsp_1\\1,1\C\H,1,1.09998926
\C,1,4.34466925,2,105.42384223\C,3,1.39811195,1,58.88526521,2,16.30653
858,0\C,4,1.39703773,3,120.11799018,1,-0.55685668,0\C,5,1.4053141,4,12
0.80078229,3,0.41509737,0\C,6,1.4063135,5,118.63658588,4,-0.35636587,0
\C,3,1.39753786,1,60.73587494,6,-17.35812919,0\H,3,1.08682096,1,178.97
698611,6,-171.82432644,0\H,4,1.08700841,3,120.21570785,1,179.37537564,
0\H,5,1.0888301,4,119.47192253,3,179.38173448,0\H,7,1.08736678,6,120.5
5668443,5,179.87356042,0\H,8,1.08716737,3,120.13609543,1,-179.39781898
,0\C,1,4.34796543,6,117.64513477,5,-101.44180915,0\C,14,1.39833062,1,5
9.31829205,6,140.71742312,0\C,15,1.39745693,14,119.97455969,1,-0.18854
661,0\C,16,1.40665475,15,120.81466741,14,0.37269234,0\C,17,1.40398027,
16,118.74433507,15,-0.846783,0\C,18,1.39799383,17,120.38337161,16,0.61
550458,0\H,14,1.08691936,1,179.38432031,6,107.62886413,0\H,15,1.087082
2,14,120.28309547,1,-179.17489964,0\H,16,1.0892553,15,119.27385338,14,
-178.19784323,0\H,18,1.08561679,17,120.27626746,16,-177.92181188,0\H,1
9,1.0871144,18,119.45983079,17,-179.34706222,0\P,1,1.8798617,6,112.258
81447,5,126.97380436,0\C,25,4.61496367,1,113.99753273,6,78.51342145,0\
C,26,1.39889164,25,59.76108291,1,69.39765795,0\C,27,1.3959634,26,120.2
2091199,25,-0.33031667,0\C,28,1.41038288,27,119.91044713,26,-0.0518014
9,0\C,29,1.40862665,28,119.58359402,27,0.11832943,0\C,30,1.39659502,29
,120.01204194,28,-0.10700101,0\H,26,1.08691237,25,179.48984395,1,123.6
4953284,0\H,27,1.08666027,26,120.22572373,25,179.62209698,0\H,28,1.086
47687,27,119.22165785,26,-179.63887896,0\H,30,1.08629131,29,120.897766
39,28,179.84089723,0\H,31,1.08666408,30,119.6082578,29,179.78866547,0\
C,25,1.83440588,1,108.74977049,6,-44.68851174,0\H,37,1.09731952,25,107
.09985515,1,61.70728257,0\H,37,1.0984386,25,107.05973742,1,176.8039319
3,0\C,25,1.8361776,1,107.58114214,6,-162.53314149,0\H,40,1.09770827,25
,107.01370855,1,-60.55276151,0\H,40,1.09897546,25,107.06438575,1,53.27
444997,0\C,40,1.54416885,25,114.36219556,1,176.3323014,0\H,43,1.099356
98,40,109.89385554,25,-60.86538551,0\H,43,1.09853983,40,109.70552899,2
5,56.30171455,0\C,37,1.54535242,25,114.50651379,1,-60.32970997,0\H,46,
1.09918942,37,110.27775078,25,-58.66873732,0\H,46,1.09861745,37,109.81
189424,25,59.28754409,0\C,43,1.54048197,40,111.59504705,25,177.8178221
9,0\H,49,1.09961211,43,109.12934052,40,-58.53237732,0\H,49,1.09975333,
43,109.32288904,40,57.53374821,0\C,46,1.54090233,37,111.30032132,25,-1
79.9346489,0\H,52,1.09932741,46,109.07233582,37,-58.22784042,0\H,52,1.
0996477,46,109.21285189,37,57.86262225,0\C,52,1.53502757,46,112.336405
34,37,179.84502694,0\H,55,1.09557109,52,110.74991829,46,179.94471519,0
\H,55,1.09741028,52,111.29347909,46,-60.25518295,0\H,55,1.09738009,52,
111.2498593,46,60.16835788,0\C,49,1.53507148,43,112.35557212,40,179.54
671111,0\H,59,1.09737288,49,111.27155367,43,59.90148178,0\H,59,1.09561
041,49,110.76720683,43,179.74244598,0\H,59,1.09742066,49,111.28086032,
43,-60.45750015,0\\Version=AM64L-G03RevD.01\State=1-A\HF=-1382.910843\
MP2=-1386.9556612\RMSD=9.082e-09\Thermal=0.\PG=C01 [X(C27H34P1)]\\@

280-TT$^+$

1\1\GINC-NODE12\SP\RMP2-FC\6-31+G(2d,p)\C33H38P1(1+)\ZIP07\19-Sep-2011
\0\\#p MP2(FC)/6-31+G(2d,p) scf=tight\\ybuttsp_19\\1,1\C\P,1,1.9638031
1\C,1,4.39029651,2,107.40497145\C,3,1.39595664,1,59.31382884,2,125.176
1904,0\C,4,1.39880282,3,120.62319012,1,-0.02626396,0\C,5,1.40691573,4,
121.07200453,3,0.0980137,0\C,6,1.41055366,5,117.58138341,4,0.46852713,

0\C,3,1.39722488,1,59.77746621,6,7.28601221,0\H,3,1.08694273,1,179.784
81437,6,-157.95107245,0\H,4,1.08718631,3,120.19106547,1,179.82292173,0
\H,5,1.08395922,4,118.54763953,3,179.98156071,0\H,7,1.08430907,6,120.5
3426419,5,178.46309954,0\H,8,1.08743541,3,120.29360865,1,-179.64367156
,0\C,1,4.38344475,6,108.08845841,5,2.84810107,0\C,14,1.39542966,1,60.5
1370004,6,88.55866122,0\C,15,1.39939062,14,120.25196823,1,1.06685928,0
\C,16,1.40441584,15,121.41979998,14,1.19760747,0\C,17,1.40868198,16,11
7.53920777,15,-2.02578631,0\C,18,1.39666294,17,121.12947786,16,1.35654
258,0\H,14,1.0867946,1,178.58695583,17,171.52367217,0\H,15,1.08719629,
14,120.32394573,1,-178.32164517,0\H,16,1.08396786,15,117.61481312,14,-
177.24933124,0\H,18,1.0855339,17,119.89812287,16,-178.10910253,0\H,19,
1.0871272,18,119.28108847,17,-179.84487289,0\C,1,4.38990975,17,108.757
38619,16,-148.75393953,0\C,25,1.39493993,1,60.11268188,17,-131.7016053
2,0\C,26,1.40065157,25,120.65863625,1,0.15410779,0\C,27,1.40377381,26,
120.96846321,25,-0.13104389,0\C,28,1.41242991,27,117.63894084,26,2.195
3182,0\C,29,1.39569852,28,121.35637993,27,-2.65853216,0\H,25,1.0869827
9,1,179.3809364,17,63.33173581,0\H,26,1.0871464,25,120.18853457,1,179.
66145531,0\H,27,1.08341996,26,118.54322812,25,178.55999033,0\H,29,1.08
588668,28,119.93094731,27,174.37719383,0\H,30,1.08737027,29,119.531947
76,28,179.72806013,0\C,2,4.63464135,1,112.98318541,17,-166.67342265,0\
C,36,1.39822958,2,60.11055332,1,-111.1790153,0\C,37,1.39627234,36,120.
16525113,2,0.94687639,0\C,38,1.40942915,37,120.29421134,36,0.00184371,
0\C,39,1.40976507,38,119.18224111,37,-0.01638848,0\C,40,1.39661194,39,
120.08311179,38,0.0370659,0\H,36,1.08696461,2,179.0534648,1,149.996431
32,0\H,37,1.08678847,36,120.28241053,2,-178.94066636,0\H,38,1.0856771,
37,118.78934039,36,179.46307138,0\H,40,1.08565331,39,120.89594902,38,1
79.49394953,0\H,41,1.08677535,40,119.41429658,39,-179.96466784,0\C,2,1
.84703603,1,110.73469146,17,-49.99284138,0\H,47,1.09770384,2,107.61170
935,1,49.87767833,0\C,2,1.85778704,1,116.368398,17,69.25867655,0\H,49,
1.09716121,2,105.59804112,1,-86.97197057,0\C,47,1.54606997,2,114.35674
984,1,173.61977701,0\H,51,1.09810506,47,110.30018893,2,-65.6728338,0\H
,51,1.09897838,47,110.05114684,2,52.15238112,0\C,49,1.54263253,2,121.0
8714656,1,38.33303583,0\H,54,1.09640938,49,109.82242299,2,-65.12938932
,0\H,54,1.09680417,49,110.95451697,2,51.63603571,0\C,51,1.54115697,47,
111.14644403,2,173.36658391,0\H,57,1.09961493,51,109.17388326,47,-59.4
9156166,0\H,57,1.09967663,51,109.31291938,47,56.6182527,0\C,57,1.53520
047,51,112.31815963,47,178.61251251,0\H,60,1.09750531,57,111.26929712,
51,-59.97471006,0\H,60,1.09741226,57,111.26581931,51,60.36929742,0\H,6
0,1.09573721,57,110.79737019,51,-179.77128409,0\C,54,1.54047668,49,110
.7103678,2,173.68263428,0\H,64,1.10035586,54,109.47122063,49,-59.58239
51,0\H,64,1.1001494,54,109.45124623,49,56.55131721,0\C,64,1.5353907,54
,112.18361643,49,178.45532638,0\H,67,1.09728034,64,111.17068224,54,-60
.15051302,0\H,67,1.0974495,64,111.21916046,54,59.95099639,0\H,67,1.095
70344,64,110.87627767,54,179.90566365,0\H,47,1.09507469,2,106.91758833
,1,-64.94506855,0\H,49,1.09900757,2,103.77619383,1,162.57476025,0\\Ver
sion=AM64L-G03RevD.01\State=1-A\HF=-1612.432341\MP2=-1617.3522679\RMSD
=9.860e-09\Thermal=0.\PG=C01 [X(C33H38P1)]\\@

281

1\1\GINC-LX64I155\SP\RMP2-FC\6-31+G(2d,p)\C16H19P1\UI271AC\06-Jul-2010
\0\\#MP2(FC)/6-31+g(2d,p) scf=tight\\xtbusp_1\\0,1\P\C,1,4.68464634\C,
2,1.3990781,1,57.9207736\C,3,1.39542561,2,120.30372973,1,2.50501143,0\
C,4,1.41236889,3,121.41975781,2,0.60828645,0\C,5,1.40706965,4,117.3958
3129,3,-1.26172015,0\C,2,1.39574886,1,61.2671496,5,52.78019417,0\H,2,1
.08761587,1,177.19130563,5,-179.2991887,0\H,3,1.08810694,2,120.1710295
7,1,-177.31198523,0\H,4,1.08799367,3,118.99135038,2,-179.70317119,0\H,
6,1.08460799,5,120.60508098,4,-178.87761401,0\H,7,1.08816653,2,120.142
63653,1,177.49254627,0\C,1,4.67714545,5,103.16854285,4,-50.90748537,0\

C,13,1.39949578,1,61.28781972,5,-33.50262832,0\C,14,1.39749064,13,120.
23552914,1,0.43617446,0\C,15,1.4086599,14,120.79379981,13,0.07661105,0
\C,16,1.40726468,15,118.22770552,14,0.64832304,0\C,13,1.39742361,1,58.
41020625,16,163.8624848,0\H,13,1.08789874,1,178.54962855,16,179.134717
32,0\H,14,1.08816878,13,120.04510113,1,-179.54649489,0\H,15,1.0871521,
14,119.5340652,13,179.03053135,0\H,17,1.0885542,16,119.26346884,15,178
.78973032,0\H,18,1.08797508,13,120.15791223,1,179.76357158,0\C,1,1.921
44401,16,102.25249913,15,77.52865831,0\C,24,1.54800016,1,107.78999274,
16,173.7774235,0\H,25,1.09713802,24,111.03190987,1,-53.73306101,0\H,25
,1.09964989,24,109.44960115,1,-172.7859232,0\H,25,1.09507051,24,113.25
453144,1,67.49696103,0\C,24,1.5405798,1,115.64656764,16,-61.25506848,0
\H,29,1.09634057,24,111.10260746,1,60.74322822,0\H,29,1.09547992,24,11
2.70843915,1,-60.59447749,0\H,29,1.10043548,24,109.66920353,1,179.5204
1694,0\C,24,1.54758151,1,105.59952209,16,59.20512661,0\H,33,1.09944405
,24,109.24752056,1,172.53015064,0\H,33,1.09544665,24,111.96882702,1,-6
7.9839627,0\H,33,1.0981132,24,111.6275393,1,53.52769356,0\\Version=IA6
4L-G03RevE.01\State=1-A\HF=-957.7306223\MP2=-960.1775523\RMSD=4.872e-0
9\Thermal=0.\PG=C01 [X(C16H19P1)]\\@

281-Me$^+$

1\1\GINC-LX64I165\SP\RMP2-FC\6-31+G(2d,p)\C17H22P1(1+)\UI271AC\06-Jul-
2010\0\\#MP2(FC)/6-31+g(2d,p) scf=tight\\xtbumesp_2\\1,1\C\C,1,1.39886
746\C,2,1.39532171,1,120.12858638\C,3,1.41122977,2,120.176329,1,-0.140
96777,0\C,4,1.40747305,3,119.33841248,2,-0.16581846,0\C,5,1.39717515,4
,120.03631768,3,0.43328067,0\H,1,1.08678859,6,119.98702036,5,-179.9672
7875,0\H,2,1.08664495,1,120.28975734,6,179.93137227,0\H,3,1.08733322,2
,119.27760181,1,179.1833582,0\H,5,1.08404761,4,121.25892914,3,-179.852
08004,0\H,6,1.0866071,5,119.45794016,4,179.57701883,0\C,3,5.29129129,2
,138.51281249,1,130.15061427,0\C,12,1.39933273,3,48.93377213,2,-62.531
18376,0\C,13,1.3959293,12,120.14630835,3,-35.47598463,0\C,14,1.4096395
6,13,119.92539846,12,-0.15504962,0\C,15,1.40787906,14,119.673244,13,0.
1583991,0\C,16,1.39685757,15,119.94671868,14,0.0374664,0\H,12,1.086844
05,3,149.11707784,2,17.81083705,0\H,13,1.08666537,12,120.21913806,3,14
4.14941274,0\H,14,1.08725252,13,119.56053618,12,178.63168745,0\H,16,1.
08684409,15,121.00279644,14,179.8247383,0\H,17,1.0866239,16,119.611899
41,15,179.60803993,0\C,15,3.02349608,14,109.61231307,13,-139.1080555,0
\C,23,1.55040408,15,83.53250169,14,112.46915565,0\H,24,1.09693506,23,1
07.89286972,15,-156.00162586,0\H,24,1.09454505,23,112.32957182,15,-37.
26229125,0\H,24,1.09670727,23,112.63928309,15,86.0058836,0\C,23,1.5487
4525,15,143.58096705,14,-134.60214936,0\H,28,1.09697039,23,108.0370082
1,15,-167.93149596,0\H,28,1.09401548,23,113.71471702,15,73.05927816,0\
H,28,1.0959401,23,111.98926705,15,-50.09485263,0\C,23,1.54657093,15,95
.45347284,14,3.87779423,0\H,32,1.0969542,23,108.32926077,15,145.205581
93,0\H,32,1.09607064,23,111.63429133,15,27.13758805,0\H,32,1.09440977,
23,113.06469704,15,-95.51453755,0\P,15,1.81344948,14,119.53609732,13,-
177.25396325,0\C,36,1.82888182,15,108.59010042,14,-158.76170896,0\H,37
,1.09440226,36,111.78897472,15,-68.2286196,0\H,37,1.09459327,36,108.84
312703,15,171.22715432,0\H,37,1.09498046,36,109.14015922,15,52.9008507
6,0\\Version=IA64L-G03RevE.01\State=1-A\HF=-997.1848768\MP2=-999.77577
96\RMSD=6.476e-09\Thermal=0.\PG=C01 [X(C17H22P1)]\\@

281-BH$^+$

1\1\GINC-GOLEM\SP\RMP2-FC\6-31+G(2d,p)\C29H30P1(1+)\CHRISTOPH\16-Jul-2
010\0\\#p MP2(FC)/6-31+g(2d,p) scf=tight\\xtbubhsp_6\\1,1\C\H,1,1.0977
661\C,1,4.34574877,2,104.99696783\C,3,1.39812284,1,58.83742888,2,13.66
25456,0\C,4,1.3974345,3,120.07071651,1,-0.59257968,0\C,5,1.40535522,4,
120.86344667,3,0.01638264,0\C,6,1.40557465,5,118.59015352,4,0.14126478
,0\C,3,1.39777878,1,60.73725817,6,-25.97901936,0\H,3,1.08682648,1,178.

90647067,6,-176.77174553,0\H,4,1.087022,3,120.24670681,1,179.20110446,
0\H,5,1.08899551,4,119.38779382,3,179.22890845,0\H,7,1.08664547,6,120.
59870238,5,-179.36503774,0\H,8,1.08720541,3,120.0871235,1,-179.4169296
7,0\C,1,4.35554013,6,114.47337095,5,-100.85033047,0\C,14,1.39744769,1,
59.26804993,6,134.19148278,0\C,15,1.39696798,14,120.08459317,1,0.57464
915,0\C,16,1.40602756,15,121.00385253,14,0.32980734,0\C,17,1.40575798,
16,118.34637698,15,-1.11644342,0\C,18,1.39735041,17,120.64090499,16,1.
06494785,0\H,14,1.08696691,1,179.00113223,6,73.34144198,0\H,15,1.08702
541,14,120.28046765,1,-178.62556447,0\H,16,1.08675216,15,118.95069919,
14,-178.67569478,0\H,18,1.08467684,17,120.44654877,16,-177.44762438,0\
H,19,1.08717454,18,119.43359993,17,-179.49942785,0\P,1,1.90246246,6,11
3.15657979,5,125.52698656,0\C,25,1.90978008,1,111.59274522,6,-51.32122
589,0\C,26,1.54957601,25,107.58850139,1,-175.34008802,0\H,27,1.0947923
6,26,112.92167008,25,-63.53595893,0\H,27,1.09513642,26,112.1233054,25,
59.34610961,0\H,27,1.0972562,26,107.91707235,25,177.68336462,0\C,26,1.
54941541,25,114.54544459,1,61.46054788,0\H,31,1.09204922,26,112.038398
91,25,-67.74978891,0\H,31,1.09764893,26,107.09348584,25,174.1650745,0\
H,31,1.09233275,26,114.30309226,25,55.39805239,0\C,26,1.54933568,25,10
7.60045512,1,-58.0375436,0\H,35,1.09669528,26,112.21211819,25,63.33364
692,0\H,35,1.09719891,26,107.66789554,25,-179.12625833,0\H,35,1.093963
3,26,112.72699892,25,-60.84829471,0\C,25,4.64114997,1,109.15973897,6,-
165.73902861,0\C,39,1.3973769,25,59.71512506,1,-142.35847588,0\C,40,1.
39725683,39,120.29144316,25,1.41752492,0\C,41,1.40886265,40,120.323243
6,39,0.18974263,0\C,42,1.40925049,41,118.88348737,40,-0.88612426,0\C,4
3,1.3961057,42,120.48942448,41,0.98163905,0\H,39,1.08684014,25,178.911
15195,1,136.8698276,0\H,40,1.08676103,39,120.24596412,25,-178.57010488
,0\H,41,1.08576865,40,119.21169878,39,-179.99667254,0\H,43,1.08601057,
42,120.92942798,41,-178.57907008,0\H,44,1.08676455,43,119.58443427,42,
179.6696531,0\C,25,4.63492546,1,109.47295375,6,78.51189488,0\C,50,1.39
815108,25,58.80987152,1,68.01700104,0\C,51,1.39620463,50,120.28722704,
25,0.56138736,0\C,52,1.41266715,51,120.24590898,50,-0.14084476,0\C,53,
1.40875168,52,118.98497209,51,0.33982414,0\C,54,1.39685393,53,120.3187
1679,52,-0.23370698,0\H,50,1.0869311,25,178.84077084,1,51.0800732,0\H,
51,1.08672796,50,120.31368682,25,-179.60358456,0\H,52,1.08537664,51,11
9.21880252,50,179.36739724,0\H,54,1.08290614,53,121.09907268,52,179.41
63688,0\H,55,1.08670504,54,119.42810511,53,179.6977246,0\\Version=AM64
L-G03RevD.01\State=1-A\HF=-1456.2875593\MP2=-1460.5824187\RMSD=9.586e-
09\Thermal=0.\PG=C01 [X(C29H30P1)]\\@

281-TT$^+$

1\1\GINC-LIEBIG\SP\RMP2-FC\6-31+G(2d,p)\C35H34P1(1+)\CHRISTOPH\26-Apr-
2012\0\\#p MP2(FC)/6-31+g(2d,p) scf=tight\\xtbuttsp_8\\1,1\C\C,1,4.384
09583\C,2,1.39939249,1,59.37797685\C,3,1.3953832,2,120.0310105,1,0.180
27345,0\C,4,1.40860521,3,121.82776906,2,1.11789685,0\C,5,1.40617832,4,
117.27139186,3,-2.91014789,0\C,2,1.39517641,1,59.65635786,5,66.2455981
1,0\H,2,1.08675432,1,179.72211935,5,-159.3237254,0\H,3,1.08722601,2,12
0.37072249,1,-178.9556075,0\H,4,1.08362136,3,118.63577879,2,-176.73179
811,0\H,6,1.08325488,5,120.44003219,4,-176.87647212,0\H,7,1.08701809,2
,120.18841549,1,179.13845614,0\C,1,4.38575143,5,109.30142515,4,-176.26
042617,0\C,13,1.39487592,1,59.49600179,5,-117.29143387,0\C,14,1.399562
86,13,120.67044495,1,-1.3373316,0\C,15,1.40667501,14,121.17061467,13,-
0.22567889,0\C,16,1.41178879,15,117.30175902,14,1.73703018,0\C,17,1.39
580101,16,121.50236544,15,-2.02492213,0\H,13,1.08689949,1,178.82716561
,5,-26.4138227,0\H,14,1.0870153,13,120.21962118,1,178.16985435,0\H,15,
1.08310486,14,118.31871762,13,178.34795029,0\H,17,1.08440766,16,120.24
159218,15,176.4678585,0\H,18,1.08734395,17,119.38597608,16,179.9061441
3,0\C,1,4.39250811,5,111.75563023,4,59.60757496,0\C,24,1.39533048,1,59
.56567892,5,4.76273437,0\C,25,1.39940184,24,120.68703875,1,-0.37962202

,0\C,26,1.40788538,25,121.22491036,24,-0.38961633,0\C,27,1.41029093,26
,117.13499387,25,2.96891113,0\C,28,1.39632843,27,121.604903,26,-3.4582
0953,0\H,24,1.08681116,1,179.8637856,5,143.07074744,0\H,25,1.08705166,
24,120.21812689,1,179.11874671,0\H,26,1.08293652,25,118.16224174,24,17
8.5768513,0\H,28,1.08226311,27,119.98543772,26,173.91772262,0\H,29,1.0
8744173,28,119.35951787,27,-179.85741597,0\P,1,2.08170483,5,109.443420
48,4,-59.63649354,0\C,35,4.66204924,1,105.64786548,5,-164.89874203,0\C
,36,1.39669962,35,59.35568344,1,82.38434896,0\C,37,1.39716839,36,120.3
7508063,35,0.65021843,0\C,38,1.40988769,37,120.64951591,36,0.11392327,
0\C,39,1.41064855,38,118.31692161,37,-0.8907643,0\C,40,1.39568566,39,1
20.78815392,38,1.11938137,0\H,36,1.08682891,35,179.46626221,1,41.30100
217,0\H,37,1.08667946,36,120.32223421,35,-179.26787286,0\H,38,1.083560
79,37,118.83170929,36,-179.93590085,0\H,40,1.08360497,39,120.76202876,
38,-177.46761976,0\H,41,1.08676097,40,119.42638352,39,179.56196283,0\C
,35,4.65685363,1,115.08318197,5,-48.04693035,0\C,47,1.3971478,35,59.33
497576,1,-142.67485535,0\C,48,1.39752665,47,120.32954511,35,2.29914129
,0\C,49,1.41089571,48,120.49152279,47,-0.04310793,0\C,50,1.41033894,49
,118.7052805,48,-0.92443645,0\C,51,1.39704291,50,120.33309708,49,1.397
19458,0\H,47,1.08681478,35,178.22612345,1,143.10516215,0\H,48,1.086828
25,47,120.31479265,35,-177.89402134,0\H,49,1.0850706,48,118.8745297,47
,179.4672362,0\H,51,1.08342168,50,120.83410377,49,-178.72061661,0\H,52
,1.08677421,51,119.24564591,50,179.42592822,0\C,35,1.95119858,1,119.63
730332,5,70.45222504,0\C,58,1.55187256,35,109.53096112,1,-86.20187601,
0\H,59,1.09364226,58,111.43975006,35,-67.9601504,0\H,59,1.09053924,58,
114.50471688,35,54.98037895,0\H,59,1.09728808,58,107.23129041,35,174.3
4856435,0\C,58,1.54517271,35,112.90634211,1,37.09785151,0\H,63,1.09352
536,58,113.14184567,35,-64.87520124,0\H,63,1.09386441,58,113.27142091,
35,57.43136116,0\H,63,1.09765276,58,107.68645487,35,176.27087602,0\C,5
8,1.55272307,35,108.38712933,1,158.97926648,0\H,67,1.09786672,58,106.8
9078856,35,170.63293992,0\H,67,1.09052102,58,114.12248321,35,-70.52374
165,0\H,67,1.09491793,58,112.17492081,35,53.54514706,0\\Version=AM64L-
G03RevD.01\State=1-A\HF=-1685.7965975\MP2=-1690.9702796\RMSD=7.958e-09
\Thermal=0.\PG=C01 [X(C35H34P1)]\\@

282

1\1\GINC-CIPCLU06\SP\RMP2-FC\6-31+G(2d,p)\C14H14N1P1\C2175\18-May-2010
\0\\#p MP2(FC)/6-31+G(2d,p) scf=tight\\xizsp_11\\0,1\P\C,1,4.67503345\
C,2,1.40014199,1,61.52489899\C,3,1.39734057,2,120.16504211,1,0.8526425
3,0\C,4,1.4091144,3,120.6879566,2,0.20327445,0\C,5,1.40749603,4,118.40
56489,3,-0.02648172,0\C,2,1.39752604,1,58.35541069,5,152.64621439,0\H,
2,1.08791252,1,178.33576891,5,174.7301011,0\H,3,1.0881477,2,119.991409
86,1,-179.23843613,0\H,4,1.08781432,3,119.76058341,2,179.25790314,0\H,
6,1.08917585,5,119.26790125,4,179.00466909,0\H,7,1.08793284,2,120.1486
4637,1,179.40560473,0\C,1,4.66128931,5,103.01183979,4,-31.7175446,0\C,
13,1.39946385,1,59.24261567,5,-53.76890648,0\C,14,1.39642173,13,120.14
872824,1,1.84036254,0\C,15,1.4083446,14,120.7822924,13,0.48181585,0\C,
16,1.40519371,15,118.58843368,14,-1.02380647,0\C,13,1.39745123,1,60.32
849998,16,70.20252835,0\H,13,1.08781355,1,178.2018393,16,179.74835835,
0\H,14,1.08806911,13,120.14290131,1,-178.0548375,0\H,15,1.08894453,14,
119.35446141,13,179.9255006,0\H,17,1.08648838,16,119.26791938,15,-178.
83803971,0\H,18,1.08825653,13,120.02664077,1,178.09168358,0\C,1,2.8037
6482,16,124.4285294,15,-158.47653696,0\C,24,1.49950484,1,77.4570404,16
,32.90304414,0\H,24,1.09189774,1,87.3847709,16,151.16741993,0\H,24,1.0
8973438,1,132.24696189,16,-87.04299147,0\H,25,1.08972615,24,119.947887
65,1,-131.24979116,0\H,25,1.09024372,24,117.40798116,1,80.25082528,0\N
,24,1.46133255,1,31.78193627,16,-17.94047815,0\\Version=AM64L-G03RevD.
01\State=1-A\HF=-933.4883745\MP2=-935.7763257\RMSD=9.489e-09\Thermal=0
.\PG=C01 [X(C14H14N1P1)]\\@

282-Me$^+$

1\1\GINC-CIPCLU06\SP\RMP2-FC\6-31+G(2d,p)\C15H17N1P1(1+)\C2175\20-May-
2010\0\\#p MP2(FC)/6-31+G(2d,p) scf=tight\\xizmesp_21\\1,1\C\C,1,1.399
75421\C,2,1.39560635,1,120.08832811\C,3,1.41178603,2,119.93194562,1,0.
06218165,0\C,4,1.40986736,3,119.64034404,2,-0.29866524,0\C,5,1.3964116
2,4,119.93875602,3,0.46701032,0\H,1,1.08687746,6,119.84781825,5,179.90
978426,0\H,2,1.08664485,1,120.19553302,6,179.63816508,0\H,3,1.08764274
,2,119.67223458,1,178.71132994,0\H,5,1.08698344,4,120.93143448,3,179.6
3648335,0\H,6,1.08658537,5,119.67940894,4,179.36002549,0\C,3,5.4270922
1,2,143.2967016,1,130.03588952,0\C,12,1.39965957,3,45.65528897,2,-52.5
5035534,0\C,13,1.39557126,12,120.08475988,3,-33.09305852,0\C,14,1.4091
3062,13,119.58655345,12,-0.08348619,0\C,15,1.4083568,14,120.2842324,13
,-0.37006615,0\C,16,1.3962368,15,119.50200665,14,0.6651074,0\H,12,1.08
694152,3,149.99446163,2,19.7956032,0\H,13,1.08656619,12,120.2189178,3,
146.70912157,0\H,14,1.0874107,13,119.6743318,12,179.43099095,0\H,16,1.
08631286,15,120.20394734,14,-179.46813855,0\H,17,1.08659417,16,119.640
85969,15,179.56013016,0\C,4,3.42561737,3,111.5283826,2,-115.34652246,0
\C,23,1.49050992,4,76.65586387,3,-28.50017194,0\H,23,1.08996654,4,61.2
4633326,3,103.4831613,0\H,23,1.08702966,4,156.74448944,3,-167.67241169
,0\H,24,1.0893474,23,117.50670219,4,47.21872571,0\H,24,1.08697381,23,1
20.04454632,4,-162.70519734,0\P,15,1.79839339,14,119.88894627,13,-176.
40668272,0\C,29,1.81569051,15,110.75478735,14,84.24419115,0\H,30,1.094
8573,29,107.86929015,15,54.83547455,0\H,30,1.09521247,29,111.08189858,
15,174.05850565,0\H,30,1.09493925,29,110.11470979,15,-64.26291052,0\N,
24,1.48034403,23,59.86046063,4,-60.96246518,0\\Version=AM64L-G03RevD.0
1\State=1-A\HF=-972.945111\MP2=-975.3748819\RMSD=6.714e-09\Thermal=0.\
PG=C01 [X(C15H17N1P1)]\\@

283

1\1\GINC-CIPCLU06\SP\RMP2-FC\6-31+G(2d,p)\C15H18N1P1\C2175\26-May-2010
\0\\#p MP2(FC)/6-31+G(2d,p) scf=tight\\xndsp_39\\0,1\C\C,1,1.39775479\
C,2,1.39872841,1,119.90208032\C,3,1.40717417,2,121.0871169,1,0.2068632
1,0\C,4,1.40798641,3,118.2531623,2,-0.50914003,0\C,5,1.39766379,4,120.
80320835,3,0.44865813,0\H,1,1.08794264,2,120.13478665,3,179.81819733,0
\H,2,1.08798741,1,120.15901453,6,-179.40078919,0\H,3,1.08917384,2,119.
68629096,1,-179.39750117,0\H,5,1.08758416,4,119.70200915,3,-178.588133
6,0\H,6,1.08819617,5,119.82143937,4,179.9877299,0\C,5,5.29834853,4,87.
83815812,3,-151.37878579,0\C,12,1.39810318,5,80.44741988,4,-65.3008107
9,0\C,13,1.39813207,12,120.28152885,5,25.34267375,0\C,14,1.40645113,13
,120.83078115,12,-0.74399826,0\C,15,1.40815253,14,118.31234545,13,1.31
689897,0\C,16,1.39752762,15,120.814619,14,-1.04391315,0\H,12,1.0877943
4,5,148.65435207,4,159.71519947,0\H,13,1.08827727,12,120.05794983,5,-1
54.77429564,0\H,14,1.08765635,13,120.07045425,12,179.36468179,0\H,16,1
.0882487,15,119.87955775,14,179.41904262,0\H,17,1.08817315,16,119.6357
1724,15,-179.65000584,0\C,4,3.84539485,3,101.6209782,2,136.78692257,0\
H,23,1.10144414,4,145.04389984,3,-156.23821488,0\P,15,1.84943785,14,12
0.38253882,13,173.26353315,0\H,23,1.09655625,4,96.9817716,3,70.2444993
8,0\C,23,2.47283837,4,57.32919688,3,-143.85843232,0\H,27,1.0989854,23,
84.81727849,4,175.54182549,0\H,27,1.10155709,23,103.18783858,4,67.9768
0638,0\H,27,1.09534555,23,139.87920284,4,-72.77858438,0\C,23,1.5378514
9,4,85.18705653,3,-38.89958981,0\H,31,1.09695665,23,111.65459076,4,-85
.65234758,0\H,31,1.09791698,23,110.25595988,4,154.64535181,0\H,31,1.09
705334,23,110.89787803,4,34.78812931,0\N,27,1.46098461,23,32.52157045,
4,-45.28919899,0\\Version=AM64L-G03RevD.01\State=1-A\HF=-973.7295333\M
P2=-976.1960205\RMSD=4.637e-09\Thermal=0.\PG=C01 [X(C15H18N1P1)]\\@

283-Me$^+$

1\1\GINC-YANG\SP\RMP2-FC\6-31+G(2d,p)\C16H21N1P1(1+)\CHRISTOPH\27-May-

2010\0\\#p MP2(FC)/6-31+g(2d,p) scf=tight\\xndmesp_21\\1,1\C\C,1,1.398
59388\C,2,1.39682324,1,120.14780818\C,3,1.40794171,2,119.69008253,1,0.
00544785,0\C,4,1.4093009,3,120.02417991,2,0.46674725,0\C,5,1.39532075,
4,119.74994744,3,-0.70842868,0\H,1,1.086935,2,119.8708212,3,179.810698
27,0\H,2,1.08660059,1,120.21257396,6,179.94618589,0\H,3,1.08672923,2,1
19.67436683,1,-179.78183615,0\H,5,1.08719929,4,120.14282502,3,179.8456
0997,0\H,6,1.0866669,5,119.71670808,4,-179.54592284,0\C,3,5.29675849,2
,144.83515511,1,-148.7877864,0\C,12,1.39951166,3,48.59725207,2,69.0389
5729,0\C,13,1.3956085,12,120.09151821,3,35.66310673,0\C,14,1.41091189,
13,120.04949031,12,0.01630421,0\C,15,1.40853752,14,119.49277361,13,0.0
5522695,0\C,16,1.39670192,15,120.04423587,14,-0.22167892,0\H,12,1.0868
5214,3,148.976022,2,-10.78481391,0\H,13,1.08667306,12,120.20429211,3,-
144.0907446,0\H,14,1.08759976,13,119.41432421,12,-178.95805634,0\H,16,
1.08668821,15,121.00509198,14,-179.69832452,0\H,17,1.08663239,16,119.6
608912,15,-179.49439216,0\C,15,3.81422297,14,117.67180765,13,127.81688
033,0\H,23,1.09691905,15,146.91521584,14,28.10852617,0\P,4,1.8060255,3
,120.69969363,2,174.69129829,0\C,25,1.81880086,4,108.31150656,3,-92.28
308622,0\H,26,1.0953257,25,108.11562983,4,58.29570915,0\H,26,1.0937530
4,25,112.72611369,4,179.12396485,0\H,26,1.09496422,25,108.67872933,4,-
59.30520414,0\H,23,1.09450855,15,98.40023327,14,165.78350891,0\C,23,2.
49182787,15,62.94992176,14,24.12223861,0\H,31,1.09512232,23,86.0208221
5,15,179.41087767,0\H,31,1.09401444,23,141.99001955,15,65.18510289,0\H
,31,1.09778363,23,98.77917755,15,-72.07882233,0\C,23,1.53360632,15,80.
516517,14,-84.51635815,0\H,35,1.09707947,23,111.81299419,15,-34.195442
36,0\H,35,1.09558051,23,111.78228623,15,87.09441722,0\H,35,1.09620698,
23,109.4877573,15,-153.7746226,0\N,31,1.47693068,23,32.88160596,15,46.
41527686,0\\Version=AM64L-G03RevD.01\State=1-A\HF=-1013.1836153\MP2=-1
015.7951621\RMSD=7.112e-09\Thermal=0.\PG=C01 [X(C16H21N1P1)]\\@

284

1\1\GINC-STEAK\SP\RMP2-FC\6-31+G(2d,p)\C19H17O1P1\CHRISTOPH\08-Feb-201
2\0\\#p MP2(FC)/6-31+g(2d,p) scf=tight\\ome1sp_6\\0,1\P\C,1,4.66963801
\C,2,1.39900064,1,58.36428916\C,3,1.39702158,2,120.00301665,1,0.023871
89,0\C,4,1.40799409,3,120.88995228,2,-0.94097994,0\C,5,1.40556451,4,11
8.5347988,3,1.07596916,0\C,2,1.39819627,1,61.32538708,5,-13.57103853,0
\H,2,1.08789794,1,178.42518421,5,-173.71366399,0\H,3,1.08798048,2,120.
15336553,1,179.3428369,0\H,4,1.08898588,3,119.64319259,2,178.79989643,
0\H,6,1.08722768,5,119.71089835,4,179.19447878,0\H,7,1.08822197,2,120.
02406351,1,-179.46993706,0\C,1,4.67034444,5,104.60452109,4,87.90950948
,0\C,13,1.39918122,1,58.31664514,5,-166.9502524,0\C,14,1.39668907,13,1
20.04043875,1,0.11038267,0\C,15,1.4081754,14,120.90327859,13,-0.924884
19,0\C,16,1.40580037,15,118.48971729,14,1.0350622,0\C,13,1.3978696,1,6
1.33598567,16,-10.68352049,0\H,13,1.08786803,1,178.42138572,16,-174.38
497425,0\H,14,1.08801605,13,120.13902538,1,179.42719102,0\H,15,1.08893
392,14,119.61431657,13,178.87037341,0\H,17,1.08724874,16,119.80614294,
15,179.29712087,0\H,18,1.0882128,13,120.05766198,1,-179.60761618,0\C,1
,4.67355478,16,104.31573276,15,86.19221891,0\C,24,1.4027809,1,61.36678
291,16,19.03812615,0\C,25,1.40024721,24,119.68574784,1,0.39309634,0\C,
26,1.40293785,25,121.57106613,24,-0.23035844,0\C,27,1.41108711,26,117.
72907755,25,-0.46450723,0\C,28,1.39000522,27,121.4379781,26,1.02202761
,0\H,25,1.08567884,24,121.06187009,1,-179.54759753,0\H,26,1.08759271,2
5,118.61552718,24,-179.83016336,0\H,28,1.0887519,27,119.4871329,26,-17
8.76497929,0\H,29,1.08672016,28,121.38700935,27,179.86326349,0\O,24,1.
36061465,1,173.8815339,27,173.66327737,0\C,34,1.41600587,24,118.240937
01,1,177.18505321,0\H,35,1.09258933,34,105.90450205,24,-179.93182155,0
\H,35,1.09928844,34,111.55326538,24,-61.12037672,0\H,35,1.09932143,34,
111.54357866,24,61.28267395,0\\Version=AM64L-G03RevD.01\State=1-A\HF=-
1145.0416173\MP2=-1148.0513908\RMSD=7.198e-09\Thermal=0.\PG=C01 [X(C19

H17O1P1)]\\@

284-Me$^+$

1\1\GINC-STEAK\SP\RMP2-FC\6-31+G(2d,p)\C20H20O1P1(1+)\CHRISTOPH\08-Feb
-2012\0\\#p MP2(FC)/6-31+g(2d,p) scf=tight\\ome1mesp_12\\1,1\P\C,1,1.8
2719513\H,2,1.09483604,1,109.73811335\H,2,1.09463321,1,109.92152636,3,
119.81372363,0\H,2,1.09467384,1,110.06601142,3,-119.93943644,0\C,1,4.6
0774332,2,107.93254466,4,179.3777692,0\C,6,1.39770147,1,60.24485517,2,
-131.25023202,0\C,7,1.39771086,6,120.14460243,1,0.14360723,0\C,8,1.406
21953,7,119.73284524,6,0.05268043,0\C,9,1.40987984,8,119.98923647,7,-0
.23498989,0\C,10,1.39479718,9,119.78478851,8,0.21686275,0\H,6,1.086916
98,1,179.82907623,9,131.85699615,0\H,7,1.08663111,6,120.22261724,1,-17
9.97294997,0\H,8,1.08659267,7,119.92864707,6,179.75963821,0\H,10,1.087
76015,9,120.88849624,8,-179.23472654,0\H,11,1.08666204,10,119.71681022
,9,179.87858156,0\C,1,4.61056427,9,110.64430037,8,-12.45055438,0\C,17,
1.40752209,1,60.1162791,9,109.26746721,0\C,18,1.39462106,17,119.764622
37,1,0.23211273,0\C,19,1.40610251,18,120.85646144,17,0.09043042,0\C,20
,1.41637612,19,118.98979106,18,-0.30113882,0\C,21,1.383781,20,120.4362
2623,19,0.25106563,0\H,18,1.08454325,17,120.98289351,1,-179.95097537,0
\H,19,1.08676562,18,118.86881926,17,179.74269516,0\H,21,1.08786141,20,
120.83686387,19,-179.37650859,0\H,22,1.0858992,21,121.25811559,20,179.
81134617,0\C,1,4.60717956,20,110.09421668,19,-14.02548743,0\C,27,1.397
82709,1,60.30187596,20,108.28604207,0\C,28,1.39758823,27,120.14590033,
1,0.26312174,0\C,29,1.40648195,28,119.72752986,27,-0.01097124,0\C,30,1
.40971948,29,119.99584923,28,-0.2562373,0\C,31,1.3949973,30,119.774934
64,29,0.31632776,0\H,27,1.08694141,1,179.77748362,20,-66.28951075,0\H,
28,1.08664365,27,120.22636002,1,-179.92478097,0\H,29,1.08650973,28,119
.84750759,27,179.65663161,0\H,31,1.08767147,30,120.85693456,29,-179.26
877721,0\H,32,1.0866631,31,119.7081061,30,179.75499306,0\O,17,1.339705
39,1,174.96175961,20,127.65900033,0\C,38,1.42768108,17,119.38446292,1,
179.69215994,0\H,39,1.09073841,38,105.48931284,17,179.79112711,0\H,39,
1.09712338,38,111.01573928,17,-61.58437002,0\H,39,1.0970876,38,110.981
04862,17,61.17040835,0\\Version=AM64L-G03RevD.01\State=1-A\HF=-1184.49
72905\MP2=-1187.6502716\RMSD=5.451e-09\Thermal=0.\PG=C01 [X(C20H20O1P1
)]\\@

285

1\1\GINC-GOLEM\SP\RMP2-FC\6-31+G(2d,p)\C18H21P1\CHRISTOPH\01-Jun-2010\
0\\#p MP2(FC)/6-31+g(2d,p) scf=tight\\x6sp_4\\0,1\P\C,1,4.67695111\C,2
,1.39936064,1,61.21080507\C,3,1.39741489,2,120.22944287,1,0.27644415,0
\C,4,1.40660815,3,120.76418324,2,0.10449305,0\C,5,1.40689694,4,118.357
71317,3,0.45763605,0\C,2,1.39750722,1,58.48233179,5,168.32995648,0\H,2
,1.08790411,1,178.65291203,5,174.66192002,0\H,3,1.08823674,2,120.02054
988,1,-179.8766119,0\H,4,1.0876168,3,119.3804577,2,179.15581487,0\H,6,
1.088715,5,119.32037876,4,178.86281651,0\H,7,1.08803386,2,120.16396872
,1,179.88908764,0\C,1,4.67764545,5,102.13438571,4,-35.58986826,0\C,13,
1.39819308,1,57.96083065,5,-95.8316628,0\C,14,1.39750825,13,120.040972
04,1,-0.46344018,0\C,15,1.40786064,14,121.1697108,13,0.91243715,0\C,16
,1.40772338,15,118.02478059,14,-1.04058266,0\C,13,1.39808389,1,61.6399
5037,16,-0.07244491,0\H,13,1.08780439,1,178.19381746,16,177.32992838,0
\H,14,1.08793955,13,120.13452786,1,-179.86870783,0\H,15,1.08871568,14,
119.61344732,13,-178.8736004,0\H,17,1.08760751,16,120.23298961,15,-179
.3953363,0\H,18,1.08818656,13,120.06387489,1,179.96134202,0\H,1,2.4833
0728,16,87.52204777,15,177.94568651,0\C,1,4.22984992,16,123.55008502,1
5,161.79477757,0\C,25,1.54112877,1,19.34442063,16,164.950444,0\C,26,1.
54674111,25,111.38172643,1,-2.87852087,0\C,27,1.54661916,26,110.152336
22,25,55.8121872,0\C,28,1.54108272,27,111.53769006,26,-55.82833765,0\C
,29,1.53867012,28,111.56695971,27,55.66178197,0\H,25,1.09896282,1,128.

9922496,16,170.37664705,0\H,25,1.10135745,1,101.10242171,16,48.1973111
4,0\H,26,1.10168054,25,109.20372506,1,117.68011783,0\H,26,1.09743396,2
5,109.97147752,1,-125.43954656,0\H,28,1.10136906,27,108.76046864,26,64
.7849892,0\H,28,1.09768909,27,110.55292918,26,-178.56536081,0\H,29,1.0
9894786,28,109.77188977,27,178.31082423,0\H,29,1.10153636,28,109.17622
796,27,-65.20636584,0\H,30,1.09888522,29,110.31835288,28,-177.24119301
,0\H,30,1.10139473,29,109.13872437,28,65.90846938,0\\Version=AM64L-G03
RevD.01\State=1-A\HF=-1034.6567907\MP2=-1037.3804497\RMSD=6.521e-09\Th
ermal=0.\PG=C01 [X(C18H21P1)]\\@

285-Me$^+$

1\1\GINC-YANG\SP\RMP2-FC\6-31+G(2d,p)\C19H24P1(1+)\CHRISTOPH\02-Jun-20
10\0\\#p MP2(FC)/6-31+g(2d,p) scf=tight\\x6mesp_1\\1,1\P\C,1,1.8265762
8\H,2,1.09379007,1,112.00725224\H,2,1.09500838,1,109.31656707,3,-121.5
7733883,0\H,2,1.09573752,1,108.40624773,4,-118.26796795,0\C,1,4.609604
57,2,110.21208308,3,60.90791383,0\C,6,1.39955148,1,59.89524222,2,159.3
942588,0\C,7,1.39551002,6,120.13720192,1,-1.10297608,0\C,8,1.41045413,
7,119.9049454,6,-0.01206,0\C,9,1.40794488,8,119.69373291,7,-0.00708441
,0\C,10,1.39696631,9,119.91947616,8,-0.0392422,0\H,6,1.08684303,1,179.
17282519,9,178.36115886,0\H,7,1.08666124,6,120.2208447,1,179.16325909,
0\H,8,1.08714807,7,119.57066935,6,-179.08219135,0\H,10,1.08646899,9,12
0.99091561,8,-179.69334087,0\H,11,1.08657361,10,119.61144348,9,-179.68
438157,0\C,1,4.61046755,9,110.72982951,8,45.963131,0\C,17,1.39890069,1
,59.88164409,9,30.72960059,0\C,18,1.39620027,17,120.1503861,1,-1.76750
313,0\C,19,1.40871893,18,119.87375638,17,-0.04105379,0\C,20,1.40829643
,19,119.7521687,18,0.40538705,0\C,21,1.39641881,20,119.90071315,19,-0.
52807033,0\H,17,1.08684411,1,178.59257,9,113.08418104,0\H,18,1.0866591
2,17,120.21986119,1,178.34825838,0\H,19,1.08716074,18,119.62719559,17,
-179.599133,0\H,21,1.08687272,20,121.03464324,19,179.58091856,0\H,22,1
.08660727,21,119.63430491,20,-179.65961924,0\C,1,4.21266244,9,101.1279
9207,8,-61.94305637,0\C,28,1.54226405,1,20.91511842,9,53.4536517,0\C,2
9,1.5503426,28,110.49777726,1,-1.78579332,0\C,30,1.55203894,29,111.175
0051,28,-56.23626889,0\C,31,1.54319916,30,110.44797959,29,55.78854316,
0\C,32,1.53712206,31,111.90942518,30,-55.30628044,0\H,28,1.10057436,1,
99.19557836,9,174.41242201,0\H,28,1.09700532,1,129.83058337,9,53.60069
833,0\H,29,1.09941026,28,109.18092926,1,-121.62960872,0\H,29,1.0974889
6,28,110.11906669,1,120.89314629,0\H,30,1.10219241,29,108.97737263,28,
62.80296263,0\H,31,1.09757116,30,110.91158399,29,177.04409075,0\H,31,1
.09982551,30,109.30581488,29,-64.78683799,0\H,32,1.10041514,31,109.065
61058,30,66.17383777,0\H,32,1.09699055,31,108.8468317,30,-177.84655994
,0\H,33,1.10047119,32,109.45959316,31,-65.74480719,0\H,33,1.09676135,3
2,109.96388868,31,177.4029462,0\\Version=AM64L-G03RevD.01\State=1-A\HF
=-1074.1099397\MP2=-1076.9794388\RMSD=5.061e-09\Thermal=0.\PG=C01 [X(C
19H24P1)]\\@

285-BH$^+$

1\1\GINC-AZAZEL\SP\RMP2-FC\6-31+G(2d,p)\C31H32P1(1+)\CHRISTOPH\20-Jul-
2010\0\\#p MP2(FC)/6-31+g(2d,p) scf=tight\\x6bhsp_7\\1,1\C\H,1,1.09780
455\C,1,4.35111937,2,106.20308988\C,3,1.39820785,1,59.38180566,2,-26.3
7436023,0\C,4,1.39726678,3,120.09531865,1,0.13746294,0\C,5,1.40645593,
4,120.76698485,3,-0.37932845,0\C,6,1.40413693,5,118.67538246,4,0.80076
818,0\C,7,1.39772523,6,120.48589316,5,-0.56125738,0\H,3,1.08701753,1,1
79.44513346,6,-144.02239129,0\H,4,1.0871456,3,120.25592004,1,179.27194
767,0\H,5,1.08749517,4,119.17939376,3,179.09609717,0\H,7,1.08554473,6,
120.24365948,5,178.0326633,0\H,8,1.0871565,7,119.45196848,6,179.373476
11,0\C,1,4.34635139,6,115.09707088,5,-142.78177874,0\C,14,1.39827824,1
,58.83068792,6,99.54286152,0\C,15,1.39686734,14,120.09626542,1,0.69589
395,0\C,16,1.40558945,15,120.86430695,14,-0.2729792,0\C,17,1.40569146,

16,118.55884239,15,-0.04221856,0\C,14,1.39725946,1,60.77308242,17,25.3
4448459,0\H,14,1.08684747,1,178.90218889,17,176.58943086,0\H,15,1.0870
1092,14,120.22573947,1,-179.24266542,0\H,16,1.08887945,15,119.46571671
,14,-179.43009055,0\H,18,1.08727608,17,120.42597887,16,-179.00839269,0
\H,19,1.08723103,14,120.12084976,1,179.1980234,0\P,1,1.89318744,17,112
.76306442,16,-130.81303375,0\C,25,4.62123471,1,109.2971012,17,-83.0939
8066,0\C,26,1.39840855,25,59.62852633,1,-71.66479496,0\C,27,1.39635663
,26,120.25386565,25,-0.63638745,0\C,28,1.41045155,27,119.99192069,26,-
0.26192927,0\C,29,1.40919691,28,119.41794488,27,0.60169415,0\C,30,1.39
653729,29,120.11127578,28,-0.53312813,0\H,27,1.08666648,26,120.2656178
9,25,179.17710836,0\H,28,1.08622206,27,119.49392158,26,179.78135301,0\
H,30,1.0861058,29,120.91127528,28,179.31352936,0\H,31,1.08670315,30,11
9.58905032,29,-179.67983885,0\C,25,4.62812228,1,110.25616732,17,155.91
779379,0\C,36,1.39895685,25,60.05326147,1,-38.11795918,0\C,37,1.396017
29,36,120.14005254,25,1.6955923,0\C,38,1.40995711,37,120.26644445,36,0
.31339706,0\C,39,1.40785636,38,119.19821105,37,-0.95582512,0\C,36,1.39
743863,25,59.92356588,1,140.50367099,0\H,36,1.08688115,25,178.66545574
,1,-129.58851078,0\H,37,1.08676771,36,120.25294944,25,-178.31333693,0\
H,38,1.08698332,37,118.72926766,36,-179.65124545,0\H,40,1.08595475,39,
120.45435707,38,-179.25919587,0\H,41,1.08673045,36,120.23301099,25,178
.32761699,0\H,26,1.08695655,25,179.40839416,1,-21.20824767,0\H,25,2.39
066936,1,98.39708031,17,15.32728791,0\C,25,4.21683696,1,129.75752896,1
7,40.68645062,0\C,49,1.5427474,25,21.01271541,1,169.10616031,0\C,50,1.
55181066,49,110.26708125,25,-3.40611071,0\C,51,1.55108139,50,110.36570
872,49,-57.67227163,0\C,52,1.54298876,51,110.20352117,50,57.46671039,0
\C,53,1.53724858,52,111.83219914,51,-56.2025522,0\H,49,1.09724687,25,1
29.94799723,1,171.80120285,0\H,49,1.10073085,25,98.4927935,1,-68.01212
208,0\H,50,1.09678907,49,110.00858835,25,119.74211076,0\H,50,1.0991516
,49,109.3844907,25,-123.22310386,0\H,52,1.09944861,51,109.26867781,50,
-62.46369829,0\H,52,1.09626345,51,110.82430963,50,178.68959591,0\H,53,
1.09730231,52,108.914735,51,-178.61179404,0\H,53,1.10027165,52,109.025
55363,51,65.41918906,0\H,54,1.10053006,53,109.37992482,52,-65.96478013
,0\H,54,1.09697751,53,110.09435001,52,177.12426162,0\\Version=AM64L-G0
3RevD.01\State=1-A\HF=-1533.2201512\MP2=-1537.7919303\RMSD=9.615e-09\T
hermal=0.\PG=C01 [X(C31H32P1)]\\@

285-TT$^+$

1\1\GINC-EVGENIX\SP\RMP2-FC\6-31+G(2d,p)\C37H36P1(1+)\CHRISTOPH\02-Oct
-2011\0\\#p MP2(FC)/6-31+g(2d,p) scf=tight\\x6ttsp_3\\1,1\C\C,1,4.3876
593\C,2,1.39652574,1,58.92681305\C,3,1.39819896,2,120.52971512,1,-1.41
779724,0\C,4,1.40782966,3,121.12497904,2,-0.18717048,0\C,5,1.40762625,
4,117.57126135,3,2.07435182,0\C,2,1.39696172,1,60.13395836,5,-65.75284
413,0\H,2,1.08702689,1,178.90968977,5,170.79051738,0\H,3,1.08724594,2,
120.23758824,1,178.34875354,0\H,4,1.08463096,3,118.66118776,2,179.2969
9813,0\H,6,1.08350538,5,120.61306893,4,175.84434397,0\H,7,1.08743499,2
,120.26048192,1,-178.39744475,0\C,1,4.38971819,5,110.81937487,4,-99.20
77257,0\C,13,1.3953723,1,59.38847577,5,-0.05086252,0\C,14,1.39938086,1
3,120.62592135,1,0.11671166,0\C,15,1.406348,14,121.14255057,13,0.08122
587,0\C,16,1.41312038,15,117.49183005,14,0.67554199,0\C,17,1.39685149,
16,121.29204178,15,-0.89595208,0\H,13,1.08715006,1,179.86359703,16,-14
5.83699582,0\H,14,1.08723316,13,120.19904723,1,179.98153283,0\H,15,1.0
8354407,14,118.53008511,13,179.77631917,0\H,17,1.08403719,16,120.39116
216,15,178.72927893,0\H,18,1.08746181,17,119.36486632,16,179.88413668,
0\C,1,4.39245805,16,114.33657834,15,-124.01610594,0\C,24,1.3993831,1,5
9.07615384,16,178.7382471,0\C,25,1.39554286,24,120.18167015,1,-0.29901
996,0\C,26,1.41226719,25,121.61491865,24,1.33674868,0\C,27,1.40481257,
26,117.24746396,25,-3.48215189,0\C,24,1.3947105,1,59.94133856,16,0.442
74931,0\H,24,1.08701366,1,179.50860813,16,-174.76579577,0\H,25,1.08743

245,24,120.27211194,1,-179.03636183,0\H,26,1.08487505,25,118.35645964,
24,-175.57468514,0\H,28,1.0831679,27,120.52251564,26,-175.80925683,0\H
,29,1.08724806,24,120.19120792,1,179.28144807,0\P,1,2.00342965,16,105.
51459244,15,118.77412969,0\C,35,4.64471783,1,111.62541408,16,68.166783
27,0\C,36,1.39870244,35,59.64945047,1,59.01269843,0\C,37,1.39617688,36
,120.40694344,35,-1.19898386,0\C,38,1.41160541,37,120.13857546,36,-0.4
9237895,0\C,39,1.40787741,38,119.07539329,37,1.20753484,0\C,36,1.39713
44,35,60.11464061,1,-120.12222659,0\H,36,1.08707993,35,178.81299482,1,
136.88300189,0\H,37,1.08695839,36,120.23385682,35,178.58528975,0\H,38,
1.08527017,37,118.85804419,36,179.89932226,0\H,40,1.08457784,39,120.62
077742,38,178.47211148,0\H,41,1.08697415,36,120.27407158,35,-178.38455
001,0\C,35,4.65286576,1,111.1256516,16,-52.26974995,0\C,47,1.39732327,
35,59.77058171,1,-121.05059551,0\C,48,1.39632986,47,120.24568902,35,-0
.14538986,0\C,49,1.41007429,48,120.68425511,47,-0.06492785,0\C,50,1.41
224324,49,118.57135079,48,0.09267076,0\C,51,1.39675565,50,120.31971915
,49,-0.11088986,0\H,47,1.08703595,35,179.82001699,1,-54.36903839,0\H,4
8,1.08689512,47,120.33059615,35,179.86465851,0\H,49,1.0825102,48,118.4
9532815,47,179.2055449,0\H,51,1.08395603,50,120.84627517,49,-179.87813
722,0\H,52,1.08689662,51,119.2087036,50,179.98934351,0\C,35,4.22901038
,1,131.71765599,16,171.09622081,0\C,58,1.5433388,35,22.41149184,1,174.
5050842,0\C,59,1.55663336,58,109.64967011,35,-13.402685,0\C,60,1.54939
491,59,111.41925425,58,-57.03223815,0\C,61,1.54370138,60,109.83774826,
59,56.90244562,0\C,62,1.53703121,61,111.94986836,60,-55.83519863,0\H,5
8,1.10072266,35,94.67618097,1,-53.87180661,0\H,58,1.09751509,35,130.68
770641,1,-170.53940139,0\H,59,1.09699346,58,109.13274478,35,-134.70955
153,0\H,59,1.09522496,58,109.75151527,35,108.34520839,0\H,60,1.1003074
3,59,105.80631182,58,59.24826412,0\H,61,1.0944584,60,111.32184247,59,1
79.02143171,0\H,61,1.09762087,60,110.61499386,59,-63.41994507,0\H,62,1
.10138768,61,109.15190073,60,65.52937059,0\H,62,1.09739857,61,108.9351
4931,60,-178.48358914,0\H,63,1.10091986,62,109.34234973,61,-65.5066537
3,0\H,63,1.09720463,62,110.12894742,61,177.69419417,0\\Version=AM64L-G
03RevD.01\State=1-A\HF=-1762.7366142\MP2=-1768.186917\RMSD=8.866e-09\T
hermal=0.\PG=C01 [X(C37H36P1)]\\@

286

1\1\GINC-YANG\SP\RMP2-FC\6-31+G(2d,p)\C17H19P1\CHRISTOPH\17-Jul-2010\0
\\#p MP2(FC)/6-31+g(2d,p) scf=tight\\xx5sp_30_2\\0,1\P\C,1,4.67592702\
C,2,1.3993969,1,61.11668345\C,3,1.39752035,2,120.21619914,1,0.45760216
,0\C,4,1.40673729,3,120.70707056,2,0.09530278,0\C,5,1.40650179,4,118.4
4776069,3,0.4584901,0\C,2,1.39758519,1,58.60569443,5,161.05185544,0\H,
2,1.08793208,1,178.71894836,5,175.03125627,0\H,3,1.08822963,2,120.0115
8995,1,-179.66476702,0\H,4,1.08790018,3,119.50558638,2,179.34443641,0\
H,6,1.08875839,5,119.36787934,4,178.85662615,0\H,7,1.08805226,2,120.14
836304,1,179.70544185,0\C,1,4.67457664,5,101.84204284,4,-34.44089642,0
\C,13,1.39915167,1,58.01199752,5,-86.51476702,0\C,14,1.39633038,13,120
.05625917,1,-0.14321019,0\C,15,1.40885412,14,121.14297787,13,0.9889876
1,0\C,16,1.40639394,15,118.07758589,14,-0.97442606,0\C,13,1.39728642,1
,61.55974058,16,9.13327562,0\H,13,1.08778301,1,178.19052353,16,175.521
53906,0\H,14,1.08795922,13,120.12909131,1,-179.50929376,0\H,15,1.08896
788,14,119.53070316,13,-178.81608134,0\H,17,1.08704254,16,120.27925178
,15,-179.81692177,0\H,18,1.08820839,13,120.07889736,1,179.63662617,0\C
,1,1.87387322,16,103.56251181,15,167.73344693,0\C,24,1.56343549,1,112.
91257497,16,-70.77107183,0\C,25,1.56003833,24,106.14536551,1,-137.9585
3385,0\C,26,1.54424527,25,105.44814511,24,-9.7872112,0\C,27,1.5397164,
26,103.49783517,25,31.9713215,0\H,24,1.10009327,1,109.9552447,16,52.04
059416,0\H,25,1.09591573,24,110.70800909,1,100.51922719,0\H,25,1.09787
455,24,111.23887956,1,-17.64781381,0\H,26,1.09639568,25,112.37002903,2
4,-132.78928274,0\H,26,1.0977874,25,109.87096654,24,108.24359175,0\H,2

7,1.0995351,26,110.34986724,25,-85.24688847,0\H,27,1.09697674,26,112.8
9636888,25,154.54870039,0\H,28,1.10073729,27,109.61029767,26,75.457320
57,0\H,28,1.09573358,27,113.74209011,26,-164.07040258,0\\Version=AM64L
-G03RevD.01\State=1-A\HF=-995.6107982\MP2=-998.1786464\RMSD=6.824e-09\
Thermal=0.\PG=C01 [X(C17H19P1)]\\@

286-Me⁺

1\1\GINC-YANG\SP\RMP2-FC\6-31+G(2d,p)\C18H22P1(1+)\CHRISTOPH\12-Jul-20
10\0\\#p MP2(FC)/6-31+g(2d,p) scf=tight\\xx5mesp_17\\1,1\C\C,1,1.39790
502\C,2,1.39724708,1,120.12671162\C,3,1.40678262,2,119.84836218,1,0.17
636379,0\C,4,1.40991021,3,119.82786789,2,-0.12224441,0\C,5,1.3950244,4
,119.8627195,3,0.00707168,0\H,1,1.08684802,2,119.89728895,3,179.997399
22,0\H,2,1.08655196,1,120.24069189,6,179.78999967,0\H,3,1.08621668,2,1
19.21338555,1,179.81741108,0\H,5,1.08785188,4,120.55738631,3,179.29797
536,0\H,6,1.0866346,5,119.69940911,4,179.899929,0\C,5,5.46455781,4,84.
09391029,3,146.35180582,0\C,12,1.39863619,5,80.18679438,4,69.88469312,
0\C,13,1.39646327,12,120.1179332,5,-23.75840857,0\C,14,1.40899924,13,1
19.94575855,12,0.09727532,0\C,15,1.40958865,14,119.6769431,13,0.199856
57,0\C,16,1.39606777,15,119.89664879,14,-0.32683204,0\H,12,1.08684533,
5,149.99391591,4,-154.72582926,0\H,13,1.08658426,12,120.24681067,5,155
.95500596,0\H,14,1.08674326,13,119.04670938,12,179.54233839,0\H,16,1.0
8701694,15,120.45521309,14,178.45285208,0\H,17,1.08663357,16,119.62358
998,15,179.75124897,0\C,4,3.00900434,3,87.24924144,2,176.3690871,0\C,2
3,1.57205736,4,96.84975433,3,-65.84112931,0\C,24,1.55101969,23,105.285
19309,4,-158.43378198,0\C,25,1.53859438,24,104.73739133,23,-22.3494685
7,0\C,26,1.54120174,25,103.21766072,24,39.76576817,0\H,23,1.0990541,4,
85.32482896,3,43.72024403,0\H,24,1.09553357,23,109.08252318,4,82.22767
994,0\H,24,1.09579364,23,113.15419496,4,-36.41417089,0\H,25,1.09471464
,24,111.89778041,23,-145.27329519,0\H,25,1.09766752,24,109.88681234,23
,95.4577404,0\H,26,1.09834353,25,110.58558592,24,-77.82366864,0\H,26,1
.0950457,25,113.18328095,24,161.27735107,0\H,27,1.09898045,26,109.0031
1525,25,76.70565408,0\H,27,1.09538386,26,113.62355496,25,-162.70451347
,0\P,15,1.80915406,14,120.56499578,13,-177.24168097,0\C,37,1.82568286,
15,109.18614633,14,-28.68770334,0\H,38,1.09452775,37,111.1737853,15,63
.41367401,0\H,38,1.09562637,37,108.55938776,15,-176.89988121,0\H,38,1.
09485719,37,109.93652273,15,-57.97562747,0\\Version=AM64L-G03RevD.01\S
tate=1-A\HF=-1035.0637015\MP2=-1037.7781333\RMSD=3.491e-09\Thermal=0.\
PG=C01 [X(C18H22P1)]\\@

286-BH⁺

1\1\GINC-CALYPSO\SP\RMP2-FC\6-31+G(2d,p)\C30H30P1(1+)\CHRISTOPH\14-Aug
-2010\0\\#p MP2(FC)/6-31+g(2d,p) scf=tight\\x5bhsp_104\\1,1\C\H,1,1.09
815118\C,1,4.35248367,2,106.24165828\C,3,1.39764881,1,60.1846195,2,151
.63367798,0\C,4,1.39790362,3,120.43215402,1,0.05704629,0\C,5,1.4041377
9,4,120.51643243,3,-0.13749742,0\C,6,1.40653586,5,118.61253208,4,-0.60
789338,0\C,7,1.39720217,6,120.8073489,5,0.89568696,0\H,3,1.08698597,1,
179.46899419,6,-147.09850901,0\H,4,1.08712426,3,120.13582862,1,-179.44
543118,0\H,5,1.08524256,4,119.16915461,3,-178.72986991,0\H,7,1.0872713
9,6,120.04728793,5,-178.59358315,0\H,8,1.08712559,7,119.63764395,6,-17
9.55240458,0\C,1,4.34681732,6,115.59149998,5,34.72855441,0\C,14,1.3981
4677,1,58.87906412,6,102.12973305,0\C,15,1.39731942,14,120.11834427,1,
0.6772052,0\C,16,1.4051868,15,120.85100518,14,-0.34296701,0\C,17,1.406
29557,16,118.55894535,15,0.19925626,0\C,14,1.39756471,1,60.71360525,17
,24.55339798,0\H,14,1.08683292,1,178.92808012,17,172.22307317,0\H,15,1
.08700981,14,120.21512722,1,-179.21841157,0\H,16,1.08881658,15,119.451
9152,14,-179.4337323,0\H,18,1.08754558,17,120.44941312,16,-179.5478837
1,0\H,19,1.08722262,14,120.12917689,1,179.23526153,0\P,1,1.89077868,17
,111.9288103,16,-127.79649989,0\C,25,4.61823353,1,110.30162081,17,-82.

89345486,0\C,26,1.39842129,25,60.37077817,1,111.84762709,0\C,27,1.3966
4975,26,120.13728994,25,0.48878475,0\C,28,1.40855526,27,120.0684817,26
,0.03222485,0\C,29,1.41088221,28,119.52179776,27,-0.05190838,0\C,30,1.
39603947,29,119.91365786,28,0.08013344,0\H,27,1.08667762,26,120.260944
47,25,-179.75738339,0\H,28,1.08558659,27,119.08506427,26,179.88646028,
0\H,30,1.08627057,29,120.5920462,28,179.77548294,0\H,31,1.08665062,30,
119.49488922,29,179.95905409,0\C,25,4.62844084,1,111.00734861,17,155.7
8086686,0\C,36,1.39893352,25,60.20557171,1,-36.34249775,0\C,37,1.39598
327,36,120.14330769,25,1.67760436,0\C,38,1.41010115,37,120.31297843,36
,0.28904125,0\C,39,1.40840487,38,119.1259948,37,-0.55374287,0\C,36,1.3
9746989,25,59.76850765,1,142.04333828,0\H,36,1.08685668,25,178.6081329
,1,-134.22665826,0\H,37,1.08673627,36,120.25384135,25,-178.38885503,0\
H,38,1.08669662,37,118.61806383,36,179.97874626,0\H,40,1.08613893,39,1
20.39641413,38,179.92448037,0\H,41,1.08670697,36,120.22901008,25,178.3
5857377,0\H,25,2.37728171,1,96.52959868,17,15.11996541,0\C,25,1.845481
44,1,109.4746374,17,38.89563827,0\C,48,1.56079409,25,113.90989302,1,53
.93063368,0\C,49,1.54146891,48,103.03620445,25,152.67722233,0\C,50,1.5
3799614,49,103.02465991,48,-41.50860781,0\C,51,1.54981193,50,104.53057
686,49,40.68386822,0\H,49,1.09779207,48,111.21727038,25,36.58146227,0\
H,49,1.09423793,48,111.79052307,25,-84.54143776,0\H,50,1.09857054,49,1
10.01108451,48,76.42562193,0\H,50,1.09530696,49,112.25863914,48,-163.6
9641127,0\H,51,1.094903,50,113.3692385,49,162.8922528,0\H,51,1.0978674
2,50,109.60906046,49,-76.98802662,0\H,52,1.09562378,51,111.52377067,50
,-146.90743443,0\H,52,1.09575817,51,110.86667716,50,94.22005857,0\H,26
,1.08696962,25,179.56492922,1,-21.19600052,0\\Version=AM64L-G03RevD.01
\State=1-A\HF=-1494.1746036\MP2=-1498.5903336\RMSD=8.929e-09\Thermal=0
.\PG=C01 [X(C30H30P1)]\\@

286-TT$^+$

1\1\GINC-NODE15\SP\RMP2-FC\6-31+G(2d,p)\C36H34P1(1+)\ZIP07\05-Oct-2011
\0\\#p MP2(FC)/6-31+G(2d,p) scf=tight\\x5ttsp_3\\1,1\C\C,1,4.38986149\
C,2,1.39554918,1,59.33298998\C,3,1.3994654,2,120.64466792,1,0.01600828
,0\C,4,1.40597406,3,121.1106105,2,-0.1559454,0\C,5,1.4126035,4,117.532
00072,3,-0.55126801,0\C,6,1.39712273,5,121.28470944,4,0.8258951,0\H,2,
1.0870035,1,179.83353625,5,156.49301326,0\H,3,1.08711049,2,120.1907395
8,1,-179.87453918,0\H,4,1.08357244,3,118.5705552,2,-179.76943975,0\H,6
,1.08419054,5,120.48205223,4,-178.99459659,0\H,7,1.08735965,6,119.3418
6782,5,-179.98215198,0\C,1,4.38665798,5,108.70577016,4,-0.89085014,0\C
,13,1.39681011,1,58.93834684,5,100.40621032,0\C,14,1.39783575,13,120.5
0991349,1,1.42434576,0\C,15,1.40794036,14,121.1271004,13,0.21988319,0\
C,16,1.40659704,15,117.59776211,14,-2.10896006,0\C,13,1.39659361,1,60.
12857464,5,-79.80942714,0\H,13,1.08690868,1,178.91004616,5,43.37870158
,0\H,14,1.08715297,13,120.25452535,1,-178.36665051,0\H,15,1.08461578,1
4,118.67086929,13,-179.26567024,0\H,17,1.08380819,16,120.63217741,15,-
175.66911285,0\H,18,1.08731724,13,120.27030766,1,178.34699735,0\C,1,4.
39478095,5,114.07234096,4,121.56333778,0\C,24,1.39472774,1,59.93132594
,5,2.21177933,0\C,25,1.40058689,24,120.6989562,1,-0.06606274,0\C,26,1.
40485766,25,121.16445717,24,0.30274785,0\C,27,1.41250931,26,117.240894
77,25,-2.7932033,0\C,28,1.39557158,27,121.61283702,26,3.2749601,0\H,24
,1.08692352,1,179.4446565,5,152.755589,0\H,25,1.08711217,24,120.199396
1,1,-179.63104635,0\H,26,1.08297873,25,118.32112539,24,-178.5320251,0\
H,28,1.08491722,27,119.92930658,26,-173.58731417,0\H,29,1.0873335,28,1
19.51813524,27,-179.99158032,0\P,1,1.98872139,5,106.40415363,4,-121.65
689933,0\C,35,4.65208721,1,112.53017844,5,51.71008986,0\C,36,1.3975749
4,35,59.95649695,1,-55.62606977,0\C,37,1.39691024,36,120.53199637,35,0
.04337037,0\C,38,1.41173155,37,120.26406072,36,-0.02435458,0\C,39,1.41
031766,38,118.69933008,37,-0.20115355,0\C,40,1.39656642,39,120.5607755
6,38,0.21135004,0\H,36,1.08689941,35,179.82364497,1,95.62250594,0\H,37

,1.08678945,36,120.27186294,35,179.96982147,0\H,38,1.08366485,37,118.9
136772,36,-179.7755393,0\H,40,1.08265654,39,121.04330847,38,179.956774
73,0\H,41,1.0868169,40,119.39432121,39,-179.98067033,0\C,35,4.64162418
,1,112.34802371,5,-69.64468803,0\C,47,1.39731051,35,60.06150188,1,119.
02753473,0\C,48,1.3971722,47,120.28998888,35,-1.22680443,0\C,49,1.4079
3238,48,120.21502152,47,-0.05973922,0\C,50,1.41113126,49,119.21250988,
48,0.74327861,0\C,51,1.39618305,50,120.0574394,49,-0.92032937,0\H,47,1
.08696474,35,178.94213885,1,-141.49494489,0\H,48,1.0868828,47,120.2650
6568,35,178.55473922,0\H,49,1.0849884,48,119.19517814,47,179.52331329,
0\H,51,1.08537812,50,120.9999093,49,179.45475898,0\H,52,1.08682423,51,
119.37812247,50,-179.78700748,0\C,35,2.96873784,1,98.04143122,5,154.01
838589,0\C,58,1.55412804,35,32.58198892,1,125.79144857,0\C,59,1.577490
79,58,106.3154247,35,131.09488262,0\C,60,1.55391222,59,104.94121188,58
,9.77823316,0\C,58,1.54162172,35,132.92930638,1,149.34058972,0\H,58,1.
09507903,35,87.79105847,1,-94.82356693,0\H,58,1.09414875,35,101.393624
44,1,11.61666225,0\H,59,1.09626821,58,107.22351178,35,-115.21233065,0\
H,60,1.0945867,59,108.9500937,58,129.52136214,0\H,60,1.09442883,59,113
.35567841,58,-112.3128649,0\H,61,1.09484619,60,111.72905515,59,138.887
91181,0\H,61,1.09861972,60,110.21016764,59,-102.50111305,0\H,62,1.0953
5437,58,112.32390388,35,151.22495831,0\H,62,1.09881652,58,109.85075646
,35,-89.28995218,0\\Version=AM64L-G03RevD.01\State=1-A\HF=-1723.693823
2\MP2=-1728.9871655\RMSD=8.347e-09\Thermal=0.\PG=C01 [X(C36H34P1)]\\@

287

1\1\GINC-AZAZEL\SP\RMP2-FC\6-31+G(2d,p)\C23H24N1O1P1\RAMAN\10-Oct-2008
\0\\#p MP2(FC)/6-31+g(2d,p) scf=tight int=finegrid\\paracatsp015\\0,1\
C\C,1,1.3979568\C,2,1.39935458,1,120.30103579\C,3,1.40579268,2,120.573
6819,1,-0.26243044,0\C,4,1.40815475,3,118.53085918,2,-0.39640055,0\C,5
,1.39678103,4,120.87970523,3,0.98296363,0\P,4,1.85324347,3,124.5971657
5,2,-178.79993382,0\C,7,1.85209368,4,102.34960461,3,12.22838413,0\C,8,
1.40570904,7,124.66544688,4,-93.65563336,0\C,9,1.39914157,8,120.574067
57,7,-178.71960364,0\C,10,1.39806099,9,120.29962109,8,-0.24985795,0\C,
11,1.39904967,10,119.68595304,9,0.40587758,0\C,12,1.39683304,11,120.01
358824,10,0.19569606,0\C,7,1.84707035,4,102.12099255,3,-94.29401007,0\
C,14,1.40625972,7,124.83847943,4,17.83004737,0\C,15,1.3929709,14,120.8
502755,7,-178.58620864,0\C,16,1.40738105,15,120.82705208,14,-0.2441822
4,0\C,17,1.4062874,16,119.09893849,15,0.16394347,0\C,18,1.39556271,17,
119.29258897,16,0.4317319,0\N,17,1.40867603,16,117.22837429,15,179.690
96004,0\C,20,1.38211398,17,129.3550974,16,-179.29741215,0\O,21,1.22158
886,20,123.08971582,17,-0.93740096,0\C,21,1.55389733,20,114.47764768,1
7,178.91633579,0\C,23,1.54903256,21,110.1258122,20,60.19936627,0\C,23,
1.54034703,21,108.01160171,20,179.52539991,0\C,23,1.54905621,21,109.92
61777,20,-61.25520317,0\H,16,1.09005987,15,119.50455717,14,179.6418258
4,0\H,15,1.08723246,14,120.03051112,7,0.91562161,0\H,19,1.08883544,18,
118.39878154,17,178.73865245,0\H,18,1.08195924,17,119.61366684,16,179.
64185756,0\H,20,1.01085784,17,114.45490525,16,-0.32776552,0\H,9,1.0873
3828,8,119.79381215,7,0.93931129,0\H,13,1.08896537,12,119.62673521,11,
178.77954106,0\H,10,1.08819177,9,119.65527788,8,179.7765006,0\H,12,1.0
8796709,11,120.14175955,10,179.50116129,0\H,11,1.08786056,10,120.14989
291,9,179.95479291,0\H,3,1.08720318,2,119.65142161,1,-179.98507334,0\H
,5,1.08892567,4,119.47003591,3,-178.80296917,0\H,2,1.08820467,1,120.04
646038,6,-179.70086835,0\H,6,1.08798975,5,119.82730094,4,179.80119652,
0\H,1,1.08788034,2,120.16698062,3,179.91813913,0\H,25,1.09473924,23,11
1.06977754,21,59.63440505,0\H,25,1.09734387,23,109.69464855,21,179.838
56963,0\H,25,1.09485611,23,111.08627106,21,-59.95822668,0\H,26,1.09755
225,23,109.71255651,21,-176.14076891,0\H,26,1.09678614,23,111.05192388
,21,-56.8206807,0\H,26,1.09798985,23,112.73940499,21,64.3634962,0\H,24
,1.09807799,23,112.78467342,21,-64.50571275,0\H,24,1.0968579,23,111.09

110735,21,56.70394995,0\H,24,1.09754766,23,109.73055837,21,176.0338031
3,0\\Version=AM64L-G03RevD.01\State=1-A\HF=-1355.1005286\MP2=-1358.893
4487\RMSD=4.607e-09\Thermal=0.\PG=C01 [X(C23H24N1O1P1)]\\@

287-Me+

1\1\GINC-CALYPSO\SP\RMP2-FC\6-31+G(2d,p)\C24H27N1O1P1(1+)\RAMAN\08-Oct
-2008\0\\#p MP2(FC)/6-31+g(2d,p) scf=tight int=finegrid\\spmethylparac
at014\\1,1\C\C,1,1.408769\C,2,1.411991,1,118.689\C,3,1.386457,2,120.29
3,1,-0.342,0\C,4,1.414782,3,120.955,2,0.145,0\C,5,1.412273,4,118.89,3,
0.113,0\P,2,1.796012,3,120.514,4,-178.357,0\C,7,1.826828,2,109.033,3,-
43.84,0\N,5,1.389844,4,117.645,3,-179.818,0\C,9,1.401679,5,128.716,4,-
179.874,0\C,10,1.54597,9,114.559,5,-179.566,0\C,11,1.550166,10,109.805
,9,-60.927,0\O,10,1.216811,9,121.788,5,0.443,0\C,11,1.550192,10,109.82
2,9,60.342,0\C,11,1.540828,10,107.998,9,179.691,0\C,7,1.810144,2,110.2
1,3,-162.57,0\C,16,1.406245,7,120.67,2,-110.023,0\C,17,1.397703,16,119
.702,7,179.265,0\C,18,1.397655,17,120.157,16,0.029,0\C,19,1.400494,18,
120.271,17,-0.203,0\C,20,1.394729,19,120.077,18,0.105,0\C,7,1.811129,1
6,110.145,17,12.879,0\C,22,1.409746,7,119.865,16,70.131,0\C,23,1.39499
,22,119.777,7,-179.352,0\C,24,1.400488,23,120.07,22,0.057,0\C,25,1.397
767,24,120.278,23,0.091,0\C,26,1.397652,25,120.144,24,-0.13,0\H,4,1.08
8216,3,119.299,2,-179.723,0\H,3,1.087554,2,121.122,1,179.376,0\H,1,1.0
86864,2,120.215,3,179.931,0\H,6,1.081663,5,119.417,4,-179.937,0\H,9,1.
011663,5,115.379,4,0.489,0\H,23,1.087768,22,120.889,27,179.323,0\H,27,
1.086645,22,120.318,23,179.932,0\H,24,1.086682,23,119.725,22,-179.88,0
\H,26,1.086655,25,120.213,24,179.962,0\H,25,1.08695,24,119.805,23,-179
.921,0\H,17,1.086493,18,119.837,19,-179.794,0\H,21,1.087793,16,120.862
,17,179.082,0\H,18,1.086621,19,120.227,20,179.879,0\H,20,1.086645,19,1
20.214,18,-179.864,0\H,19,1.086916,18,119.921,17,179.869,0\H,15,1.0947
35,11,111.23,10,60.065,0\H,15,1.096299,11,109.495,10,-179.908,0\H,15,1
.094802,11,111.197,10,-59.937,0\H,12,1.096545,11,109.38,10,-176.227,0\
H,12,1.096569,11,111.225,10,-57.092,0\H,12,1.098448,11,112.93,10,64.61
8,0\H,14,1.0984,11,112.934,10,-64.904,0\H,14,1.096582,11,111.269,10,56
.841,0\H,14,1.096464,11,109.351,10,175.957,0\H,8,1.094761,7,110.052,16
,-177.135,0\H,8,1.094649,7,109.876,16,-57.221,0\H,8,1.094632,7,109.858
,16,62.695,0\\Version=AM64L-G03RevD.01\State=1-A\HF=-1394.5566263\MP2=
-1398.4923752\RMSD=1.566e-09\Thermal=0.\PG=C01 [X(C24H27N1O1P1)]\\@

288

1\1\GINC-YANG\SP\RMP2-FC\6-31+G(2d,p)\C20H19P1\CHRISTOPH\17-Aug-2011\0
\\#p MP2(FC)/6-31+g(2d,p) scf=tight\\tol2sp_3\\0,1\P\C,1,4.67000319\C,
2,1.39788629,1,61.33255368\C,3,1.39934178,2,120.3134212,1,0.55187103,0
\C,4,1.40573543,3,120.58520221,2,-0.23425217,0\C,5,1.40819841,4,118.50
490618,3,-0.482505,0\C,6,1.39672163,5,120.89375378,4,1.077212,0\H,3,1.
08819272,2,120.05254568,1,-179.52430261,0\H,4,1.08728202,3,119.6131778
6,2,179.98442711,0\H,6,1.08895158,5,119.493131,4,-178.74321961,0\H,7,1
.08800506,6,119.82398975,5,179.72687515,0\C,1,4.69144787,5,102.9339403
1,4,13.66595182,0\C,12,1.40326637,1,57.4953427,5,90.0517366,0\C,13,1.3
9630564,12,121.01250737,1,0.27832794,0\C,14,1.40679194,13,121.03005999
,12,-1.01265057,0\C,15,1.40587018,14,117.99067989,13,1.00707433,0\C,16
,1.39721742,15,120.73900327,14,-0.49158808,0\H,13,1.08910084,12,119.49
41625,1,179.72887014,0\H,14,1.08900635,13,119.41554775,12,178.99783442
,0\H,16,1.08747362,15,119.87463039,14,179.42566819,0\H,17,1.08941773,1
6,119.32277755,15,-179.90447551,0\C,1,4.69145339,15,103.00816553,14,-1
60.2948676,0\C,22,1.40315411,1,60.46148599,15,-89.13474359,0\C,23,1.39
76291,22,121.27936862,1,0.22361464,0\C,24,1.40568688,23,120.71626321,2
2,-0.03237234,0\C,25,1.40704125,24,118.01125062,23,-0.43309191,0\C,26,
1.39585607,25,121.02945966,24,0.93642479,0\H,23,1.08932293,22,119.3720
4886,1,-179.85989289,0\H,24,1.08753849,23,119.43489493,22,-179.9282802

8,0\H,26,1.08895461,25,119.52339555,24,-179.05716293,0\H,27,1.08912462
,26,119.52052935,25,179.57440488,0\C,12,1.51388763,1,178.04195878,15,-
142.36517341,0\H,32,1.09621589,12,111.43047217,1,-70.09409613,0\H,32,1
.09936878,12,111.09313046,1,49.58732385,0\H,32,1.09665595,12,111.39835
382,1,169.07662872,0\C,22,1.51390665,1,177.91076816,15,134.25560677,0\
H,36,1.0994077,22,111.08845211,1,45.50466947,0\H,36,1.09638565,22,111.
42313578,1,165.09785564,0\H,36,1.09648487,22,111.41246819,1,-74.056317
04,0\H,2,1.08786476,1,178.40036061,15,-149.04855865,0\\Version=AM64L-G
03RevD.01\State=1-A\HF=-1109.235015\MP2=-1112.2019222\RMSD=7.798e-09\T
hermal=0.\PG=C01 [X(C20H19P1)]\\@

288-Me⁺

1\1\GINC-YIN\SP\RMP2-FC\6-31+G(2d,p)\C21H22P1(1+)\CHRISTOPH\18-Aug-201
1\0\\#p MP2(FC)/6-31+g(2d,p) scf=tight\\tol2mesp_2\\1,1\C\C,1,1.400352
59\C,2,1.39495894,1,120.07298475\C,3,1.40961124,2,119.78296545,1,-0.10
342528,0\C,4,1.40642657,3,120.00146804,2,0.29916955,0\C,5,1.39754611,4
,119.71630333,3,-0.24967177,0\H,2,1.08665979,1,120.20846153,6,179.9411
3125,0\H,3,1.08765168,2,119.31496523,1,179.4097354,0\H,5,1.0865638,4,1
20.38681502,3,-179.98941328,0\H,6,1.08663033,5,119.62383838,4,-179.852
52768,0\C,4,5.53773528,3,95.1345437,2,131.18743072,0\C,11,1.40778989,4
,76.27435572,3,112.22664522,0\C,12,1.39206605,11,121.19284901,4,3.9245
9309,0\C,13,1.41036843,12,119.97293136,11,0.06764229,0\C,14,1.40608621
,13,119.39140483,12,0.29142415,0\C,15,1.39577867,14,119.87694481,13,-0
.28310241,0\H,12,1.0877879,11,119.52428599,4,-176.03981527,0\H,13,1.08
790034,12,119.09677989,11,179.44664728,0\H,15,1.08670137,14,120.487167
5,13,179.82978506,0\H,16,1.08756131,15,119.15922224,14,179.9828209,0\C
,5,5.21157376,4,95.38580207,3,-168.61746186,0\C,21,1.40801372,5,57.464
44197,4,74.41021722,0\C,22,1.39191682,21,121.20013928,5,-38.42715112,0
\C,23,1.41062434,22,119.95112344,21,0.0451248,0\C,24,1.40580867,23,119
.402536,22,0.28590338,0\C,25,1.39594744,24,119.88844273,23,-0.26012298
,0\H,22,1.08779913,21,119.51449917,5,141.63605967,0\H,23,1.08797823,22
,119.13855935,21,179.49446354,0\H,25,1.08670804,24,120.44865188,23,179
.96080316,0\H,26,1.0875595,25,119.18671295,24,-179.96100459,0\C,11,1.5
1043553,4,162.39055142,3,-52.57571083,0\H,31,1.09869948,11,110.4524264
,4,81.95262083,0\H,31,1.09578448,11,111.34519993,4,-158.94617536,0\H,3
1,1.09478306,11,111.53386837,4,-37.66334384,0\H,1,1.08691536,6,119.911
77224,5,-179.81291938,0\P,14,1.80446286,13,119.71651656,12,179.1748943
4,0\C,36,1.82696739,14,108.47614129,13,48.20773158,0\H,37,1.0946263,36
,110.00885826,14,58.00373709,0\H,37,1.09468976,36,109.96122652,14,178.
16635525,0\H,37,1.09468133,36,109.75156582,14,-61.92463655,0\C,21,1.51
050128,5,148.48758536,4,170.96234958,0\H,41,1.09861251,21,110.47138003
,5,-154.39758348,0\H,41,1.09598443,21,111.31387032,5,-35.35287302,0\H,
41,1.09460196,21,111.54823169,5,85.87562077,0\\Version=AM64L-G03RevD.0
1\State=1-A\HF=-1148.6914196\MP2=-1151.8011001\RMSD=5.616e-09\Thermal=
0.\PG=C01 [X(C21H22P1)]\\@

289

1\1\GINC-YIN\SP\RMP2-FC\6-31+G(2d,p)\C19H23P1\CHRISTOPH\10-Jun-2010\0\
\#p MP2(FC)/6-31+g(2d,p) scf=tight\\x7sp_3\\0,1\P\C,1,4.67761395\C,2,1
.39861644,1,57.82808347\C,3,1.39678053,2,120.04562116,1,0.43622537,0\C
,4,1.40859901,3,121.19740303,2,-1.04492657,0\C,5,1.40711017,4,118.0007
5314,3,1.15643288,0\C,2,1.39762691,1,61.73613711,5,-2.16683227,0\H,2,1
.0877914,1,178.07015159,5,-177.53960125,0\H,3,1.08797955,2,120.1344883
1,1,179.79007077,0\H,4,1.08887488,3,119.54279016,2,178.80715909,0\H,6,
1.08747955,5,120.31486908,4,179.32737129,0\H,7,1.08820088,2,120.076349
24,1,-179.86252354,0\C,1,4.67638657,5,103.21364836,4,90.35938096,0\C,1
3,1.39967891,1,61.08457384,5,35.54723312,0\C,14,1.39716006,13,120.1814
7282,1,-0.49465319,0\C,15,1.40698626,14,120.75176296,13,0.02759039,0\C

,16,1.40621375,15,118.42349555,14,-0.58353485,0\C,13,1.39744248,1,58.6
4529524,5,-143.64840345,0\H,13,1.08793895,1,178.7663846,5,-159.7525436
1,0\H,14,1.08822248,13,120.02405087,1,179.69296644,0\H,15,1.08793641,1
4,119.4520136,13,-179.10356311,0\H,17,1.08859838,16,119.34102465,15,-1
78.97384092,0\H,18,1.08803807,13,120.14617347,1,-179.66949656,0\C,1,1.
89070738,5,103.70194107,4,-164.25529233,0\C,24,1.5515615,1,108.1140072
2,5,-164.75183933,0\C,25,1.54681482,24,116.5268762,1,-160.12882815,0\C
,26,1.54371287,25,116.4317105,24,-39.96036636,0\C,27,1.54145911,26,113
.36335487,25,85.99176396,0\C,28,1.53898911,27,116.24151422,26,-68.6463
0419,0\C,29,1.5442172,28,116.39169385,27,52.68640897,0\H,25,1.09767008
,24,107.76219755,1,76.90659553,0\H,25,1.10121373,24,109.57611582,1,-37
.77275195,0\H,26,1.10013407,25,109.57995159,24,83.02665332,0\H,26,1.09
912326,25,107.16538063,24,-162.85551286,0\H,27,1.10117278,26,110.47930
556,25,-38.6040454,0\H,27,1.09933204,26,108.52980701,25,-154.35913079,
0\H,28,1.10000469,27,109.1495036,26,168.78712726,0\H,28,1.10157815,27,
107.93144646,26,53.98804084,0\H,29,1.09994893,28,108.101301,27,175.245
98943,0\H,29,1.10193936,28,109.13944471,27,-70.06111156,0\H,30,1.09875
038,29,107.43222793,28,166.99494299,0\H,30,1.09980754,29,110.5538994,2
8,51.91897423,0\H,24,1.1005561,1,108.6479468,5,-48.22799756,0\\Version
=AM64L-G03RevD.01\State=1-A\HF=-1073.6823638\MP2=-1076.5630383\RMSD=7.
273e-09\Thermal=0.\PG=C01 [X(C19H23P1)]\\@

289-Me⁺

1\1\GINC-NODE3\SP\RMP2-FC\6-31+G(2d,p)\C20H26P1(1+)\ZIP07\15-Jun-2010\
0\\#p MP2(FC)/6-31+G(2d,p) scf=tight\\x7mesp_7\\1,1\C\C,1,1.39966765\C
,2,1.39533942,1,120.15811462\C,3,1.41025802,2,119.90596852,1,-0.057874
27,0\C,4,1.40798276,3,119.67189024,2,0.174746,0\C,5,1.39713376,4,119.9
4932081,3,-0.08789028,0\H,1,1.08686547,6,119.90819457,5,179.91666481,0
\H,2,1.0866545,1,120.20343822,6,179.56405666,0\H,3,1.08685621,2,119.54
400656,1,178.97154228,0\H,5,1.08643607,4,120.97191958,3,179.48594932,0
\H,6,1.08659519,5,119.6150032,4,179.69690011,0\C,3,5.48036397,2,140.36
82203,1,119.73139849,0\C,12,1.39883767,3,44.29825663,2,-45.76701161,0\
C,13,1.39618584,12,120.15365963,3,-32.23129032,0\C,14,1.40885137,13,11
9.89498245,12,-0.05282928,0\C,15,1.40818918,14,119.72755865,13,-0.2071
4502,0\C,16,1.39646779,15,119.907285,14,0.37384145,0\H,12,1.0868332,3,
150.21520147,2,23.60771253,0\H,13,1.0866479,12,120.22886458,3,147.5796
2407,0\H,14,1.08721842,13,119.58934142,12,179.43742637,0\H,16,1.086973
79,15,121.03813239,14,-179.99578657,0\H,17,1.08659832,16,119.62098093,
15,179.61459101,0\C,4,3.02011129,3,106.95226524,2,-141.62491304,0\C,23
,1.55372852,4,93.73028533,3,6.43679358,0\C,24,1.54645222,23,112.435687
83,4,-179.05675495,0\C,25,1.53817538,24,116.41403033,23,-72.42218864,0
\C,26,1.54061458,25,116.20665865,24,53.5576329,0\C,27,1.54268597,26,11
3.36439307,25,-68.35503948,0\C,28,1.54602083,27,116.30900359,26,87.087
34634,0\H,24,1.09846894,23,109.81913033,4,-59.81232266,0\H,24,1.097850
4,23,109.7217712,4,56.88491344,0\H,25,1.09819529,24,107.86147816,23,16
5.60094369,0\H,25,1.10179823,24,108.26557518,23,51.45150249,0\H,26,1.0
9820778,25,107.81122345,24,176.42853532,0\H,26,1.10043016,25,108.93332
141,24,-68.90643781,0\H,27,1.10150392,26,110.67912166,25,56.60001004,0
\H,27,1.09741383,26,107.81946648,25,171.81152704,0\H,28,1.09766629,27,
109.77533152,26,-152.20711797,0\H,28,1.09874469,27,108.75915258,26,-36
.98573668,0\H,29,1.09813887,28,108.74321002,27,78.55116962,0\H,29,1.09
997652,28,107.46238166,27,-166.2816161,0\H,23,1.100958,4,137.9774764,3
,-115.48210616,0\P,4,1.81015984,3,119.52635337,2,-177.34119977,0\C,43,
1.82643732,4,109.18012523,3,-163.72482244,0\H,44,1.09498029,43,109.588
18721,4,58.92938528,0\H,44,1.09573689,43,108.40367889,4,177.31245271,0
\H,44,1.09386579,43,111.62638474,4,-62.75627305,0\\Version=AM64L-G03Re
vD.01\State=1-A\HF=-1113.1365232\MP2=-1116.1627087\RMSD=4.311e-09\Ther
mal=0.\PG=C01 [X(C20H26P1)]\\@

289-BH$^+$

1\1\GINC-NODE4\SP\RMP2-FC\6-31+G(2d,p)\C32H34P1(1+)\ZIP07\13-Oct-2010\
0\\#p MP2(FC)/6-31+G(2d,p) scf=tight\\x7bhsp_28\\1,1\C\H,1,1.09748336\
C,1,4.35005807,2,105.71071139\C,3,1.39843793,1,59.28824959,2,-29.03053
525,0\C,4,1.39710501,3,120.06610852,1,0.28414743,0\C,5,1.40680486,4,12
0.77013109,3,-0.51051321,0\C,6,1.40375957,5,118.70876595,4,1.01444353,
0\C,3,1.39770416,1,60.27663223,6,1.87946322,0\H,3,1.08703172,1,179.428
26347,6,-151.66730328,0\H,4,1.08717637,3,120.26150045,1,179.36476396,0
\H,5,1.08776118,4,119.23079042,3,179.02589264,0\H,7,1.08548356,6,120.2
9819692,5,177.87855272,0\H,8,1.08716114,3,120.11525938,1,-179.47003743
,0\C,1,4.34654824,6,116.37632241,5,-146.20807322,0\C,14,1.39757068,1,6
0.6864018,6,-77.49242559,0\C,15,1.39883575,14,120.31150177,1,-0.111102
91,0\C,16,1.4062377,15,120.58457883,14,-0.307454,0\C,17,1.40526293,16,
118.53851647,15,0.36611971,0\C,18,1.39741751,17,120.86232295,16,-0.055
37246,0\H,14,1.08684726,1,179.06395459,17,174.98849004,0\H,15,1.087236
27,14,120.12368852,1,179.46895799,0\H,16,1.08744085,15,118.99394571,14
,179.23670039,0\H,18,1.08886553,17,119.7070165,16,179.13045983,0\H,19,
1.08705255,18,119.66878209,17,179.55186268,0\P,1,1.89485823,17,111.828
86457,16,53.62899463,0\C,25,4.62691098,1,110.51469709,17,154.25362827,
0\C,26,1.399137,25,59.98729542,1,-40.05049733,0\C,27,1.39587831,26,120
.1408342,25,1.58385216,0\C,28,1.40989311,27,120.22594524,26,0.3549485,
0\C,29,1.40755869,28,119.2628138,27,-1.04466759,0\C,26,1.39737528,25,5
9.99742006,1,138.71989656,0\H,26,1.08687738,25,178.78019997,1,-128.391
08209,0\H,27,1.08677135,26,120.24418153,25,-178.4046786,0\H,28,1.08687
552,27,118.7481074,26,-179.48434425,0\H,30,1.08592904,29,120.43887388,
28,-179.18622859,0\H,31,1.0867558,26,120.22897357,25,178.41717465,0\C,
25,4.62210826,1,108.96602176,17,-84.5890762,0\C,37,1.39855212,25,60.41
050318,1,109.91351391,0\C,38,1.39658383,37,120.1353398,25,0.73740175,0
\C,39,1.4092635,38,120.14250403,37,0.06456684,0\C,40,1.41045813,39,119
.39565233,38,-0.39126562,0\C,41,1.39639165,40,119.98642088,39,0.477411
74,0\H,37,1.08697688,25,179.40825791,1,-16.8743357,0\H,38,1.08672355,3
7,120.27417733,25,-179.49773298,0\H,39,1.08619402,38,118.96330552,37,-
179.97354187,0\H,41,1.08626655,40,120.54863931,39,-179.48411423,0\H,42
,1.08666392,41,119.46744323,40,179.93471948,0\C,25,1.86018147,1,110.44
373011,17,37.81923262,0\C,48,1.55581166,25,110.04667837,1,174.55709057
,0\C,49,1.54253727,48,114.32035122,25,162.51272574,0\C,50,1.54156412,4
9,116.92049279,48,54.93650815,0\C,51,1.54285538,50,113.76282605,49,-70
.0824387,0\C,52,1.54510657,51,116.08542946,50,84.09973851,0\C,53,1.546
88267,52,116.82032682,51,-37.29882672,0\H,48,1.09908649,25,104.3175683
1,1,-66.7695055,0\H,49,1.09834896,48,107.8717527,25,-75.21188527,0\H,4
9,1.09812535,48,110.78541303,25,40.61721308,0\H,50,1.10125049,49,109.0
0531014,48,-68.30560744,0\H,50,1.09852119,49,106.85083622,48,177.51406
352,0\H,51,1.09772267,50,107.23648349,49,169.94506519,0\H,51,1.1005428
8,50,110.40613727,49,55.01889345,0\H,52,1.10064865,51,108.29572915,50,
-39.76259126,0\H,52,1.09749423,51,109.43094871,50,-154.12999739,0\H,53
,1.09839957,52,108.61426355,51,-160.67307075,0\H,53,1.09846564,52,109.
31651022,51,83.88042617,0\H,54,1.0969676,53,106.68319871,52,-164.55164
973,0\H,54,1.09725933,53,110.19126779,52,80.65995011,0\\Version=AM64L-
G03RevD.01\State=1-A\HF=-1572.2459091\MP2=-1576.976466\RMSD=8.480e-09\
Thermal=0.\PG=C01 [X(C32H34P1)]\\@

290

1\1\GINC-CIPCLU05\SP\RMP2-FC\6-31+G(2d,p)\C15H16N1P1\C2175\18-May-2010
\0\\#p MP2(FC)/6-31+G(2d,p) scf=tight\\xazsp_21\\0,1\P\C,1,4.67553577\
C,2,1.39773766,1,58.38561129\C,3,1.39898207,2,119.91818298,1,-1.472262
33,0\C,4,1.40698126,3,121.0135305,2,0.02063216,0\C,5,1.40891976,4,118.
34469365,3,-0.14121212,0\C,6,1.39765946,5,120.7447077,4,0.14427315,0\H
,2,1.08796262,1,178.1526364,5,177.29137741,0\H,3,1.08802349,2,120.1386

5201,1,178.94105205,0\H,4,1.08905596,3,119.73481293,2,-179.35008338,0\
H,6,1.08792431,5,119.63375212,4,-178.80073911,0\H,7,1.08823587,6,119.8
4455727,5,-179.90380158,0\C,1,4.66495332,5,103.59195656,4,146.95141338
,0\C,13,1.39795478,1,59.83347632,5,132.95150472,0\C,14,1.3985364,13,12
0.30895642,1,-1.98844674,0\C,15,1.40597171,14,120.7055699,13,-0.555629
73,0\C,16,1.40791363,15,118.45194586,14,1.13353985,0\C,17,1.39713499,1
6,120.7799133,15,-0.99440867,0\H,13,1.08783411,1,178.24224751,16,-179.
6191872,0\H,14,1.08830301,13,120.03794275,1,177.98484725,0\H,15,1.0873
0569,14,120.24395638,13,179.38518466,0\H,17,1.08844214,16,119.86086452
,15,179.60513988,0\H,18,1.0881522,17,119.67297956,16,-179.62518764,0\C
,1,2.90658094,16,92.27375368,15,50.2590047,0\C,24,1.55192644,1,112.424
93109,16,-141.26632752,0\C,25,1.55312858,24,87.05329327,1,-0.82505128,
0\H,24,1.10048776,1,91.83115115,16,102.68750259,0\H,24,1.09727792,1,10
9.92948356,16,-8.22585277,0\H,25,1.09349768,24,116.45285954,1,-119.038
55172,0\H,25,1.09408353,24,113.0108855,1,112.86681918,0\H,26,1.1009759
5,25,112.70977023,24,-102.07834944,0\H,26,1.09722203,25,117.51388293,2
4,130.11045743,0\N,26,1.48305137,25,88.73994374,24,13.50902726,0\\Vers
ion=AM64L-G03RevD.01\State=1-A\HF=-972.5369641\MP2=-974.9764504\RMSD=4
.265e-09\Thermal=0.\PG=C01 [X(C15H16N1P1)]\\@

290-Me$^+$

1\1\GINC-CIPCLU09\SP\RMP2-FC\6-31+G(2d,p)\C16H19N1P1(1+)\C2175\18-May-
2010\0\\#p MP2(FC)/6-31+G(2d,p) scf=tight\\xazmesp_2\\1,1\C\C,1,1.3991
2037\C,2,1.39620113,1,120.12496972\C,3,1.40847466,2,119.64879855,1,0.0
3878792,0\C,4,1.40917851,3,120.1121016,2,0.52120959,0\C,5,1.39557405,4
,119.67467381,3,-0.79454407,0\H,1,1.08694844,2,119.82553078,3,179.7801
2979,0\H,2,1.08659498,1,120.20777632,6,179.82526758,0\H,3,1.08685437,2
,119.74031718,1,-179.65271354,0\H,5,1.08688308,4,120.13046847,3,179.28
965032,0\H,6,1.08663441,5,119.71754803,4,-179.57328269,0\C,3,5.3648717
5,2,144.88343826,1,-141.79843437,0\C,12,1.39976596,3,46.50536881,2,60.
27901361,0\C,13,1.39546415,12,120.09337023,3,33.97781192,0\C,14,1.4118
2864,13,120.00101517,12,-0.09996451,0\C,15,1.4090854,14,119.53021743,1
3,0.37280717,0\C,16,1.39666832,15,120.01996074,14,-0.5361977,0\H,12,1.
08688265,3,149.67317027,2,-14.53281145,0\H,13,1.08667184,12,120.184491
41,3,-145.69768468,0\H,14,1.08766967,13,119.53317495,12,-178.82242418,
0\H,16,1.08674563,15,120.9378344,14,-179.82427415,0\H,17,1.08661868,16
,119.6810547,15,-179.38068182,0\C,15,3.69654835,14,115.27075413,13,120
.74827021,0\C,23,1.55126703,15,110.02212921,14,4.6666503,0\C,24,1.5513
6758,23,88.04469055,15,32.02279128,0\H,23,1.09822653,15,65.01515103,14
,-102.50544366,0\H,23,1.09437697,15,128.75682266,14,161.38100575,0\H,2
4,1.09161502,23,115.9038057,15,-86.00223162,0\H,24,1.09246863,23,112.9
4852522,15,146.2880128,0\H,25,1.09776287,24,113.32710471,23,-102.37642
366,0\H,25,1.09406141,24,117.60265083,23,127.86686723,0\P,4,1.80073751
,3,120.69039518,2,175.95405796,0\C,32,1.81787673,4,110.60770045,3,-91.
9077864,0\H,33,1.0947864,32,108.09421615,4,-54.28668276,0\H,33,1.09520
318,32,111.41929398,4,-174.0910971,0\H,33,1.09488569,32,109.81470113,4
,64.55632465,0\N,25,1.49976595,24,88.66178279,23,12.35026312,0\\Versio
n=AM64L-G03RevD.01\State=1-A\HF=-1011.994493\MP2=-1014.5768142\RMSD=5.
315e-09\Thermal=0.\PG=C01 [X(C16H19N1P1)]\\@

291

1\1\GINC-SOLARIS\SP\RMP2-FC\6-31+G(2d,p)\C17H20N1P1\CHRISTOPH\27-May-2
010\0\\#p MP2(FC)/6-31+g(2d,p) scf=tight\\xpipsp_13\\0,1\C\C,1,1.39980
744\C,2,1.39717746,1,120.20092907\C,3,1.40818674,2,120.78891179,1,0.15
824359,0\C,4,1.40749819,3,118.23979017,2,0.14574425,0\C,1,1.39743317,2
,119.78079154,3,-0.25611064,0\H,1,1.08826996,6,120.16089327,5,179.7163
4863,0\H,2,1.08852085,1,120.00534855,6,179.76465075,0\H,3,1.08804936,2
,119.49902093,1,179.26532439,0\H,5,1.08929541,4,119.21258994,3,179.240

28168,0\H,6,1.08831928,1,120.16206698,2,-179.56846614,0\C,3,5.29719252
,2,142.1924801,1,135.53073308,0\C,12,1.39876369,3,45.1795787,2,-56.786
05061,0\C,13,1.39720461,12,120.26353871,3,-35.59180982,0\C,14,1.408153
75,13,120.86415469,12,0.33588125,0\C,15,1.40706345,14,118.26541287,13,
-1.16848631,0\C,12,1.39786708,3,81.16678978,2,154.32714108,0\H,12,1.08
807142,3,148.54590165,2,17.93083416,0\H,13,1.08842603,12,120.11472873,
3,144.29233699,0\H,14,1.08859628,13,119.29804569,12,179.68656148,0\H,1
6,1.08754422,15,119.01554647,14,-178.71588497,0\H,17,1.08865151,12,120
.05929671,3,-155.40279777,0\P,15,1.84829138,14,120.55997878,13,-172.87
923623,0\C,23,4.02441622,15,114.43913116,14,-171.90957848,0\C,24,1.536
79409,23,24.51600505,15,174.73272993,0\C,25,2.44935078,24,91.39145821,
23,-49.91391906,0\C,26,1.53850797,25,91.30470604,24,-0.83242862,0\C,27
,1.53744793,26,111.13659177,25,-26.80113392,0\H,25,1.1074571,24,109.10
989953,23,-143.84969758,0\H,25,1.09611437,24,110.67036715,23,98.202238
98,0\H,26,1.10638111,25,93.7369667,24,-110.61102665,0\H,26,1.09556483,
25,142.13851395,24,124.65567209,0\H,27,1.09997275,26,108.77693701,25,9
3.93021246,0\H,27,1.09904324,26,109.21235994,25,-149.22806693,0\H,28,1
.1015811,27,109.26369709,26,-66.74544085,0\H,28,1.09842513,27,110.5950
8315,26,175.92376776,0\H,24,1.09906422,23,131.91229453,15,-159.2426751
4,0\H,24,1.0997298,23,90.22552537,15,-45.18323513,0\N,26,1.47103436,25
,33.69965488,24,126.86964257,0\\Version=AM64L-G03RevD.01\State=1-A\HF=
-1050.6488407\MP2=-1053.4025484\RMSD=4.914e-09\Thermal=0.\PG=C01 [X(C1
7H20N1P1)]\\@

291-Me$^+$

1\1\GINC-YANG\SP\RMP2-FC\6-31+G(2d,p)\C18H23N1P1(1+)\CHRISTOPH\27-May-
2010\0\\#p MP2(FC)/6-31+g(2d,p) scf=tight\\xpipmesp_34\\1,1\P\C,1,1.81
948065\H,2,1.09503589,1,108.39502359\H,2,1.09331641,1,113.03509965,3,1
21.49902457,0\H,2,1.09534523,1,108.10345263,3,-117.65243017,0\C,1,4.02
443743,2,90.93478528,4,63.49070822,0\C,6,1.53565247,1,26.48819035,2,-7
0.7118347,0\C,7,2.47592244,6,90.6368209,1,-45.55760006,0\C,8,1.5343617
4,7,91.02705764,6,0.05117606,0\C,6,1.53658139,1,101.93901168,2,174.910
16663,0\H,6,1.09866472,1,90.43649367,2,64.3364429,0\H,6,1.09721329,1,1
33.55552339,2,-50.35474372,0\H,7,1.09354013,6,109.57710916,1,102.91109
233,0\H,7,1.10226844,6,110.20541322,1,-138.36604961,0\H,8,1.10232152,7
,92.03603586,6,-110.12870244,0\H,8,1.09522497,7,142.55453501,6,126.164
96709,0\H,9,1.09884997,8,109.03819432,7,93.79511384,0\H,9,1.0971844,8,
108.36616856,7,-149.75735489,0\H,10,1.10027345,6,109.5802154,1,92.2418
4821,0\H,10,1.09616363,6,110.27399121,1,-150.45944056,0\C,1,4.59926645
,2,105.23803355,7,133.72745963,0\C,21,1.39980254,1,59.8719649,2,-85.95
116799,0\C,22,1.39546112,21,120.10688963,1,-1.72796876,0\C,23,1.409103
98,22,119.7334162,21,-0.52893779,0\C,24,1.40781936,23,120.03859005,22,
0.8209099,0\C,25,1.39691704,24,119.68798268,23,-0.55199734,0\H,21,1.08
694134,1,178.4348,24,178.90215667,0\H,22,1.08668691,21,120.18513199,1,
178.21267341,0\H,23,1.08703395,22,120.17552613,21,179.95795649,0\H,25,
1.08674357,24,120.64003373,23,179.72298653,0\H,26,1.08659885,25,119.64
534968,24,-179.81106249,0\C,1,4.61289686,24,111.06815003,23,156.728766
43,0\C,32,1.39819086,1,60.47926276,24,132.74489401,0\C,33,1.39684774,3
2,120.13502041,1,-1.57014542,0\C,34,1.40847584,33,120.04926066,32,-0.3
839972,0\C,35,1.41150781,34,119.4651808,33,0.43604694,0\C,36,1.3951564
4,35,120.07024832,34,-0.28226982,0\H,32,1.08687825,1,178.58360066,24,-
121.134636,0\H,33,1.08662051,32,120.21929204,1,178.58945302,0\H,34,1.0
8644828,33,118.96717426,32,-179.76803063,0\H,36,1.08746261,35,120.4693
4018,34,-179.26068542,0\H,37,1.0866861,36,119.71786336,35,-179.6922829
1,0\N,7,1.48957332,6,110.10387606,1,-17.2392593,0\\Version=AM64L-G03Re
vD.01\State=1-A\HF=-1090.1052599\MP2=-1093.0028385\RMSD=6.497e-09\Ther
mal=0.\PG=C01 [X(C18H23N1P1)]\\@

292

1\1\GINC-LX64I155\SP\RMP2-FC\6-31+G(2d,p)\C20H25P1\UI271AC\12-Jul-2010
\0\\#MP2(FC)/6-31+g(2d,p) scf=tight\\x8sp_9\\0,1\P\C,1,4.67645778\C,2,
1.39941957,1,61.27325573\C,3,1.39743237,2,120.23203238,1,-0.31924467,0
\C,4,1.40684367,3,120.76656398,2,-0.0435369,0\C,5,1.40695345,4,118.326
83926,3,-0.56787127,0\C,2,1.39751516,1,58.42146535,5,-168.30782132,0\H
,2,1.08791595,1,178.5971095,5,-175.4318496,0\H,3,1.08822529,2,120.0119
5988,1,179.88241865,0\H,4,1.08761137,3,119.33571237,2,-179.06741158,0\
H,6,1.08865748,5,119.28699054,4,-178.83375772,0\H,7,1.08802905,2,120.1
7119187,1,-179.86231627,0\C,1,4.67847568,5,102.43284834,4,36.63864684,
0\C,13,1.39817859,1,57.88869981,5,97.00493198,0\C,14,1.39752874,13,120
.03791235,1,0.41006522,0\C,15,1.40818599,14,121.19695994,13,-0.9021585
1,0\C,16,1.40773208,15,117.97614214,14,1.00692928,0\C,13,1.39804981,1,
61.70474018,16,-1.08950467,0\H,13,1.08779698,1,178.12063055,16,-176.91
60522,0\H,14,1.08794286,13,120.13534843,1,179.78998022,0\H,15,1.088697
55,14,119.60303348,13,178.83891894,0\H,17,1.08719188,16,120.23832469,1
5,179.53980077,0\H,18,1.08818132,13,120.06945466,1,-179.90711868,0\C,1
,1.89442845,16,103.2448863,15,-156.3439437,0\C,24,1.55238923,1,107.728
93485,16,-166.99338029,0\C,25,1.54114992,24,117.32312892,1,-164.027571
01,0\C,26,3.0931913,25,102.03982114,24,-5.30084114,0\C,27,1.54446215,2
6,94.05735026,25,25.41784167,0\C,28,1.5460335,27,119.44205858,26,8.100
19719,0\H,24,1.09773531,1,108.54853484,16,-49.72170152,0\H,25,1.101980
18,24,108.31387459,1,-43.00241616,0\H,25,1.09865808,24,108.70172152,1,
71.71559072,0\H,26,1.10016446,25,106.9079963,24,174.1393591,0\H,27,1.1
0052138,26,68.77286485,25,135.1609491,0\H,27,1.09926106,26,159.3948428
5,25,-147.67081355,0\H,28,1.10034122,27,107.59369611,26,130.42405755,0
\H,28,1.10170195,27,108.1656425,26,-115.97396668,0\H,29,1.09916254,28,
110.19095236,27,58.94515326,0\H,29,1.09956905,28,105.96883396,27,172.3
0488995,0\H,26,1.09871199,25,109.57701636,24,60.58487351,0\C,26,1.5394
6801,25,117.64053678,24,-65.01404957,0\C,41,1.54645316,26,117.31321843
,25,65.49073263,0\H,42,1.09845815,41,110.28688562,26,-81.03677901,0\H,
42,1.09914368,41,107.09796617,26,164.94602518,0\H,41,1.10070582,26,106
.96079809,25,-173.26802339,0\H,41,1.10063094,26,109.32140661,25,-59.01
767641,0\\Version=IA64L-G03RevE.01\State=1-A\HF=-1112.7119874\MP2=-111
5.7490975\RMSD=5.253e-09\Thermal=0.\PG=C01 [X(C20H25P1)]\\@

292-Me$^+$

1\1\GINC-GOLEM\SP\RMP2-FC\6-31+G(2d,p)\C21H28P1(1+)\CHRISTOPH\30-Jul-2
010\0\\#p MP2(FC)/6-31+g(2d,p) scf=tight\\x8mesp_67\\1,1\C\C,1,1.39819
567\C,2,1.39674987,1,120.13547159\C,3,1.40858137,2,120.00064527,1,0.11
854694,0\C,4,1.40994925,3,119.57529502,2,0.07293545,0\C,5,1.39559795,4
,119.96392726,3,-0.15588135,0\H,1,1.08686501,2,119.91697606,3,-179.891
89348,0\H,2,1.08658716,1,120.2609148,6,179.54735503,0\H,3,1.08642283,2
,119.04658013,1,179.67115874,0\H,5,1.08666714,4,120.57653061,3,178.635
76521,0\H,6,1.08665082,5,119.61287665,4,179.74796262,0\C,5,5.42921127,
4,85.90142421,3,149.57767124,0\C,12,1.39912093,5,44.67121897,4,-85.827
40111,0\C,13,1.39588358,12,120.14461894,5,32.96266324,0\C,14,1.4093499
8,13,119.90743932,12,-0.05687126,0\C,15,1.40761684,14,119.7024107,13,0
.41169266,0\C,16,1.39665629,15,119.92942544,14,-0.53731836,0\H,12,1.08
685931,5,149.88320445,4,-156.56513328,0\H,13,1.08665517,12,120.2238711
1,5,-146.92245535,0\H,14,1.08729362,13,119.55724994,12,-179.55929585,0
\H,16,1.08630229,15,120.95768895,14,179.75844863,0\H,17,1.08659996,16,
119.6176054,15,-179.61444882,0\C,4,3.02648711,3,117.25270262,2,-137.18
281629,0\C,23,1.55785367,4,92.71416973,3,14.80389555,0\C,24,1.54227216
,23,116.10662953,4,138.1107927,0\C,25,3.09565051,24,102.36207872,23,5.
82709877,0\C,26,1.54315094,25,94.11007399,24,-26.04308024,0\C,27,1.547
3092,26,119.48231837,25,-8.21083052,0\H,23,1.09847277,4,137.79029705,3
,-107.18630418,0\H,24,1.09897313,23,109.01412344,4,-99.01338637,0\H,24

,1.10010828,23,108.74781539,4,17.29015144,0\H,25,1.09856154,24,106.150
93034,23,-173.8093743,0\H,26,1.09959137,25,69.11958112,24,-135.8154806
6,0\H,26,1.09769219,25,159.7165289,24,146.5170121,0\H,27,1.0987364,26,
107.93170714,25,-129.96974648,0\H,27,1.1016414,26,108.26407067,25,116.
18514004,0\H,28,1.09715737,27,110.77348953,26,-58.48006009,0\H,28,1.09
854518,27,105.94618159,26,-172.41584885,0\H,25,1.0974263,24,109.576480
7,23,-60.60690635,0\C,25,1.53855496,24,117.38650375,23,65.58656054,0\C
,40,1.5464209,25,117.42329948,24,-65.92197482,0\H,41,1.10044467,40,110
.67014457,25,81.5715231,0\H,41,1.09747307,40,106.94086772,25,-164.8829
4379,0\H,40,1.10000939,25,109.45439291,24,59.08599555,0\H,40,1.0989424
5,25,106.56347143,24,173.05839198,0\P,4,1.81104735,3,120.50434375,2,-1
77.11052295,0\C,46,1.82715173,4,109.04092433,3,-24.95054511,0\H,47,1.0
9501975,46,109.36896314,4,-59.71688751,0\H,47,1.09368879,46,111.917832
68,4,61.82825529,0\H,47,1.09579244,46,108.44389736,4,-178.05705119,0\\
Version=AM64L-G03RevD.01\State=1-A\HF=-1152.1658711\MP2=-1155.3491711\
RMSD=4.866e-09\Thermal=0.\PG=C01 [X(C21H28P1)]\\@

293

1\1\GINC-GOLEM\SP\RMP2-FC\6-31+G(2d,p)\C23H24N1O1P1\RAMAN\13-Oct-2008\
0\\#p MP2(FC)/6-31+g(2d,p) scf=tight int=finegrid\\metacatsp004\\0,1\C
\C,1,1.404846\C,2,1.407427,1,118.601\C,3,1.396671,2,120.69,1,-1.013,0\
C,4,1.397864,3,120.201,2,1.665,0\C,5,1.401847,4,119.905,3,-0.312,0\P,2
,1.854013,3,117.17,4,-179.401,0\C,7,1.850278,2,102.21,3,167.594,0\C,8,
1.405882,7,124.57,2,91.32,0\C,9,1.398916,8,120.516,7,179.566,0\C,10,1.
398286,9,120.295,8,0.269,0\C,11,1.398949,10,119.742,9,-0.498,0\C,12,1.
397368,11,119.988,10,-0.149,0\N,6,1.432064,5,119.711,4,-176.673,0\C,14
,1.388908,6,134.032,5,-104.417,0\O,15,1.224209,14,117.448,6,-174.158,0
\C,7,1.850915,8,102.932,9,-14.567,0\C,17,1.407699,7,116.794,8,-92.088,
0\C,18,1.397172,17,120.827,7,-179.839,0\C,19,1.398724,18,120.021,17,0.
889,0\C,20,1.398316,19,119.734,18,-0.218,0\C,21,1.398696,20,120.256,19
,-0.327,0\C,15,1.552931,14,121.365,6,7.089,0\C,23,1.549519,15,110.671,
14,-70.453,0\C,23,1.547198,15,111.595,14,52.619,0\C,23,1.54412,15,107.
049,14,171.447,0\H,3,1.088573,2,119.62,7,0.707,0\H,1,1.08663,2,120.381
,7,-2.921,0\H,5,1.087181,4,120.936,3,178.942,0\H,4,1.087646,3,119.96,2
,-179.21,0\H,14,1.015306,6,115.955,5,80.929,0\H,9,1.087244,8,119.843,7
,-0.243,0\H,13,1.088978,8,119.533,7,-0.569,0\H,10,1.087984,9,119.666,8
,-179.899,0\H,12,1.087837,11,120.166,10,-179.439,0\H,11,1.087725,10,12
0.123,9,179.929,0\H,18,1.088809,19,119.671,20,-178.832,0\H,22,1.087332
,17,119.844,18,-179.244,0\H,19,1.087835,20,120.154,21,-179.548,0\H,21,
1.088045,20,120.052,19,179.727,0\H,20,1.087771,19,120.142,18,179.384,0
\H,26,1.097749,23,109.34,15,179.586,0\H,26,1.095302,23,111.256,15,-60.
445,0\H,26,1.094172,23,111.26,15,59.351,0\H,24,1.097384,23,111.212,15,
-52.297,0\H,24,1.093784,23,112.544,15,69.471,0\H,24,1.098063,23,109.10
6,15,-171.066,0\H,25,1.093828,23,112.654,15,-67.603,0\H,25,1.097729,23
,111.06,15,54.165,0\H,25,1.097842,23,109.202,15,172.787,0\\Version=AM6
4L-G03RevD.01\State=1-A\HF=-1355.0874371\MP2=-1358.8869181\RMSD=9.019e
-09\Thermal=0.\PG=C01 [X(C23H24N1O1P1)]\\@

293-Me$^+$

1\1\GINC-GOLEM\SP\RMP2-FC\6-31+G(2d,p)\C24H27N1O1P1(1+)\RAMAN\08-Nov-2
008\0\\#p MP2(FC)/6-31+g(2d,p) scf=tight int=finegrid\\methylmetacatsp
009\\1,1\C\C,1,1.406146\C,2,1.409724,1,120.065\C,3,1.394972,2,119.759,
1,0.239,0\C,4,1.400406,3,120.051,2,-0.072,0\C,5,1.397839,4,120.292,3,-
0.126,0\P,2,1.809478,3,119.554,4,179.429,0\C,7,1.826393,2,108.318,3,48
.64,0\C,7,1.810011,2,110.433,3,167.341,0\C,9,1.403974,7,118.623,2,102.
186,0\C,10,1.409373,9,119.335,7,-178.546,0\C,11,1.409051,10,118.853,9,
-0.076,0\C,12,1.393451,11,121.09,10,0.237,0\C,13,1.396426,12,120.609,1
1,-0.143,0\N,11,1.398098,10,123.102,9,-179.8,0\C,15,1.393241,11,128.59

1,10,-1.77,0\C,16,1.54712,15,114.772,11,179.058,0\C,17,1.550872,16,108
.924,15,-66.855,0\O,16,1.220515,15,121.879,11,-0.01,0\C,17,1.549191,16
,110.822,15,54.394,0\C,17,1.541014,16,108.057,15,174.217,0\C,7,1.80724
5,9,110.503,10,-20.414,0\C,22,1.40963,7,119.274,9,-69.615,0\C,23,1.394
166,22,119.717,7,178.778,0\C,24,1.400294,23,120.077,22,-0.176,0\C,25,1
.397442,24,120.315,23,-0.044,0\C,26,1.397238,25,120.14,24,0.123,0\H,14
,1.086557,9,121.706,10,-179.392,0\H,10,1.082532,9,121.853,14,-179.899,
0\H,12,1.088536,13,119.356,14,179.802,0\H,13,1.086615,14,119.901,9,179
.728,0\H,15,1.011369,11,115.148,10,179.041,0\H,23,1.08773,22,120.911,2
7,-178.996,0\H,27,1.086409,22,120.477,23,179.785,0\H,24,1.086589,23,11
9.719,22,179.956,0\H,26,1.086528,25,120.242,24,-179.765,0\H,25,1.08686
9,24,119.796,23,-179.891,0\H,3,1.087763,2,120.888,1,-179.289,0\H,1,1.0
86469,2,120.381,3,179.935,0\H,4,1.086651,3,119.723,2,179.852,0\H,6,1.0
86632,5,120.205,4,-179.945,0\H,5,1.086935,4,119.806,3,179.889,0\H,18,1
.096602,17,109.473,16,-176.828,0\H,18,1.096633,17,111.151,16,-57.647,0
\H,18,1.097808,17,112.586,16,63.741,0\H,20,1.096642,17,111.403,16,55.9
21,0\H,20,1.096373,17,109.297,16,175.044,0\H,20,1.098587,17,113.167,16
,-66.167,0\H,21,1.095402,17,111.233,16,-61.429,0\H,21,1.094335,17,111.
202,16,58.63,0\H,21,1.096343,17,109.516,16,178.716,0\H,8,1.09463,7,109
.725,9,176.801,0\H,8,1.094479,7,110.053,9,-63.269,0\H,8,1.094648,7,109
.925,9,56.958,0\\Version=AM64L-G03RevD.01\State=1-A\HF=-1394.5548177\M
P2=-1398.4944499\RMSD=7.838e-09\Thermal=0.\PG=C01 [X(C24H27N1O1P1)]\\@

294

1\1\GINC-EDDY\SP\RMP2-FC\6-31+G(2d,p)\C22H25P1\CHRISTOPH\24-Aug-2010\0
\\#p MP2(FC)/6-31+g(2d,p) scf=tight\\xadsp_5\\0,1\P\C,1,4.67717097\C,2
,1.39976958,1,61.42071103\C,3,1.39729231,2,120.24379705,1,0.73199694,0
\C,4,1.40950728,3,120.81160581,2,-0.06833848,0\C,5,1.406978,4,118.1659
9111,3,0.68802972,0\C,2,1.39712482,1,58.27852003,5,158.6497419,0\H,2,1
.08791286,1,178.358084,5,-179.61171228,0\H,3,1.08816129,2,120.02772318
,1,-179.35928475,0\H,4,1.08707892,3,119.4316313,2,178.69994484,0\H,6,1
.0884253,5,119.19000956,4,179.00176087,0\H,7,1.08795024,2,120.17631922
,1,179.48966301,0\C,1,4.68861173,5,102.4635376,4,-44.08655377,0\C,13,1
.39799298,1,58.65830579,5,-34.44936012,0\C,14,1.39672349,13,120.407892
56,1,2.78252261,0\C,15,1.41138713,14,121.36259306,13,0.34880771,0\C,16
,1.40875294,15,117.33010189,14,-1.4955178,0\C,13,1.39670747,1,60.47980
998,5,143.36887053,0\H,13,1.08762351,1,177.56677266,5,-103.89694918,0\
H,14,1.08817781,13,120.16174674,1,-177.21590909,0\H,15,1.08700029,14,1
18.88379155,13,-179.8281509,0\H,17,1.08518107,16,120.39969606,15,-178.
13054425,0\H,18,1.08817339,13,120.13863183,1,177.33982285,0\C,1,4.3265
6889,5,96.01340118,4,56.32276249,0\C,24,1.54451316,1,97.24647767,5,57.
86090507,0\C,25,1.54498417,24,109.3755555,1,40.60170234,0\C,26,1.54774
494,25,109.32148759,24,-59.80556137,0\C,27,1.55770998,26,110.6042053,2
5,59.87187721,0\C,28,1.54875284,27,108.25937939,26,-59.15872996,0\H,24
,1.09975934,1,131.60930053,5,-66.695265,0\H,25,1.10010437,24,110.05437
32,1,-80.48183867,0\H,25,1.10037681,24,110.14607537,1,161.69768803,0\H
,26,1.09968501,25,109.69309768,24,-178.99054105,0\H,27,1.10106486,26,1
09.86715515,25,-178.56309544,0\H,27,1.09754112,26,109.33066343,25,-61.
44935846,0\H,29,1.09878607,28,109.67008553,27,-61.12736973,0\H,29,1.09
779612,28,110.82608115,27,-179.02715503,0\C,26,1.54353531,25,109.46141
595,24,60.5208467,0\H,38,1.10022215,26,110.18715276,25,178.54328676,0\
H,38,1.10030654,26,110.16997991,25,60.65778003,0\C,24,1.54522035,1,97.
39135267,5,168.66085402,0\H,41,1.10069373,24,110.11317744,1,80.4078093
1,0\H,41,1.10033038,24,110.04961706,1,-162.00896555,0\C,28,1.55799023,
27,107.34136607,26,59.24274153,0\H,44,1.09795666,28,111.10743838,27,17
8.9217148,0\H,44,1.09967611,28,109.44452612,27,61.58554635,0\C,38,1.54
341107,26,109.17062083,25,-60.48873799,0\H,47,1.09970217,38,109.738119
15,26,-179.34191128,0\\Version=AM64L-G03RevD.01\State=1-A\HF=-1188.473

3945\MP2=-1191.7933392\RMSD=5.855e-09\Thermal=0.\PG=C01 [X(C22H25P1)]\
\@

294-Me^{+}

1\1\GINC-SOLARIS\SP\RMP2-FC\6-31+G(2d,p)\C23H28P1(1+)\CHRISTOPH\24-Aug
-2010\0\\#p MP2(FC)/6-31+g(2d,p) scf=tight\\xadmesp_4\\1,1\C\C,1,1.397
9914\C,2,1.39634154,1,120.20252316\C,3,1.41014992,2,120.1575419,1,0.04
901078,0\C,4,1.40858303,3,119.26631604,2,0.55584077,0\C,5,1.39635462,4
,120.13363857,3,-0.81622943,0\H,1,1.08684758,2,120.03395166,3,179.6931
733,0\H,2,1.08669831,1,120.27189174,6,179.79514559,0\H,3,1.08640804,2,
119.30909394,1,-179.57334054,0\H,5,1.08442317,4,121.0660168,3,179.3335
9996,0\H,6,1.08667473,5,119.51393015,4,-179.51072632,0\C,3,5.21252423,
2,140.33745552,1,-143.57630575,0\C,12,1.39954793,3,49.39711947,2,69.04
718997,0\C,13,1.39572018,12,120.16626559,3,36.49774108,0\C,14,1.410311
2,13,119.98429351,12,0.11306978,0\C,15,1.4073282,14,119.55268065,13,-0
.08402996,0\C,16,1.39726146,15,120.02645327,14,-0.11039681,0\H,12,1.08
686111,3,148.45029546,2,-12.92889402,0\H,13,1.08669233,12,120.20487239
,3,-143.12344551,0\H,14,1.08715722,13,119.45972046,12,-178.61947762,0\
H,16,1.08647972,15,120.97774223,14,-179.88895765,0\H,17,1.08663815,16,
119.59439614,15,-179.59993222,0\C,5,4.81485333,4,85.37666631,3,-146.38
671063,0\C,23,1.54456758,5,84.07516637,4,-136.81436039,0\C,24,1.544315
94,23,109.59694849,5,75.43585414,0\C,25,1.55021384,24,109.62222754,23,
-60.95872468,0\C,26,1.55901726,25,109.32817748,24,59.16721871,0\C,23,1
.54930514,5,29.05011475,4,14.55123633,0\H,23,1.09777731,5,105.52637957
,4,114.17749803,0\H,24,1.09819009,23,109.92051922,5,-163.76402288,0\H,
24,1.10011253,23,110.31920702,5,-46.27344117,0\H,25,1.09786951,24,109.
91761263,23,-179.5932074,0\H,26,1.09865395,25,109.2834973,24,179.86121
737,0\H,26,1.09736333,25,109.31293652,24,-63.21242192,0\H,28,1.0973353
8,23,109.856372,5,31.91704655,0\H,28,1.09913085,23,109.51611672,5,149.
52621788,0\C,25,1.54318219,24,109.92685575,23,59.27663148,0\H,37,1.099
71934,25,110.31787958,24,178.74082878,0\H,37,1.09825929,25,109.9591206
3,24,61.12476671,0\C,23,1.54373643,5,133.75149795,4,-24.88861328,0\H,4
0,1.09953444,23,110.29668918,5,79.85081556,0\H,40,1.09830488,23,109.99
900613,5,-162.46269045,0\C,27,1.56134385,26,108.32576155,25,60.6043447
,0\H,43,1.09705464,27,110.24879918,26,179.46283303,0\H,43,1.10020939,2
7,110.55350786,26,60.82839559,0\C,37,1.5431485,25,109.25897486,24,-59.
6655586,0\H,46,1.09784217,37,109.9704834,25,-178.88402237,0\P,15,1.814
2799,14,119.64223286,13,177.16143943,0\C,48,1.82898198,15,108.39529318
,14,164.45645956,0\H,49,1.0945018,48,108.80341021,15,-170.80441147,0\H
,49,1.09467545,48,111.60225839,15,68.84972756,0\H,49,1.09498787,48,109
.22082354,15,-52.50888686,0\\Version=AM64L-G03RevD.01\State=1-A\HF=-12
27.9314982\MP2=-1231.3951729\RMSD=4.643e-09\Thermal=0.\PG=C01 [X(C23H2
8P1)]\\@

294-BH^{+}

1\1\GINC-AZAZEL\SP\RMP2-FC\6-31+G(2d,p)\C35H36P1(1+)\CHRISTOPH\01-Sep-
2010\0\\#p MP2(FC)/6-31+g(2d,p) scf=tight\\xadbhsp_16\\1,1\C\C,1,1.397
40627\C,2,1.39722193,1,120.28398445\C,3,1.40883855,2,120.38941316,1,0.
2618716,0\C,4,1.40901215,3,118.79347823,2,-1.09365358,0\C,5,1.39619576
,4,120.54300281,3,1.22024252,0\H,1,1.08686768,2,120.12608695,3,-179.81
339609,0\H,2,1.08679156,1,120.24615763,6,-179.50425419,0\H,3,1.0856644
7,2,119.24321644,1,179.98911,0\H,5,1.0856805,4,120.89969074,3,-178.308
96394,0\H,6,1.08681166,5,119.57100358,4,179.57442173,0\C,3,5.10978812,
2,139.08951571,1,146.4677878,0\C,12,1.39804306,3,46.82298472,2,-68.732
32652,0\C,13,1.39632325,12,120.30160994,3,-38.49532396,0\C,14,1.412497
4,13,120.28314885,12,-0.0717854,0\C,15,1.40875219,14,118.91092211,13,0
.05914524,0\C,16,1.39684024,15,120.38226595,14,0.06316725,0\H,12,1.086
9633,3,146.6546073,2,11.16546495,0\H,13,1.08677053,12,120.30029412,3,1

41.40781805,0\H,14,1.08530688,13,119.1676441,12,179.42833978,0\H,16,1.
08302594,15,120.96327223,14,179.82379366,0\H,17,1.08672972,16,119.4395
5486,15,179.59831019,0\C,16,4.82002657,15,94.37154402,14,-179.94944079
,0\C,23,1.54293027,16,81.75769143,15,-114.07360269,0\C,24,1.54331735,2
3,109.30406127,16,74.20392273,0\C,25,1.5492392,24,109.64535365,23,-61.
01288861,0\C,26,1.5597334,25,109.65571038,24,59.77497895,0\C,23,1.5507
771,16,31.2687883,15,41.01859042,0\H,23,1.09810386,16,106.84723597,15,
137.51846384,0\H,24,1.10022194,23,110.35545559,16,-47.23108099,0\H,24,
1.09846405,23,110.05994433,16,-164.86299274,0\H,25,1.09808362,24,109.9
6392089,23,-179.63092535,0\H,26,1.0979478,25,109.40628592,24,-179.2730
6397,0\H,26,1.09743892,25,109.53041283,24,-62.06128583,0\H,28,1.095394
52,23,109.06376733,16,37.35386341,0\H,28,1.09387067,23,109.27245917,16
,153.74775767,0\C,25,1.54351136,24,109.89953988,23,59.2990151,0\H,37,1
.09848137,25,110.03152295,24,61.23440473,0\H,37,1.09959109,25,110.2828
4745,24,178.98765509,0\C,23,1.54253597,16,134.02938608,15,-4.26579881,
0\H,40,1.09961983,23,110.24255514,16,83.64263414,0\H,40,1.09855085,23,
110.17270917,16,-158.4761877,0\C,27,1.5614968,26,108.63427942,25,59.52
013878,0\H,43,1.09997897,27,110.23142576,26,-179.50327826,0\H,43,1.096
29576,27,110.69881862,26,60.97408279,0\C,40,1.54187632,23,108.97208505
,16,-37.70522521,0\H,46,1.09822338,40,110.04361732,23,178.67153084,0\P
,15,1.82599465,14,117.75136791,13,-178.42710858,0\C,48,1.90412533,15,1
10.21120439,14,69.41246552,0\H,49,1.09790949,48,101.45071671,15,-170.2
9015943,0\C,49,4.34671141,48,115.45459581,15,77.06899222,0\C,51,1.3980
9817,49,58.78012852,48,125.2411995,0\C,52,1.39733702,51,120.06809149,4
9,-0.66499668,0\C,53,1.40542347,52,120.88761457,51,0.00916242,0\C,54,1
.40557086,53,118.5620024,52,0.22067267,0\C,51,1.3975845,49,60.78474142
,48,-54.27437217,0\H,51,1.0868867,49,178.82576921,48,157.18137047,0\H,
52,1.08706947,51,120.23791304,49,179.1647372,0\H,53,1.08894407,52,119.
42140648,51,179.3912646,0\H,55,1.08640065,54,120.60612117,53,-179.3738
3715,0\H,56,1.08725689,51,120.09073229,49,-179.37279946,0\C,49,4.35641
593,48,114.44999299,15,-56.93534333,0\C,62,1.39738796,49,59.33034421,4
8,-93.2001837,0\C,63,1.3970609,62,120.1092159,49,0.65487567,0\C,64,1.4
0585202,63,121.01467678,62,0.25883723,0\C,65,1.40577274,64,118.2999662
6,63,-1.0318727,0\C,66,1.39727257,65,120.68979185,64,1.06438161,0\H,62
,1.08697752,49,178.93745552,48,-160.9185442,0\H,63,1.0870519,62,120.27
203337,49,-178.55040396,0\H,64,1.0867043,63,118.91411283,62,-178.65988
884,0\H,66,1.08473109,65,120.38605448,64,-177.59197422,0\H,67,1.08719999,
66,119.42619999,65,-179.56131614,0\\Version=AM64L-G03RevD.01\State=
1-A\HF=-1687.0327683\MP2=-1692.2026953\RMSD=8.828e-09\Thermal=0.\PG=C0
1 [X(C35H36P1)]\\@

295

1\1\GINC-PHOENIX\SP\RMP2-FC\6-31+G(2d,p)\C21H21P1\CHRISTOPH\23-Jul-201
1\0\\#p MP2(FC)/6-31+g(2d,p) scf=tight\\tol3sp_4\\0,1\P\C,1,4.69217448
\C,2,1.40491306,1,57.52241238\C,3,1.39468719,2,121.00797892,1,0.093451
85,0\C,4,1.40811273,3,121.02388549,2,-0.99020948,0\C,5,1.40450676,4,11
8.00705461,3,0.90596381,0\C,6,1.39880419,5,120.72987669,4,-0.31272048,
0\H,3,1.0893539,2,119.50061907,1,179.45032387,0\H,4,1.08904015,3,119.4
6503263,2,178.9425355,0\H,6,1.08731549,5,119.84357027,4,179.39284115,0
\H,7,1.08919918,6,119.36183849,5,179.86966853,0\C,1,4.69164199,5,102.6
6864915,4,-160.87538088,0\C,12,1.40289586,1,60.46866829,5,-92.5456168,
0\C,13,1.3978243,12,121.29063483,1,0.27718083,0\C,14,1.40546193,13,120
.70255346,12,-0.11838416,0\C,15,1.4072017,14,118.01122686,13,-0.450708
27,0\C,16,1.39546052,15,121.03367869,14,1.04042858,0\H,13,1.08931583,1
2,119.39287302,1,-179.91695863,0\H,14,1.08749826,13,119.42544704,12,17
9.94602918,0\H,16,1.08897194,15,119.53879838,14,-178.96719493,0\H,17,1
.0891363,16,119.50976497,15,179.52293488,0\C,1,4.69160088,15,102.95146
983,14,16.24560977,0\C,22,1.40423452,1,57.4997693,15,86.44012092,0\C,2

3,1.39536722,22,121.00422619,1,0.13322531,0\C,24,1.40771102,23,121.037
47667,22,-1.11361021,0\C,25,1.40459787,24,118.00002128,23,1.06355131,0
\C,26,1.39819746,25,120.72554651,24,-0.47225403,0\H,23,1.08918396,22,1
19.49526796,1,179.56586536,0\H,24,1.08903547,23,119.40732785,22,178.98
655698,0\H,26,1.08739337,25,119.86828839,24,179.57626263,0\H,27,1.0893
7074,26,119.30941801,25,-179.92524624,0\C,2,1.51389269,1,177.92273022,
25,121.30162873,0\H,32,1.09752568,2,111.3701103,1,-80.18992784,0\H,32,
1.0989025,2,111.18848052,1,39.1890452,0\H,32,1.09579817,2,111.39889624
,1,159.19197295,0\C,12,1.51390626,1,177.9459789,25,-123.77625455,0\H,3
6,1.09940201,12,111.1182686,1,47.79981104,0\H,36,1.09645605,12,111.432
88677,1,167.39421691,0\H,36,1.0964545,12,111.4252063,1,-71.79688555,0\
C,22,1.51382284,1,177.84744015,25,-148.5613974,0\H,40,1.09663078,22,11
1.42463548,1,-77.69113423,0\H,40,1.09940988,22,111.13692855,1,41.85531
548,0\H,40,1.09629214,22,111.40435246,1,161.51191905,0\\Version=AM64L-
G03RevD.01\State=1-A\HF=-1148.2764067\MP2=-1151.3991652\RMSD=6.492e-09
\Thermal=0.\PG=C01 [X(C21H21P1)]\\@

295-Me⁺

1\1\GINC-BORIX\SP\RMP2-FC\6-31+G(2d,p)\C22H24P1(1+)\CHRISTOPH\26-Oct-2
011\0\\#p MP2(FC)/6-31+g(2d,p) scf=tight\\tol3mesp_cart_1\\1,1\C\C,1,1
.40856535\C,2,1.39163201,1,121.1957706\C,3,1.41068309,2,119.9854014,1,
0.03173749,0\C,4,1.40546738,3,119.37812951,2,0.38773696,0\C,5,1.396387
94,4,119.89523273,3,-0.3495003,0\H,2,1.08781865,1,119.53427885,6,179.6
8601071,0\H,3,1.08790531,2,119.11321014,1,179.50389157,0\H,5,1.0868620
4,4,120.43780977,3,179.8458615,0\H,6,1.08762439,5,119.17730109,4,-179.
89100997,0\C,4,5.53742203,3,94.96808682,2,131.62322351,0\C,11,1.408074
29,4,76.29988969,3,112.790254,0\C,12,1.3918829,11,121.19821776,4,4.120
11596,0\C,13,1.41050672,12,119.97918346,11,-0.15229646,0\C,14,1.405716
4,13,119.36718558,12,0.32438596,0\C,15,1.39606648,14,119.90695832,13,-
0.31495469,0\H,12,1.0878437,11,119.51728488,4,-175.63588846,0\H,13,1.0
879084,12,119.09862561,11,179.59819312,0\H,15,1.08668088,14,120.448065
99,13,-179.85533325,0\H,16,1.08756851,15,119.16935851,14,-179.60338327
,0\C,5,5.2134584,4,95.18836477,3,-168.96281368,0\C,21,1.40872879,5,57.
345652,4,74.94728056,0\C,22,1.39142084,21,121.1860728,5,-38.46490142,0
\C,23,1.41100953,22,119.99205596,21,-0.0243813,0\C,24,1.4053222,23,119
.36056888,22,0.2704971,0\C,25,1.39663307,24,119.90163984,23,-0.2508120
9,0\H,22,1.08775931,21,119.53673907,5,141.63349585,0\H,23,1.08785845,2
2,119.10392596,21,179.4608053,0\H,25,1.08667983,24,120.43699252,23,179
.98277039,0\H,26,1.08759763,25,119.17425762,24,-179.88834624,0\C,11,1.
51069103,4,162.85772976,3,-57.39419577,0\H,31,1.09855735,11,110.535642
51,4,-114.55912868,0\H,31,1.09461967,11,111.54274795,4,5.24108541,0\H,
31,1.09613747,11,111.26668665,4,126.39490584,0\P,4,1.80532207,3,119.85
091249,2,179.39741121,0\C,35,1.82707338,4,108.33598569,3,48.07545285,0
\H,36,1.09463854,35,109.92155746,4,58.27640899,0\H,36,1.09468339,35,10
9.90435354,4,178.27514854,0\H,36,1.09463934,35,109.90915695,4,-61.7264
745,0\C,21,1.51082994,5,147.76317295,4,171.44837405,0\H,40,1.09725388,
21,111.03696813,5,-21.10566079,0\H,40,1.09427752,21,111.53132013,5,99.
65952776,0\H,40,1.09782885,21,110.77964125,5,-140.07115287,0\C,1,1.511
14917,6,121.23675333,5,-178.90656489,0\H,44,1.09877925,1,110.67369276,
6,106.63795022,0\H,44,1.09670342,1,111.16955977,6,-134.29031199,0\H,44
,1.09461757,1,111.52605481,6,-13.35587705,0\\Version=AM64L-G03RevD.01\
State=1-A\HF=-1187.7354701\MP2=-1191.0005934\RMSD=3.971e-09\Thermal=0.
\PG=C01 [X(C22H24P1)]\\@

295-BH⁺

1\1\GINC-YIN\SP\RMP2-FC\6-31+G(2d,p)\C34H32P1(1+)\CHRISTOPH\08-Nov-201
0\0\\#p MP2(FC)/6-31+g(2d,p) scf=tight\\k4bhsp_5\\1,1\C\H,1,1.09933916
\C,1,4.34373164,2,105.43156046\C,3,1.39822594,1,60.70115677,2,-166.603

01051,0\C,4,1.3987766,3,120.35015031,1,0.2637537,0\C,5,1.40707863,4,12
0.46391485,3,0.13601062,0\C,6,1.40447872,5,118.63684534,4,-0.0303131,0
\C,7,1.39729868,6,120.84225846,5,-0.19002071,0\H,3,1.08697276,1,179.05
128578,6,-173.71140838,0\H,4,1.08729981,3,120.13019104,1,-179.66095101
,0\H,5,1.08686244,4,119.12996677,3,179.25684347,0\H,7,1.08841557,6,119
.69480978,5,-179.8349436,0\H,8,1.08710424,7,119.65611343,6,-179.772679
17,0\P,1,1.90113341,6,113.10416213,5,-57.72672918,0\C,1,4.35803087,6,1
15.79868604,5,75.66381214,0\C,15,1.39746424,1,60.24103264,6,-33.993889
68,0\C,16,1.39807358,15,120.45094684,1,-0.38661101,0\C,17,1.40548538,1
6,120.62397796,15,-0.25075799,0\C,18,1.40676123,17,118.36662795,16,1.1
2348772,0\C,19,1.3966328,18,120.94503195,17,-1.17242368,0\H,15,1.08703
665,1,179.13819849,6,93.2626979,0\H,16,1.08728852,15,120.15325569,1,17
8.81258929,0\H,17,1.08483916,16,118.96574777,15,178.21031164,0\H,19,1.
08678528,18,119.95868391,17,178.17333483,0\H,20,1.08718765,19,119.5813
8125,18,179.56009564,0\C,14,4.64730188,1,107.27158713,6,-168.32509465,
0\C,26,1.4016852,14,59.46720465,1,-123.4946952,0\C,27,1.39760284,26,12
1.40732473,14,0.41491836,0\C,28,1.40356474,27,120.13433563,26,0.133715
26,0\C,29,1.41181784,28,118.92730442,27,-0.84056483,0\C,30,1.39158657,
29,120.30372982,28,0.80997993,0\H,27,1.08769983,26,119.51743112,14,-17
9.33310501,0\H,28,1.08582563,27,119.36060633,26,-179.35987931,0\H,30,1
.08844145,29,120.68872418,28,-177.87023255,0\H,31,1.08812277,30,119.30
905664,29,-179.72017835,0\C,14,4.64242637,1,112.37578837,6,69.98943972
,0\C,36,1.40686843,14,59.17109022,1,57.50708734,0\C,37,1.3933854,36,12
1.41349133,14,-0.03666837,0\C,38,1.41226728,37,119.94396381,36,0.00798
456,0\C,39,1.40714509,38,119.09216973,37,0.50740684,0\C,40,1.39535937,
39,120.12663201,38,-0.49400711,0\H,37,1.08807219,36,119.52817303,14,17
9.66506045,0\H,38,1.0857702,37,119.30394949,36,179.73569017,0\H,40,1.0
8584148,39,120.39265895,38,179.32999932,0\H,41,1.08783476,40,119.09475
692,39,179.69996471,0\C,14,4.6576429,1,112.344159,6,-53.05229163,0\C,4
6,1.4047074,14,59.57946503,1,48.94289339,0\C,47,1.39441021,46,121.5756
0938,14,-0.80423952,0\C,48,1.41106362,47,120.12251492,46,-0.20723089,0
\C,49,1.41042906,48,118.63096875,47,1.06169524,0\C,50,1.39411781,49,12
0.41384721,48,-0.96525313,0\H,47,1.08793715,46,119.50687689,14,179.029
87465,0\H,48,1.0838102,47,118.91264364,46,179.02071985,0\H,50,1.085394
3,49,120.49897461,48,178.69204432,0\H,51,1.08796619,50,119.06263745,49
,179.78821398,0\C,46,1.51054163,14,178.9670523,1,-82.06204991,0\H,56,1
.09917992,46,110.36735396,14,40.24187964,0\C,36,1.51068222,14,178.7914
6556,1,-28.1758849,0\H,58,1.09569562,36,111.38460758,14,122.88472842,0
\C,26,1.51079867,14,179.07188759,1,27.16875252,0\H,60,1.09451867,26,11
1.53347164,14,-163.74239727,0\H,56,1.09523432,46,111.49870485,14,-78.9
8544566,0\H,56,1.09505285,46,111.49449312,14,159.50946753,0\H,58,1.099
11529,36,110.44520392,14,3.84542216,0\H,58,1.09473075,36,111.57039069,
14,-115.72348635,0\H,60,1.09644005,26,111.24792744,14,75.17444912,0\H,
60,1.09850836,26,110.62651726,14,-43.82376505,0\\Version=AM64L-G03RevD
.01\State=1-A\HF=-1646.8419855\MP2=-1651.810409\RMSD=9.377e-09\Thermal
=0.\PG=C01 [X(C34H32P1)]\\@

295-TT$^+$

1\1\GINC-BORIX\SP\RMP2-FC\6-31+G(2d,p)\C40H36P1(1+)\CHRISTOPH\06-Oct-2
011\0\\#p MP2(FC)/6-31+g(2d,p) scf=tight\\k4ttsp_1_3\\1,1\P\C,1,4.6679
3279\C,2,1.40658183,1,59.32071198\C,3,1.3931696,2,121.5775269,1,0.3246
194,0\C,4,1.41297502,3,120.19551633,2,0.44453504,0\C,5,1.40741598,4,11
8.53949772,3,-0.87284504,0\C,6,1.39639834,5,120.46379041,4,0.74522218,
0\H,3,1.08812603,2,119.51392383,1,-179.58000518,0\H,4,1.084357,3,118.9
4011494,2,-179.41825598,0\H,6,1.08485425,5,120.59233932,4,-179.2973714
1,0\H,7,1.08788455,6,118.99770307,5,179.79749813,0\C,1,4.66753109,5,10
6.46531007,4,-179.0624234,0\C,12,1.40592456,1,59.31157947,5,68.6657611
,0\C,13,1.39381597,12,121.55651136,1,0.2618621,0\C,14,1.41228447,13,12

0.19838114,12,0.4447111,0\C,15,1.4079602,14,118.56624388,13,-0.9012093
2,0\C,16,1.39568028,15,120.44042369,14,0.79490488,0\H,13,1.08799818,12
,119.51467048,1,-179.67876323,0\H,14,1.08445183,13,118.92944025,12,-17
9.47254819,0\H,16,1.08493419,15,120.56724161,14,-179.29925278,0\H,17,1
.08804346,16,118.99279856,15,179.75311772,0\C,1,4.66795375,15,106.3384
3462,14,-179.6570931,0\C,22,1.40569933,1,59.34427037,15,69.50170822,0\
C,23,1.3938931,22,121.56649732,1,0.35695237,0\C,24,1.41232588,23,120.2
1194324,22,0.46864928,0\C,25,1.4083457,24,118.53305231,23,-0.89670974,
0\C,26,1.39555033,25,120.45309803,24,0.76892257,0\H,23,1.08805179,22,1
19.52830331,1,-179.53338229,0\H,24,1.08437255,23,118.89086173,22,-179.
43029787,0\H,26,1.08487453,25,120.58296021,24,-179.24552344,0\H,27,1.0
8799115,26,118.9972746,25,179.79628509,0\C,1,1.99520362,15,111.9227725
8,14,-55.6370477,0\C,32,4.38986838,1,107.86892757,15,54.47341015,0\C,3
3,1.39512071,32,59.49569585,1,-125.17108086,0\C,34,1.39974429,33,120.6
1298059,32,0.48673852,0\C,35,1.40560058,34,121.13892412,33,0.13578801,
0\C,36,1.41200479,35,117.4982209,34,-2.02847605,0\C,37,1.39667283,36,1
21.28779705,35,2.50849027,0\H,33,1.08704385,32,179.7909356,1,144.19545
446,0\H,34,1.08720622,33,120.22747812,32,-179.27368492,0\H,35,1.083558
81,34,118.47250459,33,-179.19324699,0\H,37,1.084,36,120.30707249,35,-1
76.17394514,0\H,38,1.08738527,37,119.34536078,36,179.70487242,0\C,32,4
.39006551,1,107.84948256,15,174.38864677,0\C,44,1.3952084,32,59.496554
77,1,-126.0534947,0\C,45,1.39974373,44,120.61696249,32,0.44221888,0\C,
46,1.40573504,45,121.13113906,44,0.14994998,0\C,47,1.41216213,46,117.4
9663148,45,-1.99935095,0\C,48,1.39662215,47,121.28923125,46,2.45339079
,0\H,44,1.08704742,32,179.83858326,1,143.54976153,0\H,45,1.08721551,44
,120.22290411,32,-179.2978773,0\H,46,1.08356097,45,118.49154401,44,-17
9.19117669,0\H,48,1.08396248,47,120.31871444,46,-176.29271552,0\H,49,1
.08737492,48,119.34323766,47,179.69888133,0\C,32,4.38955431,1,107.8841
8404,15,-65.59400915,0\C,55,1.39523825,32,59.50870699,1,-125.48983271,
0\C,56,1.39973881,55,120.60612273,32,0.45858468,0\C,57,1.40561406,56,1
21.12747672,55,0.11495071,0\C,58,1.41175902,57,117.51709583,56,-1.9715
209,0\C,59,1.39648187,58,121.28333266,57,2.45588867,0\H,55,1.08705912,
32,179.83186698,1,139.53886755,0\H,56,1.08719455,55,120.22981833,32,-1
79.31391165,0\H,57,1.08363082,56,118.46884526,55,-179.2353173,0\H,59,1
.08388063,58,120.2919341,57,-176.29254122,0\H,60,1.08737893,59,119.345
59676,58,179.69519229,0\C,2,1.51108511,1,178.85173018,15,85.58933899,0
\H,66,1.09917314,2,110.53364252,1,20.19125895,0\H,66,1.09607963,2,111.
30502638,1,139.12956036,0\H,66,1.09440558,2,111.60442274,1,-99.5402054
6,0\C,12,1.51114635,1,178.65973627,15,-149.73374129,0\H,70,1.09943571,
12,110.44208818,1,16.07739285,0\H,70,1.09511239,12,111.48818308,1,135.
31477293,0\H,70,1.09506098,12,111.50965654,1,-103.19999639,0\C,22,1.51
088122,1,178.55672707,15,-35.27064812,0\H,74,1.09544283,22,111.4553905
8,1,134.9660886,0\H,74,1.09532661,22,111.45297026,1,-103.74254599,0\H,
74,1.09889853,22,110.52677856,1,15.62320252,0\\Version=AM64L-G03RevD.0
1\State=1-A\HF=-1876.3689765\MP2=-1882.2158551\RMSD=5.745e-09\Thermal=
0.\PG=C01 [X(C40H36P1)]\\@

296

1\1\GINC-CIPCLU06\SP\RMP2-FC\6-31+G(2d,p)\C16H18N1P1\C2175\18-May-2010
\0\\#p MP2(FC)/6-31+G(2d,p) scf=tight\\xpyrsp_11\\0,1\C\C,1,1.39875478
\C,2,1.39754454,1,120.26963844\C,3,1.40788049,2,120.79084683,1,0.27103
342,0\C,4,1.40653839,3,118.36828403,2,-1.12799033,0\C,1,1.3981436,2,11
9.48278521,3,0.38076206,0\H,1,1.08779374,6,120.28209719,5,-179.8722183
1,0\H,2,1.08814301,1,120.09526365,6,-179.74990039,0\H,3,1.08824644,2,1
19.33295972,1,179.73909391,0\H,5,1.08756122,4,119.0515233,3,-178.68818
174,0\H,6,1.08829783,1,120.05682081,2,179.70872408,0\C,3,5.35424109,2,
139.72054664,1,127.17545314,0\C,12,1.39993505,3,45.55892556,2,-51.5431
0019,0\C,13,1.39730449,12,120.15274411,3,-33.60856351,0\C,14,1.4085143

7,13,120.77881699,12,0.06355798,0\C,15,1.40694463,14,118.31324775,13,0
.12583423,0\C,12,1.39752375,3,80.28433126,2,157.4879574,0\H,12,1.08796
608,3,149.83151288,2,21.53114307,0\H,13,1.08821117,12,120.00782682,3,1
46.33635309,0\H,14,1.08778207,13,119.5633248,12,179.14131988,0\H,16,1.
08899359,15,119.23052976,14,179.21673176,0\H,17,1.08800176,12,120.1473
7997,3,-156.00403907,0\P,4,1.84743975,3,120.63540534,2,-173.25512637,0
\C,23,2.83083226,4,89.26372493,3,-132.99058053,0\C,24,1.54420157,23,13
2.91729171,4,-111.41807877,0\C,25,1.55896851,24,104.64294162,23,-30.78
966491,0\C,26,1.54912509,25,104.892569,24,4.1808934,0\H,24,1.09526915,
23,91.49193591,4,12.79100316,0\H,24,1.10448134,23,97.51029088,4,121.14
224874,0\H,25,1.0958804,24,110.02888008,23,87.7056794,0\H,25,1.0957226
2,24,111.65103084,23,-152.91904755,0\H,26,1.09530726,25,110.5857228,24
,123.5569759,0\H,26,1.0955776,25,112.24371394,24,-116.46192655,0\H,27,
1.10501037,26,109.93091016,25,-99.13776242,0\H,27,1.09527845,26,113.50
028285,25,140.01005216,0\N,24,1.46832735,23,29.898344,4,-118.15575626,
0\\Version=AM64L-G03RevD.01\State=1-A\HF=-1011.6043454\MP2=-1014.20121
4\RMSD=4.442e-09\Thermal=0.\PG=C01 [X(C16H18N1P1)]\\@

296-Me$^+$

1\1\GINC-YANG\SP\RMP2-FC\6-31+G(2d,p)\C17H21N1P1(1+)\CHRISTOPH\27-May-
2010\0\\#p MP2(FC)/6-31+g(2d,p) scf=tight\\xpyrmesp_21\\1,1\C\C,1,1.39
76837\C,2,1.39772527,1,120.15657304\C,3,1.4060561,2,119.66567049,1,-0.
00842745,0\C,4,1.41038293,3,120.04773973,2,0.20318281,0\C,5,1.39429656
,4,119.79061525,3,-0.2388177,0\H,1,1.08692924,2,119.88759613,3,179.949
79046,0\H,2,1.08659028,1,120.20321956,6,179.76669722,0\H,3,1.08643288,
2,119.81776315,1,179.85641371,0\H,5,1.08811533,4,120.59537456,3,179.21
474535,0\H,6,1.08665322,5,119.79416526,4,-179.93282364,0\C,3,5.2114771
4,2,145.05667081,1,-174.33250636,0\C,12,1.39792276,3,74.18267859,2,-12
4.86372373,0\C,13,1.39736441,12,120.03579987,3,-31.59355364,0\C,14,1.4
0626552,13,119.95929462,12,-0.01299947,0\C,15,1.40969726,14,119.794959
2,13,-0.08131313,0\C,16,1.39540998,15,119.85227095,14,0.18237392,0\H,1
2,1.08685627,3,147.89204709,2,-3.29632622,0\H,13,1.08666508,12,120.201
18388,3,148.53901101,0\H,14,1.0871674,13,119.78583474,12,-179.64290741
,0\H,16,1.0881121,15,120.89564307,14,179.71010174,0\H,17,1.08667837,16
,119.73041769,15,179.99113757,0\P,4,1.8040182,3,121.39684731,2,-179.87
162385,0\C,23,1.82073779,4,109.55477543,3,122.13885574,0\H,24,1.094968
56,23,109.22444264,4,-59.17646173,0\H,24,1.09488068,23,110.42115868,4,
-178.60739287,0\H,24,1.09487402,23,109.95879478,4,60.20111454,0\C,23,2
.77142509,4,132.2503987,3,-133.43240926,0\C,28,1.53760946,23,132.62827
353,4,26.7194601,0\C,29,1.53889428,28,103.35458933,23,33.80367412,0\C,
30,1.53769693,29,103.54632413,28,-39.50128661,0\H,28,1.09524045,23,93.
64232926,4,-98.64276447,0\H,28,1.09929435,23,95.44206124,4,152.3211045
9,0\H,29,1.09407551,28,111.75273948,23,155.86689122,0\H,29,1.09675123,
28,109.94882502,23,-84.08039505,0\H,30,1.09417724,29,113.13732836,28,-
160.64605324,0\H,30,1.09774257,29,110.25197812,28,78.44666914,0\H,31,1
.09679486,30,111.68987796,29,-87.46377325,0\H,31,1.09532032,30,112.709
94014,29,150.58150816,0\N,28,1.49131439,23,29.6515612,4,28.46194817,0\
\Version=AM64L-G03RevD.01\State=1-A\HF=-1051.0642752\MP2=-1053.8035095
\RMSD=5.884e-09\Thermal=0.\PG=C01 [X(C17H21N1P1)]\\@

297

1\1\GINC-YIN\SP\RMP2-FC\6-31+G(2d,p)\C20H20N1P1\CHRISTOPH\21-Jan-2011\
0\\#p MP2(FC)/6-31+g(2d,p) scf=tight\\am3sp_53\\0,1\P\C,1,4.67287783\C
,2,1.39919932,1,58.2850631\C,3,1.39663111,2,120.05107811,1,0.14213187,
0\C,4,1.40811709,3,120.94383423,2,-0.87550303,0\C,5,1.40583792,4,118.4
2829477,3,0.96736588,0\C,2,1.39778082,1,61.33442382,5,-8.92173345,0\H,
2,1.08792288,1,178.421067,5,-174.65762226,0\H,3,1.0880991,2,120.128707
11,1,179.47969076,0\H,4,1.08895628,3,119.6098876,2,178.95286668,0\H,6,

1.08723687,5,119.79537386,4,179.3347713,0\H,7,1.08828884,2,120.0529722
5,1,-179.69998278,0\C,1,4.70419681,5,104.13482156,4,85.50097574,0\C,13
,1.41669445,1,57.19570261,5,-154.09269146,0\C,14,1.39266152,13,120.852
68661,1,0.10936524,0\C,15,1.4065613,14,122.04836979,13,-0.34502168,0\C
,16,1.4066741,15,117.01203308,14,1.18572194,0\C,17,1.39269412,16,121.7
7440688,15,-0.72974195,0\H,14,1.08486199,13,120.51615074,1,179.4532656
,0\H,15,1.08906389,14,118.59178277,13,179.2142332,0\H,17,1.08798519,16
,119.69049487,15,178.49396697,0\H,18,1.08500236,17,118.52769712,16,179
.1946918,0\C,1,4.67184058,16,105.75156273,15,98.95113348,0\C,23,1.3977
2879,1,61.23618696,16,8.32363103,0\C,24,1.39959567,23,120.33503682,1,0
.7843701,0\C,25,1.40511402,24,120.60377429,23,-0.23037301,0\C,26,1.408
54919,25,118.46910988,24,-0.56821593,0\C,27,1.39663136,26,120.92922609
,25,1.19208459,0\H,23,1.08794411,1,178.40988165,16,-146.26722259,0\H,2
4,1.08831195,23,120.02617797,1,-179.30458426,0\H,27,1.08913319,26,119.
49186668,25,-178.71189177,0\H,28,1.08807793,27,119.83233591,26,179.635
99996,0\N,13,1.38738235,1,178.31694754,16,142.42998061,0\C,33,1.453457
39,13,119.43376635,1,44.44775766,0\H,34,1.09302108,33,109.05020573,13,
-179.09890339,0\H,34,1.10291558,33,112.76214188,13,60.58068734,0\H,34,
1.09793212,33,110.94663139,13,-60.83445846,0\C,33,1.45355585,13,119.47
345239,1,-154.25831995,0\H,38,1.09796591,33,110.95145292,13,60.7758736
9,0\H,38,1.09303402,33,109.03656744,13,179.04520662,0\H,38,1.10289557,
33,112.77642502,13,-60.62690552,0\H,25,1.08712577,24,119.71880906,23,1
79.99220967,0\\Version=AM64L-G03RevD.01\State=1-A\HF=-1164.2433474\MP2
=-1167.3950096\RMSD=7.066e-09\Thermal=0.\PG=C01 [X(C20H20N1P1)]\\@

297-Me$^+$

1\1\GINC-SOLARIS\SP\RMP2-FC\6-31+G(2d,p)\C21H23N1P1(1+)\CHRISTOPH\19-J
an-2011\0\\#p MP2(FC)/6-31+g(2d,p) scf=tight\\am3mesp_45\\1,1\P\C,1,1.
82820471\H,2,1.09481178,1,109.90545219\H,2,1.09456692,1,109.84128272,3
,-119.97440325,0\H,2,1.09466237,1,110.03017679,4,-120.13366461,0\C,1,4
.61123119,2,107.51901509,4,179.8426972,0\C,6,1.40056706,1,60.1804754,2
,-48.07112814,0\C,7,1.39505266,6,120.06243282,1,0.09082681,0\C,8,1.409
61685,7,119.82077875,6,0.0195036,0\C,9,1.40583422,8,119.95054031,7,-0.
17707605,0\C,6,1.39768013,1,60.07620081,9,104.05499214,0\H,6,1.0869721
1,1,179.93846343,9,-1.74781956,0\H,7,1.08673215,6,120.20194267,1,179.9
9095694,0\H,8,1.0877814,7,119.32114016,6,-179.45871286,0\H,10,1.086730
5,9,120.16119486,8,179.86749126,0\H,11,1.08669777,6,120.19509602,1,-17
9.96219117,0\C,1,4.6092839,9,110.3664158,8,69.27579468,0\C,17,1.397596
9,1,60.40180458,9,12.33695505,0\C,18,1.3978379,17,120.16227518,1,-0.24
221333,0\C,19,1.40595892,18,119.75172241,17,0.01268681,0\C,20,1.409486
9,19,119.943377,18,0.30800711,0\C,21,1.3949427,20,119.83218556,19,-0.3
8823234,0\H,17,1.08696582,1,179.66822858,20,-154.43221864,0\H,18,1.086
69097,17,120.22037574,1,179.85529424,0\H,19,1.08644708,18,119.82262787
,17,-179.82920568,0\H,21,1.08775071,20,120.76020214,19,179.01295707,0\
H,22,1.0867084,21,119.72387345,20,-179.81361005,0\C,1,4.63941988,20,11
0.82580156,19,-109.73977363,0\C,28,1.42507862,1,58.72834268,20,16.3306
2103,0\C,29,1.3853237,28,121.18003379,1,-0.65623663,0\C,30,1.41225045,
29,121.17065432,28,-0.11655904,0\C,31,1.41448706,30,118.07779043,29,0.
36865139,0\C,32,1.38375048,31,121.20427354,30,-0.3744838,0\H,29,1.0837
9294,28,120.29868269,1,179.61243735,0\H,30,1.0870427,29,118.6861297,28
,-179.68958775,0\H,32,1.08814298,31,120.65859372,30,179.36185275,0\H,3
3,1.08377242,32,118.57967958,31,-179.6666918,0\N,28,1.36341408,1,179.5
4358989,31,-165.63353246,0\C,38,1.46052358,28,120.26950067,1,-116.1588
9257,0\H,39,1.09854729,38,111.4461531,28,-60.77065242,0\H,39,1.0984628
5,38,111.42653556,28,60.86322061,0\H,39,1.09100151,38,109.05087778,28,
-179.97292805,0\C,38,1.46074672,28,120.37232832,1,64.37290673,0\H,43,1
.09855712,38,111.48014171,28,60.81399332,0\H,43,1.09097654,38,109.0242
7591,28,179.99783801,0\H,43,1.09851664,38,111.46906083,28,-60.86490595

,0\\Version=AM64L-G03RevD.01\State=1-A\HF=-1203.7050679\MP2=-1207.0004
32\RMSD=8.950e-09\Thermal=0.\PG=C01 [X(C21H23N1P1)]\\@

298

1\1\GINC-CIPCLU03\SP\RMP2-FC\6-31+G(2d,p)\C21H21O3P1\C2175\31-Aug-2010
\0\\#p MP2(FC)/6-31+G(2d,p) scf=tight\\k6sp_120_2\\0,1\C\C,1,1.4021246
1\C,2,1.40082466,1,119.66808892\C,3,1.40231074,2,121.61810054,1,-0.253
71352,0\C,4,1.41126037,3,117.6869434,2,-0.43378246,0\C,5,1.38997314,4,
121.44996817,3,1.02827823,0\H,2,1.08569266,1,121.0702342,6,-179.507395
48,0\H,3,1.08754067,2,118.69132191,1,-179.75792169,0\H,5,1.08883717,4,
119.47997963,3,-178.75892046,0\H,6,1.08679056,5,121.39243357,4,179.886
17727,0\C,3,5.08272855,2,139.27730676,1,-153.40082248,0\C,11,1.4062579
5,3,67.17765496,2,99.85728461,0\C,12,1.3898264,11,120.03733118,3,37.24
927687,0\C,13,1.41136986,12,121.45686691,11,-0.9505674,0\C,14,1.402078
72,13,117.68493828,12,1.03225058,0\C,15,1.40107178,14,121.61151792,13,
-0.42151298,0\H,12,1.0868098,11,118.57314769,3,-143.54310135,0\H,13,1.
08884778,12,119.05524328,11,178.85500769,0\H,15,1.0874634,14,119.71572
915,13,179.15243658,0\H,16,1.08561853,15,119.21922109,14,179.61949561,
0\C,15,5.07977659,14,95.70785652,13,162.6817052,0\C,21,1.40163394,15,6
3.53836613,14,86.15800199,0\C,22,1.40114776,21,119.67208755,15,-38.111
6314,0\C,23,1.40206075,22,121.62602305,21,-0.18907873,0\C,24,1.4115812
9,23,117.67998448,22,-0.44793434,0\C,25,1.38971702,24,121.43767235,23,
1.05697592,0\H,22,1.08581914,21,121.10621754,15,141.90489215,0\H,23,1.
08760064,22,118.62491107,21,-179.83062023,0\H,25,1.08886823,24,119.479
10147,23,-178.69061441,0\H,26,1.08679152,25,121.42612378,24,179.947481
92,0\P,24,1.84836361,23,124.92151552,22,-178.85750239,0\O,1,1.36153232
,2,124.76039238,3,179.97921769,0\O,21,1.36169663,15,146.24095003,14,-1
60.14200797,0\O,11,1.36163606,3,146.51983715,2,-2.65092644,0\C,32,1.41
557051,1,118.18586516,2,-0.3990078,0\H,35,1.09267961,32,105.92511156,1
,-179.66458664,0\H,35,1.09937325,32,111.5695854,1,-60.84543701,0\H,35,
1.09951972,32,111.5928708,1,61.54174139,0\C,34,1.41552627,11,118.20938
895,3,-93.87805639,0\H,39,1.09269824,34,105.92971658,11,-179.67682022,
0\H,39,1.09938992,34,111.56734668,11,-60.86192476,0\H,39,1.09948689,34
,111.59487025,11,61.52601678,0\C,33,1.41576898,21,118.19059469,15,-96.
09357627,0\H,43,1.09271355,33,105.91857848,21,-178.14977791,0\H,43,1.0
9905628,33,111.60596744,21,-59.32399175,0\H,43,1.09961048,33,111.57715
186,21,63.11573092,0\\Version=AM64L-G03RevD.01\State=1-A\HF=-1372.8201
155\MP2=-1376.5394455\RMSD=8.929e-09\Thermal=0.\PG=C01 [X(C21H21O3P1)]
\\@

298-Me$^+$

1\1\GINC-CIPCLU02\SP\RMP2-FC\6-31+G(2d,p)\C22H24O3P1(1+)\C2175\04-Sep-
2010\0\\#p MP2(FC)/6-31+G(2d,p) scf=tight\\k6mesp_62_2\\1,1\C\C,1,1.41
283468\C,2,1.38454273,1,120.29909295\C,3,1.41566481,2,120.50888204,1,0
.03947431,0\C,4,1.40533313,3,118.88312658,2,0.34973426,0\C,5,1.3952663
2,4,120.9374786,3,-0.39938427,0\H,2,1.08592443,1,118.42884261,6,179.87
952743,0\H,3,1.08770866,2,118.69056278,1,179.60436364,0\H,5,1.0868945,
4,120.1735968,3,179.83794537,0\H,6,1.08475725,5,119.23764062,4,-179.95
418022,0\C,4,5.5215872,3,95.50177828,2,131.23063908,0\C,11,1.40670165,
4,42.75266875,3,-69.39680351,0\C,12,1.39550201,11,119.73542153,4,-4.91
559211,0\C,13,1.40519562,12,120.93977889,11,0.0348779,0\C,14,1.4158204
2,13,118.90200163,12,-0.15818256,0\C,15,1.38442645,14,120.48236623,13,
0.10238725,0\H,12,1.0845991,11,121.03414512,4,175.01748308,0\H,13,1.08
671903,12,118.87620526,11,179.90845786,0\H,15,1.08790042,14,120.776916
82,13,-179.35921987,0\H,16,1.08593863,15,121.24115178,14,179.93545563,
0\C,5,5.20653062,4,94.81785545,3,-168.40130705,0\C,21,1.41300265,5,57.
55932548,4,75.14773318,0\C,22,1.38432027,21,120.30190696,5,-38.2527704
5,0\C,23,1.41568341,22,120.49279386,21,-0.02383747,0\C,24,1.40521314,2

3,118.91528195,22,0.30505798,0\C,25,1.39544741,24,120.9171948,23,-0.33
494287,0\H,22,1.08596205,21,118.43613527,5,141.92504226,0\H,23,1.08780
17,22,118.7671311,21,179.58938225,0\H,25,1.08674851,24,120.16886293,23
,-179.96159756,0\H,26,1.08450394,25,119.22603289,24,-179.70642717,0\O,
1,1.34140539,6,124.92643756,5,-179.93270802,0\O,21,1.34164282,5,146.13
478969,4,163.21200116,0\O,11,1.34130935,4,167.16481216,3,-50.88477484,
0\C,31,1.42672705,1,119.2599967,6,1.83020977,0\H,34,1.09084915,31,105.
53709302,1,177.97281696,0\H,34,1.09712631,31,111.05734825,1,59.3788329
9,0\H,34,1.09735725,31,111.03393622,1,-63.35531511,0\C,33,1.42665913,1
1,119.28179618,4,-14.95490903,0\H,38,1.09721982,33,111.04317038,11,61.
08664533,0\H,38,1.09085035,33,105.52323475,11,179.71870175,0\H,38,1.09
732855,33,111.04377978,11,-61.66275598,0\C,32,1.42670938,21,119.303125
2,5,111.21841691,0\H,42,1.09722951,32,111.03517726,21,61.18460073,0\H,
42,1.09085371,32,105.52105949,21,179.81005588,0\H,42,1.09727579,32,111
.05214527,21,-61.56113357,0\P,4,1.80012588,3,120.29948407,2,179.112202
85,0\C,46,1.82871655,4,108.2583636,3,47.58600026,0\H,47,1.09483212,46,
109.85313788,4,179.45975239,0\H,47,1.09468876,46,109.97230518,4,-60.69
683651,0\H,47,1.09476103,46,110.03346995,4,59.44239353,0\\Version=AM64
L-G03RevD.01\State=1-A\HF=-1412.2852321\MP2=-1416.1464836\RMSD=4.627e-
09\Thermal=0.\PG=C01 [X(C22H24O3P1)]\\@

298-BH$^+$

1\1\GINC-YANG\SP\RMP2-FC\6-31+G(2d,p)\C34H32O3P1(1+)\CHRISTOPH\09-Oct-
2010\0\\#p MP2(FC)/6-31+g(2d,p) scf=tight\\k6bhsp_154\\1,1\C\H,1,1.099
13615\C,1,4.34378681,2,105.54826309\C,3,1.39798522,1,58.97575955,2,10.
2774338,0\C,4,1.3974692,3,120.10959595,1,-0.39377118,0\C,5,1.40449785,
4,120.91486809,3,0.29883374,0\C,6,1.40721854,5,118.53157196,4,-0.39940
866,0\C,3,1.3985101,1,60.5818026,6,-14.08038944,0\H,3,1.08710915,1,179
.10644137,6,-168.13854344,0\H,4,1.08728648,3,120.22029561,1,179.650581
31,0\H,5,1.08869929,4,119.47686908,3,179.92207298,0\H,7,1.08691469,6,1
20.4328615,5,-178.96511569,0\H,8,1.08752971,3,120.13960408,1,-179.5206
5338,0\P,1,1.90752294,6,111.92410797,5,120.06305497,0\C,1,4.36181948,6
,116.64008546,5,-107.67679805,0\C,15,1.39769002,1,60.22969187,6,-28.77
185728,0\C,16,1.39748643,15,120.50138183,1,-0.22107552,0\C,17,1.406240
48,16,120.74325432,15,-0.25778907,0\C,18,1.40733654,17,118.16699703,16
,1.28917257,0\C,19,1.39745807,18,121.03249948,17,-1.3400972,0\H,15,1.0
8727739,1,179.39238183,6,112.95868006,0\H,16,1.08743035,15,120.1180303
9,1,179.14892519,0\H,17,1.0841966,16,118.81763372,15,178.38344871,0\H,
19,1.0866003,18,120.0354819,17,177.78505945,0\H,20,1.08742094,19,119.5
0192018,18,179.4739373,0\C,14,4.63197231,1,107.79705456,6,-166.3295124
8,0\C,26,1.40546368,14,60.07379028,1,-126.27669458,0\C,27,1.39641593,2
6,119.83379431,14,0.39531344,0\C,28,1.40340784,27,121.16224499,26,0.16
966907,0\C,29,1.41562555,28,118.50540577,27,-0.68933376,0\C,30,1.38527
177,29,120.78282078,28,0.66267811,0\H,27,1.08464003,26,121.03102972,14
,-179.53445689,0\H,28,1.08597475,27,118.60930296,26,-179.46245508,0\H,
30,1.08841731,29,120.57253359,28,-178.80012299,0\H,31,1.08629444,30,12
1.21187754,29,179.99433914,0\C,14,4.62216757,1,111.12311454,6,72.78403
431,0\C,36,1.40650993,14,59.94512674,1,-121.31690329,0\C,37,1.39549686
,36,119.78795474,14,-0.30772213,0\C,38,1.40559122,37,120.99893502,36,0
.27741987,0\C,39,1.41757902,38,118.81142663,37,-0.52937576,0\C,40,1.38
561799,39,120.39605034,38,0.36283506,0\H,37,1.08486716,36,121.07151298
,14,179.93140269,0\H,38,1.08645929,37,118.79742242,36,-179.94123435,0\
H,40,1.08650666,39,120.61917557,38,-179.15168989,0\H,41,1.08621242,40,
121.02608257,39,-179.74524916,0\C,14,4.64280322,1,111.64708823,6,-50.9
5816728,0\C,46,1.40559851,14,59.12099653,1,-125.77280778,0\C,47,1.3948
7778,46,119.79949615,14,0.48909737,0\C,48,1.4085198,47,121.44942082,46
,0.26250392,0\C,49,1.41822931,48,118.11825087,47,-0.89225803,0\C,50,1.
38582092,49,120.6566107,48,0.6729951,0\H,47,1.08467162,46,121.11135314

,14,-179.50343618,0\H,48,1.08539007,47,118.26096303,46,-179.72195825,0
\H,50,1.08426524,49,120.9306045,48,-178.77247649,0\H,51,1.08628317,50,
120.85931918,49,179.93830252,0\O,26,1.34349058,14,174.89879349,1,55.01
373605,0\O,36,1.3431492,14,175.15731962,1,57.27556699,0\O,46,1.3436744
8,14,175.8559708,1,55.84355495,0\C,57,1.42568814,36,119.17955263,14,-1
78.40396669,0\H,59,1.09753934,57,111.13326911,36,-61.12820079,0\H,59,1
.09115414,57,105.56652061,36,-179.81783893,0\H,59,1.09753934,57,111.08
620678,36,61.57441071,0\C,58,1.42560975,46,119.22688077,14,178.6258150
9,0\H,63,1.09116237,58,105.57610877,46,179.74043393,0\H,63,1.09763883,
58,111.18107002,46,-61.58938632,0\H,63,1.09762495,58,111.06716121,46,6
1.11249747,0\C,56,1.42617616,26,119.21366839,14,179.15556918,0\H,67,1.
09758899,56,111.07874901,26,-61.91325283,0\H,67,1.09109034,56,105.5604
3463,26,179.43869047,0\H,67,1.09750423,56,111.10329539,26,60.76100467,
0\\Version=AM64L-G03RevD.01\State=1-A\HF=-1871.391182\MP2=-1876.956946
3\RMSD=9.460e-09\Thermal=0.\PG=C01 [X(C34H32O3P1)]\\@

298-TT$^+$

1\1\GINC-PHOENIX\SP\RMP2-FC\6-31+G(2d,p)\C40H36O3P1(1+)\CHRISTOPH\05-S
ep-2011\0\\#p MP2(FC)/6-31+g(2d,p) scf=tight\\k6ttsp_11\\1,1\C\P,1,1.9
9604164\C,1,4.39017662,2,107.25942469\C,3,1.39517462,1,59.57763915,2,1
23.67924909,0\C,4,1.3997376,3,120.60579694,1,-0.26814471,0\C,5,1.40551
931,4,121.17629839,3,-0.17598836,0\C,6,1.4121296,5,117.45928935,4,2.05
386529,0\C,7,1.3965376,6,121.30213333,5,-2.47227204,0\H,3,1.08697857,1
,179.89981428,6,-134.23010827,0\H,4,1.08709012,3,120.22801782,1,179.48
232659,0\H,5,1.08345795,4,118.41946431,3,179.19797191,0\H,7,1.08386971
,6,120.28036215,5,176.27569746,0\H,8,1.08730129,7,119.34218164,6,-179.
77006668,0\C,1,4.39179378,6,111.05529791,5,6.68761512,0\C,14,1.3951606
8,1,59.4803841,6,-116.95627721,0\C,15,1.39973438,14,120.61327582,1,-0.
3800972,0\C,16,1.40613378,15,121.19602876,14,-0.15045824,0\C,17,1.4121
5034,16,117.41113688,15,1.95350445,0\C,18,1.39678174,17,121.32300175,1
6,-2.37613115,0\H,14,1.08693845,1,179.89350641,6,-25.97281794,0\H,15,1
.0870906,14,120.22960916,1,179.40757915,0\H,16,1.08327556,15,118.43333
627,14,179.25721942,0\H,18,1.08395614,17,120.29533281,16,176.39641775,
0\H,19,1.08731121,18,119.3271444,17,-179.76530226,0\C,1,4.3897709,6,11
1.67318327,5,-117.21561071,0\C,25,1.39524694,1,59.46011521,6,9.2664156
6,0\C,26,1.39975945,25,120.59563156,1,-0.57677418,0\C,27,1.40557294,26
,121.16882869,25,-0.21485578,0\C,28,1.41175573,27,117.48064572,26,2.19
107433,0\C,29,1.39670305,28,121.28417,27,-2.65143861,0\H,25,1.08695122
,1,179.72985652,6,92.75857721,0\H,26,1.08710797,25,120.23349875,1,179.
17917008,0\H,27,1.0837086,26,118.50177726,25,179.16043258,0\H,29,1.083
95881,28,120.27586464,27,176.03227738,0\H,30,1.08728728,29,119.3376356
2,28,-179.70174061,0\C,2,4.647113,1,112.47033173,6,-54.1436373,0\C,36,
1.41087714,2,59.95805483,1,54.90693667,0\C,37,1.38612104,36,120.621256
74,2,-0.30186069,0\C,38,1.41773536,37,120.64575406,36,-0.26982891,0\C,
39,1.40663842,38,118.21164036,37,0.97319185,0\C,40,1.39588026,39,121.3
948756,38,-0.91465951,0\H,37,1.08601468,36,118.50164397,2,179.50890534
,0\H,38,1.08428479,37,118.60674579,36,179.4271147,0\H,40,1.08517283,39
,120.33985759,38,178.96314318,0\H,41,1.08472686,40,119.04763033,39,179
.98992679,0\C,2,4.65052395,1,112.94773844,6,-174.36107595,0\C,46,1.410
65185,2,60.01497075,1,54.23285884,0\C,47,1.38603456,46,120.64824624,2,
-0.28988628,0\C,48,1.41786391,47,120.69278201,46,-0.27120396,0\C,49,1.
40729696,48,118.11270682,47,0.94418753,0\C,50,1.39601691,49,121.437651
99,48,-0.88193107,0\H,47,1.08604047,46,118.48713881,2,179.54250657,0\H
,48,1.08407722,47,118.52345508,46,179.5194252,0\H,50,1.08481603,49,120
.31811227,48,179.03132251,0\H,51,1.08468919,50,119.03843053,49,-179.99
829368,0\C,2,4.64911439,1,112.7043048,6,65.75136523,0\C,56,1.41083128,
2,59.92875269,1,54.51232255,0\C,57,1.38586459,56,120.63638924,2,-0.399
12903,0\C,58,1.41741565,57,120.68482637,56,-0.2518555,0\C,59,1.4071263

6,58,118.16359452,57,0.88212373,0\C,60,1.39612449,59,121.40808943,58,-
0.81314613,0\H,57,1.08604483,56,118.49828937,2,179.42928708,0\H,58,1.0
8433819,57,118.57672897,56,179.49464211,0\H,60,1.08491458,59,120.39321
465,58,179.06932776,0\H,61,1.08462933,60,119.04638276,59,-179.99908034
,0\O,56,1.34397518,2,175.58485439,1,57.16392714,0\O,46,1.34405062,2,17
5.71172717,1,55.89824314,0\O,36,1.34394686,2,175.63855041,1,56.4646877
2,0\C,66,1.42513832,56,119.09225573,2,177.12689248,0\H,69,1.09105409,6
6,105.61097024,56,179.90113463,0\H,69,1.09745397,66,111.12815489,56,-6
1.4251335,0\H,69,1.09746523,66,111.12486745,56,61.22340689,0\C,67,1.42
509967,46,119.0601716,2,178.01964052,0\H,73,1.09104657,67,105.61313466
,46,179.94339884,0\H,73,1.09743418,67,111.11747144,46,-61.37634953,0\H
,73,1.09745728,67,111.1206373,46,61.27147301,0\C,68,1.42510103,36,119.
06239427,2,178.240369,0\H,77,1.09105404,68,105.61686048,36,179.8073842
7,0\H,77,1.09744552,68,111.1213672,36,-61.51504374,0\H,77,1.09745502,6
8,111.1203718,36,61.1296907,0\\Version=AM64L-G03RevD.01\State=1-A\HF=-
2100.918974\MP2=-2107.3620993\RMSD=3.574e-10\Thermal=0.\PG=C01 [X(C40H
36O3P1)]\\@

299

1\1\GINC-YIN\SP\RMP2-FC\6-31+G(2d,p)\C22H25N2P1\CHRISTOPH\23-Jan-2011\
0\\#p MP2(FC)/6-31+g(2d,p) scf=tight\\am4sp_240\\0,1\P\C,1,4.70778935\
C,2,1.41693837,1,60.00564242\C,3,1.39334606,2,121.12078792,1,0.5788380
5,0\C,4,1.40587118,3,121.81370623,2,-0.59208042,0\C,5,1.40656365,4,116
.93319832,3,-0.72502155,0\C,6,1.39292362,5,122.09187718,4,1.23977624,0
\H,3,1.08515312,2,120.41962743,1,-179.20485817,0\H,4,1.08784607,3,118.
5204991,2,-179.6911581,0\H,6,1.08924972,5,119.3609075,4,-178.26405133,
0\H,7,1.08495317,6,118.59135621,5,-179.65608771,0\C,1,4.67549099,5,105
.14166262,4,-86.16155175,0\C,12,1.39717983,1,61.12468506,5,4.02595841,
0\C,13,1.40005781,12,120.35144392,1,1.05976376,0\C,14,1.4049491,13,120
.67311216,12,-0.24920618,0\C,15,1.40893627,14,118.36828613,13,-0.41312
247,0\C,16,1.39609773,15,120.96351687,14,1.00154048,0\H,12,1.08799407,
1,178.33875383,5,-142.44316417,0\H,13,1.08841141,12,120.03809953,1,-17
9.11213108,0\H,14,1.08713487,13,119.65015785,12,179.84635027,0\H,16,1.
0891331,15,119.47513733,14,-179.02148653,0\H,17,1.08823028,16,119.8034
1843,15,179.66407066,0\C,1,4.70794457,5,103.42916154,4,23.23832788,0\C
,23,1.41654957,1,60.03988318,5,-85.77995956,0\C,24,1.39377624,23,121.0
9487678,1,0.27461983,0\C,25,1.40623649,24,121.81650395,23,-0.66280335,
0\C,26,1.40645933,25,116.93202678,24,-0.79806533,0\C,27,1.39262762,26,
122.07530619,25,1.2892954,0\H,24,1.08514779,23,120.38270661,1,-179.556
43172,0\H,25,1.08797743,24,118.51512193,23,-179.82586436,0\H,27,1.0891
2748,26,119.35469837,25,-178.27581661,0\H,28,1.0849565,27,118.58172078
,26,-179.63631299,0\N,23,1.39011326,1,178.03581056,5,53.13976871,0\C,3
3,1.45348678,23,119.26498148,1,-148.98223652,0\H,34,1.09768495,33,110.
87189562,23,60.7801671,0\H,34,1.09326382,33,109.01771347,23,178.921388
81,0\H,34,1.1033294,33,112.90125064,23,-60.63723962,0\C,33,1.45346112,
23,119.20750311,1,52.47213328,0\H,38,1.09323761,33,109.02753353,23,-17
8.87845802,0\H,38,1.09763294,33,110.86128371,23,-60.7478343,0\H,38,1.1
0327636,33,112.8738442,23,60.67261791,0\N,2,1.38981306,1,178.37035839,
5,142.25118617,0\C,42,1.45353543,2,119.22292772,1,39.32695022,0\H,43,1
.10331401,42,112.87546566,2,60.36291255,0\H,43,1.09319983,42,109.03215
685,2,-179.20371836,0\H,43,1.09769945,42,110.88576896,2,-61.04244063,0
\C,42,1.45352186,2,119.25948459,1,-162.0334455,0\H,47,1.09771013,42,11
0.91821047,2,61.26631529,0\H,47,1.09321932,42,109.02699223,2,179.40607
427,0\H,47,1.10323837,42,112.86378911,2,-60.17567126,0\\Version=AM64L-
G03RevD.01\State=1-A\HF=-1297.3341261\MP2=-1300.9822075\RMSD=5.596e-09
\Thermal=0.\PG=C01 [X(C22H25N2P1)]\\@

299-Me+

1\1\GINC-SOLARIS\SP\RMP2-FC\6-31+G(2d,p)\C23H28N2P1(1+)\CHRISTOPH\19-J
an-2011\0\\#p MP2(FC)/6-31+g(2d,p) scf=tight\\am4mesp_114\\1,1\P\C,1,1
.82950877\H,2,1.09468298,1,109.96891202\H,2,1.09462111,1,109.79720756,
3,120.14927568,0\H,2,1.09482802,1,110.02535916,4,119.84776289,0\C,1,4.
64495825,2,109.26486189,4,60.01430975,0\C,6,1.42398776,1,58.85568058,2
,-133.02429788,0\C,7,1.38650459,6,121.18662008,1,0.52832644,0\C,8,1.41
07403,7,121.20808824,6,0.09568018,0\C,9,1.41338303,8,118.00507338,7,-0
.54651201,0\C,10,1.38458395,9,121.28669907,8,0.55177575,0\H,7,1.084043
71,6,120.37195987,1,-179.6860117,0\H,8,1.08695059,7,118.62993982,6,179
.80365034,0\H,10,1.08824594,9,120.57329624,8,-179.01479035,0\H,11,1.08
406357,10,118.53869903,9,179.70677384,0\C,1,4.6145776,9,109.82085784,8
,-17.308304,0\C,16,1.39737194,1,60.20793427,9,115.42205045,0\C,17,1.39
817748,16,120.138386,1,-0.14540921,0\C,18,1.40507358,17,119.8178433,16
,0.02333099,0\C,19,1.40931322,18,119.89272703,17,-0.20031254,0\C,20,1.
39506691,19,119.87047444,18,0.19426693,0\H,16,1.08703829,1,179.7198313
1,9,-119.4739174,0\H,17,1.08679527,16,120.1877095,1,179.81281717,0\H,1
8,1.08665904,17,120.06689166,16,179.85729245,0\H,20,1.0879273,19,120.6
6279182,18,-179.10407908,0\H,21,1.08681352,20,119.75634381,19,179.9340
741,0\C,1,4.64586811,9,111.58882458,8,105.52654376,0\C,27,1.4240194,1,
58.50440113,9,-15.18789263,0\C,28,1.38617875,27,121.16056172,1,0.53102
68,0\C,29,1.41097313,28,121.25994112,27,0.11252468,0\C,30,1.41365592,2
9,117.97829989,28,-0.11870261,0\C,31,1.38471377,30,121.26416528,29,0.0
8269692,0\H,28,1.08389808,27,120.34238036,1,-179.81556304,0\H,29,1.087
14673,28,118.80043985,27,179.63812161,0\H,31,1.08819156,30,120.6511697
7,29,-179.53092652,0\H,32,1.08398831,31,118.59567166,30,179.82076948,0
\N,27,1.36531598,1,179.54184993,9,-104.00740595,0\C,37,1.45975741,27,1
20.35019504,1,-92.21693277,0\H,38,1.0988314,37,111.54746889,27,61.7088
2454,0\H,38,1.09118123,37,109.05003453,27,-179.0814945,0\H,38,1.098603
97,37,111.46604621,27,-59.92982277,0\C,37,1.45964202,27,120.23269777,1
,88.08160579,0\H,42,1.0911407,37,109.06087008,27,-178.75273236,0\H,42,
1.09854542,37,111.40213199,27,-59.63012045,0\H,42,1.09888254,37,111.54
398634,27,61.93754324,0\N,6,1.36533963,1,179.64371955,9,146.17280788,0
\C,46,1.45952348,6,120.23060835,1,153.18303957,0\H,47,1.09113213,46,10
9.07658593,6,179.64959068,0\H,47,1.09868254,46,111.45842678,6,60.45528
932,0\H,47,1.09874858,46,111.50787335,6,-61.12751944,0\C,46,1.45954487
,6,120.34663927,1,-26.87397087,0\H,51,1.09875577,46,111.51956172,6,61.
15372352,0\H,51,1.09113652,46,109.058118,6,-179.67637107,0\H,51,1.0986
6694,46,111.49471328,6,-60.50859828,0\\Version=AM64L-G03RevD.01\State=
1-A\HF=-1336.8058395\MP2=-1340.5975448\RMSD=7.619e-09\Thermal=0.\PG=C0
1 [X(C23H28N2P1)]\\@

300

1\1\GINC-CIPCLU07\SP\RMP2-FC\6-31+G(2d,p)\C24H30N3P1\C2175\11-Nov-2010
\0\\#p MP2(FC)/6-31+G(2d,p) scf=tight\\k3sp_496\\0,1\P\C,1,4.71426105\
C,2,1.4147286,1,59.93810704\C,3,1.39504305,2,121.2356792,1,-0.79314966
,0\C,4,1.40389518,3,121.86219156,2,0.77027686,0\C,5,1.40767749,4,116.7
9798761,3,0.64805682,0\C,6,1.39194617,5,122.16361662,4,-1.2079336,0\H,
3,1.08509007,2,120.47399443,1,178.94548319,0\H,4,1.08812189,3,118.4403
1542,2,179.79289158,0\H,6,1.08963358,5,119.35869871,4,178.20424739,0\H
,7,1.08482919,6,118.52572056,5,179.62005005,0\C,1,4.71048646,5,104.862
50863,4,92.8553402,0\C,12,1.41631316,1,57.1389256,5,159.18374736,0\C,1
3,1.39212077,12,120.92505107,1,-0.28572286,0\C,14,1.40634827,13,122.10
910093,12,0.28902969,0\C,15,1.40606525,14,116.85358756,13,-1.39135486,
0\C,16,1.39396592,15,121.87991596,14,0.94921242,0\H,13,1.08517309,12,1
20.42849359,1,-179.86938848,0\H,14,1.08954009,13,118.62849263,12,-179.
46925945,0\H,16,1.08833598,15,119.59046194,14,-178.04773317,0\H,17,1.0
8512017,16,118.65275147,15,-179.31879289,0\C,1,4.70688564,15,104.67985

558,14,-93.17896178,0\C,22,1.41448116,1,57.23819385,15,162.66002656,0\
C,23,1.39051225,22,120.99791337,1,-0.38006164,0\C,24,1.41006056,23,121
.7990935,22,1.41896251,0\C,25,1.40154738,24,117.08912565,23,-1.3464825
5,0\C,26,1.39834985,25,121.68947752,24,0.24573374,0\H,23,1.08677465,22
,119.94687621,1,178.52406972,0\H,24,1.08933874,23,118.88683311,22,-179
.30189918,0\H,26,1.08811818,25,119.56124439,24,-178.39680159,0\H,27,1.
08535888,26,118.4089539,25,-178.04576402,0\N,22,1.40935962,1,177.01679
006,15,-160.16942572,0\N,2,1.3942911,1,178.39840287,15,-54.67875104,0\
N,12,1.39200214,1,177.72078567,15,-133.86908186,0\C,33,1.45596899,2,11
9.10596343,1,-46.94738446,0\H,35,1.09367831,33,108.83561006,2,177.6343
79,0\H,35,1.10392091,33,113.14727555,2,-61.74689746,0\H,35,1.09641541,
33,110.69683155,2,59.89901717,0\C,33,1.4548099,2,118.84084905,1,159.89
256254,0\H,39,1.09370809,33,108.92449763,2,-177.45989065,0\H,39,1.0965
6569,33,110.64844992,2,-59.5423949,0\H,39,1.10425204,33,113.04880386,2
,61.90376329,0\C,32,1.46059122,22,117.9366445,1,-82.76254313,0\H,43,1.
09641006,32,109.09087371,22,163.61522652,0\H,43,1.10544066,32,112.3632
0933,22,-76.16532553,0\H,43,1.09183029,32,111.05511401,22,44.99008979,
0\C,32,1.45414317,22,117.62839527,1,136.94928591,0\H,47,1.10663504,32,
113.57633195,22,70.29187925,0\H,47,1.09551447,32,110.035377,22,-51.404
19953,0\H,47,1.09578299,32,108.60244668,22,-169.5186689,0\C,34,1.45507
601,12,118.9227819,1,-63.77237775,0\H,51,1.09326704,34,108.95847476,12
,179.53931162,0\H,51,1.10409088,34,112.97472041,12,-59.93452492,0\H,51
,1.09703543,34,110.60675764,12,61.41767253,0\C,34,1.4559425,12,118.843
74712,1,141.08364308,0\H,55,1.09322666,34,109.0021162,12,-179.93828267
,0\H,55,1.09740694,34,110.57152173,12,-62.00341439,0\H,55,1.10395899,3
4,113.00878426,12,59.21352245,0\\Version=AM64L-G03RevD.01\State=1-A\HF
=-1430.4245289\MP2=-1434.56957\RMSD=8.968e-09\Thermal=0.\PG=C01 [X(C24
H30N3P1)]\\@

300-Me$^+$

1\1\GINC-YANG\SP\RMP2-FC\6-31+G(2d,p)\C25H33N3P1(1+)\CHRISTOPH\23-Oct-
2010\0\\#p MP2(FC)/6-31+G(2d,p) scf=tight\\k3mesp_186\\1,1\C\C,1,1.423
05858\C,2,1.38726889,1,121.1615515\C,3,1.40973799,2,121.26010133,1,-0.
04244271,0\C,4,1.41270813,3,117.95393823,2,0.38314416,0\C,5,1.38511177
,4,121.32240196,3,-0.37323383,0\H,2,1.08404346,1,120.32196791,6,179.92
312241,0\H,3,1.08708444,2,118.81634494,1,-179.74580925,0\H,5,1.0883032
1,4,120.53668668,3,179.13141043,0\H,6,1.08406098,5,118.57454964,4,-179
.82192264,0\C,3,5.24476551,2,142.5847193,1,-161.693413,0\C,11,1.425375
68,3,56.28026057,2,88.26661741,0\C,12,1.38528806,11,121.04497108,3,38.
48557898,0\C,13,1.41264736,12,121.32617431,11,0.04784873,0\C,14,1.4097
0721,13,117.97007084,12,-0.3491419,0\C,15,1.38727418,14,121.24657462,1
3,0.35965169,0\H,12,1.08412031,11,120.35877003,3,-141.77690293,0\H,13,
1.08831691,12,118.14408344,11,-179.47648843,0\H,15,1.08707138,14,119.9
4610636,13,-179.9269044,0\H,16,1.08408323,15,118.50396346,14,179.73851
229,0\C,15,5.24122141,14,94.74484723,13,169.54416181,0\C,21,1.42303631
,15,70.98400736,14,68.05706995,0\C,22,1.3872872,21,121.16544943,15,-33
.26179249,0\C,23,1.40967543,22,121.25142809,21,-0.06064039,0\C,24,1.41
26313,23,117.96279743,22,0.37299889,0\C,25,1.385236,24,121.32183828,23
,-0.35978035,0\H,22,1.08399053,21,120.32524698,15,146.93369767,0\H,23,
1.08709402,22,118.82451121,21,-179.76963301,0\H,25,1.08830308,24,120.5
2072522,23,179.08425205,0\H,26,1.08404066,25,118.59199282,24,-179.8236
1803,0\C,1,2.4516306,2,152.41687142,3,179.11301847,0\H,31,1.09126702,1
,137.84556069,2,0.47936336,0\H,31,1.09889746,1,96.07617084,2,125.93228
693,0\H,31,1.09888738,1,95.78939426,2,-124.62188995,0\C,11,2.45204578,
3,131.26065112,2,33.30677114,0\H,35,1.09898605,11,95.92941695,3,97.971
3546,0\H,35,1.09125981,11,137.82863819,3,-136.74880676,0\H,35,1.098914
49,11,95.96796729,3,-11.46613586,0\C,21,2.45183629,15,131.14777346,14,
-131.26395108,0\H,39,1.09126757,21,137.8330902,15,-137.08698874,0\H,39

,1.09888656,21,96.05175011,15,-11.68312662,0\H,39,1.09890455,21,95.840
92786,15,97.77006043,0\P,4,1.79479582,3,121.26376298,2,179.34906743,0\
C,43,1.83103962,4,108.06118244,3,132.28904756,0\H,44,1.09475419,43,109
.92554506,4,58.85263878,0\H,44,1.09470521,43,110.01600834,4,-61.168313
23,0\H,44,1.094731,43,109.93868809,4,178.79276753,0\N,1,1.3671189,2,12
1.50301083,3,179.68317208,0\N,11,1.36709153,3,147.84661104,2,-8.162171
42,0\N,21,1.36720823,15,147.79258811,14,-172.62992177,0\C,50,1.4588465
6,21,120.23090886,15,-104.97683068,0\H,51,1.09126954,50,109.06708815,2
1,179.87381328,0\H,51,1.09901176,50,111.54301194,21,-60.87817056,0\H,5
1,1.09884558,50,111.49515565,21,60.68319218,0\C,49,1.45902022,11,120.1
7936319,3,-104.98926957,0\H,55,1.09903704,49,111.52683209,11,-61.10487
799,0\H,55,1.09887781,49,111.45677681,11,60.41807104,0\H,55,1.09125282
,49,109.09453201,11,179.62227734,0\C,48,1.45889573,1,120.22726076,2,-0
.01774116,0\H,59,1.0990182,48,111.53577534,1,-60.83130147,0\H,59,1.098
82741,48,111.4799889,1,60.71705406,0\H,59,1.09128133,48,109.07021442,1
,179.91591447,0\\Version=AM64L-G03RevD.01\State=1-A\HF=-1469.9055604\M
P2=-1474.1934009\RMSD=7.686e-09\Thermal=0.\PG=C01 [X(C25H33N3P1)]\\@

300-BH$^+$

1\1\GINC-EDDY\SP\RMP2-FC\6-31+G(2d,p)\C37H41N3P1(1+)\CHRISTOPH\29-Dec-
2010\0\\#p MP2(FC)/6-31+g(2d,p) scf=tight\\k3bhsp_59\\1,1\C\H,1,1.0991
7314\C,1,4.36015455,2,105.42395364\C,3,1.39773775,1,59.14334293,2,-28.
15931197,0\C,4,1.39676874,3,120.20326604,1,-0.26076482,0\C,5,1.4068877
9,4,121.01832781,3,-0.32718391,0\C,6,1.40531756,5,118.21058228,4,1.065
9607,0\C,3,1.39738939,1,60.21654965,6,-31.27125423,0\H,3,1.08714414,1,
179.17932512,6,-161.8070745,0\H,4,1.08730197,3,120.2342399,1,179.03912
56,0\H,5,1.08662328,4,119.0733012,3,179.25292956,0\H,7,1.08488935,6,12
0.32386188,5,177.54639157,0\H,8,1.08740218,3,120.15415957,1,-178.92613
763,0\P,1,1.90497161,6,113.07304867,5,82.09135388,0\C,1,4.34578668,6,1
14.88057113,5,-143.45769902,0\C,15,1.39823567,1,60.66092496,6,-75.9183
9732,0\C,16,1.39895183,15,120.39047056,1,-0.25481805,0\C,17,1.40717396
,16,120.5305565,15,-0.13264519,0\C,18,1.40454627,17,118.46955171,16,0.
02962206,0\C,19,1.39706231,18,120.99085426,17,0.16272096,0\H,15,1.0870
9163,1,179.04885796,18,172.51011553,0\H,16,1.08740518,15,120.11215168,
1,179.6955084,0\H,17,1.08671605,16,119.19382659,15,-179.00943842,0\H,1
9,1.08842559,18,119.57001176,17,179.85620779,0\H,20,1.08720793,19,119.
69562173,18,179.79021776,0\C,14,4.67357009,1,111.57124497,18,52.351351
47,0\C,26,1.42295062,14,59.16021303,1,-51.29167411,0\C,27,1.38649733,2
6,121.44272602,14,0.69741767,0\C,28,1.41464495,27,121.43876831,26,0.10
192385,0\C,29,1.41338351,28,117.24275027,27,-1.04993394,0\C,30,1.38682
39,29,121.73518883,28,1.12807772,0\H,27,1.08423803,26,120.33877913,14,
-179.39443575,0\H,28,1.08415858,27,117.87010767,26,-179.46766657,0\H,3
0,1.0857627,29,120.0605213,28,-178.72000561,0\H,31,1.08418554,30,118.3
4730771,29,179.91831817,0\C,14,4.6576372,1,112.02408277,18,-70.9849132
8,0\C,36,1.42426834,14,58.78847693,1,-57.42278927,0\C,37,1.38672402,36
,121.24581525,14,0.01593511,0\C,38,1.41467193,37,121.21086112,36,0.068
3059,0\C,39,1.41062404,38,117.8056192,37,-0.39478352,0\C,40,1.38704886
,39,121.40519458,38,0.469627,0\H,37,1.08427062,36,120.37096783,14,-179
.72558506,0\H,38,1.08595893,37,118.36140551,36,-179.65275779,0\H,40,1.
08642817,39,119.90789478,38,-179.50225718,0\H,41,1.08424859,40,118.451
04223,39,-179.97644673,0\C,14,4.66272686,1,106.49515271,18,167.7127094
5,0\C,46,1.42149147,14,58.89704469,1,123.70215629,0\C,47,1.3885812,46,
121.26619626,14,-0.16998717,0\C,48,1.40796804,47,121.41755382,46,-0.09
379862,0\C,49,1.41318957,48,117.60337439,47,0.94762199,0\C,50,1.385779
16,49,121.60199598,48,-0.88183711,0\H,47,1.08399042,46,120.28805625,14
,179.54577562,0\H,48,1.08622322,47,118.52750449,46,179.4385956,0\H,50,
1.0888379,49,120.35717343,48,177.99415573,0\H,51,1.08412561,50,118.645
34449,49,179.62368404,0\C,26,2.4511598,14,149.72809553,1,-51.68252106,

0\H,56,1.09906089,26,96.06331922,14,-54.57211304,0\H,56,1.09134141,26,
137.89723504,14,179.90696265,0\H,56,1.09892876,26,95.77223827,14,54.79
781153,0\C,26,2.45025375,14,148.39448723,1,129.11459997,0\H,60,1.09895
079,26,95.70350974,14,-54.76621489,0\H,60,1.09138026,26,137.94771969,1
4,-179.85184403,0\H,60,1.09913708,26,96.03685197,14,54.57867481,0\C,36
,2.45032466,14,148.86216187,1,123.51756417,0\H,64,1.09892899,36,95.536
86726,14,53.8945593,0\H,64,1.0990317,36,96.20291219,14,-55.46068675,0\
H,64,1.09129963,36,137.94805713,14,178.73022423,0\C,36,2.45184249,14,1
49.28158887,1,-57.29125874,0\H,68,1.09134372,36,137.87145816,14,179.18
957891,0\H,68,1.09914448,36,96.21018625,14,-55.05595585,0\H,68,1.09887
321,36,95.64897132,14,54.32085348,0\C,46,2.45017624,14,149.47273327,1,
123.71002771,0\H,72,1.09927623,46,96.20574412,14,55.75849268,0\H,72,1.
09136522,46,137.95211184,14,-178.30191969,0\H,72,1.09875309,46,95.4845
2128,14,-53.57982087,0\C,46,2.45239135,14,148.66596817,1,-56.87736068,
0\H,76,1.0992669,46,96.2724677,14,-55.63689885,0\H,76,1.09883252,46,95
.64973587,14,53.74792688,0\H,76,1.09131333,46,137.84908204,14,178.5636
685,0\N,46,1.3681742,14,179.47120711,1,-25.46674488,0\N,36,1.36796512,
14,179.81064558,1,141.55610227,0\N,26,1.36779625,14,179.32758858,1,143
.79919612,0\\Version=AM64L-G03RevD.01\State=1-A\HF=-1929.0110952\MP2=-
1935.0028332\RMSD=9.676e-09\Thermal=0.\PG=C01 [X(C37H41N3P1)]\\@

300-TT^{+}

1\1\GINC-BORIX\SP\RMP2-FC\6-31+G(2d,p)\C43H45N3P1(1+)\CHRISTOPH\05-Sep
-2011\0\\#p MP2(FC)/6-31+g(2d,p) scf=tight\\k3ttsp_5\\1,1\C\P,1,2.0008
7831\C,1,4.39138538,2,107.80834256\C,3,1.39516505,1,59.54864465,2,125.
54564023,0\C,4,1.39973132,3,120.59258555,1,-0.35487656,0\C,5,1.4056903
7,4,121.23315994,3,-0.22771056,0\C,6,1.41198916,5,117.4004452,4,2.1079
81,0\C,7,1.39660605,6,121.29999324,5,-2.51408197,0\H,3,1.08713948,1,17
9.84933026,6,-165.73906092,0\H,4,1.08731168,3,120.24757568,1,179.33941
51,0\H,5,1.08363072,4,118.53804208,3,179.17556848,0\H,7,1.08374576,6,1
20.20651518,5,176.41623664,0\H,8,1.087487,7,119.286925,6,-179.67562244
,0\C,1,4.39339015,6,110.91961056,5,8.88730281,0\C,14,1.39523582,1,59.4
9097638,6,-117.61230447,0\C,15,1.39965139,14,120.61982035,1,-0.2978137
8,0\C,16,1.40585999,15,121.26935016,14,-0.17662811,0\C,17,1.4122632,16
,117.32391506,15,2.03965482,0\C,18,1.39673472,17,121.34245067,16,-2.46
977771,0\H,14,1.08712964,1,179.92307061,6,7.68284019,0\H,15,1.08733025
,14,120.2449659,1,179.45114328,0\H,16,1.08350895,15,118.33874794,14,17
9.22010636,0\H,18,1.08360503,17,120.22532913,16,176.49406607,0\H,19,1.
0874939,18,119.25331867,17,-179.66641024,0\C,1,4.39222047,6,111.633231
24,5,-115.04508416,0\C,25,1.39525697,1,59.48868075,6,8.49837878,0\C,26
,1.39973633,25,120.61840151,1,-0.49016023,0\C,27,1.40594831,26,121.218
15522,25,-0.1710002,0\C,28,1.41168533,27,117.38917396,26,1.97934633,0\
C,29,1.3967873,28,121.3275296,27,-2.43530037,0\H,25,1.08712493,1,179.7
7931997,6,104.74336625,0\H,26,1.08732133,25,120.23344154,1,179.2706666
7,0\H,27,1.08360181,26,118.39304028,25,179.24627284,0\H,29,1.08380927,
28,120.17382676,27,176.62573665,0\H,30,1.08750659,29,119.27333141,28,-
179.63284939,0\C,2,4.67966507,1,111.58758841,6,-174.5925874,0\C,36,1.4
233104,2,58.92464077,1,56.41531065,0\C,37,1.38675427,36,121.3826101,2,
-0.08833714,0\C,38,1.41492406,37,121.46301665,36,0.16836733,0\C,39,1.4
1138538,38,117.28347404,37,1.12133297,0\C,40,1.38789879,39,121.6783957
9,38,-1.12051106,0\H,37,1.08414639,36,120.42505753,2,179.69130827,0\H,
38,1.08462261,37,117.98230965,36,179.68092452,0\H,40,1.08536387,39,120
.08654217,38,178.67706415,0\H,41,1.08420623,40,118.34079324,39,179.556
4062,0\C,2,4.67973961,1,112.64652027,6,65.79694598,0\C,46,1.42337783,2
,59.01479115,1,54.08226204,0\C,47,1.3869162,46,121.39357827,2,-0.32320
065,0\C,48,1.41504401,47,121.43362343,46,-0.43403956,0\C,49,1.41100734
,48,117.30415118,47,0.69896251,0\C,50,1.38797125,49,121.7028566,48,-0.
60979471,0\H,47,1.08435642,46,120.38308385,2,179.51305231,0\H,48,1.084

76689,47,117.96702417,46,179.496325,0\H,50,1.08539238,49,120.02341494,
48,179.28826737,0\H,51,1.08429179,50,118.33763415,49,-179.88565082,0\C
,2,4.67985262,1,111.91228674,6,-54.65854047,0\C,56,1.4210877,2,57.9412
0244,1,-125.21593662,0\C,57,1.38779465,56,121.24778352,2,0.2359431,0\C
,58,1.41141116,57,121.6988421,56,-0.233235,0\C,59,1.41503614,58,117.28
387151,57,-1.11171419,0\C,60,1.3869989,59,121.44222411,58,1.22433739,0
\H,57,1.08411562,56,120.44387943,2,-179.6339213,0\H,58,1.08532201,57,1
18.24541725,56,-179.89986208,0\H,60,1.08464117,59,120.58521513,58,-178
.2771692,0\H,61,1.08424261,60,118.17786255,59,-179.6706856,0\C,56,2.45
164356,2,149.57086074,1,56.31751877,0\H,66,1.09144134,56,137.87497072,
2,-174.86087204,0\H,66,1.09810059,56,94.76146404,2,-51.46934876,0\H,66
,1.09998258,56,97.12910732,2,57.84210282,0\C,46,2.45081378,2,149.55540
467,1,52.70171917,0\H,70,1.09845128,46,95.28925363,2,52.89721909,0\H,7
0,1.09140001,46,137.95312394,2,177.17833954,0\H,70,1.09978292,46,96.50
253201,2,-56.39205603,0\C,36,2.45130249,2,149.48507251,1,56.56166489,0
\H,74,1.09139524,36,137.89880306,2,-174.76825539,0\H,74,1.0981405,36,9
4.76030925,2,-51.26784655,0\H,74,1.10001972,36,97.08979069,2,58.030137
12,0\N,56,1.36895888,2,179.0676892,1,-64.19363183,0\N,36,1.36906161,2,
178.99479887,1,-57.26752286,0\N,46,1.36884631,2,179.25031587,1,-176.89
790062,0\C,78,1.45786337,56,120.16790818,2,-63.61356209,0\H,81,1.09141
379,78,109.08960205,56,178.51902913,0\H,81,1.09986152,78,111.89894046,
56,-61.83955075,0\H,81,1.09834233,78,111.28274366,56,59.66566683,0\C,7
9,1.45780274,36,120.18879259,2,-70.14887385,0\H,85,1.09139433,79,109.0
7528776,36,178.07836656,0\H,85,1.09988683,79,111.92385016,36,-62.22145
9,0\H,85,1.09827615,79,111.24985731,36,59.28293306,0\C,80,1.45764779,4
6,120.15705021,2,53.37519291,0\H,89,1.09140137,80,109.1015198,46,-178.
40155514,0\H,89,1.09838783,80,111.28885553,46,-59.50220009,0\H,89,1.09
985121,80,111.85726849,46,61.96363447,0\\Version=AM64L-G03RevD.01\Stat
e=1-A\HF=-2158.5373613\MP2=-2165.4087924\RMSD=2.691e-09\Thermal=0.\PG=
C01 [X(C43H45N3P1)]\\@

301

1\1\GINC-EVGENIX\SP\RMP2-FC\6-31+G(2d,p)\C18H14O1P1(1-)\CHRISTOPH\08-F
eb-2012\0\\#p MP2(FC)/6-31+g(2d,p) scf=tight\\ominus1sp_1\\-1,1\P\C,1,
4.6945121\C,2,1.39740967,1,60.94110236\C,3,1.39973986,2,120.49189265,1
,2.17195769,0\C,4,1.40674977,3,121.13826706,2,0.00801078,0\C,5,1.41051
227,4,117.64401259,3,0.09786514,0\C,6,1.39616553,5,121.25858724,4,-0.0
4019738,0\H,2,1.08885075,1,177.80265075,5,177.70275578,0\H,3,1.0895854
9,2,120.0352364,1,-178.37610237,0\H,4,1.08739088,3,119.1122242,2,179.1
3837074,0\H,6,1.08809608,5,118.91934147,4,178.54454892,0\H,7,1.0891603
8,6,119.54569551,5,179.43173281,0\C,1,4.69381871,5,102.99701961,4,-23.
05208155,0\C,13,1.39915946,1,60.44568768,5,-99.82547248,0\C,14,1.39890
79,13,120.47516417,1,1.59445148,0\C,15,1.40617636,14,120.84818408,13,0
.07519983,0\C,16,1.40901664,15,117.97747767,14,-1.3187949,0\C,17,1.398
52922,16,121.2830102,15,1.91143483,0\H,13,1.088943,1,178.31869176,5,14
2.23943734,0\H,14,1.08915181,13,119.94455795,1,-178.38875292,0\H,15,1.
08786185,14,120.19496693,13,-179.31504829,0\H,17,1.08998353,16,119.234
361,15,-177.51919997,0\H,18,1.0894348,17,119.8939568,16,179.63339181,0
\C,1,4.72503697,5,106.42481969,4,-136.35186443,0\C,24,1.4576089,1,55.3
8303152,5,-141.06341787,0\C,25,1.38115304,24,122.71112563,1,0.82430356
,0\C,26,1.42393745,25,122.35438994,24,-0.20243769,0\C,27,1.42629772,26
,116.3980545,25,0.62929378,0\C,28,1.38015884,27,122.05232876,26,-0.472
94135,0\H,25,1.09023353,24,116.7527093,1,-179.56820735,0\H,26,1.092655
55,25,119.30401126,24,179.73335941,0\H,28,1.09217706,27,118.78389992,2
6,178.68035353,0\H,29,1.09049743,28,120.48231175,27,179.45714635,0\O,2
4,1.2552832,1,178.66930771,27,173.96264027,0\\Version=AM64L-G03RevD.01
\State=1-A\HF=-1105.4545557\MP2=-1108.3288163\RMSD=4.061e-09\Thermal=0
.\PG=C01 [X(C18H14O1P1)]\\@

301-Me$^+$

1\1\GINC-EVGENIX\SP\RMP2-FC\6-31+G(2d,p)\C19H17O1P1\CHRISTOPH\08-Feb-2
012\0\\#p MP2(FC)/6-31+g(2d,p) scf=tight\\ominus1mesp_10\\0,1\P\C,1,1.
8342661\H,2,1.09462747,1,109.98409885\H,2,1.09458388,1,110.33019843,3,
120.14974037,0\H,2,1.09438006,1,109.12733844,3,-119.79711116,0\C,1,4.6
2757702,2,105.89830611,5,62.02611395,0\C,6,1.39735656,1,60.96436454,2,
110.33391382,0\C,7,1.39852703,6,120.18500469,1,0.44717976,0\C,8,1.4042
2118,7,120.02980269,6,0.01266107,0\C,9,1.40820725,8,119.54855456,7,0.5
0186829,0\C,10,1.3956699,9,120.11533551,8,-0.66308525,0\H,6,1.08747955
,1,178.99841337,9,173.46996138,0\H,7,1.08729249,6,120.18338862,1,-179.
97270432,0\H,8,1.08639098,7,119.71324105,6,179.3343532,0\H,10,1.087675
46,9,119.87153254,8,177.66825546,0\H,11,1.08716603,10,119.70133435,9,1
79.75125413,0\C,1,4.63314077,9,112.99896032,8,-125.17370637,0\C,17,1.4
6733246,1,57.15605934,9,27.82130254,0\C,18,1.36885083,17,122.66502831,
1,-3.00691219,0\C,19,1.43800704,18,121.34440917,17,-0.84361776,0\C,20,
1.43721054,19,117.57927365,18,1.53554144,0\C,21,1.36854581,20,121.3758
5295,19,-1.71727998,0\H,18,1.08799522,17,116.67920571,1,177.4882418,0\
H,19,1.09036807,18,119.43653876,17,-179.78636256,0\H,21,1.0907068,20,1
19.6387262,19,179.04389332,0\H,22,1.08794094,21,120.68279142,20,-178.6
2679745,0\C,1,4.63901624,20,113.64856599,19,-86.52916969,0\C,27,1.3974
8562,1,59.0440891,20,12.4468447,0\C,28,1.39842828,27,120.13610372,1,0.
4393024,0\C,29,1.40464172,28,120.08187452,27,0.27625492,0\C,30,1.40838
038,29,119.52188517,28,-0.40989792,0\C,31,1.39637208,30,120.1394765,29
,0.32974949,0\H,27,1.08751943,1,178.88893915,20,-22.65139569,0\H,28,1.
08714616,27,120.20427668,1,-179.18062175,0\H,29,1.08775647,28,120.4288
04,27,-179.02260171,0\H,31,1.0882236,30,120.61129553,29,179.9803437,0\
H,32,1.08751881,31,119.81561412,30,-179.91687907,0\O,17,1.24083814,1,1
77.36528904,20,-178.8038894,0\\Version=AM64L-G03RevD.01\State=1-A\HF=-
1145.0231154\MP2=-1148.0399193\RMSD=9.655e-09\Thermal=0.\PG=C01 [X(C19
H17O1P1)]\\@

302

1\1\GINC-BORIX\SP\RMP2-FC\6-31+G(2d,p)\C18H12O3P1(3-)\CHRISTOPH\17-Feb
-2012\0\\#p MP2(FC)/6-31+g(2d,p) scf=tight\\ominus3sp_28\\-3,1\P\C,1,4
.80580279\C,2,1.45374979,1,54.72830552\C,3,1.39381602,2,123.02446111,1
,-0.360816,0\C,4,1.41629539,3,123.0569982,2,-0.73837283,0\C,5,1.417004
43,4,115.39471372,3,1.03414963,0\C,6,1.39369458,5,122.5310193,4,-0.718
44359,0\H,3,1.09472559,2,116.73089956,1,178.80413119,0\H,4,1.0947683,3
,118.95090222,2,179.11034262,0\H,6,1.09207185,5,118.19634104,4,177.811
15961,0\H,7,1.09405373,6,119.9592785,5,179.36949883,0\C,1,4.80918195,5
,107.25406611,4,-144.75445283,0\C,12,1.45404043,1,54.71441947,5,83.428
15152,0\C,13,1.39315032,12,123.09772435,1,-1.18987103,0\C,14,1.4171906
5,13,123.02091632,12,-1.08015085,0\C,15,1.41526299,14,115.3795986,13,1
.05979388,0\C,16,1.39568025,15,122.51389009,14,-0.55492453,0\H,13,1.09
473278,12,116.7181168,1,177.93951478,0\H,14,1.09485703,13,118.80437164
,12,178.93501624,0\H,16,1.0916826,15,118.31937242,14,178.4462272,0\H,1
7,1.09419636,16,119.84396744,15,179.51519362,0\C,1,4.80565492,5,107.90
406117,4,98.19096203,0\C,22,1.45379779,1,54.6281074,5,-150.17523197,0\
C,23,1.39357904,22,123.01943172,1,-0.53690211,0\C,24,1.41685111,23,123
.09791115,22,-0.866749,0\C,25,1.41692538,24,115.34729411,23,1.06600843
,0\C,26,1.39395415,25,122.52818024,24,-0.64899256,0\H,23,1.09468996,22
,116.75295628,1,178.61945412,0\H,24,1.09466063,23,118.9103049,22,178.9
8213117,0\H,26,1.09178317,25,118.36455175,24,178.07243097,0\H,27,1.094
02083,26,119.90989556,25,179.39883718,0\O,12,1.29297285,1,177.47010065
,25,-113.68455052,0\O,22,1.29385574,1,177.99493076,25,-170.34854546,0\
O,2,1.2938766,1,178.17496105,25,129.08505432,0\\Version=AM64L-G03RevD.
01\State=1-A\HF=-1253.8153015\MP2=-1257.1315122\RMSD=5.744e-09\Thermal
=0.\PG=C01 [X(C18H12O3P1)]\\@

302-Me^{+}

1\1\GINC-IBLIS\SP\RMP2-FC\6-31+G(2d,p)\C19H15O3P1(2-)\CHRISTOPH\16-Feb
-2012\0\\#p MP2(FC)/6-31+g(2d,p) scf=tight\\ominus3mesp_26\\-2,1\C\C,1
,1.45635777\C,2,1.38222068,1,123.09259037\C,3,1.42122611,2,121.7099295
9,1,-0.00051112,0\C,4,1.42448614,3,116.94953993,2,0.61627143,0\C,5,1.3
8018577,4,121.86364373,3,-0.5043506,0\H,2,1.09043424,1,116.76625639,6,
179.57587541,0\H,3,1.08962525,2,119.73311467,1,-179.50490643,0\H,5,1.0
9227281,4,119.21997692,3,178.34459131,0\H,6,1.09071302,5,120.3746948,4
,179.77681011,0\C,3,5.44275266,2,144.49518792,1,-159.34614784,0\C,11,1
.45626764,3,72.8028203,2,-137.79329215,0\C,12,1.38232293,11,123.091157
22,3,-28.71166431,0\C,13,1.42118441,12,121.71507201,11,0.08844248,0\C,
14,1.42467887,13,116.94929277,12,0.48138156,0\C,15,1.38013274,14,121.8
5156683,13,-0.38060814,0\H,12,1.09043123,11,116.7686402,3,151.55695107
,0\H,13,1.08972649,12,119.69927491,11,-179.37611718,0\H,15,1.09222071,
14,119.22167918,13,178.49049706,0\H,16,1.09073598,15,120.37694216,14,1
79.71257046,0\C,13,5.43689937,12,144.5618341,11,-161.44690182,0\C,21,1
.45633155,13,72.51982122,12,-136.72994585,0\C,22,1.38227694,21,123.088
99507,13,-29.07277286,0\C,23,1.42121052,22,121.70968778,21,0.00332085,
0\C,24,1.42459016,23,116.95509545,22,0.60102679,0\C,25,1.38024658,24,1
21.85488871,23,-0.4910728,0\H,22,1.09039508,21,116.75949993,13,151.242
09096,0\H,23,1.08963076,22,119.74481353,21,-179.38936585,0\H,25,1.0922
5322,24,119.23697401,23,178.44019017,0\H,26,1.09074235,25,120.37840839
,24,179.82765747,0\O,11,1.25813064,3,150.10269433,2,-9.31252724,0\O,21
,1.25813814,13,149.99039107,12,-8.90753104,0\O,1,1.2580878,2,123.54913
293,3,179.23872835,0\P,4,1.78594909,3,122.03329343,2,-177.9614962,0\C,
34,1.85266784,4,105.88790394,3,126.57906633,0\H,35,1.09568837,34,109.8
214474,4,52.50103692,0\H,35,1.09571968,34,109.67987702,4,172.52764981,
0\H,35,1.09585721,34,109.68143111,4,-67.55459266,0\\Version=AM64L-G03R
evD.01\State=1-A\HF=-1293.6081434\MP2=-1297.0594575\RMSD=5.013e-09\The
rmal=0.\PG=C01 [X(C19H15O3P1)]\\@

303

1\1\GINC-LIEBIG\SP\RMP2-FC\6-31+G(2d,p)\F3P1\CHRISTOPH\08-Feb-2012\0\\
#p MP2(FC)/6-31+g(2d,p) scf=tight\\pf3sp_1\\0,1\P\F,1,1.592664\F,1,1.5
9266395,2,97.66883222\F,1,1.59266395,3,97.66882883,2,98.8586659,0\\Ver
sion=AM64L-G03RevD.01\State=1-A1\HF=-639.1532077\MP2=-639.8993958\RMSD
=1.852e-09\Thermal=0.\PG=C03V [C3(P1),3SGV(F1)]\\@

303-Me^{+}

1\1\GINC-LIEBIG\SP\RMP2-FC\6-31+G(2d,p)\C1H3F3P1(1+)\CHRISTOPH\08-Feb-
2012\0\\#p MP2(FC)/6-31+g(2d,p) scf=tight\\pf3mesp_1\\1,1\F\F,1,2.4435
763\F,2,2.44299066,1,59.99399691\P,3,1.52760451,2,36.91006621,1,-39.74
109603,0\C,4,1.76651242,3,112.54270591,2,-123.61332277,0\H,5,1.0979118
2,4,109.17533318,3,-59.99703681,0\H,5,1.09791301,4,109.17272299,3,59.9
7904988,0\H,5,1.09788884,4,109.22919377,3,179.98981678,0\\Version=AM64
L-G03RevD.01\State=1-A\HF=-678.5036486\MP2=-679.3934188\RMSD=3.908e-09
\Thermal=0.\PG=C01 [X(C1H3F3P1)]\\@

304

1\1\GINC-NODE-17\SP\RMP2-FC\6-31+G(2d,p)\C5H9P1\ZIP04\26-Nov-2008\0\\#
p MP2(FC)/6-31+g(2d,p) scf=tight int=finegrid geom=check guess=read\\b
m_pmol3_001_spmp\\0,1\C,0,-0.0640277355,0.0831701987,0.1557033678\P,0,
-0.1209425279,0.1571008736,2.0231676177\C,0,1.7308197388,-0.0257602575
,2.3613974343\C,0,2.0092499937,-1.5133546179,2.388761462\C,0,0.9491209
059,-2.3294843307,2.3887614586\C,0,-0.4177812354,-1.6798391015,2.36139
74272\H,0,-0.9387264822,-1.8103896538,3.3215058429\H,0,-1.0747612833,-
2.1088073009,1.5910786068\H,0,1.9902680431,0.4444673144,3.3215058525\H
,0,2.3135530159,0.4996523741,1.5910786179\H,0,1.0435778256,-3.41429182

24,2.4385168048\H,0,3.0338165685,-1.8821265489,2.4385168114\H,0,-1.080
3046021,-0.0480249912,-0.2354169274\H,0,0.3227327091,1.0320891676,-0.2
354169228\H,0,0.5674026722,-0.7370397879,-0.2094920873\\Version=IA32L-
G03RevD.01\State=1-A'\HF=-535.3109781\MP2=-536.1733029\RMSD=6.328e-09\
Thermal=0.\PG=CS [SG(C1H1P1),X(C4H8)]\\@

304-Me$^+$

1\1\GINC-NODE-13\SP\RMP2-FC\6-31+G(2d,p)\C6H12P1(1+)\ZIP04\26-Nov-2008
\0\\#p MP2(FC)/6-31+g(2d,p) scf=tight int=finegrid geom=check guess=re
ad\\bm_pmol3met_spmp_001\\1,1\C,0,-0.2253131795,0.2370940882,-0.111952
6608\P,0,0.1298349386,-0.1364445628,1.6304896005\C,0,1.9260336997,-0.0
078041063,2.0133619544\C,0,2.2779068223,-1.424033526,2.4374935042\C,0,
1.3085154319,-2.3461914895,2.4373859036\C,0,-0.0883355584,-1.924029507
3,2.0131260269\H,0,-0.8345095827,-2.0642694533,2.8069382141\H,0,-0.448
0556152,-2.470220651,1.1305909744\H,0,2.1032340057,0.7303392139,2.8072
916343\H,0,2.4896073492,0.3243246395,1.1309402657\H,0,1.4764599774,-3.
3803846326,2.7266229335\H,0,3.3023954582,-1.6434194336,2.7268232057\H,
0,-1.2845532004,0.0560548017,-0.3272886721\H,0,0.0083223359,1.28601892
37,-0.3271382737\H,0,0.3855021798,-0.4048853734,-0.7566458827\C,0,-0.8
763779389,0.9212019537,2.7124500106\H,0,-0.6487168842,1.976631089,2.52
43663348\H,0,-1.9418362899,0.7469381842,2.5238627358\H,0,-0.6560288799
,0.6890612444,3.7605063059\\Version=IA32L-G03RevD.01\State=1-A\HF=-574
.750573\MP2=-575.7542772\RMSD=2.988e-09\Thermal=0.\PG=C01 [X(C6H12P1)]
\\@

305

1\1\GINC-NODE8\SP\RMP2-FC\6-31+G(2d,p)\C20H21O1P1\ZIP04\24-Jan-2009\0\
\#p MP2(FC)/6-31+g(2d,p) scf=tight int=finegrid geom=check guess=read\
\newcat_003.opt_spmp_001\\0,1\C,0,-0.0541318985,-0.3455453556,0.131392
5361\C,0,-0.0117098803,-0.2682405451,1.5394022866\C,0,1.2371623217,-0.
3357322659,2.1977010682\C,0,2.3091667697,-0.7829935185,1.4190401874\C,
0,2.2295452721,-0.8638639805,0.0133567716\C,0,1.074257959,-0.494864679
4,-0.6856432229\H,0,3.2843004997,-0.9097542648,1.8869119006\H,0,3.1430
382638,-1.0559484903,-0.5476197674\C,0,0.5047772575,2.7050622323,-0.17
82318567\C,0,0.5098119288,2.7988200103,1.2163747228\C,0,1.6508154868,2
.4501466689,1.9590298436\C,0,2.8539623422,2.3083389452,1.2496162783\C,
0,2.8489602417,2.211442703,-0.1441787175\C,0,1.6444986583,2.2570469065
,-0.8652473749\H,0,-0.4242977187,3.0058582173,1.7357339022\H,0,3.78182
36799,2.1275739208,1.7912635635\H,0,3.7733914013,1.957249078,-0.661573
1668\C,0,1.5173349927,1.9663309328,3.393209689\C,0,1.4758608623,0.3619
375963,3.5233109494\C,0,1.5316635173,1.5744995751,-2.2158384146\C,0,1.
1002142183,0.0253542007,-2.1097406649\H,0,0.5953160875,2.3719960902,3.
8269791679\H,0,2.347081731,2.3251190318,4.0160255143\H,0,2.495957592,1
.6223369677,-2.7366276785\H,0,0.7984688254,2.0849344228,-2.8527906554\
H,0,1.800033712,-0.5655464071,-2.712989405\H,0,0.1097477235,-0.0887245
65,-2.563060205\H,0,2.4351189146,0.0239590285,3.9334587725\H,0,0.70736
93271,0.1056539806,4.2642729045\H,0,-0.4324940574,2.8392245812,-0.7166
253311\C,0,-1.3522782779,0.0846168407,1.9348083056\H,0,-1.7291165028,0
.2320885999,2.9391122527\C,0,-2.0976780705,0.1872717277,0.7937085691\O
,0,-1.3106735164,-0.0557049011,-0.320186722\P,0,-3.8722031039,0.558651
6338,0.5904249085\C,0,-3.7753471877,1.8891101679,-0.7167144416\H,0,-3.
1547146781,1.594741496,-1.5712284013\H,0,-4.788976273,2.1122657932,-1.
0718965354\H,0,-3.3646735055,2.8048760856,-0.2756498993\C,0,-4.3870729
763,-0.8811826192,-0.481181276\H,0,-5.4101101343,-0.7091261203,-0.8380
265396\H,0,-3.7251928835,-1.0210475635,-1.3436970097\H,0,-4.3883879209
,-1.7986151583,0.1181571911\\Version=AM64L-G03RevD.01\State=1-A\HF=-11
85.1986983\MP2=-1188.4326543\RMSD=5.024e-09\Thermal=0.\PG=C01 [X(C20H2
1O1P1)]\\@

305-Me+

1\1\GINC-NODE7\SP\RMP2-FC\6-31+G(2d,p)\C21H24O1P1(1+)\ZIP04\20-Jan-200
9\0\\#p MP2(FC)/6-31+g(2d,p) scf=tight int=finegrid geom=check guess=r
ead\\newcat_003_met_spmp_001\\1,1\C,0,-0.0014622989,0.0119978217,-0.00
62949969\C,0,-0.0448201792,0.0324814296,1.4060465119\C,0,1.1649638219,
0.0268797751,2.1475679589\C,0,2.2978137036,-0.3216740985,1.4149071336\
C,0,2.3006730178,-0.3444022334,0.0012641614\C,0,1.1747536264,-0.024059
6323,-0.764407824\H,0,3.2530449327,-0.4091475331,1.9281478561\H,0,3.25
69444198,-0.4572563452,-0.506010282\C,0,0.3498489558,3.1051719352,-0.1
177289072\C,0,0.3289883691,3.1370855114,1.2795569272\C,0,1.4795486089,
2.8258721541,2.0236088865\C,0,2.6983190376,2.7902250916,1.3261682235\C
,0,2.7200129942,2.7588098134,-0.0709023648\C,0,1.5241011906,2.76167094
16,-0.807714265\H,0,-0.6180366069,3.2866370386,1.7973602349\H,0,3.6294
237731,2.6569203204,1.8749132259\H,0,3.667335685,2.6000906764,-0.58393
28421\C,0,1.3658321183,2.2870463463,3.4385874785\C,0,1.2734625264,0.67
54811779,3.5118119555\C,0,1.4609860668,2.145833742,-2.1928622278\C,0,1
.2685919425,0.5436313102,-2.1665659419\H,0,0.4744090941,2.7013778356,3
.9246077953\H,0,2.2275768103,2.5916570224,4.0435832969\H,0,2.379186517
4,2.3606641589,-2.7512906133\H,0,0.6314174366,2.5775829756,-2.76532872
45\H,0,2.1184167723,0.0840944733,-2.6827116139\H,0,0.3728990737,0.2920
35771,-2.7451620214\H,0,2.1712910136,0.3010351603,4.0151929334\H,0,0.4
221418107,0.4097006267,4.1509068183\H,0,-0.5805267348,3.2287166936,-0.
6720389348\C,0,-1.4211563204,0.2322839027,1.7486065318\H,0,-1.84663759
1,0.2970219584,2.742512989\C,0,-2.1027296038,0.3142016446,0.5605257963
\O,0,-1.260008281,0.196600539,-0.521649876\P,0,-3.8236779857,0.4628448
725,0.168843202\C,0,-4.1089030416,1.9338135588,-0.8634504082\H,0,-3.48
82736778,1.8673520731,-1.7638144814\H,0,-5.1634758345,1.9953998908,-1.
1555976709\H,0,-3.830108368,2.8349996067,-0.3070127472\C,0,-4.40055065
63,-0.9973903555,-0.7513934572\H,0,-5.4501901071,-0.8741933119,-1.0420
310515\H,0,-3.7881817247,-1.1216826394,-1.6512435243\H,0,-4.2954218706
,-1.8910573725,-0.1271613685\C,0,-4.7528168692,0.6143371024,1.72148129
56\H,0,-5.8194688812,0.7204909353,1.4945508984\H,0,-4.6081620077,-0.28
02312013,2.3362447276\H,0,-4.4184542092,1.4965780456,2.2772545095\\Ver
sion=AM64L-G03RevD.01\State=1-A\HF=-1224.639746\MP2=-1228.0162545\RMSD
=2.767e-09\Thermal=0.\PG=C01 [X(C21H24O1P1)]\\@

306

1\1\GINC-NODE7\SP\RMP2-FC\6-31+G(2d,p)\C7H13P1\ZIP04\24-Jan-2009\0\\#p
MP2(FC)/6-31+g(2d,p) scf=tight int=finegrid geom=check guess=read\\ne
wcat_004.opt_spmp_001\\0,1\C,0,-0.3183471454,0.0307990662,0.1614497195
\H,0,-0.9217467939,0.1848278787,1.0661910103\H,0,0.7300578503,-0.03102
88907,0.4764824324\C,0,-0.7477996471,-1.2700569734,-0.6077886544\H,0,-
1.5728619481,-1.7876996202,-0.10015592\H,0,0.0876326644,-1.9770523117,
-0.6743658979\C,0,-0.5262272019,1.2038899229,-0.8330409052\H,0,-0.0517
222557,2.1334174002,-0.50167373\C,0,-2.0440016538,1.3414987754,-1.0981
951122\H,0,-2.2608308936,2.2416041714,-1.6860681219\H,0,-2.5893568249,
1.4474573164,-0.1496675287\C,0,-1.189508534,-0.8049735951,-2.020921948
4\H,0,-1.2954611851,-1.6333846743,-2.7290813248\C,0,-2.4717369884,0.04
6034668,-1.8641667606\H,0,-3.2373961741,-0.5154242132,-1.3102045649\H,
0,-2.906361603,0.2867232472,-2.8419038672\P,0,0.2122230857,0.416364546
7,-2.3913146758\C,0,-0.40552012,1.4602723537,-3.8118060258\H,0,-0.2618
946703,0.8970762651,-4.742571344\H,0,-1.4539464958,1.7716753434,-3.752
9061194\H,0,0.2208325349,2.3588293235,-3.878283661\\Version=AM64L-G03R
evD.01\State=1-A\HF=-613.3893042\MP2=-614.5747561\RMSD=1.314e-09\Therm
al=0.\PG=C01 [X(C7H13P1)]\\@

306-Me+

1\1\GINC-NODE5\SP\RMP2-FC\6-31+G(2d,p)\C8H16P1(1+)\ZIP04\20-Jan-2009\0

\\#p MP2(FC)/6-31+g(2d,p) scf=tight int=finegrid geom=check guess=read
\\newcat_004_met_spmp_001\\1,1\C,0,0.0606781662,0.0006587186,-0.052164
1741\H,0,0.0799516894,-0.0151850336,1.0428492126\H,0,1.1037184969,0.02
18975583,-0.3860320836\C,0,-0.7038733338,-1.2436149957,-0.6354239339\H
,0,-1.0764197582,-1.8971655004,0.1606650358\H,0,-0.0498622596,-1.85548
51668,-1.266087928\C,0,-0.7090135049,1.2685146371,-0.5210620114\H,0,-0
.1601203675,2.2047369532,-0.3952270191\C,0,-2.1202406184,1.2542985601,
0.1326917596\H,0,-2.6612106403,2.1855971671,-0.0672759814\H,0,-2.00495
07443,1.1832494205,1.2195705101\C,0,-1.916054365,-0.6958725584,-1.4418
623543\H,0,-2.3923498066,-1.4280782957,-2.0981001961\C,0,-2.8849477104
,0.0097710005,-0.4506660233\H,0,-3.161456029,-0.6989377369,0.337319049
2\H,0,-3.8145877237,0.3085338172,-0.9471160053\P,0,-1.1398620845,0.773
474661,-2.2470827274\C,0,-2.2810460268,1.8864784562,-3.1255963649\H,0,
-2.6278585847,1.405427932,-4.0473554249\H,0,-3.1477281071,2.1274622274
,-2.5036257754\H,0,-1.7613754429,2.81560931,-3.3863378852\C,0,0.271265
1704,0.4190427672,-3.3407843168\H,0,-0.08666429,-0.0554362094,-4.26170
71452\H,0,0.7794624649,1.3546123842,-3.6011434109\H,0,0.9861794676,-0.
248883366,-2.8524069372\\Version=AM64L-G03RevD.01\State=1-A\HF=-652.83
28698\MP2=-654.1614729\RMSD=6.255e-09\Thermal=0.\PG=C01 [X(C8H16P1)]\\
@

307

1\1\GINC-NODE24\SP\RMP2-FC\6-31+G(2d,p)\C5H9P1\ZIP04\28-May-2012\0\\#p
MP2(FC)/6-31+G(2d,p) scf=tight geom=check guess=read\\s305_2\\0,1\C,0
,0.4078593764,-1.295180852,0.0081887972\C,0,1.7892402427,-0.5931777071
,0.1345861751\C,0,1.5352162087,0.8943652819,0.2511739899\C,0,0.3054947
447,1.323153562,-0.0704602077\H,0,0.0628461812,-1.6318474744,0.9945346
269\H,0,2.4027480129,-0.7922281275,-0.7583543294\H,0,2.3637329946,-0.9
790214175,0.9889664489\H,0,2.3464827965,1.5661063954,0.533890811\H,0,0
.0266819171,2.3752997481,-0.0860954324\H,0,0.4552493331,-2.1727976005,
-0.6447003248\P,0,-0.8096488029,-0.0086062952,-0.6576700288\C,0,-2.126
4203068,-0.0231751726,0.6717980366\H,0,-2.7765753561,0.8519383486,0.55
04479714\H,0,-2.7510865616,-0.9169313373,0.5501844302\H,0,-1.703946780
6,-0.0141913519,1.684215036\\Version=AM64L-G03RevD.01\State=1-A\HF=-53
5.3085354\MP2=-536.1703424\RMSD=2.048e-09\Thermal=0.\PG=C01 [X(C5H9P1)
]\\@

307-Me$^+$

1\1\GINC-NODE15\SP\RMP2-FC\6-31+G(2d,p)\C6H12P1(1+)\ZIP04\28-May-2012\
0\\#p MP2(FC)/6-31+G(2d,p) scf=tight geom=check guess=read\\s305_1me\\
1,1\C,0,1.1295438912,-0.0186721148,-0.0094675539\C,0,2.6833995076,0.11
88634404,-0.0128165253\C,0,3.0554504443,1.5808590986,-0.0253586107\C,0
,2.0760471039,2.5040675636,-0.0237508811\H,0,0.7626658196,-0.553710998
7,0.872923364\H,0,3.1192618825,-0.3843007698,-0.8856667315\H,0,3.12152
98338,-0.3685553767,0.8678769653\H,0,4.1078813625,1.8615876603,-0.0339
133627\H,0,2.223456283,3.579760714,-0.0303328161\H,0,0.7595081485,-0.5
508072574,-0.8922669585\P,0,0.4788926006,1.7019726445,-0.0073475073\C,
0,-0.5038924002,2.078121975,1.4771018004\H,0,-0.7685601408,3.141657684
7,1.4912685798\H,0,-1.4257286052,1.4848264577,1.4772459001\H,0,0.07885
64648,1.8448869162,2.3743997593\C,0,-0.5321293581,2.0720560286,-1.4742
555426\H,0,-1.4514363944,1.475047626,-1.4573163677\H,0,-0.8013882393,3
.134453094,-1.484620603\H,0,0.0352708855,1.8403709436,-2.3817162986\\V
ersion=AM64L-G03RevD.01\State=1-A\HF=-574.7545321\MP2=-575.7572936\RMS
D=6.377e-09\Thermal=0.\PG=C01 [X(C6H12P1)]\\@

308

1\1\GINC-NODE-17\SP\RMP2-FC\6-31+G(2d,p)\C5H11P1\ZIP04\26-Nov-2008\0\\
#p MP2(FC)/6-31+g(2d,p) scf=tight int=finegrid geom=check guess=read\\

bm_pmol1_001_spmp\\0,1\C,0,-0.0505941338,0.0610948941,-0.0403670128\P,
0,0.005886707,0.0028869323,1.8290818934\C,0,1.8754367581,-0.031681217,
2.0963008848\C,0,2.3305219872,-1.4957125801,1.9365254346\C,0,1.2498092
753,-2.3629273416,2.606331545\C,0,-0.1264700462,-1.8730506844,2.095817
7539\H,0,-0.9300052859,-2.108234952,2.8027459791\H,0,-0.3807423891,-2.
353499767,1.1414110024\H,0,2.0792342056,0.3213188991,3.1162004428\H,0,
2.3813027559,0.657663492,1.4086726702\H,0,1.396170122,-3.4329027605,2.
4070548297\H,0,1.3066843378,-2.2287461868,3.696242219\H,0,2.4008660474
,-1.7515978546,0.8695123944\H,0,3.3232711007,-1.667235095,2.3750796691
\H,0,-1.065965419,-0.1840895107,-0.3759117377\H,0,0.1721104518,1.07998
54443,-0.3801145533\H,0,0.6516403608,-0.6326655161,-0.5209709042\\Vers
ion=IA32L-G03RevD.01\State=1-A\HF=-536.495876\MP2=-537.3843366\RMSD=9.
654e-09\Thermal=0.\PG=C01 [X(C5H11P1)]\\@

308-Me$^+$

1\1\GINC-NODE-19\SP\RMP2-FC\6-31+G(2d,p)\C6H14P1(1+)\ZIP04\26-Nov-2008
\0\\#p MP2(FC)/6-31+g(2d,p) scf=tight int=finegrid geom=check guess=re
ad\\bm_pmol1met_spmp_001\\1,1\C,0,-0.1956505952,0.1831067384,-0.096226
615\P,0,0.1215111362,-0.1358661855,1.6656843836\C,0,1.9057939227,-0.00
1431355,2.0944082475\C,0,2.3590222234,-1.4775992474,2.267375534\C,0,1.
1888778726,-2.2542068076,2.9060774453\C,0,-0.0973905494,-1.9088093445,
2.1058804259\H,0,-1.0249190723,-2.0786578907,2.6628128833\H,0,-0.15300
04689,-2.4881354222,1.1752659934\H,0,1.9902113263,0.5527178271,3.03807
9427\H,0,2.4674726633,0.5478886862,1.3314543577\H,0,1.3624789092,-3.33
49747372,2.8957212665\H,0,1.0659856305,-1.9545633457,3.9550022883\H,0,
2.6063573689,-1.9076988358,1.2881233466\H,0,3.2642303306,-1.5262572079
,2.8809936201\H,0,-1.2385249421,-0.050476146,-0.3393154306\H,0,-0.0029
574773,1.2364441724,-0.3291207467\H,0,0.4612879036,-0.4433285899,-0.70
99086788\C,0,-0.9160721028,0.9631590309,2.6767849018\H,0,-0.6509942743
,2.0097953392,2.4883202106\H,0,-1.973653937,0.8138504348,2.4315995266\
H,0,-0.7622570364,0.743275493,3.7391358671\\Version=IA32L-G03RevD.01\S
tate=1-A\HF=-575.9412426\MP2=-576.972147\RMSD=3.297e-09\Thermal=0.\PG=
C01 [X(C6H14P1)]\\@

309

1\1\GINC-NODE11\SP\RMP2-FC\6-31+G(2d,p)\C18H21P1\ZIP04\28-May-2012\0\\
#p MP2(FC)/6-31+G(2d,p) scf=tight geom=check guess=read\\s308_1\\0,1\C
,0,-0.2893154342,-1.4841557268,0.4367832571\C,0,-1.1454453691,-0.37078
37752,0.4625719059\C,0,-0.7270278105,0.7820861867,1.1763442341\C,0,0.3
247505292,0.6283374029,2.0922267013\C,0,1.1544220696,-0.4956682173,2.0
673708551\C,0,0.9451947515,-1.4956441668,1.110088917\H,0,0.5973459961,
1.4694039178,2.7288181524\H,0,2.0502641344,-0.5115914203,2.6869545615\
C,0,1.5311068226,-0.2189184328,-1.8283784483\C,0,0.6529856914,0.867202
4299,-1.8567474981\C,0,0.7794728331,1.9200791829,-0.9345665296\C,0,1.9
818837108,1.9948775968,-0.2139335005\C,0,2.8559091176,0.9049951564,-0.
1800518924\C,0,2.5564125265,-0.2808032377,-0.871259305\H,0,-0.22294853
59,0.8274762259,-2.5029932737\H,0,2.1657306966,2.8436887226,0.44406619
18\H,0,3.7029692751,0.9261493057,0.504918577\C,0,-0.4384712831,2.73558
25473,-0.5382943835\C,0,-1.1520180177,2.1958762984,0.7977712253\C,0,3.
0934348125,-1.6130858714,-0.3803177006\C,0,2.0835836618,-2.3730990408,
0.6194633265\H,0,-1.1734314089,2.7054919626,-1.3504887\H,0,-0.18057189
1,3.7894703031,-0.3697005204\H,0,4.0415660849,-1.4525725714,0.14762685
22\H,0,3.3055596629,-2.2907900975,-1.2173729046\H,0,2.6750316606,-2.76
01082341,1.4593945989\H,0,1.6712723419,-3.2432492061,0.0936122405\H,0,
-0.8857706399,2.867854052,1.6231203247\P,0,-2.6674204882,-0.263697696,
-0.5882371331\C,0,-3.9814982038,-0.4063233443,0.7351985785\H,0,-3.9629
044056,0.4862547752,1.3710148812\H,0,-4.9686016979,-0.4606932121,0.259
1453426\H,0,-3.8396972073,-1.2895879603,1.3703488833\C,0,-2.7971713544

,-1.9766958459,-1.3053777799\H,0,-2.7288088701,-2.7727763976,-0.552524
3651\H,0,-3.7698451904,-2.0551662685,-1.8063480842\H,0,-2.0219059012,-
2.1338710291,-2.0643381751\H,0,-2.2345766565,2.2912192401,0.6518833741
\H,0,-0.5189362871,-2.3171656083,-0.2240592147\H,0,1.3238182739,-1.086
8149456,-2.4539775722\\Version=AM64L-G03RevD.01\State=1-A\HF=-1034.593
0662\MP2=-1037.3563611\RMSD=5.276e-09\Thermal=0.\PG=C01 [X(C18H21P1)]\
\@

309-Me$^+$

1\1\GINC-NODE8\SP\RMP2-FC\6-31+G(2d,p)\C19H24P1(1+)\ZIP04\20-Jan-2009\
0\\#p MP2(FC)/6-31+g(2d,p) scf=tight int=finegrid geom=check guess=rea
d\\newcat_007_mca_spmp_003\\1,1\C,0,-0.0080201525,0.0018791756,-0.0461
867407\C,0,0.0104768383,-0.0140413659,1.3653466349\C,0,1.255083391,-0.
0190453382,2.0578183589\C,0,2.370835133,-0.3893803844,1.2957842637\C,0
,2.3377403933,-0.4077049808,-0.0993355894\C,0,1.1798363386,-0.02844787
77,-0.7930044088\H,0,-0.9470947814,0.1325480043,-0.5788098764\H,0,3.32
71771857,-0.5176991978,1.7980700422\H,0,3.2625008145,-0.5777784599,-0.
6467767272\C,0,0.9086853518,3.0790794279,-0.2059171486\C,0,0.885203126
3,3.1136488233,1.1882966576\C,0,2.002833156,2.7133392437,1.9418130708\
C,0,3.2206218709,2.5861093235,1.2562241592\C,0,3.2440204884,2.54551289
63,-0.1428909141\C,0,2.0551225179,2.6342430367,-0.8849503661\H,0,-0.00
56967328,3.2790312687,-0.7634290041\H,0,-0.0447166759,3.3581023659,1.7
004399223\H,0,4.1363663768,2.3840036845,1.8098896613\H,0,4.1782479918,
2.3108994867,-0.6507567781\C,0,1.8182133567,2.1875782544,3.3530910744\
C,0,1.5125997932,0.6009860385,3.4225288869\C,0,1.9382180985,1.99309248
27,-2.2558018446\C,0,1.2550977294,0.5367439325,-2.2004627796\H,0,0.989
1697657,2.7157581894,3.8406784166\H,0,2.7091318378,2.3700338713,3.9645
064491\H,0,2.9336915624,1.8909485895,-2.7018316647\H,0,1.3426210553,2.
6086195604,-2.9406292052\H,0,1.8242323399,-0.1296836432,-2.8596523671\
H,0,0.2437332391,0.6065452897,-2.6177202413\H,0,2.383737879,0.10764292
65,3.8668812277\P,0,-1.562934637,-0.0500215529,2.2391265545\C,0,-1.992
1854884,1.4678363687,3.1586724391\H,0,-2.062095488,2.3113054288,2.4642
513803\H,0,-2.9621239344,1.3267436746,3.6500169917\H,0,-1.2389635245,1
.6959036778,3.9177979228\C,0,-1.5859938186,-1.4460147127,3.4109653385\
H,0,-0.780513191,-1.3459845542,4.1448975723\H,0,-2.5476429478,-1.48072
39481,3.935520085\H,0,-1.4399724598,-2.380087894,2.8577916082\H,0,0.68
76862175,0.4450022013,4.1294247737\C,0,-2.9293260619,-0.3269986746,1.0
679176783\H,0,-2.7505904953,-1.2289805841,0.4740537905\H,0,-3.85607672
43,-0.4561857316,1.6385938228\H,0,-3.0499320701,0.5311782169,0.3990018
517\\Version=AM64L-G03RevD.01\State=1-A\HF=-1074.0411574\MP2=-1076.948
8064\RMSD=4.157e-09\Thermal=0.\PG=C01 [X(C19H24P1)]\\@

310

1\1\GINC-NODE7\SP\RMP2-FC\6-31+G(2d,p)\C10H15P1\ZIP04\02-Feb-2009\0\\#
p MP2(FC)/6-31+g(2d,p) scf=tight int=finegrid geom=check guess=read\\n
ewcat_008_b98opfr_spmp_002\\0,1\C,0,0.0030656154,0.0043548004,-0.00164
09193\C,0,0.0020770015,-0.0023755757,1.3927989738\C,0,1.1953143654,-0.
0057319208,2.1342415888\C,0,2.4214247819,-0.000766034,1.427862837\C,0,
2.3988894043,0.005804813,0.0222560209\C,0,1.2093425374,0.0058633379,-0
.7163020856\H,0,-0.9438150982,0.0101526198,-0.5400988578\H,0,-0.948500
3249,-0.0013919712,1.9240717755\H,0,3.341689033,0.0126574603,-0.524039
0364\P,0,4.0309974777,0.0018881868,2.3655629039\C,0,4.9103356154,1.434
0782699,1.5512060371\H,0,5.9392054293,1.477597719,1.9302510805\H,0,4.9
435229065,1.3643467878,0.4562999809\H,0,4.4111802125,2.3704556746,1.82
60009256\C,0,4.9229031948,-1.4146473298,1.5377199142\H,0,5.9519283778,
-1.4530581739,1.916899533\H,0,4.4316876507,-2.3578747419,1.8032800804\
H,0,4.9560384812,-1.3342346969,0.4435549592\C,0,1.1227914261,-0.010124
1196,3.6504431243\H,0,1.6202845885,-0.8881400835,4.080272368\H,0,1.612

062056,0.8701420449,4.0852055822\H,0,0.0783559102,-0.0158240278,3.9830
983307\C,0,1.2224069989,-0.0128237988,-2.2308048497\H,0,2.1887493977,0
.3205108109,-2.6267092384\H,0,1.0387524962,-1.0246674702,-2.6192599771
\H,0,0.4440601768,0.6396895896,-2.6455547824\\Version=AM64L-G03RevD.01
\State=1-A\HF=-728.1884579\MP2=-729.7858665\RMSD=7.917e-09\Thermal=0.\
PG=C01 [X(C10H15P1)]\\@

310-Me$^+$

1\1\GINC-NODE8\SP\RMP2-FC\6-31+G(2d,p)\C11H18P1(1+)\ZIP04\02-Feb-2009\
0\\#p MP2(FC)/6-31+g(2d,p) scf=tight int=finegrid geom=check guess=rea
d\\newcat_008_met_b98opfr_spmp_002\\1,1\C,0,-0.022553488,0.006805038,-
0.0157925432\C,0,0.0197616309,0.4118585295,1.3171853807\C,0,1.21183951
75,0.4285965275,2.0565033519\C,0,2.3897677591,0.0151557496,1.382594050
3\C,0,2.3365268596,-0.3915481481,0.030680904\C,0,1.1378727997,-0.40441
38502,-0.6890932415\H,0,-0.9745983602,0.0133421286,-0.5426383052\H,0,-
0.9000322351,0.7258874342,1.8054095395\H,0,3.240237763,-0.7062811059,-
0.4835105836\P,0,4.0131481271,0.0100649068,2.1792641532\C,0,4.61811274
55,1.6980981017,2.5166127269\H,0,5.6206561315,1.6482662326,2.957301366
1\H,0,4.6649380274,2.2562130486,1.5750952187\H,0,3.9536935708,2.228167
6683,3.2049405571\C,0,5.2516163927,-0.7654629565,1.0925095074\H,0,6.20
9777754,-0.7872275916,1.6242035981\H,0,4.9626338379,-1.7924986272,0.84
67546548\H,0,5.3801016564,-0.1917597029,0.1692700339\C,0,1.1489293422,
0.8470853158,3.5125342462\H,0,1.0797133167,-0.0260137935,4.1758776076\
H,0,2.0076214111,1.4472764574,3.8338064609\H,0,0.2560121715,1.45371974
07,3.6917993613\C,0,1.0906790198,-0.8406102154,-2.1360878063\H,0,2.080
1239284,-1.1325236807,-2.5030877292\H,0,0.4164371081,-1.6962644258,-2.
265943828\H,0,0.7178401434,-0.0322594355,-2.777413446\C,0,4.0359000065
,-0.9490649696,3.7295281045\H,0,3.4056818525,-0.4864557141,4.493031895
5\H,0,3.6722913172,-1.9638084275,3.5335322028\H,0,5.064613279,-1.00273
75058,4.10442895\\Version=AM64L-G03RevD.01\State=1-A\HF=-767.6364807\M
P2=-769.3787893\RMSD=6.511e-09\Thermal=0.\PG=C01 [X(C11H18P1)]\\@

311

1\1\GINC-YANG\SP\RMP2-FC\6-31+G(2d,p)\C6H14N1P1\CHRISTOPH\29-Sep-2009\
0\\#p MP2(FC)/6-31+g(2d,p) scf=tight\\dxsp_11\\0,1\C\C,1,1.54325103\P,
2,1.90142631,1,108.30711948\C,3,1.88756205,2,91.33414316,1,9.52168855,
0\C,1,1.54135904,2,106.65811613,3,-33.49106283,0\H,1,1.09853699,5,111.
74666861,4,169.99794608,0\H,1,1.09934001,5,109.38600841,4,-71.75073387
,0\H,2,1.09885475,1,110.2384442,5,83.15228328,0\H,2,1.09750457,1,113.3
3424495,5,-157.22410376,0\H,4,1.0980585,3,109.26037395,2,-103.44490262
,0\H,4,1.09599178,3,110.07649161,2,139.30898301,0\H,5,1.09817462,1,112
.08716712,2,170.07178342,0\H,5,1.09995101,1,109.21535865,2,-71.6385006
3,0\C,3,2.80404769,2,85.62482135,1,-79.26677606,0\H,14,1.09973538,3,13
8.1629877,2,-175.04260519,0\H,14,1.09448914,3,86.56687981,2,72.101302,
0\H,14,1.10586383,3,104.63782509,2,-35.75668166,0\C,14,2.4247654,3,61.
84506164,2,-125.54142887,0\H,18,1.10011235,14,90.88086626,3,-144.71294
266,0\H,18,1.1057991,14,94.25622358,3,107.56507241,0\H,18,1.09437712,1
4,143.42638698,3,-21.28683981,0\N,14,1.45664842,3,31.12586947,2,-146.0
8320883,0\\Version=AM64L-G03RevD.01\State=1-A\HF=-630.5532634\MP2=-631
.7813262\RMSD=5.558e-09\Thermal=0.\PG=C01 [X(C6H14N1P1)]\\@

311-Me$^+$

1\1\GINC-GRETEL\SP\RMP2-FC\6-31+G(2d,p)\C7H17N1P1(1+)\CHRISTOPH\28-Sep
-2009\0\\#p MP2(FC)/6-31+g(2d,p) scf=tight\\dxmesp_5\\1,1\C\C,1,1.5543
7725\P,2,1.83215339,1,102.99214807\C,3,1.83733924,2,97.8722614,1,17.61
703685,0\C,1,1.54177305,2,107.2464632,3,-39.47704333,0\H,1,1.09808417,
5,110.32398392,4,-71.05230775,0\H,1,1.09470483,5,112.01628067,4,169.67
402671,0\H,2,1.09466691,1,113.85709104,5,-161.72517002,0\H,2,1.0968749

6,1,110.3390129,5,75.76410267,0\H,4,1.09422848,3,111.98464546,2,130.59
283753,0\H,4,1.09701866,3,108.48440167,2,-110.33814102,0\H,5,1.0945166
5,1,111.94654516,2,170.27435981,0\H,5,1.09687112,1,109.85051234,2,-70.
39914496,0\C,3,1.82558799,2,110.44686178,1,-97.60239557,0\H,14,1.09617
296,3,110.14641987,2,170.82508131,0\H,14,1.09600943,3,110.02060319,2,-
69.06214548,0\H,14,1.09663312,3,110.0892458,2,50.94320962,0\C,3,2.7131
7562,2,91.36774378,1,155.29360007,0\H,18,1.09498032,3,136.19379786,2,-
99.1713407,0\H,18,1.09348368,3,84.0547714,2,10.65811864,0\H,18,1.09895
33,3,106.29450498,2,118.86364131,0\C,18,2.4654243,3,62.87811292,2,-155
.1491308,0\H,22,1.09372504,18,144.25681975,3,23.16200159,0\H,22,1.0949
5341,18,89.57714578,3,144.26895698,0\H,22,1.09900361,18,93.51021338,3,
-106.96135326,0\N,22,1.47705327,18,33.44775984,3,18.89015812,0\\Versio
n=IA32L-G03RevD.01\State=1-A\HF=-670.0044616\MP2=-671.3749803\RMSD=8.9
32e-09\Thermal=0.\PG=C01 [X(C7H17N1P1)]\\@

312

1\1\GINC-NODE7\SP\RMP2-FC\6-31+G(2d,p)\C12H13P1\ZIP04\18-Jan-2009\0\\#
p MP2(FC)/6-31+g(2d,p) scf=tight int=finegrid\\newcat_006_spmp_001\\0,
1\C\C,1,1.38484583\C,2,1.42427754,1,121.9010114\C,3,1.43370165,2,118.8
9622163,1,0.,0\C,4,1.42474255,3,118.53742684,2,0.,0\C,5,1.37677737,4,1
20.99138876,3,0.,0\H,3,2.16819377,2,96.02741508,1,180.,0\H,2,1.0894693
7,1,119.58680865,6,-180.,0\C,3,1.42315054,2,122.14050947,1,180.,0\C,4,
1.42239987,3,118.95177291,2,180.,0\H,5,1.08906676,4,118.72145186,3,-18
0.,0\H,6,1.08842818,5,119.16479195,4,-180.,0\C,10,1.37961688,4,120.716
50718,3,0.,0\C,9,1.37919545,3,120.7478101,2,-180.,0\H,10,1.08867055,4,
118.84224444,3,180.,0\H,13,1.08770332,10,120.0537578,4,-180.,0\H,14,1.
08760754,9,120.12721626,3,180.,0\P,1,1.8522758,2,117.67850344,3,-180.,
0\C,18,1.86707822,1,101.10257223,2,129.06106809,0\H,19,1.097667,18,113
.58667643,1,52.42160887,0\H,19,1.09728223,18,108.78159248,1,173.339272
75,0\H,19,1.09605973,18,109.37078823,1,-69.1151416,0\C,18,1.86707573,1
,101.10321804,2,-129.06080129,0\H,23,1.09766711,18,113.58668572,1,-52.
41410149,0\H,23,1.0960594,18,109.37121859,1,69.12299788,0\H,23,1.09728
247,18,108.78119312,1,-173.33139612,0\\Version=AM64L-G03RevD.01\State=
1-A\HF=-802.7741269\MP2=-804.6097433\RMSD=3.783e-09\Thermal=0.\PG=C01
[X(C12H13P1)]\\

312-Me$^+$

1\1\GINC-NODE7\SP\RMP2-FC\6-31+G(2d,p)\C13H16P1(1+)\ZIP04\17-Jan-2009\
0\\#p MP2(FC)/6-31+g(2d,p) scf=tight int=finegrid\\newcat_006_met_spmp
_001\\1,1\C\C,1,1.38921286\C,2,1.4198227,1,120.74660832\C,3,1.43651396
,2,118.93194349,1,0.,0\C,4,1.42484232,3,118.99480447,2,0.,0\C,5,1.3751
0836,4,121.27511777,3,0.,0\H,3,2.1727453,2,95.81640305,1,-180.,0\H,2,1
.08778169,1,121.29746541,6,180.,0\C,3,1.42330975,2,121.73215419,1,-180
.,0\C,4,1.4203859,3,118.92988223,2,-180.,0\H,5,1.0875752,4,118.8892118
7,3,180.,0\H,6,1.08805599,5,119.43001733,4,-180.,0\C,10,1.37999904,4,1
20.38782997,3,0.,0\C,9,1.3784333,3,120.27133624,2,180.,0\H,10,1.087492
32,4,119.10435214,3,180.,0\H,13,1.08683161,10,119.91027722,4,180.,0\H,
14,1.08665954,9,120.0579675,3,-180.,0\P,1,1.79428904,2,121.08706554,3,
-180.,0\C,18,1.82119039,1,110.38536007,2,120.23686832,0\H,19,1.0953606
6,18,110.03112599,1,58.33014267,0\H,19,1.09598174,18,110.21984504,1,17
8.53669393,0\H,19,1.09535205,18,109.54509978,1,-61.04185756,0\C,18,1.8
2118999,1,110.38532542,2,-120.23813884,0\H,23,1.09536132,18,110.031084
14,1,-58.3243014,0\H,23,1.09535113,18,109.54521417,1,61.04765925,0\H,2
3,1.09598228,18,110.21989987,1,-178.53080274,0\C,18,1.81749199,1,111.3
6478665,2,0.,0\H,27,1.09504365,18,110.52832211,1,-60.64216451,0\H,27,1
.0950439,18,110.52830806,1,60.64616495,0\H,27,1.09586665,18,108.859555
79,1,-180.,0\\Version=AM64L-G03RevD.01\State=1-A\HF=-842.2225822\MP2=-
844.2027278\RMSD=3.144e-09\Thermal=0.\PG=C01 [X(C13H16P1)]\\@

313

1\1\GINC-NODE-10\SP\RMP2-FC\6-31+G(2d,p)\C6H13P1\ZIP04\08-Dec-2008\0\\
#p MP2(FC)/6-31+g(2d,p) scf=tight int=finegrid geom=check guess=read\\
bm_pmol4_chair_eq_spmp\\0,1\P,0,1.3079614031,0.0000000185,-0.228386063
6\C,0,0.1104902509,1.3972316184,-0.5798298201\C,0,-1.1291819528,1.2904
51443,0.3338976299\C,0,-1.9390775079,0.0000003253,0.0976893037\C,0,-1.
1291822039,-1.2904509136,0.3338977066\C,0,0.1104899796,-1.3972313887,-
0.5798297353\H,0,-0.2028647612,-1.3759535292,-1.6358811844\H,0,0.62492
8463,-2.3530061726,-0.4116711768\H,0,0.6249289202,2.3530063131,-0.4116
713182\H,0,-0.2028644963,1.3759537601,-1.6358812676\H,0,-1.7785781928,
-2.1620925189,0.1686072498\H,0,-0.8120696887,-1.3334876392,1.386899876
7\H,0,-1.7785777742,2.1620931623,0.1686071214\H,0,-0.8120694296,1.3334
881663,1.3868997975\H,0,-2.3191011208,0.0000003321,-0.9363011585\H,0,-
2.8209307891,0.000000424,0.7535505338\C,0,2.3035175705,-0.0000001157,-
1.8052409128\H,0,2.9514580185,0.8848735916,-1.8309157806\H,0,2.9514579
023,-0.8848739284,-1.8309157094\H,0,1.6735276791,-0.0000001163,-2.7052
922923\\Version=IA32L-G03RevD.01\State=1-A\HF=-575.5385621\MP2=-576.58
0136\RMSD=5.196e-09\Thermal=0.\PG=C01 [X(C6H13P1)]\\

313-Me$^+$

1\1\GINC-NODE-17\SP\RMP2-FC\6-31+G(2d,p)\C7H16P1(1+)\ZIP04\26-Nov-2008
\0\\#p MP2(FC)/6-31+g(2d,p) scf=tight int=finegrid geom=check guess=re
ad\\bm_pmol4met_chair_spmp\\1,1\C,0,-0.0964031171,0.1176921856,-0.0414
042831\P,0,0.0963849594,-0.1176698298,1.7525580239\C,0,1.8543055346,0.
0271671747,2.2238603535\C,0,2.6624589632,-1.2124593128,1.7590403243\C,
0,2.0866551265,-2.5474557578,2.272524507\C,0,0.6644093357,-2.849088531
6,1.759040208\C,0,-0.391796599,-1.812645175,2.2238602241\H,0,-0.484249
4064,-1.8246253194,3.3191517696\H,0,-1.3851157123,-2.0293380191,1.8103
397224\H,0,2.2623657842,0.9583629399,1.8103399329\H,0,1.8842573908,0.1
154503341,3.3191519043\H,0,0.3389367582,-3.832167284,2.1184955478\H,0,
0.6669953407,-2.908546049,0.6616906916\H,0,3.6903814318,-1.0869540473,
2.1184957468\H,0,2.7202430279,-1.2267033631,0.6616908121\H,0,2.0901956
177,-2.5517781802,3.3726458631\H,0,2.7521837493,-3.3599545037,1.958754
8091\H,0,-1.1485342282,-0.0050735397,-0.3223049948\H,0,0.2311508089,1.
1250450349,-0.3223049009\H,0,0.5037974236,-0.6150518611,-0.5907202078\
C,0,-0.9163302309,1.1186854161,2.6185140059\H,0,-0.5915964801,2.129337
9119,2.3455488944\H,0,-1.9711409863,0.9993346626,2.3455486355\H,0,-0.8
139111273,0.9936486134,3.7021562691\\Version=IA32L-G03RevD.01\State=1-
A\HF=-614.9881002\MP2=-616.1735163\RMSD=3.546e-09\Thermal=0.\PG=C01 [X
(C7H16P1)]\\

314

1\1\GINC-CIPCLU08\SP\RMP2-FC\6-31+G(2d,p)\C9H21P1\C2175\22-Apr-2010\0\
\#p MP2(FC)/6-31+G(2d,p) scf=tight\\knox1sp_12\\0,1\P\C,1,1.86992291\H
,2,1.09735344,1,108.16566821\H,2,1.09938202,1,113.04367876,3,119.98024
592,0\H,2,1.0953904,1,111.05111702,3,-117.3965306,0\C,1,1.86923206,2,9
6.89848432,5,-178.39047268,0\H,6,1.09747843,1,108.20387964,2,63.867179
69,0\H,6,1.09521435,1,110.95035963,2,-178.58102428,0\H,6,1.09869482,1,
113.00232873,2,-56.13035572,0\C,1,1.90237478,6,101.498315,2,101.618351
61,0\H,10,1.10310324,1,105.68690216,6,-49.54790378,0\C,10,1.56553858,1
,110.02569032,6,-163.25817449,0\C,12,1.54295145,10,111.57845984,1,162.
09786961,0\H,12,1.09956405,10,106.91322684,1,46.73401899,0\H,13,1.0979
2311,12,111.27862028,10,-55.10107861,0\H,13,1.09809834,12,110.39879407
,10,-174.78263559,0\C,10,1.57117316,1,114.44673137,6,68.26873424,0\C,1
7,1.5422785,10,111.85008562,1,-98.09919875,0\H,17,1.09994235,10,106.39
780572,1,146.64227278,0\H,18,1.09825975,17,110.62977823,10,-175.788793
8,0\H,18,1.09782099,17,111.14081594,10,-56.0813011,0\H,13,1.09781396,1
2,112.12185201,10,65.75690182,0\H,18,1.09694122,17,111.88122449,10,64.

37665854,0\C,17,1.54341017,10,115.53399934,1,29.20780817,0\H,24,1.0949
5499,17,112.19211446,10,52.09664435,0\H,24,1.09749795,17,112.07437055,
10,-68.88335768,0\H,24,1.09842537,17,109.74595201,10,172.14514072,0\C,
12,1.54103488,10,114.28925038,1,-71.47708427,0\H,28,1.09779303,12,111.
6105882,10,-66.7818539,0\H,28,1.09784888,12,110.16972214,10,174.186706
61,0\H,28,1.09677625,12,111.68412537,10,54.25084437,0\\Version=AM64L-G
03RevD.01\State=1-A\HF=-693.8006448\MP2=-695.3394135\RMSD=6.731e-09\Th
ermal=0.\PG=C01 [X(C9H21P1)]\\@

314-Me+

1\1\GINC-NAUTILUS\SP\RMP2-FC\6-31+G(2d,p)\C10H24P1(1+)\CHRISTOPH\23-Ap
r-2010\0\\#p MP2(FC)/6-31+g(2d,p) scf=tight\\knox1mesp_1\\1,1\C\H,1,1.
09577262\H,1,1.09429456,2,108.91661939\H,1,1.09599182,3,108.60170433,2
,-118.28214889,0\C,1,2.92944284,3,91.28112753,4,-88.853651,0\H,5,1.096
35395,1,88.43641065,3,109.61439491,0\H,5,1.09346724,1,147.56571513,3,-
125.8947478,0\H,5,1.0944081,1,89.4924054,3,0.93998128,0\C,1,2.97800422
,5,63.48970854,7,-33.74406032,0\H,9,1.10315295,1,75.92288371,5,163.501
46857,0\C,9,1.57151854,1,149.80110019,5,64.99031662,0\C,11,1.5420579,9
,116.20863541,1,-63.35159407,0\H,11,1.09830201,9,104.037206,1,179.7515
7678,0\H,12,1.09580324,11,109.33734729,9,-172.21727175,0\H,12,1.094742
73,11,112.85201799,9,-52.97877534,0\C,9,1.57748714,1,92.52231886,5,-91
.27689284,0\C,16,1.54062123,9,115.21745384,1,119.68856838,0\H,16,1.100
5447,9,108.13207333,1,-0.38349073,0\H,17,1.09591426,16,111.70400069,9,
64.92089271,0\H,17,1.09583558,16,113.33770996,9,-57.2950798,0\H,12,1.0
9862047,11,112.32335585,9,70.0149022,0\H,17,1.09585501,16,109.14468622
,9,-176.80721657,0\C,16,1.5437122,9,109.20661163,1,-115.55480785,0\H,2
3,1.09702072,16,111.75507907,9,-62.96322651,0\H,23,1.09577036,16,109.9
3320584,9,177.91509597,0\H,23,1.09694033,16,111.6588141,9,58.30142953,
0\C,11,1.54201037,9,112.01503358,1,65.39222266,0\H,27,1.09697397,11,11
2.61181245,9,-68.72526784,0\H,27,1.09598549,11,109.93229124,9,172.0689
4282,0\H,27,1.0969665,11,111.46301522,9,52.94864551,0\P,5,1.82263645,1
,36.61687463,9,-33.10512984,0\C,31,1.82451896,5,107.88863644,1,-112.82
500573,0\H,32,1.09511837,31,110.06930678,5,177.53842455,0\H,32,1.09644
42,31,108.61146172,5,58.60118485,0\H,32,1.09327133,31,111.4538618,5,-6
1.25448305,0\\Version=AM64L-G03RevD.01\State=1-A\HF=-733.250533\MP2=-7
34.9334317\RMSD=3.132e-09\Thermal=0.\PG=C01 [X(C10H24P1)]\\@

315

1\1\GINC-NODE26\SP\RMP2-FC\6-31+G(2d,p)\C17H37P1\ZIP07\29-Apr-2010\0\\
#p MP2(FC)/6-31+G(2d,p) scf=tight\\knox2sp_1\\0,1\P\C,1,1.87124951\H,2
,1.09877354,1,113.10785787\H,2,1.09760697,1,107.91764836,3,-119.647360
34,0\H,2,1.09480989,1,111.39284203,4,-117.56085476,0\C,1,1.86929645,2,
96.09617377,5,177.45598175,0\H,6,1.09755753,1,107.420372,2,58.55197521
,0\H,6,1.09498875,1,111.34640654,2,175.72828489,0\H,6,1.09924083,1,113
.35129315,2,-60.9780513,0\C,1,1.92058722,6,101.73266895,2,102.75364263
,0\H,10,1.09353402,1,102.68182251,6,-63.1036918,0\C,10,1.58790373,1,11
3.13793977,6,47.66229811,0\H,12,1.10006791,10,105.71664025,1,-34.71123
399,0\C,10,1.58562999,1,113.6688674,6,-178.12544329,0\H,14,1.09790101,
10,104.59841149,1,-160.85327474,0\C,14,1.5824324,10,113.37636893,1,-46
.54087439,0\H,16,1.0942086,14,105.9899395,10,-18.82825415,0\C,14,1.573
20083,10,114.24801259,1,85.12587313,0\H,18,1.09868212,14,108.63135463,
10,-77.15749132,0\C,12,1.57501205,10,113.07221755,1,-145.60183403,0\H,
20,1.09815066,12,104.24395161,10,172.23366742,0\C,12,1.56773214,10,116
.63028269,1,81.20779735,0\H,22,1.0979268,12,108.84643873,10,49.0646350
3,0\C,22,1.54633093,12,113.93821698,10,168.02612951,0\H,24,1.09512073,
22,113.38677646,12,-65.9758876,0\H,24,1.09933736,22,111.27466063,12,56
.06561294,0\H,24,1.09780327,22,109.41318165,12,174.3327447,0\C,22,1.54
553838,12,111.92094053,10,-70.69537082,0\H,28,1.09987397,22,110.129811

28,12,-67.65506434,0\H,28,1.0948159,22,113.61584416,12,52.8408782,0\H,
28,1.09811597,22,110.40946596,12,174.03715554,0\C,20,1.54294018,12,113
.05511018,10,58.81592161,0\H,32,1.09691959,20,112.78204258,12,-73.5238
4002,0\H,32,1.09816158,20,109.94909665,12,167.17664088,0\H,32,1.098478
24,20,111.39440282,12,48.02236297,0\C,20,1.54274174,12,117.16813586,10
,-70.66602806,0\H,36,1.0956909,20,112.17011086,12,74.94834641,0\H,36,1
.09858144,20,109.74316174,12,-166.28573723,0\H,36,1.09700009,20,112.48
638044,12,-46.65751792,0\C,18,1.54511452,14,115.27309062,10,42.5628499
,0\H,40,1.09828541,18,111.66894813,14,57.40839326,0\H,40,1.09794358,18
,109.37776732,14,175.81920011,0\H,40,1.09464398,18,113.49506029,14,-64
.97230953,0\C,18,1.54598076,14,111.5496181,10,164.90438888,0\H,44,1.09
927669,18,110.78548905,14,-58.51477967,0\H,44,1.09312832,18,113.063383
3,14,62.62180178,0\H,44,1.09786976,18,110.19420804,14,-177.39059125,0\
C,16,1.54562817,14,113.22405063,10,-134.13774175,0\H,48,1.09806916,16,
111.55721461,14,51.80826115,0\H,48,1.09841269,16,110.09180701,14,171.0
3920523,0\H,48,1.09558177,16,112.82210931,14,-69.12217746,0\C,16,1.543
82454,14,116.00741374,10,97.36371665,0\H,52,1.0967056,16,112.83227659,
14,67.65360408,0\H,52,1.09806605,16,109.30250471,14,-173.13937888,0\H,
52,1.09572211,16,111.91788363,14,-54.78394626,0\\Version=AM64L-G03RevD
.01\State=1-A\HF=-1006.0584993\MP2=-1008.8642313\RMSD=1.421e-09\Therma
l=0.\PG=C01 [X(C17H37P1)]\\@

315-Me$^+$

1\1\GINC-NODE28\SP\RMP2-FC\6-31+G(2d,p)\C18H40P1(1+)\ZIP07\28-Apr-2010
\0\\#p MP2(FC)/6-31+G(2d,p) scf=tight\\knox2mesp_1\\1,1\C\H,1,1.093850
38\H,1,1.09647127,2,108.8657574\H,1,1.09247128,2,109.10529744,3,118.55
815557,0\C,1,2.88566296,4,148.66640931,3,107.95722633,0\H,5,1.09577328
,1,94.60973832,4,134.4201216,0\H,5,1.09362331,1,147.9302455,4,-3.57873
74,0\H,5,1.09600175,1,82.70384961,4,-117.25017518,0\C,5,2.99054725,1,6
3.36448648,4,51.9102678,0\H,9,1.09823418,5,75.16777374,1,89.56829492,0
\C,9,1.59536598,5,87.93707013,1,-166.18840595,0\H,11,1.10130995,9,103.
65942318,5,-18.14609695,0\C,9,1.58577023,5,151.56682597,1,-4.6449333,0
\H,13,1.09998748,9,101.469753,5,162.29593005,0\C,13,1.57822376,9,114.5
0522791,5,-84.11352517,0\H,15,1.0953912,13,106.28072159,9,-20.7745581,
0\C,13,1.57537711,9,114.07568884,5,49.74125173,0\H,17,1.09831934,13,10
8.50809537,9,-77.4247508,0\C,11,1.5702403,9,112.23552121,5,-128.870305
82,0\H,19,1.09898272,11,108.79757167,9,-77.37731317,0\C,11,1.5807704,9
,115.5037484,5,97.19003801,0\H,21,1.09663337,11,107.01113671,9,-14.487
11158,0\C,21,1.54373954,11,116.42699075,9,102.65779635,0\H,23,1.096821
67,21,109.1985325,11,-170.80798302,0\H,23,1.09376756,21,112.55840902,1
1,-51.61182151,0\H,23,1.09425716,21,112.63692955,11,70.36319867,0\C,21
,1.54547604,11,111.72944095,9,-129.60483202,0\H,27,1.10011956,21,111.9
2641122,11,56.77685273,0\H,27,1.09689572,21,110.46670043,11,176.418439
94,0\H,27,1.09482094,21,112.20568368,11,-63.64585211,0\C,19,1.54628535
,11,111.13383307,9,163.79328556,0\H,31,1.09887202,19,110.68799736,11,-
62.36116,0\H,31,1.09621239,19,109.5861246,11,179.34939447,0\H,31,1.091
98302,19,113.76329098,11,59.38843496,0\C,19,1.54249089,11,113.55029273
,9,42.99245808,0\H,35,1.10011948,19,111.34463688,11,53.68041264,0\H,35
,1.09593139,19,109.66779501,11,171.8627086,0\H,35,1.09329506,19,113.43
445588,11,-68.86849538,0\C,17,1.54613514,13,115.74012999,9,43.2079478,
0\H,39,1.09688151,17,111.36264646,13,65.28900109,0\H,39,1.09641767,17,
109.08109664,13,-177.01679511,0\H,39,1.09727545,17,115.07546613,13,-57
.47743899,0\C,17,1.54677418,13,111.57463973,9,165.08119064,0\H,43,1.09
796071,17,110.97961421,13,-56.46473809,0\H,43,1.09205977,17,113.237528
81,13,65.26018912,0\H,43,1.09643189,17,109.78955985,13,-175.16589714,0
\C,15,1.54715513,13,113.06037702,9,-135.77869202,0\H,47,1.09688514,15,
109.90297929,13,167.51087331,0\H,47,1.0937157,15,112.61011715,13,-72.8
5207589,0\H,47,1.09666669,15,111.73072674,13,48.13041375,0\C,15,1.5448

0171,13,115.91100993,9,96.9364752,0\H,51,1.09491076,15,112.51398223,13
,68.18560764,0\H,51,1.09691758,15,109.43347952,13,-172.96206299,0\H,51
,1.09753009,15,113.50324917,13,-53.67371466,0\P,1,1.82778776,5,37.9076
5764,9,35.11807657,0\C,55,1.82845551,1,106.08506422,5,111.09982314,0\H
,56,1.09299533,55,111.56885746,1,75.07335572,0\H,56,1.09699817,55,107.
80355316,1,-44.31937822,0\H,56,1.0935509,55,110.65467736,1,-162.968903
59,0\\Version=AM64L-G03RevD.01\State=1-A\HF=-1045.5093266\MP2=-1048.45
92125\RMSD=1.174e-09\Thermal=0.\PG=C01 [X(C18H40P1)]\\@

316[a]

1\1\GINC-NODE21\SP\RMP2-FC\6-31+G(2d,p)\C22H30N1P1\ZIP04\07-Mar-2009\0
\\#p MP2(FC)/6-31+g(2d,p) scf=tight int=finegrid geom=check guess=read
\\newcat_001s2_spmp_005\\0,1\C,0,-0.0034597751,0.0530098812,-0.0234787
551\C,0,-0.0006898209,-0.0202678679,1.4014409111\C,0,1.2542699947,-0.0
229775378,2.0921041434\C,0,2.3886040883,-0.3641165038,1.353163959\C,0,
2.3816933209,-0.2858346999,-0.0389899695\C,0,1.2422019185,0.1296708465
,-0.7237637339\H,0,3.3351718965,-0.5192453087,1.8686300388\H,0,3.32252
9527,-0.3862481164,-0.5788265399\C,0,1.2668720735,3.174013727,-0.04428
71734\C,0,1.1249061576,3.1608955564,1.3422772946\C,0,2.157238314,2.689
8701353,2.1715096458\C,0,3.4229570816,2.536399014,1.5862168808\C,0,3.5
62254513,2.5371527615,0.1926671782\C,0,2.4446667521,2.6991518296,-0.64
10485714\H,0,0.4064921518,3.4006440741,-0.6701133536\H,0,0.1559069697,
3.3954352478,1.7811838979\H,0,4.2770080704,2.2631872811,2.2053861346\H
,0,4.5209064063,2.2571286621,-0.2431350844\C,0,1.8098837854,2.10660517
31,3.5275208207\C,0,1.5325624121,0.5239272403,3.4893203007\C,0,2.39270
47406,2.0807182073,-2.0278932533\C,0,1.4383185313,0.7964589844,-2.0873
117344\H,0,0.910102248,2.6035110986,3.9112596678\H,0,2.6084613381,2.28
1571264,4.2604226161\H,0,3.4077525423,1.793123124,-2.3302146533\H,0,2.
0275972178,2.7914000328,-2.7818059252\H,0,1.8749446725,0.0957802567,-2
.8135751767\H,0,0.4681683689,1.1178352451,-2.4692080066\H,0,0.73765301
78,0.3144640929,4.2088933181\P,0,-1.6315380857,-0.2728841923,2.2855694
238\C,0,-1.2253185586,-1.6095730774,3.5219729956\H,0,-0.9641517725,-2.
521421748,2.9725872893\H,0,-0.412758772,-1.3816539771,4.2200430157\H,0
,-2.1342794447,-1.8188380567,4.1007565926\C,0,-1.8655013235,1.20222523
05,3.40957424\H,0,-2.0022660289,2.1034717119,2.8003503472\H,0,-2.79732
40289,1.040607006,3.9670418724\H,0,-1.0612294889,1.3841676075,4.129923
6308\H,0,2.4332858545,0.0254402915,3.8689807896\C,0,-1.3128414899,-0.0
272744913,-0.8451582734\H,0,-2.1239510991,-0.2906023148,-0.14879348\C,
0,-1.216601634,-1.2022051599,-1.8413466695\H,0,-2.1894557107,-1.429454
1101,-2.291768965\H,0,-0.4942763288,-1.0168324138,-2.6443740161\H,0,-0
.8879761868,-2.0944357366,-1.2967820395\N,0,-1.651178103,1.2710481668,
-1.4914967686\C,0,-2.6200908167,1.1632901539,-2.580101619\H,0,-2.82118
21076,2.1699456604,-2.9667647514\H,0,-2.2179574683,0.5666154844,-3.404
1235161\H,0,-3.5894983083,0.7234493574,-2.2684771582\C,0,-2.1521068603
,2.2168258814,-0.4964472736\H,0,-1.4387888706,2.2976844481,0.328364084
6\H,0,-2.2656482749,3.2081773264,-0.9537913383\H,0,-3.1329042264,1.917
5056579,-0.0755423843\\Version=AM64L-G03RevD.01\State=1-A\HF=-1245.724
1608\MP2=-1249.3142071\RMSD=4.673e-09\Thermal=0.\PG=C01 [X(C22H30N1P1)
]\\@

316[a]-Me$^+$

1\1\GINC-NODE-07\SP\RMP2-FC\6-31+G(2d,p)\C23H33N1P1(1+)\ZIP04\09-Mar-2
009\0\\#p MP2(FC)/6-31+g(2d,p) scf=tight int=finegrid geom=check guess
=read\\newcat_001s2_met_spmp_002\\1,1\C,0,0.0212839095,-0.0566644533,0
.0104816009\C,0,0.0011594219,-0.054025272,1.4471147104\C,0,1.230164832
7,0.0022566613,2.1877204236\C,0,2.3748935501,-0.4428583078,1.530571217
6\C,0,2.3917796187,-0.5408323049,0.1447151697\C,0,1.2905367609,-0.1449
978539,-0.629485924\H,0,3.3063519854,-0.542638492,2.0834041545\H,0,3.3

311846992,-0.7702783963,-0.3524242241\C,0,1.1663829579,3.05574162,-0.0
257376718\C,0,1.2941554733,3.0769222136,1.3628246365\C,0,2.4561210181,
2.5796803766,1.9747242471\C,0,3.5748069666,2.3721343599,1.152269973\C,
0,3.4470947303,2.3539787485,-0.2417100761\C,0,2.18967975,2.5356397013,
-0.8367026352\H,0,0.2203786882,3.342873698,-0.4817998375\H,0,0.4403542
574,3.3701555964,1.9737759783\H,0,4.5256149444,2.0869938766,1.60039144
11\H,0,4.2999321668,2.0565168633,-0.8501228703\C,0,2.4071820896,2.0097
578251,3.3808203966\C,0,1.426828493,0.7507421903,3.5097661615\C,0,1.82
83526394,1.9087897695,-2.169567377\C,0,1.6317037952,0.3261755569,-2.04
73067111\H,0,3.4144097515,1.7001574074,3.6817179541\H,0,2.0709325587,2
.7477268452,4.1209443246\H,0,0.8965117907,2.3529557134,-2.5410255857\H
,0,2.5938568455,2.0824775835,-2.9355771555\H,0,2.5816779766,-0.1475776
188,-2.319081878\H,0,0.9118879464,0.0083593123,-2.8081094908\H,0,0.470
7322269,1.1305830199,3.8819049262\P,0,-1.4819281557,-0.2911825138,2.48
94105768\C,0,-1.0322024067,-1.1780603455,4.0280065625\H,0,-0.417595063
9,-2.0482737881,3.7754415223\H,0,-0.496546264,-0.5582968731,4.74804126
84\H,0,-1.9652353723,-1.5258535211,4.4872767124\C,0,-2.3404847197,1.23
28949963,3.0368116632\H,0,-3.1266854526,0.9563009677,3.7492913495\H,0,
-1.6328394133,1.9042923144,3.5334605635\H,0,-2.790217176,1.7598855661,
2.192745174\H,0,1.8320811202,0.0930038328,4.290142251\C,0,-1.196305432
4,0.1737028276,-0.9195459265\H,0,-0.7525820179,0.5329124139,-1.8604721
242\C,0,-2.143771217,1.305845357,-0.4581595798\H,0,-2.6320846194,1.777
2177953,-1.3168000156\H,0,-2.9417385862,0.9339046609,0.1937361547\H,0,
-1.5836677694,2.0858014446,0.0657810373\N,0,-1.9663623446,-1.064110694
4,-1.2117559603\C,0,-2.9803235102,-0.8310490193,-2.249282646\H,0,-2.54
06688207,-0.478159826,-3.2012170848\H,0,-3.5063101865,-1.7708257427,-2
.4453917998\H,0,-3.7216261518,-0.0994005156,-1.9166396795\C,0,-1.12629
29903,-2.2043192317,-1.5976183193\H,0,-1.7723997418,-3.0737453154,-1.7
588680631\H,0,-0.5562859406,-2.0271927717,-2.5275340987\H,0,-0.4179151
404,-2.4477010853,-0.8010364984\C,0,-2.6903459459,-1.4285693709,1.7414
754483\H,0,-3.6674027212,-1.2664579302,2.2116530892\H,0,-2.7396098357,
-1.3113269416,0.6466354674\H,0,-2.360840856,-2.4520878703,1.9552136151
\\Version=IA32L-G03RevD.01\State=1-A\HF=-1285.1711834\MP2=-1288.909836
7\RMSD=2.796e-09\Thermal=0.\PG=C01 [X(C23H33N1P1)]\\@

316[b]

1\1\GINC-NODE5\SP\RMP2-FC\6-31+G(2d,p)\C22H30N1P1\ZIP04\17-Jan-2009\0\
\#p MP2(FC)/6-31+g(2d,p) scf=tight int=finegrid geom=check guess=read\
\newcat_001.opt_b98opfr_spmp_004\\0,1\C,0,0.9421882519,-0.0121844078,-
0.328647375\C,0,0.0658017906,1.1137034206,-0.3867655654\C,0,-1.1819789
779,0.9926461181,-1.0768781484\C,0,-1.311128406,-0.0569522061,-1.98750
8679\C,0,-0.4643294625,-1.1604592411,-1.9086744103\C,0,0.5419966868,-1
.2359630831,-0.9410757873\H,0,-2.1871406461,-0.1047742971,-2.633593037
9\H,0,-0.6887309774,-2.0417557136,-2.5077602957\C,0,-1.2751739408,-1.4
758075413,1.738910322\C,0,-2.1861654723,-0.4348852514,1.5659315235\C,0
,-3.0868665289,-0.4500885965,0.4894411659\C,0,-3.2487303264,-1.6683601
477,-0.1890211911\C,0,-2.3356703297,-2.7147946157,-0.0129144238\C,0,-1
.2350438203,-2.5576628077,0.8426268612\H,0,-0.4993831714,-1.3828102249
,2.4956370756\H,0,-2.1001360146,0.4556575131,2.1870314979\H,0,-4.00303
21215,-1.7494315888,-0.9712172775\H,0,-2.3972466483,-3.5891055807,-0.6
602303174\C,0,-3.6098527016,0.8490074349,-0.0970659701\C,0,-2.45613394
33,1.7628315478,-0.7342991732\C,0,0.0710800003,-3.2971365274,0.6223841
393\C,0,0.9848773053,-2.6348539832,-0.5245498027\H,0,-4.3477497603,0.6
176933649,-0.8751836139\H,0,-4.1281758086,1.4586173937,0.6559559728\H,
0,0.6448716948,-3.3100873759,1.5575649196\H,0,-0.1055409341,-4.3443498
78,0.3447607426\H,0,0.9205967766,-3.2847843878,-1.4066500978\H,0,2.028
0620135,-2.6398856548,-0.2005848459\H,0,-2.2372867256,2.5589565482,-0.
0239001517\P,0,0.6687447829,2.7325816123,0.3300862516\C,0,-0.049231915

6,4.0367820457,-0.7934227636\H,0,0.3175495588,3.8561221365,-1.81060565
98\H,0,-1.1412748413,4.1077358927,-0.8239592929\H,0,0.3510861557,5.004
5986463,-0.4649884481\C,0,-0.3113489774,3.0000431226,1.8998948215\H,0,
-0.01013252,3.9672585664,2.322577937\H,0,-1.4012431531,2.9992339388,1.
7825015074\H,0,-0.0397092063,2.2252072179,2.6265646408\H,0,-2.87706241
91,2.2462808862,-1.627194753\C,0,2.2950567311,0.0727879638,0.408337482
6\H,0,2.5526106384,1.1443063892,0.4768001428\C,0,2.1940519599,-0.42813
20839,1.8642616515\H,0,3.0776624802,-0.1460555354,2.4476945329\H,0,1.3
246474828,0.0330263706,2.3444352674\H,0,2.0768174639,-1.51636257,1.919
4301751\N,0,3.3611033817,-0.6229609906,-0.3550647469\C,0,3.5693098475,
0.0089426494,-1.6595042399\H,0,3.9372159836,1.0515897851,-1.5768765364
\H,0,4.30861837,-0.5697727603,-2.2265222114\H,0,2.6351868096,0.0189941
351,-2.2276090148\C,0,4.634154884,-0.6723279594,0.3606117971\H,0,5.383
5026774,-1.1384467331,-0.2900667758\H,0,5.0145257152,0.32921234,0.6501
856375\H,0,4.5557268566,-1.2862324803,1.2628232348\\Version=AM64L-G03R
evD.01\State=1-A\HF=-1245.7268605\MP2=-1249.3155205\RMSD=6.737e-09\The
rmal=0.\PG=C01 [X(C22H30N1P1)]\\@

316[b]-Me[+]

1\1\GINC-NODE6\SP\RMP2-FC\6-31+G(2d,p)\C23H33N1P1(1+)\ZIP04\20-Jan-200
9\0\\#p MP2(FC)/6-31+g(2d,p) scf=tight int=finegrid geom=check guess=r
ead\\newcat_001_met_spmp_008\\1,1\C,0,-0.02136664,-0.01010886,0.001604
2939\C,0,-0.056713198,0.0212202569,1.4247475354\C,0,1.1768116798,0.063
4555822,2.1677764357\C,0,2.3005955626,-0.4401955293,1.5217069381\C,0,2
.3162739303,-0.5809949458,0.134137098\C,0,1.2360829578,-0.1588010187,-
0.6481158307\H,0,3.232292575,-0.5470151122,2.0725695299\H,0,3.24774960
49,-0.8579350906,-0.353899848\C,0,1.398020049,3.0495690163,-0.16937698
69\C,0,1.5035783327,3.1075293063,1.2197299679\C,0,2.6111487009,2.53921
3899,1.8698498979\C,0,3.7226096369,2.2172110965,1.0752544345\C,0,3.614
3942503,2.155760737,-0.3203013666\C,0,2.3853308719,2.416084867,-0.9430
930532\H,0,0.490938176,3.401169322,-0.6558277285\H,0,0.6703919565,3.49
51723407,1.8061695682\H,0,4.6410015849,1.8746683961,1.5500928854\H,0,4
.4496868665,1.7688871444,-0.9022330567\C,0,2.4913414561,2.0258781454,3
.2942143663\C,0,1.395204267,0.87322245,3.4512949161\C,0,1.9847791755,1
.7801058388,-2.260010209\C,0,1.5610748111,0.2409159663,-2.0879631044\H
,0,3.4620138671,1.6351692716,3.6203914441\H,0,2.2177170798,2.817568379
7,4.0041669932\H,0,2.7926703123,1.8157848378,-3.0004750541\H,0,1.13525
03335,2.3279439581,-2.6859146006\H,0,0.7494334084,0.0306999792,-2.7916
929513\H,0,2.407817137,-0.3749930657,-2.4115904728\H,0,1.7047349383,0.
2406164756,4.2937951791\P,0,-1.5687044406,-0.2451057442,2.4195911878\C
,0,-1.0342696175,-1.0147730118,4.0052299661\H,0,-0.3593040596,-1.85366
25068,3.8076845879\H,0,-0.5384276528,-0.310436345,4.6740711811\H,0,-1.
9287271256,-1.3946554862,4.5122230809\C,0,-2.5871644133,1.1771565857,2
.9648372163\H,0,-3.107575967,0.8930916642,3.8867424126\H,0,-1.94847437
87,2.0415693617,3.1735468392\H,0,-3.3165589517,1.4446279199,2.20046419
43\H,0,0.4578286653,1.3600202983,3.7428236322\C,0,-1.2600011856,0.1345
737867,-0.8963850277\H,0,-0.953553312,0.7810919554,-1.7383063108\C,0,-
1.6255172003,-1.2297106963,-1.5268004161\H,0,-0.7292146932,-1.65480635
6,-1.9886445726\H,0,-1.9833620835,-1.9544677845,-0.7896959872\H,0,-2.3
815364278,-1.1228449177,-2.3106871386\N,0,-2.3752628139,0.8084360505,-
0.1881654281\C,0,-2.1081901474,2.252745079,-0.1044235734\H,0,-2.928855
094,2.7546749735,0.4191178449\H,0,-1.1805499422,2.4304145805,0.4437985
854\H,0,-2.0172450516,2.7127827996,-1.1048072104\C,0,-3.6857490664,0.6
226546095,-0.8339299057\H,0,-3.9781983903,-0.4306937459,-0.8545290101\
H,0,-4.4406232445,1.1710288087,-0.2592933829\H,0,-3.7073033537,1.00437
0367,-1.8696615292\C,0,-2.6701030994,-1.5728470105,1.8145348466\H,0,-2
.0686127733,-2.4533879479,1.5652932375\H,0,-3.3469893574,-1.8364628098
,2.6358775969\H,0,-3.2548755974,-1.2616353774,0.9531496072\\Version=AM

64L-G03RevD.01\State=1-A\HF=-1285.1785605\MP2=-1288.919449\RMSD=3.261e
-09\Thermal=0.\PG=C01 [X(C23H33N1P1)]\\@

316[c]-Me[+]

1\1\GINC-NODE24\SP\RMP2-FC\6-31+G(2d,p)\C23H33N1P1(1+)\ZIP04\05-Mar-20
09\0\\#p MP2(FC)/6-31+g(2d,p) scf=tight int=finegrid geom=check guess=
read\\newcat_001s_nmet_spmp_004\\1,1\C,0,0.1550290103,-0.2136404248,-0
.2199978526\C,0,0.0688358742,-0.0698931022,1.2076925132\C,0,1.27189794
76,-0.0090378986,1.9718703316\C,0,2.4438535251,0.3204373579,1.28342707
15\C,0,2.5248662208,0.1495315569,-0.0947814758\C,0,1.4363314928,-0.320
5470999,-0.8437645245\H,0,3.3516499127,0.5408270668,1.8421045442\H,0,3
.4989072414,0.2273458779,-0.5728136559\C,0,1.2310088993,-3.3557264051,
0.4066986032\C,0,1.3104176083,-3.1389141573,1.7809611255\C,0,2.4618994
907,-2.5624023425,2.3403310277\C,0,3.6158437232,-2.5268813137,1.542151
4294\C,0,3.5395064765,-2.7564549069,0.1635509088\C,0,2.3007295398,-3.0
17414542,-0.4402787265\H,0,0.2909964145,-3.696886839,-0.0259063152\H,0
,0.4280089545,-3.2957593834,2.3990574868\H,0,4.5555950888,-2.182721890
8,1.9715670639\H,0,4.4212511321,-2.5914629533,-0.4541744263\C,0,2.3638
090336,-1.7345219622,3.6086660448\C,0,1.4240329994,-0.4521238208,3.426
1049377\C,0,2.0212315259,-2.666039261,-1.8869713799\C,0,1.8212302014,-
1.0922084157,-2.1124008771\H,0,3.3660439257,-1.4039093808,3.9045946871
\H,0,1.9605577596,-2.3108860927,4.4513808586\H,0,1.1145604673,-3.18526
56287,-2.2202751574\H,0,2.832903279,-2.9836745013,-2.5531389886\H,0,2.
7812840372,-0.6857357725,-2.4519441983\H,0,1.132773359,-0.963857159,-2
.9520555699\H,0,0.4496597269,-0.6927275566,3.8486511318\P,0,-1.6254187
613,0.1444292425,1.9922182527\C,0,-1.3675391996,1.3330813719,3.3965001
825\H,0,-0.9669715764,2.2737620926,3.0010699689\H,0,-0.7146615035,0.98
65095708,4.2024089318\H,0,-2.3560986359,1.5477066536,3.8209128131\C,0,
-2.0146725811,-1.4593507234,2.8527726767\H,0,-3.0008199258,-1.35247840
76,3.3212682379\H,0,-1.2966737293,-1.7596938751,3.6228424179\H,0,-2.08
6637652,-2.2612081809,2.1089973009\H,0,1.8416102702,0.3584318812,4.038
5442587\C,0,-1.1448368984,-0.2691925094,-1.0199824773\H,0,-1.960820520
2,-0.2051108521,-0.2950807121\C,0,-1.4023135341,-1.5121641834,-1.88164
74242\H,0,-2.4534481181,-1.5911091698,-2.1767808912\H,0,-1.1803505485,
-2.3832285158,-1.2574575112\H,0,-0.7801630628,-1.5897494925,-2.7757094
926\N,0,-1.3995477111,1.0782963969,-1.8380960563\C,0,-1.2034640466,2.2
620312881,-0.9238577324\H,0,-1.8625236982,2.1499564438,-0.0603996323\H
,0,-1.4512570799,3.1690813884,-1.481043839\H,0,-0.1631624307,2.2880192
608,-0.5987414949\C,0,-2.8278074709,1.0971005162,-2.3153758287\H,0,-3.
0350090121,2.0742283176,-2.7587414969\H,0,-3.4889820719,0.9306436215,-
1.4615254303\H,0,-2.9758255196,0.3209352248,-3.0664256611\C,0,-0.49318
53882,1.2521352234,-3.0210945521\H,0,0.5387400983,1.2670184069,-2.6697
764452\H,0,-0.649268459,0.435494844,-3.7267644173\H,0,-0.7372204369,2.
2018830662,-3.504773859\\Version=AM64L-G03RevD.01\State=1-A\HF=-1285.1
531638\MP2=-1288.8987283\RMSD=4.589e-09\Thermal=0.\PG=C01 [X(C23H33N1P
1)]\\@

317[a]

1\1\GINC-NODE-04\SP\RMP2-FC\6-31+G(2d,p)\C22H30N1P1\ZIP04\09-Mar-2009\
0\\#p MP2(FC)/6-31+g(2d,p) scf=tight int=finegrid geom=check guess=rea
d\\newcat_002s2_spmp_001\\0,1\C,0,0.0819411123,-0.196143527,0.06022585
82\C,0,0.0476786258,-0.1326730474,1.4623407668\C,0,1.2812440439,-0.030
6995336,2.1577622548\C,0,2.4425210581,-0.3851924626,1.4569539893\C,0,2
.4544181053,-0.4742405337,0.0607678716\C,0,1.289969792,-0.1947740414,-
0.6639112145\H,0,3.3892896007,-0.4323926453,1.994095414\H,0,3.40417018
18,-0.5997357565,-0.4576158721\C,0,0.5112563106,3.1551584258,-0.292027
2917\C,0,0.4364972487,3.1921504941,1.1085618333\C,0,1.501271501,2.7918
231662,1.9364211116\C,0,2.7528777324,2.665745221,1.323176707\C,0,2.830

114593,2.5628684169,-0.0670453574\C,0,1.6873381668,2.6204607879,-0.879
900776\H,0,-0.5152251616,3.4608246337,1.5695036846\H,0,3.6417115239,2.
4777713222,1.924031972\H,0,3.7799161632,2.2822710194,-0.5211100738\C,0
,1.2599871042,2.2877056074,3.3491135429\C,0,1.4275418945,0.700206473,3
.4873608975\C,0,1.7290222368,1.8875237003,-2.2107628883\C,0,1.33742725
87,0.3267316611,-2.0911059902\H,0,0.2410608951,2.552898489,3.653155790
2\H,0,1.946451787,2.7511721968,4.0710667253\H,0,2.7504391941,1.9401331
005,-2.6085927059\H,0,1.0723066143,2.3612963937,-2.9462496219\H,0,2.06
23384718,-0.2405681184,-2.6894582605\H,0,0.3573439788,0.1808141576,-2.
5622918457\H,0,2.4292653922,0.4918122544,3.8837064953\P,0,-1.534628257
9,0.0213697631,2.4145395213\C,0,-1.4773032219,-1.580534664,3.381372959
\H,0,-0.6623972409,-1.5515949044,4.1127625214\H,0,-2.4192909613,-1.698
5260716,3.9315561498\H,0,-1.3305284084,-2.4516513206,2.7307944977\C,0,
-2.81563463,-0.4878674108,1.161069297\H,0,-2.5383178489,-1.3949018252,
0.6083324087\H,0,-3.7483132062,-0.6823351495,1.7050694471\H,0,-3.01754
05542,0.3186890397,0.4478636524\H,0,0.7148818556,0.3628972018,4.249903
2386\C,0,-0.6964175787,3.6617668419,-1.0977106451\H,0,-1.2828976988,4.
2929992874,-0.3948910506\C,0,-1.6441545262,2.5298251023,-1.5403487282\
H,0,-2.6010943092,2.9227492162,-1.9013605341\H,0,-1.2010067256,1.91495
57266,-2.3318987984\H,0,-1.8548830649,1.8879021236,-0.6806701846\N,0,-
0.2528755564,4.4917404869,-2.2394576606\C,0,-1.3495281621,4.9174073993
,-3.1056541421\H,0,-2.1385079406,5.4848112187,-2.5689782882\H,0,-0.944
9584131,5.5689542831,-3.8889336448\H,0,-1.81656695,4.0605038376,-3.599
8523219\C,0,0.4897487502,5.6682356972,-1.7849772239\H,0,0.8559370729,6
.2224873645,-2.6573882315\H,0,-0.1313503062,6.3566156761,-1.1757978416
\H,0,1.3519004065,5.3640554732,-1.1859503041\H,0,-0.8485318393,-0.1431
347048,-0.4987306091\\Version=IA32L-G03RevD.01\State=1-A\HF=-1245.7447
115\MP2=-1249.3279962\RMSD=6.745e-09\Thermal=0.\PG=C01 [X(C22H30N1P1)]
\\@

317^{a}-Me^{+}

1\1\GINC-NODE15\SP\RMP2-FC\6-31+G(2d,p)\C23H33N1P1(1+)\ZIP04\09-Mar-20
09\0\\#p MP2(FC)/6-31+g(2d,p) scf=tight int=finegrid\\newcat_002s2_met
_spmp_004\\1,1\C\C,1,1.40989346\C,2,1.42553222,1,119.80153208\C,3,1.39
871741,2,115.56347266,1,-19.38654554,0\C,4,1.3983772,3,121.77257339,2,
16.46204454,0\C,5,1.40071911,4,120.55981372,3,2.54849097,0\H,4,1.08790
769,3,118.78422781,2,-173.4388888,0\H,5,1.08816288,4,118.91056357,3,17
2.69752866,0\C,6,3.30102574,5,106.35007545,4,69.27410438,0\C,9,1.40338
276,6,98.31137958,5,-59.82141829,0\C,10,1.40975899,9,122.39450752,6,61
.25213668,0\C,11,1.39955771,10,116.90703438,9,15.24927552,0\C,12,1.397
2764,11,119.43562812,10,-17.34318084,0\C,13,1.40245427,12,121.38523344
,11,1.51194552,0\H,10,1.08984827,9,119.31897677,6,-115.55657619,0\H,12
,1.08895045,11,120.34906976,10,171.7573844,0\H,13,1.08899851,12,119.01
323792,11,-170.04663481,0\C,11,1.51928739,10,121.21902248,9,-155.09731
335,0\C,3,1.52075391,2,124.61131642,1,145.47370419,0\C,14,1.52067348,1
3,118.17395981,12,-150.34025266,0\C,6,1.51992275,5,121.75584613,4,152.
51856225,0\H,18,1.09699093,11,111.64664751,10,-138.56986273,0\H,18,1.0
9727728,11,109.25039892,10,-20.91729266,0\H,20,1.09629633,14,113.74017
413,13,-141.33030539,0\H,20,1.09631276,14,108.67549572,13,-22.85416669
,0\H,21,1.09419164,6,108.66753801,5,176.06927342,0\H,21,1.09752946,6,1
11.28114945,5,57.61099213,0\H,19,1.09541448,3,108.18295054,2,166.07179
126,0\P,2,1.79907374,1,119.2980101,6,-171.54905398,0\C,29,1.82009592,2
,110.31572995,1,9.27551389,0\H,30,1.09471786,29,110.10301441,2,54.4901
0659,0\H,30,1.09592301,29,108.64548068,2,173.7875199,0\H,30,1.09368648
,29,110.92412871,2,-66.6733216,0\C,29,1.82367187,2,109.03840114,1,125.
66290662,0\H,34,1.09600835,29,110.03766904,2,-178.79800524,0\H,34,1.09
520868,29,109.08732725,2,-58.78551547,0\H,34,1.09428459,29,110.5884811
7,2,60.52671131,0\H,19,1.09871019,3,113.68679678,2,48.83467385,0\C,9,1

.538162,6,111.39160721,5,170.02794723,0\H,39,1.09506663,9,105.58022701
,6,-82.32456123,0\C,39,1.54576619,9,111.32851028,6,163.14695023,0\H,41
,1.09553146,39,109.74997278,9,57.97002797,0\H,41,1.09584854,39,112.916
39482,9,-61.87243645,0\H,41,1.09676868,39,110.7485664,9,176.95493906,0
\N,39,1.49039006,9,113.70805986,6,31.23909355,0\C,45,1.45646569,39,114
.19273324,9,85.00012119,0\H,46,1.10518583,45,114.37372923,39,66.185782
61,0\H,46,1.09891792,45,108.92817595,39,-174.16351278,0\H,46,1.0964441
2,45,109.6493639,39,-55.31935531,0\C,45,1.46567142,39,112.0778285,9,-1
48.75792246,0\H,50,1.09695466,45,109.22188168,39,174.88647683,0\H,50,1
.10566491,45,113.87780853,39,-64.71628512,0\H,50,1.09608767,45,109.809
61726,39,56.81344142,0\H,1,1.08647543,6,118.24580239,5,-170.62383923,0
\C,29,1.82673587,2,116.59307785,1,-112.73742152,0\H,55,1.0965176,29,10
9.07652755,2,178.23877198,0\H,55,1.09366311,29,110.73436317,2,-62.0008
9445,0\H,55,1.09431347,29,110.11863267,2,58.60685117,0\\Version=AM64L-
G03RevD.01\State=1-A\HF=-1285.1950167\MP2=-1288.9232276\RMSD=4.834e-09
\Thermal=0.\PG=C01 [X(C23H33N1P1)]\\@

317[b]

1\1\GINC-NODE5\SP\RMP2-FC\6-31+G(2d,p)\C22H30N1P1\ZIP04\19-Jan-2009\0\
\#p MP2(FC)/6-31+g(2d,p) scf=tight int=finegrid geom=check guess=read\
\newcat_002.opt_b98opfr_spmp_001\\0,1\C,0,-0.0678931757,-0.346736466,-
0.0521298766\C,0,-0.0719737053,-0.3645390206,1.3534539558\C,0,1.176991
6173,-0.3940141083,2.0269405869\C,0,2.3014965219,-0.7745943059,1.27939
13704\C,0,2.2821659909,-0.781799125,-0.1175899556\C,0,1.1233659735,-0.
3907809848,-0.799239825\H,0,3.2519717603,-0.9162567434,1.7927368122\H,
0,3.2123897884,-0.9329083256,-0.6636674726\C,0,0.622643277,2.810762139
2,-0.1922471798\C,0,0.5888912843,2.8077337685,1.2081025037\C,0,1.68525
34881,2.3960349951,1.9882394959\C,0,2.9215405719,2.2951178836,1.342541
7754\C,0,2.9692292442,2.2706182151,-0.0550200876\C,0,1.8056466441,2.35
83097613,-0.8322105682\H,0,-0.3507417638,3.0026726403,1.7184615945\H,0
,3.8230091577,2.074095584,1.9130399012\H,0,3.9102683966,2.0217233282,-
0.544690588\C,0,1.4549493426,1.8268896648,3.3769651923\C,0,1.404358196
8,0.2215267618,3.4020707483\C,0,1.800251636,1.702762008,-2.205070769\C
,0,1.1893528075,0.2162439565,-2.1904908681\H,0,0.5020713518,2.20260909
85,3.7669493437\H,0,2.2400558618,2.1402076895,4.077920986\H,0,2.835703
8494,1.636112458,-2.561508259\H,0,1.2444225265,2.3017606151,-2.9330394
738\H,0,1.8008324975,-0.397634032,-2.8651553606\H,0,0.1800482262,0.245
3494682,-2.620465594\H,0,2.364114458,-0.1443038418,3.7875874518\P,0,-1
.6231116814,-0.1268970597,2.3379528085\C,0,-1.7101679852,-1.7831338058
,3.2047513581\H,0,-0.8919265797,-1.8726402754,3.927671751\H,0,-2.65601
36772,-1.8483438206,3.7570862427\H,0,-1.6468682936,-2.6229024132,2.501
6131754\C,0,-2.9540126463,-0.4274200436,1.0695573757\H,0,-2.7784340888
,-1.3292746771,0.4686055454\H,0,-3.9034642215,-0.5445044025,1.60643621
31\H,0,-3.0614867312,0.4329162424,0.3993229236\H,0,0.6416060277,-0.069
7708376,4.1339001089\C,0,-0.6225508833,3.1426263512,-1.0266390187\H,0,
-0.8858926517,2.2311014436,-1.5868944134\C,0,-1.8647028909,3.521098539
2,-0.1898924406\H,0,-2.1716892897,2.6961087068,0.4634419222\H,0,-1.682
567235,4.3978587746,0.4428742256\H,0,-2.7096307445,3.751831854,-0.8492
248096\N,0,-0.2755066669,4.1355939679,-2.0689245626\C,0,-1.2526756085,
4.2274261268,-3.1433718419\H,0,-2.21269399,4.6988684645,-2.8530863179\
H,0,-0.8301597613,4.8237304563,-3.9625194043\H,0,-1.4714225151,3.22468
30339,-3.5328190541\C,0,0.0896183252,5.4515264137,-1.5579294933\H,0,0.
5293631238,6.0414485958,-2.372400008\H,0,-0.7648618151,6.027393435,-1.
1517511292\H,0,0.841843734,5.3449221848,-0.7695562266\H,0,-1.000727079
7,-0.1756403377,-0.5861217701\\Version=AM64L-G03RevD.01\State=1-A\HF=-
1245.7503932\MP2=-1249.3318048\RMSD=9.356e-09\Thermal=0.\PG=C01 [X(C22
H30N1P1)]\\@

317^{b}-Me^{+}

1\1\GINC-NODE6\SP\RMP2-FC\6-31+G(2d,p)\C23H33N1P1(1+)\ZIP04\19-Jan-200
9\0\\#p MP2(FC)/6-31+g(2d,p) scf=tight int=finegrid geom=check guess=r
ead\\newcat_002.optmet_spmp_002\\1,1\C,0,-0.0268318775,-0.05503941,-0.
0276297642\C,0,0.0023265858,-0.0682782209,1.3837060177\C,0,1.250928406
7,-0.0364760814,2.0677211177\C,0,2.3724172959,-0.3722686759,1.29847739
05\C,0,2.3301678749,-0.393753867,-0.0964566224\C,0,1.156906004,-0.0497
003185,-0.7823987482\H,0,3.3356833029,-0.46948775,1.7944633704\H,0,3.2
562854348,-0.5343063782,-0.6499226126\C,0,0.7399259973,3.111206866,-0.
2252128241\C,0,0.7384209077,3.1204654976,1.1738347339\C,0,1.8590280399
,2.7339803446,1.935392776\C,0,3.0812220001,2.6374739802,1.2632530448\C
,0,3.0962741021,2.5935527289,-0.1364969493\C,0,1.9125029669,2.65557673
06,-0.8837511081\H,0,-0.1832005418,3.3542393065,1.7021987644\H,0,4.000
9226884,2.4516499588,1.8159304798\H,0,4.0308349773,2.3625640884,-0.645
8589903\C,0,1.6772632166,2.205092264,3.3467664342\C,0,1.4953101345,0.6
019478241,3.4259007384\C,0,1.8663812155,1.9820793755,-2.246533144\C,0,
1.2055767045,0.5185338063,-2.1902185755\H,0,0.797785734,2.6734867919,3
.8061756795\H,0,2.5348489789,2.4589284589,3.9805695429\H,0,1.314433429
1,2.5818560563,-2.9760391703\H,0,2.8905524227,1.8750464614,-2.62144115
33\H,0,1.7802171549,-0.1352904686,-2.8574807029\H,0,0.1882816452,0.566
008623,-2.5965148191\H,0,0.7055829084,0.3830869137,4.156405272\P,0,-1.
5585214354,-0.1794892705,2.2727032223\C,0,-1.5218614682,-1.6206895261,
3.3886467718\H,0,-1.3514708757,-2.5285725088,2.8001186659\H,0,-0.70971
91848,-1.5188806519,4.1152344035\H,0,-2.4744781401,-1.707044323,3.9236
519789\C,0,-2.0231554035,1.2777613056,3.2705345141\H,0,-2.9933842316,1
.0876938387,3.7446929305\H,0,-1.2807794369,1.4733583167,4.0487101738\H
,0,-2.1057100162,2.1601869433,2.6283048711\H,0,2.4137580665,0.17610081
9,3.8440048922\C,0,-0.5051537233,3.4674884564,-1.0514627783\H,0,-0.775
4543232,2.570845702,-1.63342056\C,0,-1.7481766298,3.8321603759,-0.2077
647301\H,0,-1.5621431994,4.6779007896,0.4643352756\H,0,-2.0809820077,2
.9796472362,0.4013241229\H,0,-2.5827673201,4.1037792244,-0.8630649909\
N,0,-0.144521302,4.4764074585,-2.0649728016\C,0,-1.1126901697,4.598142
4902,-3.1489764077\H,0,-2.0698122963,5.0709857137,-2.8568758067\H,0,-0
.6761793689,5.2092574261,-3.9477783178\H,0,-1.3332907984,3.6066558964,
-3.5651429727\C,0,0.2291218037,5.7813632659,-1.5238703833\H,0,0.665341
671,6.3855903492,-2.3277981111\H,0,-0.6214102874,6.34926165,-1.1021801
842\H,0,0.9877288779,5.6596673233,-0.7437514391\H,0,-0.9740482811,0.04
38256697,-0.5527973581\C,0,-2.9320470538,-0.4500898798,1.1075706832\H,
0,-2.7412251808,-1.3295313795,0.4843919694\H,0,-3.8490481853,-0.616943
5264,1.6840995322\H,0,-3.0778892525,0.4255914891,0.4666849339\\Version
=AM64L-G03RevD.01\State=1-A\HF=-1285.2001173\MP2=-1288.9261702\RMSD=7.
770e-09\Thermal=0.\PG=C01 [X(C23H33N1P1)]\\@

317^{c}-Me^{+}

1\1\GINC-NODE22\SP\RMP2-FC\6-31+G(2d,p)\C23H33N1P1(1+)\ZIP04\06-Mar-20
09\0\\#p MP2(FC)/6-31+g(2d,p) scf=tight int=finegrid geom=check guess=
read\\newcat_002s_nmet_spmp_001\\1,1\C,0,0.003987937,0.2019368941,0.12
11209406\C,0,-0.0224557891,0.0033161058,1.5124379451\C,0,1.221982034,-
0.0571004342,2.1949140736\C,0,2.3457814409,0.4647592991,1.5361948052\C
,0,2.3452824282,0.6918194211,0.1561521339\C,0,1.2039534475,0.382233558
,-0.5925199746\H,0,3.2832332758,0.5554146871,2.0832652922\H,0,3.274819
7345,0.9621993431,-0.3422809797\C,0,0.7823616387,-2.9534046032,-0.5824
905997\C,0,0.6763231428,-3.1432424894,0.8084377261\C,0,1.7118624479,-2
.7980874122,1.6915015396\C,0,2.9662603349,-2.5488157683,1.1172550993\C
,0,3.0687557432,-2.2928840593,-0.2509846765\C,0,1.9482015117,-2.323575
6827,-1.0970573713\H,0,-0.2755712033,-3.451558347,1.2361784144\H,0,3.8
348498746,-2.3898566841,1.7534261285\H,0,4.0102547799,-1.9121651778,-0
.6419269954\C,0,1.4206019876,-2.4723222178,3.1455283674\C,0,1.44912383

67,-0.8997498733,3.444633547\C,0,1.971475396,-1.4197369444,-2.32543263
84\C,0,1.2847250987,0.0081054371,-2.0630864821\H,0,0.428490389,-2.8566
896639,3.4061796753\H,0,2.1440240966,-2.9512150917,3.8174711842\H,0,3.
0190441726,-1.2498276643,-2.5980859407\H,0,1.4986039274,-1.8585595554,
-3.2142671118\H,0,1.8525658979,0.7464259744,-2.6431268786\H,0,0.271217
5349,-0.0109559108,-2.4846502667\H,0,2.4322151198,-0.6462944088,3.8582
191362\P,0,-1.5909999648,-0.4066948408,2.4137642549\C,0,-1.6498796983,
0.980086304,3.6634201638\H,0,-0.8536325456,0.8689094852,4.4061861199\H
,0,-2.6085350154,0.9288067323,4.1935691656\H,0,-1.5538675899,1.9651360
675,3.1917465799\C,0,-2.8977222653,0.2433997663,1.2545273507\H,0,-2.65
95354475,1.238410641,0.8581576723\H,0,-3.8326775814,0.3110312571,1.823
8699397\H,0,-3.0827836868,-0.439953137,0.4179721815\H,0,0.7191135407,-
0.7135846416,4.2397985683\C,0,-0.3713675484,-3.3182492033,-1.497786651
9\H,0,-0.2719774338,-2.7765709303,-2.4431602206\C,0,-1.7700746996,-3.0
52413847,-0.9303427653\H,0,-1.7723471065,-2.0217648399,-0.5663290417\H
,0,-2.0299929932,-3.6889246161,-0.0788280391\H,0,-2.5546070119,-3.1362
09711,-1.687902296\N,0,-0.2372973851,-4.8340747713,-1.9944664902\C,0,-
1.360832629,-5.1680605346,-2.9380308344\H,0,-2.3038646576,-5.198334070
6,-2.3925195045\H,0,-1.1652821489,-6.1477733209,-3.3809568378\H,0,-1.4
037261367,-4.4102343422,-3.7244467202\C,0,-0.2575006716,-5.806411905,-
0.8478617277\H,0,-0.1607303657,-6.8171726311,-1.2521880925\H,0,-1.2034
438533,-5.7157415751,-0.3121890149\H,0,0.5746481123,-5.5822855977,-0.1
796250549\H,0,-0.9226156164,0.1331462813,-0.447079845\C,0,1.0596731329
,-5.0056544476,-2.7406298376\H,0,1.060128004,-4.3395324843,-3.60655834
21\H,0,1.8892536456,-4.754983056,-2.0795716177\H,0,1.1347332017,-6.044
3157633,-3.0719298405\\Version=AM64L-G03RevD.01\State=1-A\HF=-1285.174
8635\MP2=-1288.9128697\RMSD=6.050e-09\Thermal=0.\PG=C01 [X(C23H33N1P1)
]\\@

318

1\1\GINC-CIPCLU10\SP\RMP2-FC\6-31+G(2d,p)\C9H17P1\C2175\22-Apr-2010\0\
\#p MP2(FC)/6-31+G(2d,p) scf=tight\\kno1sp_2\\0,1\P\C,1,1.8660484\H,2,
1.09736555,1,108.564698\H,2,1.09479309,1,110.18277352,3,-117.86270502,
0\H,2,1.09896002,1,112.94416885,3,120.2271005,0\C,1,1.86604603,2,98.22
952441,4,175.88092453,0\H,6,1.0973681,1,108.56274205,2,66.25428032,0\H
,6,1.09896032,1,112.94409177,2,-53.97112907,0\H,6,1.09479439,1,110.185
04325,2,-175.88362568,0\C,1,1.89657365,6,101.23660907,2,103.26812924,0
\H,10,1.10343064,1,109.09100872,6,-50.39198145,0\C,10,1.52848801,1,109
.43290424,6,-169.63646899,0\C,12,1.51954627,10,121.13064828,1,160.4942
0002,0\C,13,1.5160124,12,60.07725514,10,-111.55496612,0\H,12,1.0911690
8,10,113.46249093,1,-56.41922779,0\H,13,1.08775627,12,117.93945506,10,
-4.21154906,0\H,13,1.08782017,12,118.32081468,10,139.86460514,0\H,14,1
.08832389,13,117.40146893,12,108.15930168,0\H,14,1.08809156,13,118.638
76717,12,-108.16119074,0\C,10,1.52847823,1,109.42959551,6,68.84940586,
0\C,20,1.51955842,10,122.11617354,1,-88.77061649,0\C,21,1.5160117,20,6
0.07640907,10,-109.95760703,0\H,20,1.09116994,10,113.46163608,1,56.426
16436,0\H,21,1.0883251,20,117.99670544,10,-2.77752891,0\H,21,1.0880910
7,20,118.43540915,10,141.54839403,0\H,22,1.08775816,21,117.51204486,20
,108.04418285,0\H,22,1.08781888,21,118.67033538,20,-108.0094374,0\\Ver
sion=AM64L-G03RevD.01\State=1-A\HF=-691.4046403\MP2=-692.8906805\RMSD=
7.502e-09\Thermal=0.\PG=C01 [X(C9H17P1)]\\@

318-Me^{+}

1\1\GINC-CIPCLU09\SP\RMP2-FC\6-31+G(2d,p)\C10H20P1(1+)\C2175\22-Apr-20
10\0\\#p MP2(FC)/6-31+G(2d,p) scf=tight\\kno1mesp_3\\1,1\C\H,1,1.09561
153\H,1,1.09394984,2,108.97698942\H,1,1.09608535,3,109.26473419,2,-118
.77602,0\C,1,2.94752133,3,90.27630432,2,151.52546539,0\H,5,1.09575205,
1,88.93831315,3,107.74711497,0\H,5,1.09508765,1,146.12984662,3,-128.88

905496,0\H,5,1.09508529,1,90.9538174,3,-1.01417954,0\C,5,3.01409791,1,
61.31996121,3,-91.28169787,0\H,9,1.10199713,5,139.17452551,1,-34.63036
802,0\P,5,1.82002132,1,35.95248419,9,-34.17352863,0\C,11,1.8207405,5,1
08.11508097,1,-116.35636468,0\H,12,1.09561033,11,110.1284861,5,179.611
68142,0\H,12,1.09608438,11,109.4927122,5,60.00608312,0\H,12,1.09395023
,11,110.14141732,5,-60.16932537,0\C,9,1.52831075,5,92.30671637,1,88.88
043656,0\C,16,1.5261825,9,122.00156718,5,-112.75611962,0\C,17,1.509878
47,16,60.08750754,9,-107.66892638,0\H,16,1.09034064,9,114.0504044,5,35
.47612266,0\H,17,1.08859838,16,118.34642753,9,-0.94613322,0\H,17,1.087
27818,16,118.10213619,9,143.7080431,0\H,18,1.0873003,17,117.68970143,1
6,108.23170918,0\H,18,1.08648073,17,118.72724775,16,-107.111779,0\C,9,
1.52829667,5,92.32448576,1,-158.16810178,0\C,24,1.52010861,9,119.42161
427,5,-176.88181556,0\C,25,1.50988247,24,60.48727477,9,-111.93550113,0
\H,24,1.09034878,9,114.04930927,5,-35.46976038,0\H,25,1.08730228,24,11
8.03361842,9,-4.26761959,0\H,25,1.08648036,24,117.47301562,9,138.90338
392,0\H,26,1.08859664,25,117.14710786,24,108.69852714,0\H,26,1.0872782
7,25,118.62408767,24,-107.7643532,0\\Version=AM64L-G03RevD.01\State=1-
A\HF=-730.8576914\MP2=-732.4879829\RMSD=4.028e-09\Thermal=0.\PG=C01 [X
(C10H20P1)]\\@

319

1\1\GINC-GOLEM\SP\RMP2-FC\6-31+G(2d,p)\C8H16N1P1\CHRISTOPH\28-Sep-2009
\0\\#p MP2(FC)/6-31+g(2d,p) scf=tight\\pxsp_18\\0,1\C\C,1,1.54133724\P
,2,1.89298355,1,108.45713298\C,3,1.90090712,2,91.05874601,1,-12.759015
75,0\C,1,1.54102151,2,106.46372043,3,36.08509341,0\H,1,1.09870311,5,11
1.77068709,4,-169.98142726,0\H,1,1.09951688,5,109.37899303,4,71.547307
7,0\H,2,1.09675261,1,113.76442152,5,159.405855,0\H,2,1.09898732,1,109.
98878375,5,-80.63099414,0\H,4,1.09621624,3,110.28978579,2,-136.4447631
3,0\H,4,1.09878373,3,108.97514208,2,106.19097267,0\H,5,1.09999929,1,10
9.15594263,2,71.51471639,0\H,5,1.09845028,1,112.0023393,2,-170.3023651
5,0\C,3,2.76928467,2,123.89823688,1,113.04528447,0\C,14,1.53621432,3,1
33.17038461,2,-22.2713035,0\C,15,1.54092165,14,102.29870656,3,-49.3685
8877,0\C,16,1.54546765,15,103.52130404,14,37.17929725,0\H,14,1.1049425
3,3,103.32429939,2,-157.23252254,0\H,14,1.09630583,3,85.68224268,2,95.
78141303,0\H,15,1.0979681,14,109.82593225,3,67.72771636,0\H,15,1.09599
528,14,112.90369153,3,-171.28681839,0\H,16,1.09800571,15,109.78069648,
14,-80.18929994,0\H,16,1.09588351,15,113.40831996,14,159.2634147,0\H,1
7,1.10082784,16,111.30645989,15,-145.29769037,0\H,17,1.10200248,16,111
.14218345,15,95.4373189,0\N,17,1.46982014,16,104.64896029,15,-23.92290
286,0\\Version=AM64L-G03RevD.01\State=1-A\HF=-707.4680779\MP2=-708.980
1698\RMSD=3.650e-09\Thermal=0.\PG=C01 [X(C8H16N1P1)]\\@

319-Me$^+$

1\1\GINC-YANG\SP\RMP2-FC\6-31+G(2d,p)\C9H19N1P1(1+)\CHRISTOPH\28-Sep-2
009\0\\#p MP2(FC)/6-31+g(2d,p) scf=tight\\pxmesp_5\\1,1\C\C,1,1.552397
79\P,2,1.851426,1,104.61286458\C,3,1.83521328,2,96.7768974,1,-9.095572
28,0\C,1,1.54528891,2,107.68729772,3,33.05429741,0\H,1,1.09458113,5,11
1.72176004,4,-169.80871085,0\H,1,1.09783261,5,109.70491896,4,71.447564
43,0\H,2,1.09793549,1,111.20762298,5,-83.79120349,0\H,2,1.0955009,1,11
3.72197673,5,154.62571199,0\H,4,1.09530451,3,111.23796671,2,-140.32005
327,0\H,4,1.09741235,3,108.4136416,2,101.48902907,0\H,5,1.09823262,1,1
10.03478333,2,71.776907,0\H,5,1.09475636,1,111.81693682,2,-169.3538373
3,0\C,3,1.81599283,2,109.6467048,1,-124.78762265,0\H,14,1.09491321,3,1
10.18510285,2,175.31574066,0\H,14,1.09535575,3,110.81630769,2,-63.3918
9574,0\H,14,1.09597517,3,108.75333604,2,55.74863433,0\C,3,2.75377956,2
,104.53155276,1,84.92310021,0\C,18,1.5397241,3,130.68151101,2,91.05273
546,0\C,19,1.53840978,18,103.66373768,3,43.73326109,0\C,20,1.53534187,
19,103.1729924,18,-39.37732394,0\H,18,1.0972902,3,85.9329582,2,-24.671

42635,0\H,18,1.09693613,3,104.27598144,2,-132.79476659,0\H,19,1.093917
58,18,111.62602671,3,165.84669914,0\H,19,1.09678201,18,110.09612311,3,
-74.24205143,0\H,20,1.09649587,19,110.66565223,18,78.16753513,0\H,20,1
.09397834,19,113.04440039,18,-160.22240107,0\H,21,1.09450642,20,112.76
830083,19,154.31160777,0\H,21,1.09971609,20,110.92647629,19,-83.303435
41,0\N,21,1.49388729,20,102.68935959,19,35.47218148,0\\Version=AM64L-G
03RevD.01\State=1-A\HF=-746.924532\MP2=-748.579567\RMSD=2.976e-09\Ther
mal=0.\PG=C01 [X(C9H19N1P1)]\\@

320
1\1\GINC-EDDY\SP\RMP2-FC\6-31+G(2d,p)\C7H16N1P1\CHRISTOPH\04-Oct-2009\
0\\#p MP2(FC)/6-31+g(2d,p) scf=tight\\dzsp_3\\0,1\C\C,1,1.54552376\C,2
,1.54050524,1,112.83357683\C,3,1.54089161,2,113.29438464,1,-59.7579589
2,0\C,4,1.54540228,3,112.94987863,2,59.73062025,0\H,1,1.10101301,2,109
.49744411,3,-59.33104155,0\H,1,1.09856569,2,110.22523767,3,-177.073438
92,0\H,2,1.10089248,1,109.4249751,3,-121.77622684,0\H,2,1.09955769,1,1
09.37794741,3,122.11692252,0\H,3,1.10160576,2,109.08645582,1,61.996603
74,0\H,3,1.09908827,2,109.33395937,1,178.05414127,0\H,4,1.09935362,3,1
09.4567605,2,-178.30807419,0\H,4,1.10075934,3,109.16849246,2,-62.28644
658,0\H,5,1.10103397,4,109.35552127,3,59.68016206,0\H,5,1.0976897,4,11
0.55772528,3,177.41539934,0\P,5,1.86841823,4,110.34181356,3,-63.231479
32,0\C,16,2.80582311,5,89.48533115,4,144.57197204,0\H,17,1.10522421,16
,108.55115886,5,-134.77837583,0\H,17,1.10026375,16,135.63714302,5,82.2
0748007,0\H,17,1.09450228,16,84.51272649,5,-27.44960795,0\C,17,2.42896
138,16,62.0714125,5,139.33635245,0\H,21,1.09448721,17,143.23167608,16,
-18.13056086,0\H,21,1.10062637,17,91.3002515,16,-141.9441198,0\H,21,1.
105524,17,94.11700732,16,110.35090024,0\N,17,1.45603755,16,30.75337569
,5,121.68475342,0\\Version=AM64L-G03RevD.01\State=1-A\HF=-669.5963816\
MP2=-670.9770286\RMSD=9.826e-09\Thermal=0.\PG=C01 [X(C7H16N1P1)]\\@

320-Me$^+$
1\1\GINC-AZAZEL\SP\RMP2-FC\6-31+G(2d,p)\C8H19N1P1(1+)\CHRISTOPH\04-Oct
-2009\0\\#p MP2(FC)/6-31+g(2d,p) scf=tight\\dzmesp_2\\1,1\C\C,1,1.5499
8857\C,2,1.5416443,1,113.14837344\C,3,1.54146014,2,114.03218791,1,61.4
2501068,0\C,4,1.55013867,3,113.17351377,2,-61.40444608,0\H,1,1.1000491
1,2,110.5029118,3,59.64740218,0\H,1,1.0958634,2,112.2501055,3,178.3700
0623,0\H,2,1.09775757,1,109.49888784,3,123.0575754,0\H,2,1.09628225,1,
107.51987519,3,-121.29413387,0\H,3,1.09611113,2,108.6404992,1,-177.265
57676,0\H,3,1.10023109,2,109.46268604,1,-61.53644539,0\H,4,1.096281,3,
109.66388979,2,178.5776295,0\H,4,1.09774122,3,109.99079938,2,61.463857
83,0\H,5,1.10006335,4,110.5577996,3,-59.66877279,0\H,5,1.09579983,4,11
2.27197974,3,-178.41836252,0\C,5,3.28644349,4,99.69561215,3,114.115932
62,0\H,16,1.09240397,5,51.91645404,4,86.53426887,0\H,16,1.09490966,5,1
30.83099057,4,6.21831638,0\H,16,1.09903742,5,120.16738601,4,178.195824
02,0\C,16,2.45913644,5,93.51502346,4,-84.18345182,0\H,20,1.0947527,16,
87.07076802,5,132.46303784,0\H,20,1.09911753,16,95.85512919,5,-119.157
75669,0\H,20,1.09240737,16,144.08565031,5,15.9566241,0\P,1,1.82286784,
2,110.87894847,3,-57.12334206,0\C,24,1.82437621,1,109.85773398,2,167.5
8146017,0\H,25,1.09618683,24,110.44931382,1,-56.77906943,0\H,25,1.0961
2059,24,109.99466418,1,63.29886803,0\H,25,1.09610694,24,110.02042426,1
,-176.88852592,0\N,20,1.47631201,16,33.60805134,5,2.98787691,0\\Versio
n=AM64L-G03RevD.01\State=1-A\HF=-709.0505301\MP2=-710.5767047\RMSD=3.4
68e-09\Thermal=0.\PG=C01 [X(C8H19N1P1)]\\@

321
1\1\GINC-NODE11\SP\RMP2-FC\6-31+G(2d,p)\C17H29P1\ZIP07\05-May-2010\0\\
#p MP2(FC)/6-31+G(2d,p) scf=tight\\kno2sp_105\\0,1\P\C,1,1.87254604\H,
2,1.09740096,1,108.14523008\H,2,1.09976632,1,113.04313406,3,119.882301

55,0\H,2,1.09586329,1,111.31311429,3,-117.38804759,0\C,1,1.86859415,2,
96.64888917,5,-178.43172341,0\H,6,1.09760073,1,108.18655704,2,62.66393
956,0\H,6,1.09455209,1,110.40929494,2,179.94944437,0\H,6,1.09886214,1,
113.10525086,2,-57.44762734,0\C,1,1.90387642,6,101.21895979,2,100.8549
9352,0\H,10,1.10071237,1,105.72561469,6,-48.78640043,0\C,10,1.5714196,
1,111.13164231,6,-162.5703102,0\H,12,1.10093984,10,107.42497701,1,42.7
9081176,0\C,10,1.57405829,1,114.48062677,6,68.38790917,0\H,14,1.102338
32,10,106.12045891,1,151.45818481,0\C,12,1.53268859,10,110.69507652,1,
158.12440514,0\C,16,1.51793298,12,120.56137353,10,-75.11209135,0\C,16,
1.51679958,12,120.65554904,10,-146.20763803,0\H,16,1.09087297,12,114.6
117039,10,69.77147617,0\H,17,1.08929879,16,117.71642208,12,-2.57771856
,0\H,17,1.08803937,16,118.76858128,12,141.72732619,0\H,18,1.08821749,1
6,117.80945251,12,2.47845501,0\H,18,1.08784105,16,118.6013007,12,-141.
80308168,0\C,12,1.5284885,10,114.98836335,1,-76.35559358,0\C,24,1.5187
172,12,120.45858414,10,153.93860202,0\C,25,1.51645661,24,60.06687802,1
2,-111.74646754,0\H,24,1.09057054,12,114.20994404,10,-63.66556592,0\H,
25,1.08831292,24,117.95312083,12,-4.23437559,0\H,25,1.08786804,24,118.
3957941,12,139.77413367,0\H,26,1.08776407,25,118.07560665,24,107.87774
277,0\H,26,1.08808508,25,118.61569201,24,-107.95366312,0\C,14,1.538494
23,10,113.21705532,1,35.53524219,0\C,32,1.52161692,14,124.34944066,10,
-107.00741521,0\C,33,1.51677475,32,59.96179143,14,-110.99432466,0\H,32
,1.08810593,14,112.68757935,10,37.191789,0\H,33,1.0873354,32,118.92839
929,14,-5.10902839,0\H,33,1.08786084,32,117.77599744,14,139.85606745,0
\H,34,1.08711453,33,116.98002936,32,108.72200053,0\H,34,1.08824309,33,
118.62636315,32,-107.71405182,0\C,14,1.52859296,10,111.48476444,1,-94.
37148536,0\C,40,1.51798265,14,119.74613156,10,-74.07141221,0\C,41,1.51
63421,40,60.17581931,14,-110.69286742,0\H,40,1.08836589,14,115.1714860
1,10,69.1512048,0\H,41,1.08873671,40,117.71197152,14,-2.7793882,0\H,41
,1.08784211,40,118.36393375,14,141.09613556,0\H,42,1.0884671,41,117.45
576139,40,107.9293886,0\H,42,1.08808882,41,118.53456211,40,-108.406686
32,0\\Version=AM64L-G03RevD.01\State=1-A\HF=-1001.2815477\MP2=-1003.97
64522\RMSD=1.356e-09\Thermal=0.\PG=C01 [X(C17H29P1)]\\@

321-Me$^+$

1\1\GINC-NODE28\SP\RMP2-FC\6-31+G(2d,p)\C18H32P1(1+)\ZIP07\01-May-2010
\0\\#p MP2(FC)/6-31+G(2d,p) scf=tight\\kno2mesp_30\\1,1\C\H,1,1.094291
58\H,1,1.09591227,2,108.52531021\H,1,1.09567622,2,109.0060162,3,-118.3
0874422,0\C,1,2.91075071,2,147.25865391,3,109.01650762,0\H,5,1.0962234
5,1,85.37326606,2,-113.47797675,0\H,5,1.09345663,1,146.80265645,2,4.39
537729,0\H,5,1.09501451,1,93.01865836,2,137.70195148,0\C,1,2.97786465,
5,61.67621425,7,-49.02577903,0\H,9,1.10271968,1,81.36498127,5,-82.6576
6946,0\P,5,1.82394319,1,37.14805642,9,-36.35853342,0\C,11,1.82147548,5
,106.65341437,1,-113.33377346,0\H,12,1.09249564,11,110.53461337,5,-176
.85496045,0\H,12,1.09662064,11,108.30148182,5,63.32563678,0\H,12,1.092
43697,11,109.94918712,5,-56.00942233,0\C,9,1.57992752,1,151.25674481,1
1,-12.7320177,0\H,16,1.09960559,9,103.66015302,1,168.46357643,0\C,9,1.
58133587,1,89.1770116,11,135.53785317,0\H,18,1.10107734,9,107.66552116
,1,13.00189335,0\C,18,1.53336513,9,108.83757132,1,127.47780479,0\C,20,
1.5215805,18,120.76119994,9,-72.01411855,0\C,21,1.51485013,20,59.95603
7,18,-109.30362051,0\H,20,1.08983403,18,114.5541555,9,74.10134078,0\H,
21,1.08995469,20,118.22868969,18,-2.42277172,0\H,21,1.08727142,20,118.
71792688,18,142.37176975,0\H,22,1.08808558,21,117.45828107,20,108.4809
5712,0\H,22,1.08675839,21,118.55992641,20,-107.84232976,0\C,18,1.52753
963,9,115.15213421,1,-107.3472847,0\C,28,1.51597949,18,119.82874296,9,
166.27545264,0\C,29,1.51302537,28,60.57367821,18,-112.48451429,0\H,28,
1.08928812,18,114.34977319,9,-52.70606032,0\H,29,1.08688532,28,118.176
12018,18,-5.11586109,0\H,29,1.08660799,28,117.97380996,18,138.90468108
,0\H,30,1.0900699,29,116.45263697,28,109.19537987,0\H,30,1.08813566,29

,118.56642756,28,-108.52868588,0\C,16,1.52847318,9,111.2325048,1,-78.1
800935,0\C,36,1.52261729,16,119.78753564,9,-70.56207187,0\C,37,1.51230
11,36,60.15346082,16,-110.2140194,0\H,36,1.0891718,16,115.55938352,9,7
3.64462383,0\H,37,1.08835633,36,117.83839392,16,-2.70302633,0\H,37,1.0
8732534,36,118.33039596,16,141.59337673,0\H,38,1.08809343,37,117.47131
38,36,108.2116253,0\H,38,1.0871009,37,118.52557654,36,-107.98441652,0\
C,16,1.53710251,9,114.51030156,1,53.1933223,0\C,44,1.53136378,16,124.4
2113226,9,-106.90582221,0\C,45,1.513101,44,59.72184822,16,-109.3836893
5,0\H,44,1.08963228,16,113.22895717,9,39.95698087,0\H,45,1.08741267,44
,119.35494303,16,-3.80919961,0\H,45,1.08878274,44,118.4547709,16,142.9
1573459,0\H,46,1.08606251,45,116.99574778,44,108.84842373,0\H,46,1.086
97852,45,118.33874783,44,-107.70442981,0\\Version=AM64L-G03RevD.01\Sta
te=1-A\HF=-1040.7355205\MP2=-1043.5760662\RMSD=9.474e-10\Thermal=0.\PG
=C01 [X(C18H32P1)]\\@

322

1\1\GINC-MOKKORI\SP\RMP2-FC\6-31+G(2d,p)\C16H25P1\CHRISTOPH\13-Feb-201
0\0\\#p MP2(FC)/6-31+g(2d,p) scf=tight\\ps11sp_1\\0,1\C\C,1,1.55369829
\C,2,1.54415731,1,110.74667905\C,3,1.55126889,2,110.61221627,1,-60.347
97745,0\C,4,1.55126652,3,108.09922437,2,58.73801219,0\C,5,1.54415597,4
,110.61226504,3,-58.73786466,0\H,2,1.10052855,1,109.77929666,6,-62.835
01762,0\H,2,1.1002237,1,110.18921241,6,179.61402879,0\H,3,1.1017878,2,
108.91201512,1,179.76567091,0\H,4,1.1013735,3,109.053697,2,-59.7021416
5,0\H,5,1.10178656,4,109.09341843,3,-178.51579339,0\H,6,1.1005289,5,10
9.91098192,4,-61.12383966,0\H,6,1.10022391,5,109.17658541,4,-178.15395
199,0\C,3,1.55142826,2,110.48214964,1,59.92374933,0\H,14,1.10175986,3,
109.07276562,2,59.65633023,0\C,1,1.5480289,2,108.40336735,3,-58.442411
42,0\H,16,1.09983642,1,110.49957637,2,179.25334749,0\H,16,1.09983706,1
,110.49960828,2,-62.47301635,0\C,5,1.55143113,4,108.59137143,3,62.6575
7946,0\H,19,1.10176062,5,109.07270687,4,178.86801181,0\C,16,1.5463637,
1,110.52174632,2,58.39009363,0\H,21,1.10189953,16,108.90613941,1,-180.
,0\C,4,1.54364406,3,110.53968876,2,179.84493391,0\H,23,1.10063041,4,10
9.91298926,3,178.87156582,0\H,23,1.10063097,4,109.91295708,3,61.481467
73,0\C,19,1.54339072,5,110.54791595,4,58.48290826,0\C,14,1.54338963,3,
110.54795309,2,-179.95835471,0\C,23,1.54379792,4,109.81430257,3,-59.82
350345,0\H,27,1.10063588,14,109.94428943,3,-178.87583841,0\H,27,1.1005
3409,14,109.91132159,3,-61.38835415,0\H,26,1.10053446,19,109.91122109,
5,61.38821672,0\H,26,1.10063546,19,109.94431689,5,178.87571557,0\H,28,
1.09944288,23,109.74542102,4,180.,0\P,1,1.89962817,16,116.35483138,21,
-180.,0\C,34,1.86742806,1,102.97169865,16,-51.03617393,0\H,35,1.096056
08,34,110.3083774,1,-68.21949515,0\H,35,1.09736756,34,113.86739837,1,5
4.50247279,0\H,35,1.09728775,34,108.09165309,1,174.80200035,0\C,34,1.8
6742735,1,102.97169684,16,51.03202358,0\H,39,1.0972877,34,108.09163268
,1,-174.80476863,0\H,39,1.09736778,34,113.86731934,1,-54.50531875,0\H,
39,1.09605587,34,110.30843569,1,68.21669147,0\\Version=IA32L-G03RevD.0
1\State=1-A\HF=-961.271753\MP2=-963.8007583\RMSD=5.851e-09\Thermal=0.\
PG=C01 [X(C16H25P1)]\\@

322-Me$^+$

1\1\GINC-GOLEM\SP\RMP2-FC\6-31+G(2d,p)\C17H28P1(1+)\CHRISTOPH\15-Feb-2
010\0\\#p MP2(FC)/6-31+g(2d,p) scf=tight\\ps11mesp_1\\1,1\C\C,1,1.5562
3535\C,2,1.54716427,1,109.29604039\C,3,1.5520847,2,110.738361,1,-60.29
543186,0\C,4,1.5513271,3,108.13475047,2,59.60015437,0\C,5,1.54658253,4
,110.49598279,3,-59.74262646,0\H,2,1.10022994,1,110.51254843,6,-61.110
2676,0\H,2,1.09836548,1,110.44976608,6,179.73308272,0\H,3,1.09948494,2
,107.85781476,1,-179.91115667,0\H,4,1.10123165,3,109.21266502,2,-59.22
294735,0\H,5,1.09933864,4,109.26415432,3,-178.39858241,0\H,6,1.0984638
9,5,109.1306695,4,-60.28896112,0\H,6,1.10073274,5,109.38989022,4,-177.

9791104,0\C,3,1.55121488,2,110.59226329,1,60.49186486,0\H,14,1.1014491
2,3,109.24043511,2,59.14239755,0\C,1,1.55668272,2,109.07090803,3,-59.6
5662841,0\H,16,1.09832684,1,110.66189563,2,-179.93366068,0\H,16,1.1000
6088,1,110.53635269,2,-60.67298786,0\C,5,1.55062401,4,109.04839464,3,6
2.19202148,0\H,19,1.10154974,5,109.31278872,4,179.05608631,0\C,16,1.54
599237,1,109.34673588,2,59.6329465,0\H,21,1.09914735,16,107.9657124,1,
179.99496948,0\C,4,1.54334234,3,110.35085723,2,-179.73360486,0\H,23,1.
09910641,4,109.75160019,3,178.77722614,0\H,23,1.09895549,4,109.6369424
5,3,61.62432435,0\C,19,1.54324064,5,110.27876838,4,58.56234824,0\C,14,
1.54329773,3,110.39629152,2,179.67546834,0\C,23,1.54444202,4,109.82145
074,3,-59.76960563,0\H,27,1.09910526,14,109.6965243,3,-178.93276447,0\
H,27,1.09929216,14,109.70134291,3,-61.71289707,0\H,26,1.09928164,19,10
9.7388249,5,61.70288487,0\H,26,1.09881673,19,109.72976561,5,178.954793
17,0\H,28,1.0974315,23,109.71629742,4,179.99384038,0\C,1,3.04092203,2,
143.60686082,3,179.38755316,0\H,34,1.09419587,1,91.54005195,2,126.5290
5829,0\H,34,1.09535475,1,91.39472972,2,-124.42619321,0\H,34,1.09567413
,1,143.90723088,2,0.88889939,0\C,34,2.93700445,1,61.05706215,2,-33.785
1737,0\H,38,1.09558209,34,88.16752235,1,160.32999548,0\H,38,1.09399069
,34,91.10052312,1,-90.89240529,0\H,38,1.09489927,34,146.76900051,1,37.
72549099,0\P,38,1.82231146,34,36.33707078,1,33.15139793,0\C,42,1.82366
393,38,107.46640865,34,115.26316044,0\H,43,1.09577225,42,109.33336049,
38,-57.86561004,0\H,43,1.09467783,42,110.57284567,38,61.87237272,0\H,4
3,1.09527974,42,110.35768666,38,-177.45981316,0\\Version=AM64L-G03RevD
.01\State=1-A\HF=-1000.7291284\MP2=-1003.4017174\RMSD=7.823e-09\Therma
l=0.\PG=C01 [X(C17H28P1)]\\@

323

1\1\GINC-AZAZEL\SP\RMP2-FC\6-31+G(2d,p)\C20H29P1\CHRISTOPH\17-Feb-2010
\0\\#p MP2(FC)/6-31+g(2d,p) scf=tight\\ps21sp_1\\0,1\P\C,1,1.867564\C,
1,4.3130879,2,92.04976839\C,3,1.55901773,1,97.7252281,2,76.26185125,0\
C,4,1.54892839,3,109.23166185,1,39.17233359,0\C,5,1.54296837,4,110.378
64099,3,-58.7704879,0\C,6,1.55307503,5,110.59319831,4,59.93242797,0\C,
3,1.54707388,1,21.71827205,2,-49.32784682,0\H,4,1.10363889,3,108.47830
804,1,-79.44512738,0\H,5,1.10192153,4,109.0085459,3,-178.47109903,0\H,
6,1.10046562,5,109.93026912,4,-178.62313811,0\H,6,1.10022506,5,109.282
53709,4,-61.58782673,0\H,8,1.10074219,3,108.73140144,1,121.9593782,0\H
,8,1.10073912,3,108.73051269,1,-121.95707381,0\C,5,1.54887029,4,108.65
061201,3,62.72441688,0\H,15,1.10123682,5,109.16275836,4,178.91277039,0
\C,3,1.55901701,1,97.72444672,2,-174.917493,0\H,17,1.10363894,3,108.47
813805,1,79.44458912,0\C,7,1.55307456,6,107.91733056,5,58.74673159,0\H
,19,1.10022777,7,110.22959672,6,-179.70763442,0\H,19,1.10046299,7,109.
82727484,6,62.75730161,0\C,19,1.54296906,7,110.59335444,6,-58.74759226
,0\H,22,1.10192066,19,109.00881329,7,-179.6323594,0\C,4,1.54983661,3,1
09.63004818,1,161.7168916,0\C,15,1.54079541,5,110.39241822,4,58.363690
86,0\C,17,1.54983626,3,109.6301808,1,-161.71761874,0\C,25,1.54126985,1
5,109.87720838,5,-59.72006042,0\H,24,1.10172063,4,108.91680611,3,178.9
8379923,0\H,25,1.10076105,15,109.97132637,5,61.54592416,0\H,25,1.10076
1,15,109.97145399,5,179.01363005,0\H,26,1.10172136,17,108.91692975,3,-
178.98366048,0\H,27,1.1013541,25,109.6407957,15,180.,0\C,3,1.54510581,
1,132.55636674,2,-49.32843253,0\C,24,1.54305621,4,110.2285378,3,58.793
90861,0\H,34,1.10052823,24,110.01597903,4,178.83081209,0\H,34,1.100637
64,24,109.97800081,4,61.2736318,0\C,26,1.54305515,17,110.22861748,3,-5
8.79362623,0\C,33,1.54374014,3,110.64918922,1,-179.99935539,0\H,33,1.1
0168072,3,109.57768538,1,-58.44570149,0\H,33,1.10168117,3,109.57776903
,1,58.44689034,0\H,37,1.10052817,26,110.01604351,17,-178.8309411,0\H,3
7,1.10063819,26,109.97788242,17,-61.27402985,0\H,38,1.09958581,33,109.
56400922,3,-180.,0\C,1,1.86756366,2,98.52394858,7,-105.53581776,0\H,44
,1.09723499,1,108.07327977,2,-69.30786897,0\H,44,1.09753403,1,113.8736

5727,2,50.98002135,0\H,44,1.09606514,1,110.31391893,2,173.69383046,0\H
,2,1.0960625,1,110.31452249,44,-173.71010134,0\H,2,1.09753615,1,113.87
434597,44,-50.99518448,0\H,2,1.09723511,1,108.0723929,44,69.2918783,0\
\Version=AM64L-G03RevD.01\State=1-A\HF=-1115.1039565\MP2=-1118.2242582
\RMSD=3.168e-09\Thermal=0.\PG=C01 [X(C20H29P1)]\\@

323-Me⁺

1\1\GINC-GOLEM\SP\RMP2-FC\6-31+G(2d,p)\C21H32P1(1+)\CHRISTOPH\18-Feb-2
010\0\\#p MP2(FC)/6-31+g(2d,p) scf=tight\\ps21mesp_2\\1,1\C\C,1,4.9027
691\C,2,1.55860959,1,89.65143897\C,3,1.54802822,2,109.36513132,1,60.53
358457,0\C,4,1.54645491,3,110.38746979,2,-59.6724068,0\C,5,1.55573467,
4,109.11922804,3,60.58333839,0\C,2,1.54859715,1,20.22292954,6,83.57858
427,0\H,3,1.10326034,2,108.67648566,1,-58.48867817,0\H,4,1.0997051,3,1
09.31674729,2,-178.21076451,0\H,5,1.09982229,4,109.52070384,3,-178.113
25987,0\H,5,1.09937956,4,109.28933011,3,-60.42507425,0\H,7,1.10087845,
2,108.93051554,1,56.40321001,0\H,7,1.10030383,2,108.7740219,1,173.2697
132,0\C,4,1.54869271,3,109.04078388,2,62.25357443,0\H,14,1.10117833,4,
109.41983082,3,178.99671186,0\C,2,1.55876998,1,119.90326935,6,19.91689
28,0\H,16,1.10326017,2,108.65905112,1,79.79000931,0\C,6,1.55581832,5,1
09.19368988,4,59.53064379,0\H,18,1.09985227,6,110.66252142,5,179.64950
714,0\H,18,1.09933549,6,110.59178849,5,60.46219774,0\C,18,1.54627981,6
,109.13217323,5,-59.79976928,0\H,21,1.09970729,18,107.90312722,6,-179.
73199511,0\C,3,1.55089378,2,109.30046908,1,-177.22374019,0\C,14,1.5411
8528,4,110.10157961,3,58.35395199,0\C,16,1.55084191,2,109.31266121,1,-
161.47876743,0\C,24,1.54071488,14,109.93109866,4,-59.55717765,0\H,23,1
.10056023,3,108.73385439,2,178.92648202,0\H,24,1.09947815,14,109.78800
291,4,61.85372769,0\H,24,1.09946975,14,109.78242618,4,179.03375154,0\H
,25,1.10057196,16,108.73582118,2,-178.95127902,0\H,26,1.09948867,24,10
9.41889179,14,-179.98657803,0\C,2,1.54450888,1,117.32721119,6,157.3848
4022,0\C,23,1.5427137,3,110.10288795,2,58.84612538,0\H,33,1.09901326,2
3,109.8087635,3,178.74637555,0\H,33,1.09999061,23,110.06901875,3,61.40
836753,0\C,25,1.54267803,16,110.08703501,2,-58.87395687,0\C,33,1.54359
019,23,109.68843389,3,-60.1131771,0\H,32,1.10119829,2,109.69582482,1,-
79.20597403,0\H,32,1.10119333,2,109.6964402,1,38.03754086,0\H,36,1.099
01074,25,109.8142987,16,-178.74573012,0\H,36,1.0999968,25,110.06527873
,16,-61.40744275,0\H,37,1.09792247,33,109.81802918,23,179.85017881,0\C
,1,2.93926375,6,61.1283034,5,-94.77092886,0\H,43,1.09495723,1,92.56150
758,6,88.86242703,0\H,43,1.0946855,1,146.40916378,6,-42.25213899,0\H,4
3,1.09593862,1,86.97938273,6,-162.46756589,0\H,1,1.09484314,43,146.600
94117,6,31.85853497,0\H,1,1.09583537,43,89.86961648,6,157.45347285,0\H
,1,1.09464287,43,89.58856299,6,-93.72851666,0\P,1,1.82295977,43,36.282
39984,6,33.14134063,0\C,50,1.82321298,1,107.31654636,43,115.15220799,0
\H,51,1.09474515,50,110.40653511,1,59.40167281,0\H,51,1.0958893,50,109
.35559348,1,-60.28453265,0\H,51,1.09493889,50,110.43659717,1,-179.9332
7191,0\\Version=AM64L-G03RevD.01\State=1-A\HF=-1154.5623173\MP2=-1157.
8260351\RMSD=5.018e-09\Thermal=0.\PG=C01 [X(C21H32P1)]\\@

324

1\1\GINC-SOLARIS\SP\RMP2-FC\6-31+G(2d,p)\C9H18N1P1\CHRISTOPH\12-Oct-20
09\0\\#p MP2(FC)/6-31+g(2d,p) scf=tight\\pzsp_2_2\\0,1\C\C,1,1.5404741
3\C,2,1.53981822,1,112.87882342\C,3,1.5396801,2,113.12127032,1,-60.883
56643,0\C,4,1.54030106,3,112.90973894,2,60.80031385,0\P,1,1.87338608,2
,115.55449438,3,58.42799717,0\H,1,1.10144603,2,110.28881361,3,-60.8000
1507,0\H,1,1.09840597,2,110.99079345,3,-177.65189189,0\H,2,1.09828219,
1,109.12509011,6,-62.9976903,0\H,2,1.10030796,1,109.15956825,6,-179.61
721861,0\H,3,1.10231512,2,109.26235644,1,61.10147275,0\H,3,1.09913198,
2,109.28332094,1,177.13391259,0\H,4,1.10030724,3,109.46514592,2,-177.4
0329105,0\H,4,1.09834006,3,109.06039666,2,-60.66739161,0\H,5,1.1014410

5,4,110.3100666,3,61.03362612,0\H,5,1.0983976,4,110.95346096,3,177.871
23352,0\C,6,2.72785176,1,128.59778402,2,46.28380246,0\C,17,1.55039204,
6,139.07714862,1,34.99103278,0\C,18,1.55511416,17,104.64837144,6,6.481
36085,0\C,19,1.5414119,18,104.12726955,17,7.1069182,0\H,17,1.09627237,
6,96.77561575,1,-101.6336349,0\H,17,1.10761582,6,85.81987359,1,151.104
0693,0\H,18,1.09576479,17,110.80269388,6,127.60803466,0\H,18,1.0953057
8,17,111.01299612,6,-113.17129265,0\H,19,1.09579104,18,112.84584828,17
,128.58327932,0\H,19,1.09565033,18,110.4051593,17,-110.75927501,0\H,20
,1.09620846,19,112.63787654,18,-150.31938945,0\H,20,1.10831364,19,110.
33018661,18,89.15542635,0\N,20,1.47236218,19,103.13226084,18,-30.97611
432,0\\Version=AM64L-G03RevD.01\State=1-A\HF=-746.506896\MP2=-748.1771
484\RMSD=3.047e-09\Thermal=0.\PG=C01 [X(C9H18N1P1)]\\@

324-Me$^+$

1\1\GINC-NAUTILUS\SP\RMP2-FC\6-31+G(2d,p)\C10H21N1P1(1+)\CHRISTOPH\12-
Oct-2009\0\\#p MP2(FC)/6-31+g(2d,p) scf=tight\\pzmesp_6\\1,1\C\C,1,1.5
4956885\C,2,1.54156559,1,113.27849941\C,3,1.54169206,2,113.7630306,1,-
62.94574727,0\C,4,1.54946146,3,113.22789259,2,62.57347646,0\P,5,1.8212
3842,4,111.15645385,3,-55.53448618,0\H,1,1.09723971,2,111.82492007,3,1
78.66031587,0\H,1,1.09954039,2,110.7217337,3,-62.19908758,0\H,2,1.0963
9452,1,107.61878804,6,177.60819168,0\H,2,1.09823076,1,109.41483797,6,-
66.79217651,0\H,3,1.09632138,2,108.67405007,1,175.89235137,0\H,3,1.100
33596,2,109.59378434,1,60.08986199,0\H,4,1.09836489,3,109.73366811,2,-
60.15704149,0\H,4,1.09640825,3,109.83132566,2,-177.12998391,0\H,5,1.09
692252,4,112.07259556,3,-178.67561468,0\H,5,1.09955053,4,110.55748287,
3,62.25382834,0\C,6,2.75406968,5,135.12607752,4,-58.5394219,0\C,17,1.5
3953828,6,130.21229931,5,-59.32418137,0\C,18,1.53817007,17,103.6262733
,6,45.40920118,0\C,19,1.5363548,18,103.20172163,17,-39.31873097,0\H,17
,1.0972514,6,85.42302947,5,-174.31533405,0\H,17,1.09734338,6,105.30679
572,5,77.87794867,0\H,18,1.09397035,17,111.70631842,6,167.54774032,0\H
,18,1.09680323,17,110.05056395,6,-72.49647771,0\H,19,1.09660716,18,110
.61771605,17,78.16638634,0\H,19,1.09395164,18,113.09425383,17,-160.264
43759,0\H,20,1.09516823,19,112.675265,18,153.81596999,0\H,20,1.0999809
9,19,110.91516395,18,-84.00274678,0\C,6,1.82504398,5,109.50477683,4,16
4.75856944,0\H,29,1.09610332,6,110.05135102,5,-177.67486944,0\H,29,1.0
9612732,6,109.87376374,5,62.6533354,0\H,29,1.09629526,6,110.55493516,5
,-57.53510002,0\N,20,1.49268289,19,102.79151682,18,34.95938982,0\\Vers
ion=AM64L-G03RevD.01\State=1-A\HF=-785.9715874\MP2=-787.7819036\RMSD=1
.732e-09\Thermal=0.\PG=C01 [X(C10H21N1P1)]\\@

325

1\1\GINC-YANG\SP\RMP2-FC\6-31+G(2d,p)\C26H23O2P1\CHRISTOPH\11-Apr-2010
\0\\#p MP2(FC)/6-31+g(2d,p) scf=tight\\buchsp_12\\0,1\C\C,1,1.40564697
\C,2,1.39790364,1,121.08374849\C,3,1.39649611,2,119.86615678,1,-0.1278
5251,0\C,4,1.396758,3,119.65380119,2,0.28680278,0\C,5,1.40220345,4,121
.09795916,3,0.1049318,0\H,2,1.08699071,1,119.53190278,6,179.49168512,0
\H,3,1.08815855,2,119.79142707,1,-179.99772081,0\H,4,1.08799722,3,120.
38263782,2,-179.96122345,0\H,5,1.08767014,4,120.119111,3,179.75869505,
0\C,6,4.30537075,5,119.06843551,4,179.34908471,0\C,11,1.39465879,6,60.
82345046,5,81.14886035,0\C,12,1.40510773,11,118.98236122,6,-0.25362213
,0\C,13,1.4096066,12,120.93964622,11,0.59058508,0\C,14,1.41051729,13,1
18.54843801,12,-1.12762479,0\C,11,1.39559714,6,60.75215947,5,-98.46605
142,0\H,11,1.08790645,6,179.79954637,5,-175.578261,0\H,12,1.08438526,1
1,119.91868173,6,179.76348365,0\H,16,1.08442854,11,119.86888773,6,-179
.81874817,0\O,15,1.36101898,14,115.25246522,13,-179.13647414,0\O,13,1.
36149044,12,123.71001445,11,-179.32741442,0\C,20,1.41474418,15,118.369
95556,14,-175.77927497,0\H,22,1.0928023,20,105.7808181,15,178.05754715
,0\H,22,1.09956482,20,111.64903648,15,-63.34976508,0\H,22,1.09888888,2

0,111.51035853,15,59.25664848,0\C,21,1.41557809,13,118.36298721,12,-8.
47979424,0\H,26,1.0985442,21,111.34577312,13,-57.19113502,0\H,26,1.099
59424,21,111.65088279,13,65.66018668,0\H,26,1.09261153,21,105.74927029
,13,-175.68972467,0\P,1,1.85701728,2,123.16951805,3,-178.53139529,0\C,
26,4.28804748,21,93.94152182,13,-98.72568857,0\C,31,1.39933111,26,62.5
0801726,21,-55.37669088,0\C,32,1.39877005,31,120.27434514,26,81.710634
74,0\C,33,1.40646363,32,120.59637965,31,0.14835428,0\C,34,1.40692741,3
3,118.52366482,32,-0.76108168,0\C,35,1.39813707,34,120.92579347,33,1.0
0510817,0\H,31,1.08810893,26,110.07041515,21,-169.74711263,0\H,32,1.08
846019,31,120.03161329,26,-98.41292759,0\H,33,1.08704163,32,119.728050
09,31,-179.45692636,0\H,35,1.08866938,34,119.35669871,33,-178.63393437
,0\H,36,1.08804541,35,119.86402794,34,179.77742178,0\C,30,4.67292358,1
,102.66297994,2,13.23078109,0\C,42,1.39774933,30,61.43719955,1,-95.285
95044,0\C,43,1.39949291,42,120.33567985,30,0.91139315,0\C,44,1.4056536
3,43,120.64878775,42,-0.35912152,0\C,45,1.40870895,44,118.38052352,43,
-0.41012527,0\C,46,1.396593,45,120.98344368,44,1.12833284,0\H,42,1.087
91501,30,178.19856069,1,109.00933528,0\H,43,1.08826024,42,120.03978103
,30,-179.25264291,0\H,44,1.08712886,43,119.54016904,42,179.77600716,0\
H,46,1.08909246,45,119.36578704,44,-178.83456217,0\H,47,1.0881011,46,1
19.84270175,45,179.58781124,0\\Version=AM64L-G03RevD.01\State=1-A\HF=-
1488.4857062\MP2=-1492.7084495\RMSD=3.934e-09\Thermal=0.\PG=C01 [X(C26
H23O2P1)]\\@

325-Me$^+$

1\1\GINC-IBLIS\SP\RMP2-FC\6-31+G(2d,p)\C27H26O2P1(1+)\CHRISTOPH\16-Apr
-2010\0\\#p MP2(FC)/6-31+g(2d,p) scf=tight\\buchmesp_28\\1,1\C\C,1,1.4
1148403\C,2,1.39376347,1,120.6449691\C,3,1.39676995,2,119.54476464,1,-
0.10291997,0\C,4,1.39531924,3,119.99629226,2,0.9703312,0\C,5,1.4041559
3,4,121.79985309,3,-0.2498099,0\H,2,1.08603228,1,120.33883905,6,178.64
066048,0\H,3,1.08657473,2,119.7768191,1,-179.9106712,0\H,4,1.08707552,
3,120.22435143,2,-179.50521338,0\H,5,1.08663559,4,119.97215193,3,179.2
3177682,0\C,6,4.29509385,5,113.81294745,4,177.84300612,0\C,11,1.395459
6,6,60.74571492,5,87.03637094,0\C,12,1.40285686,11,119.04936242,6,1.78
20892,0\C,13,1.41235361,12,120.64743112,11,0.82545682,0\C,14,1.4102306
2,13,118.81975216,12,-1.26731067,0\C,11,1.39602254,6,61.00171475,5,-94
.94067361,0\H,11,1.08725225,6,177.97109523,5,-0.2877801,0\H,12,1.08391
422,11,119.83386048,6,-177.63541417,0\H,16,1.084325,11,119.95575792,6,
177.29108711,0\O,15,1.36725265,14,115.5266522,13,-179.44957839,0\O,13,
1.35803918,12,124.27862408,11,-179.31289885,0\C,20,1.42554316,15,118.2
9092094,14,166.52919301,0\H,22,1.09205549,20,105.7210897,15,-172.16823
153,0\H,22,1.09678712,20,110.99361959,15,-53.43173942,0\H,22,1.0982340
7,20,111.62334516,15,69.0329632,0\C,21,1.42230594,13,118.45705467,12,4
.4392221,0\H,26,1.09746449,21,111.28828597,13,58.04367654,0\H,26,1.092
03997,21,105.70709417,13,176.69038162,0\H,26,1.09822757,21,111.2591593
4,13,-64.51620122,0\C,2,5.23271225,1,94.50403948,6,-161.59826601,0\C,3
0,1.39749322,2,79.9137548,1,-62.71056733,0\C,31,1.39735557,30,120.1678
4316,2,25.02671681,0\C,32,1.40668072,31,120.0502655,30,-0.24917623,0\C
,33,1.40957238,32,119.52804541,31,0.20038921,0\C,34,1.39568148,33,120.
00526668,32,-0.08723365,0\H,30,1.08686559,2,149.31527909,1,163.2910881
6,0\H,31,1.0866539,30,120.25462801,2,-154.89708992,0\H,32,1.08641123,3
1,119.00348398,30,179.79867257,0\H,34,1.08738846,33,120.55600987,32,-1
78.60569396,0\H,35,1.08675756,34,119.62021093,33,-179.62273477,0\C,12,
4.05902815,11,100.83985432,6,94.87044598,0\C,41,1.39943227,12,86.55813
316,11,-151.03352727,0\C,42,1.39536536,41,120.1614725,12,68.43804796,0
\C,43,1.40710816,42,119.87513538,41,-0.17795076,0\C,44,1.40622154,43,1
19.79940053,42,0.24086453,0\C,45,1.39615653,44,119.91056366,43,-0.2483
0459,0\H,41,1.08706348,12,110.81579977,11,88.16684726,0\H,42,1.0870151
6,41,120.20760414,12,-112.38204526,0\H,43,1.08596549,42,119.42345349,4

1,179.23866314,0\H,45,1.08572002,44,120.93149012,43,-179.24869343,0\H,
46,1.08657343,45,119.57268886,44,-179.88774405,0\P,44,1.81649675,43,11
9.36039144,42,176.92259102,0\C,52,1.82282762,44,108.92797223,43,-166.7
2499086,0\H,53,1.09527757,52,108.16014984,44,-73.97339363,0\H,53,1.094
08959,52,111.87823971,44,47.92369676,0\H,53,1.09521192,52,108.19135465
,44,168.93446069,0\\Version=AM64L-G03RevD.01\State=1-A\HF=-1527.938377
7\MP2=-1532.3144447\RMSD=7.141e-09\Thermal=0.\PG=C01 [X(C27H26O2P1)]\\
@

326

1\1\GINC-NAUTILUS\SP\RMP2-FC\6-31+G(2d,p)\C24H39P1\CHRISTOPH\13-May-20
10\0\\#p MP2(FC)/6-31+g(2d,p) scf=tight\\belsp_15\\0,1\P\C,1,1.8876148
7\H,2,1.09997024,1,111.45690083\H,2,1.09801857,1,107.36433604,3,-115.6
0535614,0\C,2,1.5422198,1,113.2027388,4,-119.11980621,0\H,5,1.10009484
,2,108.86591914,1,56.26489165,0\H,5,1.09913473,2,110.36204565,1,-59.47
621847,0\C,5,1.53872337,2,112.89132114,1,178.03883109,0\H,8,1.10080022
,5,109.33668385,2,58.08335543,0\H,8,1.10078246,5,109.24241439,2,-57.62
135095,0\C,8,1.53582145,5,112.94451559,2,-179.7110007,0\H,11,1.0981029
3,8,111.07442067,5,60.02471027,0\H,11,1.09723812,8,111.42540966,5,-179
.80994248,0\H,11,1.09815377,8,111.10239642,5,-59.64340112,0\C,1,4.3520
4947,2,88.37812549,5,-152.77777172,0\C,15,1.54383237,1,96.02092258,2,8
1.00362764,0\C,16,1.54393431,15,109.25730754,1,40.8577402,0\C,17,1.546
92355,16,109.61693322,15,-59.6659428,0\C,18,1.56247703,17,111.1614396,
16,59.54864681,0\C,15,1.55148327,1,23.28721769,2,-46.68424317,0\H,15,1
.09997606,1,132.0159121,2,-42.36164069,0\H,16,1.10046799,15,110.129787
23,1,-80.24838121,0\H,16,1.10047388,15,110.16084897,1,161.97915678,0\H
,17,1.09981092,16,109.74430605,15,-179.24254527,0\H,18,1.0993614,17,10
9.85820852,16,-179.068325,0\H,18,1.10050868,17,109.28719679,16,-62.353
13447,0\H,20,1.09887038,15,109.0242824,1,117.86940199,0\H,20,1.0966131
8,15,109.15987588,1,-125.83250617,0\C,17,1.54380083,16,109.63123535,15
,60.23345435,0\H,29,1.10020562,17,110.04973942,16,178.81411342,0\H,29,
1.10034723,17,110.21325096,16,60.97094534,0\C,15,1.54458913,1,98.25680
826,2,-168.30065141,0\H,32,1.10056284,15,110.11000111,1,81.84622561,0\
H,32,1.10036993,15,110.08360997,1,-160.4431886,0\C,19,1.5541494,18,107
.49899506,17,58.66174339,0\H,35,1.09822927,19,110.04973966,18,-178.298
21609,0\H,35,1.10039042,19,109.97008166,18,63.92198098,0\C,29,1.543345
97,17,109.22875952,16,-60.18300576,0\H,38,1.0998422,29,109.71827468,17
,-179.45568191,0\C,1,4.22302854,2,91.14468967,5,82.02715451,0\C,40,1.5
4429407,1,101.8001151,2,74.6449153,0\C,41,1.5436283,40,109.29700426,1,
42.72089664,0\C,42,1.55044444,41,109.90017983,40,-59.99777292,0\C,43,1
.5504295,42,110.72539308,41,59.82937174,0\C,40,1.54786438,1,18.1192066
3,2,-41.58001843,0\H,40,1.09986744,1,126.70624746,2,-51.15302732,0\H,4
1,1.10051752,40,110.13482258,1,-78.40822869,0\H,41,1.10038322,40,110.1
5179619,1,163.83868214,0\H,42,1.0999556,41,109.68461226,40,-179.743915
,0\H,43,1.09773853,42,108.69116052,41,-178.98503195,0\H,43,1.10026862,
42,109.7522169,41,-62.21238883,0\H,45,1.0985494,40,108.74728924,1,129.
74633109,0\H,45,1.10062514,40,109.81758335,1,-113.36784097,0\C,42,1.54
513851,41,109.45783873,40,59.9033186,0\H,54,1.10059259,42,110.06950602
,41,179.0139356,0\H,54,1.10037135,42,110.06831579,41,61.34907925,0\C,4
0,1.54356948,1,98.12382786,2,-173.30992874,0\H,57,1.10012127,40,110.13
961358,1,75.84583446,0\H,57,1.10044098,40,110.09793994,1,-166.2956019,
0\C,44,1.55837747,43,108.83421179,42,58.03417089,0\H,60,1.09974004,44,
109.40152388,43,-178.80841449,0\H,60,1.09678069,44,110.68414104,43,63.
93078473,0\C,57,1.5427006,40,109.09924284,1,-45.11866701,0\H,63,1.0997
8956,57,109.70019462,40,179.38145246,0\\Version=AM64L-G03RevD.01\State
=1-A\HF=-1272.3909616\MP2=-1276.1451772\RMSD=5.625e-09\Thermal=0.\PG=C
01 [X(C24H39P1)]\\@

326-Me^{+}

1\1\GINC-NAUTILUS\SP\RMP2-FC\6-31+G(2d,p)\C25H42P1(1+)\CHRISTOPH\25-Ma
y-2010\0\\#p MP2(FC)/6-31+g(2d,p) scf=tight\\belmesp_56\\1,1\C\H,1,1.0
9720584\H,1,1.09840825,2,106.07558942\C,1,1.54618505,2,108.85942355,3,
-119.03225969,0\H,4,1.09719255,1,110.85689973,2,-177.27851358,0\H,4,1.
09845907,1,109.57763562,2,-59.71806499,0\C,4,1.54064044,1,111.23922239
,2,60.92648099,0\H,7,1.09978834,4,109.25921486,1,-56.36780656,0\H,7,1.
09973493,4,109.2645766,1,59.68459186,0\C,7,1.53528306,4,112.33649287,1
,-178.28946791,0\H,10,1.09740155,7,111.30635637,4,-59.86009164,0\H,10,
1.0955631,7,110.76909185,4,-179.68330533,0\H,10,1.09732842,7,111.24798
408,4,60.5275338,0\C,1,5.54910473,4,143.92917265,7,144.6549161,0\C,14,
1.54404878,1,87.55511197,4,-163.45008376,0\C,15,1.54372966,14,109.4050
3459,1,31.26924784,0\C,16,1.55049182,15,109.73426988,14,-60.77150715,0
\C,17,1.56004478,16,109.66924716,15,59.62276244,0\C,14,1.5500408,1,36.
29610887,4,68.62760062,0\H,14,1.09788902,1,144.26938137,4,74.89053236,
0\H,15,1.09820994,14,109.97176535,1,152.07504348,0\H,15,1.09985902,14,
110.33723077,1,-90.31120615,0\H,16,1.09786153,15,109.845939,14,-179.35
470428,0\H,17,1.09604797,16,109.47275876,15,-62.08650325,0\H,17,1.0984
9495,16,109.06570993,15,-179.0871677,0\H,19,1.09969513,14,109.56429283
,1,-125.53687602,0\H,19,1.09693845,14,108.85500625,1,117.18771186,0\C,
16,1.54300389,15,109.88643722,14,59.62493696,0\H,28,1.09980166,16,110.
35611927,15,178.7157671,0\H,28,1.09828267,16,110.06149988,15,61.039333
38,0\C,14,1.5428681,1,91.61661274,4,-53.67565129,0\H,31,1.09974622,14,
110.3757198,1,93.17333394,0\H,31,1.09814209,14,109.99411203,1,-149.131
59278,0\C,18,1.56315135,17,108.57171603,16,59.54536924,0\H,34,1.097769
88,18,110.45873811,17,-178.82712469,0\H,34,1.09990102,18,110.8711489,1
7,62.1784404,0\C,28,1.54288593,16,109.2135534,15,-59.69517867,0\H,37,1
.09791946,28,109.96844112,16,-179.01781055,0\C,19,4.83247124,14,148.36
273761,1,138.72742308,0\C,39,1.54260146,19,134.0323138,14,-164.6245967
7,0\C,40,1.5430906,39,109.21292596,19,45.9690902,0\C,41,1.55055766,40,
109.81453935,39,-60.19541534,0\C,42,1.56195941,41,109.9696071,40,59.86
466504,0\C,39,1.55004323,19,27.58714237,14,163.92113761,0\H,39,1.09785
035,19,103.10146827,14,60.02522506,0\H,40,1.09979271,39,110.33579064,1
9,-75.54479783,0\H,40,1.09823124,39,110.11313816,19,166.74875289,0\H,4
1,1.09797038,40,109.97471326,39,-178.84599636,0\H,42,1.09807326,41,108
.89036126,40,-178.95377767,0\H,42,1.09929498,41,109.24952822,40,-61.74
958005,0\H,44,1.09847043,39,108.9942833,19,-143.77047181,0\H,44,1.0962
3947,39,109.42335655,19,-26.62202809,0\C,41,1.54331161,40,109.89045503
,39,59.98789617,0\H,53,1.09978624,41,110.37850634,40,178.48389792,0\H,
53,1.09823163,41,109.98472299,40,60.78820554,0\C,39,1.54403675,19,86.9
4025651,14,-49.69377932,0\H,56,1.09989707,39,110.3480797,19,45.0072430
6,0\H,56,1.09817692,39,109.93615777,19,162.62683645,0\C,43,1.55684229,
42,108.50731592,41,59.58637802,0\H,59,1.097127,43,110.49914618,42,-178
.92981279,0\H,59,1.10000621,43,110.53968014,42,62.30503301,0\C,53,1.54
268843,41,109.22833089,40,-59.98625472,0\H,62,1.09787264,53,109.900235
38,41,-179.3442823,0\P,1,1.8511875,4,117.02792126,7,-178.4641608,0\C,6
4,1.83184509,1,107.60572653,4,-26.83044097,0\H,65,1.09347611,64,110.23
349254,1,-47.56308191,0\H,65,1.09431943,64,110.34968942,1,-167.8593669
2,0\H,65,1.09426316,64,110.01945235,1,72.07857175,0\\Version=AM64L-G03
RevD.01\State=1-A\HF=-1311.8570619\MP2=-1315.7573997\RMSD=3.620e-09\Th
ermal=0.\PG=C01 [X(C25H42P1)]\\@

327

1\1\GINC-GOLEM\SP\RMP2-FC\6-31+G(2d,p)\C29H41P1\CHRISTOPH\17-Feb-2010\
0\\#p MP2(FC)/6-31+g(2d,p) scf=tight\\ps12sp_2\\0,1\C\C,1,1.55173711\C
,2,1.5478828,1,110.68106854\C,3,1.55092989,2,110.71580501,1,-60.775501
39,0\C,4,1.55088642,3,108.02154864,2,59.01846332,0\C,5,1.54321883,4,11
0.74938875,3,-58.43283066,0\H,2,1.09905378,1,110.33948185,6,-62.362770

65,0\H,2,1.09745784,1,110.71651601,6,179.5533804,0\H,3,1.10202163,2,10
8.72507584,1,179.40624862,0\H,4,1.10166547,3,109.05739337,2,-59.390732
84,0\H,5,1.10184793,4,109.13342597,3,-178.27084741,0\H,6,1.10055353,5,
109.26214167,4,-62.1469371,0\H,6,1.09943737,5,109.77689583,4,-178.7957
8783,0\C,3,1.55117932,2,110.72286401,1,59.64200313,0\H,14,1.10184961,3
,109.06748509,2,59.34305819,0\C,1,1.5515786,2,108.45289097,3,-57.64329
78,0\H,16,1.10074076,1,110.0667671,2,179.99212639,0\H,16,1.09851501,1,
110.19219466,2,-62.16656051,0\C,5,1.55029343,4,108.68527645,3,62.83115
58,0\H,19,1.10145888,5,109.02944837,4,178.8664317,0\C,16,1.54381115,1,
110.97045205,2,58.18305184,0\H,21,1.10186077,16,108.78600268,1,-179.79
675342,0\C,4,1.54381021,3,110.63835644,2,-179.8776374,0\H,23,1.1006195
8,4,109.89978081,3,178.95427502,0\H,23,1.10062947,4,109.94330508,3,61.
50557133,0\C,19,1.54296824,5,110.62424222,4,58.34136986,0\C,14,1.54310
625,3,110.59194593,2,179.82238092,0\C,23,1.54393044,4,109.78163222,3,-
59.79407372,0\H,27,1.10069587,14,109.92493531,3,-178.83126477,0\H,27,1
.10066031,14,109.97654278,3,-61.37888708,0\H,26,1.10059851,19,109.9271
9637,5,61.48869043,0\H,26,1.10059005,19,109.95553136,5,178.96706837,0\
H,28,1.09943995,23,109.74695423,4,179.91468714,0\C,1,2.91307278,16,146
.90593335,21,168.42623105,0\H,34,1.09506174,1,148.53130054,16,49.68941
23,0\H,34,1.0964023,1,83.00889571,16,161.03294652,0\H,34,1.09742055,1,
95.61194303,16,-91.20177018,0\P,34,1.87158708,1,40.3536651,16,30.72958
725,0\C,38,4.33491741,34,94.32948302,1,-100.36517149,0\C,39,1.55209418
,38,98.58907066,34,-170.90322186,0\C,40,1.55182945,39,108.2435182,38,3
8.96769643,0\C,41,1.54333481,40,110.70878738,39,-58.80392282,0\C,42,1.
55611747,41,111.01508648,40,58.95652263,0\C,39,1.54679044,38,22.990722
91,34,66.11478469,0\H,39,1.10196681,38,131.8161018,34,63.81064916,0\H,
40,1.10187461,39,109.02769789,38,-79.58945461,0\H,41,1.10184725,40,109
.06595645,39,-178.52728721,0\H,42,1.10000039,41,109.85502712,40,-179.8
4658799,0\H,42,1.09716318,41,109.10158491,40,-63.2535705,0\H,44,1.0985
0344,39,108.74379696,38,123.15123738,0\H,44,1.10020592,39,109.59385799
,38,-120.17471654,0\C,41,1.54959052,40,108.62022713,39,62.65512606,0\H
,52,1.10141609,41,109.0321956,40,178.9676069,0\C,39,1.5508279,38,97.91
974926,34,-60.71273425,0\H,54,1.10174519,39,109.091122,38,79.01292691,
0\C,43,1.55873814,42,107.02677649,41,59.08059525,0\H,56,1.0985233,43,1
10.24991985,42,-178.67561849,0\H,56,1.10100193,43,109.86657842,42,63.5
5418509,0\C,56,1.54422786,43,111.10635081,42,-58.26731027,0\H,59,1.101
95765,56,108.70047278,43,-179.94476002,0\C,52,1.54302141,41,110.665055
25,40,58.41304385,0\C,40,1.54318274,39,110.48485747,38,160.08242264,0\
C,54,1.54312979,39,110.50639052,38,-160.56919562,0\H,63,1.10061749,54,
109.9196087,39,-178.78780418,0\H,63,1.10063718,54,109.92123137,39,-61.
3549495,0\C,61,1.54414021,52,109.7730713,41,-59.71902193,0\H,62,1.1006
2775,40,109.93164624,39,61.35469357,0\H,62,1.10056557,40,109.95206327,
39,178.84717199,0\H,61,1.10058401,52,109.88499788,41,61.51281393,0\H,6
1,1.10066634,52,109.96602209,41,178.98732391,0\H,66,1.09943634,61,109.
76035437,52,179.90983634,0\\Version=AM64L-G03RevD.01\State=1-A\HF=-146
2.9434883\MP2=-1467.4068985\RMSD=7.467e-09\Thermal=0.\PG=C01 [X(C29H41P1)]\\@

327-Me$^+$

1\1\GINC-CALYPSO\SP\RMP2-FC\6-31+G(2d,p)\C30H44P1(1+)\CHRISTOPH\18-Feb
-2010\0\\#p MP2(FC)/6-31+g(2d,p) scf=tight\\ps12mesp_3\\1,1\C\C,1,1.56
063832\C,2,1.54654697,1,109.82733087\C,3,1.54956892,2,110.97130071,1,-
59.87898884,0\C,4,1.55003595,3,107.95680164,2,59.17570751,0\C,5,1.5463
3025,4,110.5163999,3,-59.76597416,0\H,2,1.10006062,1,110.93676106,6,-6
2.00569334,0\H,2,1.09850369,1,110.48998004,6,178.79780368,0\H,3,1.1000
4918,2,107.77681152,1,-179.65784728,0\H,4,1.10170882,3,109.29955373,2,
-59.55536375,0\H,5,1.10003811,4,109.34190503,3,-178.36264916,0\H,6,1.0
9940826,5,109.10011677,4,-59.67442763,0\H,6,1.09710468,5,109.45179589,
4,-176.88566186,0\C,3,1.55052645,2,110.26375617,1,61.04925124,0\H,14,1

.10163174,3,109.22670266,2,59.17924153,0\C,1,1.55351691,6,109.39212214
,5,58.40704729,0\H,16,1.09775293,1,110.56221091,6,61.15547529,0\H,16,1
.10047837,1,110.52004734,6,179.97664666,0\C,5,1.55076934,4,108.8975131
7,3,62.2737997,0\H,19,1.1016527,5,109.23966943,4,178.94923487,0\C,16,1
.5466798,1,109.69156835,6,-58.9793921,0\H,21,1.10003085,16,107.8815039
7,1,179.81427267,0\C,4,1.54314854,3,110.36227296,2,179.96297483,0\H,23
,1.09958458,4,109.74850254,3,178.92739094,0\H,23,1.09961112,4,109.8003
9387,3,61.72138133,0\C,19,1.54350003,5,110.34207427,4,58.50394892,0\C,
14,1.54318787,3,110.39800008,2,179.62919881,0\C,23,1.54387304,4,109.81
134341,3,-59.66843766,0\H,27,1.09964647,14,109.7418032,3,-178.97359172
,0\H,27,1.09963911,14,109.74375588,3,-61.80234952,0\H,26,1.09962525,19
,109.73418437,5,61.63771856,0\H,26,1.09964335,19,109.77638594,5,178.85
337831,0\H,28,1.09799524,23,109.6880114,4,-179.9079456,0\C,1,2.9936556
3,16,146.44707565,21,-169.98502423,0\H,34,1.09454987,1,144.43125946,16
,-45.8285238,0\H,34,1.09483134,1,98.48037092,16,94.70577639,0\H,34,1.0
9523756,1,82.46321398,16,-157.34056639,0\C,34,2.91727939,1,61.28515318
,16,-57.33567666,0\H,38,1.09465055,34,83.95589664,1,-97.03705626,0\H,3
8,1.09518161,34,94.80451244,1,154.79217742,0\H,38,1.09474062,34,146.90
972767,1,18.70961354,0\P,34,1.83143265,1,36.83103108,16,-18.89923184,0
\C,42,4.20361645,34,92.80761187,1,143.82117402,0\C,43,1.54985198,42,99
.92963607,34,164.53534206,0\C,44,1.54981577,43,107.96321254,42,42.2642
0975,0\C,45,1.54608339,44,110.49816896,43,-59.7752034,0\C,46,1.5566482
3,45,109.70980966,44,61.28425138,0\C,43,1.54668221,42,19.66531288,34,3
8.35462462,0\H,43,1.10007669,42,127.4304863,34,40.56898726,0\H,44,1.10
170934,43,109.28227793,42,-76.47129342,0\H,45,1.10009269,44,109.332578
1,43,-178.38456116,0\H,46,1.09715514,45,109.40475404,44,-176.895277,0\
H,46,1.0990212,45,109.02305417,44,-59.76035858,0\H,48,1.10020015,43,10
9.27399198,42,120.43744241,0\H,48,1.09847806,43,108.74935592,42,-122.4
4530826,0\C,45,1.55093971,44,108.89967833,43,62.25500464,0\H,56,1.1017
0268,45,109.23059413,44,178.95753746,0\C,43,1.55009565,42,100.43052223
,34,-83.81905622,0\H,58,1.1016631,43,109.24266714,42,76.84723795,0\C,4
7,1.55431001,46,109.27463632,45,58.33221361,0\H,60,1.10037459,47,110.5
2708467,46,179.91704716,0\H,60,1.09781646,47,110.47053251,46,61.156641
12,0\C,60,1.5464933,47,109.7693165,46,-58.95821835,0\H,63,1.10005003,6
0,107.88771082,47,179.87602842,0\C,56,1.54342006,45,110.32943077,44,58
.52709539,0\C,44,1.54306853,43,110.3935059,42,163.04504568,0\C,58,1.54
326281,43,110.4112617,42,-162.68769499,0\H,67,1.09963364,58,109.757842
05,43,-178.95503941,0\H,67,1.09962882,58,109.73504594,43,-61.7799263,0
\C,67,1.54387009,58,109.79260137,43,59.6037046,0\H,66,1.09961191,44,10
9.78515733,43,61.72685644,0\H,66,1.09964311,44,109.77160477,43,178.935
17858,0\H,65,1.09965715,56,109.74133011,45,61.65844806,0\H,65,1.099644
85,56,109.7768151,45,178.8682283,0\H,70,1.09797187,67,109.69559105,58,
179.95536336,0\\Version=AM64L-G03RevD.01\State=1-A\HF=-1502.4105778\MP
2=-1507.0186822\RMSD=5.162e-09\Thermal=0.\PG=C01 [X(C30H44P1)]\\@

328

1\1\GINC-CIPCLU10\SP\RMP2-FC\6-31+G(2d,p)\C15H19N1P2\C2175\18-Jun-2010
\0\\#p MP2(FC)/6-31+G(2d,p) scf=tight\\con1sp_1\\0,1\C\H,1,1.09646052\
H,1,1.09559446,2,107.95597393\H,1,1.09690436,3,109.44527194,2,118.6246
7094,0\C,1,2.89040232,3,147.18628674,2,-121.11741764,0\H,5,1.09659633,
1,86.08488414,3,-129.29255324,0\H,5,1.09567185,1,146.95008725,3,-9.041
59495,0\H,5,1.09586217,1,93.00322526,3,121.57412227,0\C,1,2.90583336,5
,60.37328839,7,27.66067733,0\H,9,1.09517946,1,146.58368287,5,-34.22748
071,0\H,9,1.0962665,1,87.66077357,5,88.56786595,0\H,9,1.09506666,1,91.
51598224,5,-162.10418729,0\P,5,3.60614557,1,70.81154618,9,94.8198353,0
\C,13,4.67117172,5,127.11519231,1,-136.01918615,0\C,14,1.39996896,13,5
9.22379428,5,-132.54129834,0\C,15,1.39651202,14,120.07799932,13,-0.663
42644,0\C,16,1.40612035,15,120.73432581,14,-0.66913228,0\C,17,1.402495

51,16,118.79473929,15,0.86916779,0\C,14,1.3978995,13,60.3606889,5,48.2
7741421,0\H,14,1.08814792,13,178.89780572,5,-76.30738667,0\H,15,1.0883
8224,14,120.08258145,13,179.06910977,0\H,16,1.08974834,15,119.46686065
,14,179.69485694,0\H,18,1.08735644,17,118.65156066,16,179.7551819,0\H,
19,1.08881961,14,119.93053895,13,-179.16995811,0\C,13,4.67976375,5,128
.08478014,1,16.32751473,0\C,25,1.40117996,13,60.49261565,5,-80.5650329
9,0\C,26,1.39662961,25,120.17516919,13,-0.40926149,0\C,27,1.40572666,2
6,120.57616537,25,0.16066512,0\C,28,1.40357698,27,118.76415487,26,0.40
991657,0\C,25,1.39727244,13,59.27115291,5,99.54754716,0\H,25,1.0881795
8,13,179.31972347,5,70.02825391,0\H,26,1.08874938,25,119.87551045,13,1
79.99303475,0\H,27,1.08823731,26,120.57988143,25,179.94918538,0\H,29,1
.08966843,28,119.46742682,27,178.68403823,0\H,30,1.08841362,25,120.105
01567,13,-179.41367359,0\P,9,1.82413086,1,37.65083631,5,-41.84167026,0
\N,36,1.58087646,9,108.86881152,1,-124.49718048,0\\Version=AM64L-G03Re
vD.01\State=1-A\HF=-1315.0695573\MP2=-1317.6623995\RMSD=4.763e-09\Ther
mal=0.\PG=C01 [X(C15H19N1P2)]\\@

328-Me⁺

1\1\GINC-NAUTILUS\SP\RMP2-FC\6-31+G(2d,p)\C16H22N1P2(1+)\CHRISTOPH\21-
Jun-2010\0\\#p MP2(FC)/6-31+g(2d,p) scf=tight\\con1mesp_17\\1,1\C\H,1,
1.09570939\H,1,1.09551033,2,108.85203397\H,1,1.0963152,3,109.02489292,
2,118.75995486,0\C,1,2.91316644,3,146.40803549,2,-124.01762223,0\H,5,1
.09606824,1,90.21515091,3,129.52247695,0\H,5,1.09633946,1,88.97672414,
3,-121.6611066,0\H,5,1.09555771,1,146.81197766,3,3.00854962,0\C,1,2.92
502853,5,60.23108543,8,35.63359213,0\H,9,1.09514462,1,146.11078292,5,-
34.08129321,0\H,9,1.09575424,1,88.5438559,5,89.57376285,0\H,9,1.095095
01,1,91.190909,5,-161.09889688,0\C,1,5.10117362,5,85.12836572,9,-148.9
1864368,0\C,13,1.39921028,1,79.03888109,5,7.67592325,0\C,14,1.39708817
,13,120.09326866,1,47.88562592,0\C,15,1.40908498,14,120.16115247,13,-0
.04932669,0\C,16,1.40821473,15,119.37413208,14,0.01179507,0\C,17,1.397
35208,16,120.1583221,15,0.10716057,0\H,13,1.08697817,1,132.03723393,5,
-113.02483452,0\H,14,1.08696339,13,120.14365947,1,-132.76765914,0\H,15
,1.08775723,14,119.66679139,13,178.63954485,0\H,17,1.08732812,16,120.7
0809097,15,179.71060065,0\H,18,1.08689703,17,119.7221532,16,179.371477
34,0\C,15,5.30382641,14,142.0630689,13,137.46847988,0\C,24,1.40015362,
15,48.99045009,14,-63.77354256,0\C,25,1.39579448,24,120.07681347,15,-3
5.84844887,0\C,26,1.40842607,25,119.8749671,24,0.12052562,0\C,27,1.404
5507,26,119.82540896,25,-0.45127473,0\C,28,1.39767044,27,119.96948763,
26,0.50998887,0\H,24,1.08702611,15,148.96629732,14,16.65715968,0\H,25,
1.08683279,24,120.15502078,15,144.04996135,0\H,26,1.08818113,25,119.53
497033,24,179.35587093,0\H,28,1.08686597,27,119.63501629,26,-178.81951
73,0\H,29,1.08692463,28,119.8184134,27,179.99021578,0\P,27,1.81479863,
26,120.54826061,25,-177.18256134,0\C,35,1.82012104,27,106.64248231,26,
69.57627564,0\H,36,1.09511665,35,110.67876258,27,-57.74678995,0\H,36,1
.09516586,35,107.75041157,27,61.59492453,0\H,36,1.09469327,35,110.8310
1269,27,-179.80144873,0\P,9,1.81556967,1,36.63254348,5,-39.38936261,0\
N,40,1.5976931,9,107.72140309,1,-122.90871,0\\Version=AM64L-G03RevD.01
\State=1-A\HF=-1354.5533053\MP2=-1357.2893409\RMSD=7.842e-09\Thermal=0
.\PG=C01 [X(C16H22N1P2)]\\@

329

1\1\GINC-NAUTILUS\SP\RMP2-FC\6-31+G(2d,p)\C9H27N3P4\CHRISTOPH\24-Jun-2
010\0\\#p MP2(FC)/6-31+g(2d,p) scf=tight\\con3sp_2\\0,1\C\H,1,1.095224
14\H,1,1.09782666,2,110.3104951\H,1,1.09612554,2,108.59153497,3,-119.8
4617396,0\C,1,2.87641988,2,90.69149394,4,-153.50991811,0\H,5,1.0957843
9,1,147.7116486,2,126.33934066,0\H,5,1.09571552,1,90.74266463,2,-1.687
44584,0\H,5,1.09701381,1,86.75185437,2,-111.32205985,0\C,1,5.00669284,
5,81.93663472,7,-7.50073816,0\H,9,1.09678633,1,147.17307459,5,-177.492

84398,0\H,9,1.09563934,1,50.451148,5,122.39998788,0\H,9,1.09530982,1,1
02.22301899,5,18.54662075,0\C,9,2.90886122,1,100.34625544,5,-74.970717
11,0\H,13,1.0956612,9,145.20549455,1,-24.09642951,0\H,13,1.09702798,9,
88.74712724,1,-148.13823593,0\H,13,1.09579897,9,91.47622846,1,102.4323
8964,0\P,9,1.83208031,1,64.50536397,5,-87.17423766,0\P,1,1.84179066,5,
38.66717156,17,-89.2043843,0\C,18,1.83190573,1,104.3126932,5,-109.0240
7049,0\H,19,1.09679197,18,111.39123836,1,52.0445165,0\H,19,1.09532456,
18,109.29574377,1,172.97186908,0\H,19,1.09560247,18,109.28497301,1,-68
.57158305,0\C,17,1.84180511,9,104.27302715,1,-92.24668304,0\H,23,1.097
88885,17,112.36695682,9,-56.92888502,0\H,23,1.09613294,17,109.30068886
,9,64.64387426,0\H,23,1.09515287,17,106.85923597,9,-178.01052165,0\P,1
7,2.92908024,9,136.7890854,1,47.2484052,0\P,27,2.92952523,17,105.93070
321,9,-160.86791601,0\C,28,1.8321634,27,136.93545517,17,-47.69787286,0
\H,29,1.09564758,28,109.29794855,27,70.48404543,0\H,29,1.09530903,28,1
09.3226867,27,-48.00024775,0\H,29,1.0968799,28,111.37228538,27,-168.94
169196,0\C,28,1.84181844,27,108.77767484,17,90.29002464,0\H,33,1.09785
841,28,112.25417649,27,151.2239118,0\H,33,1.09614377,28,109.31555308,2
7,-87.2603202,0\H,33,1.09505998,28,106.93745681,27,30.10306917,0\C,28,
1.84192639,27,94.29409146,17,-164.69556452,0\H,37,1.09697182,28,111.97
232043,27,-163.09772199,0\H,37,1.09570266,28,107.84876639,27,-42.38744
036,0\H,37,1.09577981,28,109.30890611,27,75.42341729,0\N,18,1.57677131
,1,116.93007857,5,129.44562805,0\N,28,1.57617861,27,29.10189163,17,-21
.70620004,0\N,17,1.57604763,9,109.83934598,1,33.82399825,0\\Version=AM
64L-G03RevD.01\State=1-A\HF=-1882.8909107\MP2=-1885.3768962\RMSD=6.559
e-09\Thermal=0.\PG=C01 [X(C9H27N3P4)]\\@

329-Me$^+$

1\1\GINC-NAUTILUS\SP\RMP2-FC\6-31+G(2d,p)\C10H30N3P4(1+)\CHRISTOPH\24-
Jun-2010\0\\#p MP2(FC)/6-31+g(2d,p) scf=tight\\con3mesp_1\\1,1\C\H,1,1
.09509791\H,1,1.09523419,2,108.6087157\H,1,1.09580531,2,109.22235949,3
,-118.88704924,0\C,1,2.91889529,2,146.26229909,3,123.87202938,0\H,5,1.
09506563,1,145.09777766,2,-4.47838934,0\H,5,1.09550229,1,90.61520825,2
,-129.8638997,0\H,5,1.096218,1,89.77774254,2,120.96331449,0\C,5,6.4088
3983,1,109.10340725,2,-25.13470468,0\H,9,1.09608429,5,131.97381212,1,-
168.92653509,0\H,9,1.09584686,5,99.65405931,1,64.48305538,0\H,9,1.0959
2809,5,96.24066279,1,-46.00431942,0\C,9,2.90253603,5,50.8591516,1,-132
.25218178,0\H,13,1.09546727,9,146.78947352,5,-31.88938388,0\H,13,1.096
29109,9,87.57898137,5,-154.27113209,0\H,13,1.09498137,9,91.01779435,5,
96.20227747,0\P,13,1.82743245,9,37.48372157,5,-23.4191664,0\P,1,1.8189
1024,5,36.92095876,17,-19.12270061,0\C,18,1.82907001,1,106.425774,5,-1
11.66494626,0\H,19,1.09576381,18,109.67686193,1,-63.08092876,0\H,19,1.
09579496,18,108.87684301,1,177.78696802,0\H,19,1.09615642,18,111.12547
019,1,57.45394555,0\C,17,1.8188654,13,106.3537711,9,-112.5683882,0\H,2
3,1.09579937,17,110.83759806,13,55.28764408,0\H,23,1.09513499,17,109.5
235185,13,175.87979329,0\H,23,1.09519438,17,109.50711082,13,-65.120302
27,0\P,13,4.69201025,9,81.71646543,5,66.08339729,0\C,27,1.8290063,13,1
51.83283888,9,-45.42875272,0\H,28,1.09579903,27,109.67032763,13,65.762
30652,0\H,28,1.09615715,27,111.1899572,13,-173.64843995,0\H,28,1.09589
242,27,108.82510973,13,-53.30902463,0\C,27,1.81880135,13,91.98854224,9
,86.28972233,0\H,32,1.09522977,27,109.51581743,13,24.65546979,0\H,32,1
.09576299,27,110.85002654,13,145.08679326,0\H,32,1.0951243,27,109.5181
3069,13,-94.34896098,0\C,27,1.82747339,13,89.32493004,9,-167.37743754,
0\H,36,1.09492118,27,108.38179514,13,89.22082205,0\H,36,1.09633923,27,
111.32558091,13,-150.23050249,0\H,36,1.09547127,27,109.54632718,13,-29
.40103431,0\P,17,2.91996896,13,104.33403046,9,102.9239567,0\C,40,1.829
97238,17,107.07131484,13,-148.77282799,0\H,41,1.095932,40,110.1413537,
17,39.69696552,0\H,41,1.09596612,40,110.14586181,17,159.68531747,0\H,4
1,1.09590954,40,110.16253176,17,-80.30569726,0\N,27,1.59834248,13,36.7

5265982,9,-32.10843151,0\N,18,1.59802684,1,108.18621894,5,123.45476999
,0\N,17,1.59827085,13,114.49559443,9,127.99090136,0\\Version=AM64L-G03
RevD.01\State=1-A\HF=-1922.447605\MP2=-1925.0605554\RMSD=5.301e-09\The
rmal=0.\PG=C01 [X(C10H30N3P4)]\\@

330
see reference 18

330-Me$^+$
see reference 18

331
see reference 18

331-Me$^+$
see reference 18

332
see reference 18

332-Me$^+$
see reference 18

333
see reference 18

333-Me$^+$
see reference 18

334
see reference 18

334-Me$^+$
see reference 18

335
see reference 18

335-Me$^+$
see reference 18

336
see reference 18

336-Me$^+$
see reference 18

337
see reference 18

337-Me$^+$
see reference 18

338
see reference 18

338-Me$^+$
see reference 18

339
see reference 18

339-Me$^+$
see reference 18

340
see reference 18

340-Me$^+$
see reference 18

341
see reference 18

341-Me$^+$
see reference 18

342
see reference 18

342-Me$^+$
see reference 18

343
1\1\GINC-SOLARIS\SP\RMP2-FC\6-31+G(2d,p)\H3P1\CHRISTOPH\11-Jun-2010\0\
\#p MP2(FC)/6-31+g(2d,p) scf=tight\\ph3sp\\0,1\P\H,1,1.42108956\H,1,1.
42108906,2,93.51375689\H,1,1.42108906,3,93.51376833,2,-93.74348663,0\\
Version=AM64L-G03RevD.01\State=1-A\HF=-342.4588836\MP2=-342.5942624\RM
SD=3.077e-09\Thermal=0.\PG=C01 [X(H3P1)]\\@

343-BH$^+$
1\1\GINC-CALYPSO\SP\RMP2-FC\6-31+G(2d,p)\C13H14P1(1+)\CHRISTOPH\09-Dec
-2010\0\\#p MP2(FC)/6-31+g(2d,p) scf=tight\\ph3bhsp_1\\1,1\C\H,1,1.099
2143\P,1,1.8591485,2,103.19106678\C,1,4.33296854,3,109.93846469,2,-113
.83262175,0\C,4,1.39860404,1,59.06510036,3,128.01049041,0\C,5,1.396581
66,4,120.16436219,1,-0.41014139,0\C,6,1.40410342,5,120.35754124,4,0.67
865721,0\C,7,1.40669823,6,119.18666471,5,-0.68435269,0\C,4,1.39776964,
1,60.81712603,7,-4.19764948,0\H,4,1.08662282,1,179.15810886,7,-177.084
79407,0\H,5,1.08665269,4,120.21537995,1,179.82231071,0\H,6,1.08830438,
5,119.66779406,4,179.96592692,0\H,8,1.08936894,7,121.10288408,6,179.10
290551,0\H,9,1.08666537,4,120.24952844,1,-179.74708514,0\C,1,4.3378287
2,7,121.1379022,6,-108.15805224,0\C,15,1.39919164,1,59.36846025,7,154.
66979479,0\C,16,1.39563715,15,119.9041127,1,-0.08391731,0\C,17,1.40711
162,16,120.40393798,15,-0.02081738,0\C,18,1.40328061,17,119.46293699,1
6,-0.14159423,0\C,15,1.39786117,1,60.54649649,7,-24.92907642,0\H,15,1.
0867029,1,179.35151181,7,135.00042054,0\H,16,1.08668302,15,120.3127458
2,1,-179.32193436,0\H,17,1.09012714,16,119.38446701,15,-178.47714018,0
\H,19,1.08581039,18,120.5170253,17,-177.78213965,0\H,20,1.0867487,15,1
20.11716874,1,179.39361239,0\H,3,1.40246372,1,114.83161652,7,-51.23243
27,0\H,3,1.39993833,1,112.34305303,7,-172.77823881,0\H,3,1.39672731,1,
111.54414395,7,69.49371,0\\Version=AM64L-G03RevD.01\State=1-A\HF=-840.
9787904\MP2=-842.9317493\RMSD=6.119e-09\Thermal=0.\PG=C01 [X(C13H14P1)
]\\@

343-TT$^+$
1\1\GINC-BORIX\SP\RMP2-FC\6-31+G(2d,p)\C19H18P1(1+)\CHRISTOPH\23-Apr-2

012\0\\#p MP2(FC)/6-31+g(2d,p) scf=tight\\ph3ttsp_2\\1,1\C\C,1,4.36357
452\C,2,1.39652777,1,59.7751157\C,3,1.39925966,2,120.55502403,1,-0.458
02677,0\C,4,1.40303567,3,120.33858741,2,0.60130573,0\C,5,1.40995084,4,
118.68640273,3,0.60655329,0\C,6,1.3961498,5,120.79566641,4,-1.33664743
,0\H,2,1.08667358,1,179.92338681,5,-87.37972981,0\H,3,1.08683035,2,120
.15504883,1,179.71025638,0\H,4,1.08492016,3,119.41578238,2,-179.766518
02,0\H,6,1.08912013,5,120.69207336,4,175.62625971,0\H,7,1.08682642,6,1
19.63847968,5,-179.90650968,0\C,1,4.36303291,5,113.40659201,4,15.55778
86,0\C,13,1.39651653,1,59.74820658,5,-116.89635605,0\C,14,1.39924212,1
3,120.54233584,1,-0.56602107,0\C,15,1.4029491,14,120.34478702,13,0.567
84501,0\C,16,1.40993043,15,118.70106989,14,0.75468589,0\C,17,1.3962810
1,16,120.77076692,15,-1.52488169,0\H,13,1.08669318,1,179.9754817,16,-1
54.30032421,0\H,14,1.08685553,13,120.15684835,1,179.56096079,0\H,15,1.
0850009,14,119.43434083,13,-179.90591413,0\H,17,1.08920213,16,120.7078
7115,15,175.37811596,0\H,18,1.08681886,17,119.63685601,16,-179.8580341
6,0\C,1,4.36371705,16,113.4832208,15,16.72627429,0\C,24,1.39640585,1,5
9.81637978,16,-118.36941996,0\C,25,1.39927909,24,120.55288691,1,-0.463
06254,0\C,26,1.40315312,25,120.33673612,24,0.63281357,0\C,27,1.4100808
,26,118.69442063,25,0.60533488,0\C,28,1.39616065,27,120.77729074,26,-1
.38382254,0\H,24,1.08668238,1,179.89630231,16,103.18587242,0\H,25,1.08
683643,24,120.15292254,1,179.6714003,0\H,26,1.08476943,25,119.38661661
,24,-179.91825012,0\H,28,1.0891679,27,120.67733892,26,175.56827184,0\H
,29,1.08679537,28,119.63973847,27,-179.90524074,0\P,1,1.89744511,16,10
4.61759495,15,129.77301866,0\H,35,1.3988826,1,113.48301607,16,-47.9242
2364,0\H,35,1.39884249,1,113.44665758,16,-167.78885523,0\H,35,1.398818
78,1,113.69118513,16,72.18079976,0\\Version=AM64L-G03RevD.01\State=1-A
\HF=-1070.5300041\MP2=-1073.3399726\RMSD=5.869e-09\Thermal=0.\PG=C01 [
X(C19H18P1)]\\@

344

1\1\GINC-SOLARIS\SP\RMP2-FC\6-31+G(2d,p)\H3N1\CHRISTOPH\11-Jun-2010\0\
\#p MP2(FC)/6-31+g(2d,p) scf=tight\\nh3sp\\0,1\N\H,1,1.01940111\H,1,1.
01940126,2,105.86437085\H,1,1.01940126,2,105.86437085,3,112.09838844,0
\\Version=AM64L-G03RevD.01\State=1-A\HF=-56.2029289\MP2=-56.4081914\RM
SD=1.308e-09\Thermal=0.\PG=C03 [C3(N1),X(H3)]\\@

344-BH$^+$

1\1\GINC-NAUTILUS\SP\RMP2-FC\6-31+G(2d,p)\C13H14N1(1+)\CHRISTOPH\16-Ju
n-2010\0\\#p MP2(FC)/6-31+g(2d,p) scf=tight\\nh3bhsp_2\\1,1\C\C,1,1.40
317266\C,2,1.40716924,1,119.29178327\C,3,1.39670735,2,120.24660573,1,-
0.05122202,0\C,4,1.39933894,3,120.06182583,2,0.42993453,0\C,1,1.397430
27,2,120.33992499,3,-0.53266091,0\H,1,1.0884893,6,119.70597277,5,-179.
80467598,0\H,3,1.09050754,2,121.13617372,1,178.74594946,0\H,4,1.086666
55,3,119.73669677,2,-179.947873,0\H,5,1.08666172,4,119.9357965,3,179.7
9915663,0\H,6,1.08654456,1,119.68360617,2,-179.64602715,0\C,2,1.513558
97,1,118.6483626,6,-179.85059181,0\H,12,1.09555678,2,109.2734953,1,13.
98560489,0\C,12,4.32500599,2,121.78808519,1,-113.61593057,0\C,14,1.399
9284,12,59.23341504,2,165.97346223,0\C,15,1.39495243,14,119.77009072,1
2,-0.73102217,0\C,16,1.40851439,15,120.53866697,14,0.66177056,0\C,17,1
.40231989,16,119.3555964,15,-1.43008115,0\C,14,1.39735131,12,60.758408
32,2,-12.88347231,0\H,14,1.08674741,12,179.17071389,2,177.87938403,0\H
,15,1.08664942,14,120.30128325,12,-179.65080225,0\H,16,1.09150608,15,1
19.13933091,14,-177.45259211,0\H,18,1.08567655,17,120.39279957,16,-177
.20502338,0\H,19,1.08668652,14,120.09975638,12,179.85403534,0\H,12,2.1
5870264,2,133.79699191,1,123.43793429,0\H,12,2.14677893,2,94.33011471,
1,148.38456699,0\H,12,2.16496583,2,91.90557385,1,103.10465502,0\N,12,1
.56833627,2,107.42604194,1,124.63454402,0\\Version=AM64L-G03RevD.01\St
ate=1-A\HF=-554.7331353\MP2=-556.748426\RMSD=4.605e-09\Thermal=0.\PG=C

01 [X(C13H14N1)]\\@

345
see reference 4

345-BH$^+$

1\1\GINC-YANG\SP\RMP2-FC\6-31+G(2d,p)\C20H22N1O1(1+)\CHRISTOPH\10-Jan-
2011\0\\#p MP2(FC)/6-31+g(2d,p) scf=tight\\yin5bhsp_1\\1,1\C\H,1,1.094
36888\C,1,4.34472282,2,103.50857695\C,3,1.39706669,1,58.45845854,2,3.4
0187976,0\C,4,1.39658787,3,119.94301759,1,0.69039714,0\C,5,1.40813609,
4,121.21542538,3,-0.294669,0\C,6,1.40830787,5,118.13812568,4,-0.413329
35,0\C,7,1.39724235,6,120.63685797,5,0.90971128,0\H,3,1.08671551,1,178
.57851545,6,172.58923962,0\H,4,1.0867694,3,120.34153003,1,-179.0443775
8,0\H,5,1.08897209,4,119.07235722,3,-179.34998438,0\H,7,1.08477528,6,1
21.37123072,5,-178.02468487,0\H,8,1.0868083,7,119.40289049,6,-179.9983
4133,0\C,1,4.34470897,6,118.38507046,5,118.09967469,0\C,14,1.39706815,
1,58.45902314,6,-120.63272222,0\C,15,1.39658688,14,119.94301575,1,-0.6
9057968,0\C,16,1.40813595,15,121.21499629,14,0.29442291,0\C,17,1.40830
704,16,118.13867563,15,0.41327644,0\C,18,1.39724358,17,120.636685,16,-
0.90961048,0\H,14,1.08671483,1,178.57849076,17,-172.57181963,0\H,15,1.
0867693,14,120.34121858,1,179.04385304,0\H,16,1.08897228,15,119.072799
63,14,179.35027927,0\H,18,1.08477397,17,121.37063129,16,178.02462456,0
\H,19,1.08680755,18,119.40321832,17,179.99834657,0\C,1,3.9448926,17,13
4.13718342,16,100.30468653,0\C,25,1.55235913,1,20.21610264,17,-171.835
14956,0\N,26,1.52356438,25,111.22358723,1,-2.44509928,0\C,27,1.5235649
2,26,108.32248338,25,59.10703166,0\C,28,1.55236141,27,111.22353135,26,
-59.09751964,0\H,25,1.0949819,1,119.96055921,17,123.59720879,0\H,25,1.
09557878,1,118.37475552,17,-102.08912978,0\H,26,1.09390364,25,112.2015
3277,1,116.39995729,0\H,26,1.09116562,25,112.24772461,1,-121.75592728,
0\H,28,1.091167,27,106.57694711,26,178.26137083,0\H,28,1.09390354,27,1
06.23576352,26,63.26625067,0\H,29,1.0955792,28,109.92015531,27,121.028
97358,0\H,29,1.09498071,28,109.88025103,27,-121.26670204,0\C,27,1.5073
9155,26,108.30042071,25,-58.14354802,0\H,38,1.09345523,27,108.65536006
,26,-61.87860763,0\H,38,1.09345612,27,108.65540111,26,179.15053429,0\C
,29,1.54620268,28,110.30503402,27,0.7079087,0\H,41,1.09287637,29,111.7
8946866,28,-178.81256007,0\C,41,1.51642784,29,107.13367563,28,-58.0090
4453,0\O,43,1.20485173,41,127.47396293,29,-121.81250253,0\\Version=AM6
4L-G03RevD.01\State=1-A\HF=-899.3454345\MP2=-902.5925909\RMSD=9.312e-0
9\Thermal=0.\PG=C01 [X(C20H22N1O1)]\\@

345-TT$^+$

1\1\GINC-PHOBOS\SP\RMP2-FC\6-31+G(2d,p)\C26H26N1O1(1+)\CHRISTOPH\27-Oc
t-2011\0\\#p MP2(FC)/6-31+g(2d,p) scf=tight\\yin5ttsp_5\\1,1\C\C,1,4.2
6906399\C,2,1.40147698,1,60.16332695\C,3,1.39110791,2,120.07193227,1,-
0.94285644,0\C,4,1.42113664,3,120.50268649,2,1.19996037,0\C,5,1.421459
33,4,118.48788677,3,-0.53447274,0\C,6,1.39013658,5,120.57643106,4,-0.6
9913985,0\H,2,1.08709487,1,179.57244409,5,-179.15322465,0\H,3,1.086444
12,2,120.11116239,1,178.45842133,0\H,4,1.08582173,3,119.87270781,2,177
.76219728,0\H,6,1.0854351,5,119.30161085,4,-177.79680893,0\H,7,1.08661
208,6,119.90152511,5,-178.57113104,0\C,6,5.2022844,5,96.15035194,4,-10
8.56157527,0\C,13,1.5646932,6,24.39676131,5,4.02169842,0\C,14,2.402551
6,13,90.87743727,6,-64.27152101,0\H,13,1.09643197,6,88.69415284,5,160.
98898441,0\H,14,1.09706465,13,111.11853945,6,27.3709025,0\H,15,1.09842
687,14,145.76907582,13,-132.45234018,0\C,1,4.26867425,5,120.32995841,4
,-143.97424652,0\C,19,1.40280645,1,60.15435156,5,-148.7359065,0\C,20,1
.39009662,19,120.02584585,1,-0.28532809,0\C,21,1.42179337,20,120.56929
773,19,1.22191976,0\C,22,1.42145629,21,118.46549162,20,-0.76580531,0\C
,23,1.39096442,22,120.52446943,21,-0.42176647,0\H,19,1.08703918,1,179.

71568941,22,-176.84572397,0\H,20,1.08658302,19,120.05598127,1,179.4161
507,0\H,21,1.08559352,20,120.18434034,19,178.16809905,0\H,23,1.0857894
5,22,119.54683488,21,-176.93372339,0\H,24,1.08642588,23,119.81348563,2
2,-178.30696409,0\C,1,4.26894194,22,120.17117131,21,35.2765646,0\C,30,
1.40098877,1,60.14234371,22,31.49419953,0\C,31,1.39143905,30,120.04146
975,1,-0.97880044,0\C,32,1.42082183,31,120.50279706,30,1.18172101,0\C,
33,1.42090775,32,118.5483632,31,-0.52980162,0\C,34,1.38997773,33,120.5
1160762,32,-0.67106585,0\H,30,1.08708654,1,179.58688018,22,123.1764514
2,0\H,31,1.08643894,30,120.13803318,1,178.45281705,0\H,32,1.08587091,3
1,119.9261172,30,177.80105336,0\H,34,1.08549962,33,119.22160379,32,-17
7.51985368,0\H,35,1.08662762,34,119.91976817,33,-178.55433675,0\H,13,1
.09726756,6,129.53596375,5,49.94878712,0\H,14,1.09712462,13,110.841658
92,6,146.41390284,0\H,15,1.09909694,14,90.10763351,13,105.61232901,0\C
,15,2.40041044,14,60.19851219,13,-92.63968223,0\C,44,1.56517344,15,92.
43692973,14,90.03456366,0\H,44,1.09746162,15,88.97101595,14,-159.38016
855,0\H,44,1.09666297,15,143.18628644,14,-41.43500226,0\H,45,1.0974918
2,44,111.03850802,15,-147.69718555,0\H,45,1.09610396,44,111.0104165,15
,93.40489594,0\N,15,1.47140564,14,35.58580251,13,-128.67164619,0\C,13,
1.55105839,6,109.63307687,5,-87.93036344,0\H,51,1.09395945,13,111.8321
1339,6,-154.04152319,0\C,51,1.52391366,13,107.30068401,6,-33.66540181,
0\O,53,1.20961303,51,126.3558697,13,-123.00054081,0\\Version=AM64L-G03
RevD.01\State=1-A\HF=-1128.8950622\MP2=-1132.9643605\RMSD=7.167e-09\Th
ermal=0.\PG=C01 [X(C26H26N1O1)]\\@

346
see reference 4

346-BH$^+$

1\1\GINC-CIPCLU02\SP\RMP2-FC\6-31+G(2d,p)\C20H21N2(1+)\C2175\14-Jan-20
11\0\\#p MP2(FC)/6-31+G(2d,p) scf=tight\\yin14bhsp_4\\1,1\C\H,1,1.0936
0988\C,1,4.28077667,2,104.002468\C,3,1.40534942,1,58.91972961,2,166.43
335929,0\C,4,1.37462171,3,118.17547247,1,2.90195207,0\C,5,2.38768005,4
,92.6911652,3,2.10871895,0\C,3,1.38344269,1,60.85967886,5,172.0779637,
0\H,3,1.08669145,1,177.12600266,5,-83.07727041,0\H,4,1.08434614,3,122.
09927589,1,-176.10827407,0\H,5,1.08331215,4,122.44887917,3,-176.555646
63,0\H,7,1.08345075,3,120.27109406,1,178.1725102,0\C,1,4.33764155,5,88
.28250112,4,-117.38750213,0\C,12,1.39966569,1,59.32093777,5,97.0291165
4,0\C,13,1.39583006,12,119.97159273,1,0.28387754,0\C,14,1.40497387,13,
120.45993582,12,-0.07346079,0\C,15,1.40317877,14,119.30283092,13,0.469
37775,0\C,12,1.39756378,1,60.56593104,15,16.9972042,0\H,12,1.08693909,
1,179.34708667,15,-159.50435038,0\H,13,1.0869775,12,120.18095586,1,179
.75194677,0\H,14,1.08932377,13,119.68421111,12,179.52367185,0\H,16,1.0
8745464,15,120.13033541,14,178.15047387,0\H,17,1.08697389,12,120.14378
295,1,-179.82243952,0\C,1,4.34215744,15,113.53484081,14,-154.93373694,
0\C,23,1.39511651,1,61.0460166,15,-113.72073541,0\C,24,1.40148539,23,1
20.32338406,1,-0.20554619,0\C,25,1.40173652,24,120.35716298,23,0.03974
74,0\C,26,1.40689914,25,118.95418001,24,-0.26130457,0\C,27,1.39468486,
26,120.55989073,25,0.31030888,0\H,23,1.08675392,1,178.7217962,26,179.3
6446487,0\H,24,1.08698531,23,120.2239487,1,179.87606855,0\H,25,1.08715
18,24,118.55761651,23,-179.28306435,0\H,27,1.08843015,26,119.72531497,
25,179.62775709,0\H,28,1.0869488,27,119.62280481,26,179.95569392,0\N,6
,1.37317585,5,149.40265876,4,-179.9700874,0\C,34,1.46752216,6,117.9184
0943,5,155.14084481,0\H,35,1.09309956,34,110.41609689,6,-42.02294585,0
\H,35,1.10061281,34,112.51067088,6,80.98947491,0\H,35,1.09443866,34,10
7.69984943,6,-159.97986944,0\C,34,1.4774648,6,121.22557672,5,-59.11999
755,0\H,39,1.09514454,34,109.75140787,6,141.69197396,0\H,39,1.0898597,
34,111.44426252,6,20.66183748,0\H,39,1.0990825,34,109.32254496,6,-99.0
3631395,0\N,5,1.36523114,4,122.44984684,3,0.90745876,0\\Version=AM64L-

G03RevD.01\State=1-A\HF=-878.3580898\MP2=-881.5591034\RMSD=4.570e-09\T
hermal=0.\PG=C01 [X(C20H21N2)]\\@

346-TT⁺

1\1\GINC-GOLEM\SP\RMP2-FC\6-31+G(2d,p)\C26H25N2(1+)\CHRISTOPH\07-Nov-2
011\0\\#p MP2(FC)/6-31+g(2d,p) scf=tight\\yin14ttsp_1\\1,1\C\C,1,4.371
29279\C,2,1.39832328,1,58.84848092\C,3,1.39746695,2,120.02941748,1,0.4
6091603,0\C,4,1.40880296,3,121.18383641,2,-0.11789091,0\C,5,1.40180876
,4,118.1463063,3,1.41314522,0\C,2,1.39689618,1,60.44362421,5,17.680597
27,0\H,2,1.08689268,1,178.87980129,5,157.01267763,0\H,3,1.08717692,2,1
20.35404907,1,-179.26167213,0\H,4,1.08833602,3,118.64497447,2,-179.445
35544,0\H,6,1.08356332,5,120.33193952,4,176.46563207,0\H,7,1.08724866,
2,120.10648164,1,179.69296805,0\C,1,4.39081673,5,112.68927155,4,65.765
10218,0\C,13,1.40032298,1,59.19453882,5,178.63279118,0\C,14,1.39336071
,13,120.22856108,1,0.51405013,0\C,15,1.4136741,14,121.18418346,13,1.72
324396,0\C,16,1.40400206,15,117.84842638,14,-2.33825865,0\C,13,1.39376
488,1,60.06738846,5,-1.91194367,0\H,13,1.0868987,1,178.67525658,5,111.
09133598,0\H,14,1.08708499,13,120.2691663,1,-178.19510409,0\H,15,1.087
12075,14,118.76832227,13,-177.55583761,0\H,17,1.08336245,16,120.610047
91,15,-177.24549998,0\H,18,1.08697448,13,120.26648723,1,178.14793079,0
\C,1,4.3693267,5,119.14935273,4,179.81207968,0\C,24,1.39830433,1,58.69
330262,5,-48.43479864,0\C,25,1.39581637,24,120.47319808,1,1.58517432,0
\C,26,1.40787029,25,120.94708197,24,1.81996943,0\C,27,1.40291325,26,11
7.90562187,25,-3.95631308,0\C,24,1.39500043,1,60.44818669,5,130.961377
7,0\H,24,1.08668016,1,178.05100177,5,-111.84983996,0\H,25,1.08703802,2
4,120.21152759,1,-177.2756915,0\H,26,1.08621953,25,119.01257441,24,-17
6.24392496,0\H,28,1.08679667,27,120.86035238,26,-175.21344005,0\H,29,1
.08738537,24,120.26850319,1,177.23174266,0\C,1,4.37958223,5,105.946066
,4,-54.80646696,0\C,35,1.39220827,1,59.55835646,5,-63.25232228,0\C,36,
1.39875898,35,121.85200243,1,1.80831325,0\C,37,2.36563102,36,88.734823
38,35,-0.3609104,0\C,38,1.38555864,37,92.29926518,36,1.14226375,0\H,35
,1.08677805,1,179.00785747,5,-165.67119931,0\H,36,1.08558701,35,120.57
830192,1,-179.70054515,0\H,38,1.08120684,37,146.38079751,36,179.931907
67,0\H,39,1.08504967,38,118.95011959,37,178.63837901,0\N,37,1.41056487
,36,121.64118957,35,175.91940947,0\C,44,1.4812902,37,111.82499453,36,-
70.61961558,0\H,45,1.10297599,44,111.89223929,37,72.66629781,0\H,45,1.
09392893,44,108.00744894,37,-167.6341295,0\H,45,1.09154779,44,110.5344
0498,37,-49.22239109,0\C,44,1.47429604,37,114.71826352,36,55.83782322,
0\H,49,1.10314947,44,113.23466373,37,-68.67996805,0\H,49,1.09094437,44
,110.42344817,37,54.59911226,0\H,49,1.09465453,44,107.44729361,37,172.
17047965,0\N,38,1.35706673,37,30.61554547,36,-176.52188038,0\\Version=
AM64L-G03RevD.01\State=1-A\HF=-1107.8776076\MP2=-1111.9520497\RMSD=3.4
51e-09\Thermal=0.\PG=C01 [X(C26H25N2)]\\@

347

see reference 4

347-BH⁺

1\1\GINC-NAUTILUS\SP\RMP2-FC\6-31+G(2d,p)\C20H23Cl1N1(1+)\CHRISTOPH\10
-Jan-2011\0\\#p MP2(FC)/6-31+g(2d,p) scf=tight\\yin6bhsp_24\\1,1\C\H,1
,1.09452907\C,1,4.34626826,2,103.4169867\C,3,1.39677678,1,58.46146656,
2,2.28661069,0\C,4,1.39696797,3,119.93907934,1,0.9281681,0\C,5,1.40802
307,4,121.23999211,3,-0.2796672,0\C,6,1.40824444,5,118.10205559,4,-0.2
5142531,0\C,7,1.39692113,6,120.66121357,5,0.68724476,0\H,3,1.08672389,
1,178.46159366,6,170.24823842,0\H,4,1.0867625,3,120.36124789,1,-178.79
708541,0\H,5,1.08892956,4,119.06276428,3,-179.26602038,0\H,7,1.0848035
8,6,121.25552999,5,-178.58070036,0\H,8,1.08687445,7,119.41366099,6,-17
9.92369282,0\C,1,4.34602447,6,118.13406443,5,116.07237199,0\C,14,1.396

9556,1,58.46369026,6,-121.41974208,0\C,15,1.39653658,14,119.93791687,1
,-0.59441047,0\C,16,1.40770247,15,121.25060599,14,0.23190484,0\C,17,1.
40784527,16,118.09941719,15,0.65037728,0\C,18,1.3970613,17,120.6667617
7,16,-1.16250395,0\H,14,1.08671271,1,178.63614857,17,-174.53327294,0\H
,15,1.08677244,14,120.35124673,1,179.13583256,0\H,16,1.08896036,15,118
.99812494,14,179.24610372,0\H,18,1.08480517,17,121.38005055,16,177.535
7357,0\H,19,1.08677011,18,119.38953543,17,-179.90744474,0\C,1,3.919707
5,17,134.61182165,16,101.12741569,0\C,25,1.54711906,1,21.18654321,17,-
152.5154397,0\N,26,1.52366833,25,110.22630409,1,7.91169662,0\C,27,1.52
570333,26,108.05297291,25,68.51009165,0\C,28,1.54618153,27,110.2664047
8,26,-48.81924629,0\H,25,1.09670025,1,111.69178576,17,116.41331291,0\H
,25,1.09520771,1,126.06714631,17,-110.85732633,0\H,26,1.09452271,25,11
2.17947274,1,125.7556428,0\H,26,1.09145797,25,112.56831333,1,-111.7392
0404,0\H,28,1.09146794,27,106.39249576,26,-170.73970202,0\H,28,1.09319
407,27,106.93440597,26,73.67088008,0\H,29,1.09598263,28,110.74197781,2
7,103.97345671,0\H,29,1.09344557,28,109.06159176,27,-137.96512051,0\C,
27,1.51287904,26,108.34532118,25,-48.57786113,0\H,38,1.09235952,27,106
.78316846,26,-54.89392568,0\H,38,1.09013437,27,107.66773063,26,-172.36
731172,0\C,38,1.55114093,27,110.74936856,26,65.94873408,0\H,41,1.09242
258,38,109.33311274,27,-133.63644003,0\C,41,1.53636355,38,109.29177271
,27,-12.56495729,0\H,43,1.09375421,41,110.18053976,38,-172.66111508,0\
Cl,41,1.82000674,38,110.95749061,27,111.38644638,0\\Version=AM64L-G03R
evD.01\State=1-A\HF=-1284.5511076\MP2=-1287.7697266\RMSD=4.813e-09\The
rmal=0.\PG=C01 [X(C20H23Cl1N1)]\\@

347-TT$^+$

1\1\GINC-TOFU\SP\RMP2-FC\6-31+G(2d,p)\C26H27Cl1N1(1+)\CHRISTOPH\29-Oct
-2011\0\\#p MP2(FC)/6-31+g(2d,p) scf=tight\\yin6ttsp_6\\1,1\C\C,1,4.26
80541\C,2,1.40189546,1,60.11874944\C,3,1.39054249,2,120.07221144,1,0.8
4748841,0\C,4,1.42214394,3,120.53469171,2,-1.27295919,0\C,5,1.42142079
,4,118.43421311,3,0.45610552,0\C,6,1.39015279,5,120.5984554,4,0.867689
38,0\H,2,1.08708402,1,179.86379206,5,-179.95718311,0\H,3,1.08648799,2,
120.10495067,1,-178.67154424,0\H,4,1.08586621,3,119.82996211,2,-177.78
690609,0\H,6,1.08521021,5,119.3539546,4,177.69198971,0\H,7,1.08653468,
6,119.8469686,5,178.21977774,0\C,1,4.20128411,5,107.54090887,4,-112.95
624361,0\C,1,4.268485,5,119.63754295,4,-30.06317357,0\C,14,1.40275833,
1,60.2103954,5,-37.20151297,0\C,15,1.39022257,14,120.03024785,1,0.2297
8713,0\C,16,1.42079985,15,120.50087573,14,-1.17678894,0\C,17,1.4208350
3,16,118.58082798,15,0.49990509,0\C,18,1.39122827,17,120.47465693,16,0
.72960163,0\H,15,1.08664186,14,120.05430291,1,-179.48442644,0\H,16,1.0
8525902,15,120.24816066,14,-177.95055246,0\H,18,1.08594748,17,119.5635
9464,16,177.34030783,0\H,19,1.08650441,18,119.84831671,17,178.18268146
,0\C,1,4.26737257,5,120.52790848,4,144.75436905,0\C,24,1.40155764,1,60
.21327594,5,-31.51754318,0\C,25,1.39091858,24,120.02706459,1,0.9600978
4,0\C,26,1.42126314,25,120.50691287,24,-1.17745153,0\C,27,1.42175668,2
6,118.54254829,25,0.56588256,0\C,28,1.39010426,27,120.49691973,26,0.63
092104,0\H,24,1.08715574,1,179.55715284,5,-124.91974921,0\H,25,1.08644
181,24,120.11368394,1,-178.44833484,0\H,26,1.08589016,25,119.88312702,
24,-177.68367753,0\H,28,1.08550734,27,119.26800509,26,177.37735447,0\H
,29,1.08663531,28,119.90970653,27,178.50677441,0\C,13,2.40092641,1,71.
10120382,5,1.58576666,0\C,35,1.56182556,13,93.38977433,1,-164.7401656,
0\H,35,1.09566696,13,144.36410111,1,-31.41721751,0\H,35,1.09833323,13,
87.3545976,1,85.97404753,0\H,36,1.09327997,35,111.2872259,13,-95.92426
045,0\Cl,36,1.83321653,35,111.22280035,13,148.75905386,0\C,36,1.539183
2,35,108.26917652,13,25.62535251,0\H,41,1.09500443,36,110.0046573,35,-
176.54024918,0\C,41,1.54922928,36,106.2838704,35,-56.77898536,0\H,43,1
.09810091,41,110.36610545,36,-56.03125829,0\H,43,1.09688885,41,109.481
86213,36,-173.40738946,0\H,13,1.09789601,1,78.38455528,5,99.03352168,0

\H,14,1.08715582,1,179.55978261,5,57.06624003,0\H,13,1.0974146,1,82.79
842816,5,-151.62146454,0\C,35,2.40480582,13,60.18746089,1,-76.7096539,
0\C,41,1.54277722,36,110.42411068,35,60.81751104,0\H,50,1.09470518,41,
110.498973,36,69.27579188,0\H,50,1.09728106,41,109.08891507,36,-172.99
456795,0\H,49,1.09745394,35,142.81469359,13,48.48770378,0\H,49,1.09715
321,35,86.50842245,13,163.41125403,0\N,35,1.47105436,13,35.59330329,1,
-40.89620683,0\\Version=AM64L-G03RevD.01\State=1-A\HF=-1514.0970335\MP
2=-1518.1377566\RMSD=6.560e-09\Thermal=0.\PG=C01 [X(C26H27Cl1N1)]\\@

348
see reference 4

348-BH⁺

1\1\GINC-CIPCLU07\SP\RMP2-FC\6-31+G(2d,p)\C20H21N2(1+)\C2175\26-Jan-20
11\0\\#p MP2(FC)/6-31+G(2d,p) scf=tight\\yin16bhsp_4\\1,1\C\H,1,1.0955
4188\C,1,4.33089732,2,106.39209497\C,3,1.39810108,1,60.53740694,2,148.
98715632,0\C,4,1.39871756,3,120.27175993,1,-0.22782997,0\C,5,1.4041204
3,4,120.13077485,3,0.34505979,0\C,6,1.40636885,5,119.23895419,4,-0.687
61496,0\C,7,1.39598538,6,120.52039609,5,0.56601921,0\H,3,1.08702596,1,
179.28817018,6,-142.73296808,0\H,4,1.08700563,3,120.11696085,1,-179.32
279593,0\H,5,1.08677942,4,119.78550524,3,-178.25695476,0\H,7,1.0892484
8,6,119.83067467,5,-178.98182668,0\H,8,1.08694835,7,119.84973561,6,-17
9.36436487,0\C,1,4.33021518,6,114.65755765,5,29.08096739,0\C,14,1.3983
4909,1,59.1439711,6,91.30930063,0\C,15,1.39703771,14,120.0982837,1,0.9
4875838,0\C,16,1.40298125,15,120.35738469,14,-0.36032053,0\C,17,1.4034
5848,16,119.33421082,15,0.47488723,0\C,18,1.39799621,17,120.1979015,16
,-0.25845918,0\H,14,1.08682282,1,179.08826601,6,55.46800775,0\H,15,1.0
8685517,14,120.21994222,1,-179.42200271,0\H,16,1.0887132,15,119.720416
56,14,-179.83697509,0\H,18,1.0886265,17,120.50693567,16,179.24508091,0
\H,19,1.08703586,18,119.65584569,17,179.78738759,0\C,1,4.28846346,6,11
3.46934972,5,-98.73982863,0\C,25,1.42212178,1,61.77790572,6,17.9933620
9,0\C,26,1.41778124,25,116.26749386,1,-0.52303762,0\C,27,2.36070351,26
,92.63793534,25,0.23166972,0\C,25,1.38855377,1,58.36672531,6,-161.7208
3907,0\H,25,1.08387097,1,177.75907991,6,-164.34036765,0\H,27,1.0806114
,26,123.05016512,25,-179.07347914,0\H,28,1.08318935,27,145.66625016,26
,178.06821755,0\H,29,1.08534575,25,120.41990838,1,-179.83135171,0\N,26
,1.36068933,25,122.86480379,1,179.64714676,0\C,34,1.46231775,26,119.79
720671,25,-0.04375271,0\H,35,1.09852871,34,111.45032249,26,61.61006074
,0\H,35,1.09063444,34,108.87712385,26,-179.25698458,0\H,35,1.09824906,
34,111.34086397,26,-60.29516942,0\C,34,1.46160843,26,120.20794558,25,1
78.90786639,0\H,39,1.09893641,34,111.42027614,26,61.55912075,0\H,39,1.
09056387,34,108.97624111,26,-179.40255761,0\H,39,1.09886179,34,111.433
88498,26,-60.28347666,0\N,27,1.34577688,26,121.60430078,25,0.13888241,
0\\Version=AM64L-G03RevD.01\State=1-A\HF=-878.3588799\MP2=-881.5636491
\RMSD=7.735e-09\Thermal=0.\PG=C01 [X(C20H21N2)]\\@

348-TT⁺

1\1\GINC-GOLEM\SP\RMP2-FC\6-31+G(2d,p)\C26H25N2(1+)\CHRISTOPH\10-Nov-2
011\0\\#p MP2(FC)/6-31+g(2d,p) scf=tight\\yin16ttsp_15\\1,1\C\C,1,4.37
327825\C,2,1.39385729,1,60.93734123\C,3,1.40181758,2,120.5393318,1,-0.
38624256,0\C,4,1.40174348,3,120.6995103,2,-0.05764609,0\C,5,1.40985792
,4,118.20475251,3,1.49908438,0\C,6,1.39422844,5,120.97731581,4,-2.0364
7163,0\H,2,1.08678728,1,178.65874632,5,-174.28916132,0\H,3,1.08708348,
2,120.2492726,1,179.34739255,0\H,4,1.08546567,3,117.87834972,2,178.929
6863,0\H,6,1.08712877,5,119.69801992,4,177.34317514,0\H,7,1.08702662,6
,119.44467487,5,-179.53658589,0\C,1,4.375157,5,114.88146388,4,112.1617
0292,0\C,13,1.39980404,1,59.1322602,5,-168.30848575,0\C,14,1.39456189,
13,120.11540112,1,0.11105685,0\C,15,1.41069527,14,121.04422191,13,-0.4

0370346,0\C,16,1.40196413,15,118.26695033,14,1.32678287,0\C,13,1.39459
679,1,60.24151675,5,11.08953777,0\H,13,1.08685996,1,179.38549937,5,-17
4.54301307,0\H,14,1.08714149,13,120.26258823,1,179.92236123,0\H,15,1.0
8828861,14,118.91504701,13,179.35844965,0\H,17,1.08424411,16,120.55916
848,15,178.18975633,0\H,18,1.08699858,13,120.20718665,1,-179.73358917,
0\C,1,4.37758437,5,106.58278471,4,-120.69999314,0\C,24,1.39996203,1,59
.10345816,5,42.51701289,0\C,25,1.39489281,24,120.13785477,1,-0.3197517
5,0\C,26,1.4127393,25,121.05495754,24,1.26794319,0\C,27,1.4034435,26,1
18.12027847,25,-1.86491495,0\C,24,1.39495389,1,60.28627299,5,-137.0809
8898,0\H,24,1.08702745,1,179.16255411,5,2.36003239,0\H,25,1.08723896,2
4,120.27256578,1,-178.9831761,0\H,26,1.08744537,25,119.16802539,24,-17
7.83641939,0\H,28,1.0838772,27,120.56857174,26,-177.31242541,0\H,29,1.
08704365,24,120.19978096,1,179.00009551,0\C,1,4.33566489,5,107.5421860
3,4,-0.8683681,0\C,35,1.38821396,1,58.04019321,5,70.75083052,0\C,36,1.
38931776,35,120.77633719,1,0.26770032,0\C,37,2.35316792,36,90.35376015
,35,-0.08866829,0\C,38,1.41736743,37,92.70567943,36,-0.01745174,0\H,35
,1.08401358,1,177.55310428,5,77.12442053,0\H,36,1.08542369,35,120.4437
5918,1,179.94790334,0\H,37,1.08146522,36,123.4108597,35,179.14033336,0
\H,38,1.078707,37,145.26196558,36,178.54140573,0\N,39,1.3626809,38,120
.67080367,37,179.80337505,0\C,44,1.46087958,39,120.24544314,38,-0.5993
1731,0\H,45,1.09940041,44,111.65336534,39,-62.53218741,0\H,45,1.098677
58,44,111.34910808,39,59.23997656,0\H,45,1.09067851,44,108.95556375,39
,178.15060583,0\C,44,1.4614738,39,119.72422639,38,-178.32040899,0\H,49
,1.0988001,44,111.54654586,39,61.01681137,0\H,49,1.09071436,44,108.899
2842,39,-179.79302657,0\H,49,1.09832778,44,111.32772496,39,-60.8633379
2,0\N,38,1.34669336,37,29.46274265,36,-179.55668373,0\\Version=AM64L-G
03RevD.01\State=1-A\HF=-1107.8957823\MP2=-1111.9660159\RMSD=6.728e-09\
Thermal=0.\PG=C01 [X(C26H25N2)]\\@

349
see reference 4

349-BH$^+$
1\1\GINC-CIPCLU08\SP\RMP2-FC\6-31+G(2d,p)\C23H27N4(1+)\C2175\21-Jan-20
11\0\\#p MP2(FC)/6-31+G(2d,p) scf=tight\\yin10bhsp_91\\1,1\C\H,1,1.096
70641\C,1,4.33452252,2,107.36641432\C,3,1.3987288,1,59.16604241,2,30.5
0450852,0\C,4,1.3967483,3,120.08734132,1,-0.79710335,0\C,5,1.40320524,
4,120.4659391,3,0.19625566,0\C,6,1.40280903,5,119.20564678,4,-0.328346
07,0\C,3,1.39786691,1,60.58927213,6,-38.92240363,0\H,3,1.08695308,1,17
9.09609339,6,178.5561912,0\H,4,1.08700446,3,120.1951275,1,179.452299,0
\H,5,1.08880604,4,119.7598943,3,179.69709624,0\H,7,1.088309,6,120.3490
4977,5,-179.54954424,0\H,8,1.08726484,3,120.10363453,1,-179.23678891,0
\C,1,4.33414381,6,116.51198286,5,-86.99290216,0\C,14,1.39964004,1,59.3
5647372,6,154.06811735,0\C,15,1.39575449,14,119.9417367,1,-0.22492554,
0\C,16,1.40578638,15,120.52934924,14,0.1333916,0\C,17,1.40301956,16,11
9.22264072,15,-0.46428089,0\C,14,1.39759948,1,60.51625303,6,-25.542632
9,0\H,14,1.08704842,1,179.37194123,6,139.65271983,0\H,15,1.08702148,14
,120.20332084,1,-179.63800047,0\H,16,1.08917362,15,119.6984684,14,-179
.63308935,0\H,18,1.08676109,17,120.04715915,16,-178.3708437,0\H,19,1.0
8702612,14,120.12444217,1,179.64900966,0\C,1,4.34297686,6,108.34804844
,5,143.91105586,0\C,25,1.43008669,1,58.32860836,6,109.63274704,0\C,26,
1.3716018,25,121.35710315,1,0.82371989,0\C,27,2.34859345,26,91.1457871
7,25,0.25160278,0\C,28,1.37090075,27,91.77598052,26,0.05958017,0\H,26,
1.08486816,25,118.92658245,1,-179.61948286,0\H,27,1.08349869,26,122.47
078303,25,179.78407221,0\H,28,1.08554697,27,146.12260694,26,-179.53323
081,0\H,29,1.08525977,28,118.72703774,27,178.25149201,0\C,25,2.3712288
5,1,156.10995258,6,-53.16253059,0\N,34,1.33471603,25,27.68065945,1,-17
7.18609695,0\N,34,1.37103034,25,102.0755546,1,40.04775898,0\N,34,1.356

58487,25,137.07191491,1,-120.69064545,0\C,36,1.46407551,34,123.1151611
1,25,-131.55029358,0\H,38,1.0914378,36,110.22419564,34,7.84049817,0\H,
38,1.09816346,36,108.65346306,34,126.49978645,0\H,38,1.09763857,36,112
.0124734,34,-114.10559097,0\C,36,1.46114731,34,121.31800805,25,41.3790
2072,0\H,42,1.09635628,36,108.62398434,34,140.10868749,0\H,42,1.099319
27,36,111.93005076,34,-100.12349474,0\H,42,1.09177376,36,109.92030906,
34,21.37298196,0\C,37,1.46457073,34,119.21252959,25,-7.73445979,0\H,46
,1.09794053,37,111.03574952,34,-88.06103349,0\H,46,1.09035577,37,110.1
2077518,34,32.48076136,0\H,46,1.09513979,37,108.46427956,34,152.001327
,0\C,37,1.46658142,34,122.95737792,25,-166.9812651,0\H,50,1.09699472,3
7,108.59972321,34,121.54433076,0\H,50,1.0966891,37,111.60480732,34,-11
8.94336931,0\H,50,1.09096141,37,110.39427512,34,3.07751934,0\N,28,1.36
257052,27,30.50951994,26,-179.98560427,0\\Version=AM64L-G03RevD.01\Sta
te=1-A\HF=-1104.3868001\MP2=-1108.3885617\RMSD=5.517e-09\Thermal=0.\PG
=C01 [X(C23H27N4)]\\@

349-TT^{+}

1\1\GINC-PHOBOS\SP\RMP2-FC\6-31+G(2d,p)\C29H31N4(1+)\CHRISTOPH\03-Nov-
2011\0\\#p MP2(FC)/6-31+g(2d,p) scf=tight\\yin10ttsp_5\\1,1\C\C,1,4.37
864978\C,2,1.39462644,1,60.32635519\C,3,1.40130956,2,120.57816994,1,-0
.02269308,0\C,4,1.40171736,3,120.68015809,2,-0.38268272,0\C,5,1.410559
18,4,118.17610136,3,1.35644264,0\C,6,1.39485303,5,121.08811506,4,-1.48
269998,0\H,2,1.08702442,1,179.28120512,5,-159.04554425,0\H,3,1.0871401
9,2,120.19377388,1,179.70826789,0\H,4,1.08431559,3,118.89609161,2,179.
18361135,0\H,6,1.08806668,5,119.83711363,4,178.65130771,0\H,7,1.087404
29,6,119.63255246,5,-179.37460204,0\C,1,4.37504865,5,113.18671668,4,-6
.53348576,0\C,13,1.39385034,1,60.72054463,5,-113.64158774,0\C,14,1.401
98461,13,120.51329323,1,0.42390018,0\C,15,1.40160555,14,120.7337995,13
,0.10999005,0\C,16,1.40944287,15,118.18655505,14,-1.49936277,0\C,17,1.
39432868,16,120.99817575,15,1.96863304,0\H,14,1.08724046,13,120.219750
05,1,-179.15237557,0\H,15,1.08543659,14,118.07548101,13,-178.55637699,
0\H,17,1.08725002,16,119.64920765,15,-177.22792806,0\H,18,1.08713866,1
7,119.4829217,16,179.67636556,0\C,1,4.37850225,16,106.00426918,15,120.
52110201,0\C,23,1.39987591,1,59.04836734,16,-45.35533378,0\C,24,1.3946
1819,23,120.13659269,1,0.23454769,0\C,25,1.41155574,24,121.06352933,23
,-1.28921432,0\C,26,1.40273102,25,118.15306095,24,1.89906282,0\C,23,1.
39472052,1,60.32597521,16,134.33406728,0\H,23,1.08704042,1,179.1645395
8,16,-11.06251775,0\H,24,1.08716505,23,120.27939193,1,179.21342964,0\H
,25,1.08757536,24,119.17778062,23,178.0240913,0\H,27,1.08407543,26,120
.52983651,25,177.36550312,0\H,28,1.08712958,23,120.2000033,1,-179.0532
0721,0\H,13,1.08689018,1,178.8270972,16,176.52924623,0\C,1,4.38401509,
16,108.23095899,15,-0.10627039,0\C,35,1.43400222,1,56.43696039,16,-70.
00725297,0\C,36,1.37141106,35,120.8319647,1,-0.21996209,0\C,37,2.34219
736,36,91.71780226,35,0.23521828,0\C,38,1.3723725,37,91.16032769,36,-0
.08689366,0\H,37,1.08355364,36,121.71217751,35,-179.60005207,0\H,38,1.
08170399,37,147.1958696,36,-179.00645383,0\N,35,1.34243327,1,175.78687
002,16,60.48600005,0\C,42,1.3334009,35,124.67331467,1,-151.48849325,0\
H,39,1.0849684,38,119.47743799,37,-179.94498365,0\H,36,1.08543041,35,1
20.66606173,1,178.77178465,0\N,43,1.37155281,42,123.25097808,35,-44.94
474195,0\N,43,1.35741937,42,118.33806704,35,139.81554805,0\C,47,1.4663
9787,43,122.93091489,42,140.8386385,0\H,48,1.09677604,47,111.61869986,
43,118.84774827,0\H,48,1.09703553,47,108.64131701,43,-121.62580018,0\H
,48,1.09098416,47,110.37001798,43,-3.14702199,0\C,47,1.46418205,43,119
.20786767,42,-18.35330358,0\H,52,1.09529016,47,108.49650786,43,-151.71
034465,0\H,52,1.09036118,47,110.08395425,43,-32.17549899,0\H,52,1.0980
1542,47,111.0719928,43,88.34153074,0\C,46,1.46086029,43,121.33316503,4
2,-21.71046167,0\H,56,1.0917985,46,109.89165858,43,-21.21846327,0\H,56
,1.09934316,46,111.94551772,43,100.25792969,0\H,56,1.09646657,46,108.6

3135047,43,-139.96763043,0\C,46,1.46381429,43,123.03696031,42,150.7820
8937,0\H,60,1.09828581,46,108.70974333,43,-125.82964783,0\H,60,1.09142
652,46,110.18529673,43,-7.165702,0\H,60,1.09768131,46,111.98823914,43,
114.72065581,0\N,38,1.36365467,37,30.93468135,36,179.58958447,0\\Versi
on=AM64L-G03RevD.01\State=1-A\HF=-1333.9242005\MP2=-1338.7904228\RMSD=
4.151e-09\Thermal=0.\PG=C01 [X(C29H31N4)]\\@

350
see reference 4

350-BH⁺

1\1\GINC-NODE25\SP\RMP2-FC\6-31+G(2d,p)\C22H24N3(1+)\ZIP07\13-Jan-2011
\0\\#p MP2(FC)/6-31+G(2d,p) scf=tight\\yin9bhsp_129\\1,1\C\H,1,1.09634
408\C,1,4.33453366,2,106.22161764\C,3,1.39977162,1,59.38385,2,-33.2000
0346,0\C,4,1.39603966,3,119.93458733,1,0.16868769,0\C,5,1.40576872,4,1
20.54755366,3,-0.071324,0\C,6,1.40328543,5,119.2117011,4,0.42544096,0\
C,3,1.39788662,1,60.48085057,6,9.97677543,0\H,3,1.0870785,1,179.342243
01,6,-140.51140899,0\H,4,1.08707128,3,120.21092793,1,179.47728734,0\H,
5,1.08916938,4,119.67445021,3,179.55402335,0\H,7,1.08694268,6,119.9657
1548,5,178.25498465,0\H,8,1.08709812,3,120.11079675,1,-179.46396364,0\
C,1,4.33376291,6,114.16746695,5,-152.38871071,0\C,14,1.39873564,1,59.1
2189483,6,87.01980558,0\C,15,1.39671126,14,120.0977241,1,0.77932496,0\
C,16,1.40329152,15,120.44122649,14,-0.24177416,0\C,17,1.40270498,16,11
9.23014361,15,0.32815425,0\C,14,1.3978626,1,60.63107785,17,35.66856245
,0\H,14,1.08689952,1,179.13068427,17,-174.03567347,0\H,15,1.08698508,1
4,120.20035772,1,-179.57915948,0\H,16,1.08882691,15,119.71242068,14,-1
79.85715988,0\H,18,1.08838304,17,120.36827243,16,179.3892362,0\H,19,1.
08720234,14,120.12031546,1,179.47192035,0\C,1,4.32432062,17,108.785122
72,16,-144.9361416,0\C,25,1.4145804,1,57.10133212,17,71.74667727,0\C,2
6,1.37912952,25,120.99098932,1,0.82868605,0\C,27,2.34939635,26,91.1127
0262,25,-0.42032809,0\C,28,1.38926022,27,92.6158576,26,1.1341677,0\H,2
6,1.08282333,25,120.90117618,1,179.63553522,0\H,27,1.08424499,26,122.1
8330653,25,-179.5336295,0\H,28,1.08035843,27,145.15344357,26,-179.4764
9733,0\N,29,1.38167762,28,122.26832937,27,177.48685177,0\N,25,1.353930
01,1,179.10563783,17,6.54524922,0\C,33,1.45959912,29,115.95621676,28,1
55.54923844,0\C,34,1.46532309,25,119.99547737,1,-123.4579721,0\H,36,1.
09905998,34,110.32212567,25,-88.36028098,0\H,36,1.09503435,34,108.2642
7757,25,153.38467725,0\H,35,1.0942455,33,108.50250161,29,169.50357599,
0\H,35,1.10229306,33,111.78916638,29,-71.56879856,0\C,34,1.46187647,25
,120.85265504,1,67.64253138,0\H,41,1.09625029,34,110.68775686,25,-57.5
1100498,0\H,41,1.09852497,34,111.64952087,25,64.34423576,0\H,41,1.0917
2535,34,108.66149197,25,-176.0976085,0\C,33,1.45653838,29,119.57544895
,28,5.12617371,0\H,45,1.09291017,33,108.60918339,29,-174.81344577,0\H,
45,1.10244065,33,112.77353509,29,64.83980398,0\H,45,1.09615079,33,110.
12093588,29,-56.98173982,0\N,27,1.34877828,26,121.27222104,25,-0.01225
08,0\\Version=AM64L-G03RevD.01\State=1-A\HF=-1010.3202436\MP2=-1013.99
3578\RMSD=4.339e-09\Thermal=0.\PG=C01 [X(C22H24N3)]\\@

350-TT⁺

1\1\GINC-TOFU\SP\RMP2-FC\6-31+G(2d,p)\C28H28N3(1+)\CHRISTOPH\08-Nov-20
11\0\\#p MP2(FC)/6-31+g(2d,p) scf=tight\\yin9ttsp_12\\1,1\C\C,1,4.3744
9998\C,2,1.39975725,1,58.49324917\C,3,1.39438338,2,120.26889776,1,0.02
809035,0\C,4,1.40943035,3,121.00347336,2,1.06905602,0\C,5,1.40169439,4
,118.18563236,3,-1.99409603,0\C,2,1.39402041,1,60.77421924,5,21.942744
9,0\H,2,1.08687037,1,178.75667812,5,-177.28437679,0\H,3,1.08710776,2,1
20.24842961,1,-179.21150192,0\H,4,1.08726438,3,119.33439058,2,-178.085
26719,0\H,6,1.08537659,5,121.08755914,4,-177.23115757,0\H,7,1.08728946
,2,120.20753616,1,179.02326732,0\C,1,4.37816188,5,114.66083174,4,-69.8

5737877,0\C,13,1.39996326,1,59.02139662,5,-169.87758778,0\C,14,1.39467
946,13,120.13319966,1,-0.04214541,0\C,15,1.41044464,14,121.08888409,13
,-0.54555741,0\C,16,1.40185689,15,118.18482769,14,1.40302998,0\C,13,1.
39458617,1,60.314784,5,9.77983875,0\H,14,1.08733807,13,120.24336385,1,
179.88540932,0\H,15,1.0880205,14,119.04534852,13,179.3275494,0\H,17,1.
08431715,16,120.46210699,15,178.20882093,0\H,18,1.08716223,13,120.2011
8968,1,-179.65049012,0\C,1,4.37831273,5,105.97551582,4,56.97209821,0\C
,23,1.39980016,1,59.02429191,5,44.85245821,0\C,24,1.39461491,23,120.12
678092,1,-0.22986349,0\C,25,1.41164872,24,121.0773538,23,1.21750024,0\
C,26,1.40297834,25,118.150981,24,-1.79072081,0\C,23,1.39476282,1,60.35
42624,5,-134.83860386,0\H,23,1.08702685,1,179.13888093,5,10.518458,0\H
,24,1.08718219,23,120.27537895,1,-179.1788204,0\H,25,1.08745525,24,119
.18063449,23,-178.0593126,0\H,27,1.08402047,26,120.60220167,25,-177.50
280709,0\H,28,1.08709578,23,120.19374333,1,179.05989454,0\H,13,1.08701
509,1,179.29420927,5,-170.33911771,0\C,1,4.3656691,5,108.26741371,4,17
7.43507004,0\C,35,1.4471484,1,58.02638481,5,68.63045853,0\C,36,1.38566
15,35,118.14620814,1,1.29218228,0\C,37,2.34345889,36,93.01972322,35,-1
.57571031,0\C,38,1.38237928,37,90.69636812,36,1.45047197,0\H,37,1.0800
891,36,121.65862533,35,177.175636,0\H,38,1.08105094,37,147.79670119,36
,-179.6974702,0\N,35,1.35331697,1,178.56901878,5,79.19064829,0\C,42,1.
46528021,35,120.06698778,1,-17.39181814,0\H,43,1.09510466,42,108.28964
06,35,152.65247477,0\H,42,2.11565175,35,103.64150448,1,147.44209012,0\
C,42,1.46177248,35,120.79728576,1,172.86005785,0\H,46,1.091673,42,108.
67935624,35,-178.13082295,0\C,43,1.52666353,42,110.43281047,35,32.9259
8118,0\N,36,1.38405027,35,119.36937528,1,-178.12098017,0\H,48,1.102537
2,43,110.09066202,42,69.74302074,0\H,48,1.09431531,43,108.86888304,42,
-172.0895709,0\H,43,1.09914341,42,110.31487401,35,-89.08964001,0\H,39,
1.08281887,38,117.65720008,37,179.6405102,0\H,46,1.09833857,42,111.551
33084,35,62.37596463,0\C,49,1.45711633,36,119.34273299,35,-174.6393211
6,0\H,55,1.0929747,49,108.5816227,36,-175.85280955,0\H,55,1.09613478,4
9,110.07862074,36,-58.12892025,0\H,55,1.10236224,49,112.79189414,36,63
.67631532,0\N,38,1.34727023,37,30.71825062,36,-177.74093712,0\\Version
=AM64L-G03RevD.01\State=1-A\HF=-1239.8587602\MP2=-1244.3965385\RMSD=1.
986e-09\Thermal=0.\PG=C01 [X(C28H28N3)]\\@

351-Ac⁺

see reference 9

351-MOSC_re

\\#P b98/6-31G(d) opt\\opt of cat1ph2_mosc_b.004 b98/6-31G(d) on 4p 90
0 MB\\1,1\C,0.0564330511,-0.2093523656,0.0522705965\C,0.0243512989,-0.
2030418855,1.4268136904\C,1.2094946554,-0.1159654281,2.1989275262\C,2.
4742779389,-0.1749813262,1.4836002193\C,2.4176100533,-0.1826607249,0.0
951298177\N,1.2386255356,-0.1808491466,-0.6055690774\N,3.6720526257,-0
.1936618044,2.1777108155\C,3.6095710015,-0.4922855352,3.6284009517\C,2
.4477188511,0.3077425055,4.2502808445\N,1.1941561864,0.0232075828,3.53
91522297\C,2.7262348939,1.8259098573,4.3079760891\C,4.0243913051,2.128
3864905,5.0763090965\C,5.2088142598,1.3528285311,4.4766461281\C,4.8974
177059,-0.1524278459,4.4092510566\C,4.923642726,-0.3414404757,1.427461
4508\C,5.2089825835,-1.7573790973,0.8386770932\C,-0.0536534858,0.05795
99804,4.3189651416\C,-0.5842711673,-1.3421015452,4.6561609213\H,3.4007
291337,-1.563223929,3.7658874782\H,2.3190351857,-0.07506437,5.27132087
05\H,1.8730295289,2.3336915852,4.7773298833\H,2.80148396,2.2091306462,
3.2810015989\H,4.2195981005,3.2076220696,5.059751012\H,3.9000287857,1.
8478053164,6.1332703101\H,6.1110334036,1.5096534294,5.0808027762\H,5.4
293538868,1.7451393412,3.4731725629\H,5.7431999368,-0.7318625889,4.026
7285166\H,4.7332029204,-0.5281853256,5.429869284\H,-0.8037762357,0.650
1363648,3.7820613138\H,0.1662958355,0.6087039155,5.239010334\H,-0.8202

902662,-1.9135131517,3.7511963274\H,0.1548038665,-1.9095626912,5.23334
81409\H,-1.4972215908,-1.2599052251,5.2569937831\H,5.7430820102,-0.046
434962,2.0843988532\H,4.9110896709,0.3981806098,0.616454889\H,4.334564
944,-2.0441644545,0.2369934458\H,3.3049646185,-0.2043656672,-0.5172674
174\H,-0.835937705,-0.2355096069,-0.5553220597\H,-0.9461702387,-0.2331
959429,1.9046138165\C,1.2571150945,-0.0393168671,-2.1131415536\C,2.490
0767028,-0.7238993886,-2.7101848716\C,2.4712297052,-2.1301122519,-2.74
83233688\C,3.6155662152,-0.0362634694,-3.1916573004\C,3.5585466737,-2.
8355118404,-3.2633234254\H,1.5970759019,-2.6630378193,-2.3831500171\C,
4.7042371299,-0.7506555374,-3.7035381767\H,3.6600531758,1.0471169815,-
3.1773338233\C,4.679211071,-2.145969685,-3.7413924331\H,3.5300961474,-
3.9218047072,-3.294057057\H,5.5730577605,-0.2105431105,-4.0696257357\H
,5.5304798783,-2.6939917285,-4.1353370101\C,1.1822356722,1.5073648269,
-2.3825982167\F,1.3047011607,1.7801894346,-3.687604894\F,2.1535056897,
2.1662595039,-1.7258007528\F,0.0037893907,1.9853828482,-1.9648959308\O
,0.0674268588,-0.6492827329,-2.5122345261\C,-0.2832210761,-0.628626920
8,-3.9062286166\H,-1.0684104731,-1.3797687878,-4.009236365\H,-0.671071
2353,0.351841423,-4.1990339998\H,0.5714790056,-0.8989262057,-4.5348349
011\C,6.4154213982,-1.7640427275,-0.1128963607\C,6.6964732924,-2.95201
08674,-0.813652185\C,7.2541694261,-0.659229679,-0.3220265465\C,7.77282
42488,-3.0316018699,-1.6967820961\H,6.0682376716,-3.8258633744,-0.6506
024391\C,8.3349235501,-0.7353858055,-1.2118998221\H,7.0875796108,0.277
5808395,0.2031874772\C,8.5977449121,-1.9184694073,-1.9026907624\H,7.97
43222112,-3.9642067749,-2.2192857383\H,8.9731408866,0.1336794939,-1.35
3987525\H,9.4405260217,-1.9789505232,-2.5869198898\C,5.3445151089,-2.8
197926724,1.9331201673\C,6.5257723433,-2.9439315828,2.6844161785\C,4.2
825277475,-3.6956948661,2.2054050273\C,6.6309260249,-3.9025645496,3.69
5545326\H,7.3758234369,-2.301837124,2.4596186919\C,4.3858639763,-4.659
4417493,3.2155749442\H,3.3681455782,-3.6293195663,1.6164653257\C,5.559
7136976,-4.7616637838,3.9670086649\H,7.5545273986,-3.9878313561,4.2632
897115\H,3.5550040218,-5.335145797,3.4057582033\H,5.6464361323,-5.5134
411227,4.7475585279\\Version=AM64L-G03RevD.01\State=1-A\HF=-1933.38101
23\RMSD=7.922e-09\RMSF=2.765e-05\Thermal=0.\Dipole=-1.6250488,0.037597
9,0.9177754\PG=C01 [X(C36H39F3N3O1)]\\@

351-MOSC_si

1\1\GINC-NODE8\FOpt\RB98\6-31G(d)\C36H39F3N3O1(1+)\ZIP01\24-Apr-2009\0
\\#P b98/6-31G(d) opt\\opt of cat1ph2_mosc_a1.001 b98/6-31G(d) on 4p 9
00 MB\\1,1\C,0.1238883377,-0.0711345332,-0.0290594971\C,0.0253357272,-
0.0656079247,1.3429633577\C,1.174180299,-0.0337446485,2.1737193529\C,2
.4678978399,-0.1015587732,1.5161546313\C,2.4786555686,-0.080012823,0.1
284519908\N,1.3368621876,-0.0432060447,-0.62833759\N,3.634776787,-0.15
80241671,2.2622883552\C,3.4939406408,-0.5451212916,3.6852611704\C,2.33
01475373,0.2596698852,4.2987924972\N,1.1016093102,0.0626398284,3.51588
24567\C,2.6594862437,1.7608653525,4.4534840804\C,3.9311236668,1.972206
4329,5.2932685378\C,5.1147190789,1.1875276352,4.7041743495\C,4.7555451
631,-0.3000124737,4.5399499217\C,4.9001906536,-0.3568341197,1.54756789
02\C,5.1017395815,-1.7760252534,0.9203382996\C,-0.1791629934,0.1078361
086,4.2396949101\C,-0.7690616915,-1.2856537339,4.4958129009\H,3.242173
8278,-1.6148156756,3.7478142343\H,2.1388209205,-0.173236138,5.28958806
58\H,1.8043286803,2.2751416326,4.9120682585\H,2.7960568748,2.192880388
3,3.4528238651\H,4.1640753336,3.0428329781,5.3453617772\H,3.749002945,
1.639961579,6.3265699298\H,5.9929673341,1.2784488397,5.3553131249\H,5.
3943914442,1.62354807,3.7341070461\H,5.5977513218,-0.8874757401,4.1604
579366\H,4.532607361,-0.7243107981,5.5298778528\H,-0.8844467056,0.7451
311653,3.6936793661\H,0.0150477831,0.614913412,5.1902278913\H,-0.98013
72906,-1.8137110812,3.5589648796\H,-0.0769136084,-1.8995235316,5.08364
12797\H,-1.7062733597,-1.1958731274,5.0568737522\H,5.7131433815,-0.134

5384351,2.239934348\H,4.9622561816,0.4067076066,0.7615746272\H,4.13684
99943,-2.08222182,0.4958918971\H,3.3959927906,-0.0917013279,-0.4380988
064\H,-0.7380550015,-0.0803185925,-0.6797418056\H,-0.9680819585,-0.071
3113133,1.7721911941\C,1.4438985767,-0.1265714539,-2.1369417162\C,1.47
74694271,-1.6636956358,-2.4687740065\O,0.2419726221,0.4236455143,-2.58
00997634\F,1.6991958897,-1.8761861959,-3.7710762685\F,0.305106538,-2.2
221879051,-2.1455482032\F,2.4417178968,-2.3011168568,-1.7749771397\C,2
.6644087824,0.6557047904,-2.6327536342\C,3.8677097258,0.0574869696,-3.
0381833328\C,2.5528179453,2.0580322677,-2.6406405918\C,4.9435040881,0.
8556349693,-3.4430014701\H,3.9932067233,-1.0193076452,-3.0362452843\C,
3.626001431,2.8473623142,-3.052900068\H,1.6209322857,2.5230355337,-2.3
295928012\C,4.8254241619,2.246887307,-3.4542335071\H,5.8728977416,0.37
83169085,-3.7416710895\H,3.5260917688,3.9297579876,-3.0616173324\H,5.6
621901352,2.8627390779,-3.7741227675\C,-0.0272436586,0.4407995476,-3.9
921537478\H,-0.8521116602,1.1454124054,-4.1122181767\H,0.8427813837,0.
7900064606,-4.5573739302\H,-0.3341955058,-0.5486070268,-4.3447468881\C
,5.4617356174,-2.8317032067,1.970697733\C,6.7305637761,-2.8650884469,2
.5737123001\C,4.515860421,-3.7969020489,2.3485753686\C,7.0357698375,-3
.8261588013,3.541141433\H,7.4938555632,-2.1515731371,2.2680068161\C,4.
8188807051,-4.7614958817,3.3161890978\H,3.5343878743,-3.8008466562,1.8
752110451\C,6.0795246623,-4.7758635121,3.9183376323\H,8.0252121147,-3.
8407008119,3.9923105566\H,4.0740375131,-5.5053464966,3.58994205\H,6.32
11297939,-5.5273675974,4.6659104268\C,6.098554536,-1.7503920993,-0.242
4412603\C,5.937400346,-2.6695847305,-1.2939681464\C,7.1828213112,-0.85
98057618,-0.2976176083\C,6.8294771256,-2.6986349424,-2.3684978238\H,5.
108052401,-3.3747273066,-1.2640151643\C,8.0786380311,-0.8859533916,-1.
3731319987\H,7.3429592141,-0.1353860866,0.4983272793\C,7.9054668229,-1
.8038235392,-2.412219027\H,6.689334435,-3.4236114825,-3.1671892343\H,8
.9144259027,-0.1902781568,-1.3931529899\H,8.6062098675,-1.8294944306,-
3.2432966055\\Version=AM64L-G03RevD.01\State=1-A\HF=-1933.3838496\RMSD
=4.819e-09\RMSF=2.844e-05\Thermal=0.\Dipole=-1.6610047,1.5402118,0.978
6234\PG=C01 [X(C36H39F3N3O1)]\\@

352-MOSC_re

1\1\GINC-NODE24\Freq\RB98\6-31G(d)\C38H41F3N3O1(1+)\ZIP01\13-Jul-2008\
1\\#p b98/6-31G(d) freq=noraman\\freq of cat6_1mosc_b3 b98/6-31G(d) on
4p 256 MB\\1,1\C\C,1,B1\C,2,B2,1,A1\C,3,B3,2,A2,1,D1,0\H,1,B4,4,A3,3,
D2,0\H,1,B5,4,A4,3,D3,0\H,4,B6,3,A5,2,D4,0\H,4,B7,3,A6,2,D5,0\C,3,B8,2
,A7,1,D6,0\C,2,B9,1,A8,4,D7,0\H,10,B10,2,A9,1,D8,0\C,3,B11,2,A10,1,D9,
0\H,12,B12,3,A11,2,D10,0\H,3,B13,2,A12,1,D11,0\C,9,B14,3,A13,2,D12,0\H
,15,B15,9,A14,3,D13,0\H,15,B16,9,A15,3,D14,0\H,15,B17,9,A16,3,D15,0\C,
9,B18,3,A17,2,D16,0\H,19,B19,9,A18,3,D17,0\H,19,B20,9,A19,3,D18,0\H,19
,B21,9,A20,3,D19,0\C,2,B22,1,A21,4,D20,0\H,23,B23,2,A22,1,D21,0\H,23,B
24,2,A23,1,D22,0\H,23,B25,2,A24,1,D23,0\N,10,B26,2,A25,1,D24,0\N,12,B2
7,3,A26,2,D25,0\C,27,B28,10,A27,2,D26,0\C,28,B29,12,A28,3,D27,0\C,29,B
30,27,A29,10,D28,0\H,31,B31,29,A30,27,D29,0\C,31,B32,29,A31,27,D30,0\H
,33,B33,31,A32,29,D31,0\N,33,B34,31,A33,29,D32,0\C,35,B35,33,A34,31,D3
3,0\H,36,B36,35,A35,33,D34,0\C,28,B37,12,A36,3,D35,0\H,38,B38,28,A37,1
2,D36,0\H,38,B39,28,A38,12,D37,0\C,27,B40,10,A39,2,D38,0\H,41,B41,27,A
40,10,D39,0\H,41,B42,27,A41,10,D40,0\C,41,B43,27,A42,10,D41,0\C,44,B44
,41,A43,27,D42,0\C,44,B45,41,A44,27,D43,0\C,45,B46,44,A45,41,D44,0\H,4
5,B47,44,A46,41,D45,0\C,46,B48,44,A47,41,D46,0\H,46,B49,44,A48,41,D47,
0\C,49,B50,46,A49,44,D48,0\H,47,B51,45,A50,44,D49,0\H,49,B52,46,A51,44
,D50,0\H,51,B53,49,A52,46,D51,0\C,38,B54,28,A53,12,D52,0\C,55,B55,38,A
54,28,D53,0\C,55,B56,38,A55,28,D54,0\C,56,B57,55,A56,38,D55,0\H,56,B58
,55,A57,38,D56,0\C,57,B59,55,A58,38,D57,0\H,57,B60,55,A59,38,D58,0\C,6
0,B61,57,A60,55,D59,0\H,58,B62,56,A61,55,D60,0\H,60,B63,57,A62,55,D61,
0\H,62,B64,60,A63,57,D62,0\C,35,B65,33,A64,31,D63,0\C,66,B66,35,A65,33

,D64,0\C,67,B67,66,A66,35,D65,0\C,67,B68,66,A67,35,D66,0\C,68,B69,67,A
68,66,D67,0\H,68,B70,67,A69,66,D68,0\C,69,B71,67,A70,66,D69,0\H,69,B72
,67,A71,66,D70,0\C,72,B73,69,A72,67,D71,0\H,70,B74,68,A73,67,D72,0\H,7
2,B75,69,A74,67,D73,0\H,74,B76,72,A75,69,D74,0\C,66,B77,35,A76,33,D75,
0\F,78,B78,66,A77,35,D76,0\F,78,B79,66,A78,35,D77,0\F,78,B80,66,A79,35
,D78,0\O,66,B81,35,A80,33,D79,0\C,82,B82,66,A81,35,D80,0\H,83,B83,82,A
82,66,D81,0\H,83,B84,82,A83,66,D82,0\H,83,B85,82,A84,66,D83,0\\B1=1.56
807269\B2=2.27943019\B3=1.54722688\B4=1.0973188\B5=1.09486844\B6=1.096
57257\B7=1.09387316\B8=1.5605718\B9=1.58528395\B10=1.09914381\B11=1.55
905902\B12=1.10097972\B13=1.09525934\B14=1.54738881\B15=1.09295165\B16
=1.09698421\B17=1.09769962\B18=1.54245575\B19=1.09791706\B20=1.0974454
1\B21=1.09077543\B22=1.5285634\B23=1.09810687\B24=1.09448332\B25=1.096
64515\B26=1.48325431\B27=1.47057262\B28=1.35914812\B29=1.37794831\B30=
1.41703572\B31=1.08159831\B32=1.37779064\B33=1.07948439\B34=1.35266306
\B35=1.36633783\B36=1.07939784\B37=1.47199373\B38=1.10350411\B39=1.094
95549\B40=1.47797697\B41=1.092012\B42=1.10148377\B43=1.52671931\B44=1.
40462135\B45=1.40102089\B46=1.39637546\B47=1.0894392\B48=1.39997721\B4
9=1.08672904\B50=1.39662767\B51=1.08715628\B52=1.08739539\B53=1.086960
77\B54=1.52566266\B55=1.4020815\B56=1.40358883\B57=1.39851471\B58=1.08
609131\B59=1.3983625\B60=1.08929532\B61=1.39901723\B62=1.0876318\B63=1
.08728796\B64=1.08728578\B65=1.52601165\B66=1.53408711\B67=1.402456\B6
8=1.40290337\B69=1.39669526\B70=1.08695699\B71=1.39695163\B72=1.084536
12\B73=1.39768649\B74=1.08696384\B75=1.08694205\B76=1.08703166\B77=1.5
7302329\B78=1.33110446\B79=1.34345661\B80=1.34658606\B81=1.38236461\B8
2=1.4401871\B83=1.0937968\B84=1.09121128\B85=1.09538664\A1=75.24867954
\A2=78.04975178\A3=112.72663038\A4=111.7280706\A5=112.1239881\A6=111.2
7862525\A7=43.55559641\A8=103.21496574\A9=107.96633201\A10=77.36269079
\A11=109.28495754\A12=158.8868496\A13=112.99772024\A14=114.37240369\A1
5=110.35542324\A16=109.74453298\A17=115.67954012\A18=109.6908072\A19=1
10.00921849\A20=114.47683564\A21=111.63178865\A22=111.95869998\A23=113
.35460332\A24=108.96744454\A25=118.35355339\A26=113.26042923\A27=121.3
4931307\A28=119.88149461\A29=121.94611939\A30=120.76109151\A31=122.023
74282\A32=121.12187264\A33=120.57336453\A34=119.69484775\A35=114.57142
031\A36=113.82093898\A37=109.69743326\A38=106.49406695\A39=114.9235035
1\A40=106.64280415\A41=108.34453409\A42=116.81499155\A43=117.56509809\
A44=123.40496174\A45=120.59017406\A46=119.8367873\A47=120.34352968\A48
=119.93245256\A49=120.32882389\A50=119.71827415\A51=119.57015519\A52=1
20.23078052\A53=115.76985199\A54=121.98207767\A55=118.92847922\A56=120
.39511705\A57=119.60087456\A58=120.60764427\A59=119.80471997\A60=120.0
5694468\A61=119.68094052\A62=119.74759562\A63=120.18221859\A64=124.964
83521\A65=110.12934726\A66=121.54681771\A67=118.7052846\A68=120.073756
61\A69=120.76250894\A70=119.94139982\A71=119.7836775\A72=120.31042166\
A73=119.59976881\A74=119.50418317\A75=120.13532182\A76=110.07299142\A7
7=108.45474892\A78=111.80259288\A79=112.29515551\A80=108.96182502\A81=
120.39218361\A82=110.81312847\A83=104.67654582\A84=111.55934208\D1=2.3
4236693\D2=-117.86950701\D3=122.38728438\D4=-123.23300598\D5=117.06294
748\D6=125.68255213\D7=-74.43885653\D8=-43.79717897\D9=-106.54675044\D
10=112.73673901\D11=130.90583587\D12=-117.82137557\D13=60.76974324\D14
=-177.99653846\D15=-60.2287203\D16=120.14430305\D17=71.92605834\D18=-1
70.89719184\D19=-49.11770859\D20=157.06807186\D21=54.68853715\D22=177.
55110743\D23=-62.7399567\D24=-162.35415266\D25=-128.19389467\D26=-136.
08242484\D27=140.63886734\D28=-164.69592504\D29=-2.43805458\D30=179.58
534382\D31=179.86172339\D32=-0.73120768\D33=-0.36668061\D34=179.907858
13\D35=-70.99646447\D36=-98.52082768\D37=15.76367208\D38=72.1186804\D3
9=-7.83826599\D40=105.90737197\D41=-130.05102696\D42=-169.22658898\D43
=11.78583387\D44=-178.31913359\D45=1.29856182\D46=178.49049037\D47=-1.
73624499\D48=-0.00759701\D49=179.67972686\D50=179.99446927\D51=-179.90
953918\D52=136.80696935\D53=-32.93826768\D54=149.97299111\D55=-176.759

74271\D56=3.5273391\D57=176.51563421\D58=-3.02148718\D59=0.48725518\D6
0=179.73034817\D61=-179.45993321\D62=-179.73271828\D63=-175.29092116\D
64=97.40982282\D65=-32.72993214\D66=150.08281658\D67=-177.91382656\D68
=2.41077237\D69=178.00938041\D70=-2.35359312\D71=-0.37286739\D72=-179.
78765749\D73=179.78646669\D74=-179.90333839\D75=-23.45026827\D76=-168.
34442096\D77=72.0828035\D78=-48.68172082\D79=-145.07883757\D80=67.3370
8407\D81=76.72301385\D82=-164.54156565\D83=-46.35763657\\Version=AM64L
-G03RevD.01\State=1-A\HF=-2010.7399505\RMSD=4.404e-09\RMSF=2.302e-06\Z
eroPoint=0.7238183\Thermal=0.7626616\Dipole=-0.4159386,-0.1683875,-0.8
198664\\\@

352-MOSC_si

1\1\GINC-NODE22\Freq\RB98\6-31G(d)\C38H41F3N3O1(1+)\ZIP01\13-Jul-2008\
1\\#p b98/6-31G(d) freq=noraman\\freq of cat6_1mosc_a3 b98/6-31G(d) on
4p 256 MB\\1,1\C\C,1,B1\C,2,B2,1,A1\C,3,B3,2,A2,1,D1,0\H,1,B4,4,A3,3,
D2,0\H,1,B5,4,A4,3,D3,0\H,4,B6,3,A5,2,D4,0\H,4,B7,3,A6,2,D5,0\C,3,B8,2
,A7,1,D6,0\C,2,B9,1,A8,4,D7,0\H,10,B10,2,A9,1,D8,0\C,3,B11,2,A10,1,D9,
0\H,12,B12,3,A11,2,D10,0\H,3,B13,2,A12,1,D11,0\C,9,B14,3,A13,2,D12,0\H
,15,B15,9,A14,3,D13,0\H,15,B16,9,A15,3,D14,0\H,15,B17,9,A16,3,D15,0\C,
9,B18,3,A17,2,D16,0\H,19,B19,9,A18,3,D17,0\H,19,B20,9,A19,3,D18,0\H,19
,B21,9,A20,3,D19,0\C,2,B22,1,A21,4,D20,0\H,23,B23,2,A22,1,D21,0\H,23,B
24,2,A23,1,D22,0\H,23,B25,2,A24,1,D23,0\N,10,B26,2,A25,1,D24,0\N,12,B2
7,3,A26,2,D25,0\C,27,B28,10,A27,2,D26,0\C,28,B29,12,A28,3,D27,0\C,29,B
30,27,A29,10,D28,0\H,31,B31,29,A30,27,D29,0\C,31,B32,29,A31,27,D30,0\H
,33,B33,31,A32,29,D31,0\N,33,B34,31,A33,29,D32,0\C,35,B35,33,A34,31,D3
3,0\H,36,B36,35,A35,33,D34,0\C,28,B37,12,A36,3,D35,0\H,38,B38,28,A37,1
2,D36,0\H,38,B39,28,A38,12,D37,0\C,27,B40,10,A39,2,D38,0\H,41,B41,27,A
40,10,D39,0\H,41,B42,27,A41,10,D40,0\C,41,B43,27,A42,10,D41,0\C,44,B44
,41,A43,27,D42,0\C,44,B45,41,A44,27,D43,0\C,45,B46,44,A45,41,D44,0\H,4
5,B47,44,A46,41,D45,0\C,46,B48,44,A47,41,D46,0\H,46,B49,44,A48,41,D47,
0\C,49,B50,46,A49,44,D48,0\H,47,B51,45,A50,44,D49,0\H,49,B52,46,A51,44
,D50,0\H,51,B53,49,A52,46,D51,0\C,38,B54,28,A53,12,D52,0\C,55,B55,38,A
54,28,D53,0\C,55,B56,38,A55,28,D54,0\C,56,B57,55,A56,38,D55,0\H,56,B58
,55,A57,38,D56,0\C,57,B59,55,A58,38,D57,0\H,57,B60,55,A59,38,D58,0\C,6
0,B61,57,A60,55,D59,0\H,58,B62,56,A61,55,D60,0\H,60,B63,57,A62,55,D61,
0\H,62,B64,60,A63,57,D62,0\C,35,B65,33,A64,31,D63,0\C,66,B66,35,A65,33
,D64,0\C,67,B67,66,A66,35,D65,0\C,67,B68,66,A67,35,D66,0\C,68,B69,67,A
68,66,D67,0\H,68,B70,67,A69,66,D68,0\C,69,B71,67,A70,66,D69,0\H,69,B72
,67,A71,66,D70,0\C,72,B73,69,A72,67,D71,0\H,70,B74,68,A73,67,D72,0\H,7
2,B75,69,A74,67,D73,0\H,74,B76,72,A75,69,D74,0\C,66,B77,35,A76,33,D75,
0\F,78,B78,66,A77,35,D76,0\F,78,B79,66,A78,35,D77,0\F,78,B80,66,A79,35
,D78,0\O,66,B81,35,A80,33,D79,0\C,82,B82,66,A81,35,D80,0\H,83,B83,82,A
82,66,D81,0\H,83,B84,82,A83,66,D82,0\H,83,B85,82,A84,66,D83,0\\B1=1.56
757895\B2=2.28032672\B3=1.54684399\B4=1.09732181\B5=1.09491836\B6=1.09
670025\B7=1.0939392\B8=1.5601191\B9=1.58529369\B10=1.09926991\B11=1.55
862031\B12=1.10128243\B13=1.09529129\B14=1.54703109\B15=1.09297245\B16
=1.09710875\B17=1.09773111\B18=1.54280151\B19=1.09799895\B20=1.0976872
8\B21=1.09088949\B22=1.52873343\B23=1.09810759\B24=1.09435195\B25=1.09
663095\B26=1.4827407\B27=1.47047688\B28=1.3598182\B29=1.37933734\B30=1
.41789169\B31=1.08145701\B32=1.37675554\B33=1.08110109\B34=1.35032132\
B35=1.36559628\B36=1.07873846\B37=1.47342327\B38=1.10407008\B39=1.0949
134\B40=1.4777567\B41=1.09201128\B42=1.10159562\B43=1.52653804\B44=1.4
04597\B45=1.40108794\B46=1.39653823\B47=1.08947364\B48=1.3997233\B49=1
.08666029\B50=1.39677011\B51=1.08719403\B52=1.08737657\B53=1.08698604\
B54=1.52440393\B55=1.40170154\B56=1.40374451\B57=1.39775242\B58=1.0865
8829\B59=1.3981056\B60=1.08964676\B61=1.39795231\B62=1.08743039\B63=1.
08735779\B64=1.08710959\B65=1.5307089\B66=1.53366173\B67=1.40783951\B6
8=1.40466831\B69=1.39578077\B70=1.08719395\B71=1.39817899\B72=1.083256

47\B73=1.39653843\B74=1.08705988\B75=1.08685347\B76=1.08700009\B77=1.5
7274223\B78=1.33606509\B79=1.33815477\B80=1.34065816\B81=1.38731182\B8
2=1.4341794\B83=1.09373101\B84=1.09163532\B85=1.09693191\A1=75.2398872
\A2=78.02203068\A3=112.71730576\A4=111.70329855\A5=112.12550722\A6=111
.30031714\A7=43.57050052\A8=103.15552322\A9=107.89469727\A10=77.362242
11\A11=109.26415099\A12=158.85692182\A13=113.12831481\A14=114.41020491
\A15=110.28189151\A16=109.82669647\A17=115.60780952\A18=109.72362059\A
19=109.95646511\A20=114.60358035\A21=111.65780032\A22=111.88406255\A23
=113.31364778\A24=109.03765311\A25=118.535825\A26=113.19457541\A27=121
.36168646\A28=119.71595078\A29=121.88307964\A30=120.76181555\A31=121.8
0856824\A32=122.91510167\A33=120.55322189\A34=120.0625905\A35=115.5342
5703\A36=113.33325376\A37=109.49958739\A38=106.4321272\A39=114.8970994
5\A40=106.59390445\A41=108.42091222\A42=116.69720341\A43=117.71884576\
A44=123.27294371\A45=120.59106153\A46=119.83876324\A47=120.36710878\A4
8=119.9365943\A49=120.33060649\A50=119.70963122\A51=119.55190749\A52=1
20.23465388\A53=115.85606751\A54=121.86968624\A55=119.02273623\A56=120
.35357226\A57=119.64649443\A58=120.54773049\A59=119.82362634\A60=120.0
604629\A61=119.67795016\A62=119.73121717\A63=120.18851687\A64=119.8168
77\A65=109.88127421\A66=119.55596876\A67=121.5188993\A68=120.73250293\
A69=120.24177333\A70=120.26159269\A71=120.94255138\A72=120.56227746\A7
3=119.61887497\A74=119.23929636\A75=120.23401702\A76=104.65743489\A77=
111.02370589\A78=109.86898368\A79=110.99465996\A80=108.88123489\A81=12
0.22718641\A82=111.22192554\A83=104.54414132\A84=111.75324247\D1=2.277
05495\D2=-117.97612059\D3=122.32419678\D4=-123.16681785\D5=117.1269413
7\D6=125.68156196\D7=-74.35690754\D8=-43.47334947\D9=-106.67239675\D10
=112.90536952\D11=130.73519307\D12=-117.73154297\D13=59.99294916\D14=-
178.83497897\D15=-61.09714962\D16=120.14932048\D17=72.07699643\D18=-17
0.72268436\D19=-48.83787168\D20=157.1860833\D21=54.82307015\D22=177.61
922817\D23=-62.63652518\D24=-162.06207053\D25=-128.09697603\D26=-136.1
9845598\D27=140.92090634\D28=-164.37402804\D29=-2.49828567\D30=-179.94
323085\D31=177.13633403\D32=-2.10825165\D33=2.51885124\D34=177.2775914
2\D35=-72.3442027\D36=-96.54571145\D37=17.79630488\D38=72.0301786\D39=
-8.42583179\D40=105.36427138\D41=-130.64434356\D42=-167.33334234\D43=1
3.68848113\D44=-178.33703433\D45=1.31881533\D46=178.51451156\D47=-1.87
248612\D48=-0.01582013\D49=179.71119385\D50=179.90956518\D51=-179.9515
3548\D52=139.10869215\D53=-35.32217709\D54=146.79335056\D55=-177.77236
364\D56=2.979324\D57=177.5082598\D58=-2.56261869\D59=0.4250333\D60=-17
9.79704613\D61=-179.80338046\D62=179.7584052\D63=175.13480235\D64=142.
26840543\D65=-39.69397519\D66=144.62697673\D67=-176.3120738\D68=3.7626
7357\D69=176.43592268\D70=-3.48406746\D71=-0.42848673\D72=179.79180054
\D73=179.75668819\D74=-179.82627156\D75=-94.36384758\D76=170.33134462\
D77=50.95784265\D78=-68.74924312\D79=15.10679794\D80=91.54432646\D81=6
1.33890319\D82=179.83374869\D83=-61.78830945\\Version=AM64L-G03RevD.01
\State=1-A\HF=-2010.7394586\RMSD=4.164e-09\RMSF=5.675e-06\\\@

352-Ac^{+}
see reference 9

353-MOSC_re
1\1\GINC-NODE15\SP\RMP2-FC\6-31+G(2d,p)\C34H32F3N2O1(1+)\ZIP01\12-Mar-
2009\0\\#P MP2/6-31+G(2d,p) scf=(direct,tight) int=finegrid geom=check
guess=read\\sp of spi1_mosc_a3.358 MP2-5\\1,1\N,0,-0.09508341,-0.0718
872757,0.0296385364\C,0,-0.071702773,0.0550188804,1.3866744562\C,0,1.0
723988458,0.0079820417,2.1522337816\C,0,2.354714441,-0.1102340671,1.47
60265025\C,0,2.261645967,-0.3740754554,0.0642988796\C,0,1.0767905512,-
0.3419917122,-0.6133263857\N,0,3.566383498,0.0028966585,2.0555515312\C
,0,3.8204481571,0.5179013369,3.4192537802\C,0,4.084956758,2.029387154,
3.4260013375\C,0,0.8178622977,-0.0161302964,3.6356661781\C,0,0.3358831

225,1.1715358573,4.290363739\C,0,0.0395047216,1.1198644173,5.695151851
5\C,0,0.2370214963,-0.0994382993,6.3969280342\C,0,0.7045513428,-1.2182
980203,5.7492361846\C,0,0.9937014181,-1.2048281649,4.3531699942\C,0,-0
.4364874287,2.2867606539,6.3557838904\C,0,-0.6049605048,3.4699132409,5
.6702715632\C,0,-0.2985799204,3.5322913748,4.2881298056\C,0,0.15904252
92,2.417134584,3.6161117078\C,0,1.5333630217,-2.4610090482,3.743887258
4\C,0,2.6842162542,-3.0517647089,4.3003761785\C,0,3.2079312815,-4.2361
169781,3.7748256689\C,0,2.5843080749,-4.8571595138,2.6878454331\C,0,1.
4300410922,-4.2904724844,2.1376593557\C,0,0.9079099983,-3.1038393714,2
.6599469772\C,0,4.8052989696,-0.2653727388,1.2887327157\C,0,5.11257051
04,-1.7640131903,1.1736307871\H,0,3.142906608,-0.5904433,-0.5228078151
\H,0,-1.0353706299,0.1760498176,1.8657650801\H,0,1.0113240162,-0.50190
31247,-1.6802707943\H,0,5.6146983911,0.240954297,1.8213563623\H,0,4.74
95780334,0.2164682074,0.3067474179\H,0,4.6878582342,-0.030723991,3.802
9277596\H,0,2.9815436836,0.279320945,4.0667302416\H,0,0.385874493,2.49
13875193,2.5561187877\H,0,0.8374592701,-2.1494577188,6.2939032334\H,0,
-0.4245300012,4.4698114635,3.7518628258\H,0,-0.6622317512,2.2266267494
,7.418366528\H,0,0.0089855819,-0.138020193,7.4600533417\H,0,-0.9668815
531,4.3559381198,6.1852671865\H,0,3.1708773977,-2.5762585617,5.1499034
451\H,0,-0.0072048616,-2.6943120748,2.2408623985\H,0,4.0964946515,-4.6
775907942,4.220184887\H,0,0.9239654885,-4.7809755807,1.3093278073\H,0,
2.9853846035,-5.783096431,2.2831141671\H,0,4.3211014615,2.3518362391,4
.4466468643\H,0,4.9295711518,2.301483429,2.7818151666\H,0,3.1997278561
,2.582512523,3.0930809048\H,0,6.0513990265,-1.9084742012,0.6265998859\
H,0,4.3183359522,-2.3061744505,0.6483251593\H,0,5.2169254305,-2.214165
5252,2.1668663884\C,0,-1.4060246217,-0.0785513627,-0.7197402116\C,0,-1
.9054951981,-1.5666576855,-0.6723180221\O,0,-1.033751499,0.2542555756,
-2.0225370967\C,0,-2.0555389753,0.3483416464,-3.0303002326\H,0,-2.3547
878681,-0.6448528628,-3.3781716091\H,0,-1.5926847684,0.9008990262,-3.8
49861345\H,0,-2.9271746549,0.8972910806,-2.6600294727\C,0,-2.367601347
1,0.9415825733,-0.1029889997\C,0,-3.5357278703,0.60470775,0.5972088251
\C,0,-2.0264508325,2.2970481449,-0.2678425744\C,0,-4.3484216812,1.6149
402265,1.1247297028\H,0,-3.8303046109,-0.4291045988,0.7373349201\C,0,-
2.8400735811,3.2978745967,0.2610920403\H,0,-1.1293398996,2.5600277484,
-0.8231163004\C,0,-4.0045091154,2.9578037725,0.9603574425\H,0,-5.25348
95415,1.3438855644,1.6616939001\H,0,-2.5702698126,4.3417026225,0.12248
02315\H,0,-4.6412063191,3.737468085,1.3704718606\F,0,-1.0370760874,-2.
3568383064,-1.313303522\F,0,-2.0069447997,-2.0084320473,0.5977731949\F
,0,-3.1039875357,-1.7013147025,-1.2512698457\\Version=AM64L-G03RevD.01
\State=1-A\HF=-1788.1819706\MP2=-1794.2184032\RMSD=4.027e-09\Thermal=0
.\PG=C01 [X(C34H32F3N2O1)]\\@

353-MOSC_si

1\1\GINC-NODE5\SP\RMP2-FC\6-31+G(2d,p)\C34H32F3N2O1(1+)\ZIP01\08-Mar-2
009\0\\#P MP2/6-31+G(2d,p) scf=(direct,tight) int=finegrid geom=check
guess=read\\sp of spi1_mosc_a2.272 MP2-5\\1,1\N,0,-0.0279894412,0.0376
613124,0.0653971067\C,0,-0.0179239324,-0.024063848,1.4255902011\C,0,1.
1233985695,-0.0957235656,2.1951024884\C,0,2.416449321,-0.0870956373,1.
5306471434\C,0,2.3473946543,0.0182460259,0.0974999889\C,0,1.166690453,
0.0718988614,-0.5871633258\N,0,3.6281085482,-0.1758708703,2.1261087735
\C,0,3.8786363635,-0.4798265849,3.5552589071\C,0,4.5804956659,0.650512
69,4.3177884646\C,0,0.8490148568,-0.1181316522,3.6756798143\C,0,0.9489
172921,1.1190613281,4.4046391066\C,0,0.6247011681,1.1347052657,5.80243
37988\C,0,0.1845066535,-0.068326897,6.4184391212\C,0,0.0511691481,-1.2
254108538,5.6898592309\C,0,0.3752963331,-1.2771313932,4.2997112335\C,0
,0.7316372099,2.3506070173,6.532994745\C,0,1.125894311,3.5165966316,5.
9128141821\C,0,1.4244081844,3.512576343,4.527353628\C,0,1.3393290371,2
.3467667348,3.7926265328\C,0,0.1759298785,-2.580224383,3.5932242193\C,

0,-1.049046082,-3.2587866769,3.7401694991\C,0,-1.2616809766,-4.4967748
645,3.1285147199\C,0,-0.2525079731,-5.0845753094,2.3597343628\C,0,0.97
13797554,-4.4246377581,2.2092012904\C,0,1.1830426902,-3.1860401994,2.8
195205121\C,0,4.8708559331,-0.0668289229,1.3241279174\C,0,5.2186763651
,1.3665947404,0.8977101406\H,0,3.2452351586,0.0426544562,-0.5029114538
\H,0,-0.9887663666,-0.0183920856,1.9044081359\H,0,1.1224893007,0.13125
78823,-1.6654182641\H,0,5.6730648134,-0.4648744108,1.9510281917\H,0,4.
8061697725,-0.7381545938,0.4594997615\H,0,4.4970732078,-1.3877767999,3
.5743450715\H,0,2.9368696216,-0.7245395674,4.0376881859\H,0,1.55772049
32,2.3696313498,2.7281797736\H,0,-0.2847554204,-2.1378647636,6.1751549
452\H,0,1.7175653675,4.4383946524,4.0382763365\H,0,0.4867051321,2.3442
323227,7.5930222502\H,0,-0.0534256985,-0.0601120326,7.4800808651\H,0,1
.1994100073,4.4413663618,6.4789887097\H,0,-1.8433023479,-2.8056750157,
4.3295038397\H,0,2.1528604307,-2.705707734,2.7178611858\H,0,-2.2146296
964,-5.0043160111,3.2595008006\H,0,1.7693721158,-4.8824388807,1.629181
2678\H,0,-0.4127350037,-6.0530462306,1.8922268584\H,0,3.9862035535,1.5
695203995,4.2980191779\H,0,4.7024729546,0.3449569779,5.3633860783\H,0,
5.5788721939,0.8640441863,3.9194690093\H,0,6.1519433738,1.3587837764,0
.3226652615\H,0,4.4398556932,1.816482191,0.2723548514\H,0,5.3607831876
,2.0094518269,1.7721837945\C,0,-1.3213372676,0.2115632198,-0.697554466
4\C,0,-1.5232665031,1.7652421696,-0.8063608443\O,0,-1.0252839297,-0.32
06545967,-1.9530372949\C,0,-2.047313624,-0.3087257869,-2.9644994964\H,
0,-2.1475170188,0.6860716229,-3.4090935544\H,0,-1.7029898158,-1.016501
0727,-3.7206029758\H,0,-3.0088498934,-0.6396690209,-2.5591981398\C,0,-
2.4619599588,-0.5315504412,0.0028599891\C,0,-2.4004890876,-1.936829273
7,-0.022695521\C,0,-3.5366812003,0.1008736822,0.6474209843\C,0,-3.4002
134388,-2.6966968424,0.5827150739\H,0,-1.5695004464,-2.429421554,-0.52
11930382\C,0,-4.5380139617,-0.6685989707,1.2501708057\H,0,-3.614810120
6,1.1814217221,0.6855895871\C,0,-4.4740411663,-2.0630753434,1.21898655
63\H,0,-3.3392169803,-3.781298878,0.5576252197\H,0,-5.3700148555,-0.16
9815535,1.7402277219\H,0,-5.2575301247,-2.6545975429,1.6859200547\F,0,
-1.5176288048,2.3396859779,0.4094732739\F,0,-2.6793092586,2.0691159228
,-1.4079843585\F,0,-0.5269610215,2.3031582049,-1.521763815\\Version=AM
64L-G03RevD.01\State=1-A\HF=-1788.1808831\MP2=-1794.2172517\RMSD=5.979
e-09\Thermal=0.\PG=C01 [X(C34H32F3N2O1)]\\@

354
see reference 29

354-MOSC_re
see reference 29

354-MOSC_si
see reference 29

355
see reference 29

355-MOSC_re
see reference 29

355-MOSC_si
see reference 29

356
see reference 29

356-MOSC_re
see reference 29

356-MOSC_si
see reference 29

357
see reference 29

357-MOSC_re
see reference 29

357-MOSC_si
see reference 29

358
1\1\GINC-YIN\SP\RHF\6-31G(d)\C5H4Br2N2\RAMAN\10-Sep-2011\0\\#P RHF/6-3
1G(d) scf=tight int=finegrid SCRF=(PCM,Read,Solvent=Chloroform) geom=c
heck guess=read\\PCM of dibromampy1 b98/6-31G(d) structures on 4p 900
MB\\0,1\C,0,0.3093468408,-1.111174296,-0.0187258871\C,0,2.2882812981,0
.0279143217,-0.0237752778\C,0,1.6354164888,1.2537502525,0.0415027478\C
,0,0.2234510639,1.3270028139,0.0750369511\C,0,-0.422480566,0.069266613
2,0.0463716928\H,0,-0.2110475402,-2.0665064606,-0.0395770918\H,0,3.375
6682498,-0.0020650094,-0.0480791397\N,0,1.6471080722,-1.1469087065,-0.
0545040063\N,0,-0.4553386213,2.5062970073,0.0957438811\H,0,-1.43819819
44,2.4821510929,0.3279439125\H,0,0.0600239287,3.3445361708,0.324300320
3\Br,0,2.6561248631,2.8681188746,0.0949341014\Br,0,-2.3309201837,-0.00
23741144,0.1066883058\\Version=AM64L-G03RevD.01\State=1-A\HF=-5440.342
5733\RMSD=2.595e-09\Thermal=0.\Dipole=-0.5137373,0.894032,0.3426514\PG
=C01 [X(C5H4Br2N2)]\\@

358-Ac^{+}
1\1\GINC-EDDY\SP\RHF\6-31G(d)\C7H7Br2N2O1(1+)\RAMAN\26-Sep-2011\0\\#P
RHF/6-31G(d) scf=tight int=finegrid SCRF=(PCM,Read,Solvent=Chloroform)
geom=check guess=read\\PCM of dibromampy1.ac1 b98/6-31G(d) structures
on 2p 512 MB\\1,1\C,0,1.1525563548,1.8378910508,0.0109203456\C,0,-1.2
144663902,1.8100225998,0.0149251405\C,0,-1.2286414316,0.4395789813,0.0
236466386\C,0,-0.004862494,-0.3073656339,0.0264285048\C,0,1.1999190855
,0.4700120248,0.019541148\H,0,2.0376937876,2.4637588909,0.0055091617\H
,0,-2.1339070002,2.3801540174,0.0127897803\N,0,-0.0398060788,2.5061870
303,0.0086071057\N,0,0.0118609352,-1.6348673519,0.0348213771\H,0,0.892
4225488,-2.1368784465,0.0365219933\H,0,-0.853337508,-2.1624344032,0.03
94809137\Br,0,-2.8804926762,-0.4719655807,0.032062694\Br,0,2.871175423
5,-0.4036032871,0.0223707041\C,0,0.0235727948,4.0065918597,-0.00123162
04\C,0,-1.2918887159,4.737309213,-0.0037300138\H,0,-1.8849702141,4.493
8242765,-0.8937959583\H,0,-1.8832192617,4.5033969606,0.8900575181\H,0,
-1.0663898886,5.8053662236,-0.0096587019\O,0,1.111830649,4.5004516645,
-0.006458291\\Version=AM64L-G03RevD.01\State=1-A\HF=-5592.5348722\RMSD
=5.540e-09\Thermal=0.\Dipole=-1.2486958,0.4969376,-0.0010374\PG=C01 [X
(C7H7Br2N2O1)]\\@

359
1\1\GINC-CALYPSO\SP\RHF\6-31G(d)\C7H8N2O1\RAMAN\12-Sep-2011\0\\#P RHF/
6-31G(d) scf=tight int=finegrid SCRF=(PCM,Read,Solvent=Chloroform) geo
m=check guess=read\\PCM of aminoacyl2 b98/6-31G(d) structures on 4p 90
0 MB\\0,1\C,0,-2.1032956673,1.0718143317,0.0597908946\C,0,-0.725269585
5,1.299401752,0.0591570363\C,0,0.1232487756,0.1822596818,-0.0098100876
\C,0,-0.4716529391,-1.0900500084,-0.0742882633\C,0,-1.8611842791,-1.18

55944406,-0.0664088257\H,0,-2.7795882082,1.9247334019,0.1128920726\H,0
,-0.3190871482,2.3004951936,0.1097614693\H,0,0.1383158813,-1.990390501
4,-0.1294128307\H,0,-2.3347985471,-2.1659033368,-0.1158122522\N,0,1.52
42441104,0.2570758294,-0.0193910661\N,0,-2.6880425591,-0.1326538161,-0
.0007493532\C,0,2.3337964331,1.3803876946,0.0502129801\H,0,1.999202949
6,-0.634720896,-0.068885641\O,0,1.9080835506,2.5201405974,0.1121783937
\C,0,3.8258275178,1.0637660315,0.0799621129\H,0,4.1523513992,0.9495656
54,1.1217088905\H,0,4.0854496056,0.1486571456,-0.464979665\H,0,4.36542
33803,1.9093156758,-0.3530206851\\Version=AM64L-G03RevD.01\State=1-A\H
F=-453.5196496\RMSD=4.633e-09\Thermal=0.\Dipole=1.8893453,-1.4077172,-
0.0459153\PG=C01 [X(C7H8N2O1)]\\@

359-Ac⁺

1\1\GINC-CALYPSO\SP\RHF\6-31G(d)\C9H11N2O2(1+)\RAMAN\13-Sep-2011\0\\#P
RHF/6-31G(d) scf=tight int=finegrid SCRF=(PCM,Read,Solvent=Chloroform
) geom=check guess=read\\PCM of aminoacyl2.ac1 b98/6-31G(d) structures
on 2p 512 MB\\1,1\C,0,-0.0528977113,0.017724786,0.0413827284\C,0,-0.0
025029857,0.010014183,1.4131973484\C,0,1.2625671622,-0.0363442154,2.06
3371866\C,0,2.4211626538,-0.0726277616,1.2388660686\C,0,2.3010065676,-
0.0625616886,-0.1252638555\H,0,-0.9996242597,0.0525445648,-0.482453383
\H,0,-0.9163202916,0.0389601642,1.9909855208\H,0,3.4156652974,-0.10873
09175,1.6744334967\H,0,3.1459337027,-0.0886944556,-0.8042920766\N,0,1.
4182127471,-0.0476257834,3.4178765695\N,0,1.0752784971,-0.0177538944,-
0.7265835705\C,0,0.400123383,-0.0163940568,4.4221065116\H,0,2.37367750
13,-0.0820119535,3.7580319163\O,0,-0.7703393886,0.0254321918,4.1269434
649\C,0,0.9490409736,-0.0411741147,5.8302887134\H,0,1.5368377982,-0.95
14792692,6.0052252804\H,0,1.5980212131,0.8250780306,6.0125597785\H,0,0
.1107890181,-0.0151473324,6.5283294558\C,0,1.0521921721,-0.0104522161,
-2.2271603648\O,0,2.1098234782,-0.0445947366,-2.7838982842\C,0,-0.3006
036256,0.0391668366,-2.8839115231\H,0,-0.8479784443,0.9486691674,-2.60
76182761\H,0,-0.1350972329,0.0384342724,-3.9628845462\H,0,-0.909254427
3,-0.8331046406,-2.6158501244\\Version=AM64L-G03RevD.01\State=1-A\HF=-
605.7218443\RMSD=4.030e-09\Thermal=0.\Dipole=0.6758229,-0.0237984,0.10
43992\PG=C01 [X(C9H11N2O2)]\\@

361

1\1\GINC-NODE6\SP\RMP2-FC\6-31+G(2d,p)\C11H17N1\ZIP01\01-Oct-2008\0\\#
P MP2/6-31+G(2d,p) scf=tight int=finegrid\\Single point of cat12_004_s
p MP2/6-31+G(2d,p)\\0,1\C,0,1.134973,-1.820328,-0.422195\C,0,1.210098,
-0.435327,-0.214742\C,0,0.,0.285058,-0.104334\C,0,-1.210099,-0.435325,
-0.214746\C,0,-1.134974,-1.820325,-0.422199\N,0,-0.000001,-2.517847,-0
.526555\H,0,2.057729,-2.396585,-0.514461\H,0,-2.057729,-2.396582,-0.51
4462\C,0,-2.582164,0.20502,-0.081828\H,0,-2.587484,1.20942,-0.522668\H
,0,-3.301567,-0.385897,-0.664582\C,0,0.000006,1.796236,0.073556\H,0,-0
.87481,2.104293,0.657791\H,0,0.874851,2.104284,0.657754\C,0,2.582162,0
.205022,-0.081831\H,0,3.301564,-0.38589,-0.664593\H,0,2.587473,1.20942
4,-0.522668\C,0,-3.072881,0.27961,1.379865\H,0,-2.393509,0.876005,2.00
1476\H,0,-4.070317,0.733701,1.433372\H,0,-3.128899,-0.723022,1.820734\
C,0,-0.000009,2.553008,-1.272911\H,0,0.000001,3.637614,-1.107726\H,0,0
.884017,2.298961,-1.870319\H,0,-0.884055,2.298973,-1.870293\C,0,3.0728
86,0.279611,1.379859\H,0,2.39351,0.875995,2.001477\H,0,3.128919,-0.723
023,1.820723\H,0,4.070317,0.733715,1.433361\\Version=AM64L-G03RevD.01\
State=1-A\HF=-480.9513338\MP2=-482.7582923\RMSD=2.251e-09\Thermal=0.\P
G=C01 [X(C11H17N1)]\\@

361-Ac⁺

1\1\GINC-NODE6\SP\RMP2-FC\6-31+G(2d,p)\C13H20N1O1(1+)\ZIP01\02-Oct-200
8\0\\#P MP2/6-31+G(2d,p) scf=tight int=finegrid\\Single point of cat12

ac_41_sp MP2/6-31+G(2d,p)\\1,1\C,0,-0.865391,-1.246752,-0.121928\C,0,0
.521205,-1.217493,-0.108309\C,0,1.176373,0.04436,-0.100175\C,0,0.38703
9,1.227866,-0.103778\C,0,-0.994393,1.105317,-0.119214\N,0,-1.603528,-0
.108084,-0.129544\H,0,-1.443474,-2.164589,-0.133301\H,0,-1.639133,1.97
5326,-0.127529\C,0,0.974535,2.628607,-0.050502\H,0,1.897857,2.669612,-
0.637699\H,0,0.276322,3.322521,-0.535061\C,0,2.684955,0.127497,-0.1517
89\H,0,3.028646,1.021019,0.378123\H,0,3.123568,-0.729874,0.36733\C,0,1
.257165,-2.54594,-0.062322\H,0,0.634859,-3.308158,-0.546625\H,0,2.1784
17,-2.484795,-0.651442\C,0,1.243988,3.106231,1.393936\H,0,1.952352,2.4
51693,1.91499\H,0,1.665024,4.116986,1.378849\H,0,0.318047,3.132436,1.9
8043\C,0,3.20664,0.164304,-1.612141\H,0,4.299851,0.224279,-1.606781\H,
0,2.916076,-0.736034,-2.164997\H,0,2.819078,1.033198,-2.15591\C,0,1.57
8985,-2.995717,1.380507\H,0,2.213406,-2.269955,1.90294\H,0,0.661848,-3
.124692,1.966934\H,0,2.106938,-3.954868,1.361052\C,0,-3.10498,-0.26784
4,-0.157894\O,0,-3.529098,-1.384366,-0.16366\C,0,-3.922115,0.995288,-0
.175563\H,0,-4.972406,0.69862,-0.197626\H,0,-3.740826,1.602158,0.71977
1\H,0,-3.703015,1.602284,-1.062323\\Version=AM64L-G03RevD.01\State=1-A
\HF=-633.1189622\MP2=-635.4027988\RMSD=7.565e-09\Thermal=0.\PG=C01 [X(
C13H20N1O1)]\\@

362

1\1\GINC-CIP-D-04\SP\RHF\6-31G(d)\C14H23N1\C2253\17-Sep-2010\0\\#P RHF
/6-31G(d) scf=tight int=finegrid SCRF=(PCM,Read,Solvent=Chloroform) ge
om=check guess=read\\PCM of cat13_001 b98/6-31G(d) structures on 4p 90
0 MB\\0,1\C,0,1.65375,-1.59689,-1.03859\C,0,1.42232,-0.31431,-0.5192\C
,0,0.08272,0.07033,-0.2807\C,0,-0.93606,-0.86431,-0.57125\C,0,-0.55616
,-2.11401,-1.08284\N,0,0.70246,-2.49209,-1.32199\H,0,2.67844,-1.91216,
-1.24293\H,0,-1.32721,-2.85002,-1.31943\C,0,-2.41126,-0.60374,-0.31941
\H,0,-2.6675,0.44423,-0.52385\H,0,-3.00144,-1.20324,-1.02711\C,0,-0.25
738,1.46956,0.20937\H,0,-1.16116,1.4437,0.83078\H,0,0.54085,1.84968,0.
85811\C,0,2.61309,0.58254,-0.22218\H,0,3.44909,0.27929,-0.86852\H,0,2.
3842,1.62182,-0.49077\C,0,-2.85373,-0.96662,1.11736\H,0,-2.25646,-0.39
324,1.84089\H,0,-2.62046,-2.02475,1.30146\C,0,-0.4759,2.4709,-0.95033\
H,0,0.42714,2.50182,-1.57613\H,0,-1.28155,2.10187,-1.60045\C,0,3.09656
,0.54005,1.2509\H,0,2.26818,0.81925,1.91744\H,0,3.87021,1.31114,1.3770
9\C,0,-4.34794,-0.71051,1.35795\H,0,-4.96691,-1.29697,0.66604\H,0,-4.6
3832,-0.98367,2.37991\H,0,-4.59835,0.34871,1.21065\C,0,-0.81327,3.8830
4,-0.45208\H,0,-0.96385,4.57358,-1.29095\H,0,-1.73136,3.88267,0.15042\
H,0,-0.00584,4.28661,0.17324\C,0,3.66002,-0.82044,1.68402\H,0,2.90433,
-1.61138,1.6082\H,0,4.51376,-1.11235,1.05744\H,0,4.00645,-0.7855,2.724
35\\Version=IA32L-G03RevE.01\State=1-A\HF=-598.0069273\RMSD=7.826e-09\
Thermal=0.\Dipole=-0.3057879,1.2259483,0.5131618\PG=C01 [X(C14H23N1)]\
\@

362-Ac^{+}

1\1\GINC-NODE24\FOpt\RB98\6-31G(d)\C16H26N1O1(1+)\ZIP01\05-Oct-2008\0\
\#p b98/6-31G(d) opt freq=noraman\\optimization + freq of cat13_005_ac
b98/6-31G(d) on 4p 256 MB\\1,1\C,-0.0068609952,-0.0056521682,0.009390
0237\C,0.0081677192,-0.0000387993,1.3960791674\C,1.263452367,0.0017631
466,2.0650744256\C,2.4549388956,-0.0043407247,1.2867179485\C,2.3460626
593,-0.011552144,-0.0959380487\N,1.1390168524,-0.0130319495,-0.7183441
698\H,-0.9201973099,-0.0100963975,-0.575285633\H,3.2238207349,-0.02013
43364,-0.7297645164\C,3.8484845719,0.0426215926,1.8868745851\H,3.87776
0721,-0.5325767729,2.8189175598\H,4.5487903085,-0.4538935072,1.2021276
019\C,1.3319504545,-0.0560017182,3.5718715493\H,2.2251502718,0.4675656
738,3.9290541095\H,0.4716596675,0.4626981883,4.0075285886\C,-1.3269111
64,0.0480838449,2.1160573414\H,-2.0850223128,-0.4377987493,1.488307411
5\H,-1.2783961076,-0.5359372505,3.0421252242\C,4.3419174684,1.48835216

9,2.1480853107\H,3.6320980495,2.0076702661,2.8070878716\H,4.3404465543
,2.0430172449,1.1989134986\C,1.3589629732,-1.5216347201,4.0968012963\H
,0.4631142062,-2.0506724037,3.7443689899\H,2.219110736,-2.0487685985,3
.6618112316\C,-1.7882933787,1.4939913122,2.4303261973\H,-1.8643903953,
2.0560219967,1.4888960192\H,-1.0234215913,2.0046403327,3.0325885326\C,
5.7457656495,1.5129737002,2.7674582625\H,6.4787173559,1.0246529512,2.1
131615391\H,6.0772922208,2.5441637106,2.930971289\H,5.763471073,0.9977
989401,3.7359815284\C,1.4309951233,-1.5727754313,5.628854061\H,1.44709
27039,-2.6116605601,5.9763580489\H,2.3367435542,-1.0794155515,6.002644
2885\H,0.564391832,-1.0791883519,6.0856918325\C,-3.1351428742,1.517935
2391,3.1653894022\H,-3.9224529146,1.0409489061,2.5685707506\H,-3.07463
9081,0.991176305,4.1260419301\H,-3.4461879824,2.5487056187,3.367399087
9\C,0.9959906993,-0.0301619378,-2.2197417707\O,-0.1153281583,-0.030472
1255,-2.6583343511\C,2.2680507214,-0.0446968135,-3.0239322251\H,1.9826
065931,-0.0531477965,-4.077493052\H,2.8779758889,0.8448111724,-2.82546
81713\H,2.8678330143,-0.9371957303,-2.8084257636\\Version=AM64L-G03Rev
D.01\State=1-A\HF=-754.8468938\RMSD=4.578e-09\RMSF=1.766e-06\Thermal=0
.\Dipole=0.8867141,-0.1966967,-1.0450267\PG=C01 [X(C16H26N1O1)]\\@

363

1\1\GINC-YANG\SP\RHF\6-31G(d)\C7H9Br1N2\RAMAN\10-Sep-2011\0\\#P RHF/6-
31G(d) scf=tight int=finegrid SCRF=(PCM,Read,Solvent=Chloroform) geom=
check guess=read\\PCM of bromdmap1 b98/6-31G(d) structures on 4p 900 M
B\\0,1\C,0,-2.0539990716,1.0898688674,-0.0714288661\C,0,-0.6813817438,
1.2122067696,-0.2891256483\C,0,0.1777064296,0.1021059188,-0.1101851015
\C,0,-0.4756918955,-1.096443711,0.2484783183\C,0,-1.8582103323,-1.1200
716665,0.4231088682\H,0,-2.6939863782,1.9590152705,-0.212909102\H,0,0.
0958078595,-2.0052464914,0.4086273178\H,0,-2.3505870947,-2.0504004468,
0.7048862422\N,0,1.560027045,0.1698783268,-0.328813932\N,0,-2.65371755
47,-0.0525178027,0.2831930442\C,0,2.2541451037,-1.0859065161,-0.581877
141\H,0,1.7037163687,-1.6771448033,-1.32021995\H,0,2.4049151241,-1.697
9038938,0.326327469\H,0,3.2418910732,-0.8532162733,-0.9959152998\C,0,2
.3410912004,1.0630284921,0.5337283206\H,0,2.550218092,0.5959604664,1.5
119267543\H,0,3.2952981555,1.288196307,0.04335443\H,0,1.8133496347,2.0
028999672,0.6973083334\Br,0,-0.0454780754,2.8950203789,-0.9304126375\\
Version=AM64L-G03RevD.01\State=1-A\HF=-2949.0801766\RMSD=5.345e-09\The
rmal=0.\Dipole=1.3377634,-0.8297486,0.3263722\PG=C01 [X(C7H9Br1N2)]\\@

363-Ac^{+}

1\1\GINC-AZAZEL\SP\RHF\6-31G(d)\C9H12Br1N2O1(1+)\RAMAN\13-Sep-2011\0\\
#P RHF/6-31G(d) scf=tight int=finegrid SCRF=(PCM,Read,Solvent=Chlorofo
rm) geom=check guess=read\\PCM of bromdmap1.ac2 b98/6-31G(d) structure
s on 2p 512 MB\\1,1\C,0,0.0154340548,-0.1299341124,0.0143846105\C,0,0.
0339644365,-0.1683384111,1.3841869603\C,0,1.2875336895,-0.1007349035,2
.1041332347\C,0,2.4375697482,0.120737516,1.2677976834\C,0,2.351451616,
0.1491762082,-0.0940123335\H,0,-0.906287081,-0.1708160403,-0.554577083
1\H,0,3.4223588141,0.2092681153,1.7085833976\H,0,3.2310205919,0.267542
6053,-0.713463333\N,0,1.4367853567,-0.2100894016,3.4328958986\N,0,1.15
30633554,0.0002505637,-0.7343806268\C,0,2.682175997,0.225862758,4.0841
913892\H,0,3.055322624,1.14736059,3.6308518171\H,0,3.4535823098,-0.554
5165079,4.0419624562\H,0,2.4555416397,0.4362946994,5.1320350296\C,0,0.
5132387984,-0.925597555,4.333865293\H,0,1.1285566246,-1.4873369225,5.0
436322808\H,0,-0.1261323912,-0.2279314987,4.8844034727\H,0,-0.10283784
49,-1.6334378655,3.7828004326\Br,0,-1.672028847,-0.1251322807,2.212083
7584\C,0,0.9972213994,0.0073615737,-2.2138107026\O,0,-0.1093471829,-0.
1282537047,-2.651260055\C,0,2.2539113752,0.1845000251,-3.0255951187\H,
0,2.9739766876,-0.6202129999,-2.8329548166\H,0,1.9646322531,0.15706542
99,-4.0776923616\H,0,2.7364361611,1.1465223017,-2.8139076058\\Version=

AM64L-G03RevD.01\State=1-A\HF=-3101.2816444\RMSD=2.476e-09\Thermal=0.\
Dipole=3.3629843,0.1170395,0.150003\PG=C01 [X(C9H12Br1N2O1)]\\@

364

1\1\GINC-NODE18\SP\RHF\6-31G(d)\C8H12N2\ZIP08\08-Oct-2010\0\\#P RHF/6-
31G(d) scf=tight int=finegrid SCRF=(PCM,Read,Solvent=Chloroform) geom=
check guess=read\\PCM of t1ap1 b98/6-31G(d) structures on 4p 900 MB\\0
,1\C,0,0.0148716477,0.1950482337,-0.040686672\C,0,0.0276310255,0.17160
45998,1.3591523849\C,0,1.2603427351,-0.1358611084,1.9918796593\C,0,2.3
577291644,-0.4211298493,1.1585168693\C,0,2.2061566309,-0.3649882205,-0
.2270354076\H,0,-0.9260226934,0.4130535783,-0.5495854253\H,0,3.3280646
075,-0.669195351,1.5772368502\H,0,3.0580134775,-0.5752943205,-0.874222
7242\N,0,1.059547441,-0.0543107565,-0.8415899695\N,0,1.346771944,-0.20
9912091,3.4015677925\C,0,-1.2598679779,0.3861441687,2.1213012149\H,0,-
2.119951939,0.124752361,1.4941234577\H,0,-1.2834610865,-0.2369843312,3
.0231533683\H,0,-1.3997195548,1.4302674769,2.4363206698\C,0,1.14440442
82,1.0420558768,4.1332657862\H,0,0.2990463647,1.5993244162,3.726656796
9\H,0,0.932867805,0.816104505,5.185555315\H,0,2.0363678363,1.693064744
8,4.0904630148\C,0,2.4519239245,-0.9802346685,3.9568322756\H,0,2.24838
90709,-1.1623571736,5.0188276304\H,0,2.5247344645,-1.9476040775,3.4500
5655\H,0,3.4292663531,-0.4675108241,3.885098374\\Version=AM64L-G03RevD
.01\State=1-A\HF=-418.8116705\RMSD=4.043e-09\Thermal=0.\Dipole=0.28120
44,0.1383609,1.5239847\PG=C01 [X(C8H12N2)]\\@

364-Ac$^+$

1\1\GINC-NODE18\SP\RHF\6-31G(d)\C8H12N2\ZIP08\08-Oct-2010\0\\#P RHF/6-
31G(d) scf=tight int=finegrid SCRF=(PCM,Read,Solvent=Chloroform) geom=
check guess=read\\PCM of t1ap1 b98/6-31G(d) structures on 4p 900 MB\\0
,1\C,0,0.0148716477,0.1950482337,-0.040686672\C,0,0.0276310255,0.17160
45998,1.3591523849\C,0,1.2603427351,-0.1358611084,1.9918796593\C,0,2.3
577291644,-0.4211298493,1.1585168693\C,0,2.2061566309,-0.3649882205,-0
.2270354076\H,0,-0.9260226934,0.4130535783,-0.5495854253\H,0,3.3280646
075,-0.669195351,1.5772368502\H,0,3.0580134775,-0.5752943205,-0.874222
7242\N,0,1.059547441,-0.0543107565,-0.8415899695\N,0,1.346771944,-0.20
9912091,3.4015677925\C,0,-1.2598679779,0.3861441687,2.1213012149\H,0,-
2.119951939,0.124752361,1.4941234577\H,0,-1.2834610865,-0.2369843312,3
.0231533683\H,0,-1.3997195548,1.4302674769,2.4363206698\C,0,1.14440442
82,1.0420558768,4.1332657862\H,0,0.2990463647,1.5993244162,3.726656796
9\H,0,0.932867805,0.816104505,5.185555315\H,0,2.0363678363,1.693064744
8,4.0904630148\C,0,2.4519239245,-0.9802346685,3.9568322756\H,0,2.24838
90709,-1.1623571736,5.0188276304\H,0,2.5247344645,-1.9476040775,3.4500
5655\H,0,3.4292663531,-0.4675108241,3.885098374\\Version=AM64L-G03RevD
.01\State=1-A\HF=-418.8116705\RMSD=4.043e-09\Thermal=0.\Dipole=0.28120
44,0.1383609,1.5239847\PG=C01 [X(C8H12N2)]\\@

365

1\1\GINC-YIN\SP\RHF\6-31G(d)\C9H12N2O1\RAMAN\25-Aug-2011\0\\#P RHF/6-3
1G(d) scf=tight int=finegrid SCRF=(PCM,Read,Solvent=Chloroform) geom=c
heck guess=read\\PCM of morpho3 b98/6-31G(d) structures on 4p 900 MB\\
0,1\C,0,-1.4923181683,1.0131889739,0.5462392754\C,0,-0.850251197,-0.03
75882483,-0.1240480662\C,0,-1.6434291625,-1.042063438,-0.687732217\C,0
,-3.0302685087,-0.9562765883,-0.5475863947\N,0,-3.6619559539,0.0420665
963,0.0896247305\C,0,-2.8880843179,1.0003209101,0.6189395257\H,0,-0.93
16755331,1.8248461753,0.9967930455\H,0,-1.1772799107,-1.8587170491,-1.
2313830967\H,0,-3.6703195646,-1.7244072297,-0.9830403262\H,0,-3.412021
8791,1.8096479876,1.1296717606\C,0,2.7431490828,0.8009118561,-0.827242
1389\C,0,1.2818862871,1.1281191767,-0.5162418457\N,0,0.5718573318,-0.1
31412296,-0.2546890466\C,0,1.2055131613,-0.8481158402,0.8649652577\C,0

,2.6735129143,-1.103037715,0.5263653202\O,0,3.3615034239,0.1120772131,
0.2530004511\H,0,3.3106909693,1.7269100856,-0.9737702639\H,0,2.8009452
932,0.19732376,-1.7487009195\H,0,0.8110824351,1.6292012404,-1.37065911
32\H,0,1.2619290954,1.8154770131,0.3505455075\H,0,1.1403795605,-0.2756
594553,1.8103343189\H,0,0.6843662208,-1.8022079803,1.014312778\H,0,3.1
920656201,-1.5777460091,1.366780986\H,0,2.7302401106,-1.7686208673,-0.
351390374\\Version=AM64L-G03RevD.01\State=1-A\HF=-531.5346842\RMSD=2.1
65e-09\Thermal=0.\Dipole=0.6386038,-0.0357399,-0.0893554\PG=C01 [X(C9H
12N2O1)]\\@

365-Ac$^+$

1\1\GINC-AZAZEL\SP\RHF\6-31G(d)\C11H15N2O2(1+)\RAMAN\26-Aug-2011\0\\#P
RHF/6-31G(d) scf=tight int=finegrid SCRF=(PCM,Read,Solvent=Chloroform
) geom=check guess=read\\PCM of morpho3.ac2 b98/6-31G(d) structures on
2p 512 MB\\1,1\C,0,-0.0092352029,0.3077183238,-0.0744867362\C,0,-0.19
32345462,-0.4164690635,1.1568243441\C,0,1.0044668055,-1.0194419245,1.6
814451703\C,0,2.2044358865,-0.8767555987,1.0506377328\N,0,2.3374670082
,-0.1633248991,-0.1133368604\C,0,1.2201700215,0.4124535512,-0.65735150
6\H,0,-0.8334814185,0.7870542016,-0.5846112494\H,0,0.9976558572,-1.608
7695425,2.5881816743\H,0,3.1240396091,-1.3152706345,1.421283724\H,0,1.
3534285817,0.958360947,-1.5825850405\C,0,-3.3755948524,0.8285405963,2.
3366458859\C,0,-2.6446721734,0.0445336758,1.2334915305\N,0,-1.38538822
71,-0.5235905436,1.7621208227\C,0,-1.6284121302,-1.3031607713,2.995786
9782\C,0,-2.4086333397,-0.4541263539,4.0136382983\O,0,-3.6188469656,0.
013494957,3.460909082\H,0,-4.3487418557,1.1582520962,1.9609818547\H,0,
-2.7835921493,1.7162367269,2.6167509336\H,0,-2.4534588605,0.70238825,0
.3869189327\H,0,-3.2733369424,-0.7889629024,0.8932064729\H,0,-2.218503
5485,-2.1880061198,2.7226901022\H,0,-0.6921517347,-1.6359293852,3.4411
289891\H,0,-2.6671849676,-1.0732740125,4.8776739186\H,0,-1.7825989209,
0.3877027868,4.3545002048\C,0,3.6874519181,-0.0703131774,-0.7110903669
\O,0,4.5864041271,-0.6175934882,-0.1361364779\C,0,3.8120839046,0.70714
61703,-1.9964836271\H,0,3.5125086205,1.754079161,-1.8645223863\H,0,4.8
617665278,0.6735545053,-2.2935093308\H,0,3.2018261707,0.2654720487,-2.
7937484951\\Version=AM64L-G03RevD.01\State=1-A\HF=-683.7530885\RMSD=5.
900e-09\Thermal=0.\Dipole=0.3095605,0.5063706,-1.5140933\PG=C01 [X(C11
H15N2O2)]\\@

366

1\1\GINC-NODE9\SP\RHF\6-31G(d)\C11H18N2\ZIP08\12-Oct-2010\0\\#P RHF/6-
31G(d) scf=tight int=finegrid SCRF=(PCM,Read,Solvent=Chloroform) geom=
check guess=read\\PCM of t2ap3 b98/6-31G(d) structures on 4p 900 MB\\0
,1\C,0,-0.0044165306,0.2257459459,-0.1206750881\C,0,0.0150292239,0.201
3643397,1.2802275876\C,0,1.2562520632,-0.0848277471,1.905761779\C,0,2.
3494066372,-0.3638131872,1.0666165473\C,0,2.1876214994,-0.3262318919,-
0.3191519506\H,0,-0.9461350389,0.4504274184,-0.6253368543\H,0,3.326207
4917,-0.5969372981,1.4793269664\H,0,3.0360039133,-0.5379027984,-0.9704
502693\N,0,1.0354997156,-0.0259802915,-0.927530285\N,0,1.3558180935,-0
.101501137,3.3247956284\C,0,1.2656153764,1.2279473254,3.9665735567\H,0
,2.2813508532,1.6379187832,4.1167375964\H,0,0.7662910379,1.9096705637,
3.271518353\C,0,2.411710757,-0.9352066002,3.9127177985\H,0,3.420916846
9,-0.6097992059,3.5967849955\H,0,2.3722553632,-0.7697801643,4.99527808
98\C,0,-1.2904129334,0.3789282945,2.0351238188\H,0,-2.0250532126,0.826
5444534,1.3520630414\H,0,-1.1822724836,1.0838106093,2.8674003754\C,0,-
1.8482742011,-0.95475063,2.5751319913\H,0,-2.7999835132,-0.7927839526,
3.0972452035\H,0,-2.0242801822,-1.6620579934,1.7557145275\H,0,-1.14179
18908,-1.4115106908,3.2765288327\C,0,0.5096146359,1.2073432593,5.30009
1251\H,0,-0.5183362369,0.8534216369,5.1603423796\H,0,0.9926738424,0.55
31890876,6.0356340893\H,0,0.4763586839,2.2171665096,5.7284281559\C,0,2

.2178153803,-2.4303976835,3.6420948057\H,0,1.2498501809,-2.7648201891,
4.0329893222\H,0,2.2492009297,-2.6599990612,2.5716057613\H,0,3.0101805
291,-3.0061432437,4.1360313513\\Version=AM64L-G03RevD.01\State=1-A\HF=
-535.9125806\RMSD=3.150e-09\Thermal=0.\Dipole=0.3409866,0.1455971,1.50
98359\PG=C01 [X(C11H18N2)]\\@

366-Ac+

1\1\GINC-NODE11\SP\RHF\6-31G(d)\C13H21N2O1(1+)\ZIP08\20-Oct-2010\0\\#P
RHF/6-31G(d) scf=tight int=finegrid SCRF=(PCM,Read,Solvent=Chloroform
) geom=check guess=read\\PCM of t2ap3.ac1 b98/6-31G(d) structures on 4
p 900 MB\\1,1\C,0,-0.0507637213,0.0730549013,0.0255215671\C,0,0.010052
9453,0.1422190682,1.3971323187\C,0,1.3184357112,0.0190098903,2.0266474
945\C,0,2.3886351788,-0.3915243928,1.1581458402\C,0,2.2354102237,-0.43
79028548,-0.1957221833\H,0,-1.0052433959,0.1603243541,-0.4787348652\H,
0,3.3747208553,-0.6019741537,1.5491086809\H,0,3.0434564593,-0.66209015
13,-0.8824120946\N,0,1.0312730906,-0.16376549,-0.7819552923\N,0,1.5653
791591,0.2477241038,3.3334298101\C,0,0.8111697576,1.2258772499,4.15518
80696\H,0,1.5595373478,1.9431121662,4.5177664322\H,0,0.1486734702,1.79
48042891,3.5021303145\C,0,2.8234853601,-0.2264276892,3.9635544201\H,0,
3.6869599304,0.1661793198,3.410896891\H,0,2.8621303475,0.237502681,4.9
52017818\C,0,-1.3343368588,0.1046639234,2.1194314742\H,0,-2.1055111038
,0.4459901715,1.4179182867\H,0,-1.3851981402,0.7908655016,2.965200578\
C,0,-1.6800896378,-1.3260812577,2.5899043176\H,0,-2.6471883628,-1.3272
990475,3.1049428987\H,0,-1.746480134,-2.0136337724,1.7389775179\H,0,-0
.9259774242,-1.7195168362,3.2813948381\C,0,0.063883878,0.6158414951,5.
3448018396\H,0,-0.7110280115,-0.0888154955,5.027477556\H,0,0.747718643
4,0.088240233,6.0195919943\H,0,-0.413255738,1.417343728,5.9200552483\C
,0,2.8873940752,-1.7506604895,4.1152067305\H,0,2.0575953815,-2.1120580
426,4.7325226232\H,0,2.8467882718,-2.2706896264,3.1517305647\H,0,3.826
3421198,-2.0274455718,4.6075797364\C,0,0.9666574752,-0.1809206679,-2.2
581103754\O,0,1.9697653995,-0.4581180864,-2.8553034209\C,0,-0.35954543
05,0.1603238153,-2.889294235\H,0,-0.7137005655,1.1497502721,-2.5768417
83\H,0,-0.2133107221,0.1557177822,-3.9708554105\H,0,-1.1257905385,-0.5
82204731,-2.6336135647\\Version=AM64L-G03RevD.01\State=1-A\HF=-688.122
919\RMSD=7.646e-09\Thermal=0.\Dipole=-0.5909807,0.6488772,0.2219441\PG
=C01 [X(C13H21N2O1)]\\@

367

1\1\GINC-NODE23\SP\RHF\6-31G(d)\C14H24N2\ZIP08\09-Mar-2011\0\\#P RHF/6
-31G(d) scf=tight int=finegrid SCRF=(PCM,Read,Solvent=Chloroform) geom
=check guess=read\\PCM of t3ap42 b98/6-31G(d) structures on 4p 900 MB\
\0,1\C,0,-0.0121746326,0.0051179719,-0.0746357129\C,0,0.0007347421,0.0
029881018,1.328132416\C,0,1.2660729066,0.0907060312,1.9615265564\C,0,2
.3942856607,0.2427893486,1.1338359932\C,0,2.2384385693,0.2608266374,-0
.2512647706\H,0,-0.9679789945,-0.0846016315,-0.5933475666\H,0,3.388969
0018,0.3313316678,1.5612394493\H,0,3.1101595405,0.3720703682,-0.896526
7557\N,0,1.0586365217,0.1262504225,-0.8698945072\N,0,1.361770588,0.035
661431,3.3792461954\C,0,-1.3097587364,-0.0409515236,2.0988586148\H,0,-
1.0950235826,0.0363025131,3.1702747824\H,0,-1.8032205229,-1.0119071218
,1.9391365068\C,0,1.1210373119,-1.2949011617,3.966707646\H,0,0.2088231
15,-1.7066506803,3.5187160865\H,0,0.9108006696,-1.1510009093,5.0369021
721\C,0,2.4434021475,0.7892360329,4.021062512\H,0,2.3761128135,0.57073
18666,5.0957840824\H,0,3.4515204506,0.4581522057,3.7086684795\C,0,-2.2
991255576,1.0888353979,1.7243671828\H,0,-3.2407901472,0.9047725119,2.2
612458868\H,0,-2.5423977742,1.0381589149,0.6540649256\C,0,-1.778462104
3,2.4913353223,2.0685707767\H,0,-1.5602108835,2.5767559962,3.141572913
\H,0,-0.8549615088,2.7174562904,1.5215088966\H,0,-2.5183381871,3.25996
68783,1.8128760749\C,0,2.2631453482,-2.3170539277,3.7970798601\H,0,2.4

870490912,-2.4300638154,2.7271485387\H,0,3.1773902166,-1.9352883592,4.
272960922\C,0,1.9004868802,-3.681139391,4.4023247245\H,0,1.0086500247,
-4.1036071302,3.9206799052\H,0,2.7186426161,-4.4006256892,4.2783788617
\H,0,1.6898843477,-3.5972701159,5.4770250855\H,0,2.3348855752,2.536633
6311,2.7428794073\H,0,1.3188432151,2.6162294966,4.1779070771\C,0,2.308
1993976,2.3054875232,3.8159346227\C,0,3.4128072141,3.0882770691,4.5397
65717\H,0,3.3908268634,2.9043470145,5.6223786421\H,0,4.4088058583,2.80
43369158,4.1744424676\H,0,3.2968561024,4.1672572863,4.3830358485\\Vers
ion=AM64L-G03RevD.01\State=1-A\HF=-653.0147349\RMSD=2.643e-09\Thermal=
0.\Dipole=0.2667751,-0.1955121,1.4407918\PG=C01 [X(C14H24N2)]\\@

367-Ac$^+$

1\1\GINC-NODE22\SP\RHF\6-31G(d)\C16H27N2O1(1+)\ZIP08\24-Jun-2011\0\\#P
RHF/6-31G(d) scf=tight int=finegrid SCRF=(PCM,Read,Solvent=Chloroform
) geom=check guess=read\\PCM of t3ap184.ac1 b98/6-31G(d) structures on
4p 900 MB\\1,1\C,0,-0.0299043111,-0.0025714613,0.0434480118\C,0,0.002
5702217,0.0275694968,1.4184133748\C,0,1.3050716883,-0.0538243584,2.070
0887918\C,0,2.4241840505,-0.2756914615,1.1915253988\C,0,2.2944506745,-
0.2927849799,-0.16379574\H,0,-0.9805232708,0.0447858837,-0.4724250074\
H,0,3.4269025828,-0.3660024325,1.5863879124\H,0,3.1304468457,-0.404805
868,-0.843974854\N,0,1.0733501083,-0.1330514777,-0.757997316\N,0,1.517
9561256,0.0445386605,3.3993145926\C,0,-1.3553664668,-0.0306016518,2.10
60598811\H,0,-2.123502798,0.1701969606,1.3479252537\H,0,-1.4738025143,
0.7646925591,2.8487943849\C,0,2.8022242444,-0.3936371085,3.9989275238\
H,0,3.2037881298,-1.2202716358,3.405080752\H,0,2.5491090027,-0.8233978
118,4.9752431316\C,0,0.6222587845,0.7636672231,4.3381564375\H,0,-0.347
62469,0.2623206863,4.4000858153\H,0,1.0784298961,0.6566002826,5.326468
607\C,0,-1.6552842341,-1.4095477277,2.7434626878\H,0,-0.8694932457,-1.
6732318856,3.4649485744\H,0,-1.6153117079,-2.1761095199,1.9575972785\C
,0,-3.0268257822,-1.4351399676,3.4325638739\H,0,-3.0804579345,-0.70078
37467,4.2469257934\H,0,-3.8327609991,-1.2075048238,2.7237343767\H,0,-3
.2274441096,-2.4234088143,3.8604221138\C,0,3.8473594058,0.7220031001,4
.1776321581\H,0,4.0594548407,1.193588971,3.2081539735\H,0,3.4321601606
,1.5089502483,4.8212486871\C,0,5.1430647184,0.1763620741,4.7971678302\
H,0,5.6017725486,-0.5900532015,4.159663806\H,0,5.8737883292,0.98097089
53,4.9299627819\H,0,4.9569160362,-0.2714715248,5.7811850825\H,0,1.4507
157971,2.7364921991,4.034767317\H,0,0.0708193068,2.3906731615,3.000879
97\C,0,0.4615626356,2.260662817,4.0191758329\C,0,-0.4703026754,2.94628
36378,5.0295362773\H,0,-1.478325504,2.5119616519,5.008311296\H,0,-0.08
69596491,2.8541148957,6.0533275272\H,0,-0.5628808062,4.0137887095,4.80
36653526\C,0,1.0224042858,-0.1293061827,-2.233005186\O,0,2.0544546758,
-0.2831581174,-2.8261881996\C,0,-0.328275754,0.072248378,-2.873017595\
H,0,-0.7836788888,1.0210658215,-2.5654341845\H,0,-0.17501303,0.0823778
905,-3.9535518007\H,0,-1.0161602102,-0.7440488865,-2.6201610957\\Versi
on=AM64L-G03RevD.01\State=1-A\HF=-805.2273932\RMSD=5.715e-09\Thermal=0
.\Dipole=-0.6001644,-0.0492618,-0.5628289\PG=C01 [X(C16H27N2O1)]\\@

368

1\1\GINC-NAUTILUS\SP\RHF\6-31G(d)\C23H24N2\EVGENY\15-Dec-2009\0\\#P RH
F/6-31G(d) scf=tight int=finegrid SCRF=(PCM,Read,Solvent=Chloroform) g
eom=check guess=read\\PCM of para1.3 b98/6-31G(d) structures on 4p 900
MB\\0,1\C,0,-0.0027963863,-0.062917945,0.1936931207\C,0,0.0090609333,
0.0347804263,1.5960095916\C,0,1.2649595827,0.074124513,2.2134037329\C,
0,2.4037903534,0.3571970899,1.454288399\C,0,2.3327118018,0.5871144079,
0.0732887371\C,0,1.1398531929,0.1873566005,-0.5874456364\H,0,-0.948852
5695,-0.2395563323,-0.3170172216\H,0,1.3402522987,0.0482429959,3.29963
34073\H,0,3.3405047338,0.560226968,1.972052925\C,0,3.3824858255,1.4841
714867,-0.5638820277\H,0,3.6229929518,1.1703796831,-1.5841281047\H,0,4

.3073889647,1.4018334243,0.020705102\C,0,2.9607944351,3.0368807985,-0.
6054861762\H,0,3.8467911086,3.6262703514,-0.3351906785\H,0,2.708449473
3,3.2930095177,-1.6413768398\C,0,-1.2597293051,0.3552627217,2.36586069
73\H,0,-2.130900783,0.0413685577,1.7778683472\H,0,-1.2987665176,-0.197
9355869,3.3132451935\C,0,-1.4186415316,1.9217326272,2.7086394569\H,0,-
2.4580850338,2.203683746,2.4968599242\H,0,-1.2661461108,2.0532708446,3
.7869587303\C,0,-0.443471623,2.8021053128,1.948317226\C,0,0.8012757379
,3.1395204357,2.5055484866\C,0,-0.6219754999,3.0719251679,0.581997908\
C,0,1.9003424626,3.4208740891,1.6881792885\H,0,0.9526638146,3.03769937
26,3.5797069557\C,0,0.4765963912,3.3507855043,-0.2345056914\H,0,-1.600
8346675,2.9251084095,0.1258241293\C,0,1.7797977874,3.365468557,0.29083
49515\H,0,2.8842388696,3.5335470279,2.1425951456\H,0,0.3356864053,3.41
90344069,-1.3116844424\C,0,1.0220436182,0.1704759645,-2.0770131702\C,0
,-0.0228354511,0.8820169333,-2.6883600876\C,0,1.9240889168,-0.52348077
13,-2.9391804694\H,0,-0.7317193921,1.4056957249,-2.0486549563\C,0,1.73
04378704,-0.3599136424,-4.3248674691\C,0,0.6660091043,0.407798045,-4.7
954174336\H,0,2.3782244219,-0.8562518293,-5.0400105611\H,0,0.514780098
,0.5200277725,-5.8693667986\N,0,-0.2255827845,1.0160917339,-4.00599173
2\N,0,2.9795050562,-1.3001868147,-2.4333966158\C,0,4.0506535088,-1.667
0181314,-3.347847943\H,0,4.8920634811,-2.0479447903,-2.7574426279\H,0,
3.7635151223,-2.452980956,-4.0707721419\H,0,4.3936297746,-0.7877690018
,-3.9028865851\C,0,2.6534872525,-2.3889710822,-1.5084112114\H,0,2.3728
981781,-3.3061857234,-2.0558143864\H,0,3.527369473,-2.6105491254,-0.88
39102147\H,0,1.8292316622,-2.1069276938,-0.8535944567\\Version=AM64L-G
03RevD.01\State=1-A\HF=-993.7729523\RMSD=3.524e-09\Thermal=0.\Dipole=1
.1212722,-1.0938253,0.5125518\PG=C01 [X(C23H24N2)]\\@

368-Ac^{+}

1\1\GINC-NAUTILUS\SP\RHF\6-31G(d)\C25H27N2O1(1+)\EVGENY\23-Dec-2009\0\
\#P RHF/6-31G(d) scf=tight int=finegrid SCRF=(PCM,Read,Solvent=Chlorof
orm) geom=check guess=read\\PCM of para1.3.ac2 b98/6-31G(d) structures
on 4p 900 MB\\1,1\C,0,0.0039210737,-0.0272267534,0.0093391873\C,0,-0.
0005732376,-0.0023047605,1.4138530605\C,0,1.2527849005,0.0106097473,2.
0418283941\C,0,2.3983694542,0.3492812825,1.3179308677\C,0,2.3457293562
,0.650458797,-0.051156448\C,0,1.1604872594,0.2696684124,-0.7330309571\
H,0,-0.9333074355,-0.1928010849,-0.52134727\H,0,1.3179833588,-0.084472
5266,3.1239998667\H,0,3.3232893776,0.5356642485,1.8608907393\C,0,3.387
8274619,1.5970188498,-0.6291551658\H,0,3.6354241653,1.3641699473,-1.67
2396413\H,0,4.315516836,1.4803337248,-0.0571291131\C,0,2.956019833,3.1
49612463,-0.5631071791\H,0,3.8384330287,3.7169969468,-0.2432147004\H,0
,2.7173434389,3.4804479716,-1.5815070103\C,0,-1.2789832608,0.257484343
3,2.1898041273\H,0,-2.1417908739,-0.0192589916,1.5725666421\H,0,-1.322
8783853,-0.361437542,3.0940860841\C,0,-1.4445174366,1.7967189945,2.636
7342692\H,0,-2.4831894749,2.0870618955,2.4375849839\H,0,-1.2987952987,
1.8549197736,3.721342282\C,0,-0.4666846108,2.7261513962,1.9412335722\C
,0,0.7726159971,3.028606257,2.5287146092\C,0,-0.6405090292,3.096405723
9,0.5966256616\C,0,1.8776889844,3.3659304982,1.7402408886\H,0,0.914078
6514,2.8652324019,3.5959980383\C,0,0.4633248111,3.4374692479,-0.188651
438\H,0,-1.6229485189,3.008386527,0.1329626353\C,0,1.76525902,3.409087
1971,0.3425320331\H,0,2.8568643894,3.4559127827,2.208621047\H,0,0.3241
978608,3.6334696293,-1.2514548061\C,0,1.0655513594,0.3396709623,-2.223
3543882\C,0,0.0996115719,1.1595307902,-2.7571042461\C,0,1.9533562606,-
0.3527315077,-3.1458161173\H,0,-0.5967810983,1.6990775633,-2.128686022
7\C,0,1.8449283683,0.0223467317,-4.5301261193\C,0,0.8582831687,0.85537
01746,-4.9693334691\H,0,2.4983240306,-0.4118625643,-5.2759207608\H,0,0
.7415993083,1.0913947594,-6.019065104\N,0,-0.0435442036,1.404140619,-4
.1003694262\N,0,2.8390947128,-1.2979062806,-2.7847220806\C,0,3.8927350
688,-1.7386381552,-3.7090726594\H,0,4.6816156807,-2.2120798503,-3.1199

262778\H,0,3.5137500908,-2.4698008469,-4.4360534502\H,0,4.331764132,-0
.88661795,-4.2346940522\C,0,2.7372026221,-2.1218183642,-1.5681599424\H
,0,2.9077147404,-3.1625405854,-1.8649174535\H,0,3.4890880301,-1.827170
7543,-0.8286243659\H,0,1.7481800515,-2.0402142378,-1.1225349981\C,0,-1
.1361166538,2.3011233212,-4.5175260496\O,0,-1.8460954272,2.7560709328,
-3.662446562\C,0,-1.2718449962,2.5734492418,-5.9952311466\H,0,-0.38252
33415,3.076637916,-6.3940798742\H,0,-1.4342058262,1.6489627758,-6.5625
597539\H,0,-2.1358317098,3.2274827203,-6.1251978218\\Version=AM64L-G03
RevD.01\State=1-A\HF=-1145.9868935\RMSD=5.449e-09\Thermal=0.\Dipole=1.
3414933,-2.218469,-4.6156527\PG=C01 [X(C25H27N2O1)]\\@

369

1\1\GINC-NODE-21\SP\RMP2-FC\6-31+G(2d,p)\C8H12N2\ZIP08\26-Aug-2010\0\\
#P MP2/6-31+G(2d,p) scf=(direct,tight) int=finegrid geom=check guess=r
ead\\sp of d15ap1 MP2-5/6-31+G(2d,p)\\0,1\C,0,-0.0418231561,0.19619398
13,0.0658543946\C,0,-0.0022267567,0.3233263035,1.4520860249\C,0,2.2187
101404,-0.1035902541,1.5350514607\C,0,2.3102635069,-0.2561351402,0.154
6376194\C,0,1.1498375993,-0.0948668036,-0.6430280406\H,0,-0.9880555318
,0.3272683312,-0.4477027864\H,0,-0.9240994796,0.5522686955,1.988064914
9\H,0,3.1175725934,-0.2273623344,2.1401641336\H,0,3.2722776237,-0.5038
864556,-0.2808900952\N,0,1.09509811,0.1837652893,2.2096486939\N,0,1.16
54966954,-0.1967990857,-2.0192597092\C,0,-0.0921212293,-0.1780481053,-
2.7512781437\H,0,-0.6178657939,0.7768056179,-2.6140294082\H,0,0.117779
7144,-0.2940084877,-3.8179431558\H,0,-0.7699398011,-0.9897719634,-2.44
48194616\C,0,2.3656084964,-0.6163703978,-2.7389180768\H,0,3.2343970903
,-0.1091493707,-2.3038592611\H,0,2.2804049104,-0.2370194562,-3.7645241
014\C,0,2.5887829724,-2.137974394,-2.7627665999\H,0,1.7524778727,-2.64
80810525,-3.2565997232\H,0,3.5076510238,-2.3822385353,-3.3110243637\H,
0,2.6773151313,-2.5389813422,-1.7465116064\\Version=IA32L-G03RevD.01\S
tate=1-A\HF=-418.8550441\MP2=-420.3748493\RMSD=7.125e-09\Thermal=0.\PG
=C01 [X(C8H12N2)]\\@

369-Ac$^+$

1\1\GINC-NODE-25\SP\RMP2-FC\6-31+G(2d,p)\C10H15N2O1(1+)\ZIP08\29-Aug-2
010\0\\#P MP2/6-31+G(2d,p) scf=(direct,tight) int=finegrid geom=check
guess=read\\sp of d15ap1.ac1 MP2-5/6-31+G(2d,p)\\1,1\C,0,-0.0070173387
,-0.0120860116,0.038235804\C,0,-0.0315024617,0.0224955715,1.401372699\
C,0,2.3267647523,-0.0481961706,1.503345156\C,0,2.4212098315,-0.0804959
786,0.1420335216\C,0,1.239289212,-0.0584258301,-0.6770220002\H,0,-0.95
47445828,0.0138799747,-0.4844912328\H,0,-0.948423822,0.0685907549,1.97
75641139\H,0,3.2115363429,-0.0714543333,2.1263979361\H,0,3.4108027927,
-0.1437098367,-0.2918161027\N,0,1.1187649532,0.0062795983,2.1493735436
\N,0,1.2820303052,-0.0776897488,-2.0141229926\C,0,0.043102651,-0.16399
20576,-2.8026640946\H,0,-0.5292252239,0.7694201201,-2.737457951\H,0,0.
3074715452,-0.3368303397,-3.8468038781\H,0,-0.5781718288,-1.0001686338
,-2.4654459978\C,0,2.5512998994,-0.0557016578,-2.7740903822\H,0,3.2814
656312,0.5443647894,-2.2236878113\H,0,2.3487376817,0.4891079113,-3.701
5215736\C,0,3.0896369678,-1.4592198965,-3.0775079857\H,0,2.3675546454,
-2.0442028821,-3.6583107112\H,0,4.0104310553,-1.3791947989,-3.66586604
37\H,0,3.3162043071,-2.0115522055,-2.1581450868\C,0,0.9779161149,0.048
2955648,3.6228745056\O,0,-0.1329965763,0.0871996337,4.0724806403\C,0,2
.2507076494,0.0400296116,4.4306134242\H,0,2.8828407831,0.9048818742,4.
1952447883\H,0,1.964992521,0.0850560196,5.4830214704\H,0,2.8299960271,
-0.8754229754,4.2584001443\\Version=IA32L-G03RevD.01\State=1-A\HF=-571
.0387989\MP2=-573.0329019\RMSD=2.477e-09\Thermal=0.\PG=C01 [X(C10H15N2
O1)]\\@

370

1\1\GINC-NODE-28\SP\RMP2-FC\6-31+G(2d,p)\C11H18N2\ZIP08\06-Aug-2010\0\
\#P MP2/6-31+G(2d,p) scf=(direct,tight) int=finegrid geom=check guess=
read\\sp of d3ap37 MP2-5/6-31+G(2d,p)\\0,1\C,0,0.0126165156,0.06855707
5,-0.0428585818\C,0,-0.0131874721,0.0744698151,1.3489654755\C,0,1.2032
965356,0.0006147983,2.0744651705\C,0,2.3754943472,-0.0723585101,1.2793
151654\C,0,2.2687344893,-0.0648521851,-0.1086405334\H,0,-0.9308590578,
0.1236788679,-0.5873346546\H,0,-0.972197029,0.1390319505,1.8508006595\
H,0,3.3620818672,-0.1374487731,1.7244170326\H,0,3.1789228413,-0.119310
1562,-0.7071638292\N,0,1.2435020247,-0.0001804705,3.4548237235\N,0,1.1
196708635,0.0022644052,-0.7969811319\C,0,2.51456369,-0.1421447898,4.16
80604858\H,0,3.2625347451,0.5257571273,3.7189672348\H,0,2.3573622388,0
.2244110619,5.1889394693\C,0,0.0161206314,0.1409578513,4.2410006021\H,
0,0.2324577361,-0.2267734675,5.2505752541\H,0,-0.756763955,-0.52643054
87,3.8354388766\C,0,3.0646698433,-1.5830378251,4.224928934\H,0,3.23970
81704,-1.9471866859,3.2042080991\H,0,4.0476503121,-1.5439950116,4.7173
897966\C,0,-0.5296562339,1.5817804843,4.3314451228\H,0,-1.4822981521,1
.5421683905,4.880259118\H,0,-0.7638082207,1.9470873,3.3230611482\C,0,0
.4266163599,2.5654035345,5.01988778\H,0,0.655430048,2.2503925028,6.047
3575089\H,0,1.3726081964,2.6466989604,4.4706635847\H,0,-0.0144572946,3
.5681388383,5.070389073\C,0,2.1500917269,-2.5674954433,4.9667624817\H,
0,2.5933454591,-3.5702635811,4.9903592104\H,0,1.9814918645,-2.25366273
01,6.0061684811\H,0,1.1737269861,-2.6482184636,4.4734551028\\Version=I
A32L-G03RevD.01\State=1-A\HF=-535.9698216\MP2=-537.954451\RMSD=7.430e-
09\Thermal=0.\PG=C01 [X(C11H18N2)]\\@

370-Ac$^+$

1\1\GINC-NODE-28\SP\RMP2-FC\6-31+G(2d,p)\C13H21N2O1(1+)\ZIP08\07-Aug-2
010\0\\#P MP2/6-31+G(2d,p) scf=(direct,tight) int=finegrid geom=check
guess=read\\sp of d3ap7.ac1 MP2-5/6-31+G(2d,p)\\1,1\C,0,-0.0251965261,
-0.045825337,0.0101166035\C,0,0.0001674465,-0.0455184554,1.3725197617\
C,0,1.2470479749,-0.0046364,2.0911555862\C,0,2.427254145,0.0343209634,
1.2668405176\C,0,2.3326637717,0.0309556511,-0.0939799925\H,0,-0.942234
2026,-0.069727676,-0.5673951642\H,0,-0.9493103115,-0.0587168453,1.8916
895204\H,0,3.4183739265,0.048524785,1.7011036849\H,0,3.2179865813,0.05
33594727,-0.7163444924\N,0,1.3059172408,-0.003187447,3.426731581\N,0,1
.124136038,-0.0084170823,-0.7410288581\C,0,0.0944431302,-0.1092894712,
4.2693158814\H,0,-0.614695525,-0.7969248725,3.7962541212\H,0,0.4067932
714,-0.5894174534,5.2030864698\C,0,2.5851336769,0.1025184533,4.1613286
48\H,0,2.355430869,0.5849610336,5.1175817506\H,0,3.2525607734,0.788084
3322,3.6280785961\C,0,-0.5581502721,1.2525265339,4.5646486451\H,0,0.18
64542464,1.9136150832,5.0287432766\H,0,-0.8532205568,1.7310322106,3.62
04738132\C,0,3.2580613198,-1.2601298062,4.4025297703\H,0,3.4706011683,
-1.7408973907,3.437463952\H,0,2.55378469,-1.9188581961,4.9286877422\C,
0,4.5510478795,-1.1145248152,5.218360364\H,0,5.2826595423,-0.478387450
4,4.7040677543\H,0,4.3542120079,-0.6733074486,6.2031749878\H,0,5.01472
67052,-2.0934316222,5.3787621708\C,0,-1.7775171653,1.1047387877,5.4867
619763\H,0,-2.2291579988,2.0826641038,5.6833246581\H,0,-2.5474592014,0
.4655742207,5.0364021132\H,0,-1.4974334281,0.6663091246,6.45250589\C,0
,0.9802317893,-0.0121580688,-2.2108441538\O,0,-0.1312644231,-0.0516287
159,-2.6609627083\C,0,2.2502900319,0.0349072507,-3.0224648691\H,0,2.88
76495769,-0.8352249683,-2.8230827134\H,0,1.9608908434,0.0283266057,-4.
0747791643\H,0,2.8258296039,0.9456261069,-2.8167139766\\Version=IA32L-
G03RevD.01\State=1-A\HF=-688.1604523\MP2=-690.6157294\RMSD=1.652e-09\T
hermal=0.\PG=C01 [X(C13H21N2O1)]\\@

371

1\1\GINC-YANG\SP\RHF\6-31G(d)\C25H26N2\EVGENY\22-Dec-2009\0\\#P RHF/6-

31G(d) scf=tight int=finegrid SCRF=(PCM,Read,Solvent=Chloroform) geom=
check guess=read\\PCM of para2.4 b98/6-31G(d) structures on 4p 900 MB\
\0,1\C,0,-0.7933578206,0.4104293297,0.1238350797\C,0,-0.5780678506,0.3
062740879,1.5080349529\C,0,0.6252593547,-0.2824475974,1.9178638233\C,0
,1.6750623295,-0.415564622,1.0072056054\C,0,1.5534064635,0.0044610742,
-0.3266633253\C,0,0.2414777577,0.2444917918,-0.8160937081\H,0,-1.76915
81085,0.7340320995,-0.2368892231\H,0,0.8035956543,-0.4834338778,2.9733
327887\H,0,2.6575823115,-0.7051055515,1.3784225759\C,0,2.8145096066,0.
435956059,-1.0610784657\H,0,2.7795101008,0.1872025645,-2.1270282321\H,
0,3.6653143795,-0.1098623297,-0.6340171232\C,0,3.1241820662,2.00963672
12,-0.9307937406\H,0,4.2074251385,2.1198111901,-0.7889962646\H,0,2.874
2532811,2.4856296143,-1.8863130101\C,0,-1.4700845792,1.0387135552,2.49
46646292\H,0,-2.4404466127,1.2410972347,2.0249210829\H,0,-1.6683781163
,0.4264690129,3.384244161\C,0,-0.8483544921,2.43757792,2.9958138526\H,
0,-1.6602526453,3.1762875891,3.0055203564\H,0,-0.5184527619,2.31360725
74,4.034716818\C,0,0.3199308315,2.9106185998,2.1491610086\C,0,1.640222
5282,2.5910519873,2.5075855585\C,0,0.1201281324,3.4262003709,0.8585351
072\C,0,2.641481337,2.4817489634,1.5372001605\H,0,1.8574469468,2.28047
20851,3.5291111476\C,0,1.1204239495,3.3173589787,-0.110365633\H,0,-0.8
662303233,3.7864523548,0.5668540726\C,0,2.3415560297,2.6862949881,0.18
14012431\H,0,3.6166882463,2.0889657684,1.8235561049\H,0,0.8990974944,3
.5982950903,-1.1380355948\C,0,-0.0291423444,0.5169474745,-2.2614283298
\C,0,-0.6754923118,1.7204879387,-2.5957544685\C,0,0.3540228145,-0.3348
996799,-3.3479615733\H,0,-1.0086810315,2.3600383179,-1.7799915066\C,0,
0.1718258975,0.196280455,-4.6501330797\C,0,-0.4450707789,1.4286050416,
-4.8256659343\H,0,0.4785253706,-0.3627008984,-5.5268677927\H,0,-0.5952
245179,1.8139792595,-5.8346582407\N,0,-0.903952333,2.193900189,-3.8259
44445\N,0,0.8763768354,-1.603847082,-3.1905658722\C,0,0.5724066052,-2.
4920672656,-2.0467765228\H,0,-0.4077098605,-2.2513254831,-1.6208553154
\H,0,1.3145614801,-2.3706986229,-1.2487222035\C,0,1.2983663766,-2.3877
723402,-4.3600435195\H,0,0.4741037934,-2.4953581115,-5.0861662254\H,0,
2.1356855577,-1.8990980173,-4.8748635994\C,0,1.666655886,-3.7563978166
,-3.7738206223\H,0,1.6178668136,-4.5539360318,-4.5227926906\H,0,2.6846
629493,-3.7335163666,-3.3635042466\C,0,0.6359942327,-3.9088590429,-2.6
428536739\H,0,0.9156418085,-4.6581431344,-1.8944196841\H,0,-0.33906422
58,-4.1936013262,-3.0602160117\\Version=AM64L-G03RevD.01\State=1-A\HF=
-1070.6841007\RMSD=3.996e-09\Thermal=0.\Dipole=0.6637252,-1.9405269,0.
2717488\PG=C01 [X(C25H26N2)]\\@

371-Ac$^+$

1\1\GINC-YANG\SP\RHF\6-31G(d)\C25H26N2\EVGENY\22-Dec-2009\0\\#P RHF/6-
31G(d) scf=tight int=finegrid SCRF=(PCM,Read,Solvent=Chloroform) geom=
check guess=read\\PCM of para2.4 b98/6-31G(d) structures on 4p 900 MB\
\0,1\C,0,-0.7933578206,0.4104293297,0.1238350797\C,0,-0.5780678506,0.3
062740879,1.5080349529\C,0,0.6252593547,-0.2824475974,1.9178638233\C,0
,1.6750623295,-0.415564622,1.0072056054\C,0,1.5534064635,0.0044610742,
-0.3266633253\C,0,0.2414777577,0.2444917918,-0.8160937081\H,0,-1.76915
81085,0.7340320995,-0.2368892231\H,0,0.8035956543,-0.4834338778,2.9733
327887\H,0,2.6575823115,-0.7051055515,1.3784225759\C,0,2.8145096066,0.
435956059,-1.0610784657\H,0,2.7795101008,0.1872025645,-2.1270282321\H,
0,3.6653143795,-0.1098623297,-0.6340171232\C,0,3.1241820662,2.00963672
12,-0.9307937406\H,0,4.2074251385,2.1198111901,-0.7889962646\H,0,2.874
2532811,2.4856296143,-1.8863130101\C,0,-1.4700845792,1.0387135552,2.49
46646292\H,0,-2.4404466127,1.2410972347,2.0249210829\H,0,-1.6683781163
,0.4264690129,3.384244161\C,0,-0.8483544921,2.43757792,2.9958138526\H,
0,-1.6602526453,3.1762875891,3.0055203564\H,0,-0.5184527619,2.31360725
74,4.034716818\C,0,0.3199308315,2.9106185998,2.1491610086\C,0,1.640222
5282,2.5910519873,2.5075855585\C,0,0.1201281324,3.4262003709,0.8585351

072\C,0,2.641481337,2.4817489634,1.5372001605\H,0,1.8574469468,2.28047
20851,3.5291111476\C,0,1.1204239495,3.3173589787,-0.110365633\H,0,-0.8
662303233,3.7864523548,0.5668540726\C,0,2.3415560297,2.6862949881,0.18
14012431\H,0,3.6166882463,2.0889657684,1.8235561049\H,0,0.8990974944,3
.5982950903,-1.1380355948\C,0,-0.0291423444,0.5169474745,-2.2614283298
\C,0,-0.6754923118,1.7204879387,-2.5957544685\C,0,0.3540228145,-0.3348
996799,-3.3479615733\H,0,-1.0086810315,2.3600383179,-1.7799915066\C,0,
0.1718258975,0.196280455,-4.6501330797\C,0,-0.4450707789,1.4286050416,
-4.8256659343\H,0,0.4785253706,-0.3627008984,-5.5268677927\H,0,-0.5952
245179,1.8139792595,-5.8346582407\N,0,-0.903952333,2.193900189,-3.8259
44445\N,0,0.8763768354,-1.603847082,-3.1905658722\C,0,0.5724066052,-2.
4920672656,-2.0467765228\H,0,-0.4077098605,-2.2513254831,-1.6208553154
\H,0,1.3145614801,-2.3706986229,-1.2487222035\C,0,1.2983663766,-2.3877
723402,-4.3600435195\H,0,0.4741037934,-2.4953581115,-5.0861662254\H,0,
2.1356855577,-1.8990980173,-4.8748635994\C,0,1.666655886,-3.7563978166
,-3.7738206223\H,0,1.6178668136,-4.5539360318,-4.5227926906\H,0,2.6846
629493,-3.7335163666,-3.3635042466\C,0,0.6359942327,-3.9088590429,-2.6
428536739\H,0,0.9156418085,-4.6581431344,-1.8944196841\H,0,-0.33906422
58,-4.1936013262,-3.0602160117\\Version=AM64L-G03RevD.01\State=1-A\HF=
-1070.6841007\RMSD=3.996e-09\Thermal=0.\Dipole=0.6637252,-1.9405269,0.
2717488\PG=C01 [X(C25H26N2)]\\@

372

1\1\GINC-NODE21\SP\RMP2-FC\6-31+G(2d,p)\C26H28N2\ZIP01\21-Dec-2009\0\\
#P MP2/6-31+G(2d,p) scf=(direct,tight) int=finegrid geom=check guess=r
ead\\sp of para3_1 MP2-5/6-31+G(2d,p)\\0,1\C,0,0.0402154986,-0.0146044
679,0.0293655797\C,0,-0.1016989064,-0.0107348099,1.427819593\C,0,1.077
2067508,0.0602782068,2.1814127952\C,0,2.2624536515,0.4747656505,1.5718
468614\C,0,2.3178934817,0.7948302937,0.2064225103\C,0,1.2392726857,0.3
589716595,-0.6079925714\H,0,-0.8320267049,-0.2162055101,-0.5918663185\
H,0,1.039458429,-0.0381959891,3.2654358016\H,0,3.122216,0.7048586478,2
.1999068445\C,0,3.3593466532,1.8046832213,-0.2540420578\H,0,4.20072537
78,1.7744079059,0.4499107332\H,0,3.7641293259,1.5663984931,-1.24362347
91\C,0,2.8066315417,3.3119485345,-0.2976348187\H,0,3.6244080643,3.9687
2181,0.0280188254\H,0,2.5860191799,3.5650226706,-1.3412996385\C,0,-1.4
596533162,0.1884977859,2.0797090082\H,0,-2.2478539811,-0.0557892418,1.
3569140063\H,0,-1.5984996235,-0.4829139512,2.9376507832\C,0,-1.6862393
012,1.6907338035,2.6056056337\H,0,-2.7414977767,1.9437199069,2.4399268
854\H,0,-1.5275850717,1.697900101,3.6911165337\C,0,-0.7406458037,2.696
9906052,1.9676306589\C,0,0.4398569219,3.0536236788,2.6372645\C,0,-0.86
39560349,3.0803791078,0.6124429987\C,0,1.5784525415,3.4657336623,1.937
9731035\H,0,0.5190096483,2.8625926086,3.7071506969\C,0,0.2895342751,3.
4801275979,-0.0754570263\C,0,1.5537647278,3.5130507214,0.5378989896\H,
0,2.5189602567,3.5883876085,2.4740304791\H,0,0.2231861441,3.6454920593
,-1.1502052569\C,0,1.2814136451,0.4697532324,-2.0989151095\C,0,0.26831
32273,1.2080969276,-2.7351316096\C,0,2.3063279364,-0.0780179444,-2.937
0143273\H,0,-0.5366581548,1.6004529174,-2.1167674285\C,0,2.2653060717,
0.3036779634,-4.3020286173\C,0,1.2173657639,1.0786743893,-4.783036163\
H,0,3.0291492601,-0.0288031567,-4.9957086195\H,0,1.1904481055,1.347363
3355,-5.8395479536\N,0,0.1960015285,1.5175368685,-4.0348824454\N,0,3.2
997635758,-0.9273590233,-2.4902590808\C,0,4.3557072438,-1.3874352341,-
3.4030767826\H,0,4.9662259499,-0.5432790231,-3.7490007512\H,0,3.927843
1821,-1.8796430208,-4.2936119905\C,0,3.1437093793,-1.8635799436,-1.355
0409697\H,0,3.4742562193,-1.4001721801,-0.4178661261\H,0,2.0925496885,
-2.1449963105,-1.2284376106\C,0,4.0408186987,-3.0569948674,-1.72602493
51\H,0,4.4170577192,-3.5807258442,-0.8406403285\H,0,3.4828454436,-3.77
92518334,-2.3366267039\C,0,5.1475451064,-2.4024883483,-2.5692085639\H,
0,5.699822727,-3.1113034634,-3.1952894223\H,0,5.8665276822,-1.88445668

73,-1.921277697\C,0,-2.1727192193,2.9218005708,-0.1345596016\H,0,-2.42
62130723,1.8639057133,-0.2967172808\H,0,-3.0095431889,3.3713711652,0.4
158834988\H,0,-2.1229754748,3.4022506336,-1.1181751722\\Version=AM64L-
G03RevD.01\State=1-A\HF=-1109.8100183\MP2=-1113.952361\RMSD=3.115e-09\
Thermal=0.\PG=C01 [X(C26H28N2)]\\@

372-Ac$^+$

1\1\GINC-NODE26\SP\RMP2-FC\6-31+G(2d,p)\C28H31N2O1(1+)\ZIP01\12-Jan-20
10\0\\#P MP2/6-31+G(2d,p) scf=(direct,tight) int=finegrid geom=check g
uess=read\\sp of para3_1.ac2 MP2-5/6-31+G(2d,p)\\1,1\C,0,0.1015281663,
-0.0052877037,-0.0527037538\C,0,0.1189884141,-0.0263704226,1.35243357\
C,0,1.3778468853,0.0608940848,1.9633625016\C,0,2.4810675382,0.51388891
75,1.2377690226\C,0,2.3824147637,0.8537368724,-0.1206025282\C,0,1.2215
071754,0.4021213171,-0.7988307063\H,0,-0.8279065976,-0.2240206335,-0.5
778835675\H,0,1.4675145285,-0.064938816,3.040687408\H,0,3.3976459269,0
.7532084509,1.7739528901\C,0,3.3412079829,1.8922954207,-0.6842733018\H
,0,4.3047491105,1.7942983593,-0.1708943905\H,0,3.5391230055,1.73828922
88,-1.7524074641\C,0,2.8301721778,3.4121442991,-0.4922407664\H,0,3.688
2361547,3.9999109676,-0.1446648122\H,0,2.5552335181,3.8039029774,-1.47
94362907\C,0,-1.1592667979,0.1126088629,2.1612019318\H,0,-2.0173512627
,-0.1522118959,1.5321779067\H,0,-1.1668263537,-0.5774161552,3.01394162
52\C,0,-1.3713796575,1.5997760457,2.7362112235\H,0,-2.4397647512,1.833
7932401,2.657411236\H,0,-1.1294512925,1.5879164552,3.8051130935\C,0,-0
.4969933161,2.6339678288,2.0450017438\C,0,0.7272712753,2.9990045866,2.
6232223103\C,0,-0.7466227646,3.0544749699,0.7170436177\C,0,1.796246786
4,3.4514818309,1.8418315174\H,0,0.897354365,2.791344797,3.6787849777\C
,0,0.3360649787,3.501501796,-0.0510161393\C,0,1.6490175621,3.543265768
2,0.4523790734\H,0,2.7744451739,3.5886363119,2.3002887259\H,0,0.168324
2768,3.7423196437,-1.1013343203\C,0,1.1089090161,0.4939933607,-2.28927
65656\C,0,0.1543373637,1.3389822566,-2.79850921\C,0,1.9484112724,-0.23
37817746,-3.2332919316\H,0,-0.5159975392,1.886910729,-2.1500731055\C,0
,1.8131309871,0.1471534046,-4.6176361221\C,0,0.8460242918,1.0128942863
,-5.0305146635\H,0,2.4522590265,-0.2849761646,-5.3762165189\H,0,0.7209
942437,1.2655501465,-6.0752711982\N,0,-0.0177023841,1.590299504,-4.139
4990742\N,0,2.7950477512,-1.2169194451,-2.9095086001\C,0,3.6785879245,
-1.8618373401,-3.9187873415\H,0,4.3015222332,-1.1079943406,-4.41285931
\H,0,3.0589765052,-2.3595177784,-4.6778052105\C,0,2.8784944985,-1.9403
208137,-1.6093715119\H,0,3.5026741769,-1.3710088177,-0.9125300765\H,0,
1.8844396083,-2.0396416672,-1.1704796193\C,0,3.5296082798,-3.277442743
,-1.9849613589\H,0,4.0319243038,-3.7338715754,-1.1271052048\H,0,2.7728
872967,-3.9821069373,-2.3518894779\C,0,4.4943258211,-2.8831337445,-3.1
141222621\H,0,4.807959892,-3.7271483915,-3.7349595472\H,0,5.3940150647
,-2.4098520408,-2.7028632521\C,0,-2.1181973582,2.9082663366,0.08935186
2\H,0,-2.4006675945,1.8532371269,-0.0449527534\H,0,-2.8955723298,3.365
7697049,0.714160891\H,0,-2.1578801748,3.3900165102,-0.8939922677\C,0,-
1.0876747566,2.5170239067,-4.532464869\O,0,-1.7556220145,3.0124317169,
-3.6646410749\C,0,-1.2662007738,2.7729896602,-6.0094531766\H,0,-0.3805
997131,3.2532765692,-6.4434959912\H,0,-1.4654108958,1.8456026748,-6.55
97328147\H,0,-2.1198328701,3.4439919036,-6.1194527522\\Version=AM64L-G
03RevD.01\State=1-A\HF=-1262.0008482\MP2=-1266.6178584\RMSD=7.951e-09\
Thermal=0.\PG=C01 [X(C28H31N2O1)]\\@

373

1\1\GINC-YIN\SP\RMP2-FC\6-31+G(2d,p)\C9H14N2\RAMAN\01-Jun-2010\0\\#P M
P2/6-31+G(2d,p) scf=(direct,tight) int=finegrid geom=check guess=read\
\sp of d2ap4 mp2-5 on 2p 900 MB\\0,1\C,0,0.339189923,0.1045160185,-0.0
508099047\C,0,0.3656013428,0.7393099621,1.2168153949\C,0,1.4942523264,
0.6195134241,2.0226630601\C,0,2.5780335356,-0.6569790112,0.5027038833\

C,0,1.5113758215,-0.6172319268,-0.3907792768\H,0,-0.469080179,1.333490
2525,1.5728140506\H,0,1.5024570966,1.1139081434,2.9948677958\H,0,3.473
3451866,-1.2170137287,0.2298275118\H,0,1.5954063939,-1.156613535,-1.32
80525134\N,0,-0.749397333,0.1835555428,-0.8934037159\C,0,-1.9911970846
,0.835997293,-0.4836599029\H,0,-2.812160956,0.3495292182,-1.0260243976
\H,0,-2.1728693774,0.6341273198,0.578719102\C,0,-0.719279545,-0.381464
8868,-2.2408082108\H,0,0.2790349976,-0.2383662498,-2.6712922146\H,0,-1
.4017169083,0.2132635383,-2.8615566375\C,0,-1.1247114037,-1.8633202162
,-2.299951123\H,0,-2.1427781468,-2.0042004805,-1.9162639211\H,0,-1.096
9932169,-2.2282303897,-3.3346918687\H,0,-0.4499754987,-2.4815437006,-1
.6970700162\C,0,-2.0150499368,2.3487800793,-0.7568257536\H,0,-1.211322
1899,2.8603261453,-0.2152509811\H,0,-1.883139428,2.5535957376,-1.82648
52635\H,0,-2.9738370802,2.7809249113,-0.4428430073\N,0,2.6055513747,-0
.0601764947,1.7034763805\\Version=AM64L-G03RevD.01\State=1-A\HF=-457.8
949688\MP2=-459.5694926\RMSD=7.552e-09\Thermal=0.\PG=C01 [X(C9H14N2)]\
\@

373-Ac⁺

1\1\GINC-GOLEM\SP\RMP2-FC\6-31+G(2d,p)\C11H17N2O1(1+)\RAMAN\10-Jul-201
0\0\\#P MP2/6-31+G(2d,p) scf=(direct,tight) int=finegrid geom=check gu
ess=read\\sp of d2ap4.ac1 mp2-5 on 2p 900 MB\\1,1\C,0,-0.0104187226,0.
001982288,0.0071882934\C,0,-0.0192655032,-0.0229492585,1.4466319418\C,
0,1.1468628896,-0.0165793546,2.1550844316\C,0,2.4230769727,0.033748608
3,0.169494355\C,0,1.2957444442,0.0309595709,-0.5966168104\H,0,-0.94585
63056,-0.0271862045,2.0057991043\H,0,1.143824884,-0.0271383218,3.23745
15324\H,0,3.4242158983,0.0478634181,-0.2460460874\H,0,1.4204187171,0.0
314251398,-1.6717042285\N,0,-1.1353608557,-0.0013421797,-0.7158457758\
C,0,-2.4741965127,-0.1112554548,-0.095294882\H,0,-3.1163827914,-0.6028
732051,-0.8329806372\H,0,-2.4168488343,-0.792392888,0.7593733384\C,0,-
1.1255127592,0.1052975309,-2.1923176003\H,0,-0.3248794497,0.7868660289
,-2.4953084159\H,0,-2.0639972086,0.594803002,-2.4716146464\C,0,-1.0033
464283,-1.2557510582,-2.889077541\H,0,-1.8280192931,-1.9205288733,-2.6
078948844\H,0,-1.0384275474,-1.1146620009,-3.9749990857\H,0,-0.0611881
242,-1.756767343,-2.6389557928\C,0,-3.0618057045,1.248405675,0.3035646
273\H,0,-2.4436683377,1.7532062347,1.054864873\H,0,-3.1511692318,1.911
3411,-0.5644729647\H,0,-4.0634844961,1.1049943525,0.7236418451\N,0,2.3
723677098,0.011340863,1.5413572432\C,0,3.6573253852,0.017788026,2.2728
910203\O,0,4.665444021,0.0456522069,1.6235146019\C,0,3.5866272197,-0.0
118257435,3.7786604583\H,0,3.0830513682,-0.916702809,4.1400038565\H,0,
4.6129526503,-0.0048402155,4.1498730528\H,0,3.0591098945,0.8648456731,
4.1740107242\\Version=AM64L-G03RevD.01\State=1-A\HF=-610.080645\MP2=-6
12.2297885\RMSD=2.173e-09\Thermal=0.\PG=C01 [X(C11H17N2O1)]\\@

374

1\1\GINC-NODE11\SP\RMP2-FC\6-31+G(2d,p)\C15H26N2\ZIP08\25-Aug-2010\0\\
#P MP2/6-31+G(2d,p) scf=(direct,tight) int=finegrid geom=check guess=r
ead\\sp of d5ap37 MP2-5/6-31+G(2d,p)\\0,1\C,0,0.0014867751,-0.01645941
62,0.0236060982\C,0,0.0013212044,-0.0177323691,1.4156315125\C,0,1.2333
979044,-0.0000661134,2.1184253738\C,0,2.3926011911,0.020405548,1.30106
78093\C,0,2.2592800064,0.0242580522,-0.0845535698\H,0,-0.9535421003,-0
.0320336188,-0.5030596191\H,0,-0.9501710928,-0.0283832922,1.9354548191
\H,0,3.3894565134,0.0293113068,1.7275270399\H,0,3.159553088,0.04193999
95,-0.7000981652\N,0,1.2995116585,-0.0026122162,3.4975716883\N,0,1.095
8245125,0.0052292411,-0.7513748222\C,0,2.5909752144,-0.0397516187,4.18
72997346\H,0,3.273261426,0.6855923901,3.7232784684\H,0,2.4235330637,0.
3173455932,5.2098877405\C,0,0.0799530219,0.0317594668,4.3078049334\H,0
,0.3443783439,-0.3290799126,5.3083626208\H,0,-0.6437228645,-0.69198442
58,3.9085023403\C,0,3.2592352099,-1.4298316156,4.2375712437\H,0,3.4377

762748,-1.7887123781,3.2145863446\H,0,4.2495784316,-1.3062471894,4.702
9517227\C,0,-0.5801809201,1.4215292439,4.4268984141\H,0,-1.5214962783,
1.2960548669,4.9844047276\H,0,-0.8556791396,1.784159416,3.4270131944\C
,0,0.2930932516,2.4765193867,5.1246466715\H,0,0.5735451251,2.117926334
7,6.1286179957\H,0,1.2315667579,2.6018771329,4.5642983252\C,0,2.456516
9288,-2.4875553513,5.0117347567\H,0,2.2734190467,-2.1327225414,6.03924
12415\H,0,1.4687377943,-2.6110232977,4.5432420846\C,0,-0.4020904844,3.
8424575033,5.2525972659\H,0,-1.3431010934,3.72053,5.8100434727\H,0,-0.
6832409042,4.1987997035,4.2503928899\C,0,3.1605276351,-3.8538235852,5.
0675779869\H,0,4.1505611444,-3.7337658211,5.5329092119\H,0,3.344475279
2,-4.2064234614,4.0417729996\C,0,2.3571799497,-4.9121271593,5.83723587
61\H,0,2.8824527893,-5.8749190225,5.8581565104\H,0,2.1866908877,-4.601
5133231,6.8767081889\H,0,1.3752269402,-5.0768655661,5.3742711178\C,0,0
.4713908599,4.8980518764,5.9457751434\H,0,-0.0492960141,5.8606702458,6
.0203968747\H,0,0.7404760041,4.583633449,6.9630184918\H,0,1.4045846628
,5.0646657892,5.3916121463\\Version=AM64L-G03RevD.01\State=1-A\HF=-692
.1259876\MP2=-694.7220286\RMSD=7.521e-09\Thermal=0.\PG=C01 [X(C15H26N2
)]\\@

374-Ac⁺

1\1\GINC-NODE10\SP\RMP2-FC\6-31+G(2d,p)\C17H29N2O1(1+)\ZIP08\04-Sep-20
10\0\\#P MP2/6-31+G(2d,p) scf=(direct,tight) int=finegrid geom=check g
uess=read\\sp of d5ap7.ac2 MP2-5/6-31+G(2d,p)\\1,1\C,0,-0.0319818393,-
0.0558168741,-0.0119047114\C,0,-0.0049573136,-0.0658633564,1.350186253
8\C,0,1.2424576282,-0.0134709761,2.0680359177\C,0,2.4216337234,0.04020
70351,1.2422933396\C,0,2.3255236662,0.0435326518,-0.118107064\H,0,-0.9
497426424,-0.0839641266,-0.5877736852\H,0,-0.9537193775,-0.0916987048,
1.8703591211\H,0,3.4130915596,0.0572176361,1.6757777708\H,0,3.20963411
06,0.0754054519,-0.7417557538\N,0,1.3030373178,-0.0145004235,3.4026037
364\N,0,1.1163164496,-0.0011458133,-0.7644631649\C,0,0.0967804149,-0.1
518053253,4.2484568781\H,0,-0.5990327821,-0.8499658906,3.7715569514\H,
0,0.4242512585,-0.634533295,5.1755646203\C,0,2.579111064,0.1259386996,
4.1371056199\H,0,2.3366909066,0.6189206779,5.084809309\H,0,3.232497453
,0.8170187352,3.5942131164\C,0,-0.5834066373,1.1923228959,4.5613536311
\H,0,0.1496294613,1.867405846,5.025917313\H,0,-0.8981921469,1.67419267
04,3.6242505699\C,0,3.2814139535,-1.217107834,4.4020548496\H,0,3.49736
3528,-1.716432661,3.4461464077\H,0,2.596725373,-1.8801453002,4.9498937
159\C,0,4.5816339166,-1.0327425364,5.2042641342\H,0,5.2627164336,-0.36
56319239,4.6531125633\H,0,4.3556334848,-0.525266465,6.1544601006\C,0,-
1.7940287753,1.0166587189,5.4946853387\H,0,-2.5200917811,0.3302327061,
5.0318537847\H,0,-1.4673855649,0.5355233635,6.4292061053\C,0,-2.491249
1305,2.3480796811,5.8246224213\H,0,-1.762818077,3.0334048219,6.2823612
084\H,0,-2.8206786889,2.8261143334,4.8902106671\C,0,5.2977436612,-2.36
30069212,5.4960012605\H,0,4.6168436904,-3.025658707,6.0499082844\H,0,5
.5191585305,-2.8717307925,4.5459392689\C,0,6.5956509829,-2.1744898913,
6.2939496942\H,0,6.3999010613,-1.700309324,7.2641510255\H,0,7.08199076
5,-3.1377373527,6.4852685082\H,0,7.3086749099,-1.5419581405,5.74962824
46\C,0,-3.6924256948,2.1713674789,6.7639422424\H,0,-3.3866135642,1.728
8395474,7.7205862416\H,0,-4.1673294087,3.1353718944,6.9787104802\H,0,-
4.452395725,1.5159481928,6.3193216463\C,0,0.9723463059,0.0086896772,-2
.2326966231\O,0,-0.1385187846,-0.0351223056,-2.6847500447\C,0,2.242319
2551,0.0743302082,-3.0437172334\H,0,2.8853881645,-0.7939270325,-2.8546
269022\H,0,1.953138434,0.0784680575,-4.0960857933\H,0,2.8119996813,0.9
861483873,-2.8268079555\\Version=AM64L-G03RevD.01\State=1-A\HF=-844.31
80435\MP2=-847.3840125\RMSD=7.028e-09\Thermal=0.\PG=C01 [X(C17H29N2O1)
]\\@

375

1\1\GINC-NODE17\SP\RMP2-FC\6-31+G(2d,p)\C13H22N2\ZIP08\06-Aug-2010\0\\
#P MP2/6-31+G(2d,p) scf=(direct,tight) int=finegrid geom=check guess=r
ead\\sp of d4ap48 MP2-5/6-31+G(2d,p)\\0,1\C,0,-0.0055057985,0.01443493
43,0.0051449803\C,0,-0.0033652776,0.0054740343,1.3974313126\C,0,1.2290
102669,0.0037438309,2.1002276139\C,0,2.3860604401,0.0110397965,1.28055
63205\C,0,2.2515805281,0.0127296105,-0.1046772797\H,0,-0.9617484113,0.
0155359263,-0.5195634944\H,0,-0.9551451296,-0.0103327931,1.9165868202\
H,0,3.3836477667,0.0380090717,1.7036786877\H,0,3.1518159468,0.01916628
58,-0.7204058495\N,0,1.2867496497,0.011977725,3.4806718417\N,0,1.08744
78493,0.014616457,-0.7713365086\C,0,2.5275890863,-0.2018709713,4.22818
90987\H,0,2.3123962584,-0.9225342578,5.0306423727\H,0,3.2640056973,-0.
6892071844,3.5811683076\C,0,0.0651167233,0.0176636889,4.2840373014\H,0
,-0.6570886367,0.7145350766,3.8402905364\H,0,0.3198880703,0.4379796098
,5.2658273639\C,0,3.1237915258,1.0745663606,4.8561634805\H,0,2.3689143
913,1.5355592938,5.511942865\H,0,3.9523785127,0.7691581868,5.513982420
8\C,0,-0.5761402318,-1.3713776812,4.4748778922\H,0,0.1537158712,-2.041
289532,4.9539747839\H,0,-0.7973673464,-1.8093328294,3.4917255303\C,0,-
1.8580138323,-1.3152514856,5.3220949715\H,0,-1.6304755696,-0.865337635
7,6.3006189041\H,0,-2.5844959571,-0.6444578146,4.8386613587\C,0,3.6261
448637,2.1216833122,3.8496842324\H,0,2.8136041658,2.3838946141,3.15877
47708\H,0,4.4267661999,1.6830673985,3.2352517397\C,0,4.1504038017,3.39
22785889,4.5350375647\H,0,4.9827019977,3.1643084,5.2143291845\H,0,4.50
95745418,4.1214017764,3.7985471248\H,0,3.3618661029,3.8760967056,5.126
8197966\C,0,-2.4974441764,-2.6957789305,5.5301446028\H,0,-3.409887763,
-2.6250843424,6.1348054761\H,0,-2.7658019758,-3.1554817087,4.570038865
\H,0,-1.806933573,-3.3776791195,6.0435585072\\Version=AM64L-G03RevD.01
\State=1-A\HF=-614.0482204\MP2=-616.3371329\RMSD=8.983e-09\Thermal=0.\
PG=C01 [X(C13H22N2)]\\@

375-Ac$^+$

1\1\GINC-NODE15\SP\RMP2-FC\6-31+G(2d,p)\C15H25N2O1(1+)\ZIP08\18-Aug-20
10\0\\#P MP2/6-31+G(2d,p) scf=(direct,tight) int=finegrid geom=check g
uess=read\\sp of d4ap37.ac2 MP2-5/6-31+G(2d,p)\\1,1\C,0,-0.0757037759,
0.0135610438,0.0083584341\C,0,-0.0383736016,0.0182216473,1.3720416662\
C,0,1.2163250856,0.0013211416,2.079735735\C,0,2.3879123992,-0.01491518
43,1.2424297056\C,0,2.28157847,-0.0138266221,-0.1161641448\H,0,-1.0174
326774,0.0242450504,-0.5250497564\H,0,-0.9830379451,0.0429099138,1.898
6781106\H,0,3.3831593161,-0.0368910875,1.6660249537\H,0,3.1393118195,-
0.0250961761,-0.7786122773\N,0,1.2872245616,0.0005902813,3.4154671841\
N,0,1.0647897646,-0.0016341942,-0.7530145918\C,0,2.5921409537,-0.02283
1794,4.1210682937\H,0,3.2657870606,0.6913916566,3.6342078454\H,0,2.406
3067207,0.3685881452,5.1249657388\C,0,0.0662266572,0.022468841,4.25676
59158\H,0,0.357609685,-0.3735169809,5.2334147539\H,0,-0.6570344515,-0.
6891684915,3.8422667317\C,0,3.2367520626,-1.4193204655,4.2114740984\H,
0,3.4290913667,-1.8109130311,3.2022317284\H,0,4.221614373,-1.278882749
1,4.6796553073\C,0,-0.5629809497,1.4193285684,4.4210167403\H,0,-1.4925
231596,1.2787934728,4.9912354698\H,0,-0.8608105992,1.8148478617,3.4390
918661\C,0,0.325790642,2.4505983377,5.1376705405\H,0,0.630145469,2.051
7044903,6.1169125238\H,0,1.2485910947,2.6112555495,4.5610279005\C,0,2.
4309854845,-2.4541613301,5.0157311988\H,0,2.2336987161,-2.0593625947,6
.0236618961\H,0,1.4513985097,-2.6137576984,4.5412941413\C,0,-0.3891404
915,3.7955560497,5.3340905443\H,0,-1.2949992342,3.6771574586,5.9415378
08\H,0,0.2626583426,4.5148718353,5.8420661888\H,0,-0.6845770894,4.2329
178527,4.3717276672\C,0,3.163876634,-3.799118275,5.1283749226\H,0,4.12
99163618,-3.6819237887,5.6348845893\H,0,2.5713847581,-4.5213461715,5.7
008189783\H,0,3.3542768333,-4.2319046401,4.137906785\C,0,1.065381106,-
0.0048814515,-2.2294502945\O,0,2.1284781788,-0.015808894,-2.785867815\

C,0,-0.2782551615,0.0048498041,-2.9141902868\H,0,-0.8547936452,0.90187
10234,-2.6571978988\H,0,-0.0921480719,-0.0007501365,-3.9896036481\H,0,
-0.8707083975,-0.8797582302,-2.6504673194\\Version=AM64L-G03RevD.01\St
ate=1-A\HF=-766.2351721\MP2=-769.000087\RMSD=1.939e-09\Thermal=0.\PG=C
01 [X(C15H25N2O1)]\\@

376

1\1\GINC-NODE11\SP\RMP2-FC\6-31+G(2d,p)\C17H30N2\ZIP08\16-Oct-2010\0\\
#P MP2/6-31+G(2d,p) scf=(direct,tight) int=finegrid geom=check guess=r
ead\\sp of d6ap37 MP2-5/6-31+G(2d,p)\\0,1\C,0,-0.0008612061,0.00164042
27,-0.0183173317\C,0,-0.0009031108,0.0017299524,1.3737069883\C,0,1.231
4169253,0.0000327204,2.076297911\C,0,2.3907117131,0.0008402649,1.25881
12021\C,0,2.257231308,0.0055068517,-0.1267908074\H,0,-0.9560974936,0.0
011599592,-0.54487079\H,0,-0.9523031832,0.0072582398,1.8937809847\H,0,
3.3875836019,-0.0062478211,1.6852684709\H,0,3.1575935499,0.007869503,-
0.7424834203\N,0,1.2976698848,-0.0022399167,3.4554065112\N,0,1.0935520
444,0.0047615914,-0.7934671364\C,0,2.5888052951,-0.0535941334,4.144673
969\H,0,3.2803307153,0.6596526002,3.6756867537\H,0,2.4275043463,0.3120
339593,5.1653116651\C,0,0.0785610738,0.0466434431,4.2654321992\H,0,0.3
368990523,-0.3223133129,5.2646997358\H,0,-0.6548491318,-0.6651665793,3
.8625509806\C,0,3.2376931137,-1.4526847639,4.2012903602\H,0,3.40198557
73,-1.8221104115,3.179685777\H,0,4.2336688959,-1.3401452612,4.65730994
59\C,0,-0.5616904777,1.4454377584,4.3885899561\H,0,-1.509383611,1.3312
420195,4.9376107434\H,0,-0.8230985425,1.8181945199,3.3886592237\C,0,0.
3223204965,2.4821320346,5.1003596208\H,0,0.5843283733,2.1138857952,6.1
057474781\H,0,1.2690820631,2.5917577407,4.5509353868\C,0,2.4258191669,
-2.4918532838,4.9911105647\H,0,2.2614523633,-2.1269563344,6.0181898895
\H,0,1.4307325807,-2.5998210246,4.5346153331\C,0,-0.3495738276,3.85971
81114,5.2254219696\H,0,-1.3025404118,3.7539262013,5.7677995502\H,0,-0.
6087454141,4.2289396756,4.2208207181\C,0,3.1063897476,-3.8697371869,5.
0466588664\H,0,4.1069766873,-3.7655790994,5.4955325123\H,0,3.268008548
6,-4.2356124745,4.0206311934\C,0,2.3028581334,-4.9154614071,5.83731177
83\H,0,2.1432126395,-4.5499591346,6.8631447486\H,0,1.3028921021,-5.017
94726,5.3894180071\C,0,0.52621406,4.9029639821,5.9388417999\H,0,0.7834
029561,4.5341157154,6.9434395799\H,0,1.4786561974,5.007080446,5.397493
4964\C,0,2.9864372149,-6.2896418759,5.8838036422\H,0,3.9760710537,-6.2
237328465,6.3552640435\H,0,2.3915747784,-7.0131778699,6.454989632\H,0,
3.1269982607,-6.6956025093,4.873257972\C,0,-0.1495437755,6.2768758619,
6.055165929\H,0,-1.0894364782,6.209260132,6.6191019735\H,0,0.497447014
2,6.9986238728,6.5690743\H,0,-0.3862642304,6.6861350714,5.064088575\\V
ersion=AM64L-G03RevD.01\State=1-A\HF=-770.2040654\MP2=-773.105282\RMSD
=1.955e-09\Thermal=0.\PG=C01 [X(C17H30N2)]\\@

376-Ac^{+}

1\1\GINC-NODE23\SP\RMP2-FC\6-31+G(2d,p)\C19H33N2O1(1+)\ZIP08\13-Nov-20
10\0\\#P MP2/6-31+G(2d,p) scf=(direct,tight) int=finegrid geom=check g
uess=read\\sp of d6ap37.ac2 MP2-5/6-31+G(2d,p)\\1,1\C,0,-1.2666768835,
-2.7301695689,-1.1570890447\C,0,-0.5251732238,-1.5872364919,-1.1712406
069\C,0,-0.4612799956,-0.7280646332,-0.0169328547\C,0,-1.2273141453,-1
.1749504297,1.1187899724\C,0,-1.9453415906,-2.333732192,1.0694893056\H
,0,-1.3401521253,-3.4000491164,-2.0058445635\H,0,0.0171751554,-1.35430
64235,-2.0779272437\H,0,-1.2601820034,-0.6037576536,2.0370483225\H,0,-
2.5199762645,-2.6726653419,1.9217801349\N,0,0.2564025239,0.3997682891,
-0.0000917617\N,0,-1.9809125844,-3.123282183,-0.0515500397\C,0,0.29945
90774,1.2753485405,1.1957445271\H,0,0.4213934565,0.6475481452,2.085749
6941\H,0,1.2155235858,1.8663345722,1.1121364761\C,0,1.0470133525,0.831
2983757,-1.1787598832\H,0,1.1919059723,1.9097846558,-1.0734274167\H,0,
0.4378828089,0.6907559699,-2.0786974774\C,0,-0.9191432488,2.2082449206

,1.3372981671\H,0,-1.8386622637,1.6125623947,1.4321152389\H,0,-0.79716
94309,2.7392711449,2.29230307\C,0,2.4100709269,0.1262619509,-1.3172435
706\H,0,2.8432757768,0.4694541328,-2.2676646546\H,0,2.2614966718,-0.95
83932774,-1.4205192133\C,0,3.4019679671,0.4003768921,-0.1738469236\H,0
,3.5440899867,1.4870559939,-0.064892641\H,0,2.9844390313,0.0403049715,
0.779202152\C,0,-1.0857498227,3.2293715938,0.1991377127\H,0,-0.1587670
448,3.8145571309,0.0939255865\H,0,-1.2364342141,2.7027860015,-0.755890
6921\C,0,4.7685904475,-0.2677454962,-0.4050339867\H,0,5.1908237055,0.0
900873851,-1.3564115886\H,0,4.6279514555,-1.353901053,-0.5198676754\C,
0,-2.2653780314,4.1898265277,0.4316391558\H,0,-2.1146887708,4.72660874
59,1.3806323491\H,0,-3.1920199692,3.6069295261,0.5515181785\C,0,-2.447
8933851,5.2076741953,-0.7065233668\H,0,-1.5202338212,5.7867770293,-0.8
263132014\H,0,-2.5996174439,4.6688253036,-1.6540285526\C,0,5.772062668
5,-0.0010378331,0.7293716354\H,0,5.9109004408,1.0845550604,0.841871025
3\H,0,5.3480043231,-0.3578233942,1.6802410465\C,0,-3.6234057981,6.1659
014141,-0.4671355737\H,0,-3.4810069505,6.7454601347,0.4539710359\H,0,-
3.7289328098,6.87618698,-1.2953228777\H,0,-4.569979711,5.6177104025,-0
.3740372901\C,0,7.1331311382,-0.6702050902,0.4908696844\H,0,7.59646538
99,-0.3066309899,-0.4353167679\H,0,7.8255579644,-0.4617058522,1.314641
7718\H,0,7.0305854011,-1.7599877951,0.4075659664\C,0,-2.7462177851,-4.
3812688446,-0.1434525972\O,0,-2.6994252328,-4.9885032144,-1.1774848031
\C,0,-3.5263295242,-4.7988325069,1.0780472504\H,0,-4.2841883817,-4.052
8005611,1.3465973189\H,0,-4.0246425525,-5.7392045355,0.8361755214\H,0,
-2.8681979458,-4.956074659,1.9412839844\\Version=AM64L-G03RevD.01\Stat
e=1-A\HF=-922.3923377\MP2=-925.7679439\RMSD=2.247e-09\Thermal=0.\PG=C0
1 [X(C19H33N2O1)]\\@

377

1\1\GINC-NODE13\SP\RMP2-FC\6-31+G(2d,p)\C21H38N2\ZIP08\08-Jul-2011\0\\
#P MP2/6-31+G(2d,p) scf=(direct,tight) int=finegrid geom=check guess=r
ead\\sp of d8ap37 MP2-5/6-31+G(2d,p)\\0,1\C,0,-0.3397207321,3.79863572
77,-1.0782454907\C,0,-0.3624654111,2.408235675,-1.1410778161\C,0,-0.00
0110676,1.6475139473,-0.0000297685\C,0,0.3621676508,2.4082304017,1.141
0461062\C,0,0.3392826773,3.7986305122,1.0782645804\H,0,-0.6186028938,4
.3705127829,-1.9641801942\H,0,-0.6627474688,1.9339571227,-2.0686292385
\H,0,0.6624982576,1.9339482954,2.0685799783\H,0,0.6181075035,4.3705032
465,1.9642201262\N,0,-0.000041543,0.2669181642,-0.0000557679\N,0,-0.00
02554598,4.5203354706,0.0000227731\C,0,0.4340653915,-0.4843515779,1.17
97632453\H,0,-0.028209122,-0.0520302241,2.0778099543\H,0,0.0238976865,
-1.4966508459,1.0889252433\C,0,-0.4340631309,-0.4843493775,-1.17990751
87\H,0,-0.0237897337,-1.4966092977,-1.0891085588\H,0,0.0281696055,-0.0
519426927,-2.0779347909\C,0,1.9635686451,-0.5695199565,1.3677337267\H,
0,2.3763386392,0.4426791608,1.4776375272\H,0,2.1530319907,-1.089795986
9,2.3195526422\C,0,-1.9635564489,-0.5696721992,-1.3678873514\H,0,-2.15
29609284,-1.0899278879,-2.3197291601\H,0,-2.3764343416,0.4424874117,-1
.4777491866\C,0,-2.6999214316,-1.2943348058,-0.2296810567\H,0,-2.28605
20203,-2.3088299429,-0.1091128065\H,0,-2.5140763014,-0.7662007195,0.71
71382924\C,0,2.7000073265,-1.2940555756,0.2294943622\H,0,2.2862459443,
-2.3085898352,0.1088844605\H,0,2.5141032311,-0.7659012433,-0.717302095
2\C,0,-4.2170330379,-1.3918493089,-0.4627869637\H,0,-4.4074236794,-1.9
305761636,-1.4043969911\H,0,-4.6275192633,-0.3794123932,-0.6008722312\
C,0,4.2171303224,-1.3914171927,0.4625904094\H,0,4.4075831465,-1.930162
3223,1.4041773892\H,0,4.6275081417,-0.3789417606,0.6007155433\C,0,4.96
62299627,-2.0901263613,-0.684200044\H,0,4.5506214069,-3.0999830942,-0.
8293191738\H,0,4.7808211077,-1.5451753001,-1.6231868423\C,0,-4.9660630
652,-2.0906859025,0.6839713116\H,0,-4.5503459558,-3.1005034303,0.82905
2372\H,0,-4.7807185322,-1.5457522972,1.6229808581\C,0,6.4820232749,-2.
1945508716,-0.4488211144\H,0,6.6684308355,-2.7468529788,0.4858787803\H

,0,6.8968322551,-1.1857640684,-0.2951114975\C,0,-6.4818436439,-2.19526
577,0.448579065\H,0,-6.6681848689,-2.7475498087,-0.4861447171\H,0,-6.8
967619814,-1.1865180236,0.2949083446\C,0,-7.2342355164,-2.8813566851,1
.6008186857\H,0,-6.817495193,-3.8878727571,1.7576259928\H,0,-7.0519714
447,-2.3260495374,2.5332828652\C,0,-8.7463228133,-2.9861483211,1.35456
18502\H,0,-8.9599332174,-3.5672632888,0.4475760987\H,0,-9.255657331,-3
.4765754042,2.1934831937\H,0,-9.1956770384,-1.992542063,1.2250350426\C
,0,7.2344827077,-2.8805120319,-1.6010937869\H,0,6.817846829,-3.8870646
274,-1.7579443053\H,0,7.0521572948,-2.325182468,-2.5335326557\C,0,8.74
6581825,-2.9851565959,-1.3548470912\H,0,8.9602567802,-3.5662924677,-0.
4478899432\H,0,9.2559650761,-3.475489799,-2.1937937237\H,0,9.195831759
6,-1.9915091353,-1.225274289\\Version=AM64L-G03RevD.01\State=1-A\HF=-9
26.3601634\MP2=-929.8716183\RMSD=7.857e-09\Thermal=0.\PG=C01 [X(C21H38
N2)]\\@

377-Ac⁺

1\1\GINC-NODE26\SP\RMP2-FC\6-31+G(2d,p)\C23H41N2O1(1+)\ZIP08\27-Aug-20
11\0\\#P MP2/6-31+G(2d,p) scf=(direct,tight) int=finegrid geom=check g
uess=read\\sp of d8ap7.ac1 MP2-5/6-31+G(2d,p)\\1,1\C,0,-0.0381534653,-
0.0604995258,-0.0577718022\C,0,-0.0084796152,-0.0637436516,1.304129516
3\C,0,1.2401909269,-0.0020954733,2.0193385593\C,0,2.417217525,0.060271
2927,1.1908491809\C,0,2.3182962786,0.0569830318,-0.1693277103\H,0,-0.9
562872918,-0.097833137,-0.6325834108\H,0,-0.955845853,-0.0933374146,1.
8265955918\H,0,3.4094183225,0.0902293283,1.6219331431\H,0,3.2011229592
,0.0953347735,-0.7942737818\N,0,1.3031949426,-0.0026584315,3.353568036
5\N,0,1.1082207288,-0.0020996584,-0.8131810069\C,0,0.0981635206,-0.143
7506143,4.2007364099\H,0,-0.5958412918,-0.8438249934,3.7240229772\H,0,
0.4274992341,-0.6257149401,5.1275448766\C,0,2.5811810149,0.1365784672,
4.0854407079\H,0,2.3395864659,0.6158973753,5.040190806\H,0,3.228306226
4,0.8384508698,3.5488012483\C,0,-0.5847343948,1.1988477594,4.512997616
7\H,0,0.1444799282,1.8722489592,4.9858619318\H,0,-0.8918249615,1.68422
80827,3.5751481135\C,0,3.2901280601,-1.2067025317,4.3294185535\H,0,3.5
102662249,-1.6887399229,3.365652434\H,0,2.6069133745,-1.8821763422,4.8
638301932\C,0,4.5882785431,-1.0323748951,5.1377275894\H,0,5.2631303907
,-0.3434609887,4.6060936958\H,0,4.3560511434,-0.5536731983,6.101112272
5\C,0,-1.804015262,1.0208139721,5.4353104681\H,0,-2.5256345729,0.33648
31258,4.9626503677\H,0,-1.4858099192,0.5353892224,6.3704218538\C,0,-2.
5023995942,2.3518167858,5.7625493634\H,0,-1.778817108,3.0353377688,6.2
326685966\H,0,-2.8202679683,2.836005551,4.8261655654\C,0,5.3151332199,
-2.3642548541,5.3910345842\H,0,4.6377106923,-3.0525203851,5.9193019019
\H,0,5.5477418616,-2.8412594011,4.4262453992\C,0,6.6103966835,-2.19928
36499,6.2038875129\H,0,6.3751601018,-1.7255475673,7.1695201463\H,0,7.2
847562039,-1.5056330113,5.6777266701\C,0,-3.7194051709,2.1820607898,6.
6875765359\H,0,-3.3992444791,1.6991626519,7.6238557262\H,0,-4.44025055
21,1.4945097517,6.2182246609\C,0,-4.4213789858,3.5105896264,7.01359991
6\H,0,-3.700011475,4.198893885,7.4813077047\H,0,-4.7422398993,3.992994
528,6.0769003412\C,0,7.3412584327,-3.5288576529,6.4531314051\H,0,7.577
5740518,-4.0017972046,5.4868829098\H,0,6.6659808547,-4.2233314913,6.97
70820298\C,0,-5.6375041372,3.3439558313,7.9401936603\H,0,-6.3563792568
,2.6538263856,7.4735335327\H,0,-5.3158818353,2.8646700038,8.876962863\
C,0,-6.3355510373,4.6739690111,8.2578010439\H,0,-6.7004982468,5.158714
973,7.3427555865\H,0,-7.1954786937,4.5226981988,8.9211099334\H,0,-5.64
96265183,5.373076055,8.7539846712\C,0,8.6353251523,-3.3677865116,7.268
488144\H,0,8.398076127,-2.898922732,8.2351865061\H,0,9.3080029139,-2.6
708222109,6.7463408566\C,0,9.3616975036,-4.6989034032,7.5084447557\H,0
,10.27756831,-4.5518392656,8.0931298196\H,0,8.7245617789,-5.4047733858
,8.0572618343\H,0,9.6435908577,-5.1735533809,6.5593669525\C,0,0.959829
8917,-0.0051686355,-2.2805071737\O,0,-0.1521544341,-0.0623561609,-2.72

86989842\C,0,2.2261407759,0.0648319286,-3.096899459\H,0,2.8770925151,-
0.7967667197,-2.904298079\H,0,1.9328230381,0.0591655067,-4.1481156836\
H,0,2.789305014,0.9826855466,-2.8886603559\\Version=AM64L-G03RevD.01\S
tate=1-A\HF=-1078.5529514\MP2=-1082.5342746\RMSD=7.366e-10\Thermal=0.\
PG=C01 [X(C23H41N2O1)]\\@

378
see reference 9

378-Ac^{+}
see reference 9

379
1\1\GINC-NODE12\SP\RHF\6-31G(d)\C19H30N2\ZIP08\01-Dec-2010\0\\#P
RHF/6-31G(d) scf=tight int=finegrid SCRF=(PCM,Read,Solvent=Chloroform)
geom=check guess=read\\PCM of TCAP41 b98/6-31G(d) structures on
4p 900MB\ \0,1\C,0,1.2431083136,-2.611555249,-0.3092960538\C,0,1.2517
259498,-1.2 323019151,-.0725371854\C,0,0.0067490815,-0.5265574094,-0.0
642132881\C ,0,-1.1541532578,-1.3670351543,-0.1205088033\C,0,-0.99519
15554,-2.7343 935439,-0.3405874598\N,0,0.1685105968,-3.3816326501,-0.4
777101281\N,0 -0.0714311601,0.874906906,0.020434817\C,0,-1.393678764
8,1.5870193454,- 0.1039753838\C,0,-2.5085520614,0.6005595252,-0.542257
7947\C,0,-2.54493 64562,.7896781309,0.1008122781\C,0,2.5952763947,-0.58
0176756,0.25190 12857\C,0,2.3033778977,0.850199188,0.7253280274\C,0,1.
2181263806,1.646 8248083,-0.0407471388\H,0,-1.8813501104,-3.3609830363,
-0.4154419373\H, 0,2.1989236336,-3.133184321,-0.3625556554\H,0,-3.468945
9488,1.11345953 3,-0.3980833043\H,0,3.2312021088,1.4370382887,0.7203795
681\H,0,1.96905 77454,0.7984295713,1.7718582926\H,0,-2.4020547853,0.435
7106807,-1.6242 956201\C,0,-3.6679814999,-1.5818164148,-0.6124940638\H,
0,-3.8515423351 ,-2.5550512944,-0.1436796739\H,0,-4.6071123808,-1.015197
2571,-0.556265 0773\H,0,-3.4322965345,-1.7494652331,-1.6704458573\C,0,-2.
8677512398,- 0.7778860257,1.617785937\H,0,-3.8083057824,-0.2462052957,1.8
190544512\ H,0,-2.9748361009,-1.8084890777,1.9777974441\H,0,-2.07361040
52,-0.3120 644947,2.2067526905\C,0,3.580751751,-0.6427932967,-0.94536344
12\H,0,4. 5076756808,-0.1023842563,-0.7067988786\H,0,3.8510699487,-1.6841
681888, -1.1586770755\H,0,3.1645318716,-0.2222770946,-1.8632529783\C,0,3.2
7740 50695,-1.309224828,1.4451345244\H,0,3.582849662,-2.3282382451,1.1839
554476\H,0,4.1791769319,-0.7618102001,1.7530378124\H,0,2.6014563167,-1.3
701833123,2.306552075\C,0,1.6469264526,1.911298613,-1.5129454548\H,0,1 .0
968861315,2.7483376762,-1.9510556613\H,0,2.7139180713,2.1589752085,- 1.5
624498534\H,0,1.4688589822,1.0307902463,-2.1383192094\C,0,1.1804788818,3
.0018821958,0.7143825111\H,0,2.1916649358,3.4256286226,0.6811352167\H,0,
0.5161685938,3.7465950296,0.2737009601\H,0,0.9099987758,2.86884 88344,1.
7669053992\C,0,-1.423752643,2.6547407547,-1.2339480461\H,0,-0. 7867434699
,3.5205600252,-1.0516879514\H,0,-1.1547062154,2.2162400882,- 2.2008017867\
H,0,-2.4518296662,3.0313626857,-1.3135315863\C,0,-1.79185 64107,2.28179446
82,1.2339149564\H,0,-2.8769721126,2.4353380109,1.27129 33659\H,0,-1.50377
02125,1.6800494028,2.0993262287\H,0,-.3253530888,3.2640165635,1.3442078928
\\Version=AM64L-G03RevD.01\State=1-A\HF=-845.83 80641\RMSD=3.951e-
09\Thermal=0.\Dipole=-0.1477124,2.0352236,0.2143952\ PG=C01 [X(C19H30N2)]\\@

379-Ac^{+}
1\1\GINC-NODE15\SP\RHF\6-31G(d)\C21H33N2O1(1+)\ZIP08\02-Dec-2010\0\\#P
RHF/6-31G(d) scf=tight int=finegrid SCRF=(PCM,Read,Solvent=Chloroform
) geom=check guess=read\\PCM of TCAP41.ac2 b98/6-31G(d) structures on
4p 900 MB\\1,1\C,0,-0.0584844034,-0.1099494849,0.0498907806\C,0,-0.029
5343966,0.0112334281,1.4141425064\C,0,1.2571891839,-0.0045637708,2.097
7898637\C,0,2.4081004628,0.2161884107,1.2330494635\C,0,2.2729598581,0.

0852127859,-0.1190059556\N,0,1.0660220387,-0.1508537342,-0.7244983573\
N,0,1.3696039275,-0.1965795872,3.4330840961\C,0,2.7365921136,-0.334101
6639,4.1105744068\C,0,3.8699472486,-0.3706472336,3.0513402533\C,0,3.75
07463134,0.5746137923,1.8478562488\C,0,-1.3558084604,0.2591392763,2.13
91511486\C,0,-1.0307269994,0.4195632222,3.632911219\C,0,0.0827287263,-
0.4864971469,4.2094192505\H,0,3.1050480921,0.1554782122,-0.8068523893\
H,0,-1.0012542465,-0.155080907,-0.4787195805\H,0,4.8112150702,-0.18979
70276,3.5834623566\H,0,-1.9405303712,0.2559131348,4.2221819656\H,0,-0.
7229101875,1.4580431548,3.8179581957\H,0,3.9346359869,-1.3908653288,2.
6480625397\C,0,4.9478597579,0.3038637942,0.9110148035\H,0,4.9439127203
,0.9646672976,0.0361571062\H,0,5.8803961721,0.4991941406,1.4531884003\
H,0,4.9717031804,-0.7367215915,0.5645402836\C,0,3.7547699015,2.0823347
441,2.2150877102\H,0,4.641735823,2.3292061358,2.8112660326\H,0,3.78385
28884,2.6864647094,1.3006599922\H,0,2.8672673183,2.3842386504,2.779580
8749\C,0,-2.3989592588,-0.8562634758,1.8710019843\H,0,-3.3064829915,-0
.6560499015,2.4537325796\H,0,-2.6964328074,-0.8743317158,0.8150706517\
H,0,-2.0395155139,-1.8541481632,2.1328330823\C,0,-1.9619918252,1.60311
96464,1.6458206373\H,0,-2.2727866815,1.5475827234,0.5954987072\H,0,-2.
8514734966,1.8490592474,2.2391289934\H,0,-1.2456851271,2.4271264259,1.
7471854586\C,0,-0.2509990208,-1.9934700646,4.0852676512\H,0,0.40653869
04,-2.6001832724,4.7122742031\H,0,-1.2798631533,-2.1715275589,4.418434
4251\H,0,-0.1556822939,-2.3509464661,3.0549866236\C,0,0.1340535221,-0.
1007287953,5.7051123286\H,0,-0.8576477633,-0.3142604285,6.1198479567\H
,0,0.8470638838,-0.6752827524,6.2949636204\H,0,0.3265642867,0.96630694
63,5.8481640604\C,0,2.8786540762,-1.6934035041,4.8436967507\H,0,2.2486
734031,-1.7962730632,5.7274339343\H,0,2.6837406108,-2.5325083789,4.167
6648043\H,0,3.9177928806,-1.7762552821,5.1841643607\C,0,2.9729320526,0
.8306028309,5.1149284979\H,0,4.0497899176,0.985404946,5.240317975\H,0,
2.5393594623,1.7727642946,4.7702712137\H,0,2.5658433623,0.6131955351,6
.1038701667\C,0,1.05361605,-0.3564939338,-2.1757298357\O,0,2.102230311
3,-0.3149370968,-2.7624641338\C,0,-0.2868646692,-0.6109837805,-2.82227
1623\H,0,-0.9466924644,0.2607118577,-2.7295338634\H,0,-0.1039464913,-0
.801328696,-3.8812321315\H,0,-0.7918194948,-1.478683115,-2.3816928243\
\Version=AM64L-G03RevD.01\State=1-A\HF=-998.0626155\RMSD=1.981e-09\The
rmal=0.\Dipole=-1.1473145,-0.2043542,0.0082611\PG=C01 [X(C21H33N2O1)]\
\@

380

1\1\GINC-CALYPSO\SP\RHF\6-31G(d)\C27H29N3O1\EVGENY\12-Feb-2010\0\\#P R
HF/6-31G(d) scf=tight int=finegrid SCRF=(PCM,Read,Solvent=Chloroform)
geom=check guess=read\\PCM of para4_21 b98/6-31G(d) structures on 4p 9
00 MB\\0,1\C,0,0.0934539127,-0.0348565539,0.0559401781\C,0,-0.03368696
69,-0.0125932401,1.4538276013\C,0,1.1547243875,0.1045263481,2.18918983
81\C,0,2.3177910985,0.5419409558,1.55738483\C,0,2.3528201712,0.8387748
916,0.1846029721\C,0,1.2782422001,0.3523543783,-0.6042711078\H,0,-0.78
14537801,-0.2590884147,-0.5535267579\H,0,1.1359512856,0.0204544529,3.2
747276644\H,0,3.1840263053,0.7939655668,2.1676195008\C,0,3.3853503726,
1.8456434929,-0.2911851576\H,0,4.3193134898,1.6899576262,0.2626161908\
H,0,3.6160164587,1.7156713223,-1.3540087019\C,0,2.9323630038,3.3802408
559,-0.0562809605\H,0,2.8218742828,3.8549687375,-1.0391061186\H,0,3.75
53380638,3.8968187757,0.4526893536\C,0,-1.3868955401,0.1538081457,2.12
68877753\H,0,-2.17780371,-0.0323476793,1.3906312916\H,0,-1.5293461766,
-0.5735171969,2.9378620853\C,0,-1.5894170546,1.6111207694,2.7524278769
\H,0,-2.6465416446,1.8821143159,2.6722766363\H,0,-1.349389385,1.560790
9511,3.8218247514\C,0,-0.6827614969,2.656945916,2.1232382535\C,0,0.467
511083,3.0824759285,2.8100821026\C,0,-0.7480635967,3.0006615167,0.7568
755664\C,0,1.6188843475,3.5053943843,2.1388561124\H,0,0.5187978546,2.9
189668202,3.8857491739\C,0,0.4056640806,3.4285421817,0.0867864622\C,0,

1.6436753779,3.5242105746,0.7363625879\H,0,2.5356580441,3.6702859755,2
.7029547123\H,0,0.3515507308,3.5662605707,-0.9917048859\C,0,1.28675382
13,0.3001711734,-2.1047756279\C,0,1.3321229875,1.4836764348,-2.8556310
149\C,0,1.1324568282,-0.925940079,-2.8339287279\H,0,1.4736905843,2.422
5178424,-2.3261506421\C,0,0.9331151921,-0.7978133313,-4.2321363273\C,0
,0.9720139234,0.4525967039,-4.8361424715\H,0,0.7741684143,-1.672381333
,-4.8528747967\H,0,0.8314962521,0.5318629578,-5.9146388142\N,0,1.19141
2746,1.6011992938,-4.1830644284\N,0,1.1570425706,-2.1780459829,-2.2565
261375\C,0,1.8827048505,-2.5211709404,-1.0133387848\H,0,2.7660578215,-
1.885658885,-0.8931176011\H,0,1.2448478975,-2.370916591,-0.1337854703\
C,0,0.8941135329,-3.37902034,-3.0598115116\H,0,1.6071959914,-3.4645581
657,-3.8981670063\H,0,-0.1178882474,-3.3488376721,-3.4844222549\C,0,1.
0969004974,-4.5345188316,-2.0714645122\H,0,1.3322883107,-5.4764767107,
-2.5781404573\H,0,0.1897185797,-4.6851608428,-1.4720091181\C,0,2.24053
76868,-4.0074506124,-1.1892303385\H,0,3.1981537663,-4.1098476122,-1.71
68290168\H,0,2.3258105902,-4.5267214291,-0.2286390731\H,0,-1.695728791
5,2.5223735335,-1.0117442214\N,0,-1.8920686254,2.734282267,-0.04074876
53\C,0,-3.2031764577,3.0658994109,0.2520450034\O,0,-3.5681447767,3.496
4138095,1.3329249142\C,0,-4.1654806415,2.8222940933,-0.9095355216\H,0,
-3.9397449177,3.4918980615,-1.749780132\H,0,-5.1820325561,3.0181499983
,-0.5632668622\H,0,-4.0973190951,1.7897819794,-1.2747120961\\Version=A
M64L-G03RevD.01\State=1-A\HF=-1277.4973684\RMSD=2.057e-09\Thermal=0.\D
ipole=0.7676048,-2.3733529,-0.4468047\PG=C01 [X(C27H29N3O1)]\\@

380-Ac^{+}

1\1\GINC-GOLEM\SP\RHF\6-31G(d)\C29H32N3O2(1+)\EVGENY\25-Feb-2010\0\\#P
RHF/6-31G(d) scf=tight int=finegrid SCRF=(PCM,Read,Solvent=Chloroform
) geom=check guess=read\\PCM of para4_29.ac1 b98/6-31G(d) structures o
n 4p 900 MB\\1,1\C,0,0.1110846385,0.0887980384,-0.2284501205\C,0,0.209
8698721,0.0318182956,1.1755578583\C,0,1.4848780686,0.2233916911,1.7252
878452\C,0,2.4879068838,0.8272719022,0.9660160306\C,0,2.279042775,1.18
43486257,-0.374344986\C,0,1.1542141286,0.6050661904,-1.0171229458\H,0,
-0.8195688484,-0.1978346882,-0.7164909297\H,0,1.6458505807,0.069974651
4,2.7908129991\H,0,3.3978150785,1.156868292,1.4642379951\C,0,3.0619820
885,2.3573860049,-0.9416887066\H,0,4.034303086,2.4089162314,-0.4384332
796\H,0,3.2645259609,2.2532693032,-2.0143989095\C,0,2.3126225381,3.765
4714084,-0.7212813405\H,0,3.0847885466,4.5136218799,-0.5041276946\H,0,
1.8468702132,4.0560042274,-1.6712625711\C,0,-1.0168626358,0.0120811584
,2.0759684848\H,0,-1.9100453749,-0.1461311133,1.4604858748\H,0,-0.9783
882703,-0.8123586969,2.7999779622\C,0,-1.1880428268,1.3674088817,2.905
963983\H,0,-2.2583066574,1.4889373653,3.1241622926\H,0,-0.6970669154,1
.2503288806,3.8787094467\C,0,-0.5554886104,2.5588023462,2.1970958846\C
,0,0.6842651929,3.0551738747,2.6213024538\C,0,-1.0039847077,2.97815469
71,0.9241895054\C,0,1.5789577674,3.647738264,1.723490827\H,0,1.0198993
185,2.8363192844,3.6336123696\C,0,-0.1016678252,3.5404146048,0.0140381
217\C,0,1.2477405165,3.7284463806,0.3640733817\H,0,2.5844856987,3.8965
301532,2.0574763084\H,0,-0.4335599465,3.7446619075,-0.9969466426\C,0,1
.0764721234,0.5980185884,-2.5192629045\C,0,0.2490205674,1.5004659841,-
3.1496827437\C,0,1.8945706631,-0.2852574104,-3.3414917485\H,0,-0.42314
26472,2.1175317373,-2.5632498129\C,0,1.834733699,-0.0545212341,-4.7607
241445\C,0,0.9947238584,0.8690036244,-5.3010598167\H,0,2.4370792993,-0
.6387165161,-5.4435097315\H,0,0.9038638156,1.0419976614,-6.3667410883\
N,0,0.1891145688,1.6451907491,-4.5146862118\N,0,2.6726328932,-1.278354
5691,-2.8856330601\C,0,2.6922417069,-1.8679868378,-1.517716951\H,0,3.2
997841385,-1.2430399986,-0.8549735826\H,0,1.6810906576,-1.9144795399,-
1.1116529794\C,0,3.5622706108,-2.0526147048,-3.7934118828\H,0,4.214597
3462,-1.3739270608,-4.3538894879\H,0,2.9514208777,-2.6218731035,-4.508
1027259\C,0,4.3362051257,-2.9912960287,-2.8592341767\H,0,4.6545965029,

-3.9016793716,-3.3752822945\H,0,5.2310310121,-2.4883569838,-2.47277048
36\C,0,3.3309351349,-3.2473783881,-1.7268512283\H,0,3.797096344,-3.617
5303997,-0.8089432532\H,0,2.5735882329,-3.975855596,-2.0427020826\H,0,
-2.9920094556,2.7176319323,1.3944589243\N,0,-2.364036562,2.718892088,0
.6018918992\C,0,-2.943789721,2.5009745445,-0.6163201647\O,0,-2.3087083
657,2.4353025179,-1.6689966037\C,0,-4.4571658706,2.3560285565,-0.59923
37542\H,0,-4.9018711713,3.2476179448,-1.0576670883\H,0,-4.8798692445,2
.2339608036,0.4032499154\H,0,-4.7356303948,1.4962762093,-1.2161939974\
C,0,-0.6696002607,2.6298637384,-5.2116423102\O,0,-0.7210471943,2.56370
9374,-6.4106309848\C,0,-1.3732656159,3.6510002239,-4.3649365263\H,0,-2
.0719590364,3.1880261853,-3.6582426342\H,0,-0.6498345506,4.2339164915,
-3.7805509805\H,0,-1.9096057706,4.3170162707,-5.0432901199\\Version=AM
64L-G03RevD.01\State=1-A\HF=-1429.7266402\RMSD=5.811e-10\Thermal=0.\Di
pole=0.401866,-1.4726041,-0.4380999\PG=C01 [X(C29H32N3O2)]\\@

381

1\1\GINC-GOLEM\SP\RHF\6-31G(d)\C15H22N2\RAMAN\18-Nov-2010\0\\#P RHF/6-
31G(d) scf=tight int=finegrid SCRF=(PCM,Read,Solvent=Chloroform) geom=
check guess=read\\PCM of TCAP2 b98/6-31G(d) structures on 4p 900 MB\\0
,1\N,0,-0.0030182346,-0.0949218456,-0.0773875369\N,0,0.0749150485,0.03
36611878,4.2033976821\C,0,1.3534026827,-0.2012928336,-0.7249615472\C,0
,2.2289063965,0.9364239183,-0.1345073152\C,0,2.4560970376,0.7596479758
,1.3630897071\C,0,1.1743528803,0.3502392578,2.0523310245\C,0,1.1245416
692,0.371610211,3.4487820011\C,0,-1.0042346173,-0.3578427832,3.5147214
206\C,0,-1.0938547274,-0.4420886678,2.1268595325\C,0,-2.3714618453,-0.
8830280151,1.4668386861\C,0,-2.4877010442,-0.1343024646,0.1456201787\C
,0,-1.2854190432,-0.3795073483,-0.8058657319\C,0,0.0210585479,-0.06454
02722,1.3285228619\H,0,3.1897906824,0.9671243216,-0.664600695\H,0,-2.3
808583925,-1.970344412,1.2956420036\H,0,-1.8775178686,-0.6346227951,4.
1091604394\H,0,2.0147606531,0.6990991774,3.9907188691\H,0,2.8433225144
,1.6889005524,1.8029149409\H,0,-3.2312289026,-0.6649571237,2.113728287
5\H,0,3.2319494887,-0.0020578049,1.5349467063\H,0,1.7154254558,1.88687
24473,-0.3320051916\H,0,-2.5614972839,0.9428919203,0.3514496129\H,0,-3
.3970659721,-0.423852686,-0.3956577055\C,0,1.3877881384,0.0058914015,-
2.2521312239\H,0,2.4337869994,-0.0980206231,-2.5655952938\H,0,0.815807
698,-0.7388629949,-2.8112305829\H,0,1.0568381862,1.0072965573,-2.54337
13291\C,0,1.978217083,-1.589639554,-0.4242371751\H,0,1.4576291373,-2.3
785073048,-0.9786746679\H,0,3.0341237962,-1.606072413,-0.7257643962\H,
0,1.9240107353,-1.8377544758,0.6405975313\C,0,-1.5308356176,0.60875813
3,-1.9809046718\H,0,-1.2639511779,1.6288719419,-1.6818930893\H,0,-0.99
13931137,0.3558117708,-2.8940339438\H,0,-2.5989622591,0.6002093897,-2.
2315039297\C,0,-1.3495125481,-1.8395677454,-1.3203842032\H,0,-2.331076
9467,-2.0333714432,-1.7732164638\H,0,-0.5925899171,-2.0416342527,-2.08
43581562\H,0,-1.2002876769,-2.5567214772,-0.5057309018\\Version=AM64L-
G03RevD.01\State=1-A\HF=-689.7257218\RMSD=1.014e-09\Thermal=0.\Dipole=
-0.0330992,-0.1506185,-2.0195182\PG=C01 [X(C15H22N2)]\\@

381-Ac$^+$

1\1\GINC-YANG\SP\RHF\6-31G(d)\C17H25N2O1(1+)\RAMAN\28-Nov-2010\0\\#P R
HF/6-31G(d) scf=tight int=finegrid SCRF=(PCM,Read,Solvent=Chloroform)
geom=check guess=read\\PCM of TCAP1.ac2 b98/6-31G(d) structures on 2p
512 MB\\1,1\C,0,-0.0230801612,0.0095197629,0.0213034859\C,0,-0.0044547
029,0.016227727,1.3879738665\C,0,1.2538744303,0.0043238029,2.110181165
9\C,0,2.4354077711,-0.0248429862,1.2676009352\C,0,2.3182689339,-0.0529
292873,-0.0950627226\N,0,1.1129632343,-0.0307676322,-0.7397411009\N,0,
1.3219727544,0.0189310667,3.4660296913\C,0,2.6788778723,-0.1368075621,
4.1644538209\C,0,3.6766814385,-0.8238197331,3.2010123084\C,0,3.8084320
586,-0.0782638506,1.8818608114\C,0,-1.3100505469,0.0895277858,2.132744

2226\C,0,-1.0476176492,0.8602114169,3.4173998279\C,0,0.0392741285,0.18
96460314,4.2917209544\H,0,3.2022866235,-0.0887197787,-0.7195788635\H,0
,-0.9458943207,0.0299019548,-0.5478135852\H,0,4.6435391381,-0.88188146
1,3.7113984035\H,0,-1.9592026971,0.931926958,4.0196035751\H,0,-0.73930
74095,1.8862751433,3.1743308731\H,0,3.3491450634,-1.8547917476,3.00955
51126\H,0,4.2038140426,0.9334610667,2.0440558529\H,0,4.508520159,-0.58
79788918,1.209793589\H,0,-1.6914335401,-0.9168416816,2.3514468349\H,0,
-2.0688566894,0.5890899992,1.5201317138\C,0,0.2151316374,1.1512902858,
5.4894967026\H,0,-0.7674082945,1.2597005343,5.9628961174\H,0,0.8975104
089,0.7975946617,6.2618031724\H,0,0.5338212968,2.1454673243,5.15973129
\C,0,-0.4710098251,-1.1923706187,4.7718384148\H,0,0.1168021901,-1.5907
448563,5.6000082771\H,0,-1.5042997492,-1.0898651353,5.1248941381\H,0,-
0.4597832804,-1.9328061093,3.9648329242\C,0,2.624513063,-1.0774618227,
5.3904677382\H,0,2.0242406199,-0.7095661637,6.2218844124\H,0,2.2730947
689,-2.0762225389,5.1119300196\H,0,3.6497880229,-1.1801515916,5.764030
1873\C,0,3.2321019321,1.2537524848,4.5670064515\H,0,2.7272354483,1.667
528483,5.4410953975\H,0,4.2952400274,1.1579631051,4.8190138682\H,0,3.1
394739627,1.9789570798,3.7513434679\C,0,0.9627940201,-0.0464602013,-2.
2025102078\O,0,-0.1502768123,-0.0201645456,-2.6534283371\C,0,2.2296259
644,-0.094919501,-3.0207576682\H,0,2.8117684556,-0.9995346428,-2.80706
41728\H,0,1.935339341,-0.1018313227,-4.0716724746\H,0,2.8634526221,0.7
803466297,-2.8332897951\\Version=AM64L-G03RevD.01\State=1-A\HF=-841.94
87188\RMSD=2.660e-09\Thermal=0.\Dipole=1.2210602,-0.0415682,-0.4659468
\PG=C01 [X(C17H25N2O1)]\\@

382

1\1\GINC-SOLARIS\SP\RHF\6-31G(d)\C15H22N2\RAMAN\01-Dec-2010\0\\#P RHF/
6-31G(d) scf=tight int=finegrid SCRF=(PCM,Read,Solvent=Chloroform) geo
m=check guess=read\\PCM of TCAP22 b98/6-31G(d) structures on 4p 900 MB
\\0,1\N,0,-0.0025674685,0.0170093833,-0.0037603066\N,0,0.0544059564,-0
.2895550795,4.2137080199\C,0,1.2610521121,-0.0146479498,-0.726393202\C
,0,2.2901335614,0.8701565027,-0.0282355503\C,0,2.5616135391,0.41830540
78,1.4257394571\C,0,1.2206369941,0.1158413034,2.1145673725\C,0,1.15444
39396,-0.0108550425,3.5038658783\C,0,-1.0691873209,-0.4424799394,3.502
5261768\C,0,-1.1763720988,-0.349391765,2.1131233065\C,0,-2.532346365,-
0.5703496299,1.4226795434\C,0,-2.4478990599,-0.0495237053,-0.031116336
2\C,0,-1.1617225823,-0.485050766,-0.7277982882\C,0,0.0153022042,-0.079
3106316,1.3818245672\H,0,3.2290236757,0.8686967997,-0.5985478185\H,0,-
1.1232629091,-1.5854795604,-0.8292290925\H,0,-1.1256745414,-0.07098460
19,-1.7438690398\H,0,-1.9599411442,-0.6652421227,4.0901018955\H,0,2.06
31713506,0.1156774566,4.0925214562\H,0,1.637180247,-1.0495140175,-0.82
78222764\H,0,1.0738464499,0.3562609967,-1.7425296366\H,0,1.9100016754,
1.901236798,-0.0252602799\H,0,-2.4810635549,1.0488954817,-0.0280282206
\H,0,-3.3174035234,-0.4020120808,-0.602506451\C,0,3.3212178916,1.56190
97729,2.1404617488\H,0,3.6466727874,1.2771415804,3.147371466\H,0,4.220
7986463,1.8265374621,1.568107235\H,0,2.6937325777,2.4573376785,2.22678
30626\C,0,3.4521627326,-0.8505496045,1.4344652089\H,0,4.4220957605,-0.
6434763333,0.9603985878\H,0,3.6386909069,-1.1846376263,2.4622814436\H,
0,2.980023786,-1.6840787409,0.9008453976\C,0,-2.8836311864,-2.08020793
42,1.4307196824\H,0,-2.9333431136,-2.4598989194,2.4584250828\H,0,-3.85
98248072,-2.2509452772,0.9550290168\H,0,-2.1330919265,-2.6765654372,0.
8982827525\C,0,-3.6654085364,0.2059928834,2.1362091191\H,0,-4.59808435
48,0.1147421603,1.562925932\H,0,-3.8616729888,-0.1797833452,3.14295326
8\H,0,-3.4186500323,1.2711741963,2.2226909269\\Version=AM64L-G03RevD.0
1\State=1-A\HF=-689.744899\RMSD=3.961e-09\Thermal=0.\Dipole=0.0060509,
-0.0201583,-1.8912312\PG=C01 [X(C15H22N2)]\\@

382-Ac^{+}

1\1\GINC-YANG\SP\RHF\6-31G(d)\C17H25N2O1(1+)\RAMAN\02-Dec-2010\0\\#P R
HF/6-31G(d) scf=tight int=finegrid SCRF=(PCM,Read,Solvent=Chloroform)
geom=check guess=read\\PCM of TCAP21.ac2 b98/6-31G(d) structures on 2p
512 MB\\1,1\C,0,-0.035535383,0.0143592343,0.027639164\C,0,-0.01386302
22,0.014894543,1.3939270499\C,0,1.2704158123,0.0026971008,2.0611769214
\C,0,2.4673150058,-0.0186861269,1.2461670915\C,0,2.3257132612,-0.03718
08097,-0.1141556829\N,0,1.104170003,-0.0160848297,-0.7354974613\N,0,1.
3506453407,0.0105596565,3.3995029063\C,0,2.6232553726,-0.1318767872,4.
1260280609\C,0,3.7245998138,-0.7190939663,3.2516366315\C,0,3.852692738
,0.0241536669,1.9015661553\C,0,-1.3120842854,-0.0199616744,2.207651699
3\C,0,-1.0280747301,0.7327475925,3.5281815753\C,0,0.1720004112,0.15734
50239,4.2706426932\H,0,3.1886039886,-0.0587366224,-0.7644973318\H,0,-0
.9508734754,0.0262231123,-0.5500209449\H,0,2.4304994155,-0.7849286955,
4.9861453435\H,0,2.9073706339,0.851677447,4.5251251205\H,0,0.463978400
5,0.8193535321,5.09532313\H,0,-0.0574194116,-0.8234432966,4.7098609097
\H,0,4.6725500995,-0.6661529037,3.8001589474\H,0,-1.9040278333,0.67865
83288,4.1854715814\H,0,-0.8516263299,1.7950498936,3.3106939257\H,0,3.5
17878291,-1.7816418791,3.063806401\C,0,4.9324803881,-0.6817774526,1.05
41246221\H,0,5.1462670777,-0.1464792383,0.1208441169\H,0,5.8719937359,
-0.714853874,1.6178350467\H,0,4.6477096712,-1.7128334784,0.8110330576\
C,0,4.2647249924,1.5044280929,2.1194414606\H,0,5.2155954764,1.55544195
85,2.664683782\H,0,4.3990623464,2.0111995196,1.1566035324\H,0,3.515676
2609,2.0705058106,2.6853313456\C,0,-1.6969268575,-1.4981536526,2.48197
55227\H,0,-2.5788692916,-1.542225841,3.1333380072\H,0,-1.9427112337,-2
.0098511992,1.5442063989\H,0,-0.8888108038,-2.0627772057,2.9621321237\
C,0,-2.4814857512,0.6806122412,1.4836345024\H,0,-2.7930912831,0.142925
7753,0.5806025926\H,0,-3.3520435919,0.7138950123,2.148983261\H,0,-2.22
76447406,1.7109198434,1.2059341238\C,0,0.9406812099,-0.0267790174,-2.1
971978002\O,0,-0.1759576497,-0.0027439637,-2.6404565703\C,0,2.19781025
92,-0.0672843887,-3.0321424759\H,0,2.7898293568,-0.9674678558,-2.82741
30044\H,0,1.8886246516,-0.0759636819,-4.0787660924\H,0,2.8276803244,0.
8128592722,-2.854390675\\Version=AM64L-G03RevD.01\State=1-A\HF=-841.97
0051\RMSD=4.943e-09\Thermal=0.\Dipole=1.2822339,-0.0324791,0.704642\PG
=C01 [X(C17H25N2O1)]\\@

383

1\1\GINC-NODE-04\SP\RMP2-FC\6-31+G(2d,p)\C18H16F6N4O1\ZIP01\13-Jan-201
0\0\\#P MP2/6-31+G(2d,p) scf=(direct,tight) int=finegrid geom=check gu
ess=read\\sp of cat81ur1f_5c1 MP2-5/6-31+G(2d,p)\\0,1\C,0,-1.707463976
3,-1.3003076531,0.7902176259\N,0,-1.8959180602,-2.0002382158,-0.429531
2812\C,0,-2.3718887456,-0.0507144407,0.9234148867\C,0,-0.9750516837,-1
.8088034236,1.8631995924\C,0,-3.2571704777,-1.8888683125,-0.9519741503
\C,0,-3.5445624476,-0.4107880717,-1.2332833359\H,0,-3.3322025863,-2.48
52087968,-1.8627335429\H,0,-3.9614408896,-2.2874722984,-0.2106458116\N
,0,-3.1330681718,0.4403924183,-0.1144002333\H,0,-4.6228748031,-0.27896
57416,-1.4099567729\H,0,-3.0233562125,-0.0997003853,-2.152592169\C,0,-
3.6580193998,1.8043526338,-0.1111614159\C,0,-5.0254617766,1.9474425259
,0.575835114\H,0,-3.7323662407,2.1219922217,-1.1598653558\H,0,-2.91411
8806,2.4625764825,0.3509540611\H,0,-5.3715738289,2.987027325,0.5207702
987\H,0,-5.7795272088,1.3153736039,0.0904555667\H,0,-4.9723568628,1.65
74220719,1.6312888785\C,0,-2.16833274,0.6271951033,2.1444531061\C,0,-1
.3688355438,0.0418706925,3.1235584015\H,0,-2.6229693333,1.59356496,2.3
352162645\N,0,-0.7801278355,-1.1582491325,3.0164145022\H,0,-1.20713187
86,0.5696304927,4.0631803974\H,0,-0.5026022984,-2.7853011275,1.7666176
39\C,0,-0.9614025257,-2.7335096196,-1.1399074868\O,0,-1.2748038921,-3.
5809120157,-1.9635696937\N,0,0.3934221959,-2.4934634598,-0.8539092031\
H,0,0.9612940461,-3.2353211532,-1.2463582417\C,0,1.0565986535,-1.26349
76658,-0.6725836538\C,0,0.4477571577,-0.0263982134,-0.9309071612\C,0,2

.3904958691,-1.2846083133,-0.2345937898\C,0,1.1487246478,1.1595153683,
-0.7076833881\H,0,-0.5675085604,0.0173010959,-1.303647942\C,0,3.090394
332,-0.0909027654,-0.0522378517\H,0,2.8732783522,-2.2338319712,-0.0193
375137\C,0,2.4751390429,1.1448699118,-0.2722402801\H,0,3.016369902,2.0
700442195,-0.1071905759\C,0,4.5481795339,-0.1356934996,0.3492253842\F,
0,5.3537292668,-0.1219149574,-0.7329797525\F,0,4.8421165971,-1.2494902
181,1.0459190697\F,0,4.8871600577,0.9235678732,1.1069114169\C,0,0.4253
867595,2.47535505,-0.8826287752\F,0,-0.1890957251,2.8548643512,0.26045
2971\F,0,-0.5341025854,2.401481268,-1.8293171428\F,0,1.2624636521,3.46
92714626,-1.2278566795\\Version=IA32L-G03RevD.01\State=1-A\HF=-1580.38
16131\MP2=-1585.0855729\RMSD=7.881e-09\Thermal=0.\PG=C01 [X(C18H16F6N4
O1)]\\@

383-Ac^{+}

1\1\GINC-NAUTILUS\SP\RHF\6-31G(d)\C20H19F6N4O2(1+)\EVGENY\20-Jan-2010\
0\\#P RHF/6-31G(d) scf=tight int=finegrid SCRF=(PCM,Read,Solvent=Chlor
oform) geom=check guess=read\\PCM of cat81ur1f_5c1.ac2 b98/6-31G(d) st
ructures on 4p 900 MB\\1,1\C\N,1,1.3981282\C,1,1.44906481,2,117.275859
77\C,1,1.37132861,2,123.08344897,3,179.1282029,0\C,2,1.4660295,1,113.8
6687803,4,142.93556145,0\C,5,1.52636521,2,108.24604901,1,59.49047887,0
\H,5,1.09118174,2,108.27054185,1,179.96748622,0\H,5,1.09737654,2,110.0
2691228,1,-62.02859972,0\N,3,1.33876729,1,120.58811771,4,-177.7422787,
0\H,6,1.09668476,5,110.04280768,2,-168.36011906,0\H,6,1.09827282,5,110
.90245559,2,72.36282796,0\C,9,1.47798198,3,122.75924682,1,-172.9400994
1,0\C,12,1.53374329,9,113.1788375,3,-87.819048,0\H,12,1.09540078,9,106
.07643133,3,150.97502163,0\H,12,1.09395351,9,108.53504977,3,37.2909715
1,0\H,13,1.09555942,12,109.44804121,9,-177.99609301,0\H,13,1.096077,12
,111.3012747,9,-58.8949935,0\H,13,1.09592988,12,111.82995502,9,62.1234
5005,0\C,3,1.42916844,1,116.20113678,4,3.24091762,0\C,19,1.36747322,3,
120.93551918,1,-0.8391017,0\H,19,1.08244641,3,121.03042721,1,178.73617
851,0\N,20,1.36632896,19,121.50856799,3,-0.876504,0\H,20,1.08188187,19
,121.41569122,3,179.37340082,0\H,4,1.08247818,1,122.96155468,2,-0.2530
4968,0\C,2,1.41337633,1,127.61171465,4,-30.11252026,0\O,25,1.21386649,
2,120.90292602,1,153.26731797,0\N,25,1.39131641,2,116.88658135,1,-26.2
0125387,0\H,27,1.01504573,25,110.59764421,2,169.90437352,0\C,27,1.4190
3322,25,128.21756925,2,-36.04112605,0\C,29,1.39970811,27,121.78163559,
25,-21.62645177,0\C,29,1.40322902,27,118.80568013,25,157.83035633,0\C,
30,1.3965958,29,120.00933848,27,175.84284838,0\H,30,1.0843606,29,120.3
2021442,27,-3.75498575,0\C,31,1.39701101,29,119.87175774,27,-178.87763
349,0\H,31,1.08661836,29,120.04874564,27,0.27150603,0\C,32,1.39568679,
30,120.92348822,29,3.7446709,0\H,36,1.08479727,32,120.69722245,30,179.
1357884,0\C,34,1.51778085,31,119.53520174,29,-177.00136931,0\F,38,1.34
070906,34,111.0691907,31,90.89837142,0\F,38,1.34549284,34,111.23753586
,31,-29.07070121,0\F,38,1.34496773,34,111.0817401,31,-149.20542598,0\C
,32,1.51245495,30,117.99533886,29,-172.30498441,0\F,42,1.36314957,32,1
09.88383523,30,77.63393035,0\F,42,1.34437357,32,111.77702203,30,-40.17
607248,0\F,42,1.33488988,32,112.69635521,30,-162.76730965,0\C,22,1.478
29256,20,123.38046502,19,179.31716625,0\O,46,1.2000287,22,117.6321607,
20,-176.81727111,0\C,46,1.50799483,22,117.00852855,20,3.26053601,0\H,4
8,1.09694477,46,111.42116,22,-63.35360917,0\H,48,1.09648995,46,111.648
54752,22,58.34511479,0\H,48,1.09142441,46,107.1584214,22,177.73546964,
0\\Version=AM64L-G03RevD.01\State=1-A\HF=-1732.4930154\RMSD=8.475e-09\
Thermal=0.\Dipole=2.3829323,-3.6463745,-2.1382732\PG=C01 [X(C20H19F6N4
O2)]\\@

384

1\1\GINC-NODE10\SP\RMP2-FC\6-31+G(2d,p)\C31H32N4O2\ZIP01\22-Dec-2010\0
\\#P MP2/6-31+G(2d,p) scf=(direct,tight) int=finegrid geom=check guess

=read\\sp of cat81ur8_19 MP2-5/6-31+G(2d,p)\\0,1\C,0,0.082968353,-0.28
12497279,-0.0556491015\C,0,-0.0435207977,-0.170751815,1.3305321595\C,0
,1.088555393,0.2106089653,2.1098643404\C,0,2.2616868119,0.5056171078,1
.3778979874\C,0,2.2462537158,0.4082555778,-0.0115141141\H,0,-0.7781889
062,-0.6167266277,-0.6358090126\H,0,3.1790895113,0.797831682,1.8785682
305\H,0,3.1525497084,0.6449003593,-0.5691476502\C,0,-1.0061632053,-1.1
451692132,3.3003053714\H,0,-1.9651811343,-1.3755793262,3.7647774738\H,
0,-0.4576462622,-2.0762690786,3.1172355976\C,0,-0.1916922868,-0.195821
4859,4.1827518541\H,0,0.1220202685,-0.7172735074,5.0984934522\H,0,-0.8
183184433,0.6594661157,4.4811547637\N,0,1.0046653106,0.2699673916,3.47
74217922\N,0,-1.2545769159,-0.4984872381,2.0083281606\N,0,1.1926958722
,0.0209053708,-0.7448621404\C,0,2.0624444438,0.8517918217,4.2987120877
\H,0,2.5413976325,1.6636564118,3.7387213561\H,0,1.5774717659,1.3240829
362,5.1632110834\C,0,3.1089045664,-0.1689698572,4.7726923535\H,0,3.624
2745424,-0.6301838297,3.922301745\H,0,2.6392898884,-0.9698136125,5.357
0229411\H,0,3.8586637007,0.318800399,5.4081882838\C,0,-2.4157890453,0.
2824194617,1.9289718617\O,0,-3.3652861161,0.0513488774,2.6923943786\H,
0,-1.5730851389,1.4044551372,0.4677457689\N,0,-2.4236662054,1.26559602
91,0.993497941\C,0,-3.5257568448,2.2026565421,0.7422648013\H,0,-3.1045
7289,2.9247443282,0.0346195459\C,0,-4.7188696662,1.5029239164,0.039783
3637\H,0,-5.0949932288,0.7102699848,0.6934449634\H,0,-5.5242382944,2.2
388743526,-0.0690968313\C,0,-3.5348284019,-0.0778196817,-3.821780538\C
,0,-3.87919525,1.2711468377,-3.6866325866\C,0,-4.2734752574,1.77317124
92,-2.4432435173\C,0,-4.3308503019,0.9406810138,-1.3133261742\C,0,-3.9
889690763,-0.4126190585,-1.4640644465\C,0,-3.5931640884,-0.9193688679,
-2.7066158864\H,0,-3.2281555462,-0.4704641227,-4.7886133432\H,0,-3.844
7382051,1.9318701682,-4.5504297983\H,0,-4.5454319555,2.8239683152,-2.3
457862559\H,0,-4.0413801305,-1.0745738117,-0.6009872277\H,0,-3.3354239
665,-1.9720017869,-2.8037511864\C,0,-3.9969116413,3.0192955724,2.01827
13113\C,0,-0.6758023311,4.2166040935,4.608417851\C,0,-1.9124829862,3.8
383769776,5.1405465512\C,0,-2.9585716184,3.456099248,4.2950210758\C,0,
-2.7943867313,3.4433832683,2.901104066\C,0,-1.5475863956,3.8291236,2.3
789851692\C,0,-0.4990863192,4.2117021897,3.2214698935\H,0,0.1381170208
,4.5174383888,5.2649327483\H,0,-2.0680719354,3.8437895277,6.2176520513
\H,0,-3.9176277872,3.1567790026,4.7061604874\H,0,-1.3723783378,3.82773
83529,1.3056355345\H,0,0.4564313547,4.5024723386,2.7895159356\C,0,-6.2
026490316,6.5365519206,0.6526189798\C,0,-4.8395003665,6.4104585626,0.3
678805387\C,0,-4.1291190832,5.2917667104,0.8100378708\C,0,-4.765342034
5,4.2734103639,1.539302256\C,0,-6.1328503418,4.4102723091,1.821270094\
C,0,-6.8432482807,5.5330247834,1.3829632542\H,0,-6.7562340801,7.408565
9633,0.3114737322\H,0,-4.3236435936,7.1862077431,-0.194123682\H,0,-3.0
65165562,5.2305072425,0.594729251\H,0,-6.6319166551,3.6305778344,2.385
6625072\H,0,-7.9029386183,5.6191067172,1.6147003551\O,0,-4.9082972205,
2.2524108941,2.7742952624\H,0,-4.4800310992,1.3762283328,2.9069829582\
\Version=AM64L-G03RevD.01\State=1-A\HF=-1560.2267683\MP2=-1565.78558\R
MSD=6.811e-09\Thermal=0.\PG=C01 [X(C31H32N4O2)]\\@

384-Ac$^+$

1\1\GINC-CIPCLU09\SP\RHF\6-31G(d)\C33H35N4O3(1+)\C2253\24-Jan-2011\0\\
#P RHF/6-31G(d) scf=tight int=finegrid SCRF=(PCM,Read,Solvent=Chlorofo
rm) geom=check guess=read\\PCM of cat81ur8_58.ac2 b98/6-31G(d) structu
res on 4p 900 MB\\1,1\C,0,2.11967,2.00623,0.53563\C,0,2.5212,0.70891,0
.73078\C,0,3.45371,0.09961,-0.20375\C,0,3.80404,0.89143,-1.34447\C,0,3
.31666,2.15941,-1.50036\H,0,1.47131,2.52434,1.23165\H,0,4.452,0.50252,
-2.119\H,0,3.5756,2.75809,-2.36345\C,0,3.13611,-0.94538,2.33024\H,0,3.
99711,-0.36507,2.68217\H,0,2.71663,-1.50644,3.16613\C,0,3.53901,-1.896
02,1.20545\H,0,2.70781,-2.56765,0.95455\H,0,4.39327,-2.50043,1.51916\N
,0,3.93119,-1.13269,-0.00065\N,0,2.10571,-0.02515,1.84741\N,0,2.48899,

2.72947,-0.57338\C,0,4.76516,-1.77735,-1.05039\H,0,4.175,-1.786,-1.976
42\H,0,5.6469,-1.14478,-1.21685\C,0,5.21897,-3.20287,-0.73676\H,0,5.89
795,-3.25114,0.12077\H,0,4.37209,-3.8756,-0.56745\H,0,5.76929,-3.57303
,-1.60874\C,0,0.75037,-0.40588,2.12046\O,0,0.52746,-1.21287,3.00722\H,
0,0.05666,0.6457,0.49144\N,0,-0.20879,0.19843,1.36144\C,0,-1.56151,-0.
36682,1.30773\H,0,-1.56827,-1.20079,2.01325\C,0,-2.62009,0.65325,1.820
59\H,0,-3.58902,0.14465,1.86611\H,0,-2.33396,0.88306,2.85488\C,0,-3.06
218,4.36346,-0.39933\C,0,-2.04056,4.2264,0.54686\C,0,-1.89842,3.03218,
1.26\C,0,-2.77163,1.95149,1.04379\C,0,-3.80716,2.11056,0.1027\C,0,-3.9
4764,3.30396,-0.61658\H,0,-3.17983,5.29517,-0.94749\H,0,-1.35905,5.051
42,0.73885\H,0,-1.11986,2.94474,2.01553\H,0,-4.51765,1.30064,-0.05498\
H,0,-4.76047,3.40842,-1.33158\C,0,-1.76154,-0.90586,-0.15814\C,0,1.128
37,-4.04661,-1.11054\C,0,0.3323,-4.14408,0.03709\C,0,-0.582,-3.13427,0
.34902\C,0,-0.71667,-2.00293,-0.47353\C,0,0.08903,-1.91102,-1.61964\C,
0,1.00144,-2.92511,-1.93601\H,0,1.81511,-4.84947,-1.37124\H,0,0.40712,
-5.01591,0.68286\H,0,-1.19748,-3.24722,1.23694\H,0,-0.0192,-1.05388,-2
.27546\H,0,1.59102,-2.84939,-2.84835\C,0,-5.70673,-2.57337,-0.9766\C,0
,-5.13452,-2.73299,0.28867\C,0,-3.87701,-2.18983,0.56704\C,0,-3.1714,-
1.47268,-0.41409\C,0,-3.75015,-1.32906,-1.68525\C,0,-5.00847,-1.87109,
-1.96269\H,0,-6.6855,-2.99457,-1.19189\H,0,-5.66624,-3.27919,1.06409\H
,0,-3.45747,-2.3312,1.56046\H,0,-3.20774,-0.79582,-2.46089\H,0,-5.4396
3,-1.74727,-2.95341\O,0,-1.46874,0.17172,-1.05349\H,0,-2.17473,0.83471
,-0.95339\C,0,1.98687,4.11108,-0.67646\O,0,1.31387,4.53172,0.22468\C,0
,2.3665,4.88863,-1.91247\H,0,2.01206,4.39401,-2.8247\H,0,1.89546,5.869
89,-1.83311\H,0,3.4527,5.02231,-1.98516\\Version=AM64L-G03RevD.01\Stat
e=1-A\HF=-1712.2972102\RMSD=5.442e-09\Thermal=0.\Dipole=5.6291515,1.50
18217,-2.709305\PG=C01 [X(C33H35N4O3)]\\@

385

1\1\GINC-NODE-12\SP\RMP2-FC\6-31+G(2d,p)\C17H20N4S1\ZIP01\22-Nov-2009\
0\\#P MP2/6-31+G(2d,p) scf=(direct,tight) int=finegrid geom=check gues
s=read\\sp of cat81ur4_66.sps MP2-5/6-31+G(2d,p)\\0,1\C,0,-0.007194874
,-0.0456580565,0.0131430299\N,0,-0.010379045,-0.0303399224,1.435863886
5\C,0,1.2522296657,-0.0510396302,-0.6551009189\C,0,-1.1782778454,0.053
8053986,-0.743240205\C,0,1.1152395983,0.7270311494,1.9897036168\C,0,2.
4096500277,0.0685143862,1.5134627918\H,0,1.0573470483,1.7687449828,1.6
484118471\H,0,1.0469001636,0.7112214882,3.0779747039\N,0,2.4242361946,
-0.0662813219,0.0582394937\H,0,3.2601865295,0.6954176576,1.8170567482\
H,0,2.524422062,-0.9154101129,1.9930204484\C,0,3.726395373,-0.24516590
46,-0.5761261157\C,0,4.3894107028,1.072163611,-1.0091329562\H,0,4.3679
696541,-0.7687473613,0.1447711324\H,0,3.617244671,-0.9217756448,-1.432
1713325\H,0,3.7762876014,1.5980205312,-1.7498224964\H,0,4.528592053,1.
7408573301,-0.1507880895\H,0,5.374741004,0.8774408976,-1.4509016149\C,
0,1.197737467,-0.0356667053,-2.067623992\C,0,-0.0371024677,0.003784898
4,-2.7088747735\H,0,2.1055854235,-0.0392356369,-2.6618162298\N,0,-1.22
12617552,0.0574147177,-2.0820738978\H,0,-0.0724569744,0.0089322635,-3.
7982405989\H,0,-2.1349078023,0.1086217019,-0.2264501821\C,0,-0.9722511
542,-0.5772441463,2.2501218474\N,0,-1.6948482704,-1.6343501169,1.75493
69157\H,0,-2.4196808414,-1.9000483234,2.4083248462\S,0,-1.3028853002,-
0.0065384657,3.8073192766\C,0,-1.1940049608,-2.7184264985,0.8965717647
\H,0,-1.9354156577,-3.5216611624,0.9949454346\H,0,-1.2034338432,-2.413
4079871,-0.155676904\C,0,2.7205324512,-4.2931846185,1.9066570616\C,0,1
.8097895299,-3.9788458879,2.9224740875\C,0,0.5506828516,-3.462279159,2
.6045871547\C,0,0.1870002105,-3.2471023932,1.2653290115\C,0,1.10668047
03,-3.5596184282,0.2546200555\C,0,2.3648058597,-4.0833496,0.570968743\
H,0,3.6985967339,-4.6988890896,2.1558441573\H,0,2.0784098175,-4.138103
7598,3.9645326707\H,0,-0.1454022973,-3.2080258855,3.4014358171\H,0,0.8
402738676,-3.3817537189,-0.7860392703\H,0,3.0659475388,-4.3232510331,-

0.2257378814\\Version=IA32L-G03RevD.01\State=1-A\HF=-1270.7799942\MP2=
-1274.083967\RMSD=7.066e-09\Thermal=0.\PG=C01 [X(C17H20N4S1)]\\@

385-Ac+

1\1\GINC-NODE15\SP\RMP2-FC\6-31+G(2d,p)\C19H23N4O1S1(1+)\ZIP01\28-Nov-
2009\0\\#P MP2/6-31+G(2d,p) scf=(direct,tight) int=finegrid geom=check
guess=read\\sp of cat81ur4_66.ac2 MP2-5/6-31+G(2d,p)\\1,1\C,0,-0.7580
632562,0.0580982821,0.4927171101\N,0,0.3848112749,-0.4934590299,1.0715
542308\C,0,-0.7892054362,1.4939485735,0.2707512691\C,0,-1.8637285439,-
0.6921531798,0.1651712241\C,0,1.076623882,0.4033808207,2.0062140059\C,
0,1.4422712384,1.6783281099,1.2563587558\H,0,0.4471823464,0.6166956446
,2.8801807788\H,0,1.9813632572,-0.0950294206,2.3555699684\N,0,0.262593
6096,2.2570728548,0.5898106313\H,0,1.8225911833,2.4229539237,1.9652142
287\H,0,2.2172499182,1.4804406097,0.504882521\C,0,0.3271195253,3.70267
41667,0.3010966951\C,0,-0.2658645782,4.5660189648,1.4221107804\H,0,1.3
869650156,3.9375322326,0.1553952445\H,0,-0.1645714302,3.8953636084,-0.
6578455528\H,0,-1.3305503272,4.3557502168,1.5746999909\H,0,0.254794934
7,4.3954277949,2.3713118113\H,0,-0.158609842,5.6255774109,1.1646937574
\C,0,-1.9844945467,2.0209381799,-0.3069344771\C,0,-3.0351615421,1.2066
247859,-0.6317747007\H,0,-2.0872131244,3.0813195981,-0.4986542728\N,0,
-2.9830785348,-0.1380258888,-0.4087236875\H,0,-3.9399488785,1.60060426
63,-1.0747353721\H,0,-1.9111011837,-1.7628205933,0.3187552166\C,0,0.60
06547605,-1.8876163429,1.1670871547\N,0,0.5309831505,-2.5548078394,-0.
0166175375\H,0,0.6339752878,-3.5561974337,0.0978247669\S,0,0.920383636
7,-2.6402819059,2.6146548245\C,0,0.791162489,-2.0133439756,-1.36066619
71\H,0,0.9368115737,-2.8922794994,-1.9997516546\H,0,-0.1010242704,-1.5
005138406,-1.7448614964\C,0,4.2262528941,0.6066966898,-1.7264257821\C,
0,4.3074233134,-0.546051855,-0.9349544659\C,0,3.2017567792,-1.39080859
74,-0.8047572483\C,0,1.9964619648,-1.0874805448,-1.4579592968\C,0,1.92
16254635,0.0684893698,-2.247291749\C,0,3.0315767987,0.9098979324,-2.38
70651999\H,0,5.0921241631,1.2548953887,-1.8364974272\H,0,5.2369086604,
-0.7944930066,-0.4284081757\H,0,3.2777270618,-2.2863639147,-0.19124544
73\H,0,0.9949393285,0.3068659283,-2.768432038\H,0,2.964285959,1.794673
3932,-3.0161461517\C,0,-4.0886124387,-1.0571027067,-0.7463039547\O,0,-
3.9151015628,-2.2308902768,-0.5662262866\C,0,-5.3541502251,-0.43822948
59,-1.2871084805\H,0,-5.7829258191,0.284420851,-0.5826411721\H,0,-5.17
84705695,0.0673762908,-2.2445049702\H,0,-6.0668866807,-1.2498825399,-1
.4432295723\\Version=AM64L-G03RevD.01\State=1-A\HF=-1422.9584083\MP2=-
1426.7421027\RMSD=4.834e-09\Thermal=0.\PG=C01 [X(C19H23N4O1S1)]\\@

386

1\1\GINC-NODE18\SP\RMP2-FC\6-31+G(2d,p)\C18H22N4O1\ZIP01\18-Jan-2010\0
\\#P MP2/6-31+G(2d,p) scf=(direct,tight) int=finegrid geom=check guess
=read\\sp of cat81ur5_51.sps MP2-5/6-31+G(2d,p)\\0,1\C,0,0.7001015871,
-1.4708389543,0.3462889954\N,0,-0.0041614733,-0.9131131461,1.439122841
2\C,0,1.8251504916,-0.7652438445,-0.17737584\C,0,0.3797078173,-2.72455
27719,-0.1840997125\C,0,0.8150709832,-0.0830466387,2.3154142117\C,0,1.
4749946107,1.0094123541,1.4721314795\H,0,0.1667965273,0.3472145373,3.0
799334507\H,0,1.5804673054,-0.6973033481,2.8084518518\N,0,2.2779427657
,0.4044311345,0.4087543767\H,0,0.6998493919,1.6796694926,1.0670237782\
H,0,2.133082719,1.601264042,2.1163969437\C,0,3.220271767,1.246458254,-
0.3362389114\C,0,3.8142572962,2.4048435355,0.4707558717\H,0,2.73677172
66,1.6442752393,-1.2459421414\H,0,4.0506295726,0.6115006952,-0.6667356
331\H,0,4.5690192327,2.9064457911,-0.1461223957\H,0,4.3090112227,2.048
2402409,1.3820928537\H,0,3.0641798671,3.15375393,0.7474127843\C,0,2.44
42226793,-1.3583955868,-1.3003329888\C,0,1.9929126683,-2.5881188074,-1
.7756549878\H,0,3.2719857526,-0.876616411,-1.8076204481\N,0,0.98780891
18,-3.2891087267,-1.2362103823\H,0,2.4812730948,-3.0365978361,-2.64088

08368\H,0,-0.4451760352,-3.280599091,0.2579650566\C,0,-1.3647424532,-1
.0067063206,1.7185778781\N,0,-2.2196215465,-1.3723360861,0.6805547634\
H,0,-3.1562247257,-1.4405091139,1.0639234343\O,0,-1.8005942459,-0.7862
344366,2.8442180784\C,0,-2.1809266182,-0.8217684573,-0.6875646519\H,0,
-1.4220576462,-1.3650603743,-1.2615505681\C,0,-1.2511589028,3.41615486
64,-0.9582194295\C,0,-0.6828871345,2.5057375735,-1.8548726981\C,0,-0.9
728070635,1.1414301714,-1.7469614202\C,0,-1.8384068126,0.6675236588,-0
.7501760639\C,0,-2.4030081281,1.5891765481,0.1465430603\C,0,-2.1102933
098,2.9523388971,0.0446108692\H,0,-1.0257461171,4.477348063,-1.0379400
914\H,0,-0.0109020524,2.8552674861,-2.6362163674\H,0,-0.5137424764,0.4
342976589,-2.4364617204\H,0,-3.0571707495,1.238084871,0.9421439396\H,0
,-2.5539582567,3.6523680947,0.749412164\C,0,-3.5477512886,-1.106052151
9,-1.3436076434\H,0,-3.5317879315,-0.7949257889,-2.3934671577\H,0,-3.7
756622113,-2.1783565428,-1.2988914415\H,0,-4.3479139851,-0.5491123255,
-0.8375313852\\Version=AM64L-G03RevD.01\State=1-A\HF=-987.1920067\MP2=
-990.7092722\RMSD=4.158e-09\Thermal=0.\PG=C01 [X(C18H22N4O1)]\\@

386-Ac⁺

1\1\GINC-NODE20\SP\RMP2-FC\6-31+G(2d,p)\C20H25N4O2(1+)\ZIP01\20-Jan-20
10\0\\#P MP2/6-31+G(2d,p) scf=(direct,tight) int=finegrid geom=check g
uess=read\\sp of cat81ur5_51.ac2.sps MP2-5/6-31+G(2d,p)\\1,1\C,0,0.029
6976877,0.0960987189,-0.0400745545\N,0,0.0377817426,0.1669536662,1.347
7174684\C,0,1.3084339989,0.0055063305,-0.7265143927\C,0,-1.1299005791,
0.0594394656,-0.7794770127\C,0,1.1759557595,-0.494843739,1.9866528123\
C,0,2.4615800518,0.0980911476,1.4236760215\H,0,1.1086681407,-0.3184791
106,3.0607649268\H,0,1.1374658501,-1.5791240952,1.8171255842\N,0,2.466
5217552,0.0046069757,-0.0494636096\H,0,2.5659960313,1.1476217826,1.726
9047715\H,0,3.3151334242,-0.4645264064,1.8079120994\C,0,3.7518080213,0
.0408014455,-0.7948963256\C,0,5.0091082187,0.0620349297,0.0746419245\H
,0,3.7409472509,0.9356182216,-1.4323304123\H,0,3.785856917,-0.83749746
4,-1.4526459852\H,0,5.8719208123,0.1460797461,-0.5950316125\H,0,5.1352
488202,-0.8588429424,0.6533652609\H,0,5.0319922504,0.9213661481,0.7526
836459\C,0,1.2559014704,-0.0687479911,-2.1526053934\C,0,0.0620275988,-
0.0703702636,-2.8223856861\H,0,2.1627059697,-0.1143298156,-2.740941578
5\N,0,-1.1237048519,-0.0029014692,-2.1523802024\H,0,0.0200314114,-0.11
96951875,-3.9021010425\H,0,-2.1120182693,0.1077008153,-0.3257601833\C,
0,-1.021160962,0.5899509354,2.1937301614\N,0,-1.8311190093,1.598756046
8,1.7162581903\H,0,-2.5671382111,1.7805614979,2.3909638181\O,0,-1.1693
571816,0.0759003721,3.2871903514\C,0,-1.3974158609,2.7890769633,0.9476
36997\H,0,-1.4009413499,2.5361919973,-0.1220515641\C,0,2.5792924394,4.
2374325722,1.928140175\C,0,2.1788868577,4.1136467856,0.59392312\C,0,0.
9006359296,3.6315809415,0.2889830012\C,0,0.0061843797,3.2740445322,1.3
078130774\C,0,0.4138418242,3.4089196092,2.6454621967\C,0,1.69191644,3.
8842061829,2.9528975531\H,0,3.5682971267,4.6190709871,2.1700504533\H,0
,2.8552115132,4.4014923978,-0.2080890314\H,0,0.5918851802,3.5430620448
,-0.7524186025\H,0,-0.2650570569,3.1364082539,3.4514071159\H,0,1.99139
86755,3.9890970244,3.9929824614\C,0,-2.4535715611,3.8889703093,1.16261
75488\H,0,-2.2085903751,4.7646353803,0.5537006184\H,0,-3.44929058,3.53
17765565,0.8728295666\H,0,-2.4789242436,4.2056956329,2.2126043227\C,0,
-2.4353427392,0.0092342967,-2.8295761833\O,0,-3.4154605595,0.140240385
5,-2.1492563155\C,0,-2.4346437132,-0.1511544758,-4.3301653076\H,0,-1.9
173016114,0.6805491289,-4.823891759\H,0,-1.9579224611,-1.0902096499,-4
.6353311942\H,0,-3.4771897897,-0.1586368539,-4.6529265875\\Version=AM6
4L-G03RevD.01\State=1-A\HF=-1139.3738282\MP2=-1143.3699869\RMSD=4.192e
-09\Thermal=0.\PG=C01 [X(C20H25N4O2)]\\@

387

1\1\GINC-NODE23\SP\RMP2-FC\6-31+G(2d,p)\C22H22F6N4O1\ZIP01\06-Feb-2010

\0\\#P MP2/6-31+G(2d,p) scf=(direct,tight) int=finegrid geom=check gue
ss=read\\sp of cat11ur3_3 MP2-5/6-31+G(2d,p)\\0,1\C,0,-0.023265347,-0.
008586542,-0.0042323175\C,0,-0.0324195469,-0.0026467825,1.5431858333\C
,0,1.4245550211,-0.0232905022,2.064027504\C,0,2.3231710717,1.065089465
2,1.4468667665\C,0,2.301676432,0.994033847,-0.0888510736\C,0,0.8612496
925,1.0912617583,-0.6167437394\N,0,1.4033692434,0.0660588089,3.5291563
079\C,0,0.7418327232,1.2207717656,4.0229017041\C,0,-0.371562251,1.7253
825487,3.2924424445\N,0,-0.7774236445,1.1428035108,2.1082092474\C,0,-1
.0064120823,2.8552369066,3.8549578562\C,0,-0.5090448697,3.4176535326,5
.0286749759\N,0,0.5798165031,2.985900502,5.679637662\C,0,1.1915992977,
1.9193469927,5.1491016103\C,0,-2.1726903615,1.3167072668,1.67627666\C,
0,-2.4122064659,2.4649444059,0.6835325837\H,0,-0.5150787256,-0.9352795
296,1.8837424195\H,0,1.8415828797,-1.0045015735,1.8235265944\H,0,3.341
1136156,0.9269193798,1.832542102\H,0,1.9893345124,2.0601222834,1.77204
01318\H,0,2.9200727253,1.7982572223,-0.508079585\H,0,2.7499301121,0.04
46990085,-0.4203389047\H,0,0.845903451,1.0053030054,-1.7113376456\H,0,
0.451450602,2.0800640249,-0.3671239319\H,0,-1.0467004883,0.049804095,-
0.3950492058\H,0,0.3600117366,-0.993046679,-0.3118155176\H,0,-2.502807
9352,0.3693031076,1.2319525356\H,0,-2.7946464193,1.4627857389,2.568840
877\H,0,-1.89053984,2.2937495764,-0.2638064749\H,0,-3.4851918548,2.553
5179084,0.4702156289\H,0,-2.0646836692,3.4222748515,1.0879825286\H,0,-
1.8697255926,3.307583691,3.3789318456\H,0,2.0949985092,1.5760144597,5.
6544733275\H,0,-1.00979296,4.2850037375,5.4579659459\C,0,1.6634258612,
-1.1059625333,4.2475877891\O,0,2.2134322308,-2.0675178674,3.7255399091
\H,0,0.7083743398,-0.2828439768,5.864313587\N,0,1.2473745638,-1.086382
9773,5.5684870599\C,0,1.7496317294,-3.9538154462,8.628696508\C,0,2.313
2072357,-4.1754460109,7.3702590035\C,0,2.1706066786,-3.2574686821,6.32
66689604\C,0,1.4427293668,-2.0745121214,6.5444009318\C,0,0.8703217266,
-1.8456658658,7.8081180971\C,0,1.0246044912,-2.7777187934,8.8337522747
\H,0,1.8591992943,-4.6830241061,9.4232555406\H,0,2.5982360087,-3.45131
52424,5.3520352887\H,0,0.2949501878,-0.9404184438,7.9888277896\C,0,0.4
396906,-2.472651618,10.1941894694\C,0,3.1487684648,-5.4149564832,7.132
8391625\F,0,-0.7333196074,-1.8146696914,10.0938520313\F,0,0.2189606175
,-3.5917073047,10.9077558625\F,0,1.2645476247,-1.692680873,10.92198919
52\F,0,2.8101477201,-6.414462096,7.9705415078\F,0,3.0149063164,-5.8753
204327,5.875318739\F,0,4.4603561836,-5.1657170188,7.3245359715\\Versio
n=AM64L-G03RevD.01\State=1-A\HF=-1735.384226\MP2=-1740.6806006\RMSD=6.
541e-09\Thermal=0.\PG=C01 [X(C22H22F6N4O1)]\\@

387-Ac$^+$

1\1\GINC-NODE17\SP\RMP2-FC\6-31+G(2d,p)\C24H25F6N4O2(1+)\ZIP01\14-Feb-
2010\0\\#P MP2/6-31+G(2d,p) scf=(direct,tight) int=finegrid geom=check
guess=read\\sp of cat11ur3_2.ac2 MP2-5/6-31+G(2d,p)\\1,1\C,0,-0.13681
22201,-0.0123812295,0.1514228213\C,0,-0.0343625892,0.0024535567,1.6916
46894\C,0,1.4568893273,-0.0060390988,2.0889071473\C,0,2.272396219,1.12
29197478,1.4305481907\C,0,2.1210109241,1.1007309272,-0.1014131097\C,0,
0.640586453,1.1340928401,-0.5186603074\N,0,1.5391735432,0.0189674786,3
.5581884519\C,0,0.8878969832,1.0902796836,4.1691760217\C,0,-0.30811738
46,1.6160430211,3.5370593048\N,0,-0.7239953785,1.1641276567,2.34114551
52\C,0,-0.9995687166,2.629282865,4.2751591555\C,0,-0.5182351162,3.1071
250576,5.4632210771\N,0,0.6542734446,2.6493867318,5.9879873468\C,0,1.3
52212304,1.6732083837,5.3236835949\C,0,-2.0187944613,1.6129287677,1.78
38262549\C,0,-3.2054232523,0.7820179088,2.2918962542\H,0,-0.5095475974
,-0.9071496417,2.0858078873\H,0,1.8730473702,-0.9677818791,1.777603315
\H,0,3.321652295,0.9953707214,1.7216745201\H,0,1.9428862955,2.09762417
82,1.8196066348\H,0,2.591220467,0.1904373897,-0.5013784996\H,0,2.66177
76215,1.9493098993,-0.5369872571\H,0,0.5491010582,1.0454451956,-1.6077
75493\H,0,0.2005723257,2.1047865291,-0.2439801985\H,0,-1.1806957169,-0

.0526643212,-0.1794501563\H,0,0.2994733849,-0.9723603889,-0.1598287926
\H,0,-2.1444329102,2.6746343726,2.0115074994\H,0,-1.9479566921,1.55636
61644,0.6978321584\H,0,-3.2979251664,0.8238614686,3.383046129\H,0,-3.1
048972616,-0.271161329,2.0076484865\H,0,-4.1337340767,1.163890088,1.85
23237972\H,0,-1.9459795422,3.0238555034,3.9319654843\H,0,2.2717381725,
1.3661729435,5.8030634015\H,0,-1.0556472309,3.8560906327,6.0292152992\
C,0,2.4159644713,-0.9067813543,4.1762411862\O,0,3.3319143628,-1.410166
0225,3.5573774389\H,0,2.9021144176,-1.7685913112,5.9164384901\N,0,2.16
39653528,-1.2036401724,5.5091007027\C,0,-1.6462704058,-1.0669997084,7.
3369308277\C,0,-1.5027566527,-1.5776868495,6.0407352931\C,0,-0.2436204
355,-1.6782944203,5.4513171173\C,0,0.8892730258,-1.2209773473,6.139994
3581\C,0,0.759529711,-0.7304419717,7.4429256086\C,0,-0.5078442441,-0.6
602007066,8.033958491\H,0,-2.6282673436,-0.9866524825,7.7926750425\H,0
,-0.1395107732,-2.0991365603,4.4573395917\H,0,1.6347596111,-0.37348912
83,7.9795616321\C,0,1.1986930604,3.1024674075,7.2837927787\O,0,2.19738
00896,2.5694771615,7.684651609\C,0,0.4492001468,4.198633621,7.99633213
53\H,0,0.3396902058,5.0924539267,7.3714476609\H,0,-0.545002936,3.85317
76718,8.3037633794\H,0,1.0224875629,4.4525445318,8.8896294926\C,0,-2.7
37715109,-2.0140237033,5.2791974042\C,0,-0.639108112,0.0038837613,9.38
821464\F,0,-3.2691939866,-3.1336456578,5.7801148792\F,0,-2.4637967064,
-2.2340024386,3.9742059266\F,0,-3.6879758066,-1.0558045522,5.331382705
6\F,0,0.3785405625,-0.3101695293,10.198481354\F,0,-1.7862538784,-0.312
3371898,9.9986322312\F,0,-0.6239408361,1.3582676689,9.2400452518\\Vers
ion=AM64L-G03RevD.01\State=1-A\HF=-1887.5511267\MP2=-1893.3457655\RMSD
=7.765e-09\Thermal=0.\PG=C01 [X(C24H25F6N4O2)]\\@

388
see reference 9

388-Ac$^+$
see reference 9

389
1\1\GINC-NODE22\SP\RMP2-FC\6-31+G(2d,p)\C21H26N4O1\ZIP01\02-Jan-2010\0
\\#P MP2/6-31+G(2d,p) scf=(direct,tight) int=finegrid geom=check guess
=read\\sp of cat11ur2_52 MP2-5/6-31+G(2d,p)\\0,1\C,0,-0.0745711214,0.1
090745053,-0.0334732547\C,0,-0.0433622235,0.0691795475,1.5134039462\C,
0,1.423999351,-0.0203440057,1.9899067299\C,0,2.3516534763,1.0468145379
,1.3769417748\C,0,2.2810950601,1.0334009054,-0.158554773\C,0,0.8297152
979,1.1966808597,-0.639421489\N,0,1.4334141643,0.0186118916,3.45573175
98\C,0,0.8306829105,1.1675305367,4.0166072604\C,0,-0.2718819906,1.7446
026249,3.3272948608\N,0,-0.7243756779,1.2288219931,2.1232537518\C,0,-0
.856563298,2.8739138727,3.9395097003\C,0,-0.3265989611,3.3717832339,5.
1281138045\N,0,0.7452334381,2.863919008,5.7498833232\C,0,1.3095380375,
1.7940939811,5.172774622\C,0,-2.1361647374,1.4263368265,1.7658662027\C
,0,-2.4087178654,2.6016816517,0.8140538853\H,0,-0.5528222121,-0.851215
4866,1.8451773235\H,0,1.7992891894,-1.0069482442,1.7048850404\H,0,3.37
18621971,0.8434179362,1.7251871772\H,0,2.0725796977,2.0418215427,1.750
2556615\H,0,2.9140969926,1.8308943232,-0.5696137041\H,0,2.6863919442,0
.0822851768,-0.5376939013\H,0,0.7794837728,1.1456323194,-1.7355681628\
H,0,0.4606735799,2.1901038764,-0.3477104913\H,0,0.2644302046,-0.878200
2353,-0.3823572497\H,0,-1.1062964738,0.215026012,-0.3916474077\H,0,-2.
7137004722,1.5556124198,2.6905430128\H,0,-2.4988673282,0.4937073853,1.
3147796918\H,0,-3.4881691193,2.6973832406,0.6376864924\H,0,-1.91761090
01,2.4564966802,-0.1541094959\H,0,-2.0462891983,3.5474981855,1.2325018
389\H,0,-1.7050673534,3.3762761052,3.486921373\H,0,2.1811935748,1.3757
884026,5.6733956689\H,0,-0.7849973706,4.243299964,5.5954077889\C,0,2.1
372054873,-0.9806868368,4.1218337621\O,0,3.0046471395,-1.6415939573,3.

5582934956\N,0,1.8093046116,-1.2215586807,5.4509442827\H,0,2.408993341
1,-1.9563325446,5.804034867\C,0,0.4543760299,-1.1951942659,6.013321001
8\H,0,0.5192057777,-1.7480423102,6.9596608844\H,0,0.1724625067,-0.1691
985123,6.2754663949\C,0,-2.6889959019,-2.9487021141,3.5853354551\C,0,-
2.9196876664,-1.7702763964,4.3015248208\C,0,-1.895391832,-1.2036230188
,5.0678237989\C,0,-0.6329289554,-1.8092669912,5.1370555996\C,0,-0.4102
128515,-2.9927938273,4.4154336981\C,0,-1.4294871384,-3.556784779,3.643
0822335\H,0,-3.482621435,-3.3905454606,2.9868371859\H,0,-3.894226638,-
1.287999524,4.260912426\H,0,-2.0741759132,-0.2762990066,5.6099454975\H
,0,0.569648068,-3.4655221978,4.4434719905\H,0,-1.2405629836,-4.4731108
43,3.08792883\\Version=AM64L-G03RevD.01\State=1-A\HF=-1103.1460162\MP2
=-1107.1116177\RMSD=4.023e-09\Thermal=0.\PG=C01 [X(C21H26N4O1)]\\@

389-Ac⁺

1\1\GINC-NODE16\SP\RMP2-FC\6-31+G(2d,p)\C23H29N4O2(1+)\ZIP01\13-Jan-20
10\0\\#P MP2/6-31+G(2d,p) scf=(direct,tight) int=finegrid geom=check g
uess=read\\sp of cat11ur2_52.ac2 MP2-5/6-31+G(2d,p)\\1,1\C,0,-3.193291
528,-1.7986641358,0.1973936241\C,0,-1.9883927168,-0.8415254058,0.02543
37502\C,0,-1.3786715452,-0.5281123367,1.4060424514\C,0,-1.0983431115,-
1.7649241287,2.282367752\C,0,-2.3559924991,-2.6337428561,2.4389614536\
C,0,-2.9020082544,-3.0387009783,1.0617910659\N,0,-0.1720022348,0.29154
20623,1.2047463485\C,0,0.8046356541,-0.2894748928,0.4057471662\C,0,0.3
616675792,-1.138140788,-0.6850910747\N,0,-0.9426041094,-1.3676807155,-
0.9036927856\C,0,1.3888920685,-1.6782358811,-1.5195693796\C,0,2.711998
5199,-1.4274716295,-1.275284895\N,0,3.1015683237,-0.6485705708,-0.2254
317883\C,0,2.1528806421,-0.1039116762,0.6078694816\C,0,-1.392269789,-1
.8634051598,-2.2262299701\C,0,-1.4719696592,-3.3894478775,-2.365417091
9\H,0,-2.3446009035,0.0996852973,-0.4169499439\H,0,-2.0944354352,0.110
1889137,1.9307494074\H,0,-0.739860618,-1.406647537,3.2540915542\H,0,-0
.2916080249,-2.3667716239,1.8374598085\H,0,-2.1225991285,-3.5225388704
,3.0373915221\H,0,-3.1246647055,-2.0744268616,2.9924925661\H,0,-3.8246
449511,-3.6224402576,1.1687814234\H,0,-2.1728159582,-3.6914654832,0.56
06291228\H,0,-3.9762792509,-1.2006577862,0.6853787031\H,0,-3.607397225
9,-2.0821465125,-0.7771088093\H,0,-0.7227527423,-1.4404328933,-2.98369
49338\H,0,-2.3722923311,-1.4127269028,-2.4090412627\H,0,-1.7366862893,
-3.6406071092,-3.399146428\H,0,-2.2342236725,-3.8176138441,-1.70867487
57\H,0,-0.5172535369,-3.8744174452,-2.1321799707\H,0,1.1440772845,-2.3
153791015,-2.359279721\H,0,2.5449238852,0.5153196142,1.4048604693\H,0,
3.4910767192,-1.8410256596,-1.9014565476\C,0,0.0066630145,1.389231557,
2.0860544337\O,0,-0.5610357898,1.4161344509,3.1634333358\N,0,0.8213989
636,2.4078254689,1.6398514462\H,0,0.8898532694,3.1253204446,2.35211670
76\C,0,0.9317807041,2.8794494469,0.2494329179\H,0,1.320695954,3.902385
6372,0.3195722513\H,0,1.6958413755,2.3017851002,-0.2878777843\C,0,-2.7
322620956,2.8794795157,-2.0747018564\C,0,-1.5347474235,2.4325202878,-2
.6415407821\C,0,-0.3606434926,2.4226236736,-1.8794303903\C,0,-0.365793
4577,2.8664383265,-0.5496727829\C,0,-1.5707607539,3.3220151355,0.00939
67303\C,0,-2.7460246123,3.3246204841,-0.7468788126\H,0,-3.6452930556,2
.8947587533,-2.6648929974\H,0,-1.5111083044,2.1007593137,-3.6773143982
\H,0,0.5705361077,2.0774913084,-2.328035871\H,0,-1.5939111803,3.673821
8364,1.039070411\H,0,-3.670599497,3.6863205894,-0.3032791307\C,0,4.513
1276212,-0.3482660914,0.0774198561\O,0,4.7461959783,0.3820402432,1.001
360132\C,0,5.5564351022,-0.991392882,-0.8035591188\H,0,5.4934247272,-2
.0856614773,-0.771444601\H,0,5.4595319853,-0.6658402308,-1.8464006465\
H,0,6.5317975899,-0.6800187222,-0.4256834834\\Version=AM64L-G03RevD.01
\State=1-A\HF=-1255.3288036\MP2=-1259.7734758\RMSD=4.473e-09\Thermal=0
.\PG=C01 [X(C23H29N4O2)]\\@

390

1\1\GINC-NODE13\SP\RMP2-FC\6-31+G(2d,p)\C20H24N4O1\ZIP01\26-Dec-2009\0
\\#P MP2/6-31+G(2d,p) scf=(direct,tight) int=finegrid geom=check guess
=read\\sp of cat11ur1_3 MP2-5/6-31+G(2d,p)\\0,1\C,0,0.0591724852,0.018
1364177,0.0228544813\C,0,0.1349484733,-0.0125188315,1.5685964995\C,0,1
.6135613708,0.0870510656,2.0129289295\C,0,2.3762073947,1.2655181718,1.
3788781351\C,0,2.2732297245,1.2350572569,-0.155559797\C,0,0.8027158402
,1.2109597066,-0.6016919993\N,0,1.6662331122,0.1436443735,3.4778408741
\C,0,0.93334808,1.2236479656,4.0327220728\C,0,-0.257549152,1.644374569
9,3.3750713199\N,0,-0.6762872609,1.0501163707,2.200213883\C,0,-0.95641
59392,2.7035341984,3.9953534254\C,0,-0.4482284387,3.2834124596,5.15598
86238\N,0,0.7047409012,2.932621232,5.7415772706\C,0,1.3770436058,1.933
659929,5.1542865829\C,0,-2.1029597426,1.1033944179,1.8496846485\C,0,-2
.5014117432,2.239106609,0.893927972\H,0,-0.24386919,-0.9917487584,1.90
94282265\H,0,2.103069383,-0.8475627406,1.7272499857\H,0,3.4228563158,1
.2087152008,1.703639784\H,0,1.9765011856,2.2180573485,1.7535954695\H,0
,2.7901218179,2.1047537883,-0.5820610661\H,0,2.7870299611,0.3412817033
,-0.5422017192\H,0,0.7341669355,1.1523787792,-1.6962512704\H,0,0.31850
18225,2.1505972928,-0.3007131804\H,0,-0.9849220065,-0.0111455859,-0.31
34620094\H,0,0.5156987469,-0.917754878,-0.3328429661\H,0,-2.3716996403
,0.1364398258,1.405545289\H,0,-2.6842795109,1.1801834704,2.7776731375\
H,0,-2.019408139,2.1303475387,-0.0832556363\H,0,-3.5882708792,2.231622
2348,0.7403445658\H,0,-2.22048309,3.2184938371,1.2974344935\H,0,-1.879
3622701,3.0882320565,3.5745520338\H,0,2.3290193929,1.65533213,5.607436
5933\H,0,-0.9976523832,4.096374304,5.6302878132\C,0,2.040336928,-1.033
3074754,4.1523700434\O,0,2.6290628376,-1.9338525245,3.5658706835\H,0,1
.1158103718,-0.3176228449,5.8269744733\N,0,1.6908049759,-1.0758720503,
5.4844202821\C,0,2.4979599582,-3.9582754655,8.4639902766\C,0,3.0184708
813,-4.1340128648,7.1789919418\C,0,2.7741672185,-3.2089051057,6.160294
4621\C,0,1.9889752581,-2.0753260321,6.4336963993\C,0,1.4638128416,-1.8
952057615,7.7270295098\C,0,1.7169447094,-2.8293730666,8.7304757232\H,0
,2.6966327192,-4.6877703577,9.2452009034\H,0,3.6278384532,-5.007283918
8,6.955590516\H,0,3.1750450749,-3.3536036064,5.1658837543\H,0,0.857196
144,-1.016715467,7.9447787093\H,0,1.3016612515,-2.6698492719,9.7231211
816\\Version=AM64L-G03RevD.01\State=1-A\HF=-1064.117393\MP2=-1067.9156
04\RMSD=8.345e-09\Thermal=0.\PG=C01 [X(C20H24N4O1)]\\@

390-Ac$^+$

1\1\GINC-NODE11\SP\RMP2-FC\6-31+G(2d,p)\C22H27N4O2(1+)\ZIP01\31-Dec-20
09\0\\#P MP2/6-31+G(2d,p) scf=(direct,tight) int=finegrid geom=check g
uess=read\\sp of cat11ur1_2.ac2 MP2-5/6-31+G(2d,p)\\1,1\C,0,-0.1398973
814,-0.1076427831,0.0843012445\C,0,-0.0969646064,-0.0129612696,1.62420
55877\C,0,1.3737098115,-0.0513778405,2.0804829386\C,0,2.2489469434,1.0
210959207,1.4033014487\C,0,2.1669745844,0.9149764759,-0.1302173116\C,0
,0.7088654376,0.9678728541,-0.618161412\N,0,1.4056601966,0.0270075023,
3.5508851204\C,0,0.7468784993,1.1250484678,4.1026956744\C,0,-0.3934976
478,1.6739795621,3.3905917915\N,0,-0.7619906933,1.2047932304,2.1841805
895\C,0,-1.0860366446,2.736968273,4.0521169719\C,0,-0.6484175192,3.249
7537782,5.2420188696\N,0,0.4757612581,2.7684008092,5.8443835805\C,0,1.
1637894593,1.7332801082,5.2647256333\C,0,-1.9816846321,1.7137068284,1.
5229574132\C,0,-3.2535354863,0.9745851214,1.9612653245\H,0,-0.61686496
06,-0.8851357985,2.0457474654\H,0,1.7690968768,-1.0370611609,1.8222815
058\H,0,3.2789592731,0.8812568043,1.7497074621\H,0,1.9275855008,2.0231
089517,1.7240259413\H,0,2.6251104821,-0.0312024787,-0.4535534454\H,0,2
.753767497,1.7197459487,-0.5888635624\H,0,0.663096044,0.8114997932,-1.
7027005619\H,0,0.2928401208,1.9688610187,-0.428134782\H,0,-1.171387277
6,-0.1255886506,-0.2860568502\H,0,0.2693989855,-1.0990167604,-0.156814
3933\H,0,-2.0568594021,2.7875608153,1.7129807573\H,0,-1.8383933093,1.6
192781843,0.4467316313\H,0,-3.4208968049,1.0627064212,3.0411222304\H,0

,-3.1972735497,-0.0908982247,1.710576709\H,0,-4.1222276802,1.396593542
6,1.4434559117\H,0,-1.9959892185,3.1516585039,3.6407062345\H,0,2.04273
56097,1.4119996032,5.8059101965\H,0,-1.1815969977,4.0451880356,5.74477
96801\C,0,2.3809899685,-0.7829733656,4.1936909231\O,0,3.3099978139,-1.
2453627302,3.5569617901\H,0,3.0325242856,-1.5024185301,5.9167261249\N,
0,2.2273495949,-1.0063247394,5.5473373979\C,0,-1.301902673,-1.45048245
62,7.8405003941\C,0,-1.2882845451,-1.8675470611,6.5048872739\C,0,-0.13
27573683,-1.7298813807,5.7317804794\C,0,1.0125747071,-1.1450495091,6.2
913780932\C,0,1.001424315,-0.7216383756,7.6285233509\C,0,-0.1517992495
,-0.8864158618,8.4011845113\H,0,-2.197464114,-1.5786031529,8.442883064
6\H,0,-2.170987704,-2.3303492447,6.0701363707\H,0,-0.1097488292,-2.096
6258737,4.7088952298\H,0,1.8940059469,-0.2715101768,8.0572749774\H,0,-
0.1479500521,-0.572808249,9.4421625686\C,0,0.9944673709,3.2872459213,7
.1212863854\O,0,1.9651974049,2.7590440672,7.5919056249\C,0,0.261134959
1,4.4542466607,7.7360919185\H,0,0.2352955248,5.3171062832,7.0600692735
\H,0,-0.7690420998,4.1861825005,8.0008040288\H,0,0.7985440941,4.729503
3375,8.6452148201\\Version=AM64L-G03RevD.01\State=1-A\HF=-1216.2931835
\MP2=-1220.5803238\RMSD=2.566e-09\Thermal=0.\PG=C01 [X(C22H27N4O2)]\\@

391

1\1\GINC-NODE20\FOpt\RB98\6-31G(d)\C16H18N4S1\ZIP01\22-Oct-2009\0\\#P
b98/6-31G(d) opt=(maxCycle=60) freq=noraman\\opt and freq of cat81ur2_
5c1 b98/6-31G(d) on 4p 900 MB\\0,1\C,-0.0636705969,-0.0322076973,0.051
5375877\N,-0.0459746309,-0.0500386826,1.476316029\C,1.1877522356,0.024
4393615,-0.6230880821\C,-1.2356566216,-0.1741510162,-0.6957760614\C,1.
0909569437,-0.8083866787,2.0051399735\C,2.3794022113,-0.1360608664,1.5
314164412\H,1.0334301963,-0.8179840531,3.0944270464\H,1.0349515162,-1.
8432456653,1.6431819295\N,2.3644121242,0.0947171464,0.0881430638\H,3.2
296463418,-0.7870597871,1.7821079995\H,2.522653938,0.8133213858,2.0717
295406\C,3.660762004,0.2532132464,-0.5634275795\C,4.3012435162,-1.0709
484636,-1.0115995723\H,4.3208450746,0.7634188347,0.1507160279\H,3.5480
647503,0.9348143889,-1.4146564765\H,5.2802127192,-0.8852058035,-1.4707
974533\H,4.4508825898,-1.7440423279,-0.1583262139\H,3.6692538417,-1.58
80329345,-1.7422633823\C,1.128093557,0.0082982568,-2.0339171411\C,-0.1
083829296,-0.080046017,-2.6668368187\H,2.0303266236,0.0524441933,-2.63
49691655\N,-1.284383592,-0.1853143945,-2.0324920002\H,-0.1505942302,-0
.0863224395,-3.7558440698\H,-2.1861785338,-0.2679779681,-0.1745642616\
C,-1.0349203597,0.4231683104,2.3049851299\N,-1.8465480783,1.4259041747
,1.8229198025\H,-2.6573124624,1.5543455734,2.4163485267\C,-1.550723877
9,2.5221692878,0.9691627397\C,-0.2518144857,3.0243342254,0.7972506135\
C,-2.6281698315,3.1555201316,0.3301767309\C,-0.0416062304,4.136900896,
-0.0197303486\H,0.5841386832,2.5556056646,1.3066719518\C,-2.408812488,
4.2773980616,-0.4702869718\H,-3.6330258764,2.7581596113,0.4574649218\C
,-1.1136403343,4.770567578,-0.6554264495\H,0.9693559159,4.5173341241,-
0.1476565736\H,-3.2530278276,4.7579510774,-0.9589893253\H,-0.941973353
,5.638928314,-1.2864078492\S,-1.3263914128,-0.1882800496,3.8502412267\
\Version=AM64L-G03RevD.01\State=1-A\HF=-1237.3493692\RMSD=4.094e-09\RM
SF=3.596e-06\Thermal=0.\Dipole=1.9767456,0.7218227,-0.8956742\PG=C01 [
X(C16H18N4S1)]\\@

392

1\1\GINC-NODE23\SP\RMP2-FC\6-31+G(2d,p)\C22H22F6N4O1\ZIP01\06-Feb-2010
\0\\#P MP2/6-31+G(2d,p) scf=(direct,tight) int=finegrid geom=check gue
ss=read\\sp of cat11ur3_3 MP2-5/6-31+G(2d,p)\\0,1\C,0,-0.023265347,-0.
008586542,-0.0042323175\C,0,-0.0324195469,-0.0026467825,1.5431858333\C
,0,1.4245550211,-0.0232905022,2.064027504\C,0,2.3231710717,1.065089465
2,1.4468667665\C,0,2.301676432,0.994033847,-0.0888510736\C,0,0.8612496
925,1.0912617583,-0.6167437394\N,0,1.4033692434,0.0660588089,3.5291563

079\C,0,0.7418327232,1.2207717656,4.0229017041\C,0,-0.371562251,1.7253
825487,3.2924424445\N,0,-0.7774236445,1.1428035108,2.1082092474\C,0,-1
.0064120823,2.8552369066,3.8549578562\C,0,-0.5090448697,3.4176535326,5
.0286749759\N,0,0.5798165031,2.985900502,5.679637662\C,0,1.1915992977,
1.9193469927,5.1491016103\C,0,-2.1726903615,1.3167072668,1.67627666\C,
0,-2.4122064659,2.4649444059,0.6835325837\H,0,-0.5150787256,-0.9352795
296,1.8837424195\H,0,1.8415828797,-1.0045015735,1.8235265944\H,0,3.341
1136156,0.9269193798,1.832542102\H,0,1.9893345124,2.0601222834,1.77204
01318\H,0,2.9200727253,1.7982572223,-0.508079585\H,0,2.7499301121,0.04
46990085,-0.4203389047\H,0,0.845903451,1.0053030054,-1.7113376456\H,0,
0.451450602,2.0800640249,-0.3671239319\H,0,-1.0467004883,0.049804095,-
0.3950492058\H,0,0.3600117366,-0.993046679,-0.3118155176\H,0,-2.502807
9352,0.3693031076,1.2319525356\H,0,-2.7946464193,1.4627857389,2.568840
877\H,0,-1.89053984,2.2937495764,-0.2638064749\H,0,-3.4851918548,2.553
5179084,0.4702156289\H,0,-2.0646836692,3.4222748515,1.0879825286\H,0,-
1.8697255926,3.307583691,3.3789318456\H,0,2.0949985092,1.5760144597,5.
6544733275\H,0,-1.00979296,4.2850037375,5.4579659459\C,0,1.6634258612,
-1.1059625333,4.2475877891\O,0,2.2134322308,-2.0675178674,3.7255399091
\H,0,0.7083743398,-0.2828439768,5.864313587\N,0,1.2473745638,-1.086382
9773,5.5684870599\C,0,1.7496317294,-3.9538154462,8.628696508\C,0,2.313
2072357,-4.1754460109,7.3702590035\C,0,2.1706066786,-3.2574686821,6.32
66689604\C,0,1.4427293668,-2.0745121214,6.5444009318\C,0,0.8703217266,
-1.8456658658,7.8081180971\C,0,1.0246044912,-2.7777187934,8.8337522747
\H,0,1.8591992943,-4.6830241061,9.4232555406\H,0,2.5982360087,-3.45131
52424,5.3520352887\H,0,0.2949501878,-0.9404184438,7.9888277896\C,0,0.4
396906,-2.472651618,10.1941894694\C,0,3.1487684648,-5.4149564832,7.132
8391625\F,0,-0.7333196074,-1.8146696914,10.0938520313\F,0,0.2189606175
,-3.5917073047,10.9077558625\F,0,1.2645476247,-1.692680873,10.92198919
52\F,0,2.8101477201,-6.414462096,7.9705415078\F,0,3.0149063164,-5.8753
204327,5.875318739\F,0,4.4603561836,-5.1657170188,7.3245359715\\Versio
n=AM64L-G03RevD.01\State=1-A\HF=-1735.384226\MP2=-1740.6806006\RMSD=6.
541e-09\Thermal=0.\PG=C01 [X(C22H22F6N4O1)]\\@

392-Ac^{+}

1\1\GINC-NODE23\SP\RMP2-FC\6-31+G(2d,p)\C22H22F6N4O1\ZIP01\06-Feb-2010
\0\\#P MP2/6-31+G(2d,p) scf=(direct,tight) int=finegrid geom=check gue
ss=read\\sp of cat11ur3_3 MP2-5/6-31+G(2d,p)\\0,1\C,0,-0.023265347,-0.
008586542,-0.0042323175\C,0,-0.0324195469,-0.0026467825,1.5431858333\C
,0,1.4245550211,-0.0232905022,2.064027504\C,0,2.3231710717,1.065089465
2,1.4468667665\C,0,2.301676432,0.994033847,-0.0888510736\C,0,0.8612496
925,1.0912617583,-0.6167437394\N,0,1.4033692434,0.0660588089,3.5291563
079\C,0,0.7418327232,1.2207717656,4.0229017041\C,0,-0.371562251,1.7253
825487,3.2924424445\N,0,-0.7774236445,1.1428035108,2.1082092474\C,0,-1
.0064120823,2.8552369066,3.8549578562\C,0,-0.5090448697,3.4176535326,5
.0286749759\N,0,0.5798165031,2.985900502,5.679637662\C,0,1.1915992977,
1.9193469927,5.1491016103\C,0,-2.1726903615,1.3167072668,1.67627666\C,
0,-2.4122064659,2.4649444059,0.6835325837\H,0,-0.5150787256,-0.9352795
296,1.8837424195\H,0,1.8415828797,-1.0045015735,1.8235265944\H,0,3.341
1136156,0.9269193798,1.832542102\H,0,1.9893345124,2.0601222834,1.77204
01318\H,0,2.9200727253,1.7982572223,-0.508079585\H,0,2.7499301121,0.04
46990085,-0.4203389047\H,0,0.845903451,1.0053030054,-1.7113376456\H,0,
0.451450602,2.0800640249,-0.3671239319\H,0,-1.0467004883,0.049804095,-
0.3950492058\H,0,0.3600117366,-0.993046679,-0.3118155176\H,0,-2.502807
9352,0.3693031076,1.2319525356\H,0,-2.7946464193,1.4627857389,2.568840
877\H,0,-1.89053984,2.2937495764,-0.2638064749\H,0,-3.4851918548,2.553
5179084,0.4702156289\H,0,-2.0646836692,3.4222748515,1.0879825286\H,0,-
1.8697255926,3.307583691,3.3789318456\H,0,2.0949985092,1.5760144597,5.
6544733275\H,0,-1.00979296,4.2850037375,5.4579659459\C,0,1.6634258612,

-1.1059625333,4.2475877891\O,0,2.2134322308,-2.0675178674,3.7255399091
\H,0,0.7083743398,-0.2828439768,5.864313587\N,0,1.2473745638,-1.086382
9773,5.5684870599\C,0,1.7496317294,-3.9538154462,8.628696508\C,0,2.313
2072357,-4.1754460109,7.3702590035\C,0,2.1706066786,-3.2574686821,6.32
66689604\C,0,1.4427293668,-2.0745121214,6.5444009318\C,0,0.8703217266,
-1.8456658658,7.8081180971\C,0,1.0246044912,-2.7777187934,8.8337522747
\H,0,1.8591992943,-4.6830241061,9.4232555406\H,0,2.5982360087,-3.45131
52424,5.3520352887\H,0,0.2949501878,-0.9404184438,7.9888277896\C,0,0.4
396906,-2.472651618,10.1941894694\C,0,3.1487684648,-5.4149564832,7.132
8391625\F,0,-0.7333196074,-1.8146696914,10.0938520313\F,0,0.2189606175
,-3.5917073047,10.9077558625\F,0,1.2645476247,-1.692680873,10.92198919
52\F,0,2.8101477201,-6.414462096,7.9705415078\F,0,3.0149063164,-5.8753
204327,5.875318739\F,0,4.4603561836,-5.1657170188,7.3245359715\\Versio
n=AM64L-G03RevD.01\State=1-A\HF=-1735.384226\MP2=-1740.6806006\RMSD=6.
541e-09\Thermal=0.\PG=C01 [X(C22H22F6N4O1)]\\@

393

1\1\GINC-MORITZ\SP\RB98\6-31G(d)\C17H20N4O1\EVGENY\23-Oct-2009\0\\#P b
98/6-31G(d) scf=(conver=5) int=finegrid\\sp of cat81ur3_66 b98/6-31G(d
) on 2p 900 MB\\0,1\C\N,1,1.3962\C,1,1.4096,2,122.48\C,1,1.4026,2,119.
02,3,182.78,0\C,2,1.4537,1,105.54,3,36.02,0\C,5,1.5175,2,109.47,1,296.
81,0\H,5,1.1156,2,111.43,1,58.8,0\H,5,1.1141,2,111.84,1,176.76,0\N,3,1
.401,1,121.55,2,0.82,0\H,6,1.1147,5,108.72,2,180.92,0\H,6,1.1149,5,108
.83,2,295.88,0\C,9,1.4605,3,121.27,1,169.02,0\C,12,1.5212,9,112.57,3,9
0.34,0\H,12,1.1151,9,109.37,3,210.12,0\H,12,1.1127,9,111.94,3,325.55,0
\H,13,1.1123,12,112.08,9,299.69,0\H,13,1.1132,12,111.5,9,59.84,0\H,13,
1.1133,12,111.4,9,179.57,0\C,3,1.4009,1,116.5,2,180.47,0\C,19,1.3929,3
,120.8,1,0.88,0\H,19,1.0988,3,121.3,1,180.16,0\N,20,1.3447,19,123.11,3
,0.6,0\H,20,1.1027,19,120.4,3,179.77,0\H,4,1.1032,1,119.77,2,0.37,0\C,
2,1.3915,1,132.95,3,211.88,0\N,25,1.3842,2,121.59,1,27.1,0\H,26,1.0266
,25,115.25,2,183.99,0\O,25,1.2086,2,120.3,1,205.35,0\C,26,1.4568,25,12
4.1,2,21.9,0\H,29,1.1146,26,109.53,25,169.88,0\H,29,1.1084,26,111.81,2
5,285.38,0\C,11,3.3313,6,141.82,5,117.35,0\C,32,1.3939,11,73.38,6,219.
47,0\C,33,1.3953,32,120.12,11,73.9,0\C,34,1.4022,33,120.8,32,359.87,0\
C,35,1.4022,34,118.43,33,0.4,0\C,32,1.3938,11,84.97,6,342.53,0\H,32,1.
1027,11,111.89,6,103.04,0\H,33,1.1029,32,119.98,11,253.97,0\H,34,1.102
9,33,119.21,32,180.22,0\H,36,1.1033,35,120.,34,180.23,0\H,37,1.1027,32
,120.05,11,112.27,0\\Version=IA32L-G03RevD.01\State=1-A\HF=-953.700207
6\RMSD=2.541e-06\Thermal=0.\Dipole=1.6389773,-0.7246235,-0.2481071\PG=
C01 [X(C17H20N4O1)]\\@

393-Ac$^+$

1\1\GINC-NODE24\SP\RMP2-FC\6-31+G(2d,p)\C19H23N4O2(1+)\ZIP01\30-Oct-20
09\0\\#P MP2/6-31+G(2d,p) scf=(direct,tight) int=finegrid geom=check g
uess=read\\sp of cat81ur3_43.ac2.sps MP2-5/6-31+G(2d,p)\\1,1\C,0,0.028
1068591,-0.087993646,-0.0047874484\N,0,0.0313410679,-0.1478749824,1.38
53887646\C,0,1.3089309122,-0.0444120879,-0.6933219748\C,0,-1.12899387,
-0.012614604,-0.745439206\C,0,1.1866254638,0.4796077439,2.030482267\C,
0,2.4520107594,-0.1535390049,1.4644228102\H,0,1.1826907779,1.564692483
2,1.8630910216\H,0,1.1124323856,0.3033237385,3.1040263732\N,0,2.460989
0761,-0.0511913119,-0.0073100813\H,0,3.3336135204,0.3767952505,1.83910
55701\H,0,2.5223150787,-1.2072362847,1.7636106094\C,0,3.77858699,-0.10
52749154,-0.6712619425\C,0,4.1901997823,-1.5266146167,-1.0789155624\H,
0,3.7704165882,0.5736911308,-1.5306148143\H,0,4.5002773706,0.308756830
5,0.0392802968\H,0,4.2095348264,-2.1942106865,-0.2106329958\H,0,3.5015
023066,-1.951022658,-1.818649803\H,0,5.1935610463,-1.5048898575,-1.518
7127712\C,0,1.2582237808,0.0047044514,-2.1200797801\C,0,0.0657078614,0
.043242906,-2.7897083621\H,0,2.1656958428,-0.0062965296,-2.7094916632\

N,0,-1.1217128116,0.0341322168,-2.1185329982\H,0,0.0245461494,0.073548 2534,-3.8702350244\H,0,-2.1123071381,-0.0139059367,-0.2917086872\C,0,- 1.0544315679,-0.5083900151,2.2274583429\N,0,-1.8968657259,-1.496064647 4,1.761218156\H,0,-2.6441184791,-1.6397469377,2.4307227466\O,0,-1.1965 83911,0.0338058613,3.307288128\C,0,-1.4864324342,-2.6973092721,1.01276 8044\H,0,-2.280857445,-3.4327510965,1.1879580926\H,0,-1.4963490506,-2. 4956431438,-0.066547104\C,0,2.3416707368,-4.4315597781,2.0916174984\C, 0,1.9648802544,-4.3257596465,0.7491065323\C,0,0.7354228987,-3.74813652 2,0.410460298\C,0,-0.1362078306,-3.2839453174,1.4053742766\C,0,0.24530 20824,-3.4027354949,2.7520542219\C,0,1.4776580183,-3.967764023,3.09184 47778\H,0,3.2930902686,-4.8846172773,2.3593679929\H,0,2.6210254707,-4. 6998507201,-0.0337381392\H,0,0.4461086864,-3.6721123565,-0.6372394792\ H,0,-0.4190558227,-3.0512267776,3.5391144207\H,0,1.7574929393,-4.05906 9192,4.1385993042\C,0,-2.4336818678,0.0716798735,-2.7953063018\O,0,-3. 4178612605,-0.0037802115,-2.1126965595\C,0,-2.4265328336,0.2080575401, -4.2980698519\H,0,-1.9047059699,1.1173347842,-4.6192846677\H,0,-1.9516 994706,-0.6564017835,-4.7780958358\H,0,-3.4678353713,0.2613653558,-4.6 20554675\\Version=AM64L-G03RevD.01\State=1-A\HF=-1100.3359569\MP2=-110 4.1725549\RMSD=3.934e-09\Thermal=0.\PG=C01 [X(C19H23N4O2)]\\@

394

1\1\GINC-NODE9\SP\RMP2-FC\6-31+G(2d,p)\C16H18N4O1\ZIP01\16-Sep-2009\0\ \#P MP2/6-31+G(2d,p) scf=(direct,tight) int=finegrid geom=check guess= read\\sp of cat81ur1_2c2.sps MP2-5/6-31+G(2d,p)\\0,1\C,0,-0.042973403, -0.0037669164,0.1860245599\N,0,0.0716151038,-0.2306278105,1.5833254265 \C,0,1.1305024662,-0.1214420951,-0.6189679158\C,0,-1.232286468,0.42640 95112,-0.4103309455\C,0,1.3663647298,0.1773158995,2.1233036396\C,0,2.4 768562654,-0.5869379771,1.3985863496\H,0,1.4844905707,1.2579849074,1.9 761306984\H,0,1.3773018779,-0.0440346483,3.1910599368\N,0,2.333445623, -0.4667965258,-0.0536647301\H,0,2.4523105988,-1.6454423607,1.702156695 2\H,0,3.4557731352,-0.1802433468,1.6893141953\C,0,3.5361466687,-0.6800 670808,-0.8541640888\C,0,4.3306496235,0.6065665507,-1.1301792492\H,0,3 .258930553,-1.1723281078,-1.793885928\H,0,4.1637504888,-1.397219236,-0 .3095490918\H,0,5.2319236371,0.3815268977,-1.7139654376\H,0,3.72795489 18,1.3303434641,-1.6904292754\H,0,4.6437664293,1.0818922898,-0.1923984 961\C,0,0.9584446904,0.1419075341,-1.996653105\C,0,-0.2989951193,0.497 5797221,-2.4787535138\H,0,1.7911150608,0.0769554251,-2.6892473609\N,0, -1.3937102635,0.6526988252,-1.7212249925\H,0,-0.4253548553,0.687500713 6,-3.5445225763\H,0,-2.1063929551,0.5874464779,0.2217236825\C,0,-0.670 2208219,-1.1562010523,2.3418620429\O,0,-0.4212658911,-1.3359755396,3.5 277421677\N,0,-1.6638933358,-1.8088925751,1.6474284724\H,0,-1.69971611 29,-1.6492216961,0.6493809702\C,0,-2.6149237326,-2.7259213879,2.141848 5768\C,0,-2.7358769822,-3.0759449048,3.4979270084\C,0,-3.4892965942,-3 .2993589553,1.1999817537\C,0,-3.723110872,-3.9868328661,3.8838320365\H ,0,-2.0626912118,-2.6403596204,4.2242621679\C,0,-4.4677855276,-4.20691 22886,1.6033080679\H,0,-3.4014084444,-3.0290049994,0.1481977968\C,0,-4 .5926331493,-4.5584095552,2.9509991155\H,0,-3.8074434736,-4.250062081, 4.9361650621\H,0,-5.1335836984,-4.6375519834,0.8586691856\H,0,-5.35494 97899,-5.2659197289,3.26756945\\Version=AM64L-G03RevD.01\State=1-A\HF= -909.1272343\MP2=-912.3179178\RMSD=7.795e-09\Thermal=0.\PG=C01 [X(C16H 18N4O1)]\\@

394-Ac^{+}

1\1\GINC-NODE7\SP\RMP2-FC\6-31+G(2d,p)\C18H21N4O2(1+)\ZIP01\17-Sep-200 9\0\\#P MP2/6-31+G(2d,p) scf=(direct,tight) int=finegrid geom=check gu ess=read\\sp of cat81ur1_5c1.ac2.sps MP2-5/6-31+G(2d,p)\\1,1\C,0,0.055 8758093,0.053803855,0.0120251985\N,0,0.0746375609,0.1159749231,1.40797 36246\C,0,1.3314220626,-0.0161053792,-0.6764729386\C,0,-1.1023332464,0

.0163586451,-0.7266086377\C,0,1.184143205,-0.6211740884,2.0233315846\C
,0,2.4925712344,-0.061854059,1.4827262719\H,0,1.1223802301,-0.49054922
5,3.1042345819\H,0,1.0989620143,-1.6948517991,1.8079624175\N,0,2.48361
73081,-0.0226439864,0.0097092981\H,0,3.3268238718,-0.7004166607,1.7955
251735\H,0,2.6743720038,0.9503908783,1.8673360763\C,0,3.8087363639,-0.
0222399037,-0.6407556152\C,0,4.3141021257,-1.4319833207,-0.9747309271\
H,0,4.4921079237,0.4737613063,0.0569643113\H,0,3.7678190831,0.61220203
92,-1.5318101543\H,0,5.3133545935,-1.3679358052,-1.4195039028\H,0,4.38
59017886,-2.0513792218,-0.0733499418\H,0,3.6538796023,-1.9407674076,-1
.6862479798\C,0,1.2819650919,-0.0652038781,-2.1032144519\C,0,0.0891530
725,-0.0461947234,-2.7718595282\H,0,2.1899367638,-0.1141400182,-2.6902
100708\N,0,-1.0971098174,-0.0055002355,-2.0990235609\H,0,0.0458102218,
-0.0675470947,-3.8524244612\H,0,-2.0840530439,0.0300260032,-0.27398012
1\C,0,-1.0214297973,0.4279202518,2.260689731\O,0,-1.1292303677,-0.1189
92491,3.3415010574\N,0,-1.9349874296,1.3524524588,1.7976417312\H,0,-2.
7229276761,1.4174601315,2.4347301888\C,0,-1.7015640968,2.5010970009,0.
9755080726\C,0,-0.5083291088,3.2338509024,1.0497923416\C,0,-2.72696032
52,2.9155889233,0.1128191829\C,0,-0.3386319472,4.3632635461,0.24486656
58\H,0,0.2687362931,2.9365308506,1.7489181483\C,0,-2.557476844,4.06137
67998,-0.6697081717\H,0,-3.6513774999,2.3444743925,0.062876799\C,0,-1.
3608476507,4.7825891096,-0.6136343046\H,0,0.5842750388,4.9342364937,0.
3120312118\H,0,-3.363109309,4.387661043,-1.3226945741\H,0,-1.230491893
8,5.6734607169,-1.2223880422\C,0,-2.4082578846,0.0281931267,-2.7738629
391\O,0,-3.3914133985,0.0996982798,-2.0883176148\C,0,-2.405313856,-0.0
334517412,-4.2815367255\H,0,-1.8816073559,0.8256007152,-4.7181548178\H
,0,-1.9338703419,-0.9537782629,-4.6466661798\H,0,-3.4470693047,-0.0152
529637,-4.6063877026\\Version=AM64L-G03RevD.01\State=1-A\HF=-1061.3017
974\MP2=-1064.9796316\RMSD=3.380e-09\Thermal=0.\PG=C01 [X(C18H21N4O2)]
\\@

395

1\1\GINC-NODE3\FOpt\RB98\6-31G(d)\C53H49N3\ZIP01\25-Dec-2010\0\\#P b98
/6-31G(d) opt=(maxCycle=60) scf=tight\\opt of cat6ph_3 b98/6-31G(d) on
4p 900 MB\\0,1\C,3.0108438415,0.5024885335,-2.3452091915\C,2.93827646
18,0.4939955242,-0.7876010018\C,2.1139274767,-1.5437655286,-1.37823090
07\C,2.4273861297,-0.8903612459,-2.7519462622\H,2.442309569,1.33984723
95,-2.7707839663\H,4.0459365924,0.6231792851,-2.6844464901\H,1.5260913
287,-0.8022619745,-3.3715285401\H,3.1483405292,-1.4821981397,-3.325411
6531\C,3.2731008081,-1.0131541594,-0.4770646075\C,1.3824878247,0.60191
17686,-0.4801037724\H,0.9592248198,1.2943046025,-1.2157861543\C,0.8359
061076,-0.8183357039,-0.8478488553\H,0.1271383468,-0.6964696514,-1.680
5392142\H,2.0088367,-2.633840321,-1.4160615173\C,4.6692773126,-1.46890
97935,-0.9670308703\H,4.8674493128,-1.2709882663,-2.0238419455\H,5.458
8974068,-0.97644746,-0.3845893785\H,4.7795325425,-2.550074895,-0.80852
09088\C,3.212876622,-1.4132126529,1.0113022318\H,3.2669643192,-2.50580
12927,1.1086964228\H,4.0801166052,-0.9964234824,1.5410742591\H,2.32217
19733,-1.0825010645,1.5424326145\C,3.8089948696,1.554745872,-0.1201688
266\H,3.5928052463,2.5585289415,-0.5111526043\H,3.6558387058,1.5748460
135,0.9651905346\H,4.8716299824,1.3578857952,-0.3125285907\N,1.0030665
826,1.1213442244,0.8300759221\N,0.1302314061,-1.5408005855,0.205132312
9\C,0.3703078348,0.3194226195,1.7813062865\C,-0.1059673576,-0.98475635
57,1.4635994947\C,0.1928073515,0.7593222522,3.1056701804\H,0.579390264
4,1.7238187643,3.4182578989\C,-0.472402067,-0.0383174623,4.0331964539\
H,-0.605991717,0.3084152595,5.0575907955\N,-0.9663931666,-1.2453601191
,3.7389902359\C,-0.7679194216,-1.6887014601,2.4928211736\H,-1.17814691
53,-2.6760349457,2.2945934515\C,-0.6958229936,-2.6308665812,-0.3167925
015\H,-0.0667849505,-3.2445792194,-0.9735358276\C,0.742851362,2.579761
7819,0.8650837736\H,1.4093924232,3.0380294229,0.1292701454\H,-0.990164

4425,-3.2877174324,0.5053207709\H,1.0423256156,2.9826834821,1.83811465
13\C,-1.9300185682,-2.1859541961,-1.1048562977\C,-2.1125961401,-2.6351
534689,-2.4178777033\C,-2.881208423,-1.3231518086,-0.5401246868\C,-3.2
365810591,-2.2499337771,-3.1716366258\H,-1.3569360772,-3.2723152076,-2
.8750983169\C,-4.0231194933,-0.929536663,-1.2598012956\H,-2.7200831554
,-0.9322636085,0.4611327744\C,-4.1850074844,-1.4086343323,-2.572078241
7\H,-5.0877377557,-1.1559682533,-3.1218759607\C,-0.7080318772,2.943716
1403,0.5665027874\C,-1.6128924665,3.1687405278,1.6116413621\C,-1.17574
16375,3.010760186,-0.7543428419\C,-2.972853371,3.4225678033,1.36301375
2\H,-1.2583695414,3.1578829236,2.638464056\C,-2.5301433309,3.254670576
2,-1.0376746284\H,-0.4799684675,2.8967876,-1.5831387173\C,-3.416411197
1,3.4604156066,0.0321347325\H,-4.4768565624,3.5810553479,-0.172616076\
C,-3.9274225346,3.6051714156,2.4907027407\C,-4.9582237536,4.5600152357
,2.4211730326\C,-3.8283923779,2.8107870374,3.6486745745\C,-5.862213210
4,4.716185432,3.4742516577\H,-5.0370941463,5.1972465714,1.5430331847\C
,-4.732736504,2.9674081923,4.7018735724\H,-3.0576573261,2.0448554865,3
.708233209\C,-5.7536431149,3.9200713317,4.6192644615\H,-6.6468175134,5
.466741087,3.4041194218\H,-4.6441464249,2.3371638801,5.5842002674\H,-6
.4573464864,4.0417824696,5.4397341203\C,-3.0244591486,3.254725604,-2.4
423837423\C,-3.9710253961,4.1995952634,-2.8799217549\C,-2.572408212,2.
2891657686,-3.3614401558\C,-4.4501673304,4.1799045098,-4.1917845186\H,
-4.3177590435,4.9651052674,-2.1891294569\C,-3.0496266308,2.2700913965,
-4.6747792495\H,-1.8695946962,1.5270047281,-3.0321561262\C,-3.99177368
69,3.2150089581,-5.0951916857\H,-5.1760871723,4.9245823626,-4.51142234
97\H,-2.6907149738,1.5099888022,-5.3660151857\H,-4.3638599233,3.201101
5417,-6.1172074365\C,-5.0479010768,-0.0370110434,-0.648958932\C,-5.766
1473581,0.8830582139,-1.4366021675\C,-5.3239369431,-0.0869103077,0.730
8933878\C,-6.7286028233,1.7186361667,-0.8648202048\H,-5.545591743,0.97
25525864,-2.4975201834\C,-6.2776713469,0.7562185729,1.3043376294\H,-4.
7993324858,-0.803130902,1.3589400725\C,-6.9869515508,1.661732525,0.508
7029481\H,-7.2654038107,2.4254585353,-1.4943936862\H,-6.4649081305,0.7
076264363,2.3743983479\H,-7.7273430111,2.3198878012,0.9574736103\C,-3.
4151342899,-2.7160997798,-4.5742402475\C,-3.9296398714,-1.8501081137,-
5.5580675129\C,-3.0730561376,-4.0271477193,-4.9544787549\C,-4.09845261
82,-2.2817459922,-6.8755280885\H,-4.1765015951,-0.8247972886,-5.289592
1113\C,-3.2403208119,-4.4582618682,-6.2724249473\H,-2.6992794535,-4.72
08635944,-4.2045018619\C,-3.7541822079,-3.587699078,-7.2387860647\H,-4
.4922442597,-1.5942394869,-7.6211845465\H,-2.9781286313,-5.4790657775,
-6.5422187473\H,-3.8854386618,-3.9238266633,-8.2648119621\\Version=AM6
4L-G03RevD.01\State=1-A\HF=-2212.5210311\RMSD=5.824e-09\RMSF=1.543e-06
\Thermal=0.\Dipole=0.3438543,0.6246874,-1.4365535\PG=C01 [X(C53H49N3)]
\\@

395-Ac$^+$

1\1\GINC-NODE9\SP\RMP2-FC\6-31+G(2d,p)\C55H52N3O1(1+)\ZIP01\20-Jan-201
1\0\\#P MP2/6-31+G(2d,p) scf=(direct,tight) int=finegrid geom=check gu
ess=read\\sp of cat6ph_1.ac1 MP2-5/6-31+G(2d,p)\\1,1\C,0,1.2085268031,
-0.7226334104,5.1806393979\C,0,1.5138694532,-0.2425772116,3.719280686\
C,0,-0.7590762646,-0.1797438188,3.8873876172\C,0,-0.3436722702,-0.6232
356861,5.3107611499\H,0,1.5853475648,-1.7375288678,5.359514084\H,0,1.7
180601135,-0.0728886835,5.8995233292\H,0,-0.8071640747,-1.5746627456,5
.5974091274\H,0,-0.6428824506,0.1149992864,6.060156833\C,0,0.374288262
1,0.816872718,3.4921363432\C,0,1.0430365065,-1.4747767843,2.8366613243
\H,0,1.4057540155,-2.3926782884,3.3197670784\C,0,-0.5228587013,-1.4315
02009,2.987623484\H,0,-0.8263419696,-2.335804087,3.5379391212\H,0,-1.7
767463115,0.2180422412,3.8198191466\C,0,0.4573594523,2.0404140992,4.43
5982635\H,0,0.6532628657,1.7933232674,5.4826135894\H,0,1.2497529998,2.
7228213567,4.104854039\H,0,-0.4858056779,2.6002443385,4.3981095337\C,0

,0.2940441145,1.3971588047,2.0654753937\H,0,-0.5123189352,2.1392718279
,2.0089179006\H,0,1.22460802,1.9244060639,1.819834291\H,0,0.1098277988
,0.653265381,1.2883051024\C,0,2.9499958845,0.2638544819,3.5811960913\H
,0,3.684311848,-0.4835454946,3.9097523022\H,0,3.2041533932,0.576027694
2,2.5631703746\H,0,3.0819296769,1.1342126289,4.2349436635\N,0,1.529136
4584,-1.5875303676,1.4365751299\N,0,-1.2944705318,-1.4115361463,1.7360
085771\C,0,0.7048082198,-1.9406382757,0.4268167414\C,0,-0.7338292217,-
1.904099921,0.5813248102\C,0,1.2061588099,-2.3640478838,-0.8383664915\
H,0,2.2683648856,-2.3618419976,-1.0408166398\C,0,0.3687605851,-2.75586
99811,-1.8499104214\H,0,0.749695067,-3.0738112128,-2.8105543444\N,0,-0
.9797799314,-2.7635098196,-1.6695194118\C,0,-1.5138317529,-2.353815096
7,-0.473386943\H,0,-2.5927633116,-2.3730265011,-0.4459654476\C,0,-2.75
09783931,-1.5620081662,1.9164737968\H,0,-3.0366739892,-2.6170962377,1.
7669835049\H,0,-2.9603425366,-1.3361348706,2.9664521887\C,0,2.97380630
65,-1.8656984266,1.2840845719\H,0,3.409304719,-1.820487771,2.283510563
9\H,0,3.0903170297,-2.9076439881,0.9485694057\C,0,3.7412755516,-0.9337
295469,0.3482517541\C,0,5.0647283533,-1.2696381159,0.0328904458\C,0,3.
1840379628,0.2370276069,-0.1727362639\C,0,5.8434599917,-0.4402050333,-
0.7924379228\H,0,5.5117448696,-2.1678850311,0.4561654195\C,0,3.9330799
977,1.0832956599,-1.0120855609\H,0,2.1668448855,0.5145973545,0.0841560
907\C,0,5.2580276158,0.7305890639,-1.3085319532\H,0,5.8306328403,1.347
739223,-1.9960538334\C,0,-3.6030076749,-0.6640051101,1.025892309\C,0,-
3.2457621704,0.6685291841,0.7956505405\C,0,-4.7924761857,-1.1635769476
,0.4826247764\C,0,-4.0642152635,1.5139302016,0.0269555786\H,0,-2.33220
82358,1.0603750467,1.2306410659\C,0,-5.6350155952,-0.3436508048,-0.290
4107469\H,0,-5.0874598099,-2.1942025251,0.6751088993\C,0,-5.2554048922
,0.9913050311,-0.5053958256\H,0,-5.8719941182,1.618442766,-1.144604403
4\C,0,3.3137164827,2.3117554599,-1.5816873607\C,0,1.9692838224,2.31152
27932,-1.9994544788\C,0,4.0545803726,3.5004603183,-1.716431397\C,0,1.3
841749117,3.4608457147,-2.5358697689\H,0,1.3881555845,1.3936242008,-1.
9336478707\C,0,3.4696818359,4.6505760696,-2.2505256055\H,0,5.087696080
4,3.5301730181,-1.3777228613\C,0,2.1330398879,4.6358098583,-2.66218906
49\H,0,0.3479329711,3.4359079936,-2.8663526025\H,0,4.057498517,5.56093
95018,-2.340023632\H,0,1.6807504065,5.5303494922,-3.0834804905\C,0,7.2
491755901,-0.7990343066,-1.1248888558\C,0,8.2432503449,0.1939386403,-1
.2023223771\C,0,7.6163823739,-2.1355418421,-1.3681468798\C,0,9.5630166
26,-0.1398739139,-1.5124737219\H,0,7.9858417841,1.229477744,-0.9912116
084\C,0,8.9360036947,-2.4687991244,-1.6797905586\H,0,6.8578607782,-2.9
151769352,-1.3420869711\C,0,9.9141862209,-1.4720531595,-1.752727506\H,
0,10.3190035673,0.6403164596,-1.5586453774\H,0,9.1987032371,-3.5059569
678,-1.8744331699\H,0,10.9417552351,-1.7313867874,-1.9952158738\C,0,-3
.6653338629,2.9266362301,-0.2232300524\C,0,-4.6239049861,3.95653933,-0
.2316985945\C,0,-2.3180846192,3.2670120018,-0.4509999551\C,0,-4.247212
8926,5.2824812166,-0.4570270923\H,0,-5.66689614,3.7199275083,-0.033737
9634\C,0,-1.9403958858,4.5933062601,-0.6732346962\H,0,-1.5635451701,2.
4832370681,-0.4796538014\C,0,-2.9047522211,5.6064947229,-0.6770938135\
H,0,-5.0027806373,6.0645033903,-0.4511441502\H,0,-0.8936817542,4.83154
20081,-0.8504845273\H,0,-2.6130128497,6.6395883625,-0.8497429713\C,0,-
6.8865242174,-0.8878353955,-0.8840853482\C,0,-6.9082721708,-2.17008645
15,-1.463355928\C,0,-8.0720007091,-0.1304909464,-0.8790907157\C,0,-8.0
843067875,-2.6804851164,-2.0173032672\H,0,-5.9907015296,-2.7532926532,
-1.5108909155\C,0,-9.2475238195,-0.6428852956,-1.4317940495\H,0,-8.077
8386848,0.8534023246,-0.4149905079\C,0,-9.2579202773,-1.919928995,-2.0
017728568\H,0,-8.0819220383,-3.6695190728,-2.4696870873\H,0,-10.157383
7234,-0.0477227261,-1.4099436289\H,0,-10.1734071587,-2.3186540761,-2.4
320276928\C,0,-1.9236551336,-3.2181261662,-2.7029738521\O,0,-3.0935655
48,-3.2492167199,-2.4302929063\C,0,-1.3417572173,-3.6153039364,-4.0387
089801\H,0,-0.6664611889,-4.4741211588,-3.9432722702\H,0,-2.1774257326

,-3.8927384523,-4.6833872933\H,0,-0.7909273979,-2.78793385,-4.50136878
69\\Version=AM64L-G03RevD.01\State=1-A\HF=-2351.2736894\MP2=-2359.8786
513\RMSD=4.284e-09\Thermal=0.\PG=C01 [X(C55H52N3O1)]\\@

397

1\1\GINC-GOLEM\SP\RHF\6-31G(d)\C20H24N4O3\EVGENY\13-Mar-2010\0\\#P RHF
/6-31G(d) scf=tight int=finegrid SCRF=(PCM,Read,Solvent=Chloroform) ge
om=check guess=read\\PCM of cat81ur7_37 b98/6-31G(d) structures on 4p
900 MB\\0,1\C,0,-0.0954090373,0.385297462,-0.162213952\C,0,-0.09659405
32,0.1574253074,1.2169355727\C,0,1.1309543261,-0.1695388761,1.86581823
93\C,0,2.2460304224,-0.3343129413,1.0126713331\C,0,2.104117927,-0.1362
551203,-0.3578443656\H,0,-1.0254107847,0.6953963502,-0.6368633473\H,0,
3.215079046,-0.6233449321,1.4046417553\H,0,2.969471428,-0.2718414853,-
1.0065584967\C,0,-1.0080610407,0.7932693737,3.3597619259\H,0,-0.584593
7133,1.8038862678,3.3125994027\H,0,-1.9564466214,0.8213827657,3.897700
9845\C,0,-0.0247180157,-0.1694839531,4.0323688447\H,0,-0.512895641,-1.
1489127708,4.1612817462\H,0,0.2471648296,0.205741894,5.0279172449\N,0,
1.1987594896,-0.2767963812,3.2374377061\N,0,-1.2712949979,0.321247181,
2.0023829666\N,0,0.9653510673,0.2279711907,-0.9649667683\C,0,2.3829486
337,-0.8145165675,3.9029883913\H,0,2.338932162,-0.4819407985,4.9475227
073\H,0,3.277154556,-0.3472091242,3.4725834994\C,0,2.4942981681,-2.347
0668834,3.8461955472\H,0,2.5514064154,-2.7004322276,2.8102999606\H,0,1
.6252420415,-2.821749632,4.3166512793\H,0,3.3953488581,-2.6825048035,4
.3749721275\C,0,-2.4620340882,-0.3836412141,1.8031114695\O,0,-3.411198
0294,-0.2785358167,2.5755323294\H,0,-1.6356934777,-1.5509442368,0.3480
646353\N,0,-2.5225549989,-1.1725998166,0.667165529\C,0,-3.6236904668,-
2.1137051091,0.6081214249\H,0,-4.5374779199,-1.5473607981,0.7966428879
\C,0,-3.7258897608,-2.7430616093,-0.8148803729\H,0,-4.5611005221,-3.45
57894321,-0.8140290257\H,0,-3.9932949461,-1.9295951476,-1.5004194659\C
,0,-3.4475056713,-3.2095508919,1.6854016775\O,0,-2.3589231479,-3.55335
104,2.0769056657\C,0,-0.0804310313,-4.6718339422,-2.153291433\C,0,-1.0
530301491,-5.4078142004,-1.4680261007\C,0,-2.2310954285,-4.7899969026,
-1.0417450262\C,0,-2.4610143034,-3.4290607258,-1.2996782743\C,0,-1.481
8779657,-2.7034603613,-1.9988301429\C,0,-0.296146045,-3.3162214012,-2.
4161244778\H,0,0.8385081144,-5.1529873451,-2.4806899621\H,0,-0.8931016
815,-6.463565561,-1.2607091104\H,0,-2.9774763407,-5.3685469407,-0.5000
089007\H,0,-1.6458369256,-1.6500819743,-2.2193478661\H,0,0.4529725626,
-2.72874087,-2.9415383077\O,0,-4.5478320354,-3.8597695945,2.1346919601
\C,0,-5.8506657238,-3.2885401025,1.9869933225\H,0,-6.1690717328,-3.252
7830347,0.9376679745\H,0,-5.889552697,-2.2859576904,2.4272966034\H,0,-
6.5214090846,-3.9528368281,2.5367247678\\Version=AM64L-G03RevD.01\Stat
e=1-A\HF=-1213.7515594\RMSD=1.715e-09\Thermal=0.\Dipole=0.2034681,-0.0
477148,0.4640657\PG=C01 [X(C20H24N4O3)]\\@

397-Ac$^+$

#P b98/6-31G(d) opt=(maxCycle=60) scf=tight freq=noraman\\opt of cat81
ur7_22c.ac2 b98/6-31G(d) on 4p 900 MB\\1,1\C,0.2609558574,1.0893720409
,0.7347403916\C,0.0770976523,0.2507188201,1.8152762869\C,1.2336083262,
-0.3424825801,2.4618116285\C,2.5079194253,0.1266559736,2.0213205707\C,
2.6145960788,1.0225456749,0.9934957212\H,-0.5907033569,1.4785450863,0.
1825415996\H,3.42097632,-0.2013700981,2.500951418\H,3.5605486409,1.408
7927518,0.6331857691\C,-1.2826620623,-1.5022737022,2.7498505764\H,-2.2
971766218,-1.6617893936,3.1183098259\H,-1.0994310757,-2.20670517,1.928
086538\C,-0.2572182964,-1.6951922391,3.8635886399\H,-0.1998795464,-2.7
530527573,4.1416677971\H,-0.5460591218,-1.122165244,4.754506295\N,1.08
54270855,-1.2760760499,3.4162400696\N,-1.1894298582,-0.1253222912,2.26
81306226\N,1.5121929995,1.4884021469,0.3388545528\C,2.2357812531,-1.84

85873455,4.143791243\H,1.9218465512,-2.8371981158,4.4927007585\H,3.052
2180482,-2.0175863207,3.4340590197\C,2.6914314917,-0.986499055,5.32902
04541\H,1.8830776352,-0.8564730714,6.0570694688\H,3.5287763906,-1.4767
513406,5.8380098542\H,3.0238040662,0.0059996294,5.0036013671\C,-2.3802
031323,0.6474349184,2.225114318\O,-3.4694227823,0.1045413105,2.2608541
285\H,-1.2720092765,2.3946715294,2.190135462\N,-2.2056079896,2.0063435
893,2.2225400509\C,-3.2698651784,2.8720578184,1.7534225252\H,-4.217887
9828,2.4916403128,2.1464177105\C,-3.0599311955,4.325769001,2.267022354
\H,-3.9007965937,4.9286227123,1.907942308\H,-3.1198901132,4.2952838135
,3.3611703559\C,-3.3498818943,2.8196094719,0.2154195502\O,-2.517919528
7,2.2927360282,-0.5010875116\C,0.7302721807,6.0033802438,0.9730410725\
C,0.6377216171,5.3425226234,2.2017389294\C,-0.5891440713,4.8178863655,
2.6264227953\C,-1.7403802968,4.9368561691,1.8276080824\C,-1.6350061969
,5.6136066602,0.5994449931\C,-0.4126283043,6.1448347207,0.1764977481\H
,1.677953229,6.4263337617,0.6489035283\H,1.5130751172,5.2566346164,2.8
416962225\H,-0.6619378286,4.3406341867,3.6033432407\H,-2.5227020959,5.
7451878151,-0.01705524\H,-0.35524697,6.6831221591,-0.7668376556\O,-4.4
440034428,3.4321144134,-0.2288795921\C,-4.6489041109,3.4320251619,-1.6
574248541\H,-3.8316827028,3.9614107185,-2.1557367367\H,-5.596150785,3.
9485146782,-1.8125771748\H,-4.7009780114,2.404979839,-2.0278941731\C,1
.7397041949,2.4216713007,-0.79338926\O,2.8693001516,2.7725030741,-0.99
80019638\C,0.5335647973,2.8299289533,-1.5923158369\H,-0.0141336889,1.9
615418725,-1.9749997885\H,-0.163390867,3.4233126154,-0.9890657776\H,0.
8930363007,3.4344447318,-2.4270203713\\Version=AM64L-G03RevD.01\State=
1-A\HF=-1373.81987\RMSD=8.496e-09\RMSF=2.341e-06\Thermal=0.\Dipole=1.8
144195,-1.6446806,0.751559\PG=C01 [X(C22H27N4O4)]\\@

398

1\1\GINC-NODE13\SP\RMP2-FC\6-31+G(2d,p)\C33H41N3\ZIP01\29-Dec-2010\0\\
#P MP2/6-31+G(2d,p) scf=(direct,tight) int=finegrid geom=check guess=r
ead\\sp of cat6me_1 MP2-5/6-31+G(2d,p)\\0,1\C,0,-0.7106530989,3.686417
4514,-1.1982326807\C,0,-1.0839149998,2.4682496514,-0.2910800405\C,0,1.
1922582452,2.4491241738,-0.3610054297\C,0,0.850317444,3.6960401929,-1.
211984564\H,0,-1.1405563572,3.5808411894,-2.2027710404\H,0,-1.11907382
38,4.6133995431,-0.7798416062\H,0,1.2631109222,3.622275232,-2.22581379
89\H,0,1.2562364151,4.6109993717,-0.7680850348\C,0,0.0967761434,2.4772
937253,0.749236049\C,0,-0.7549536736,1.2061871607,-1.1952328045\H,0,-1
.1093316715,1.4240423134,-2.2163441144\C,0,0.8198282827,1.2191586112,-
1.2418361496\H,0,1.1102828298,1.4204892324,-2.2878483689\H,0,2.2247288
031,2.4210670158,0.0037012444\C,0,0.1629527851,3.7504898402,1.62635236
39\H,0,0.0664268522,4.6893338776,1.0732306574\H,0,-0.6290193942,3.7337
893052,2.3862744826\H,0,1.1217919773,3.7793241669,2.1612287275\C,0,0.1
091604015,1.28926138,1.7333957087\H,0,1.0029821061,1.3451844892,2.3694
289432\H,0,-0.765766379,1.3452450652,2.3953999143\H,0,0.1030925773,0.3
129717289,1.2505751398\C,0,-2.4931569894,2.5967272002,0.2869973261\H,0
,-3.2453659841,2.7544169955,-0.4977663284\H,0,-2.7918837523,1.72250623
87,0.8741078285\H,0,-2.538810053,3.4744647747,0.9447351945\N,0,-1.3589
483875,-0.0798463928,-0.8019582232\N,0,1.4967019357,-0.0089188784,-0.8
129597543\C,0,-0.608124903,-1.2541687719,-0.9572366367\C,0,0.813384347
3,-1.2245401728,-0.9721168794\C,0,-1.2154907612,-2.5177633945,-1.07585
17546\H,0,-2.2931065557,-2.6248690946,-1.0451016194\C,0,-0.4296359039,
-3.663206607,-1.1998953094\H,0,-0.9084873208,-4.6380864776,-1.28916596
89\N,0,0.9046811374,-3.6501037341,-1.2160757441\C,0,1.4914016334,-2.45
08365269,-1.1031151883\H,0,2.5775912412,-2.4681552961,-1.0928233507\C,
0,2.9261270635,-0.0004106365,-1.1389971713\H,0,3.1097609729,-0.5668930
212,-2.0690860225\H,0,3.1982546924,1.0374231507,-1.3645829207\C,0,-2.7
817010887,-0.1869146931,-1.1488193688\H,0,-3.1316173377,0.8159622532,-
1.4084293006\H,0,-2.8965835662,-0.7909028166,-2.0655022854\C,0,-3.6989

335111,-0.7466661005,-0.0643510734\C,0,-5.037242848,-1.0144931335,-0.4
000920252\C,0,-3.2667482853,-0.9831200633,1.2414664985\C,0,-5.93990868
25,-1.5004568227,0.549705369\H,0,-5.3795896034,-0.8389622943,-1.420477
5571\C,0,-4.1510093751,-1.476785161,2.2178972715\H,0,-2.2274961291,-0.
7934787834,1.4991971967\C,0,-5.4784766223,-1.7256327218,1.8588268976\H
,0,-6.1709722378,-2.1084425478,2.608439679\C,0,3.8645475866,-0.5029650
473,-0.0460000401\C,0,3.461861464,-0.6307439004,1.2839406279\C,0,5.194
658327,-0.7974509195,-0.3889761845\C,0,4.3679715866,-1.0433747792,2.27
64037526\H,0,2.4287371955,-0.4204263626,1.5466903554\C,0,6.1200704424,
-1.2023529274,0.577417674\H,0,5.5135642472,-0.7067462048,-1.4278023142
\C,0,5.689312036,-1.316934312,1.910373088\H,0,6.3998978697,-1.63340758
64,2.6740336716\C,0,-3.6564461536,-1.7449621578,3.6243629687\H,0,-2.92
52161303,-2.5646002282,3.6369575953\H,0,-3.1588323226,-0.8626026467,4.
0476267581\H,0,-4.4799865359,-2.0211981346,4.2924017228\C,0,-7.3840368
559,-1.7797221405,0.1875440918\H,0,-7.6549933644,-2.8216604593,0.40354
5298\H,0,-8.06938107,-1.1423254038,0.7625264972\H,0,-7.5720190437,-1.5
9778198,-0.8765677307\C,0,3.9050753634,-1.208712077,3.7094826903\H,0,3
.2791010738,-0.3654689732,4.0279946945\H,0,3.3030144808,-2.1205899207,
3.826350184\H,0,4.7539875919,-1.2809390983,4.3989900653\C,0,7.55256170
52,-1.5240157728,0.2041308737\H,0,8.2627364989,-0.9522855799,0.8159723
942\H,0,7.7748046329,-2.5883827006,0.3607601253\H,0,7.7532704518,-1.29
35284753,-0.8482189364\\Version=AM64L-G03RevD.01\State=1-A\HF=-1436.97
72414\MP2=-1442.3571384\RMSD=5.223e-09\Thermal=0.\PG=C01 [X(C33H41N3)]
\\@

398-Ac⁺

1\1\GINC-NODE7\SP\RMP2-FC\6-31+G(2d,p)\C35H44N3O1(1+)\ZIP01\12-Jan-201
1\0\\#P MP2/6-31+G(2d,p) scf=(direct,tight) int=finegrid geom=check gu
ess=read\\sp of cat6me_1.ac1 MP2-5/6-31+G(2d,p)\\1,1\C,0,-1.3836895052
,4.0952373676,-1.6525255901\C,0,-1.636837107,2.8908655254,-0.681238372
\C,0,0.6255027645,3.177300411,-0.6735398434\C,0,0.1591587949,4.3243734
526,-1.602231887\H,0,-1.7475521618,3.8789061683,-2.6649333957\H,0,-1.9
367198078,4.9748647714,-1.3075323034\H,0,0.6287904761,4.2697402783,-2.
591594913\H,0,0.4122557518,5.3032587044,-1.1848641808\C,0,-0.509985363
4,3.1132964985,0.3947866198\C,0,-1.1037018144,1.6470522298,-1.50793872
95\H,0,-1.4615840895,1.7500791569,-2.541586927\C,0,0.4510846276,1.8750
609633,-1.5138210864\H,0,0.7488608651,2.0727582898,-2.5551400276\H,0,1
.638209505,3.300874385,-0.2757854288\C,0,-0.658654253,4.4270768526,1.1
973801439\H,0,-0.8755764284,5.3074303886,0.587152823\H,0,-1.4628302046
,4.3335937824,1.9377257287\H,0,0.2672004,4.6289529343,1.7512595363\C,0
,-0.3721132772,1.9942505707,1.4483644321\H,0,0.4503716342,2.2336103381
,2.1345912885\H,0,-1.2848369655,1.9336597132,2.0553092813\H,0,-0.17391
3525,1.0042313664,1.0357969728\C,0,-3.0776745867,2.8731527792,-0.17036
05112\H,0,-3.8053118173,2.8888175008,-0.9925102345\H,0,-3.3003223372,2
.014527124,0.4705212269\H,0,-3.2570740315,3.7805892661,0.4186922109\N,
0,-1.5358418104,0.2846680688,-1.1073933896\N,0,1.2789539899,0.75783144
28,-1.0380289835\C,0,-0.6606817452,-0.7420566326,-1.0268341064\C,0,0.7
70260061,-0.5197479049,-1.0272016241\C,0,-1.1038395428,-2.0932746145,-
0.9483789361\H,0,-2.1595395497,-2.3236048287,-0.9033738824\C,0,-0.2197
729023,-3.1384878333,-0.8942603931\H,0,-0.5557992796,-4.1640874999,-0.
8307575205\N,0,1.1215858971,-2.9134457904,-0.9257843554\C,0,1.60175743
72,-1.6299764829,-0.9997028335\H,0,2.6801643068,-1.5615031368,-0.99716
5172\C,0,2.7192988673,0.9317927553,-1.3076752135\H,0,2.9883597064,0.38
06775295,-2.2249057548\H,0,2.8626908817,1.992584746,-1.5374282857\C,0,
-2.9550715037,-0.0284814625,-1.3919120637\H,0,-3.4173052167,0.90688904
8,-1.7128495296\H,0,-2.9886292987,-0.7013453844,-2.2626481365\C,0,-3.7
732028378,-0.6277952993,-0.2514181846\C,0,-5.0036250674,-1.2166976482,
-0.582075964\C,0,-3.3657608715,-0.592689714,1.0858574841\C,0,-5.836506

0154,-1.7531563762,0.4074277796\H,0,-5.3223978693,-1.2546345561,-1.623
79659\C,0,-4.1795358688,-1.1270160242,2.0996886929\H,0,-2.4057291219,-
0.1553039691,1.35039658\C,0,-5.4047242086,-1.7019292669,1.7428476754\H
,0,-6.0393034226,-2.1246924224,2.5209607939\C,0,3.6612484384,0.5347445
662,-0.1772770312\C,0,3.3375210274,0.7653172024,1.1663939737\C,0,4.903
5659915,-0.0249249777,-0.4990802976\C,0,4.2423459205,0.4469178754,2.18
73824085\H,0,2.3676726344,1.1847803564,1.4208601441\C,0,5.8336615808,-
0.3479155484,0.5022496441\H,0,5.1585190116,-0.2134734762,-1.5422051878
\C,0,5.4829491665,-0.1088440566,1.836714969\H,0,6.1903847107,-0.365955
0402,2.6244564853\C,0,-3.7488782526,-1.0585238305,3.549892573\H,0,-2.6
578837784,-1.0095443388,3.644335852\H,0,-4.1617742987,-0.1656694655,4.
0395206325\H,0,-4.1016067211,-1.9295205405,4.1136932768\C,0,-7.1730644
947,-2.369508067,0.05379925\H,0,-7.282880478,-3.365822472,0.4996640788
\H,0,-8.0003923199,-1.7536534641,0.4305342326\H,0,-7.2973981317,-2.466
8956171,-1.0300423505\C,0,3.8999991671,0.7029502724,3.6402820027\H,0,2
.8521372258,1.0006305796,3.7597118681\H,0,4.0706105474,-0.1901496069,4
.2543996781\H,0,4.523917452,1.5047806215,4.0571039298\C,0,7.1866569532
,-0.9238269137,0.1430237731\H,0,7.583810607,-1.5457796904,0.952796673\
H,0,7.1334598106,-1.5370505249,-0.7638914125\H,0,7.9158531122,-0.12336
29292,-0.0439008679\C,0,2.1148594575,-3.9999694016,-0.9043171573\O,0,3
.2775261555,-3.7080247174,-0.9796274009\C,0,1.5878714829,-5.410860942,
-0.7898078388\H,0,0.9595217253,-5.6764106097,-1.6484873826\H,0,2.45320
9517,-6.0753661836,-0.7658656214\H,0,1.0030539046,-5.5508472303,0.1269
090908\\Version=AM64L-G03RevD.01\State=1-A\HF=-1589.1709308\MP2=-1595.
0331677\RMSD=5.563e-09\Thermal=0.\PG=C01 [X(C35H44N3O1)]\\@

399
see reference 9

399-Ac$^+$
see reference 9

400
see reference 9

400-Ac$^+$
see reference 9

401
see reference 9

401-Ac$^+$
see reference 9

402
see reference 9

402-Ac$^+$
see reference 9

403
1\1\GINC-NODE10\SP\RMP2-FC\6-31+G(2d,p)\C24H30N6\ZIP01\10-Apr-2010\0\\
#P MP2/6-31+G(2d,p) scf=(direct,tight) int=finegrid geom=check guess=r
ead\\sp of triaz2_3 MP2-5/6-31+G(2d,p)\\0,1\C,0,0.3612556644,0.3586022
366,-0.1962293594\C,0,0.1816069959,0.188885457,1.3277669578\C,0,1.5790
363738,0.1229542717,1.9839228257\C,0,2.4410235985,1.356174784,1.625909
8037\C,0,2.5859689347,1.5265998171,0.1034451377\C,0,1.2088655912,1.584
2331683,-0.579923089\C,0,0.4647716181,0.659520041,4.0939246713\C,0,-0.

5730857344,1.3199727093,3.3590664871\N,0,-0.5884529018,1.2766011464,1.
9603884414\C,0,-1.5256851949,2.0198418354,4.1197548562\C,0,-1.42947173
01,2.0621122985,5.5129656487\N,0,-0.4690522892,1.4528774125,6.20804681
33\C,0,0.4443491513,0.7762274852,5.4953321413\C,0,-1.7739534896,1.7764
987751,1.2630251008\C,0,-2.9890646653,0.8316010139,1.2890314809\H,0,-0
.3142086624,-0.7805363807,1.5073533861\H,0,2.0651796289,-0.7784348715,
1.5850377596\H,0,3.4291096512,1.2497202702,2.0947589072\H,0,1.97333705
45,2.2496292607,2.0606892052\H,0,3.164585047,2.4325842533,-0.121474233
6\H,0,3.1576951464,0.6787840227,-0.3060915859\H,0,1.3254110482,1.62424
35461,-1.6716043055\H,0,0.6935097054,2.5080112067,-0.2804191357\H,0,-0
.6086446932,0.3642423631,-0.7084807437\H,0,0.8709830382,-0.5515579612,
-0.5455761963\H,0,-2.0359805259,2.7484845679,1.6941427214\H,0,-1.49408
8959,1.988898634,0.2274510413\H,0,-3.2941743034,0.6072290544,2.3175720
976\H,0,-3.840184706,1.2935928747,0.7719248215\H,0,-2.7643551203,-0.11
81446165,0.7875934149\H,0,-2.3547439738,2.5330508974,3.6456165559\H,0,
1.2181624758,0.2889507304,6.0843573828\H,0,-2.1742119546,2.6126012288,
6.0877398855\N,0,2.3762846678,-3.6818976837,2.7999106351\N,0,0.3814454
256,-4.365171778,2.4038469692\N,0,1.6742120278,-4.455227949,2.01113649
32\C,0,-0.6533370659,-5.0861478086,1.6668430931\H,0,-0.1373213198,-5.9
2427671,1.1872876843\H,0,-1.3662614092,-5.4954069163,2.3907872704\C,0,
-1.3684063275,-4.2241819599,0.6384015129\C,0,-2.7461900699,-3.99398702
31,0.7344002558\C,0,-0.6514516705,-3.6636234912,-0.4311677876\C,0,-3.4
057371605,-3.2174221645,-0.2254105199\H,0,-3.3100191124,-4.4263950463,
1.5596825168\C,0,-1.308349043,-2.8881150102,-1.388802877\H,0,0.4204813
056,-3.8379004595,-0.504329128\C,0,-2.687589125,-2.6635062151,-1.28861
993\H,0,-4.4767678416,-3.0481846584,-0.1414186701\H,0,-0.7463112629,-2
.4636539431,-2.2177132718\H,0,-3.1986146921,-2.0644560396,-2.038991320
9\C,0,1.5463040634,-3.0836924023,3.7087610803\C,0,2.0550373622,-2.1226
668854,4.7428583564\H,0,2.9198838902,-2.5624083826,5.2581519313\N,0,1.
4351992458,-0.0775813452,3.4188519372\C,0,2.4984454789,-0.770668955,4.
1273687814\H,0,1.2773364794,-1.9545857193,5.4964079976\H,0,3.298960116
,-0.970933364,3.4072736817\H,0,2.9336103899,-0.1351030941,4.9135538493
\C,0,0.2574434195,-3.5122356956,3.4523803935\H,0,-0.692383635,-3.28836
12701,3.9163315337\\Version=AM64L-G03RevD.01\State=1-A\HF=-1253.048889
1\MP2=-1257.6208498\RMSD=7.170e-09\Thermal=0.\PG=C01 [X(C24H30N6)]\\@

403-Ac$^+$

1\1\GINC-NODE23\SP\RMP2-FC\6-31+G(2d,p)\C26H33N6O1(1+)\ZIP01\18-May-20
10\0\\#P MP2/6-31+G(2d,p) scf=(direct,tight) int=finegrid geom=check g
uess=read\\sp of triaz2_98.ac2 MP2-5/6-31+G(2d,p)\\1,1\C,0,-0.09381991
48,0.1685164184,0.0600560984\C,0,-0.0210324328,0.0070717595,1.59680384
73\C,0,1.4553277496,-0.039405739,2.0449180359\C,0,2.3150517617,1.11046
0365,1.4743621563\C,0,2.2324500663,1.1732760525,-0.0581253308\C,0,0.76
99599812,1.3094679617,-0.5074875285\C,0,0.8121356507,0.8129900331,4.21
71189088\C,0,-0.3368514233,1.4402350079,3.5768065199\N,0,-0.7411219803
,1.0722697313,2.3461859436\C,0,-1.0311174005,2.4289800797,4.3323239363
\C,0,-0.6960640583,2.7124632472,5.6282709149\C,0,1.0789671881,1.145260
0411,5.533173004\C,0,-2.1278272982,1.3809205317,1.9199420248\C,0,-2.29
46280933,2.6874180104,1.1333586967\H,0,-0.4951304913,-0.9405417412,1.8
878223522\H,0,1.8505870586,-0.9880006006,1.6611459532\H,0,3.3513031252
,0.9663650499,1.8073181378\H,0,1.9760384354,2.0650484724,1.9027780685\
H,0,2.8301751179,2.0139294712,-0.4307828307\H,0,2.6693383266,0.2602038
293,-0.4905822781\H,0,0.7009522351,1.2995678365,-1.6023071683\H,0,0.38
18154387,2.2828324035,-0.176242778\H,0,-1.1334831239,0.2614574753,-0.2
739998484\H,0,0.2601795491,-0.7865048544,-0.3553532259\H,0,-2.46874109
44,0.5311010953,1.3219204575\H,0,-2.7575927653,1.3853228842,2.81599447
76\H,0,-1.7823900737,2.6486057741,0.1676195088\H,0,-1.9065201491,3.551
3086305,1.6851288117\H,0,-3.3607846246,2.8576039792,0.9428807321\H,0,-

1.8595153213,2.9731230558,3.8986477066\H,0,1.8869869633,0.6820740407,6
.0763212181\H,0,-1.2108643438,3.444337311,6.2378901095\N,0,-0.82784652
8,-1.8995296651,4.3692924216\N,0,-1.3113460493,-2.5416148242,6.3558291
274\N,0,-1.7977222946,-1.9416965778,5.249041509\C,0,-2.1596399784,-2.6
914687905,7.5444364112\H,0,-3.1848713896,-2.5711734225,7.182730554\H,0
,-2.0455280414,-3.715763082,7.914232539\C,0,-1.8284381452,-1.680621197
7,8.6286907515\C,0,-1.1847809541,-2.0818726799,9.8066695839\C,0,-2.173
0047397,-0.3295237667,8.4601926123\C,0,-0.8883527712,-1.14816588,10.80
65916736\H,0,-0.9283239382,-3.1299702905,9.9539048816\C,0,-1.881757433
9,0.6028413709,9.4591403191\H,0,-2.6819500456,-0.0170900079,7.54959154
76\C,0,-1.2365877093,0.1942206396,10.6342816544\H,0,-0.3990137306,-1.4
73083861,11.7215465463\H,0,-2.1659238948,1.6444636174,9.3311715253\H,0
,-1.022007978,0.9185819387,11.4164046346\C,0,0.2973599684,-2.464972160
1,4.905290371\C,0,1.5892913507,-2.5281308012,4.1446689856\H,0,2.258895
9625,-3.274044524,4.5913492714\N,0,1.5506604278,-0.0983331314,3.503206
959\C,0,2.3410223823,-1.1705088942,4.0996569697\H,0,1.3864900188,-2.86
10892981,3.1186264641\H,0,2.6545643469,-0.8920926513,5.1096634198\H,0,
3.2628902632,-1.2834663897,3.514943367\C,0,-0.0087609604,-2.8789142193
,6.1890717893\H,0,0.5647761207,-3.3804348512,6.9556472738\N,0,0.329300
7943,2.0591514551,6.2406250509\C,0,0.5859599421,2.376614734,7.64953702
37\O,0,-0.0657521612,3.2477091652,8.1636583761\C,0,1.6449805717,1.5664
230318,8.3557030273\H,0,1.3919027333,0.499366242,8.3445409081\H,0,2.63
2604271,1.7041135825,7.8991985567\H,0,1.6807256722,1.9130561168,9.3895
944726\\Version=AM64L-G03RevD.01\State=1-A\HF=-1405.2444763\MP2=-1410.
2979169\RMSD=8.649e-09\Thermal=0.\PG=C01 [X(C26H33N6O1)]\\@

404
see reference 9

404-Ac^{+}
see reference 9

405
see reference 9

405-Ac^{+}
see reference 9

406
see reference 9

406-Ac^{+}
see reference 9

407
1\1\GINC-AZAZEL\SP\RHF\6-31G(d)\C11H15N3\RAMAN\18-Jul-2011\0\\#P RHF/6
-31G(d) scf=tight int=finegrid SCRF=(PCM,Read,Solvent=Chloroform) geom
=check guess=read\\PCM of rtcapme14 b98/6-31G(d) structures on 4p 900
MB\\0,1\C,0,-1.0568865323,1.8141250692,-0.2781054635\C,0,-0.7604108446
,0.4459871739,-0.2784248701\C,0,0.5917016568,0.0627001811,-0.039985799
3\C,0,1.5412870115,1.0851917064,0.1623051467\C,0,1.1091390923,2.413092
266,0.0963442635\N,0,-0.1529731097,2.7938279419,-0.1119768999\N,0,0.94
88921333,-1.2720788095,-0.0157995246\C,0,2.3368324573,-1.6984508313,0.
1256328928\C,0,3.3200129724,-0.6037721622,-0.3036561238\C,0,-1.4366283
955,-1.7971017972,0.2163215232\C,0,-0.0509377478,-2.3127739847,-0.1734
652979\H,0,1.8391751932,3.2142017632,0.2275202445\H,0,-2.0810632156,2.
1464406136,-0.4332250663\H,0,2.4835079007,-2.5943068896,-0.4948700774\
H,0,2.5380745061,-1.9960828978,1.1703425862\H,0,0.2130679256,-3.161356

4298,0.4759640718\H,0,-0.0747719506,-2.6829708538,-1.211855156\H,0,4.3
42118627,-0.9318067882,-0.0772590692\H,0,-2.1867051083,-2.5474520438,-
0.059001998\H,0,-1.487990688,-1.6537223708,1.3120232213\H,0,3.25049953
63,-0.4588513909,-1.390045444\N,0,-1.7160231027,-0.5648493936,-0.51112
2792\C,0,-3.115111422,-0.175617111,-0.5558821721\H,0,-3.2747569086,0.5
577017337,-1.3539829398\H,0,-3.7175052347,-1.0605703201,-0.7874877011\
C,0,2.987580609,0.7147781071,0.4125341902\H,0,3.6483591549,1.520623123
3,0.0679949685\H,0,3.1750564925,0.5963685461,1.4918776124\H,0,-3.48075
57184,0.2594902785,0.3924086841\\Version=AM64L-G03RevD.01\State=1-A\HF
=-588.6235353\RMSD=7.070e-09\Thermal=0.\Dipole=0.3128376,-2.1353997,0.
2504223\PG=C01 [X(C11H15N3)]\\@

407-Ac⁺

1\1\GINC-NAUTILUS\SP\RHF\6-31G(d)\C13H18N3O1(1+)\RAMAN\19-Jul-2011\0\\
#P RHF/6-31G(d) scf=tight int=finegrid SCRF=(PCM,Read,Solvent=Chlorofo
rm) geom=check guess=read\\PCM of rtcapme14.ac2 b98/6-31G(d) structure
s on 2p 512 MB\\1,1\C,0,-0.0323770139,0.0527963374,0.0099664609\C,0,-0
.0159055012,0.0614280838,1.3893500341\C,0,1.2696099643,0.0279619088,2.
0604510626\C,0,2.4526005748,-0.0051095305,1.2572662895\C,0,2.335569150
9,-0.053890558,-0.1098234756\N,0,1.1221772401,-0.0304487905,-0.7317092
582\N,0,1.3338513721,0.0181580525,3.4022112518\C,0,2.6076993178,-0.098
1025616,4.1373878948\C,0,3.7058630203,-0.7349244594,3.2857054009\C,0,-
1.0307495005,0.7143075548,3.4635191743\C,0,0.1194588246,0.0631564469,4
.2243862608\H,0,3.2109268812,-0.1092110558,-0.7438953596\H,0,-0.937050
8622,0.0899563926,-0.5806082649\H,0,2.4110644932,-0.7068210184,5.02741
59162\H,0,2.9038990677,0.9038911566,4.4806986881\H,0,0.3441194615,0.64
35759979,5.127220614\H,0,-0.1484464759,-0.9572000083,4.5297135809\H,0,
4.6548462999,-0.6755393339,3.8289058842\H,0,-1.9570941621,0.5607844203
,4.0245828394\H,0,-0.8652869911,1.8007156123,3.3743887221\H,0,3.481898
1909,-1.797577192,3.1279414471\N,0,-1.1669839251,0.0782028861,2.155073
1612\C,0,-2.459234328,0.1892407439,1.4875534823\H,0,-2.5800644508,-0.6
306638504,0.7711086789\H,0,-3.2482551387,0.0964643191,2.2379720281\C,0
,3.8077794013,-0.0134373936,1.9337082764\H,0,4.5509107481,-0.496132666
3,1.2889517654\H,0,4.1472004357,1.0208376226,2.0925840922\H,0,-2.58501
73558,1.1482206673,0.959352656\C,0,0.9711191306,-0.0751181897,-2.19520
73073\O,0,-0.1416357726,-0.0459542685,-2.6476085326\C,0,2.2342382782,-
0.1531253412,-3.0183515012\H,0,2.8106412818,-1.0571938324,-2.788526462
\H,0,1.9321651643,-0.1821193291,-4.0666680637\H,0,2.8756208306,0.72161
00178,-2.8569401684\\Version=AM64L-G03RevD.01\State=1-A\HF=-740.849051
6\RMSD=2.875e-09\Thermal=0.\Dipole=1.4138666,0.155854,1.053636\PG=C01
[X(C13H18N3O1)]\\@

408

1\1\GINC-GOLEM\SP\RHF\6-31G(d)\C12H17N3\RAMAN\05-Jul-2011\0\\#P RHF/6-
31G(d) scf=tight int=finegrid SCRF=(PCM,Read,Solvent=Chloroform) geom=
check guess=read\\PCM of rtcapet2 b98/6-31G(d) structures on 4p 900 MB
\\0,1\N,0,0.0396874564,1.5831121674,-0.0228410965\N,0,0.0019646878,-2.
6384204891,-0.1312161181\C,0,1.2253189391,2.2569324785,-0.5199811841\C
,0,2.4712515552,1.5387425036,-0.0015828335\C,0,1.2351199939,-0.5425063
419,-0.2432830487\C,0,1.1436282301,-1.9437219377,-0.245335725\C,0,-1.1
222301766,-1.934288415,0.0005443953\C,0,-1.1845285217,-0.5361773072,0.
0201499436\C,0,-2.3172391072,1.617304106,0.670567557\C,0,-1.2166094952
,2.3134807232,-0.1357421576\C,0,0.0177091718,0.1884101328,-0.091209315
6\H,0,3.3670654017,2.0108779497,-0.425010121\H,0,-1.5307369083,2.40883
93555,-1.1928792069\H,0,-1.0452366487,3.3298948315,0.2433606556\H,0,-2
.0446895846,-2.5094411726,0.0990908477\H,0,2.0429703752,-2.5470033012,
-0.3453146319\H,0,1.2516137471,2.2925243286,-1.6237758536\H,0,1.215782
5233,3.2910095883,-0.1509737449\H,0,2.5194134736,1.6495056192,1.096074

6867\H,0,-2.0240415121,1.5984750086,1.7286005134\H,0,-3.2489227054,2.1
914815599,0.593698078\N,0,2.443817242,0.148351326,-0.4260053905\C,0,3.
7168500256,-0.5628992476,-0.3845428026\H,0,3.651929792,-1.4322147832,-
1.0498615393\H,0,4.466232419,0.1055599087,-0.829727047\C,0,4.183923691
6,-0.9976911001,1.0172692998\H,0,5.1410808457,-1.5304122478,0.95081847
33\H,0,4.3266518052,-0.1297144098,1.6727883927\H,0,3.4537567995,-1.663
3195414,1.4910158008\C,0,-2.51327749,0.1856021302,0.1515776173\H,0,-3.
0171740848,0.2217931723,-0.8271244391\H,0,-3.1795588312,-0.3773600253,
0.8185017849\\Version=AM64L-G03RevD.01\State=1-A\HF=-627.6591355\RMSD=
6.211e-09\Thermal=0.\Dipole=0.310909,2.0282089,0.0256046\PG=C01 [X(C12
H17N3)]\\@

408-Ac$^+$

1\1\GINC-AZAZEL\SP\RHF\6-31G(d)\C14H20N3O1(1+)\RAMAN\07-Jul-2011\0\\#P
RHF/6-31G(d) scf=tight int=finegrid SCRF=(PCM,Read,Solvent=Chloroform
) geom=check guess=read\\PCM of rtcapet14.ac2 b98/6-31G(d) structures
on 2p 512 MB\\1,1\C,0,-0.0253852733,0.0502143408,0.013086183\C,0,-0.00
97057291,0.0507602157,1.393884258\C,0,1.2813156456,0.0085764356,2.0586
664775\C,0,2.4616272315,0.0067175777,1.2513702424\C,0,2.3428790651,-0.
0263768799,-0.1162322692\N,0,1.1271684862,-0.014622128,-0.7326659966\N
,0,1.3530117763,-0.0429425497,3.4003164099\C,0,2.6316170526,-0.1658896
523,4.1263295039\C,0,3.7353629886,-0.7607182845,3.2522348974\C,0,-1.01
75997462,0.6133295276,3.5045091226\C,0,0.1424888936,-0.0614509746,4.22
82918691\H,0,3.2171447378,-0.0615642413,-0.7529597086\H,0,-0.930291945
6,0.0844298621,-0.5763541474\H,0,2.4468094757,-0.8050051037,4.99731849
2\H,0,2.9161761714,0.8284880516,4.5002960017\H,0,0.3619128636,0.473044
5265,5.1602943553\H,0,-0.1094270236,-1.1006391027,4.4795530273\H,0,4.6
857989705,-0.7033000518,3.7932235054\H,0,-1.9421167399,0.4214568405,4.
0577124246\H,0,-0.8615443162,1.7034452485,3.4764453628\H,0,3.526610616
8,-1.8214218041,3.0634308913\N,0,-1.1540985345,0.0482456878,2.16539555
82\C,0,-2.4864212276,0.0944237233,1.5488161681\H,0,-2.496870461,-0.619
787744,0.7170680494\H,0,-3.1864006678,-0.2990452968,2.295475561\C,0,-2
.9432452598,1.4877736334,1.0869314778\H,0,-2.2644783325,1.9199655187,0
.3425999272\H,0,-3.9389242692,1.4176353115,0.6344418023\H,0,-3.0096506
535,2.1843005867,1.9309074235\C,0,3.8194648294,0.0024096071,1.92229006
57\H,0,4.5683541619,-0.4474106206,1.2605932317\H,0,4.1413457054,1.0373
04055,2.1109587774\C,0,0.9717481234,-0.0477137031,-2.1956200853\O,0,-0
.1424876137,-0.0245474619,-2.6452897208\C,0,2.2325696138,-0.1077188333
,-3.0240735712\H,0,2.8184480605,-1.007910174,-2.8033246897\H,0,1.92661
43707,-0.1315653804,-4.0713825107\H,0,2.8663399119,0.7717681575,-2.858
5674359\\Version=AM64L-G03RevD.01\State=1-A\HF=-779.8829238\RMSD=2.974
e-09\Thermal=0.\Dipole=1.7644378,-0.0453075,1.0616283\PG=C01 [X(C14H20
N3O1)]\\@

409

1\1\GINC-NODE11\SP\RMP2-FC\6-31+G(2d,p)\C19H25N5O1\ZIP01\19-Feb-2010\0
\\#P MP2/6-31+G(2d,p) scf=(direct,tight) int=finegrid geom=check guess
=read\\sp of cat81ur6_63 MP2-5/6-31+G(2d,p)\\0,1\C,0,0.0444084181,0.87
92248811,0.3284684939\C,0,0.168299805,0.6611590392,3.024362357\C,0,1.3
070040971,0.725179734,2.2180665102\H,0,0.0487288114,0.9538833057,-0.75
74111051\H,0,0.2895757149,0.5975298511,4.1002858719\H,0,2.2963279456,0
.6894724579,2.6736911032\C,0,-1.1131719045,0.7129137554,2.4511400689\C
,0,-1.1792928541,0.8195242443,1.019456131\N,0,-2.2843571895,0.64885777
54,3.1924506754\N,0,-2.4187978556,0.883441481,0.3839703119\N,0,1.26537
02791,0.8442422554,0.8896199368\C,0,-2.2534778518,0.6515248354,4.65279
85835\H,0,-3.2144808045,0.238144113,4.9875510869\H,0,-1.485300127,-0.0
579384801,4.9825747627\C,0,-2.0313412686,2.0306822959,5.299246074\H,0,
-2.824756911,2.7321991813,5.016027197\H,0,-2.035761485,1.9381134139,6.

392958903\H,0,-1.0763488743,2.4722152906,4.9941659727\C,0,-3.488411547
5,1.1876192472,2.5675511853\H,0,-3.4772279033,2.2868242943,2.516778870
3\H,0,-4.3530152969,0.876970505,3.1669718018\C,0,-3.6244015329,0.62263
98997,1.1546294241\H,0,-4.4692502365,1.1152368894,0.6593704629\H,0,-3.
8399191326,-0.4579096023,1.2055349322\C,0,-2.5672372678,1.1478508513,-
1.034862718\H,0,-3.4556403411,0.6113554787,-1.3954162301\H,0,-1.721855
6008,0.7247315371,-1.5914341301\C,0,-2.7274287207,2.6590767057,-1.3845
53563\H,0,-3.6835449222,3.0121446305,-0.9850169526\C,0,-2.692980525,2.
8767286144,-2.9026428154\H,0,-2.8703224746,3.9301017795,-3.1429250971\
H,0,-1.7193517587,2.5888634717,-3.3234804694\H,0,-3.4620991952,2.27197
15377,-3.3996283173\H,0,-0.7558019109,3.2348628612,-0.9146351196\N,0,-
1.7194054274,3.4763291449,-0.7162431209\C,0,-1.9918385904,4.019167588,
0.5292030597\O,0,-3.1340435377,4.2403468997,0.9202651921\H,0,0.0018706
648,3.8458047416,0.9717370251\N,0,-0.8469801621,4.3188374538,1.2570971
142\C,0,-0.4959804213,6.0167520795,5.1092170474\C,0,0.5418707318,5.265
8092022,4.5478762709\C,0,0.3995887936,4.7015265479,3.2798471911\C,0,-0
.7872211454,4.8855573567,2.5463597091\C,0,-1.8309162934,5.6452863276,3
.1041471133\C,0,-1.6742413505,6.1966886272,4.3786397081\H,0,-0.3868244
667,6.4552052941,6.0980750714\H,0,1.468492322,5.1131442555,5.097052129
\H,0,1.2061934576,4.1045007418,2.8562652658\H,0,-2.74731788,5.78638497
56,2.5459435784\H,0,-2.4896638442,6.7814634092,4.7995195168\\Version=A
M64L-G03RevD.01\State=1-A\HF=-1081.266008\MP2=-1085.1214935\RMSD=7.520
e-09\Thermal=0.\PG=C01 [X(C19H25N5O1)]\\@

409-Ac$^+$

1\1\GINC-CALYPSO\SP\RHF\6-31G(d)\C21H28N5O2(1+)\EVGENY\21-Feb-2010\0\\
#P RHF/6-31G(d) scf=tight int=finegrid SCRF=(PCM,Read,Solvent=Chlorofo
rm) geom=check guess=read\\PCM of cat81ur6_143.ac2 b98/6-31G(d) struct
ures on 4p 900 MB\\1,1\C,0,0.4227395239,0.1556347681,1.452681361\C,0,0
.9922691141,-2.3754000936,0.5523938785\C,0,-0.1074338678,-2.1516189912
,1.3369465796\H,0,0.0804382767,1.1091180065,1.8225673903\H,0,1.1876410
591,-3.3932198055,0.2417570574\H,0,-0.7776784151,-2.9514116553,1.61906
73405\C,0,1.8543063944,-1.3147826411,0.1547005563\C,0,1.5336451948,0.0
207871829,0.6455650823\N,0,2.9237290884,-1.5180466102,-0.6357006431\N,
0,2.3519637357,1.0759234771,0.2993597938\N,0,-0.3943591211,-0.89919302
2,1.7867583749\C,0,3.3053090768,-2.8530098471,-1.1317725256\H,0,2.3975
459649,-3.3962968408,-1.4130303199\H,0,3.8637218444,-2.6907480482,-2.0
59309482\C,0,4.1543497079,-3.6540305796,-0.1345724265\H,0,3.6147336195
,-3.8354485713,0.8019705139\H,0,4.4168855738,-4.623895897,-0.571816421
3\H,0,5.0855517053,-3.1280904566,0.1037702141\C,0,3.8592243066,-0.4180
253425,-0.9010947717\H,0,4.3329625665,-0.6030231883,-1.8705761764\H,0,
4.6434256256,-0.399105269,-0.1324639125\C,0,3.1124165354,0.9098594685,
-0.9374862036\H,0,2.4485791792,0.9563274407,-1.8116269514\H,0,3.842100
6037,1.7233501336,-1.0053068317\C,0,2.1096518747,2.456366582,0.7260101
195\H,0,3.0919740191,2.9475136878,0.7368274166\H,0,1.7522406723,2.4535
657613,1.762687927\C,0,1.1536484249,3.3023658529,-0.1635750556\H,0,1.4
546444485,3.1835584476,-1.2111560699\C,0,1.2453136541,4.7842128058,0.2
277287818\H,0,0.9527333864,4.94015711,1.2754295004\H,0,2.2688617452,5.
1555736406,0.1093347797\H,0,0.590279689,5.3889795021,-0.408671693\H,0,
-0.8637546378,3.4263311699,0.4875615745\N,0,-0.2335952418,2.8538883466
,-0.0590807291\C,0,-0.7442278878,1.8584450519,-0.8723421946\O,0,-0.061
5828818,1.2847557013,-1.7158668201\H,0,-2.4747110465,2.0137231577,0.21
1677996\N,0,-2.0641425831,1.5506291163,-0.5893103826\C,0,-4.3796616093
,-1.7613896025,-1.8263255523\C,0,-3.2529780945,-1.392405337,-2.5672854
419\C,0,-2.4620195338,-0.3014815783,-2.194663845\C,0,-2.8022391578,0.4
337364556,-1.0468048721\C,0,-3.9377930695,0.0685988296,-0.2994964897\C
,0,-4.7212718554,-1.0187075734,-0.6914926896\H,0,-4.9914779421,-2.6042
732549,-2.1365945917\H,0,-2.9861436554,-1.9512392118,-3.4614518099\H,0

,-1.5960788356,-0.0166168548,-2.7779072711\H,0,-4.2144863377,0.6472226
404,0.5817071759\H,0,-5.6054991246,-1.2754831793,-0.1124545099\C,0,-1.
6253824234,-0.5814237677,2.5142283985\O,0,-1.8559489131,0.5745375958,2
.765257446\C,0,-2.5332059622,-1.7315140217,2.8751152183\H,0,-2.9802958
591,-2.1635102667,1.9705290617\H,0,-2.0083562504,-2.5196791719,3.42627
29406\H,0,-3.3307167398,-1.3274402862,3.5011478008\\Version=AM64L-G03R
evD.01\State=1-A\HF=-1233.4001814\RMSD=1.506e-09\Thermal=0.\Dipole=1.8
500817,-1.6174838,1.6103016\PG=C01 [X(C21H28N5O2)]\\@

410

1\1\GINC-AZAZEL\SP\RHF\6-31G(d)\C17H19N3\RAMAN\19-Jul-2011\0\\#P RHF/6
-31G(d) scf=tight int=finegrid SCRF=(PCM,Read,Solvent=Chloroform) geom
=check guess=read\\PCM of rtcapbn5 b98/6-31G(d) structures on 4p 900 M
B\\0,1\N,0,2.4030517794,-1.1320390627,0.0663092698\N,0,0.5868773626,2.
6453837852,-0.4501444789\C,0,3.7046885982,-1.2175148332,0.716231331\C,
0,4.6187331151,-0.0873030922,0.2319635581\C,0,2.524840139,1.3113178722
,0.1175869797\C,0,1.8683173335,2.5275822075,-0.1013775745\C,0,-0.10594
39331,1.5074012345,-0.6079571977\C,0,0.4261252744,0.2200930627,-0.4415
437922\C,0,0.3931796969,-2.1815064053,-0.8147070421\C,0,1.5288875085,-
2.2821688338,0.2025247257\C,0,1.7986405798,0.1188480165,-0.0688849848\
H,0,5.6016066539,-0.1773212039,0.7109698899\H,0,1.1070741006,-2.367145
0762,1.2204383595\H,0,2.1159035144,-3.1891665845,0.0070442874\H,0,-1.1
552756344,1.6299758442,-0.8639462086\H,0,2.4235825826,3.4595877971,0.0
200505407\H,0,3.6062978477,-1.1748512034,1.8178407315\H,0,4.1369433404
,-2.1957582033,0.4673020588\H,0,4.7653479612,-0.190923144,-0.851289504
2\H,0,0.8140462068,-2.246081611,-1.8342712018\H,0,-0.2908770237,-3.027
4628932,-0.6770606421\N,0,-0.349178738,-0.9463319278,-0.5988438854\C,0
,-1.6390787783,-0.8447939466,-1.2712850984\H,0,-1.8844938656,-1.850138
2589,-1.6396066133\H,0,-1.5710137611,-0.2000686762,-2.1645445752\C,0,3
.979189085,1.2721476007,0.5503940704\H,0,4.5366941053,2.0822664782,0.0
618977522\H,0,4.0500357514,1.460078277,1.6332802267\C,0,-2.7882570701,
-0.3660617239,-0.3864038264\C,0,-3.9080222766,0.240638023,-0.974083008
8\C,0,-2.7781828823,-0.5694562422,0.9994937042\C,0,-5.0010295541,0.628
9178386,-0.1942776251\H,0,-3.923483504,0.4142871656,-2.0497354222\C,0,
-3.8679817953,-0.1768940229,1.7821499684\H,0,-1.9030702138,-1.02214958
81,1.459186983\C,0,-4.9842287497,0.4205126131,1.188445967\H,0,-5.86030
07699,1.1014791704,-0.6654113283\H,0,-3.8429861317,-0.3345680213,2.858
4614599\H,0,-5.830541734,0.728313955,1.7985259404\\Version=AM64L-G03Re
vD.01\State=1-A\HF=-818.1692826\RMSD=1.809e-09\Thermal=0.\Dipole=0.763
5159,-1.9799971,0.1391064\PG=C01 [X(C17H19N3)]\\@

410-Ac$^+$

1\1\GINC-NAUTILUS\SP\RHF\6-31G(d)\C19H22N3O1(1+)\RAMAN\23-Jul-2011\0\\
#P RHF/6-31G(d) scf=tight int=finegrid SCRF=(PCM,Read,Solvent=Chlorofo
rm) geom=check guess=read\\PCM of rtcapbn16.ac2 b98/6-31G(d) structure
s on 2p 512 MB\\1,1\C,0,-0.1238156739,0.0138382108,0.068282879\C,0,-0.
0696438178,0.0423241421,1.4478022654\C,0,1.2343098622,0.0190982346,2.0
802790793\C,0,2.3944916905,-0.0036377989,1.243357744\C,0,2.2409819481,
-0.068428991,-0.1173298928\N,0,1.0093760433,-0.0706141039,-0.703521893
7\N,0,1.3411093152,0.0106936102,3.4203440085\C,0,2.6363479555,-0.10273
9156,4.1179892343\C,0,3.7161926935,-0.7214612561,3.2302849674\C,0,-1.0
146550247,0.713134834,3.5477865136\C,0,0.1528369625,0.0582024145,4.276
5220763\H,0,3.0760410105,-0.1234034354,-0.8060307221\H,0,-1.0654361722
,0.0037126581,-0.4568700503\H,0,2.4691598561,-0.7206077454,5.008078632
6\H,0,2.9343203216,0.8985371089,4.4620686239\H,0,0.4031692952,0.639452
1061,5.1722246811\H,0,-0.1104379857,-0.9605310853,4.592359312\H,0,4.67
98776729,-0.654009899,3.746076484\H,0,-1.9222508362,0.5768002253,4.142
7122466\H,0,-0.8378040841,1.7963514363,3.4424562857\H,0,3.5007258187,-

1.7858284528,3.0720117402\N,0,-1.2055664558,0.0658518896,2.2487787762\
C,0,-2.5236036904,0.2305560647,1.6224111338\H,0,-3.2343309039,0.380687
6317,2.4439343355\H,0,-2.5547916058,1.1508066496,1.0125967059\C,0,3.76
77880886,0.0096959592,1.8808000158\H,0,4.4991860275,-0.4547181877,1.21
04439318\H,0,4.0922737588,1.0491219657,2.0366636609\C,0,-2.9717739933,
-0.960457821,0.7834480954\C,0,-2.7116572548,-2.2770703336,1.1905876599
\C,0,-3.7010684354,-0.7411238342,-0.3954078692\C,0,-3.1700179057,-3.35
50481179,0.4286063435\H,0,-2.1474272506,-2.4551714388,2.1032619302\C,0
,-4.1651060821,-1.8206510125,-1.1556235044\H,0,-3.9218530532,0.2773864
088,-0.7142855441\C,0,-3.8972193346,-3.1303391585,-0.7458820718\H,0,-2
.9643338486,-4.3718334995,0.7543501632\H,0,-4.7375520661,-1.6371428696
,-2.0616369765\H,0,-4.2564834231,-3.9707227193,-1.3343256038\C,0,0.958
3186475,-0.173770527,-2.1727022705\O,0,1.9990620652,-0.2321464268,-2.7
702758841\C,0,-0.4081002638,-0.1969950904,-2.8121371693\H,0,-0.9481053
77,0.7397417578,-2.6262995999\H,0,-0.2581053089,-0.3118208223,-3.88704
0638\H,0,-1.0207428146,-1.0267864602,-2.4395145803\\Version=AM64L-G03R
evD.01\State=1-A\HF=-970.3943172\RMSD=2.303e-09\Thermal=0.\Dipole=0.78
1003,1.0193124,1.7651652\PG=C01 [X(C19H22N3O1)]\\@

411-Ac⁺

1\1\GINC-NODE22\SP\RMP2-FC\6-31+G(2d,p)\C23H24N5O1(1+)\ZIP01\29-Jan-20
11\0\\#P MP2/6-31+G(2d,p) scf=(direct,tight) int=finegrid geom=check g
uess=read\\sp of diaza4ci_1.ac2 MP2-5/6-31+G(2d,p)\\1,1\C,0,0.02221855
46,-0.0590336109,-0.020316358\C,0,0.0218007478,-0.020758612,2.73395774
03\C,0,1.2019817108,-0.0635613443,2.0360621397\H,0,0.1485668205,-0.067
3139679,-1.0922984263\H,0,0.0798172946,0.0148443723,3.8131035649\H,0,2
.1567649014,-0.0759089405,2.5445273722\C,0,-1.2035257489,-0.056312609,
0.6160894421\C,0,-1.2327479916,-0.0063651961,2.0597159983\C,0,-3.62996
10241,0.238679787,1.9920884922\C,0,-3.6114501738,0.144880554,0.5788972
354\C,0,-4.7952841665,0.3476888109,-0.1313839891\C,0,-5.9990897292,0.6
271926642,0.5400157853\C,0,-6.0181781155,0.6902342554,1.9353641924\C,0
,-4.8312360201,0.5288439183,2.6488644317\H,0,-4.8058686348,0.299187863
,-1.2124744338\H,0,-6.9548550093,0.8701718684,2.4538100762\H,0,-4.8655
075679,0.6218388074,3.7278701847\N,0,-2.3999647241,-0.164209084,-0.074
5271967\N,0,-2.4139287708,0.0274250306,2.7109033222\N,0,1.2154502929,-
0.0772667762,0.6794987837\C,0,-2.3843504028,-0.4490963482,-1.518275676
8\H,0,-1.5609972268,-1.1498367736,-1.6989671457\H,0,-3.2993985921,-1.0
023768745,-1.7483236061\C,0,-2.2540167615,0.794836959,-2.4113307444\H,
0,-2.2382250334,0.4879963737,-3.4633972007\H,0,-3.0931708367,1.4847539
304,-2.2692553517\H,0,-1.3299423586,1.3476753493,-2.2074431473\C,0,-2.
4586154653,-0.082648186,4.187210353\H,0,-1.6700690648,-0.7771475839,4.
4898025982\H,0,-3.3987958919,-0.5780748246,4.4384803469\C,0,-2.3253619
169,1.2645019049,4.9084761749\H,0,-3.1261519975,1.9538246359,4.6214424
647\H,0,-2.3827455509,1.1042655848,5.9909653783\H,0,-1.3686234542,1.75
03801173,4.685758521\N,0,-7.2609569804,0.6284694244,-0.1368072987\N,0,
-7.5076971294,1.2684566593,-1.1821239138\C,0,-5.1507593924,4.259063682
6,-3.0058983884\C,0,-5.2807395159,4.2621449456,-1.611096133\C,0,-6.011
8676462,3.2624962881,-0.967584902\C,0,-6.6358695962,2.2613396936,-1.73
34022108\C,0,-6.5579377708,2.292296528,-3.1356847574\C,0,-5.7884900875
,3.2709427092,-3.7661719724\H,0,-4.579279889,5.0409766853,-3.499737066
2\H,0,-4.8196069422,5.0525625482,-1.0237198964\H,0,-6.1287958712,3.276
1387588,0.1122401416\H,0,-7.1011373509,1.5446755997,-3.7078414924\H,0,
-5.7123289387,3.2819912394,-4.8504983393\C,0,2.452588548,-0.1039009664
,-0.1120297796\O,0,2.3532969083,-0.1065450353,-1.3103241995\C,0,3.7580
118401,-0.1247131636,0.6467494956\H,0,3.8425238383,-1.014125393,1.2826
89452\H,0,4.558667548,-0.143160113,-0.094677421\H,0,3.8757895504,0.767
2939101,1.2737528758\\Version=AM64L-G03RevD.01\State=1-A\HF=-1231.8887
598\MP2=-1236.2499948\RMSD=6.107e-09\Thermal=0.\PG=C01 [X(C23H24N5O1)]

\\@

412

1\1\GINC-CIP-F-08\SP\RHF\6-31G(d)\C21H21N5\C2253\31-Dec-2010\0\\#P hf/
6-31G(d) int=finegrid\\sp of diaza4tr_1 b98/6-31G(d) on 2p 900 MB\\0,1
\C\C,1,2.7385\C,2,1.402,1,57.\H,1,1.0787,2,175.59,3,4.55,0\H,2,1.0766,
1,175.19,3,352.44,0\H,3,1.08,2,120.3,1,180.25,0\C,1,1.4187,2,62.46,3,1
80.03,0\C,2,1.4118,1,62.18,3,179.88,0\C,8,2.3754,2,150.94,1,165.09,0\C
,9,1.4303,8,89.97,2,193.31,0\C,10,1.4178,9,118.97,8,172.44,0\C,11,1.40
7,10,121.66,9,0.39,0\C,12,1.4118,11,119.12,10,359.82,0\C,13,1.4051,12,
120.1,11,359.87,0\H,11,1.0774,10,121.13,9,180.96,0\H,13,1.0801,12,121.
24,11,180.23,0\H,14,1.0773,13,117.08,12,180.74,0\N,7,1.3678,1,123.68,2
,180.31,0\N,8,1.368,2,122.75,1,179.84,0\N,3,1.3417,2,123.48,1,0.1,0\C,
18,1.4693,7,119.3,1,345.42,0\H,21,1.0907,18,110.35,7,212.78,0\H,21,1.0
907,18,110.36,7,326.35,0\C,21,1.5372,18,112.46,7,89.48,0\H,24,1.0894,2
1,111.95,18,299.69,0\H,24,1.0895,21,111.82,18,59.98,0\H,24,1.09,21,110
.62,18,179.82,0\C,19,1.4696,8,119.54,2,16.24,0\H,28,1.0903,19,110.13,8
,145.42,0\H,28,1.0905,19,110.04,8,32.02,0\C,28,1.539,19,112.87,8,268.8
5,0\H,31,1.0896,28,111.82,19,60.29,0\H,31,1.09,28,110.63,19,180.1,0\H,
31,1.0894,28,111.99,19,300.04,0\N,12,1.4162,11,117.45,10,180.03,0\N,35
,1.2472,12,117.85,11,180.11,0\C,36,4.2443,35,119.84,12,180.03,0\C,37,1
.4078,36,61.04,35,359.97,0\C,38,1.409,37,120.13,36,359.97,0\C,36,1.416
,35,117.8,12,180.03,0\C,40,1.4103,36,117.4,35,179.97,0\C,37,1.4077,36,
58.87,35,179.97,0\H,37,1.0807,36,178.92,35,179.28,0\H,38,1.0803,37,119
.97,36,179.97,0\H,39,1.0803,38,118.63,37,179.97,0\H,41,1.0805,40,119.9
1,36,359.97,0\H,42,1.0803,37,120.,36,180.03,0\\Version=IA32L-G03RevE.0
1\State=1-A\HF=-1079.6331202\RMSD=6.592e-06\Thermal=0.\Dipole=-0.73270
44,0.2290865,0.5851562\PG=C01 [X(C21H21N5)]\\@

412-Ac⁺

1\1\GINC-NODE24\SP\RMP2-FC\6-31+G(2d,p)\C23H24N5O1(1+)\ZIP01\03-Jan-20
11\0\\#P MP2/6-31+G(2d,p) scf=(direct,tight) int=finegrid geom=check g
uess=read\\sp of diaza4tr_1.ac1 MP2-5/6-31+G(2d,p)\\1,1\C,0,-0.0320402
559,-0.0199524023,-0.0177298008\C,0,-0.0084749101,0.1372302262,2.73316
35033\C,0,1.1625158599,0.1734339456,2.0217725403\H,0,0.0420656472,-0.0
891338578,-1.0902961618\H,0,0.0585579878,0.1784431979,3.8117489506\H,0
,2.1394754197,0.2542061531,2.483539322\C,0,-1.2527721405,-0.0234695826
,0.6325010129\C,0,-1.2666954589,0.0338995878,2.0761638666\C,0,-3.65543
8822,-0.2942680899,2.0577779137\C,0,-3.6516099304,-0.3203638331,0.6443
049003\C,0,-4.8339697545,-0.6322888172,-0.0279651342\C,0,-6.0199754232
,-0.9027241464,0.673416746\C,0,-6.0175956788,-0.8802833848,2.074350533
7\C,0,-4.8391154575,-0.5853629484,2.7525830574\H,0,-4.8814588513,-0.68
31844692,-1.1093175024\H,0,-6.9320202964,-1.102100885,2.6132002506\H,0
,-4.8542918022,-0.5937572494,3.8363112344\N,0,-2.4595293868,-0.0104066
338,-0.0471552052\N,0,-2.4418121578,0.016003212,2.7412296024\N,0,1.166
2123421,0.0953314202,0.666776952\C,0,-2.4821083074,0.2120986871,-1.498
8879726\H,0,-3.4314833888,0.702089527,-1.7344403285\H,0,-1.7008667431,
0.9494135898,-1.7233774728\C,0,-2.3037593765,-1.0566245272,-2.34729076
87\H,0,-1.3541739274,-1.5640325251,-2.1397756802\H,0,-3.1067738334,-1.
7772488115,-2.1603492366\H,0,-2.3230851247,-0.7929846088,-3.4110166644
\C,0,-2.4736152134,0.2453246179,4.2046750923\H,0,-3.4264775113,0.73085
12603,4.4261127494\H,0,-1.7033246406,0.9856555933,4.4371866203\C,0,-2.
290669296,-1.0324634318,5.033504511\H,0,-1.3199385274,-1.5040117469,4.
8434293864\H,0,-2.345751134,-0.7838855714,6.0993051041\H,0,-3.06998305
28,-1.7687939121,4.8122387736\N,0,-7.1414441988,-1.1989651368,-0.14217
54224\N,0,-8.1977088333,-1.4274590975,0.5169210002\C,0,-11.720507327,-
2.3589569685,-1.5660911337\C,0,-10.5535277534,-2.1117756808,-2.3084875
878\C,0,-9.3633087352,-1.7986414673,-1.6621387361\C,0,-9.3378264316,-1

.7318251092,-0.2531542561\C,0,-10.5067895233,-1.9795311343,0.487773475
3\C,0,-11.6967990329,-2.2926223432,-0.1692162626\H,0,-12.6462522332,-2
.6029136713,-2.0815523897\H,0,-10.5822342229,-2.1658419006,-3.39400587
13\H,0,-8.4510198934,-1.6041989151,-2.2169485967\H,0,-10.4546785265,-1
.920135917,1.5715653527\H,0,-12.6007006374,-2.4840179862,0.4029222183\
C,0,2.4738855096,0.1311606916,-0.0073379637\O,0,3.4584287824,0.2287247
48,0.6752819567\C,0,2.4711394849,0.040635072,-1.5129367168\H,0,1.91585
2709,0.8721378504,-1.9639340696\H,0,3.5106936926,0.0867013551,-1.84187
69662\H,0,2.0289623867,-0.9023470042,-1.8573080523\\Version=AM64L-G03R
evD.01\State=1-A\HF=-1231.9170455\MP2=-1236.267209\RMSD=6.513e-09\Ther
mal=0.\PG=C01 [X(C23H24N5O1)]\\@

413

1\1\GINC-CIP-F-09\SP\RHF\6-31G(d)\C33H26N6\C2253\22-Aug-2010\0\\#P RHF
/6-31G(d) scf=tight int=finegrid SCRF=(PCM,Read,Solvent=Chloroform) ge
om=check guess=read\\PCM of diaza2cn_ci_7 b98/6-31G(d) structures on 4
p 900 MB\\0,1\C,0,-4.2449263336,2.676815489,-0.9892930467\C,0,-3.29560
41349,1.6378563792,-0.9541173825\C,0,-2.0413396719,1.9225518196,-0.339
1152014\C,0,-1.8348520428,3.2279313828,0.12694143\C,0,-2.8521047505,4.
1773496169,0.008654194\H,0,-5.2272544317,2.4903732421,-1.416483167\H,0
,-0.8895934373,3.5068595175,0.579792429\H,0,-2.6901769566,5.1914687712
,0.3721349792\N,0,-1.0809503306,0.9010916064,-0.1999487209\N,0,-4.0452
449662,3.9197808544,-0.531344625\N,0,-3.5415302973,0.3845463151,-1.518
7672527\C,0,-1.2176410859,-0.2519450665,-1.107840007\H,0,-1.0344037297
,0.0662358687,-2.1477199108\C,0,-2.706470684,-0.7376625675,-1.10646816
05\H,0,-2.7528714062,-1.4768047117,-1.918370467\H,0,-5.6289623787,0.06
01563567,-1.1711616214\H,0,-4.8625600197,-0.9340032067,-2.4334958259\C
,0,2.8232941549,1.8903296944,1.1161690427\C,0,1.7684068416,1.786146607
2,2.0366865424\C,0,0.4922060745,1.4388287243,1.597946127\C,0,0.2357752
842,1.2654701242,0.2295658868\C,0,1.2747645819,1.4620794019,-0.6940353
335\C,0,2.5648168591,1.7558141826,-0.2584006712\H,0,1.9735186792,1.945
0069548,3.0921074243\H,0,-0.320151268,1.3043330886,2.3074821753\H,0,1.
0734411505,1.3729349231,-1.758288788\H,0,3.3633978576,1.8966605689,-0.
9806805153\N,0,4.094859105,2.2801326939,1.6500153032\N,0,5.1787771292,
1.7727404871,1.2803708468\C,0,5.7447879508,-1.6378322058,-1.1517833939
\C,0,6.4557988873,-0.4469763759,-1.3952661324\C,0,6.2365728902,0.66656
64309,-0.5900918699\C,0,5.2709290237,0.6219197669,0.4290639791\C,0,4.5
828579906,-0.5758334341,0.6937002183\C,0,4.8211227841,-1.6989905051,-0
.0903995836\H,0,7.1811522109,-0.4082948161,-2.2028409425\H,0,6.7970448
805,1.5845758676,-0.7447313849\H,0,3.8618240156,-0.6242489441,1.503718
7729\H,0,4.2788177867,-2.620000203,0.1000802584\C,0,-4.8813820477,0.06
23598659,-1.9811839663\H,0,-5.2005963529,0.7768558631,-2.7493884517\C,
0,-4.0740341289,-2.8087240141,2.4659476603\C,0,-3.729572825,-3.5222554
28,1.3145448939\C,0,-3.2916817674,-2.8405692194,0.1757830033\C,0,-3.18
16031295,-1.4411929473,0.1662168936\C,0,-3.5404938154,-0.7326475779,1.
3262547984\C,0,-3.9812398145,-1.4129910854,2.4655927103\H,0,-4.4194671
518,-3.3341172823,3.353482417\H,0,-3.8047484941,-4.6073405509,1.299496
5586\H,0,-3.0219599675,-3.4035836927,-0.7165389615\H,0,-3.4907613356,0
.3522876224,1.339719953\H,0,-4.2579630388,-0.8486536552,3.3535865752\C
,0,1.4524765275,-3.582760054,-0.3340254042\C,0,1.2695065478,-3.0996989
175,-1.6330052239\C,0,0.4234231807,-2.0089894967,-1.8575426962\C,0,-0.
2536391873,-1.3890984074,-0.7966823686\C,0,-0.0583454312,-1.8790702004
,0.5052806223\C,0,0.790141189,-2.9651950999,0.7334920439\H,0,2.1017136
341,-4.4377173093,-0.1559694269\H,0,1.7863595749,-3.5661907186,-2.4685
200746\H,0,0.2879163464,-1.6343585461,-2.8713921975\H,0,-0.5780698804,
-1.4152267509,1.3383239595\H,0,0.9233143618,-3.3379101792,1.7468298734
\C,0,5.9580644045,-2.7920740987,-1.9787000153\N,0,6.1178319987,-3.7295
709289,-2.6507098611\\Version=IA32L-G03RevE.01\State=1-A\HF=-1591.4003

087\RMSD=5.638e-09\Thermal=0.\Dipole=-0.4463435,-0.9085002,0.2833728\P
G=C01 [X(C33H26N6)]\\@

413-Ac$^+$

1\1\GINC-PHOENIX\SP\RHF\6-31G(d)\C35H29N6O1(1+)\EVGENY\02-Oct-2010\0\\
#P RHF/6-31G(d) scf=tight int=finegrid SCRF=(PCM,Read,Solvent=Chlorofo
rm) geom=check guess=read\\PCM of diaza2cn_ci_7.ac1.sp b98/6-31G(d) st
ructures on 2p 900 MB\\1,1\C,0,4.6371,-0.87114,-0.93802\C,0,3.41371,-0
.2195,-0.9184\C,0,2.27553,-0.92815,-0.36061\C,0,2.49292,-2.25635,0.091
21\C,0,3.72846,-2.84688,0.0047\H,0,5.54879,-0.41551,-1.29675\H,0,1.669
08,-2.82982,0.49706\H,0,3.89494,-3.86505,0.32844\N,0,1.06702,-0.32573,
-0.28464\N,0,3.2571,1.05125,-1.4131\C,0,0.83475,0.93179,-1.06003\H,0,0
.75842,0.63827,-2.11622\C,0,2.09802,1.8441,-0.98323\H,0,1.93245,2.5883
9,-1.77158\H,0,5.1451,1.99187,-1.08003\H,0,4.0935,2.737,-2.30502\C,0,-
2.25968,-2.44291,1.26376\C,0,-1.30614,-1.89552,2.13666\C,0,-0.22691,-1
.1752,1.62692\C,0,-0.06453,-1.05424,0.24104\C,0,-0.97743,-1.65522,-0.6
3475\C,0,-2.07887,-2.3416,-0.12614\H,0,-1.43324,-2.02419,3.20817\H,0,0
.49317,-0.71226,2.29728\H,0,-0.83355,-1.58085,-1.70968\H,0,-2.7913,-2.
80413,-0.80269\N,0,-3.28381,-3.26167,1.84787\N,0,-4.4961,-3.16275,1.55
782\C,0,-6.29437,-0.14052,-0.77846\C,0,-6.62385,-1.49662,-0.96555\C,0,
-6.01372,-2.46956,-0.17905\C,0,-5.02909,-2.10366,0.75342\C,0,-4.72513,
-0.74786,0.96784\C,0,-5.36116,0.22841,0.20916\H,0,-7.36313,-1.77445,-1
.71089\H,0,-6.27775,-3.51823,-0.2839\H,0,-4.00534,-0.45941,1.72743\H,0
,-5.1293,1.27732,0.36524\C,0,4.42651,1.78436,-1.8869\H,0,4.92843,1.220
44,-2.68202\C,0,2.72071,4.09733,2.67853\C,0,2.17599,4.70755,1.54529\C,
0,1.98586,3.96692,0.37572\C,0,2.32978,2.60729,0.31996\C,0,2.8883,2.006
08,1.46091\C,0,3.07914,2.74619,2.63179\H,0,2.87451,4.67159,3.58855\H,0
,1.9033,5.7596,1.5678\H,0,1.55618,4.44889,-0.50073\H,0,3.19604,0.96356
,1.44461\H,0,3.51622,2.26856,3.50549\C,0,-2.79619,3.11744,-0.17281\C,0
,-2.49981,2.71722,-1.47877\C,0,-1.3349,1.98771,-1.73474\C,0,-0.45124,1
.6557,-0.69591\C,0,-0.7607,2.05748,0.61452\C,0,-1.92809,2.77967,0.8722
2\H,0,-3.69507,3.69584,0.02858\H,0,-3.17156,2.96964,-2.29528\H,0,-1.11
103,1.67838,-2.75475\H,0,-0.08787,1.82067,1.43332\H,0,-2.15159,3.09219
,1.8894\C,0,-6.9004,0.87714,-1.59081\N,0,-7.36401,1.71423,-2.25347\N,0
,4.79067,-2.16648,-0.5044\C,0,6.14135,-2.74887,-0.60749\O,0,7.01579,-2
.06404,-1.06455\C,0,6.31808,-4.16893,-0.12716\H,0,6.07851,-4.26748,0.9
3836\H,0,5.68949,-4.86716,-0.69271\H,0,7.36627,-4.43124,-0.28071\\Vers
ion=AM64L-G03RevD.01\State=1-A\HF=-1743.6136141\RMSD=4.193e-09\Thermal
=0.\Dipole=5.7324427,-2.4612484,0.095365\PG=C01 [X(C35H29N6O1)]\\@

414

1\1\GINC-CIP-D-01\SP\RHF\6-31G(d)\C32H27N5\C2253\31-Jul-2010\0\\#P RHF
/6-31G(d) scf=tight int=finegrid SCRF=(PCM,Read,Solvent=Chloroform) ge
om=check guess=read\\PCM of diaza2ci_7 b98/6-31G(d) structures on 4p 9
00 MB\\0,1\C,0,-0.3286473915,-0.0942977188,-0.117216954\C,0,-0.3395586
513,0.0123514184,1.286062337\C,0,0.8861869079,-0.2411985498,1.96872255
53\C,0,1.9874405693,-0.6259185261,1.1915692153\C,0,1.8604881907,-0.717
9557015,-0.1960125995\H,0,-1.2344479998,0.1225330069,-0.6782805674\H,0
,2.938434512,-0.8509670778,1.6621910711\H,0,2.7174996885,-1.0169339353
,-0.798537597\N,0,0.9620862306,-0.0705944608,3.3637934469\N,0,0.730165
628,-0.4542455299,-0.8553738332\N,0,-1.4987987807,0.3237250804,2.00170
62331\C,0,-0.315402482,-0.1151346897,4.0957191726\H,0,-0.7476879336,-1
.128151393,4.0339654855\C,0,-1.35916946,0.8005843808,3.3730884994\H,0,
-2.310699269,0.582028993,3.8777706115\H,0,-2.5268717158,1.7017020114,0
.7256467113\H,0,-3.489535149,0.9213487914,2.0032284603\C,0,4.434459064
6,-1.3898026505,5.4107595672\C,0,4.4191997268,-0.1449781704,4.76284955
64\C,0,3.2661205002,0.2892199721,4.110814635\C,0,2.1389301612,-0.54087
79719,4.0375382588\C,0,2.1888192821,-1.8195430718,4.6139845675\C,0,3.3

207342129,-2.2402194919,5.3099080708\H,0,5.3074818822,0.4801009515,4.8
036899398\H,0,3.2288669092,1.2702408508,3.6437809185\H,0,1.332458714,-
2.4835368072,4.5263506992\H,0,3.3468686172,-3.2255900146,5.7660241551\
N,0,5.6808074114,-1.7909888058,5.9976786069\N,0,5.7790482988,-2.328637
9548,7.1249196227\C,0,2.8047607221,-2.8023840504,10.0950713581\C,0,3.7
074291404,-3.8286588524,9.7948660129\C,0,4.666868186,-3.6454655047,8.7
981573791\C,0,4.6921157344,-2.4536712613,8.0562638068\C,0,3.7981147872
,-1.4144581933,8.3694841746\C,0,2.8643850311,-1.5937821011,9.390787221
1\H,0,2.068111749,-2.9373354941,10.8836255729\H,0,3.67748447,-4.763306
9488,10.3501009302\H,0,5.401624123,-4.414712814,8.5740851439\H,0,3.835
2112175,-0.4767947872,7.8231699146\H,0,2.1718981898,-0.7888171896,9.62
46145202\C,0,-2.6824214698,0.7623914255,1.2813495736\H,0,-3.007628368,
-0.0112104955,0.575341733\C,0,-0.7690320275,5.0933534096,3.7584510253\
C,0,-1.7361067936,4.434065674,4.5223290188\C,0,-1.9118997101,3.0541980
641,4.3862187273\C,0,-1.1245039404,2.3074701172,3.4958654654\C,0,-0.16
16046192,2.9822974511,2.7251907386\C,0,0.0136800608,4.3631826812,2.858
0391006\H,0,-0.6308870155,6.1677067469,3.8576599648\H,0,-2.3559561486,
4.9918264614,5.2209805517\H,0,-2.6651443718,2.5470692406,4.9876235358\
H,0,0.4442349929,2.4340058328,2.0099557215\H,0,0.7618003256,4.86945835
81,2.2516847078\C,0,-0.1055616012,0.8880307326,8.3112562568\C,0,-0.842
4208792,-0.2160440092,7.8736551705\C,0,-0.8764282495,-0.5366557237,6.5
129438117\C,0,-0.1862936345,0.2397499649,5.5707877212\C,0,0.5501404716
,1.3477646395,6.0208085961\C,0,0.5933221828,1.6652504596,7.3803586431\
H,0,-0.0787994338,1.1447146943,9.3683438764\H,0,-1.3860983863,-0.83084
14405,8.5875084984\H,0,-1.4455992685,-1.4033363167,6.1790700831\H,0,1.
0845726793,1.9641486104,5.3044329218\H,0,1.1663456459,2.5284750264,7.7
116872047\\Version=IA32L-G03RevE.01\State=1-A\HF=-1499.6653958\RMSD=4.
540e-09\Thermal=0.\Dipole=-1.9903486,0.512039,2.2141482\PG=C01 [X(C32H
27N5)]\\@

414-Ac^{+}

1\1\GINC-LX64I155\SP\RHF\6-31G(d)\C34H30N5O1(1+)\LU23QOW\04-Sep-2010\0
\\#P RHF/6-31G(d) scf=tight int=finegrid SCRF=(PCM,Read,Solvent=Chloro
form) geom=check guess=read\\PCM of diaza2ci_7.ac2.sp b98/6-31G(d) str
uctures on lrz\\1,1\C,0,-0.235791994,0.1785207074,-0.1500758165\C,0,-0
.3040828767,0.1899780142,1.2338687195\C,0,0.8933382538,-0.1634377552,1
.9763985505\C,0,2.0416211069,-0.5294803693,1.2245255891\C,0,2.02035603
64,-0.5501693705,-0.1464633766\H,0,-1.0594047737,0.4595674572,-0.79038
06771\H,0,2.9511551749,-0.8166993623,1.7369663266\H,0,2.8849893513,-0.
84770938,-0.7235898725\N,0,0.892704018,-0.1375386792,3.3271373926\N,0,
0.8965821618,-0.2028122319,-0.8309506175\N,0,-1.4581962029,0.511333437
2,1.9054981936\C,0,-0.4122430635,-0.0486564432,4.0493418456\H,0,-0.890
3708671,-1.0336170316,3.9547333303\C,0,-1.3678757645,0.9375744325,3.30
78484374\H,0,-2.350481454,0.7324086422,3.7497061489\H,0,-2.4532803735,
1.901820962,0.6255581535\H,0,-3.4583224539,1.0874125093,1.8455848747\C
,0,4.3827981463,-1.2609366063,5.4562927798\C,0,4.2864145483,0.01668465
57,4.8803483643\C,0,3.1279662899,0.3949084934,4.2044277996\C,0,2.08180
12046,-0.5235888739,4.0529262198\C,0,2.1930623165,-1.8175098221,4.5780
221505\C,0,3.3366054222,-2.1850233556,5.2849802084\H,0,5.121404234,0.7
048591753,4.9805849521\H,0,3.0342555591,1.3943008643,3.7860615101\H,0,
1.3866125311,-2.53361484,4.4400852644\H,0,3.4245711322,-3.1856745324,5
.6976754309\N,0,5.655436941,-1.6352510556,5.9975425997\N,0,5.818755838
5,-2.1964612236,7.1036810548\C,0,2.9919042283,-2.8503684562,10.1701498
341\C,0,3.9619114099,-3.8075861266,9.8508324631\C,0,4.8767089797,-3.55
75786851,8.8281492928\C,0,4.784135781,-2.37349586,8.0771586223\C,0,3.8
215570104,-1.4022558691,8.4083238382\C,0,2.9368815969,-1.6450523318,9.
4594133328\H,0,2.2958340254,-3.032949508,10.985123733\H,0,4.021979296,
-4.7346267106,10.4152028572\H,0,5.6662177099,-4.2653989454,8.589867896

7\H,0,3.7812230587,-0.460017734,7.8705223029\H,0,2.2009204232,-0.88921
01634,9.722372887\C,0,-2.6269161492,0.9504570446,1.1503079586\H,0,-2.9
154405114,0.1833071131,0.4220399709\C,0,-0.670072875,5.1905486708,3.85
84668883\C,0,-1.6727802711,4.5302373402,4.5740348776\C,0,-1.884411945,
3.1625536195,4.3809456041\C,0,-1.0969017573,2.4318536162,3.4776524917\
C,0,-0.0996005237,3.1076402681,2.7539706034\C,0,0.1128271217,4.4763017
186,2.9458633019\H,0,-0.5056227908,6.2553286055,4.002616318\H,0,-2.293
8512124,5.0780365809,5.2782383878\H,0,-2.6654957131,2.6561394385,4.945
3602329\H,0,0.5084306663,2.5808740549,2.0229396896\H,0,0.8858252703,4.
9861931307,2.3757882527\C,0,-0.1838898539,0.7567322761,8.2979443697\C,
0,-0.8887874432,-0.3481595667,7.8133543513\C,0,-0.9282732645,-0.600585
0861,6.4388990572\C,0,-0.2755154604,0.2495369187,5.5334003064\C,0,0.42
88219811,1.3593321776,6.0296628819\C,0,0.4780482901,1.6055305353,7.403
3179092\H,0,-0.1540468526,0.9600815291,9.3658815564\H,0,-1.4046461739,
-1.0148087111,8.4997189393\H,0,-1.4739506389,-1.4676388941,6.069232120
5\H,0,0.9353325523,2.035857786,5.3480732251\H,0,1.025030705,2.46889560
6,7.7740708433\C,0,0.8246123326,-0.197534295,-2.3017027659\O,0,-0.2123
775909,0.1266746482,-2.8147073115\C,0,2.0692532184,-0.602401539,-3.054
4765916\H,0,2.3677272272,-1.6305084304,-2.8168973764\H,0,1.8353647199,
-0.5379707333,-4.118506039\H,0,2.910173608,0.0654432982,-2.8328981225\
\Version=IA64L-G03RevE.01\State=1-A\HF=-1651.8801674\RMSD=3.217e-09\Th
ermal=0.\Dipole=-1.4967669,-0.210571,-3.7627494\PG=C01 [X(C34H30N5O1)]
\\@

415

1\1\GINC-CIP-F-14\SP\RHF\6-31G(d)\C33H26N6\C2253\21-Aug-2010\0\\#P RHF
/6-31G(d) scf=tight int=finegrid SCRF=(PCM,Read,Solvent=Chloroform) ge
om=check guess=read\\PCM of diaza2cn_tr_1 b98/6-31G(d) structures on 4
p 900 MB\\0,1\C,0,-3.8673623967,3.4615489664,-0.358153422\C,0,-3.34947
85587,2.1644385505,-0.5415313632\C,0,-1.9330901915,2.0126677353,-0.471
7881402\C,0,-1.171019875,3.1771923092,-0.3004151089\C,0,-1.8075385658,
4.4110246737,-0.1556426727\H,0,-4.9438862247,3.6135825004,-0.362585875
9\H,0,-0.0879988467,3.1282464935,-0.2796209823\H,0,-1.2128752245,5.313
3998349,-0.0189936915\N,0,-1.3610366595,0.7290766033,-0.5767153262\N,0
[truncated: 67,556 more chars]
